# Short Scalable Route to Bis-morpholine Spiroacetals and Oxazepane Analogues: Useful 3D-Scaffolds for Compound Library Assembly

**DOI:** 10.1021/acs.joc.4c02690

**Published:** 2025-02-10

**Authors:** Daniel Kovari, Louise Male, Kimberley A. Roper, Christian P. Mang, Oliver Kunz, Liam R. Cox

**Affiliations:** † School of Chemistry, The University of Birmingham, Edgbaston, Birmingham B15 2TT, U.K.; ‡ School of Pharmacy, The University of Birmingham, Edgbaston, Birmingham B15 2TT, U.K.; § AnalytiCon Discovery GmbH, Hermannswerder 17, 14473 Potsdam, Germany

## Abstract

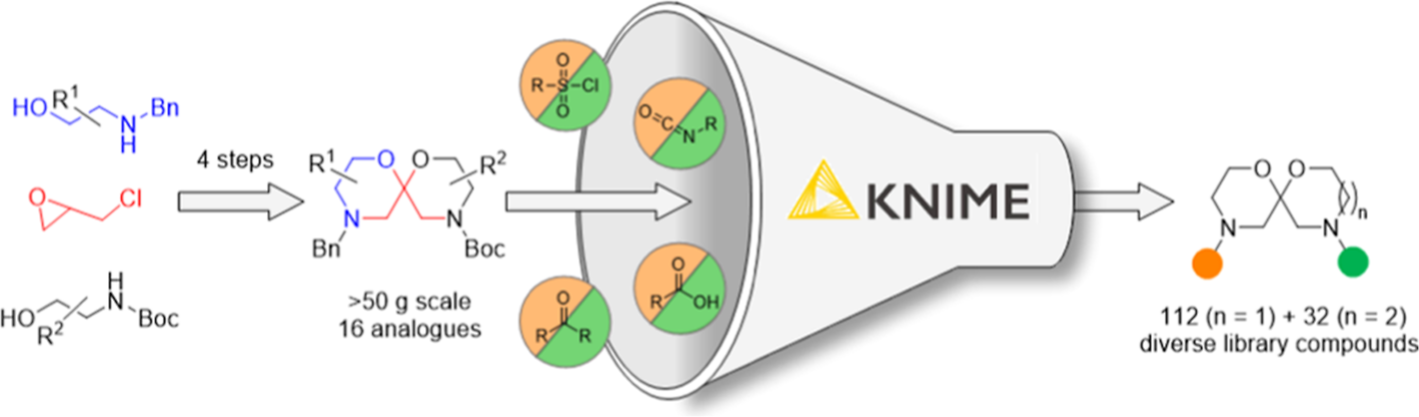

sp^3^-Rich molecular scaffolds incorporating nitrogen heterocycles represent important starting points for assembling compound screening libraries and drug discovery. Herein, we report a four-step synthesis of a conformationally well-defined sp^3^-rich scaffold incorporating two morpholine rings embedded within a spiroacetal framework. The synthesis involves the intermediacy of a 2-chloromethyl-substituted morpholine, accessed from epichlorohydrin and readily available β-aminoalcohols. Base-mediated dehydrochlorination affords an exocyclic enol ether, from which the second morpholine ring is constructed in two steps. Scaffold synthesis is high-yielding and can be performed on a large scale. The methodology allows ready substitution of one–or both– of the morpholine rings for 1,4-oxazepanes and the generation of 6,7- and 7,7-spiroacetal analogues, which are virtually unexplored in drug discovery. Substituted 6,6-systems can be prepared and, in some instances, undergo acid-mediated anomerization to deliver the scaffolds in high diastereoselectivity. The two amine functionalities embedded in the 6,6- and 6,7-spiroacetal scaffolds were sequentially functionalized to provide a diverse physical compound library. These library compounds occupy a similar chemical space to small-molecule drugs that have been approved for clinical application by the Food and Drug Administration yet are structurally dissimilar and may therefore act upon novel targets, representing attractive starting materials for drug discovery.

## Introduction

Conformationally well-defined three-dimensional scaffolds are attractive starting points for compound library assembly and applications in early-stage drug discovery. Conformational rigidity in these sp^3^-rich systems can be achieved in various ways, through bridged systems and ring fusion in polycycles,^1^ as well as through spiro systems. Spirocyclic molecules are widespread among bioactive natural products^2^ and have received growing interest as scaffolds for drug discovery.^3^ The presence of a spiranic center within the core of a molecular scaffold necessarily introduces a high degree of three-dimensionality, typically significant conformational rigidity, and a limited number of low-energy conformations. Functionality embedded within the constituent rings then introduces exit vectors and a means to predictably orient recognition motifs into well-defined regions of three-dimensional space, which is attractive for structure-based drug design.^3a,4^ Moreover, there are examples in which the incorporation of a spirocycle imparts favorable physicochemical properties^5^ and an improved pharmacokinetics profile compared with nonspirocyclic analogues.^3a^ A number of groups have reported diversity libraries of spiro(hetero)cyclic compounds^6^ and bis-spirocyclic frameworks.^7^ Müller and co-workers used a cheminformatics approach to classify systematically the structures and chemical space interrogated by spirocycles of known biological activity.^3b^ This study highlighted the capacity for this type of scaffold to access a significant volume of biologically relevant chemical space, reinforcing the attraction of deploying spirocycles for drug discovery.

Spiroacetals are an important class of spirocycle;^8^ they are ubiquitous across natural products, many of which display potent and diverse biological activities.^9^ While the hydrolytic instability of acyclic acetals might raise a safety flag owing to the aldehyde/ketone hydrolysis product, the increased stability of spiroacetals and their abundance in natural products justifies their inclusion in compound screening libraries and potential to deliver attractive starting points for drug discovery.^10^ Aliphatic nitrogen heterocycles also abound in bioactive natural products and are privileged structural motifs for drug discovery. The tetrahydro-1,4-oxazine–or morpholine– framework is particularly widespread, and synthetic routes to this nitrogen heterocycle, its prevalence in drugs, and pharmacological activity have been extensively reviewed.^11^ Azaspiro systems have been accessed in various ways,^12^ and azaspiro scaffolds incorporating a morpholine exhibit diverse bioactivities. Examples include AZD-2115, a dual-acting M3 receptor antagonist/β2-adrenergic receptor agonist,^3a^ NK1-antagonists,^13^ and potent HIV protease inhibitors,^14^ while a pyrrolomorpholine spiroacetal forms the core of the shensongine and acortatarin classes of natural products, which display antioxidant activity.^15^

## Results and Discussion

**Figure 1:**
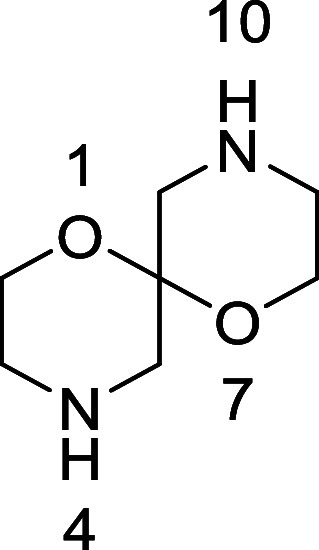
1,7-Dioxa-4,10-diazaspiro[5.5]undecane as a scaffold for compound library synthesis.

**Scheme 1:**
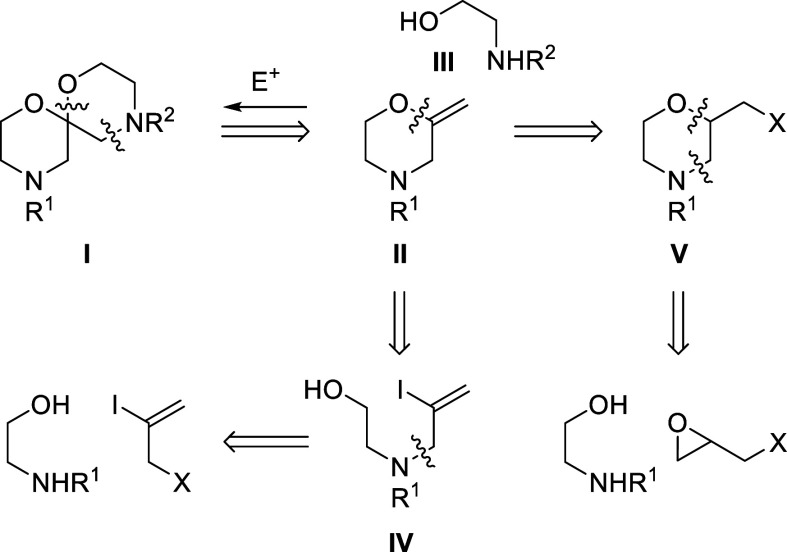
Retrosynthetic Analysis of Target Bis-morpholine Spiroacetal **I**

We postulated that a spiro-bis-morpholine [1,7-dioxa-4,10-diazaspiro[5.5]undecane] framework (Figure 1) would provide an attractive scaffold for compound library assembly,^16^ especially if a scalable route could be developed that enabled sequential functionalization of the two amines. We envisaged orthogonally protected spiro bis-morpholine **I** could be accessed in two steps from 2-methylidenemorpholine **II**, using an electrophile-mediated regioselective functionalization of the exocyclic alkene with an ethanolamine bis-nucleophile **III**(17) to assemble the spiroacetal and second morpholine ring (Scheme 1). Two approaches to enol ether **II** were explored.

Stoltz and co-workers reported the synthesis of a range of 2-methylidenemorpholines, including enol ether **II** (*R*^1^ = Bn), via an intramolecular Ni(COD)_2_-catalyzed cross-coupling reaction of the corresponding iodo-aminoalcohol **IV** (Scheme 1).^18^ This reaction was successfully applied to a range of vinyl iodides (see the Supporting Information), including one containing a secondary amine [**IV** (*R*^1^ = H)], which extends the scope of this methodology; however, we were unable to access unsubstituted 2-methylidenemorpholine **II** (*R*^1^ = Bn) efficiently, achieving 20% conversion at best.^19^ A second approach was therefore explored, which ultimately provided a higher yielding, more practical, and scalable synthesis of this enol ether.

**Scheme 2:**
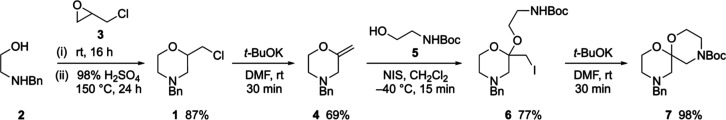
Synthesis of Bis-morpholine Spiroacetal **7**

We envisaged enol ether **II** (*R*^1^ = Bn) could also be accessed via dehydrohalogenation^20^ of the corresponding 2-halomethyl-substituted morpholine **V** (Scheme 1).^21^ 2-Chloromethylmorpholine **1** was therefore prepared in a two-step, one-pot operation,^22^ first introduced by Loftus^22a^ and later improved by Matsumoto and co-workers;^22b^ thus, treatment of *N*-benzylethanolamine (**2**) with an equimolar quantity of epichlorohydrin (**3**) in the absence of solvent at room temperature, followed by the addition of 98% H_2_SO_4_ and heating at 170 °C, afforded 2-chloromethylmorpholine **1** in 87% yield (Scheme 2). While this reaction sequence employs particularly harsh conditions in the second step, it proved remarkably scalable and was performed on a 0.37 mol scale, providing 72 g of the target product. Subsequent dehydrochlorination on **1** using *t*-BuOK as the base,^23^ and DMF as solvent,^24^ afforded 2-methylidenemorpholine **4** (Scheme 2). Unlike the corresponding 2-methylidenetetrahydropyran, which needs to be stored over KOH,^25^ 2-methylidenemorpholine **4** could be purified by column chromatography using a basic eluent (see the Supporting Information for full details) and the purified product was stored under nitrogen at 4 °C for several weeks without any noticeable decomposition.^26^

**Figure 2:**
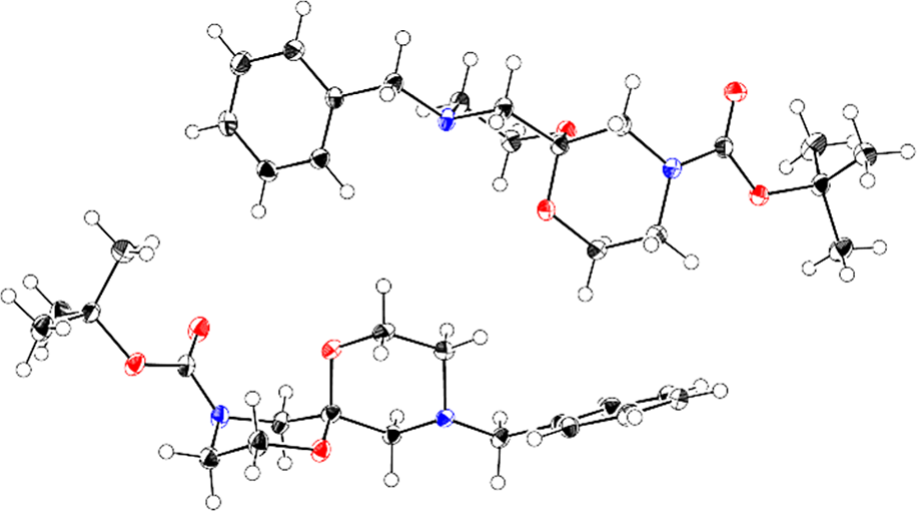
ORTEP plot of **7** with ellipsoids drawn at the 50% probability level, confirming the double anomeric stabilization of the spiroacetal. The structure contains two crystallographically independent molecules as shown. Atomic displacement parameters at 100 K.

Adapting a methodology introduced by Nishi and co-workers to assemble 2,2-disubstituted morpholines,^27^ 2-methylidenemorpholine **4** underwent regioselective iodoacetalization upon treatment with *N*-iodosuccinimide in the presence of *N*-Boc-ethanolamine (**5**) to give iodide **6**. Subsequent treatment with *t*-BuOK in DMF effected ring closure to provide bis-morpholine spiroacetal **7**. Starting from 17 g (92 mmol) of enol ether **4** delivered target scaffold **7** in 75% yield over the two steps (Scheme 2).^28^ The structural identity of **7** was confirmed by single-crystal X-ray diffraction, which revealed both rings adopt chair conformations and the spiroacetal benefits from double anomeric stabilization (Figure 2). A solution of **7** in DMSO left in an NMR tube at room temperature on an open bench over 3 weeks showed no evidence of decomposition, as determined by ^1^H- and ^13^C{^1^H}-NMR spectroscopy; this scaffold was therefore deemed sufficiently stable for compound library synthesis.

**Scheme 3:**
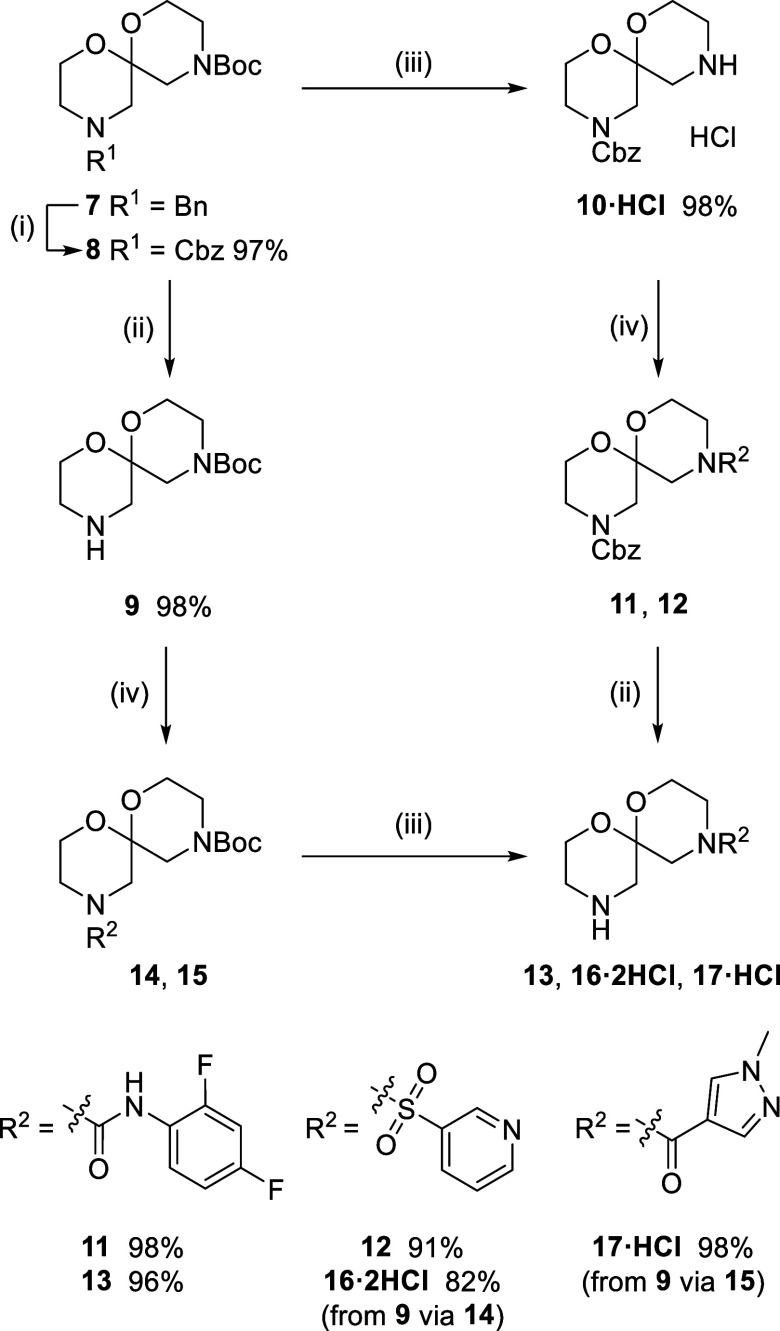
Orthogonal Deprotection and Sequential Functionalization of Spiro-bis-morpholine **8** Reaction conditions: (i) CbzCl, CH_2_Cl_2_, rt, 24 h; (ii) H_2_, 10% Pd/C, THF/H_2_O 4/1, rt; (iii) HCl (4 M in 1,4-dioxane), THF/H_2_O 4/1, rt; (iv) 2,4-difluoro-1-isocyanatobenzene, Et_3_N, CH_2_Cl_2_, rt, 1 h, or pyridine-3-sulfonyl chloride, Et_3_N, CH_2_Cl_2_, 0 °C–rt, 16 h, or 1-methyl-1*H*-pyrazole-4-carboxylic acid, HATU, *i*Pr_2_NEt, CH_2_Cl_2_, 0 °C–rt, 16 h.

Orthogonal deprotection of the two amines in spiroacetal **7** was next explored. Standard benzylamine deprotection conditions (H_2_/Pd/C in MeOH with or without acetic acid and the use of 1-chloroethyl chloroformate in the presence of acid scavengers) proved ineffective.^29^ As Cbz carbamates undergo hydrogenolysis more readily,^30^ benzylamine **7** was converted to carbamate **8** (Scheme 3).^31^ Now, hydrogenolysis of **8**, performed on a 33 mmol scale, proceeded without event under neutral conditions^32^ to deliver secondary amine **9** with the Boc group intact.

While a protecting group exchange is undesirable on efficiency grounds, the reaction sequence was straightforward and provided **9** in 95% yield over the two steps. The selective deprotection of the Boc carbamate in **8** was also confirmed: treatment with 4 M HCl in 1,4-dioxane^33^ led to rapid Boc deprotection, leaving the 6,6-spiroacetal intact, to provide **10**, which was conveniently isolated as its HCl salt (Scheme 3).

Having access to two differently monoprotected bis-morpholine spiroacetals offered useful flexibility for scaffold decoration. Thus, treatment of amine HCl salt **10·HCl** with 2,4-difluoro-1-isocyanatobenzene and pyridine-3-sulfonyl chloride under standard conditions provided urea **11** and sulfonamide **12**, respectively (Scheme 3). Hydrogenolysis of the Cbz carbamate in urea **11** provided **13** in excellent yield; however, the same reaction with sulfonamide **12** saw recovery of the starting material, even when the carbamate deprotection was performed under acidic conditions to protonate the basic nitrogen in the embedded pyridine (Scheme 3). Turning instead to Boc carbamate **9**, reaction with pyridine-3-sulfonyl chloride and 1-methyl-1*H*-pyrazole-4-carboxylic acid provided sulfonamide **14** and amide **15**, respectively. This time, subsequent Boc deprotections proceeded without event, affording sulfonamide **16·2HCl** in 82% yield and amide **17·HCl** in 98% yield, over the two steps.

Having successfully functionalized one of the morpholine rings and deprotected the resulting products, the KNIME analytics platform^34^ was used to enumerate a virtual library of 630 compounds, from which 124 diverse^35^ compounds were selected for physical synthesis (see the Supporting Information). Compounds were chosen to occupy drug-like chemical space, obeying Lipinski’s rule of five and Veber’s rules.^36^ Urea **13** and 12 singly decorated scaffolds, synthesized from Boc carbamate **9** in yields ranging between 66 and 98% over the two steps, were applied in amidation, sulfonylation, urea formation, and reductive amination reactions to assemble a compound library (see the Supporting Information).^37^ Of the 124 reactions performed, only one failed to deliver any product after purification by HPLC, confirming bis-morpholine spiroacetal **I** as a viable scaffold for compound library assembly.^38^

**Figure 3:**
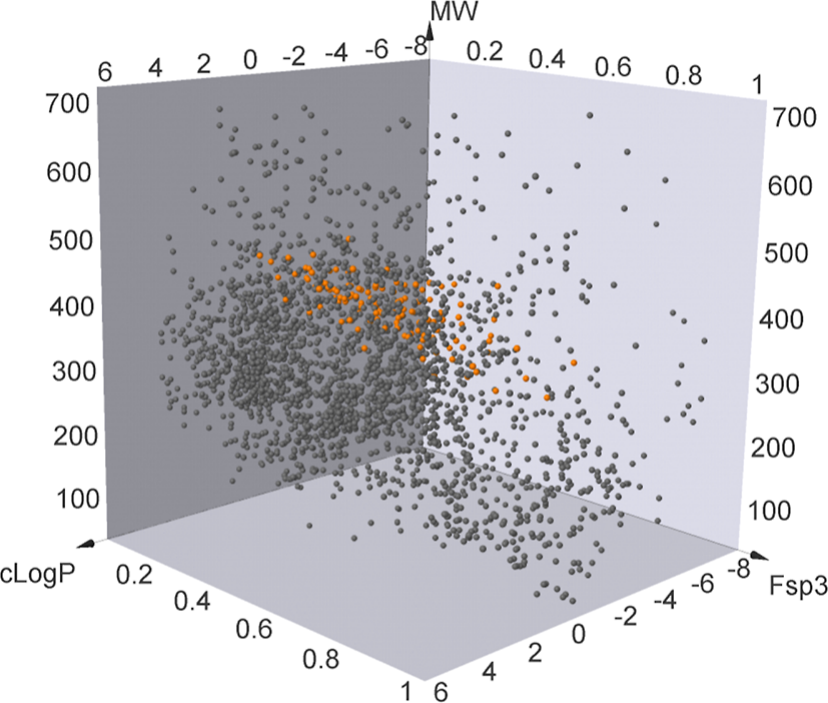
Comparison of library of synthesized spiro-bis-morpholine compounds (*n* = 124) with the FDA-approved small-molecule drugs^39^ (*n* = 2709) visualized in c Log *P*/MW/Fsp^3^ space [FDA-approved drugs (dark gray), spiro-bis-morpholine library compounds (orange)].

To assess its chemical space coverage, the bis-morpholine spiroacetal library was compared with the collection of small-molecule drugs (molecular weight (*M*_W_) < 700 Da^39^) that have been approved by the Food and Drug Administration,^40^ in the fraction of sp^3^ carbon atoms (Fsp^3^)—lipophilicity (c Log *P*)—MW chemical space (Figure 3). The 3D scatter plot indicates that the library occupies similar physicochemical space to the FDA-approved list of small-molecule drugs. To evaluate the dissimilarity of the compound library from this set of FDA-approved drugs,^41^ Tanimoto coefficients^42^ were calculated (see the Supporting Information); the obtained scores of 0.2–0.4 indicate significant dissimilarity; thus, while our library compounds have similar physicochemical properties to clinically approved small-molecule drugs, they are structurally distinct. We hypothesize that they may act on new targets and, therefore, represent attractive starting points for drug discovery.

**Scheme 4:**
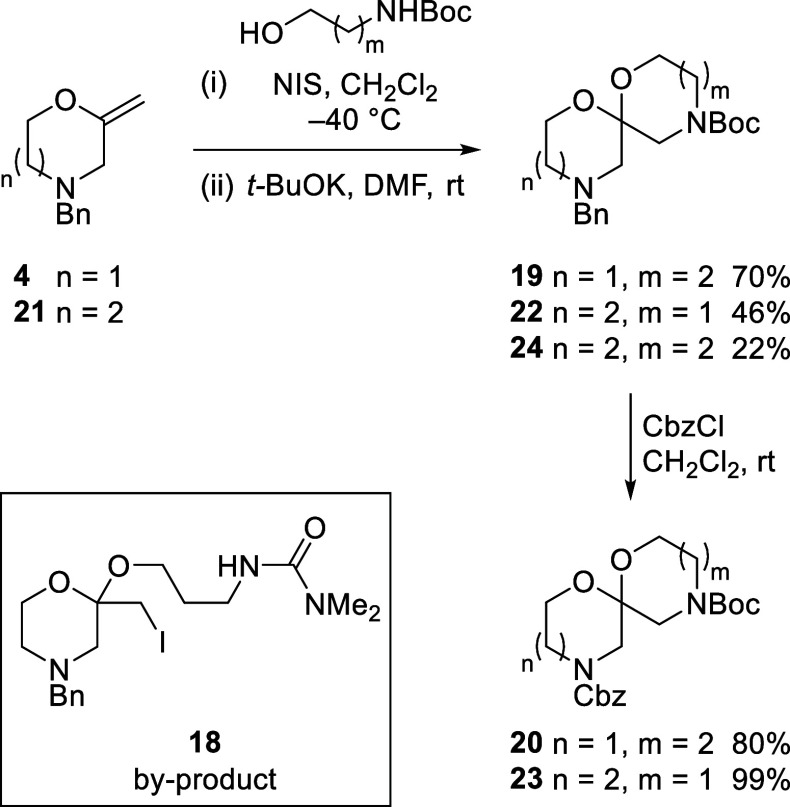
Formation of Spiroacetals Incorporating 1,4-Oxazepanes

Six-membered cyclic amines (piperidines, piperazines, and morpholines) abound in bioactive compounds. By contrast, seven-membered cyclic amines are far less common,^43^ and their incorporation into 6,7-spirocycles is even more unusual;^3b,10a,12a^ thus, the 6,7-spirocyclic homologue of 6,6-spiroacetal **8** would represent a novel scaffold for compound library assembly. Iodoacetalization of enol ether **4** using *N*-Boc-propanolamine proceeded uneventfully to provide the corresponding iodide. Subsequent formation of the 1,4-oxazepane using our established cyclization conditions proved inefficient; the slow reaction led to competing formation of a urea byproduct **18** (Scheme 4).^44^ The base-mediated elimination of *t*-BuOH from Boc carbamates to afford an isocyanate has been reported.^45^ We postulate urea **18** arises from isocyanate trapping with dimethylamine, which originates from the slow decomposition of the DMF solvent under the reaction conditions.^46^ Urea formation was suppressed by performing the reaction at higher concentration (0.2 M) and using 2 equiv of *t*-BuOK; the increased rate of cyclization now allowed isolation of oxazepane **19** in 70% yield. Exchanging the benzyl protecting group in **19** for a Cbz carbamate provided orthogonally protected 6,7-spiroacetal **20** in a high yield (Scheme 4). Efforts to synthesize the corresponding 6,8- and 6,9-spiroacetals failed (see the Supporting Information).

**Scheme 5:**
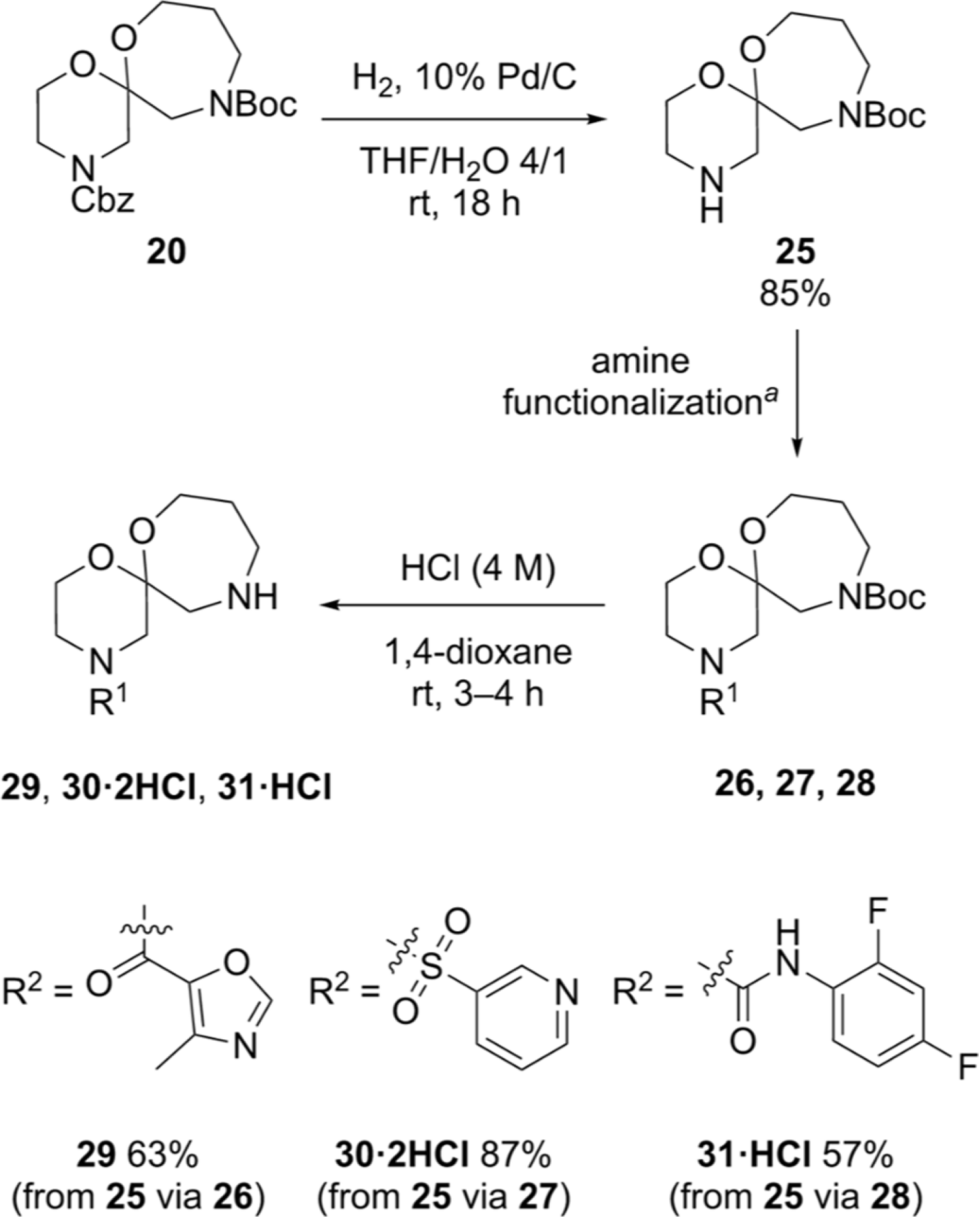
Orthogonal Deprotection and Functionalization of 6,7-Spiroacetal Scaffold **20** Reaction conditions: 4-methyloxazole-5-carboxylic acid, HATU, *i*Pr_2_NEt, CH_2_Cl_2_, 0 °C–rt, 16 h, 79% (**26**); or pyridine-3-sulfonyl chloride, Et_3_N, CH_2_Cl_2_, 0 °C–rt, 16 h, 89% (**27**); or 2,4-difluoro-1-isocyanatobenzene, Et_3_N, CH_2_Cl_2_, rt, 2 h, 89% (**28**).

As a complementary strategy, 2-methylidene-1,4-oxazepane **21** was synthesized from 3-benzylaminopropan-1-ol and epichlorohydrin, using our established method (see the Supporting Information). Iodoacetalization with *N*-Boc-ethanolamine (**5**), followed by morpholine ring formation, provided 7,6-spiroacetal **22** in 46% yield over two steps. Exchanging the benzyl protecting group in **22** for a Cbz carbamate provided the orthogonally protected 7,6-spiroacetal scaffold **23**. Crystals suitable for analysis by single-crystal X-ray diffraction confirmed the structure of **23**, with the spiroacetal benefiting from double anomeric stabilization (see the Supporting Information). As a final example, bis-oxazepane spiroacetal **24** was also synthesized via this route. While the formation of the intermediate iodoacetal proceeded in good yield from enol ether **21** and *N*-Boc-propanolamine, subsequent cyclization to install the second oxazepane ring furnished **24** in low yield. No efforts were made to optimize this reaction. 6,7-Spiroacetal **20** was accessed on >50 g scale.^47^ Hydrogenolysis of the Cbz carbamate afforded secondary amine **25** in good yield. Amine functionalization with a representative amide (**26**), sulfonamide (**27**) and urea (**28**), followed by Boc deprotection, provided 6,7-spiroacetals **29**,^48^**30·2HCl**, and **31·HCl**, respectively (Scheme 5), from which a small library of 33 compounds was synthesized without event (see the Supporting Information).^49^ This small study suggests that spiroacetal scaffolds containing 1,4-oxazepanes are viable starting materials for compound library assembly.

**Scheme 6:**
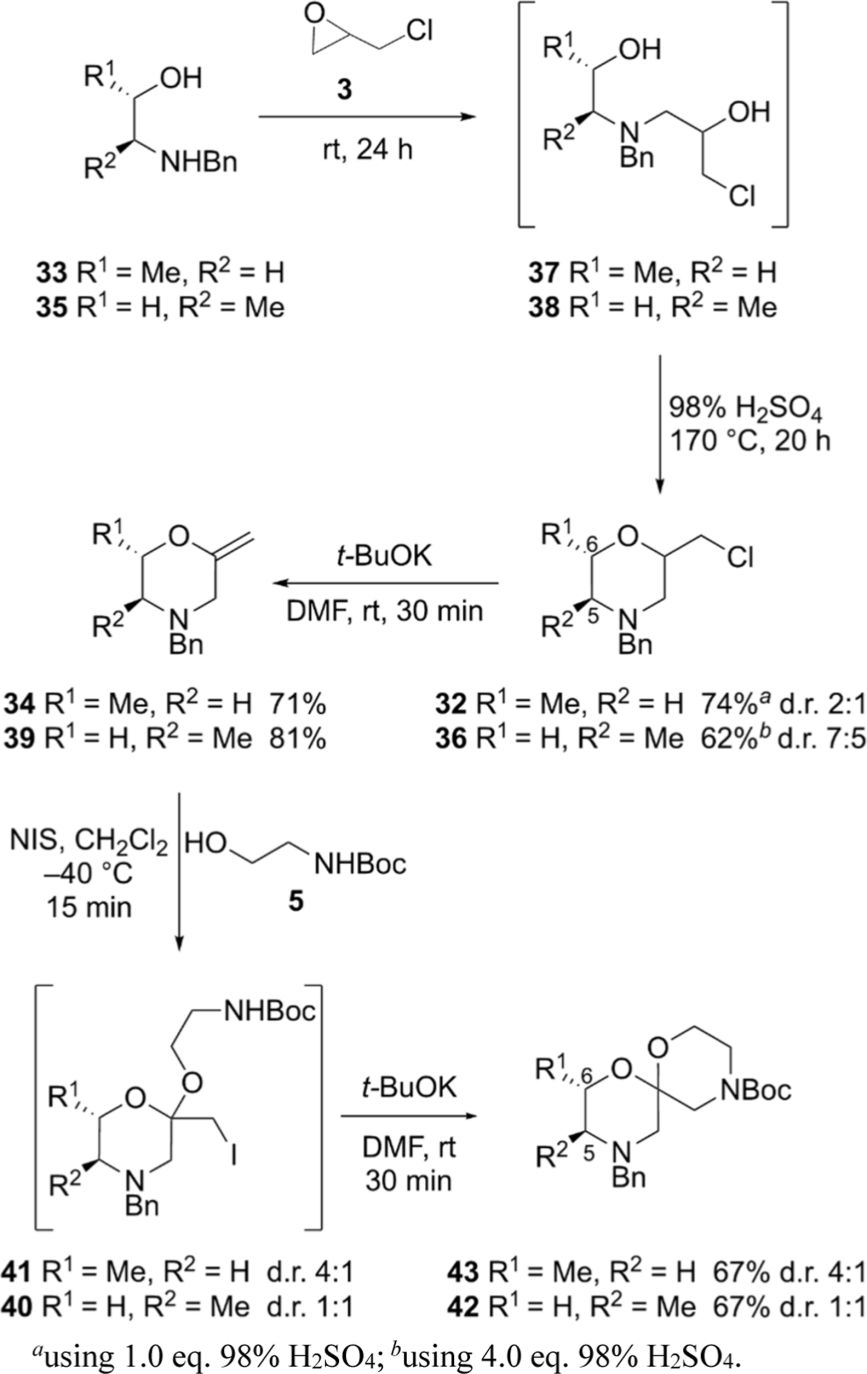
Formation of Methyl-Substituted Bis-morpholine Spiroacetals **42** and **43**

The introduction of even a methyl substituent^50,51^ can drastically affect lipophilicity, solubility, bioavailability, and selectivity of bioactive compounds;^52^ thus, being able to access methylated and similarly substituted spiroacetal scaffolds would potentially be useful for future hit optimization studies. 2-Chloromethyl-6-methylmorpholine **32** was produced in good yield under our standard conditions from (*S*)-1-benzylaminopropan-2-ol (**33**) and *rac*-epichlorohydrin (**3**) and isolated as a ∼2:1 mixture of diastereoisomers^53^ (Scheme 6). Dehydrochlorination provided enol ether **34** in 71% yield.^54^ Incorporating a methyl substituent at the 5-position of the morpholine proved more challenging: analogous reaction of (*S*)-2-benzylaminopropan-1-ol (**35**) with *rac*-epichlorohydrin (**3**) afforded 6-regioisomer **32**, again as a ∼2:1 mixture of diastereomers; the desired 2,5-disubstituted regioisomer **36** was not observed. LCMS analysis of the first step in both reactions revealed the formation of a 1:1 mixture of two chlorohydrin diastereoisomers (**37** from **33** and **38** from **35**), consistent with both enantiomerically pure aminoalcohols reacting similarly with the racemic epoxide. This observation suggested that the formation of the 2,6-regioisomer from **35** was occurring in the second step of the reaction. Performing this step at different temperatures (150, 170, and 190 °C) had no impact on the outcome; however, the stoichiometry of acid did: using 1.0 equiv of 98% H_2_SO_4_ led to exclusive formation of 2,6-disubstituted regioisomer **32**; using 2.0 equiv of acid, both morpholine regioisomers were observed in a 2:1 ratio, with the 2,6-regioisomer now the minor product. Formation of this regioisomer was suppressed completely when 4.0 (or greater) equiv of acid was employed;^55,56^ under these conditions, morpholine **36** was obtained in 62% yield (Scheme 6).^54^ In the presence of excess acid, we tentatively propose that the amine in **38** is effectively permanently protonated, suppressing the formation of aziridinium species,^57^ which provide a pathway for isomerization (see the Supporting Information).

Dehydrohalogenation of regioisomers **32** and **36** provided 2-methylidenemorpholines **34** and **39**, respectively, in good yields (Scheme 6). Iodoacetalization proceeded efficiently on both enol ethers. No stereoinduction was observed in the reaction of 5-methyl-substituted enol ether **39**; the iodoacetal product **40** was isolated as a 1:1 mixture of diastereoisomers. Moving the methyl substituent to the 6-position of the morpholine ring (**34**) led to modest diastereoselectivity, with iodoacetal **41** now formed as a 4:1 mixture of diastereoisomers. In both cases, the diastereoisomeric ratio did not change when the reaction was performed at 0 or −78 °C. Finally, cyclization to introduce the second morpholine ring afforded 5- and 6-methyl-substituted spiroacetals, **42** and **43**, respectively (Scheme 6). In both cases, the diastereoisomeric ratio of the iodoacetal starting materials was preserved in the spiroacetal products, suggesting efforts to improve the diastereomeric ratio, for example, using a chiral electrophilic iodine reagent would be worth exploring in the future. It was not possible to determine by NMR spectroscopy the orientation of the methyl substituent in the two diastereoisomers of spiroacetal **42** (5-Me) owing to resonance overlap and the presence of rotamers; however, the appearance of the resonance for one of the C(5)Hs in the major diastereoisomer of spiroacetal **43** (6-Me) as an apparent triplet [δ_H_ = 1.81 (app t, *J* = 10.9 Hz)] is consistent with an axially oriented hydrogen substituent at C(6) (Scheme 6). We were unable to confirm the stereochemistry of the spiroacetal; however, based on the crystal structure of spiroacetal **7** (Figure 2) and literature precedent,^58^ we hypothesize that this diastereoisomer is the double anomerically stabilized spiroacetal with an equatorial methyl substituent.

**Figure 4:**
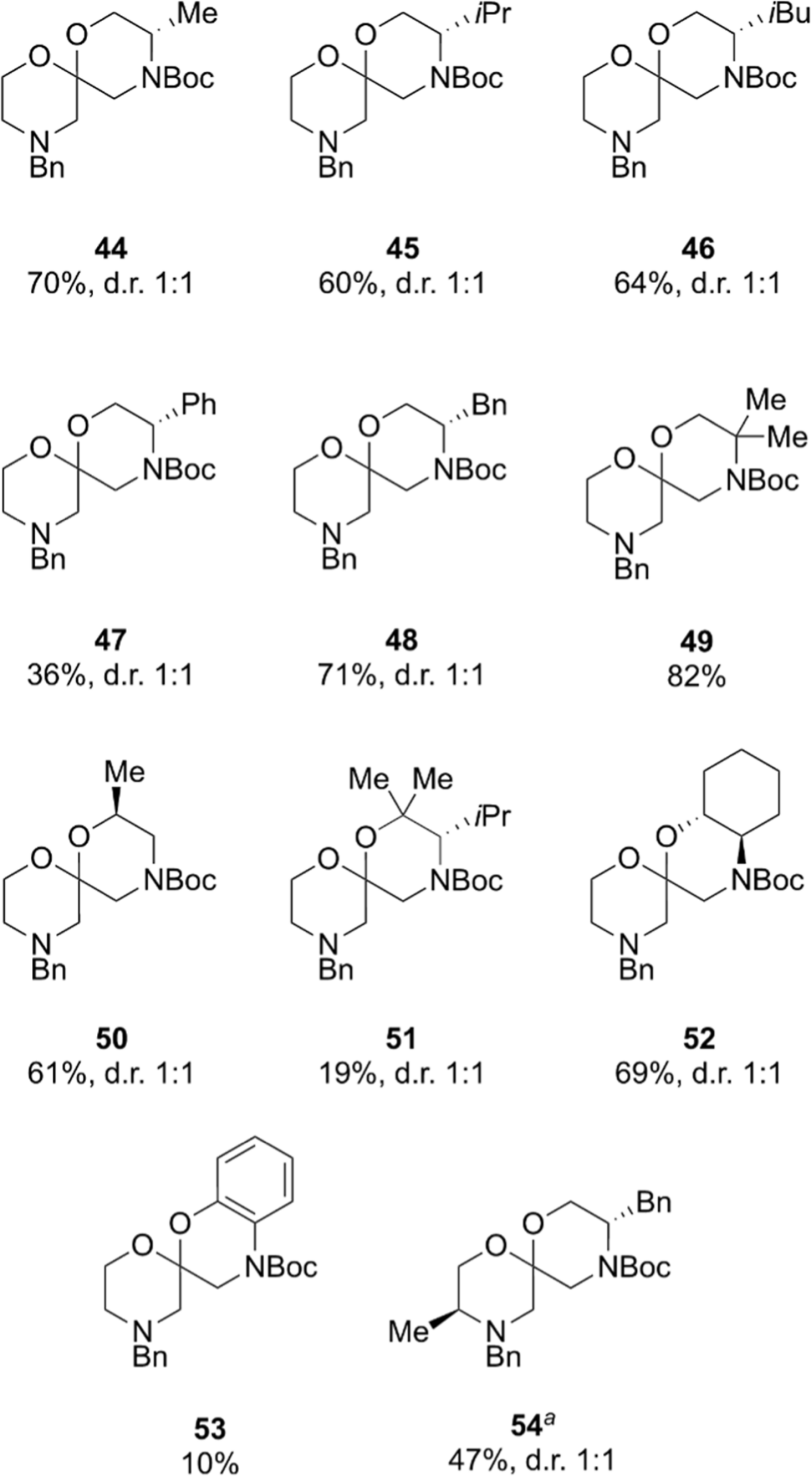
Synthesized substituted bis-morpholine spiroacetals. ^a^Synthesized from enol ether **39**.

Incorporating substituents into the ring formed in the final cyclization step proved to be more straightforward. Ten commercially available, enantiopure (where applicable) Boc-protected aminoalcohols were used to produce substituted spiroacetals **44**–**54** (Figure 4). In most cases, the spiroacetals were isolated in greater than 60% yield from 2-methylidenemorpholine **4** and, where relevant, as 1:1 mixtures of diastereoisomers, highlighting that the stereochemical information embedded in the nucleophile imparted no stereoinduction. The formation of 1,1-dimethyl-2-isopropyl-substituted spiroacetal **51** was low-yielding, presumably because of the sterically hindered tertiary alcohol employed in the iodoacetalization step. The reaction sequence to form benzene-fused spiroacetal **53** was also low-yielding, which is likely a consequence of the lower nucleophilicity of both the phenol in the iodoacetalization step and the aniline carbamate in the cyclization. The highest overall yield was observed for the formation of gem-dimethyl-substituted spiroacetal **49**, which may be attributed to the Thorpe–Ingold effect facilitating ring closure.^59^

**Scheme 7:**
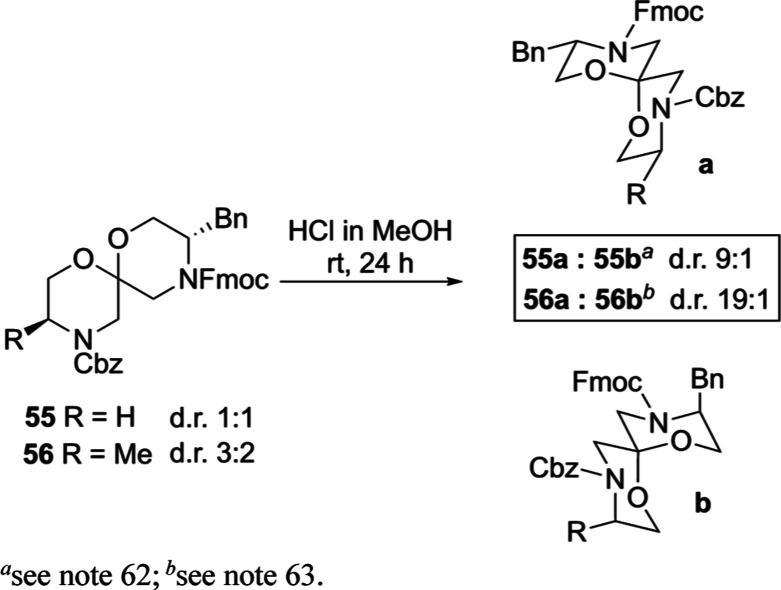
Anomerization of Spiroacetals **55** and **56**

Racemates are commonly employed in high-throughput screens of compound libraries; however, the inclusion of diastereoisomeric mixtures is generally avoided. Lewis acids have been used to epimerize heterocyclic acetals;^60^ however, we focused on the use of Brønsted acids, pioneered by Deslongchamps on similarly substituted 6,6-spiroacetals (1,7-dioxaspiro[5.5]undecanes), to explore the possibility of improving the diastereoisomeric ratio of substituted bis-morpholine spiroacetals by anomerization.^58^ Unsurprisingly, the attempted anomerization of spiroacetal **48** using 3 M HCl in MeOH led to Boc deprotection. An analogue (**55**) containing Cbz and Fmoc carbamate protecting groups was therefore synthesized (see the Supporting Information). A 1:1 diastereoisomeric mixture of **55** was treated with 3 M HCl in MeOH. After 24 h at room temperature, the ratio had increased to 9:1, as determined by LCMS.^61^ This ratio did not change over the course of 1 week, suggesting the reaction had reached equilibrium. It was not possible to confirm the structure of the two diastereoisomers by ^1^H NMR spectroscopy owing to extensive resonance overlap, compounded by the presence of rotamers; however, based on the crystal structure of spiroacetal **7** (Figure 2) and literature precedent,^58^ we postulate that the major diastereoisomer is the double anomerically stabilized spiroacetal **55a**, in which the benzyl group occupies an equatorial orientation (Scheme 7).^62^ Subjecting a 3:2 diastereoisomeric mixture of doubly substituted bis-morpholine spiroacetal **56** (see the Supporting Information) to 3 M HCl in MeOH at room temperature delivered a 19:1 (by LCMS) ratio of products after 22 h; this ratio did not change upon extension of the reaction time. We again hypothesize the major diastereoisomer is **56a**, which benefits from double anomeric stabilization and now two substituents adopting equatorial positions;^63^ this result suggests that in some instances (especially matched cases), it should be possible to access highly diastereoisomerically enriched products by anomerization.

## Conclusions

Morpholines are ubiquitous in drug discovery. In this study, we report a four-step synthesis of a scaffold containing two such heterocycles embedded within a spiroacetal framework. The synthesis involves the intermediacy of a 2-chloromethyl-substituted morpholine, which undergoes elimination to afford an exocyclic enol ether, from which the second morpholine ring is constructed in two steps. The overall synthesis is high-yielding and can be performed on a large scale from readily available starting materials. The method can be extended to the generation of 6,7- and 7,7-spiroacetal analogues and to substituted 6,6-systems. Substitution introduces issues of diastereoselectivity, which in some instances can be improved by acid-mediated anomerization. The two amine functionalities embedded in the 6,6- and 6,7-spiroacetal scaffolds can be sequentially functionalized, providing entry into compound libraries that occupy drug-like chemical space. These spiroacetals are hydrolytically stable and, therefore, represent attractive starting materials for drug discovery.

## Data Availability

A preprint of this article is available on ChemRxiv.^64^ The data underlying this study are available in the published article, in its Supporting Information, and openly available in UBIRA, the University of Birmingham’s eData repository at 10.25500/edata.bham.00001159.

## Supporting Information Available

Synthesis of the Stoltz cross-coupling precursors and formation of the corresponding 2-methylidenemorpholines, attempted synthesis of 2-iodomethylmorpholines by iodoetherification routes, significance of solvent choice for hydrogenolysis of carbamate **8**, Tanimoto dissimilarity comparison of the spiro-bis-morpholine library with small-molecule FDA-approved drugs, optimization of the synthesis of 6,7-spiroacetal **19**, synthesis of 7,6-spiroacetal **23** and attempted synthesis of larger ring systems, synthesis of spiroacetals **55** and **56**, discussion of a mechanism for the formation of methyl-substituted 2-chloromethylmorpholines **32** and **36**, discussion of the diastereoselectivity of the iodoacetalization of methyl-substituted 2-methylidenemorpholines **34** and **39**, general experimental details, experimental procedures and complete compound characterization data, NMR data for all compounds, X-ray crystallographic data for spiroacetals **7** and **23**, library enumeration including KNIME workflows, and library analysis and selection of diversity subset for physical library synthesis (PDF)
NMR spectra (PDF)

## Abbreviations

HATU: hexafluorophosphate azabenzotriazole tetramethyl uronium
KNIME: Konstanz Information Miner
LCMS: liquid chromatography–mass spectrometry
NK-1: neurokinin-1.
