# Supplementary material for: Short Scalable Route to Bis-morpholine Spiroacetals and Oxazepane Analogues: Useful 3D-Scaffolds for Compound Library Assembly
Source: J Org Chem. 2025 Feb 10;90(7):2652–61. doi: 10.1021/acs.joc.4c02690 (PMC11852203; doi:10.1021/acs.joc.4c02690)
Supplement: Supplementary file 1 — jo4c02690_si_001.pdf [file jo4c02690_si_001.pdf]

A short scalable route to bis-morpholine spiroacetals and oxazepane analogues: useful 3D-scaffolds for compound library assembly

Supporting Information

Daniel Kovari,[a,c] Louise Male,[a] Kimberley A. Roper,[b] Christian P. Mang,[c] Oliver Kunz,[c] Liam R. Cox[a,*]

[a]School of Chemistry, The University of Birmingham, Edgbaston, Birmingham, B15 2TT, United Kingdom, [b]School of Pharmacy, The University of Birmingham, Edgbaston, Birmingham, B15 2TT, United Kingdom, [c]Analyticon Discovery GmbH, Hermannswerder 17, 14473, Potsdam, Germany

Contents

1 Additional Chemistry Discussion S3

1.1 Synthesis of Stoltz cross-coupling precursors S3

1.2 Stoltz cross-coupling reactions S4

1.3 Attempted iodomethylmorpholine formation S5

1.4 Importance of solvent choice for hydrogenolysis of Cbz-Carbamate S6

1.5 Comparison of the spiro-bis-morpholine library with small-molecule FDA-approved drugs S7

1.6 Formation of 6,7-spiroacetal S8

1.7 Attempted synthesis of larger ring systems – side-products S10

1.8 Formation of 7- and 8-membered ring exocyclic enol ethers S12

1.9 7,6-Spiroacetal **23** S12

1.10 Synthesis of Spiroacetals **55** and **56** S13

1.11 Anomerization of monosubstituted 6,6-spiroacetal S15

1.12 Anomerization of disubstituted 6,6-spiroacetal S16

1.13 Summary of compound library synthesis S17

1.14 Formation of 5-methyl-2-methylidenemorpholine **36** – a possible mechanism for the formation of 6-regioisomer **32** from aminoalcohol **35** S17

1.15 Diastereoselectivity of the iodoacetalization of methyl-substituted 2-methylidenemorpholines **34** and **39** S20

2 Experimental Section S24

2.1 General Experimental S24

2.2 Stoltz chemistry S31

2.3 Aminoalcohols S43

2.4 Chloromethyl-substituted heterocycles S45

2.5 Enol ethers S49

2.6 Spiroacetals S53

2.7 Library compounds S85

3 Library Enumeration S139

3.1 Virtual library enumeration and selection of compounds for physical synthesis S139

3.2 Library synthesis results S153

3.3 Library comparisons with FDA-approved drugs S167

3.4 KNIME S168

3.5 DataWarrior methods S193

4 Crystal structures S194

5 References S211

1 Additional Chemistry Discussion

1.1 Synthesis of Stoltz cross-coupling precursors

The synthesis of the Stoltz cross-coupling precursors is summarized in Scheme S1. Bromide **S1** was synthesized in three steps on multigram scale from *trans*-cinnamaldehyde (**S2**):[1] thus, reaction with iodine monochloride afforded vinyliodide **S3** in quantitative yield. Attempted reductive amination [$NaBH(OAc)_3$, AcOH, CH_2Cl_2, rt] of **S3** with *N*-methylethanolamine, resulted in the formation of many products; the desired product was not observed.[a] Instead, Luche reduction provided allylic alcohol **S4**, from which bromide **S1** was obtained via an Appel reaction. Chemoselective *N*-alkylation of **S1** with ethanolamine and its *N*-methyl and *N*-benzyl analogues, afforded the corresponding iodoalcohols **S5**, **S6** and **S7**. Reaction of **S7** with Boc_2O provided Boc-amide **S8**, and with TsCl provided sulfonamide **S9**.

Scheme S1. Synthesis of Stoltz cross-coupling precursors S5–S9

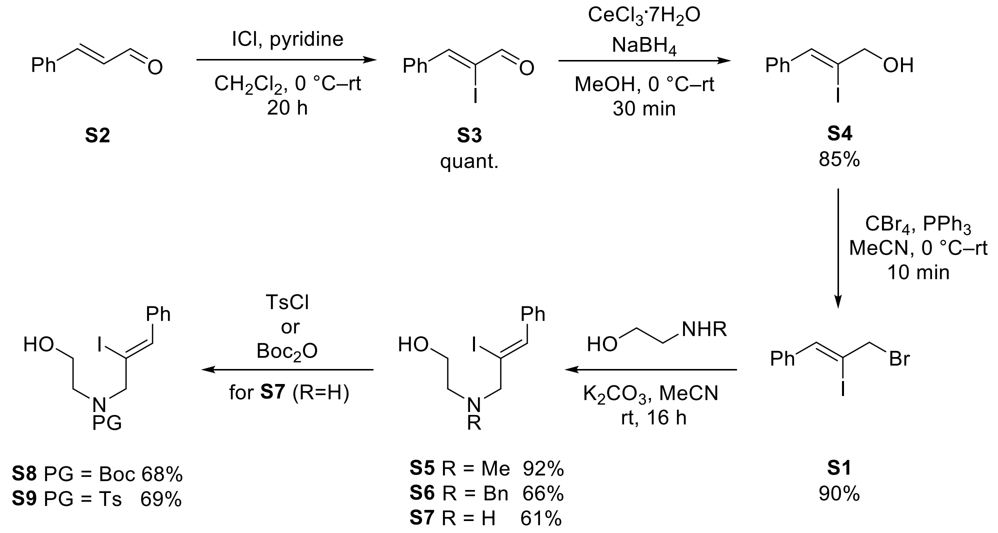

The synthesis of iodoalcohol **S10** started with regioselective hydroiodination of propargyl alcohol (**S11**) to afford iodoalcohol **S12** (Scheme S2).[2] Owing to its low boiling point (50 °C at 7 mbar),[3] **S12** was directly tosylated without purification[4] to provide tosylate **S13**, which reacted with *N*-benzylethanolamine (**2**) to provide Stoltz cross-coupling precursor **S10** in good yield.[5]

[a] *trans*-Cinnamaldehyde underwent successful reductive amination with 2-(methylamino)ethanol; the 3° amine product was not isolated, but was positively identified in the crude reaction mixture by ^{1}H- and ^{13}C{^{1}H}-NMR spectroscopy, where data were in accordance with those reported in the literature. Reddy, K. A.; Bhushan, L. V.; Reddy, A. S.; Harikishore, P.; Rajagopalan, R.; Rao, C. S. Compounds Having Antidiabetic, Hypolipidemic, Antihypertensive Properties, Process for Their Preparation and Pharmaceutical Compositions Containing Them. US005925656A, 1999.

Scheme S2. Synthesis of iodoalcohol S10

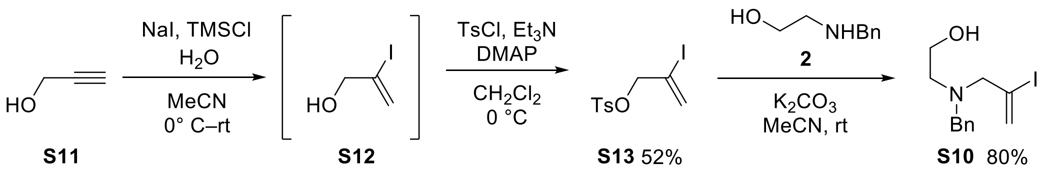

1.2 Stoltz cross-coupling reactions

The results of the cross-coupling reaction are summarized in Table S1. The reaction was first validated with N-methyl-substituted iodo-aminoalcohol **S5**. Stoltz reported the formation of the corresponding 2-methylidenemorpholine **S14** in 86% yield after 24 h;[5] in our hands, full consumption of starting material was observed in less than 30 min and 2-methylidenemorpholine **S14** was isolated in 76% yield. Iodo-aminoalcohol **S6** reacted similarly, affording N-benzyl-protected 2-methylidenemorpholine **S15** in excellent yield. Both Boc-protected aminoalcohol **S8** and tosyl analogue **S9** failed to react; neither substrate showed any conversion after 24 h at room temperature nor after heating the reaction mixtures for an additional 24 h at 60 °C.[b] Secondary amine **S7** was therefore targeted. While the reaction time proved variable (1.5–24 h), clean cyclization of **S7** afforded 2-methylidenemorpholine **S16**.[c] Subsequent protection of the embedded secondary amine as its Boc carbamate **S17** and tosyl sulfonamide **S18** proceeded without event. The successful application of a secondary amine substrate (which allows post-coupling derivatization) usefully extends the scope of this methodology.[d] Finally, Stoltz reported the formation of target molecule **4** from iodoalcohol **S10** in 40% yield; however, we were only able to achieve 20% conversion at best.[e]

[b] We tentatively propose that the electron-withdrawing nature of the sulfonamide and carbamate groups in the chain – and not sterics – suppresses the cross-coupling reaction, possibly by reducing the nucleophilicity of the alcohol, rendering formation of the Ni-alkoxide intermediate more difficult.
[c] No obvious trend was observed, which would correlate with the varied reaction time.
[d] Stoltz reported the cyclization of tertiary amine substrates. See ref. 5.
[e] As determined by analysis of the reaction mixture by ^{1}H-NMR spectroscopy.

Table S1. Ni-catalyzed C–O cross-coupling approach to 2-methylidenemorpholines

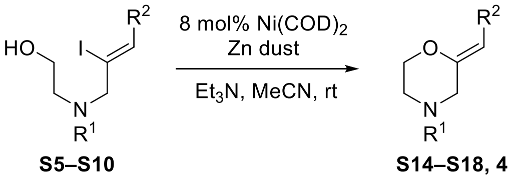

iodo-alcohol	R^1	R^2	2-methylidene-morpholine	% yield[a]
**S5**	Me	Ph	**S14**	76 (86)[b]
**S6**	Bn	Ph	**S15**	83
**S7**	H	Ph	**S16**	61[c]
**S8**	Boc	Ph	**S17**	no reaction
**S9**	Ts	Ph	**S18**	no reaction
**S10**	Bn	H	**4**	20[d] (40)[b]

[a]Isolated yield after column chromatography, [b]Yields in parentheses are those reported by Stoltz, see ref. 5. [c]The combined product from two reactions (using 50 mg and 250 mg of substrate **S7**) was purified once by column chromatography to afford **S16** in 61% combined yield; however, ^{1}H-NMR spectroscopic analysis of the crude reaction mixture revealed just the presence of product **S16**. [d]Conversion as determined by ^{1}H-NMR spectroscopic analysis of the reaction mixture.

1.3 Attempted iodomethylmorpholine formation

Chemoselective allylation of *N*-benzylethanolamine (**2**) afforded alkenol **S19** in 92% yield (Scheme S3).

Scheme S3: Chemoselective allylation of *N*-benzylethanolamine

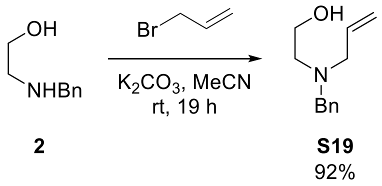

Iodoetherification was attempted on alkenol **S19** using conditions described by Bera and Panda,[6] Doveston *et al.*[7] and Eli Lilly[8]; however, in all three cases, decomposition of the starting alkenol **S19** or the formation of complex mixtures of unidentified products was observed (Table S2).

Table S2: Attempted iodoetherification of S19

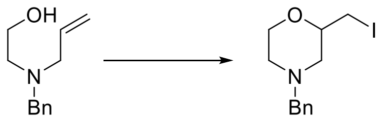

S19

temp. (°C)	iodine source (1.5 eq)	solvent[a]	observations
60	I_2	THF	mixture of unidentified products
23	I_2	MTBE/$NaHCO_{3(aq)}$ (1/4 mixture)	mixture of unidentified products
60	NIS	MeCN	decomposition of alkenol starting material

[a]Reaction concentration 0.2 mmol mL^{-1}.

1.4 Importance of solvent choice for hydrogenolysis of Cbz-Carbamate 8

Hydrogenolysis of Cbz-carbamate **8** on 0.5 g scale using H_2 / Pd / C in MeOH solvent led to a by-product, which was identified as methylamine **S20**. The formation of methylamine products in Pd-catalyzed reduction reactions has been reported,[9] and is proposed to arise from the oxidation of MeOH to formaldehyde. In our case, condensation with the 2° amine hydrogenolysis product **9** presumably affords iminium species **S21** and thence methylamine **S20** upon reduction (Scheme S4). Changing the solvent from MeOH to a THF/H_2O (4:1) mixture eliminated this by-product.

Scheme S4. Methylamine formation from hydrogenolysis of Cbz-carbamate 8 using MeOH as solvent

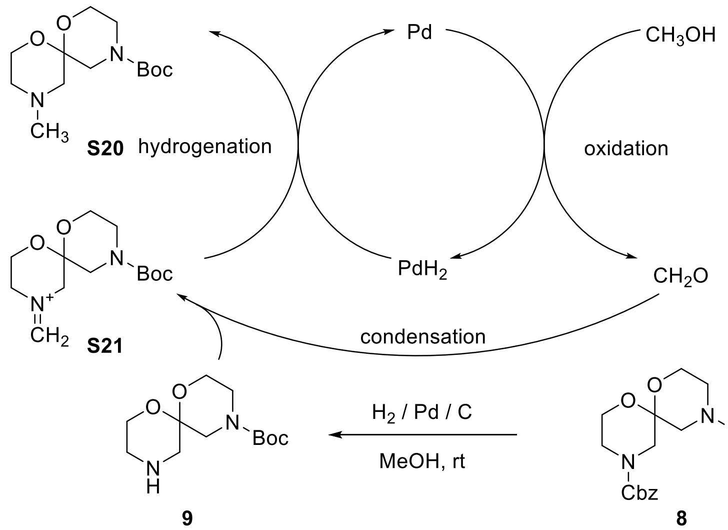

1.5 Comparison of the spiro-bis-morpholine library with small-molecule FDA-approved drugs

To evaluate the dissimilarity[10] of our compound library from the set of small-molecule FDA-approved drugs,[f] each compound in both libraries was translated into its Morgan 2 (an Extended Connectivity Fingerprint (ECFP_4) like) fingerprint using the KNIME platform (see Section 3.4.5); this allowed the calculation of Tanimoto coefficients. Tanimoto coefficients range from 0 to 1. Scores closer to 1 represent higher fingerprint similarity.

Tanimoto coefficient, T, is calculated according to the following equation:

$$T = [c / (a + b - c)]$$

where: a = the number of bits contained in the fingerprint of molecule A; b = the number of bits contained in the fingerprint of molecule B; c = the number of bits contained in the fingerprints of both molecules A and B.

The calculated Tanimoto scores are represented in histogram form in Figure S1; scores of 0.2–0.4 indicate significant dissimilarity between compounds in our library and those on the FDA list.

[f] This filtering of the FDA collection removed biologics and other macromolecular therapeutics, which frequently have different modes of administration. The collection was exported from CDD vault on 05.05.2022 based on the following public collections “FDA Approved: Tox”, “FDA Approved: Approved drugs” and “FDA Approved: Orphan drugs”. Further information about these collections can be found at: https://www.collaborativedrug.com/public-access/. Date of access: 05.05.2022.

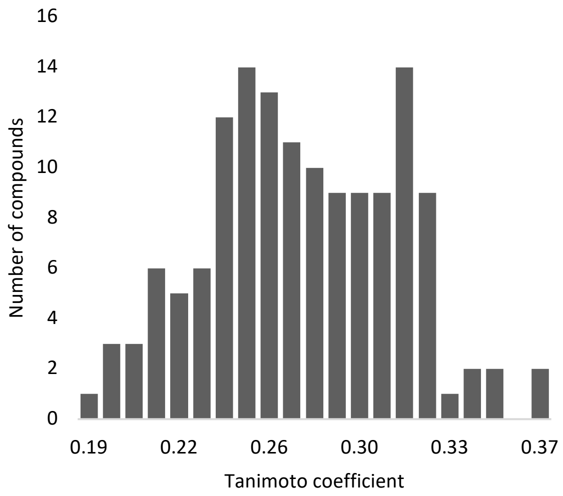

Figure S1. Histogram of Tanimoto coefficients calculated using Morgan 2 fingerprints, comparing the spiro-bis-morpholine physical library with small-molecule drugs approved by the FDA.

1.6 Formation of 6,7-spiroacetal 19

Iodoacetalization of 2-methylidenemorpholine **4** using *N*-Boc-propanolamine proceeded uneventfully to provide iodide **S22** in 79% yield. Subsequent cyclization to 1,4-oxazepane **19** required optimization as the slow rate of reaction under the conditions used successfully to access 6,6-spiroacetal **7**, resulted in the formation of urea **18**, which co-eluted with the target product. The effects of base, reaction temperature and solvent were therefore assessed in a qualitative study, analyzing reactions by LCMS (Table S3). The best results were achieved using *t*-BuOK in DMF at room temperature (Table S3, entry 10).

Table S3: Iodoacetalization of 2-methylidenemorpholine 4 with *N*-Boc-propanolamine and optimization of the subsequent ring closure to oxazepane 19

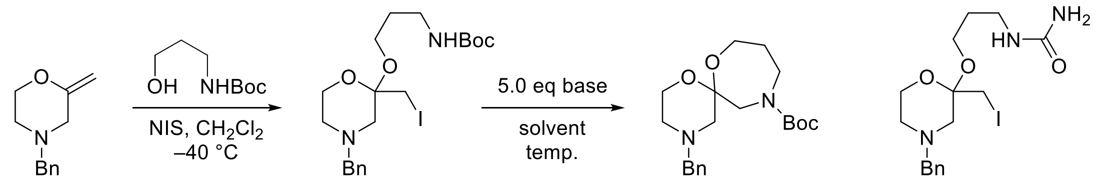

4 **S22** 79% **19** **18**

entry[a]	temp. and time	base	solvent[b]	observations
1	rt (5 days), then 90 °C (1 day)	Ag_2CO_3	MeCN	decomposition of iodide **S22**
2	rt (5 days), then 90 °C (1 day)	K_2CO_3	MeCN	low conversion[c] to oxazepane **19**
3	rt (5 days), then 90 °C (1 day)	Cs_2CO_3	MeCN	low conversion[c] to oxazepane **19**
4	rt (1 day), then 90 °C (1 day)	Ag_2CO_3	DMF	decomposition of iodide **S22**
5	rt (1 day), then 90 °C (1 day)	K_2CO_3	DMF	low conversion[c] to oxazepane **19**
6	rt (1 day), then 90 °C (1 day)	Cs_2CO_3	DMF	consumption of iodide **S22**, ~1:1[d] oxazepane **19** : urea **18**
7	rt (5 days)	NaH	THF	decomposition of iodide **S22**
8	rt (5 days)	*t*-BuOK	THF	decomposition of iodide **S22**
9	rt (2 days)	NaH	DMF	consumption of iodide **S22**, ~1:1[d] oxazepane **19** : urea **18**
10	rt (1 day)	*t*-BuOK	DMF	consumption of iodide **S22**, ~7:3[d] oxazepane **19** : urea **18**

[a]Reactions were performed on 50 mg scale. [b]Reaction concentration: 0.05 mmol mL^{-1}. [c] <25% by LCMS. [d]Ratio by LCMS.

Performing the cyclization in DMF at higher reaction concentrations and using different stoichiometries of *t*-BuOK led to further improvements (Table S4). The best results were obtained using 2.0 equivalents of *t*-BuOK and a reaction concentration of 0.05 or 0.20 mmol mL^{-1} (Table S4, entries 1, 5). Mindful of a future need to scale-up this reaction, performing the cyclization at the higher concentration on a 0.5 g scale afforded analytically pure 6,7-spiroacetal **19** in 70% yield, without the need for purification.

Table S4: Effect of reaction concentration and base stoichiometry on the cyclization of iodide S22

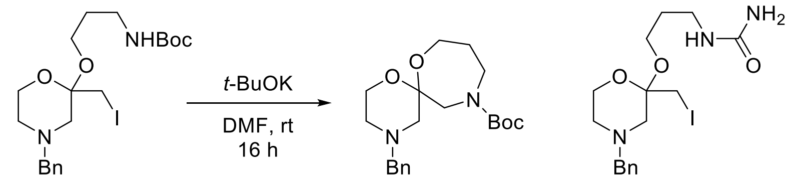

S22 **19** **18**

entry[a]	reaction concentration (mmol mL^{-1})	*t*-BuOK (eq)	observations
1	0.05	2.0	consumption of iodide **S22**, formation of oxazepane **19,** trace urea side-product **18**
2	0.10	2.0	incomplete consumption of iodide **S22**
3	0.15	2.0	incomplete consumption of iodide **S22**
4	0.20	2.0	consumption of iodide **S22**, formation of oxazepane **19**, trace urea side-product **18**
5	0.15	1.5	incomplete consumption of iodide **S22**
6	0.15	3.0	incomplete consumption of iodide **S22**
7	0.15	4.0	incomplete consumption of iodide **S22**

[a]Reactions were performed on 100 mg scale.

1.7 Attempted synthesis of larger ring systems – side-products

Efforts to synthesize 6,8- and 6,9-spiroacetals failed. While the desired iodoacetal cyclization precursors, **S23** and **S24**, were formed uneventfully,[g] their attempted cyclization led in both cases to

[g] The reported yields are estimates, given the compounds were treated as intermediates and after purification were only analyzed by LCMS. The LCMS data of the products did not show any major impurities.

products arising from degradation of the Boc-carbamate. Urea and 1° amine side-products were observed by LCMS analysis of the reaction mixtures, and in the case of urea **S25** and 1° amine **S26**, isolated and characterized by NMR spectroscopy (see experimental). A urea product (**S27**) was also observed as a side-product in the formation of 7,7-spiroacetal **24** (see experimental).

Scheme S5. Attempted synthesis of 6,8- and 6,9-spiroacetals

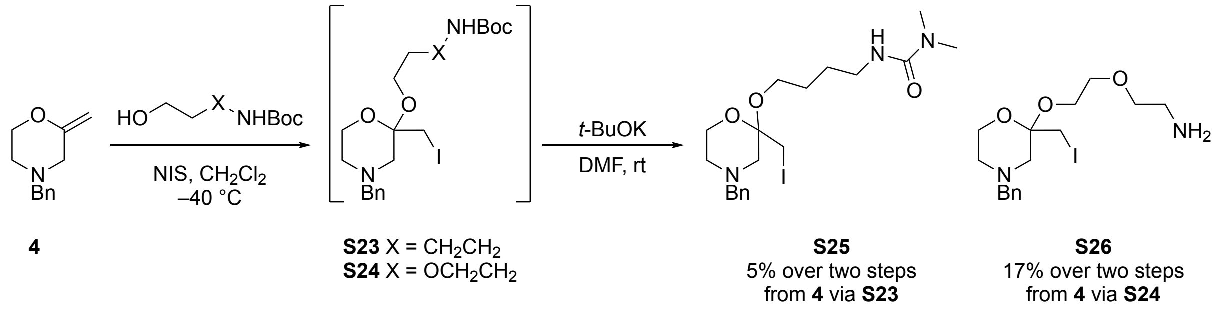

The formation of these side-products can be rationalized through base-mediated elimination of *t*-BuOH from the Boc-carbamate and formation of the corresponding isocyanate **S28** (Scheme S6);[11] trapping with dimethylamine (a decomposition product of DMF under basic conditions[12]) would provide urea **S29**, while reaction with adventitious H_2O would afford the corresponding carbamic acid, which undergoes decarboxylation to afford 1° amine **S30**. While we did not investigate these substrates further, we hypothesize that formation of the isocyanate should be rendered reversible (therefore representing a redundant pathway) by performing the reaction in *t*-BuOH under rigorously anhydrous conditions. In the absence of an external nucleophile, slow cyclization of the deprotonated carbamate should deliver the target products.

Scheme S6. Proposed mechanism of side-product formation

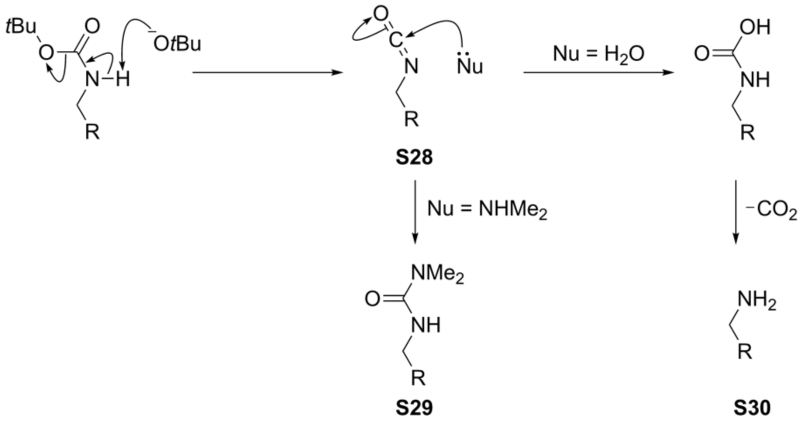

1.8 Formation of 7- and 8-membered ring exocyclic enol ethers

Formation of 2-methylidene-1,4-oxazepane **21** began with the reaction of commercially available *N*-benzylaminopropanol **S31** with epichlorohydrin under our standard conditions. Without purification, 2-chloromethyl-substituted 1,4-oxazepane **S32** underwent dehydrochlorination to furnish 2-methylidene-1,4-oxazepane **21** in 44% yield. Attempted formation of 2-methylidene-1,5-oxazocine began with condensation of benzaldehyde with 4-aminobutanol (**S33**) to provide the corresponding imine, which, without isolation, was reduced with $NaBH_4$ to provide 2° amine **S34** in quantitative yield. Attempted formation of 2-chloromethyl-substituted 1,5-oxazocine **S35** was unsuccessful: based on TLC analysis, the first step (reaction with epichlorohydrin) proceeded with complete consumption of *N*-benzylaminobutanol **S34**; however, attempted ring closure of the intermediate diol led to an intractable tar after 4 h; the corresponding 2-chloromethyl-substituted 1,5-oxazocine 1,5-oxazocine **S35** was not observed by LCMS analysis of the reaction mixture and therefore this substrate was not investigated further.

Scheme S7. Formation of larger ring exocyclic enol ethers

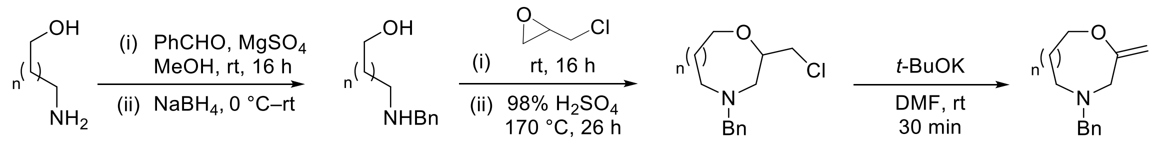

S33 n = 2

S31 n = 1
S34 n = 2, quant

S32 n = 1 51%
S35 n = 2 not observed

21 n = 1, 44%

1.9 7,6-Spiroacetal 23

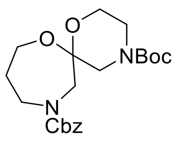

23

Stability studies: A solution of spiroacetal **23** in DMSO was monitored by NMR spectroscopy over 3 weeks: as there was no change in the ^{1}H-NMR spectrum or the ^{13}C{^{1}H}-NMR spectrum of **23** over this period, this scaffold was deemed suitable for future compound library synthesis.

Structural studies: Spiroacetal **23** crystallized from the *n*-heptane/EtOAc eluent mixture used for its purification, allowing analysis by single-crystal X-ray crystallography (Figure S2). The crystal structure reveals the six-membered ring assumes a chair conformation and the seven-membered ring a twist-chair conformation while the spiroacetal benefits from double anomeric stabilization, similar to the

6,6 spiroacetal analogue **7** (see Figure 2 in the article). The C(spiro)–O bond lengths of 1.429 (2) Å for the morpholine and 1.414 (2) Å for the 1,4-oxazepane are comparable with those in bis-morpholine spiroacetal **7** [1.4220 (16) Å, 1.4234 (16) Å in molecule 1, and 1.4229 (16) Å and 1.4237 (16) Å in molecule 2 of the asymmetric unit, mean C(spiro)–O bond length: 1.4230 Å] and other axially *O*-substituted 1,3-dioxanes.[13] The O–C(spiro)–O bond angle is 111.04 (15)°, again similar to the O–C(spiro)–O bond angle in 6,6-spiroacetal **7** [110.79 (11)° for molecule 1 and 110.91 (11)° for molecule 2 of the asymmetric unit, mean angle: 110.85 °].

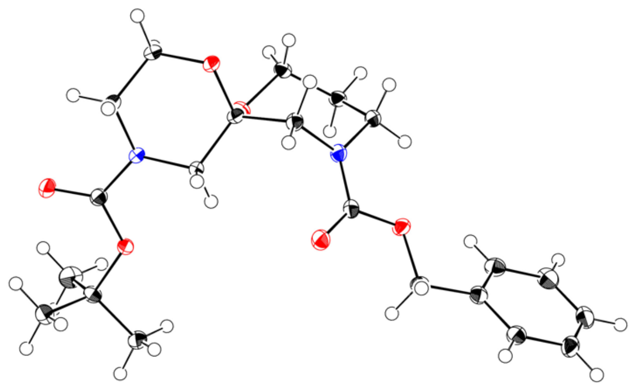

Figure S2. ORTEP plot of **23** with ellipsoids drawn at the 50% probability level, confirming the double anomeric stabilization of the spiroacetal. Atomic displacement parameters at 100 K.

1.10 Synthesis of Spiroacetals 55 and 56

Monosubstituted spiroacetal 55: The synthesis of spiroacetal **55** from spiroacetal **48** is summarized in Scheme S8. Treatment of benzylamine **48** (1:1 mixture of diastereoisomers as determined by LCMS analysis) with CbzCl effected protecting group exchange to provide Cbz-carbamate **S36** (d.r. 1:1) in excellent yield. Subsequent acid-mediated deprotection of the Boc group in **S36** afforded amine HCl salt **S37·HCl** (d.r. 1:1) which, without purification, was treated with FmocCl to provide Fmoc-carbamate **55** as a 1:1 mixture of diastereoisomers based on LCMS analysis.

Scheme S8. Formation of monosubstituted spiroacetal 55

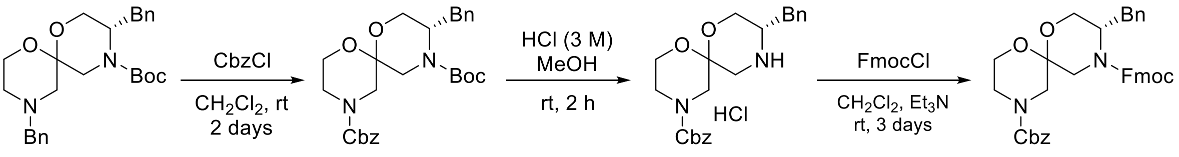

48 d.r. 1:1 **S36** d.r. 1:1, 99% **S37·HCl** d.r. 1:1, quant. **55** d.r. 1:1, 60%

Disubstituted Spiroacetal 56: The synthesis of spiroacetal **56** from 2-methylidenemorpholine **39** is summarized in Scheme S9. Note, the absolute stereochemistry in the two building blocks was chosen to deliver the 'matched' diastereoisomer upon anomerization, i.e., a doubly anomerically stabilized spiroacetal in which both ring substituents occupy equatorial positions. Iodoacetalization of (*S*)-5-methyl-substituted enol ether **39** with *tert*-butyl (*S*)-(1-hydroxy-3-phenylpropan-2-yl)carbamate delivered the intermediate iodide as a 1:1 mixture of diastereoisomers (determined by ^{1}H-NMR spectroscopy). Subsequent cyclization under standard conditions provided spiroacetal **54** as a 1:1 mixture of diastereoisomers (determined by ^{1}H-NMR spectroscopy). The presence of the methyl substituent in spiroacetal **54** significantly reduced the rate of reaction with CbzCl; indeed, we were unable to drive the reaction to completion. As the Cbz-carbamate product was inseparable from the starting material, we instead exchanged first the Boc group in spiroacetal **54** for an Fmoc group; thus, Boc deprotection of spiroacetal **54** and subsequent Fmoc re-protection of 2° amine **S38·HCl** provided Fmoc-carbamate **S39**[h] (Scheme S9). Finally, the benzyl group in spiroacetal **S39** was exchanged for a Cbz carbamate. While this reaction again proved very slow (starting material remained after 5 days), it was now possible to isolate a small sample of bis-carbamate **56** as a 3:2 mixture of diastereoisomers, sufficient for anomerization studies.

Scheme S9. Formation of disubstituted spiroacetal 56

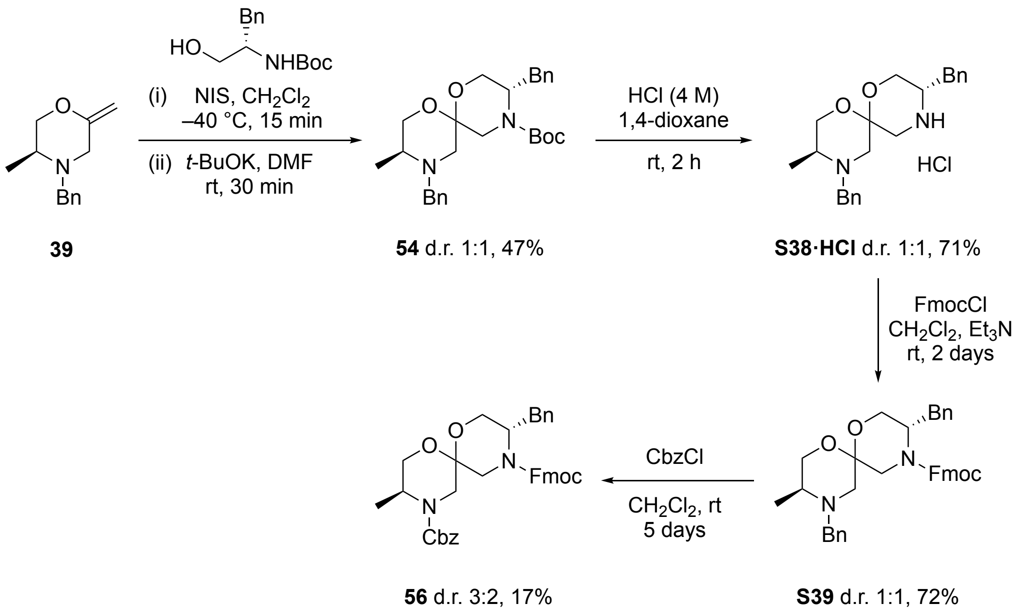

[h] Attempted anomerization of carbamate **S39** with 3 M HCl in MeOH at room temperature was unsuccessful; after four days, no change was observed in the diastereoisomeric ratio. Presumably, protonation of the benzylamine to afford the corresponding quaternary ammonium salt, suppresses anomerization.

1.11 Anomerization of monosubstituted 6,6-spiroacetal 55

A 1:1 diastereoisomeric mixture of **55** was treated with 3 м HCl in MeOH. After 24 h at room temperature, the ratio had increased to 9:1 as determined by LC-MS analysis of the reaction mixture.[i] This ratio did not change over the course of one week, suggesting the reaction had reached equilibrium (Scheme S10).

Scheme S10. Anomerization of spiroacetal 55

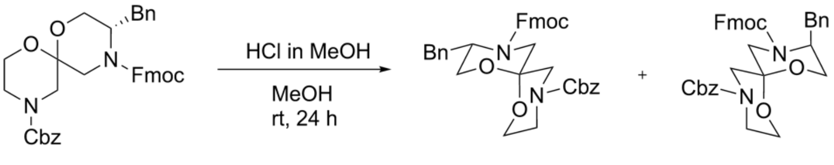

55 d.r. 1:1 **55a** d.r. 9:1 **55b**

LCMS data:

Diastereoisomeric ratio of the starting material (**55**):

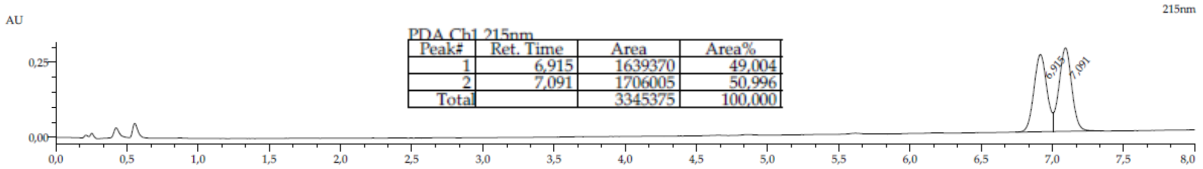

Peak#	Ret. Time	Area	Area%
1	6,915	1639370	49,004
2	7,091	1706005	50,996
Total		3345375	100,000

Diastereoisomeric ratio after 1 day:

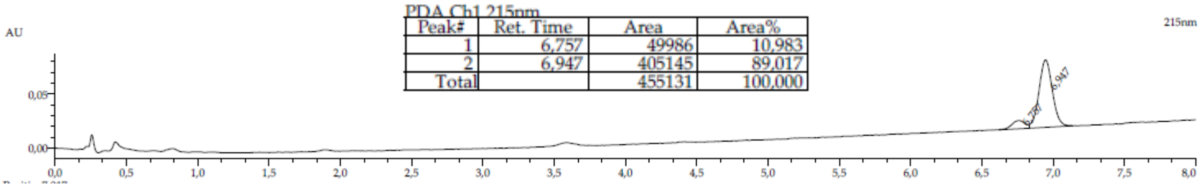

Peak#	Ret. Time	Area	Area%
1	6,757	49986	10,983
2	6,947	405145	89,017
Total		455131	100,000

Diastereoisomeric ratio after 6 days:

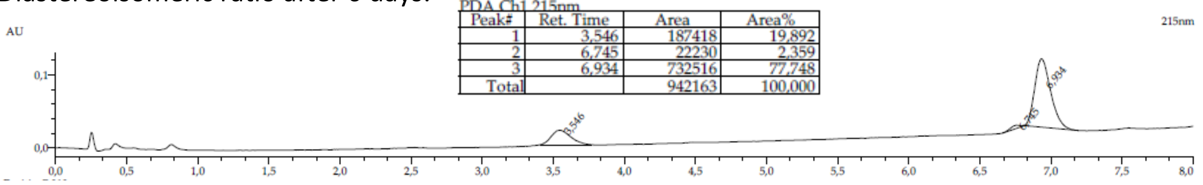

Peak#	Ret. Time	Area	Area%
1	3,546	187418	19,892
2	6,745	22230	2,359
3	6,934	732516	77,748
Total		942163	100,000

The peak at 3.6 min is an unidentified compound, which we postulate may result from decomposition of the spiroacetal over the extended reaction time.

[i] There was no evidence for preferential decomposition of one diastereoisomer.

1.12 Anomerization of disubstituted 6,6-spiroacetal 56

A 3:2 diastereoisomeric mixture of **56** was treated with 3 M HCl in MeOH at room temperature. After 22 h, the diastereoisomeric ratio was 19:1 as determined by LC-MS analysis of the reaction mixture; this ratio did not change upon extending the reaction time (Scheme S11).

Scheme S11. Anomerization of spiroacetal 56

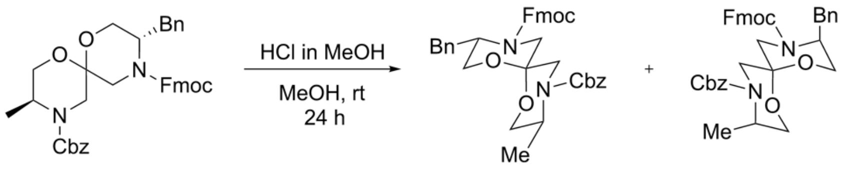

56 d.r. 3:2 **56a** d.r. 19:1 **56b**

LCMS data:

Diastereoisomeric ratio of the starting material (**56**):

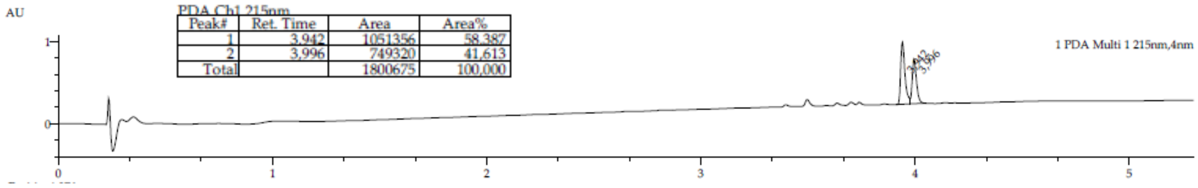

Diastereoisomeric ratio after 1 day:

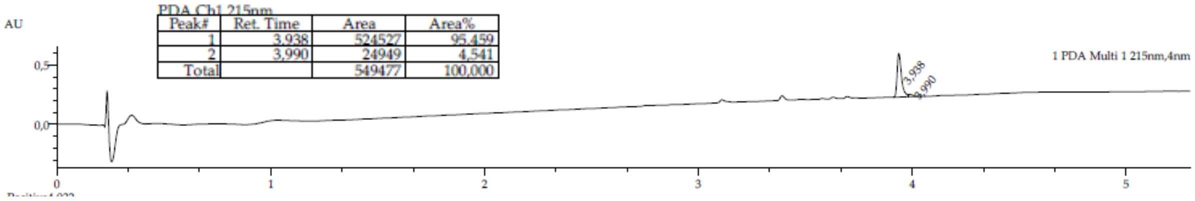

Diastereoisomeric ratio after 13 days:

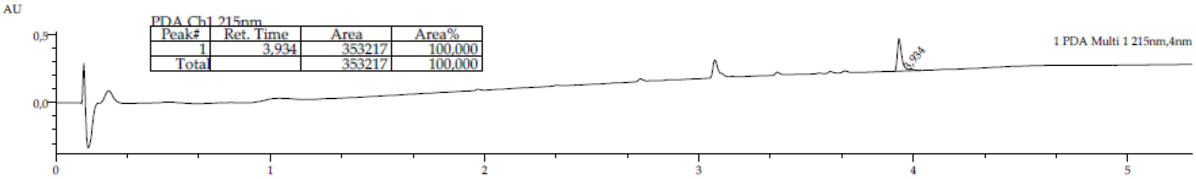

The peak at 3.1 min is an unidentified compound, which we postulate may result from decomposition of the spiroacetal over the extended reaction time.

1.13 Summary of compound library synthesis

A reaction was deemed successful when the library compound was isolated in >10 mg and >90% purity (by LCMS, evaporative light scattering detection). Of the 124 reactions performed, only one failed to deliver any product while eleven reductive amination products were isolated in less than 10 mg. Four products did not satisfy our purity threshold but were deemed of sufficient purity (>80%) for inclusion (with a flag) in the library. These results translate to a 90% success rate for compound library synthesis. Table S5 summarizes the composition of the assembled compound library.

Table S5. Composition of compound library synthesized from spiro-bis-morpholine 8

	R^2 = amide	R^2 = sulfonamide	R^2 = urea	R^2 = amine
R^1 = amide	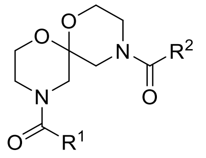 28 examples (56%)[a]	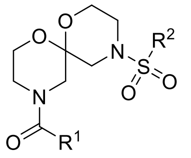 6 examples (66%)[a]	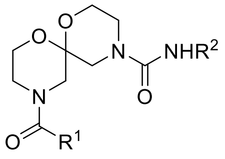 3 examples (53%)[a]	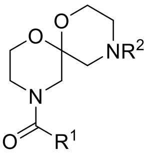 27 examples (58%)[a]
R^1 = sulfonamide	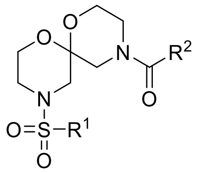 7 examples (55%)[a]	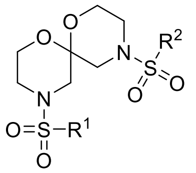 2 examples (54%)[a]	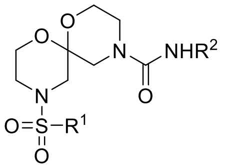 1 example 69%	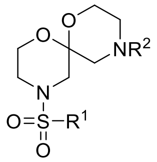 11 examples (56%)[a]
R^1 = urea	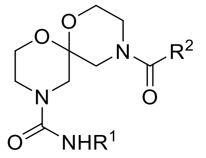 10 examples (51%)[a]	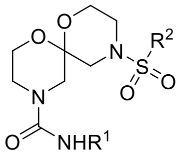 3 examples (54%)[a]	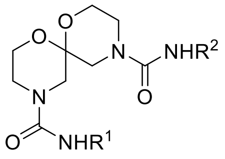 2 examples (61%)[a]	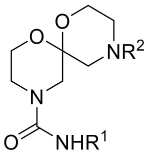 12 examples (48%)[a]

[a]Average isolated yields after HPLC purification, isolated yields for each library product are included in Section 3.2 of the Supporting Information.

1.14 Formation of 5-methyl-2-methylidenemorpholine 36 – a possible mechanism for the formation of 6-regioisomer 32 from aminoalcohol 35

Reaction of (*S*)-2-benzylaminopropan-1-ol (**35**) with *rac*-epichlorohydrin (**3**) in 98% H_2SO_4 (1.0 equiv) at 170 °C afforded 6-methyl regioisomer **32** in 74% yield as a ~2:1 mixture of diastereomers (Scheme S12):[j]

[j] The relative stereochemistry of the major diastereoisomer was assigned as 2,6-*cis* morpholine **32** as follows: assuming a chair conformation, a large (~10.5 Hz) vicinal coupling between C(2)H and C(3)H and between C(5)H and C(6)H in the ^{1}H-NMR spectrum (appearing as an apparent triplet) suggests axial hydrogen substituents; this is consistent with both substituents occupying equatorial positions.

Scheme S12: Formation of isomerized product 32

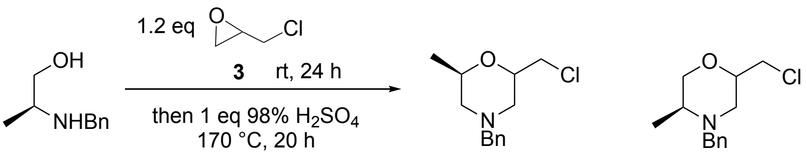

35 32 36
74% d.r. 2:1 not observed

LCMS analysis of Step 1, *i.e.*, reaction of **35** with **3**, revealed the formation of a 1:1 mixture of two chlorohydrin diastereoisomers **38** (Scheme S13):

Scheme S13: Formation of chlorohydrin intermediates from Step 1

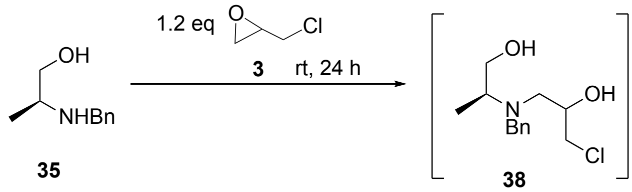

35 38
d.r. 1:1 (by LCMS)

This observation is consistent with enantiomerically pure **35** reacting with the racemic epoxide **3** and suggests the formation of the 6-methyl regioisomer **32** from **35** is occurring in Step 2 of the reaction.

While performing Step 2 step at 150 °C, 170 °C and 190 °C had no impact on the outcome, changing the stoichiometry of 98% H_2SO_4 did:

- 1.0 equiv. of acid → exclusive formation of the 6-methyl regioisomer **32**
- 2.0 equiv. of acid → both regioisomers observed: 5-regioisomer (**36**): 6-methyl regioisomer (**32**), 2:1
- 4.0 equiv. of acid → exclusive formation of 5-methyl regioisomer **36** (isolated in 62% yield, d.r. 7:5) (Scheme S14).[k]

[k] The absolute stereochemistry was not confirmed; however, we tentatively assign the stereochemistry shown in Scheme S14 based on the proposed mechanism, summarized in Scheme S15.

Scheme S14: Selective formation of 5-methyl regioisomer 36 in the presence of excess acid

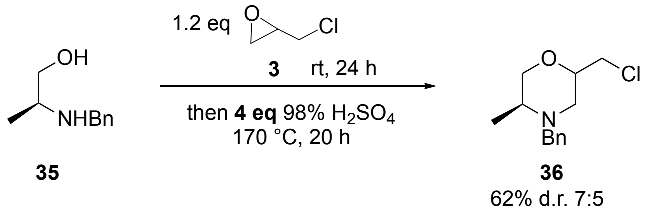

Possible mechanism

A tentative mechanism for the formation of 5- and 6-methyl-substituted 2-chloromethyl morpholines, **32** and **36**, including the formation of **32** from **38**, is summarized in Scheme S15, and is based on work from Cossy and co-workers,[14,15] who studied the rearrangement of β-amino alcohols via the intermediacy of aziridinium ions, notably in the presence of sulfuric acid.

Scheme S15. Formation of 5- and 6-methyl-substituted 2-chloromethyl morpholines and proposed mechanism for the formation of 32 from 35

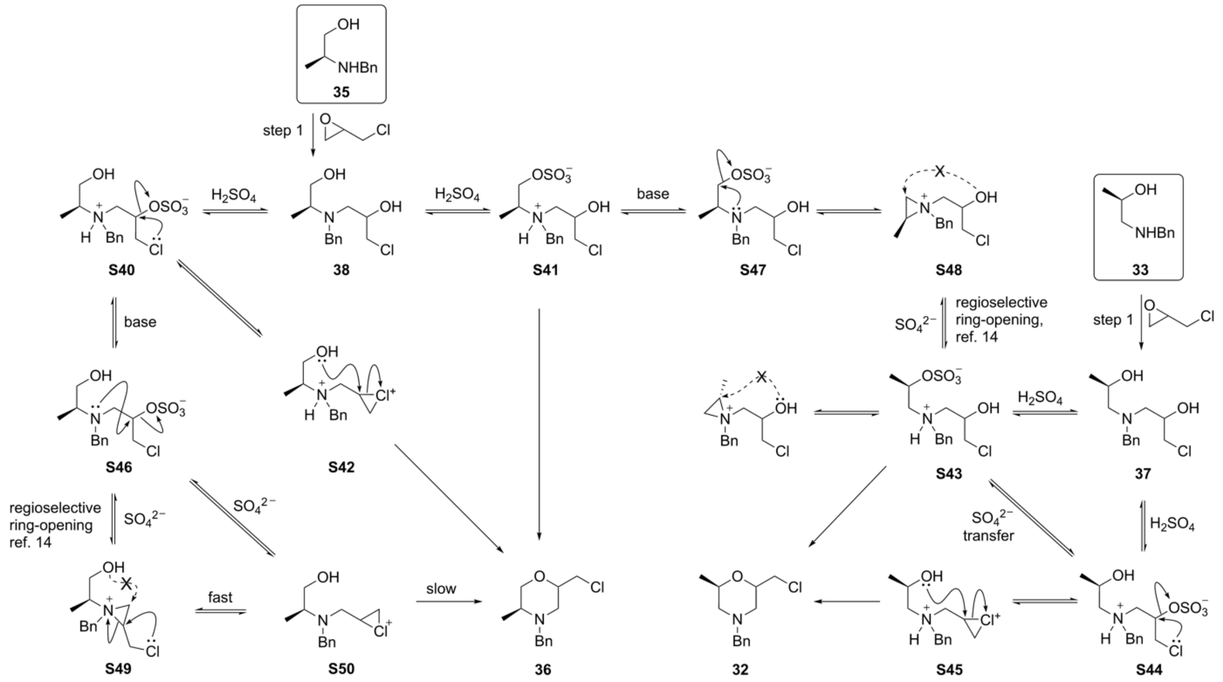

Focusing on the reaction of aminoalcohol **35**, chlorohydrin **38**, the product from step 1 of the reaction sequence, reacts with sulfuric acid in step 2 to provide the corresponding ammonium sulfates **S40** and **S41**. Both sulfates can cyclize to 5-methyl morpholine regioisomer **36**, in the case of 2° sulfate **S40**, potentially via the intermediacy of a chloronium ion **S42**. Similarly, chlorohydrin **37**, the product from

step 1 of the reaction sequence involving aminoalcohol **33**, reacts in step 2 with sulfuric acid to provide the corresponding ammonium sulfates **S43** and **S44**. Both sulfates can cyclize to 6-methyl morpholine regioisomer **32**,[l] in the case of 2° sulfate **S44**, potentially via the intermediacy of a chloronium ion **S45**. We propose these routes are possible pathways to **32** (from **37**) and **36** (from **38**) when the reaction is performed in the presence of an excess of sulfuric acid. Under these conditions, the amine in **37** and **38** is effectively permanently protonated, suppressing the formation of aziridinium ions (see below).

An alternative reaction pathway opens up when the reaction is performed with 1 equivalent of sulfuric acid. Now, when chlorohydrin **38** is treated with 1 equivalent of sulfuric acid, there is a shift in the equilibrium between the ammonium species **S40** and **S41** and their conjugate bases, amines **S46** and **S47**, respectively. The formation of tertiary amine **S47** leads to rapid cyclization to aziridinium species **S48**. Similarly, **S46** can form aziridinium species **S49**. Intramolecular ring-opening of aziridinium ion **S48** (and **S49**) by the pendant alcohol is disfavored on geometric grounds; however, Cossy and co-workers have reported the intermolecular reaction of aziridinium ions with sulfuric acid.[14] Notably, ring-opening by sulfate occurs regioselectively at the more substituted carbon. Thus, irrespective of the starting aminodiol (**37 or 38**), the formation of aziridinium species **S48** delivers the same ammonium sulfate species **S43** converging on morpholine **32**, either directly by intramolecular nucleophilic substitution,[m] or via sulfate transfer to afford ammonium sulfate **S44** which can be intercepted directly by the pendant alcohol to afford morpholine product **32** or react via chloronium ion **S45**.

Ammonium sulfate **S40** can react similarly via **S46** to provide aziridinium species **S49** and potentially chloronium ion **S50**. While interception of this chloronium ion by the pendant alcohol would afford 5-methyl regioisomer **36**, we postulate formation of the six-membered morpholine ring is slow relative to re-formation of the aziridinium species **S49** and the reverse reaction. In this way, **38** is channeled through aziridinium species **S48** and the isomerization pathway.

1.15 Diastereoselectivity of the iodoacetalization of methyl-substituted 2-methylidenemorpholines 34 and 39

Experimental observations: 5-Methyl-substituted 2-methylidenemorpholine **39** afforded a 1:1 mixture (by LCMS) of acetal diastereoisomers **40a** and **40b**; 6-methyl-substituted 2-methylidenemorpholine **34** afforded a 4:1 mixture (by LCMS) of acetal diastereoisomers **41a** and **41b** (major diastereoisomer not determined) (Scheme S16).

---

[l] Cyclization of **S43** via an S_N2 pathway would proceed with inversion of configuration, not shown in Scheme S15.

Scheme S16. Iodoacetalization of methyl-substituted 2-methylidenemorpholines

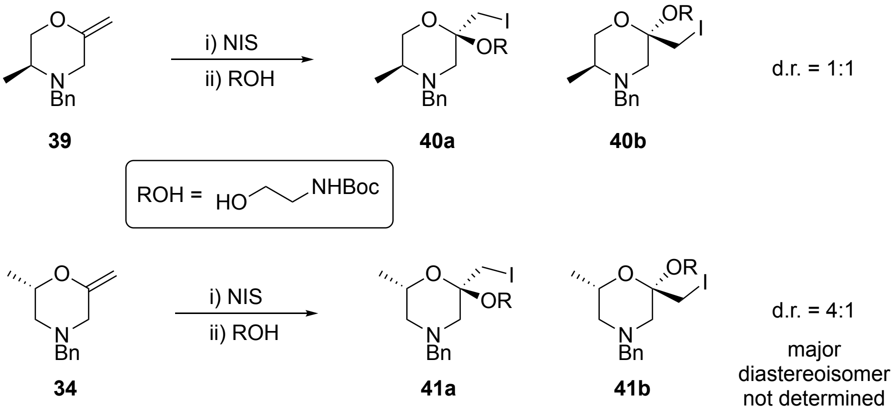

Mechanism: A possible mechanism for the iodoacetalization reaction is summarized in Scheme S17. Reaction of the enol ether with NIS affords oxacarbenium ion **I**, potentially via an iodonium intermediate. Assuming **I** is the reactive electrophile and adopts a half-chair conformation, axial attack of the nucleophile [*N*-Boc-ethanolamine (**5**)] would be expected to deliver preferentially the acetal product in a chair (and not a twist boat) product conformation (Scheme S17).

Scheme S17. Possible mechanism for the iodoacetalization of methyl-substituted 2-methylidenemorpholines

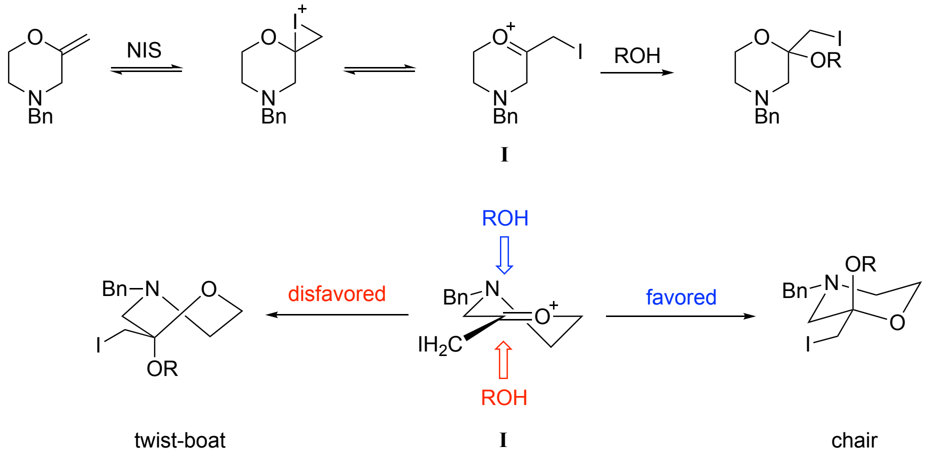

Rationalizing the difference in diastereoselectivity of the iodoacetalization of the 5-methyl- and 6-methyl regioisomers: Methyl substitution on the 2-methylidenemorpholine ring affords two half-chair conformations (Scheme S18). Conformational energy differences between half-chair conformations of substituted cyclohexenes (crudely modeling oxacarbenium ion **I**) are typically lower than those between chair conformations of the corresponding saturated analogues. Moreover, heteroatom substitution in a six-membered ring can also reduce conformational energy differences ring conformations (e.g., through the absence of axial substituents, bond length and angle differences). If

the conformational energy differences between the two half-chair conformations of the oxacarbenium ions derived from **34** and **39** are small, then we postulate reaction proceeds similarly on both conformations. Assuming the two diastereoisomeric acetal products derive from axial attack on these two half-chair conformations, then 5-methyl substitution does not appear to have significant impact on the two transition states; this would be consistent with the formation of acetal **40** as a 1:1 mixture of diastereoisomers. By contrast, there are unfavorable interactions between the nucleophile and the pseudoaxially oriented methyl substituent in the half-chair conformation of the oxacarbenium ion that would deliver acetal **41b**; this might be expected to better differentiate the two transition states and would be consistent with one of the acetal diastereoisomers (**41a** according to this analysis) being formed preferentially (observed d.r. = 4:1).

Scheme S18. Rationalizing the difference in diastereoselectivity in the iodoacetalization of methyl-substituted 2-methylidenemorpholines

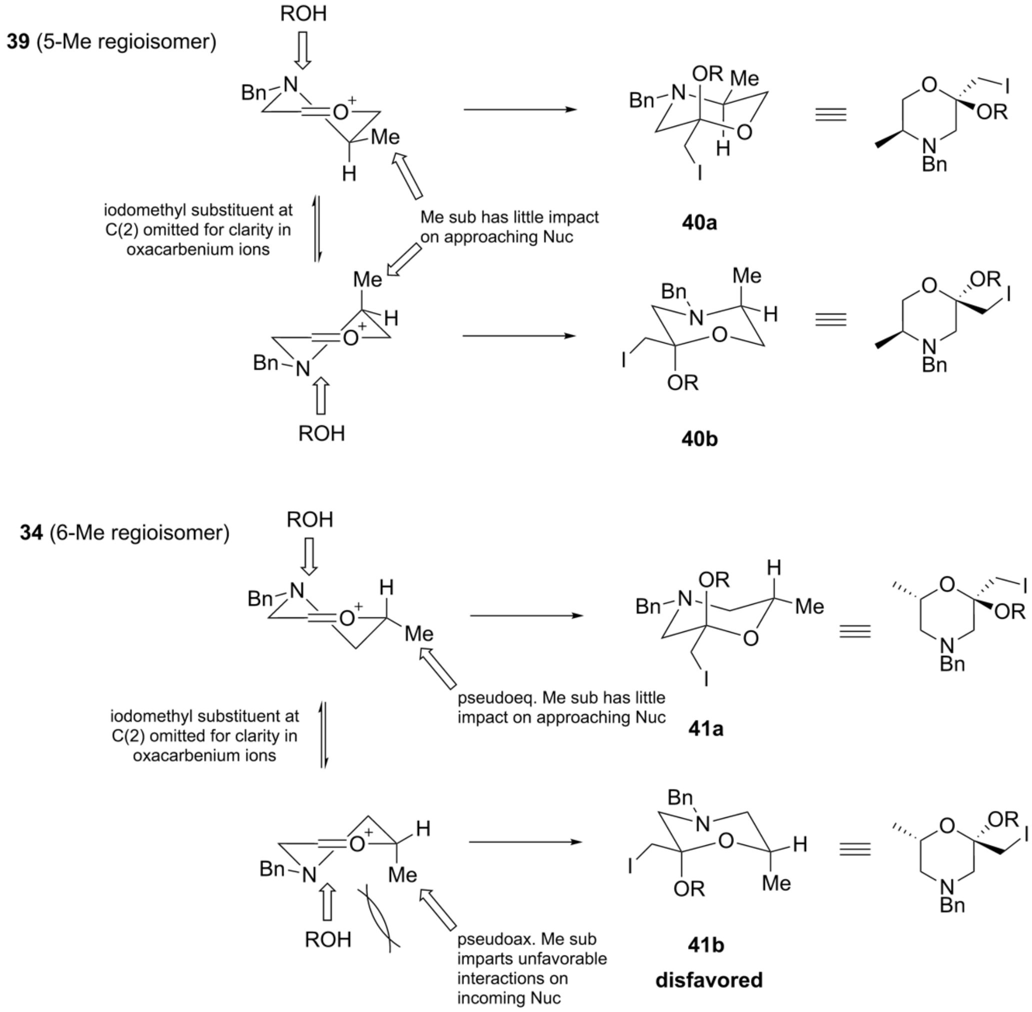

This analysis offers one possible explanation for the observed difference in diastereoselectivity in the iodoacetalization of **34** and **39**. However, it is noted that the acetal products were used directly to form bis-spiro-morpholines **43** and **42**, respectively, and whilst the diastereoisomeric ratio was preserved in these spiroacetal products, the relative stereochemistry in the diastereoisomers could not be determined.

2 Experimental Section

2.1 General Experimental

Unless stated otherwise, all reactions were carried out in oven-dried glassware under an argon atmosphere using anhydrous solvents. Anhydrous THF and CH_2Cl_2 were collected from a solvent purification system and stored over activated 4 Å molecular sieves. Molecular sieves were activated by heating (using a heating block fitted to a stirrer hotplate) at 250 °C under high vacuum (< 2 mbar) for at least 6 h prior to use, following a procedure by Williams and Lawton.[16] All other anhydrous solvents (toluene, 1,2-dichloroethane, DMF, MeOH) were purchased stored on molecular sieves (size not specified) and used without further drying. All other reagents and solvents were purchased from commercial suppliers and used without further purification unless stated otherwise. $Ni(COD)_2$ was stored and weighed in a nitrogen-filled glove box.

All solutions used in reaction work-up procedures are aqueous and saturated unless stated otherwise. PTFE syringe filters (45 µm pore size) were used for the filtration of small quantities.

The progress of reactions was determined by thin-layer chromatography (TLC), which was performed on silica gel 60 F_{254} plates. TLC plates were visualized under UV irradiation (254 nm) and by staining with either potassium permanganate, ninhydrin or vanillin solutions. Flash column chromatography was performed either using hand-filled silica gel columns (230–400 mesh, 40–63 µm) with the indicated eluent, or using a chromatography purification system (equipped with a UV-vis detector) in which case, the choice of column size, i.e., the amount of silica gel used, was decided based on the scale of the reaction as follows:

amount of silica in the column (g)	reaction size (mmol)	flow rate (mL min^{-1})
10	<2	32
25–50	2–7	70
100	7–15	90
340	15	90

Typical gradient flash column chromatography elution methods on a 10 g column are listed below. For reactions performed on larger scale, the number of solvent column volumes (CV) used was proportionately decreased:

- Elution with EtOAc in *n*-heptane: starting from 0% EtOAc (2 CV), 0–50% EtOAc (5 CV), 50% EtOAc (2 CV), 50–100% EtOAc (5 CV) and finally 100% EtOAc (2 CV).
- Elution with MeOH in CH_2Cl_2: starting from 0% MeOH (2 CV), 0–5% MeOH (5 CV), 5% MeOH (2 CV), 5–10% MeOH (5 CV) and finally 10% MeOH (2 CV).

General methods have been provided for frequently used experimental procedures; any deviations from the general method are specified when discussing the synthesis of individual compounds.

Evaporation of solvents containing hydrogen chloride (primarily for Boc-group deprotection) was carried out using a rotary evaporator housed in a ducted fume cupboard and equipped with a trap containing 2 M NaOH solution to avoid hydrogen chloride progressing into the vacuum pump.

Solvents were degassed, where specified, with a portable vacuum pump (~200 mbar) connected to the reaction vessel. For small-scale reactions (0.1 – 0.4 mmol), the reaction vessel was evacuated for 10 s (for multigram reactions, this time was extended) and then refilled with N_2 or Ar for reactions performed under an inert atmosphere. This process was repeated at least twice before initiating the reaction. For Pd/C hydrogenolysis reactions, the reaction solution was first degassed with Ar prior to adding the Pd/C catalyst. The resulting mixture was evacuated and flushed with Ar one more time and then the reaction vessel was evacuated and refilled twice with H_2 which was retained over the reaction mixture. Upon completion of the reaction, the vessel was evacuated and flushed with Ar twice before opening it to air.

"rt" refers to ambient room temperature, i.e., around 23 °C.

^{1}H-NMR, ^{13}C{^{1}H}-NMR and ^{19}F-NMR spectra were recorded in commercially available deuterated solvents on 300 MHz (^{1}H = 300 MHz) or 400 MHz (^{1}H = 400 MHz, ^{13}C = 101 MHz, ^{19}F = 376 MHz) spectrometers. Spectra recorded in different deuterated solvents were calibrated using the following reference signals:[17]

solvent	δ_H (ppm)	δ_C (ppm)
$CDCl_3$	7.26	77.2
CD_3OD	3.31	49.00
C_6D_6	7.16	128.1
$(CD_3)_2SO$	2.50	39.5

Chemical shifts are reported in ppm. Coupling constants (J) are averages of the couplings ($J_{A–B}$ and $J_{B–A}$) and are reported in Hz to the nearest 0.1 Hz. It is acknowledged that in ABX systems, the experimentally measured and reported $J_{A–X}$ and $J_{B–X}$ values are approximations of their true values. The following abbreviations are used to describe the multiplicity of resonances in ^{1}H-NMR spectra: s (singlet), d (doublet), t (triplet), q (quartet), m (multiplet), app (apparent) and br (broad). Multiplicities are reported as observed, and do not necessarily represent the true multiplicity of the resonance; for example, a resonance reported as a triplet may in fact be a doublet of doublets. 'Stack' is used to describe resonances from two or more non-equivalent protons which are coincident (including rotamer and diastereoisomer resonances of protons attached to the same carbon atom).

Assignments in NMR spectra are based on definitive chemical shifts and couplings, or from 2D experiments (COSY, HSQC, HMBC, NOESY) or based on analogy with previously assigned structurally very similar compounds. In those instances where resonances cannot be assigned with certainty, spectra are not assigned. Library compounds were only characterized by ^{1}H- and ^{13}C{^{1}H}-NMR spectroscopy without 2D-NMR measurements and therefore resonances are unassigned. The numbering of hydrogen and carbon atoms in compounds is arbitrary and not related to the numbering used within the compound name. Hydrogen atoms attached to the same carbon atom, e.g., CH_2, C-2, but which are in different environments (diastereotopic protons) are assigned as "H-2a" and "H-2b" without explicitly defining them. In cases where the hydrogen resonances from the same C-2 carbon atom are inside the same stack, no annotation is written, i.e., "(stack, 2H, H-2)" and not "(stack, 2H, H-2a, H-2b)".

In assigning NMR spectra of those compounds which existed as a mixture of rotamers (typically amides and carbamates) or were isolated as a mixture of diastereoisomers, the rotameric/diastereoisomeric ratios were assigned based on the relative integration of distinct resonances in the ^{1}H-NMR spectrum and are reported as fractional integrations. Where possible and appropriate, resonances belonging to different rotamers/diastereoisomers are distinguished with maj (major) and min (minor) notations. This notation is used to describe resonances in both the ^{1}H-NMR and ^{13}C{^{1}H}-NMR spectra, where appropriate. In the case of a ~1:1 ratio of rotamers/diastereoisomers, "rotA" and "rotB" or "diastA" and "diastB" are used to distinguish rotamers/diastereoisomers although this notation is not used explicitly to connect a resonance to a specific rotamer as this was usually not possible with 1:1 mixtures. As an example, when two hydrogen atoms are attached to the same carbon atom, e.g., C-2 and the compound exists as rotamers, then the assignment may appear as: "(stack, 1H, H-2a rotA, H-2a rotB)". Due to rotameric effects, the presence of diastereoisomers and the symmetry of some compounds, NMR assignments were not always possible. In the case of ^{13}C{^{1}H}-NMR spectroscopic

data, where it is clear, resonances for rotamers for a single carbon environment are collected in square brackets. In those cases where rotamers were not observed or could not be determined, then these are acknowledged. The absence of rotamers is acknowledged where rotamers might otherwise be expected.

In those instances, where compounds were isolated as a ~1:1 mixture of diastereoisomers or characterized as a ~1:1 mixture of rotamers (principally substituted spiroacetals), it was typically not possible to assign resonances definitively to a particular diastereoisomer or rotamer; assignments are therefore made to the hydrogen/carbon environment (e.g., H-2a, H-2b) in the general structure. In those instances where resonance broadening was observed without separation of rotamers, resonances are reported as a range of chemical shifts; where rotamers can be clearly distinguished, coincident resonances are described as stacks.

The crystallographic datasets for **7** and **23** were measured on a diffractometer. The data collections were driven and processed and absorption corrections were applied using CrysAlisPro.[18] Both structures were solved using ShelXT[19] and refined by a full-matrix least-squares procedure on F^2 in ShelXL.[20] Figures and reports were produced using OLEX2[21] and ORTEP-3 for Windows.[22] All non-hydrogen atoms were refined with anisotropic displacement parameters and all hydrogen atoms in both structures were fixed as riding models and the isotropic thermal parameters (U_{iso}) were based on the U_{eq} of the parent atoms.

Melting points were measured using open capillaries.

Infrared spectra were recorded as thin films, neat or KBr discs, either by one of the authors (Daniel Kovari) or by Dr Holly Adcock at The University of Birmingham, or by Dr Maik Icker at the University of Leipzig. Wavelengths (ν_{max}) are reported in cm^{-1}.

HRMS and LRMS measurements (EI, ES, CI, ASAP) were recorded at the University of Birmingham either by one of the authors (Daniel Kovari) or by Dr Holly Adcock or Dr Christopher Williams. In some instances, diethylamine (MW = 73.14 g mol^{-1}) was used as an ion source during mass spectrum acquisition; in these cases, its adduct with the compound was typically observed in the mass spectrum.

LCMS measurements, primarily for library compounds, were carried out by the analytical team at AnalytiCon Discovery GmbH. Routinely basic and neutral measurements were carried out with the following specifications: MS pump: Shimadzu 2020 single quadrupole, LC pump: Shimadzu LC-20 AD

XR, Autosampler: Shimadzu SiL-20 A XR, PDA detector: Shimadzu SPD-M20 A (210–400 nm), ELSD detector: Sedere Sedex85 (pressure 4 bar, nebulizer temperature 35–50 °C).

- Basic LCMS method: column: Gemini NX C18 (3 µm, 50 × 3 mm), eluents: A: 5 mmol mL^{-1} $NH_4HCO_{3(aq)}$ + $NH_4OH_{(aq)}$ (pH = 10.4); B: MeOH + 5 mmol mL^{-1} $NH_4HCO_{3(aq)}$, injection volume: 5 µL, flow-rate: 1.2 mL min^{-1}, scan range: 100–1500 g mol^{-1}.
- Neutral LCMS method: column: Cortecs UPLC C18 (1.6 µm, 50 × 2.1 mm), eluents: A: 5 mmol mL^{-1} $NH_4HCO_{2(aq)}$ + 0.1% $HCO_2H_{(aq)}$ (pH not defined); B: MeOH:MeCN 1:1 + 5 mmol mL^{-1} $NH_4HCO_{2(aq)}$ and 0.1% $HCO_2H_{(aq)}$, injection volume: 0.1–5 µL, flow-rate: 1.0 mL min^{-1}, scan range: 100–1500 g mol^{-1}.

Preparative HPLC was conducted by the analytical team at AnalytiCon Discovery GmbH. Dried reaction mixtures were dissolved in DMSO (1–2 mL) and eluted through a syringe filter (pore size 45 µm, PTFE) before loading on to a preparative HPLC purification system with the following specifications: LC pump: Knauer K1800, UV detector: Knauer K2500 UV (210–400 nm), ELSD detector: Sedere Sedex75 (pressure 4 bar, nebulizer temperature 35–50 °C).

- Basic preparative HPLC method: Gemini NX C18 (5 µm, 50 mm × 21.2 mm), eluents: A: H_2O + 1 v/v% $NH_4OH_{(aq)}$ (26 wt%), B: MeCN + 1 v/v% $NH_4OH_{(aq)}$ (26 wt%), flow-rate: 35 mL min^{-1}, using a fitted gradient system for each compound.
- Neutral preparative HPLC method: Phenomenex LunaC8 column (5 µm, 50 mm × 21.2 mm), eluents: A: H_2O + 0.1% $HCO_2H_{(aq)}$ (pH not defined), B: MeOH + 0.1% $HCO_2H_{(aq)}$ (pH not defined), flow-rate: 70 mL min^{-1}, using a fitted gradient system for each compound.

Fraction collection was based on UV absorbance (215 nm) of the preparative HPLC purifications. The combined fractions were concentrated with a blowdown evaporator (using 2-dram vials maintained at rt or heated on a heating block at 50 °C while exposing to a pressurized jet of air to facilitate the evaporation of solvent). The analytical purity of the isolated compounds was assessed based on UV absorbance of basic and neutral LCMS measurements. The retention times reported for library compounds are based on these LCMS data.

Unless otherwise stated, the appearance of library compounds purified by preparative HPLC is not reported; these samples were only handled by the analytical team at AnalytiCon Discovery GmbH and then immediately stored in the company's compound respiratory. ^{19}F-NMR spectra were not recorded for library compounds containing fluorine.

Library compounds are numbered as follows:

- The first group of digits refers to the scaffold, for example, 6,6-spiroacetal **9 (L1)** or 6,7-spiroacetal **25 (L2)**.

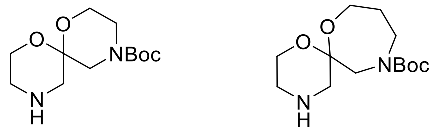

9 **25**

- Next, a letter and integer refer to the first decorating reagent (e.g., -**B03**, or -**I05**).
- Finally, a letter and integer refer to the second decorating reagent.
- The letters are defined as follows: **I** = isocyanate (for urea formation), **B** = sulfonyl chloride (for sulfonamide formation), **C** = carboxylic acid (for amide formation), **A** = aldehyde, **K** = ketone (for 2° and 3° amine formation).

As an example, compound **L1-I03-B05** refers to the library compound derived from 6,6-spiroacetal scaffold **L1**. **I03** is the isocyanate used in the first decoration step, and **B05** refers to the sulfonyl chloride used in the second decoration step:

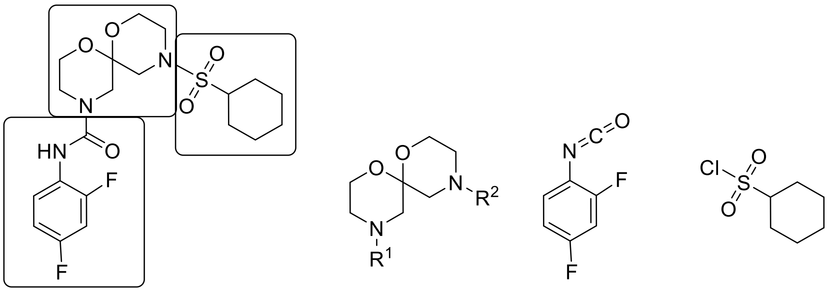

L1-I03-B05 **L1** **I03** **B05**

Those library compounds named in the main article are described with standard compound numbers; for simplicity, these are renumbered with their library descriptors in Table S6.

Table S6: Library compounds described in the main article and in the supporting information.

Structure	Cpd number in main article	Cpd number in SI	Structure	Cpd number in main article	Cpd number in SI
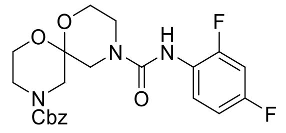	**11**	**10-I03**	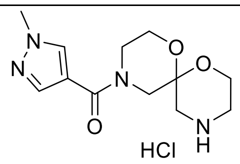	**17·HCl**	**L1-C28**
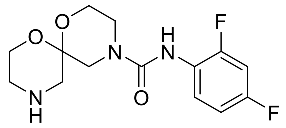	**13**	**L1-I03**	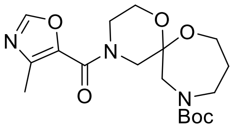	**26**	**25-C30**
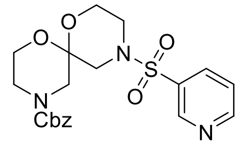	**12**	**10-B08**	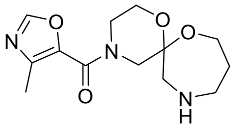	**29**	**L2-C30**
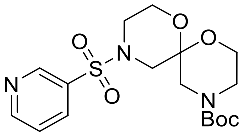	**14**	**9-B08**	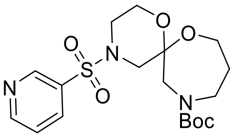	**27**	**25-B08**
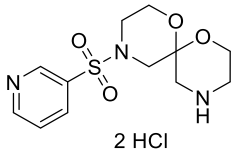	**16·2HCl**	**L1-B08·2HCl**	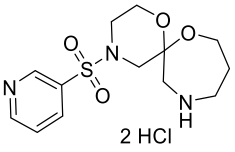	**30·2HCl**	**L2-B08·2HCl**
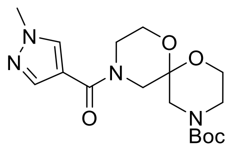	**15**	**9-C28**	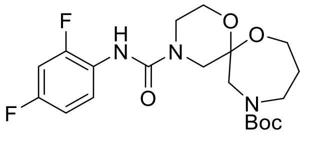	**28**	**25-I03**
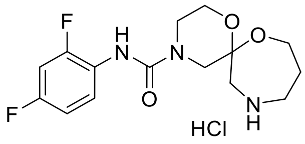	**31·HCl**	**L2-I03·HCl**			

2.2 Stoltz chemistry

(*Z*)-2-iodo-3-phenylacrylaldehyde (S3):

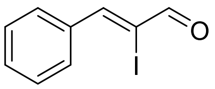

Under an Ar atmosphere, a solution of ICl (4.9 g, 30 mmol) in CH_2Cl_2 (30 mL) was added dropwise over 15 min (or slower when precipitation occurred) to a solution of pyridine (4.9 mL, 61 mmol) in CH_2Cl_2 (45 mL) at 0 °C. *Trans*-Cinnamaldehyde (**S2**) (2.00 g, 15.1 mmol) was then added over 2 min to the clear orange solution at 0 °C. The reaction vessel was then covered with aluminum foil and the reaction mixture was allowed to warm to rt without removing the ice-H_2O bath (~2 h to reach rt). After 20 h, hydrochloric acid (40 mL, 2 M) was added to the reaction mixture, resulting in a yellow precipitate, which was removed by filtration, washing with CH_2Cl_2 (2 × 10 mL). The resulting biphasic filtrate was partitioned, and the aqueous layer was extracted with CH_2Cl_2 (3 × 50 mL). The organic phases were combined and washed with hydrochloric acid (40 mL, 2 M). H_2O (100 mL) was then added to the organic phase, followed by the addition of $Na_2S_2O_3 \cdot 5H_2O$ (~7.5 g, ~30 mmol) to the biphasic system. The mixture was swirled until the solution became pale orange in color. The organic layer was separated and concentrated under reduced pressure. The resulting yellow-orange crystals were washed with a mixture of H_2O/EtOH (2/1 mixture, 2 × 15 mL). The crystals were dissolved in CH_2Cl_2 (30 mL), dried over $MgSO_4$, filtered and the solution was concentrated under reduced pressure to provide iodide **S3** as a light orange solid (4.29 g, quant.), which was used in the next step without further purification.

^{1}H-NMR (400 MHz, $CDCl_3$) δ_H 8.80 (d, *J* = 0.7 Hz, 1H), 8.11 – 8.08 (m, 1H), 8.06 – 7.97 (m, 2H), 7.59 – 7.41 (stack, 3H).

^{13}C{^{1}H}-NMR (101 MHz, $CDCl_3$) δ_C 189.1 (CH), 155.8 (CH), 134.1 (C), 131.6 (CH), 130.5 (CH), 128.6 (CH), 105.9 (C).

LRMS (ES+): 313.1 [$(M + Na + MeOH)^+$, 30%], 281.1 [100, $(M + Na)^+$], 259.1 [40, $(M + H)^+$].

Data were in agreement with those reported in the literature.[1]

(*Z*)-2-iodo-3-phenylprop-2-en-1-ol (S4):

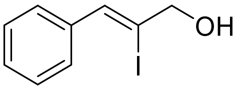

$CeCl_3 \cdot 7H_2O$ (2.89 g, 7.75 mmol) was added to a solution of aldehyde **S3** (2.00 g, 7.75 mmol) in MeOH (78 mL) at 0 °C. After 10 min, $NaBH_4$ (440 mg, 11.6 mmol) was added portionwise over 10 min. After 10 min, the reaction was quenched by the dropwise addition of H_2O (40 mL), and the resulting mixture

was concentrated under reduced pressure to remove the majority of the MeOH. The remaining solution was extracted with EtOAc (3 × 50 mL). The organic layers were combined, dried over $MgSO_4$, filtered and concentrated under reduced pressure to produce alcohol **S4** as a light brown oil (1.73 g, 85%), which solidified upon storage at 4 °C after 16 h. Alcohol **S4** was used in the next step without further purification.

R_f (*n*-hexane/EtOAc, 3/2) = 0.5.

^{1}H-NMR (400 MHz, $CDCl_3$) δ_H 7.56 – 7.47 (m, 2H), 7.43 – 7.27 (stack, 3H), 7.12 (br s, 1H), 4.44 (s, 2H), 2.17 – 2.05 (br s, 1H).

^{13}C{^{1}H}-NMR (101 MHz, $CDCl_3$) δ_C 137.0 (C), 134.0 (CH), 128.6 (CH), 128.22 (CH), 128.18 (CH), 106.4 (C), 72.8 (CH_2).

LRMS (ES+): 315.2 [$(M + Na + MeOH)^+$, 10%], 283.1 [60, $(M + Na)^+$], 243.1 [100, $(M – OH)^+$], 116.1 [60, $(M – OH – I)^+$].

Data were in agreement with those reported in the literature.[1]

(*Z*)-(3-bromo-2-iodoprop-1-en-1-yl)benzene (S1):

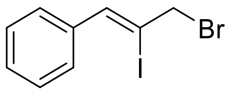

PPh_3 (2.52 g, 9.61 mmol) was added in one portion to a solution of alcohol **S4** (2.00 g, 7.69 mmol) and CBr_4 (3.19 g, 16.8 mmol) in MeCN (77 mL) at 0 °C. After 10 min, the mixture was concentrated under reduced pressure and the residue was flushed through a column (*n*-hexane/EtOAc, 4/1), producing bromide **S1** as a light-yellow oil (2.58 g, quant.).

R_f (*n*-hexane/EtOAc, 3/2) = 0.8.

^{1}H-NMR (400 MHz, $CDCl_3$) δ_H 7.60 – 7.55 (m, 2H), 7.46 – 7.33 (stack, 3H), 7.14 (br s, 1H), 4.58 (d, J = 1.0 Hz, 2H).

^{13}C{^{1}H}-NMR (101 MHz, $CDCl_3$) δ_C 138.6 (CH), 136.7 (C), 128.8 (CH), 128.7 (CH), 128.2 (CH), 99.5 (C), 45.1 (CH_2).

LRMS (EI): 323.9 [$(M[^{81}Br])^+$, 10%], 321.9 [10, $(M[^{79}Br])^+$], 243.0 [50, $(M – Br)^+$], 116.1 [100, $(M – Br – I)^+$].

Data were in agreement with those reported in the literature.[1]

(*Z*)-2-((2-iodo-3-phenylallyl)(methyl)amino)ethan-1-ol (S5):

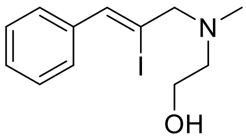

K_2CO_3 (4.28 g, 31.0 mmol) and bromide **S1** (1.00 g, 3.10 mmol) were added to a solution of *N*-methylethanolamine (279 mg, 3.72 mmol) in MeCN (21 mL) at rt. After 16 h, the reaction mixture was filtered through a Celite plug, which was washed with EtOAc (2 × 10 mL). The filtrate was concentrated under reduced pressure. The resulting crude product was dissolved in EtOAc (20 mL) and extracted with H_2O (2 × 10 mL). The organic layer was washed with brine (30 mL), dried over $MgSO_4$, filtered and concentrated under reduced pressure to produce 3° amine **S5** as a light-yellow oil (908 mg, 92%), which was used in the next step without further purification.

R_f (*n*-hexane/EtOAc, 3/2) = 0.3.

^{1}H-NMR (400 MHz, $CDCl_3$) δ_H 7.58 – 7.51 (m, 2H), 7.40 – 7.29 (stack, 3H), 7.00 (br s, 1H), 3.70 – 3.64 (m, 2H), 3.40 (d, *J* = 1.3 Hz, 2H), 3.16 – 2.95 (br s, 1H), 2.73 – 2.65 (m, 2H), 2.34 (s, 3H).

^{13}C{^{1}H}-NMR (101 MHz, $CDCl_3$) δ_C 137.2 (C), 136.5 (CH), 128.7 (CH), 128.2 (CH), 128.1 (CH), 106.4 (C), 70.3 (CH_2), 58.4 (CH_2), 57.9 (CH_2), 40.8 (CH_3).

LRMS (EI): 286.0 [$(M - CH_2OH)^+$, 100%], 243.0 [30, $(M - N(CH_3)CH_2CH_2OH)^+$], 190.1 [20, $(M - I)^+$].

Data were in agreement with those reported in the literature.[5]

(*Z*)-2-(benzyl(2-iodo-3-phenylallyl)amino)ethan-1-ol (S6):

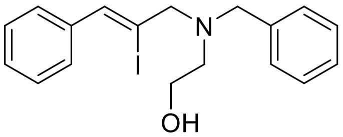

K_2CO_3 (428 mg, 3.10 mmol) and bromide **S1** (0.10 g, 0.31 mmol) were added to a solution of *N*-benzylethanolamine (70 mg, 0.46 mmol) in MeCN (2.1 mL) at rt. After 20 h, the reaction mixture was filtered through a Celite plug, which was washed with EtOAc (2 × 5 mL). The filtrate was concentrated under reduced pressure. The resulting crude product was dissolved in EtOAc (10 mL), washed sequentially with H_2O (10 mL) and brine (10 mL), dried over $MgSO_4$, filtered and concentrated under reduced pressure to give 3° amine **S6** as a light-brown oil (80 mg, 66%), which was used in the next step without further purification.

R_f (*n*-hexane/EtOAc, 3/2) = 0.5.

ν_{max} (thin film/cm^{-1}): 3425 br w, 2925 br m, 1730 w, 1492 m, 1056 m, 737 s, 695 s.

^{1}H-NMR (400 MHz, $CDCl_3$) δ_H 7.55 – 7.50 (m, 2H), 7.42 – 7.27 (stack, 8H), 7.02 (s, 1H), 3.71 (s, 2H), 3.66 – 3.59 (m, 2H), 3.47 (d, *J* = 1.2 Hz, 2H), 2.79 – 2.75 (m, 1H), 2.74 – 2.70 (m, 2H).

^{13}C{^{1}H}-NMR (101 MHz, $CDCl_3$) δ_C 138.0 (C), 137.3 (C), 136.7 (CH), 129.4 (CH), 128.7 (CH), 128.5 (CH), 128.2 (CH), 128.1 (CH), 127.4 (CH), 107.4 (C), 66.7 (CH_2), 58.6 (CH_2), 57.7 (CH_2), 54.3 (CH_2).

LRMS (ES+): 394.1 [$(M + H)^+$, 100%], 386.2 (30).

HRMS (ES+): calcd for $C_{18}H_{21}INO$ $[M + H]^+$ 394.0668, found 394.0669.

(*Z*)-2-((2-iodo-3-phenylallyl)amino)ethan-1-ol (S7):

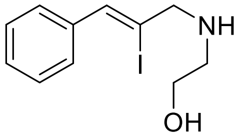

K_2CO_3 (6.42 g, 46.4 mmol) and bromide **S1** (1.50 g, 4.64 mmol) were added to a solution of ethanolamine (698 mg, 9.29 mmol) in MeCN (30 mL) at rt. After 19 h, the reaction mixture was filtered through a Celite plug, which was washed with EtOAc (2 × 15 mL). The filtrate was concentrated under reduced pressure. The resulting crude product was dissolved in EtOAc (30 mL) and extracted with hydrochloric acid (2 × 15 mL, 0.1 M). The combined aqueous layers were washed with EtOAc (15 mL) and then basified to pH 9–10 with NaOH solution (~40 mL, 2 M). The resulting mixture was extracted with EtOAc (3 × 30 mL). The combined organic layers were washed with brine (30 mL), dried over $MgSO_4$, filtered and concentrated under reduced pressure to give 2° amine **S7** as a light-brown oil (870 mg, quant.), which was used in the next step without further purification.

R_f (CH_2Cl_2/MeOH, 20/1) = 0.2.

ν_{max} (thin film/cm^{-1}): 3140 br m, 2848 br m, 1730 w, 1438 m, 1049 m, 743 s.

^{1}H-NMR (400 MHz, $CDCl_3$) δ_H 7.53 – 7.48 (m, 2H), 7.39 – 7.29 (stack, 3H), 6.96 (br s, 1H), 3.70 – 3.61 (stack, 4H), 2.84 – 2.70 (m, 2H), 2.27 – 2.02 (stack, 2H).

^{13}C{^{1}H}-NMR (101 MHz, $CDCl_3$) δ_C 137.4 (C), 135.4 (CH), 128.6 (CH), 128.2 (CH), 128.1 (CH), 108.9 (C), 62.1 (CH_2), 61.0 (CH_2), 48.7 (CH_2).

LRMS (ES+): 304.0 [$(M + H)^+$, 100%], 243.0 [15, $(M - NHCH_2CH_2OH)^+$].

HRMS (ES+): calcd for $C_{11}H_{15}INO$ $[M + H]^+$ 304.0198, found 304.0193.

tert-butyl (Z)-(2-hydroxyethyl)(2-iodo-3-phenylallyl)carbamate (S8):

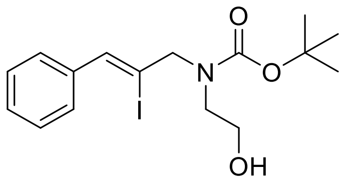

A solution of Boc_2O (43 mg, 0.20 mmol) in CH_2Cl_2 (0.8 mL) was added dropwise over 1 min to a solution of Et_3N (28 µL, 0.20 mmol) and aminoalcohol **S7** (50 mg, 0.16 mmol) in CH_2Cl_2 (0.8 mL) at 0 °C. The reaction mixture was then warmed to rt without removing the ice-H_2O bath (~1 h). After 5 h, the reaction mixture was extracted with $NaHCO_3$ solution (3 × 2 mL). The organic layer was dried over $MgSO_4$, filtered, concentrated under reduced pressure and the crude product was purified by flash column chromatography (*n*-hexane/EtOAc, 2/1) to afford carbamate **S8** as a light-yellow oil (45 mg, 68%).

R_f (*n*-hexane/EtOAc, 3/2) = 0.3.

ν_{max} (thin film/cm^{-1}): 3422 br w, 2928 br w, 1669 s, 1160 s, 740 s, 693 s.

^{1}H-NMR (400 MHz, $CDCl_3$, resonance broadening observed because of rotamers) δ_H 7.47 – 7.39 (stack, 2H), 7.33 – 7.22 (stack, 3H), 6.82 (s, 1H), 4.39 – 4.19 (stack, 2H), 3.73 (br s, 2H), 3.48 – 3.32 (stack, 2H), 2.90 (br s, 1H), 1.43 (s, 9H).

^{13}C{^{1}H}-NMR (101 MHz, $CDCl_3$, rotamers not observed) δ_C 156.8 (C), 137.2 (C), 134.6 (CH), 128.7 (CH), 128.2 (2 × CH, resonance overlap as evidenced by analysis of the ^{13}C{^{1}H}-NMR spectrum), 103.3 (C), 81.0 (C), 62.3 (CH_2), 61.2 (CH_2), 49.8 (CH_2), 28.4 (CH_3).

LRMS (ES+): 426.1 [(M + Na)$^+$], 100%].

HRMS (ES+): calcd for $C_{16}H_{22}INO_3Na$ [M + Na]$^+$ 426.0542, found 426.0540.

(Z)-N-(2-hydroxyethyl)-N-(2-iodo-3-phenylallyl)-4-methylbenzenesulfonamide (S9):

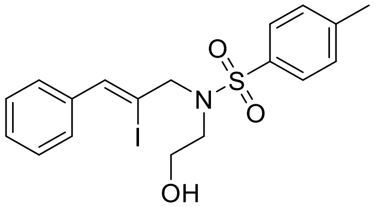

TsCl (31 mg, 0.16 mmol) and Et_3N (45 µL, 0.33 mmol) were added to a solution of aminoalcohol **S7** (50 mg, 0.16 mmol) in CH_2Cl_2 (3.3 mL) at 0 °C. After 4 h, the reaction mixture was diluted with CH_2Cl_2 (10 mL) and washed sequentially with H_2O (10 mL) and brine (10 mL). The organic layer was dried over $MgSO_4$, filtered and concentrated under reduced pressure. The crude product was purified by flash

column chromatography (*n*-hexane/EtOAc, 4/1) to afford sulfonamide **S9** as a white viscous oil (52 mg, 69%).

R_f (*n*-hexane/EtOAc, 3/2) = 0.3.

ν_{max} (thin film/cm^{-1}): 3530 br w, 2924 br w, 1727 w, 1331 s, 1153 s.

^{1}H-NMR (400 MHz, $CDCl_3$) δ_H 7.80 – 7.73 (m, 2H), 7.48 – 7.40 (m, 2H), 7.40 – 7.28 (stack, 5H), 6.98 (s, 1H), 4.34 (d, *J* = 1.5 Hz, 2H), 3.81 (q, *J* = 5.6 Hz, 2H), 3.38 (t, *J* = 5.6 Hz, 2H), 2.43 (s, 3H), 2.22 (t, *J* = 5.6 Hz, 1H).

^{13}C{^{1}H}-NMR (101 MHz, $CDCl_3$) δ_C 143.9 (C), 137.1 (CH), 136.8 (C), 136.2 (C), 129.8 (CH), 128.6 (CH), 128.5 (CH), 128.2 (CH), 127.6 (CH), 100.8 (C), 62.4 (CH_2), 61.2 (CH_2), 50.8 (CH_2), 21.6 (CH_3).

LRMS (ES+): 480.0 [$(M + Na)^+$, 100%], 425.3 [20, $(M - CH_2OH)^+$].

HRMS (ES+): calcd for $C_{18}H_{20}INO_3SNa$ $[M + Na]^+$ 480.0106, found 480.0110.

2-iodoallyl 4-methylbenzenesulfonate (S13):

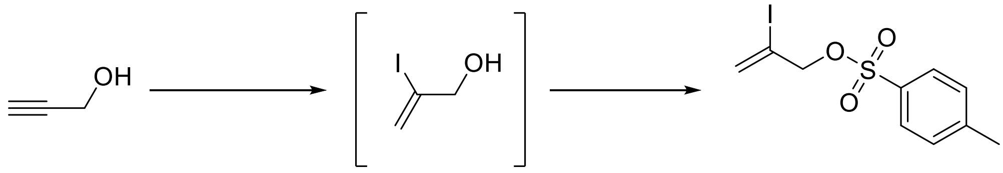

S11 **S12** **S13**

In an aluminum foil-covered flask, TMSCl (2.8 mL, 21 mmol) was added to a cooled (0 °C) suspension of NaI (3.21 g, 21.4 mmol) in MeCN (89 mL). After 15 min, H_2O (0.19 mL, 11 mmol) was added, followed by dropwise addition of propargyl alcohol (**S11**) (1.0 mL, 17 mmol) over ~ 1 min. The cooling bath was removed and the reaction mixture allowed to warm to rt. After 5 h, the reaction was not complete, as evidenced by ^{1}H-NMR spectroscopic analysis; therefore, additional NaI (1.06 g, 7.06 mmol) and TMSCl (0.91 mL, 7.1 mmol) in MeCN (30 mL) were added. After 23 h, another portion of NaI (1.06 g, 7.06 mmol), TMSCl (0.91 mL, 7.1 mmol) and H_2O (50 µL, 2.8 mmol) in MeCN (25 mL) were added to the reaction mixture. 47 h after the initial addition of TMSCl, the reaction mixture was diluted with H_2O (50 mL) and the resulting mixture was extracted with Et_2O (3 × 50 mL). The combined organic layers were washed sequentially with $NaHCO_3$ solution (100 mL), $Na_2S_2O_3$ solution (100 mL), and brine (50 mL), dried over Na_2SO_4, filtered and carefully concentrated under reduced pressure[m] to produce crude iodo alcohol **S12** as a brown oil (1.65 g, 50%), which was used in the next step without purification. The identity of iodo alcohol **S12** was confirmed by ^{1}H-NMR spectroscopic analysis, which was in agreement with data reported in the literature:[2] ^{1}H-NMR (400 MHz, $CDCl_3$) δ_H 6.39 (q, *J* = 1.7 Hz, 1H), 5.91 – 5.83 (m, 1H), 4.18 (br s, 2H), 2.11 (br s, 1H).

[m] The boiling point of the product is low (lit.[2] 50 °C, 7 mbar).

Under an Ar atmosphere, Et_3N (2.0 mL, 15 mmol), DMAP (0.12 g, 0.98 mmol, 20 mol%) and TsCl (1.40 g, 7.34 mmol) were added sequentially to a solution of iodo alcohol **S12** (900 mg, 4.89 mmol) in CH_2Cl_2 (50 mL) at 0 °C. After 4 h, the reaction mixture was diluted with CH_2Cl_2 (20 mL) and washed sequentially with H_2O (20 mL) and brine (20 mL). The organic layer was dried over $MgSO_4$, filtered, concentrated under reduced pressure and the product was purified by flash column chromatography (gradient, 5–10% EtOAc in *n*-hexane) to give tosylate **S13** as a colorless oil (859 mg, 52%).

R_f (*n*-hexane/EtOAc, 3/2) = 0.7.

^{1}H-NMR (400 MHz, $CDCl_3$) δ_H 7.86 – 7.76 (AA' of AA'BB', 2H), 7.39 – 7.30 (BB' of AA'BB', 2H), 6.41 (dt, *J* = 2.2, 1.6 Hz, 1H), 5.91 (dt, *J* = 2.2, 1.1 Hz, 1H), 4.61 – 4.55 (m, 2H), 2.46 (s, 3H).

^{13}C{^{1}H}-NMR (101 MHz, $CDCl_3$) δ_C 145.2 (C), 132.9 (C), 130.0 (CH), 128.6 (CH_2), 128.1 (CH), 98.6 (C), 75.5 (CH_2), 21.7 (CH_3).

LRMS (ES+): 360.9 [$(M + Na)^+$], 100%], 180.1 (20).

Data were in agreement with those reported in the literature.[4]

2-(benzyl(2-iodoallyl)amino)ethan-1-ol (S10):

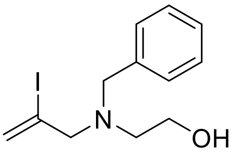

K_2CO_3 (570 mg, 4.13 mmol) and tosylate **S13** (670 mg, 1.98 mmol) were added to a solution of *N*-benzylethanolamine (250 mg, 1.66 mmol) in MeCN (17 mL) at rt. The resulting mixture was heated in an oil bath at 80 °C. After 14 h, the mixture was cooled to rt, diluted with H_2O (20 mL) and extracted with Et_2O (3 × 20 mL). The combined organic layers were washed with brine (30 mL), dried over $MgSO_4$, filtered, concentrated under reduced pressure and the crude product was purified by flash column chromatography (*n*-hexane/EtOAc 4/1 + 1 v/v% Et_3N) to produce 3° amine **S10** as a clear oil (415 mg, 80%).

R_f (*n*-hexane/EtOAc, 3/2) = 0.6.

^{1}H-NMR (400 MHz, $CDCl_3$) δ_H 7.40 – 7.23 (stack, 5H), 6.34 (q, *J* = 1.4 Hz, 1H), 5.99 – 5.87 (m, 1H), 3.64 (s, 2H), 3.61 – 3.56 (m, 2H), 3.17 (br s, 2H), 2.69 (br s, 1H), 2.64 – 2.55 (m, 2H).

^{13}C{^{1}H}-NMR (101 MHz, $CDCl_3$) δ_C 137.9 (C), 129.3 (CH), 128.4 (CH), 127.9 (CH_2), 127.4 (CH), 112.0 (C), 64.9 (CH_2), 58.6 (CH_2), 57.6 (CH_2), 54.2 (CH_2).

LRMS (ES+): 318.0 [$(M + H)^+$, 100%].

Data were in agreement with those reported in the literature.[5]

2.2.1 General procedure A – Intramolecular $Ni(COD)_2$-catalyzed cross-coupling

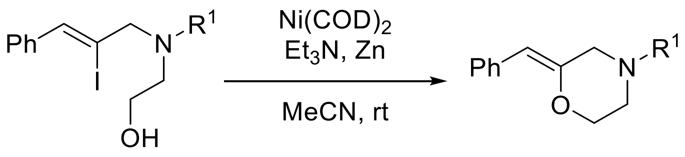

Following the procedure reported by Stoltz and co-workers,[5] in a Schlenk tube under an Ar atmosphere, anhydrous Et_3N (1.73 mmol, 1.10 eq) and activated (see below) Zn powder (3.15 mmol, 2.00 eq) were added to a solution of the iodo alcohol (1.58 mmol, 1.00 eq) in anhydrous MeCN (11 mL, 0.15 mmol mL^{-1}). With Ar gas flowing through the Schlenk tube, $Ni(COD)_2$ (0.13 mmol, 8 mol%) was added to the reaction mixture at rt. After 0.5–24 h (typical reaction time), the reaction mixture was filtered through a Celite plug, which was washed with EtOAc (3 × reaction volume). The organic layers were combined and the volatiles were removed under reduced pressure. The crude mixture was redissolved in EtOAc (2 × reaction volume), washed with H_2O (2 × reaction volume), dried over $MgSO_4$, filtered and concentrated under reduced pressure to produce the enol ether product.

Zn activation was performed in a fume cupboard as follows: Zn powder was poured on a sinter, swirled in 2 M hydrochloric acid for 30 s and then washed with deionized H_2O. Then, it was swirled in 6 M hydrochloric acid for 30 s and then washed with deionized H_2O. Finally, the Zn powder was swirled in 12 M hydrochloric acid for 30 s and then washed sequentially with deionized H_2O and twice with acetone. The activated zinc powder was transferred to a flask and placed on the high vacuum line to remove residual solvent.

(*Z*)-2-benzylidene-4-methylmorpholine (S14):

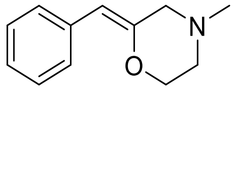

Following general procedure A (p 38), $Ni(COD)_2$ (35.0 mg, 0.13 mmol, 8 mol%), activated Zn powder (206 mg, 3.15 mmol) and Et_3N (245 µL, 1.73 mmol) were added to a solution of iodo alcohol **S5** (500 mg, 1.58 mmol) in MeCN (11 mL). After 30 min, the reaction mixture was worked up according to the general procedure to produce enol ether **S14** as a brown oil (299 mg, quant.).
R_f (*n*-hexane/EtOAc, 3/2) = 0.1.

^{1}H-NMR (400 MHz, $CDCl_3$ (filtered through basic alumina)) δ_H 7.59 – 7.54 (m, 2H), 7.33 – 7.23 (m, 2H), 7.20 – 7.09 (m, 1H), 5.46 (s, 1H), 4.10 – 4.01 (m, 2H), 3.03 (s, 2H), 2.63 – 2.54 (m, 2H), 2.34 (s, 3H).

^{13}C{^{1}H}-NMR (101 MHz, $CDCl_3$) δ_C 149.7 (C), 135.7 (C), 128.5 (CH), 128.1 (CH), 126.0 (CH), 107.9 (CH), 67.5 (CH_2), 58.4 (CH_2), 54.2 (CH_2), 46.1 (CH_3).

LRMS (ES+): 190.1 [$(M + H)^+$, 100%].

Data were in agreement with those reported in the literature.[5]

(*Z*)-4-benzyl-2-benzylidenemorpholine (S15):

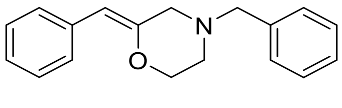

Following general procedure A (p 38), $Ni(COD)_2$ (4.5 mg, 0.016 mmol, 8 mol%), activated Zn powder (27 mg, 0.41 mmol) and Et_3N (32 µL, 0.22 mmol) were added to a solution of iodo alcohol **S6** (80.0 mg, 0.20 mmol) in MeCN (1.4 mL) at rt. After 30 min, the reaction mixture was worked up according to the general procedure and the crude product was purified by flash column chromatography (*n*-hexane/EtOAc 10/1 + 1 v/v% Et_3N) to afford enol ether **S15** as a viscous white oil (45 mg, 83%).

R_f (*n*-heptane/EtOAc, 3/2) = 0.6.

ν_{max} (thin film/cm^{-1}) 2920 br m, 1661 s, 1335 s, 1169 s, 1053 s, 694 s.

^{1}H-NMR (400 MHz, $CDCl_3$ (filtered through basic alumina)) δ_H 7.64 – 7.51 (m, 2H), 7.42 – 7.21 (stack, 7H), 7.20 – 7.08 (m, 1H), 5.41 (s, 1H), 4.13 – 4.02 (m, 2H), 3.57 (s, 2H), 3.12 (s, 2H), 2.70 – 2.58 (m, 2H).

^{13}C{^{1}H}-NMR (101 MHz, $CDCl_3$) δ_C 149.9 (C), 137.3 (C), 135.8 (C), 129.2 (CH), 128.4 (2 × CH, resonance overlap as evidenced by analysis of the ^{13}C{^{1}H}-NMR spectrum), 128.1 (CH), 127.4 (CH), 125.9 (CH), 107.7 (CH), 67.4 (CH_2), 63.1 (CH_2), 56.6 (CH_2), 52.2 (CH_2).

LRMS (ES+): 266.2 [$(M + H)^+$, 100%].

HRMS (ES+): calcd for $C_{18}H_{20}NO$ $[M + H]^+$ 266.1545, found 266.1543.

(*Z*)-2-benzylidenemorpholine (S16):

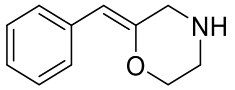

Following general procedure A (p 38), Ni(COD)$_2$ (18 mg, 0.066 mmol, 9 mol%), activated Zn powder (0.11 g, 1.7 mmol) and Et$_3$N (125 µL, 0.91 mmol) were added to a solution of iodo alcohol **S7** (0.23 g, 0.76 mmol) in MeCN (2.2 mL) at rt. After 24 h, the reaction mixture was worked up according to the general procedure to produce enol ether **S16** as a brown oil (133 mg, quant.).[n]

R$_f$ (CH_2Cl_2/MeOH, 10/1) = 0.3.

ν_{max} (thin film/cm^{-1}) 3306 br w, 2957 br m, 1659 s, 1307 s, 1163 s, 754 s, 694 s.

^{1}H-NMR (400 MHz, $CDCl_3$ (filtered through basic alumina)) δ_H 7.52 – 7.45 (m, 2H), 7.31 – 7.23 (m, 2H), 7.12 – 7.03 (m, 1H), 5.32 (s, 1H), 4.02 – 3.95 (m, 2H), 3.51 (br s, 2H), 3.06 – 2.99 (m, 2H), 2.43 (br s, 1H).

^{13}C{^{1}H}-NMR (101 MHz, $CDCl_3$) δ_C 151.0 (C), 135.6 (C), 128.4 (CH), 128.2 (CH), 126.0 (CH), 106.7 (CH), 68.2 (CH_2), 48.6 (CH_2), 45.0 (CH_2).

LRMS (ES+): 176.1 [(M + H)$^+$, 100%], 133.1 [95, (M – CH_2CH_2O)$^+$].

HRMS (ES+): calcd for $C_{11}H_{14}NO$ [M + H]$^+$ 176.1075, found 176.1072.

***tert*-butyl (*Z*)-2-benzylidenemorpholine-4-carboxylate (S17):**

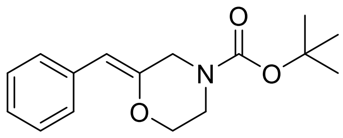

A solution of Boc$_2$O (37 mg, 0.17 mmol) in CH_2Cl_2 (0.7 mL) was added dropwise over 1 min to a solution of enol ether **S16** (25 mg, 0.14 mmol) and Et$_3$N (17 mg, 0.17 mmol) in CH_2Cl_2 (0.7 mL) at 0 °C. After 3 h, the reaction mixture was diluted with CH_2Cl_2 (2 mL) and washed with $NaHCO_3$ solution (3 × 2 mL). The organic layer was dried over $MgSO_4$, filtered and concentrated under reduced pressure. The crude mixture was purified by flash column chromatography (*n*-hexane/EtOAc 10/1 + 1 v/v% Et$_3$N) to afford carbamate **S17** as a colorless oil (17.5 mg, 44%).

Rf (*n*-hexane/EtOAc, 3/2) = 0.5.

ν_{max} (thin film/cm^{-1}) 2932 br w, 1696 s, 1158 s, 754 s, 697 s.

[n] NMR spectroscopic analysis of the crude reaction mixture revealed just the presence of product **S16**; however, the combined product from two reactions (using 50 mg and 250 mg of substrate **S7**) was purified once by column chromatography (CH_2Cl_2/MeOH 20/1) to afford **S16** in 61% combined yield.

^{1}H-NMR (400 MHz, $CDCl_3$ (filtered through basic alumina)) δ_H 7.58 – 7.53 (m, 2H), 7.32 – 7.26 (m, 2H), 7.19 – 7.09 (m, 1H), 5.42 (s, 1H), 4.26 – 4.12 (stack, 4H), 3.65 – 3.57 (m, 2H), 1.49 (s, 9H).
^{13}C{^{1}H}-NMR (101 MHz, $CDCl_3$) δ_C 154.4 (C), 149.0 (C), 135.5 (C), 128.2 (2 × CH, resonance overlap as evidenced by analysis of the ^{13}C{^{1}H}-NMR spectrum), 125.9 (CH), 105.2 (CH), 80.4 (C), [65.5, 64.8 (CH_2)], [45.7, 44.1 (CH_2)], [43.8, 42.7 (CH_2)], [28.4, 28.3 (CH_3)].
LRMS (AP+): 218.1 [$(M – t\text{-Bu})^+$, 50%], 176.15 [100, $(M – Boc)^+$] 91.1 [25, $(C_7H_7)^+$].
HRMS (AP+): calcd for $C_{12}H_{12}NO_3$ $[M – t\text{-Bu}]^+$ 218.0817, found 218.0822.

Attempted synthesis of S17 from iodo alcohol S8

Following general procedure A (p 38), $Ni(COD)_2$ (4.5 mg, 0.016 mmol, 6 mol%), activated Zn powder (32 mg, 0.50 mmol) and Et_3N (38 µL, 0.27 mmol) were added to a solution of iodo alcohol **S8** (0.10 g, 0.25 mmol) in MeCN (1.7 mL) at rt. After 24 h, TLC analysis of the reaction mixture revealed the presence of starting material only. The reaction mixture was therefore heated at 60 °C (in sand). As there was no evidence of product formation after heating for 24 h, the reaction mixture was worked up and the starting material was recovered.

(*Z*)-2-benzylidene-4-tosylmorpholine (S18):

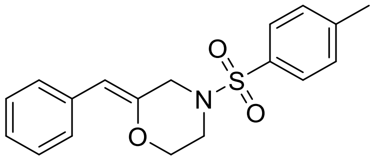

TsCl (0.16 g, 0.86 mmol) was added to a solution of Et_3N (0.17 g, 1.7 mmol), DMAP (11 mg, 0.09 mmol, 10 mol%) and enol ether **S7** (0.15 g, 0.86 mmol) in CH_2Cl_2 (4.3 mL) at 0 °C. After 1.5 h, the reaction mixture was diluted with CH_2Cl_2 (10 mL) and washed sequentially with H_2O (10 mL), brine (10 mL), dried over $MgSO_4$, filtered and concentrated under reduced pressure to give the crude product, which was purified by flash column chromatography (*n*-hexane/EtOAc 6/1 to 3/2 + 1 v/v% Et_3N) to afford sulfonamide **S18** as a pale orange solid (114 mg, 40%).
R_f (*n*-hexane/EtOAc, 3/2) = 0.7.
ν_{max} (neat/cm^{-1}) 2934 br w, 1671 m, 1339 br, 1158 s.
^{1}H-NMR (400 MHz, $CDCl_3$ (filtered through basic alumina)) δ_H 7.74 – 7.65 (m, 2H), 7.54 – 7.47 (m, 2H), 7.38 – 7.33 (m, 2H), 7.31 – 7.25 (m, 2H), 7.20 – 7.14 (m, 1H), 5.54 (s, 1H), 4.12 – 4.04 (m, 2H), 3.70 (br s, 2H), 3.30 – 3.17 (m, 2H), 2.44 (s, 3H).
^{13}C{^{1}H}-NMR (101 MHz, $CDCl_3$) δ_C 146.2 (C), 144.2 (C), 134.7 (C), 132.2 (C), 129.9 (CH), 128.6 (CH), 128.2 (CH), 128.0 (CH), 126.6 (CH), 109.9 (CH), 66.1 (CH_2), 48.3 (CH_2), 45.1 (CH_2), 21.6 (CH_3).

LRMS (ES+): 398.1 [(M + Na + HCOOH)$^+$, 20%], 352.1 [100, (M + Na)$^+$], 330.1 [20, (M + H)$^+$].

HRMS (ES+): calcd for $C_{18}H_{19}NO_3SNa$ [M + Na]$^+$ 352.0983, found: 352.0986.

Attempted synthesis of S18 from iodo alcohol S9

Following general procedure A (p 38), Ni(COD)$_2$ (1.5 mg, 0.005 mmol, 5 mol%), activated Zn powder (14 mg, 0.22 mmol) and Et_3N (17 µL, 0.12 mmol) were added to a solution of iodo alcohol **S9** (50 mg, 0.11 mmol) in MeCN (0.7 mL) at rt. After 24 h, TLC analysis of the reaction mixture revealed the presence of starting material only. The reaction mixture was therefore heated at 60 °C (in sand). As there was no evidence of product formation after heating for 40 h, the reaction mixture was worked up and the starting material was recovered.

2-(allyl(benzyl)amino)ethan-1-ol (S19):

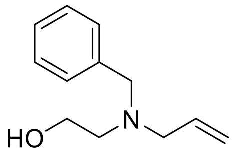

K_2CO_3 (3.83 g, 27.7 mmol) and allyl bromide (335 mg, 2.77 mmol) were added to a solution of *N*-benzylethanolamine (503 mg, 3.32 mmol) in MeCN (19 mL) at rt. After 19 h, the reaction mixture was filtered through a Celite plug, which was washed with EtOAc (2 × 10 mL). The filtrate was concentrated under reduced pressure. The resulting crude product was dissolved in EtOAc (20 mL) and washed with H_2O (2 × 10 mL). The organic layer was washed with brine (10 mL), dried over $MgSO_4$, filtered, concentrated under reduced pressure and the crude product was purified by column chromatography (*n*-hexane/EtOAc, 3/2) to produce aminoalcohol **S19** as a colorless oil (497 mg, 92%).

R_f (*n*-heptane/EtOAc, 3/2) = 0.2.

^{1}H-NMR (400 MHz, $CDCl_3$) δ_H 7.36 – 7.22 (stack, 5H), 6.04 – 5.78 (m, 1H), 5.25 – 5.18 (stack, 2H), 3.64 (s, 2H), 3.61 – 3.55 (m, 2H), 3.14 (dt, J = 6.5, 1.3 Hz, 2H), 2.80 – 2.53 (stack, 3H).

^{13}C{^{1}H}-NMR (101 MHz, $CDCl_3$) δ_H 138.8 (C), 135.1 (CH), 129.0 (CH), 128.4 (CH), 127.2 (CH), 118.2 (CH_2), 58.4 (CH_2), 58.0 (CH_2), 56.5 (CH_2), 54.6 (CH_2).

LRMS (EI): 160.1 [(M – CH_2OH)$^+$, 90%], 91.0 [100, [C_7H_7]$^+$].

Data were in agreement with those reported in the literature.[23]

2.3 Aminoalcohols

2.3.1 General procedure B – Benzyl protection of 1° amines

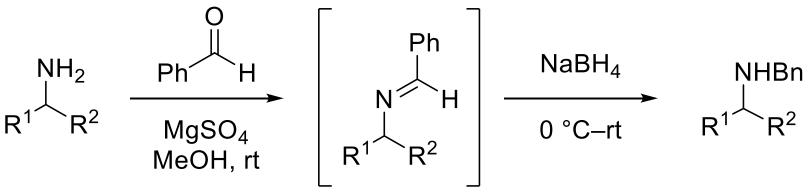

Following a procedure reported by Paquin,[17] $MgSO_4$ (56 mmol, 2.0 eq) and benzaldehyde (28 mmol, 1.0 eq) were added to a solution of the 1° amine (42 mmol, 1.5 eq) in MeOH (55 mL, 0.5 mmol mL^{-1}) at rt. After 16–24 h (typical reaction time), the solution was cooled to 0 °C and $NaBH_4$ (34 mmol, 1.2 eq) was added portionwise. After 0.5–4 h (typical reaction time) at 0 °C, H_2O (0.5 × reaction volume) was added portionwise over 5 min and the mixture was filtered through a sinter. The residue was washed sequentially with H_2O (0.5 × reaction volume) and EtOAc (2 × 0.5 × reaction volume). The filtrate was extracted with EtOAc (2 × reaction volume). The combined organic layers were washed with brine (1 × reaction volume), dried over Na_2SO_4, filtered and concentrated under reduced pressure to give the corresponding benzylamine.

4-(benzylamino)butan-1-ol (S34):

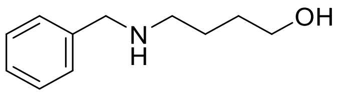

Following general procedure B (p 43), benzaldehyde (100 mg, 0.94 mmol) and $MgSO_4$ (227 mg, 1.88 mmol) were added to a solution of 4-aminobutanol (**S33**) (101 mg, 1.13 mmol) in MeOH (1.9 mL) at rt. After 20 h, the reaction mixture was cooled to 0 °C and $NaBH_4$ (39.0 mg, 1.04 mmol) was added portionwise over 1 min to the mixture. After 4 h at 0 °C, H_2O (1 mL) was added and the reaction mixture was worked up according to the general procedure to produce aminoalcohol **S34** as a pale-yellow oil (167 mg, quant.), which was used in the next step without further purification.

^{1}H-NMR (400 MHz, $CDCl_3$) δ_H 7.32 – 7.11 (stack, 5H), 3.71 (s, 2H), 3.66 – 3.56 (stack, 2H), 3.57 – 3.46 (m, 2H), 2.70 – 2.55 (m, 2H), 1.66 – 1.48 (stack, 4H).

^{13}C{^{1}H}-NMR (101 MHz, $CDCl_3$) δ_C 139.2 (C), 128.6 (CH), 128.4 (CH), 127.3 (CH), 62.7 (CH_2), 53.9 (CH_2), 49.3 (CH_2), 32.5 (CH_2), 28.7 (CH_2).

LRMS (ES+): 180.1 [$(M + H)^+$, 100%], 91.1 [10, $[C_7H_7]^+$].

Data were in agreement with those reported in the literature.[24]

(*S*)-1-(benzylamino)propan-2-ol (33):

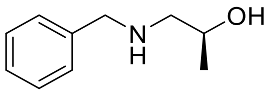

Following general procedure B (p 43), benzaldehyde (3.00 g, 28.3 mmol) and $MgSO_4$ (6.81 g, 56.5 mmol) were added to a solution of (*S*)-1-aminopropan-2-ol (2.55 g, 33.9 mmol) in MeOH (55 mL) at rt. After 16 h, the reaction mixture was cooled to 0 °C and $NaBH_4$ (1.18 g, 31.1 mmol) was added portionwise over 5 min. After 30 min at 0 °C, H_2O (25 mL) was added and the reaction mixture was worked up according to the general procedure to produce aminoalcohol **33** as a colorless oil (4.67 g, quant.), which was used in the next step without further purification.

^{1}H-NMR (400 MHz, $CDCl_3$) δ_H 7.40 – 7.23 (stack, 5H), 3.90 – 3.76 (stack, 3H), 2.74 (dd, *J* = 12.0, 3.0 Hz, 1H), 2.70 – 2.51 (stack, 2H), 2.46 (dd, *J* = 12.0, 9.4 Hz, 1H), 1.17 (d, *J* = 6.2 Hz, 3H).

^{13}C{^{1}H}-NMR (101 MHz, $CDCl_3$) δ_C 140.1 (C), 128.5 (CH), 128.1 (CH), 127.1 (CH), 65.7 (CH_2), 56.3 (CH), 53.7 (CH_2), 20.5 (CH_3).

LRMS (ES+): 166.0 [$(M + H)^+$, 100%].

Data were in agreement with those reported in the literature.[25]

(*S*)-2-(benzylamino)propan-1-ol (35):

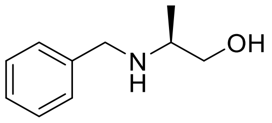

Following general procedure B (p 43), benzaldehyde (3.00 g, 28.3 mmol) and $MgSO_4$ (6.81 g, 56.5 mmol) were added to a solution of (*S*)-alaninol (2.55 g, 33.9 mmol) in MeOH (55 mL) at rt. After 16 h, the reaction mixture was cooled to 0 °C and $NaBH_4$ (1.18 g, 31.1 mmol) was added portionwise over 5 min. After 30 min at 0 °C, H_2O (25 mL) was added and the reaction mixture was worked up according to the general procedure to produce aminoalcohol **35** as a colorless oil, which was used in the next step without further purification (4.67 g, quant.).

^{1}H-NMR (400 MHz, $CDCl_3$) δ_H 7.48 – 7.14 (stack, 5H), 3.89 (A of AB, $J_{A–B}$ = 13.0 Hz, 1H), 3.75 (B of AB, $J_{B–A}$ = 13.0 Hz, 1H), 3.59 (dd, *J* = 10.6, 4.1 Hz, 1H), 3.28 (dd, *J* = 10.6, 7.0 Hz, 1H), 2.95 – 2.80 (m, 1H), 2.51 – 1.95 (stack, 2H), 1.11 (d, *J* = 6.5 Hz, 3H).

^{13}C{^{1}H}-NMR (101 MHz, $CDCl_3$) δ_C 140.3 (C), 128.5 (CH), 128.2 (CH), 127.1 (CH), 65.5 (CH_2), 53.8 (CH_2), 51.1 (CH), 17.1 (CH_3).

LRMS (ES+): 165.9 [$(M + H)^+$, 100%].

Data were in agreement with those reported in the literature.[25]

2.4 Chloromethyl-substituted heterocycles

4-benzyl-2-(chloromethyl)morpholine (1):

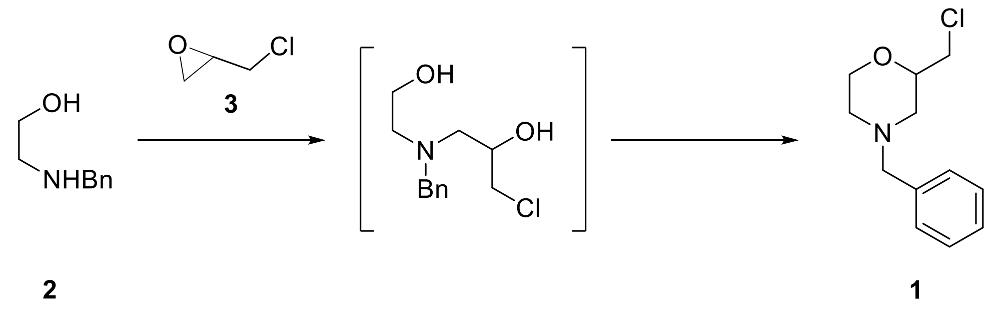

Rac-Epichlorohydrin (**3**) (30 mL, 0.37 mol) was added via a dropping funnel (~25 min) to *N*-benzylethanolamine (**2**) (58 g, 0.37 mol) at rt. After 16 h, H_2SO_4 (20 mL, 0.55 mol, 98%) was added dropwise (~25 min) to the viscous diol intermediate, while gradually heating in an oil bath (***Caution!*** *Use silicone oil with a flashpoint that exceeds the maximum reaction temperature, i.e., 170 °C in this experiment.*) to 90 °C to avoid solidification of the reaction mixture. After the addition of the acid, the mixture was heated at 170 °C. After 6 h, further H_2SO_4 (9.5 mL, 0.27 mol, 98%) was added to the heated mixture via a dropping funnel at 170 °C. After 18 h, the reaction mixture was left to cool to ~90 °C and ice-H_2O (250 mL) was added slowly over 3 min (***Caution!*** *The addition of water to concentrated acid is a highly exothermic process, so ensure the reaction mixture is stirring efficiently throughout.*) Note: the addition of further acid and ice-H_2O prevented the reaction mixture from turning into a sparingly soluble black solid.] The mixture was diluted with H_2O (1.0 L) and the pH adjusted to 8–9 by the addition of solid NaOH. The resulting mixture was extracted with EtOAc (3 × 500 mL). The combined organic layers were washed with brine (500 mL), dried over Na_2SO_4, filtered and concentrated under reduced pressure to give morpholine **1** as a dark-brown oil (71.9 g, 87%). The crude product was used in the next step without further purification.

R_f (*n*-hexane/EtOAc 3/2) = 0.6.

^{1}H-NMR (400 MHz, $CDCl_3$) δ_H 7.37 – 7.23 (stack, 5H), 3.91 (ddd, *J* = 11.3, 3.4, 1.9 Hz, 1H), 3.84 – 3.77 (m, 1H), 3.71 (td, *J* = 11.3, 2.6 Hz, 1H), 3.55 – 3.52 (stack, 3H), 3.50 (B of ABX, J_{B-A} = 11.6 Hz, J_{B-X} = 5.2 Hz, 1H), 2.84 (dt, *J* = 11.0, 2.0 Hz, 1H), 2.66 (ddd, *J* = 11.3, 4.0, 2.1 Hz, 1H), 2.21 (td, *J* = 11.3, 3.4 Hz, 1H), 2.01 (dd, *J* = 11.2, 9.7 Hz, 1H).

^{13}C{^{1}H}-NMR (101 MHz, $CDCl_3$) δ_C 137.5 (C), 129.1 (CH), 128.4 (CH), 127.3 (CH), 75.2 (CH), 66.9 (CH_2), 63.2 (CH_2), 55.9 (CH_2), 52.7 (CH_2), 45.0 (CH_2).

LRMS (EI): 227.1 [(M[^{37}Cl])$^+$, 10%], 225.1 [25, (M[^{35}Cl])$^+$], 190.1 [100, (M – Cl)$^+$], 91.1 (85, [C_7H_7]$^+$).

Data were in agreement with those reported in the literature.[26]

4-benzyl-2-(chloromethyl)-1,4-oxazepane (S32):

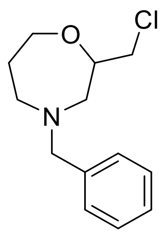

Rac-Epichlorohydrin (**3**) (3.0 mL, 38 mmol) was added via a dropping funnel (10 min) to *N*-benzyl aminopropanol (**S31**) (4.35 g, 25.0 mmol) at rt. After 16 h, H_2SO_4 (1.5 mL, 38 mmol, 98%) was added dropwise (over 10 min) to the viscous diol intermediate while gradually heating in an oil bath (***Caution!*** *Use silicone oil with a flashpoint that exceeds the maximum reaction temperature, i.e., 170 °C in this experiment.*) to 90 °C to avoid solidification of the reaction mixture. After the addition of the acid, the mixture was heated at 170 °C. After 6 h, further H_2SO_4 (1.5 mL, 38 mmol, 98%) was added to the heated mixture via a dropping funnel at 170 °C. After a further 20 h, the reaction mixture was cooled to ~90 °C and ice-H_2O (50 mL) was added over 2 min (***Caution!*** *The addition of water to concentrated acid is a highly exothermic process, so ensure the reaction mixture is stirring efficiently throughout.*). Note, the addition of further acid and the ice-H_2O prevented the reaction mixture from turning into a sparingly soluble black solid. The mixture was diluted with H_2O (150 mL) and the pH adjusted to 8–9 by the addition of solid NaOH. The resulting mixture was extracted with EtOAc (3 × 50 mL). The combined organic layers were washed with brine (50 mL), dried over Na_2SO_4, filtered and concentrated under reduced pressure to give oxazepane **S32** as a dark-brown oil (3.06 g, 51%). The crude product was used in the next step without further purification.

R_f (*n*-hexane/EtOAc 3/2) = 0.5.

ν_{max} (thin film/cm^{-1}): 2932 m, 1728 w, 1631 m, 1296 m, 1126 m, 1092 s.

^{1}H-NMR (400 MHz, $CDCl_3$) δ_H 7.36 – 7.30 (stack, 4H), 7.29 – 7.23 (m, 1H), 3.98 – 3.90 (m, 1H), 3.89 – 3.79 (stack, 2H), 3.68 (s, 2H), 3.47 (A of ABX, J_{A-B} = 11.1 Hz, J_{A-X} = 5.9 Hz, 1H), 3.41 (B of ABX, J_{B-A} 11.1 = Hz, J_{B-X} = 5.9 Hz, 1H), 2.92 (ddd, J = 13.6, 2.5, 1.2 Hz, 1H), 2.78 – 2.72 (m, 1H), 2.68 – 2.56 (stack, 2H), 1.98 – 1.79 (m, 2H).

^{13}C{^{1}H}-NMR (101 MHz, $CDCl_3$) δ_C 139.1 (C), 128.8 (CH), 128.3 (CH), 127.1 (CH), 78.3 (CH), 67.5 (CH_2), 62.8 (CH_2), 58.6 (CH_2), 54.3 (CH_2), 45.8 (CH_2), 30.6 (CH_2).

LRMS (ES+): 242.1 [(M[^{37}Cl] + H)$^+$, 40%], 240.1 [100, (M[^{35}Cl] + H)$^+$], 204.1 (15).

HRMS (ES+): calcd for $C_{13}H_{19}{}^{35}ClNO$ [M + H]$^+$ 240.1150, found 240.1151.

Data were in agreement with those reported in the literature.[27]

(6*S*)-4-benzyl-2-(chloromethyl)-6-methylmorpholine (32):

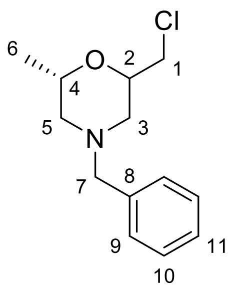

Rac-Epichlorohydrin (**3**) (2.3 mL, 29 mmol) was added dropwise over ~1 min to (*S*)-1-(benzylamino)propan-2-ol (**33**) (3.40 g, 19.6 mmol) at rt. After 3 days, H_2SO_4 (1.0 mL, 20 mmol, 98%) was added dropwise over ~2 min to the viscous diol intermediate, while gradually heating in an oil bath (***Caution!*** *Use silicone oil with a flashpoint that exceeds the maximum reaction temperature, i.e., 170 °C in this experiment.*) to 90 °C to avoid solidification of the reaction mixture. After the addition of the acid, the mixture was heated at 170 °C. After 2 h, further H_2SO_4 (1.0 mL, 20 mmol, 98%) was added dropwise over ~1 min to the heated mixture at 170 °C. After a further 20 h, the reaction mixture was cooled to ~90 °C and ice-H_2O (10 mL) was added over 3 min (***Caution!*** *The addition of water to concentrated acid is a highly exothermic process, so ensure the reaction mixture is stirring efficiently throughout.*). Note, the addition of further acid and ice-H_2O prevented the reaction mixture from turning into a sparingly soluble black solid. Then the pH of the mixture was adjusted to 8–9 by the addition of NaOH (150 mL, 2 M). The resulting mixture was extracted with EtOAc (3 × 150 mL). The combined organic layers were washed with brine (200 mL), dried over Na_2SO_4, filtered and concentrated under reduced pressure to give morpholine **32** as a dark-brown oil (3.47 g, 74%, d.r. ~2:1). The crude product was used in the next step without further purification.

^{1}H-NMR (400 MHz, $CDCl_3$, ~2:1 mixture of diastereomers based on the relative integration of the resonances for H-3b at δ_H 2.43 ppm and 1.91 ppm) δ_H 7.42 – 7.21 (stack, 5H, Ph), 4.05 – 3.89 (m, 1H, H-4), 3.88 – 3.61 (stack, 1.8H, H-1 min, H-2), 3.60 – 3.40 (stack, 3.2H, H-1 maj, H-7), 2.94 (app dt, *J* = 11.0, 2.0 Hz, 0.6H, H-3a maj), 2.74 (app dt, *J* = 11.3, 2.0 Hz, 0.6H, H-5a maj), 2.70 – 2.59 (stack, 0.8H, H-3a min, H-5a min), 2.43 (dd, *J* = 11.6, 3.6 Hz, 0.4H, H-3b min), 2.02 (dd, *J* = 11.2, 8.1 Hz, 0.4H, H-5b min),1.91 (app t, *J* = 10.6 Hz, 0.6H, H-3b maj), 1.81 (app t, *J* = 10.7 Hz, 0.6H, H-5b maj), 1.23 – 1.16 (stack, 3H [including 1.20 (d, *J* = 6.2 Hz, 1.2H, H-6 min), 1.18 (d, *J* = 6.4 Hz, 1.8H, H-6 maj)], H-6).

^{13}C{^{1}H}-NMR (101 MHz, $CDCl_3$, mixture of diastereomers) δ_C 137.9 (C, C-8 min), 137.6 (C, C-8 maj), 129.2 (CH, C-9 maj), 128.8 (CH, C-9 min), 128.4 (CH, C-10 maj), 128.3 (CH, C-10 min), 127.3 (CH, C-11 maj), 127.2 (CH, C-11 min), 75.4 (CH, C-2 maj), 72.28 (CH, C-4 maj), 72.25 (CH, C-2 min), 66.9 (CH, C-4 min),

63.0 (CH_2, C-7 maj), 62.9 (CH_2, C-7 min), 59.3 (CH_2, C-5 maj), 59.2 (CH_2, C-5 min), 55.6 (CH_2, C-3 maj), 53.6 (CH_2, C-3 min), 45.0 (CH_2, C-1 maj), 43.7 (CH_2, C-1 min), 19.0 (CH_3, C-6 maj), 18.7 (CH_3, C-6 min). LRMS (ES+): 241.9 [(M[^{37}Cl] + H)$^+$, 40%], 239.9 [100, (M[^{35}Cl] + H)$^+$].

(5*S*)-4-benzyl-2-(chloromethyl)-5-methylmorpholine (36):

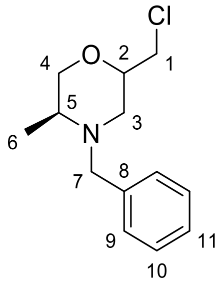

Rac-Epichlorohydrin (**3**) (1.0 mL, 13 mmol) was added dropwise over ~1 min to a solution of (*S*)-2-(benzylamino)propan-1-ol (**35**) (1.10 g, 6.33 mmol) in CH_2Cl_2 (2.5 mL) at rt.[o] After 2 days, extra *rac*-epichlorohydrin (0.31 mL, 3.9 mmol) was added to the mixture. After a further 5 days, H_2SO_4 (1.3 mL, 25 mmol, 98%) was added dropwise over ~2 min to the viscous diol intermediate, while gradually heating in an oil bath (***Caution!*** *Use silicone oil with a flashpoint that exceeds the maximum reaction temperature, i.e., 170 °C in this experiment.*) to 90 °C to avoid solidification of the reaction mixture. After the addition of the acid, the mixture was heated at 170 °C. After 16 h, the reaction mixture was cooled to ~90 °C and ice-H_2O (5 mL) was added over 1 min (***Caution!*** *The addition of water to concentrated acid is a highly exothermic process, so ensure the reaction mixture is stirring efficiently throughout.*) Then, the pH of the mixture was adjusted to 8–9 by the addition of NaOH (100 mL, 2 M) and EtOAc (100 mL) was added. The resulting biphasic system was filtered over cotton wool into a separating funnel and the solid filtrate was suspended in a mixture of NaOH (2 M) and EtOAc (50 mL of a 1/1 mixture) and sonicated for 5 min. This mixture was filtered and collected with the previous mother liquors. The solids were suspended and sonicated again in the same way as before and this procedure was repeated twice. After the final filtration, the solids were removed by filtration and the biphasic system was separated. The aqueous phase was extracted with EtOAc (2 × 50 mL). The organic layers were combined, washed sequentially with H_2O (100 mL) and brine (100 mL), dried over Na_2SO_4, filtered and concentrated under reduced pressure to give morpholine **36** as a dark-brown oil (970 mg, 62%, d.r. ~7:5). The crude product was used in the next step without further purification.

[o] The CH_2Cl_2 was necessary to facilitate stirring of the gummy intermediate.

Major diastereomer

^{1}H-NMR (400 MHz, $CDCl_3$) δ_H 7.47 – 7.16 (stack, 5H, Ph), 3.88 – 3.61 (stack, 5H, H-1a, H-2, H-4, H-7a), 3.59 – 3.49 (stack, 2H, H-1b, H-7b), 2.87 – 2.69 (m, 1H, H-5), 2.60 – 2.35 (stack, 2H, H-3), 1.12 (d, *J* = 6.5 Hz, 3H, H-6).

Minor diastereomer

^{1}H-NMR (400 MHz, $CDCl_3$) δ_H 7.47 – 7.16 (stack, 5H, Ph), 4.17 (d, *J* = 13.3 Hz, 1H, H-7a), 3.88 – 3.61 (stack, 2H, H-2, H-4a), 3.45 (stack, 2H, H-1), 3.39 (dd, *J* = 11.3, 10.3 Hz, 1H, H-4b), 3.11 (d, *J* = 13.3 Hz, 1H, H-7b), 2.87 – 2.69 (m, 1H, H-3a), 2.60 – 2.35 (m, 1H, H-5), 1.97 (dd, *J* = 11.5, 10.3 Hz, 1H, H-3b), 1.12 (d, *J* = 6.2 Hz, 3H, H-6).

^{13}C{^{1}H}-NMR (101 MHz, $CDCl_3$) δ_C 138.6 (C, C-8 maj), 138.2 (C, C-8 min), 129.1 (CH, C-9 min), 128.7 (CH, C-9 maj), 128.3 (CH, C-10, for both diastereoisomers), 127.1 (CH, C-11 for both diastereoisomers), 75.7 (CH, C-2 min), 75.1 (CH, C-2 maj), 73.1 (CH_2, C-4 min), 71.4 (CH_2, C-4 maj), 58.7 (CH_2, C-7 maj), 58.0 (CH_2, C-7 min), 55.2 (CH, C-5 min), 54.4 (CH_2, C-3 min), 52.4 (CH, C-5 maj), 49.1 (CH_2, C-3 maj), 45.1 (CH_2, C-1 min), 44.6 (CH_2, C-1 maj), 15.2 (CH_3, C-6 min), 9.3 (CH_3, C-6 maj).

LRMS (ES+): 242.0 [(M[^{37}Cl] + H)$^+$, 40%], 240.0 [100, (M[^{35}Cl] + H)$^+$].

2.5 Enol ethers

2.5.1 General procedure C – Dehydrochlorination to form enol ethers

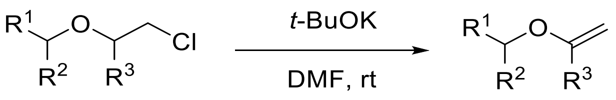

t-BuOK (0.3 mol, 3.0 eq) was added portionwise to a solution of the β-chloro ether (0.1 mol, 1.0 eq) in DMF (250 mL, 0.4 mmol mL^{-1}) at rt. After 30–60 min (typical reaction time), the reaction mixture was diluted with H_2O (2 × reaction volume) and extracted with EtOAc (3 × reaction volume). The organic layers were combined, washed sequentially with H_2O (2 × reaction volume) and brine (1 × reaction volume), dried over Na_2SO_4, filtered and concentrated under reduced pressure. The crude product was purified by flash column chromatography to give the enol ether product.

4-benzyl-2-methylidenemorpholine (4):

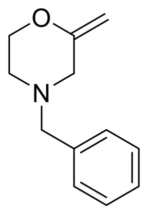

Following general procedure C (p 49), *t*-BuOK (44.7 g, 0.399 mol) was added over 20 min to a solution of chloride **1** (30.0 g, 0.133 mol) in DMF (350 mL) at rt. Given the scale of the reaction, a slight exotherm was observed during the addition of *t*-BuOK, therefore the reaction vessel was cooled with an ice bath to prevent a temperature rise above rt. After 30 min, the reaction was worked up according to the general procedure and the crude product was purified by flash column chromatography (*n*-hexane/EtOAc 6/1 + 1 v/v% Et_3N) to produce enol ether **4** as a light-yellow oil (17.5 g, 69%).

R_f (*n*-heptane/EtOAc, 3/2) = 0.5.

^{1}H-NMR (400 MHz, $CDCl_3$ (filtered through basic alumina)) δ_H 7.36 – 7.24 (stack, 5H), 4.42 – 4.38 (m, 1H), 4.15 – 4.08 (m, 1H), 3.95 – 3.86 (m, 2H), 3.52 (s, 2H), 2.99 (br s, 2H), 2.57 – 2.50 (m, 2H).

^{13}C{^{1}H}-NMR (101 MHz, $CDCl_3$) δ_C 155.9 (C), 137.3 (C), 129.2 (CH), 128.4 (CH), 127.3 (CH), 92.0 (CH_2), 67.8 (CH_2), 63.1 (CH_2), 55.2 (CH_2), 52.0 (CH_2).

LRMS (EI): 189.1 [$(M)^+$, 100%], 98.1 [70, $(M – Bn)^+$], 91.1 [65, $(C_7H_7)^+$].

Data were in agreement with those reported in the literature.[5]

4-benzyl-2-methylidene-1,4-oxazepane (21):

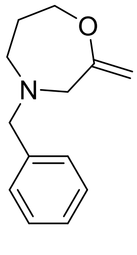

Following general procedure C (p 49), *t*-BuOK (2.11 g 18.8 mmol) was added over 5 min to a solution of chloride **S32** (1.50 g, 6.26 mmol) in DMF (18 mL) at rt. After 30 min, the reaction was worked up according to the general procedure and the crude product was purified by flash column chromatography (gradient, 0–50% EtOAc in *n*-heptane + 1 v/v% Et_3N) to produce enol ether **21** as a light-yellow oil (554 mg, 44%).

R_f (*n*-heptane/EtOAc, 3/2) = 0.5.

ν_{max} (thin film/cm^{-1}): 2935 br s, 1724 w, 1632 br s, 1094 s.

^{1}H-NMR (400 MHz, $CDCl_3$ (filtered through basic alumina)) δ_H 7.35 – 7.14 (stack, 5H), 4.21 (s, 1H), 4.05 – 3.92 (stack, 2H), 3.79 (s, 1H), 3.64 (s, 2H), 3.36 (s, 2H), 2.86 – 2.73 (m, 2H), 1.90 – 1.74 (m, 2H).

^{13}C{^{1}H}-NMR (101 MHz, $CDCl_3$) δ_C 160.3 (C), 139.0 (C), 128.9 (CH), 128.3 (CH), 127.0 (CH), 90.8 (CH_2), 68.2 (CH_2), 57.2 (CH_2), 55.6 (CH_2), 55.3 (CH_2), 29.3 (CH_2).

LRMS (ASAP+): 204.1 [$(M + H)^+$, 100%].

HRMS (ASAP+): calcd for $C_{13}H_{18}NO$ $[M + H]^+$ 204.1388, found 204.1393.

(*S*)-4-benzyl-2-methyl-6-methylidenemorpholine (34):

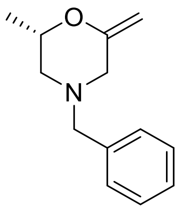

Following general procedure C (p 49), *t*-BuOK (2.11 g 18.8 mmol) was added over 5 min to a solution of chloride **32** (1.50 g, 6.26 mmol) in DMF (18 mL) at rt. After 30 min, the reaction was worked up according to the general procedure and the crude product was purified by flash column chromatography (gradient, 0–50% EtOAc in *n*-heptane + 1 v/v% Et_3N) to produce enol ether **34** as a light-yellow oil (1.05 g, 71%).

R_f (*n*-heptane/EtOAc, 3/2) = 0.6.

ν_{max} (thin film/cm^{-1}): 2945 w, 2762 w, 1654 br s, 1454 m, 1273 m, 1065 s.

^{1}H-NMR (400 MHz, C_6D_6) δ_H 7.39 – 7.31 (m, 2H), 7.28 – 7.19 (m, 2H), 7.19 – 7.11 (m, 1H), 4.70 – 4.63 (m, 1H), 4.12 – 4.08 (m, 1H), 3.98 – 3.83 (m, 1H), 3.28 (A of AB, $J_{A–B}$ = 13.0 Hz, 1H), 3.20 (B of AB, $J_{B–A}$ = 13.0 Hz, 1H), 3.14 (dd, J = 12.2, 1.7 Hz), 2.68 – 2.54 (m, 1H), 2.49 (dt, J = 11.5, 2.1 Hz, 1H), 1.78 (dd, J = 11.5, 9.5 Hz, 1H), 1.03 (d, J = 6.3 Hz, 3H).

^{13}C{^{1}H}-NMR (101 MHz, C_6D_6) δ_C 156.5 (C), 138.0 (C), 128.9 (CH), 128.3 (CH), 127.2 (CH), 91.2 (CH_2), 72.9 (CH), 62.6 (CH_2), 58.2 (CH_2), 54.7 (CH_2), 18.6 (CH_3).

LRMS (ASAP+): 204.1 [$(M + H)^+$, 100%].

HRMS (ASAP+): calcd for $C_{13}H_{18}NO$ $[M + H]^+$ 204.1388, found 204.1394.

(*S*)-4-benzyl-5-methyl-2-methylidenemorpholine (39):

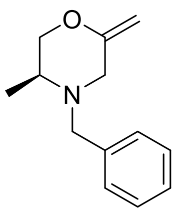

Following general procedure C (p 49), *t*-BuOK (1.69 g 15.0 mmol) was added over 5 min to a solution of chloride **36** (1.20 g, 5.01 mmol) in DMF (14 mL) at rt. After 10 min, the reaction was worked up according to the general procedure and the crude product was purified by flash column chromatography (gradient, 0–50% EtOAc in *n*-heptane + 1 v/v% Et_3N) to produce enol ether **39** as a colorless oil (825 mg, 81%).

R_f (*n*-heptane/EtOAc, 3/2) = 0.6.

ν_{max} (thin film/cm^{-1}): 2972 m, 2796 w, 1949 w, 1742 w, 1658 br s, 1455 s.

^{1}H-NMR (400 MHz, $CDCl_3$ (filtered through basic alumina)) δ_H 7.44 – 7.15 (stack, 5H), 4.34 (s, 1H), 4.07 – 3.95 (stack, 2H), 3.91 (dd, *J* = 10.8, 3.5 Hz, 1H), 3.58 (dd, *J* = 10.8, 9.1 Hz, 1H), 3.21 (d, *J* = 13.1 Hz, 1H), 3.18 (d, *J* = 13.0 Hz, 1H), 2.75 (d, *J* = 13.0 Hz, 1H), 2.71 – 2.61 (m, 1H), 1.12 (d, *J* = 6.3 Hz, 3H).

^{13}C{^{1}H}-NMR (101 MHz, $CDCl_3$) δ_C 156.3 (C), 138.1 (C), 129.1 (CH), 128.4 (CH), 127.2 (CH), 91.2 (CH_2), 72.7 (CH_2), 58.1 (CH_2), 54.4 (CH), 52.1 (CH_2), 14.3 (CH_3).

LRMS (CI): 204.1 [(M + H)$^+$, 100%], 131.0 (20).

HRMS (CI): calcd for $C_{13}H_{18}NO$ [M + H]$^+$ 204.1388, found 204.1395.

2.6 Spiroacetals

2.6.1 General procedure D – Iodoacetalization and spiroacetal formation

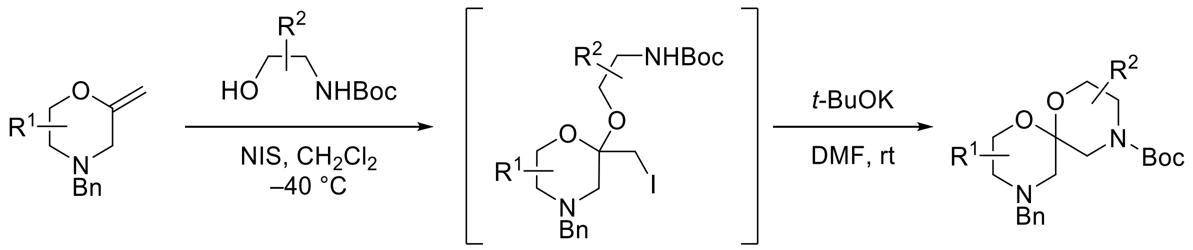

In an aluminum foil-covered flask and under an Ar atmosphere, NIS (1.0 mmol, 1.0 eq) was added portionwise (rate of addition specified for each reaction) to a cooled (–40 °C, dry ice in MeCN) solution of the Boc-protected aminoalcohol (1.2 mmol, 1.2 eq) and enol ether (1.0 mmol, 1.0 eq) in CH_2Cl_2 (7.0 mL, 0.15 mmol mL^{-1}). After the addition of the NIS, the color of the reaction mixture usually turned a shade of red. After 10–30 min (typical reaction time) at –40 °C, the reaction mixture was diluted with CH_2Cl_2 (1 × reaction volume) and washed sequentially with 10 wt% $Na_2S_2O_3$ solution (2 × reaction volume, the mixture usually lost its red color after these washes) and brine (1 × reaction volume). The organic phase was dried over Na_2SO_4, filtered and concentrated under reduced pressure. The crude product was purified by flash column chromatography (gradient, 0–100% EtOAc in *n*-heptane + 1 v/v% Et_3N) to give the iodo acetal intermediate, which was used directly in the next step: *t*-BuOK (2.0 mmol, 2.0 eq) was added portionwise (rate of addition specified for each reaction) to a solution of the iodo acetal intermediate in DMF (5 mL, 0.2 mmol mL^{-1}) at rt. After 0.5–24 h (typical reaction time) at rt, the reaction mixture was diluted with H_2O (1 × reaction volume) and extracted with EtOAc (2 × reaction volume). The combined organic phases were washed with brine (1 × reaction volume), dried over Na_2SO_4, filtered, concentrated under reduced pressure and the crude product was purified by flash column chromatography (gradient, 0–100% EtOAc in *n*-heptane) to give the spiroacetal product.

Unless stated otherwise, diastereoisomeric ratios were calculated based on the relative areas under the curve of the UV absorbance (215 nm) of basic or neutral LCMS measurements of each compound.

2.6.2 Iodoacetalization and unsubstituted spiroacetal formation

***tert*-butyl (2-((4-benzyl-2-(iodomethyl)morpholin-2-yl)oxy)ethyl)carbamate (6):**

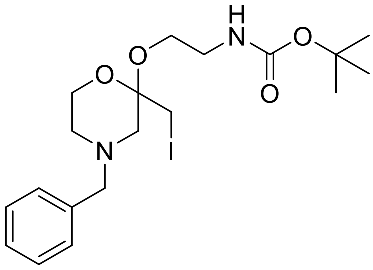

Following general procedure D (p 53), NIS (22.8 g, 92.4 mmol) was added over 15 min to a solution of *N*-Boc-ethanolamine (17.9 g, 111 mmol) and enol ether **4** (17.5 g, 92.4 mmol) in CH_2Cl_2 (620 mL) at –30 °C. After 15 min, the reaction was not complete as evidenced by TLC analysis; therefore, more NIS (2.28 g, 9.24 mmol) and *N*-Boc-ethanolamine (**5**) (1.79 g, 11.1 mmol) were added. After 30 min, the reaction was worked up according to the general procedure to provide iodo acetal **6** as a light-yellow oil (34.0 g, 77%).

R_f (*n*-heptane/EtOAc, 3/2) = 0.5.

ν_{max} (neat/cm^{-1}): 3356 m, 2974 w, 2814 w, 1709 s, 1162 s, 1059 s.

^{1}H-NMR (400 MHz, C_6D_6, resonance broadening observed because of rotamers) δ_H 7.28 – 7.02 (stack, 5H), 5.03 (br s, 1H), 3.62 – 3.51 (m, 1H), 3.36 – 3.13 (stack, 6H), 3.09 – 2.98 (stack, 3H), 2.74 (d, *J* = 11.4 Hz, 1H), 2.13 (d, *J* = 11.4 Hz, 1H), 2.06 – 1.97 (m, 1H), 1.86 – 1.76 (m, 1H), 1.45 (s, 9H).

^{13}C{^{1}H}-NMR (101 MHz, C_6D_6, rotamers not observed) δ_C 156.0 (C), 138.0 (C), 129.3 (CH), 128.6 (CH), 127.5 (CH), 95.6 (C), 78.6 (C), 62.7 (CH_2), 62.4 (CH_2), 60.1 (CH_2), 58.9 (CH_2), 51.7 (CH_2), 41.0 (CH_2), 28.6 (CH_3), 8.3 (CH_2).

LRMS (ES+): 477.1 [(M + H)$^+$, 100%], 316.0 [30, (M + H – OCH_2CH_2NHBoc)$^+$].

HRMS (ES+): calcd for $C_{19}H_{30}IN_2O_4$ [M + H]$^+$ 477.1250, found 477.1233.

***tert*-butyl 10-benzyl-1,7-dioxa-4,10-diazaspiro[5.5]undecane-4-carboxylate (7):**

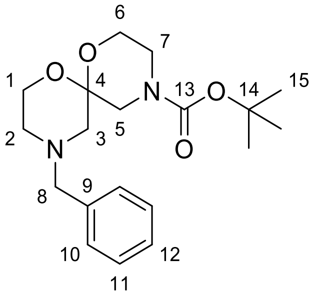

Following general procedure D (p 53), *t*-BuOK (16.0 g, 143 mmol) was added over 30 min to a solution of iodo acetal **6** (34.0 g, 71.3 mmol) in DMF (480 mL) at rt. After 1 h, the reaction was worked up

according to the general procedure to produce spiroacetal **7** as a yellow oil, which solidified upon storage at rt (24.4 g, 76% over the two steps). The product was used in the next step without further purification. Crystals of spiroacetal **7** suitable for analysis by X-ray analysis were grown by slow evaporation of a solution of **7** in $CDCl_3$, see Section 4.1.1 for full details.

R_f (*n*-heptane/EtOAc, 3/2) = 0.2.

MP: 83 – 85 °C

ν_{max} (neat/cm^{-1}): 2971 m, 2932 w, 1695 s, 1055 s.

^{1}H-NMR (400 MHz, C_6D_6, ~1:1 mixture of rotamers based on the relative integration of the resonances for H-5a at δ_H 4.45 – 4.30 ppm and H-7a at 3.67 – 3.57 ppm) δ_H 7.36 – 7.26 (stack, 2H, H-10), 7.20 – 7.12 (stack, 2H, H-11), 7.11 – 7.04 (stack, 1H, H-12), 4.45 – 4.30 (m, 0.5H, H-5a rotA), 4.12 – 3.92 (stack, 1H, H-5a rotB, H-7a rotA), 3.88 – 3.71 (stack, 2H, H-1a, H-6a), 3.67 – 3.57 (m, 0.5H, H-7a rotB), 3.50 – 3.30 (stack, 1H, H-1b), 3.26 – 3.10 (stack, 3H, H-6b, H-8), 2.80 – 2.58 (stack, 1H, H-7b), 2.58 – 2.41 (stack, 2H, H-3a, H-5b), 2.30 – 2.17 (stack, 1H, H-2a), 2.01 – 1.90 (stack, 1H, H-2b), 1.90 – 1.73 (stack, 1H, H-3b), 1.44 (br s, 9H, H-15).

^{13}C{^{1}H}-NMR (101 MHz, C_6D_6, mixture of rotamers) δ_C [155.0, 154.7 (C, C-13)], 138.5 (C, C-9), 129.2 (CH, C-10), 128.6 (CH, C-11), 127.4 (CH, C-12), [93.4, 92.7 (C, C-4)], 79.3 (C, C-14), 63.1 (CH_2, C-8), 60.8 (CH_2, broad, C-1), 59.3 (CH_2, C-6), 58.8 (CH_2, C-3), 52.6 (CH_2, C-2), [50.1, 48.6 (CH_2, C-5)], [43.9, 42.8 (CH_2, C-7)], 28.5 (CH_3, C-15).

LRMS (ES+): 349.2 [$(M + H)^+$, 100%], 293.1 [80, $(M + H – t\text{-Bu})^+$], 188.1 (20).

HRMS (ES+): calcd for $C_{19}H_{29}N_2O_4$ $[M + H]^+$ 349.2127, found 349.2136.

4-benzyl 10-(*tert*-butyl) 1,7-dioxa-4,10-diazaspiro[5.5]undecane-4,10-dicarboxylate (8):

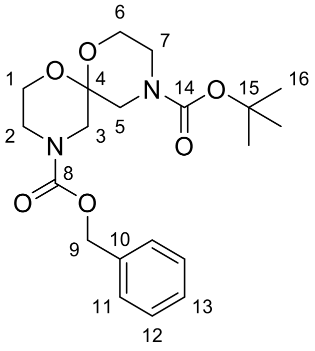

Under an Ar atmosphere, CbzCl (21 mL, 0.15 mol) was added dropwise via a dropping funnel over 2 h to a solution of benzylamine **7** (24.4 g, 70.1 mmol) in CH_2Cl_2 (350 mL) at rt. After 24 h, the reaction mixture was diluted with CH_2Cl_2 (100 mL) and extracted with $NaHCO_3$ solution (3 × 150 mL). The organic layer was dried over Na_2SO_4, filtered, and concentrated under reduced pressure. The crude product

was purified by flash column chromatography (*n*-heptane/EtOAc 4/1 + 1 v/v% Et_3N) to produce carbamate **8** as a light-yellow oil (26.6 g, 97%).

R_f (*n*-heptane/EtOAc, 3/2) = 0.4.

ν_{max} (neat/cm^{-1}): 2975 w, 2929 w, 1691 s, 1428 m, 1270 m, 1135 s, 1052 s.

^{1}H-NMR (400 MHz, C_6D_6, resonance broadening observed because of rotamers) δ_H 7.26 – 6.99 (stack, 5H, Ph), 5.23 – 4.93 (stack, 2H, H-9), 4.23 – 3.63 (stack, 3H, H-2a or H-7a, H-3a, H-5a), 3.62 – 3.41 (stack, 3H, H-1a, H-2a or H-7a, H-6a), 3.21 – 2.97 (stack, 2H, H-1b, H-6b), 2.81 – 2.25 (stack, 4H, H-2b, H-3b, H-5b, H-7b), 1.41 (s, 9H, H-16).

^{13}C{^{1}H}-NMR (101 MHz, C_6D_6, resonance broadening observed because of rotamers) δ_C [155.5, 155.2, 154.9, 154.6, (C, C-8, C-14)], [137.5, 137.4 (C, C-10)], [91.9, 91.3 (C, C-4)], 79.5 (C, C-15), [67.4, 67.3 (CH_2, C-9)], [59.5, 59.3 (CH_2, C-1, C-6)], [49.6, 49.3, 48.8, 48.2 (CH_2, C-3, C-5)], [43.5, 43.1, 43.0, 42.3 (CH_2, C-2, C-7)], 28.4 (CH_3, C-16), resonances for the aromatic CHs (C-11, C-12, C-13) overlapped with the C_6D_6 resonances, however HMBC and HSQC measurements confirmed their presence between δ_C 129.0 – 127.0 ppm.

LRMS (ASAP+): 337.1 [(M + H – *t*-Bu)$^+$, 100%], 293.2 [80, (M + H – Boc)$^+$], 188.1 (60).

HRMS (ASAP+): calcd for $C_{20}H_{28}N_2O_6$ [M]$^+$ 392.1947, found 392.1949.

***tert*-butyl 1,7-dioxa-4,10-diazaspiro[5.5]undecane-4-carboxylate (9):**

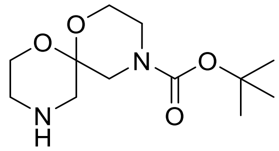

Under an Ar atmosphere, Pd/C (10 wt%, 2.01 g, 1.89 mmol, 6 mol%) was added to a degassed solution of carbamate **8** (12.8 g, 32.6 mmol) in THF/H_2O (4/1 mixture, 370 mL) at rt. The reaction vessel was sequentially evacuated and flushed with H_2 gas twice and the mixture then stirred under an H_2 atmosphere. After 20 h, the reaction vessel was evacuated and flushed with Ar gas twice. The reaction mixture was filtered through a pad of Celite (paying attention to never let the Celite go dry), washing with NH_3 in MeOH (1 mmol mL^{-1} solution, 3 × 150 mL). The filtrate was concentrated under reduced pressure and the residue purified by flash column chromatography (gradient, 0–10% MeOH in CH_2Cl_2) to give 2° amine **9** as a pale-yellow oil (8.28 g, 98%).

ν_{max} (thin film/cm^{-1}): 3545 w, 3312 w, 2973 m, 1695 s, 1454 s, 1427 s, 1284 s, 1044 s.

^{1}H-NMR (400 MHz, CD_3OD, resonance broadening observed because of rotamers) δ_H 3.94 – 3.69 (stack, 4H), 3.65 – 3.55 (m, 1H), 3.55 – 3.44 (m, 1H), 3.12 – 2.87 (stack, 1H), 2.85 – 2.55 (stack, 5H), 1.45 (s, 9H), 1H (probably NH) not observed.

^{13}C{^{1}H}-NMR (101 MHz, CD_3OD, resonance broadening observed because of rotamers) δ_C 157.0 (C), 92.1 (C), 81.4 (C), 61.2 (CH_2), 60.2 (CH_2), 51.4 (CH_2), 50.8 (CH_2), 45.3 (CH_2), [44.7, 43.4 (CH_2)], 28.6 (CH_3).

LRMS (ES+): 281.2 [$(M + Na)^+$, 10%], 259.2 [15, $(M + H)^+$], 203.1 [100, $(M + H – Bn)^+$].

HRMS (ES+): calcd for $C_{12}H_{23}N_2O_4$ $[M + H]^+$ 259.1658, found 259.1659.

***tert*-butyl 10-methyl-1,7-dioxa-4,10-diazaspiro[5.5]undecane-4-carboxylate (S20):**

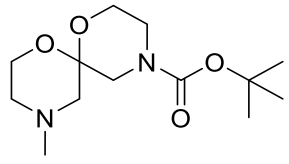

Methylamine **S20** was isolated as a by-product of the hydrogenolysis of Cbz-carbmate **8** (see p 56).

R_f (CH_2Cl_2/MeOH, 9/1) = 0.5.

ν_{max} (thin film/cm^{-1}): 2975 w, 2359 w, 1697 s, 1171 m.

^{1}H-NMR (400 MHz, CD_3OD, resonance broadening observed because of rotamers) δ_H 3.94 – 3.76 (stack, 4H), 3.66 – 3.52 (stack, 2H), 3.12 – 2.62 (stack, 4H), 2.28 – 2.13 (stack, 4H), 1.98 (d, J = 11.5 Hz, 1H), 1.46 (s, 9H).

^{13}C{^{1}H}-NMR (101 MHz, CD_3OD, resonance broadening observed because of rotamers) δ_C 156.8 (C), 93.6 (C), 81.4 (C), 61.0 (CH_2), 60.9 (CH_2), 60.2 (CH_2), 55.0 (CH_2), 51.0 (CH_2), 46.2 (CH_3), [44.7, 43.5 (CH_2)], 28.6 (CH_3), one of the CH_2 resonances overlapped with the CD_3OD resonances, however HSQC confirms its presence around δ_C 48.5 ppm.

LRMS (ES+): 273.2 [$(M + H)^+$, 40%], 217.1 [100, $(M + H – t\text{-Bu})^+$], 112.1 (60).

HRMS (ES+): calcd for $C_{13}H_{25}N_2O_4$ $[M + H]^+$ 273.1814, found 273.1819.

benzyl 1,7-dioxa-4,10-diazaspiro[5.5]undecane-4-carboxylate hydrochloride (10·HCl):

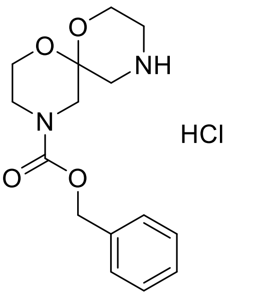

Boc-Protected amine **8** (4.04 g, 10.8 mmol) was added to a solution of HCl in 1,4-dioxane (14 mL of a 4 M solution, 56 mmol) at rt. After 3 h, more HCl in 1,4-dioxane (5 mL of a 4 M solution, 20 mmol) was added to the mixture, and after 17 h, a further volume of HCl in 1,4-dioxane (5 mL of a 4 M solution, 20 mmol) was added. After 4 h, the reaction mixture was concentrated under reduced pressure using a rotary evaporator maintained in a ducted fume cupboard, and the crude product was washed sequentially with Et_2O (2 × 30 mL), Et_2O/CH_2Cl_2 (1/1 mixture, 2 × 30 mL) and dried under reduced pressure to produce amine HCl salt **10·HCl** as a white solid (3.31 g, 98%).

ν_{max} (KBr/cm^{-1}): 3435 br m, 2934 w, 1701 br s, 1048 m.

^{1}H-NMR (400 MHz, CD_3OD, resonance broadening observed because of rotamers) δ_H 7.50 – 7.21 (stack, 5H), 5.13 (s, 2H), 4.15 – 3.89 (stack, 3H), 3.89 – 3.63 (stack, 3H), 3.28 – 2.92 (stack, 6H), 1H (probably NH) not observed.

^{13}C{^{1}H}-NMR (101 MHz, CD_3OD, resonance broadening observed because of rotamers) δ_C 157.2 (C), 137.9 (C), 129.5 (CH), 129.2 (CH), 128.9 (CH), [92.5, 92.1 (C)], 68.6 (CH_2), 60.7 (CH_2), 57.5 (CH_2), 48.2 (CH_2), [44.1, 43.7 (CH_2)], 43.4 (CH_2), the resonance for one CH_2 overlapped with the CD_3OD resonances, however HSQC confirms its presence around δ_C 49.4 ppm.

LRMS (ES+): 293.2 [(M + H)$^+$, 100%].

HRMS (ES+): calcd for $C_{15}H_{21}N_2O_4$ [M + H]$^+$ 293.1501, found 293.1508.

***tert*-butyl 4-benzyl-1,7-dioxa-4,11-diazaspiro[5.6]dodecane-11-carboxylate (19):**

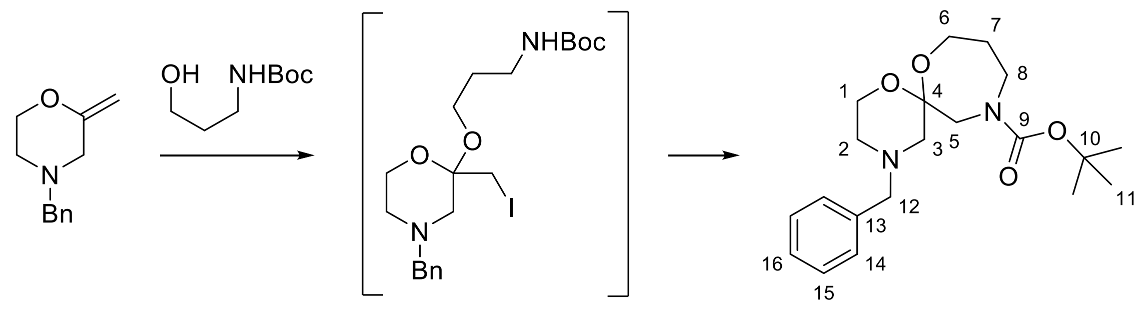

4 **19**

Following general procedure D (p 53), NIS (11.9 g, 52.8 mmol) was added over 10 min to a solution of *N*-Boc-propanolamine (11.1 g, 63.4 mmol) and enol ether **4** (10.0 g, 52.8 mmol) in CH_2Cl_2 (11 mL) at –30 °C. After 30 min, the reaction was worked up according to the general procedure to provide the iodo acetal intermediate as a colorless oil (20.6 g, 79%), which was used directly in the next step: *t*-BuOK (3.57 g, 31.8 mmol) was added over 15 min to a solution of the iodo acetal (10.4 g, 21.2 mmol) in DMF (140 mL) at rt. After 4 h, more *t*-BuOK (1.19 g, 10.6 mmol) was added to the reaction mixture. After 18 h, the reaction was worked up according to the general procedure to produce spiroacetal **19** as a light-yellow oil (6.87 g, 70% over the two steps), which was used in the next step without further purification.

R_f (*n*-heptane/EtOAc, 3/2) = 0.3.

ν_{max} (thin film/cm^{-1}): 2972 m, 1697 br s, 1419 s, 1161 s, 1061 s.

^{1}H-NMR (400 MHz, $CDCl_3$, ~3:2 mixture of rotamers based on the relative integration of the resonances for H-8a maj at δ_H 4.35 – 4.22 ppm and H-8b min at δ_H 2.91 – 2.79 ppm) δ_H 7.40 – 7.19 (stack, 5H, Ph), 4.35 – 4.22 (m, 0.6 H, H-8a maj), 4.11 – 3.77 (stack, 4H, H-1, H-3a, H-5a, H-6, H-8a min), 3.70 – 3.54 (stack, 2H, H-3b, H-12a), 3.54 – 3.34 (stack, 1H, H-12b), 3.13 – 2.92 (stack, 2H, H-1, H-5b, H-6), 2.91 – 2.79 (m, 0.4H, H-8b min), 2.79 – 2.58 (stack, 2H, H-2, H-8b maj), 2.29 – 1.92 (stack, 3H, H-1, H-2b, H-6, H-7a), 1.64 – 1.53 (stack, 1H, H-7b), 1.41 (s, 3.6H, H-11 min), 1.32 (s, 5.4H, H-11 maj).

^{13}C{^{1}H}-NMR (101 MHz, $CDCl_3$, mixture of rotamers) δ_C [155.1, 154.5 (C, C-9)], [137.0, 136.7 (C, C-13)], [129.5, 129.2 (CH, C-14)], [128.3, 128.1 (CH, C-15)], [127.2, 127.0 (CH, C-16)], [99.0, 98.6 (C, C-4)], [79.9, 79.6 (C, C-10)], [63.5, 63.2 (CH_2, C-12)], [61.7, 61.3, 61.2 (CH_2, C-1, C-6)], [61.0, 60.8 (CH_2, C-3)], [55.5, 55.2 (CH_2, C-5)], [52.5, 52.0 (CH_2, C-2)], [49.3, 48.7 (CH_2, C-8)], [30.3, 30.2 (CH_2, C-7)], [28.4, 28.3 (CH_3, C-11)].

LRMS (ES+): 363.2 [(M + H)$^+$, 100%], 307.2 [30, (M + H – *t*-Bu)$^+$].

HRMS (ES+): calcd for $C_{20}H_{31}N_2O_4$ [M + H]$^+$ 363.2284, found 363.2285.

4-benzyl 11-(*tert*-butyl) 1,7-dioxa-4,11-diazaspiro[5.6]dodecane-4,11-dicarboxylate (20):

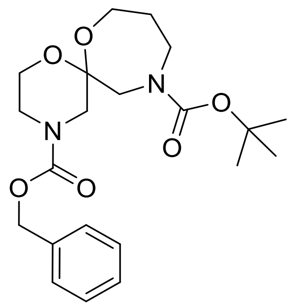

Under an Ar atmosphere, CbzCl (0.45 mL, 3.1 mmol) was added dropwise over 5 min to a solution of benzylamine **19** (540 mg, 1.49 mmol) in CH_2Cl_2 (7.5 mL) at rt. After 3 days, the reaction mixture was diluted with CH_2Cl_2 (15 mL) and extracted with $NaHCO_3$ solution (3 × 15 mL). The organic layer was dried over Na_2SO_4, filtered, and concentrated under reduced pressure. The crude product was purified by flash column chromatography (gradient, 0–75% EtOAc in *n*-heptane) to produce carbamate **20** as a light-yellow oil (483 mg, 80%).

R_f (*n*-heptane/EtOAc, 3/2) = 0.4.

ν_{max} (thin film/cm^{-1}): 2932 s, 1695 br s, 1470 br m, 1057 m.

^{1}H-NMR (400 MHz, $CDCl_3$, mixture of rotamers) δ_H 7.37 – 7.14 (stack, 5H), 5.20 – 4.93 (stack, 2H), 4.31 – 4.04 (stack, 2H), 4.02 – 3.60 (stack, 5H), 3.60 – 3.42 (stack, 1H), 3.09 – 2.51 (stack, 4H), 2.06 – 1.76 (stack, 1H), 1.60 – 1.11 (stack, 10H).

^{13}C{^{1}H}-NMR (101 MHz, $CDCl_3$, mixture of rotamers) δ_C [155.9, 155.2, 154.3 (2 × C)], [136.7, 136.5 (C)], [128.5, 128.3, 128.2, 128.0, 127.6, 127.4 (3 × CH)], [97.9, 97.4 (C)], [80.2, 79.9 (C)], [67.4, 67.0 (CH_2)], [61.4, 61.2 (CH_2)], 59.8 (CH_2, broad), [54.9, 54.3 (CH_2)], 52.4 (CH_2, broad), [49.4, 49.0 (CH_2)], 42.8 (CH_2, broad), [30.4, 30.1 (CH_2)], 28.2 (CH_3).

LRMS (ES+): 429.2 [(M + Na)$^+$ 100%], 351.2 [45, (M + H – *t*-Bu)$^+$], 307.2 [15, (M + H – Boc)$^+$], 126 (20).

HRMS (ES+): calcd for $C_{21}H_{30}N_2O_6Na$ [M + Na]$^+$ 429.2002, found 429.2010.

***tert*-butyl 1,7-dioxa-4,11-diazaspiro[5.6]dodecane-11-carboxylate (25):**

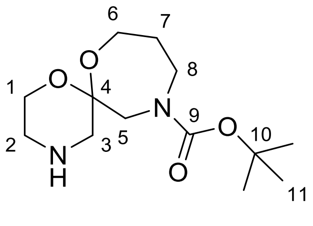

Under an Ar atmosphere, Pd/C (10 wt%, 240 mg, 0.22 mmol, 5 mol%) was added to a degassed solution of carbamate **20** (1.76 g, 4.48 mmol) in THF/H_2O (4/1 mixture, 45 mL) at rt. The reaction vessel was

sequentially evacuated and flushed with H_2 gas twice and the mixture was then stirred under an H_2 atmosphere. After 18 h, the reaction vessel was evacuated and flushed with Ar gas twice. The reaction mixture was filtered through a pad of Celite (paying attention to never let the Celite go dry), washing with NH_3 in MeOH (1 mmol mL^{-1}, 3 × 30 mL). The filtrate was concentrated under reduced pressure and the residue purified by flash column chromatography (gradient, 0–10% MeOH in CH_2Cl_2 + 1 v/v% Et_3N)[p] to give 2° amine **25** as a white solid (1.04 g, 85%).

ν_{max} (KBr/cm^{-1}): 3466 br m, 2946 m, 1685 br s, 1423 m, 1159 s.

^{1}H-NMR (400 MHz, CD_3OD, ~3/2 mixture of rotamers based on the relative integration of the resonances for H-8a maj at δ_H 4.20 – 4.11 ppm and H-8a min at 4.09 – 4.01 ppm) δ_H 4.20 – 4.11 (m, 0.6H, H-8a maj), 4.09 – 4.01 (m, 0.4H, H-8a min) 3.99 – 3.84 (stack, 3H, H-1a, H-5a, H-6a), 3.84 – 3.74 (stack, 1H, H-6b), 3.58 – 3.47 (stack, 1H, H-1b), 3.09 – 2.65 (stack, 6H, H-2, H-3, H-5b, H-8b), 1.99 – 1.81 (stack, 1H, H-7a), 1.69 – 1.58 (stack, 1H, H-7b), 1.53 – 1.41 (stack, 9H, H-11), NH not observed.

^{13}C{^{1}H}-NMR (101 MHz, CD_3OD, mixture of rotamers) δ_C [156.9, 156.3 (C, C-9)], [98.3, 97.9 (C, C-4)], [81.6, 81.1 (C, C-10)], [62.23, 62.18 (CH_2, C-6)], [61.9, 61.8 (CH_2, C-1)], [56.2, 55.8, (CH_2, C-5)], [54.8, 54.7 (CH_2, C-3)], [50.5, 49.7 (CH_2, C-8)], 45.2 (CH_2, C-2), [31.5, 31.3 (CH_2, C-7)], 28.7 (CH_3, C-11).

LRMS (ES+): 273.2 [(M + H)$^+$, 30%], 217.1 [100, (M + H – *t*-Bu)$^+$].

HRMS (ES+): calcd for $C_{13}H_{25}N_2O_4$ [M + H]$^+$ 273.1814, found 273.1815.

***tert*-butyl 11-benzyl-1,7-dioxa-4,11-diazaspiro[5.6]dodecane-4-carboxylate (22):**

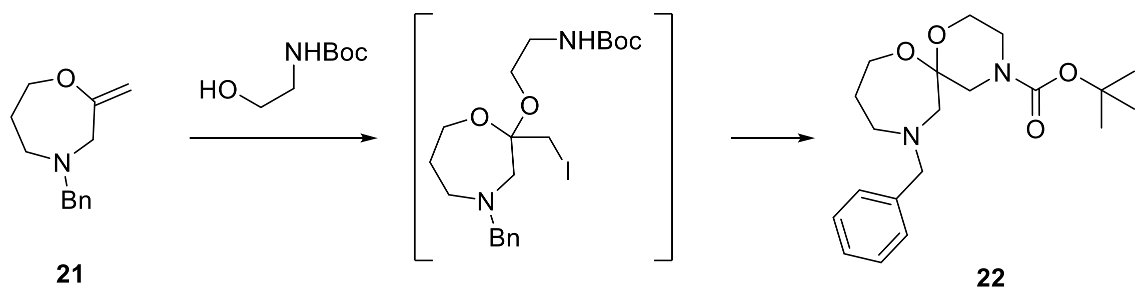

21 **22**

Following general procedure D (p 53), NIS (244 mg, 1.08 mmol) was added in one batch to a solution of *N*-Boc-ethanolamine (209 mg, 1.30 mmol) and enol ether **21** (220 mg, 1.08 mmol) in CH_2Cl_2 (7.0 mL) at –40 °C. After 10 min, the reaction was worked up according to the general procedure to provide the iodo acetal intermediate as a colorless oil (310 mg, 58%), which was used directly in the next step: *t*-BuOK (133 mg, 1.18 mmol) was added in one batch to a solution of the iodo acetal (290 mg, 0.59 mmol) in DMF (3.0 mL) at rt. After 30 min, the reaction was worked up according to the general procedure.

[p] Despite the use of Et_3N in the eluent mixture, the product still showed some tailing during column chromatography.

The product was purified by flash column chromatography (gradient, 0–100% EtOAc in *n*-heptane) to produce spiroacetal **22** as a yellow oil (163 mg, 46% over the two steps).

R_f (*n*-heptane/EtOAc, 3/2) = 0.4.

ν_{max} (thin film/cm^{-1}): 2932 m, 1698 br s, 1420 m, 1060 s.

^{1}H-NMR (400 MHz, $CDCl_3$, resonance broadening observed because of rotamers) δ_H 7.44 – 7.19 (stack, 5H), 4.07 – 3.80 (stack, 3H), 3.80 – 3.57 (stack, 4H), 3.57 – 3.47 (stack, 1H), 3.09 – 2.92 (stack, 2H), 2.92 – 2.76 (stack, 1H), 2.76 – 2.58 (stack, 2H), 2.48 – 2.30 (stack, 1H), 2.07 – 1.89 (stack, 1H), 1.64 – 1.38 (stack, 10H).

^{13}C{^{1}H}-NMR (101 MHz, $CDCl_3$, resonance broadening observed because of rotamers) δ_C 155.6 (C), 139.3 (C), 129.0 (CH), 128.3 (CH), 127.1 (CH), 97.8 (C, broad), 79.7 (C), 63.2 (CH_2), [62.5, 61.8 (CH_2)], 61.6 (CH_2), 59.4 (CH_2), [57.4, 57.0 (CH_2)], [51.6, 50.1 (CH_2)], [43.7, 42.7 (CH_2)], 31.3 (CH_2, broad), 28.4 (CH_3).

LRMS (ES+): 363.2 [(M + H)$^+$, 70%], 307.2 [60, (M + H – *t*-Bu)$^+$], 278.2 (35), 204.1 (90), 178.1 (100).

HRMS (ES+): calcd for $C_{20}H_{31}N_2O_4$ [M + H]$^+$ 363.2284, found 363.2287.

11-benzyl 4-(*tert*-butyl) 1,7-dioxa-4,11-diazaspiro[5.6]dodecane-4,11-dicarboxylate (23):

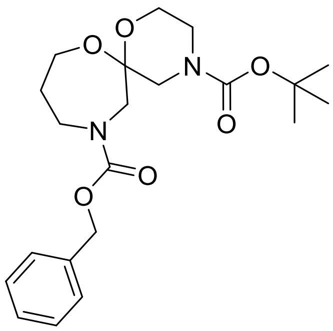

Under an Ar atmosphere, CbzCl (105 µL, 0.73 mmol) was added dropwise over 2 min to a solution of benzylamine **22** (125 mg, 0.345 mmol) in CH_2Cl_2 (1.7 mL) at rt. After 16 h, the reaction mixture was diluted with CH_2Cl_2 (10 mL) and extracted with $NaHCO_3$ solution (3 × 10 mL). The organic layer was dried over Na_2SO_4, filtered, concentrated under reduced pressure and the crude product was purified by flash column chromatography (gradient, 0–75% EtOAc in *n*-heptane) to produce carbamate **23** as a white solid (139 mg, 99%). Crystals of spiroacetal **23** suitable for analysis by X-ray analysis were grown by evaporation of a solution of **23** in EtOAc/*n*-heptane, see Section 4.1.2 for full details.

R_f (*n*-heptane/EtOAc, 3/2) = 0.5.

ν_{max} (neat/cm^{-1}): 2960 w, 2681 w, 1698 s, 1683 s, 1059 s.

^{1}H-NMR (400 MHz, $CDCl_3$, mixture of rotamers) δ_H 7.38 – 7.16 (stack, 5H), 5.27 – 4.82 (stack, 2H), 4.29 – 3.56 (stack, 7H), 3.54 – 3.39 (stack, 1H), 3.20 – 2.60 (stack, 4H), 2.11 – 1.80 (stack, 1H), 1.62 – 1.51 (stack, 1H), 1.37 (s, 9H).

$^{13}C\{^1H\}$-NMR (101 MHz, $CDCl_3$, mixture of rotamers) δ_C 155.9 (C), 155.3 (C), 136.8 (C), [128.5, 128.2, 128.0, 127.8 (3 × CH)], 97.7 (C), [79.9, 79.7 (C)], [67.5, 67.3 (CH_2)], [61.1, 61.0 (CH_2)], [59.9, 59.8 (CH_2)], [54.8, 54.6 (CH_2)], [52.4, 50.6 (CH_2)], [49.3, 48.9 (CH_2)], [43.3, 42.2 (CH_2)], [30.3, 29.9 (CH_2)], 28.4 (CH_3).

LRMS (ES+): 429.2 [$(M + Na)^+$, 100%], 407.2 [40, $(M + H)^+$], 351.2 [100, $(M + H – t\text{-Bu})^+$], 307.2 [20, $(M + H – Boc)^+$].

HRMS (ES+): calcd for $C_{21}H_{31}N_2O_6$ $[M + H]^+$ 407.2195, found 407.2191.

***tert*-butyl 12-benzyl-1,8-dioxa-5,12-diazaspiro[6.6]tridecane-5-carboxylate (24):**

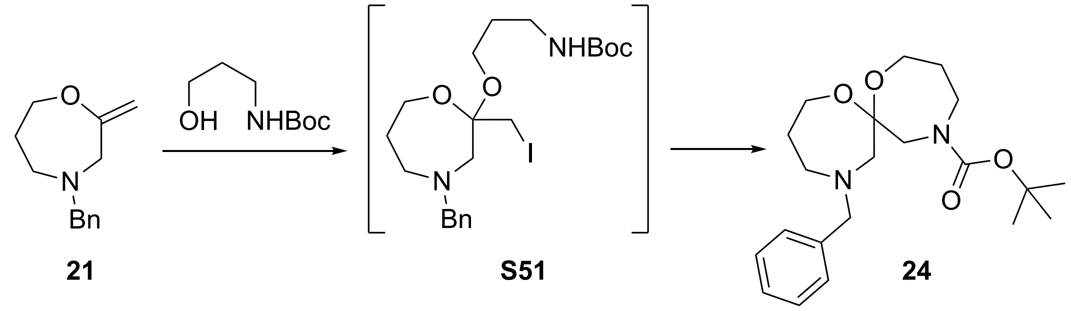

21 **S51** **24**

Following general procedure D (p 53), NIS (199 mg, 0.885 mmol) was added in one batch to a solution of *N*-Boc-propanolamine (186 mg, 1.06 mmol) and enol ether **21** (180 mg, 0.885 mmol) in CH_2Cl_2 (6.0 mL) at –40 °C. After 30 min, the reaction was worked up according to the general procedure to provide iodo acetal intermediate **S51** as a colorless oil (340 mg, 76%), which was used directly in the next step: *t*-BuOK (147 mg, 1.31 mmol) was added in two portions to a solution of iodo acetal **S51** (330 mg, 0.654 mmol) in DMF (3.3 mL) at rt. After 2 days, more *t*-BuOK (75 mg, 0.65 mmol) was added. After 4 days, the reaction was still not complete as evidenced by LCMS analysis, so more *t*-BuOK (75 mg, 0.65 mmol) was added. After a further 7 days, the reaction was worked up according to the general procedure and the crude product was purified by flash column chromatography (gradient, 0–100% EtOAc in *n*-heptane) to produce spiroacetal **24** as a yellow oil (55 mg, 17% over the two steps).

R_f (*n*-heptane/EtOAc, 3/2) = 0.5.

ν_{max} (KBr/cm^{-1}): 2935 m, 1690 s, 1421 s, 1162 s, 1055 s.

^{1}H-NMR (400 MHz, $CDCl_3$, mixture of rotamers) δ_H 7.49 – 7.14 (stack, 5H), 4.93 – 4.59 (stack, 1H), 4.27 – 3.76 (stack, 4H), 3.76 – 3.46 (stack, 3H), 3.32 – 2.33 (stack, 5H), 2.29 – 2.03 (stack, 1H), 2.01 – 1.31 (stack, 13H).

$^{13}C\{^1H\}$-NMR (101 MHz, $CDCl_3$, mixture of rotamers) δ_C 155.2 (C), [139.8, 139.3 (C)], [129.3, 129.2, 128.8, 128.0, 126.7 (3 × CH)], [103.5, 102.2 (C)], [80.0, 79.3 (C)], 63.5 (CH_2), 62.6 (CH_2), 61.4 (CH_2), 61.2 (CH_2), [56.5, 56.0 (CH_2)], [54.1, 53.1 (CH_2)], [49.7, 48.5 (CH_2)], [31.7, 31.2 (CH_2)], [30.8, 30.0 (CH_2)], 28.5 (CH_3).

LRMS (ES+): 377.2 [$(M + H)^+$, 100%], 321.2 [20, $(M + H – t\text{-Bu})^+$].

HRMS (ES+): calcd for $C_{21}H_{33}N_2O_4$ $[M + H]^+$ 377.2440, found 377.2446.

Isolated spiroacetal **24** contained an unidentified iodide impurity (<10%). The $^{13}C\{^1H\}$-NMR spectrum is consistent with the structure shown below: $^{13}C\{^1H\}$-NMR (101 MHz, $CDCl_3$) δ_C 156.0 (C), 139.2 (C), 128.5 (CH), 127.3 (CH), 100.7 (C), 64.3, 63.0, 60.9, 57.7, 57.4, 9.3 (CH_2).

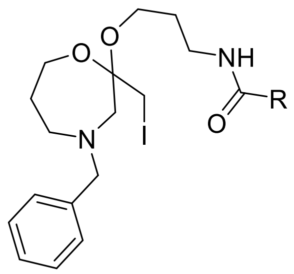

3-(3-((4-benzyl-2-(iodomethyl)-1,4-oxazepan-2-yl)oxy)propyl)-1,1-dimethylurea (S27):

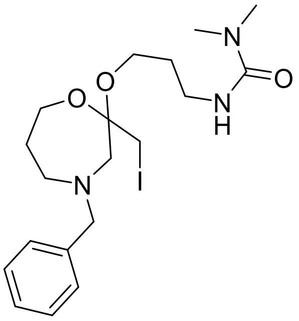

Urea **S27** was isolated as a by-product of the ring closure of iodo acetal **S51** (see p 63) as a yellow oil (51 mg 17% over the two steps).

ν_{max} (thin film/cm^{-1}): 3353 br s, 2950 m, 2812 w, 2234 w, 1746 w, 1633 br s, 1538 m.

^1H-NMR (400 MHz, $CDCl_3$) δ_H 7.35 – 7.12 (stack, 5H), 4.91 (br s, 1H), 3.77 (t, J = 12.2 Hz, 1H), 3.69 – 3.48 (stack, 4H), 3.43 (A of AB, $J_{A–B}$ = 11.0 Hz, 1H), 3.38 – 3.11 (stack, 5H), 2.92 – 2.76 (stack, 7H), 2.42 – 2.33 (m, 1H), 2.19 (td, J = 12.2, 3.4 Hz, 1H), 1.88 – 1.77 (m, 1H), 1.76 – 1.60 (m, 2H), 1.59 – 1.46 (m, 1H).

$^{13}C\{^1H\}$-NMR (101 MHz, $CDCl_3$) δ_C 158.5 (C), 139.0 (C), 129.2 (CH), 128.5 (CH), 127.4 (CH), 100.7 (C), 64.3 (CH_2), 63.0 (CH_2), 60.9 (CH_2), 58.5 (CH_2), 57.6 (CH_2), 39.1 (CH_2), 36.2 (CH_3), 31.7 (CH_2), 29.5 (CH_2), 9.7 (CH_2).

LRMS (ES+): 476.1 [(M + H)$^+$, 100%], 330.0 (20).

HRMS (ES+): calcd for $C_{19}H_{31}IN_3O_3$ [M + H]$^+$ 476.1410, found 476.1424.

3-(4-((4-benzyl-2-(iodomethyl)morpholin-2-yl)oxy)butyl)-1,1-dimethylurea (S25):

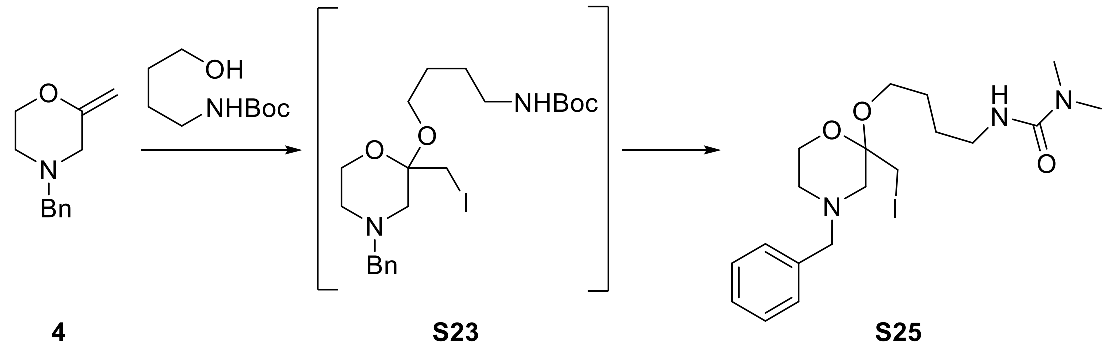

4 **S23** **S25**

Following general procedure D (p 53), NIS (238 mg, 1.06 mmol) was added in one batch to a solution of *N*-Boc-butanolamine (240 mg, 1.27 mmol) and enol ether **4** (200 mg, 1.06 mmol) in CH_2Cl_2 (7.0 mL) at –40 °C. After 30 min, the reaction was worked up according to the general procedure to provide iodo acetal intermediate **S23** as a colorless oil (439 mg, 82%), which was used directly in the next step: *t*-BuOK (148 mg, 1.33 mmol) was added in two portions to a solution of the iodo acetal **S23** (0.34 g, 0.66 mmol) in DMF (15 mL) at rt. After 2 days, more *t*-BuOK (75 mg, 0.65 mmol) was added. After 1 day, the reaction was worked up according to the general procedure and the crude product was purified by flash column chromatography (gradient, 0–100% EtOAc in *n*-heptane) to afford urea **S25** as a yellow oil (27 mg, 5% over the two steps).

Selected data:

ν_{max} (thin film/cm^{-1}): 3354 br m, 2939 m, 1738 w, 1633 s, 1538 s.

^{1}H-NMR (400 MHz, $CDCl_3$) δ_H 7.42 – 7.15 (stack, 5H), 4.61 (br s, 1H), 3.93 – 3.83 (m, 1H), 3.68 – 3.56 (stack, 2H), 3.56 – 3.41 (stack, 4H), 3.41 – 3.26 (stack, 4H), 2.89 (s, 6H), 2.51 – 2.43 (m, 1H), 2.34 (d, *J* = 11.5 Hz, 1H), 2.30 – 2.20 (m, 1H), 1.77 – 1.55 (stack, 4H).

LRMS (ES+): 476.1 [(M + H)$^+$, 100%], 316.0 (10).

HRMS (ES+): calcd for $C_{19}H_{31}IN_3O_3$ [M + H]$^+$ 476.1410, found 476.1418.

2-(2-((4-benzyl-2-(iodomethyl)morpholin-2-yl)oxy)ethoxy)ethan-1-amine (S26):

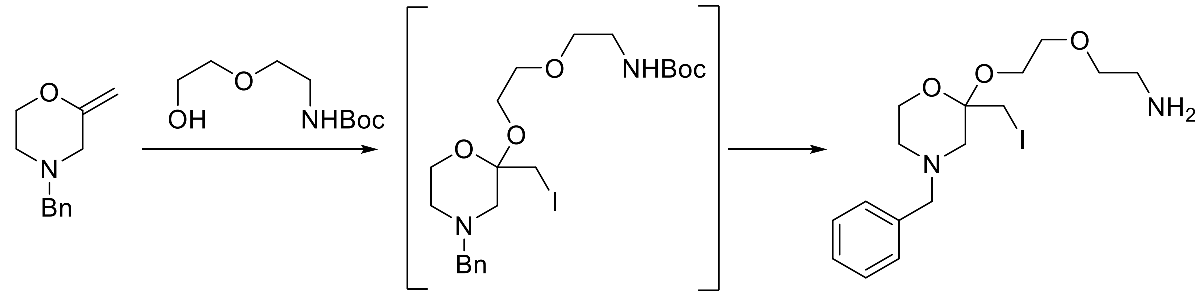

4 **S24** **S26**

Following general procedure D (p 53), NIS (238 mg, 1.06 mmol) was added in one batch to a solution of 2-(Boc-amino)ethoxy ethanol (260 mg, 1.27 mmol) and enol ether **4** (200 mg, 1.06 mmol) in CH_2Cl_2 (7.0 mL) at –40 °C. After 30 min, the reaction was worked up according to the general procedure to provide the iodo acetal intermediate **S24** as a colorless oil (429 mg, 78%), which was used directly in the next step: *t*-BuOK (259 mg, 2.31 mmol) was added in two portions to a solution of the iodo acetal **S24** (0.40 g, 0.77 mmol) in DMF (15 mL) at rt. After 16 h, more *t*-BuOK (259 mg, 2.31 mmol) was added. After 7 days, the reaction was worked up according to the general procedure and the crude product was purified by flash column chromatography (gradient, 0–20% MeOH in CH_2Cl_2) to produce 1° amine **S26** as a yellow oil (91 mg, 17% over the two steps).

Selected data:

^{1}H-NMR (400 MHz, $CDCl_3$) δ_H 7.50 – 6.90 (stack, 5H), 3.92 – 3.81 (m, 1H), 3.68 – 3.48 (stack, 8H), 3.40 (d, J = 13.1 Hz, 1H), 3.28 (A of AB, $J_{A–B}$ = 11.2 Hz, 1H), 3.24 (B of AB, $J_{B–A}$ = 11.2 Hz, 1H), 2.91 (d, J = 11.5 Hz, 1H), 2.86 – 2.79 (m, 2H), 2.54 (br s, 2H), 2.48 – 2.40 (m, 1H), 2.26 (d, J = 11.5 Hz, 1H), 2.22 – 2.12 (m, 1H).

^{13}C{^{1}H}-NMR (101 MHz, $CDCl_3$) δ_C 137.0 (C), 129.2 (CH), 128.3 (CH), 127.3 (CH), 95.6 (C), 72.8 (CH_2), 69.9 (CH_2), 62.6 (CH_2), 62.4 (CH_2), 60.3 (CH_2), 58.8 (CH_2), 51.5 (CH_2), 41.6 (CH_2), 7.8 (CH_2).

LRMS (ES+): 421.1 [$(M + H)^+$, 100%], 316.0 (20).

2.6.3 Substituted 6,6-spiroacetals

***tert*-butyl (3*S*)-10-benzyl-3-methyl-1,7-dioxa-4,10-diazaspiro[5.5]undecane-4-carboxylate (44):**

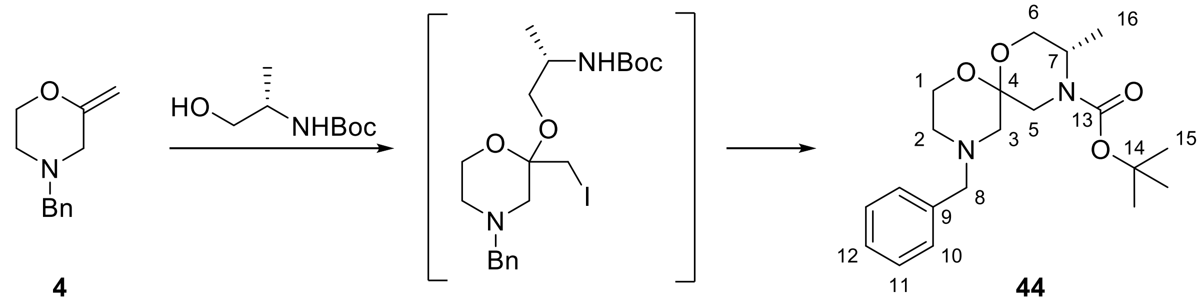

4 **44**

Following general procedure D (p 53), NIS (238 mg, 1.06 mmol) was added to a solution of *tert*-butyl (*S*)-(1-hydroxypropan-2-yl)carbamate (222 mg, 1.27 mmol) and enol ether **4** (200 mg, 1.06 mmol) in CH_2Cl_2 (7.0 mL) at –40 °C. After 30 min, the reaction was worked up according to the general procedure to provide the iodo acetal intermediate as a colorless oil (400 mg, 77%), which was used directly in the next step.[q] *t*-BuOK (268 mg, 2.39 mmol) was added to a solution of the iodo acetal (390 mg, 0.80 mmol) in DMF (4.0 mL) at rt. After 1 h, the reaction was worked up according to the general procedure to produce spiroacetal **44** as a yellow oil (269 mg, 70% over the two steps, d.r. ~1:1, based on ^{1}H-NMR spectroscopy data).

R_f (*n*-heptane/EtOAc, 3/2) = 0.4.

ν_{max} (thin film/cm^{-1}): 2973 m, 1694 br s, 1415 s, 1058 s.

^{1}H-NMR (400 MHz, $CDCl_3$, resonance broadening observed because of rotamers, ~1:1 mixture of diastereoisomers, based on the relative integration of the resonances for H-16 diastA at δ_H 1.22 ppm and H-16 diastB at 1.07 ppm) δ_H 7.46 – 7.10 (stack, 5H, Ph), 4.24 – 3.84 (stack, 3H, H-1 or H-6, H-5a, H-7), 3.80 – 3.37 (stack, 5H, H-1, H-6, H-8), 2.96 – 2.59 (stack, 3H, H-2a, H-3a, H-5b), 2.34 – 2.18 (stack, 1H, H-2b), 2.18 – 2.02 (stack, 1H, H-3b), 1.48 (s, 4.5H, H-15 diastA), 1.44 (s, 4.5H, H-15 diastB), 1.22 (d, *J* = 7.1 Hz, 1.5H, H-16 diastA), 1.07 (d, *J* = 6.3 Hz, 1.5H, H-16 diastB).

^{13}C{^{1}H}-NMR (101 MHz, $CDCl_3$, mixture of rotamers and diastereoisomers) δ_C 155.1 (C, C-13), 136.9 (C, C-9), 129.5 (CH, C-10), [128.39 128.36 (CH, C-11)], [127.38, 127.35 (CH, C-12)], 96.6 (C, C-4), [80.0, 79.9 (C, C-14)], [63.4, 63.3 (CH_2, C-8, C-6)], [61.5, 60.7 (CH_2, C-1)], [59.7, 58.6 (CH_2, C-3)], [52.4, 52.0 (CH_2, C-2)], [48.7, 46.5 (CH, C-7)], [45.2, 45.0, 44.5, 43.8 (CH_2, C-5)], [28.54, 28.50 (CH_3, C-15)], 14.7 (CH_3, C-16).

LRMS (ES+): 363.2 [(M + H)$^+$], 100%], 307.2 [40, (M + H – *t*-Bu)$^+$].

HRMS (ES+): calcd for $C_{20}H_{31}N_2O_4$ [M + H]$^+$ 363.2284, found 363.2287.

[q] The d.r. of the iodo acetal intermediate could not be determined by LCMS methods because the peaks for the two diastereoisomers did not separate, nor by ^{1}H-NMR spectroscopy, because of resonance overlap; however, we presume the d.r. to be ~1:1, in analogy with other examples, which showed no diastereoselectivity in the iodoacetalization step.

tert-butyl (3S)-10-benzyl-3-isopropyl-1,7-dioxa-4,10-diazaspiro[5.5]undecane-4-carboxylate (45):

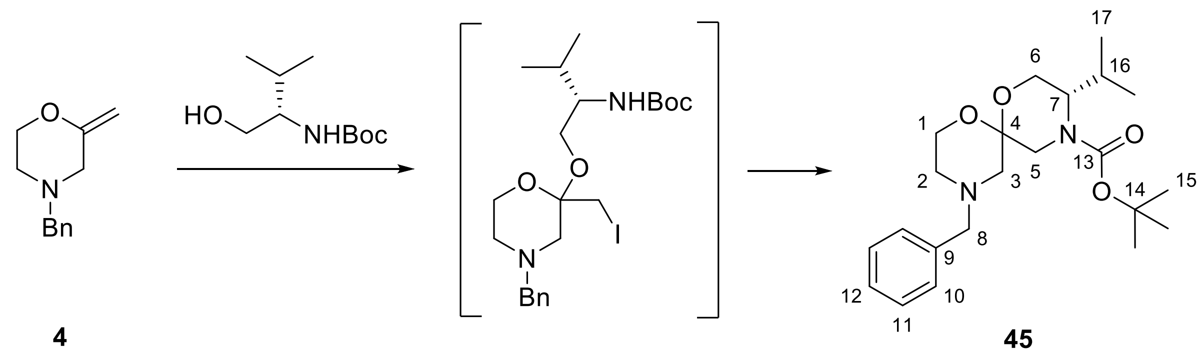

Following general procedure D (p 53), NIS (238 mg, 1.06 mmol) and *tert*-butyl (*S*)-(1-hydroxy-3-methylbutan-2-yl)carbamate (258 mg, 1.27 mmol) were added to a solution of enol ether **4** (200 mg, 1.06 mmol) in CH_2Cl_2 (7.0 mL) at –40 °C. After 30 min, the reaction was worked up according to the general procedure to provide the iodo acetal intermediate as a colorless oil (380 mg, 69%, d.r. ~1:1, neutral LCMS method), which was used directly in the next step: *t*-BuOK (189 mg, 1.69 mmol) was added to a solution of the iodo acetal (360 mg, 0.69 mmol) in DMF (3.5 mL) at rt. After 1 h, the reaction was worked up according to the general procedure to produce spiroacetal **45** as a yellow oil (249 mg, 60% over the two steps, d.r. ~1:1, based on ^{1}H-NMR spectroscopy data).

R_f (*n*-heptane/EtOAc, 3/2) = 0.5.

ν_{max} (thin film/cm^{-1}): 2968 m, 1695 br s, 1155 m, 1062 m.

^{1}H-NMR (400 MHz, $CDCl_3$, resonance broadening observed because of rotamers, ~1:1 mixture of diastereoisomers, based on the relative integration of the resonances for H-15 diastA at δ_H 1.47 ppm and H-15 diastB at 1.40 ppm) δ_H 7.41 – 7.16 (stack, 5H, Ph), 4.19 – 3.39 (stack, 8H, H-1, H-6, H-5a, H-7, H-8), 2.96 (d, *J* = 11.6 Hz, 0.5H, H-3a), 2.86 – 2.62 (stack, 2.5H, H-2a, H-3a, H-5b), 2.32 – 1.73 (stack, 3H, H-2b, H-3b, H-16), 1.47 (s, 4.5H, H-15 diastA), 1.40 (s, 4.5H, H-15 diastB), 1.02 – 0.95 (stack, 3H, H-17), 0.93 – 0.82 (stack, 3H, H-17).

^{13}C{^{1}H}-NMR (101 MHz, $CDCl_3$, mixture of rotamers and diastereoisomers) δ_C [155.5, 155.4 (C, C-13)], [137.0, 136.7 (C, C-9)], [129.6, 129.5, 129.4, 128.4, 127.4, 127.3 (CH, C-10, C-11, C-12)], [96.2, 92.6, 92.2 (C, C-4)], [79.9, 79.8 (C, C-14)], [63.4, 63.3 (CH_2, C-8)], [61.5, 60.8, 60.7, 60.4, 59.7, 59.6 (CH_2, C-1, C-6)], [58.6, 58.4 (CH_2, C-3)], [57.2, 55.4 (CH, C-7], [52.5, 52.1, 52.0 (CH_2, C-2)], [45.8, 44.3 (CH_2, C-5], 29.3 (CH, C-16), [28.54, 28.52, 28.4 (CH_3, C-15)], [25.1, 25.0 (CH, C-16)], [20.3, 20.2, 19.6, 19.3, 18.9, 18.4 (CH_3, C-17)].

LRMS (ES+): 391.3 [$(M + H)^+$], 100%], 335.2 [20, $(M + H – t\text{-Bu})^+$].

HRMS (ES+): calcd for $C_{22}H_{35}N_2O_4$ $[M + H]^+$ 391.2597, found 391.2608.

***tert*-butyl (3*S*)-10-benzyl-3-isobutyl-1,7-dioxa-4,10-diazaspiro[5.5]undecane-4-carboxylate (46):**

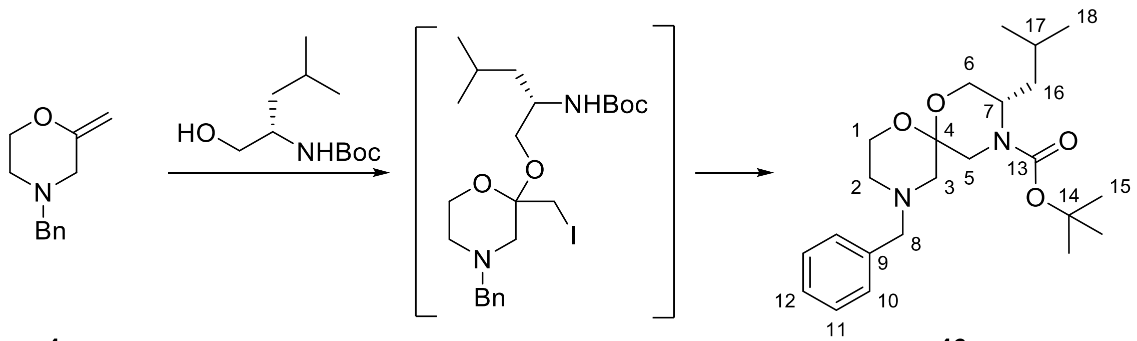

4 **46**

Following general procedure D (p 53), NIS (238 mg, 1.06 mmol) and *tert*-butyl (*S*)-(1-hydroxy-4-methylpentan-2-yl)carbamate (276 mg, 1.27 mmol) were added to a solution of enol ether **4** (200 mg, 1.06 mmol) in CH_2Cl_2 (7.0 mL) at –40 °C. After 15 min, the reaction was worked up according to the general procedure to provide the iodo acetal intermediate as a colorless oil (406 mg, 69%, d.r. ~1:1, neutral LCMS method), which was used directly in the next step: *t*-BuOK (169 mg, 1.50 mmol) was added to a solution of the iodo acetal (400 mg, 0.75 mmol) in DMF (3.8 mL) at rt. After 1 h, the reaction was worked up according to the general procedure to produce spiroacetal **46** as a yellow oil (266 mg, 62% over the two steps, d.r. ~1:1, neutral LCMS method).

R_f (*n*-heptane/EtOAc, 3/2) = 0.4.

ν_{max} (thin film/cm^{-1}): 2957 s, 1697 br s, 1062 s.

^{1}H-NMR (400 MHz, $CDCl_3$, mixture of rotamers and diastereoisomers, ratios could not be accurately determined) δ_H 7.37 – 7.16 (stack, 5H, Ph), 4.16 – 3.71 (stack, 4H, H-1a, H-5a, H-6a, H-7), 3.70 – 3.38 (stack, 4H, H-1b, H-6b, H-8), 2.93 (app d, *J* = 11.6 Hz, 0.5H, H-3a), 2.80 (app d, *J* = 14.3 Hz, 0.5H, H-5b), 2.75 – 2.57 (stack, 2H, H-2a, H-3a, H-5b), 2.31 – 2.14 (stack, 1H, H-2b), 2.14 – 2.01 (stack, 1H, H-3b), 1.74 – 1.34 (stack, 11.5H, H-15, H-16, H-17), 1.27 – 1.12 (stack, 0.5H, H-17), 0.97 – 0.86 (stack, 6H, H-18).

^{13}C{^{1}H}-NMR (101 MHz, $CDCl_3$, mixture of rotamers and diastereoisomers) δ_C 155.3 (C, C-13), [137.1, 136.8 C, C-9)], [129.6, 129.48, 129.46, 128.4, 127.4 (CH, C-10, C-11, C-12)], [96.5, 92.8, 92.3 (C, C-4)], [80.04, 80.01, 79.8 (C, C-14)], [63.4, 63.3 (CH_2, C-8)], [62.5, 62.0, 61.9, 61.5, 60.8, 60.7 (CH_2, C-1, C-6)], [59.1, 58.7, 58.5 (CH_2, C-3)], [52.5, 52.0 (CH_2, C-2)], [50.7, 48.9, 47.5 (CH, C-7)], [45.4, 44.9, 43.9 (CH_2, C-5)], [39.4, 37.8, 37.2 (CH_2, C-16)], [28.54, 28.49 (CH_3, C-15)], [25.1, 24.8, 24.6 (CH, C-17)], [23.3, 23.0, 22.9, 22.8, 22.5 (CH_3, C-18)].

LRMS (ES+): 405.3 [(M + H)$^+$], 100%], 349.2 [20, (M + H – *t*-Bu)$^+$].

HRMS (ES+): calcd for $C_{23}H_{37}N_2O_4$ [M + H]$^+$ 405.2753, found 405.2762.

tert-butyl (3S)-10-benzyl-3-phenyl-1,7-dioxa-4,10-diazaspiro[5.5]undecane-4-carboxylate (47):

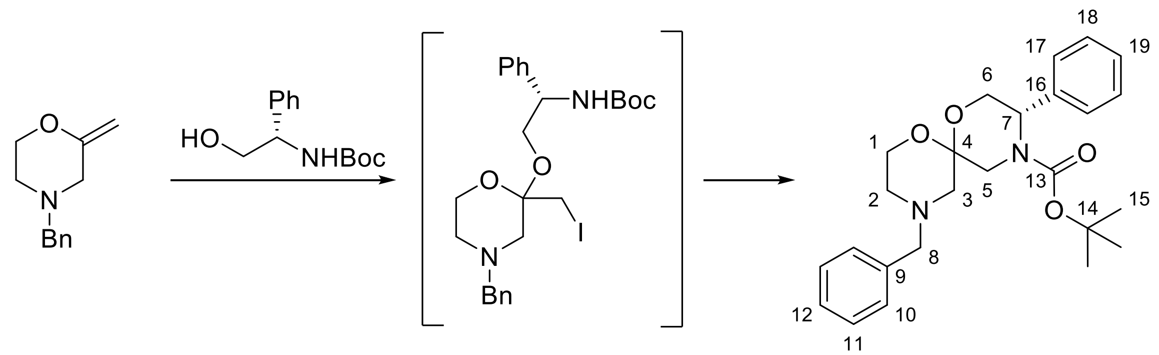

4 **47**

Following general procedure D (p 53), NIS (238 mg, 1.06 mmol) and *tert*-butyl (*S*)-(2-hydroxy-1-phenylethyl)carbamate (301 mg, 1.27 mmol) were added to a solution of enol ether **4** (200 mg, 1.06 mmol) in CH_2Cl_2 (7.0 mL) at –40 °C. After 15 min, the reaction was worked up according to the general procedure to provide the iodo acetal intermediate as a colorless oil (285 mg, 49%, d.r. ~1:1, neutral LCMS method), which was used directly in the next step: *t*-BuOK (116 mg, 1.03 mmol) was added to a solution of the iodo acetal (285 mg, 0.52 mmol) in DMF (2.6 mL) at rt. After 1 h, the reaction was worked up according to the general procedure to produce spiroacetal **47** as a yellow oil (161 mg, 36% over the two steps, d.r. ~1:1, neutral LCMS method).

R_f (*n*-heptane/EtOAc, 3/2) = 0.5.

ν_{max} (KBr/cm^{-1}): 2975 w, 1697 br s, 1152 m.

^{1}H-NMR (400 MHz, $CDCl_3$, mixture of rotamers and diastereoisomers, ratios could not be accurately determined) δ_H 7.58 – 7.16 (stack, 10H, Ph), 5.37 – 4.86 (stack, 1H, H-7), 4.38 – 3.42 (stack, 7H, H-1, H-5a, H-6, H-8), 3.20 (app d, *J* = 14.5 Hz, 0.3H, H-5b), 2.95 (app d, *J* = 11.3 Hz, 0.3H, H-3a), 2.82 – 2.53 (stack, 2.4H, H-2a, H-3a, H-5b), 2.37 – 2.16 (stack, 1.3H, H-2b, H-3b), 2.15 – 1.96 (stack, 0.7H, H-3b), 1.47 (app s, 6.5H, H-15), 1.28 (app s, 2.5H, H-15).

^{13}C{^{1}H}-NMR (101 MHz, $CDCl_3$, mixture of rotamers and diastereoisomers) δ_C 155.4 (C, C-13), [139.5, 139.0, 136.9, 136.5 (C, C-9, C-16)], [129.51, 129.47, 128.7, 128.5, 128.4, 128.3, 127.8, 127.6, 127.4, 127.3, 125.9 (CH, C-10, C-11, C-12, C-17, C-18, C-19)], [96.8, 93.3, 92.8 (C, C-4)], [80.42, 80.36 (C, C-14)], [63.3, 63.1 (CH_2, C-8, C-6)], [61.5, 61.0 (CH_2, C-1)], [60.3, 58.4 (CH_2, C-3)], [57.4, 53.3 (CH, C-7)], [52.3, 51.9 (CH_2, C-2)], 51.3 (CH, C-7), [46.2, 45.7, 44.4 (CH_2, C-5)], [28.5, 28.3 (CH_3, C-15)].

LRMS (ES+): 425.2 [$(M + H)^+$], 100%], 369.2 [20, $(M + H – t\text{-Bu})^+$].

HRMS (ES+): calcd for $C_{25}H_{33}N_2O_4$ $[M + H]^+$ 425.2440, found 425.2445.

tert-butyl (3_S_)-3,10-dibenzyl-1,7-dioxa-4,10-diazaspiro[5.5]undecane-4-carboxylate (48):

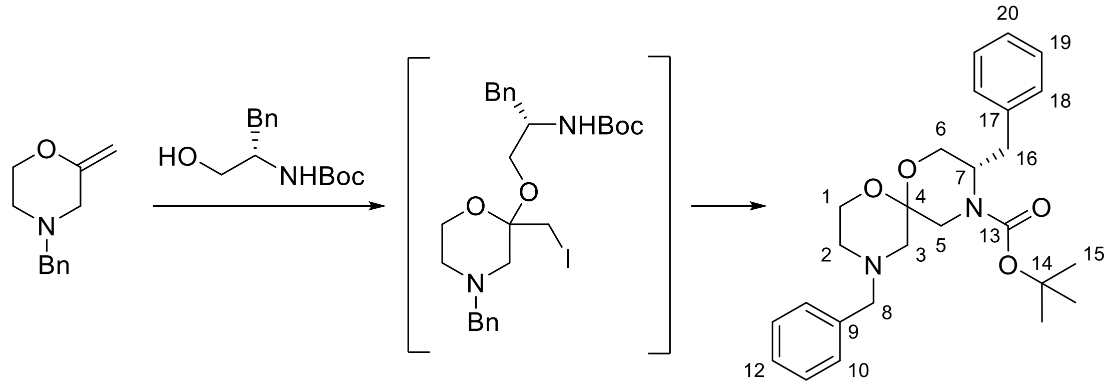

4 **48**

Following general procedure D (p 53), NIS (238 mg, 1.06 mmol) and *tert*-butyl (*S*)-(1-hydroxy-3-phenylpropan-2-yl)carbamate (319 mg, 1.27 mmol) were added to a solution of enol ether **4** (200 mg, 1.06 mmol) in CH_2Cl_2 (7.0 mL) at –40 °C. After 15 min, the reaction was worked up according to the general procedure to provide the iodo acetal intermediate as a light-green oil (520 mg, 87%, d.r. ~1:1, neutral LCMS method), which was used directly in the next step: *t*-BuOK (297 mg, 2.65 mmol) was added to a solution of the iodo acetal (0.50 g, 0.88 mmol) in DMF (4.4 mL) at rt. After 30 min, the reaction was worked up according to the general procedure to produce spiroacetal **48** as a light-green oil (332 mg, 72% over the two steps, d.r. ~1:1, neutral LCMS method). It was possible to separate one of the diastereoisomers of the mixture and the following characterization data are reported on that single diastereoisomer. It was not possible to assign the relative stereochemistry of this diastereoisomer.

R_f (*n*-heptane/EtOAc, 3/2) = 0.4.

ν_{max} (thin film/cm^{-1}): 2975 w, 1693 br s, 1152 m, 1060 m, 754 s.

^{1}H-NMR (400 MHz, $CDCl_3$, rotamers not observed) δ_H 7.33 – 6.99 (stack, 10H, Ph), 4.19 – 4.06 (m, 1H, H-7), 4.00 (app td, *J* = 11.1, 2.7 Hz, 1H, H-1 or H-6), 3.95 – 3.84 (m, 1H, H-5a), 3.61 – 3.47 (stack, 3H, H-1, H-6), 3.43 (br s, 2H, H-8), 2.89 – 2.76 (stack, 2H, H-3a, H-16a), 2.76 – 2.61 (stack, 2H [including 2.72 (d, *J* = 14.4 Hz, 1H, H-5b)], H-5b, H-16b), 2.60 – 2.53 (m, 1H, H-2a), 2.14 (app td, *J* = 11.2, 3.2 Hz, 1H, H-2b), 2.08 – 1.98 (m, 1H, H-3b), 1.30 (s, 9H, H-15).

^{13}C{^{1}H}-NMR (101 MHz, $CDCl_3$, rotamers not observed) δ_C 155.1 (C, C-13), [137.4, 136.9 (C, C-9, C-17)], [129.3, 129.2, 128.5, 128.2, 127.2, 126.5 (CH, C-10, C-11, C-12, C-18, C-19, C-20)], 96.3 (C, C-4), 80.0 (C, C-14), 63.2 (CH_2, C-8), [61.6, 61.4 (CH_2, C-1, C-6)], 59.0 (CH_2, C-3), 53.6 (CH, C-7), 52.3 (CH_2, C-2), 45.0 (CH_2, C-5), 36.3 (CH_2, C-16), 28.3 (CH_3, C-15).

LRMS (ES+): 439.3 [(M + H)$^+$], 100%], 383.2 [20, (M + H – *t*-Bu)$^+$].

HRMS (ES+): calcd for $C_{26}H_{35}N_2O_4$ [M + H]$^+$ 439.2597, found 439.2598.

10-benzyl 4-(*tert*-butyl) (3*S*)-3-benzyl-1,7-dioxa-4,10-diazaspiro[5.5]undecane-4,10-dicarboxylate (S36):

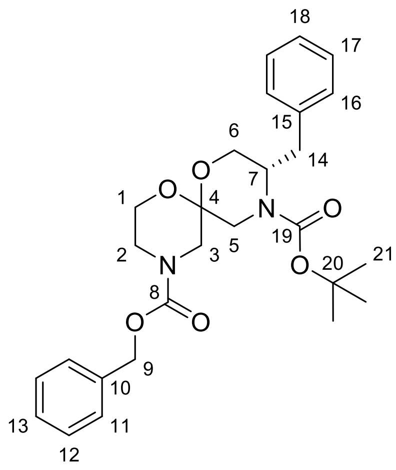

Under an Ar atmosphere, CbzCl (1.4 mL, 9.9 mmol) was added dropwise over 10 min to a solution of benzylamine **48** (1.9 g, 4.3 mmol) in CH_2Cl_2 (20 mL) at rt. After 2 days, the reaction mixture was diluted with CH_2Cl_2 (20 mL) and washed with $NaHCO_3$ solution (3 × 20 mL). The organic layer was dried over Na_2SO_4, filtered, and concentrated under reduced pressure. The crude product was purified by flash column chromatography (gradient, 0–75% EtOAc in *n*-heptane) to produce carbamate **S36** as a yellow oil (139 mg, 99%, d.r. ~1:1, neutral LCMS method).

R_f (*n*-heptane/EtOAc, 3/2) = 0.4.

ν_{max} (thin film/cm^{-1}): 2974 w, 1697 br s, 1417 m, 1136 m, 1061 m.

^{1}H-NMR (400 MHz, $CDCl_3$, mixture of rotamers and diastereoisomers) δ_H 7.41 – 6.99 (stack, 10H, Ph), 5.21 – 4.95 (stack, 2H, H-9), 4.33 – 3.30 (stack, 8H), 3.14 – 2.56 (stack, 5H), 1.45 – 1.13 (stack, 9H, H-21).

^{13}C{^{1}H}-NMR (101 MHz, $CDCl_3$, mixture of rotamers and diastereoisomers) δ_C [155.5, 154.8 (C, C-8, C-19)], [138.4, 138.1, 137.4, 136.5 (C, C-10, C-15)], [129.5, 129.4, 129.2, 128.6, 128.54, 128.47, 128.1, 127.9, 126.6 (CH, Ph)], [94.9, 91.8, 91.4 (C, C-4)], [80.4, 80.2 (C, C-20)], [67.5, 67.3 (CH_2, C-9)], [62.4, 61.8, 60.5, 60.2, 59.3, 53.1, 52.2, 50.4, 49.4, 48.9, 47.9, 45.3, 44.4, 43.7, 43.5, 43.1, 42.8 (CH_2, C-1, C-2, C-3, C-5, C-6)(CH, C-7)], [36.1, 35.9, 35.0, 34.4 (CH_2, C-14)], [28.3, 28.2 (CH_3, C-21)].

LRMS (ES+): 505.2 [(M + Na)$^+$], 100%], 427.2 [20, (M + H – *t*-Bu)$^+$].

HRMS (ES+): calcd for $C_{27}H_{34}N_2O_6Na$ [M + Na]$^+$ 505.2315, found 505.2325.

4-((9*H*-fluoren-9-yl)methyl) 10-benzyl (3*S*)-3-benzyl-1,7-dioxa-4,10-diazaspiro[5.5]undecane-4,10-dicarboxylate (55):

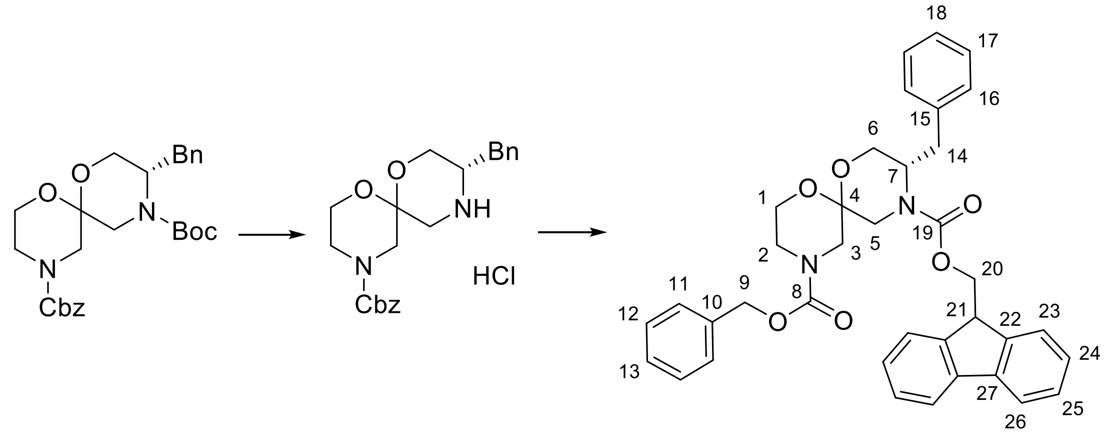

S36 **S37·HCl** **55**

Boc-protected amine **S36** (200 mg, 0.41 mmol) was added to a solution of HCl in 1,4-dioxane (1.0 mL of a 3 M solution, 3.0 mmol) at rt. After 1 h, the reaction mixture was concentrated under reduced pressure using a rotary evaporator maintained in a ducted fume cupboard to produce the crude 2° amine HCl salt **S37·HCl** (189 mg, quant., d.r. ~1:1, neutral LCMS method) as a yellow solid, which was used without further purification in the next step: FmocCl (167 mg, 0.64 mmol) and Et_3N (190 µL, 1.36 mmol) were added to a solution of the amine HCl salt **S37·HCl** (180 mg, 0.43 mmol) in CH_2Cl_2 (2.1 mL) at rt. After 1 day, more FmocCl (56 mg, 0.21 mmol) and Et_3N (65 µL, 0.45 mmol) were added to the reaction mixture. After 2 days, no further progress was observed, therefore the mixture was diluted with CH_2Cl_2 (10 mL) and extracted with hydrochloric acid (3 × 10 mL, 1 M). The combined organic phases were dried over Na_2SO_4, filtered, concentrated under reduced pressure and the residue was purified by flash column chromatography (gradient, 0–100% EtOAc in *n*-heptane) to produce Fmoc carbamate **55** as a light-yellow oil (105 mg, 60% yield over the two steps based on recovered amine **S37**, d.r. ~1:1, basic LCMS method). Intermediate amine **S37** was also recovered (45 mg, 0.12 mmol).

R_f (*n*-heptane/EtOAc, 3/2) = 0.7.

ν_{max} (KBr/cm^{-1}): 2930 w, 1701 br s, 1451 m, 1427 m, 1265 m.

^{1}H-NMR (400 MHz, $CDCl_3$, mixture of rotamers and diastereoisomers) δ_H 7.79 – 7.61 (stack, 2H), 7.59 – 7.40 (stack, 2H), 7.40 – 7.00 (stack, 12H), 6.98 – 6.69 (stack, 2H), 5.24 – 4.93 (stack, 2H), 4.58 – 4.20 (stack, 2H), 4.20 – 3.18 (stack, 9H), 3.12 – 2.28 (stack, 5H).

^{13}C{^{1}H}-NMR (101 MHz, $CDCl_3$, mixture of rotamers and diastereoisomers) δ_C [155.6, 155.3 (C, C-8, C-19)], [144.1, 143.9, 141.4, 141.3 (C, C-22, C-27)], [137.8, 136.6 (C, C-10, C-15)], [129.5, 129.4, 129.2, 128.6, 128.5, 127.9, 127.8, 127.7, 127.2, 127.1, 127.0, 126.6, 125.2, 124.7, 124.4, 120.0 (CH, Ar)], 94.7

(C, broad, C-4), [67.6, 67.3, 66.9 (CH_2, C-9)], [61.6, 60.2, 59.4, 53.2, 52.0, 51.1, 48.8, 47.4, 47.2, 44.8, 44.3, 42.8 (CH_2, C-1, C-2, C-3, C-5, C-6, C-20)(CH, C-7, C-21)], [35.5, 34.7, 34.5 (CH_2, C-14)].

LRMS (ES+): 627.3 [$(M + Na)^+$], 100%], 605.3 [85, $(M + H)^+$], 322.1 (30).

HRMS (ES+): calcd for $C_{37}H_{37}N_2O_6$ $[M + H]^+$ 605.2652, found 605.2654.

***tert*-butyl 10-benzyl-3,3-dimethyl-1,7-dioxa-4,10-diazaspiro[5.5]undecane-4-carboxylate (49):**

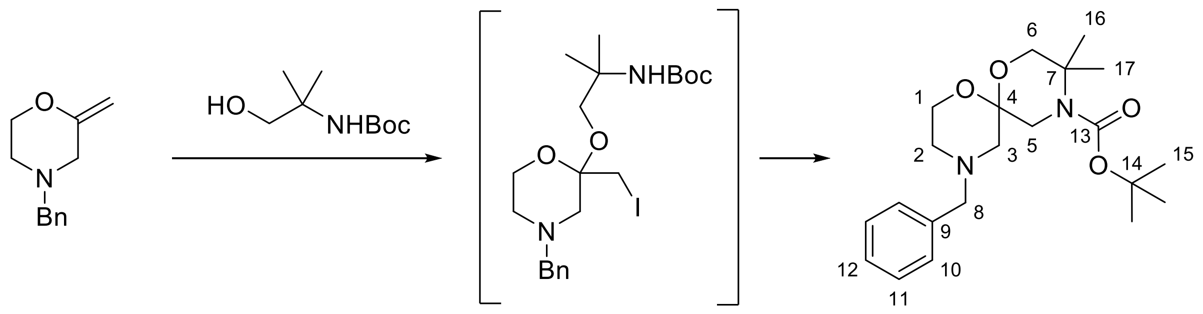

4 **49**

Following general procedure D (p 53), NIS (238 mg, 1.06 mmol) and *tert*-butyl (1-hydroxy-2-methylpropan-2-yl)carbamate (240 mg, 1.27 mmol) were added to a solution of enol ether **4** (200 mg, 1.06 mmol) in CH_2Cl_2 (7.0 mL) at –40 °C. After 30 min, the reaction was worked up according to the general procedure to provide the iodo acetal intermediate as a colorless oil (443 mg, 83%), which was used directly in the next step: *t*-BuOK (189 mg, 1.69 mmol) was added to a solution of the iodo acetal (425 mg, 0.84 mmol) in DMF (4.2 mL) at rt. After 30 min, the reaction was worked up according to the general procedure to produce spiroacetal **49** as a yellow oil (327 mg, 82% over the two steps).

R_f (*n*-heptane/EtOAc, 3/2) = 0.6.

ν_{max} (thin film/cm^{-1}): 2973 w, 1687 br s, 1393 s, 1147 s, 1060 s.

^{1}H-NMR (400 MHz, $CDCl_3$, rotamers not observed) δ_H 7.39 – 7.17 (stack, 5H, Ph), 4.04 (app td, J = 11.0, 2.5 Hz, 1H, H-1a), 3.76 (d, J = 11.9 Hz, 1H, H-6a), 3.71 – 3.50 (stack, 4H, H-1b, H-5a, H-8), 3.28 (d, J = 11.9 Hz, 1H, H-6b), 3.25 (d, J = 14.3 Hz, 1H, H-5b), 2.78 (d, J = 11.1 Hz, 1H, H-3a), 2.71 – 2.56 (m, 1H, H-2a), 2.36 – 2.23 (m, 1H, H-2b), 2.15 (d, J = 11.1 Hz, 1H, H-3b), 1.44 (s, 9H, H-15), [1.38 (s, 3H), 1.26 (s, 3H), H-16 or H-17].

^{13}C{^{1}H}-NMR (101 MHz, $CDCl_3$, rotamers not observed) δ_C 155.6 (C, C-13), 136.8 (C, C-9), 129.5 (CH, C-10), 128.3 (CH, C-11), 127.3 (CH, C-12), 96.3 (C, C-4), 80.1 (C, C-14), 70.5 (CH_2, C-6), 63.1 (CH_2, C-8), 61.2 (CH_2, C-1), 59.6 (CH_2, C-3), 55.0 (C, C-7), 52.3 (CH_2, C-2), 46.1 (CH_2, C-5), 28.6 (CH_3, C-15), [23.9, 22.6 (CH_3, C-16, C-17)].

LRMS (ES+): 377.2 [$(M + H)^+$], 100%], 321.2 [15, $(M + H – t\text{-Bu})^+$].

HRMS (ES+): calcd for $C_{21}H_{33}N_2O_4$ $[M + H]^+$ 377.2440, found 377.2444.

tert-butyl (2S)-10-benzyl-2-methyl-1,7-dioxa-4,10-diazaspiro[5.5]undecane-4-carboxylate (50):

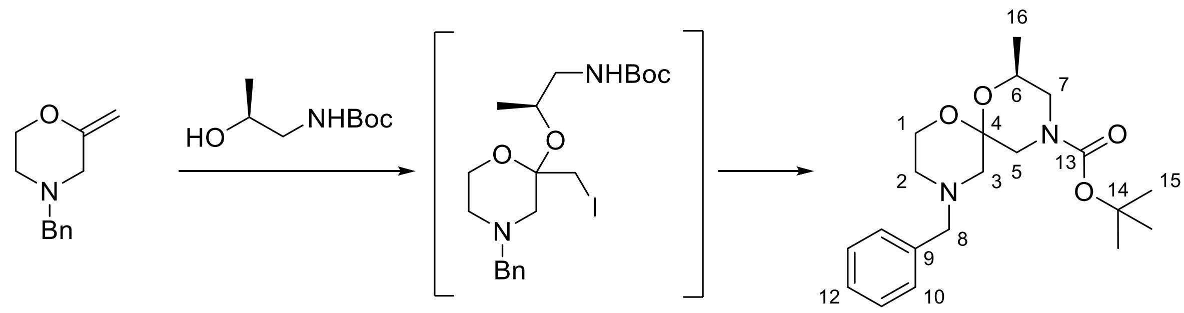

4 **50**

Following general procedure D (p 53), NIS (238 mg, 1.06 mmol) was added to a solution of *tert*-butyl (*S*)-(2-hydroxypropyl)carbamate (222 mg, 1.27 mmol) and enol ether **4** (200 mg, 1.06 mmol) in CH_2Cl_2 (7.0 mL) at –40 °C. After 10 min, the reaction was worked up according to the general procedure to provide the iodo acetal intermediate as a colorless oil (343 mg, 66%, d.r. ~1:1, neutral LCMS method), which was used directly in the next step: *t*-BuOK (156 mg, 1.39 mmol) was added in two portions to a solution of the iodo acetal (340 mg, 0.69 mmol) in DMF (3.5 mL) at rt. After 30 min, the reaction was worked up according to the general procedure to produce spiroacetal **50** as a colorless oil (197 mg, 51% over the two steps, d.r. ~1:1, based on ^{1}H-NMR spectroscopy data).

R_f (*n*-heptane/EtOAc, 3/2) = 0.4.

ν_{max} (thin film/cm^{-1}): 2973 m, 1699 br s, 1073 m, 1060 m.

^{1}H-NMR (400 MHz, $CDCl_3$, resonance broadening observed because of rotamers, ~1:1 mixture of diastereoisomers, based on the relative integration of the resonances for H-16 diastB at δ_H 1.23 ppm and H-16 diastA at 1.16 ppm) δ_H 7.40 – 7.18 (stack, 5H, Ph), 4.27 – 3.36 (stack, 7H, H-1, H-5a, H-6, H-7a, H-8), 3.20 – 2.40 (stack, 4H, H-2a, H-3a, H-5b, H-7b), 2.40 – 2.29 (stack, 1H, H-2b), 2.25 – 1.96 (stack, 1H, H-3b), 1.46 (s, 4.5H, H-15 diastA), 1.42 (s, 4.5H, H-15 diastB), 1.23 (d, *J* = 6.2 Hz, 1.5H, H-16 diastB), 1.16 (d, *J* = 6.3 Hz, 1.5H, H-16 diastA).

^{13}C{^{1}H}-NMR (101 MHz, $CDCl_3$, mixture of rotamers and diastereoisomers) δ_C 154.7 (C, C-13), 137.3 (C, C-9), [129.1, 128.9, 128.3, 128.3, 127.2 (CH, C-10, C-11, C-12)], [95.0, 94.4, 93.4 (C, C-4)], [80.1, 79.9 (C, C-14)], [67.7, 64.3 (CH, C-6)], [62.89, 62.87 (CH_2, C-8)], [61.6, 61.1 (CH_2, C-1)], [58.6, 55.4, 55.0, 54.9 (CH_2, C-3)], [52.8, 52.6, 52.2 (CH_2, C-2)], [49.9, 49.6, 48.5, 48.2, 47.7 (CH_2, C-5, C-7)], [28.42, 28.37 (CH_3, C-15)], [18.8, 18.3 (CH_3, C-16)].

LRMS (ES+): 363.2 [(M + H)$^+$], 100%], 307.2 [80, (M + H – *t*-Bu)$^+$].

HRMS (ES+): calcd for $C_{20}H_{31}N_2O_4$ [M + H]$^+$ 363.2284, found 363.2294.

tert-butyl (3S)-10-benzyl-3-isopropyl-2,2-dimethyl-1,7-dioxa-4,10-diazaspiro[5.5]undecane-4-carboxylate (51):

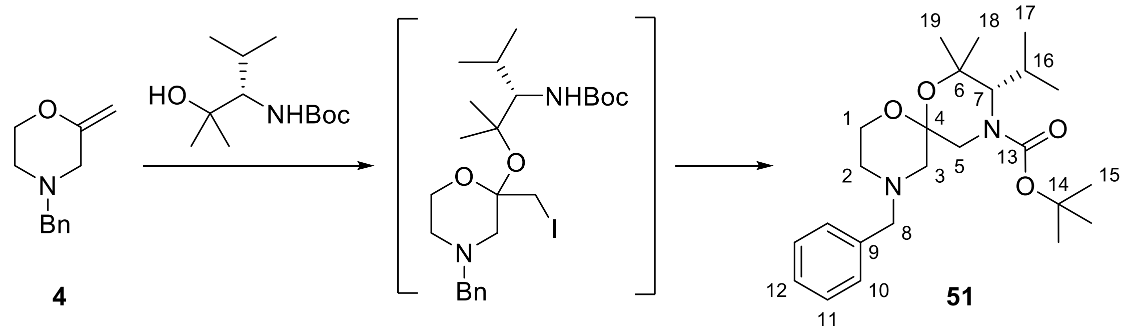

Following general procedure D (p 53), NIS (238 mg, 1.06 mmol) and *tert*-butyl (*S*)-(2-hydroxy-2,4-dimethylpentan-3-yl)carbamate (293 mg, 1.27 mmol) were added to a solution of enol ether **4** (200 mg, 1.06 mmol) in CH_2Cl_2 (7.0 mL) at –40 °C. After 15 min, the reaction was worked up according to the general procedure to provide the iodo acetal intermediate as a colorless oil (131 mg, 23%, d.r. ~1:1, neutral LCMS method), which was used directly in the next step: *t*-BuOK (42 mg, 0.40 mmol) was added to a solution of the iodo acetal (110 mg, 0.20 mmol) in DMF (3.8 mL) at rt. After 1 h, the reaction was worked up according to the general procedure to produce spiroacetal **51** as a red oil (83 mg, 19% over the two steps).[r]

R_f (*n*-heptane/EtOAc, 3/2) = 0.4.

ν_{max} (thin film/cm^{-1}): 2974 m, 1694 br s, 1165 m, 1149 m, 1062 m.

^{1}H-NMR (400 MHz, $CDCl_3$, mixture of rotamers and diastereoisomers, ratios could not be accurately determined) δ_H 7.25 – 6.97 (stack, 5H, Ph), 4.09 – 3.74 (stack, 2H, H-1a or H-3a, H-5a), 3.61 – 3.17 (stack, 4H, H-1, H-3, H-8), 2.81 – 2.70 (stack, 0.5H, H-5b), 2.60 – 2.28 (stack, 2.5H, H-1b or H-3b, H-2a, H-5b), 2.27 – 2.08 (stack, 1H, H-2b), 2.07 – 1.71 (stack, 2H, H-7, H-16), 1.35 – 1.29 (stack, 5H, H-15, H-18, H-19), 1.28 – 1.17 (stack, 8H, H-15, H-18, H-19), 1.16 – 1.10 (stack, 2H, H-15, H-18, H-19), 0.91 – 0.82 (stack, 3H, H-17a), 0.78 – 0.69 (stack, 3H, H-17b).

^{13}C{^{1}H}-NMR (101 MHz, $CDCl_3$, mixture of rotamers and diastereoisomers) δ_C [155.9, 155.4 (C, C-13)], [137.4, 137.1, 137.0 (C, C-9)], [129.11, 129.08, 129.0 (CH, C-10)], [128.29, 128.25, 128.2, 128.1 (CH, C-11)], [127.2, 127.14, 127.07, 127.0 (CH, C-12)], [95.4, 95.2, 93.5, 93.2 (C, C-4)], [79.9, 79.7, 79.6, 79.5 (C, C-14)], [75.4, 75.0, 74.7 (C, C-6)], [65.0, 63.9, 63.0, 62.8, 62.0, 61.3, 61.1, 60.8, 60.3, 60.1 (CH, C-7, CH_2, C-1, C-3, C-8)], [52.5, 52.2 (CH_2, C-2)], [45.4, 45.2, 44.3, 44.1 (CH_2, C-5)], [32.3, 32.0 (CH, C-16)], [28.74, 28.72, 28.5, 28.4, 28.3, 28.2 (CH_3, C-15, C-18, C-19)], 28.1 (CH, C-16), [27.9, 27.8 (CH_3, C-15, C-

[r] The d.r. of the product spiroacetal could not be determined, neither by LCMS methods because the peaks for the two diastereoisomers did not separate, nor by ^{1}H-NMR spectroscopy, because of resonance overlap. However, we expect the d.r. to remain ~1:1 after the ring closure of the iodoacetal intermediate, in analogy with other examples.

18, C-19)], 27.1 (CH, C-16), [26.7, 25.9, 25.7 (CH_3, C-15, C-18, C-19)], [23.4, 22.9, 22.8, 20.2, 20.1, 20.0, 19.8 (CH_3, C-17)].

LRMS (ES+): 419.3 [$(M + H)^+$], 100%], 363.2 [20, $(M + H – t\text{-Bu})^+$].

HRMS (ES+): calcd for $C_{24}H_{39}N_2O_4$ $[M + H]^+$ 419.2910, found 419.2922.

***tert*-butyl (4*aS*,8*aS*)-4'-benzylhexahydrospiro[benzo[b][1,4]oxazine-2,2'-morpholine]-4(3*H*)-carboxylate (52):**

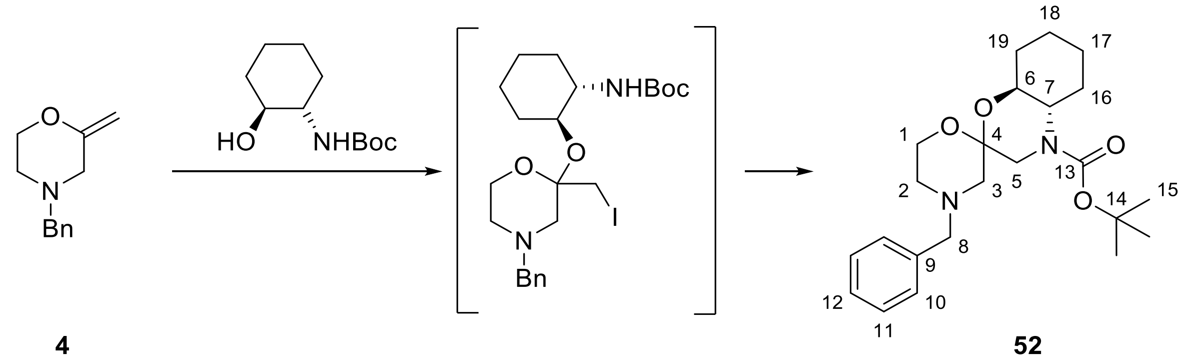

4 **52**

Following general procedure D (p 53), NIS (238 mg, 1.06 mmol) and *tert*-butyl ((1*R*,2*R*)-2-hydroxycyclohexyl)carbamate (273 mg, 1.27 mmol) were added to a solution of enol ether **4** (200 mg, 1.06 mmol) in CH_2Cl_2 (7.0 mL) at –40 °C. After 15 min, the reaction was worked up according to the general procedure to provide the iodo acetal intermediate as a colorless oil (360 mg, 64%, d.r. ~1:1, neutral LCMS method), which was used directly in the next step: *t*-BuOK (131 mg, 1.17 mmol) was added to a solution of the iodo acetal (310 mg, 0.584 mmol) in DMF (3.9 mL) at rt. After 1 h, the reaction was worked up according to the general procedure to produce spiroacetal **52** as a yellow oil (282 mg, 69% over the two steps).[s]

R_f (*n*-heptane/EtOAc, 3/2) = 0.7.

ν_{max} (thin film/cm^{-1}): 2937 m, 1695 br s, 1151 s, 1057 s.

^{1}H-NMR (400 MHz, $CDCl_3$, mixture of rotamers and diastereoisomers, ratios could not be accurately determined) δ_H 7.38 – 7.18 (stack, 5H, Ph), 4.56 (br d, *J* = 14.5 Hz, 0.5H, H-5a), 4.25 – 4.03 (stack, 1H, H-1a, H-5a), 3.86 (app dd, *J* = 6.1, 3.5 Hz, 1H, H-1b), 3.76 – 3.37 (stack, 4H, H-1a, H-6, H-7, H-8), 3.31 (app td, *J* = 10.9, 3.2 Hz, 0.5H, H-7), 3.07 (d, *J* = 8.0 Hz, 0.5H, H-5b), 3.04 (d, *J* = 8.2 Hz, 0.5H, H-5b), 2.68 (d, *J* = 11.2 Hz, 0.5H, H-3a), 2.62 – 2.45 (stack, 1.5H, H-3a, H-2), 2.44 – 2.17 (stack, 2.5H, H-2, H-3, H-16

[s] The d.r. of the product spiroacetal could not be determined, neither by LCMS methods because the peaks for the two diastereomers did not separate, nor by ^{1}H-NMR spectroscopy, because of resonance overlap. However, we expect the d.r. likely to remain ~1:1 after the ring closure of the iodoacetal intermediate, in analogy with other examples.

or H-19), 2.17 – 1.93 (stack, 1.5H, H-16, H-19), 1.88 – 1.62 (stack, 2.5H, H-17, H-18), 1.55 – 1.00 (stack, 12.5H, H-15, H-16, H-17, H-18, H-19).

$^{13}C\{^1H\}$-NMR (101 MHz, $CDCl_3$, mixture of rotamers and diastereoisomers) δ_C [156.6, 155.3 (C, C-13)], [137.8, 136.8 (C, C-9)], [129.3, 128.9 (CH, C-10)], [128.3, 128.2 (CH, C-11)], [127.18, 127.16 (CH, C-12)], [98.1, 97.8 (C, C-4)], [79.83, 79.77 (C, C-14)], [74.0, 72.1 (CH, C-6)], [63.0, 62.7 (CH_2, C-8)], [62.5, 61.4 (CH_2, C-1)], 60.3 (CH, C-7), 60.0 (CH_2, C-3), 59.4 (CH, C-7), 58.8 (CH_2, C-3), [52.5, 52.1 (CH_2, C-2)], [45.1, 42.8 (CH_2, C-5)], [32.1, 31.6, 30.7, 29.1 (CH_2, C-16, C-19)], 28.4 (CH_3, C-15), [24.7, 24.5, 24.4, 24.3 (CH_2, C-17, C-18)].

LRMS (ES+): 403.3 [$(M + H)^+$], 100%], 347.2 [45, $(M + H – t\text{-Bu})^+$].

HRMS (ES+): calcd for $C_{23}H_{35}N_2O_4$ $[M + H]^+$ 403.2597, found 403.2606.

***tert*-butyl 4'-benzylspiro[benzo[b][1,4]oxazine-2,2'-morpholine]-4(3*H*)-carboxylate (53):**

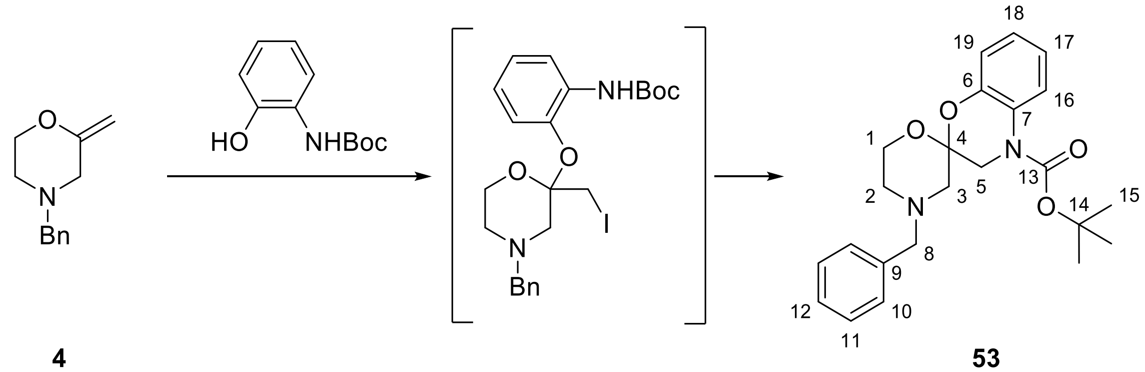

4 **53**

Following general procedure D (p 53), NIS (238 mg, 1.06 mmol) and Boc-anilinophenol (265 mg, 1.27 mmol) were added to a solution of enol ether **4** (200 mg, 1.06 mmol) in CH_2Cl_2 (7.0 mL) at –40 °C. After 45 min, the reaction was worked up according to the general procedure to provide the iodo acetal intermediate as a colorless oil (272 mg, 49%), which was used directly in the next step: *t*-BuOK (111 mg, 0.99 mmol) was added to a solution of the iodo acetal (260 mg, 0.496 mmol) in DMF (2.5 mL) at rt. After 30 min, the reaction was worked up according to the general procedure. After purification via column chromatography, it was necessary to further purify the compound using the neutral preparative HPLC method to produce spiroacetal **53** as a brown oil (43 mg, 10% over the two steps).

R_f (*n*-heptane/EtOAc, 3/2) = 0.6.

ν_{max} (thin film/cm^{-1}): 2976 m, 1701 br s, 1496 s, 1369 s, 1082 s.

^1H-NMR (400 MHz, $CDCl_3$, rotamers not observed) δ_H 7.62 (br s, 1H, H-16), 7.46 – 7.19 (stack, 5H, Ph), 7.06 – 6.95 (stack, 2H, Ar), 6.95 – 6.84 (m, 1H, Ar), 4.51 (app s, 1H, H-5a), 4.12 – 3.94 (m, 1H, H-1a), 3.79 – 3.70 (m, 1H, H-1b), 3.66 (A of AB, $J_{A–B}$ = 13.2 Hz, 1H, H-8a), 3.59 (B of AB, $J_{B–A}$ = 13.2 Hz, 1H, H-

8b), 3.08 – 2.87 (m, 1H, H-5b), 2.79 (app d, J = 11.3 Hz, 1H, H-3a), 2.71 – 2.57 (m, 1H, H-2a), 2.55 – 2.43 (m, 1H, H-2b), 2.43 – 2.35 (m, 1H, H-3b), 1.53 (s, 9H, H-15).

$^{13}C\{^{1}H\}$-NMR (101 MHz, $CDCl_3$, rotamers not observed) δ_C 153.6 (C, C-13), 144.4 (C, C-6), 136.9 (C, C-9), 129.3 (CH, C-10), 128.5 (CH, C-11), 127.5 (CH, C-12), 126.1 (C, C-7), 124.7 (CH, Ar), 124.1 (CH, C-16), 120.7 (CH, Ar), 117.5 (CH, Ar), 94.4 (C, C-4), 81.7 (C, C-14), 62.8 (CH_2, C-8), 62.3 (CH_2, C-1), 57.5 (CH_2, C-3), 52.1 (CH_2, C-2), 46.9 (CH_2, C-5), 28.4 (CH_3, C-15).

LRMS (ES+): 397.2 [$(M + H)^+$, 100%], 341.2 [10, $(M + H – t\text{-Bu})^+$].

HRMS (ES+): calcd for $C_{23}H_{29}N_2O_4$ $[M + H]^+$ 397.2127, found 397.2133.

***tert*-butyl (8*S*)-10-benzyl-8-methyl-1,7-dioxa-4,10-diazaspiro[5.5]undecane-4-carboxylate (43):**

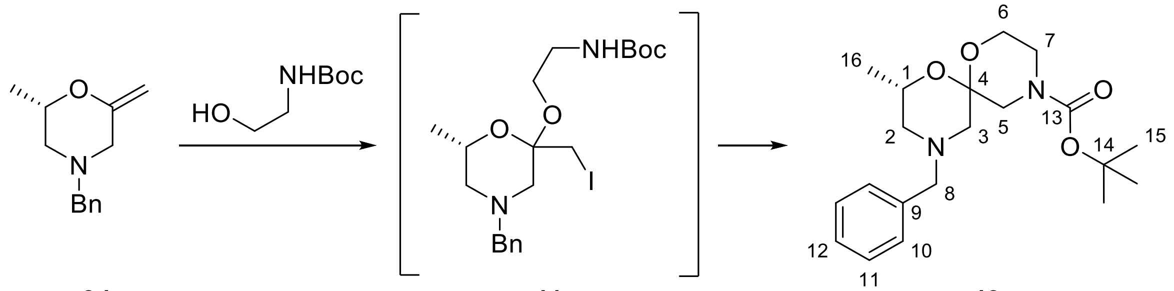

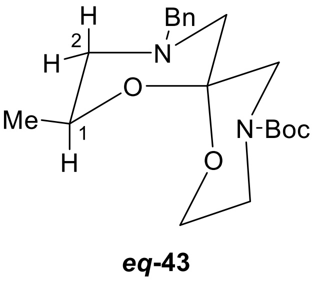

***eq*-43**

Following general procedure D (p 53), NIS (587 mg, 2.61 mmol) and *N*-Boc-ethanolamine (504 mg, 3.13 mmol) were added to a solution of enol ether **34** (530 mg, 2.61 mmol) in CH_2Cl_2 (17 mL) at –40 °C. After 15 min, the reaction was worked up according to the general procedure to provide iodo acetal **41** as a light-brown oil (950 mg, 74%, d.r. ~4:1, basic LCMS method), which was used directly in the next step: *t*-BuOK (435 mg, 3.87 mmol) was added to a solution of iodo acetal **41** (950 mg, 1.94 mmol) in DMF (10 mL) at rt. After 30 min, the reaction was worked up according to the general procedure to produce spiroacetal **43** as a colorless oil (692 mg, 67% over the two steps, d.r. ~4:1, basic LCMS method). It was possible to separate the major diastereoisomer from the mixture and the following characterization data are reported on this isomer (***eq*-43**).

R_f (*n*-heptane/EtOAc, 3/2) = 0.4.

ν_{max} (thin film/cm^{-1}): 2974 m, 2777 w, 1697 s, 1455 m, 1270 m, 1058 s.

^{1}H-NMR (400 MHz, $CDCl_3$, rotamers not observed) δ_H 7.39 – 7.12 (stack, 5H, Ph), 4.03 – 3.93 (m, 1H, H-1), 3.93 – 3.62 (stack, 3H, H-5a, H-6a, H-7a), 3.60 – 3.41 (stack, 3H, H-6b, H-8), 3.11 – 2.86 (m, 1H, H-7b), 2.86 – 2.62 (stack, 3H, H-2a, H-3a, H-5b), 1.93 – 1.72 (stack, 2H [including 1.81 (app t, *J* = 10.9 Hz, 1H, H-2b)], H-2b, H-3b), 1.43 (s, 9H, H-15), 1.08 (d, *J* = 6.3 Hz, 3H, H-16).

^{13}C{^{1}H}-NMR (101 MHz, $CDCl_3$, mixture of rotamers) δ_C 155.5 (C, C-13), 136.7 (C, C-9), 129.5 (CH, C-10), 128.3 (CH, C-11), 127.3 (CH, C-12), 93.0 (C, C-4), 79.8 (C, C-14), 65.2 (CH, C-1), 63.1 (CH_2, C-8), 59.4 (CH_2, C-6), 58.7 (CH_2, C-2), 57.2 (CH_2, C-3), [50.7, 49.3 (CH_2, C-5)], [43.7, 42.6 (CH_2, C-7)], 28.4 (CH_3, C-15), 18.7 (CH_3, C-16).

LRMS (ES+): 363.2 [(M + H)$^+$, 100%], 307.2 [50, (M + H – *t*-Bu)$^+$].

HRMS (ES+): calcd for $C_{20}H_{31}N_2O_4$ [M + H]$^+$ 363.2284, found 363.2286.

tert-butyl (9*S*)-10-benzyl-9-methyl-1,7-dioxa-4,10-diazaspiro[5.5]undecane-4-carboxylate (42):

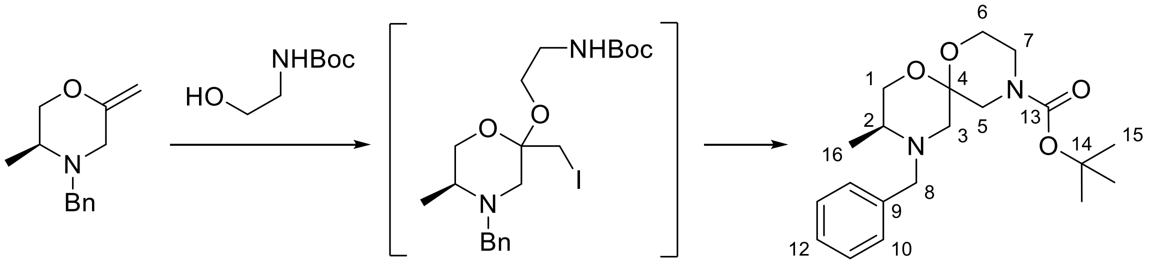

39 **40** **42**

Following the general procedure for spiroacetal formation (p 53), NIS (332 mg, 1.48 mmol) and *N*-Boc-ethanolamine (286 mg, 1.77 mmol) were added to a solution of enol ether **39** (300 mg, 1.48 mmol) in CH_2Cl_2 (10 mL) at –40 °C. After 15 min, the reaction was worked up according to the general procedure to provide iodo acetal **40** as a colorless oil (520 mg, 72%, d.r. ~1:1, basic LCMS method), which was used directly in the next step: *t*-BuOK (238 mg, 2.12 mmol) was added to a solution of iodo acetal **40** (520 mg, 1.06 mmol) in DMF (5.0 mL) at rt. After 2 h, the reaction was worked up according to the general procedure to produce spiroacetal **42** as a colorless oil (360 mg, 67% over the two steps, d.r. ~1:1, basic LCMS method). It was possible to isolate a sample of each diastereoisomer (diastA, 46 mg, 9% yield over two steps, diastB 43 mg, 8% yield over two steps) and the data are reported on each isomer. It was not possible to assign, which diastereoisomer is which.

ν_{max} (thin film/cm^{-1}): 2972 m, 2808 w, 1698 br s, 1454 m, 1174 m, 1059 m.

diastA:

^{1}H-NMR (400 MHz, $CDCl_3$, resonance broadening observed because of rotamers) δ_H 7.33 – 7.03 (stack, 5H, Ph), 4.30 – 4.01 (m, 1H, H-5a), 4.01 – 3.61 (stack, 4H, H-1a, H-6a, H-7a, H-8a), 3.57 – 3.30 (stack, 2H, H-1b, H-6b), 3.29 – 3.11 (m, 1H, H-8b), 2.99 – 2.75 (m, 1H, H-7b), 2.66 – 2.38 (stack, 3H, H-2, H-3a, H-5b), 2.15 (d, *J* = 11.8 Hz, 1H, H-3b), 1.40 (s, 9H, H-15), 1.01 (d, *J* = 6.3 Hz, 3H, H-16).

^{13}C{^{1}H}-NMR (101 MHz, $CDCl_3$, mixture of rotamers) δ_C 155.0 (C, C-13), 138.5 (C, C-9), 128.8 (CH, C-10), 128.3 (CH, C-11), 127.0 (CH, C-12), [93.6, 93.1 (C, C-4)], 79.9 (C, C-14), 67.2 (CH_2, C-1), 59.7 (CH_2, C-6), 58.2 (CH_2, C-8), 54.8 (CH_2, C-3), [53.4, 53.1 (CH, C-2)], [48.0, 46.5 (CH_2, C-5)], [43.7, 42.6 (CH_2, C-7)], 28.4 (CH_3, C-15), 11.6 (CH_3, C-16).

diastB:

^{1}H-NMR (400 MHz, $CDCl_3$, mixture of rotamers, ratio could not be accurately determined) δ_H 7.34 – 7.10 (stack, 5H, Ph), 4.15 – 3.97 (stack, 1H, H-8a), 3.90 – 3.35 (stack, 6H, H-1, H-5a, H-6, H-7a), 3.30 – 3.01 (stack, 1H, H-8b), 2.99 – 2.75 (stack, 1H, H-7b), 2.73 – 2.51 (stack, 2H, H-3a, H-5b), 2.49 – 2.33 (stack, 1H, H-2), 1.89 (d, *J* = 11.5 Hz, 1H, H-3b), 1.36 (s, 9H, H-15), 1.08 (d, *J* = 5.5 Hz, 3H, H-16).

^{13}C{^{1}H}-NMR (101 MHz, $CDCl_3$, mixture of rotamers) δ_C 155.0 (C, C-13), 137.2 (C, C-9), 129.4 (CH, C-10), 128.3 (CH, C-11), 127.2 (CH, C-12), 93.2 (C, C-4), 80.0 (C, C-14), 66.2 (CH_2, C-1), 59.4 (CH_2, C-6), 58.0 (CH_2, C-8), 56.6 (CH_2, C-3), 54.6 (CH, C-2), [50.0, 48.8 (CH_2, C-5)], [43.6, 42.2 (CH_2, C-7)], 28.4 (CH_3, C-15), 14.9 (CH_3, C-16).

LRMS (ES+): 363.2 [$(M + H)^+$, 100%], 307.2 [30, $(M + H – t\text{-Bu})^+$].

HRMS (ES+): calcd for $C_{20}H_{31}N_2O_4$ $[M + H]^+$ 363.2284, found 363.2289.

tert-butyl (3_S_,9_S_)-3,10-dibenzyl-9-methyl-1,7-dioxa-4,10-diazaspiro[5.5]undecane-4-carboxylate (54):

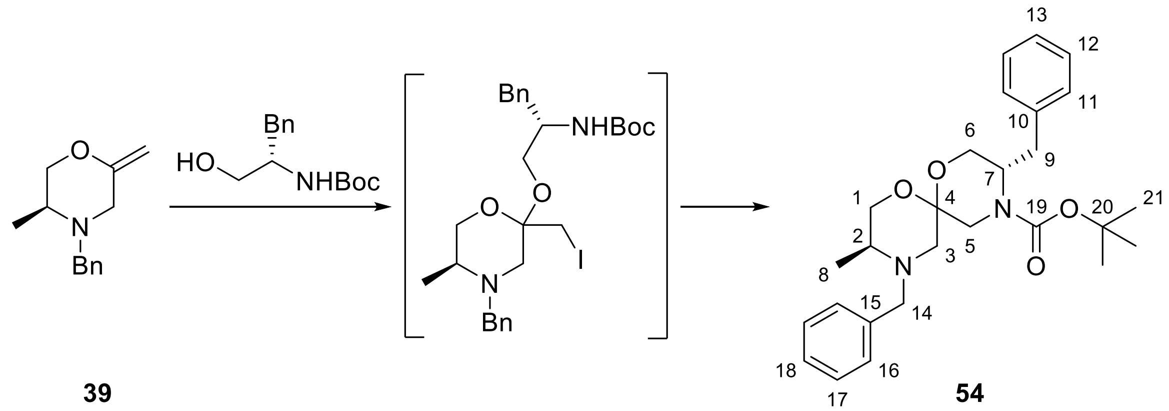

39 54

Following general procedure D (p 53), NIS (1.49 g, 6.64 mmol) and *tert*-butyl (*S*)-(1-hydroxy-3-phenylpropan-2-yl)carbamate (2.00 g, 7.97 mmol) were added to a solution of enol ether **39** (1.35 g,

6.64 mmol) in CH_2Cl_2 (44 mL) at –40 °C. After 15 min, the reaction was worked up according to the general procedure to provide the iodo acetal intermediate as a brown glass (2.76 g, 70%, d.r. ~1:1, basic LCMS method), which was used directly in the next step: *t*-BuOK (1.60 g, 14.3 mmol) was added to a solution of the iodo acetal (2.76 g, 4.75 mmol) in DMF (23.8 mL) at rt. After 30 min, the reaction was worked up according to the general procedure to produce spiroacetal **54** as an orange oil (1.71 g, 47% over the two steps, d.r. ~1:1, basic LCMS method).

R_f (*n*-heptane/EtOAc, 3/2) = 0.5.

ν_{max} (thin film/cm^{-1}): 2976 w, 1692 br s, 1151 m.

^{1}H-NMR (400 MHz, $CDCl_3$, mixture of rotamers and diastereoisomers) δ_H 7.47 – 6.95 (stack, 10H, Ph), 4.29 – 3.66 (stack, 4H), 3.55 – 3.01 (stack, 3H), 2.95 – 2.18 (stack, 5H, including H-9), 2.05 – 1.49 (stack, 2H), 1.42 – 0.79 (stack, 12H, H-8, H-21).

^{13}C{^{1}H}-NMR (101 MHz, $CDCl_3$, mixture of rotamers and diastereoisomers) δ_C [155.0, 154.8 (C, C-19)], [138.4, 137.3 (C, C-10, C-15)], [129.5, 129.4, 129.2, 128.54, 128.48, 126.5 (CH, C-11, C-12, C-13, C-16, C-17, C-18)], [96.2, 93.0 (C, C-4)], 80.1 (C, C-20), [66.8, 61.7, 60.6, 58.3, 57.9, 53.5, 52.9, 52.3 (CH_2, C-1, C-3, C-6, C-14)(CH, C-2, C-7)], [44.9, 42.5 (CH_2, C-5)], [36.2, 35.0, 34.5 (CH_2, C-9)], [28.4, 28.2 (CH_3, C-21)], 14.7 (CH_3, C-8).

LRMS (ES+): 453.3 [(M + H)$^+$, 100%], 397.2 [15, (M + H – *t*-Bu)$^+$].

HRMS (ES+): calcd for $C_{27}H_{37}N_2O_4$ [M + H]$^+$ 453.2753, found 453.2763.

(3*S*,9*S*)-4,9-dibenzyl-3-methyl-1,7-dioxa-4,10-diazaspiro[5.5]undecane (S38):

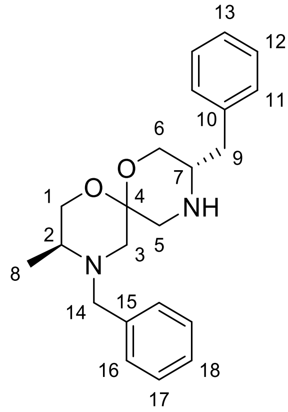

Boc-protected amine **54** (500 mg, 1.10 mmol) was added to a solution of HCl in 1,4-dioxane (1.2 mL of a 4 M solution, 4.8 mmol) at rt. After 30 min, the reaction mixture turned into a thick paste, therefore MeOH (0.5 mL) was added as a co-solvent. After 2 h, the volatiles were removed under reduced pressure using a rotary evaporator maintained in a ducted fume cupboard and the crude product was

purified by flash column chromatography (gradient, 0–10% MeOH in CH_2Cl_2 + 1 v/v% Et_3N) to provide 2° amine **S38** as a brown oil (275 mg, 71%, d.r. ~1:1, based on ^{1}H-NMR spectroscopy data).

ν_{max} (neat/cm^{-1}): 3322 w, 2964 m, 1602 w, 1494 m, 1453 s.

^{1}H-NMR (400 MHz, CD_3OD, ~1:1 mixture of diastereoisomers, based on the relative integration of the resonances for H-14a diastA at δ_H 3.56 ppm and H-14b diastB at 3.05 – 2.95 ppm) δ_H 7.42 – 6.93 (stack, 10H, Ph), 3.99 – 3.87 (stack, 1H, H-1a diastA, H-14a diastB), 3.75 (app br d, *J* = 11.6 Hz, 0.5H, H-6a diastA), 3.56 (app d, *J* = 13.2 Hz, 0.5H, H-14a diastA), 3.49 – 3.22 (stack, 2.5H, H-1 diastB, H-1b diastA, H-6 diastB, H-14b diastA), 3.18 – 3.15 (m, 0.5H, H-6b diastA), 3.05 – 2.95 (m, 0.5H, H-14b diastB), 2.88 – 2.47 (stack, 5H), 2.47 – 2.33 (stack, 2.5H), 2.33 – 2.23 (m, 0.5H), 1.80 – 1.68 (m, 0.5H), 1.04 – 0.92 (stack, 3H, H-8), NH was not observed.

^{13}C{^{1}H}-NMR (101 MHz, CD_3OD, mixture of diastereoisomers) δ_C [140.3, 139.5, 139.0, 137.9 (C, C-10, C-15)], [130.8, 130.3, 130.2, 130.1, 129.6, 129.3 (CH, C-11, C-12, C-16, C-17)], [128.3, 128.2, 127.5, 127.4 (CH, C-13, C-18)], [93.9, 93.0 (C, C-4)], [66.7, 66.5 (CH_2, C-1)], [65.3, 62.6 (CH_2, C-6)], [59.7, 58.8 (CH_2, C-14)], [57.8, 56.5, 56.2, 54.1, 53.0, 52.8, 52.5, 47.2 (CH_2, C-3, C-5)(CH, C-2, C-7)], [39.3, 36.6 (CH_2, C-9)], [14.9, 9.4 (CH_3, C-8)].

LRMS (ES+): 353.2 [(M + H)$^+$, 100%].

HRMS (ES+): calcd for $C_{22}H_{29}N_2O_2$ [M + H]$^+$ 353.2229, found 353.2238.

(9*H*-fluoren-9-yl)methyl (3*S*,9*S*)-3,10-dibenzyl-9-methyl-1,7-dioxa-4,10-diazaspiro[5.5]undecane-4-carboxylate (S39):

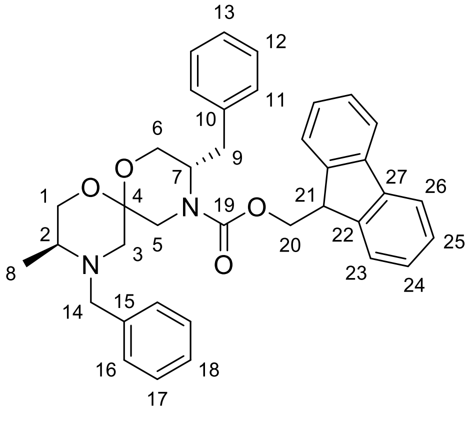

Under an Ar atmosphere, FmocCl (275 mg, 1.06 mmol) was added in one portion to a solution of Et_3N (0.31 mL, 2.2 mmol) and 2° amine **S38** (250 mg, 0.709 mmol) in CH_2Cl_2 (3.5 mL) at rt. After 2 days, more FmocCl (275 mg, 1.06 mmol) and Et_3N (0.31 mL, 2.2 mmol) were added to the mixture. After a further 3 days, the reaction mixture was diluted with CH_2Cl_2 (10 mL) and washed with hydrochloric acid (3 × 15 mL of a 1 M solution). The organic layer was washed with brine (20 mL), dried over Na_2SO_4,

concentrated under reduced pressure and the crude product was purified by flash column chromatography (gradient, 0–100% EtOAc in *n*-heptane) to produce carbamate **S39** as a colorless oil (293 mg, 72%, d.r. ~1:1, neutral LCMS method). The final product contained an unidentified minor impurity.

ν_{max} (thin film/cm^{-1}): 3445 w, 2872 w, 1698 s, 1450 m.

^{1}H-NMR (400 MHz, $CDCl_3$, mixture of rotamers and diastereoisomers) δ_H 7.74 – 7.62 (stack, 4H, Fmoc), 7.60 – 7.40 (stack, 4H, Fmoc), 7.37 – 7.02 (stack, 10H, Ph), 4.44 – 4.30 (stack, 1H), 4.29 – 4.08 (stack, 2H), 4.08 – 3.97 (stack, 1H), 3.97 – 3.89 (stack, 2H), 3.87 – 3.65 (stack, 2H), 3.48 – 3.19 (stack, 3H), 2.96 – 2.44 (stack, 5H), 2.44 – 2.19 (stack, 1H), 1.12 – 0.84 (stack, 3H, H-8).

^{13}C{^{1}H}-NMR (101 MHz, $CDCl_3$, mixture of rotamers and diastereoisomers) δ_C [155.6, 155.4 (C, C-19)], [144.4, 144.2, 144.1, 143.92, 143.87, 141.6, 141.40, 141.37, 138.4, 138.0, 129.5, 129.4, 129.3, 128.9, 128.54, 128.46, 127.7, 127.6, 127.1, 126.6, 126.5, 125.2, 125.1, 124.9, 124.8, 120.1, 120.0 (C, CH, Ar, Ph)], [93.0, 92.4 (C, C-4)], [67.4, 66.9, 65.2, 59.9, 59.7, 58.4, 58.3, 54.5, 53.9, 53.4, 52.8, 52.2, 51.4, 50.4, 47.4, 47.3, 44.0, 43.1, 34.8, 34.6 (CH_2, C-1, C-3, C-5, C-6, C-9, C-14, C-20)(CH, C-2, C-7, C-21)], [14.9, 10.9, 10.5 (CH_3, C-8)].

LRMS (ES+): 575.3 [$(M + H)^+$, 100%], 414.3 (20).

HRMS (ES+): calcd for $C_{37}H_{39}N_2O_4$ $[M + H]^+$ 575.2910, found 575.2924.

4-((9*H*-fluoren-9-yl)methyl) 10-benzyl (3*S*,9*S*)-3-benzyl-9-methyl-1,7-dioxa-4,10-diazaspiro[5.5]undecane-4,10-dicarboxylate (56):

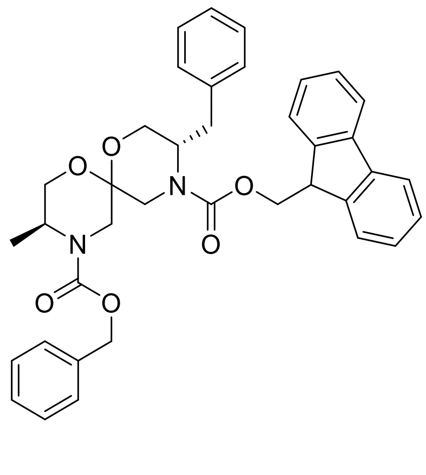

Under an Ar atmosphere, CbzCl (145 µL, 1.01 mmol) was added dropwise over 5 min to a solution of benzylamine **S39** (220 mg, 0.38 mmol) in CH_2Cl_2 (1.9 mL) at rt. After 2 days, more CbzCl (145 µL, 1.01 mmol) was added to the mixture. After a further 3 days, no further progress was observed by LCMS, therefore the reaction mixture was diluted with CH_2Cl_2 (5 mL) and washed with $NaHCO_3$ solution (3 ×

5 mL). The organic layer was dried over Na_2SO_4, concentrated under reduced pressure and the crude product was purified by flash column chromatography (gradient, 0–100% EtOAc in *n*-heptane) to produce carbamate **56** as a light-yellow oil (41 mg, 17%, d.r. ~1:1, neutral LCMS method). An analytically pure sample of carbamate **56** could not be obtained, thus, only selected data are reported:
ν_{max} (KBr/cm^{-1}): 3439 s, 1700 s, 1422 m.
LRMS (ES+): 641.3 [(M + Na)$^+$, 35%], 480.2 (100).
HRMS (ES+): calcd for $C_{38}H_{38}N_2O_6Na$ [M + Na]$^+$ 641.2628, found 641.2625.

2.7 Library compounds

2.7.1 General procedure E – Urea formation

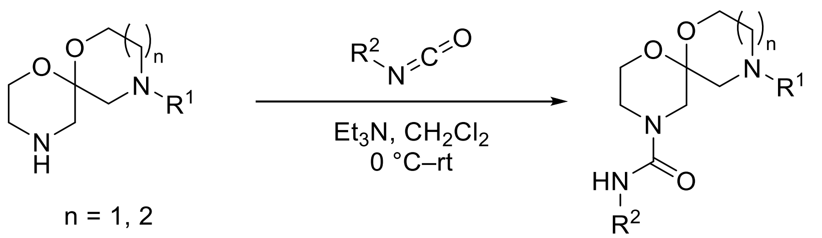

Under an Ar atmosphere, the isocyanate (5.0 mmol, 1.1 eq) was added to a solution of Et_3N (9.0 mmol, 2.0 eq) and the 2° amine (4.5 mmol, 1.0 eq) in CH_2Cl_2 (45 mL, 0.1 mmol mL^{-1}) at 0 °C. The resulting mixture was allowed to gradually warm to rt without removing the ice bath (~2 h). After 1–4 h (typical reaction time) at rt, the reaction was complete. The reaction mixture was diluted with CH_2Cl_2 (1 × reaction volume) and washed sequentially with H_2O (2 × reaction volume) and brine (1 × reaction volume). The combined organic phases were dried over Na_2SO_4 and concentrated under reduced pressure. The residue was purified by flash column chromatography.

2.7.2 General procedure F – Sulfonamide formation

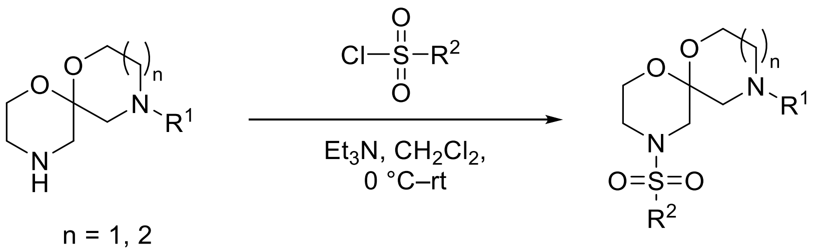

Under an Ar atmosphere, the sulfonyl chloride (0.45 mmol, 1.5 eq) was added to a solution of Et_3N (0.90 mmol, 3.0 eq) and the 2° amine (0.30 mmol, 1.0 eq) in CH_2Cl_2 (3 mL, 0.1 mmol mL^{-1}) at 0 °C. The

resulting mixture was allowed to gradually warm to rt without removing the ice bath (~2 h). After 18–24 h (typical reaction time) at rt, the reaction was complete. The reaction mixture was diluted with CH_2Cl_2 (1 × reaction volume) and washed sequentially with H_2O (2 × reaction volume) and brine (1 × reaction volume). The combined organic phases were dried over Na_2SO_4 and concentrated under reduced pressure. The residue was purified by flash column chromatography.

2.7.3 General procedure G – Amide formation

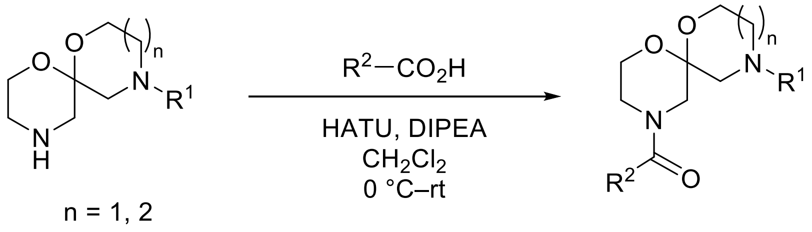

Under an Ar atmosphere, *i*-Pr_2NEt (18 or 36 mmol, 1.5 or 3.0 eq, see individual experimental procedures) and HATU (14 mmol, 1.2 eq) were added sequentially to a solution of the carboxylic acid (14 mmol, 1.2 eq) in CH_2Cl_2 (108 mL, 0.11 mmol mL^{-1}) at 0 °C. A solution of the 2° amine (12 mmol, 1.0 eq) in CH_2Cl_2 (12 mL, 1.0 mmol mL^{-1}) was then added at 0 °C (final reaction concentration 0.1 mmol mL^{-1}). After 30 min, the pH of the mixture was checked and adjusted, if necessary, to pH 8–9 by the addition of more *i*-Pr_2NEt. The resulting mixture was allowed to gradually warm to rt without removing the ice bath (~2 h). After 14–18 h (typical reaction time) at rt, the reaction was complete. The reaction mixture was diluted with CH_2Cl_2 (1 × reaction volume) and washed sequentially with H_2O (2 × reaction volume) and brine (1 × reaction volume). The combined organic phases were dried over Na_2SO_4 and concentrated under reduced pressure. The residue was purified by flash column chromatography.

2.7.4 General procedure H – Boc-deprotection of mono-decorated scaffolds

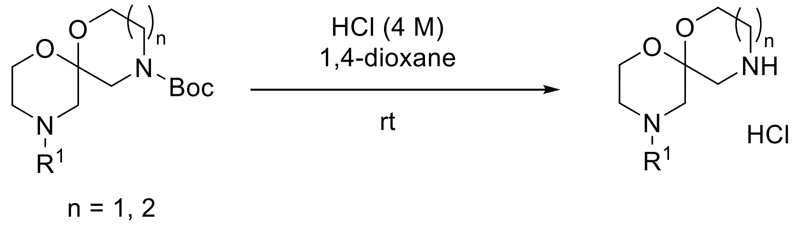

Boc-Protected amine (6.0 mmol, 1.0 eq) was added to a solution of HCl in 1,4-dioxane (4.0 M solution, 30 mmol, 5.0 eq) at rt. In those cases, where the amine did not completely dissolve, CH_2Cl_2 was included as a co-solvent, see the individual experimental procedures. After 1–4 h (typical reaction

time), the volatiles were removed under reduced pressure using a rotary evaporator maintained in a ducted fume cupboard. The crude product was then placed on a sinter and washed sequentially with mixtures of Et_2O and CH_2Cl_2 (3–5 × reaction volume, specified for each example). The purity of the product was assessed by ^{1}H-NMR spectroscopy and used in library synthesis without further purification, unless otherwise stated.

2.7.5 General procedure I – Library synthesis – Amidation

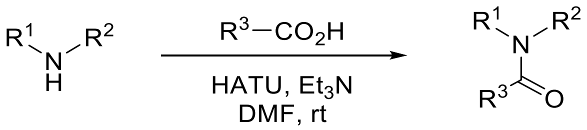

In a 2-dram vial, a solution of HATU (0.24 mmol, 1.2 eq) in DMF (0.5 mL, 0.5 mmol mL^{-1}) was added to a solution of Et_3N (0.40 mmol, 2.0 eq for free amines, 0.60 mmol, 3.0 eq for single HCl salts, 0.80 mmol, 4.0 eq for double HCl salts), the carboxylic acid (0.24 mmol, 1.2 eq) and the 2° amine (0.20 mmol, 1.0 eq) in DMF (2 mL, 0.1 mmol mL^{-1}) at rt (final reaction concentration 0.08 mmol mL^{-1}). The resulting mixture was shaken overnight (16–24 h typical reaction time) at rt. The progress of the reaction was followed by TLC and if necessary, excess carboxylic acid and HATU solution were added to the mixture to ensure complete consumption of the starting amine. Upon completion, the reaction mixture was diluted with a toluene/MeOH mixture (2/1, 1–2 mL) and concentrated using a TurboVap. After 1–2 h in the TurboVap, further toluene/MeOH (1/3, 1–2 mL) was added to the sample and this sequence was repeated until the sample was deemed sufficiently free from volatiles to place it under high vacuum (16–24 h). The resulting mixture was purified by the neutral or basic preparative HPLC method to give the corresponding amide library compound.

2.7.6 General procedure J – Library synthesis – Sulfonylation

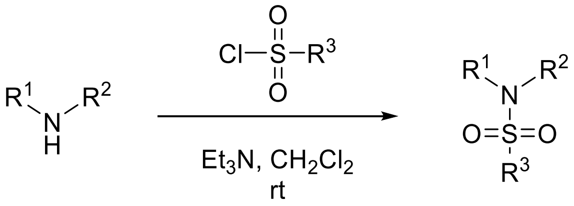

In a 2-dram vial, the sulfonyl chloride (0.40 mmol, 2.0 eq) was added to a solution of Et_3N (0.40 mmol, 2.0 eq for free amines, 0.60 mmol, 3.0 eq for single HCl salts, 0.80 mmol, 4.0 eq for double HCl salts) and the 2° amine (0.20 mmol, 1.0 eq) in CH_2Cl_2 (2 mL, 0.1 mmol mL^{-1}) at rt. The resulting mixture was

shaken overnight (16–24 h typical reaction time) at rt. The progress of the reaction was followed by TLC and if necessary, excess sulfonyl chloride was added to the mixture to ensure full consumption of the starting amine. Upon completion, the reaction mixture was concentrated using a TurboVap and then under high vacuum (16–24 h). The resulting mixture was purified by the neutral or basic preparative HPLC method to give the corresponding sulfonamide library compound.

2.7.7 General procedure K – Library synthesis – Urea formation

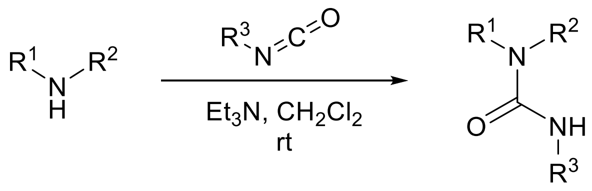

In a 2-dram vial, the isocyanate (0.24 mmol, 1.2 eq) was added to a solution of Et_3N (0.40 mmol, 2.0 eq for free amines, 0.60 mmol, 3.0 eq for single HCl salts, 0.80 mmol, 4.0 eq for double HCl salts) and the 2° amine (0.20 mmol, 1.0 eq) in CH_2Cl_2 (2 mL, 0.1 mmol mL^{-1}) at rt. The resulting mixture was shaken overnight (16–24 h typical reaction time) at rt. The progress of the reaction was followed by TLC and if necessary, excess isocyanate was added to the mixture to ensure full consumption of the starting amine. Upon completion, the reaction mixture was concentrated using a TurboVap and then under high vacuum (16–24 h). The resulting mixture was purified by the neutral or basic preparative HPLC method to give the corresponding urea library compound.

2.7.8 General procedure L – Library synthesis – Reductive amination using a suspension of $NaBH(OAc)_3$

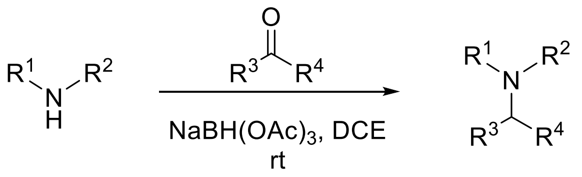

In a 2-dram vial, a suspension of $NaBH(OAc)_3$ (0.40 mmol, 2.0 eq for aldehydes, 0.80 mmol, 4.0 eq for ketones) in 1,2-dichloroethane (2.7 mL for aldehydes, 5.4 mL for ketones, 0.15 mmol mL^{-1}) was added to a solution of the aldehyde (0.40 mmol, 2.0 eq) or ketone (0.80 mmol, 4.0 eq) and the 2° amine (0.20 mmol, 1.0 eq) in 1,2-dichloroethane (1 mL, 0.2 mmol mL^{-1}) at rt (final reaction concentration for

aldehydes: 0.05 mmol mL^{-1}, and for ketones: 0.03 mmol mL^{-1}). The resulting mixture was shaken overnight (16–24 h typical reaction time) at rt. The progress of the reaction was followed by TLC and if necessary, excess NaBH(OAc)$_3$ was added to the mixture to ensure complete consumption of the starting amine. Upon completion, the reaction mixture was extracted with H_2O (2 × 1 mL). The organic phase was dried using a TurboVap and subsequently under high vacuum (16–24 h). The resulting mixture was purified by the neutral or basic preparative HPLC method to give the amine library compound.

2.7.9 General procedure M – Library synthesis – Reductive amination using solid NaBH(OAc)$_3$

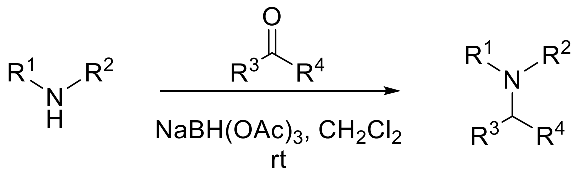

In a 2-dram vial, NaBH(OAc)$_3$ (0.40 mmol, 2.0 eq for aldehydes, 0.80 mmol, 4.0 eq for ketones) was added to a solution of the aldehyde (0.40 mmol, 2.0 eq) or ketone (0.80 mmol, 4.0 eq) and the 2° amine (0.20 mmol, 1.0 eq) in CH_2Cl_2 (2 mL, 0.1 mmol mL^{-1}) at rt. The resulting mixture was shaken overnight (16–24 h typical reaction time) at rt. The progress of the reaction was followed by TLC and if necessary, excess NaBH(OAc)$_3$ was added to ensure complete consumption of the starting amine. Upon completion, the reaction mixture was extracted with H_2O (2 × 1 mL). The organic phase was dried using a TurboVap and subsequently under high vacuum (16–24 h). The resulting mixture was purified by the neutral or basic preparative HPLC method to give the amine library compound.

2.7.10 First decoration step – Urea formation

benzyl 10-((2,4-difluorophenyl)carbamoyl)-1,7-dioxa-4,10-diazaspiro[5.5]undecane-4-carboxylate (10-I03 *or* 11 in the main article):

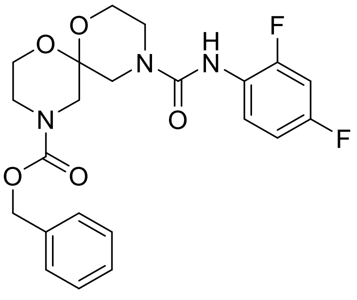

Following general procedure E (p 85), isocyanate **I03** (778 mg, 5.02 mmol) was added to a solution of Et_3N (1.3 mL, 9.1 mmol) and amine HCl salt **10·HCl** (1.50 g, 4.56 mmol) in CH_2Cl_2 (45 mL) at 0 °C. After 1 h, the reaction was worked up according to the general procedure and the crude product was purified by flash column chromatography (gradient, 0–5% MeOH in CH_2Cl_2) to produce urea **10-I03** as a white foam (2.03 g, 98%).

R_f (CH_2Cl_2/MeOH, 20/1) = 0.7.

ν_{max} (KBr/cm^{-1}): 3458 br s, 2927 w, 1699 s, 1650 s, 1518 s, 1287 s, 1270 s.

^{1}H-NMR (500 MHz, C_6D_6, ~1:1 mixture of rotamers, based on the presence of relative half integrals) δ_H 8.18 (stack, 1H), 7.29 – 7.00 (stack, 5H), 6.61 – 6.48 (stack, 2H), 6.48 – 6.34 (m, 1H), 5.26 – 4.95 (stack, 2H), 4.17 – 3.65 (stack, 1.5H), 3.65 – 3.31 (stack, 4.5H, including H-1), 3.15 – 2.93 (stack, 2H), 2.70 – 2.21 (stack, 4H).

^{13}C{^{1}H}-NMR (126 MHz, C_6D_6, mixture of rotamers) δ_C 158.1 (dd, *J* = 243.2, 11.6 Hz, C), [155.5, 155.4, 154.6 (2 × C)], 152.9 (d, *J* = 246.0 Hz, C), [137.4, 137.2 (C)], 124.7 (d, *J* = 9.4 Hz, C), 123.4 (CH), 111.3 (dd, *J* = 17.2, 2.8 Hz, CH), 103.3 (t, *J* = 20.3 Hz, CH), [92.3, 91.7 (C)], 67.5 (CH_2), 59.5 (2 × CH_2, resonance overlap), [49.4, 49.3, 48.7 (2 × CH_2)], 43.4 (CH_2), [43.1, 43.0 (CH_2)], resonances for three aromatic CHs overlapped with the C_6D_6 resonances, however HMBC and HSQC measurements confirmed their presence between δ_C 129.0 – 127.5 ppm.

^{19}F-NMR (376 MHz, C_6D_6, mixture of rotamers) δ_F −117.82 (s), −117.93 (s), −128.82 (s), −128.87 (s).

LRMS (ES+): 521.3 [(M + Et_2NH_2)$^+$, 25%], 448.2 [100, (M + H)$^+$].

HRMS (ES+): calcd for $C_{22}H_{24}F_2N_3O_5$ [M + H]$^+$ 448.1684, found 448.1698.

_N_-(2,4-difluorophenyl)-1,7-dioxa-4,10-diazaspiro[5.5]undecane-4-carboxamide (L1-I03 _or_ 13 in the main article):

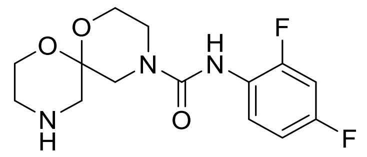

Under an Ar atmosphere, Pd/C (10 wt%, 59.0 mg, 0.056 mmol, 5 mol%) was added to a solution of carbamate **10-I03** (500 mg, 1.12 mmol) in THF/H_2O (1/1, 11 mL) at rt. H_2 was bubbled through the reaction mixture for 2 min from a balloon, venting the reaction vessel to displace any Ar. This balloon was then used to retain a H_2 atmosphere over the reaction mixture. After 3 h, the reaction mixture was purged with Ar gas for 5 min. The reaction mixture was filtered through a pad of Celite (paying attention to never let the Celite go dry), washing with Et_3N in MeOH (10 v/v% mixture, 2 × 10 mL). The filtrate was concentrated under reduced pressure to give amine **L1-I03** as a white foam (340 mg, 96%).

R_f (CH_2Cl_2/MeOH, 20/1) = 0.1.

ν_{max} (neat/cm^{-1}): 3321 w, 3195 br w, 3021 w, 2929 w, 1632 m, 1512 s, 1049 s.

^{1}H-NMR (400 MHz, CD_3OD) δ_H 7.45 – 7.32 (m, 1H), 7.03 – 6.94 (m, 1H), 6.94 – 6.87 (m, 1H), 4.05 – 3.98 (m, 1H), 3.97 – 3.87 (stack, 2H), 3.83 (ddd, _J_ = 11.4, 10.3, 4.6 Hz, 1H), 3.73 – 3.63 (m, 1H), 3.62 – 3.49 (m, 1H), 3.13 (ddd, _J_ = 13.4, 11.9, 3.6 Hz, 1H), 2.95 (d, _J_ = 13.5 Hz, 1H), 2.89 – 2.70 (stack, 3H), 2.67 (d, _J_ = 13.2 Hz, 1H), 2Hs (probably NHs) not observed.

^{13}C{^{1}H}-NMR (101 MHz, CD_3OD) δ_C 161.3 (dd, _J_ = 245.8, 11.3 Hz, C), 158.4 (C), 157.7 (dd, _J_ = 249.5, 12.4 Hz, C), 129.1 (dd, _J_ = 9.6, 2.7 Hz, CH), 124.5 (dd, _J_ = 12.2, 3.9 Hz, C), 111.8 (dd, _J_ = 22.2, 3.9 Hz, CH), 104.8 (t, _J_ = 24.0 Hz, CH), 92.4 (C), 61.2 (CH_2), 60.4 (CH_2), 51.3 (CH_2), 50.7 (CH_2), 45.3 (CH_2), 44.5 (CH_2).

^{19}F-NMR (376 MHz, CD_3OD) δ_F –116.47 (d, _J_ = 5.5 Hz), –120.84 (d, _J_ = 5.5 Hz).

LRMS (ES+): 336.1 [(M + Na)$^+$, 30%], 314.1 [100, (M + H)$^+$].

HRMS (ES+): calcd for $C_{14}H_{18}F_2N_3O_3$ [M + H]$^+$ 314.1316, found 314.1324.

***N*-(2,4-difluorophenyl)-10-(2-(pyridine-3-yl)acetyl)-1,7-dioxa-4,10-diazaspiro[5.5] undecane-4-carboxamide (L1-I03-C39):**

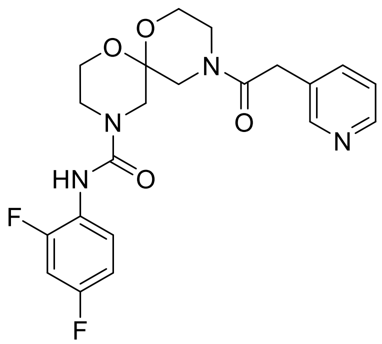

Following general procedure I (p 87), carboxylic acid **C39** (32 mg, 0.23 mmol), Et_3N (53 µL, 0.38 mmol) and a solution of HATU (87 mg, 0.23 mmol) in DMF (0.46 mL) were added sequentially to a solution of amine **L1-I03** (60 mg, 0.19 mmol) in DMF (2 mL) at rt. After 24 h, the reaction was worked up according to the general procedure and the crude product was purified by the neutral preparative HPLC method to produce amide **L1-I03-C39** (56 mg, 68%).

ν_{max} (neat/cm^{-1}): 3253 br w, 2981 w, 1642 s, 1514 s, 1430 s, 1052 s.

^{1}H-NMR (400 MHz, CD_3OD, mixture of rotamers) δ_H 8.52 – 8.42 (stack, 2H), 7.87 – 7.75 (stack, 1H), 7.50 – 7.34 (stack, 2H), 7.06 – 6.97 (stack, 1H), 6.97 – 6.89 (stack, 1H), 4.47 – 4.35 (stack, 1H), 4.10 – 3.62 (stack, 9H), 3.48 – 3.30 (stack, 0.8H), 3.25 – 3.10 (stack, 2H), 3.05 – 2.94 (stack, 0.8H), 2.86 (d, *J* = 13.3 Hz, 0.4H), 1H (probably NH) not observed.

^{13}C{^{1}H}-NMR (101 MHz, CD_3OD, mixture of rotamers) δ_C 172.0, 171.6, 161.4 (dd, *J* = 246.4, 11.0 Hz), 158.24, 158.21, 157.8 (dd, *J* = 248.5, 12.5 Hz), 150.7, 150.5, 148.1, 148.0, 139.9, 139.6, 133.8, 133.5, 129.1 (d, *J* = 10.0 Hz), 125.23, 125.15, 124.4 (d, *J* = 13.2 Hz), 111.8 (d, *J* = 22.7 Hz), 104.8 (t, *J* = 25.6 Hz), 93.9, 93.4, 60.7, 60.6, 52.6, 50.3, 50.0, 47.9, 46.4, 44.5, 44.4, 43.0, 37.8, 37.3.

LRMS (ES+): 455.2 [(M + Na)$^+$, 20%], 433.2 [100, (M + H)$^+$], 412.2 (40).

HRMS (ES+): calcd for $C_{21}H_{23}F_2N_4O_4$ [M + H]$^+$ 433.1687, found 433.1693.

Retention time (neutral LCMS method): 3.83 min.

10-(4,4-difluorocyclohexyl)-*N*-(2,4-difluorophenyl)-1,7-dioxa-4,10-diazaspiro[5.5] undecane-4-carboxamide (L1-I03-K01):

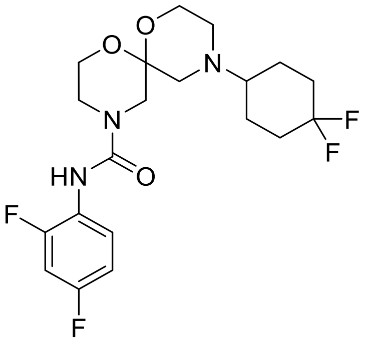

Following general procedure L (p 88), a suspension of $NaBH(OAc)_3$ (163 mg, 0.77 mmol) in 1,2-dichloroethane (5.1 mL) was added to a solution of ketone **K01** (103 mg, 0.77 mmol) and amine **L1-I03** (60 mg, 0.19 mmol) in 1,2-dichloroethane (1 mL) at rt. After 16 h, the reaction was worked up according to the general procedure and the crude product was purified by basic preparative HPLC method to provide 3° amine **L1-I03-K01** (45 mg, 55%).

ν_{max} (KBr/cm^{-1}): 3445 br s, 2942 w, 1651 br s, 1517 s, 1257 m, 1101 m, 1059 m.

^{1}H-NMR (400 MHz, CD_3OD) δ_H 7.48 – 7.30 (m, 1H), 7.05 – 6.97 (m, 1H), 6.97 – 6.89 (m, 1H), 4.10 – 3.83 (stack, 4H), 3.77 – 3.60 (stack, 2H), 3.19 (td, *J* = 13.2, 3.5 Hz, 1H), 3.06 (d, *J* = 13.5 Hz, 1H), 2.94 (d, *J* = 11.3 Hz, 1H), 2.85 (d, *J* = 11.4 Hz, 1H), 2.63 – 2.44 (stack, 2H), 2.37 (d, *J* = 11.2 Hz, 1H), 2.21 – 2.08 (stack, 2H), 2.03 – 1.50 (stack, 6H), 1H (probably NH) not observed.

^{13}C{^{1}H}-NMR (101 MHz, CD_3OD) δ_C 161.3 (dd, *J* = 244.8, 11.3 Hz), 158.2, 157.7 (dd, *J* = 248.7, 12.3 Hz), 129.1 (dd, *J* = 9.5, 2.7 Hz) 126.4 – 121.7 (m), 124.4 (dd, *J* = 12.2, 3.8 Hz), 111.8 (dd, *J* = 22.2, 3.9 Hz), 104.8 (app t, *J* = 25.6 Hz), 94.2, 62.4, 61.4, 60.4, 55.4, 50.7, 49.9, 44.6, 33.2 (dd, *J* = 24.7, 8.1 Hz), 24.9 (dd, *J* = 49.4, 9.2 Hz).

LRMS (ES+): 432.2 [$(M + H)^+$, 100%].

HRMS (ES+): calcd for $C_{20}H_{26}F_4N_3O_3$ $[M + H]^+$ 432.1910, found 432.1911.

Retention time (basic LCMS method): 4.85 min.

***N*-(2,4-difluorophenyl)-10-(imidazo[1,5-a]pyridin-3-ylmethyl)-1,7-dioxa-4,10-diazaspiro [5.5]undecane-4-carboxamide (L1-I03-A11):**

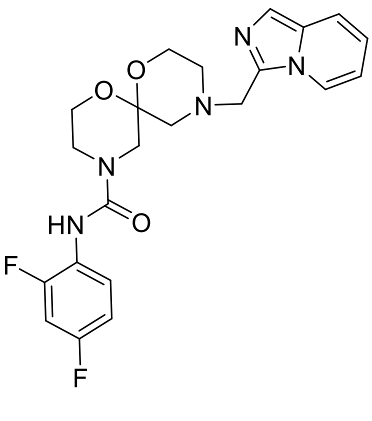

Following general procedure L (p 88), a suspension of $NaBH(OAc)_3$ (81 mg, 0.38 mmol) in 1,2-dichloroethane (2.6 mL) was added to a solution of aldehyde **A11** (56 mg, 0.38 mmol) and amine **L1-I03** (60 mg, 0.19 mmol) in 1,2-dichloroethane (1 mL) at rt. After 16 h, the reaction was worked up according to the general procedure and the crude product was purified by basic preparative HPLC method to provide 3° amine **L1-I03-A11** (32 mg, 38%).

ν_{max} (KBr/cm^{-1}): 3444 br s, 2930 w, 1646 br s, 1515 s, 1257 m, 1058 m.

^{1}H-NMR (400 MHz, CD_3OD) δ_H 8.49 – 8.28 (m, 1H), 7.61 – 7.47 (m, 1H), 7.45 – 7.31 (stack, 2H), 7.05 – 6.96 (m, 1H), 6.96 – 6.89 (m, 1H), 6.89 – 6.81 (m, 1H), 6.75 – 6.68 (m, 1H), 4.07 – 3.82 (stack, 6H), 3.70 – 3.58 (stack, 2H), 3.13 (ddd, *J* = 15.4, 7.3, 3.6 Hz, 1H), 3.00 (d, *J* = 13.5 Hz, 1H), 2.72 (d, *J* = 11.4 Hz, 1H), 2.62 (d, *J* = 11.7 Hz, 1H), 2.38 (td, *J* = 11.1, 3.1 Hz, 1H), 2.25 (d, *J* = 11.4 Hz, 1H), 1H (probably NH) not observed.

^{13}C{^{1}H}-NMR (101 MHz, CD_3OD) δ_C 161.3 (dd, *J* = 244.8, 11.2 Hz), 158.2, 157.8 (dd, *J* = 248.5, 12.4 Hz), 135.6, 133.0, 129.1 (dd, *J* = 9.5, 2.6 Hz), 124.4 (dd, *J* = 12.3, 3.7 Hz), 124.0, 120.7, 119.1, 118.6, 113.9, 111.8 (dd, *J* = 22.2, 3.8 Hz), 104.8 (dd, *J* = 26.9, 24.4 Hz), 94.2, 61.5, 60.4, 59.1, 55.1, 53.0, 50.7, 44.5.

LRMS (ES+): 476.2 [(M + H + MeOH)$^+$ 20%], 444.2 [100, (M + H)$^+$].

HRMS (ES+): calcd for $C_{22}H_{24}F_2N_5O_3$ [M + H]$^+$ 444.1847, found 444.1845.

Retention time (basic LCMS method): 4.68 min.

tert-butyl 10-(benzylcarbamoyl)-1,7-dioxa-4,10-diazaspiro[5.5]undecane-4-carboxylate (9-I04):

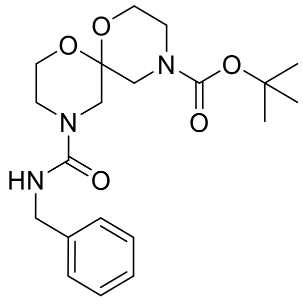

Following general procedure E (p 85), isocyanate **I04** (1.36 g, 10.2 mmol) was added to a solution of Et_3N (2.6 mL, 19 mmol) and 2° amine **9** (2.39 g, 9.25 mmol) in CH_2Cl_2 (93 mL) at 0 °C. After 2 h, the reaction was worked up according to the general procedure and the crude product was purified by flash column chromatography (gradient, 0–10% MeOH in CH_2Cl_2) to produce a colorless oil (3.19 g), which was a 4:1 mixture[t] of urea **9-I04** and by-product urea **S52**. Calculated yield of urea **9-I04** is 75%.
R_f (CH_2Cl_2/MeOH, 9/1) = 0.7.
ν_{max} (thin film/cm^{-1}): 3351 br m, 2977 m, 1697 s, 1629 s, 1541 s, 1276 s, 1058 s.
^{1}H-NMR (400 MHz, C_6D_6, ~1:1 mixture of rotamers based on the presence of relative half integrals) δ_H 7.22 – 6.99 (stack, 5H), 4.72 – 4.57 (stack, 1H), 4.42 – 4.35 (stack, 2H), 4.18 – 3.67 (stack, 1.5H, H-5a, H-7a rotA), 3.64 – 3.32 (stack, 4.5H), 3.16 – 3.03 (stack, 2H), 2.76 – 2.35 (stack, 4H), 1.43 (br s, 9H).
^{13}C{^{1}H}-NMR (101 MHz, C_6D_6, mixture of rotamers) δ_C 157.8 (C), [155.0, 154.6 (C)], 140.7 (C), [128.7, 127.9, 127.3 (CH)], [92.4, 91.8 (C)], 79.6 (C), [59.6, 59.4 (CH_2)], 49.8 (CH_2), 49.3 (CH_2), 48.3 (CH_2), 45.0 (CH_2), 43.6 (CH_2), 43.1 (CH_2), 42.4 (CH_2), 28.4 (CH_3).

LRMS (ES+): 414.2 [(M + Na)$^+$, 40%], 392.2 [80, (M + H)$^+$], 336.2 [100, (M + H – _t_-Bu)$^+$].
HRMS (ES+): calcd for $C_{20}H_{30}N_3O_5$ [M + H]$^+$ 392.2185, found 392.2187.

Selected data for the 1,3-dibenzylurea (**S52**) by-product:

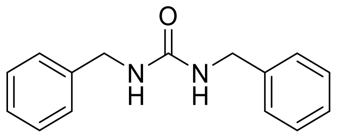

^{1}H-NMR (400 MHz, C_6D_6) δ_H 7.22 – 6.99 (stack, 10H), 5.20 (m, 2H), 4.33 (d, _J_ = 5.9 Hz, 4H).
^{13}C{^{1}H}-NMR (101 MHz, C_6D_6) δ_C 158.4 (C), 140.9 (C), 128.7 (CH), 127.1 (CH), 44.5 (CH_2), CH, Ph was not observed because of resonance overlap with urea **9-I04** and C_6D_6 resonances.

[t] Ratio was assigned based on the relative integration of the resonances at δ_H 4.33 ppm for by-product and at 4.37 ppm for product.

LRMS (ES+): 241.1 [(M + H), 50%].[u]
Data are in accordance with those reported in the literature.[28]

***N*-benzyl-1,7-dioxa-4,10-diazaspiro[5.5]undecane-4-carboxamide hydrochloride (L1-I04·HCl):**

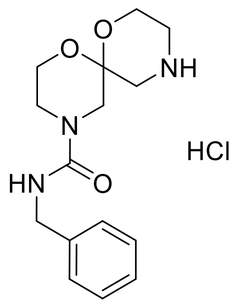

Following general procedure H (p 86), Boc-protected amine **9-I04** (3.17 g, 8.10 mmol) was added to a solution of HCl in 1,4-dioxane (11 mL of a 4 M solution, 44 mmol) at rt. After 2 h, a further volume of HCl in 1,4-dioxane (2 mL of a 4 M solution, 8 mmol) was added. After 2 h, the reaction was worked up according to the general procedure and the crude product was washed with CH_2Cl_2 (3 × 10 mL) to produce amine amine HCl salt **L1-I04·HCl** as an off-white solid (2.39 g, 90%).
R_f (CH_2Cl_2/MeOH, 9/1) = 0.1.
ν_{max} (neat/cm^{-1}): 3345 br w, 2929 w, 2885 w, 1627 m, 1531 s, 1265 s, 1056 s.
^{1}H-NMR (400 MHz, CD_3OD) δ_H 7.36 – 7.28 (stack, 4H), 7.28 – 7.18 (m, 1H), 4.41 (A of AB, $J_{A–B}$ = 15.4 Hz, 1H), 4.35 (B of AB, $J_{B–A}$ = 15.4 Hz, 1H), 4.10 – 3.92 (stack, 3H), 3.92 – 3.80 (stack, 2H), 3.76 (app dd, J = 11.5, 2.7 Hz, 1H), 3.30 – 3.01 (stack, 6H), 3Hs (probably NHs) not observed.
^{13}C{^{1}H}-NMR (101 MHz, CD_3OD) δ_C 160.1 (C), 141.3 (C), 129.3 (CH), 128.1 (CH), 127.9 (CH), 92.5 (C), 60.9 (CH_2), 57.4 (CH_2), 49.4 (CH_2), 48.4 (CH_2), 45.2 (CH_2), 43.9 (CH_2), 43.4 (CH_2).
LRMS (ES+): 314.2 [(M + Na)$^+$, 10%], 292.2 [100, (M + H)$^+$].
HRMS (ES+): calcd for $C_{15}H_{22}N_3O_3$ [M + H]$^+$ 292.1661, found 292.1667.

[u] The base peak was for the product 336.2 [100, (M + H – *t*-Bu)$^+$].

N-benzyl-10-((1,3,5-trimethyl-1H-pyrazol-4-yl)sulfonyl)-1,7-dioxa-4,10-diazaspiro[5.5] undecane-4-carboxamide (L1-I04-B10):

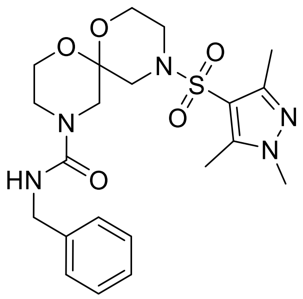

Following general procedure J (p 87), sulfonyl chloride **B10** (0.10 g, 0.49 mmol) was added to a solution of Et_3N (0.10 mL, 0.73 mmol) and amine HCl salt **L1-I04·HCl** (80 mg, 0.24 mmol) in CH_2Cl_2 (2 mL) at rt. After 18 h, the reaction was worked up according to the general procedure and the crude product was purified by the neutral preparative HPLC method to produce sulfonamide **L1-I04-B10** (81 mg, 72%).

ν_{max} (neat/cm^{-1}): 3348 br w, 2932 w, 2885 w, 1628 m, 1529 m, 1266 m, 1151 s, 1057 s.

^{1}H-NMR (400 MHz, CD_3OD) δ_H 7.35 – 7.26 (stack, 4H), 7.25 – 7.18 (m, 1H), 4.40 (A of AB, J_{A-B} = 15.4 Hz, 1H), 4.33 (B of AB, J_{B-A} = 15.4 Hz, 1H), 3.93 – 3.74 (stack, 7H), 3.73 – 3.65 (m, 1H), 3.65 – 3.57 (m, 1H), 3.52 – 3.40 (stack, 2H), 3.11 (td, J = 13.1, 3.6 Hz, 1H), 3.01 (d, J = 13.4 Hz, 1H), 2.69 (td, J = 11.6, 3.3 Hz, 1H), 2.52 (d, J = 11.7 Hz, 1H), 2.47 (s, 3H), 2.35 (s, 3H), 1H (probably NH) not observed.

^{13}C{^{1}H}-NMR (101 MHz, CD_3OD) δ_C 160.2, 149.4, 144.7, 141.4, 129.3, 128.1, 127.8, 112.8, 93.4, 60.7, 60.4, 51.0, 50.0, 45.5, 45.2, 44.2, 36.6, 13.5, 11.0.

LRMS (ES+): 502.2 [(M + K)$^+$, 10%], 486.2 [100, (M + Na)$^+$], 464.2 [60, (M + H)$^+$], 126 (25).

HRMS (ES+): calcd for $C_{21}H_{30}N_5O_5S$ [M + H]$^+$ 464.1968, found 464.1967.

Retention time (neutral LCMS method): 4.22 min.

N-benzyl-10-((2,3-dihydrobenzo[b][1,4]dioxin-6-yl)methyl)-1,7-dioxa-4,10-diazaspiro[5.5]undecane-4-carboxamide (L1-I04-A13):

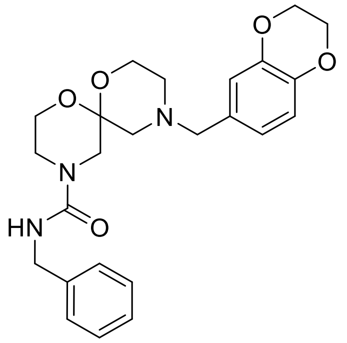

Following general procedure L (p 88), a suspension of $NaBH(OAc)_3$ (104 mg, 0.49 mmol) in 1,2-dichloroethane (3.3 mL) was added to a solution of aldehyde **A13** (80 mg, 0.49 mmol) and amine HCl

salt **L1-I04·HCl** (80 mg, 0.24 mmol) in 1,2-dichloroethane (2 mL) at rt. After 16 h, the reaction was worked up according to the general procedure and the crude product was purified by basic preparative HPLC method to produce 3° amine **L1-I04-A13** (57 mg, 53%).

ν_{max} (KBr/cm^{-1}): 3433 br s, 1631 s, 1542 m, 1508 s, 1286 s, 1264 s, 1057 s.

^{1}H-NMR (400 MHz, CD_3OD) δ_H 7.37 – 7.26 (stack, 4H), 7.25 – 7.17 (m, 1H), 6.87 (s, 1H), 6.78 (s, 2H), 4.40 (A of AB, J_{A-B} = 15.4 Hz, 1H), 4.33 (B of AB, J_{B-A} = 15.4 Hz, 1H), 4.23 (s, 4H), 3.96 – 3.80 (stack, 4H), 3.69 – 3.55 (stack, 2H), 3.49 (d, J = 12.6 Hz, 1H), 3.32 – 3.30 (m, 1H), 3.05 (td, J = 12.6, 3.6 Hz, 1H), 2.88 (d, J = 13.7 Hz, 1H), 2.74 – 2.63 (stack, 2H), 2.26 (td, J = 11.5, 3.4 Hz, 1H), 1.99 (d, J = 11.4 Hz, 1H), 1H (probably NH) not observed.

^{13}C{^{1}H}-NMR (101 MHz, CD_3OD) δ_C 160.3, 144.8, 144.5, 141.5, 130.7, 129.3, 128.1, 127.8, 123.6, 119.4, 117.9, 94.2, 65.6 (resonance overlap), 63.4, 61.3, 60.4, 58.8, 53.4, 50.6, 45.2, 44.3.

LRMS (ES+): 440.2 [(M + H)$^+$, 100%].

HRMS (ES+): calcd for $C_{24}H_{30}N_3O_5$ [M + H]$^+$ 440.2185, found 440.2176.

Retention time (basic LCMS method): 4.95 min.

***N*-benzyl-10-((3-methoxypyridin-2-yl)methyl)-1,7-dioxa-4,10-diazaspiro[5.5]undecane-4-carboxamide (L1-I04-A08):**

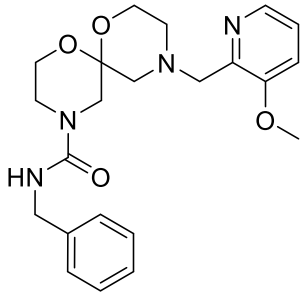

Following general procedure L (p 88), a suspension of $NaBH(OAc)_3$ (104 mg, 0.49 mmol) in 1,2-dichloroethane (3.3 mL) was added to a solution of aldehyde **A08** (67 mg, 0.49 mmol) and amine HCl salt **L1-I04·HCl** (80 mg, 0.24 mmol) in 1,2-dichloroethane (2 mL) at rt. After 16 h, the reaction was worked up according to the general procedure and the crude product was purified by basic preparative HPLC method to produce 3° amine **L1-I04-A08** (41 mg, 41%).

ν_{max} (neat/cm^{-1}): 3341 br w, 2934 w, 2876 w, 2486 w, 1623 m, 1539 m, 1432 m, 1052 s.

^{1}H-NMR (400 MHz, CD_3OD) δ_H 8.15 – 8.07 (m, 1H), 7.51 – 7.41 (m, 1H), 7.38 – 7.14 (stack, 6H), 4.39 (A of AB, J_{A-B} = 15.4 Hz, 1H), 4.32 (B of AB, J_{B-A} = 15.4 Hz, 1H), 3.97 – 3.69 (stack, 9H), 3.64 – 3.51 (stack, 2H), 3.11 – 2.95 (m, 1H), 2.95 – 2.70 (stack, 3H), 2.39 (td, J = 11.3, 3.3 Hz, 1H), 2.21 (d, J = 11.5 Hz, 1H), 1H (probably NH) not observed.

$^{13}C\{^1H\}$-NMR (101 MHz, CD_3OD) δ_C 160.2, 156.5, 146.8, 141.5, 140.8, 129.3, 128.1, 127.8, 125.3, 119.8, 94.1, 61.3, 60.3, 59.1, 58.3, 56.1, 53.1, 50.5, 45.1, 44.2.

LRMS (ES+): 435.2 [$(M + Na)^+$, 10%], 413.2 [100, $(M + H)^+$], 126.0 (25).

HRMS (ES+): calcd for $C_{22}H_{29}N_4O_4$ $[M + H]^+$ 413.2189, found 413.2194.

Retention time (basic LCMS method): 4.48 min.

***tert*-butyl 10-((3,5-dimethylisoxazol-4-yl)carbamoyl)-1,7-dioxa-4,10-diazaspiro[5.5]undecane-4-carboxylate (9-I08):**

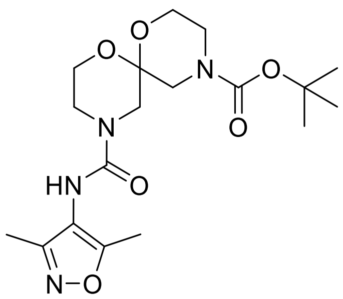

Following general procedure E (p 85), isocyanate **I08** (1.76 g, 12.8 mmol) was added to a solution of Et_3N (3.2 mL, 23 mmol) and 2° amine **9** (3.00 g, 11.6 mmol) in CH_2Cl_2 (120 mL) at 0 °C. After 1 h, more isocyanate (352 mg, 2.55 mmol) and Et_3N (0.64 mL, 4.6 mmol) were added to the reaction mixture. After a further 3 h, the reaction was worked up according to the general procedure and the crude product was purified by flash column chromatography two times (gradient, 0–10% MeOH in CH_2Cl_2 and gradient, 0–100% EtOAc in *n*-heptane) to produce urea **9-I08** as a yellow foam (3.88 g, 84%).

R_f (CH_2Cl_2/MeOH, 9/1) = 0.7.

ν_{max} (KBr/cm^{-1}): 3445 br s, 2977 w, 1697 br s, 1662 br s, 1524 m, 1277 s, 1059 s.

^1H-NMR (400 MHz, C_6D_6, resonance broadening observed because of rotamers) δ_H 5.90 (br s, 1H), 4.06 – 3.78 (stack, 5H), 3.78 – 3.56 (stack, 3H), 3.27 – 2.93 (stack, 3H), 2.88 (d, *J* = 13.3 Hz, 1H), 2.31 (s, 3H), 2.18 (s, 3H), 1.49 (s, 9H).

$^{13}C\{^1H\}$-NMR (101 MHz, C_6D_6, resonance broadening observed because of rotamers) δ_C [163.5, 158.4 (C)], 156.3 (C), 155.1 (C), 114.6 (C), [92.4, 91.7 (C)], 80.5 (C), 59.5 (2 × CH_2, broad, resonance overlap), 50.0 (CH_2), [49.6, 47.9 (CH_2)], 43.8 (CH_2), [43.4, 42.1 (CH_2)], 28.4 (CH_3), 10.8 (CH_3), 9.6 (CH_3).

LRMS (ES+): 419.2 [$(M + Na)^+$, 25%], 397.2 [30, $(M + H)^+$], 341.2 [100, $(M + H – t\text{-Bu})^+$].

HRMS (ES+): calcd for $C_{18}H_{29}N_4O_6$ $[M + H]^+$ 397.2087, found 397.2086.

***N*-(3,5-dimethylisoxazol-4-yl)-1,7-dioxa-4,10-diazaspiro[5.5]undecane-4-carboxamide (L1-I08):**

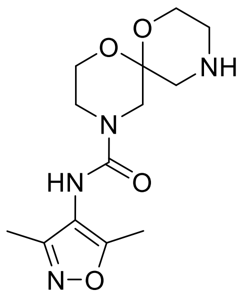

Following general procedure H (p 86), a solution of HCl in 1,4-dioxane (12 mL of a 4 M solution, 60 mmol) was added to a solution of Boc-protected amine **9-I08** (3.88 g, 9.79 mmol) in a mixture of CH_2Cl_2/MeOH (3:1, 40 mL) at rt. After 2 h, an additional volume of HCl in 1,4-dioxane (4 mL of a 4 M solution, 20 mmol) was added. After a further 2 h, the reaction mixture was worked up according to the general procedure and the crude product was washed with CH_2Cl_2 (5 × 10 mL) to produce amine HCl salt **L1-I08·HCl** as a light-orange foam (3.12 g, 96%).

100 mg of the crude product was purified by basic preparative HPLC method for characterization purposes and the rest of the product was used for library synthesis without further purification. The characterization data are reported on the free amine **L1-I08**. After purification by preparative HPLC, the sample containing the product amine, was enriched with ~20% of the urea dimer by-product **S53** (determined by ^{1}H-NMR spectroscopy). The crude amine HCl salt contained only traces of this impurity, thus the reported yield is accurate.

R_f (CH_2Cl_2/MeOH, 9/1) = 0.1.

ν_{max} (neat/cm^{-1}): 3310 w, 3054 w, 2953 w, 1576 w, 1448 m, 1330 s, 1167 s, a strong urea C=O stretch was expected between 1700–1600 cm^{-1}; however, it was not observed.

^{1}H-NMR (400 MHz, CD_3OD) δ_H 4.07 – 3.96 (m, 1H), 3.94 – 3.77 (stack, 3H), 3.66 (dd, *J* = 11.3, 2.9 Hz, 1H), 3.58 – 3.49 (m, 1H), 3.10 (td, *J* = 13.0, 3.5 Hz, 1H), 2.98 (d, *J* = 13.6 Hz, 1H), 2.89 – 2.76 (stack, 3H), 2.73 – 2.63 (m, 1H), 2.27 (s, 3H), 2.12 (s, 3H), 2Hs (probably NHs) not observed.

^{13}C{^{1}H}-NMR (101 MHz, CD_3OD) δ_C 165.4 (C), 160.3 (C), 158.9 (C), 116.4 (C), 92.4 (C), 61.0 (CH_2), 60.3 (CH_2), 51.2 (CH_2), 50.9 (CH_2), 45.2 (CH_2), 44.5 (CH_2), 10.5 (CH_3), 9.47 (CH_3).

LRMS (ES+): 319.1 [$(M + Na)^+$, 10%], 297.2 [100, $(M + H)^+$], 200.1 (15).

HRMS (ES+): calcd for $C_{13}H_{21}N_4O_4$ $[M + H]^+$ 297.1563, found 297.1570.

Selected data for the 1,3-bis(3,5-dimethylisoxazol-4-yl)urea (**S53**) urea by-product**:**

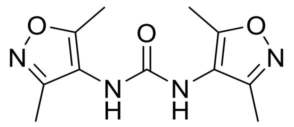

^{1}H-NMR (400 MHz, CD_3OD) δ_H 2.32 (s, 6H), 2.18 (s, 6H), NHs not observed.

^{13}C{^{1}H}-NMR (101 MHz, CD_3OD) δ_C 159.9 (C), 115.5 (C), [10.7, 9.54 (CH_3), 2C resonances (probably those for the urea and one of the quaternary aromatic carbons) not observed.

***N*^4,*N*^10-bis(3,5-dimethylisoxazol-4-yl)-1,7-dioxa-4,10-diazaspiro[5.5]undecane-4,10-dicarboxamide (L1-I08-I08):**

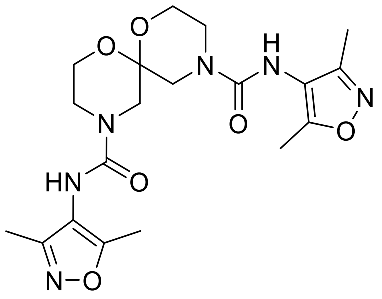

Following general procedure K (p 88), isocyanate **I08** (40 mg, 0.29 mmol) was added to a solution of Et_3N (67 µL, 0.48 mmol) and amine HCl salt **L1-I08·HCl** (80 mg, 0.24 mmol) in CH_2Cl_2 (2 mL) at rt. After 16 h, the reaction was worked up according to the general procedure and the crude product was purified by the neutral preparative HPLC method to produce urea **L1-I08-I08** (50 mg, 48%).

ν_{max} (neat/cm^{-1}): 3284 w, 2928 w, 2423 w, 1629 s, 1461 s, 1428 s, 1271 s, 1055 s, 1020 s.

^{1}H-NMR (400 MHz, CD_3OD) δ_H 4.08 – 3.97 (stack, 4H), 3.92 (td, *J* = 11.6, 2.5 Hz, 2H), 3.69 (dd, *J* = 11.4, 2.5 Hz, 2H), 3.19 (td, *J* = 13.4, 3.4 Hz, 2H), 3.11 (d, *J* = 13.6 Hz, 2H), 2.31 (s, 6H), 2.17 (s, 6H), 2Hs (probably NHs) not observed.

^{13}C{^{1}H}-NMR (101 MHz, CD_3OD) δ_C 165.4, 160.2, 158.8, 116.3, 93.5, 60.5, 50.5, 44.5, 10.5, 9.5.

LRMS (ES+): 457.2 [$(M + Na)^+$, 60%], 435.2 [100, $(M + H)^+$], 126.0 (35).

HRMS (ES+): calcd for $C_{19}H_{27}N_6O_6$ $[M + H]^+$ 435.1992, found 435.2002.

Retention time (neutral LCMS method): 2.93 min.

10-(3-chloro-4-(trifluoromethoxy)benzyl)-*N*-(3,5-dimethylisoxazol-4-yl)-1,7-dioxa-4,10-diazaspiro[5.5]undecane-4-carboxamide (L1-I08-A09):

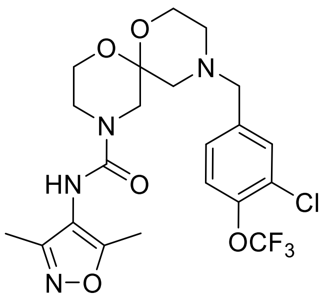

Following general procedure L (p 88), a suspension of $NaBH(OAc)_3$ (102 mg, 0.48 mmol) in 1,2-dichloroethane (3.2 mL) was added to a solution of aldehyde **A09** (108 mg, 0.48 mmol) and amine HCl salt **L1-I08·HCl** (80 mg, 0.24 mmol) in 1,2-dichloroethane (2 mL) at rt. After 16 h, the reaction was worked up according to the general procedure and the crude product was purified by basic preparative HPLC method to produce 3° amine **L1-I08-A09** (62 mg, 55%).

ν_{max} (KBr/cm^{-1}): 3445 br m, 1637 br m, 1264 s, 1220 m, 1060 m.

^{1}H-NMR (400 MHz, CD_3OD) δ_H 7.70 – 7.62 (m, 1H), 7.48 – 7.36 (stack, 2H), 4.06 – 3.90 (stack, 4H), 3.73 – 3.64 (stack, 2H), 3.62 (A of AB, $J_{A–B}$ = 13.5 Hz, 1H), 3.51 (B of AB, $J_{B–A}$ = 13.5 Hz, 1H), 3.21 – 3.10 (m, 1H), 3.04 (d, J = 13.6 Hz, 1H), 2.74 (t, J = 10.8 Hz, 2H), 2.38 – 2.26 (stack, 4H), 2.18 – 2.08 (stack, 4H), 1H (probably NH) not observed.

^{13}C{^{1}H}-NMR (101 MHz, CD_3OD) δ_C 165.5, 160.3, 158.8, 145.4, 139.8, 132.7, 130.3, 128.2, 123.8, 122.0 (q, J = 258.3 Hz), 116.3, 94.3, 62.3, 61.5, 60.4, 59.0, 53.3, 51.0, 44.6, 10.5, 9.4.

LRMS (ES+): 505.2 [(M + H)$^+$, 100%].

HRMS (ES+): calcd for $C_{21}H_{25}{}^{35}ClF_3N_4O_5$ [M + H]$^+$ 505.1466, found 505.1467.

Retention time (basic LCMS method): 5.31 min.

tert-butyl 4-((2,4-difluorophenyl)carbamoyl)-1,7-dioxa-4,11-diazaspiro[5.6]dodecane-11-carboxylate (25-I03 _or_ 28 in the main article):

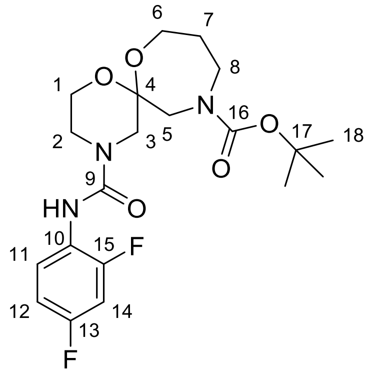

Following general procedure E (p 85), isocyanate **I03** (1.25 g, 8.08 mmol) was added to a solution of Et_3N (2.0 mL, 15 mmol) and amine **25** (2.00 g, 7.34 mmol) in CH_2Cl_2 (73 mL) at 0 °C. After 2 h, the reaction was worked up according to the general procedure and the crude product was purified by flash column chromatography (gradient, 0–75% EtOAc in *n*-heptane) to produce urea **25-I03** as a white foam (2.78 g, 89%).

R_f (*n*-heptane/EtOAc, 3/2) = 0.2.

ν_{max} (KBr/cm^{-1}): 3445 br m, 1682 br s, 1613 w, 1531 br s.

^{1}H-NMR (400 MHz, $CDCl_3$, ~1:2 mixture of rotamers based on the relative integration of the resonances for H-18 min at δ_H 1.42 ppm and H-18 maj at 1.40 ppm) δ_H 7.91 – 7.64 (stack, 1H, Ar), 7.53 – 7.38 (m, 0.66H, NH maj), 6.92 – 6.84 (m, 0.34H, NH min), 6.83 – 6.69 (stack, 2H, Ar), 4.34 – 3.99 (stack, 2H, H-2a min, H-5a maj, H-8a), 3.96 – 3.68 (stack, 4.4H, H-1a, H-2a maj, H-3a min, H-5a min, H-6), 3.65 – 3.50 (stack, 1.6H, H-1b, H-3a maj), 3.45 (d, *J* = 14.5 Hz, 0.7H, H-3b maj), 3.27 (ddd, *J* = 13.5, 10.9, 4.1 Hz, 0.7H, H-2b maj), 3.11 – 2.88 (stack, 1H, H-2b min, H-3b min, H-5b min), 2.83 – 2.57 (stack, 1.6H, H-5b maj, H-8b), 2.03 – 1.79 (stack, 1H, H-7a), 1.62 – 1.47 (stack, 1H, H-7b), 1.42 (s, 3H, H-18 min), 1.40 (s, 6H, H-18 maj).

^{13}C{^{1}H}-NMR (101 MHz, $CDCl_3$, mixture of rotamers) δ_C 157.8 (dd, *J* = 242.4, 11.6 Hz, C, C-13 or C-15), [155.8, 155.7, 155.6 (C, C-9, C-16)], 153.0 (dd, *J* = 246.4, 11.5 Hz, C, C-13 or C-15), 124.2 (dd, *J* = 10.5, 3.7 Hz, C, C-10), 123.0 (dd, *J* = 9.1, 2.5 Hz, CH, C-11, rotamer), 122.3 (app d, *J* = 6.9 Hz, CH, C-11, rotamer), 110.8 (dd, *J* = 21.4, 3.3 Hz, CH, C-12), 103.3 (app t, *J* = 25.0 Hz, CH, C-14), [99.1, 98.0 (C, C-4)], [80.6, 80.5 (C, C-17)], [62.0, 61.9 (CH_2, C-6)], [60.5, 60.3 (CH_2, C-1)], 54.3 (CH_2, C-5 min), 53.4 (CH_2, C-3 min), 53.2 (CH_2, C-5 maj), 52.8 (CH_2, C-3 maj), [49.8, 48.9 (CH_2, C-8)], [44.1, 43.7 (CH_2, C-2)], [30.6, 30.3 (CH_2, C-7)], [28.5, 28.3 (CH_3, C-18)].

^{19}F-NMR (376 MHz, $CDCl_3$) δ_F −117.76 to −117.93 (m), −118.04 to −118.18 (m), −126.28 to −126.43 (m), −128.27 to −128.43 (m).

LRMS (ES+): 877.4 [$(2M + Na)^+$, 30%], 450.2 [100, $(M + Na)^+$], 428.2 [40, $(M + H)^+$], 372.2 [30, (M + H – *t*-Bu$)^+$].

HRMS (ES+): calcd for $C_{20}H_{27}F_2N_3O_5Na$ $[M + Na]^+$ 450.1816, found 450.1825.

_N_-(2,4-difluorophenyl)-1,7-dioxa-4,11-diazaspiro[5.6]dodecane-4-carboxamide hydrochloride (L2-I03·HCl _or_ 31·HCl in the main article):

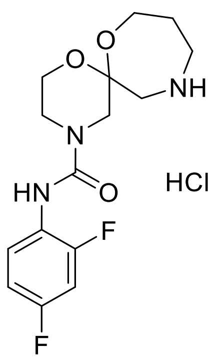

Following general procedure H (p 86), a solution of HCl in 1,4-dioxane (7.6 mL of a 4 M solution, 30 mmol) was added to a solution of Boc-protected amine **25-I03** (2.60 g, 6.08 mmol) in CH_2Cl_2 (10 mL) at rt. After 3 h, the reaction was worked up according to the general procedure and the crude product was washed with MeOH (5 × 20 mL) to produce amine HCl salt **L2-I03·HCl** as a white solid (1.44 g, 65%).

R_f (CH_2Cl_2/MeOH, 9/1) = 0.1.

ν_{max} (KBr/cm^{-1}): 3443 br m, 2941 w, 2738 w, 1651 br s, 1516 s, 1068 s.

^{1}H-NMR (400 MHz, D_2O) δ_H 7.27 – 7.14 (m, 1H), 7.03 – 6.92 (m, 1H), 6.92 – 6.84 (m, 1H), 4.06 – 3.63 (stack, 6H), 3.52 (d, *J* = 14.8 Hz, 1H), 3.44 – 3.06 (stack, 5H), 2.14 – 1.77 (stack, 2H), 3Hs (probably NHs) not observed.

^{13}C{^{1}H}-NMR (101 MHz, D_2O) δ_C 161.7 (dd, *J* = 246.1, 11.7 Hz, C), 158.1 (C), 156.8 (dd, *J* = 250.4, 13.0 Hz, C), 128.6 (dd, *J* = 10.0, 2.3 Hz, CH), 121.4 (dd, *J* = 12.7, 3.8 Hz, C), 111.3 (dd, *J* = 22.4, 3.8 Hz, CH), 104.3 (dd, *J* = 26.8, 24.3 Hz, CH), 95.4 (C), 61.6 (CH_2), 59.8 (CH_2), 51.8 (CH_2), 49.3 (CH_2), 48.0 (CH_2), 43.2 (CH_2), 26.3 (CH_2).

^{19}F-NMR (376 MHz, D_2O) δ_F −112.98 to −113.15 (m), −119.36 to −119.51 (m).

LRMS (ES+): 328.2 [$(M + H)^+$, 100%], 126.0 (20).

HRMS (ES+): calcd for $C_{15}H_{20}F_2N_3O_3$ $[M + H]^+$ 328.1473, found 328.1477.

***N*-(2,4-difluorophenyl)-11-((3-phenylisoxazol-5-yl)methyl)-1,7-dioxa-4,11-diazaspiro [5.6]dodecane-4-carboxamide (L2-I03-A05):**

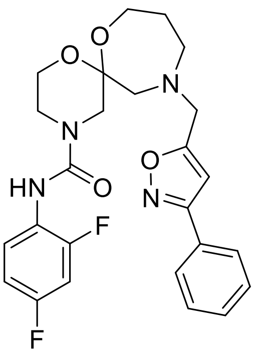

Following general procedure M (p 89), $NaBH(OAc)_3$ (70 mg, 0.33 mmol) was added to a solution of aldehyde **A05** (46 mg, 0.33 mmol) and amine HCl salt **L2-I03·HCl** (60 mg, 0.17 mmol) in CH_2Cl_2 (2 mL) at rt. After 16 h, the reaction was worked up according to the general procedure and the crude product was purified by basic preparative HPLC method to provide 3° amine **L2-I03-A05** (59 mg, 73%).

ν_{max} (KBr/cm^{-1}): 3435 br m, 2948 w, 1668 br m, 1610 w, 1519 br s, 1058 m.

^{1}H-NMR (400 MHz, CD_3OD) δ_H 7.87 – 7.77 (stack, 2H), 7.55 – 7.26 (stack, 4H), 7.02 – 6.93 (m, 1H), 6.93 – 6.85 (m, 1H), 6.83 – 6.78 (m, 1H), 4.04 – 3.79 (stack, 6H), 3.73 – 3.63 (m, 1H), 3.61 – 3.52 (m, 1H), 3.23 – 3.09 (m, 2H), 3.09 – 2.89 (m, 2H), 2.76 – 2.66 (m, 1H), 2.58 – 2.42 (m, 1H), 2.11 – 1.92 (m, 1H), 1.71 – 1.54 (m, 1H, H-7b), 1H (probably NH) not observed.

^{13}C{^{1}H}-NMR (101 MHz, CD_3OD) δ_C 172.0, 163.8, 161.0 (dd, J = 244.7, 11.3 Hz), 158.2, 157.4 (dd, J = 248.7, 12.4 Hz), 131.3, 130.2, 130.1, 128.5 (app d, J = 9.8 Hz), 127.8, 124.6 (dd, J = 12.2, 3.7 Hz), 111.8 (dd, J = 22.2, 3.7 Hz), 104.8 (dd, J = 26.8, 24.4 Hz), 102.8, 99.2, 63.0, 62.5, 60.4, 58.7, 55.1, 51.9, 45.0, 32.1.

LRMS (ES+): 507.2 [(M + Na)$^+$, 25%], 485.2 [100, (M + H)$^+$].

HRMS (ES+): calcd for $C_{25}H_{27}F_2N_4O_4$ [M + H]$^+$ 485.2000, found 485.2002.

Retention time (basic LCMS method): 3.14 min.

N-(2,4-difluorophenyl)-11-((1,3,5-trimethyl-1H-pyrazol-4-yl)sulfonyl)-1,7-dioxa-4,11-diazaspiro[5.6]dodecane-4-carboxamide (L2-I03-B10):

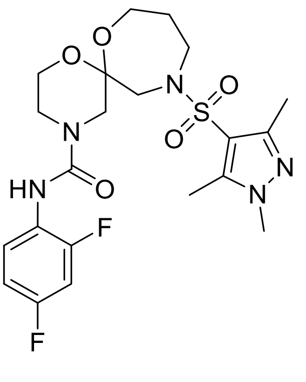

Following general procedure J (p 87), sulfonyl chloride **B10** (69 mg, 0.33 mmol) was added to a solution of Et_3N (92 µL, 0.66 mmol) and amine HCl salt **L2-I03·HCl** (60 mg, 0.17 mmol) in CH_2Cl_2 (2 mL) at rt. After 18 h, the reaction was worked up according to the general procedure and the crude product was purified by the neutral preparative HPLC method to produce sulfonamide **L2-I03-B10** (63 mg, 76%).

ν_{max} (KBr/cm^{-1}): 3435 br s, 1660 br m, 1528 s, 1148 m.

^{1}H-NMR (400 MHz, CD_3OD) δ_H 7.53 – 7.39 (m, 1H), 7.06 – 6.97 (m, 1H), 6.97 – 6.89 (m, 1H), 4.12 (d, J = 13.7 Hz, 1H), 4.04 – 3.84 (stack, 3H), 3.83 – 3.71 (stack, 6H), 3.70 – 3.59 (m, 1H), 3.25 – 3.13 (stack, 2H), 3.03 (d, J = 15.4 Hz, 1H), 2.78 (td, J = 12.6, 3.4 Hz, 1H), 2.50 (s, 3H), 2.36 (s, 3H), 2.14 – 1.93 (m, 1H), 1.79 – 1.61 (m, 1H), 1H (probably NH) not observed.

^{13}C{^{1}H}-NMR (101 MHz, CD_3OD) δ_C 161.0 (dd, J = 244.2, 11.5 Hz), 158.2, 157.3 (dd, J = 247.8, 12.6 Hz), 148.6, 144.2, 128.4 (app d, J = 9.6 Hz), 124.6 (dd, J = 12.1, 3.7 Hz), 115.5, 111.8 (dd, J = 22.2, 3.8 Hz), 104.8 (dd, J = 26.8, 24.4 Hz), 98.7, 62.1, 60.9, 55.9, 52.2, 51.2, 44.7, 36.6, 32.2, 13.5, 11.0.

LRMS (ES+): 522.2 [(M + Na)$^+$, 100%], 500.2 [50, (M + H)$^+$].

HRMS (ES+): calcd for $C_{21}H_{27}F_2N_5O_5SNa$ [M + Na]$^+$ 522.1599, found 522.1613.

Retention time (neutral LCMS method): 2.33 min.

***N*-(2,4-difluorophenyl)-11-(2-methylthiazole-4-carbonyl)-1,7-dioxa-4,11-diazaspiro[5.6]dodecane-4-carboxamide (L2-I03-C42):**

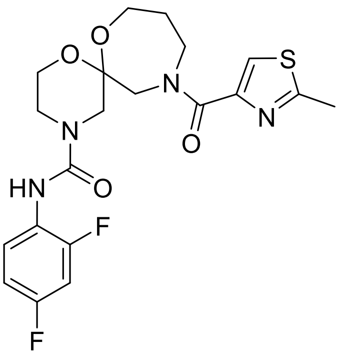

Following general procedure I (p 87), carboxylic acid **C42** (29 mg, 0.20 mmol), Et_3N (69 µL, 0.50 mmol) and a solution of HATU (76 mg, 0.20 mmol) in DMF (0.40 mL) were added sequentially to a solution of amine HCl salt **L2-I03·HCl** (60 mg, 0.17 mmol) in DMF (2 mL) at rt. After 24 h, the reaction was worked up according to the general procedure and the crude product was purified by the neutral preparative HPLC method to provide amide **L2-I03-C42** (56 mg, 75%).

ν_{max} (KBr/cm^{-1}): 3444 br s, 1670 br s, 1623 br s, 1517 br s, 1431 m, 1061 m.

^{1}H-NMR (400 MHz, CD_3OD, ~4:1 mixture of rotamers based on the relative integration of the resonances for δ_H 7.93 ppm and 7.86 ppm) δ_H 7.93 (s, 0.2H), 7.86 (s, 0.8H), 7.49 – 7.34 (m, 1H), 7.03 – 6.96 (m, 1H), 6.95 – 6.85 (m, 1H), 4.66 – 4.35 (stack, 2H), 4.22 – 3.54 (stack, 7H), 3.51 – 3.35 (m, 0.2H), 3.51 – 3.14 (stack, 2.4H), 3.10 – 2.98 (m, 0.2H), 2.80 – 2.65 (stack, 3.2H), 2.22 – 2.04 (m, 1H), 1.97 – 1.83 (m, 0.2H), 1.75 – 1.62 (m, 0.8H), 1H (probably NH) not observed.

^{13}C{^{1}H}-NMR (101 MHz, CD_3OD, mixture of rotamers) δ_C 168.0, 166.3, 161.2 (dd, *J* = 244.7, 11.3 Hz), 158.3, 158.0, 157.6 (dd, *J* = 248.6, 12.3 Hz), 150.3, 150.2, 128.8, 128.7, 125.6, 124.6, 124.5, 124.3, 111.8 (dd, *J* = 22.2, 3.9 Hz), 104.8 (dd, *J* = 26.8, 24.4 Hz), 99.4, 98.4, 62.3, 62.0, 60.9, 60.8, 57.1, 54.4, 53.5, 53.0, 51.2, 44.5, 32.0, 29.7, 18.8.

LRMS (ES+): 475.1 [$(M + Na)^+$, 100%], 453.1 [75, $(M + H)^+$].

HRMS (ES+): calcd for $C_{20}H_{22}F_2N_4O_4SNa$ $[M + Na]^+$ 475.1228, found 475.1234.

Retention time (neutral LCMS method): 2.30 min.

2.7.11 First decoration step – Sulfonylations

benzyl 10-(pyridin-3-ylsulfonyl)-1,7-dioxa-4,10-diazaspiro[5.5]undecane-4-carboxylate (10-B08 *or* 12 in the main article):

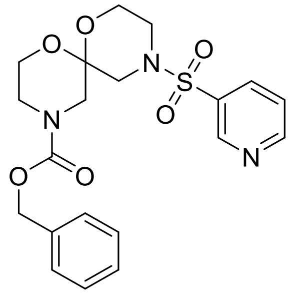

Following general procedure F (p 85), sulfonyl chloride **B08** (81 mg, 0.46 mmol) was added to a solution of Et_3N (130 µL, 0.91 mmol) and amine HCl salt **10·HCl** (100 mg, 0.30 mmol) in CH_2Cl_2 (3.0 mL) at 0 °C. After 16 h, the reaction was worked up according to the general procedure and the crude product was purified by flash column chromatography (gradient, 0–100% EtOAc in *n*-heptane) to produce sulfonamide **10-B08** as a white oil (118 mg, 91%).

R_f (*n*-heptane/EtOAc, 3/2) = 0.3.

ν_{max} (KBr/cm^{-1}): 2931 w, 1702 s, 1355 s, 1060 s.

^{1}H-NMR (400 MHz, C_6D_6, resonance broadening observed because of rotamers) δ_H 8.98 (s, 1H), 8.27 (s, 1H), 7.48 (br s, 1H), 7.16 – 6.85 (stack, 5H), 6.45 (br s, 1H), 5.11 – 4.76 (stack, 2H), 3.96 – 3.73 (m, 1H), 3.59 – 3.06 (stack, 4H), 3.06 – 2.71 (stack, 3H), 2.55 – 2.38 (m, 1H), 2.30 – 2.03 (m, 1H), 2.03 – 1.68 (stack, 2H).

^{13}C{^{1}H}-NMR (101 MHz, C_6D_6, mixture of rotamers) δ_C 156.0 (C), 153.9 (CH), 149.5 (CH), 137.8 (C), 135.6 (CH), 134.9 (C), 129.3 (CH), 124.0 (CH), [92.5, 91.9 (C)], 68.0 (CH_2), 59.9 (CH_2), [59.4, 59.3 (CH_2)], 50.7 (CH_2), [49.6, 49.2 (CH_2)], 44.9 (CH_2), [43.6, 43.4 (CH_2)], resonances of two aromatic CHs overlapped with the C_6D_6 resonances, however HMBC and HSQC measurements confirmed their presence between δ_C 129.0 – 128.0 ppm.

LRMS (ES+): 456.1 [(M + Na)$^+$, 15%], 434.1 [100, (M + H)$^+$].

HRMS (ES+): calcd for $C_{20}H_{24}N_3O_6S$ [M + H]$^+$ 434.1386, found 434.1391.

***tert*-butyl 10-(pyridin-3-ylsulfonyl)-1,7-dioxa-4,10-diazaspiro[5.5]undecane-4-carboxylate (9-B08 *or* 14 in the main article):**

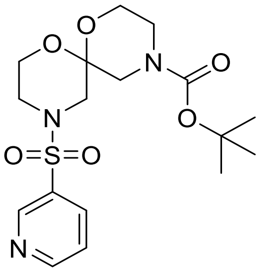

Following general procedure F (p 85), sulfonyl chloride **B08** (618 mg, 3.48 mmol) was added to a solution of Et_3N (0.97 mL, 7.0 mmol) and amine **9** (600 mg, 2.21 mmol) in CH_2Cl_2 (22 mL) at 0 °C. After 18 h, the reaction was worked up according to the general procedure and the crude product was purified by flash column chromatography (gradient, 0–10% MeOH in CH_2Cl_2) to produce sulfonamide **9-B08** as a light-yellow solid (770 mg, 87%).

R_f (CH_2Cl_2/MeOH, 9/1) = 0.9.

ν_{max} (KBr/cm^{-1}): 2976 m, 2930 m, 1696 br s, 1418 m, 1174 s.

^{1}H-NMR (400 MHz, C_6D_6, mixture of rotamers) δ_H 9.10 (s, 1H), 8.51 – 8.26 (stack, 1H), 7.59 (app br s, 1H), 6.65 – 6.42 (stack, 1H), 4.09 – 3.76 (stack, 1H), 3.76 – 3.18 (stack, 4H), 3.18 – 2.83 (stack, 3H), 2.76 – 2.45 (stack, 1H), 2.45 – 2.27 (stack, 1H), 2.23 – 1.88 (stack, 2H), 1.39 (s, 9H).

^{13}C{^{1}H}-NMR (101 MHz, C_6D_6, mixture of rotamers) δ_C 154.4 (C), 152.9 (CH), 148.5 (CH), 134.6 (CH), 133.8 (C), 123.0 (CH), [91.6, 91.1 (C)], 79.3 (C), 59.0 (CH_2), 58.4 (CH_2), 49.9 (CH_2), [49.0, 47.8 (CH_2)], 44.0 (CH_2), [43.1, 41.9 (CH_2)], 28.0 (CH_3).

LRMS (ES+): 400.2 [$(M + H)^+$, 20%], 344.1 [100, $(M + H – t\text{-Bu})^+$].

HRMS (ES+): calcd for $C_{17}H_{26}N_3O_6S$ $[M + H]^+$ 400.1542, found 400.1540.

4-(pyridin-3-ylsulfonyl)-1,7-dioxa-4,10-diazaspiro[5.5]undecane dihydrochloride (L1-B08·2HCl *or* 16 in the main article):

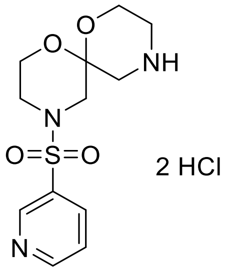

Following general procedure H (p 86), Boc-protected amine **9-B08** (720 mg, 1.80 mmol) was added to a solution of HCl in 1,4-dioxane (2.3 mL of a 4 M solution, 9.2 mmol) at rt. After 1 h, the reaction was worked up according to the general procedure and the crude product was washed with CH_2Cl_2 (2 × 10 mL) to produce amine HCl salt **L1-B08·2HCl** as an off-white solid (635 mg, 95%). The yield was calculated for the 2HCl salt. The 2HCl form is an assumption based on the resonance shift of the pyridyl ring compared with sulfonamide precursor **9-B08**.

ν_{max} (neat/cm^{-1}): 3311 br w, 3054 w, 2953 w, 1576 w, 1330 s, 1167 s.

^{1}H-NMR (400 MHz, CD_3OD) δ_H 9.27 (s, 1H), 9.07 (d, *J* = 5.3 Hz, 1H), 8.80 (d, *J* = 8.0 Hz, 1H), 8.15 (dd, *J* = 8.0, 5.3 Hz, 1H), 3.93 – 3.82 (stack, 3H), 3.82 – 3.63 (stack, 3H), 3.28 – 3.15 (stack, 4H), 3.12 – 3.04 (m, 1H), 2.99 (d, *J* = 13.0 Hz, 1H), 3Hs (probably NHs) not observed.

^{13}C{^{1}H}-NMR (101 MHz, CD_3OD) δ_C 148.7 (CH), 144.4 (CH), 144.0 (CH), 139.3 (C), 128.6 (CH), 92.3 (C), 60.2 (CH_2), 57.6 (CH_2), 50.6 (CH_2), 47.9 (CH_2), 45.3 (CH_2), 43.2 (CH_2).

LRMS (ES+): 300.1 [$(M + H)^+$, 100%].

HRMS (ES+): calcd for $C_{12}H_{18}N_3O_4S$ $[M + H]^+$ 300.1018, found 300.1025.

2-(pyridin-3-yl)-1-(10-(pyridin-3-ylsulfonyl)-1,7-dioxa-4,10-diazaspiro[5.5]undecan-4-yl)ethan-1-one (L1-B08-C39):

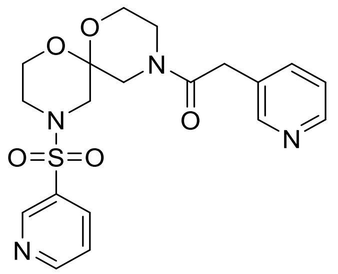

Following general procedure I (p 87), carboxylic acid **C39** (35 mg, 0.26 mmol), Et_3N (0.12 mL, 0.86 mmol) and a solution of HATU (99 mg, 0.26 mmol) in DMF (0.52 mL) were added sequentially to a solution of amine HCl salt **L1-B08·2HCl** (80 mg, 0.21 mmol) in DMF (2 mL) at rt. After 24 h, the reaction

was worked up according to the general procedure and the crude product was purified by the neutral preparative HPLC method to produce amide **L1-B08-C39** (58 mg, 64%).

ν_{max} (neat/cm^{-1}): 2922 br w, 1675 m, 1577 w, 1330 m, 1168 s, 1049 s.

^{1}H-NMR (400 MHz, CD_3OD, mixture of rotamers) δ_H 9.01 – 8.93 (stack, 1H), 8.87 – 8.80 (stack, 1H), 8.51 – 8.37 (stack, 2H), 8.32 – 8.18 (stack, 1H), 7.85 – 7.73 (stack, 1H), 7.73 – 7.63 (stack, 1H), 7.51 – 7.39 (stack, 1H), 4.44 – 4.25 (stack, 1H), 4.02 – 3.48 (stack, 9H), 3.44 – 3.27 (stack, 0.8H), 3.01 – 2.89 (stack, 0.6H), 2.89 – 2.76 (stack, 1.6H), 2.75 – 2.64 (stack, 1H).

^{13}C{^{1}H}-NMR (101 MHz, CD_3OD, mixture of rotamers) δ_C 171.9, 171.5, 154.3, 151.8, 150.1, 149.2, 149.17, 149.13, 147.5, 140.3, 137.4, 137.3, 135.7, 133.7, 129.8, 125.6, 125.3, 122.0, 93.6, 93.0, 60.6, 60.1, 52.3, 51.3, 51.1, 47.7, 46.2, 45.7, 45.6, 42.8, 37.7, 37.1.

LRMS (ES+): 441.1 [(M + Na)$^+$, 20%], 419.1 [100, (M + H)$^+$].

HRMS (ES+): calcd for $C_{19}H_{23}N_4O_5S$ [M + H]$^+$ 419.1389, found 419.1388.

Retention time (neutral LCMS method): 3.32 min.

4-(methylsulfonyl)-10-(pyridin-3-ylsulfonyl)-1,7-dioxa-4,10-diazaspiro[5.5]undecane (L1-B08-B09)

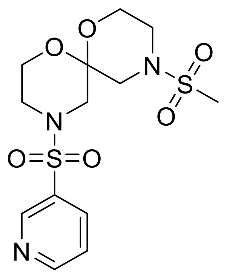

Following general procedure J (p 87), sulfonyl chloride **B09** (49 mg, 0.43 mmol) was added to a solution of Et_3N (0.12 mL, 0.86 mmol) and amine HCl salt **L1-B08·2HCl** (80 mg, 0.21 mmol) in CH_2Cl_2 (2 mL) at rt. After 18 h, the reaction was worked up according to the general procedure and the crude product was purified by the neutral preparative HPLC method to produce sulfonamide **L1-B08-B09** (36 mg, 44%). The compound contained a major impurity (~40%) after the preparative HPLC purification, thus no NMR data are reported.

Selected data:

ν_{max} (KBr/cm^{-1}): 1636 br w, 1575 w, 1333 s, 1171 s, 984 s.

LRMS (ES+): 378.1 [(M + H)$^+$, 100%].

HRMS (ES+): calcd for $C_{13}H_{20}N_3O_6S_2$ [M + H]$^+$ 378.0794, found 378.0801.

Retention time (neutral LCMS method): 2.88 min.

tert-butyl 10-(cyclohexylsulfonyl)-1,7-dioxa-4,10-diazaspiro[5.5]undecane-4-carboxylate (9-B05):

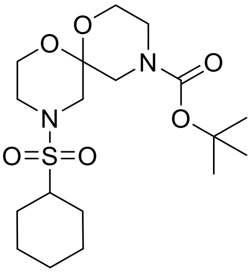

Following general procedure F (p 85),[v] sulfonyl chloride **B05** (3.18 g, 15.7 mmol) was added to a solution of Et_3N (2.9 mL, 21 mmol) and amine **9** (2.70 g, 10.5 mmol) in CH_2Cl_2 (110 mL) at 0 °C. After 24 h, the reaction was worked up according to the general procedure and the crude product was purified by flash column chromatography (*n*-heptane/EtOAc, 3/1 + 1 v/v% Et_3N) to produce sulfonamide **9-B05** as a viscous yellow oil (2.89 g, 68%).

R_f (*n*-heptane/EtOAc, 3/2) = 0.3.

ν_{max} (KBr/cm^{-1}): 2978 m, 2931 m, 2858 m, 1694 br s, 1427 m, 1281 m, 1156 s.

^{1}H-NMR (400 MHz, C_6D_6, ~1:1 mixture of rotamers based on the presence of relative half integrals) δ_H 4.20 – 3.63 (stack, 1.5H), 3.63 – 3.30 (stack, 3.5H), 3.30 – 2.98 (stack, 3H), 2.78 – 2.40 (stack, 5H), 2.19 – 1.89 (stack, 2H), 1.66 – 1.25 (stack, 14H), 1.00 – 0.75 (stack, 3H).

^{13}C{^{1}H}-NMR (101 MHz, C_6D_6, mixture of rotamers) δ_C 154.4 (C, broad), [91.5, 91.2 (C)], 79.2 (C), 61.3 (CH), 59.5 (CH_2), 59.2 (CH_2), [50.3, 49.2, 47.9, 44.5, 43.2, 42.0 (4 × CH_2)], 28.1 (CH_3), [26.42, 26.38, 25.1, 25.0 (5 × CH_2)].

LRMS (ES+): 427.2 [$(M + Na)^+$, 100%], 405.2 [20, $(M + H)^+$].

HRMS (ES+): calcd for $C_{18}H_{32}N_2O_6SNa$ $[M + Na]^+$ 427.1879, found 427.1873.

4-(cyclohexylsulfonyl)-1,7-dioxa-4,10-diazaspiro[5.5]undecane (L1-B05):

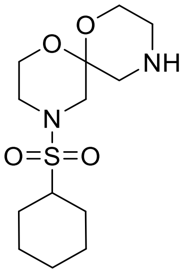

Following general procedure H (p 86), Boc-protected amine **9-B05** (2.30 g, 5.69 mmol) was added to a solution of HCl in 1,4-dioxane (7.1 mL of a 4 M solution, 28 mmol) at rt. After 1 h, the reaction was

[v] 2.0 equivalents of Et_3N were used instead of the 3.0 equivalents described in General procedure F.

worked up according to the general procedure and the crude product was washed with a mixture of Et_2O/CH_2Cl_2 (1:1, 20 mL) to produce amine HCl salt **L1-B05·HCl** as an off-white solid (1.90 g, 98%).
100 mg of the product was purified by the basic preparative HPLC method for characterization purposes and the rest of the product was used for library synthesis without further purification. The characterization data are reported on the free amine.

ν_{max} (neat/cm^{-1}): 2972 w, 2938 m, 2859 m, 1322 s, 1145 s, 970 s, N–H stretch not observed.

^{1}H-NMR (400 MHz, CD_3OD) δ_H 3.92 (app td, J = 11.6, 2.9 Hz, 1H), 3.87 – 3.79 (m, 1H), 3.73 – 3.64 (m, 1H), 3.63 – 3.53 (stack, 2H), 3.53 – 3.45 (m, 1H), 3.20 – 3.01 (stack, 2H), 2.93 – 2.74 (stack, 4H), 2.66 (d, J = 13.2 Hz, 1H), 2.23 – 2.06 (m, 2H), 1.95 – 1.80 (m, 2H), 1.76 – 1.64 (m, 1H), 1.58 – 1.42 (m, 2H), 1.42 – 1.14 (stack, 3H), 1H (probably NH) not observed.

^{13}C{^{1}H}-NMR (101 MHz, CD_3OD) δ_C 91.9 (C), 62.4 (CH), 61.2 (CH_2), 60.7 (CH_2), 51.6 (CH_2), 51.2 (CH_2), 45.9 (CH_2), 45.2 (CH_2), [27.7, 27.6, 26.4, 26.2 (5 × CH_2)].

LRMS (ES+): 305.2 [$(M + H)^+$, 100%].

HRMS (ES+): calcd for $C_{13}H_{25}N_2O_4S$ $[M + H]^+$ 305.1535, found 305.1540.

4-(cyclohexylsulfonyl)-10-((1,3,5-trimethyl-1*H*-pyrazol-4-yl)sulfonyl)-1,7-dioxa-4,10-diazaspiro[5.5]undecane (L1-B05-B10):

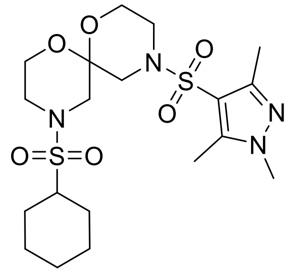

Following general procedure J (p 87), sulfonyl chloride **B10** (98 mg, 0.47 mmol) was added to a solution of Et_3N (98 µL, 0.70 mmol) and amine HCl salt **L1-B05·HCl** (80 mg, 0.23 mmol) in CH_2Cl_2 (2 mL) at rt. After 18 h, the reaction was worked up according to the general procedure and the crude product was purified by the neutral preparative HPLC method to produce sulfonamide **L1-B05-B10** (73 mg, 65%).

ν_{max} (neat/cm^{-1}): 2934 w, 2885 w, 1503 w, 1324 m, 1147 s, 1065 s, 983 s.

^{1}H-NMR (400 MHz, $(CD_3)_2SO$) δ_H 3.80 – 3.60 (stack, 7H), 3.42 – 3.25 (m, 4H), 3.15 – 2.99 (stack, 3H), 2.49 – 2.43 (m, 1H), 2.42 – 2.34 (stack, 4H), 2.24 (s, 3H), 2.04 – 1.91 (m, 2H), 1.80 – 1.68 (m, 2H), 1.64 – 1.55 (m, 1H), 1.39 – 1.02 (stack, 5H).

^{13}C{^{1}H}-NMR (101 MHz, $(CD_3)_2SO$) δ_C 146.8, 142.9, 110.1, 91.5, 59.9, 59.5, 58.8, 49.9, 49.4, 44.4, 44.2, 36.5, 26.2, 24.9, 24.7, 13.3, 10.7.

LRMS (ES+): 515.1 [$(M + K)^+$, 10%], 499.2 [85, $(M + Na)^+$], 477.2 [100, $(M + H)^+$].

HRMS (ES+): calcd for $C_{19}H_{33}N_4O_6S_2$ $[M + H]^+$ 477.1842, found 477.1845.

Retention time (neutral LCMS method): 4.61 min.

10-(cyclohexylsulfonyl)-*N*-(4-methoxybenzyl)-1,7-dioxa-4,10-diazaspiro[5.5]undecane-4-carboxamide (L1-B05-I11):

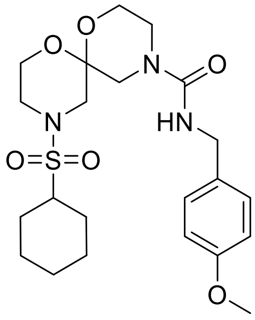

Following general procedure K (p 88), isocyanate **I11** (46 mg, 0.28 mmol) was added to a solution of Et_3N (98 µL, 0.70 mmol) and amine HCl salt **L1-B05·HCl** (80 mg, 0.23 mmol) in CH_2Cl_2 (2 mL) at rt. After 16 h, the reaction was worked up according to the general procedure and the crude product was purified by the neutral preparative HPLC method to produce urea **L1-B05-I11** (76 mg, 69%).

ν_{max} (neat/cm^{-1}): 3331 br w, 2933 m, 2857 w, 1626 m, 1537 m, 1511 s.

^{1}H-NMR (400 MHz, CD_3OD) δ_H 7.27 – 7.18 (AA' of AA'BB', 2H), 6.92 – 6.82 (BB' of AA'BB', 2H), 4.34 (A of AB, $J_{A–B}$ = 15.0 Hz, 1H), 4.27 (B of AB, $J_{B–A}$ = 15.0 Hz, 1H), 3.92 – 3.79 (stack, 4H), 3.78 (s, 3H), 3.69 – 3.61 (stack, 2H), 3.60 – 3.52 (stack, 2H), 3.21 – 3.01 (stack, 3H), 3.01 – 2.93 (stack, 2H), 2.24 – 2.04 (m, 2H), 1.94 – 1.79 (m, 2H), 1.78 – 1.65 (m, 1H), 1.59 – 1.06 (stack, 5H), 1H (probably NH) not observed.

^{13}C{^{1}H}-NMR (101 MHz, CD_3OD) δ_C 160.2, 160.1, 133.4, 129.5, 114.7, 93.0, 62.4, 60.9, 60.7, 55.7, 51.4, 49.8, 45.9, 44.7, 44.1, 27.7, 27.6, 26.4, 26.2.

LRMS (ES+): 957.4 [$(2M + Na)^+$, 10%], 506.2 [10, $(M + K)^+$], 490.2 [85, $(M + Na)^+$], 468.2 [100, $(M + H)^+$].

HRMS (ES+): calcd for $C_{22}H_{34}N_3O_6S$ $[M + H]^+$ 468.2168, found 468.2163.

Retention time (neutral LCMS method): 4.88 min.

4-(cyclohexylsulfonyl)-10-(pyrimidin-2-ylmethyl)-1,7-dioxa-4,10-diazaspiro[5.5] undecane (L1-B05-A06):

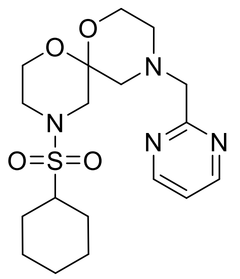

Following general procedure L (p 88), a suspension of $NaBH(OAc)_3$ (100 mg, 0.47 mmol) in 1,2-dichloroethane (3.1 mL) was added to a solution of the aldehyde **A06** (51 mg, 0.47 mmol) and the amine HCl salt **L1-B05·HCl** (80 mg, 0.23 mmol) in 1,2-dichloroethane (2 mL) at rt. After 16 h, the reaction was worked up according to the general procedure and the crude product was purified by the basic preparative HPLC method to produce 3° amine **L1-B05-A06** (60 mg, 65%).

ν_{max} (neat/cm^{-1}): 2972 w, 2934 w, 1634 w, 1562 m, 1320 m, 1139 s, 1055 s.

^{1}H-NMR (400 MHz, CD_3OD) δ_H 8.80 (d, J = 5.0 Hz, 2H), 7.42 (t, J = 5.0 Hz, 1H), 4.00 – 3.75 (stack, 4H), 3.72 – 3.61 (stack, 2H), 3.61 – 3.53 (stack, 2H), 3.19 – 3.00 (stack, 2H), 2.95 – 2.88 (m, 1H), 2.88 – 2.78 (stack, 2H), 2.54 (app td, J = 11.1, 3.4 Hz, 1H), 2.30 (d, J = 11.4 Hz, 1H), 2.19 – 2.07 (m, 2H), 1.93 – 1.83 (m, 2H), 1.76 – 1.66 (m, 1H), 1.56 – 1.41 (m, 2H), 1.40 – 1.13 (stack, 3H).

^{13}C{^{1}H}-NMR (101 MHz, CD_3OD) δ_C 167.5, 158.5, 121.3, 93.6, 65.0, 62.3, 61.3, 60.7, 58.9, 53.2, 51.6, 46.0, 27.64, 27.60, 26.4, 26.2.

LRMS (ES+): 419.2 [(M + Na)$^+$, 35%], 397.2 [100, (M + H)$^+$].

HRMS (ES+): calcd for $C_{18}H_{29}N_4O_4S$ [M + H]$^+$ 397.1910, found 397.1908.

Retention time (basic LCMS method): 4.37 min.

***tert*-butyl 4-(pyridin-3-ylsulfonyl)-1,7-dioxa-4,11-diazaspiro[5.6]dodecane-11-carboxylate (25-B08 *or* 27 in the main article):**

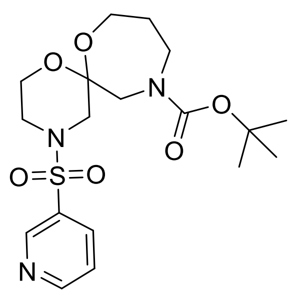

Following general procedure F (p 85), sulfonyl chloride **B08** (1.86 g, 10.5 mmol) was added to a solution of Et_3N (2.9 mL, 21 mmol) and amine **25** (2.00 g, 6.98 mmol) in CH_2Cl_2 (70 mL) at 0 °C. After 16 h, the

reaction was worked up according to the general procedure and the crude product was purified by flash column chromatography (gradient, 0–100% EtOAc in *n*-heptane) to produce sulfonamide **25-B08** as a light-yellow solid (2.57 g, 89%).

R_f (CH_2Cl_2/MeOH, 20/1) = 0.6.

ν_{max} (KBr/cm^{-1}): 2978 m, 1693 s, 1573 m, 1417 s, 1167 s, 1059 s.

^{1}H-NMR (400 MHz, $CDCl_3$, ~1:1 mixture of rotamers based on the relative half integrals) δ_H 9.03 – 8.85 (stack, 1H), 8.82 – 8.65 (stack, 1H), 8.12 – 7.95 (stack, 1H), 7.53 – 7.28 (stack, 1H), 4.26 – 4.16 (m, 0.5H), 4.03 – 3.91 (m, 0.5H), 3.91 – 3.33 (stack, 7H), 2.98 – 2.43 (stack, 4H), 2.02 – 1.77 (stack, 1H), 1.58 – 1.30 (stack, 10H).

^{13}C{^{1}H}-NMR (101 MHz, $CDCl_3$, mixture of rotamers) δ_C [155.3, 154.2 (C)], [152.7, 152.4 (CH)], 148.0 (CH), [136.0, 135.8 (CH)], [135.1, 134.5 (C)], [123.9, 123.6 (CH)], [97.9, 97.5 (C)], [80.6, 80.2, (C)] 61.5 (CH_2), [59.2, 59.0 (CH_2)], [54.6, 54.2 (CH_2)], [53.5, 53.3 (CH_2)], [49.3, 48.8 (CH_2)], [44.6, 44.4 (CH_2)], [30.4, 30.1 (CH_2)], [28.5, 28.4 (CH_3)].

LRMS (ES+): 436.2 [(M + Na)$^+$, 35%], 414.2 (15), 358.1 [100, (M + H – *t*-Bu)$^+$].

HRMS (ES+): calcd for $C_{18}H_{27}N_3O_6SNa$ [M + Na]$^+$ 436.1518, found 436.1526.

4-(pyridin-3-ylsulfonyl)-1,7-dioxa-4,11-diazaspiro[5.6]dodecane dihydrochloride (L2-B08·2HCl *or* 30·2HCl in the main article):

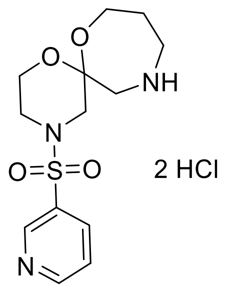

Following general procedure H (p 86), a solution of HCl in 1,4-dioxane (7.7 mL of a 4 M solution, 31 mmol) was added to a solution of Boc-protected amine **25-B08** (2.55 g, 6.17 mmol) in CH_2Cl_2 (10 mL) at rt. After 4 h, the reaction was worked up according to the general procedure and the crude product was washed with CH_2Cl_2 (2 × 20 mL) to produce amine HCl salt **L2-B08·2HCl** as a light-yellow foam (2.24 g, 94%). The yield was calculated for 2HCl salt. The 2HCl form is an assumption based on the resonance shift of the pyridyl carbon resonances compared with precursor sulfonamide **25-B08**.

R_f (CH_2Cl_2/MeOH, 20/1) = 0.1.

ν_{max} (thin film/cm^{-1}): 3418 br s, 2939 w, 1627 br m, 1462 m, 1634 s, 1173 s, 1069 s.

^{1}H-NMR (400 MHz, CD_3OD) δ_H 9.42 (br s, 1H), 9.18 – 9.11 (m, 1H), 9.06 – 8.94 (m, 1H), 8.28 (dd, J = 8.1, 5.7 Hz, 1H), 4.28 – 4.15 (m, 1H), 3.91 – 3.66 (stack, 4H), 3.62 – 3.44 (stack, 3H), 3.35 – 3.27 (m, 1H), 3.22 – 3.01 (stack, 3H), 2.23 – 2.04 (m, 1H), 2.00 – 1.89 (m, 1H), 3Hs (probably NHs) not observed.

^{13}C{^{1}H}-NMR (101 MHz, CD_3OD) δ_C 147.6 (CH), 145.1 (CH), 143.8 (CH), 140.4 (C), 128.9 (CH), 96.2 (C), 62.2 (CH_2), 60.5 (CH_2), 53.5 (CH_2), 52.6 (CH_2), 49.5 (CH_2), 45.4 (CH_2), 28.0 (CH_2).

LRMS (ES+): 314.1 [$(M + H)^+$, 100%], 240.1 (30).

HRMS (ES+): calcd for $C_{13}H_{20}N_3O_4S$ $[M + H]^+$ 314.1175, found 314.1183.

(1-(4-fluorophenyl)cyclopropyl)(4-(pyridin-3-ylsulfonyl)-1,7-dioxa-4,11-diazaspiro[5.6]dodecan-11-yl)methanone (L2-B08-C34):

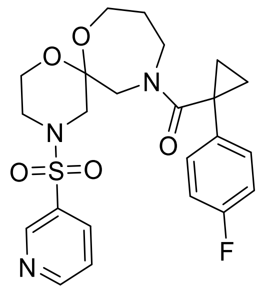

Following general procedure I (p 87), carboxylic acid **C34** (26 mg, 0.19 mmol), Et_3N (66 µL, 0.47 mmol) and a solution of HATU (72 mg, 0.19 mmol) in DMF (0.38 mL) were added sequentially to a solution of amine HCl salt **L2-B08·2HCl** (60 mg, 0.16 mmol) in DMF (2 mL) at rt. After 24 h, the reaction was worked up according to the general procedure and the crude product was purified by the neutral preparative HPLC method to provide amide **L2-B08-C34** (51 mg, 57%).

ν_{max} (KBr/cm^{-1}): 2945 w, 1634 s, 1573 w, 1510 s, 1173 s, 1061 s.

^{1}H-NMR (400 MHz, CD_3OD, resonance broadening observed because of rotamers) δ_H 9.03 – 8.89 (m, 1H), 8.86 – 8.63 (m, 1H), 8.32 – 8.07 (m, 1H), 7.64 (dd, J = 8.0, 4.9 Hz, 1H), 7.40 – 7.26 (m, 2H), 7.20 – 7.07 (m, 2H), 4.38 – 4.20 (stack, 2H), 3.81 (d, J = 13.0 Hz, 1H), 3.79 – 3.63 (stack, 3H), 3.61 – 3.50 (m, 1H), 3.31 – 3.17 (m, 1H), 3.05 – 2.91 (stack, 2H), 2.91 – 2.79 (stack, 2H), 1.61 – 1.48 (stack, 2H), 1.36 – 1.20 (stack, 2H), 1.15 – 0.87 (stack, 2H).

^{13}C{^{1}H}-NMR (101 MHz, CD_3OD, resonance broadening observed because of rotamers) δ_C 174.4, 163.15 (d, J = 244.8 Hz), 154.0, 149.2, 137.4 (d, J = 3.2 Hz), 137.3, 136.5, 129.3 (d, J = 8.0 Hz), 125.4, 116.8 (d, J = 21.7 Hz), 98.7, 61.6, 60.1, 55.0, 53.6, 50.9, 45.7, 30.5, 30.2, 16.3, 13.8.

LRMS (ES+): 498.2 [$(M + Na)^+$, 20%], 476.2 [100, $(M + H)^+$].

HRMS (ES+): calcd for $C_{23}H_{27}FN_3O_5S$ $[M + H]^+$ 476.1655, found 476.1669.

Retention time (neutral LCMS method): 2.45 min.

(2-methylpyridin-4-yl)(4-(pyridin-3-ylsulfonyl)-1,7-dioxa-4,11-diazaspiro[5.6]dodecan-11-yl)methanone (L2-B08-C38):

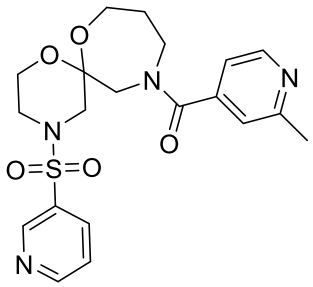

Following general procedure I (p 87), carboxylic acid **C38** (34 mg, 0.19 mmol), Et_3N (66 µL, 0.47 mmol) and a solution of HATU (72 mg, 0.19 mmol) in DMF (0.38 mL) were added sequentially to a solution of amine HCl salt **L2-B08·2HCl** (60 mg, 0.16 mmol) in DMF (2 mL) at rt. After 24 h, the reaction was worked up according to the general procedure and the crude product was purified by the neutral preparative HPLC method to provide amide **L2-B08-C38** (59 mg, 72%).

ν_{max} (thin film/cm^{-1}): 2923 w, 2359 m, 1635 br m, 1177 m, 1061 m.

^{1}H-NMR (400 MHz, CD_3OD, ~4:1 mixture of rotamers based on the relative integration of the resonances at δ_H 8.31 – 8.17 ppm and at 8.13 – 8.05 ppm) δ_H 9.02 – 8.94 (m, 0.8H), 8.91 – 8.71 (stack, 1.2H), 8.61 – 8.43 (stack, 1H), 8.31 – 8.20 (m, 0.8H), 8.14 – 8.02 (m, 0.2H), 7.77 – 7.53 (stack, 1H), 7.45 – 7.20 (stack, 2H), 4.39 (d, *J* = 14.9 Hz, 0.8H), 4.01 – 3.41 (stack, 7.6H), 3.27 – 3.11 (stack, 1.8H), 2.88 – 2.74 (stack, 1.6H), 2.69 – 2.53 (stack, 3H), 2.22 – 1.74 (stack, 1.4H), 1.64 – 1.48 (m, 0.8H).

^{13}C{^{1}H}-NMR (101 MHz, CD_3OD, mixture of rotamers) δ_C 171.6, 171.1, 160.6, 160.2, 154.2, 150.3, 150.0, 149.2, 149.0, 146.1, 137.3, 137.1, 135.9, 125.6, 125.5, 122.9, 121.7, 120.4, 119.3, 98.7, 97.2, 61.9, 61.3, 60.4, 60.2, 58.2, 54.8, 53.8, 52.7, 51.8, 45.8, 45.3, 31.8, 29.1, 24.0.

LRMS (ES+): 433.2 [(M + H)$^+$, 100%].

HRMS (ES+): calcd for $C_{20}H_{25}N_4O_5S$ [M + H]$^+$ 433.1546, found 433.1550.

Retention time (neutral LCMS method): 1.80 min.

2.7.12 First decoration step – Amidations

***tert*-butyl 10-(1-methyl-1*H*-pyrazole-4-carbonyl)-1,7-dioxa-4,10-diazaspiro[5.5]undecane-4-carboxylate (9-C28 *or* 15 in the main article):**

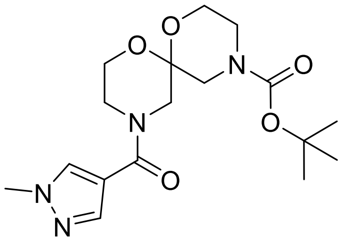

Following general procedure G (p 86), *i*-Pr_2NEt (5.1 mL, 29 mmol), carboxylic acid **C28** (2.26 g, 13.9 mmol) and HATU (5.30 g, 13.9 mmol) were added to a solution of amine **9** (3.00 g, 11.6 mmol) in CH_2Cl_2 (115 mL) at 0 °C. After 16 h, the reaction was worked up according to the general procedure and the crude product was purified by flash column chromatography two times (gradient, 0–5% MeOH in CH_2Cl_2) to provide amide **9-C28** as a light-yellow foam (4.25 g, quant.).

R_f (CH_2Cl_2/MeOH, 9/1) = 0.5.

ν_{max} (KBr/cm^{-1}): 2977 w, 1697 s, 1612 s, 1550 m, 1432 s, 1272 s, 1057 s.

^{1}H-NMR (400 MHz, C_6D_6, resonance broadening observed because of rotamers) δ_H 7.77 (br s, 1H), 7.40 (s, 1H), 4.75 – 3.39 (stack, 6H), 3.22 – 2.98 (stack, 5H), 2.84 – 2.12 (stack, 4H), 1.43 (br s, 9H).

^{13}C{^{1}H}-NMR (101 MHz, C_6D_6, mixture of rotamers) δ_C 163.8 (C), [154.9, 154.6 (C)], 139.2 (CH), 133.2 (CH), 117.7 (C), 91.8 (C, broad), 79.5 (C), 59.7 (CH_2), 59.4 (CH_2), 49.7 (CH_2), 48.3 (CH_2), 43.4 (CH_2), 42.3 (CH_2), 38.3 (CH_3), 28.4 (CH_3).

LRMS (ES+): 440.3 [(M + Et_2NH_2)$^+$, 30%], 367.2 [60, (M + H)$^+$], 311.1 [100, (M + H – *t*-Bu)$^+$], 140.0 (20).

HRMS (ES+): calcd for $C_{17}H_{27}N_4O_5$ [M + H]$^+$ 367.1981, found 367.1985.

(1-methyl-1*H*-pyrazol-4-yl)(1,7-dioxa-4,10-diazaspiro[5.5]undecan-4-yl)methanone (L1-C28 *or* 17·HCl in the main article):

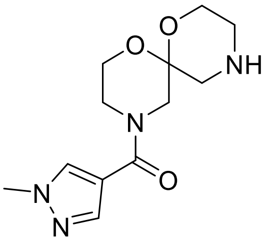

Following general procedure H (p 86), a solution of HCl in 1,4-dioxane (15 mL of a 4 M solution, 58 mmol) was added to a solution of Boc-protected amine **9-C28** (4.25 g, 11.6 mmol) in CH_2Cl_2 (20 mL) at

rt. After 2 h, the reaction was worked up according to the general procedure and the crude product was washed with Et_2O (2 × 15 mL) to produce amine HCl salt **L1-C28·HCl** as a pale-yellow solid (3.50 g, yield over two steps: 98%).

100 mg of the product was purified for characterization purposes by the basic preparative HPLC method and the rest of the product was used for library synthesis without further purification. The characterization data are reported on the free amine.

ν_{max} (neat/cm^{-1}): 3317 br w, 2910 w, 2868 w, 1595 s, 1548 s, 1439 m, 1049 s.

^{1}H-NMR (400 MHz, CD_3OD, resonance broadening observed because of rotamers) δ_H 7.93 (s, 1H), 7.71 (s, 1H), 4.54 – 4.20 (m, 1H), 4.05 – 3.76 (stack, 6H), 3.74 – 3.65 (m, 1H), 3.57 – 3.48 (m, 1H), 3.25 – 3.11 (m, 1H), 3.11 – 2.92 (m, 1H), 2.88 – 2.58 (stack, 4H), 1H (probably NH) not observed.

^{13}C{^{1}H}-NMR (101 MHz, CD_3OD, resonance broadening observed because of rotamers) δ_C 166.3 (C), 140.8 (CH), 133.9 (CH), 117.4 (C), 92.3 (C), 61.3 (CH_2), 60.5 (CH_2), 54.1 (CH_2), 51.2 (CH_2), 45.2 (CH_2), 43.2 (CH_2), 39.1 (CH_3).

LRMS (ES+): 267.2 [(M + H)$^+$, 100%], 126.0 (25).

HRMS (ES+): calcd for $C_{12}H_{19}N_4O_3$ [M + H]$^+$ 267.1457, found 267.1461.

***tert*-butyl 10-(1-phenylcyclopropane-1-carbonyl)-1,7-dioxa-4,10-diazaspiro[5.5]undecane-4-carboxylate (9-C14):**

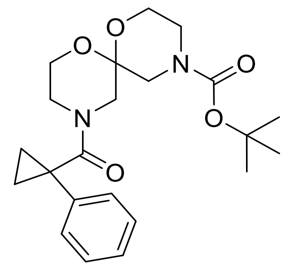

Following general procedure G (p 86), *i*-Pr_2NEt (0.59 mL, 3.4 mmol), carboxylic acid **C14** (437 mg, 2.69 mmol), and HATU (1.02 g, 2.69 mmol) were added to a solution of amine **9** (580 mg, 2.25 mmol) in CH_2Cl_2 (22 mL) at 0 °C. After 20 h, the reaction was worked up according to the general procedure and the crude product was purified by flash column chromatography (*n*-heptane/EtOAc, 2/1) to produce amide **9-C14** as a colorless oil (769 mg, 87%).

R_f (*n*-heptane/EtOAc, 3/2) = 0.3.

ν_{max} (KBr/cm^{-1}): 2975 m, 1698 s, 1645 s, 1427 s, 1278 s, 1056 s.

^{1}H-NMR (400 MHz, C_6D_6, mixture of rotamers, ratio could not be accurately determined) δ_H 7.12 – 7.04 (stack, 4H), 7.04 – 6.95 (stack, 1H), [4.71 – 4.25 (stack, 1H), 4.24 – 2.78 (stack, 7H), 2.76 – 1.99 (stack, 4H), 1.97 – 1.70 (br s, 0.5H), 1.68 – 1.10 (stack, 10.7H), 1.05 – 0.61 (stack, 1.8H).
^{13}C{^{1}H}-NMR (101 MHz, C_6D_6, mixture of rotamers) δ_C 170.9 (C), 154.9 (C), 141.5 (C), 128.9 (CH), 126.4 (CH), 125.7 (CH), 92.4 (C, broad), 79.5 (C), 59.4 (CH_2, broad, 2 × resonance overlap), [51.0, 49.6, 48.2, 47.2 (2 × CH_2)], [44.8, 43.5, 42.3, 41.5 (2 × CH_2)], 29.5 (C), 28.4 (CH_3), [16.9, 16.2, 14.9 (2 × CH_2)].
LRMS (ES+): 476.3 [(M + Et_2NH_2)$^+$, 40%], 403.2 [70, (M + H)$^+$], 347.2 [100, (M + H – *t*-Bu)$^+$].
HRMS (ES+): calcd for $C_{22}H_{31}N_2O_5$ [M + H]$^+$ 403.2233, found 403.2239.

1-phenylcyclopropyl-(1,7-dioxa-4,10-diazaspiro[5.5]undecan-4-yl)methanone (L1-C14):

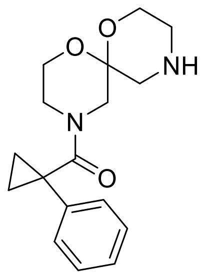

Following general procedure H (p 86), Boc-protected amine **9-C14** (647 mg, 1.61 mmol) was added to a solution of HCl in 1,4-dioxane (2.0 mL of a 4 M solution, 8.1 mmol) at rt. After 1 h, the reaction was worked up according to the general procedure and the crude product was washed with a mixture of Et_2O/CH_2Cl_2 (1:1, 2 × 20 mL) to produce amine HCl salt **L1-C14·HCl** as an off-white solid (471 mg, 87%). 100 mg of the product was purified for characterization purposes by the basic preparative HPLC method and the rest of the product was used for library synthesis without further purification. The characterization data are reported on the free amine:
ν_{max} (neat/cm^{-1}): 3319 w, 2938 w, 1629 s, 1057 s.
^{1}H-NMR (400 MHz, CD_3OD, ~3:2 mixture of rotamers based on the relative integration of the resonances at δ_H 3.16 – 3.01 ppm and at 2.52 – 2.43 ppm) δ_H 7.37 – 7.25 (stack, 2H), 7.24 – 7.11 (stack, 3H), 4.46 – 4.26 (stack, 1H), 4.02 – 3.84 (stack, 1H), 3.84 – 3.60 (stack, 1.8H), 3.60 – 3.32 (stack, 2.2H), 3.16 – 3.01 (m, 0.6H), 2.96 – 2.54 (stack, 5H), 2.52 – 2.43 (m, 0.4H), 1.60 – 1.20 (stack, 3H), 1.20 – 0.98 (m, 0.6H), 0.95 – 0.79 (m, 0.4H), 1H (probably NH) not observed.
^{13}C{^{1}H}-NMR (101 MHz, CD_3OD, mixture of rotamers) δ_C [174.2, 173.9, (C)], 141.7 (C), [129.8, 127.5, 126.6, 126.2 (3 × CH, Ph)], [93.0, 92.3, (C)], 61.1 (CH_2), [60.1, 59.8, (CH_2)], [52.3, 51.3, 48.6, 46.4, 45.2, 42.8 (4 × CH_2)], [30.3, 30.1 (C)], [17.1, 17.0, 15.5 (2 × CH_2)].
LRMS (ES+): 303.2 [(M + H)$^+$, 100%].
HRMS (ES+): calcd for $C_{17}H_{23}N_2O_3$ [M + H]$^+$ 303.1709, found 303.1713.

(2-methylthiazol-4-yl)(10-(1-phenylcyclopropane-1-carbonyl)-1,7-dioxa-4,10-diazaspiro[5.5]undecan-4-yl)methanone (L1-C14-C42):

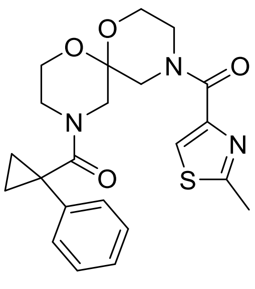

Following general procedure I (p 87), carboxylic acid **C42** (41 mg, 0.28 mmol), Et_3N (0.10 mL, 0.71 mmol) and a solution of HATU (106 mg, 0.28 mmol) in DMF (0.57 mL) were added sequentially to a solution of amine HCl salt **L1-C14·HCl** (80 mg, 0.24 mmol) in DMF (2 mL) at rt. After 24 h, the reaction was worked up according to the general procedure and the crude product was purified by the neutral preparative HPLC method to provide amide **L1-C14-C42** (76 mg, 75%).

ν_{max} (neat/cm^{-1}): 2928 w, 2878 w, 1623 s, 1488 m, 1427 s, 1051 s.

^{1}H-NMR (400 MHz, CD_3OD, mixture of rotamers) δ_H 7.86 (s, 1H), 7.39 – 7.08 (stack, 5H), 4.57 – 3.88 (stack, 4H), 3.87 – 2.78 (stack, 8H), 2.72 (s, 3H), 1.59 – 1.24 (stack, 3H), 1.21 – 0.80 (stack, 1H).

^{13}C{^{1}H}-NMR (101 MHz, CD_3OD, mixture of rotamers) δ_C 174.1, 173.8, 168.1, 167.9, 165.7, 165.2, 149.9, 149.6, 141.6, 129.8, 127.6, 126.6, 126.2, 125.4, 94.4, 93.9, 93.4, 60.9, 60.3, 60.0, 53.6, 53.3, 52.1, 51.8, 48.1, 47.6, 46.2, 43.2, 42.5, 30.2, 30.1, 18.9, 17.0, 15.4.

LRMS (ES+): 450.2 [(M + Na)$^+$, 55%], 428.2 [100, (M + H)$^+$].

HRMS (ES+): calcd for $C_{22}H_{26}N_3O_4S$ [M + H]$^+$ 428.1644, found 428.1646.

Retention time (neutral LCMS method): 4.62 min.

***tert*-butyl 10-(2-phenoxyacetyl)-1,7-dioxa-4,10-diazaspiro[5.5]undecane-4-carboxylate (9-C08):**

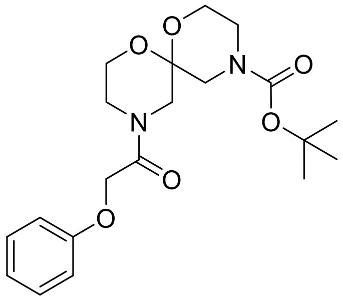

Following general procedure G (p 86), *i*-Pr_2NEt (4.0 mL, 23 mmol), carboxylic acid **C08** (2.12 g, 13.9 mmol) and HATU (5.30 g, 13.9 mmol) were added to a solution of amine **9** (3.00 g, 11.6 mmol) in CH_2Cl_2

(22 mL) at 0 °C. After 16 h, the reaction was worked up according to the general procedure and the crude product was purified by flash column chromatography two times (gradient, 0–100% EtOAc in *n*-heptane, gradient, 0–10% MeOH in CH_2Cl_2) to produce amide **9-C08** as a light-green oil (4.45 g, calculated yield 90%). After purification by column chromatography, the product was still contaminated with a small amount of tetramethylurea [8.5% as determined by ^{1}H-NMR spectroscopy (methyl resonance appears as a singlet at δ_H 2.82 ppm)]. Therefore, 100 mg of the product was purified for characterization purposes by the neutral preparative HPLC method and the rest of the product was used in the Boc-deprotection step without further purification.

R_f (*n*-heptane/EtOAc, 3/2) = 0.6.

ν_{max} (KBr/cm^{-1}): 2932 w, 1686 br s, 1656 s, 1056 m.

^{1}H-NMR (400 MHz, $CDCl_3$, mixture of rotamers, ratio could not be accurately determined) δ_H 7.35 – 7.17 (stack, 2H), 7.06 – 6.81 (stack, 3H), 4.85 – 4.60 (stack, 2H), 4.50 – 4.28 (stack, 1H), 4.07 – 3.42 (stack, 7H), 3.41 – 3.29 (m, 0.5H), 3.26 – 3.12 (m, 0.5H), 3.11 – 2.65 (stack, 3H), 1.45 (stack, 9H).

$^{13}C\{^1H\}$-NMR (101 MHz, $CDCl_3$, mixture of rotamers) δ_C [168.0, 167.4 (C)], [157.9, 157.8 (C)], 155.1 (C), [129.7, 129.6 (CH)], [121.8, 121.7 (CH)], [114.73, 114.67 (CH)], 91.9 (C, broad), [80.4, 80.3 (C)], [67.9, 67.2 (CH_2)], [59.8, 59.7, 59.6, 59.5 (2 × CH_2], [51.0, 49.4, 48.3, 47.1 (2 × CH_2), [45.0, 43.4, 42.2, 41.9 (2 × CH_2), 28.4 (CH_3).

LRMS (ES+): 415.2 [(M + Na)$^+$, 60%], 393.2 [80, (M + H)$^+$], 337.1 [100, (M + H – *t*-Bu)$^+$], 126.0 (25).

HRMS (ES+): calcd for $C_{20}H_{29}N_2O_6$ [M + H]$^+$ 393.2026, found 393.2025.

2-phenoxy-1-(1,7-dioxa-4,10-diazaspiro[5.5]undecan-4-yl)ethan-1-one (L1-C08):

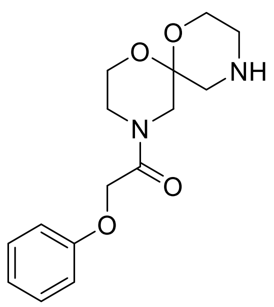

Following general procedure H (p 86), a solution of HCl in 1,4-dioxane (14 mL of a 4 M solution, 56 mmol) was added to a solution of the Boc-protected amine **9-C08** (4.33 g, 11.0 mmol) in CH_2Cl_2 (30 mL) at rt. After 2 h, the reaction was worked up according to the general procedure and the crude product was washed with a mixture of Et_2O/CH_2Cl_2 (5:1, 2 × 25 mL) to produce the amine HCl salt **L1-C08·HCl** as a white solid (3.24 g, 86%).

100 mg of the product was purified for characterization purposes by the basic preparative HPLC method and the rest of the product was used for library synthesis without further purification. The data are reported on the free amine:

R_f (CH_2Cl_2/MeOH, 9/1) = 0.2.

ν_{max} (neat/cm^{-1}): 3651 w, 2981 m, 1654 m, 1599 m, 1043 s.

^{1}H-NMR (400 MHz, CD_3OD, ~7:3 mixture of rotamers based on the relative integration of the resonances at δ_H 4.37 ppm and at 4.25 ppm) δ_H 7.34 – 7.18 (stack, 2H), 6.96 (stack, 3H), 4.78 (stack, 2H), 4.37 (app d, J = 13.3 Hz, 0.7H), 4.25 (d, J = 13.3 Hz, 0.3H), 3.93 – 3.62 (stack, 4H), 3.62 – 3.53 (m, 0.7H), 3.49 – 3.41 (m, 0.3H), 3.39 – 3.28 (m, 0.3H), 3.16 (d, J = 13.9 Hz, 0.7H), 2.99 – 2.61 (stack, 5H), 1H (probably NH) not observed.

^{13}C{^{1}H}-NMR (101 MHz, CD_3OD) δ_C [170.1, 169.5 (C)], [159.6, 159.4 (C)], [130.5, 130.4 (CH)], [122.5, 122.4 (CH)], [115.8, 115.7 (CH)], [92.8, 92.3 (C)], [67.5, 66.7 (CH_2)], [61.3, 61.1 (CH_2], [60.5, 60.4 (CH_2)], [51.7, 51.2, 51.0, 48.2, 45.5, 45.2, 45.1, 43.1 (4 × CH_2)].

LRMS (ES+): 315.1 [(M + Na)$^+$, 35%], 293.2 [100, (M + H)$^+$], 196.1 (20), 126.0 (50).

HRMS (ES+): calcd for $C_{15}H_{21}N_2O_4$ [M + H]$^+$ 293.1501, found 293.1505.

***tert*-butyl 10-(4-(trifluoromethoxy)benzoyl)-1,7-dioxa-4,10-diazaspiro[5.5]undecane-4-carboxylate (9-C16):**

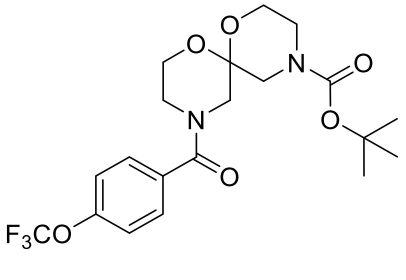

Following general procedure G (p 86), i-Pr_2NEt (4.2 mL, 24 mmol), carboxylic acid **C16** (2.40 g, 11.6 mmol) and HATU (4.42 g, 11.6 mmol) were added to a solution of amine **9** (2.50 g, 9.68 mmol) in CH_2Cl_2 (100 mL) at 0 °C. After 16 h, the reaction was worked up according to the general procedure and the crude product was purified by flash column chromatography (gradient, 0–100% EtOAc in n-heptane) to provide amide **9-C16** as a pale-yellow solid (4.02 g, 93%).

R_f (CH_2Cl_2/MeOH, 9/1) = 0.5.

ν_{max} (KBr/cm^{-1}): 2981 w, 1700 s, 1621 br s, 1261 br s, 1159 s, 1058 s.

^{1}H-NMR (400 MHz, C_6D_6, mixture of rotamers, ratio could not be accurately determined) δ_H 7.53 – 6.97 (stack, 2H), 6.91 – 6.71 (stack, 2H), 4.77 – 3.81 (stack, 2H), 3.79 – 3.32 (stack, 3H), 3.32 – 2.90 (stack, 2.7H), 2.86 – 2.21 (stack, 3.6H), 2.21 – 1.87 (stack, 0.7H), 1.43 (s, 9H).
^{13}C{^{1}H}-NMR (101 MHz, C_6D_6, mixture of rotamers) δ_C 169.1 (C), [154.8, 154.5 (C)], 150.1 (C), 134.9 (C), 130.0 (CH, broad), 121.04 (q, J = 258.6 Hz), 120.8 (CH), 92.0 (C, broad), 79.6 (C), 59.5 (CH_2), 59.3 (CH_2), [52.6, 49.5, 48.1 (2 × CH_2)], [43.4, 42.2, 41.7 (2 × CH_2)], 28.4 (CH_3).
^{19}F-NMR (376 MHz, C_6D_6) δ_F −57.64.
LRMS (ES+): 520.3 [$(M + Et_2NH_2)^+$, 45%], 447.2 [40, $(M + H)^+$], 391.1 [100, $(M + H – t\text{-Bu})^+$], 140.0 (20).
HRMS (ES+): calcd for $C_{20}H_{26}F_3N_2O_6$ $[M + H]^+$ 447.1743, found 447.1740.

(1,7-dioxa-4,10-diazaspiro[5.5]undecan-4-yl)(4-(trifluoromethoxy)phenyl)methanone hydrochloride (L1-C16·HCl):

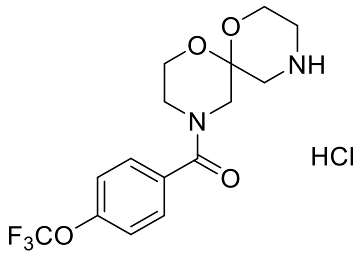

Following general procedure H (p 86), Boc-protected amine **9-C16** (3.92 g, 8.79 mmol) was added to a solution of HCl in 1,4-dioxane (11 mL of a 4 M solution, 44 mmol) at rt. After 1 h, the reaction was worked up according to the general procedure and the crude product was washed with CH_2Cl_2 (2 × 15 mL) to provide amine HCl salt **L1-C16·HCl** as a white solid (3.17 g, 94%).
R_f (CH_2Cl_2/MeOH, 9/1) = 0.3.
ν_{max} (neat/cm^{-1}): 3340 w, 2920 w, 1610 m, 1438 m, 1151 s, 1051 s.
^{1}H-NMR (400 MHz, CD_3OD, ~1:1 mixture of rotamers based on the presence of relative half integrals) δ_H 7.64 – 7.48 (stack, 2H), 7.45 – 7.26 (stack, 2H), 4.64 – 4.45 (stack, 1H), 4.09 – 3.97 (stack, 1H), 3.96 – 3.80 (stack, 2.5H), 3.77 – 3.57 (stack, 1.5H), 3.52 – 2.95 (stack, 6H), 2Hs (probably NHs) not observed.
^{13}C{^{1}H}-NMR (101 MHz, CD_3OD, mixture of rotamers) δ_C 172.1 (C), [151.6, 151.6 (C)], 135.1 (C), [130.8, 130.4 (CH)], 122.2 (CH), 121.8 (q, J = 257.5 Hz, C), [92.9, 92.4 (C)], 60.7 (CH_2), 57.7 (CH_2), 53.3 (CH_2), 48.0 (CH_2), 43.3 (CH_2), 42.7 (CH_2).
^{19}F-NMR (376 MHz, CD_3OD) δ_F –59.40 (br s).
LRMS (ES+): 347.1 [$(M + H)^+$, 100%]
HRMS (ES+): calcd for $C_{15}H_{18}F_3N_2O_4$ $[M + H]^+$ 347.1219, found 347.1225.

(4-(trifluoromethoxy)phenyl)(10-((1,3,5-trimethyl-1*H*-pyrazol-4-yl)sulfonyl)-1,7-dioxa-4,10-diazaspiro[5.5]undecan-4-yl)methanone (L1-C16-B10):

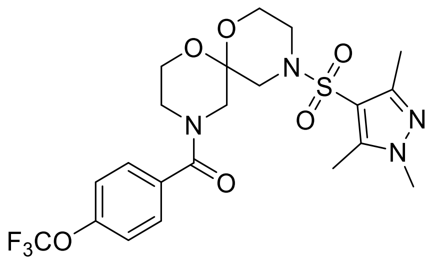

Following general procedure J (p 87), sulfonyl chloride **B10** (87 mg, 0.42 mmol) was added to a solution of Et_3N (87 µL, 0.63 mmol) and amine HCl salt **L1-C16·HCl** (80 mg, 0.21 mmol) in CH_2Cl_2 (2 mL) at rt. After 18 h, the reaction was worked up according to the general procedure and the crude product was purified by the neutral preparative HPLC method to produce sulfonamide **L1-C16-B10** (99 mg, 91%).

ν_{max} (neat/cm^{-1}): 3125 w, 2911 w, 1595 m, 1438 m, 1253 m, 1050 s.

^{1}H-NMR (400 MHz, CD_3OD, mixture of rotamers) δ_H 7.64 – 7.50 (stack, 2H), 7.45 – 7.25 (stack, 2H), 4.59 – 4.35 (stack, 1H), 3.98 – 3.67 (stack, 7H), 3.63 – 3.26 (stack, 4H), 3.25 – 2.97 (stack, 1H), 2.82 – 2.24 (stack, 8H).

^{13}C{^{1}H}-NMR (101 MHz, CD_3OD, mixture of rotamers) δ_C 171.9, 171.5, 151.4, 149.2, 144.7, 135.2, 130.8, 130.3, 122.2, 122.0, 121.8 (q, *J* = 256.5 Hz), 112.7, 93.8, 93.2, 60.7, 60.6, 60.5, 60.4, 54.0, 51.1, 50.7, 48.2, 48.0, 45.4, 43.0, 36.6, 13.5, 11.1.

LRMS (ES+): 541.1 [(M + Na)$^+$, 70%], 519.2 [100, (M + H)$^+$].

HRMS (ES+): calcd for $C_{21}H_{26}F_3N_4O_6S$ [M + H]$^+$ 519.1525, found 519.1533.

Retention time (neutral LCMS method): 4.81 min.

***tert*-butyl 10-(3-methylbutanoyl)-1,7-dioxa-4,10-diazaspiro[5.5]undecane-4-carboxylate (9-C09):**

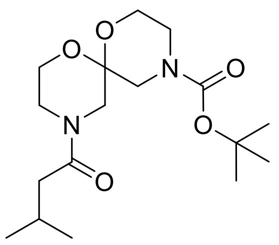

Following general procedure G (p 86), *i*-Pr_2NEt (0.66 mL, 3.8 mmol), carboxylic acid **C09** (308 mg, 3.02 mmol) and HATU (1.15 g, 3.02 mmol) were added to a solution of amine **9** (650 mg, 2.52 mmol) in

CH_2Cl_2 (25 mL) at 0 °C. After 40 h, the reaction was worked up according to the general procedure and the crude product was purified by flash column chromatography (gradient, 0–100% EtOAc in *n*-heptane) to produce amide **9-C09** as a white solid (806 mg, 94%).

R_f (*n*-heptane/EtOAc, 3/2) = 0.2.

ν_{max} (KBr/cm^{-1}): 2962 m, 1688 s, 1640 s, 1426 s, 1057 s.

^{1}H-NMR (400 MHz, C_6D_6, mixture of rotamers, ratio could not be accurately determined) δ_H 4.61 – 4.38 (stack, 1H), 4.24 – 3.71 (stack, 1.6H), 3.71 – 3.31 (stack, 2.5H), 3.27 – 2.91 (stack, 2.5H), 2.86 – 2.47 (stack, 2.2H), 2.47 – 2.28 (stack, 2.8H), 2.24 – 2.04 (stack, 1H), 1.96 – 1.71 (stack, 1.4H), 1.44 (br s, 9H), 0.99 – 0.84 (stack, 6H).

^{13}C{^{1}H}-NMR (101 MHz, C_6D_6, mixture of rotamers) δ_C 170.7 (C), 154.7 (C), [91.8, 91.2 (C, broad)], [79.6, 79.4 (C)], [59.7, 59.6, 59.2 (2 × CH_2)], 51.0 (CH_2), [49.7, 48.4 (CH_2)], [46.3, 44.5, 43.4, 42.4, 41.9, 41.7, 41.0 (3 × CH_2)], 28.4 (CH_3), [25.7, 25.5 (CH)], [23.03, 22.98, 22.92, 22.88 (CH_3)].

LRMS (ES+): 416.3 [(M + Et_2NH_2)$^+$, 100%], 343.2 [50, (M + H)$^+$], 287.2 [90, (M + H – *t*-Bu)$^+$], 140.0 (30).

HRMS (ES+): calcd for $C_{17}H_{31}N_2O_5$ [M + H]$^+$ 343.2233, found 343.2234.

3-methyl-1-(1,7-dioxa-4,10-diazaspiro[5.5]undecan-4-yl)butan-1-one hydrochloride (L1-C09·HCl):

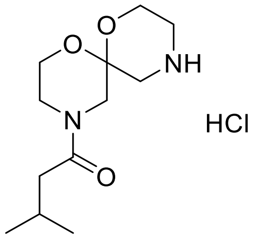

Following general procedure H (p 86), a solution of HCl in 1,4-dioxane (2.8 mL of a 4 M solution, 11 mmol) was added to a solution of Boc-protected amine **9-C09** (760 mg, 2.22 mmol) in CH_2Cl_2 (10 mL) at rt. After 2 h, an extra volume of HCl in 1,4-dioxane (1.1 mL of a 4 M solution, 4.4 mmol) was added to the mixture. After 1 h, the reaction was worked up according to the general procedure and the HCl salt was washed with CH_2Cl_2 (2 × 15 mL) to provide amine HCl salt **L1-C09·HCl** as a white solid (615 mg, 99%).

R_f (CH_2Cl_2/MeOH, 9/1) = 0.1.

ν_{max} (neat/cm^{-1}): 3296 w, 2957 m, 1619 s, 1461 m, 1053 s.

^{1}H-NMR (400 MHz, CD_3OD, ~1:1 mixture of rotamers based on the presence of relative half integrals) δ_H 4.47 – 4.35 (stack, 1H), 4.03 – 3.86 (stack, 2H), 3.86 – 3.67 (stack, 3H), 3.41 – 3.06 (stack, 5H), 2.94 – 2.82 (m, 0.5H), 2.79 (app d, *J* = 13.5 Hz, 0.5H), 2.36 – 2.15 (stack, 2H), 2.12 – 1.93 (stack, 1H), 0.98 – 0.82 (stack, 6H), 2Hs (probably NHs) not observed.

$^{13}C\{^1H\}$-NMR (101 MHz, CD_3OD, mixture of rotamers) δ_C [174.5, 174.3 (C)], [92.9, 92.3 (C)], [61.2, 61.0 (CH_2)], [57.6, 57.5 (CH_2)], [51.7, 48.2, 47.0, 46.1, 43.5, 43.4, 42.59, 42.55, 42.1 (5 × CH_2)], [27.1, 26.7 (CH)], [23.0, 22.9, 22.82, 22.78 (CH_3)], the other rotamer resonance of a CH_2 carbon overlaps with the CD_3OD resonances, however HMBC and HSQC measurements confirmed its presence between δ_C 49.5 – 49.0 ppm.
LRMS (ES+): 243.2 [$(M + H)^+$, 100%], 126.0 (50).
HRMS (ES+): calcd for $C_{12}H_{23}N_2O_3$ $[M + H]^+$ 243.1709, found 243.1708.

tert-butyl 10-(1-methylcyclohexane-1-carbonyl)-1,7-dioxa-4,10-diazaspiro[5.5]undecane-4-carboxylate (9-C10):

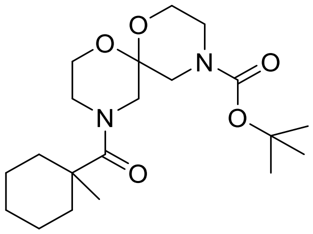

Following general procedure G (p 86), *i*-Pr_2NEt (3.6 mL, 20 mmol), carboxylic acid **C10** (1.74 g, 12.3 mmol) and HATU (4.66 g, 12.3 mmol) were added to a solution of amine **9** (2.64 g, 10.2 mmol) in CH_2Cl_2 (100 mL) at 0 °C. After 5 days, the reaction was worked up according to the general procedure and the product purified by flash column chromatography two times (gradient, 0–10% MeOH in CH_2Cl_2 and gradient, 0–100% EtOAc in *n*-heptane) to provide amide **9-C10** as a white solid (3.27 g, 84%). After purification by column chromatography, the product was still contaminated with a small amount of tetramethylurea [5.3% as determined by ^1H-NMR spectroscopy (methyl resonance appears as a singlet at δ_H 2.82 ppm)] and by-product **S54** [5.8% as determined by ^1H-NMR spectroscopy]. Therefore, 100 mg of the product was purified by the neutral preparative HPLC method for characterization purposes and the rest of the product was used in the Boc-deprotection step without further purification.
R_f (CH_2Cl_2/MeOH, 9/1) = 0.9.
ν_{max} (KBr/cm^{-1}): 2967 m, 2937 m, 1696 s, 1624 s, 1429 s, 1130 s, 1055 s.
^1H-NMR (400 MHz, $CDCl_3$, mixture of rotamers, ratio could not be accurately determined) δ_H 4.37 – 4.14 (stack, 2H), 4.04 – 3.73 (stack, 4H), 3.73 – 3.50 (stack, 2H), 3.26 – 2.76 (stack, 4H), 2.13 – 1.93 (stack, 2H), 1.62 – 1.42 (stack, 14H), 1.42 – 1.30 (stack, 3H), 1.25 (s, 3H).
$^{13}C\{^1H\}$-NMR (101 MHz, $CDCl_3$, mixture of rotamers) δ_C 176.5 (C), 155.1 (C), 92.0 (C), 80.2 (C), 60.0 (CH_2), 59.4 (CH_2), [49.9, 49.3, 48.3 (2 × CH_2)], [44.9, 43.6, 42.7, 42.2 (2 x CH_2 and CH), 37.4 (CH_2), 37.0 (CH_2), 28.4 (CH_3), 25.9 (CH_2), 24.4 (CH_3), 23.00 (CH_2), 22.98 (CH_2)].

LRMS (ES+): 405.2 [(M + Na)$^+$, 40%], 383.3 [60, (M + H)$^+$], 327.2 [100, (M + H – *t*-Bu)$^+$], 126.0 (20).

HRMS (ES+): calcd for $C_{20}H_{35}N_2O_5$ [M + H]$^+$ 383.2546, found 383.2547.

3*H*-[1,2,3]triazolo[4,5-b]pyridine-3-yl 1-methylcyclohexane-1-carboxylate (S54):

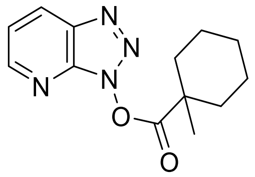

By-product formed during the amidation of **9** with **C10** in the presence of HATU (see p 128). By-product **S54** was a white solid.

ν_{max} (KBr/cm^{-1}): 2935 s, 2849 m, 1803 s, 1598 m, 1454 m.

^{1}H-NMR (400 MHz, $CDCl_3$, resonance broadening observed because of rotamers) δ_H 8.66 (dd, *J* = 4.5, 1.4 Hz, 1H), 8.35 (dd, *J* = 8.4, 1.4 Hz, 1H), 7.36 (dd, *J* = 8.4, 4.5 Hz, 1H), 2.33 – 2.19 (stack, 2H), 1.75 – 1.39 (stack, 10H), 1.38 – 1.22 (stack, 1H).

^{13}C{^{1}H}-NMR (101 MHz, $CDCl_3$, resonance broadening observed because of rotamers) δ_C 173.5, 151.9, 140.9, 135.2, 129.4, 120.9, 43.7, 35.7, 26.4, 25.5, 23.1.

LRMS (ES+): 283.1 [(M + Na)$^+$, 30%], 261.1 [95, (M + H)$^+$], 137.1 (100).

HRMS (ES+): calcd for $C_{13}H_{17}N_4O_2$ [M + H]$^+$ 261.1352, found 261.1354.

(1-methylcyclohexyl)(1,7-dioxa-4,10-diazaspiro[5.5]undecan-4-yl)methanone (L1-C10):

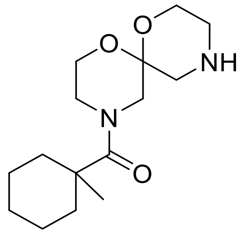

Following general procedure H (p 86), Boc-protected amine **9-C10** (2.50 g, 6.50 mmol) was added to a solution of HCl in 1,4-dioxane (9.0 mL of a 4 M solution, 36 mmol) at rt. After 1 h, the reaction was worked up according to the general procedure and the crude product was washed with CH_2Cl_2 (2 × 25 mL) to provide amine HCl salt **L1-C10·HCl** as an off-white solid (1.64 g, 79%).

100 mg of the product was purified by the basic preparative HPLC method for characterization purposes and the rest of the product was used for library synthesis without further purification. The data are reported on the free amine:

ν_{max} (neat/cm^{-1}): 3659 w, 2919 br m, 1623 s, 1412 m, 1046 s.

^{1}H-NMR (400 MHz, CD_3OD, resonance broadening observed because of rotamers) δ_H 4.34 (d, J = 13.6 Hz, 1H), 4.23 (d, J = 13.4 Hz, 1H), 3.91 – 3.76 (stack, 2H), 3.73 – 3.66 (m, 1H), 3.58 – 3.46 (m, 1H), 3.20 – 2.99 (m, 1H), 2.95 – 2.76 (stack, 4H), 2.69 (d, J = 13.2 Hz, 1H), 2.18 – 2.01 (stack, 2H), 1.64 – 1.44 (stack, 5H), 1.44 – 1.29 (stack, 3H), 1.26 (s, 3H), 1H (probably NH) not observed.

^{13}C{^{1}H}-NMR (101 MHz, CD_3OD, resonance broadening observed because of rotamers) δ_C 178.4 (C), 92.5 (C), 61.1 (CH_2), 60.7 (CH_2), 51.6 (CH_2), 51.4 (CH_2), 46.0 (CH_2), 45.3 (CH_2), 44.0 (C), 38.7 (CH_2), 38.2 (CH_2), 27.0 (CH_2), 25.2 (CH_3), 24.3 (CH_2), 24.2 (CH_2).

LRMS (ES+): 283.2 [$(M + H)^+$, 100%].

HRMS (ES+): calcd for $C_{15}H_{27}N_2O_3$ $[M + H]^+$ 283.2022, found 283.2025.

(10-(2,3-dihydro-1*H*-inden-2-yl)-1,7-dioxa-4,10-diazaspiro[5.5]undecan-4-yl)(1-methylcyclohexyl)methanone (L1-C10-K02):

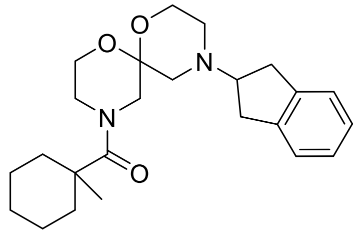

Following general procedure L (p 88), a suspension of $NaBH(OAc)_3$ (212 mg, 1.0 mmol) in 1,2-dichloroethane (6.7 mL) was added to a solution of ketone **K02** (0.13 g, 1.0 mmol) and amine HCl salt **L1-C10·HCl** (80 mg, 0.25 mmol) in 1,2-dichloroethane (0.5 mL) at rt. After 16 h, the reaction was worked up according to the general procedure and the product purified by the basic preparative HPLC method to provide 3° amine **L1-C10-K02** (68 mg, 68%).

ν_{max} (neat/cm^{-1}): 3422 br w, 2926 m, 1627 m, 1415 m, 1058 s.

^{1}H-NMR (400 MHz, CD_3OD, mixture of rotamers) δ_H 7.22 – 7.15 (stack, 2H), 7.15 – 7.10 (stack, 2H), 4.38 – 4.23 (stack, 2H), 3.97 – 3.77 (stack, 2H), 3.73 – 3.58 (stack, 2H), 3.22 – 3.01 (stack, 4H), 3.01 – 2.76 (stack, 5H), 2.39 – 2.23 (m, 1H), 2.20 – 2.01 (stack, 3H), 1.66 – 1.44 (stack, 5H), 1.44 – 1.30 (stack, 3H), 1.26 (s, 3H).

$^{13}C\{^1H\}$-NMR (101 MHz, CD_3OD, mixture of rotamers) δ_C 178.4, 142.22, 142.18, 127.64, 127.63, 125.36, 125.35, 94.1, 68.5, 61.0, 60.7, 58.0, 52.1, 51.9, 46.2, 44.1, 38.7, 38.2, 37.50, 37.47, 27.0, 25.3, 24.3, 24.2.

LRMS (ES+): 399.3 [$(M + H)^+$, 100%], 126.0 (50).

HRMS (ES+): calcd for $C_{24}H_{35}N_2O_3$ $[M + H]^+$ 399.2648, found 399.2650.

Retention time (basic LCMS method): 5.82 min.

tert-butyl 10-(4-methyloxazole-5-carbonyl)-1,7-dioxa-4,10-diazaspiro[5.5]undecane-4-carboxylate (9-C30):

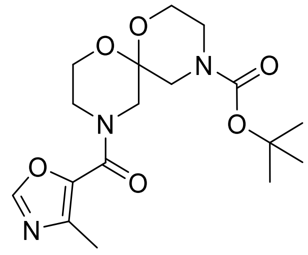

Following general procedure G (p 86), _i_-Pr_2NEt (5.5 mL, 32 mmol), carboxylic acid **C30** (1.95 g, 15.3 mmol) and HATU (5.83 g, 15.3 mmol) were added to a solution of amine **9** (3.30 g, 12.8 mmol) in CH_2Cl_2 (130 mL) at 0 °C. After 16 h, the reaction was worked up according to the general procedure and the crude product was purified by flash column chromatography (gradient, 0–5% MeOH in CH_2Cl_2) to provide amide **9-C30** as a light-yellow oil (4.69 g, quant.).

R_f (CH_2Cl_2/MeOH, 9/1) = 0.5.

ν_{max} (thin film/cm^{-1}): 2978 m, 1696 s, 1633 s, 1455 s, 1287 s, 1055 s.

^1H-NMR (400 MHz, C_6D_6, mixture of rotamers) δ_H 6.99 (s, 1H), 4.60 – 3.38 (stack, 6H), 3.18 – 2.90 (stack, 2H), 2.76 – 2.26 (stack, 7H), 1.43 (br s, 9H).

$^{13}C\{^1H\}$-NMR (101 MHz, C_6D_6, mixture of rotamers) δ_C 159.1 (C), 154.8 (C), 149.3 (CH), 143.3 (C), 140.1 (C), 91.8 (C), 79.6 (C), 59.5 (CH_2, 2 × resonance overlap), 49.6 (CH_2), 48.2 (CH_2), 43.5 (CH_2), 42.2 (CH_2), 28.4 (CH_3), 13.4 (CH_3).

LRMS (ES+): 441.3 [$(M + Et_2NH_2)^+$, 30%], 368.2 [30, $(M + H)^+$], 312.1 [100, $(M + H - t\text{-Bu})^+$].

HRMS (ES+): calcd for $C_{17}H_{26}N_3O_6$ $[M + H]^+$ 368.1822, found 368.1819.

(4-methyloxazol-5-yl)(1,7-dioxa-4,10-diazaspiro[5.5]undecan-4-yl)methanone (L1-C30):

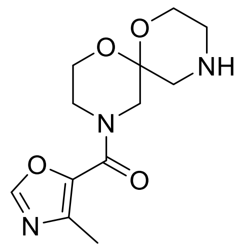

Following general procedure H (p 86), Boc-protected amine **9-C30** (4.69 g, 12.8 mmol) was added to a solution of HCl in 1,4-dioxane (16 mL of a 4 M solution, 64 mmol) at rt. After 1 h, the reaction was worked up according to the general procedure and the crude product was washed sequentially with Et_2O (2 × 15 mL) and CH_2Cl_2 (2 × 15 mL) to produce amine HCl salt **L1-C30·HCl** as a pale-yellow solid (2.84 g, yield over two steps 73%). The product was still contaminated with a small amount of tetramethylurea [3.0% as determined by ^{1}H-NMR spectroscopy (methyl resonance appears as a singlet at δ_H 2.82 ppm)]. Therefore, 100 mg of the product was purified by the basic preparative HPLC method for characterization purposes and the rest of the product was used for library synthesis without further purification. The data are reported on the free amine:

ν_{max} (neat/cm^{-1}): 3306 br w, 2927 w, 1626 s, 1440 m, 1045 s.

^{1}H-NMR (400 MHz, CD_3OD, mixture of rotamers, ratio could not be accurately determined) δ_H 8.23 (s, 1H), 4.51 – 3.42 (stack, 6H), 3.22 – 2.54 (stack, 6H), 2.39 – 2.30 (stack, 3H), 1H (probably NH) not observed.

^{13}C{^{1}H}-NMR (101 MHz, CD_3OD, mixture of rotamers) δ_C [164.0, 161.5 (C)], 152.6 (CH), 142.5 (C), 140.7 (C), [92.8, 92.2 (C, broad)], 61.3 (CH_2), 60.1 (CH_2), [53.6, 51.5, 51.1 (2 × CH_2)], [47.3, 45.3, 45.1, 43.2 (2 × CH_2)], 12.7 (CH_3).

LRMS (ES+): 268.1 [$(M + H)^+$, 100%].

HRMS (ES+): calcd for $C_{12}H_{18}N_3O_4$ $[M + H]^+$ 268.1297, found 268.1305.

tert-butyl 10-(1-methyl-1H-imidazole-2-carbonyl)-1,7-dioxa-4,10-diazaspiro[5.5]undecane-4-carboxylate (9-C27):

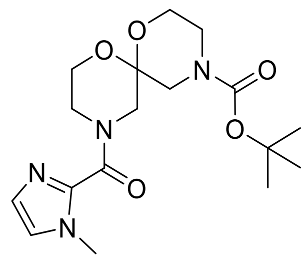

Following general procedure G (p 86), i-Pr_2NEt (5.5 mL, 32 mmol), carboxylic acid **C27** (1.93 g, 15.3 mmol) and HATU (5.83 g, 15.3 mmol) were added to a solution of the amine **9** (3.30 g, 12.8 mmol) in CH_2Cl_2 (130 mL) and DMF (20 mL)[w] at 0 °C. After 16 h, the reaction was worked up according to the general procedure and the product purified by flash column chromatography (gradient, 0–5% MeOH in CH_2Cl_2) to provide amide **9-C27** as a white solid (3.83 g, 82%).

R_f (CH_2Cl_2/MeOH, 9/1) = 0.6.

ν_{max} (KBr/cm^{-1}): 2978 w, 1695 s, 1633 s, 1474 s, 1054 s.

^{1}H-NMR (400 MHz, C_6D_6, mixture of rotamers, ratio could not be accurately determined) δ_H 7.06 – 6.86 (stack, 1H), 6.25 – 6.08 (stack, 1H), 5.33 – 5.06 (stack, 1H), 4.68 – 4.56 (m, 0.4H), 4.49 – 4.31 (m, 0.6H), 4.21 – 4.06 (m, 0.4H), 4.01 – 2.95 (stack, 9.6H), 2.80 – 2.32 (stack, 3H), 1.41 (br s).

^{13}C{^{1}H}-NMR (101 MHz, C_6D_6, mixture of rotamers) δ_C 160.4, 159.3, 154.9, 154.5, 140.1, 140.0, 127.4, 127.3, 124.2, 124.1, 93.0, 92.5, 91.8, 79.4, 60.4, 59.6, 59.5, 53.0, 49.7, 49.5, 48.4, 48.1, 47.3, 46.7, 43.5, 42.2, 41.6, 35.2, 35.1, 28.41, 28.39.

LRMS (ES+): 367.2 [$(M + H)^+$, 100%], 311.1 [35, $(M + H – t\text{-Bu})^+$].

HRMS (ES+): calcd for $C_{17}H_{27}N_4O_5$ $[M + H]^+$ 367.1981, found 367.1991.

(1-methyl-1H-imidazol-2-yl)(1,7-dioxa-4,10-diazaspiro[5.5]undecan-4-yl)methanone (L1-C27):

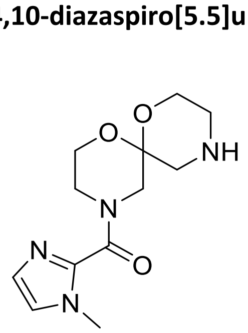

Following general procedure H (p 86), a solution of HCl in 1,4-dioxane (13 mL of a 4 M solution, 52 mmol) was added to a solution of Boc-protected amine **9-C27** (3.82 g, 10.4 mmol) in CH_2Cl_2 (30 mL) at

[w] The carboxylic acid did not dissolve in CH_2Cl_2, therefore DMF was included as a co-solvent.

rt. After 1 h, an extra volume of HCl in 1,4-dioxane (5.5 mL of a 4 M solution, 21 mmol) was added. After 1 h, the reaction was worked up according to the general procedure and the crude product was washed with CH_2Cl_2 (2 × 50 mL) to provide amine HCl salt **L1-C27·HCl** salt as a light-green foam (2.73 g, 86%).

100 mg of the product was purified by the basic preparative HPLC method for characterization purposes and the rest of the product was used for library synthesis without further purification. The characterization data are reported on the free amine:

ν_{max} (neat/cm^{-1}): 3296 br w, 1927 w, 1623 s, 1467 m, 1044 s.

^{1}H-NMR (400 MHz, CD_3OD, ~2:1 mixture of rotamers based on the relative integration of the resonances at δ_H 4.34 – 4.25 ppm and at 3.16 ppm) δ_H 7.24 (br s, 1H), 7.07 – 6.99 (stack, 1H), 4.53 – 4.40 (stack, 1H), 4.34 – 4.25 (m, 0.4H), 4.03 – 3.49 (stack, 7.6H), 3.42 (app td, *J* = 13.4, 3.6 Hz, 0.4H), 3.36 – 3.29 (m, 0.6H), 3.16 (td, *J* = 12.8, 3.8 Hz, 0.6H), 2.96 – 2.70 (stack, 3.8H), 2.54 (d, *J* = 13.2 Hz, 0.6H), 1H (probably NH) not observed.

^{13}C{^{1}H}-NMR (101 MHz, CD_3OD, mixture of rotamers) δ_C [163.1, 161.8 (C)], [141.3, 141.0 (C)], [128.0, 127.9 (CH)], [125.3, 125.1 (CH)], [92.9, 92.2 (C)], [61.2, 60.9, 60.8, 60.2 (2 × CH_2)], [54.5, 51.1, 50.7, 48.3 (2 × CH_2)], [47.7, 45.0, 44.9, 43.1 (2 × CH_2)], [34.8, 34.5 (CH_3)].

LRMS (ES+): 289.1 [(M + Na)$^+$, 25%], 267.2 [100, (M + H)$^+$], 206.1 (70), 130.2 (30).

HRMS (ES+): calcd for $C_{12}H_{19}N_4O_3$ [M + H]$^+$ 267.1457, found 267.1464.

***N*-(4-methoxybenzyl)-10-(1-methyl-1*H*-imidazole-2-carbonyl)-1,7-dioxa-4,10-diazaspiro[5.5]undecane-4-carboxamide (L1-C27-I11):**

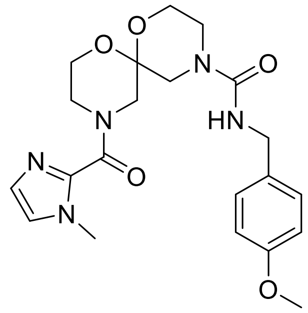

Following general procedure K (p 88), isocyanate **I11** (52 mg, 0.32 mmol) was added to a solution of Et_3N (74 µL, 0.53 mmol) and amine HCl salt **L1-C27·HCl** (80 mg, 0.26 mmol) in CH_2Cl_2 (2 mL) at rt. After 16 h, the reaction was worked up according to the general procedure and the crude product was purified by the neutral preparative HPLC method to provide urea **L1-C27-I11** (67 mg, 59%).

ν_{max} (neat/cm^{-1}): 3319 br w, 2936 w, 1715 w, 1627 s, 1049 s.

^{1}H-NMR (400 MHz, CD_3OD, mixture of rotamers, ratio could not be accurately determined) δ_H 7.28 – 7.16 (stack, 3H), 7.07 – 7.00 (stack, 1H), 6.89 – 6.84 (stack, 2H), 4.52 – 4.42 (stack, 1H), 4.38 – 4.18 (stack, 2.8H), 4.07 – 3.76 (stack, 10H), 3.75 – 3.52 (stack, 2.5H), 3.48 – 3.36 (stack, 1H), 3.24 – 3.04 (stack, 1.2H), 3.04 – 2.87 (stack, 1H), 2.85 – 2.78 (stack, 0.5H), 1H (probably NH) was not observed.
^{13}C{^{1}H}-NMR (101 MHz, CD_3OD, mixture of rotamers) δ_C 163.0, 161.8, 160.3, 160.1, 141.2, 140.9, 133.4, 132.6, 129.6, 129.5, 128.0, 127.9, 125.3, 125.2, 114.8, 114.7, 93.9, 93.1, 61.0, 60.9, 60.7, 60.3, 55.7, 54.3, 52.5, 50.0, 49.7, 48.2, 47.7, 45.0, 44.7, 44.1, 43.9, 43.1, 40.4, 34.8, 34.5.
LRMS (ES+): 452.2 [$(M + Na)^+$, 40%], 430.2 [100, $(M + H)^+$], 126.0 (60).
HRMS (ES+): calcd for $C_{21}H_{28}N_5O_5$ $[M + H]^+$ 430.2090, found 430.2095.
Retention time (neutral LCMS method): 4.42 min.

***tert*-butyl 4-(4-methyloxazole-5-carbonyl)-1,7-dioxa-4,11-diazaspiro[5.6]dodecane-11-carboxylate (25-C30 *or* 26 in the main article):**

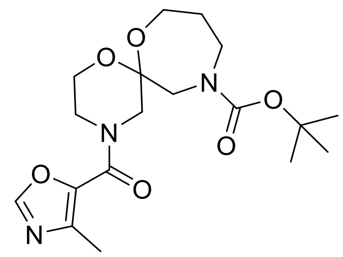

Following general procedure G (p 86), *i*-Pr_2NEt (3.2 mL, 18 mmol), carboxylic acid **C30** (1.26 g, 9.91 mmol) and HATU (3.77 g, 9.91 mmol) were added to a solution of amine **25** (2.25 g, 8.26 mmol) in CH_2Cl_2 (85 mL) at 0 °C. After 16 h, the reaction was worked up according to the general procedure and the crude product was purified by flash column chromatography (gradient, 0–10% MeOH in CH_2Cl_2) to provide amide **25-C30** as a brown oil (2.55 g, calculated yield 78%). After purification by column chromatography, the product was still contaminated with a small amount of tetramethylurea [3.5% as determined by ^{1}H-NMR spectroscopy (methyl resonance appears as a singlet at δ_H 2.82 ppm)]. Therefore, 100 mg of the product was purified by the neutral preparative HPLC method for characterization purposes and the rest of the product was used in the Boc-deprotection step without further purification.
R_f (CH_2Cl_2/MeOH, 9/1) = 0.7.
ν_{max} (thin film/cm^{-1}): 2933 m, 1686 s, 1635 s, 1056 m.

^{1}H-NMR (400 MHz, $CDCl_3$, ~1:1 mixture of rotamers based on the relative half integral) δ_H 7.84 – 7.59 (stack, 1H), 4.75 – 4.32 (stack, 1H), 4.31 – 4.12 (stack, 1H), 4.09 – 3.87 (stack, 3H), 3.86 – 3.58 (stack, 3H), 3.40 – 2.59 (stack, 4H), 2.42 (s, 1.5H), 2.37 (s, 1.5H), 2.20 – 1.66 (stack, 1.5H), 1.64 – 1.21 (stack, 9.5H).

^{13}C{^{1}H}-NMR (101 MHz, $CDCl_3$, mixture of rotamers) δ_C 160.0 (C), [155.1, 154.2 (C)], [149.5, 149.4 (CH)], [142.5, 141.1 (C)], [139.74, 139.66 (C)], [98.0, 97.4 (C)], [80.2, 79.9 (C)], 61.5 (CH_2), 59.9 (CH_2), [55.4, 54.7 (CH_2)], [54.3, 54.1 (CH_2)], [49.7, 48.7 (CH_2)], 42.0 (CH_2), [30.4, 30.2 (CH_2)], [28.4, 28.3 (CH_3)], [13.1, 13.0 (CH_3)].

LRMS (ES+): 785.4 [$(2M + Na)^+$, 30%], 404.2 [100, $(M + Na)^+$], 326.1 [60, $(M + H – t\text{-Bu})^+$].

HRMS (ES+): calcd for $C_{18}H_{27}N_3O_6Na$ $[M + Na]^+$ 404.1798, found 404.1808.

(4-methyloxazol-5-yl)(1,7-dioxa-4,11-diazaspiro[5.6]dodecan-4-yl)methanone (L2-C30 *or* 29 in the main article):

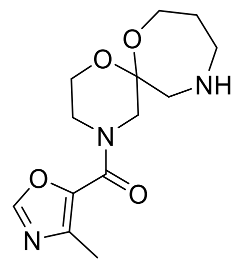

Following general procedure H (p 86), Boc-protected amine **25-C30** (2.60 g, 6.82 mmol) was added to a solution of HCl in 1,4-dioxane (8.5 mL of a 4 M solution, 34 mmol) at rt. After 10 min, MeOH (5 mL) was added as a co-solvent since the reaction mixture had formed a paste, which was affecting stirring. After 4 h, the reaction mixture was neutralized with Et_3N (~8–10 mL) and concentrated under reduced pressure. The product was dissolved in EtOAc (~100 mL) and the $Et_3N{\cdot}HCl$ salt was separated by filtration. The filtrate was concentrated under reduced pressure and the residue purified by flash column chromatography (gradient, 0–10% MeOH in CH_2Cl_2) to provide amine **L2-C30** as a brown oil (1.62 g, 84%). After purification by column chromatography, the product was still contaminated with unidentified minor impurities (<5%). Therefore, 100 mg of the product was purified by the basic preparative HPLC method for characterization purposes and the rest of the product was used for library synthesis without further purification. The data are reported on the free amine:

ν_{max} (thin film/cm^{-1}): 3442 br m, 2944 w, 1630 br s, 1058 m.

^{1}H-NMR (400 MHz, CD_3OD, resonance broadening observed because of rotamers) δ_H 8.22 (s, 1H), 4.61 – 3.78 (stack, 5H), 3.75 – 3.61 (stack, 2H), 3.55 – 2.94 (stack, 3H), 2.89 – 2.58 (stack, 2H), 2.36 (s, 3H), 2.00 – 1.58 (stack, 2H), 1H (probably NH) not observed.

^{13}C{^{1}H}-NMR (101 MHz, CD_3OD, resonance broadening observed because of rotamers) δ_C 161.6 (C), 152.6 (CH), 141.8 (C), 140.8 (C), 99.1 (C, broad), 62.8 (CH_2), 60.3 (CH_2, broad), 57.2 (CH_2), 55.1 (CH_2), 51.6 (CH_2), 43.5 (CH_2), 33.1 (CH_2), 12.7 (CH_3).

LRMS (ES+): 282.2 [100, $(M + H)^+$], 207.1 (35).

HRMS (ES+): calcd for $C_{13}H_{20}N_3O_4$ $[M + H]^+$ 282.1454, found 282.1462.

(4-methyloxazol-5-yl)(11-(methylsulfonyl)-1,7-dioxa-4,11-diazaspiro[5.6]dodecan-4-yl)methanone (L2-C30-B09):

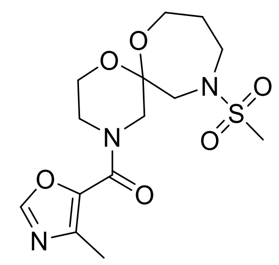

Following general procedure J (p 87), sulfonyl chloride **B09** (41 mg, 0.36 mmol) was added to a solution of Et_3N (74 µL, 0.53 mmol) and 2° amine **L2-C30** (50 mg, 0.18 mmol) in CH_2Cl_2 (2 mL) at rt. After 18 h, the reaction was worked up according to the general procedure and the crude product was purified by the neutral preparative HPLC method to provide sulfonamide **L2-C30-B09** (33 mg, 52%).

ν_{max} (KBr/cm^{-1}): 2930 w, 1631 br s, 1466 m, 1329 s, 1150 s, 1059 s.

^{1}H-NMR (400 MHz, CD_3OD, resonance broadening observed because of rotamers) δ_H 8.23 (s, 1H), 4.41 – 3.66 (stack, 8H), 3.53 – 3.35 (stack, 1H), 3.23 – 2.81 (stack, 6H), 2.36 (s, 3H), 2.11 – 1.65 (stack, 2H).

^{13}C{^{1}H}-NMR (101 MHz, CD_3OD, resonance broadening observed because of rotamers) δ_C 161.5, 152.7, 141.6, 140.8, 98.3, 62.3, 60.8, 55.9, 55.0, 51.5, 50.4, 47.1, 43.4, 37.1, 32.4, 12.8.

LRMS (ES+): 382.1 [$(M + Na)^+$, 100%], 360.1 [60, $(M + H)^+$].

HRMS (ES+): calcd for $C_{14}H_{21}N_3O_6SNa$ $[M + Na]^+$ 382.1049, found 382.1052.

Retention time (neutral LCMS method): 1.74 min.

(4-methyloxazol-5-yl)(11-((3-phenylisoxazol-5-yl)methyl)-1,7-dioxa-4,11-diazaspiro[5.6]dodecan-4-yl)methanone (L2-C30-A05):

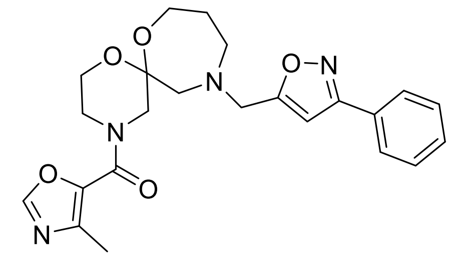

Following general procedure M (p 89), $NaBH(OAc)_3$ (75 mg, 0.36 mmol) was added to a solution of aldehyde **A05** (62 mg, 0.36 mmol) and 2° amine **L2-C30** (50 mg, 0.18 mmol) in CH_2Cl_2 (2 mL) at rt. After 16 h, the reaction was worked up according to the general procedure and the crude product was purified by the basic preparative HPLC method to provide 3° amine **L2-C30-A05** (46 mg, 59%).

ν_{max} (thin film/cm^{-1}): 3477 br w, 2944 w, 2359 w, 1630 br s, 1467 m, 1058 s.

^{1}H-NMR (400 MHz, CD_3OD, resonance broadening observed because of rotamers) δ_H 8.33 – 8.10 (stack, 1H), 8.01 – 7.72 (stack, 2H), 7.56 – 7.45 (stack, 3H), 6.91 – 6.68 (stack, 1H), 4.44 – 4.20 (stack, 1H), 4.13 – 3.78 (stack), 3.73 – 3.57 (stack, 2H), 3.53 – 3.39 (stack, 1H), 3.25 – 2.86 (stack, 3H), 2.85 – 2.64 (stack, 1H), 2.63 – 2.44 (stack, 1H), 2.35 (s, 3H), 2.14 – 1.73 (stack, 1H), 1.73 – 1.53 (stack, 1H).

^{13}C{^{1}H}-NMR (101 MHz, CD_3OD, resonance broadening observed because of rotamers) δ_C 172.1, 163.8, 161.5, 152.6, 141.6, 140.9, 131.3, 130.2, 130.1, 127.8, 102.7, 98.4, 62.5, 60.3, 59.0, 55.5, 54.9, 49.9, 43.6, 32.2, 12.7.

LRMS (ES+): 461.2 [(M + Na)$^+$, 30%], 439.2 [100, (M + H)$^+$].

HRMS (ES+): calcd for $C_{23}H_{27}N_4O_5$ [M + H]$^+$ 439.1981, found 439.1991.

Retention time (basic LCMS method): 2.80 min.

3 Library Enumeration

3.1 Virtual library enumeration and selection of compounds for physical synthesis

Virtual libraries based on scaffolds **9** and **25** were generated using the KNIME analytics platform (http://www.knime.com) and designed to be broadly (some compounds exceeded 500 Da in MW) Lipinski rule-compliant.[29] The basic workflow is summarized in Scheme S19. First, the 2° amine in ring A was derivatized (enumeration 1) using a range of commercially available carboxylic acids, sulfonyl chlorides and isocyanates (see Figure S13, p 166), to provide a series of the corresponding amides, sulfonamides and ureas. 3° Amines, introduced through a virtual reductive amination, were not incorporated into this first enumeration given we expected this basic functional group might cause problems with the second amine derivatization step in the synthesis of the physical compound library. Moreover, compounds containing two basic sites (and protonated at physiological pH) can show a propensity to function as hERG blockers and so were deprioritized for a compound screening library.[30,31]

Scheme S19. Virtual library assembly

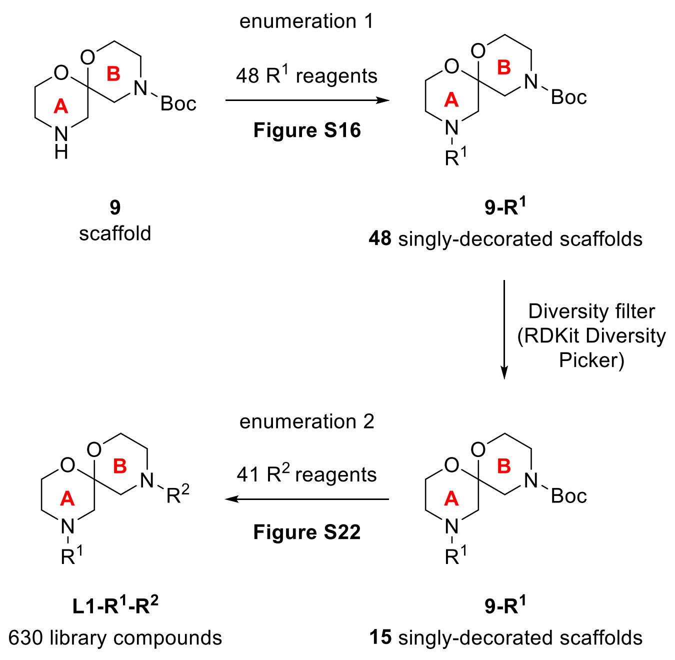

48 achiral (to avoid the formation of diastereoisomers) reagents were selected for the first enumeration step. Given the physical synthesis of 48 different compounds from this first enumeration was deemed impractical, a more synthetically tractable, representative diverse set of 15 compounds (**9-R^1**) were chosen using the "RDKit Diversity Picker" node (Scheme S19, 3.4.1 KNIME methods, p 169).

Virtual deprotection of the Boc carbamate in these 15 singly decorated scaffolds **9-R^1** delivered the corresponding 2° amines. Each compound next underwent virtual derivatization of ring B with a diverse set of 41 achiral decorating reagents (see Figure S11, p 162), comprising aldehydes and symmetrical ketones (reductive amination), carboxylic acids (amidation), sulfonyl chlorides (sulfonylation) and isocyanides (urea formation). This second enumeration delivered 615 (15 × 41) compounds to which were added 15 compounds containing a 2° amine (second derivatization omitted), providing a final virtual library of 630 compounds (Scheme S19, 3.4.1 KNIME methods, p 169) from which a diverse subset of compounds was chosen for physical synthesis.

Assuming a 70% success rate, 138 different reactions would need to be performed to deliver a library of 96 compounds that would occupy every position in a 96-well-plate for screening. Ultimately, a more conservative figure of 150 different decoration reactions was chosen as it would allow us to target 10 compounds from each of our 15 singly decorated scaffolds. Using the Cluster-SkelSphereFP selection method in DataWarrior (https://openmolecules.org/datawarrior/, 3.5 Data Warrior methods, p 193), a diverse set of 150 compounds was identified that was representative of the whole virtual library. This subset was further appraised based on practical considerations; thus, to reduce the number of reactions needed to assemble the physical library, we targeted those singly decorated scaffolds that participated in >7 second-decoration steps. This constraint had little impact, reducing the number of singly decorated scaffolds from 15 to 13 and the final subset to 141 compounds.[x] The final selection of 141 compounds required 13 reagents for the first scaffold-decoration step and 34 reagents for the second decoration step.

Physical Compound Library Synthesis using scaffold 9

A representative amide (**9-C28**), sulfonamide (**9-B08**) and urea (**10-I03**), all products from the first decoration step, were chosen for a validation study. Boc-deprotection of amide **9-C28** and sulfonamide **9-B08** worked well and produced the corresponding HCl salts **L1-C28·HCl** and **L1-B08·2HCl** in excellent yield. In the case of urea **10-I03**, hydrogenolysis of the Cbz carbamate also worked well, furnishing the free amine **L1-I03** in excellent yield (Scheme S20).

[x] We also examined whether the nine filtered compounds should be substituted for compounds derived from our 13 selected singly decorated scaffolds; however, this had little impact on the overall chemical space coverage of the final library.

Scheme S20. Singly decorated sulfonamide, amide and urea scaffold synthesis and subsequent deprotection at the second decorating site

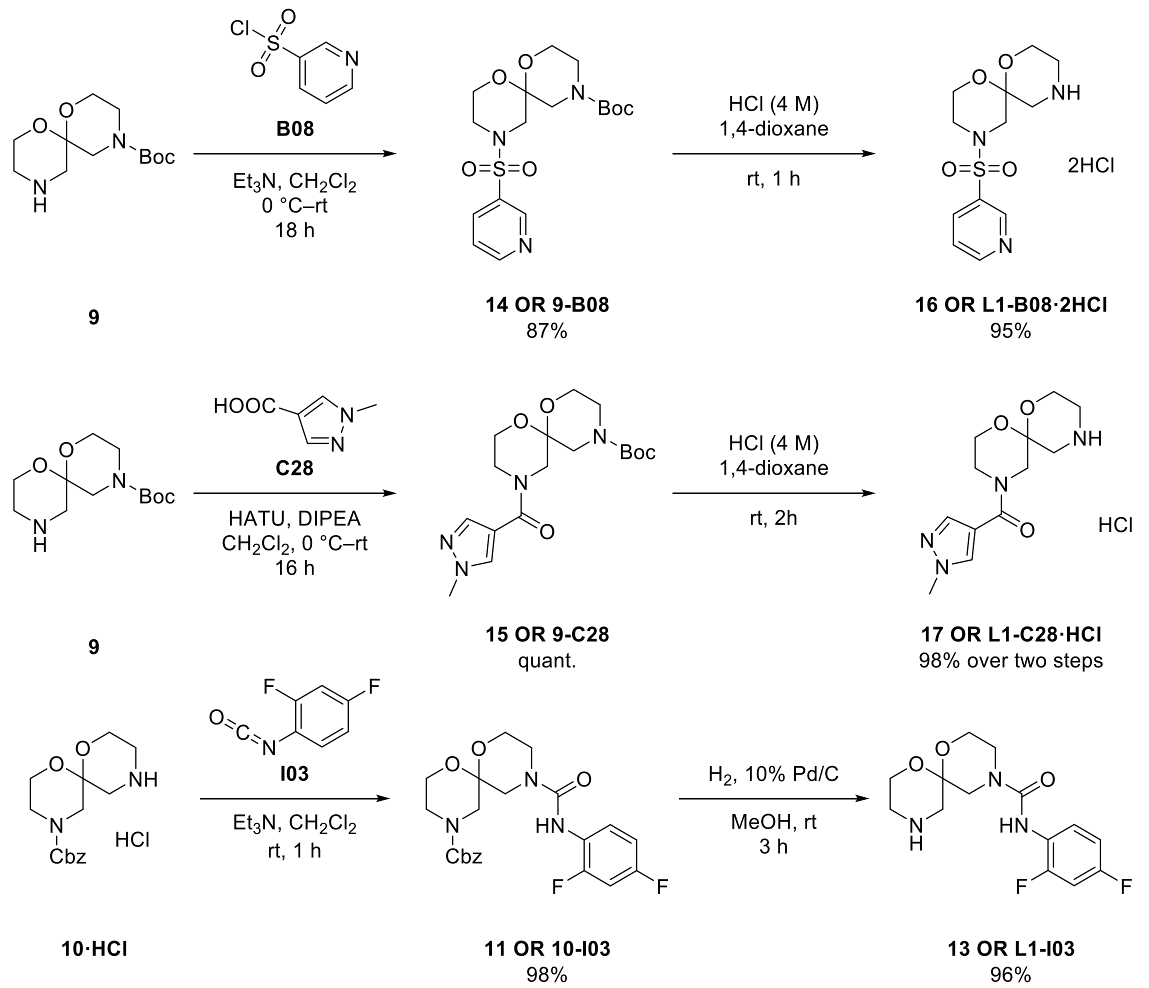

One of each type of the second decoration steps was next tested on urea scaffold **L1-I03** on 50 mg scale in 0.1 mmol mL^{-1} concentration, using reaction protocals that are commonly used at AnalytiCon Discovery GmbH for compound library assembly (Scheme S21). Sulfonylation, amidation and urea formation all worked without event, affording the desired products after purification via preparative HPLC; however, reductive amination proved problematic.

Scheme S21. General secondary (R^2) decoration conditions

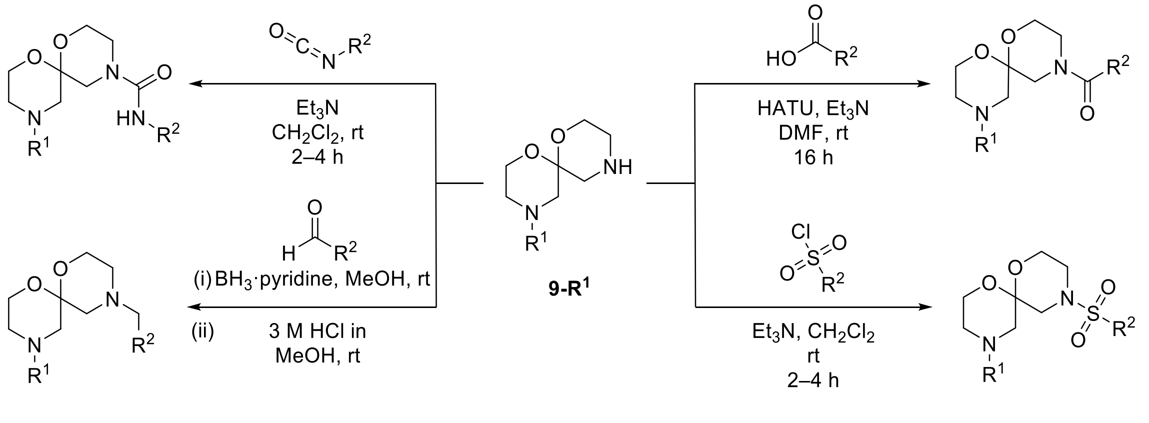

Using BH_3·pyridine complex as the reducing agent and MeOH as the solvent did not effect reductive amination without destroying the spiroacetal moiety, not entirely surprising given the Lewis acidity of borane. Analysis of the reaction mixture by LCMS indicated the formation of methyl acetal by-products arising from transacetalization. These problems were not observed using $NaBH(OAc)_3$ in 1,2-dichloroethane, which delivered the 3° amine product in moderate yield (Scheme S22), which was not reflective of the efficiency of the transformation, more the fact that the reaction was performed on small scale (50 mg) and the product purified by column chromatography.[y]

---

[y] **benzyl 10-(pyrimidin-2-ylmethyl)-1,7-dioxa-4,10-diazaspiro[5.5]undecane-4-carboxylate (10-A06):**

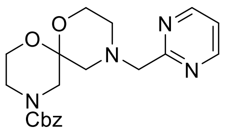

Following general procedure L (p 89), a suspension of $NaBH(OAc)_3$ (63 mg, 0.30 mmol) in 1,2-dichloroethane (2 mL) was added to a mixture of aldehyde **A06** (33 mg, 0.30 mmol) and 2° amine HCl salt **10·HCl** (50 mg, 0.15 mmol) in 1,2-dichloroethane (2 mL) at rt. After 15 h, the reaction mixture was washed with H_2O (2 × 1 mL). The organic layer was then dried over Na_2SO_4, filtered, concentrated under reduced pressure and the residue was purified by flash column chromatography (gradient, 0–10% MeOH in CH_2Cl_2) to produce 3° amine **10-A06** as a colorless oil (25 mg, 43%). General procedure L ($NaBH(OAc)_3$ suspension) was optimized on this compound, hence this compound was only partially characterized.
Selected data:
R_f (CH_2Cl_2/MeOH, 9/1) = 0.6.
^{1}H-NMR (400 MHz, C_6D_6, ~1:1 mixture of rotamers, based on the relative integration of the resonances for δ_H 4.42 ppm, and δ_H 4.10 – 3.94 ppm) δ_H 8.15 (s, 2H), 7.27 – 7.17 (stack, 2H), 7.12 – 6.98 (stack, 3H), 6.24 – 6.08 (stack, 1H), 5.23 – 4.93 (stack, 2H), 4.42 (d, *J* = 13.4 Hz, 0.5H), 4.10 – 3.94 (stack, 1H), 3.88 – 3.65 (stack, 4H), 3.63 – 3.24 (stack, 1.5H), 3.21 – 3.08 (stack, 1H), 2.83 – 2.70 (stack, 1H), 2.70 – 2.40 (stack, 3H), 2.39 – 2.06 (stack, 2H).
$^{13}C\{^1H\}$-NMR (101 MHz, C_6D_6, mixture of rotamers) δ_C 167.5 (C), 156.4 (CH), [155.3, 155.0 (C)], [137.3, 137.2 (C)], 128.3 (CH), 118.8 (CH), [93.0, 92.3 (C)], [66.9, 66.8 (CH_2)], 64.5 (CH_2), [60.7, 60.5 (CH_2)], 58.8 (CH_2), [58.3, 58.1 (CH_2)], 51.9 (CH_2), [49.4, 48.8 (CH_2)], [43.2, 43.0 (CH_2)], resonances for the three phenyl CHs partially overlapped with the C_6D_6 resonances, however HMBC and HSQC measurements confirmed their presence between δ_C 128.3 – 127.4 ppm.
LRMS (ES+): 385.0 [$(M + H)^+$, 100%].

Scheme S22. Reductive amination with NaBH(OAc)$_3$

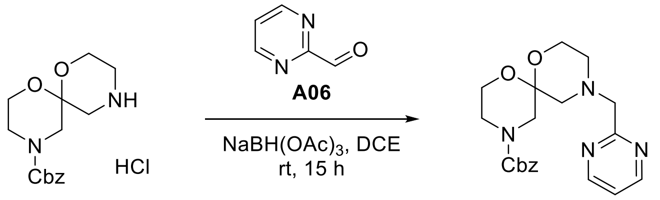

10

10-A06
43%

Having validated all four reactions that would be used in our second decoration step, a library synthesis feasibility study was conducted on three differently functionalized mono-decorated scaffolds, namely urea **L1-I03** (Figure S3), amide **L1-C14·HCl** (Figure S4) and sulfonamide **L1-B05·HCl** (Figure S5), which would deliver a library of 32 compounds. Reactions were performed on 60–80 mg scale in the same format that would be used for full library synthesis. Only one reaction, mesylation (**B09**) of **L1-B05**, failed. After purification via preparative HPLC, 31 products were isolated in 21–77% yield and >90% purity as determined by by ^{1}H-NMR spectroscopy and LCMS analysis.

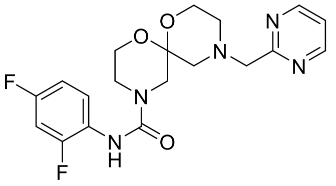

L1-I03-A06
64% yield
100% purity

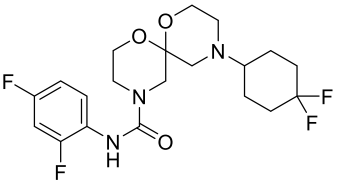

L1-I03-K01
55% yield
100% purity

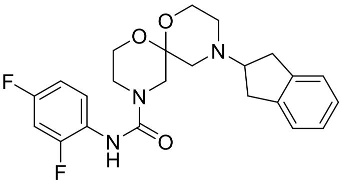

L1-I03-K02
62% yield
98% purity

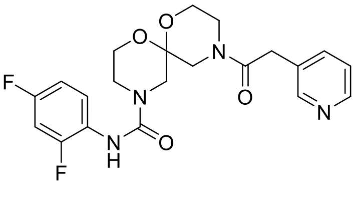

L1-I03-C39
68% yield
99% purity

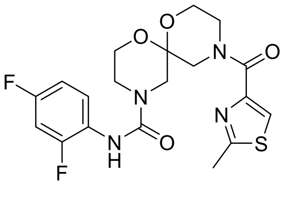

L1-I03-C42
63% yield
100% purity

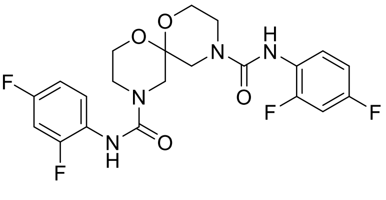

L1-I03-I03
73% yield
100% purity

Figure S3: Library compounds synthesized from urea scaffold L1-I03.

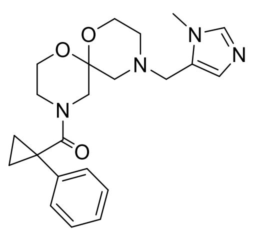

L1-C14-A01
71% yield
97% purity

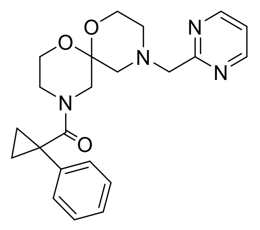

L1-C14-A06
77% yield
98% purity

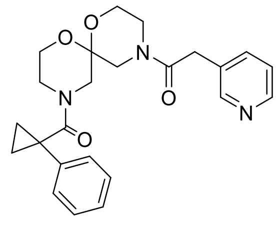

L1-C14-C39
69% yield
98% purity

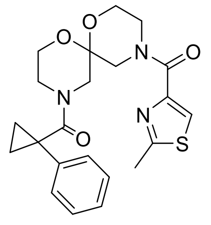

L1-C14-C42
75% yield
99% purity

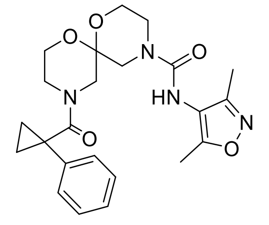

L1-C14-I08
74% yield
98% purity

Figure S4: Library compounds synthesized from amide scaffold L1-C14.

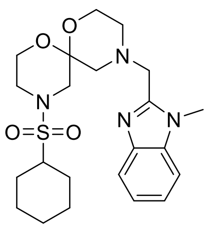

L1-B05-A10
69% yield
94% purity

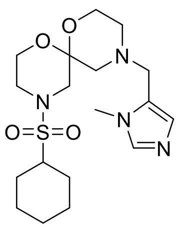

L1-B05-A01
54% yield
98% purity

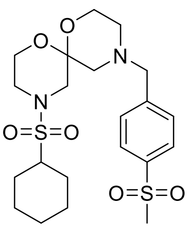

L1-B05-A07
69% yield
96% purity

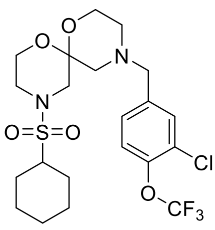

L1-B05-A09
53% yield
94% purity

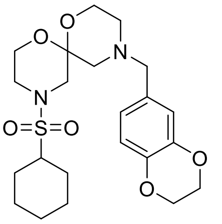

L1-B05-A13
59% yield
92% purity

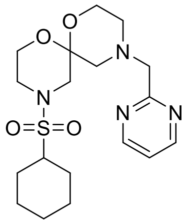

L1-B05-A06
65% yield
98% purity

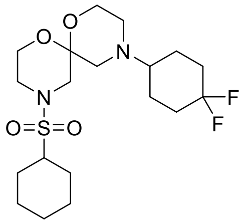

L1-B05-K01
58% yield
100% purity

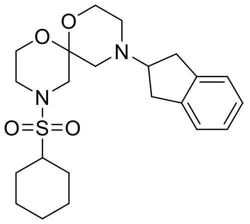

L1-B05-K02
75% yield
95% purity

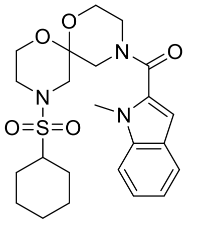

L1-B05-C46
74% yield
98% purity

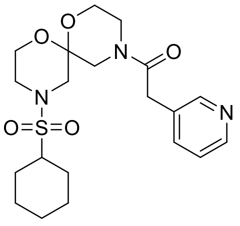

L1-B05-C39
60% yield
93% purity

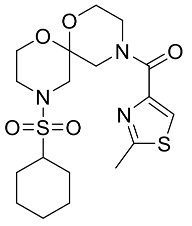

L1-B05-C42
71% yield
99% purity

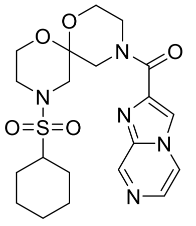

L1-B05-C45
21% yield
94% purity

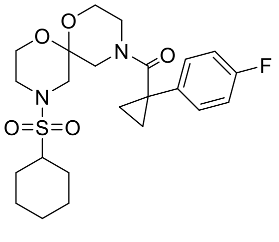

L1-B05-C34
41% yield
100% purity

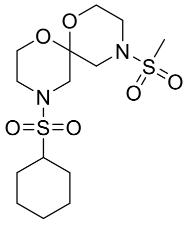

L1-B05-B09
reaction failed

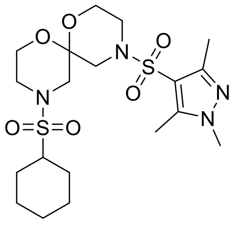

L1-B05-B10
65% yield
98% purity

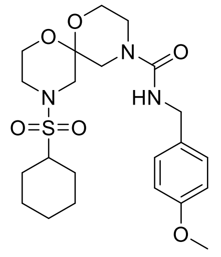

L1-B05-I11
69% yield
90% purity

Figure S5: Library compounds synthesized from sulfonamide scaffold L1-B05.

After the success of the feasibility study, the remaining 10 singly decorated scaffolds were synthesized on multigram scale. The amidation, sulfonylation and urea formation reactions were all performed on amine **9** under standard conditions to afford the corresponding Boc-protected scaffolds in good yields. Subsequent treatment with 4 M HCl in 1,4-dioxane afforded the singly decorated products as their hydrochloride salts **L1-R^1·HCl** (Scheme S23).

Scheme S23. First decoration of bis-morpholine spiroacetal scaffold 9

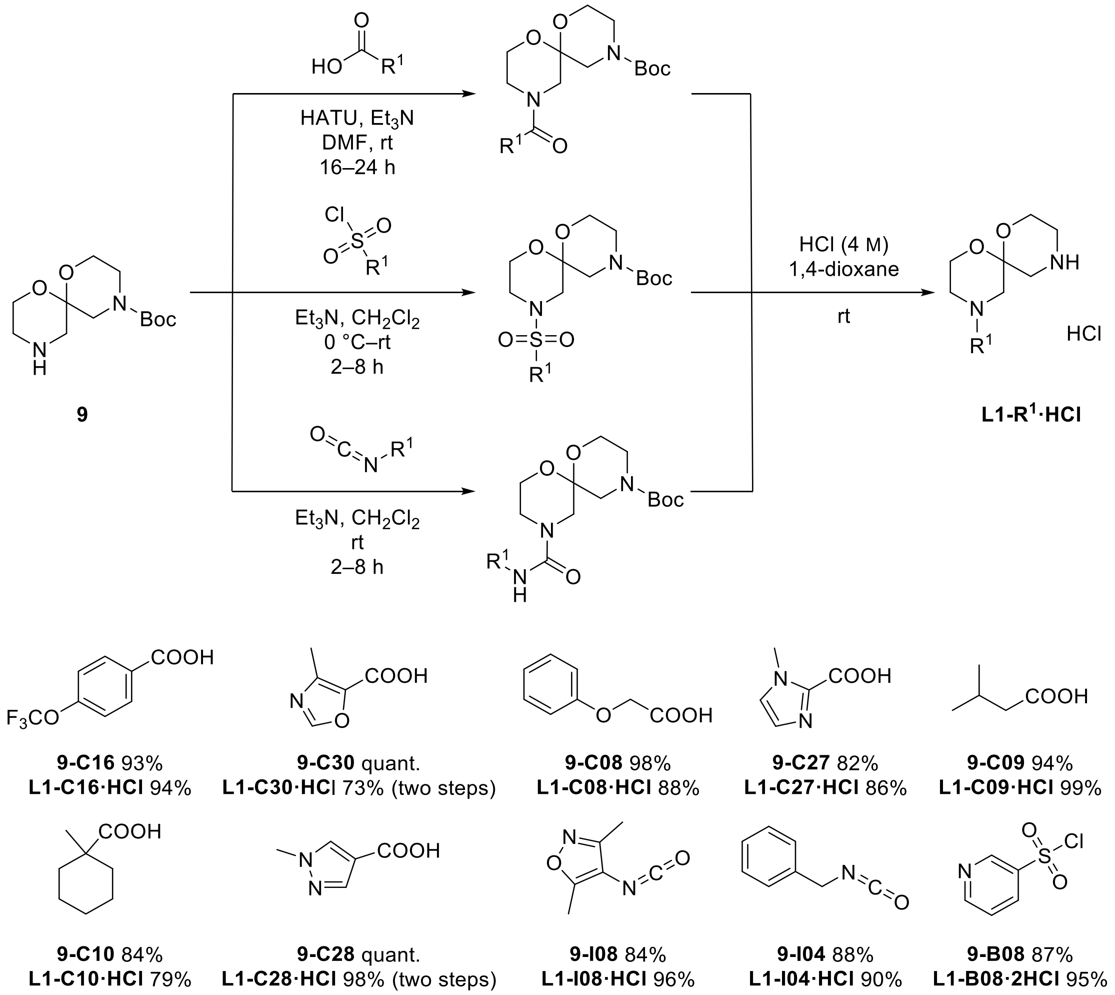

137 compounds from our target of 141 were successfully synthesized. Of the four compounds that we were unable to access, one reaction (a mesylation) failed and the required decorating reagents for the other three were not available. Of the 137 compounds that were synthesized, 13, all reductive amination products, were isolated in <10 mg (<10% isolated yield). We postulate the reason behind their low yield is problematic purification, rather than efficiency of the reaction, given the consumption of the starting material was confirmed by TLC in every case. The yields for the remaining 111 reactions are summarized in Table S7 and a summary of the reagents used, product purity and isolated yields of

each library compound can be found in Table S9, p 156. In summary, 138 reactions were performed in our library synthesis. 124 compounds were successfully synthesized and isolated; this result translates to a 90% success rate, which confirmed bis-morpholine **9** as a valid starting point for compound library assembly.

Table S7: Library synthesis summary

reaction type	average isolated yield (%)	max. isolated yield (%)	min. isolated yield (%)	number of examples
amidation	61	90	16	45
reductive amination	57	99	15	48
sulfonylation	61	91	35	12
urea formation	64	74	28	6

Physical Compound Library Synthesis using 6,7-spiroacetal scaffold 25

Guided by our synthesis of the bis-morpholine spiroacetal library, three singly decorated analogues were chosen to validate the synthesis of a compound library from 6,7-spiroacetal scaffold **25**, namely amide **L2-C30**, sulfonamide **L2-B08·2HCl** and urea **L2-I03·HCl**. Their synthesis is discussed in the main article and summarized in Scheme S24.

Scheme S24. Synthesis of singly decorated 6,7-spiroacetal scaffolds

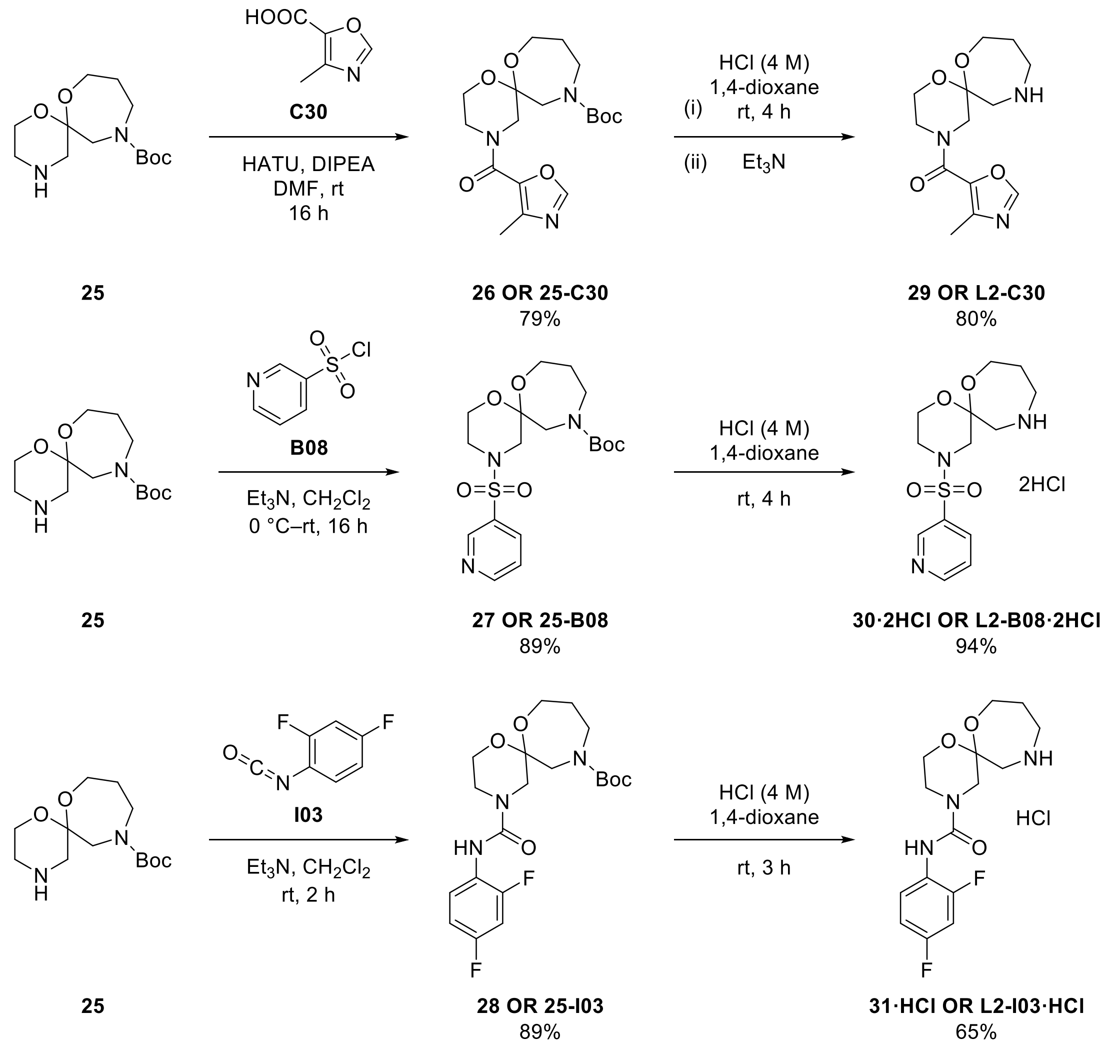

20 reagents from the 42 [41 + skip (i.e., the monodecorated scaffold **L2-C30, L2-B08** and **L2-I03**)] that were used to generate the virtual library, were selected for the second decoration step to deliver 11 library compounds from each singly decorated scaffold. Assuming a 100% success rate, this would generate 33 compounds in total. The selection of the 20 reagents was as follows: each of the three singly decorated scaffolds (**L2-C30**, **L2-B08** and **L2-I03**) added 42 enumerated compounds to the 6,7-

spiroacetal virtual library. From each of these, 10 clusters of compounds were generated using the DW-Cluster-SkelSphereFP method and representative compounds were selected from each cluster. Analysis of the resulting compound selection revealed almost the same 10 groups had been used in the second decoration step on all three singly decorated scaffolds. To generate a more diverse set of compounds, the initially selected 10 decorating groups were applied to the urea scaffold **L2-I03**. For the sulfonamide scaffold **L2-B08**, the last 5 cluster representatives were deleted from the 10 representatives and the remaining 37 compounds were clustered again into 10 clusters; representative compounds were selected from each cluster. Now, only three were the same as those used to decorate urea **L2-I03**. Similarly, for amide scaffold **L2-C30**, the first 5 cluster representatives were removed and the remaining 37 compounds clustered again into 10 clusters from which 10 representatives were selected. Three of these compounds had the same second decorations as urea scaffold **L2-I03** and five were the same as sulfonamide scaffold **L2-B08**. From all of these common decorating groups (3+3+5), none were present on all three scaffolds. In conclusion, 19 different reagents were selected for the second decoration step, of which 11 were used on two scaffolds and eight on one scaffold. An additional reagent was selected based on an *in silico* study that will not be discussed here, resulting in 20 reagents in total and 33 compounds for synthesis (list of reagents Figure S12, p 165).

The same methods were used for these decoration steps as had been used to prepare the bis-morpholine spiroacetal library from scaffold **9**, except for the reductive amination. For this transformation, instead of using a suspension of $NaBH(OAc)_3$ in 1,2-dichloroethane, solid reducing agent was added to the reaction mixtures in CH_2Cl_2 (Scheme S25).

Scheme S25. General R^2 decoration conditions for the 6,7-spiroacetal

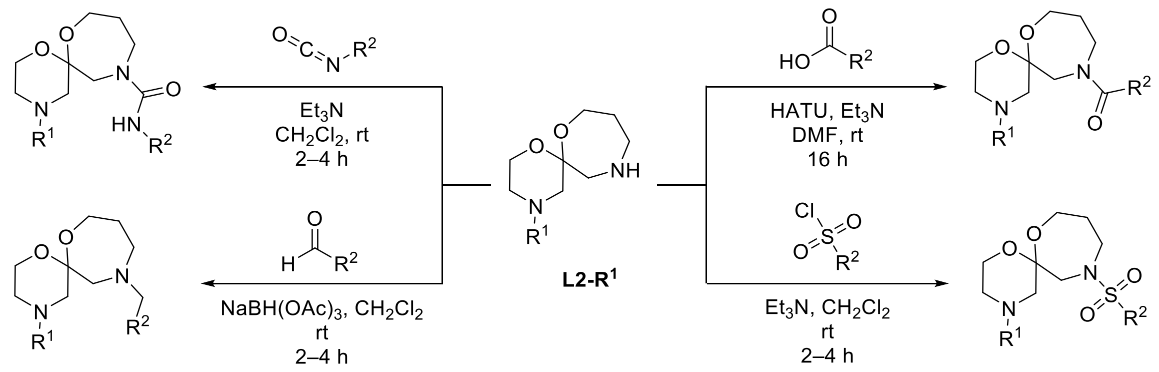

Of the 33 reactions, only one reaction failed, a urea formation on spiroacetal **L2-I03**. The average isolated product yield from the successful reactions was 58%, ranging between 31–84%, similar to

those reactions used to prepare the 6,6-spiroacetal library. The products were purified by preparative HPLC and their purity measured by LCMS and ^{1}H-NMR spectroscopy, before transfer to AnalytiCon's compound repository. The reagents used, product purity and isolated product yields of all compounds are summarized in Table S10, p 163.

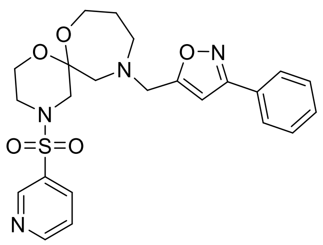

L2-B08-A05
54% yield
100% purity

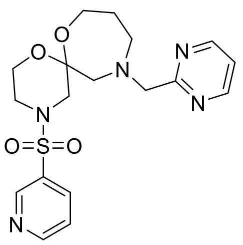

L2-B08-A06
26% yield
98% purity

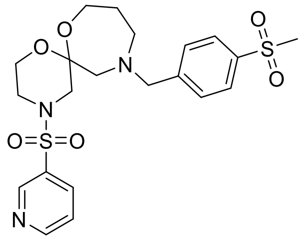

L2-B08-A07
31% yield
94% purity

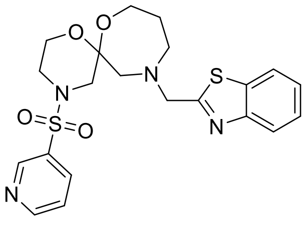

L2-B08-A12
60% yield
94% purity

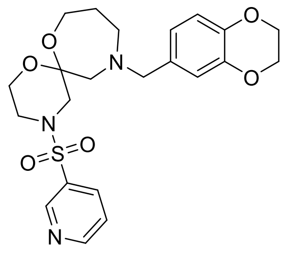

L2-B08-A13
53% yield
96% purity

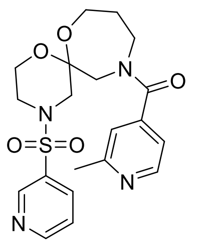

L2-B08-C38
59% yield
100% purity

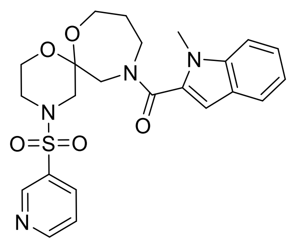

L2-B08-C46
28% yield
97% purity

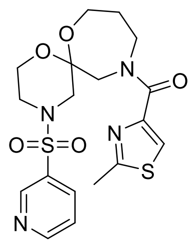

L2-B08-C42
59% yield
100% purity

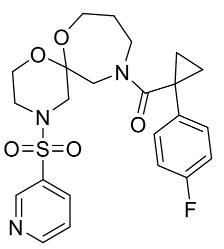

L2-B08-C34
51% yield
97% purity

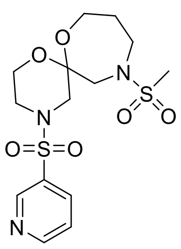

L2-B08-B09
49% yield
100% purity

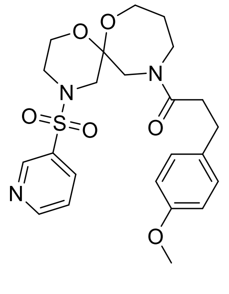

L2-B08-I11
62% yield
95% purity

Figure S6: Library compounds synthesized from sulfonamide scaffold L2-B08.

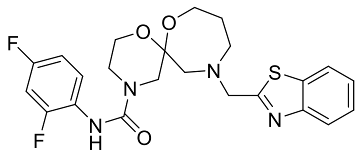

L2-I03-A12
53% yield
99% purity

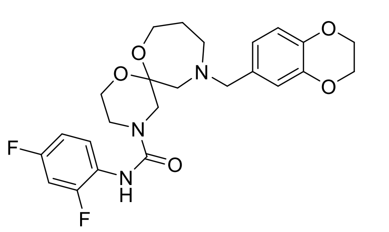

L2-I03-A13
59% yield
98% purity

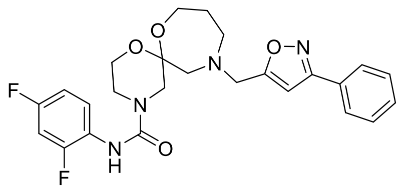

L2-I03-A05
59% yield
98% purity

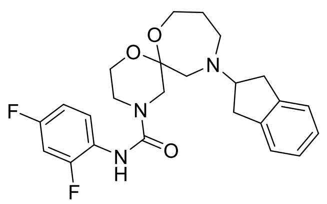

L2-I03-K02
53% yield
100% purity

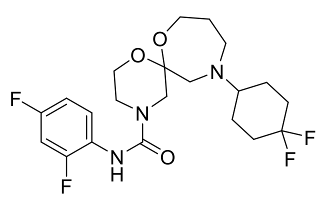

L2-I03-K01
55% yield
100% purity

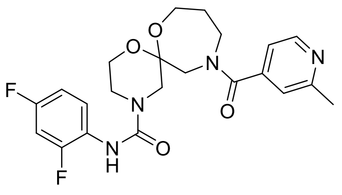

L2-I03-C38
55% yield
100% purity

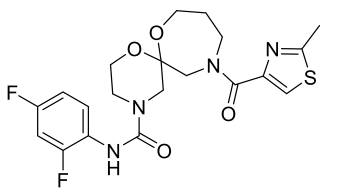

L2-I03-C42
56% yield
100% purity

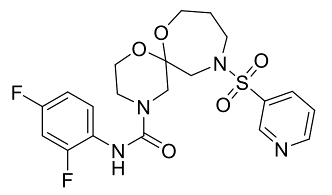

L2-I03-B08
33% yield
95% purity

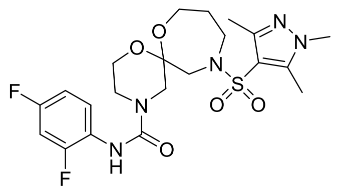

L2-I03-B10
64% yield
100% purity

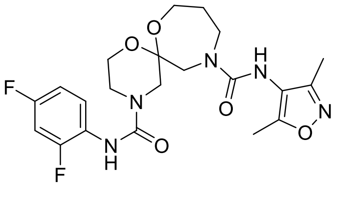

L2-I03-I08
64% yield
87% purity

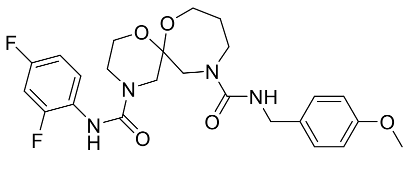

L2-I03-I11
reaction failed

Figure S7: Library compounds synthesized from urea scaffold L2-I03.

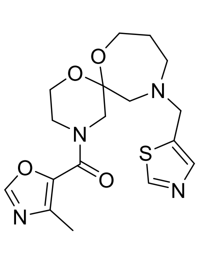

L2-C30-A04
43% yield
97% purity

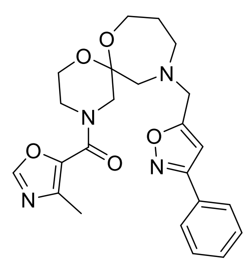

L2-C30-A05
46% yield
98% purity

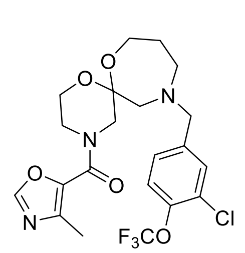

L2-C30-A09
44% yield
98% purity

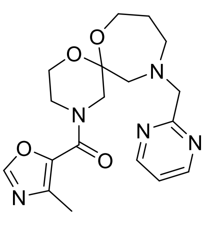

L2-C30-A06
30% yield
100% purity

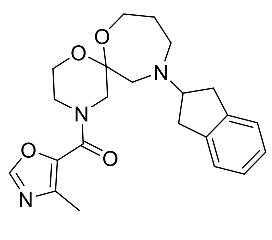

L2-C30-K02
44% yield
99% purity

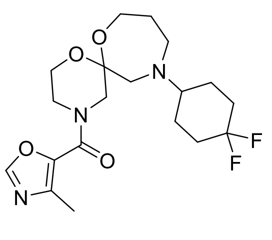

L2-C30-K01
43% yield
99% purity

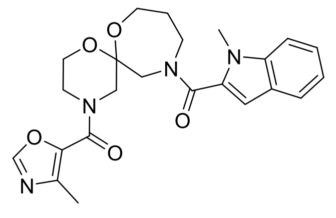

L2-C30-C46
51% yield
96% purity

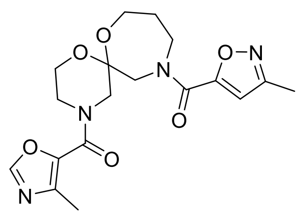

L2-C30-C40
45% yield
98% purity

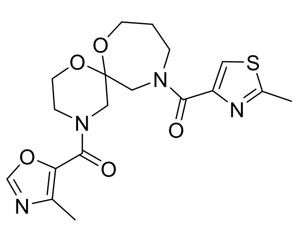

L2-C30-C42
27% yield
95% purity

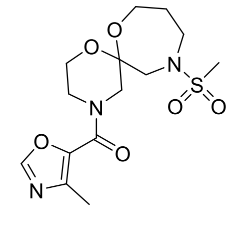

L2-C30-B09
33% yield
98% purity

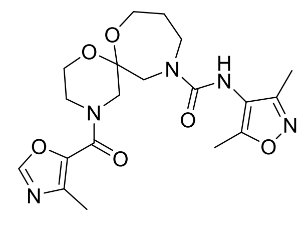

L2-C30-I08
46% yield
99% purity

Figure S8: Library compounds synthesized from amide scaffold L2-C30.

3.2 Library synthesis results

Table S8: Feasibility study results for the library derived from bis-morpholine spiroacetal 9.

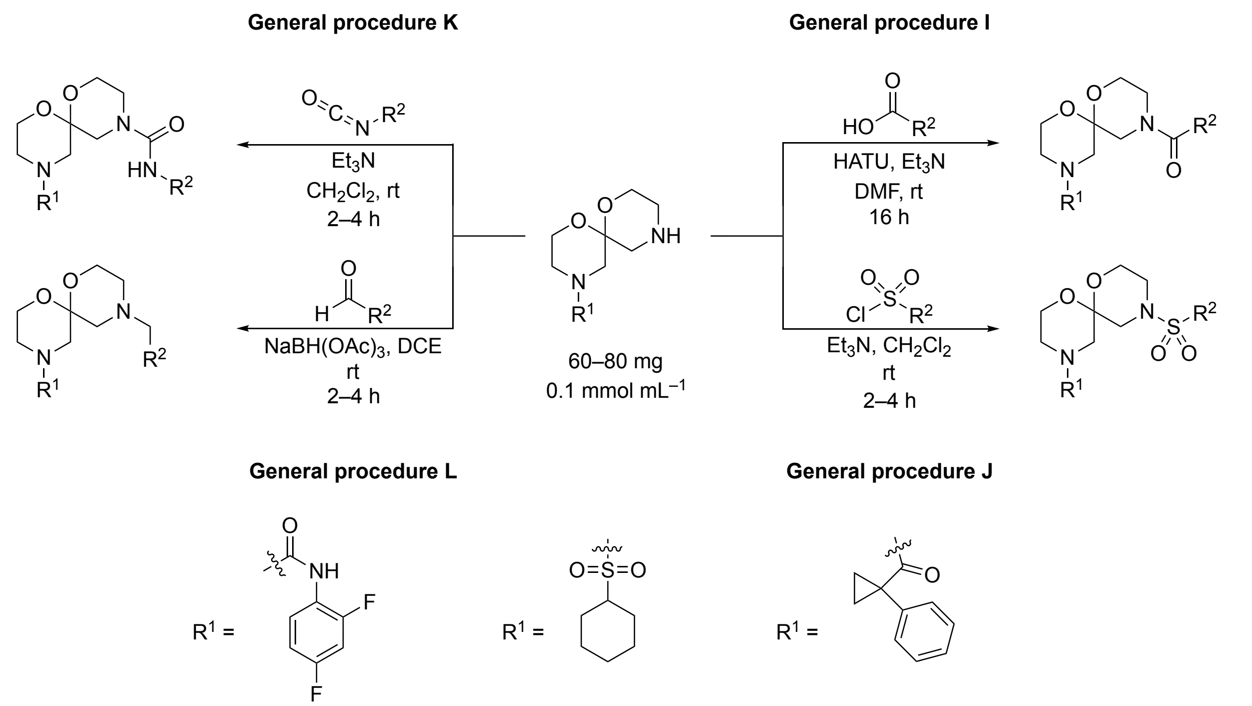

I03 B05 C14

compound[a]	method	MW (g mol −1)	purity (%)[b]	quantity (mg)	isolated yield (%)	retention time (min)	HPLC method[c]
**L1-C14**	-	302.37	98	35.6	66	4.13	N
**L1-I03**	-	313.31	98	29.8	60	3.31	N
**L1-B05**	-	304.41	90	17.6	33	3.99	N
**L1-C14-A01**	L	396.49	97	50.0	71	4.54	B
**L1-C14-A06**	L	394.48	98	53.8	77	4.41	B
**L1-I03-A06**	L	405.41	100	41.2	64	3.86	B
**L1-B05-A10**	L	448.58	94	54.1	69	5.21	B
**L1-B05-A01**	L	398.52	98	50.9	54	4.49	B
**L1-B05-A07**	L	472.62	96	57.8	69	4.70	B
**L1-B05-A09**	L	512.97	94	48.2	53	5.95	B

**L1-B05-A05**	L	461.58	93	53.0	65	5.38	B
**L1-B05-A13**	L	452.57	92	47.0	59	5.40	B
**L1-B05-A06**	L	396.51	98	60.8	65	4.37	B
**L1-I03-K01**	L	431.43	100	37.6	55	4.85	B
**L1-I03-K02**	L	429.47	98	42.6	62	5.24	B
**L1-B05-K01**	L	422.53	100	57.1	58	5.26	B
**L1-B05-K02**	L	420.57	95	55.5	75	5.61	B
**L1-C14-C39**	I	421.50	98	51.5	69	4.37	N
**L1-C14-C42**	I	427.52	99	75.8	75	4.62	N
**L1-I03-C39**	I	432.43	99	65.8	68	3.83	N
**L1-I03-C42**	I	438.45	100	44.0	63	4.09	N
**L1-B05-C46**	I	461.58	98	59.8	74	5.33	N
**L1-B05-C39**	I	423.53	93	44.4	60	4.29	N
**L1-B05-C42**	I	429.55	99	71.3	71	4.55	N
**L1-B05-C45**	I	449.53	94	16.2	21	4.26	N
**L1-B05-C34**	I	466.57	100	34.0	41	5.28	N
**L1-C14-I08**	K	440.50	98	57.4	74	4.40	N
**L1-I03-I03**	K	468.41	100	54.6	73	4.48	N
**L1-B05-I11**	K	467.58	90	56.8	69	4.88	N
**L1-B05-B09**	J	382.49			reaction failed		
**L1-B05-B10**	J	476.61	98	54.3	65	4.61	N

[a]A = aldehyde, K = ketone, C = carboxylic acid, I = isocyanate, S = sulfonyl chloride (the R^2 decorating reagents are listed in Figure S9). [b]Purity was measured by the percentage of total UV absorbance between 320 and 220 nm. [c]N = neutral preparative HPLC method, B = basic preparative HPLC method (General experimental 2.1, p 24).

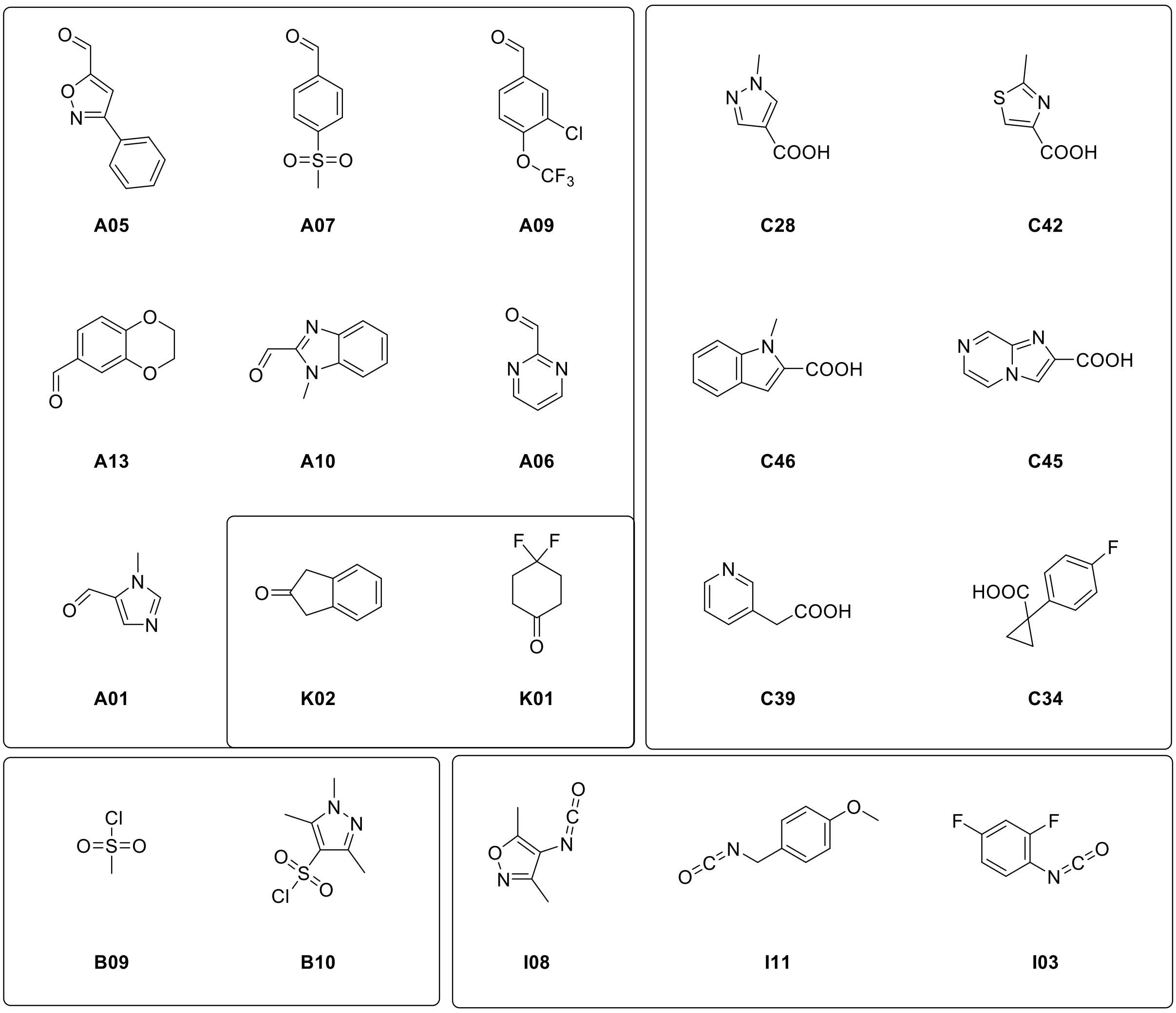

Figure S9: R^2 decorating reagents used for the feasibility study on 6,6-spiroacetal scaffold 9.

Table S9: Library compounds derived from 6,6-spiroacetal 9.

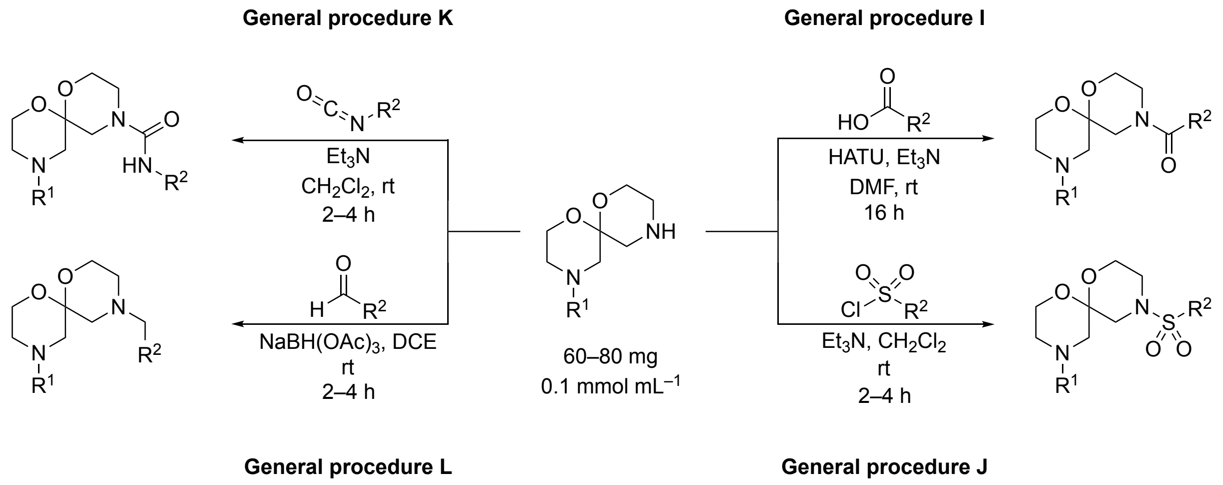

compound[a]	method	MW (g mol $^{-1}$)	purity (%)[b]	quantity (mg)	isolated yield (%)	retention time (min)	HPLC method[c]
**L1-C28**	-	266.30	100	39.9	46	2.11	N
**L1-C16**	-	346.31	100	79.3	87	4.40	N
**L1-I04**	-	291.35	99	51.1	51	3.35	N
**L1-C30**	-	267.29	91	39.3	66	3.62	N
**L1-C27**	-	266.30	98	69.3	66	2.24	N
**L1-B08**	-	299.35	100	24.0	22	2.63	N
**L1-I08**	-	296.33	99	93.7	94	3.17	N
**L1-C08**	-	292.34	100	61.4	69	4.11	N
**L1-C08**	-	292.34	100	61.4	69	3.54	N
**L1-C10**	-	282.38	96	89.7	90	4.52	N
**L1-C28-A01**	L	360.42	96	9.2	13	3.04	B
**L1-C28-A06**	L	358.40	90	0.7	1	2.88	B
**L1-C28-A02**	L	360.42	99	1.9	3	3.11	B

**L1-C28-A04**	L	363.44	100	5.3	7	3.10	B
**L1-C28-A08**	L	387.44	100	8.8	12	3.65	B
**L1-C09-A01**	L	336.44	98	7.1	10	4.01	B
**L1-C09-A06**	L	334.42	99	17.5	24	3.86	B
**L1-C16-A01**	L	440.42	99	38.2	55	4.75	B
**L1-C16-A13**	L	494.47	100	57.9	75	5.50	B
**L1-I04-A07**	L	459.56	100	56.7	60	4.25	B
**L1-I04-A09**	L	499.92	100	15.5	15	5.67	B
**L1-I04-A05**	L	448.52	100	60.0	65	5.02	B
**L1-I04-A13**	L	439.51	97	47.6	53	4.95	B
**L1-I04-A03**	L	400.48	99	38.5	47	4.26	B
**L1-I04-A08**	L	412.49	99	46.8	41	4.48	B
**L1-I04-A11**	L	421.50	76	33.6	39	4.70	B
**L1-C30-A01**	L	361.40	95	4.0	5	3.22	B
**L1-C30-A07**	L	435.50	85	19.9	20	3.60	B
**L1-C30-A05**	L	424.46	99	37.8	40	4.53	B
**L1-C30-A06**	L	359.39	98	6.5	8	3.07	B
**L1-C27-A10**	L	410.48	98	84.5	83	4.22	B
**L1-C27-A09**	L	474.87	99	121.9	99	5.28	B
**L1-C27-A13**	L	414.46	94	89.6	87	4.37	B
**L1-C27-A03**	L	375.43	98	47.6	51	3.50	B
**L1-C27-A11**	L	396.45	80	7.6	8	4.02	B
**L1-C27-A12**	L	413.50	93	64.3	63	4.48	B
**L1-B08-A01**	L	393.46	96	5.4	6	3.48	B

**L1-B08-A06**	L	391.45	100	24.5	28	3.31	B
**L1-I08-A01**	L	390.44	91	0.4	1	3.14	B
**L1-I08-A09**	L	504.89	99	51.8	51	5.31	B
**L1-I08-A06**	L	388.43	90	1.2	2	3.01	B
**L1-C08-A10**	L	436.51	98	57.2	72	4.92	B
**L1-C08-A01**	L	386.45	94	6.5	9	4.15	B
**L1-C10-A01**	L	376.50	100	38.0	48	4.87	B
**L1-C10-A07**	L	450.59	98	60.2	63	4.99	B
**L1-C10-A09**	L	490.95	94	53.9	52	6.08	B
**L1-C10-A06**	L	374.49	99	53.5	67	4.76	B
**L1-C10-A12**	L	429.58	90	50.0	55	5.66	B
**L1-C28-C28**	I	374.40	99	17.6	24	2.71	N
**L1-C28-C39**	I	385.42	97	21.3	28	2.94	N
**L1-C09-C31**	I	334.36	90	42.2	59	4.35	N
**L1-C09-C39**	I	361.44	99	55.8	72	3.79	N
**L1-C09-C42**	I	367.46	100	61.1	77	4.06	N
**L1-C16-C39**	I	465.43	100	61.2	84	4.55	N
**L1-C16-C42**	I	471.45	100	66.4	90	4.77	N
**L1-I04-C31**	I	383.40	86	41.8	53	4.35	N
**L1-I04-C32**	I	411.47	99	48.1	57	3.30	N
**L1-I04-C46**	I	448.52	100	60.4	65	5.01	N
**L1-I04-C37**	I	399.45	77	19.0	23	3.45	N
**L1-I04-C41**	I	410.47	92	19.6	17	3.97	N
**L1-I04-C45**	I	436.47	95	30.7	34	3.76	N

**L1-I04-C34**	I	453.51	98	47.5	51	4.94	N
**L1-C30-C28**	I	375.39	94	30.0	36	2.88	N
**L1-C30-C46**	I	424.46	98	53.4	56	4.52	N
**L1-C30-C39**	I	386.41	95	17.0	20	3.12	N
**L1-C30-C42**	I	392.43	96	54.4	62	3.36	N
**L1-C30-C45**	I	412.41	78	16.8	18	3.04	N
**L1-C30-C34**	I	429.45	97	57.9	60	4.43	N
**L1-C27-C31**	I	358.35	75	22.3	25	3.53	N
**L1-C27-C32**	I	386.42	99	33.5	35	2.35	N
**L1-C27-C33**	I	414.46	99	73.9	72	4.07	N
**L1-C27-C36**	I	434.88	99	73.8	68	4.15	N
**L1-C27-C37**	I	374.40	60	15.3	16	2.58	N
**L1-C27-C42**	I	391.45	97	71.2	73	3.27	N
**L1-C27-C35**	I	420.42	100	68.0	65	3.97	N
**L1-B08-C39**	I	418.47	100	79.1	64	3.32	N
**L1-B08-C42**	I	424.49	100	58.9	63	3.56	N
**L1-I08-C42**	I	421.47	94	64.7	76	3.31	N
**L1-C08-C26**	I	411.46	99	45.6	61	4.03	N
**L1-C08-C42**	I	417.48	100	57.4	75	4.19	N
**L1-C08-C35**	I	446.45	100	46.1	57	4.68	N
**L1-C10-C31**	I	374.43	96	45.2	57	5.08	N
**L1-C10-C42**	I	407.53	100	59.1	68	4.87	N
**L1-C27-I11**	K	429.48	90	62.7	59	4.42	N
**L1-I08-I08**	K	434.45	98	56.4	48	2.93	N

**L1-C08-I11**	K	455.51	83	30.8	28	4.55	N
**L1-C16-K01**	L	464.43	100	57.2	78	5.37	B
**L1-C27-K01**	L	384.43	97	18.4	19	4.13	B
**L1-C27-K02**	L	382.46	92	92.0	97	4.67	B
**L1-B08-K01**	L	417.47	100	27.8	23	4.39	B
**L1-I08-K02**	L	412.49	95	21.3	26	4.67	B
**L1-C08-K01**	L	410.46	100	50.5	67	4.94	B
**L1-C08-K02**	L	408.50	96	37.0	50	5.31	B
**L1-C10-K01**	L	400.51	100	41.9	49	5.50	B
**L1-C10-K02**	L	398.55	90	57.9	68	5.82	B
**L1-C09-B09**	J	320.40	100	54.2	78	3.42	N
**L1-C16-B10**	J	518.51	100	74.0	91	4.81	N
**L1-I04-B09**	J	369.44	99	41.7	55	3.52	N
**L1-I04-B10**	J	463.55	99	69.2	72	4.22	N
**L1-C30-B10**	J	439.49	94	60.1	61	3.56	N
**L1-C27-B09**	J	344.39	96	52.4	61	2.53	N
**L1-B08-B09**	J	377.43	96	36.6	44	2.88	N
**L1-I08-B09**	J	374.41	99	35.0	35	2.54	N
**L1-C10-B09**	J	360.47	99	54.3	71	4.46	N
**L1-C10-B10**	J	454.59	100	45.4	35	4.90	N

[a]A = aldehyde, K = ketone, C = carboxylic acid, I = isocyanate, B = sulfonyl chloride (the R^1 and R^2 reagents are listed in Figure S10 and Figure S11). [b]Purity was measured by the percentage of total UV absorbance between 320 and 220 nm. [c]N = neutral preparative HPLC method, B = basic preparative HPLC method (General experimental 2.1, p 24).

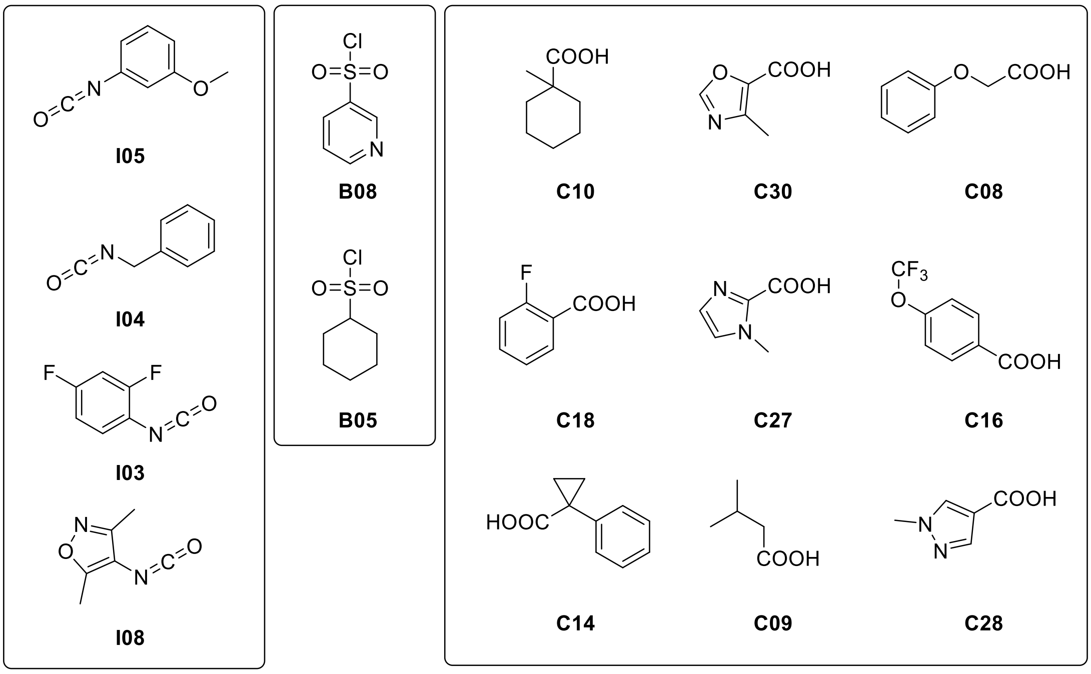

Figure S10: R[1] reagents (4 isocyanates, 2 sulfonyl chlorides and 9 carboxylic acids) selected as first decorating reagents, for the singly decorated scaffolds, that were used in further library enumeration.

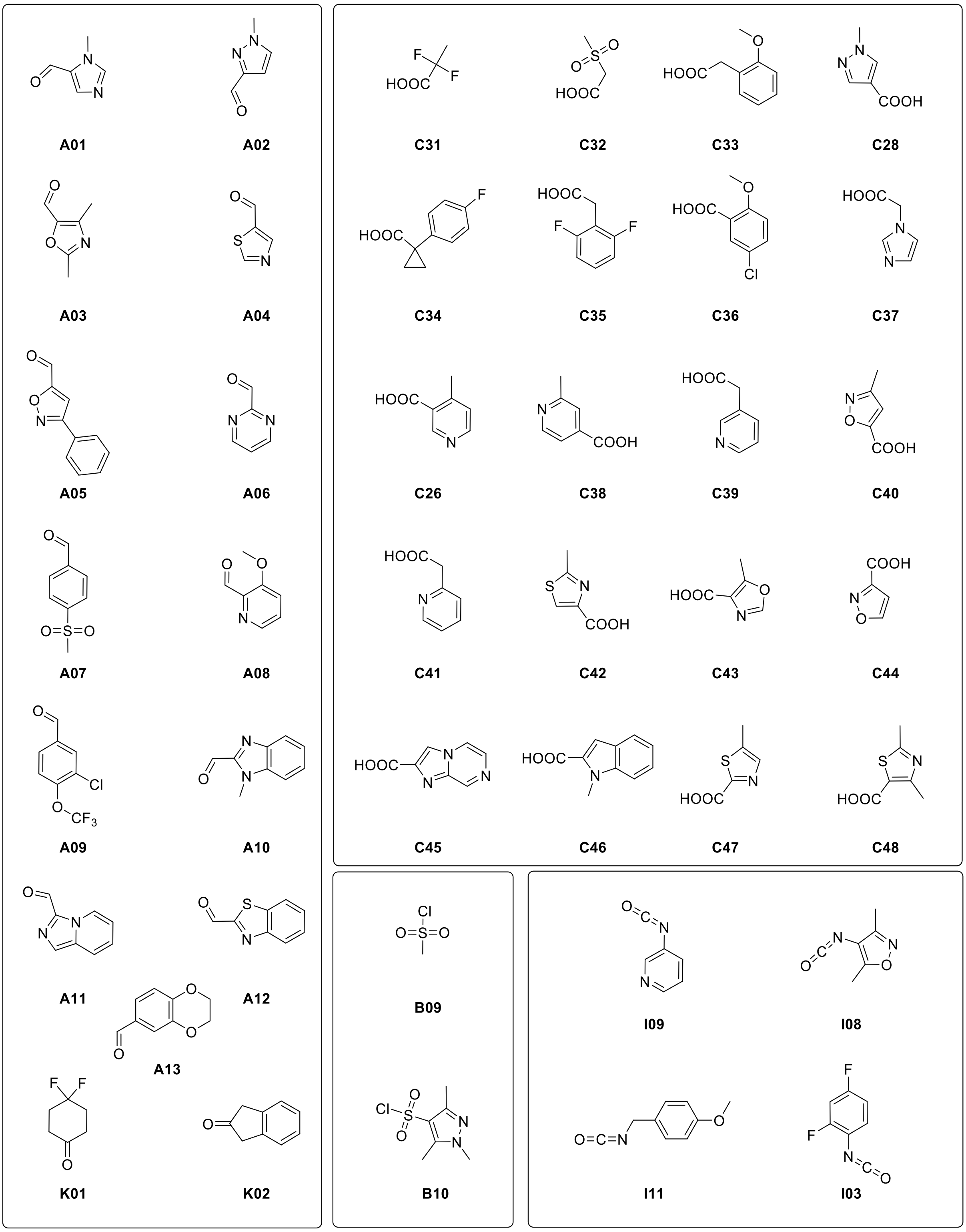

Figure S11: R^2 reagents (13 aldehydes, 4 isocyanates, 20 carboxylic acids, 2 ketones and 2 sulfonyl chlorides) used for the enumeration in the second derivatisation point of the virtual library.

Table S10: Feasibility study results for the library derived from 6,7-spiroacetal 25.

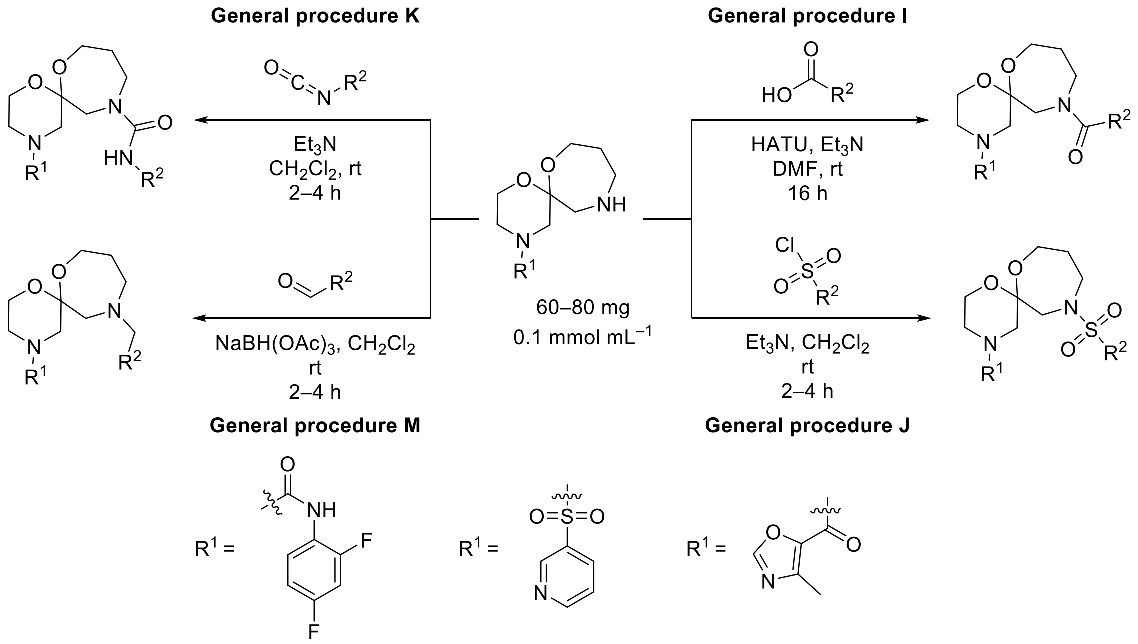

I03 B08 C30

compound[a]	method	MW (g mol $^{-1}$)	purity (%)[b]	quantity (mg)	isolated yield (%)	retention time (min)	HPLC method[c]
**L2-B08-A06**	M	405.47	98	1.61	26.0	31	B
**L2-B08-A13**	M	461.53	96	2.02	53.6	55	B
**L2-B08-A07**	M	481.58	94	2.06	30.9	31	B
**L2-B08-A12**	M	460.57	94	2.94	59.5	61	B
**L2-B08-A05**	M	470.54	100	2.97	53.6	54	B
**L2-C30-A04**	M	378.45	97	1.68	43.3	54	B
**L2-C30-A06**	M	373.41	100	1.45	29.6	38	B
**L2-C30-A05**	M	438.48	98	2.80	45.9	50	B
**L2-C30-A09**	M	489.88	98	3.12	44.0	43	B
**L2-I03-A13**	M	475.49	98	2.21	58.6	68	B
**L2-I03-A12**	M	474.53	99	3.18	53.4	62	B

**L2-I03-A05**	M	484.50	98	3.14	59.3	68	B
**L2-B08-C38**	I	432.50	100	1.80	58.7	65	N
**L2-B08-C46**	I	470.54	97	2.46	28.1	28	N
**L2-B08-C42**	I	438.52	100	2.08	59.2	64	N
**L2-B08-C34**	I	475.54	97	2.45	50.8	51	N
**L2-C30-C46**	I	438.48	96	2.38	50.5	55	N
**L2-C30-C41**	I	400.44	95	1.61	26.5	32	N
**L2-C30-C40**	I	390.40	98	1.94	44.6	54	N
**L2-I03-C38**	I	446.46	100	2.02	54.5	68	N
**L2-I03-C42**	I	452.48	100	2.30	56.0	69	N
**L2-B08-I11**	K	476.55	95	2.22	61.6	62	N
**L2-C30-I08**	K	419.44	99	1.81	46.0	52	N
**L2-I03-I08**	K	465.46	87	2.15	64.3	77	N
**L2-I03-I11**	K	490.51			reaction failed		
**L2-C30-K01**	M	399.44	99	1.71	42.5	51	B
**L2-C30-K02**	M	397.48	99	1.92	43.6	52	B
**L2-I03-K01**	M	445.46	100	2.11	55.1	69	B
**L2-I03-K02**	M	443.50	100	2.33	52.8	66	B
**L2-B08-B09**	J	391.46	100	1.86	49.3	60	N
**L2-C30-B09**	J	359.40	98	1.74	33.4	44	N
**L2-I03-B08**	J	468.48	95	2.26	32.9	39	N
**L2-I03-B10**	J	499.53	100	2.33	63.5	71	N

[a]A = aldehyde, K = ketone, C = carboxylic acid, I = isocyanate, B = sulfonyl chloride (the R^2 decorating reagents are listed in Figure S12). [b]Purity was measured by the percentage of total UV absorbance between 320 and 220 nm. [c]N = neutral preparative HPLC method, B = basic preparative HPLC method (General experimental 2.1, p 24).

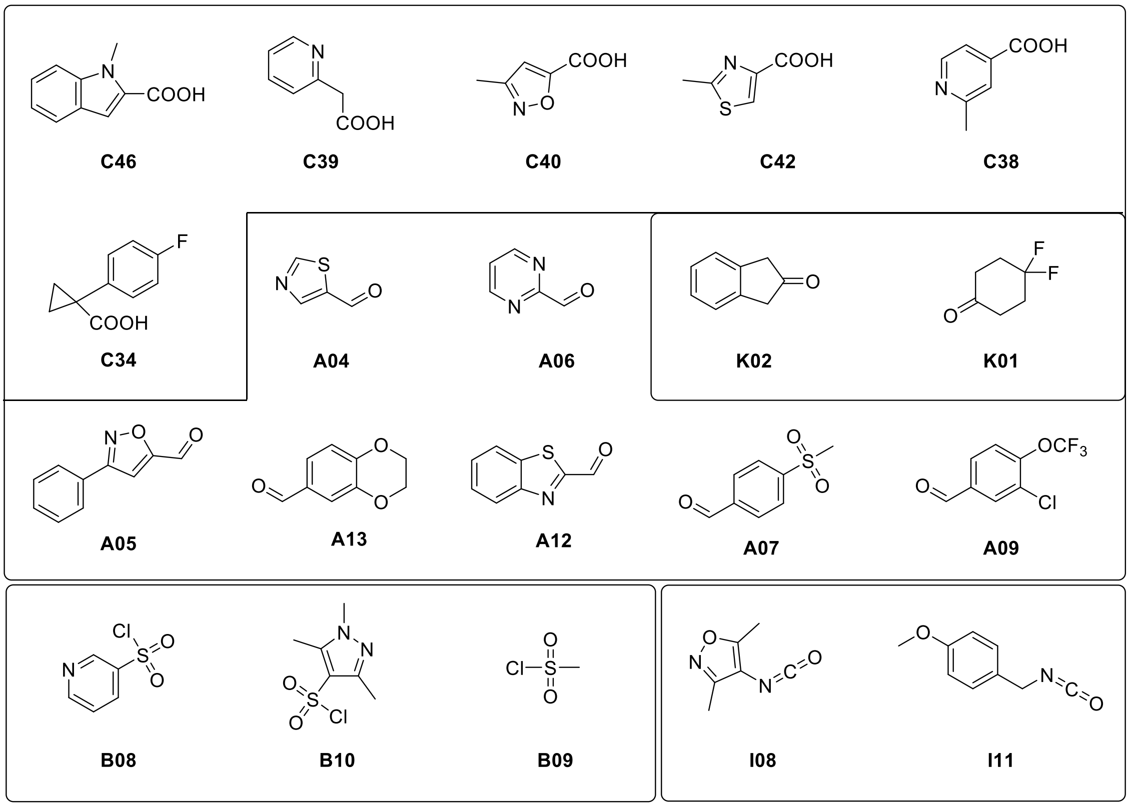

Figure S12: R^2 decorating reagents used for the 6,7-spiroacetal library feasibility study.

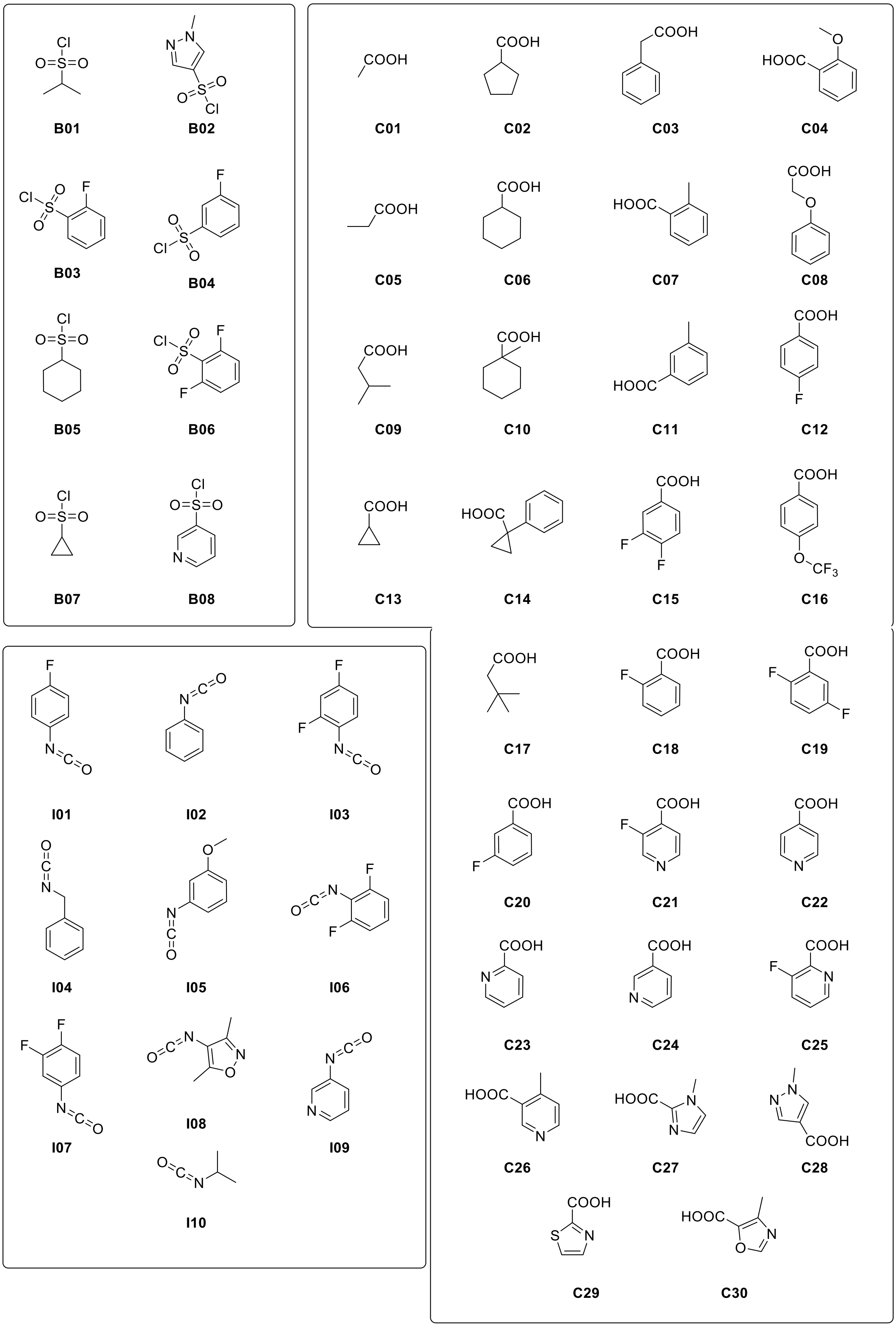

Figure S13: R[1] reagents (8 sulfonyl chlorides, 10 isocyanates, and 30 carboxylic acids used for the first decoration of the scaffold, from which 15 were selected for further library enumeration).

3.3 Library comparisons with FDA-approved drugs

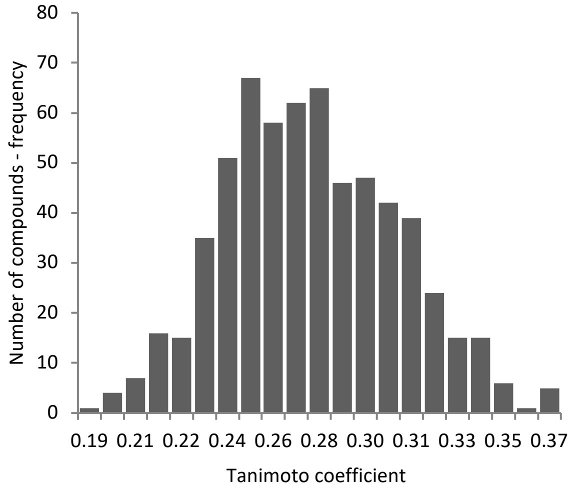

Figure S14: Histogram of the Tanimoto coefficients calculated using Morgan 2 fingerprints, comparing the bis-morpholine spiroacetal virtual library with the FDA-approved small-molecule drugs.

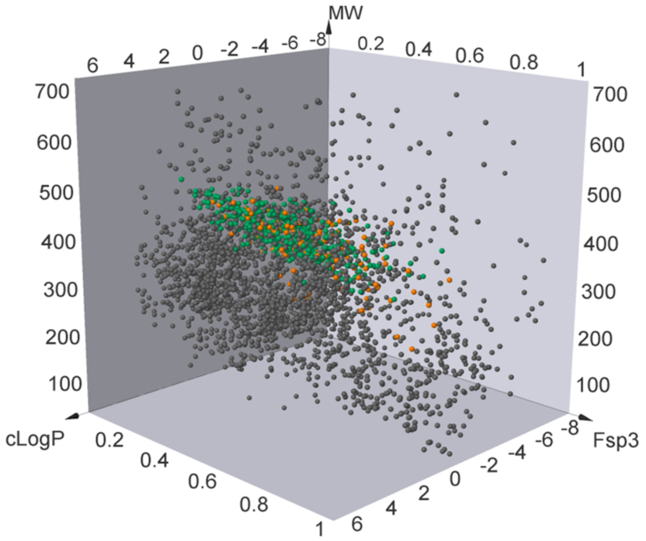

Figure S15: Comparison of bis-morpholine spiroacetal compound library (n = 124) with the virtual library (n = 630) and FDA-approved drugs[z] (n = 2709), visualized in cLogP / MW / Fsp^3 space [FDA-approved drugs (black), physical bis-morpholine spiroacetal library (orange), virtual bis-morpholine spiroacetal library (green)].

[z] This filtering of the FDA collection removed biologics and other macromolecular therapeutics, which frequently have different modes of administration (H-bond donors <5, H-bond acceptors <10, cLogP <5.0, MW <700). The collection was exported from CDD vault on 05.05.2022 based on the following public collections “FDA Approved: Tox”, “FDA Approved: Approved drugs” and “FDA Approved: Orphan drugs”. Further information about these collections can be found at: https://www.collaborativedrug.com/public-access/.

3.4 KNIME

Unless stated otherwise, all calculations and enumerations were performed in the KNIME analytics platform version 4.1.2. Copyright by KNIME AG, Zürich, Switzerland. This version of KNIME is licensed under the GNU General Public License, Version 3. More details can be found at http://www.knime.com

The following node packages were used in KNIME:

KNIME base nodes: These nodes are included within the software installation. Base nodes include "Column Rename" (renames column headings or changes their data types, for example converting an integer number to a string value), "Reference Row Filter" (filters rows from a table using another table as reference) and "Concatenate" (concatenates tables). The full list of base nodes and their individual descriptions can be found at: https://nodepit.com/iu/org.knime.base

KNIME chemistry base nodes: These are chemistry-related base nodes, and include "SDF Writer" (writes sdf or Mol cells into a continuous sdf file) and "SDF Reader" (reads an sdf file and creates several columns with each molecule in a new row). The full list of chemistry base nodes and their individual descriptions can be found at: https://nodepit.com/iu/org.knime.chem.base

RDKit node package: RDKit is an open-source cheminformatics toolkit, which includes "RDKit Diversity Picker" (picks diverse rows from an input table based on the Tanimoto distance between the used fingerprints; the picking is performed using the MaxMin algorithm) and "RDKit Functional Group Filter" (filters sets of molecules based on named substructures) amongst others. The full list of nodes and their individual descriptions can be found at: https://nodepit.com/iu/org.rdkit.knime.nodes

Vernalis node package: The Vernalis nodes provide a cheminformatics toolkit, which include "Principal Moment of Inertia (PMI)-Derived Properties" (calculates PMI-Derived Properties such as npr1 - First Normalized PMI (i.e., I_1 / I_3), npr2 - Second Normalized PMI (i.e., I_2 / I_3)) and other nodes. The full list of nodes and their individual descriptions can be found at: https://nodepit.com/iu/com.vernalis.knime.database

CDK node package: CDK nodes provide another cheminformatics toolkit, and include "Fingerprint Similarity" (calculates the dissimilarity between the input fingerprints using the Tanimoto coefficient) and other nodes. The full list of nodes and their individual descriptions can be found at: https://nodepit.com/category/community/cdk

ChemAxon node packages:

- Marvin: Marvin nodes are chemistry-related and free-to-access nodes, which include "MarvinSketch" (chemical editor for drawing structures, queries and reactions), "MolConverter" (converts between various data types (mrv, sdf, SMILES, etc.)) and two other nodes. Further information can be found at: https://nodepit.com/iu/jp.co.infocom.cheminfo.marvin

- JChem: JChem nodes are license-protected cheminformatics tools, and include "Advanced MolConverter" (converts between various data types (mrv, sdf, SMILES, etc.)), "Bi Reactor (By Input)" (virtual reaction-processing tool which transforms starting compounds to products according to a given chemical reaction) and other nodes. The full list of nodes and their individual descriptions can be found at: https://nodepit.com/iu/jp.co.infocom.cheminfo.jchem

3.4.1 KNIME Methods

Disclaimer:

Some of the nodes in the workflows summarized in the following figures show the error sign . These nodes were obtained under a time-limited license and were functional when performing the library enumerations and calculations, but the license had expired when generating the figures for this article, hence the error sign.

3.4.2 Enumeration and selection of singly decorated bis-morpholine spiroacetal scaffolds:

The workflow in Figure S16 was constructed to produce an sdf file of 15 diverse mono-decorated scaffolds (Scheme S26), see Section 3.1. Note, this workflow does not give identical results if repeated. This is because the "RDKit diversity picker" node (Node 13 in Figure S16) selects the first compound randomly. The second compound is selected as the most dissimilar (Tanimoto scoring) from the first; the third the most dissimilar from the first two, and so on.

The workflow and associated data files are freely available from UBIRA, the University of Birmingham's eData repository at: https://doi.org/10.25500/edata.bham.00001159

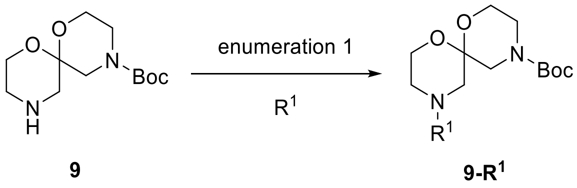

Scheme S26. The enumeration performed by the workflow

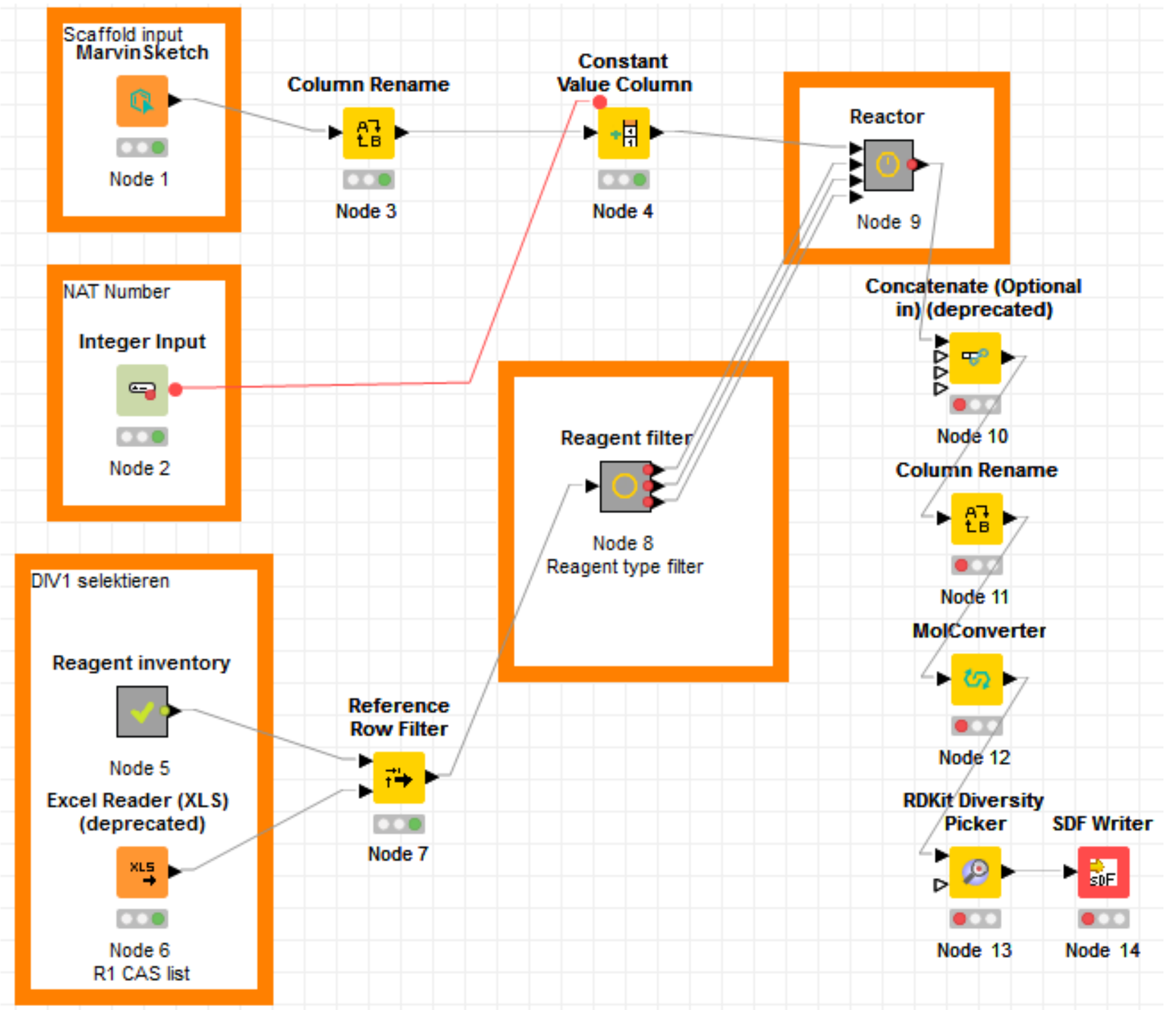

Figure S16: Workflow used to produce 15 singly decorated scaffolds 9-R¹.

node name	node ID	description (shorter)	notes
**MarvinSketch (ChemAxon)**	Node 1	Chemical editor, for drawing structures, queries and reactions.	
**Integer Input**	Node 2	Outputs an integer flow variable with a given number value.	Library code name
**Column Rename**	Node 3	Renames column headings or changes their types.	
**Constant Value Column**	Node 4	Adds/replaces a column containing the same value in each row.	The column name is received as a flow variable from Node 2.
**Metanode**	Node 5	Metanode for inventory reading.	
**Excel Reader (XLS) (deprecated)**	Node 6	This node reads a spreadsheet and provides it at its output port.	
**Reference Row Filter**	Node 7	This node filters rows from the first table using the second table as a reference.	
**Metanode**	Node 8	Metanode for filtering the reagent type	
**Metanode Reactor**	Node 9	Metanode for reactions	Enumeration 1
**Concatenate (Optional in) (deprecated)**	Node 10	This node concatenates tables.	
**Column Rename**	Node 11	Renames column headings or changes their types.	
**MolConverter (ChemAxon)**	Node 12	MolConverter converts between various data types (mrv, sdf, smiles, *etc.*).	
**RDKit Diversity Picker**	Node 13	Picks diverse rows from an input table based on the Tanimoto distance between fingerprints. The picking is performed using the MaxMin algorithm (Ashton, M. *et al.*, *Quant. Struct.-Act. Relat.*, 2002, 21, 598–604).	15 diverse compounds were chosen. In the absence of a reference compound (second input port), the picking starts from a randomly selected compound,

			therefore the selection is not reproducible.
**SDF Writer**	Node 14	This node writes sdf or mol cells into a continuous sdf file.	

Node 5 (**Figure S16**): Metanode for reading the reagent inventory

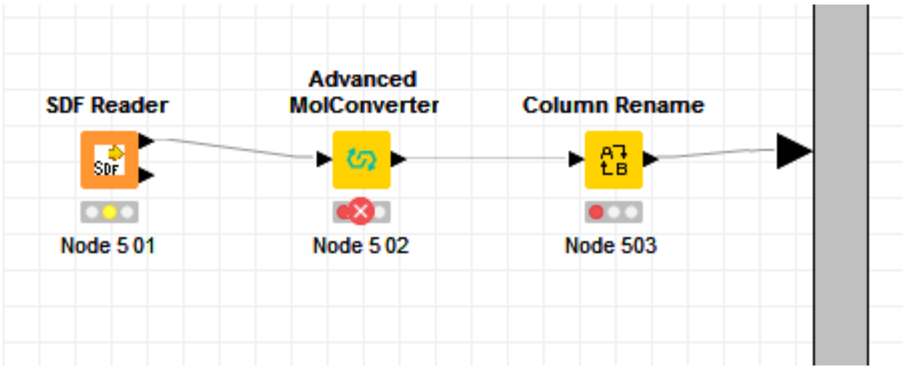

Figure S17: **Workflow for reading the chemical inventory (Node 5, Figure S16).**

node name	node ID	description (short)	notes
**SDF Reader**	Node 501	This node reads an sdf file and creates several columns with each molecule in a new row.	
**Advanced MolConverter (ChemAxon)**	Node 502	Advanced MolConverter converts between various data types (mrv, sdf, Smiles, etc.).	
**Column Rename**	Node 503	Renames column headings or changes their types.	

Node 8 (**Figure S16**): Metanode for filtering reagents

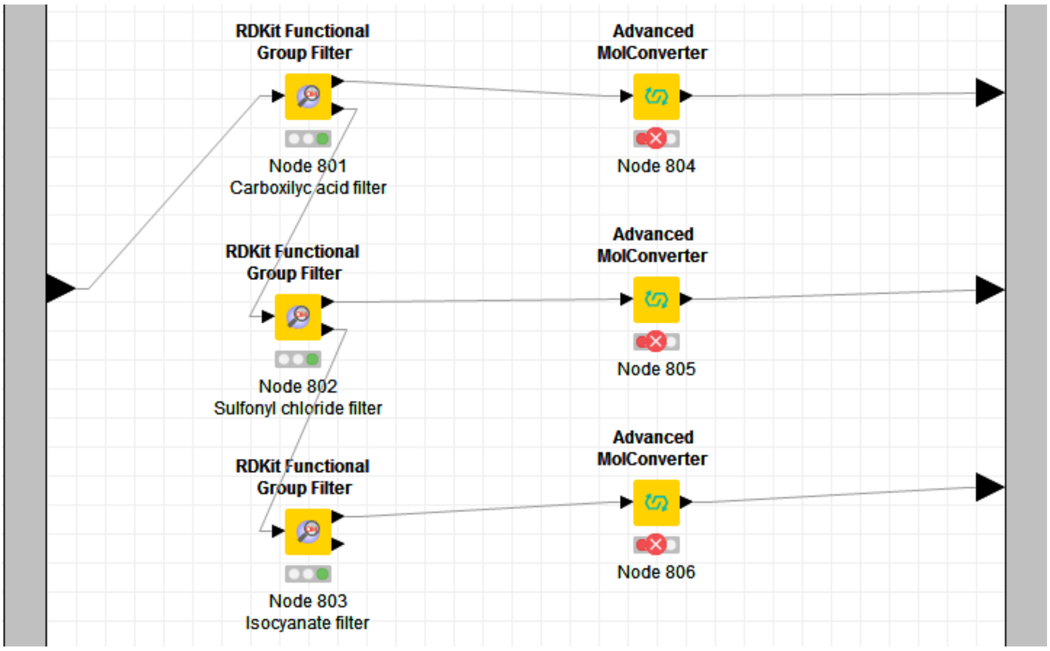

Figure S18: **Workflow for filtering reagents (Node 8, Figure S16).**

node name	node ID	description (short)	notes
**RDKit Functional Group Filter**	Node 801<br>Node 802<br>Node 803	This node was used to filter sets of molecules based on named substructures.	
**Advanced MolConverter (ChemAxon)**	Node 804<br>Node 805<br>Node 806	Advanced MolConverter converts between various data types (mrv, sdf, Smiles, *etc.*).	

Node 9 (**Figure S16**): Metanode Reactor (scaffold decoration)

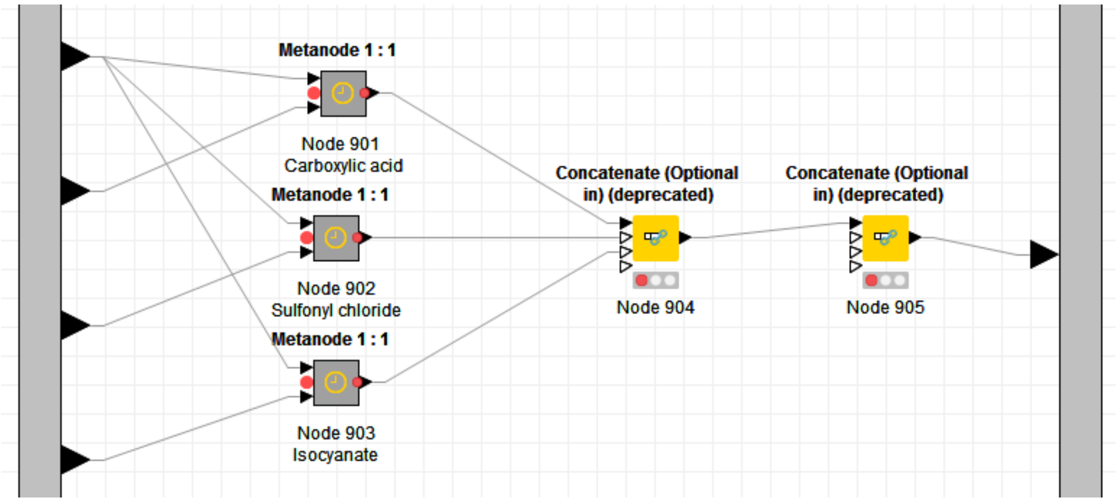

Figure S19: **Workflow of the reactor used to perform virtual scaffold decoration (Node 9, Figure S16).**

Node type	node ID	description (short)	notes
**Metanode 901**<br>**Metanode 902**<br>**Metanode 903**	Node 901<br>Node 902<br>Node 903	The reaction happens in these metanodes.	
**Concatenate (Optional in) (deprecated)**	Node 904<br>Node 905	This node concatenates tables.	

Node 901 / 902 / 903: Metanode 901 / 902 / 903 reaction of scaffold **9** with carboxylic acids (901, amidation) / sulfonyl chlorides (902, sulfonylation) and isocyanates (903, urea formation)

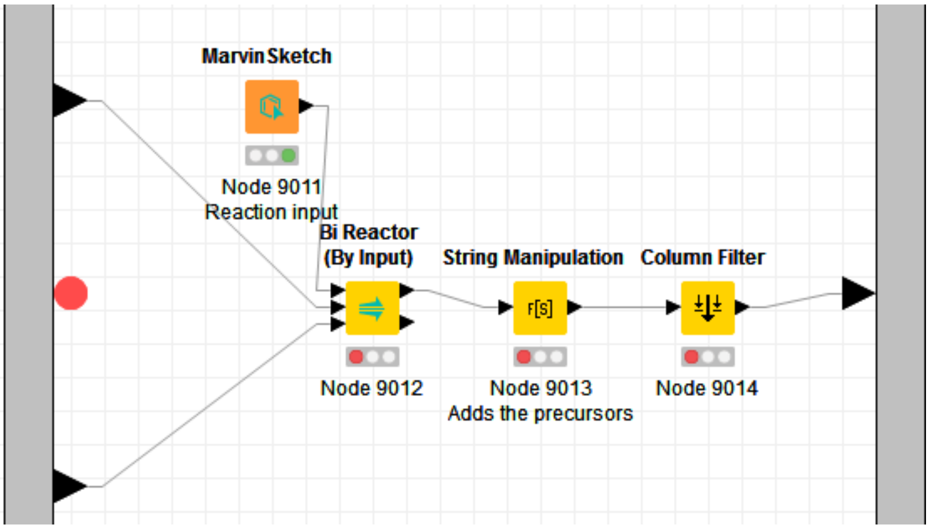

Figure S20: Representative workflow of the compound enumeration shown for amidation (Node 901).

node name	node ID	description (short)	notes
**MarvinSketch (ChemAxon)**	Node 9011<br>Node 9021<br>Node 9031	Chemical editor for drawing structures, queries and reactions.	Scheme about a reaction between a free amine and a carboxylic acid, forming an amid. Figure S21
**Bi Reactor (By Input) (ChemAxon)**	Node 9012<br>Node 9022<br>Node 9032	Reactor is a virtual reaction processing tool which transforms starting compounds to products according to a given chemical reaction definition.	MarvinSketch provided the input for the reaction.
**String Manipulation**	Node 9013<br>Node 9023<br>Node 9033	Manipulates strings, for example by searching and replacing or capitalizing letters.	Used to add the CAS number of the reagents used into a new column.

**Column Filter**	Node 9014<br>Node 9024<br>Node 9034	This node was used to filter columns from the input table while only the remaining columns are passed to the output table.	

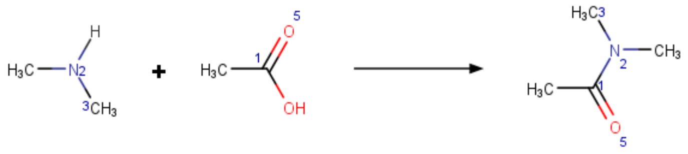

Figure S21: **Drawing in the MarvinSketch Node 9011.**

3.4.3 Complete enumeration of virtual libraries for scaffolds 9 and 25

The workflow in Figure S22 was developed to produce an sdf file of 630-630 library compounds from 6,6-spiroacetal scaffold **9** and 6,7-spiroacetal scaffold **25**. After the selection and filtering of 15 amine decorating reagents, the workflow produces 15 singly decorated spiroacetals by Enumeration 1 (Node 9, Scheme S27). Enumeration 2 is performed from Node 15 to Node 20 to produce the final virtual library of 630 compounds. The workflow and associated data files are freely available from UBIRA, the University of Birmingham's eData repository at: https://doi.org/10.25500/edata.bham.00001159

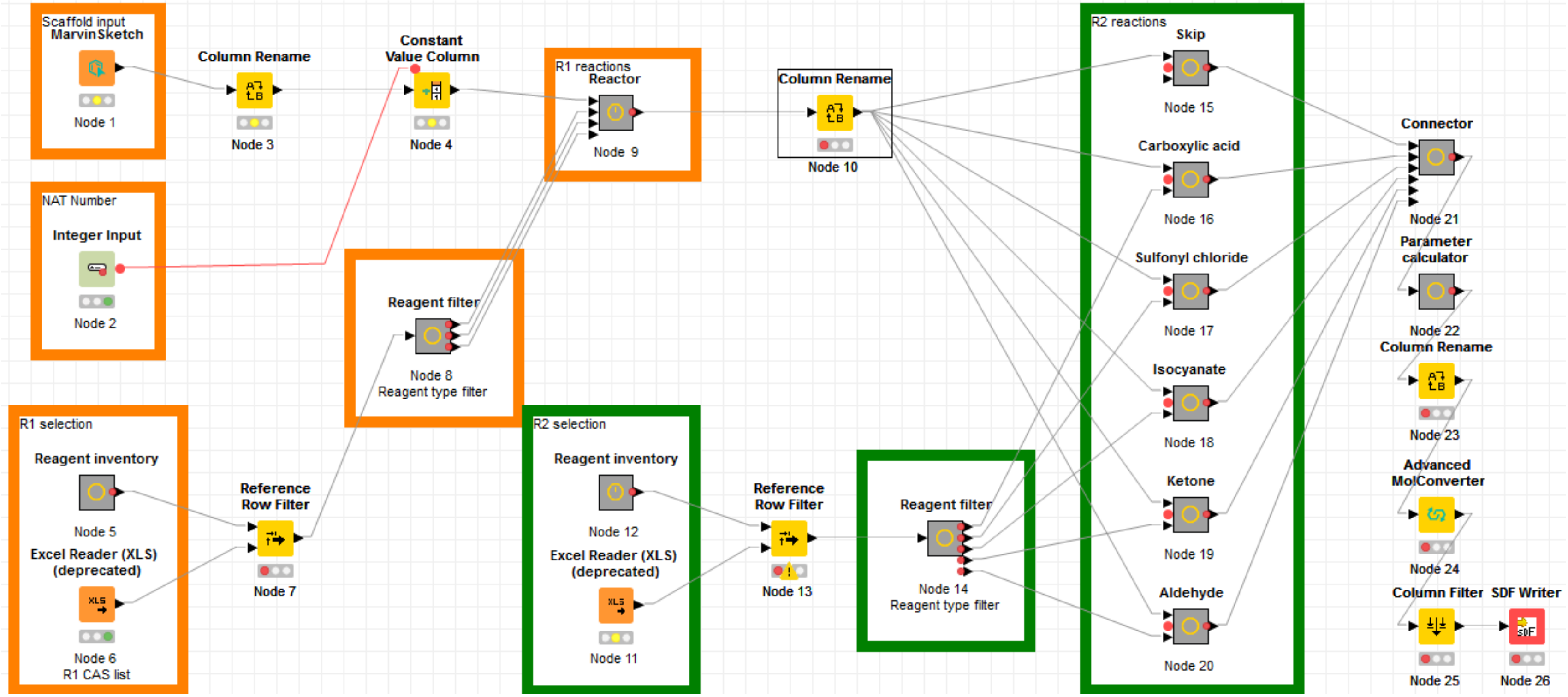

Figure S22: **Workflow for enumerating the full virtual libraries of 630 compounds for the 6,6-spiroacetal (scaffold 9) and 6,7-spiroacetal scaffold (25).**

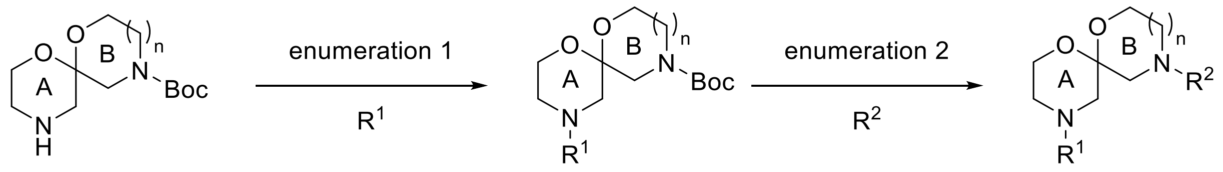

9 n = 1
25 n = 2

9-R¹ n = 1
25-R¹ n = 2

L1-R¹-R² n = 1
L2-R¹-R² n = 2

Scheme S27. The enumeration performed by the workflow summarised in Figure S22

node name	node ID	description (shorter)	notes
**MarvinSketch (ChemAxon)**	Node 1	Chemical editor, for drawing structures, queries and reactions.	Drawing of the scaffold
**Integer Input**	Node 2	Outputs an integer flow variable with a given number value.	Library code name
**Column Rename**	Node 3	Renames column headings or changes their types.	
**Constant Value Column**	Node 4	Adds/replaces a column containing the same value in each row.	
**Metanode**	Node 5<br>Node 12	Metanode for reagent inventory check for R^1 and R^2	
**Excel Reader (XLS) (deprecated)**	Node 6<br>Node 11	This node reads a spreadsheet and provides it at its output port.	Reads in the R^1 and R^2 selected reagent CAS numbers
**Reference Row Filter**	Node 7<br>Node 13	This node filters rows from the first table using the second table as a reference.	
**Metanode**	Node 8	Metanode for filtering reagents for R^1	
**Metanode Reactor**	Node 9	Metanode for performing reactions for the first enumeration (R^1 position)	Enumeration 1
**Column Rename**	Node 10<br>Node 23	Renames column headings or changes their types.	
**Metanode**	Node 14	Metanode for filtering reagents for R^2	

**Metanode Skip**	Node 15	Metanode for performing no reaction in the second enumeration (R^2 position – Skip)	Leaves a free 2° amine
**Metanode Carboxylic acid**	Node 16	Metanode for performing an amidation reaction in R^2 position	Amide formation
**Metanode Sulfonyl chloride**	Node 17	Metanode for performing a sulfonylation reaction in R^2 position	Sulfonamide formation
**Metanode Isocyanate**	Node 18	Metanode for introducing a urea into R^2 position	Urea formation
**Metanode Keton**	Node 19	Metanode for performing a reductive amination reaction in R^2 position with a ketone	3° amine formation
**Metanode Aldehyde**	Node 20	Metanode for performing a reductive amination reaction in R^2 position with an aldehyde	3° amine formation
**Metanode Concatenate**	Node 21	Metanode for concatenating datasets	
**Metanode Parameter**	Node 22	Metanode for parameter calculations	HBD, HBA, TPSA, clogP, logD, Fsp^3
**Advanced MolConverter (ChemAxon)**	Node 24	Advanced MolConverter converts between various data types (mrv, sdf, SMILES, *etc.*).	
**Column Filter**	Node 25	This node was used to filter columns from the input table while only the remaining columns are passed to the output table.	
**SDF Writer**	Node 26	This node writes sdf or mol cells into a continuous sdf file.	

Node 5 / 12 (Figure S22): Metanode for reading AnalytiCon Discovery's reagent inventory

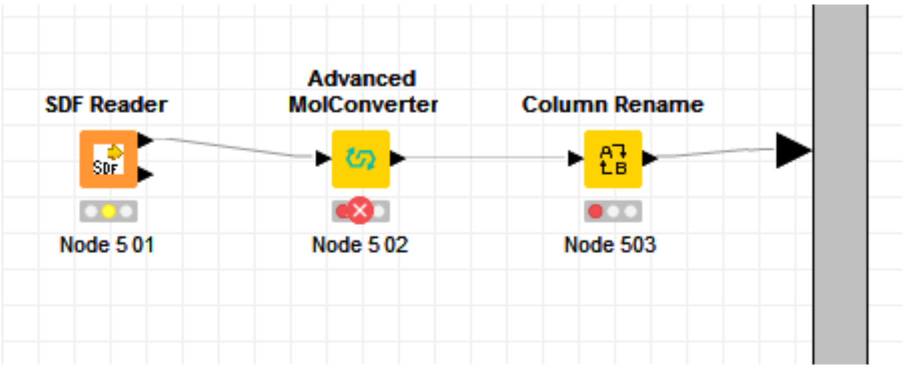

Figure S23: Workflow for reading the chemical inventory (Node 5, Figure S22).

node name	node ID	description (short)	notes
**SDF Reader**	Node 501<br>Node 1201	This node reads an sdf file and creates several columns with each molecule in a new row.	
**Advanced MolConverter (ChemAxon)**	Node 502<br>Node 1202	Advanced MolConverter converts between various data types (mrv, sdf, SMILES, *etc.*).	
**Column Rename**	Node 503<br>Node 1203	Renames column headings or changes their types.	

Node 8 (**Figure S22**): Metanode for filtering reagent type (introducing R^1)

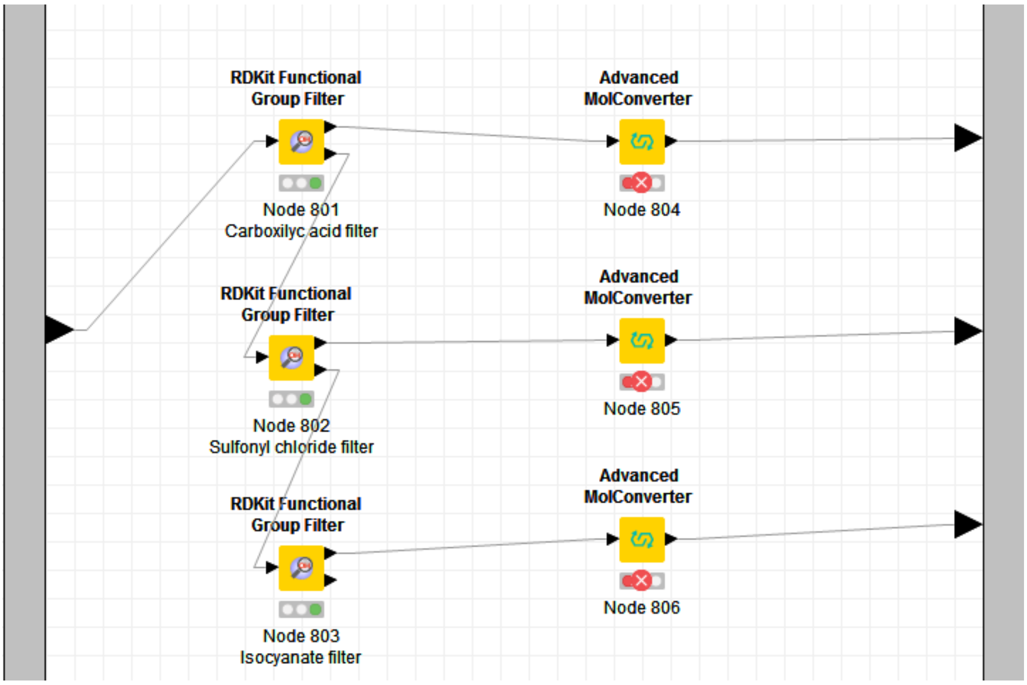

Figure S24: Workflow of filtering reagents (Node 8, Figure S22).

node name	node ID	description (short)	notes
**RDKit Functional Group Filter**	Node 801<br>Node 802<br>Node 803	This node was used to filter sets of molecules based on named substructures.	
**Advanced MolConverter (ChemAxon)**	Node 804<br>Node 805<br>Node 806	Advanced MolConverter converts between various data types (mrv, sdf, SMILES, etc.).	

Node 9 (**Figure S22)**: Metanode for performing the first decoration step (introducing R^1)

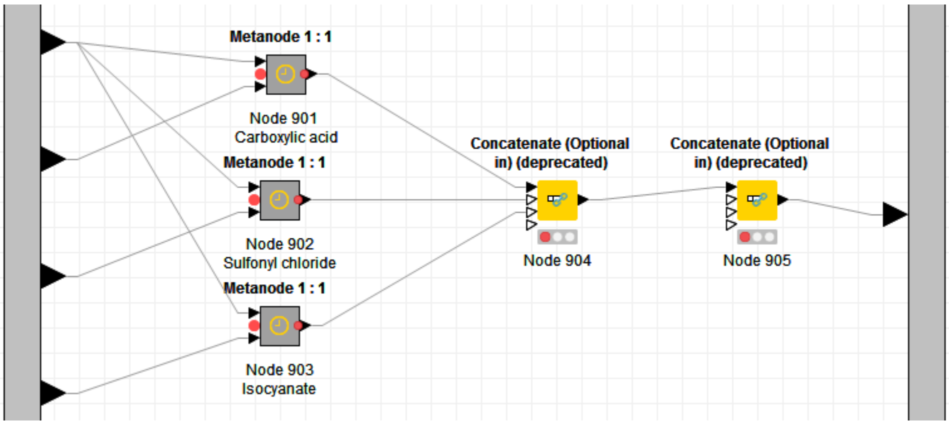

Figure S25: Workflow of the reactor metanode (Node 9, Figure S22).

node name	node ID	description (short)	notes
**Metanode 901**<br>**Metanode 902**<br>**Metanode 903**	Node 901<br>Node 902<br>Node 903	The virtual reaction is performed in these metanodes.	
**Concatenate (Optional in) (deprecated)**	Node 904<br>Node 905	This node concatenates tables.	

Node 901 / 902 / 903: Metanode 901 / 902 / 903 reaction of the scaffold with carboxylic acids (901, amidation) / sulfonyl chlorides (902, sulfonylation) and isocyanates (903, urea formation)

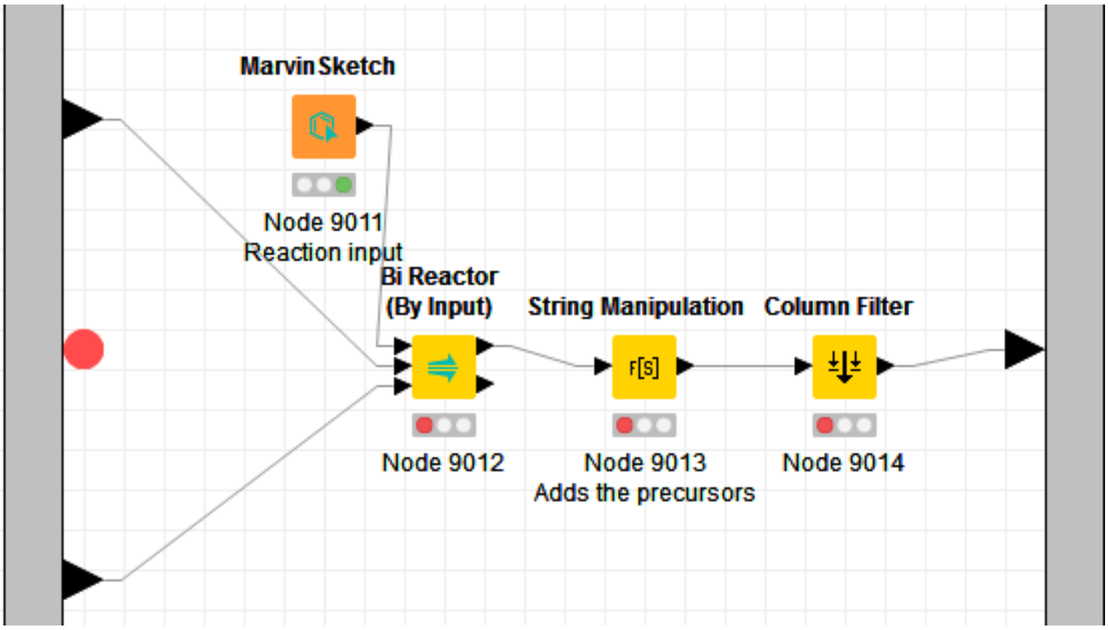

Figure S26: **Workflow of the compound enumeration shown for the amidation as an example (Node 901).**

node name	node ID	description (short)	notes
**MarvinSketch (ChemAxon)**	Node 9011 Node 9021 Node 9031	Chemical editor, for drawing structures, queries and reactions.	Reaction between the free amine and the reagent
**Bi Reactor (By Input) (ChemAxon)**	Node 9012 Node 9022 Node 9032	Reactor is a virtual reaction processing tool which transforms starting compounds to products according to a given chemical reaction definition.	
**String Manipulation**	Node 9013 Node 9023 Node 9033	Manipulates strings, for example by searching and replacing or capitalizing letters.	Adding the used reagents into a new column.
**Column Filter**	Node 9014 Node 9024 Node 9034	This node was used to filter columns from the input table while only the remaining columns are passed to the output table.	

Node 14 (Figure S22): Metanode for filtering reagents for second decoration step (introducing R^2)

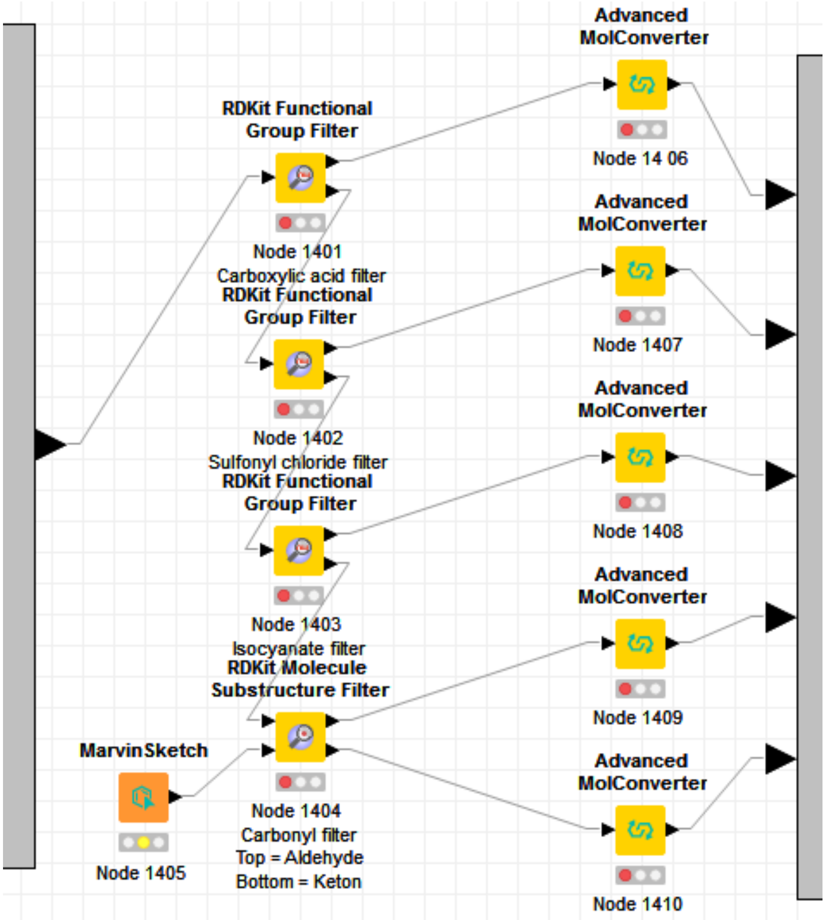

Figure S27: **Workflow for filtering reagents (introduction of R^2) (Node 14, Figure S22).**

node name	node ID	description (short)	notes
**RDKit Functional Group Filter**	Node 1401<br>Node 1402<br>Node 1403	This node was used to filter sets of molecules based on named substructures.	
**RDKit Molecule Substructure Filter**	Node 1404	Applies a substructure filter to an input column.	Top output port filters the ketones. Bottom output port filters aldehydes.
**MarviSketch (ChemAxon)**	Node 1405	Chemical editor, for drawing structures, queries and reactions.	Drawing of a general ketone

**Advanced MolConverter (ChemAxon)**	Node 1406<br>Node 1407<br>Node 1408<br>Node 1409<br>Node 1410	Advanced MolConverter converts between various data types (mrv, sdf, SMILES, etc.).	

Node 15 (**Figure S22**): Metanode for performing no reaction in the second decoration step – Skip – introducing a 2° amine in R^2 position

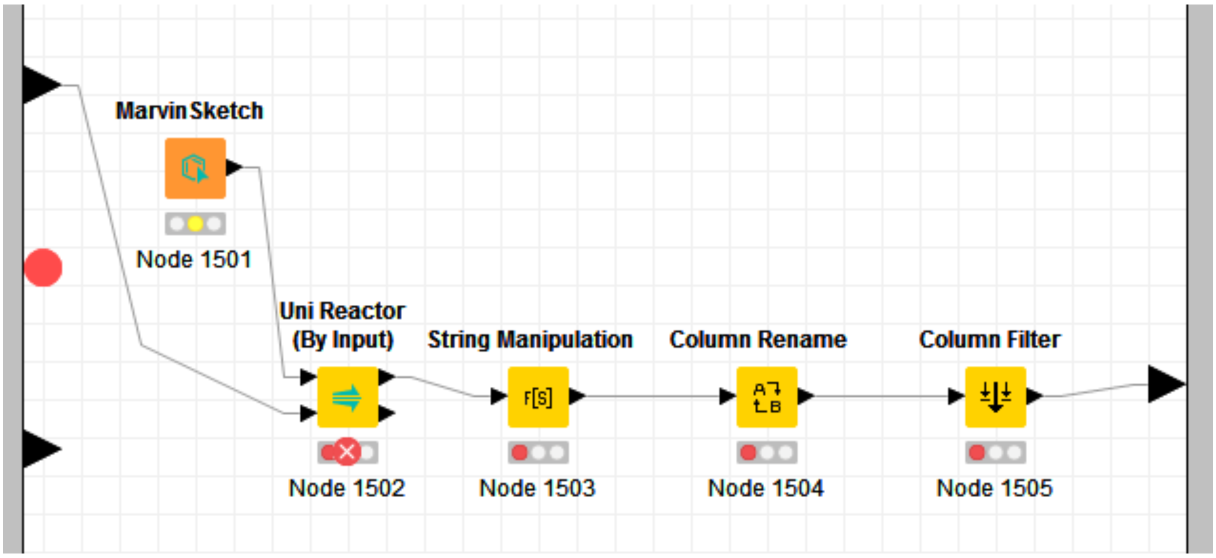

Figure S28: **Workflow for introducing a 2° amine in the second decoration step (Node 15, Figure S22).**

node name	node ID	description (short)	notes
**MarvinSketch (ChemAxon)**	Node 1501	Chemical editor for drawing structures, queries and reactions.	Drawing of the deprotection of the R^2 amine
**Uni Reactor (By Input) (ChemAxon)**	Node 1502	Reactor is a virtual reaction processing tool which transforms starting compounds to products according to a given chemical reaction definition.	

**String Manipulation**	Node 1503	Manipulates strings, for example by searching and replacing or capitalizing letters.	Adding the used reagents into a new column.
**Column Rename**	Node 1504	Renames column headings or changes their types.	
**Column Filter**	Node 1505	This node was used to filter columns from the input table while only the remaining columns are passed to the output table.	

Node 16 / 17 / 18 / 19 / 20: Metanode 16 / 17 / 18 / 19 / 20 reaction of the second decoration step – introduction of R^2 – amidation (Node 16), sulfonylation (Node 17), urea formation (Node 18), reductive amination using a ketone (Node 19), reductive amination using an aldehyde (Node 20)

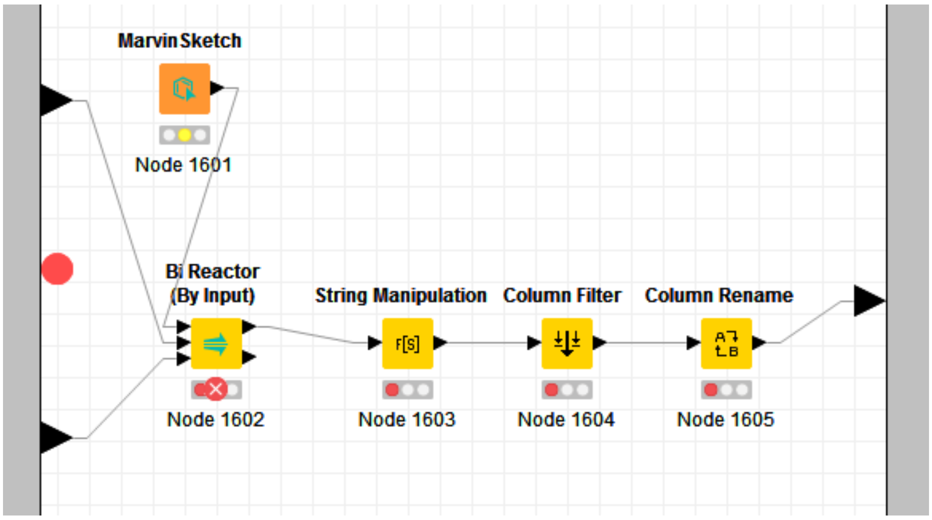

Figure S29: **Workflow for the 2nd decoration step with amidation as an example (Node 16, Figure S22).**

node name	node ID	description (short)	notes
**MarvinSketch (ChemAxon)**	Node 1601<br>Node 1701<br>Node 1801<br>Node 1901	Chemical editor for drawing structures, queries and reactions.	Drawing of the deprotection of the R^2 amine and reaction of the

	Node 2001		resulting 2° amine with the chosen type of reagent. Figure S30
**Bi Reactor (By Input) (ChemAxon)**	Node 1602<br>Node 1702<br>Node 1802<br>Node 1902<br>Node 2002	Reactor is a virtual reaction processing tool which transforms starting compounds to products according to a given chemical reaction definition.	
**String Manipulation**	Node 1603<br>Node 1703<br>Node 1803<br>Node 1903<br>Node 2003	Manipulates strings, for example by searching and replacing or capitalizing letters.	Adding the used reagents into a new column.
**Column Rename**	Node 1604<br>Node 1704<br>Node 1804<br>Node 1904<br>Node 2004	Renames column headings or changes their types.	
**Column Filter**	Node 1605<br>Node 1705<br>Node 1805<br>Node 1905<br>Node 2005	This node was used to filter columns from the input table while only the remaining columns are passed to the output table.	

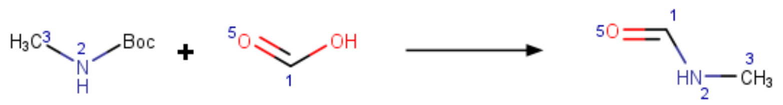

Figure S30: **Representative drawing in MarvinSketch (Amidation, Node 1601).**

Node 21: Metanode for concatenating the enumerated datasets

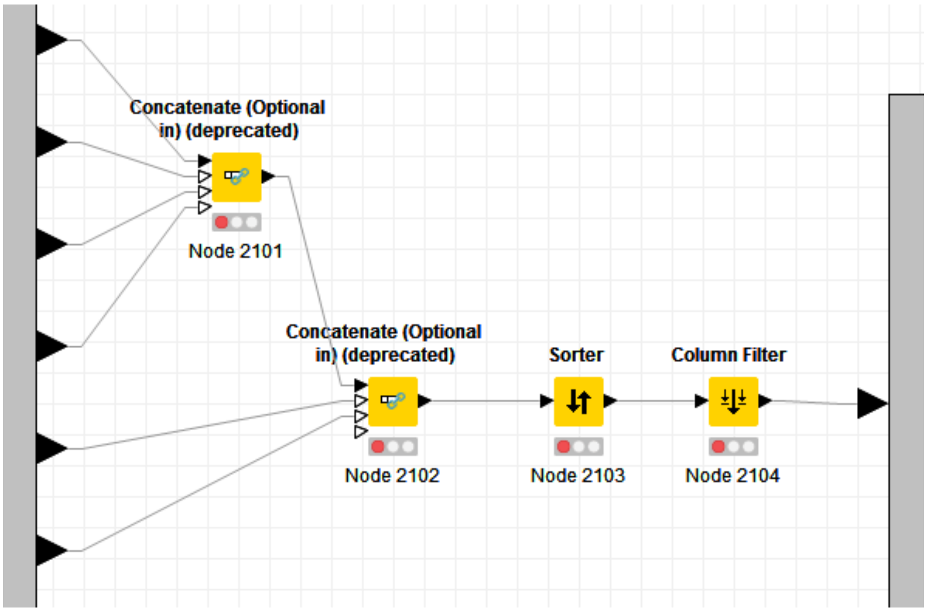

Figure S31: Workflow for concatenation and data sorting (Node 21, Figure S22).

node name	node ID	description (short)	notes
**Concatenate (Optional in) (deprecated)**	Node 2101<br>Node 2102	This node concatenates tables.	
**Sorter**	Node 2103	This node sorts rows according to user-defined criteria.	
**Column Filter**	Node 2104	This node was used to filter columns from the input table while only the remaining columns are passed to the output table.	

Node 22: Metanode parameter calculator

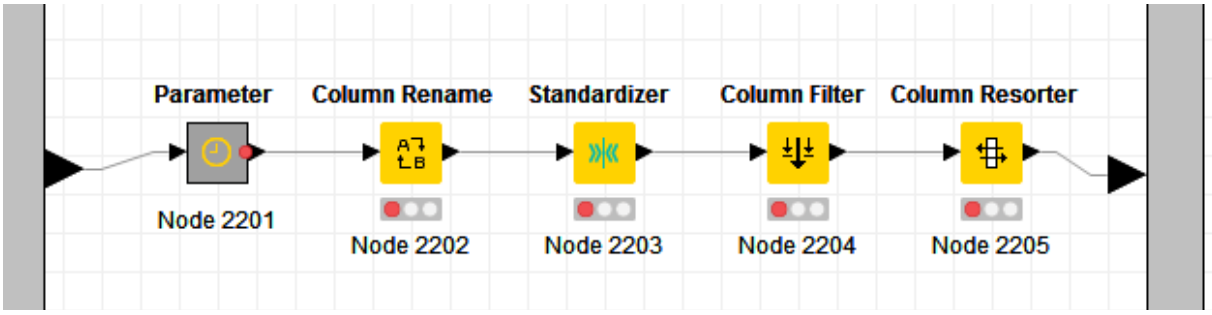

Figure S32: Workflow for calculating parameters for library compounds (Node 22, Figure S22).

node name	node ID	description (short)	notes
**Metanode**	Node 2201	Metanode for physicochemical parameter calculations	
**Column Rename**	Node 2202	Renames column headings or changes their types.	
**Standardizer (ChemAxon)**	Node 2203	Standardizer is a structure canonicalization tool that converts molecules from different sources into standard representational forms.	
**Column Filter**	Node 2204	This node was used to filter columns from the input table while only the remaining columns are passed to the output table.	
**Column Resorter**	Node 2205	This node changes the order of the input columns, based on user-defined settings.	

Node 2201: Metanode for calculating physicochemical parameters

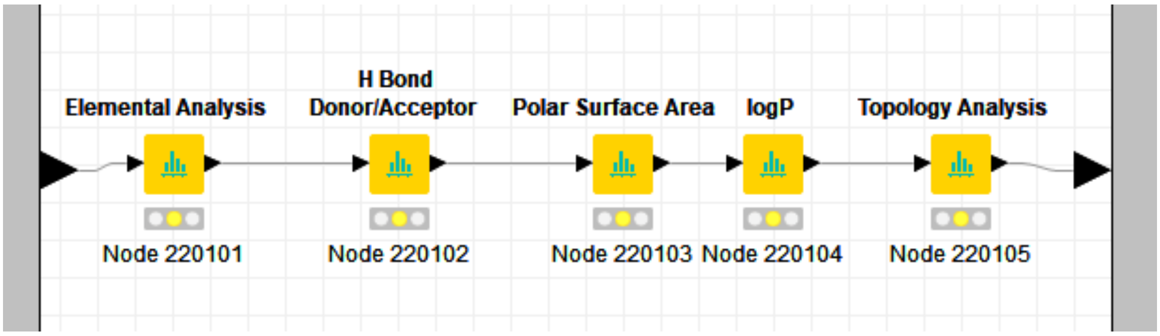

Figure S33: Workflow for calculating physicochemical parameters (Node 2201, Figure S22).

node name	node ID	description (short)	notes
**Elemental Analysis (ChemAxon)**	Node 220101	The Elemental Analysis Plugin calculates basic descriptors related to the elemental composition of a molecule.	
**H Bond Donor/Acceptor (ChemAxon)**	Node 220102	The Hydrogen Bond Donor-Acceptor Plugin calculates atomic hydrogen bond donor and acceptor inclination.	
**Polar Surface Area (ChemAxon)**	Node 220103	Estimation of Topological Polar Surface Area (TPSA) is based on the method described by Ertl *et al. J. Med. Chem.,* 2000, 43, 3714–3717	
**logP (ChemAxon)**	Node 220104	The logP node calculates the *n*-octanol/water partition coefficient as a measure of molecular hydrophobicity. (Klopman *et al. J. Chem. Inf. Comput. Sci.* 1994, 34, 752–781)	cLogP values were calculated exclusively with this node, with the consensus method.
**Topology Analysis (ChemAxon)**	Node 220106	The Topology Analysis Plugin calculates different topological descriptors for a molecule.	Rotatable bonds and Fsp^3 values were calculated with this node.

3.4.4 Filtering of the FDA-approved list of drugs

To compare our virtual library of bis-morpholine spiroacetals with the Food and Drug Administration (FDA) approved drugs the two collections were filtered in KNIME, first to eliminate all salt forms and then by the Ro5 guidelines (H-Bond donors <5, H-bond acceptors <10, cLogP <5.0) with a molecular weight threshold of 700 g mol^{-1}, given the molecular weight of several orally marketed, orally administered drugs is higher than the 500 g mol^{-1}.[29] This filtering reduced the FDA list from 4568[aa] to 2709 compounds. Of the 630 compounds in the bis-morpholine spiroacetal virtual library, only six compounds had cLogP values exceeding 5.0 but these were retained in the library for the diversity selection.

3.4.5 Measurement of fingerprint-based dissimilarity

The workflow summarized in Figure S34 was used to measure the fingerprint-based dissimilarity between two sets of compounds in KNIME. Nodes were executed in numerical order to produce an Excel file. The histogram of the Tanimoto coefficients was produced in Excel. Figure S35 shows the settings of the Fingerprint Similarity node. The workflow and associated data files are freely available from UBIRA, the University of Birmingham's eData repository at: https://doi.org/10.25500/edata.bham.00001159

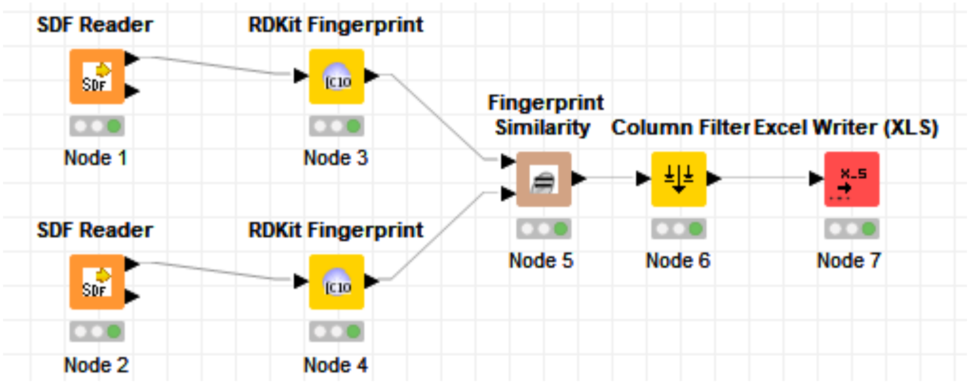

Figure S34: Workflow used to measure fingerprint-based dissimilarity.

[aa] Exported from CDD vault on 05.05.2022 based on the following public collections "FDA Approved: Tox", "FDA Approved: Approved drugs" and "FDA Approved: Orphan drugs". Further information about these collections can be found at: https://www.collaborativedrug.com/public-access/

node name	node ID	description (short)	notes
**SDF Reader**	Node 1<br>Node 2	This node reads an sdf file and creates several columns with each molecule in a new row.	Node 2 contains the reference compounds that Node 1 compounds are compared to.
**RDKit Fingerprint**	Node 3<br>Node 4	This node produces Morgan_2 fingerprints which are the equivalents of the ECFP_4 circular fingerprints.	1024 bits long fingerprints were calculated.
**Fingerprint Similarity**	Node 5	This node calculates the dissimilarity between the input fingerprints using the Tanimoto coefficient.	
**Column Filter**	Node 6	This node was used to filter columns from the input table while only the remaining columns are passed to the output table.	
**Excel Writer (XLS)**	Node 7	This node writes the input data table into an Excel spreadsheet.	

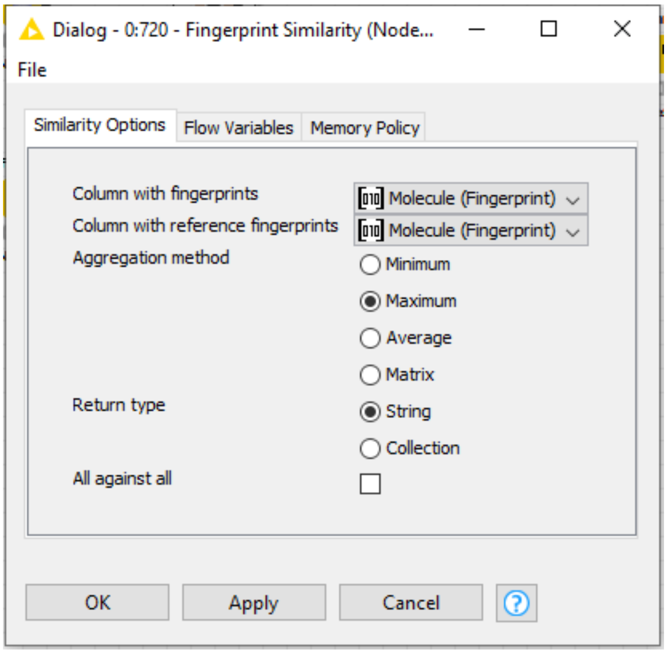

Figure S35: Configuration of the Fingerprint Similarity node.

3.5 DataWarrior methods

3.5.1 Diversity subset selection for physical compound synthesis

Description of the clustering algorithm:

After calculating a chosen fingerprint descriptor, the algorithm calculates a full similarity matrix. Using this matrix, similar compounds are placed into the same cluster. The similarity between any compound and a cluster is calculated as a mean of the cluster members. Compounds are placed into the clusters until one of the stop criteria is met (Figure S36). If none of the stop criteria is reached before placing every compound into a cluster, the algorithm merges clusters until one of the stop criteria is reached. A fuller description of the method can be read at: http://www.openmolecules.org/help/chemistry.html#ClusterCompounds

Step by step description to produce subset **DW-Cluster-SkelSphereFP**:

The dataset sdf file of the full virtual library was opened within DataWarrior. Then the "SkelSphereFP" descriptor was calculated for the compounds (Chemistry => From Chemical Structure => Calculate Descriptor => SkelSphereFP). After the fingerprint calculation, the diversity subset of 150 compounds was performed (Figure S36, Chemistry => Cluster Compounds/Reactions). The software made two new columns, "Cluster No" and "Is Representative." Each compound was assigned to a cluster which is indicated in the "Cluster No" column. For each cluster, a representative compound was selected which is shown in the "Is representative" column. These representative compounds were exported into a new sdf file as the diversity subset **DW-Cluster-SkelSphereFP**. The data files are freely available from UBIRA, the University of Birmingham's eData repository at: https://doi.org/10.25500/edata.bham.00001159

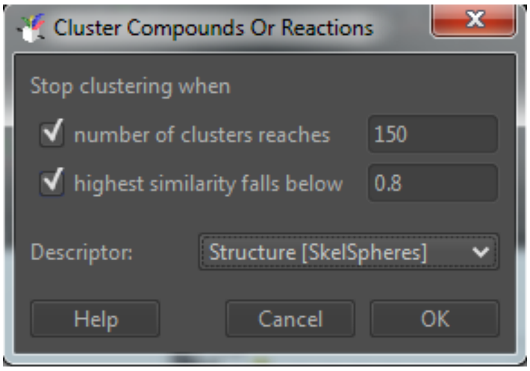

Figure S36: Setup for clustering the virtual library into 150 clusters, with 0.8 Tanimoto coefficient as highest similarity and utilizing the SkelSphereFP descriptor.

4 Crystal structures

Crystals of spiroacetal **7**, suitable for analysis by X-ray analysis, were grown by slow evaporation of a solution of **7** in $CDCl_3$. Crystals of spiroacetal **23**, suitable for analysis by X-ray analysis, were grown by evaporation of a solution of **23** in EtOAc/*n*-heptane. The datasets for both spiroacetals, **7** and **23**, were measured on an Agilent SuperNova diffractometer using an Atlas detector. The data collections were driven and processed and absorption corrections were applied using CrysAlisPro.[18] Both structures were solved using ShelXT[19] and refined by a full-matrix least-squares procedure on F^2 in ShelXL.[20] Figures and reports were produced using OLEX2[21] and ORTEP-3 for Windows.[22] All non-hydrogen atoms were refined with anisotropic displacement parameters and all hydrogen atoms in both structures were fixed as riding models and the isotropic thermal parameters (U_{iso}) were based on the U_{eq} of the parent atoms.

Both structures occupy centrosymmetric space groups such that there is a racemic mixture of enantiomers.

Compound 7: The structure contains two crystallographically-independent molecules. The crystal was twinned with the two components being related by 180° about the reciprocal direction [1 0 0]. The refined percentage ratio of the two twin components is 52.18 (7) : 47.82 (7).

Compound 23: The crystal was twinned with the two components being related by 180° about the reciprocal direction [0 0 1]. The refined percentage ratio of the two twin components is 76.84 (11) : 23.16 (11).

Crystal data for **7**: $C_{19}H_{28}N_2O_4$ (M =348.43 g mol^{-1}): triclinic, space group P-1 (no. 2), a = 9.0866(5) Å, b = 12.2999(7) Å, c = 16.7315(10) Å, α = 87.234(5)°, β = 87.223(5)°, γ = 88.732(4)°, V = 1865.24(19) Å^3, Z = 4, T = 99.98(10) K, μ(Cu Kα) = 0.705 mm^{-1}, *Dcalc* = 1.241 g cm^{-3}, 12360 reflections measured (7.196° ≤ 2Θ ≤ 149.678°), 12360 unique (R_{int} = ?, R_{sigma} = 0.0262) which were used in all calculations. The final R_1 was 0.0380 (I > 2σ(I)) and wR_2 was 0.1038 (all data).

Crystal data for **23**: $C_{21}H_{30}N_2O_6$ (M =406.47 g mol^{-1}): monoclinic, space group $P2_1/c$ (no. 14), a = 8.6370(4) Å, b = 9.7490(5) Å, c = 24.4388(11) Å, β = 94.118(4)°, V = 2052.49(17) Å^3, Z = 4, T = 100.01(10) K, μ(Cu Kα) = 0.795 mm^{-1}, *Dcalc* = 1.315 g cm^{-3}, 5829 reflections measured (7.254° ≤ 2Θ ≤ 146.186°), 5829 unique (R_{int} = ?, R_{sigma} = 0.0337) which were used in all calculations. The final R_1 was 0.0518 (I > 2σ(I)) and wR_2 was 0.1449 (all data).

CCDC 2375037 and CCDC 2375038 contain the supplementary crystallographic data for this paper. These data can be obtained free of charge from The Cambridge Crystallographic Data Centre via www.ccdc.cam.ac.uk/data_request/cif.

4.1.1 Single Crystal X-ray structure of bis-morpholine spiroacetal 7

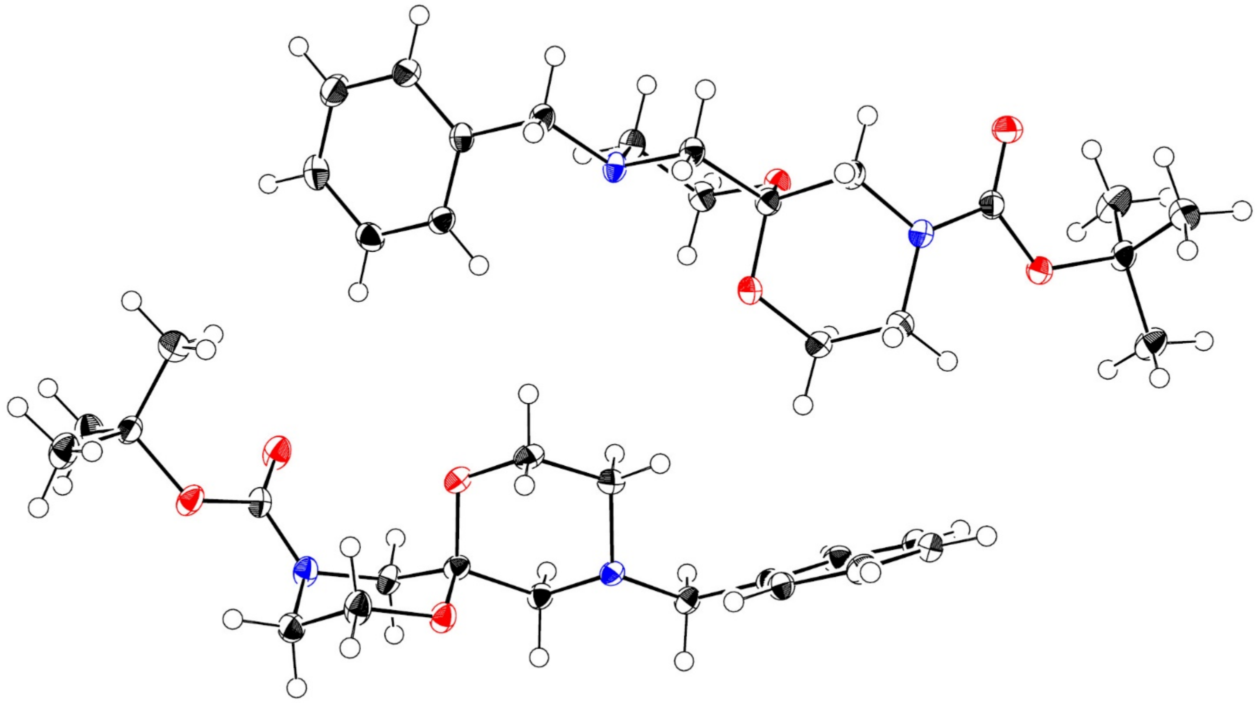

Figure S37: ORTEP plot of 7 with ellipsoids drawn at the 50% probability level, confirming the double anomeric stabilization of the spiroacetal. The structure contains two crystallographically-independent molecules as shown. Atomic displacement parameters at 100 K.

Table S11 Crystal data and structure refinement for **7**.

Identification code	**7**
Empirical formula	$C_{19}H_{28}N_2O_4$
Formula weight	348.43
Temperature/K	99.98(10)
Crystal system	triclinic
Space group	P-1
a/Å	9.0866(5)
b/Å	12.2999(7)
c/Å	16.7315(10)
α/°	87.234(5)
β/°	87.223(5)
γ/°	88.732(4)
Volume/Å^3	1865.24(19)
Z	4
ρ_{calc}g/cm^3	1.241
μ/mm^{-1}	0.705
F(000)	752.0
Crystal size/mm^3	0.184 × 0.153 × 0.091
Radiation	Cu Kα (λ = 1.54184)
2Θ range for data collection/°	7.196 to 149.678
Index ranges	–11 ≤ h ≤ 8, –15 ≤ k ≤ 15, –20 ≤ l ≤ 20
Reflections collected	12360
Independent reflections	12360 [R_{int} = ?, R_{sigma} = 0.0262]
Data/restraints/parameters	12360/0/458
Goodness-of-fit on F^2	0.999
Final R indexes [I>=2σ (I)]	R_1 = 0.0380, wR_2 = 0.0994
Final R indexes [all data]	R_1 = 0.0481, wR_2 = 0.1038
Largest diff. peak/hole / e Å^{-3}	0.35/–0.23

Table S12 Fractional Atomic Coordinates (×10^4) and Equivalent Isotropic Displacement Parameters (Å^2×10^3) for compound **7**. U_{eq} is defined as 1/3 of the trace of the orthogonalized U_{IJ} tensor.

Atom	*x*	*y*	*z*	U(eq)
C1	1872.9(14)	1173.4(11)	3498.6(8)	17.8(3)
C2	2114.2(15)	680.7(12)	2680.4(8)	20.6(3)
C3	83.3(15)	1798.1(13)	2190.5(9)	23.7(3)
C4	-33.5(15)	2345.5(13)	2976.6(9)	24.1(3)
C5	2058.8(14)	305.0(11)	4173.0(8)	18.1(3)
C6	3260.6(15)	1564.0(12)	4940.9(8)	20.2(3)
C7	2955.5(15)	2438.0(11)	4301.6(9)	21.1(3)
C8	2599.9(15)	1958.7(11)	1536.1(8)	19.0(3)

Table S12 Fractional Atomic Coordinates (×10[4]) and Equivalent Isotropic Displacement Parameters ($Å^2×10^3$) for compound **7**. U_{eq} is defined as 1/3 of the trace of the orthogonalized U_{IJ} tensor.

Atom	*x*	*y*	*z*	U(eq)
C9	2715.4(15)	3466.1(12)	514.6(8)	20.4(3)
C10	1503.1(17)	4236.4(13)	226.3(9)	27.8(3)
C11	3376.2(17)	2819.7(14)	-171.3(9)	27.7(3)
C12	3856.2(18)	4087.2(14)	935.5(10)	30.1(3)
C13	2337.4(14)	-134.6(11)	5558.1(8)	18.7(3)
C14	2402.0(14)	210.1(11)	6407.0(8)	18.0(3)
C15	1494.7(14)	1047.9(12)	6704.8(8)	19.4(3)
C16	1542.4(15)	1335.6(12)	7494.2(9)	21.6(3)
C17	2493.4(15)	779.9(12)	8009.5(9)	21.6(3)
C18	3401.0(15)	-61.5(13)	7722.6(9)	22.4(3)
C19	3359.7(14)	-334.0(12)	6928.3(8)	20.4(3)
N1	1608.3(13)	1419.8(10)	2040.4(7)	20.7(2)
N2	2091.5(12)	758.3(9)	4961.9(7)	17.0(2)
O1	423.1(10)	1612.9(8)	3619.6(6)	20.7(2)
O2	2939.3(10)	1999.8(8)	3524.7(6)	19.7(2)
O3	3911.3(11)	1737.4(9)	1478.8(6)	26.1(2)
O4	1899.2(10)	2738.9(9)	1095.3(6)	24.2(2)
C101	6999.3(14)	3677.3(11)	6385.6(8)	17.9(3)
C102	7032.2(16)	4085.6(12)	7230.0(8)	20.9(3)
C103	5274.6(15)	2691.5(13)	7687.7(9)	24.3(3)
C104	5378.7(16)	2261.4(12)	6857.6(8)	23.3(3)
C105	7048.9(15)	4636.8(11)	5769.5(8)	18.8(3)
C106	8570.7(15)	3605.2(12)	4880.4(8)	20.1(3)
C107	8430.7(15)	2637.2(11)	5468.4(8)	20.6(3)
C108	7605.3(15)	2907.6(11)	8398.6(8)	19.2(3)
C109	7833.7(15)	1600.1(12)	9544.7(8)	20.8(3)
C110	7714.1(17)	2450.9(14)	10172.0(9)	27.8(3)
C111	6924.9(17)	611.1(13)	9803.5(10)	27.4(3)
C112	9424.2(17)	1263.5(14)	9341.9(10)	31.5(4)
C113	7276.4(15)	5226.0(11)	4379.3(8)	19.3(3)
C114	7351.8(14)	4895.7(11)	3520.7(8)	18.0(3)
C115	6483.3(14)	4055.3(12)	3279.4(8)	19.6(3)
C116	6522.8(15)	3761.3(12)	2492.1(9)	23.0(3)
C117	7442.5(17)	4306.0(14)	1920.9(9)	26.3(3)
C118	8327.4(17)	5137.4(13)	2153.3(9)	27.3(3)
C119	8282.5(15)	5423.6(12)	2943.7(9)	22.8(3)
N101	6649.4(13)	3230.5(10)	7829.5(7)	21.7(3)
N102	7229.4(12)	4284.7(9)	4952.2(7)	18.0(2)
O101	5674.3(10)	3124.4(8)	6266.4(6)	20.1(2)
O102	8250.1(10)	2973.1(8)	6276.6(6)	19.5(2)
O103	8743.8(11)	3373.3(9)	8506.2(6)	26.2(2)

Table S12 Fractional Atomic Coordinates (×10[4]) and Equivalent Isotropic Displacement Parameters (Å[2]×10[3]) for compound **7**. U_{eq} is defined as 1/3 of the trace of the orthogonalized U_{IJ} tensor.

Atom	*x*	*y*	*z*	U(eq)
O104	7107.9(11)	2016.6(8)	8819.5(6)	23.1(2)

Table S13. Anisotropic Displacement Parameters (Å[2]×10[3]) for **7**. The Anisotropic displacement factor exponent takes the form: $-2\pi^2[h^2a^{*2}U_{11}+2hka^*b^*U_{12}+\ldots]$.

Atom	U_{11}	U_{22}	U_{33}	U_{23}	U_{13}	U_{12}
C1	18.2(6)	14.9(6)	19.8(7)	1.7(5)	0.0(5)	-0.6(5)
C2	25.0(7)	17.3(7)	19.0(7)	3.1(6)	-0.4(5)	1.8(5)
C3	17.3(6)	31.6(8)	21.7(7)	4.7(6)	-1.9(5)	0.4(5)
C4	21.2(7)	28.0(8)	22.2(8)	5.3(6)	-0.9(5)	7.1(5)
C5	20.9(6)	14.5(6)	18.7(7)	1.2(5)	-1.0(5)	-0.6(5)
C6	20.0(6)	19.6(7)	21.3(7)	-0.2(6)	-2.3(5)	-3.8(5)
C7	23.4(7)	15.4(6)	24.4(7)	0.8(6)	0.6(5)	-4.4(5)
C8	22.5(7)	18.5(7)	15.9(7)	0.6(5)	-1.8(5)	2.8(5)
C9	21.1(6)	21.1(7)	18.6(7)	4.5(6)	0.0(5)	-1.1(5)
C10	29.2(7)	25.0(8)	28.1(8)	7.5(6)	-1.2(5)	4.4(6)
C11	29.5(7)	31.4(8)	22.3(8)	-3.0(6)	-2.1(5)	4.0(6)
C12	34.9(8)	28.9(8)	27.0(8)	-0.1(7)	-6.0(6)	-7.6(6)
C13	20.4(6)	16.1(6)	19.0(7)	2.1(5)	-0.1(5)	0.9(5)
C14	16.8(6)	16.8(6)	20.0(7)	3.2(5)	-0.6(5)	-2.4(5)
C15	17.6(6)	19.9(7)	20.4(7)	3.4(6)	-2.8(5)	0.3(5)
C16	21.7(7)	19.1(7)	23.5(8)	1.6(6)	1.4(5)	0.4(5)
C17	23.9(7)	24.4(7)	16.6(7)	1.3(6)	-0.9(5)	-3.9(5)
C18	19.8(6)	24.2(7)	22.7(7)	6.3(6)	-3.5(5)	-1.3(5)
C19	18.2(6)	18.4(7)	24.1(7)	3.8(6)	-0.1(5)	0.0(5)
N1	21.3(6)	22.3(6)	18.0(6)	3.1(5)	-0.9(4)	2.9(4)
N2	18.5(5)	14.2(5)	18.1(6)	2.5(5)	-1.6(4)	-2.1(4)
O1	18.6(5)	23.6(5)	19.3(5)	3.6(4)	0.4(3)	3.1(4)
O2	21.9(5)	16.7(5)	20.2(5)	2.6(4)	1.1(3)	-2.4(4)
O3	21.0(5)	29.9(6)	26.0(6)	7.0(4)	1.4(4)	7.0(4)
O4	18.1(5)	26.5(5)	26.3(6)	11.4(4)	1.1(4)	2.9(4)
C101	20.0(6)	15.1(6)	18.7(7)	1.1(5)	-3.0(5)	0.8(5)
C102	26.1(7)	17.5(7)	19.0(7)	2.1(6)	-3.1(5)	-2.0(5)
C103	19.6(7)	32.1(8)	21.2(8)	5.3(6)	-4.5(5)	-5.7(6)
C104	24.3(7)	24.3(7)	21.2(7)	5.1(6)	-5.0(5)	-7.1(5)
C105	23.8(6)	14.5(6)	18.0(7)	1.3(5)	-2.5(5)	2.2(5)
C106	20.7(6)	18.9(7)	20.3(7)	0.7(6)	-1.3(5)	4.6(5)
C107	23.8(7)	15.4(6)	22.5(7)	-0.3(6)	-3.9(5)	4.2(5)
C108	23.8(7)	17.6(6)	16.3(7)	-0.4(5)	-2.3(5)	-0.5(5)
C109	20.9(6)	21.6(7)	19.3(7)	6.3(6)	-3.2(5)	2.2(5)

Table S13. Anisotropic Displacement Parameters (Å^2×10^3) for **7**. The Anisotropic displacement factor exponent takes the form: $-2\pi^2[h^2a^{*2}U_{11}+2hka^*b^*U_{12}+\ldots]$.

Atom	U_{11}	U_{22}	U_{33}	U_{23}	U_{13}	U_{12}
C110	27.1(7)	33.3(9)	23.5(8)	-2.9(7)	-2.4(5)	-3.4(6)
C111	31.5(8)	21.5(7)	28.4(8)	7.5(6)	-0.2(6)	-0.6(6)
C112	26.5(8)	31.3(9)	34.8(9)	10.0(7)	4.3(6)	7.6(6)
C113	23.1(7)	15.0(6)	19.6(7)	2.8(5)	-3.9(5)	2.0(5)
C114	18.8(6)	16.4(6)	18.6(7)	2.3(5)	-3.6(5)	4.0(5)
C115	17.1(6)	19.3(7)	21.8(7)	3.8(6)	-1.4(5)	0.8(5)
C116	21.9(7)	21.5(7)	26.0(8)	0.3(6)	-7.1(5)	0.6(5)
C117	32.1(8)	29.1(8)	17.6(7)	-1.2(6)	-3.3(5)	2.2(6)
C118	29.8(8)	28.8(8)	22.5(8)	4.2(6)	1.7(5)	-2.4(6)
C119	23.9(7)	20.3(7)	24.1(8)	2.7(6)	-3.4(5)	-2.2(5)
N101	23.6(6)	23.7(6)	17.6(6)	4.7(5)	-4.2(4)	-4.9(5)
N102	22.2(5)	15.3(5)	16.0(6)	1.9(5)	-1.6(4)	3.6(4)
O101	21.7(5)	21.5(5)	17.1(5)	3.3(4)	-4.9(3)	-2.1(4)
O102	20.9(5)	16.6(5)	20.9(5)	2.7(4)	-4.8(3)	3.1(4)
O103	26.6(5)	25.4(6)	26.9(6)	5.9(4)	-8.2(4)	-8.0(4)
O104	27.1(5)	21.7(5)	20.7(5)	6.4(4)	-8.0(4)	-5.5(4)

Table S14. Bond Lengths for compound **7**.

Atom	Atom	Length/Å	Atom	Atom	Length/Å
C1	C2	1.528(2)	C101	C102	1.524(2)
C1	C5	1.5288(18)	C101	C105	1.5291(18)
C1	O1	1.4220(16)	C101	O101	1.4229(16)
C1	O2	1.4234(16)	C101	O102	1.4237(16)
C2	N1	1.4559(17)	C102	N101	1.4539(18)
C3	C4	1.504(2)	C103	C104	1.508(2)
C3	N1	1.4659(17)	C103	N101	1.4618(17)
C4	O1	1.4403(16)	C104	O101	1.4363(17)
C5	N2	1.4593(18)	C105	N102	1.4549(18)
C6	C7	1.5103(19)	C106	C107	1.5110(19)
C6	N2	1.4668(17)	C106	N102	1.4659(17)
C7	O2	1.4320(18)	C107	O102	1.4337(17)
C8	N1	1.3646(18)	C108	N101	1.3591(17)
C8	O3	1.2167(17)	C108	O103	1.2185(17)
C8	O4	1.3491(17)	C108	O104	1.3483(17)
C9	C10	1.518(2)	C109	C110	1.516(2)
C9	C11	1.520(2)	C109	C111	1.519(2)
C9	C12	1.520(2)	C109	C112	1.521(2)
C9	O4	1.4729(16)	C109	O104	1.4738(16)
C13	C14	1.506(2)	C113	C114	1.510(2)
C13	N2	1.4684(17)	C113	N102	1.4670(17)
C14	C15	1.4007(19)	C114	C115	1.4006(19)

Table S14. Bond Lengths for compound **7**.

Atom	Atom	Length/Å	Atom	Atom	Length/Å
C14	C19	1.3991(19)	C114	C119	1.3978(19)
C15	C16	1.387(2)	C115	C116	1.381(2)
C16	C17	1.395(2)	C116	C117	1.395(2)
C17	C18	1.398(2)	C117	C118	1.396(2)
C18	C19	1.389(2)	C118	C119	1.383(2)

Table S15. Bond Angles for compound **7**.

Atom	Atom	Atom	Angle/˚	Atom	Atom	Atom	Angle/˚
C2	C1	C5	110.80(11)	C102	C101	C105	110.19(11)
O1	C1	C2	112.33(11)	O101	C101	C102	111.85(11)
O1	C1	C5	105.74(10)	O101	C101	C105	106.04(10)
O1	C1	O2	110.79(11)	O101	C101	O102	110.91(11)
O2	C1	C2	106.12(10)	O102	C101	C102	106.96(10)
O2	C1	C5	111.16(11)	O102	C101	C105	110.96(11)
N1	C2	C1	111.49(11)	N101	C102	C101	111.29(11)
N1	C3	C4	108.98(11)	N101	C103	C104	108.75(11)
O1	C4	C3	110.85(12)	O101	C104	C103	110.78(12)
N2	C5	C1	113.09(11)	N102	C105	C101	112.25(11)
N2	C6	C7	108.97(11)	N102	C106	C107	108.92(11)
O2	C7	C6	111.61(11)	O102	C107	C106	111.34(11)
O3	C8	N1	124.54(13)	O103	C108	N101	123.86(13)
O3	C8	O4	125.55(13)	O103	C108	O104	125.66(12)
O4	C8	N1	109.87(11)	O104	C108	N101	110.48(11)
C10	C9	C11	110.71(12)	C110	C109	C111	111.09(12)
C10	C9	C12	110.33(13)	C110	C109	C112	112.29(13)
C11	C9	C12	113.13(12)	C111	C109	C112	110.59(13)
O4	C9	C10	101.93(11)	O104	C109	C110	109.46(12)
O4	C9	C11	110.02(12)	O104	C109	C111	102.35(11)
O4	C9	C12	110.16(12)	O104	C109	C112	110.62(12)
N2	C13	C14	114.82(11)	N102	C113	C114	112.38(11)
C15	C14	C13	121.93(12)	C115	C114	C113	120.86(12)
C19	C14	C13	119.83(12)	C119	C114	C113	120.99(12)
C19	C14	C15	118.22(13)	C119	C114	C115	118.15(13)
C16	C15	C14	121.13(12)	C116	C115	C114	121.30(13)
C15	C16	C17	120.05(13)	C115	C116	C117	119.86(13)
C16	C17	C18	119.53(13)	C116	C117	C118	119.55(14)
C19	C18	C17	120.00(13)	C119	C118	C117	120.13(14)
C18	C19	C14	121.06(13)	C118	C119	C114	120.99(13)
C2	N1	C3	112.36(11)	C102	N101	C103	113.61(11)
C8	N1	C2	120.36(11)	C108	N101	C102	120.81(11)
C8	N1	C3	123.58(12)	C108	N101	C103	125.12(12)

Table S15. Bond Angles for compound **7**.

Atom	Atom	Atom	Angle/˚	Atom	Atom	Atom	Angle/˚
C5	N2	C6	108.56(10)	C105	N102	C106	108.80(10)
C5	N2	C13	108.66(10)	C105	N102	C113	110.67(10)
C6	N2	C13	111.99(10)	C106	N102	C113	111.69(11)
C1	O1	C4	113.91(10)	C101	O101	C104	113.13(10)
C1	O2	C7	111.80(10)	C101	O102	C107	111.92(10)
C8	O4	C9	121.30(10)	C108	O104	C109	120.93(10)

Table S16. Torsion Angles for compound **7**.

A	B	C	D	Angle/˚	A	B	C	D	Angle/˚
C1	C2	N1	C3	-52.83(15)	C101	C102	N101	C103	-51.64(16)
C1	C2	N1	C8	106.24(14)	C101	C102	N101	C108	120.97(13)
C1	C5	N2	C6	-55.53(13)	C101	C105	N102	C106	-56.76(14)
C1	C5	N2	C13	-177.56(11)	C101	C105	N102	C113	-179.83(11)
C2	C1	C5	N2	170.11(10)	C102	C101	C105	N102	171.89(10)
C2	C1	O1	C4	-50.95(15)	C102	C101	O101	C104	-53.64(14)
C2	C1	O2	C7	-172.71(10)	C102	C101	O102	C107	-173.22(10)
C3	C4	O1	C1	56.31(15)	C103	C104	O101	C101	58.44(14)
C4	C3	N1	C2	57.53(16)	C104	C103	N101	C102	55.50(16)
C4	C3	N1	C8	-100.75(15)	C104	C103	N101	C108	-116.73(15)
C5	C1	C2	N1	166.50(11)	C105	C101	C102	N101	166.89(11)
C5	C1	O1	C4	-171.92(11)	C105	C101	O101	C104	-173.78(11)
C5	C1	O2	C7	-52.17(14)	C105	C101	O102	C107	-53.01(14)
C6	C7	O2	C1	57.86(14)	C106	C107	O102	C101	57.58(14)
C7	C6	N2	C5	58.55(13)	C107	C106	N102	C105	59.16(14)
C7	C6	N2	C13	178.52(11)	C107	C106	N102	C113	-178.38(11)
C10	C9	O4	C8	-175.78(12)	C110	C109	O104	C108	62.52(16)
C11	C9	O4	C8	66.73(16)	C111	C109	O104	C108	-179.58(12)
C12	C9	O4	C8	-58.66(17)	C112	C109	O104	C108	-61.73(17)
C13	C14	C15	C16	178.32(12)	C113	C114	C115	C116	178.71(12)
C13	C14	C19	C18	-177.48(12)	C113	C114	C119	C118	-178.54(12)
C14	C13	N2	C5	179.86(10)	C114	C113	N102	C105	-175.84(10)
C14	C13	N2	C6	59.94(14)	C114	C113	N102	C106	62.77(14)
C14	C15	C16	C17	-0.7(2)	C114	C115	C116	C117	0.0(2)
C15	C14	C19	C18	0.73(19)	C115	C114	C119	C118	1.1(2)
C15	C16	C17	C18	0.4(2)	C115	C116	C117	C118	0.7(2)
C16	C17	C18	C19	0.5(2)	C116	C117	C118	C119	-0.5(2)
C17	C18	C19	C14	-1.0(2)	C117	C118	C119	C114	-0.4(2)
C19	C14	C15	C16	0.15(19)	C119	C114	C115	C116	-0.91(19)
N1	C3	C4	O1	-57.94(15)	N101	C103	C104	O101	-57.41(15)
N1	C8	O4	C9	178.36(12)	N101	C108	O104	C109	-171.15(12)
N2	C6	C7	O2	-60.95(14)	N102	C106	C107	O102	-60.29(14)

Table S16. Torsion Angles for compound **7**.

A	B	C	D	Angle/˚	A	B	C	D	Angle/˚
N2	C13	C14	C15	37.22(17)	N102	C113	C114	C115	42.37(16)
N2	C13	C14	C19	-144.64(12)	N102	C113	C114	C119	-138.03(13)
O1	C1	C2	N1	48.48(15)	O101	C101	C102	N101	49.21(14)
O1	C1	C5	N2	-67.93(13)	O101	C101	C105	N102	-66.89(13)
O1	C1	O2	C7	65.10(13)	O101	C101	O102	C107	64.58(13)
O2	C1	C2	N1	-72.73(13)	O102	C101	C102	N101	-72.41(13)
O2	C1	C5	N2	52.38(14)	O102	C101	C105	N102	53.62(14)
O2	C1	O1	C4	67.54(14)	O102	C101	O101	C104	65.67(14)
O3	C8	N1	C2	13.7(2)	O103	C108	N101	C102	8.5(2)
O3	C8	N1	C3	170.33(14)	O103	C108	N101	C103	-179.80(14)
O3	C8	O4	C9	-3.9(2)	O103	C108	O104	C109	8.5(2)
O4	C8	N1	C2	-168.55(12)	O104	C108	N101	C102	-171.85(12)
O4	C8	N1	C3	-11.92(19)	O104	C108	N101	C103	-0.14(19)

Table S17. Hydrogen Atom Coordinates (Å×10[4]) and Isotropic Displacement Parameters (Å[2]×10[3]) for compound **7**.

Atom	*x*	*y*	*z*	U(eq)
H2A	3176.19	512.58	2582.15	25
H2B	1574.82	-9.8	2679.39	25
H3A	-587.47	1173.08	2208.81	28
H3B	-206.87	2317.59	1752.83	28
H4A	593.93	2995.46	2943.26	29
H4B	-1065.48	2589.58	3087.98	29
H5A	1235.69	-209.27	4174.43	22
H5B	2987.02	-111.01	4066.37	22
H6A	4228.05	1206.94	4821.22	24
H6B	3290.28	1891.7	5468.64	24
H7A	1991.02	2794.86	4429.07	25
H7B	3722.51	2997.73	4297.84	25
H10A	740.03	3819.02	-8.58	42
H10B	1917.9	4760.52	-178.62	42
H10C	1068.88	4627.03	679.93	42
H11A	4097.22	2285.68	34.67	42
H11B	3865.26	3315.87	-570.74	42
H11C	2592.25	2440.63	-420.25	42
H12A	3387.36	4421.42	1405.67	45
H12B	4271.47	4656.17	566.7	45
H12C	4645.01	3584.2	1105.35	45
H13A	3274.69	-515.69	5409.21	22
H13B	1535.65	-662.71	5535.69	22

Table S17. Hydrogen Atom Coordinates (Å×10[4]) and Isotropic Displacement Parameters (Å[2]×10[3]) for compound **7**.

**Atom**	***x***	***y***	***z***	**U(eq)**
H15	835.46	1425.48	6360.1	23
H16	926.97	1911.43	7684.3	26
H17	2523.94	972.14	8551.48	26
H18	4046.16	-446.99	8071	27
H19	3991.99	-898.85	6735.86	24
H10D	8030.24	4347.79	7320.15	25
H10E	6327.98	4705.98	7285.96	25
H10F	4435.43	3215.12	7736.32	29
H10G	5109.12	2084.79	8090.44	29
H10H	6176.22	1701.52	6824.71	28
H10I	4441.3	1914.21	6748.37	28
H10J	6124.75	5073.53	5826.82	23
H10K	7877.23	5109.27	5878.16	23
H10L	9439.07	4033.28	4995.76	24
H10M	8707.31	3353.71	4327.96	24
H10N	7572.08	2205.88	5342	25
H10O	9323.86	2165.75	5414.68	25
H11D	8284.02	3088.27	9983.71	42
H11E	8102.9	2146.19	10673.04	42
H11F	6678.14	2668.11	10264.91	42
H11G	5914.97	845.69	9952.28	41
H11H	7362.34	230.14	10265.02	41
H11I	6911.07	119.56	9360.19	41
H11J	9448.26	711.79	8937.77	47
H11K	9876.39	961.26	9826.32	47
H11L	9972.36	1900.68	9131	47
H11M	8147.91	5663.66	4472.5	23
H11N	6386.18	5689.75	4472.88	23
H115	5854.56	3679.97	3664.86	23
H116	5924.87	3189.32	2339.86	28
H117	7466.47	4112.4	1377.49	32
H118	8961.68	5507.45	1767.85	33
H119	8893.45	5987.43	3096.55	27

Refinement model description

Number of restraints - 0, number of constraints - unknown.

Details:

```
1. Twinned data refinement
 Scales: 0.5218(7)
 0.4782(7)
```

2. Fixed Uiso
At 1.2 times of:
All C(H) groups, All C(H,H) groups
At 1.5 times of:
All C(H,H,H) groups
3.a Secondary CH2 refined with riding coordinates:
C2(H2A,H2B), C3(H3A,H3B), C4(H4A,H4B), C5(H5A,H5B), C6(H6A,H6B), C7(H7A,H7B),
C13(H13A,H13B), C102(H10D,H10E), C103(H10F,H10G), C104(H10H,H10I), C105(H10J,
H10K), C106(H10L,H10M), C107(H10N,H10O), C113(H11M,H11N)
3.b Aromatic/amide H refined with riding coordinates:
C15(H15), C16(H16), C17(H17), C18(H18), C19(H19), C115(H115), C116(H116),
C117(H117), C118(H118), C119(H119)
3.c Idealized Me refined as rotating group:
C10(H10A,H10B,H10C), C11(H11A,H11B,H11C), C12(H12A,H12B,H12C), C110(H11D,H11E,
H11F), C111(H11G,H11H,H11I), C112(H11J,H11K,H11L)

4.1.2 Single Crystal X-ray structure of 7,6-spiroacetal 23

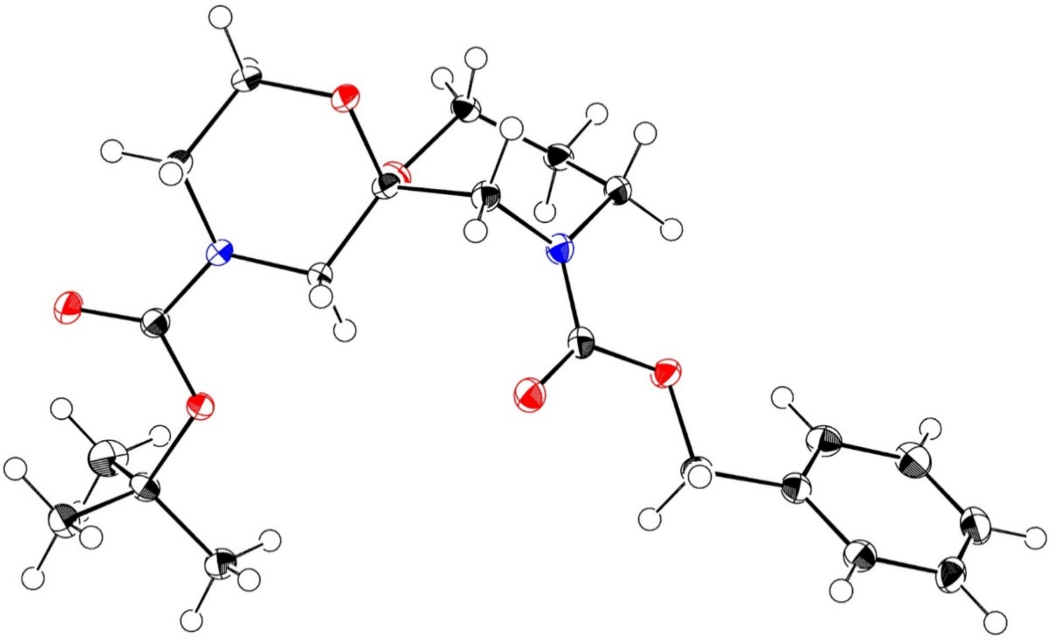

Figure S38: **ORTEP plot of 23 with ellipsoids drawn at the 50% probability level, confirming the double anomeric stabilization of the spiroacetal. Atomic displacement parameters at 100 K.**

Table S18. Crystal data and structure refinement for compound **23**.

Identification code	23
Empirical formula	$C_{21}H_{30}N_2O_6$
Formula weight	406.47
Temperature/K	100.01(10)
Crystal system	monoclinic
Space group	$P2_1/c$
a/Å	8.6370(4)
b/Å	9.7490(5)
c/Å	24.4388(11)
α/°	90
β/°	94.118(4)
γ/°	90
Volume/Å^3	2052.49(17)
Z	4
ρ_{calc}g/cm^3	1.315
μ/mm^{-1}	0.795
F(000)	872.0
Crystal size/mm^3	0.223 × 0.151 × 0.06
Radiation	Cu Kα (λ = 1.54184)
2Θ range for data collection/°	7.254 to 146.186
Index ranges	–10 ≤ h ≤ 10, –11 ≤ k ≤ 11, –30 ≤ l ≤ 23

Reflections collected	5829
Independent reflections	5829 [R_{int} = ?, R_{sigma} = 0.0337]
Data/restraints/parameters	5829/0/266
Goodness-of-fit on F^2	1.032
Final R indexes [I>=2σ (I)]	R_1 = 0.0518, wR_2 = 0.1375
Final R indexes [all data]	R_1 = 0.0619, wR_2 = 0.1449
Largest diff. peak/hole / e Å^{-3}	0.33/–0.32

Table S19. Fractional Atomic Coordinates (×10^4) and Equivalent Isotropic Displacement Parameters (Å^2×10^3) for compound **23**. U_{eq} is defined as 1/3 of the trace of the orthogonalized U_{IJ} tensor.

**Atom**	***x***	***y***	***z***	**U(eq)**
C1	7502(2)	7514(2)	5049.5(8)	14.5(4)
C2	6531(2)	8462(2)	4659.6(8)	14.8(4)
C3	8894(2)	9085(2)	4226.9(8)	18.5(4)
C4	9786(2)	8068(2)	4595.8(8)	18.4(4)
C5	7009(2)	7613(2)	5639.5(8)	17.3(4)
C6	6141(3)	5338(2)	5958.1(8)	18.9(4)
C7	6710(2)	4449(2)	5498.8(8)	18.7(4)
C8	7975(3)	5086(2)	5179.2(8)	17.7(4)
C9	6483(2)	9021(2)	3667.2(8)	17.0(4)
C10	3887(2)	9143(2)	3203.5(8)	18.5(4)
C11	4196(3)	8128(3)	2753.0(9)	28.9(5)
C12	4026(3)	10623(2)	3021.0(9)	24.2(5)
C13	2300(3)	8893(2)	3419.2(9)	22.3(5)
C14	4318(2)	7320(2)	5801.3(8)	16.5(4)
C15	1871(2)	7165(2)	6156.1(8)	18.5(4)
C16	989(2)	6228(2)	6511.3(8)	18.7(4)
C17	-94(3)	6796(2)	6838.2(8)	21.1(5)
C18	-953(3)	5970(3)	7166.9(8)	26.3(5)
C19	-722(3)	4567(3)	7172.9(9)	28.9(5)
C20	360(3)	3990(3)	6850.2(10)	27.1(5)
C21	1216(3)	4811(2)	6514.2(9)	22.5(5)
N1	7287(2)	8635.4(18)	4146.1(7)	16.1(4)
N2	5729(2)	6733.6(18)	5766.4(7)	17.2(4)
O1	9090.4(16)	7934.9(16)	5109.4(6)	17.4(3)
O2	7362.2(17)	6176.4(14)	4828.1(5)	16.6(3)
O3	7121.9(18)	9424.6(18)	3268.4(6)	22.8(4)
O4	4946.7(17)	8858.0(16)	3693.2(5)	17.8(3)
O5	3965.9(19)	8454.2(16)	5625.0(6)	21.9(4)
O6	3333.6(17)	6516.5(15)	6063.1(6)	18.7(3)

Table S20. Anisotropic Displacement Parameters (Å^2×10^3) for compound **23**. The Anisotropic displacement factor exponent takes the form: $-2\pi^2[h^2a^{*2}U_{11}+2hka^*b^*U_{12}+…]$.

Atom	U_{11}	U_{22}	U_{33}	U_{23}	U_{13}	U_{12}
C1	11.7(9)	14.8(9)	17.2(9)	-0.2(7)	1.8(7)	-0.5(8)
C2	12.9(9)	16.9(10)	14.9(8)	3.4(7)	2.8(7)	1.4(8)
C3	13.0(10)	22.9(11)	19.9(9)	3.6(8)	2.8(7)	-1.5(8)
C4	11.2(9)	24.2(11)	20.1(9)	2.6(8)	2.2(7)	1.1(9)
C5	16.0(10)	19.5(10)	16.2(9)	0.3(8)	0.2(7)	-4.2(8)
C6	17.5(11)	18.2(10)	21.5(9)	7.3(8)	4.0(8)	2.5(8)
C7	15.7(10)	16.0(10)	24.2(10)	5.4(8)	1.0(8)	1.5(8)
C8	16.7(10)	16.6(10)	19.6(9)	2.2(8)	0.3(7)	2.6(8)
C9	16.1(10)	17.5(10)	17.7(9)	0.1(8)	2.3(8)	0.1(8)
C10	16.9(10)	23.9(11)	14.0(8)	0.3(8)	-3.4(7)	-0.6(9)
C11	28.8(13)	35.9(14)	21.6(10)	-8.6(10)	-0.5(9)	1.6(11)
C12	22.4(11)	27.0(12)	23.0(10)	6.7(9)	-0.1(8)	-0.9(10)
C13	15.7(10)	28.9(12)	22.0(10)	2.9(9)	-1.3(8)	-4.5(9)
C14	18.4(10)	17.6(10)	13.4(8)	0.7(7)	1.5(7)	-1.8(8)
C15	14.9(10)	19.5(10)	21.0(9)	1.7(8)	1.2(8)	1.1(9)
C16	16.8(10)	22.9(11)	16.0(9)	-0.1(8)	-1.6(7)	-2.6(9)
C17	18.3(11)	26.8(11)	18.0(9)	-1.6(9)	-0.8(8)	-0.7(9)
C18	18.9(11)	45.0(15)	15.0(9)	-0.4(9)	0.8(8)	-4.0(10)
C19	20.5(11)	44.0(15)	21.5(10)	10.6(10)	-3.6(9)	-11.9(11)
C20	23.8(11)	26.3(12)	30.4(11)	6.3(9)	-3.6(9)	-7.4(10)
C21	19.5(11)	23.0(11)	24.4(10)	-0.6(9)	-1.8(8)	-1.1(9)
N1	12.1(8)	20.0(9)	16.4(8)	2.5(7)	3.0(6)	0.7(7)
N2	17.9(9)	14.8(8)	19.1(8)	3.6(7)	3.2(6)	-0.3(7)
O1	12.1(7)	22.6(8)	17.3(6)	2.7(6)	0.8(5)	-3.1(6)
O2	18.8(8)	13.6(7)	16.9(6)	0.6(6)	-1.7(5)	1.4(6)
O3	18.4(8)	33.7(9)	16.8(7)	3.9(6)	4.2(6)	-0.8(7)
O4	13.7(7)	25.2(8)	14.2(6)	3.2(6)	-0.8(5)	-1.1(6)
O5	23.4(8)	17.6(8)	25.3(7)	5.0(6)	6.3(6)	2.8(6)
O6	15.4(8)	17.3(7)	23.8(7)	3.6(6)	4.4(6)	0.3(6)

Table S21. Bond Lengths for compound **23**.

Atom	Atom	Length/Å	Atom	Atom	Length/Å
C1	C2	1.533(3)	C10	C11	1.518(3)
C1	C5	1.535(3)	C10	C12	1.517(3)
C1	O1	1.429(2)	C10	C13	1.523(3)
C1	O2	1.414(2)	C10	O4	1.480(2)
C2	N1	1.466(2)	C14	N2	1.354(3)
C3	C4	1.513(3)	C14	O5	1.217(3)

Table S21. Bond Lengths for compound **23**.

Atom	Atom	Length/Å	Atom	Atom	Length/Å
C3	N1	1.456(3)	C14	O6	1.351(3)
C4	O1	1.436(2)	C15	C16	1.505(3)
C5	N2	1.450(3)	C15	O6	1.445(3)
C6	C7	1.527(3)	C16	C17	1.388(3)
C6	N2	1.474(3)	C16	C21	1.395(3)
C7	C8	1.521(3)	C17	C18	1.389(3)
C8	O2	1.443(2)	C18	C19	1.383(4)
C9	N1	1.370(3)	C19	C20	1.384(4)
C9	O3	1.219(3)	C20	C21	1.396(3)
C9	O4	1.342(3)			

Table S22. Bond Angles for compound **23.**

Atom	Atom	Atom	Angle/˚	Atom	Atom	Atom	Angle/˚
C2	C1	C5	111.69(16)	O5	C14	N2	124.4(2)
O1	C1	C2	111.65(16)	O5	C14	O6	123.0(2)
O1	C1	C5	102.40(15)	O6	C14	N2	112.61(17)
O2	C1	C2	106.85(15)	O6	C15	C16	108.02(17)
O2	C1	C5	113.30(16)	C17	C16	C15	118.7(2)
O2	C1	O1	111.04(15)	C17	C16	C21	119.5(2)
N1	C2	C1	110.14(16)	C21	C16	C15	121.8(2)
N1	C3	C4	108.84(17)	C16	C17	C18	120.8(2)
O1	C4	C3	110.67(17)	C19	C18	C17	119.8(2)
N2	C5	C1	115.52(17)	C18	C19	C20	119.9(2)
N2	C6	C7	111.94(16)	C19	C20	C21	120.7(2)
C8	C7	C6	115.24(18)	C16	C21	C20	119.4(2)
O2	C8	C7	111.10(17)	C3	N1	C2	113.45(16)
O3	C9	N1	122.73(19)	C9	N1	C2	122.24(17)
O3	C9	O4	125.58(19)	C9	N1	C3	116.94(17)
O4	C9	N1	111.66(17)	C5	N2	C6	116.29(18)
C11	C10	C13	110.87(19)	C14	N2	C5	117.84(18)
C12	C10	C11	112.61(19)	C14	N2	C6	124.59(18)
C12	C10	C13	110.26(19)	C1	O1	C4	113.28(14)
O4	C10	C11	109.47(18)	C1	O2	C8	115.74(15)
O4	C10	C12	111.03(17)	C9	O4	C10	119.71(16)
O4	C10	C13	102.12(15)	C14	O6	C15	114.04(16)

Table S23. Torsion Angles for compound **23**.

A	B	C	D	Angle/˚	A	B	C	D	Angle/˚
C1	C2	N1	C3	-53.4(2)	C18	C19	C20	C21	-0.5(3)
C1	C2	N1	C9	157.56(19)	C19	C20	C21	C16	0.9(3)
C1	C5	N2	C6	88.1(2)	C21	C16	C17	C18	-0.1(3)
C1	C5	N2	C14	-104.2(2)	N1	C3	C4	O1	-57.4(2)
C2	C1	C5	N2	85.8(2)	N1	C9	O4	C10	-176.43(17)
C2	C1	O1	C4	-54.2(2)	N2	C6	C7	C8	50.5(2)
C2	C1	O2	C8	-169.94(16)	N2	C14	O6	C15	174.15(16)
C3	C4	O1	C1	57.9(2)	O1	C1	C2	N1	50.4(2)
C4	C3	N1	C2	56.8(2)	O1	C1	C5	N2	-154.62(17)
C4	C3	N1	C9	-152.40(18)	O1	C1	O2	C8	68.1(2)
C5	C1	C2	N1	164.40(16)	O2	C1	C2	N1	-71.2(2)
C5	C1	O1	C4	-173.86(16)	O2	C1	C5	N2	-35.0(2)
C5	C1	O2	C8	-46.5(2)	O2	C1	O1	C4	64.9(2)
C6	C7	C8	O2	-72.2(2)	O3	C9	N1	C2	165.4(2)
C7	C6	N2	C5	-70.5(2)	O3	C9	N1	C3	17.4(3)
C7	C6	N2	C14	122.7(2)	O3	C9	O4	C10	1.7(3)
C7	C8	O2	C1	94.7(2)	O4	C9	N1	C2	-16.4(3)
C11	C10	O4	C9	64.8(3)	O4	C9	N1	C3	-164.46(17)
C12	C10	O4	C9	-60.1(2)	O5	C14	N2	C5	16.1(3)
C13	C10	O4	C9	-177.64(18)	O5	C14	N2	C6	-177.27(19)
C15	C16	C17	C18	179.04(19)	O5	C14	O6	C15	-4.9(3)
C15	C16	C21	C20	-179.71(19)	O6	C14	N2	C5	-162.92(16)
C16	C15	O6	C14	-172.81(16)	O6	C14	N2	C6	3.7(3)
C16	C17	C18	C19	0.5(3)	O6	C15	C16	C17	153.90(18)
C17	C16	C21	C20	-0.6(3)	O6	C15	C16	C21	-27.0(3)
C17	C18	C19	C20	-0.2(3)					

Table S24. Hydrogen Atom Coordinates (Å×10[4]) and Isotropic Displacement Parameters (Å[2]×10[3]) for compound **23**.

Atom	*x*	*y*	*z*	U(eq)
H2A	5483.91	8063.61	4580.91	18
H2B	6411.45	9366.27	4835.72	18
H3A	8941.61	10007.3	4397.22	22
H3B	9360.03	9141.36	3869.06	22
H4A	9795.86	7163.61	4411.93	22
H4B	10873.77	8380.2	4663.21	22
H5A	7916.79	7386.91	5894.03	21
H5B	6718.25	8575.4	5710.71	21
H6A	5220.89	4901.94	6103.85	23

Table S24. Hydrogen Atom Coordinates (Å×10^4) and Isotropic Displacement Parameters (Å^2×10^3) for compound **23**.

Atom	*x*	*y*	*z*	U(eq)
H6B	6964.23	5395.96	6260.65	23
H7A	7104.86	3573.78	5660.05	22
H7B	5812.66	4228.94	5238.59	22
H8A	8804.9	5454.16	5439	21
H8B	8437.86	4372.06	4953.58	21
H11A	3415.18	8245.16	2445.44	43
H11B	4141.74	7191.75	2895.88	43
H11C	5231.89	8293.49	2626.94	43
H12A	5062.51	10778.28	2894.17	36
H12B	3864.48	11234.6	3329.95	36
H12C	3240.53	10811.53	2720.86	36
H13A	1488.37	9066.29	3126.18	34
H13B	2157.2	9511.23	3727.72	34
H13C	2231.94	7939.45	3542.6	34
H15A	1267	7326.47	5802.11	22
H15B	2055.18	8060.42	6340.8	22
H17	-249.35	7760.87	6837.17	25
H18	-1697.42	6368.01	7386.83	32
H19	-1304.5	3998.57	7398.08	35
H20	521.51	3026.38	6857.6	33
H21	1946.06	4408.6	6289.62	27

Refinement model description

Number of restraints - 0, number of constraints - unknown.

Details:

```
1. Twinned data refinement
 Scales: 0.7684(11)
 0.2316(11)
2. Fixed Uiso
 At 1.2 times of:
 All C(H) groups, All C(H,H) groups
 At 1.5 times of:
 All C(H,H,H) groups
3.a Secondary CH2 refined with riding coordinates:
 C2(H2A,H2B), C3(H3A,H3B), C4(H4A,H4B), C5(H5A,H5B), C6(H6A,H6B),
C7(H7A,H7B),
 C8(H8A,H8B), C15(H15A,H15B)
3.b Aromatic/amide H refined with riding coordinates:
```

```
 C17(H17), C18(H18), C19(H19), C20(H20), C21(H21)
3.c Idealized Me refined as rotating group:
 C11(H11A,H11B,H11C), C12(H12A,H12B,H12C), C13(H13A,H13B,H13C)
```

5 References

[1] Bowman, W. R.; Bridge, C. F.; Brookes, P.; Cloonan, M. O.; Leach, D. C. Cascade Radical Synthesis of Heteroarenes *via* Iminyl Radicals. *J. Chem. Soc. Perkin Trans. 1* **2002**, 58–68.

[2] Yamada, R.; Fukuyama, T.; Yokoshima, S. Synthetic Studies of the Daphniphyllum Alkaloids: A Cooperative Reaction of Proximal Functional Groups Forming a Tetracyclic System. *Org. Lett.* **2018**, *20*, 4504–4506.

[3] Kurosu, M.; Lin, M.-H.; Kishi, Y. Fe/Cr- and Co/Cr-Mediated Catalytic Asymmetric 2-Haloallylations of Aldehydes. *J. Am. Chem. Soc.* **2004**, *126*, 12248–12249.

[4] Ichikawa, M.; Takahashi, M.; Aoyagi, S.; Kibayashi, C. Total Synthesis of (–)-Incarvilline, (+)-Incarvine C, and (–)-Incarvillateine. *J. Am. Chem. Soc.* **2004**, *126*, 16553–16558.

[5] Han, S.-J.; Doi, R.; Stoltz, B. M. Nickel-Catalyzed Intramolecular C–O Bond Formation: Synthesis of Cyclic Enol Ethers. *Angew. Chem. Int. Ed.* **2016**, *55*, 7437–7440.

[6] Bera, S. and Panda, G. I_2-Mediated Diversity Oriented Diastereoselective Synthesis of Amino Acid Derived *trans*-2,5-Disubstituted Morpholines, Piperazines, and Thiomorpholines. *ACS Comb. Sci.*, **2012**, *14*, 1–4.

[7] Doveston, R. G.; Tosatti, P.; Dow, M.; Foley, D. J.; Li, H. Y.; Campbell, A. J.; House, D.; Churcher, I.; Marsden, S. P.; Nelson, A. A Unified Lead-Oriented Synthesis of Over Fifty Molecular Scaffolds. *Org. Biomol. Chem.*, **2015**, *13*, 859–865.

[8] Amegadzie, A. K.; Beck, J. P.; Gardinier, K. M.; Hembre, E. J.; Ruble, J. C.; Savin, K. A.; Wakefield, B. D. Thiazolopyridinone Derivates as Mch Receptor Antagonists. WO2006066174, June 22, 2006.

[9] Goyal, V.; Gahtori, J.; Narani, A.; Gupta, P.; Bordoloi, A.; Natte, K. Commercial Pd/C-Catalyzed *N*-Methylation of Nitroarenes and Amines Using Methanol as Both C1 and H_2 Source. *J. Org. Chem.* **2019**, *84*, 15389–15398.

[10] Vogt, M.; Stumpfe, D.; Geppert, H.; Bajorath, J. Scaffold Hopping Using Two-Dimensional Fingerprints: True Potential, Black Magic, or a Hopeless Endeavor? Guidelines for Virtual Screening. *J. Med. Chem.* **2010**, *53*, 5707–5715.

[11] Tom, N. J.; Simon, W. M.; Frost, H. N.; Ewing, M. Deprotection of a Primary Boc Group under Basic Conditions. *Tetrahedron Lett.* **2004**, *45*, 905–906.

[12] Comins, D. L.; Joseph, S. P. Dimethylformamide. In *Encyclopedia of Reagents for Organic Synthesis*; Charette, A. B., Ed.; John Wiley & Sons, Ltd: Hoboken (USA), 1999; pp 1–4.

[13] Wiberg, K. B.; Bailey, W. F.; Lambert, K. M.; Stempel, Z. D. The Anomeric Effect: It's Complicated. *J. Org. Chem.*, **2018**, *83*, 5242–5255.

[14] Métro, T. X.; Pardo, D. G.; Cossy, J. Highly Enantioselective Synthesis of Linear β-Amino Alcohols. *Chem. Eur. J.* **2009**, *15*, 1064–1070.

[15] Métro, T. X.; Duthion, B.; Pardo, D. G.; Cossy, J. Rearrangement of β-Amino Alcohols via Aziridiniums: A Review. *Chem. Soc. Rev.* **2009**, *39*, 89–102.

[16] Williams, D. B. G.; Lawton, M. Drying of Organic Solvents: Quantitative Evaluation of the Efficiency of Several Desiccants. *J. Org. Chem.* **2010**, *75*, 8351–8354.

[17] Hemelaere, R.; Desroches, J.; Paquin, J.-F. Introduction of the 4,4,4-Trifluorobut-2-ene Chain Exploiting a Regioselective Tsuji–Trost Reaction Catalyzed by Palladium Nanoparticles. *Org. Lett.* **2015**, *17*, 1770–1773.

[18] CrysAlisPro, Rigaku Oxford Diffraction, 2015.

[19] Sheldrick, G. M. Crystal structure refinement with *SHELXL*. *Acta Cryst.*, **2015**, *A71*, 3-8.

[20] Sheldrick, G. M. Crystal structure refinement with *SHELXL*. *Acta Cryst.* **2015**, *C71*, 3-8.

[21] Dolomanov, O. V.; Bourhis, L. J.; Gildea, R. J.; Howard, J. A. K.; Puschmann, H. *OLEX2*: a complete structure solution, refinement and analysis program. *J. Appl. Crystallogr.* **2009**, *42*, 339-341.

[22] Farrugia, L. J. WinGX and ORTEP for Windows: an update. *J. Appl. Cryst.* **2012**, *45*, 849-854.

[23] Médoc, M.; Sobrio, F. Nucleophilic Radiofluorination at Room Temperature via Aziridinium Intermediates. *RSC Adv.* **2014**, *4*, 35371–35374.

[24] Huy, P. H.; Koskinen, A. M. P. Efficient, Stereodivergent Access to 3-Piperidinols by Traceless $P(OEt)_3$ Cyclodehydration. *Org. Lett.* **2013**, *15*, 5178–5181.

[25] Li, X.; Chen, N.; Xu, J. Microwave-Assisted CuCl-Catalyzed Three-Component Reactions of Alkynes, Aldehydes, and Amino Alcohols. *Synthesis* **2019**, *51*, 3336–3344.

[26] Loftus, F. The Synthesis of Some 2-Substituted Morpholines. *Synth. Commun.* **1980**, *10*, 59–73.

[27] Araki, K.; Kuroda, T.; Uemori, S.; Moriguchi, A.; Ikeda, Y.; Hirayama, F.; Yokoyama, Y.; Iwao, E.; Yakushiji, T. Quinolone Antimicrobial Agents Substituted with Morpholines at the 7-Position. Syntheses and Structure-Activity Relationships. *J. Med. Chem.* **1993**, *36*, 1356–1363.

[28] Wilson, A. A.; Garcia, A.; Houle, S.; Sadovski, O.; Vasdev, N. Synthesis and Application of Isocyanates Radiolabeled with Carbon-11. *Chem. Eur. J.* **2011**, *17*, 259–264.

[29] Doak, B. C.; Over, B.; Giordanetto, F.; Kihlberg, J. Oral Druggable Space Beyond the Rule of 5: Insights from Drugs and Clinical Candidates. *Chem. Biol.* **2014**, *21*, 1115–1142.

[30] Stocks, M. J.; Wilden, G. R. H. H.; Pairaudeau, G.; Perry, M. W. D. D.; Steele, J.; Stonehouse, J. P. A Practical Method for Targeted Library Design Balancing Lead-like Properties with Diversity. *ChemMedChem* **2009**, *4*, 800–808.

[31] Jamieson, C.; Moir, E. M.; Rankovic, Z.; Wishart, G. Medicinal Chemistry of hERG Optimizations: Highlights and Hang-Ups. *J. Med. Chem.* **2006**, *49*, 5029–5046.
