# Supplementary material for: Short Scalable Route to Bis-morpholine Spiroacetals and Oxazepane Analogues: Useful 3D-Scaffolds for Compound Library Assembly
Source: J Org Chem. 2025 Feb 10;90(7):2652–61. doi: 10.1021/acs.joc.4c02690 (PMC11852203; doi:10.1021/acs.joc.4c02690)

A short scalable route to bis-morpholine spiroacetals and oxazepane analogues: useful 3D-scaffolds for compound library assembly

Supporting Information – NMR Spectra

Daniel Kovari,[a,c] Louise Male,[a] Kimberley A. Roper,[b] Christian P. Mang,[c]
Oliver Kunz,[c] Liam R. Cox[a,*]

[a]School of Chemistry, The University of Birmingham, Edgbaston, Birmingham, B15 2TT, United Kingdom, [b]School of Pharmacy, The University of Birmingham, Edgbaston, Birmingham, B15 2TT, United Kingdom, [c]Analyticon Discovery GmbH, Hermannswerder 17, 14473, Potsdam, Germany

Contents

1.1 Stoltz chemistry S3

1.2 Aminoalcohols S20

1.3 Chloromethyl-substituted heterocycles S23

1.4 Enol ethers S31

1.5 Spiroacetals S35

1.6 Library compounds S111

1.1 Stoltz chemistry

(*Z*)-2-iodo-3-phenylacrylaldehyde (S3) [^{1}H-NMR data: 400 MHz, $CDCl_3$; $^{13}C\{^1H\}$-NMR data: 101 MHz, $CDCl_3$]:

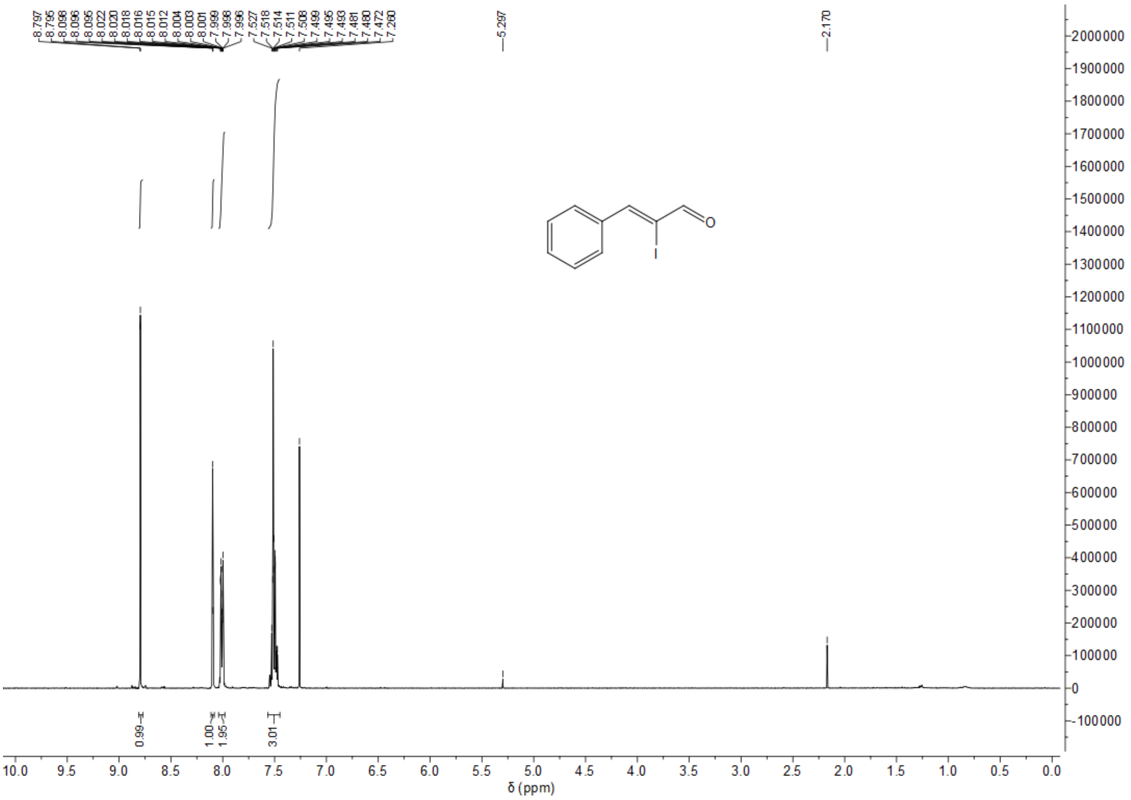

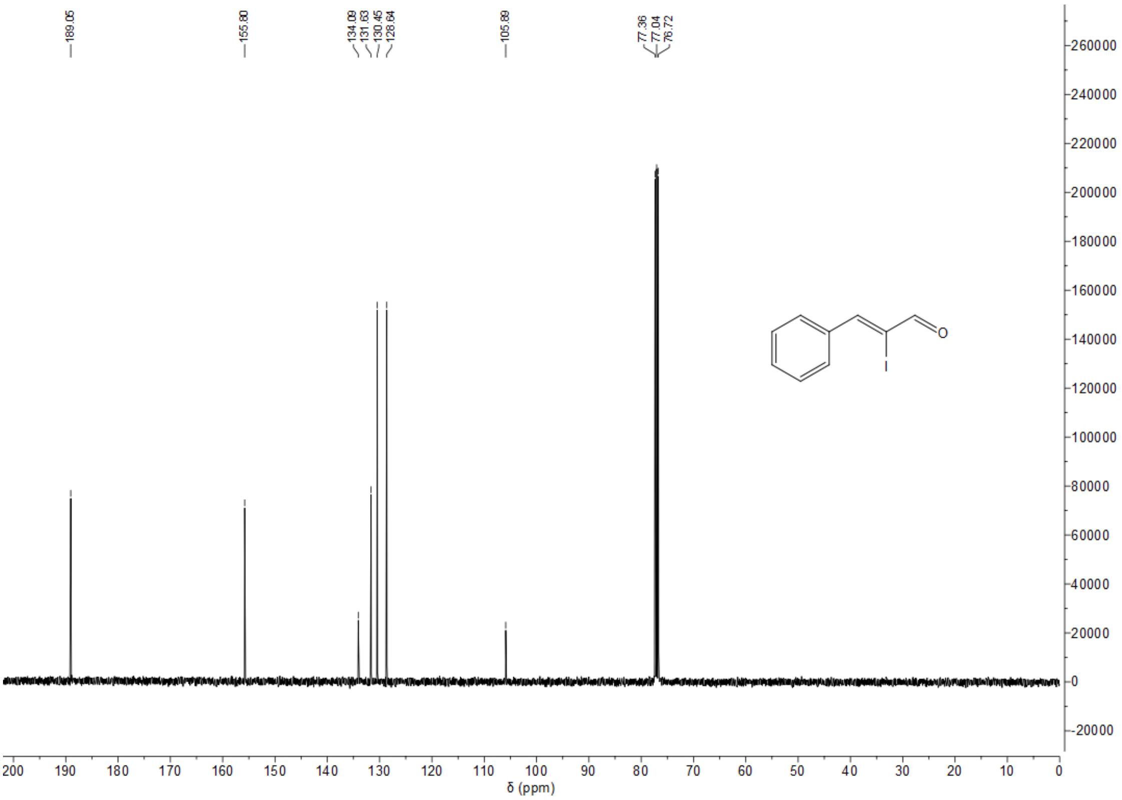

(*Z*)-2-iodo-3-phenylprop-2-en-1-ol (S4) [^{1}H-NMR data: 400 MHz, $CDCl_3$; $^{13}C\{^{1}H\}$-NMR data: 101 MHz, $CDCl_3$]:

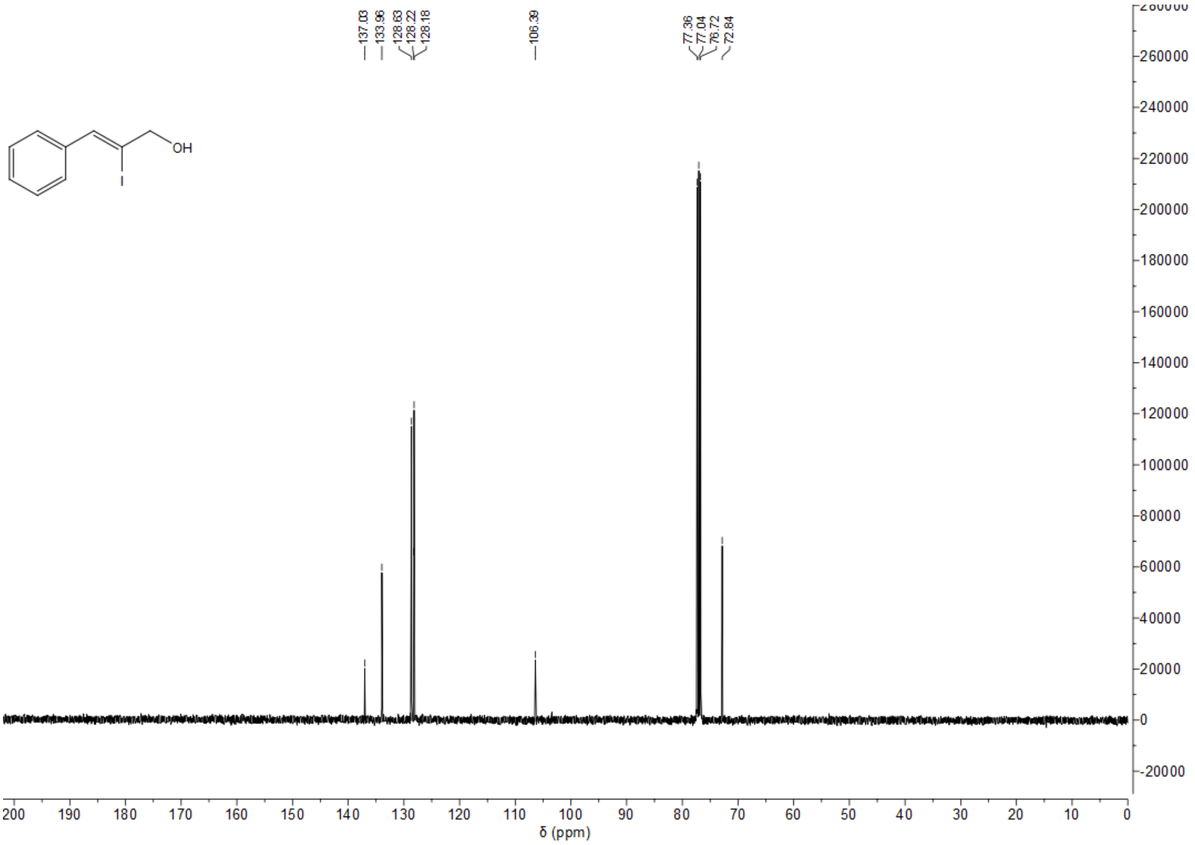

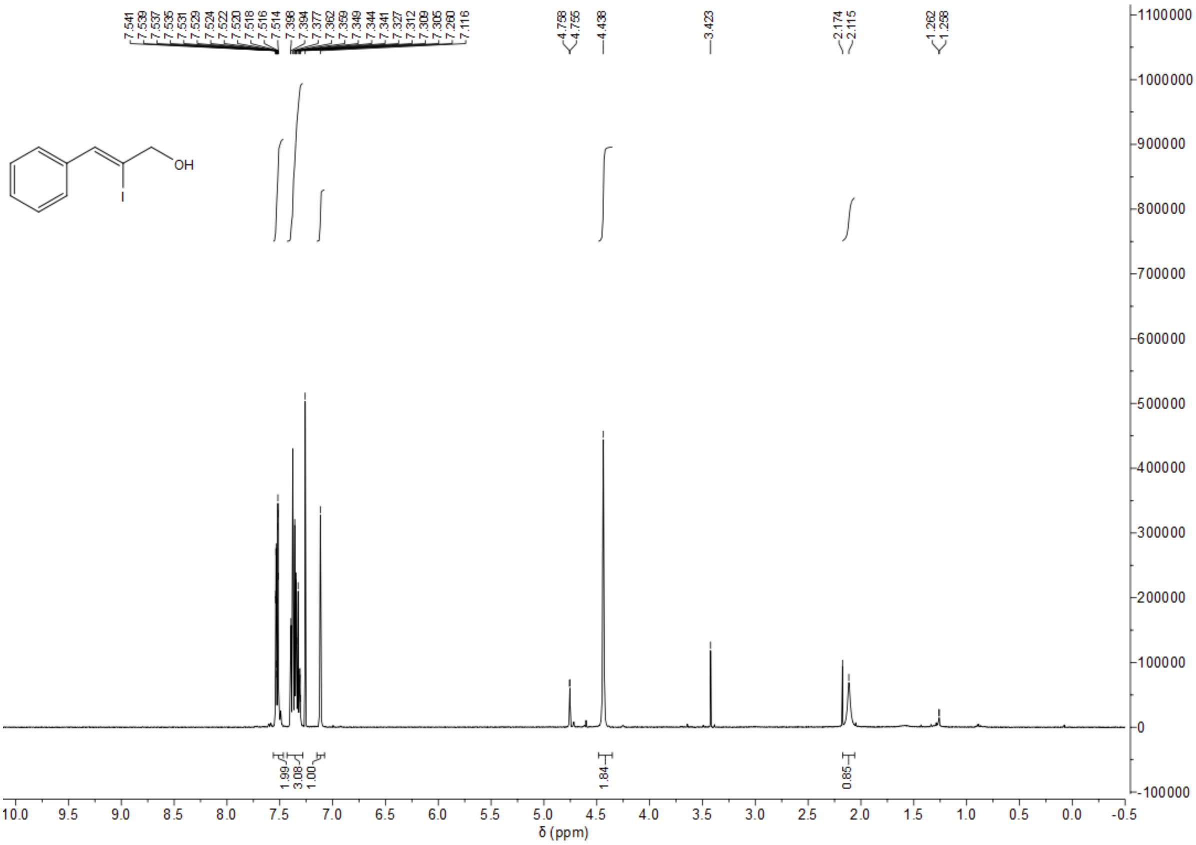

(*Z*)-(3-bromo-2-iodoprop-1-en-1-yl)benzene (S1) [^{1}H-NMR data: 400 MHz, CDCl$_3$; ^{13}C{^{1}H}-NMR data: 101 MHz, CDCl$_3$]:

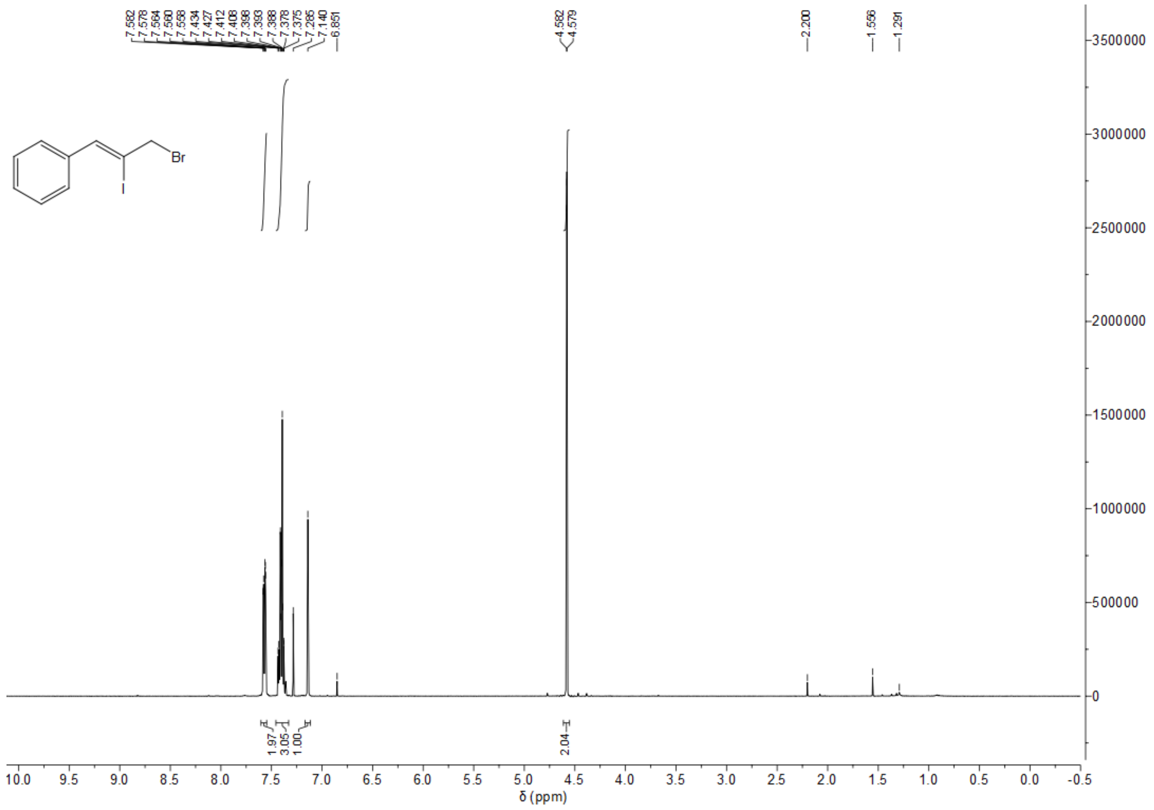

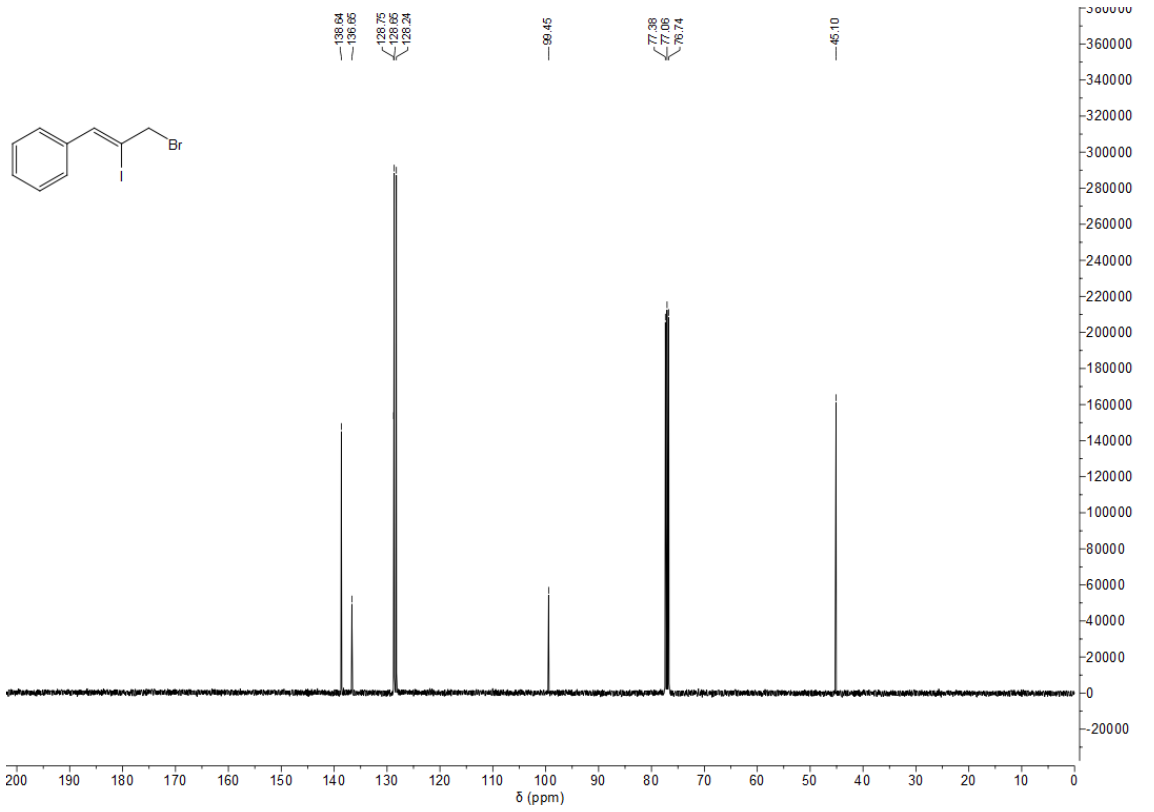

(*Z*)-2-((2-iodo-3-phenylallyl)(methyl)amino)ethan-1-ol (S5) [^{1}H-NMR data: 400 MHz, $CDCl_3$; ^{13}C{^{1}H}-NMR data: 101 MHz, $CDCl_3$]:

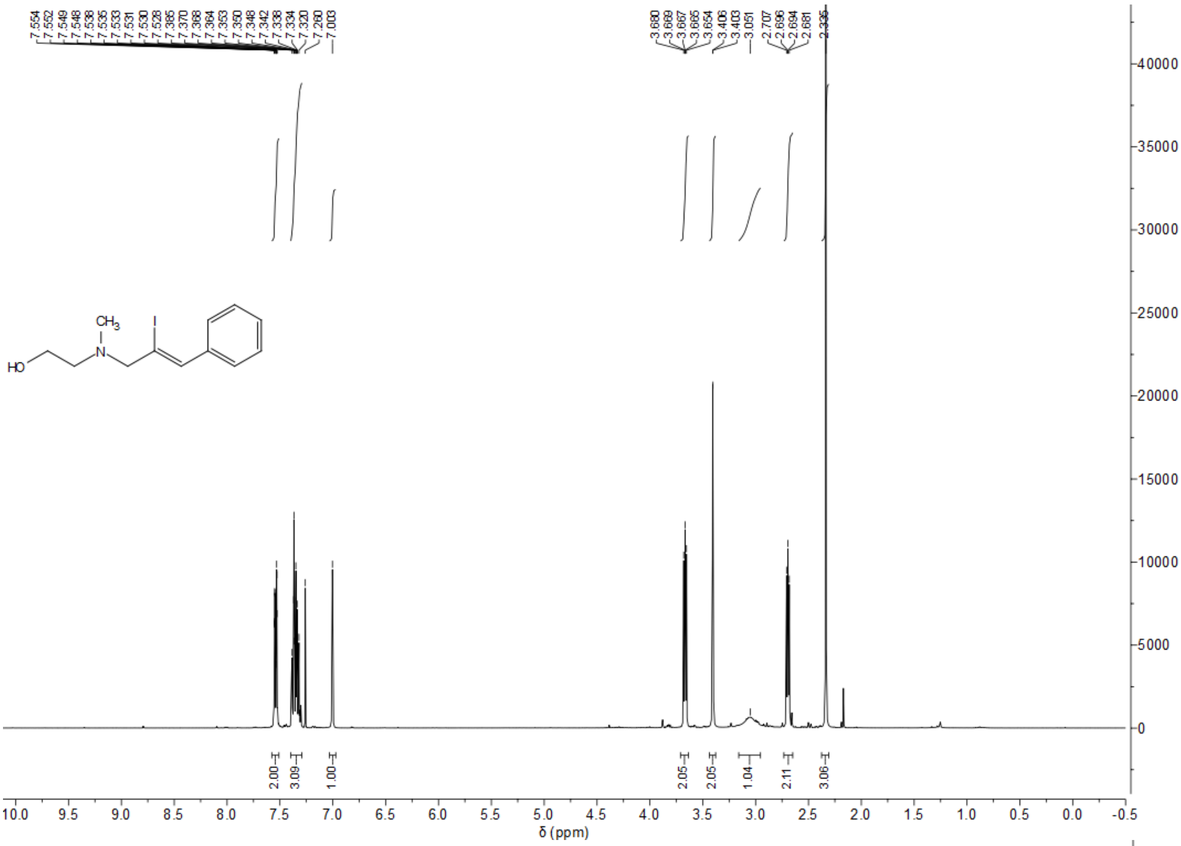

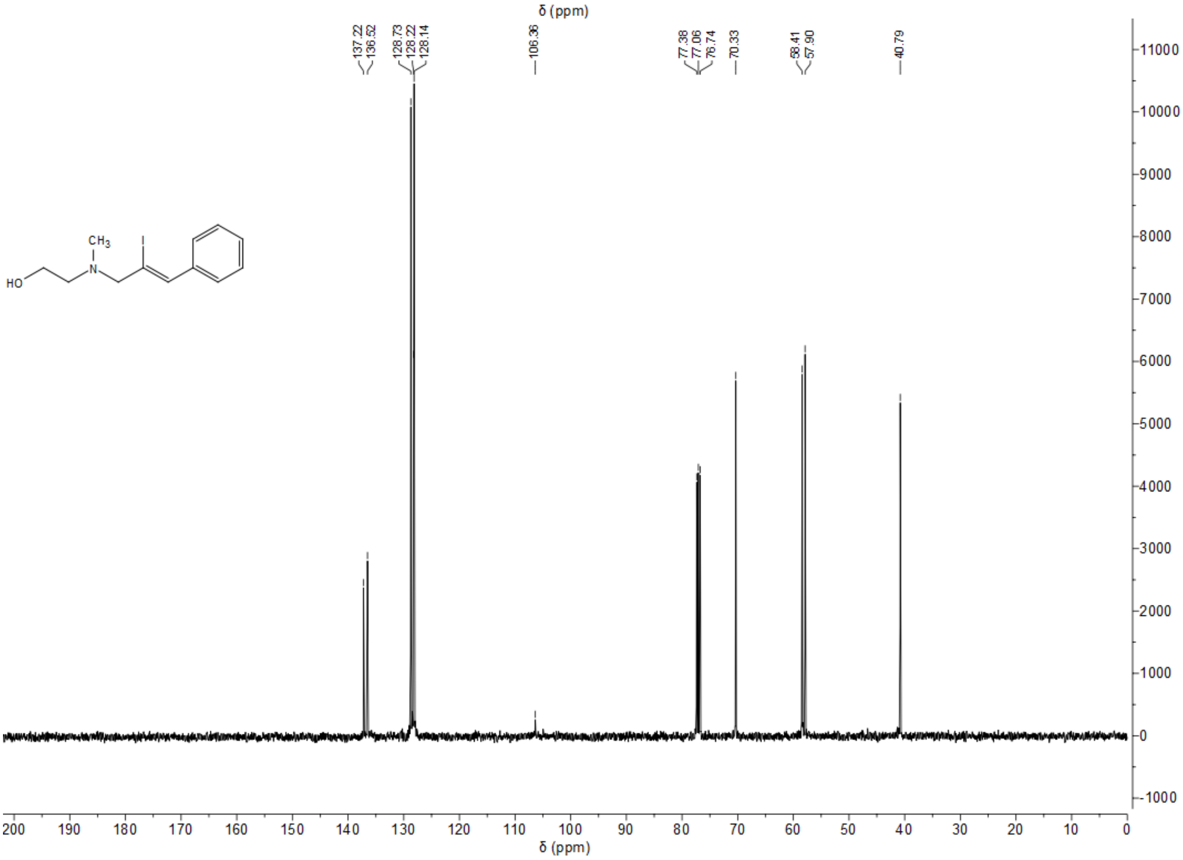

(*Z*)-2-(benzyl(2-iodo-3-phenylallyl)amino)ethan-1-ol (S6) [^{1}H-NMR data: 400 MHz, $CDCl_3$; $^{13}C\{^1H\}$-NMR data: 101 MHz, $CDCl_3$]:

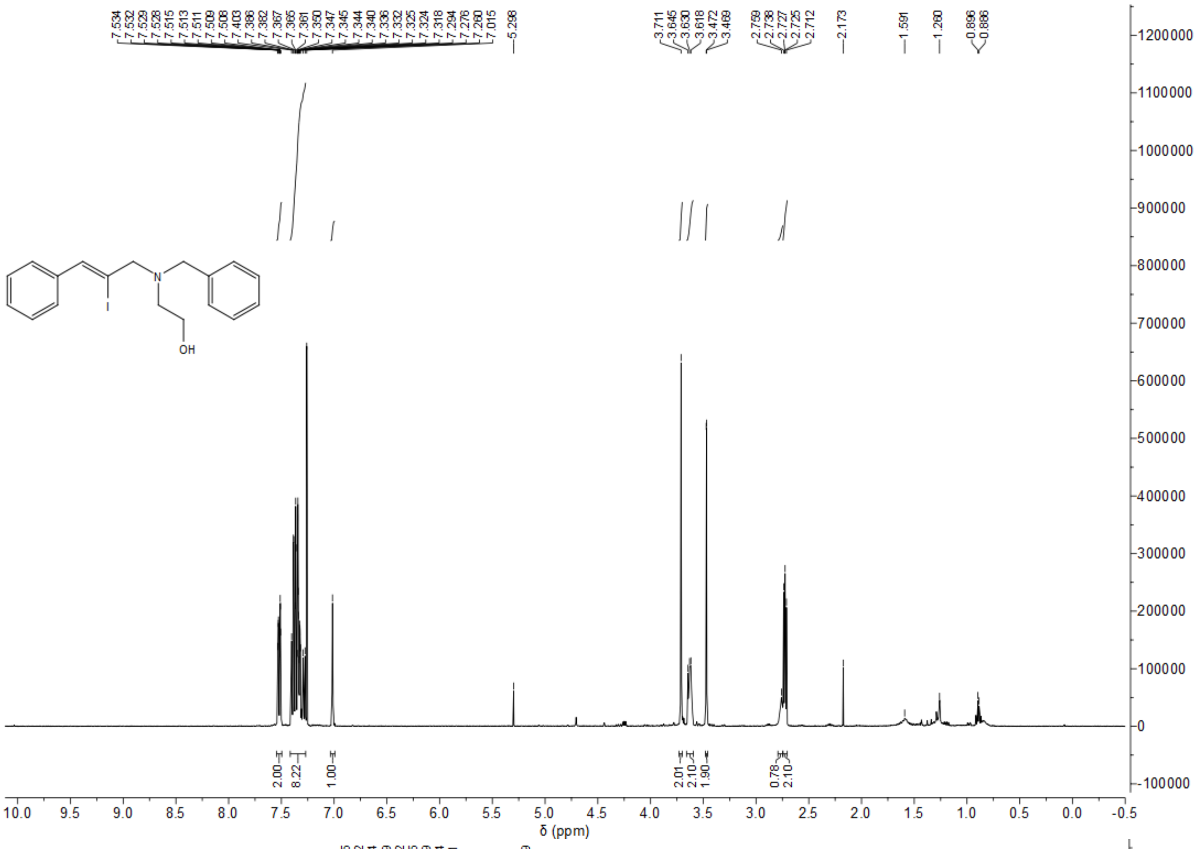

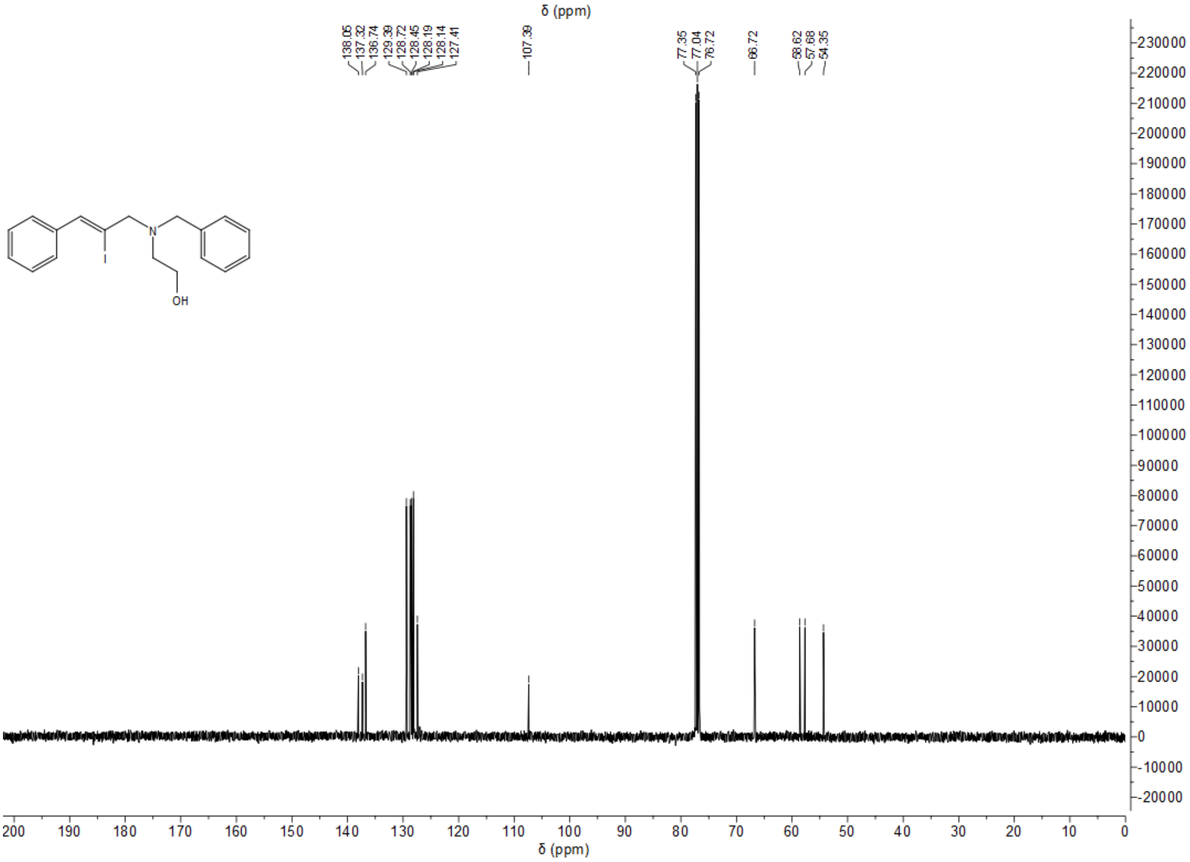

(*Z*)-2-((2-iodo-3-phenylallyl)amino)ethan-1-ol (S7) [^{1}H-NMR data: 400 MHz, CDCl$_3$; ^{13}C{^{1}H}-NMR data: 101 MHz, CDCl$_3$]:

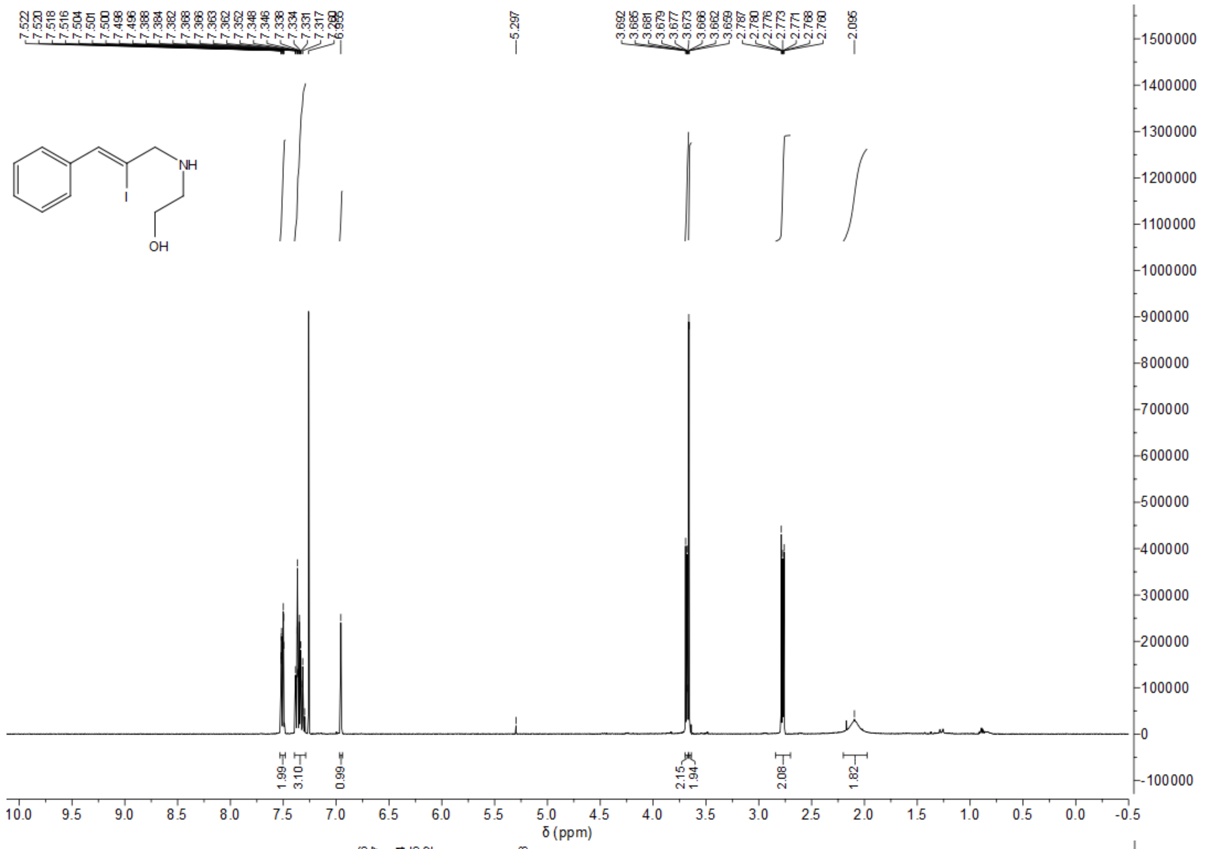

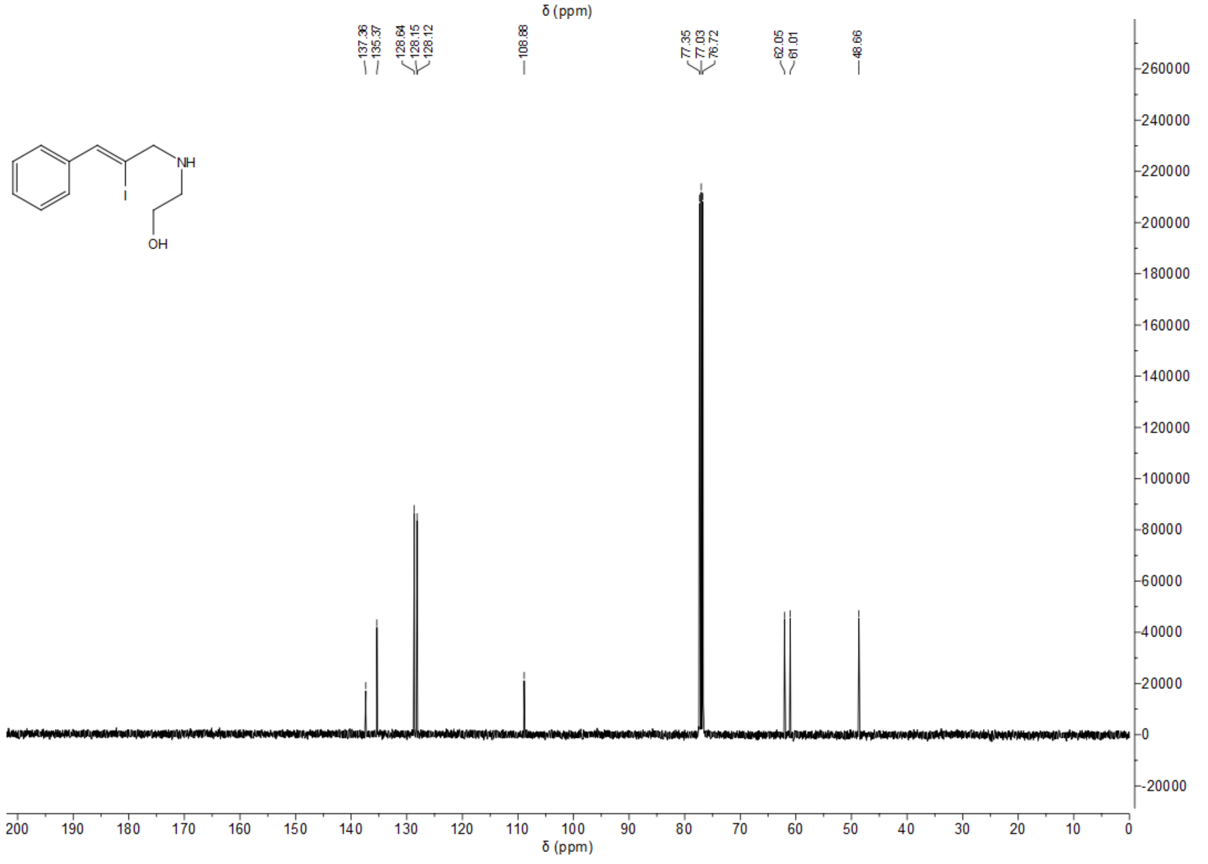

***tert*-butyl (*Z*)-(2-hydroxyethyl)(2-iodo-3-phenylallyl)carbamate (S8) [^{1}H-NMR data: 400 MHz, CDCl$_3$; ^{13}C{^{1}H}-NMR data: 101 MHz, CDCl$_3$]:**

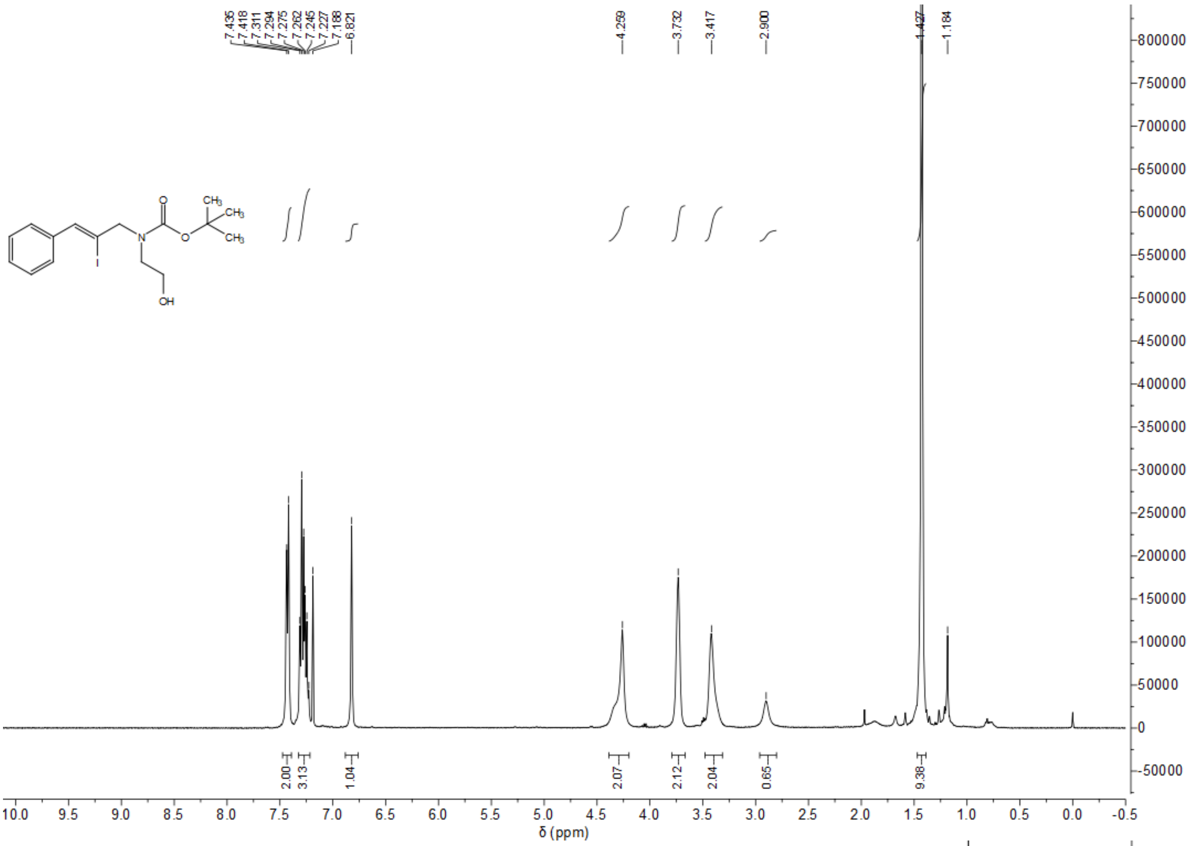

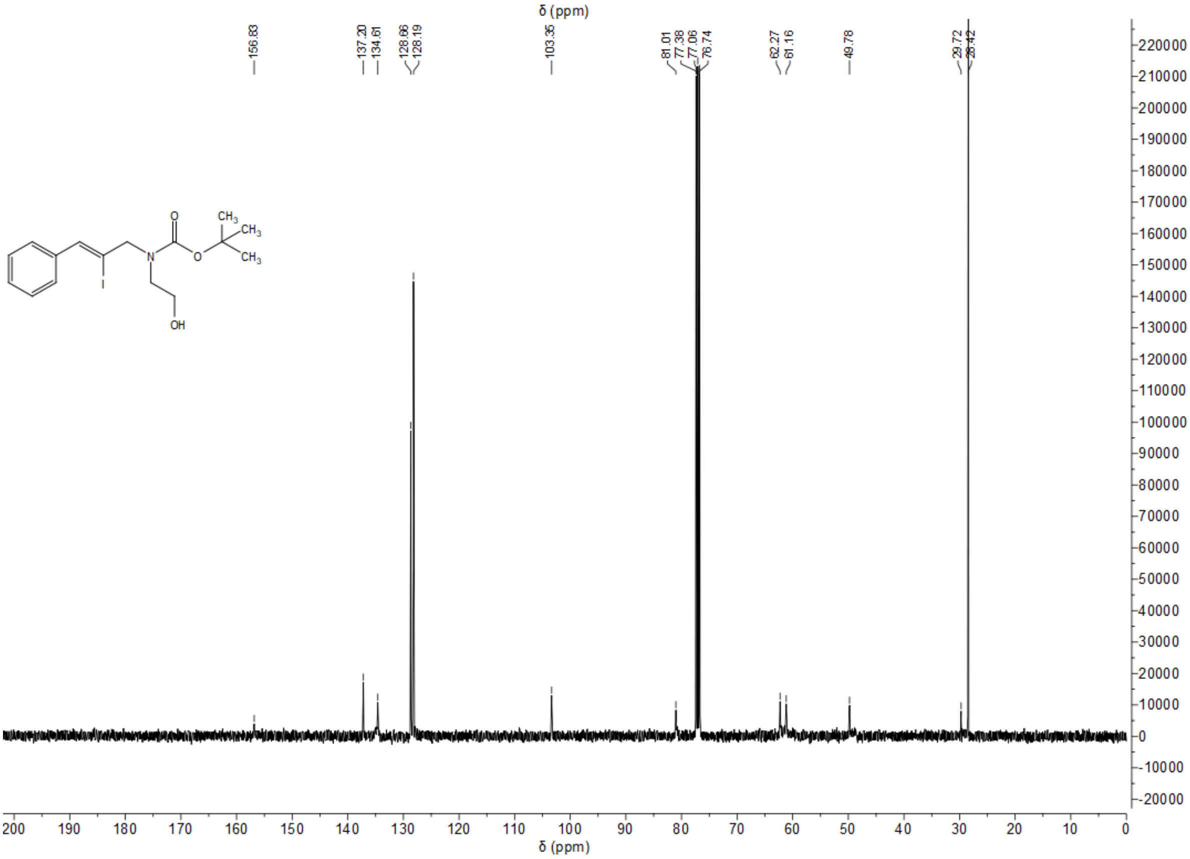

(*Z*)-*N*-(2-hydroxyethyl)-*N*-(2-iodo-3-phenylallyl)-4-methylbenzenesulfonamide (*S*9) [^{1}H-NMR data: 400 MHz, CDCl$_3$; ^{13}C{^{1}H}-NMR data: 101 MHz, CDCl$_3$]:

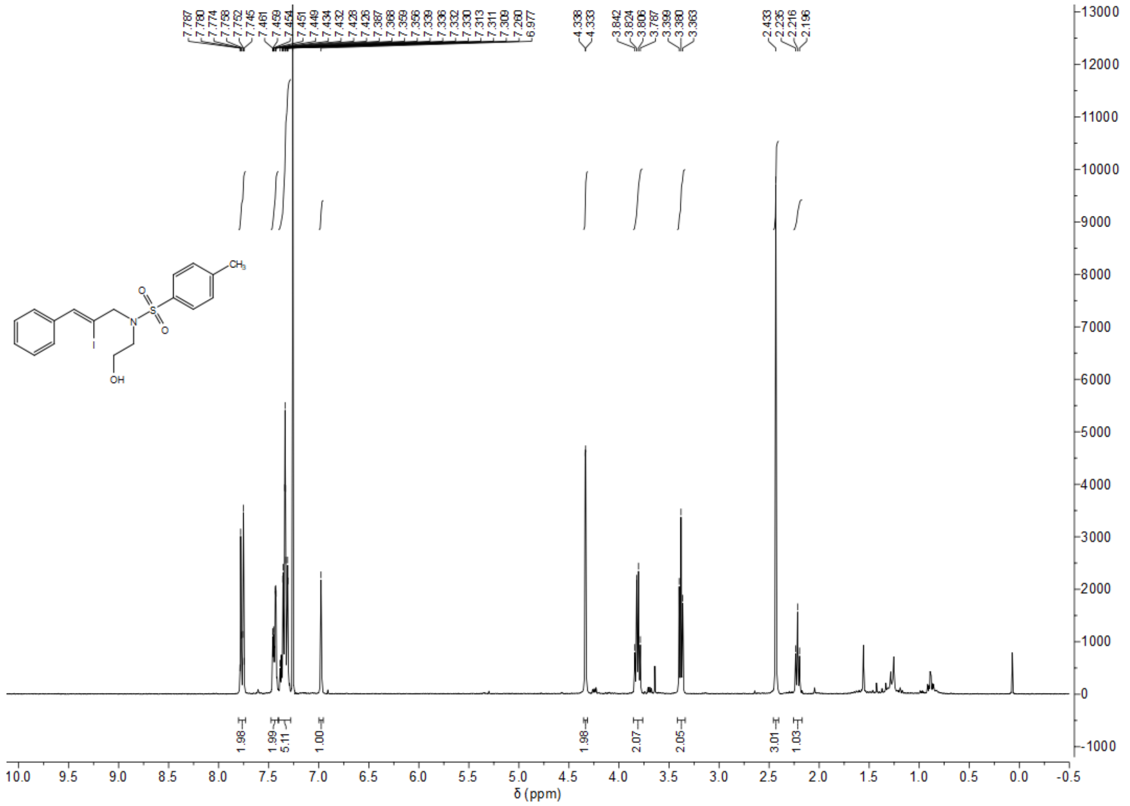

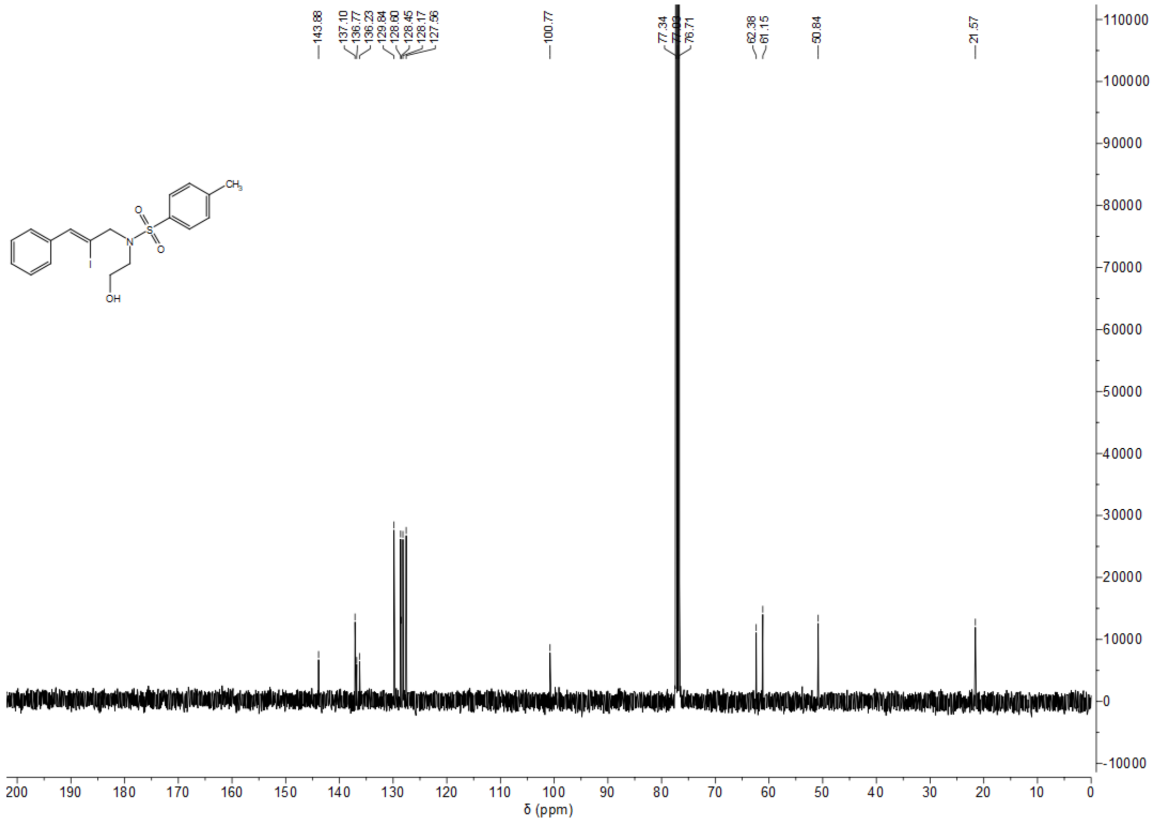

2-iodoprop-2-en-1-ol (S12) [^{1}H-NMR data: 400 MHz, $CDCl_3$]:

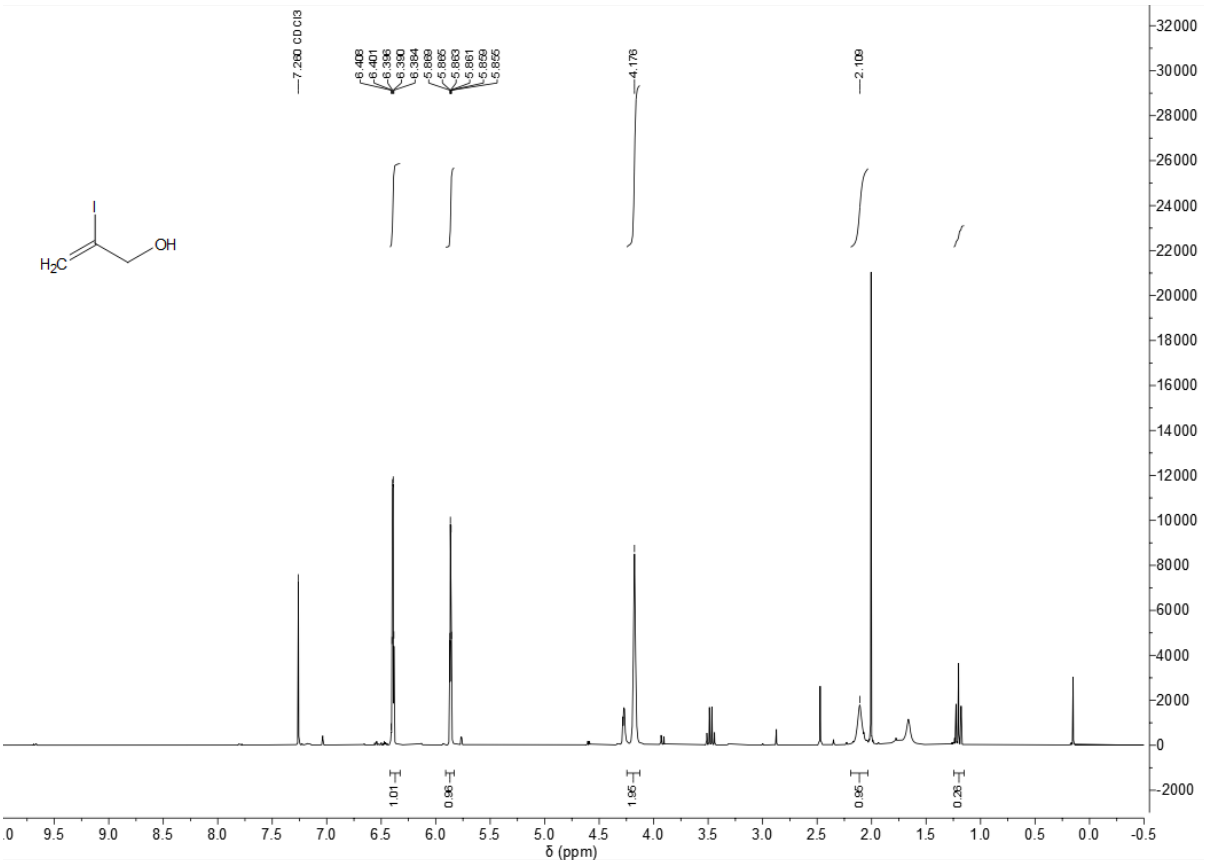

2-iodoallyl 4-methylbenzenesulfonate (S13) [^{1}H-NMR data: 400 MHz, $CDCl_3$; $^{13}C\{^{1}H\}$-NMR data: 101 MHz, $CDCl_3$]:

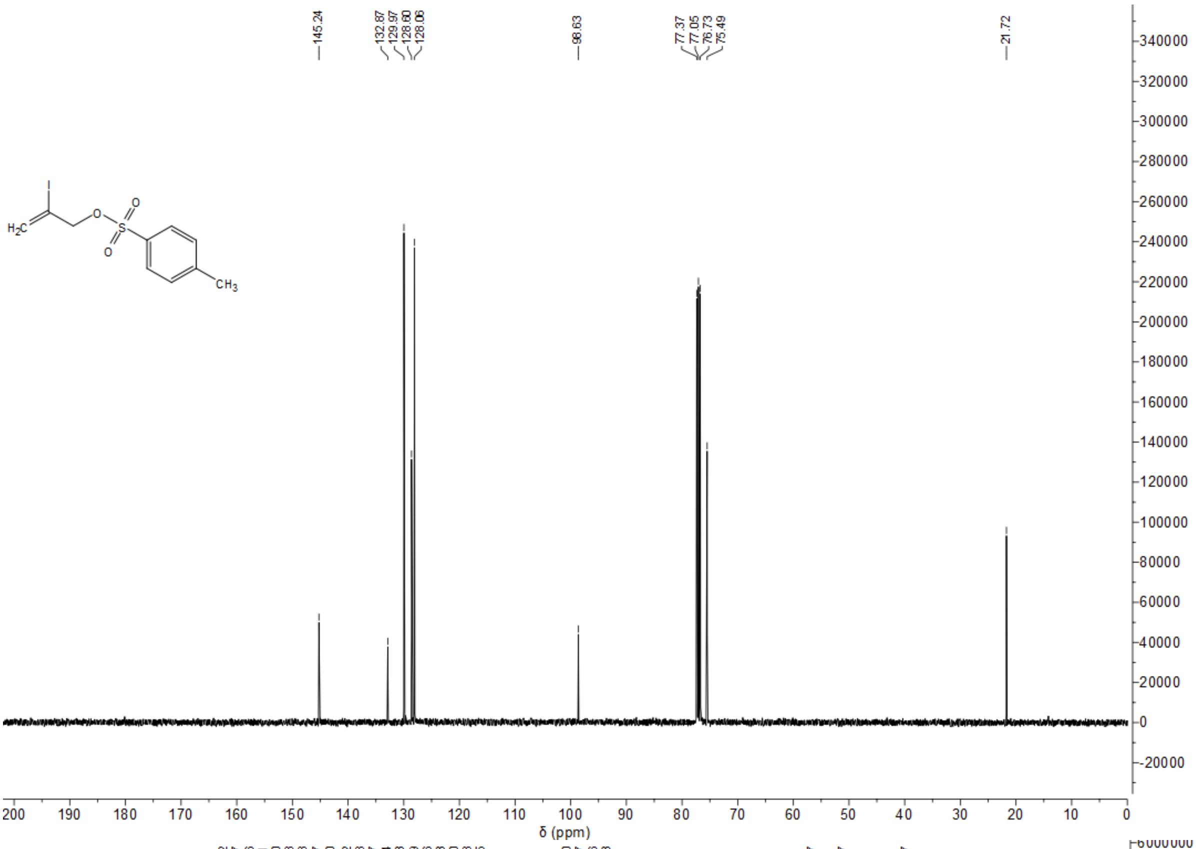

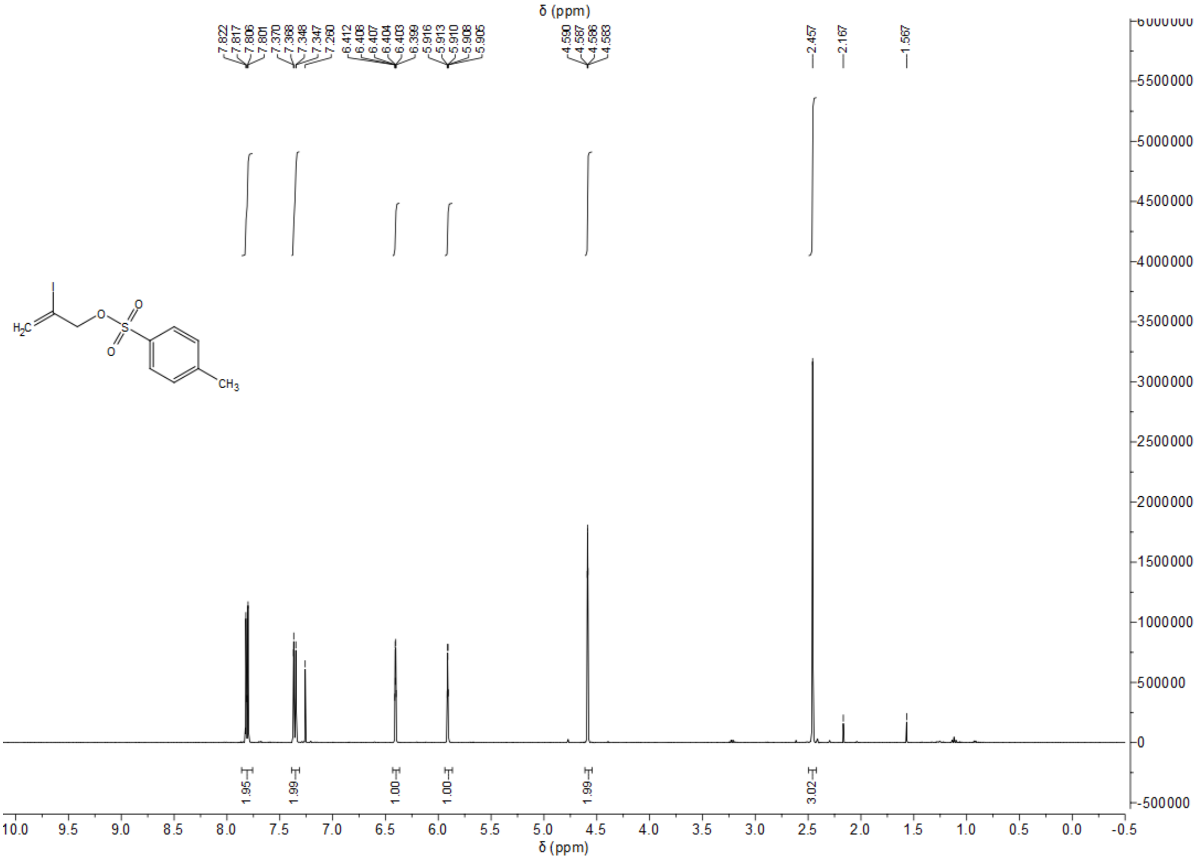

2-(benzyl(2-iodoallyl)amino)ethan-1-ol (S10) [^{1}H-NMR data: 400 MHz, $CDCl_3$; $^{13}C\{^{1}H\}$-NMR data: 101 MHz, $CDCl_3$]:

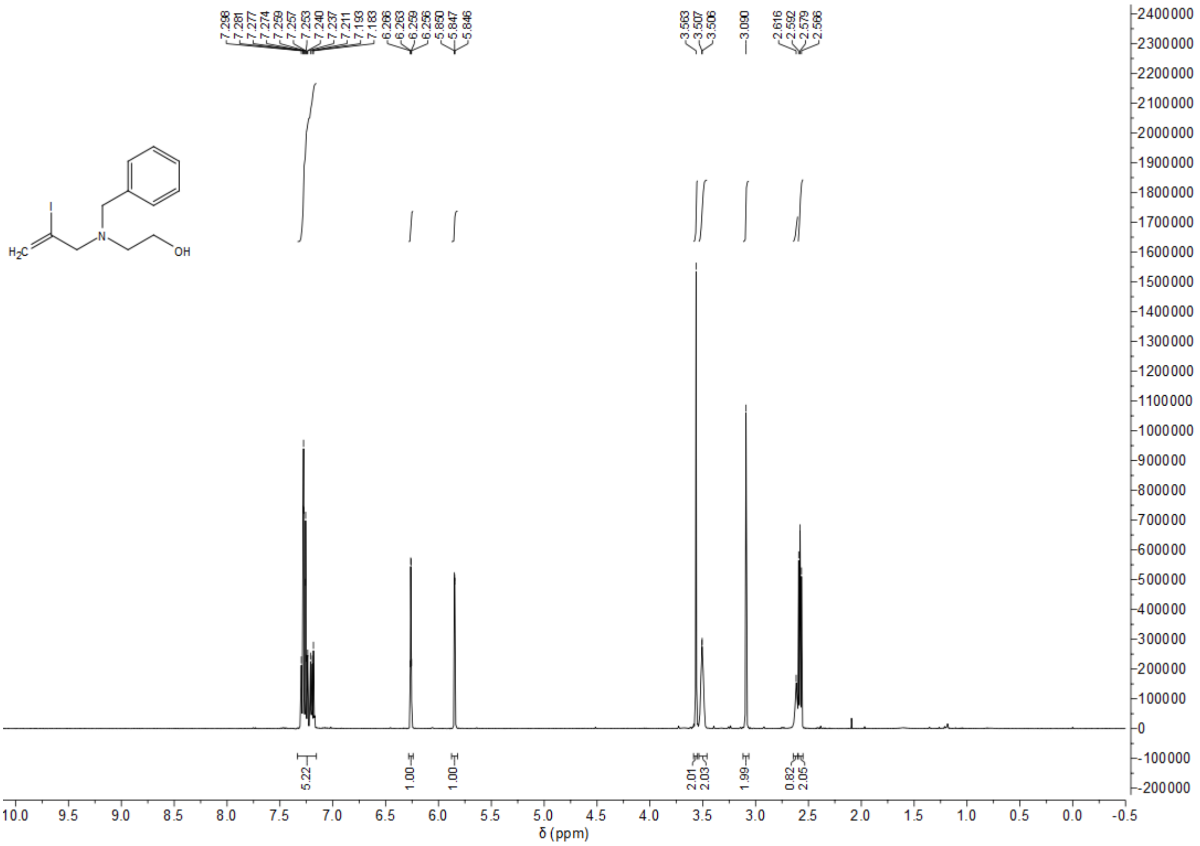

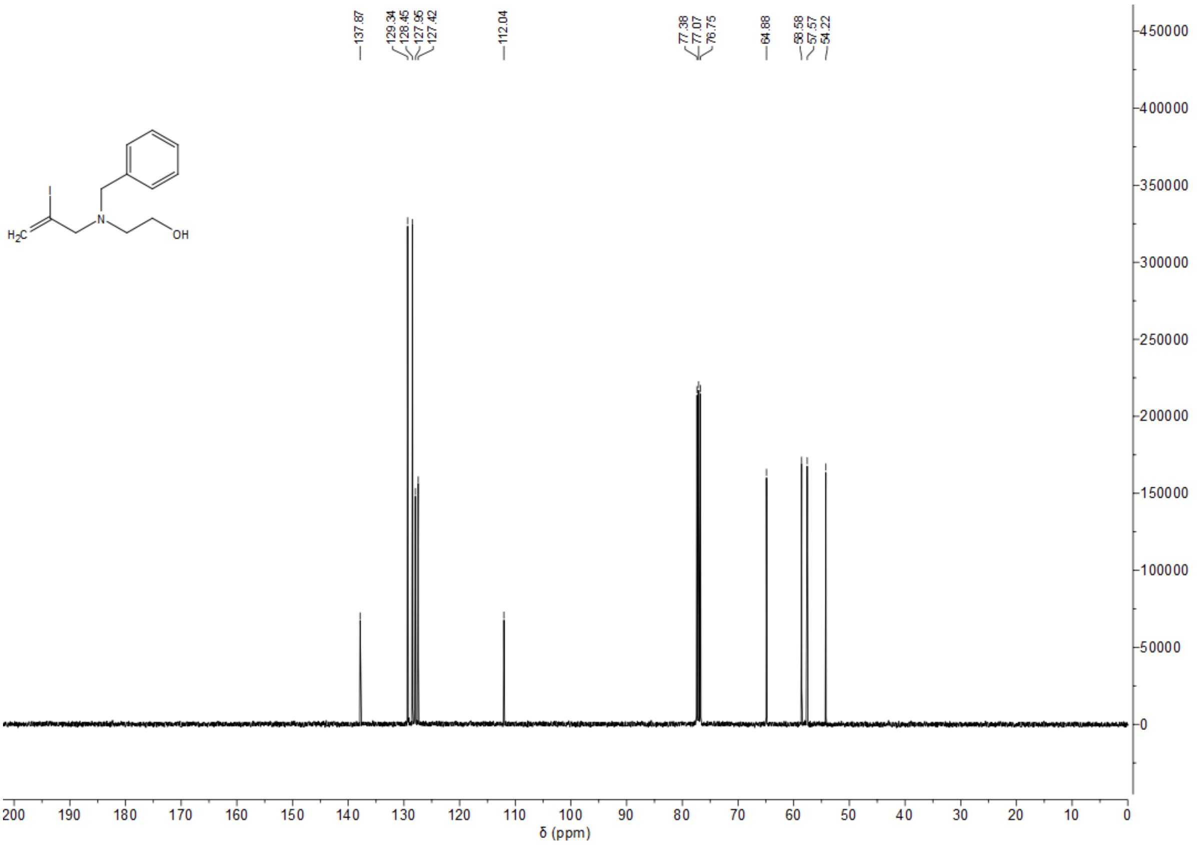

(*Z*)-2-benzylidene-4-methylmorpholine (S14) [^{1}H-NMR data: 400 MHz, $CDCl_3$; $^{13}C\{^1H\}$-NMR data: 101 MHz, $CDCl_3$]:

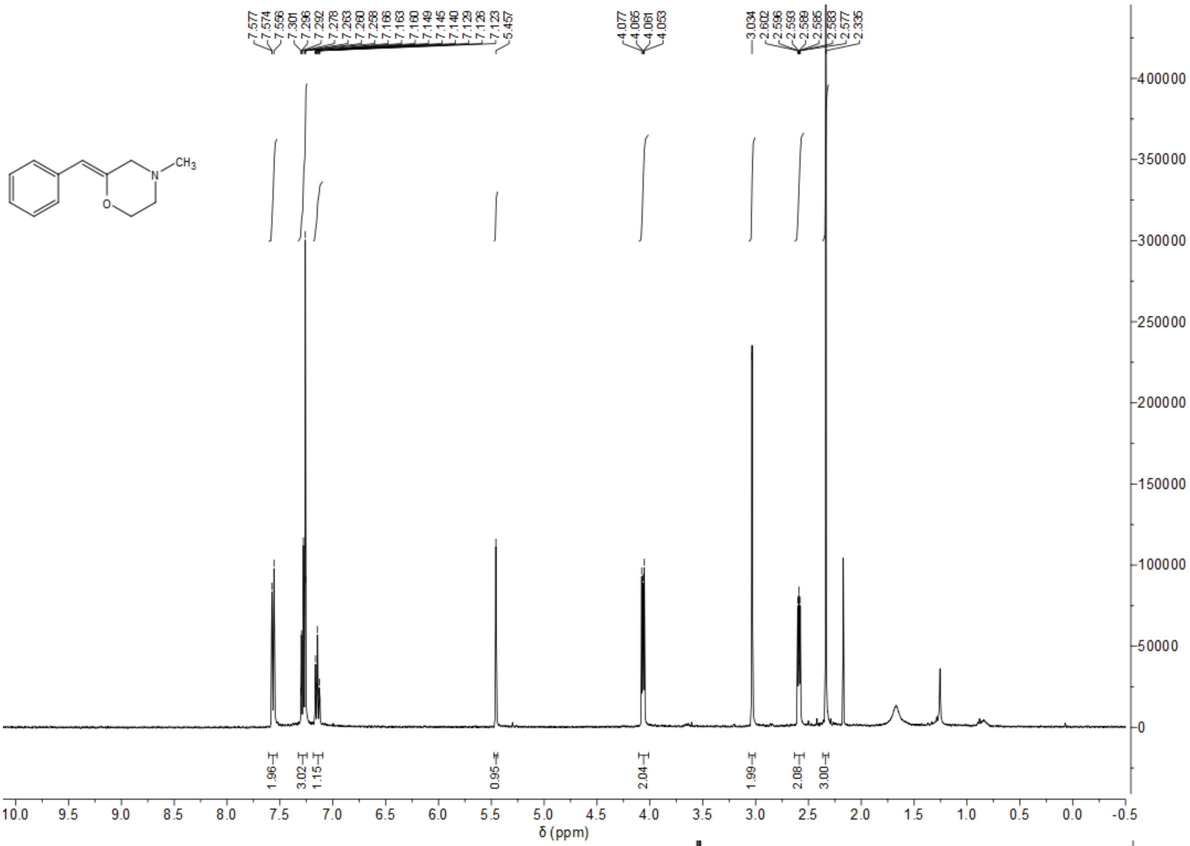

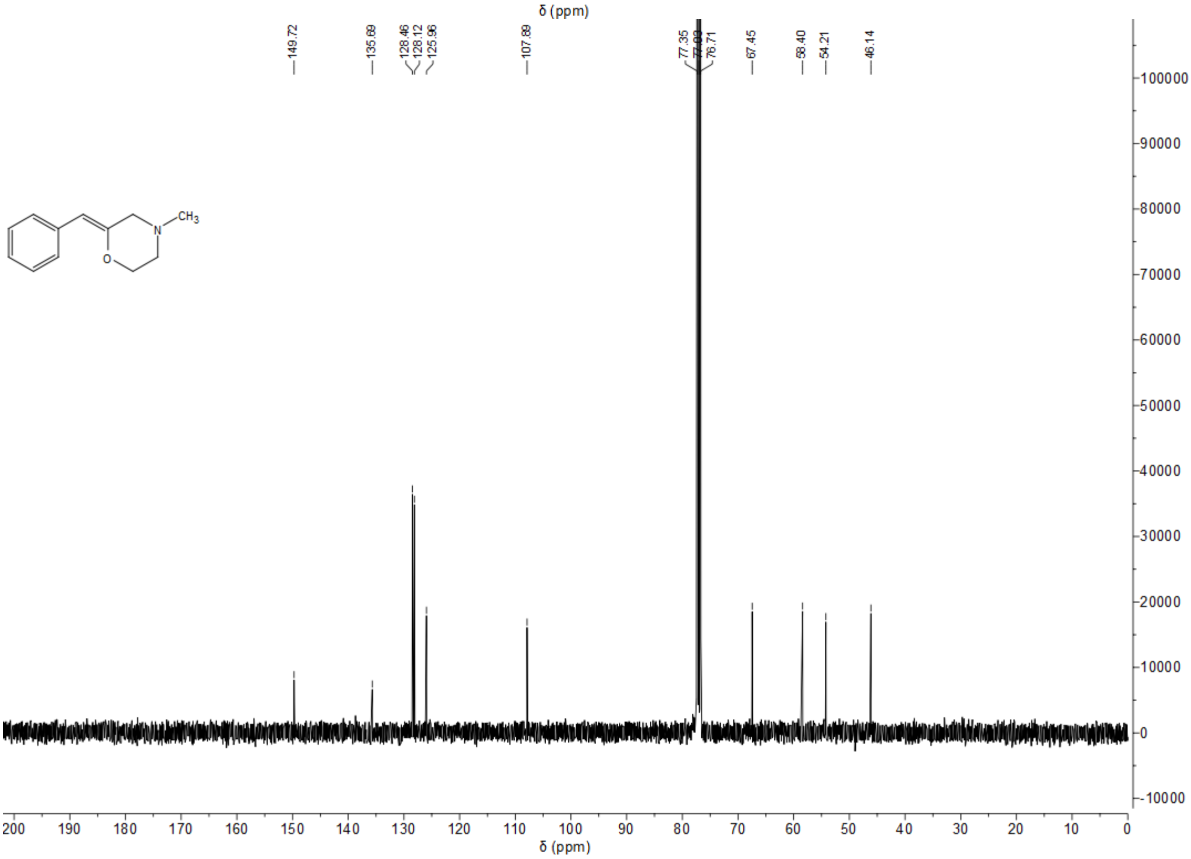

(*Z*)-4-benzyl-2-benzylidenemorpholine (S15) [^{1}H-NMR data: 400 MHz, $CDCl_3$; $^{13}C\{^1H\}$-NMR data: 101 MHz, $CDCl_3$]:

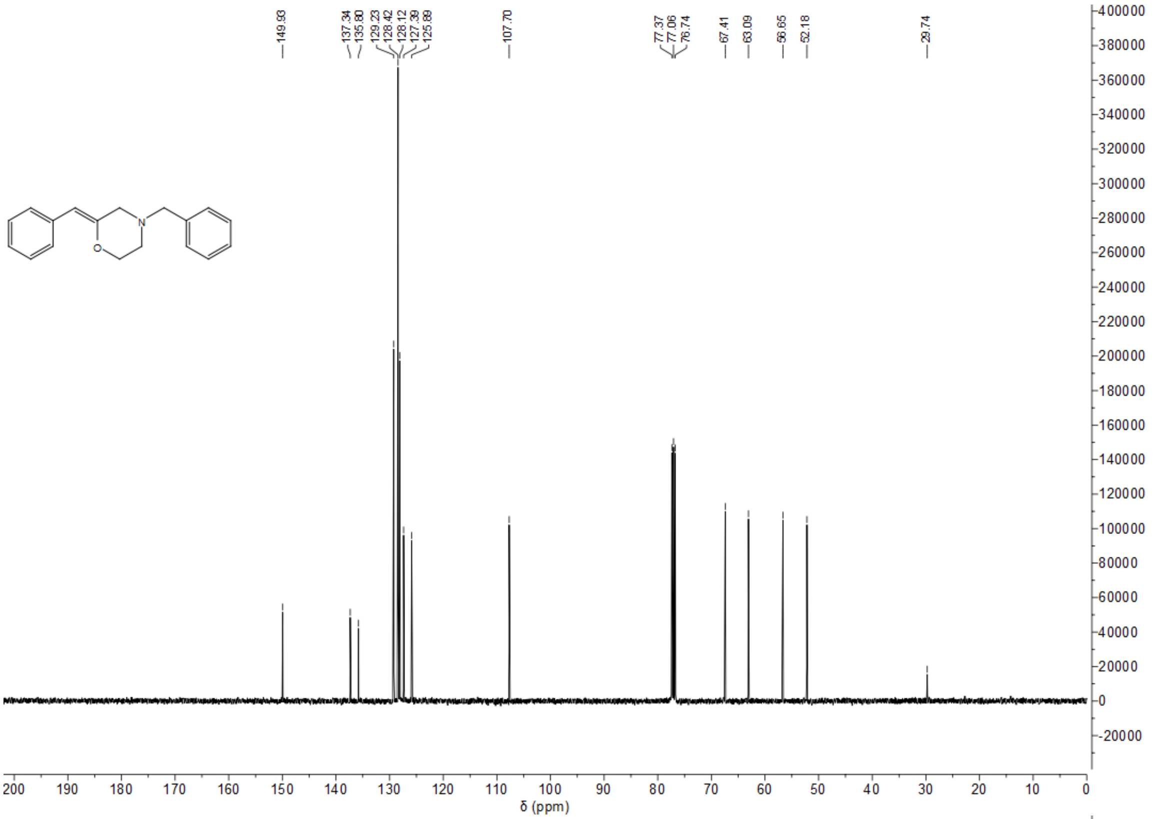

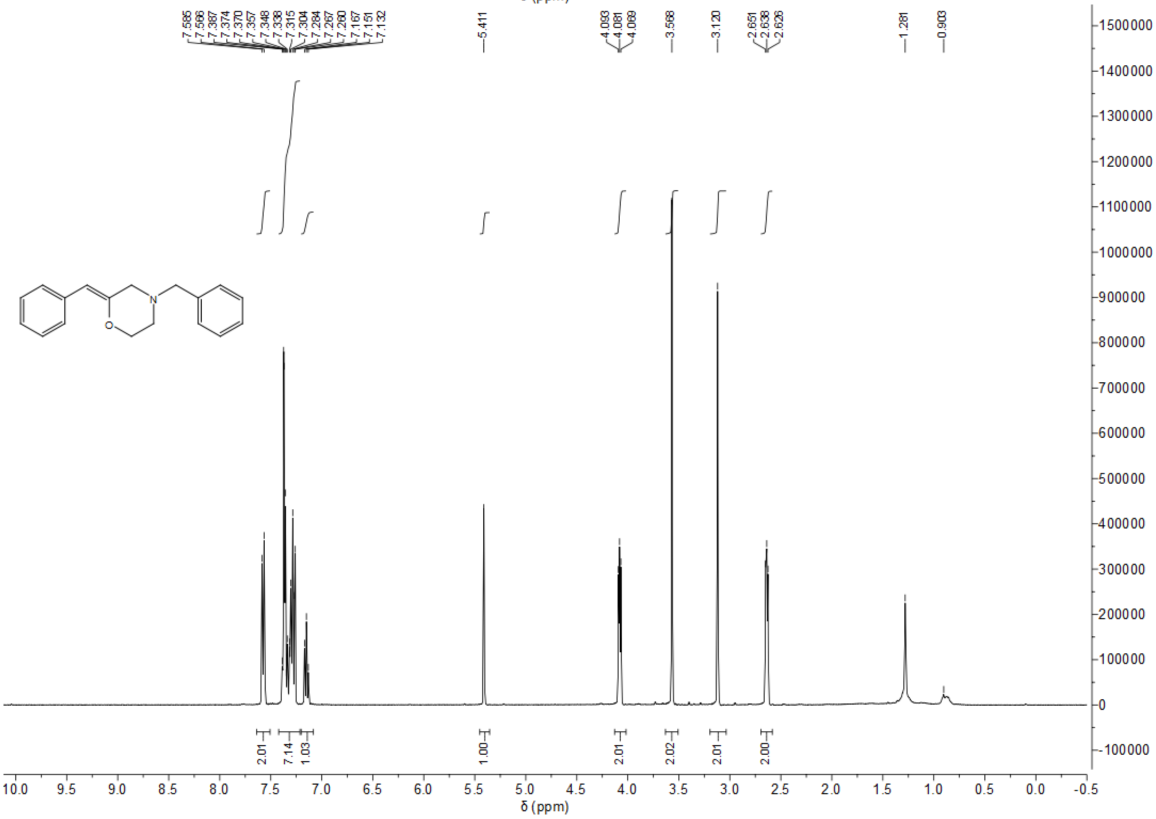

(*Z*)-2-benzylidenemorpholine (S16) [^{1}H-NMR data: 400 MHz, $CDCl_3$; $^{13}C\{^1H\}$-NMR data: 101 MHz, $CDCl_3$]:

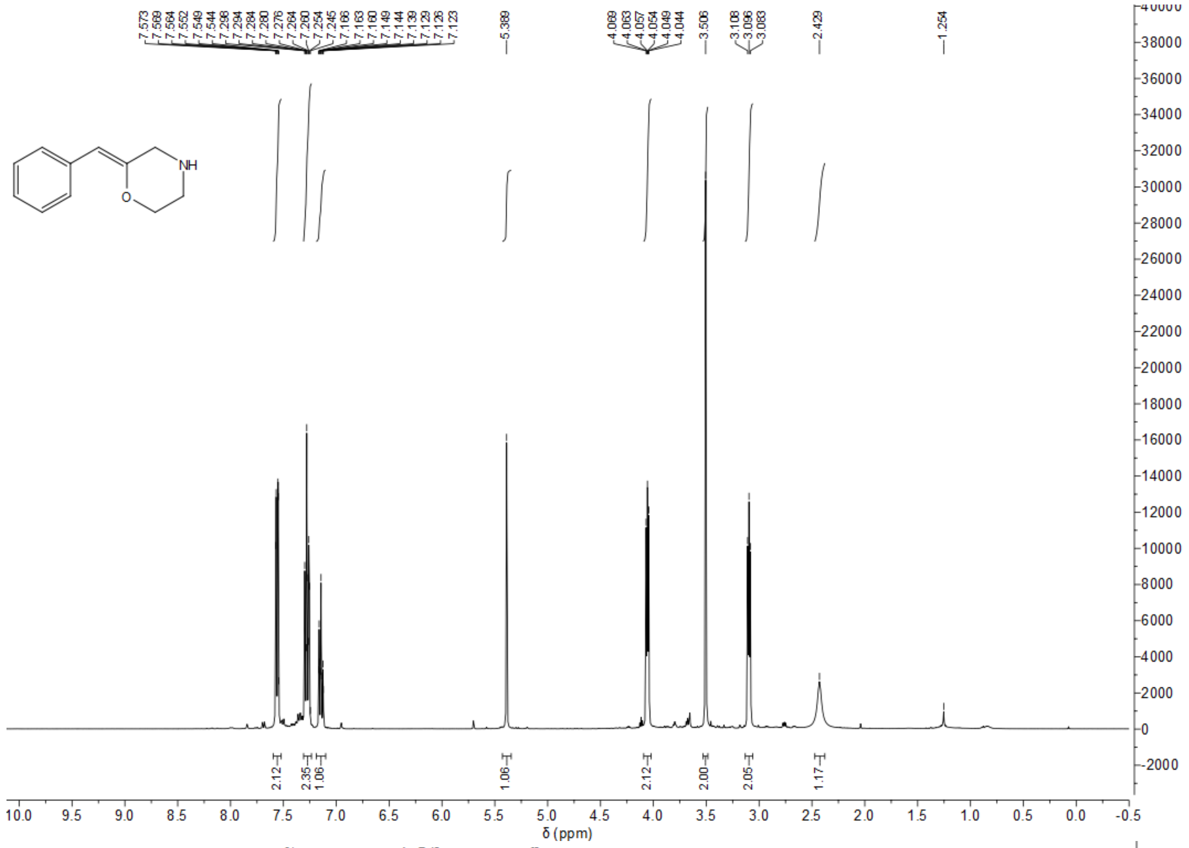

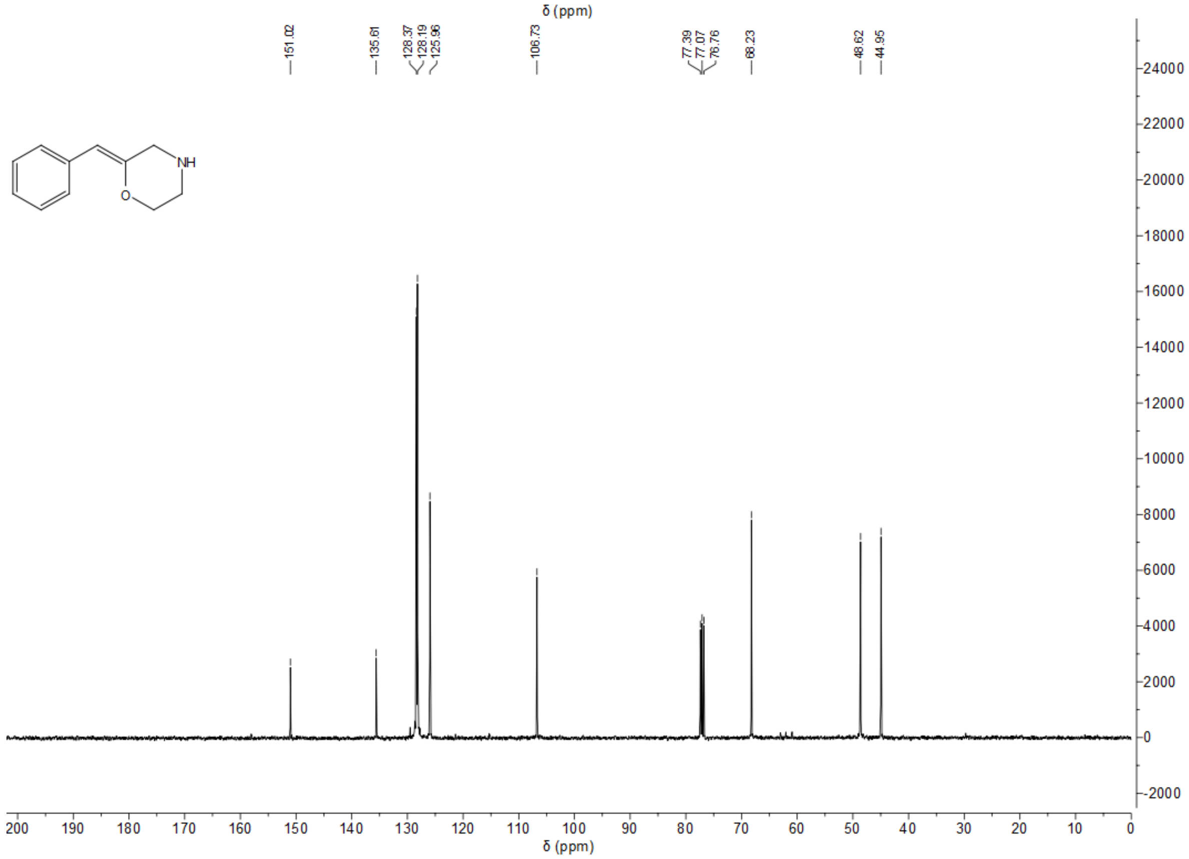

***tert*-butyl(*Z*)-2-benzylidenemorpholine-4-carboxylate (S17) [^{1}H-NMR data: 400 MHz, $CDCl_3$; $^{13}C\{^{1}H\}$-NMR data: 101 MHz, $CDCl_3$]:**

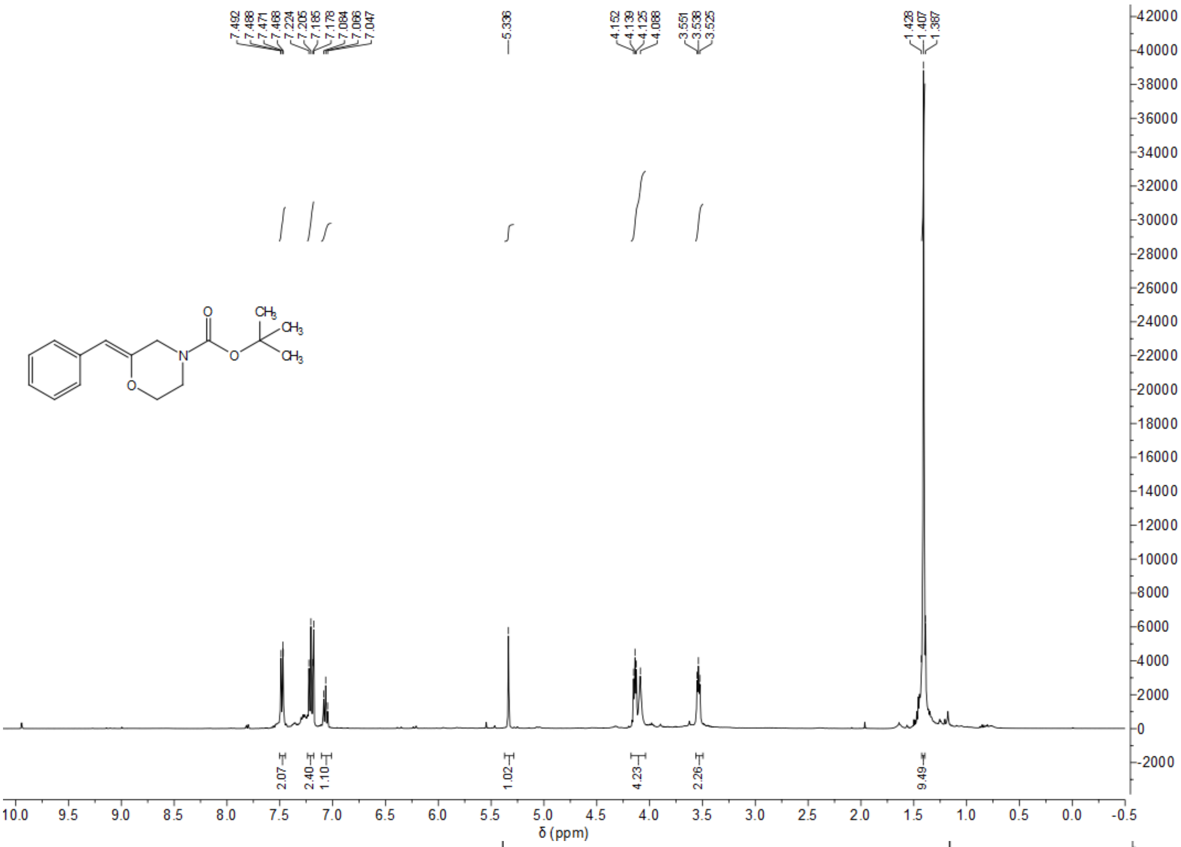

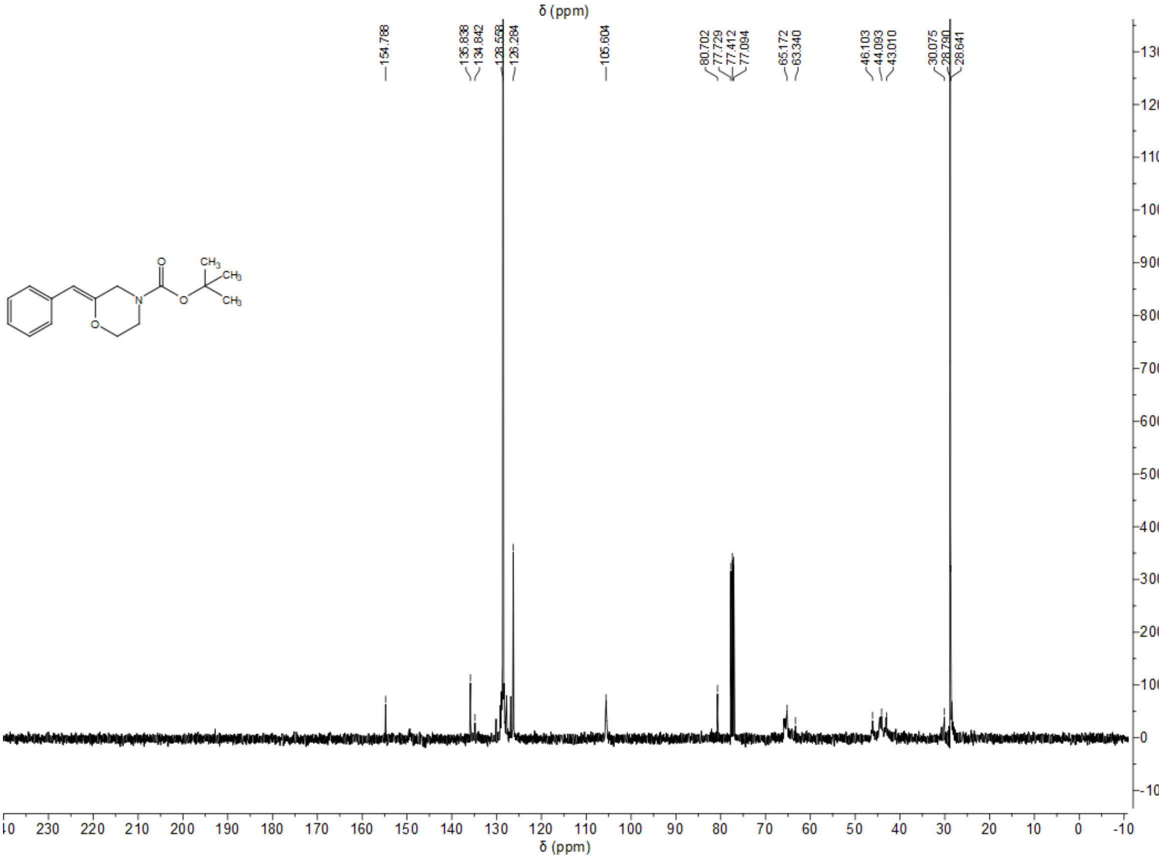

(*Z*)-2-benzylidene-4-tosylmorpholine (S18) [^{1}H-NMR data: 400 MHz, $CDCl_3$; $^{13}C\{^{1}H\}$-NMR data: 101 MHz, $CDCl_3$]:

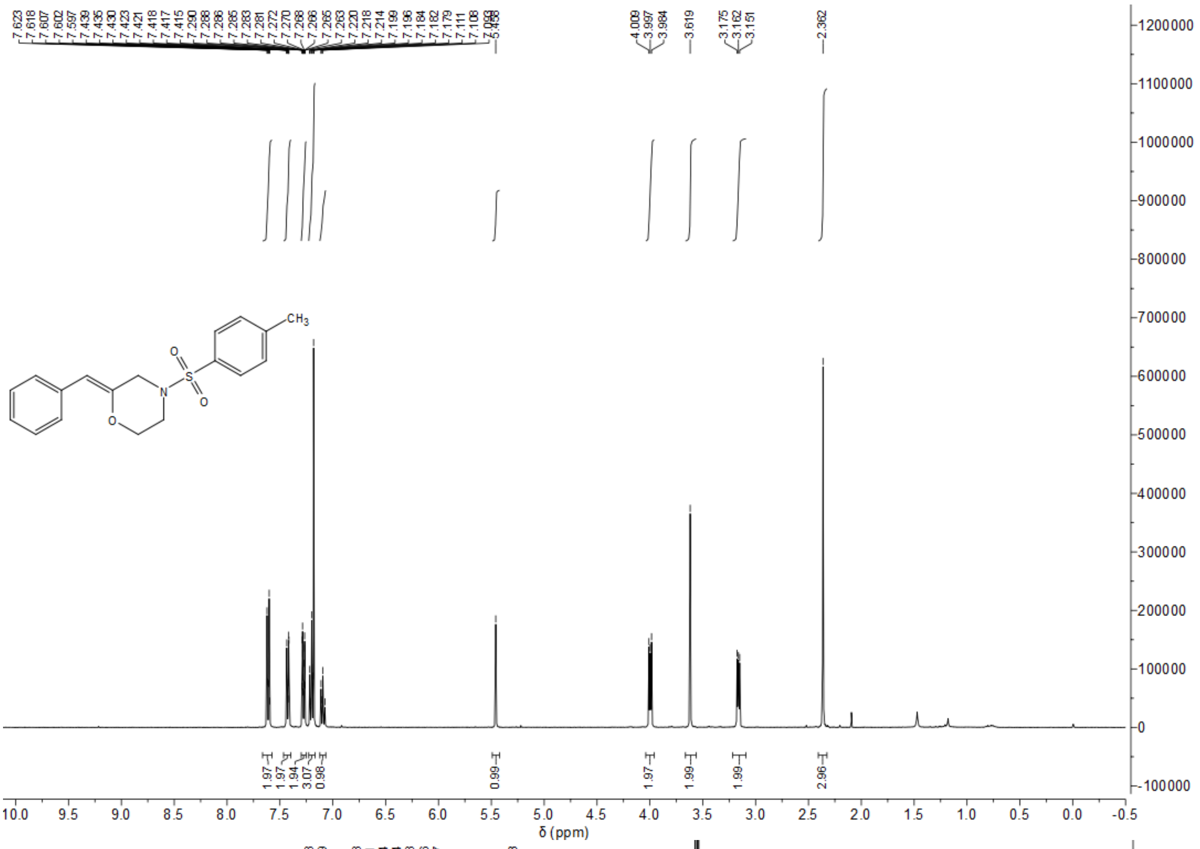

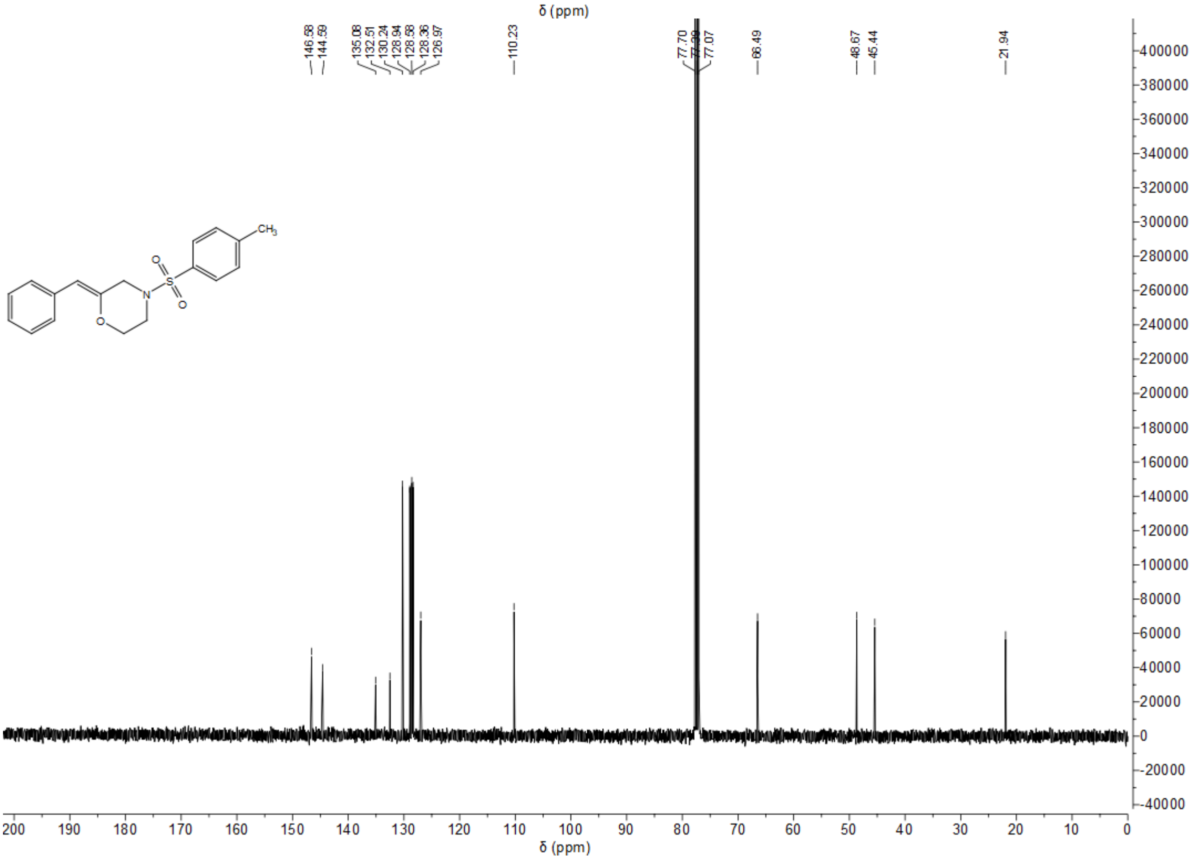

2-(allyl(benzyl)amino)ethan-1-ol (S19) [^{1}H-NMR data: 400 MHz, $CDCl_3$; $^{13}C\{^{1}H\}$-NMR data: 101 MHz, $CDCl_3$]:

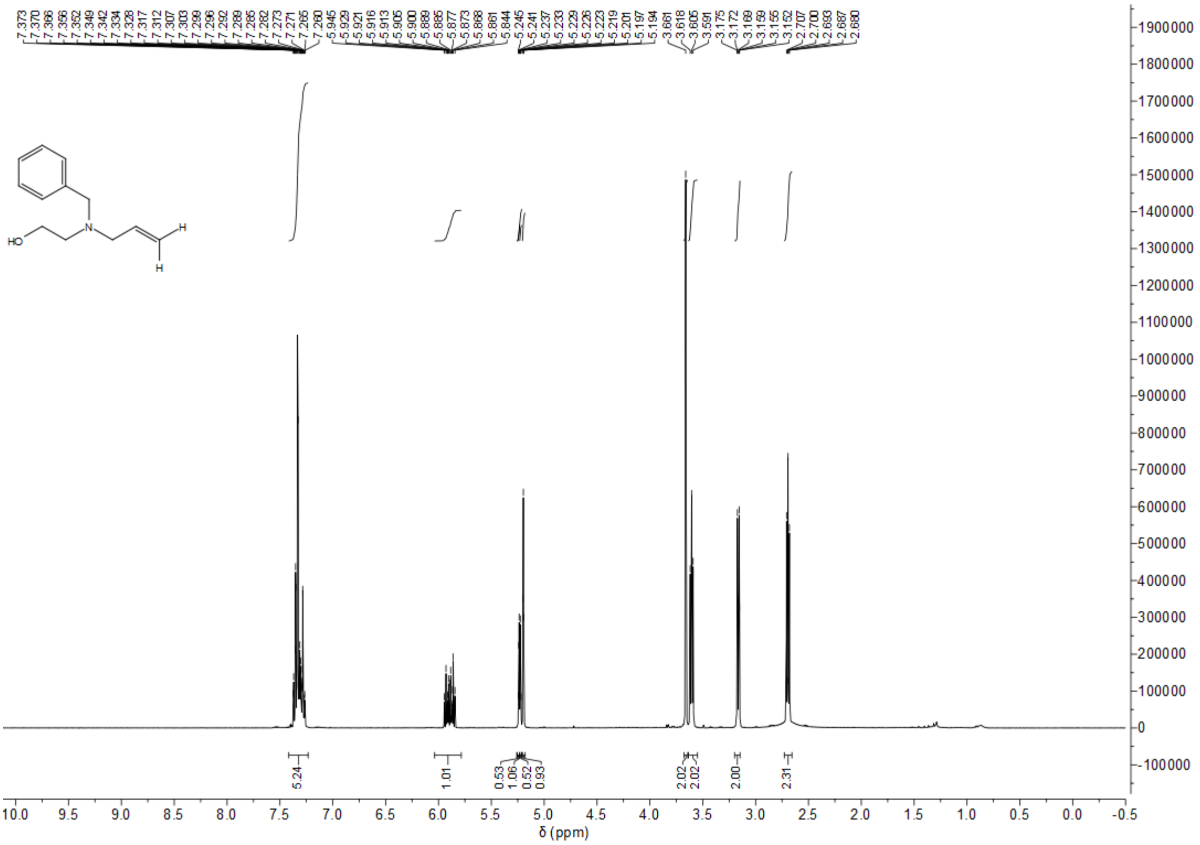

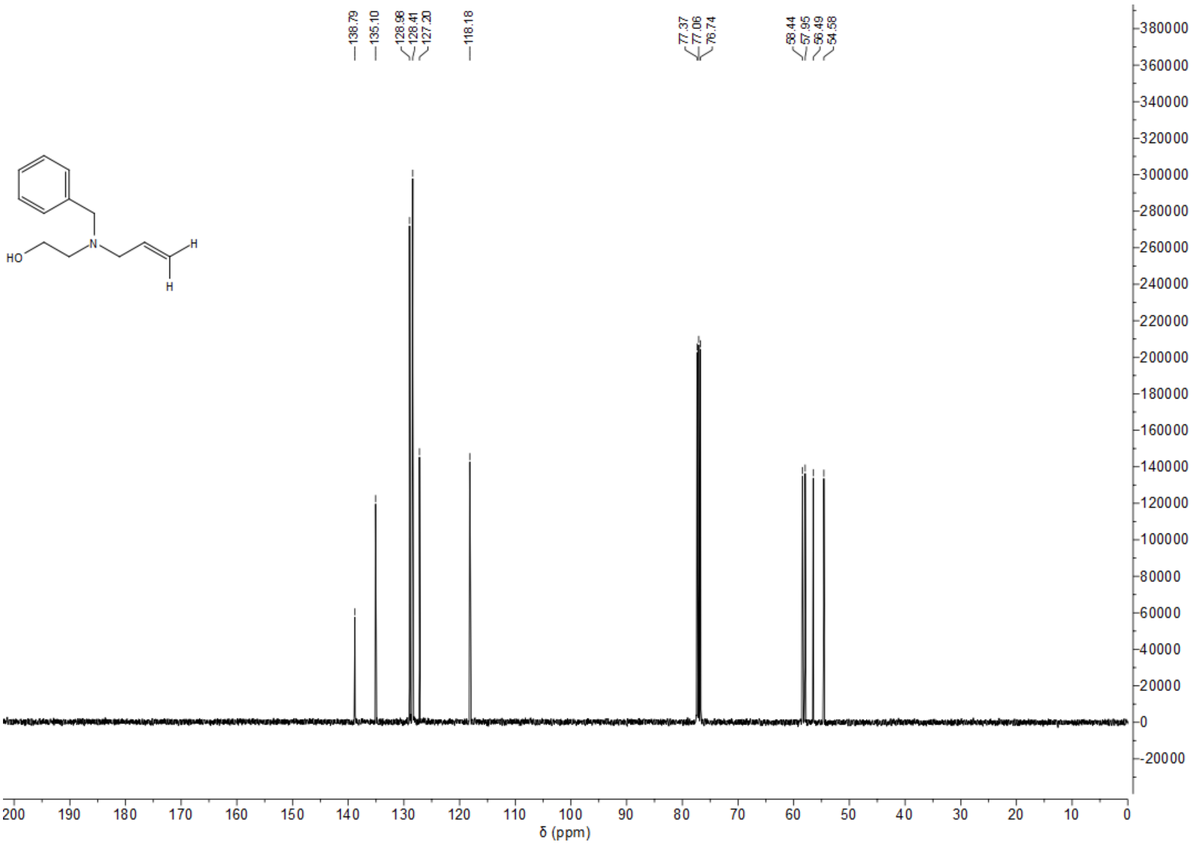

1.2 Aminoalcohols

4-(benzylamino)butan-1-ol (S34) [^{1}H-NMR data: 400 MHz, $CDCl_3$; $^{13}C\{^1H\}$-NMR data: 101 MHz, $CDCl_3$]:

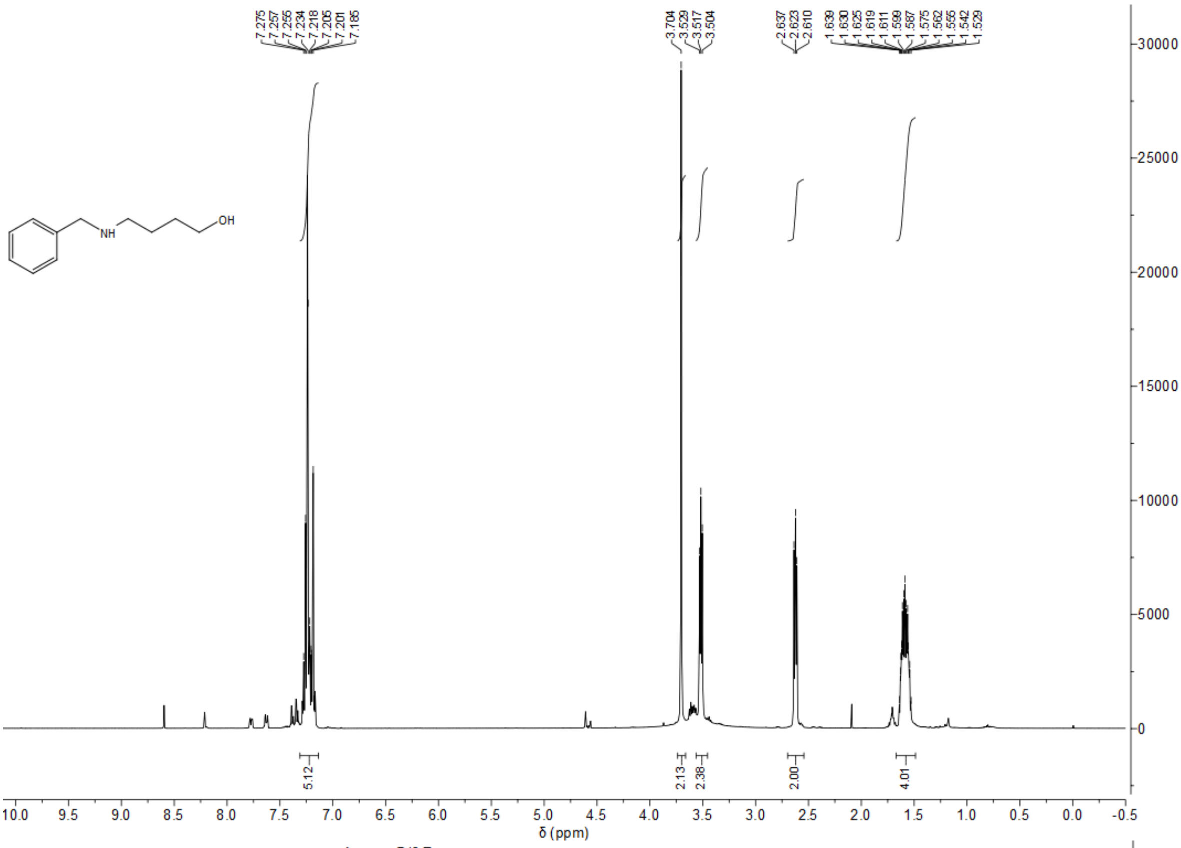

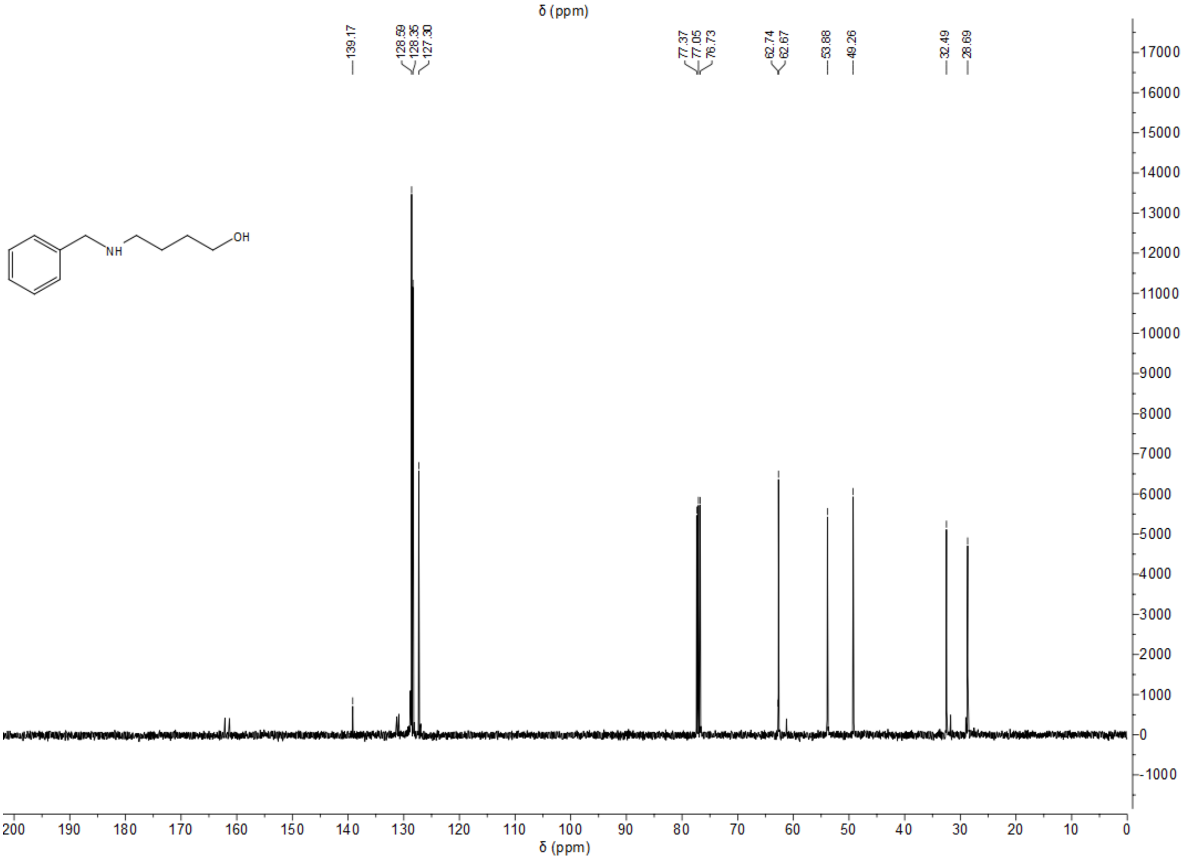

(*S*)-1-(benzylamino)propan-2-ol (33) [^{1}H-NMR data: 400 MHz, $CDCl_3$; $^{13}C\{^{1}H\}$-NMR data: 101 MHz, $CDCl_3$]:

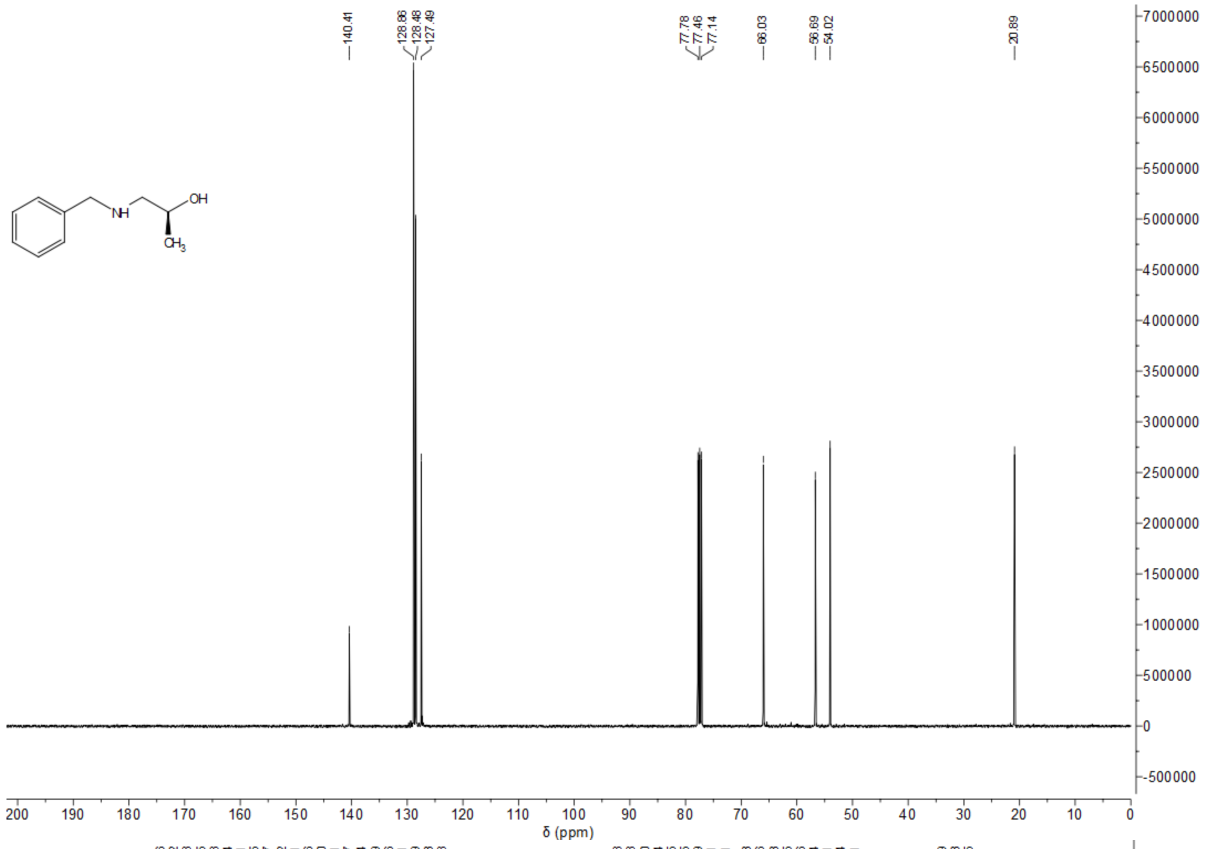

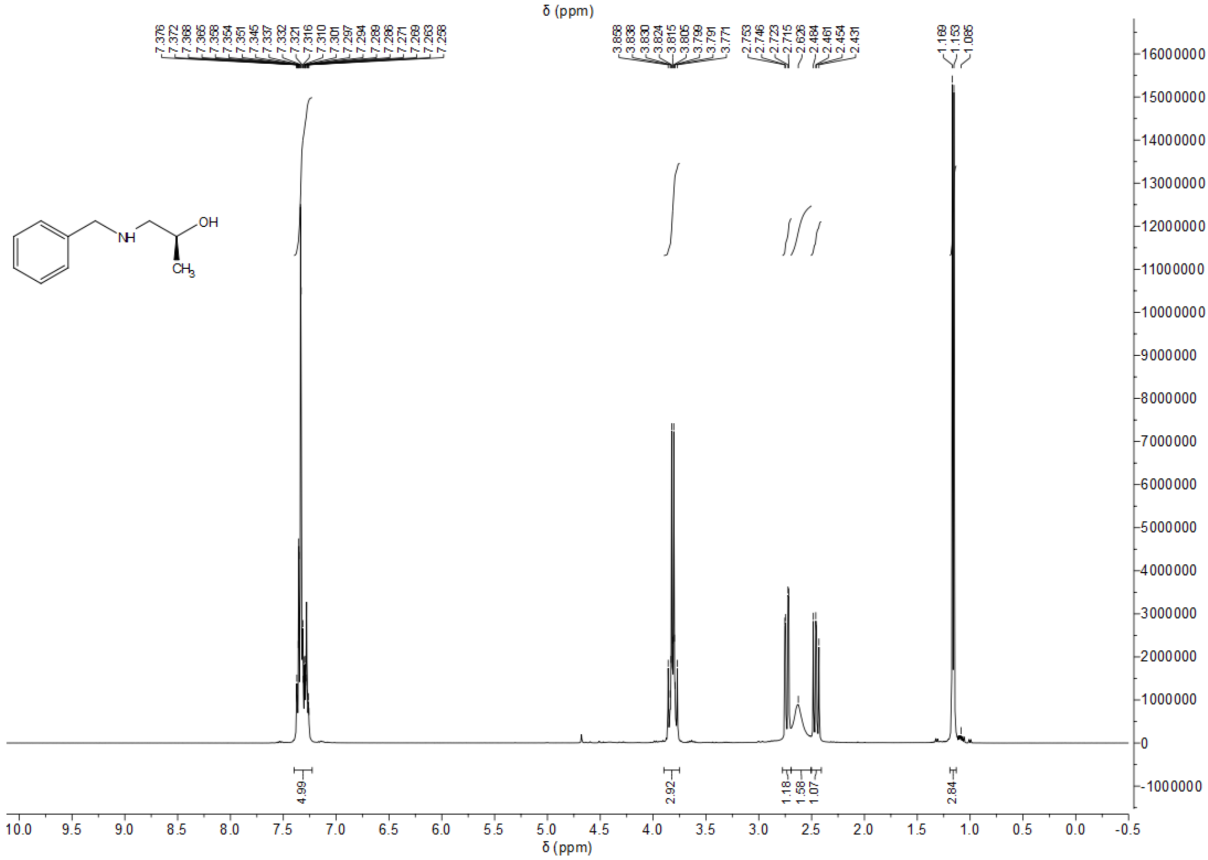

(*S*)-2-(benzylamino)propan-1-ol (35) [^{1}H-NMR data: 400 MHz, $CDCl_3$; $^{13}C\{^{1}H\}$-NMR data: 101 MHz, $CDCl_3$]:

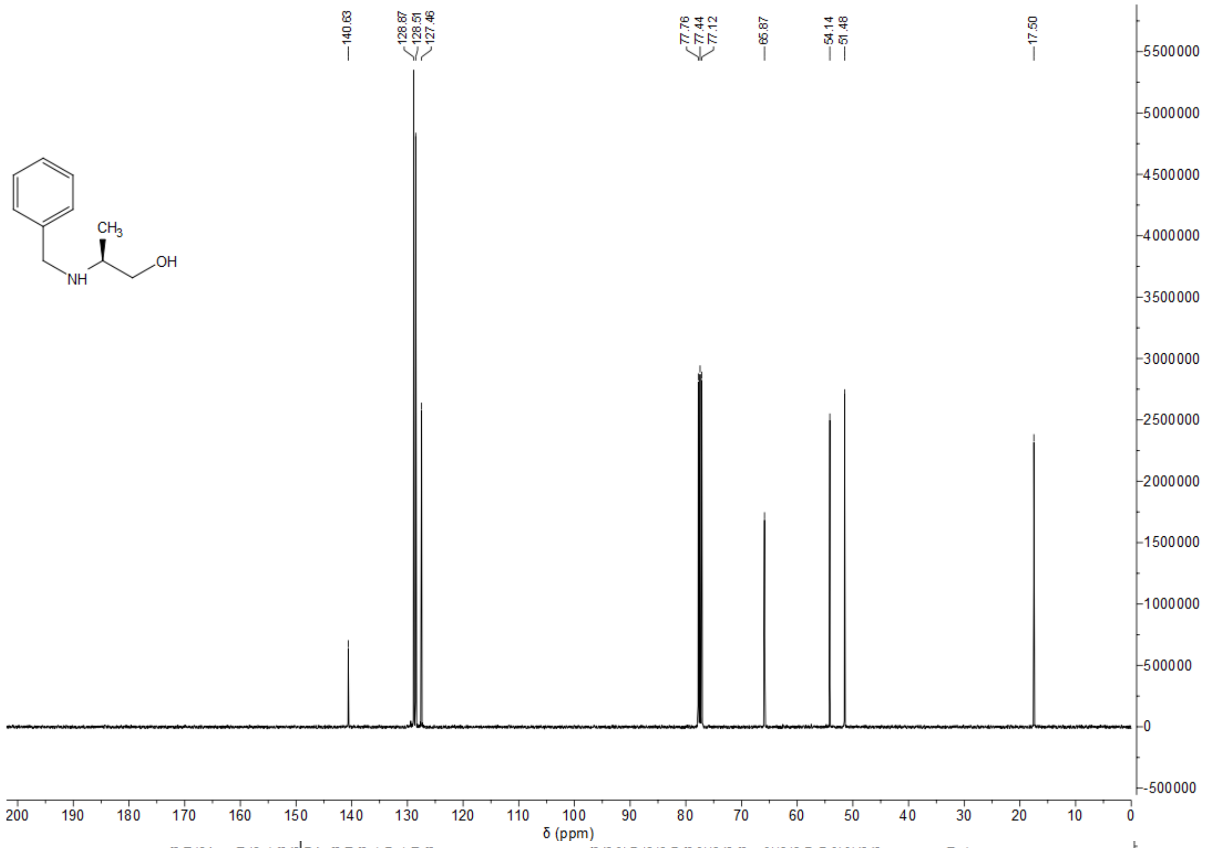

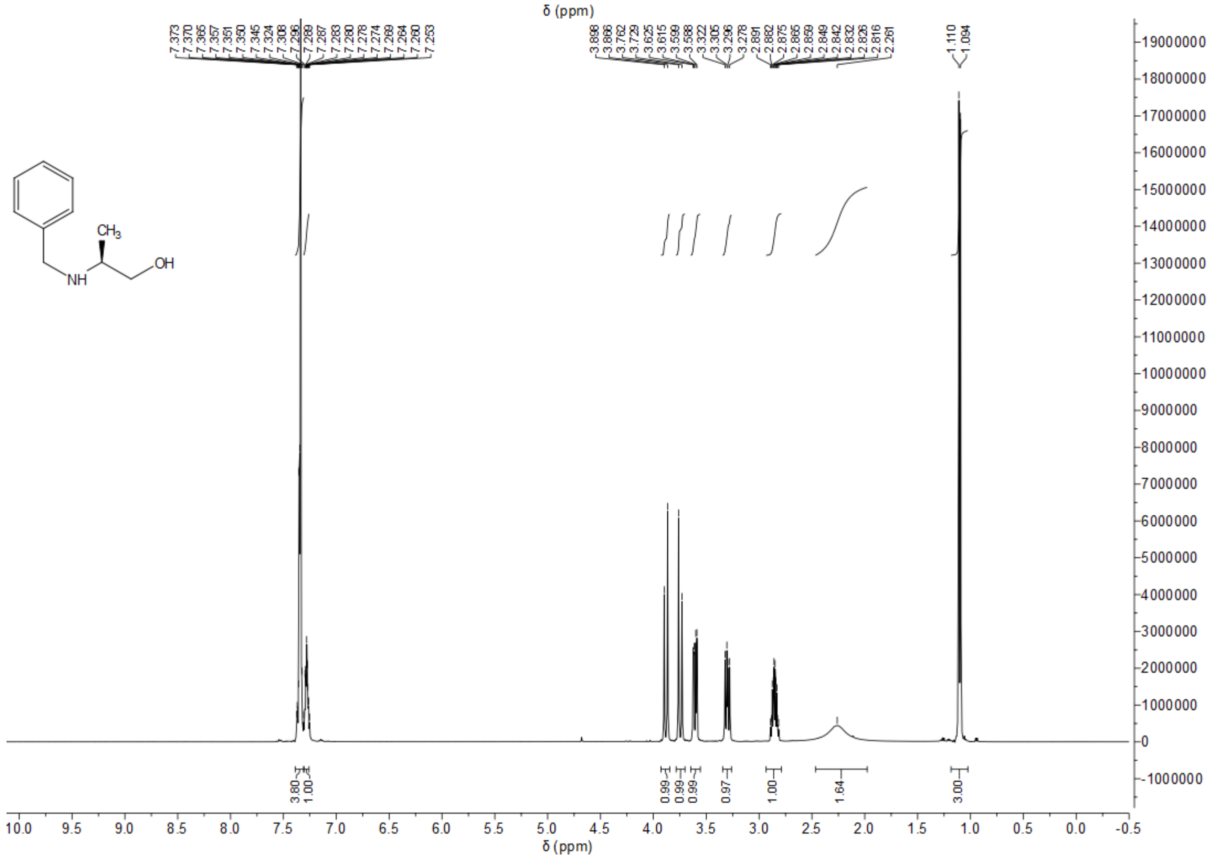

1.3 Chloromethyl-substituted heterocycles

4-benzyl-2-(chloromethyl)morpholine (1) [^{1}H-NMR data: 400 MHz, CDCl$_3$; ^{13}C{^{1}H}-NMR data: 101 MHz, CDCl$_3$]:

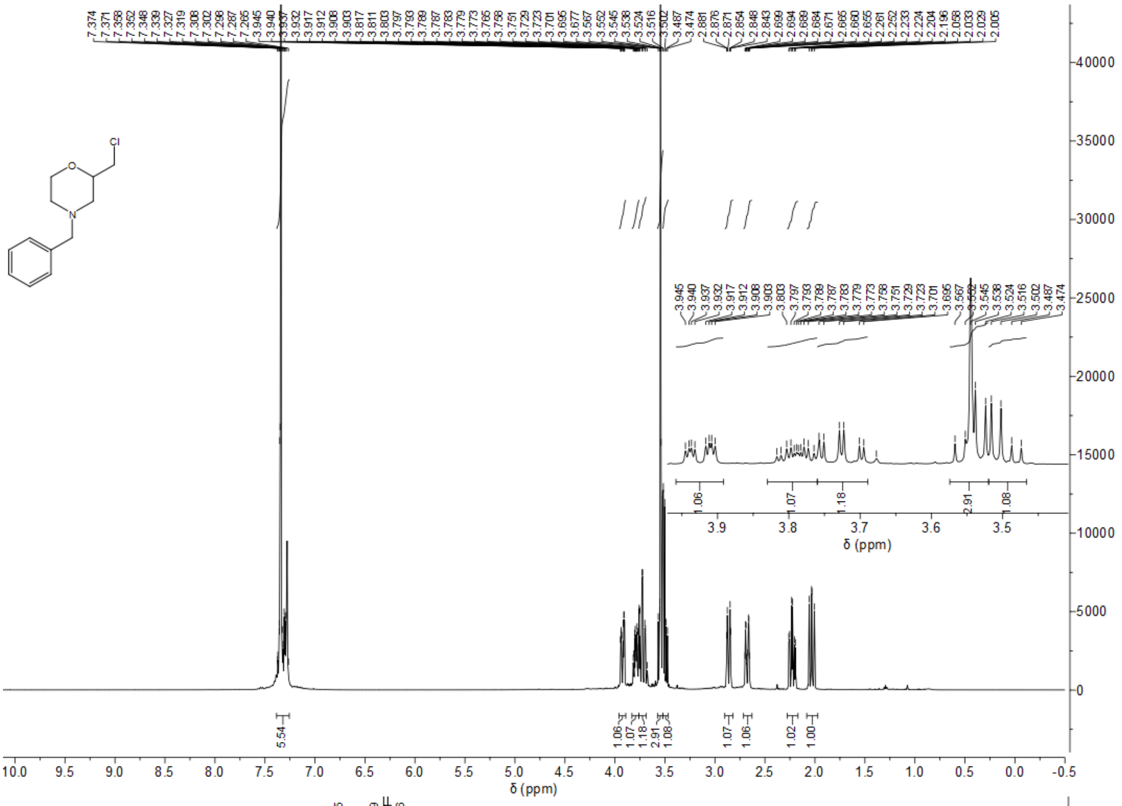

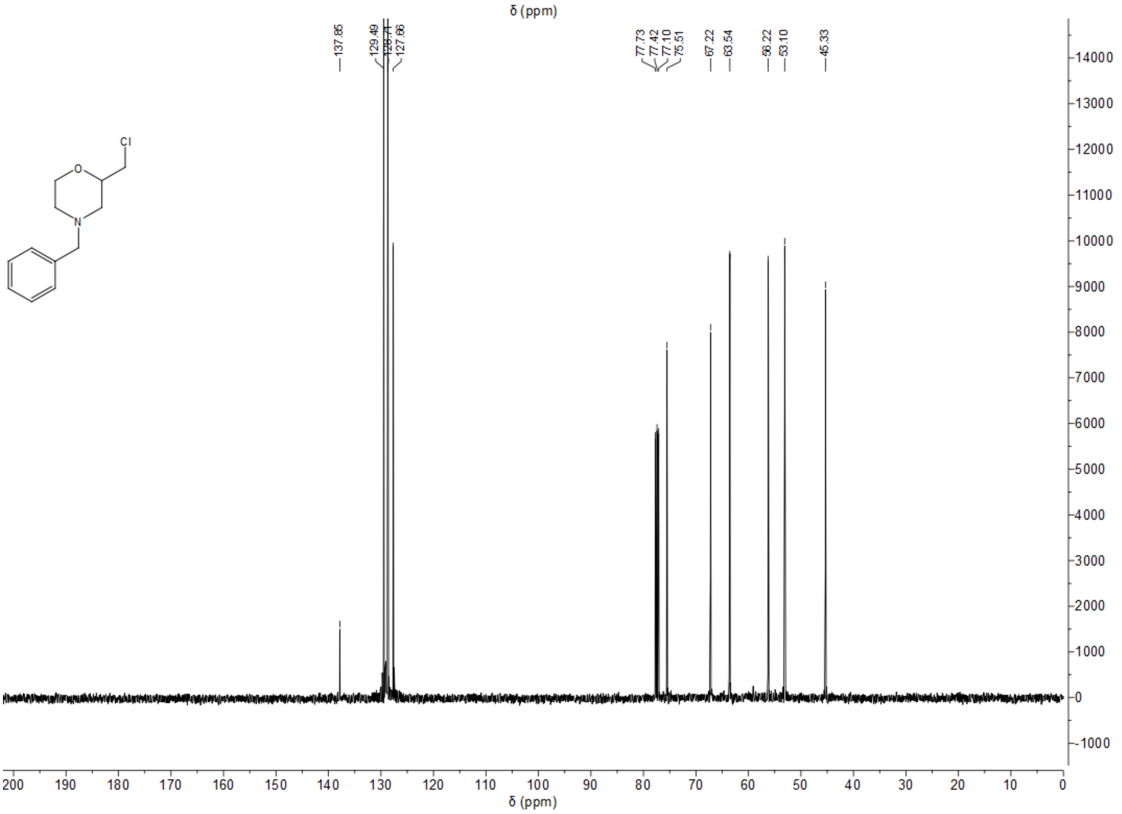

4-benzyl-2-(chloromethyl)-1,4-oxazepane (S32) [^{1}H-NMR data: 400 MHz, $CDCl_3$; ^{13}C{^{1}H}-NMR data: 101 MHz, $CDCl_3$]:

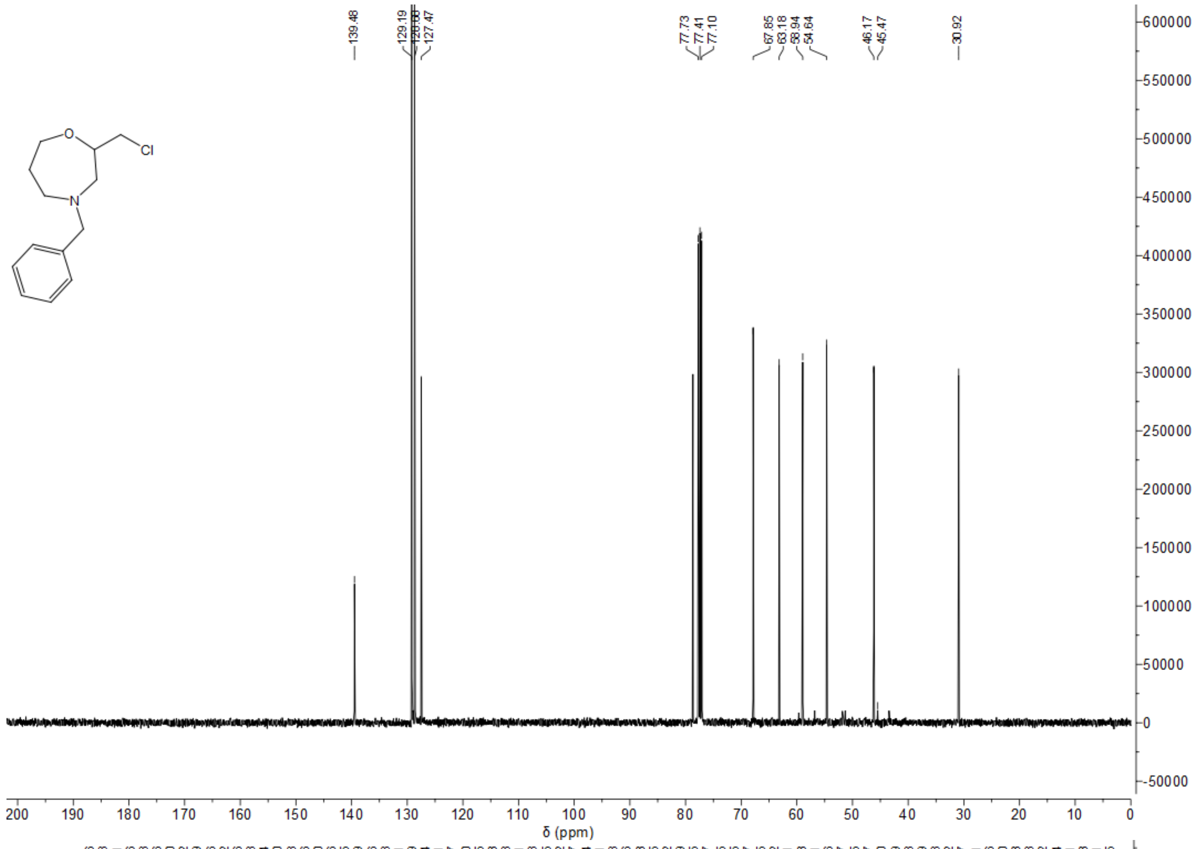

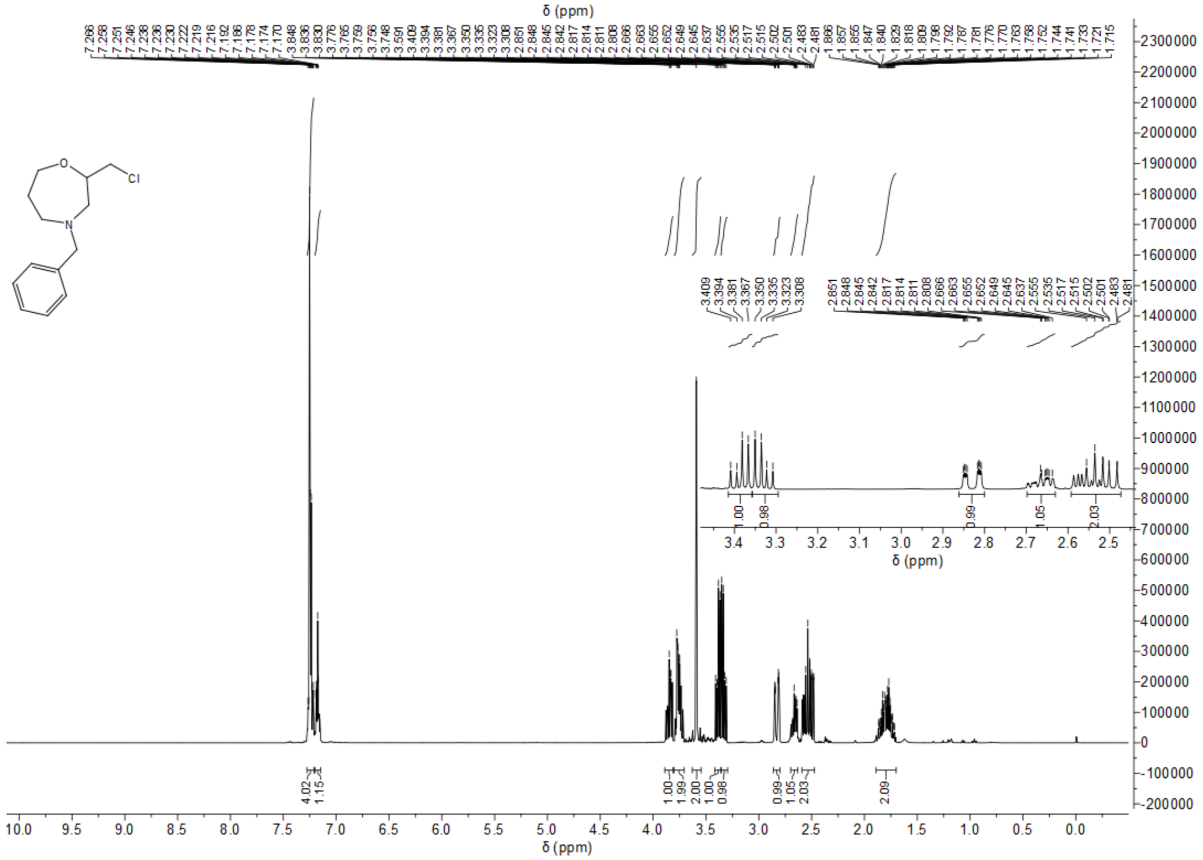

(6*S*)-4-benzyl-2-(chloromethyl)-6-methylmorpholine (32) [^{1}H-NMR data: 400 MHz, $CDCl_3$; $^{13}C\{^1H\}$-NMR data: 101 MHz, $CDCl_3$; 2D NMR spectra: HSQC, HMBC, COSY, all in $CDCl_3$]:

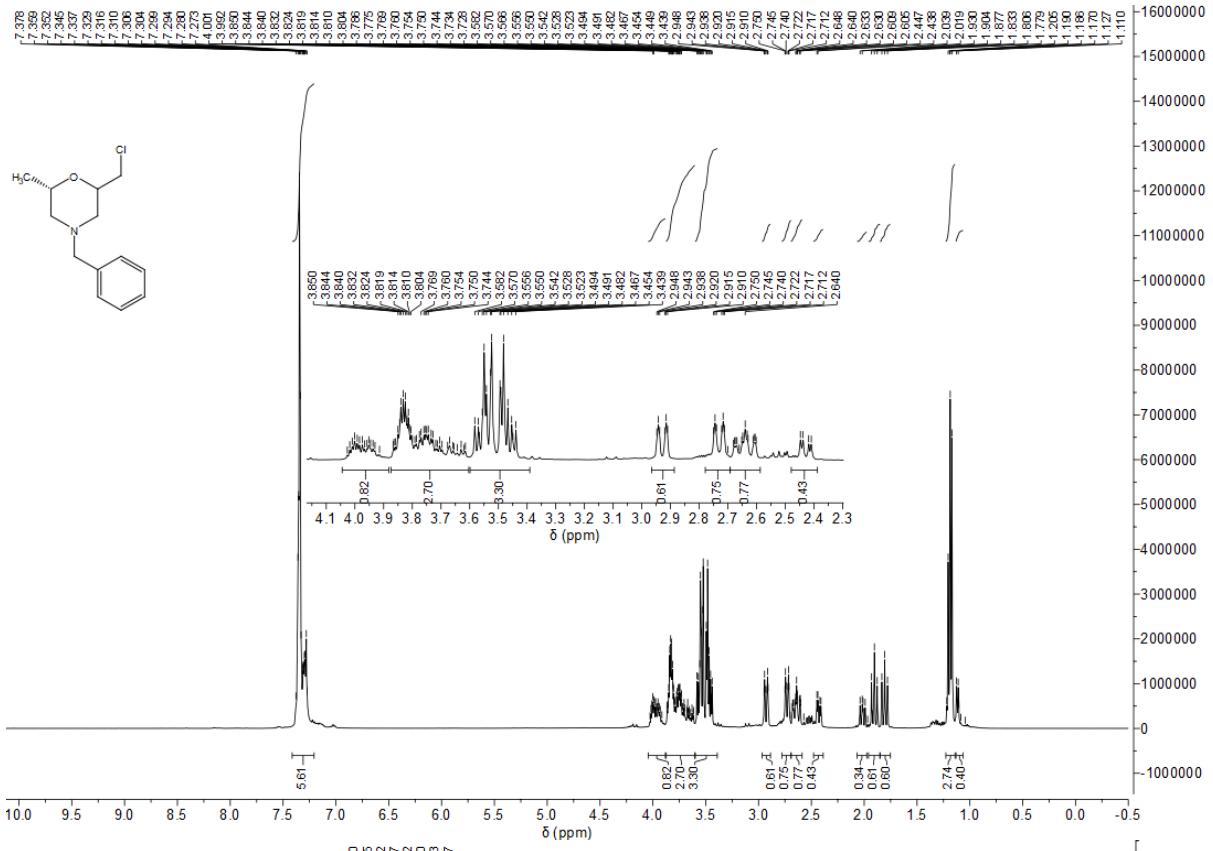

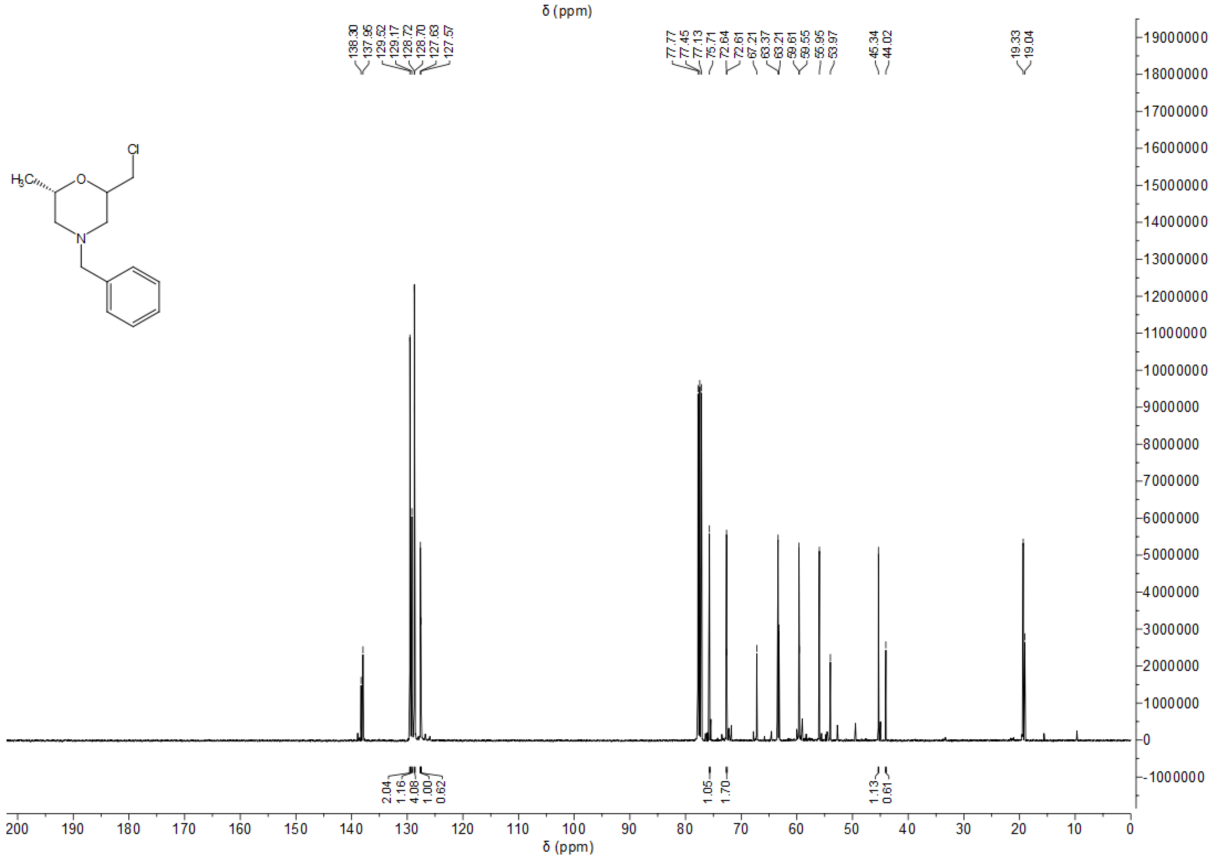

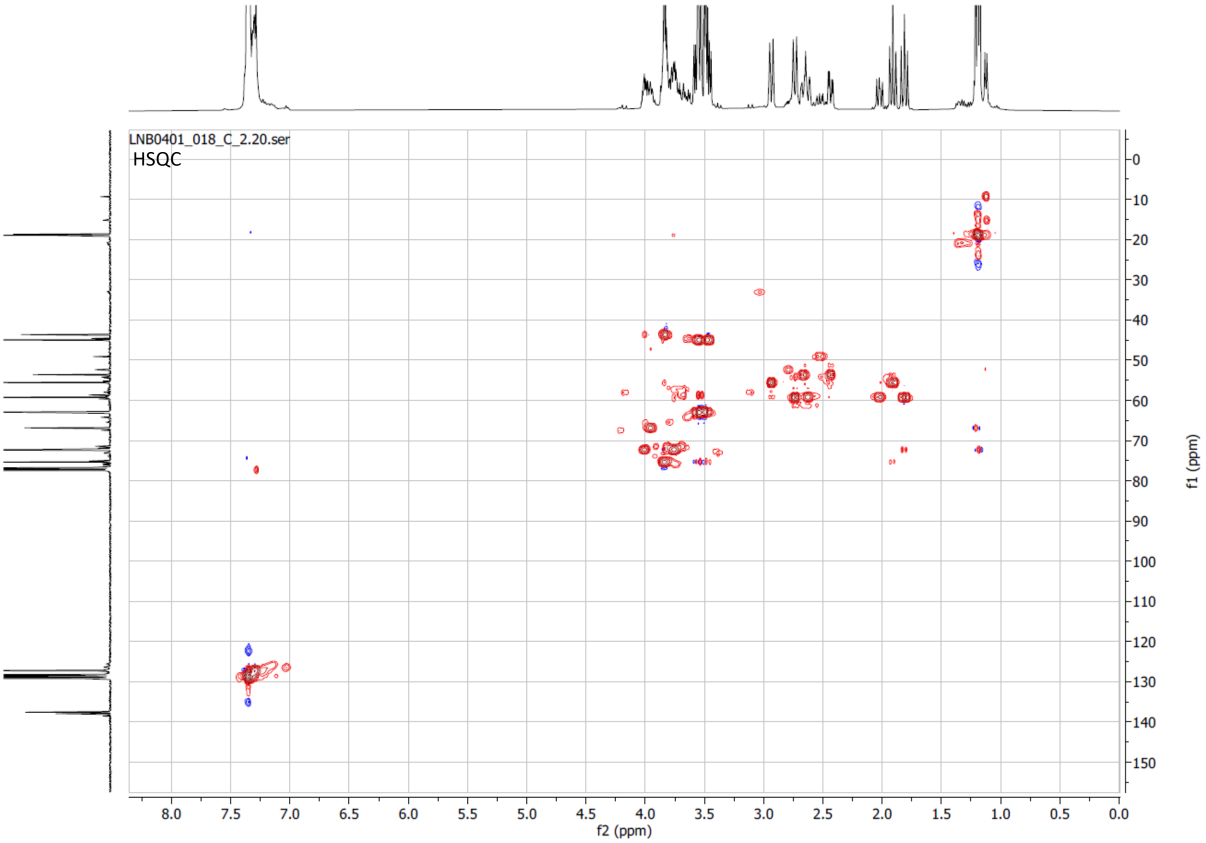
LNB0401_018_C_2.20.ser
HSQC
f2 (ppm)
f1 (ppm)

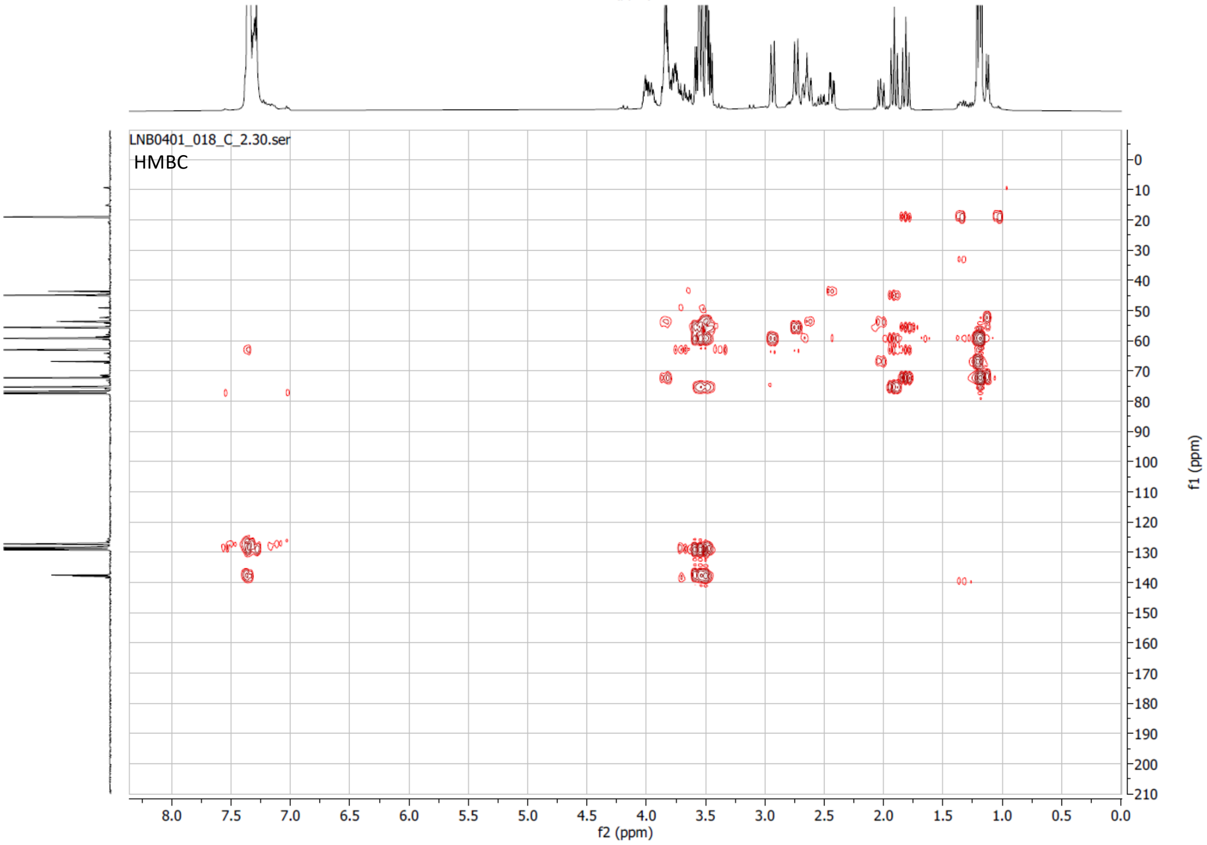
LNB0401_018_C_2.30.ser
HMBC
f2 (ppm)
f1 (ppm)

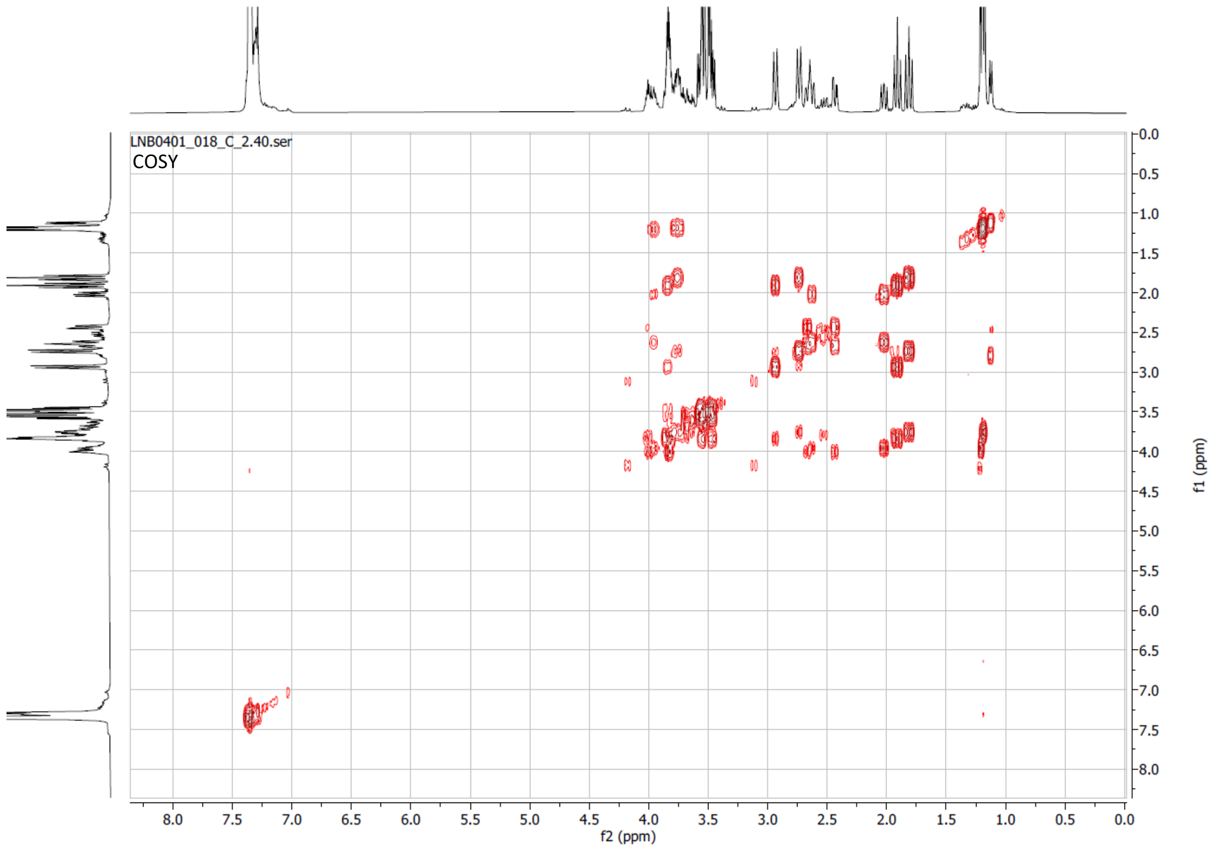
LNB0401_018_C_2.40.ser
COSY
f2 (ppm)
f1 (ppm)

(5*S*)-4-benzyl-2-(chloromethyl)-5-methylmorpholine (36) [^{1}H-NMR data: 400 MHz, $CDCl_3$; $^{13}C\{^{1}H\}$-NMR data: 101 MHz, $CDCl_3$; 2D NMR spectra: HSQC, HMBC, COSY, all in $CDCl_3$]:

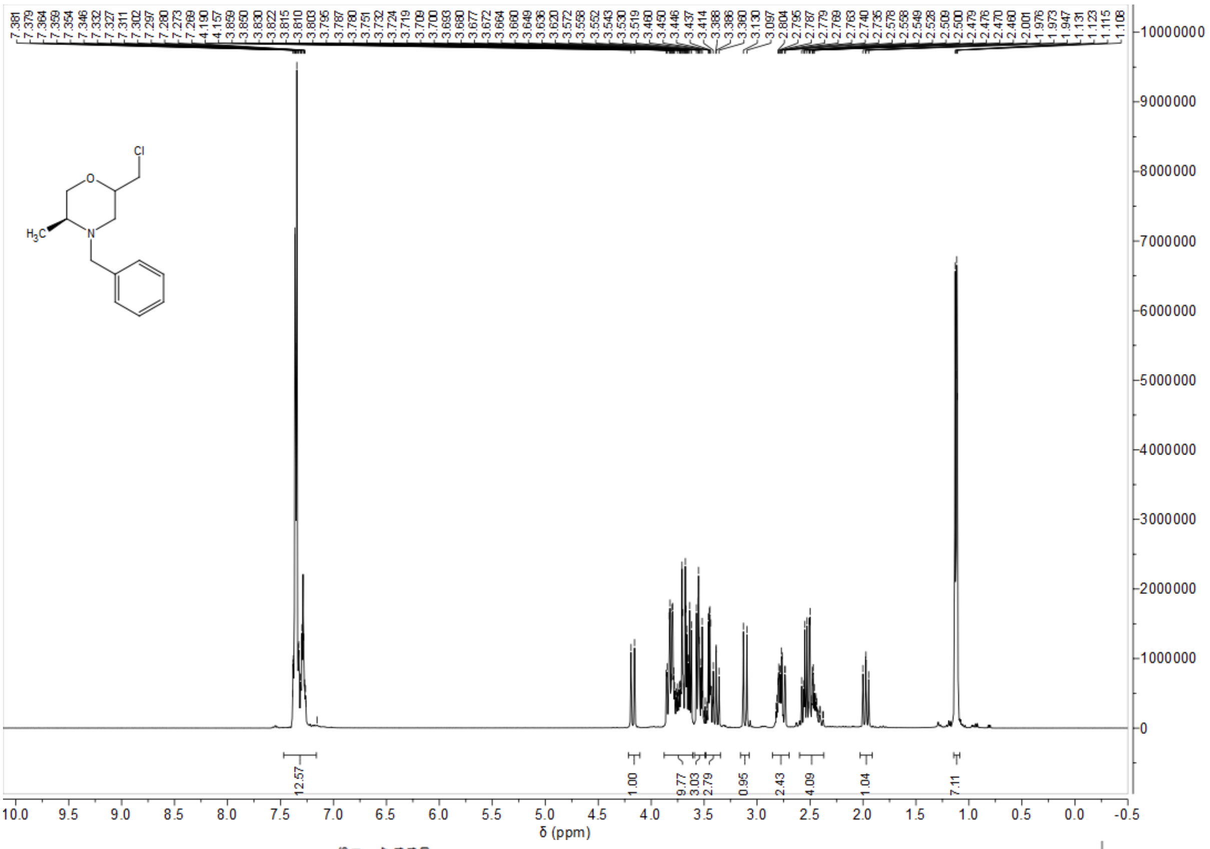

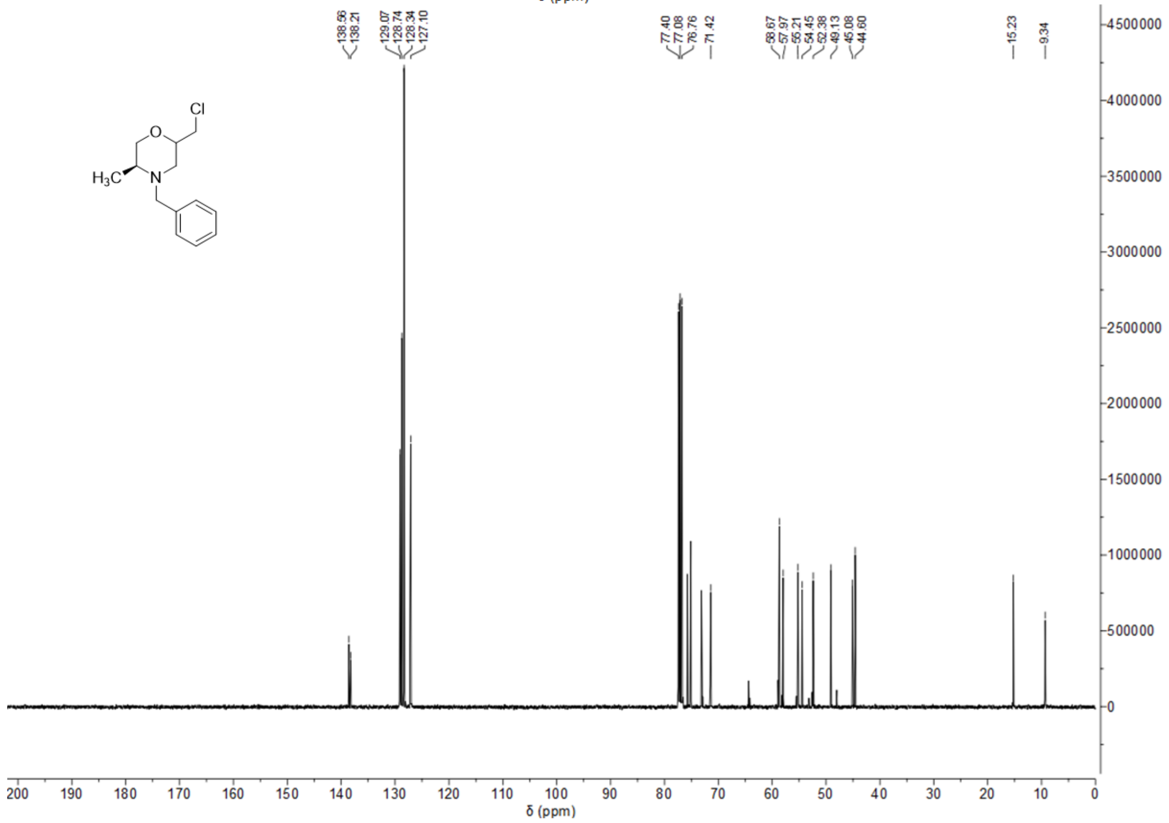

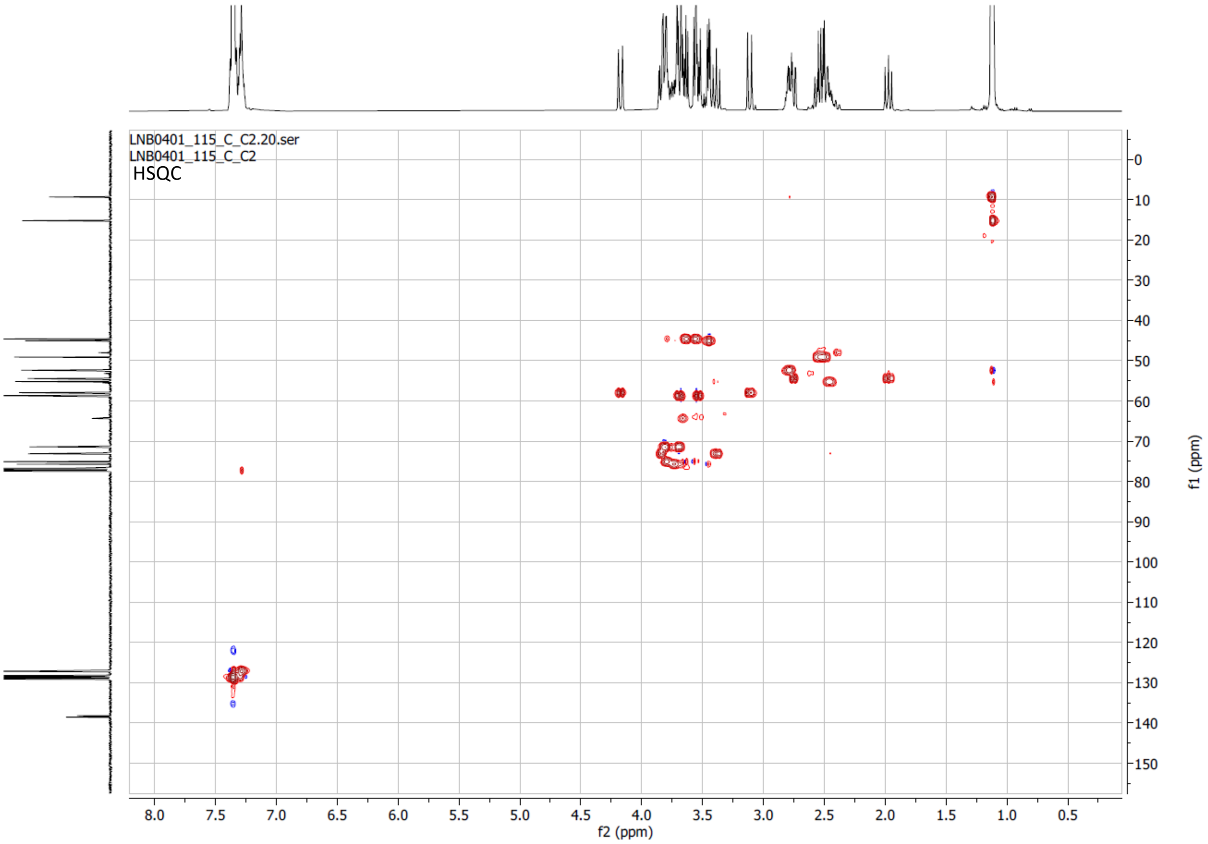
LNB0401_115_C_C2.20.ser
LNB0401_115_C_C2
HSQC
f2 (ppm)
f1 (ppm)

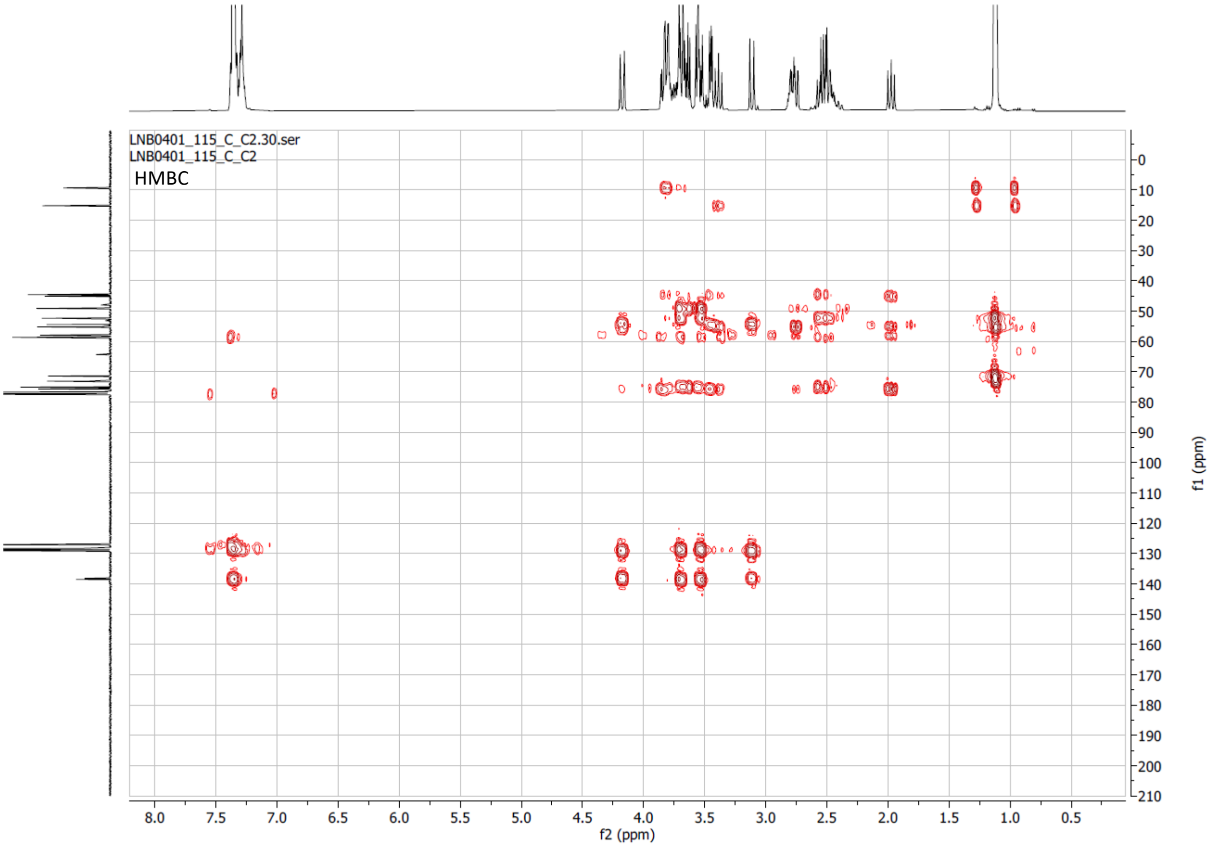
LNB0401_115_C_C2.30.ser
LNB0401_115_C_C2
HMBC
f2 (ppm)
f1 (ppm)

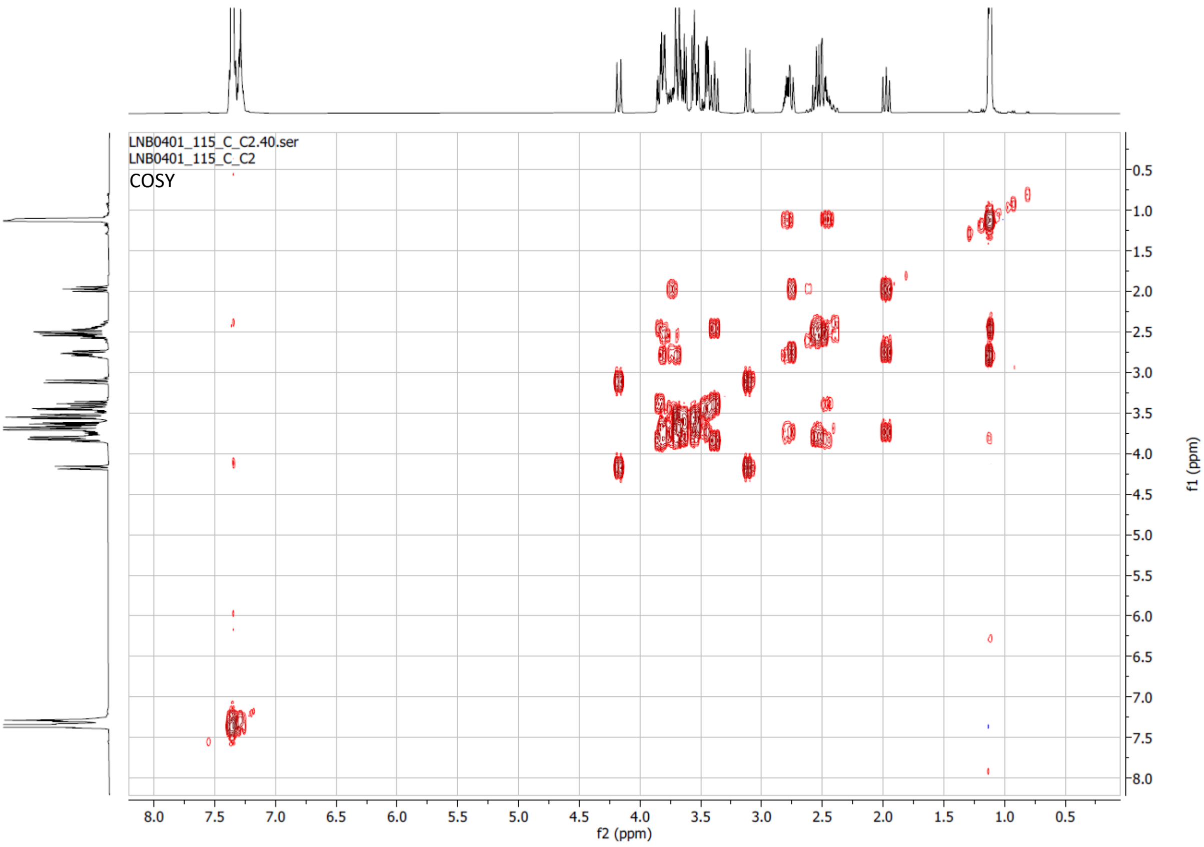

LNB0401_115_C_C2.40.ser
LNB0401_115_C_C2
COSY
f2 (ppm)
f1 (ppm)

1.4 Enol ethers

4-benzyl-2-methylenemorpholine (4) [^{1}H-NMR data: 400 MHz, $CDCl_3$; $^{13}C\{^1H\}$-NMR data: 101 MHz, $CDCl_3$]:

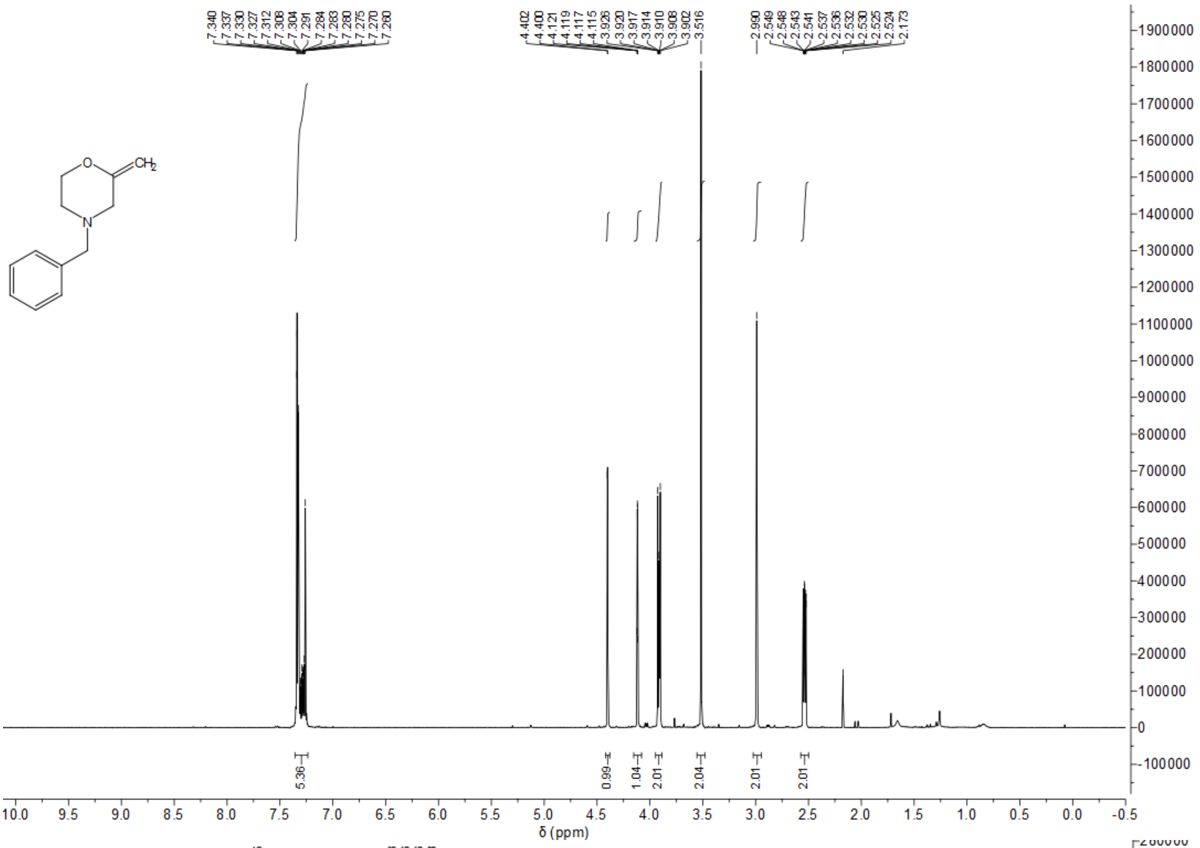

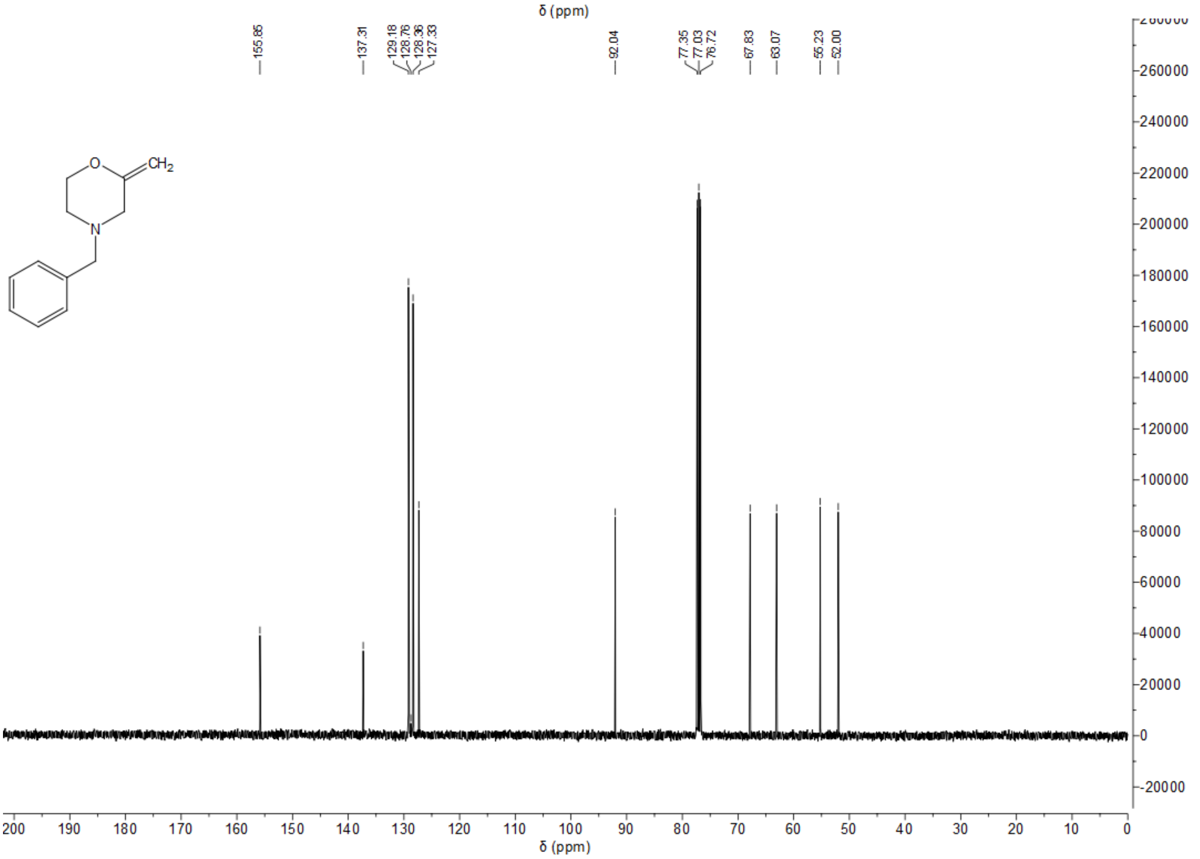

4-benzyl-2-methylene-1,4-oxazepane (21) [^{1}H-NMR data: 400 MHz, $CDCl_3$; $^{13}C\{^{1}H\}$-NMR data: 101 MHz, $CDCl_3$]:

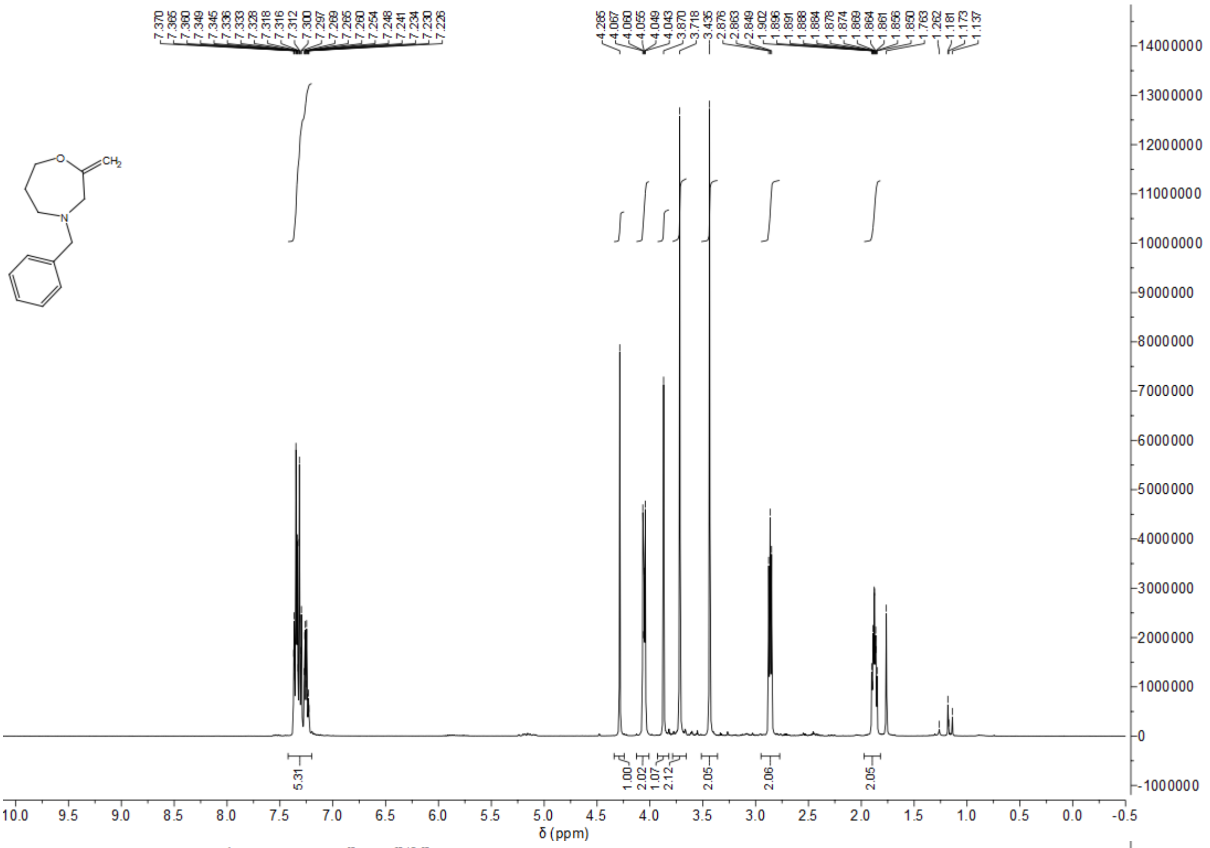

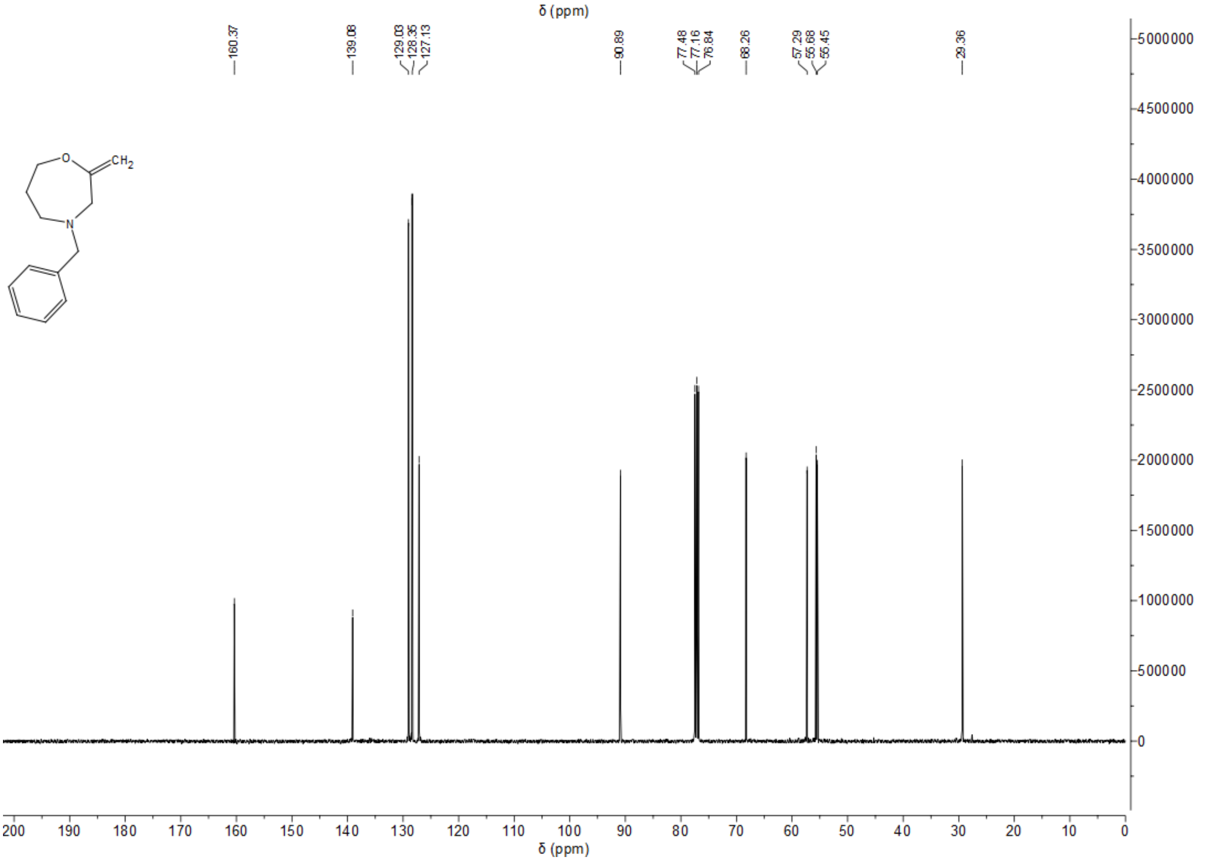

(*S*)-4-benzyl-2-methyl-6-methylenemorpholine (34) [^{1}H-NMR data: 400 MHz, C_6D_6; ^{13}C{^{1}H}-NMR data: 101 MHz, C_6D_6]:

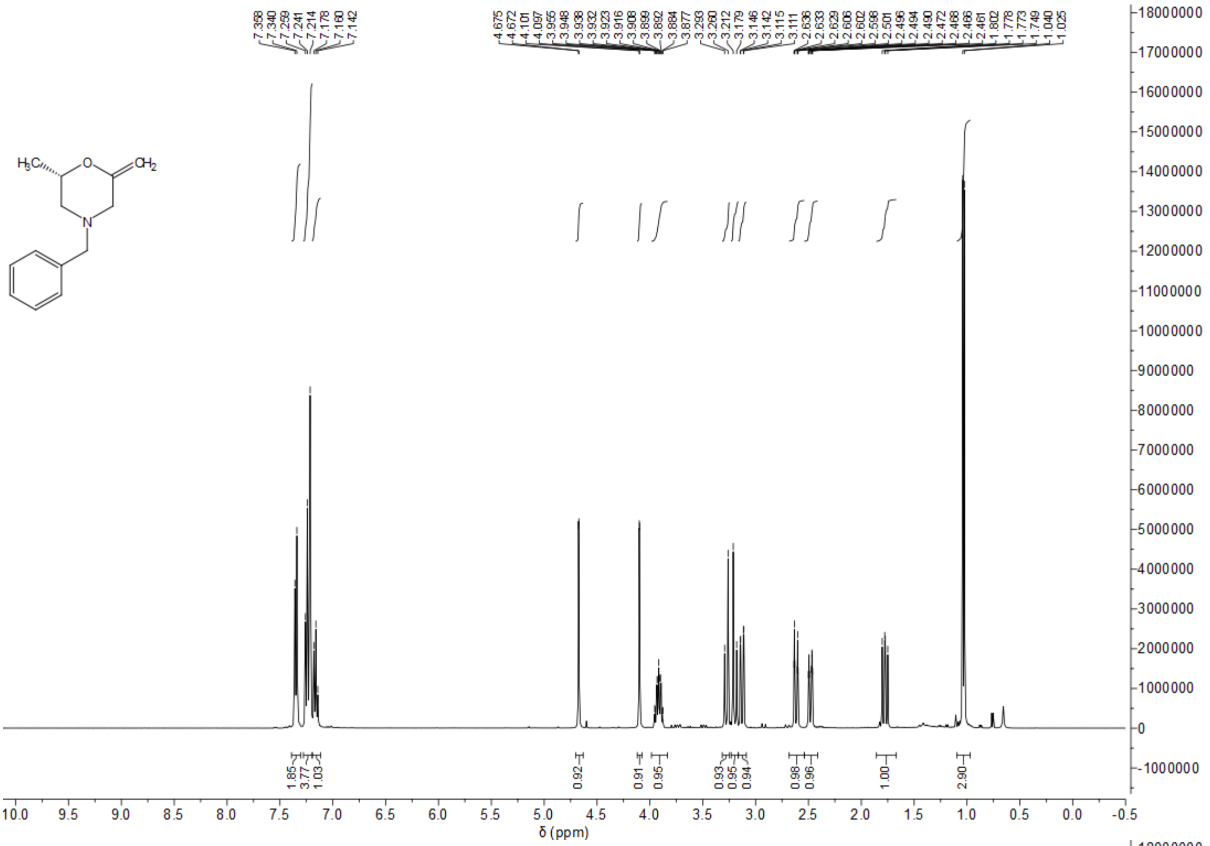

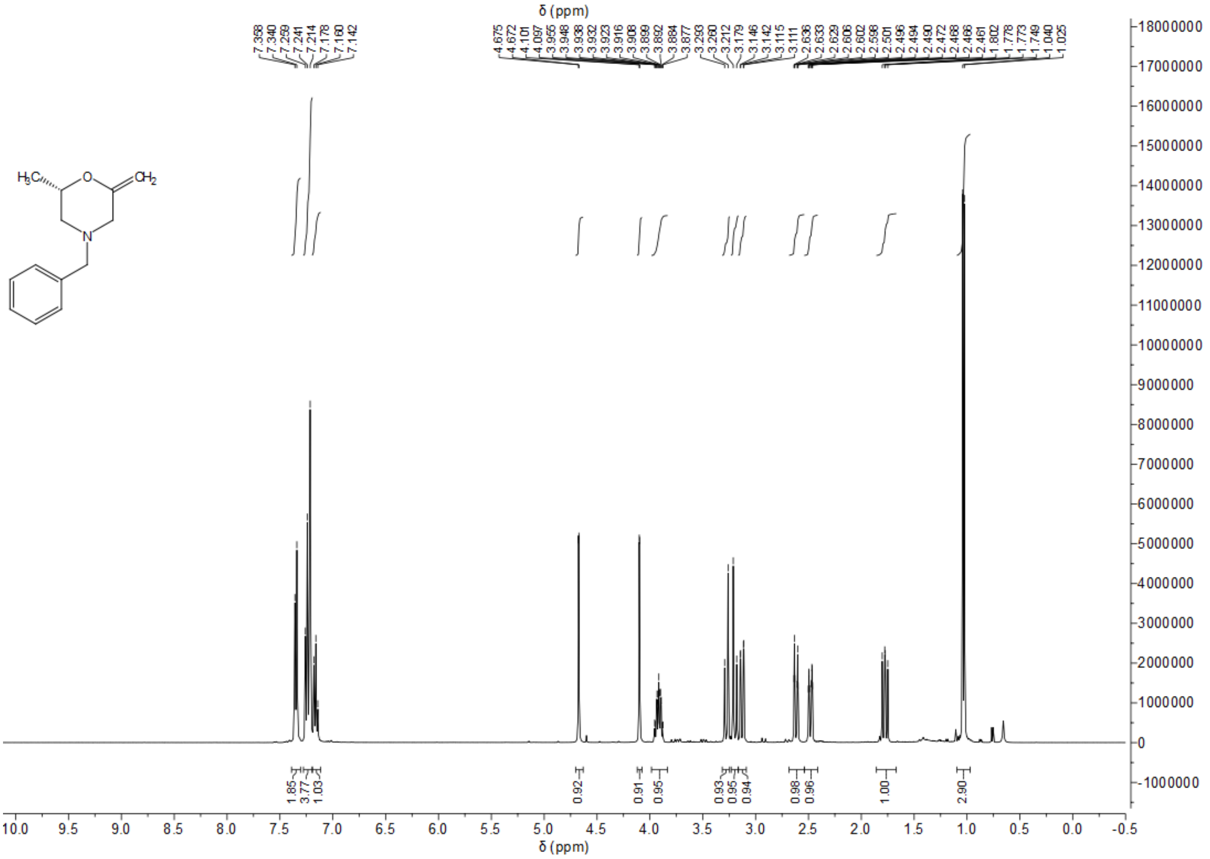

(*S*)-4-benzyl-5-methyl-2-methylenemorpholine (39) [^{1}H-NMR data: 400 MHz, $CDCl_3$; ^{13}C{^{1}H}-NMR data: 101 MHz, $CDCl_3$]:

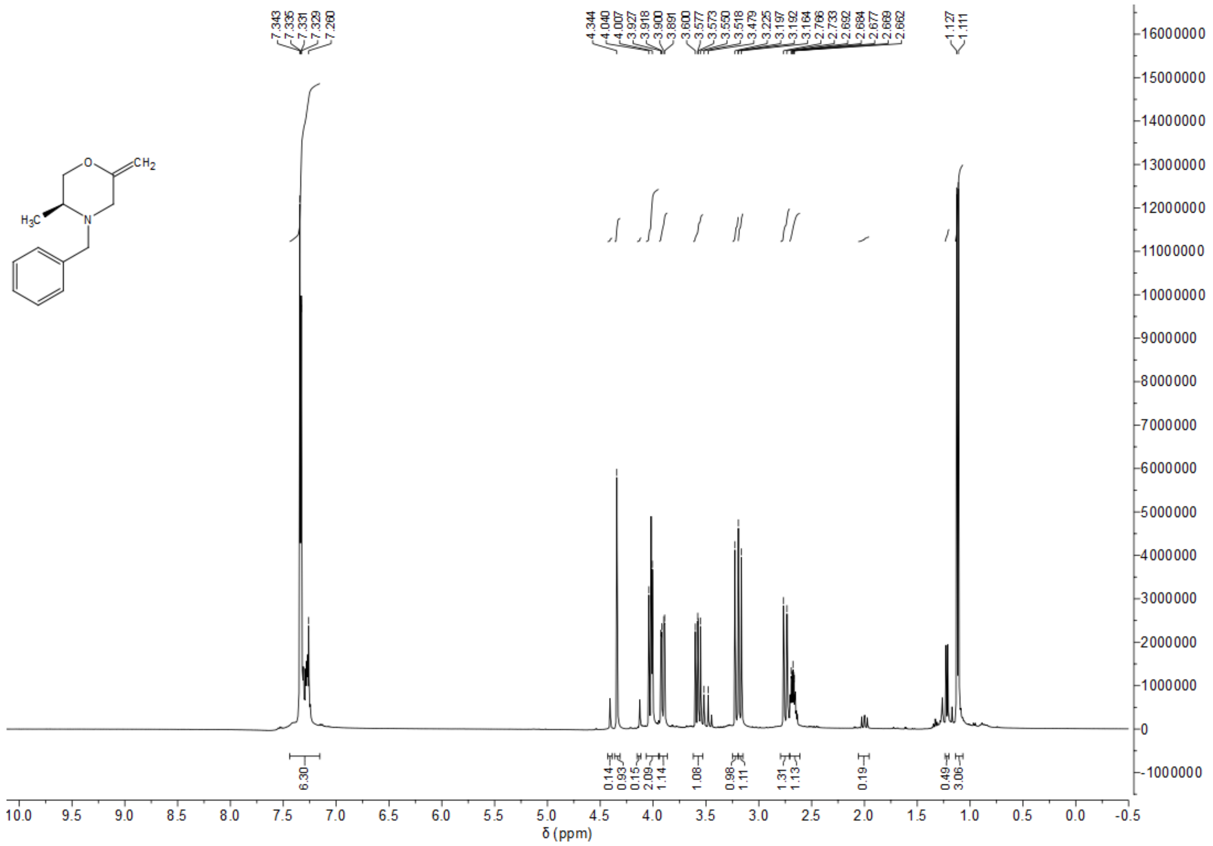

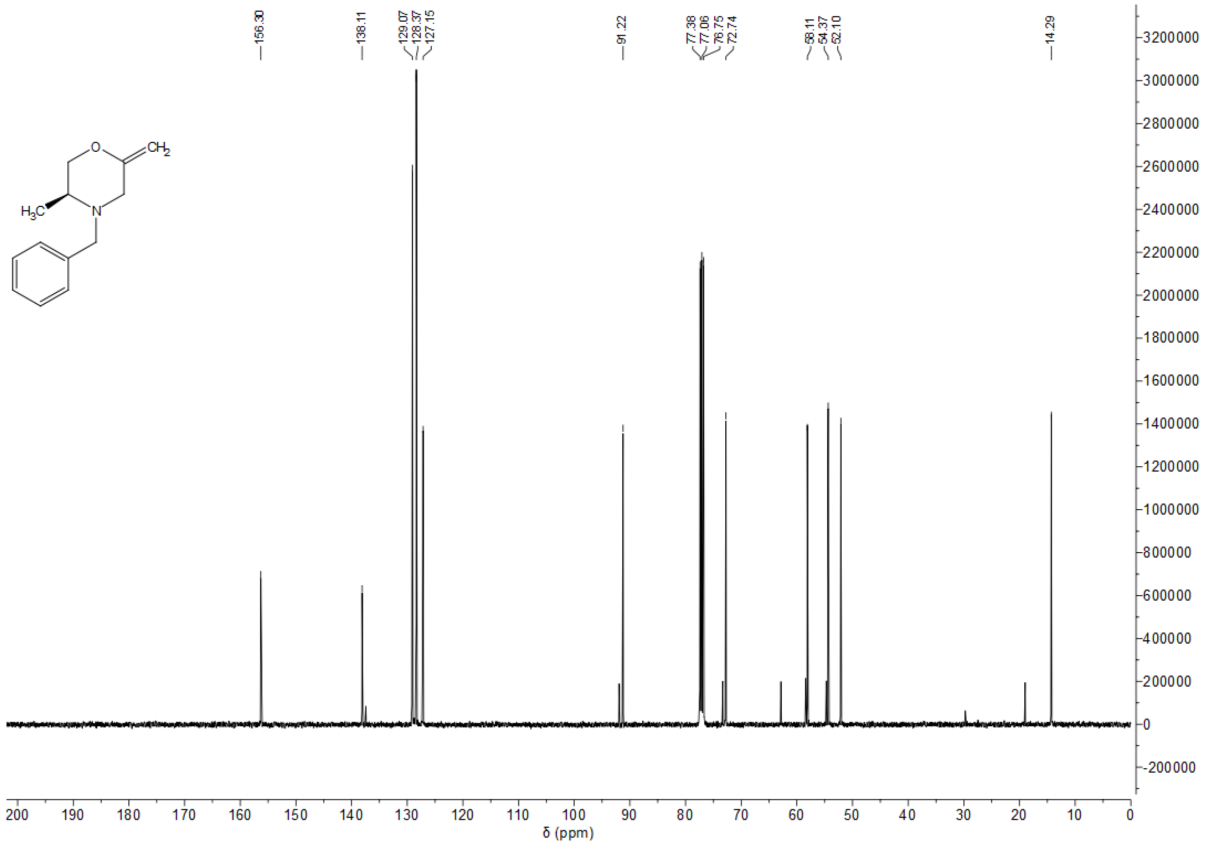

1.5 Spiroacetals

1.5.1 Iodoacetals and unsubstituted spiroacetals

***tert*-butyl (2-((4-benzyl-2-(iodomethyl)morpholin-2-yl)oxy)ethyl)carbamate (6) [^{1}H-NMR data: 400 MHz, C_6D_6; ^{13}C{^{1}H}-NMR data: 101 MHz, C_6D_6]:**

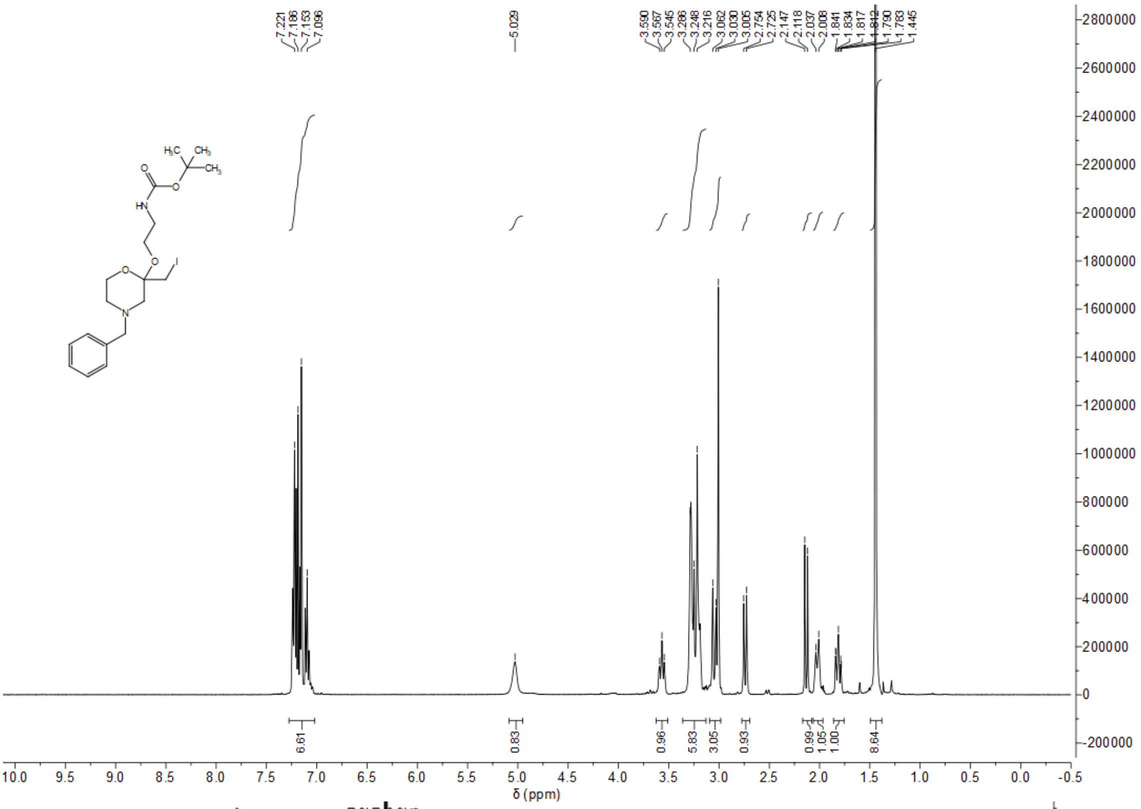

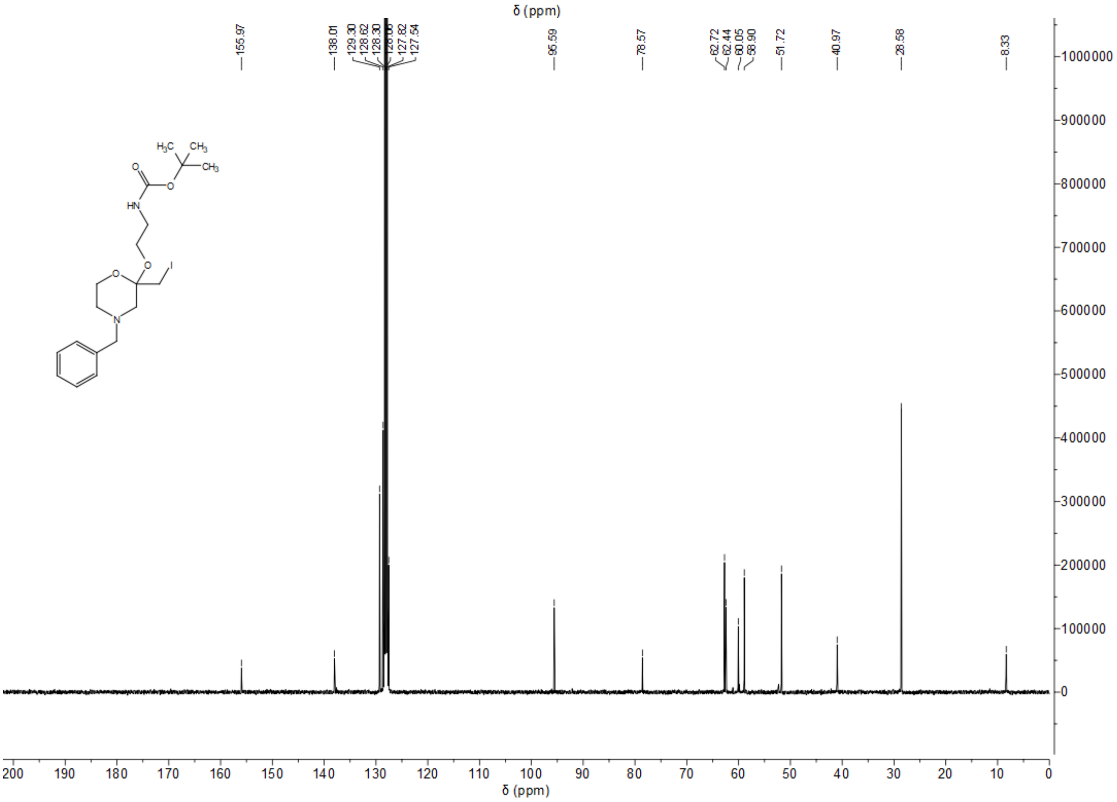

***tert*-butyl 10-benzyl-1,7-dioxa-4,10-diazaspiro[5.5]undecane-4-carboxylate (7) [^{1}H-NMR spectrum: 400 MHz, C_6D_6; $^{13}C\{^{1}H\}$-NMR spectrum: 101 MHz, C_6D_6; 2D NMR spectra: HSQC, HMBC, COSY, NOESY, all in C_6D_6]:**

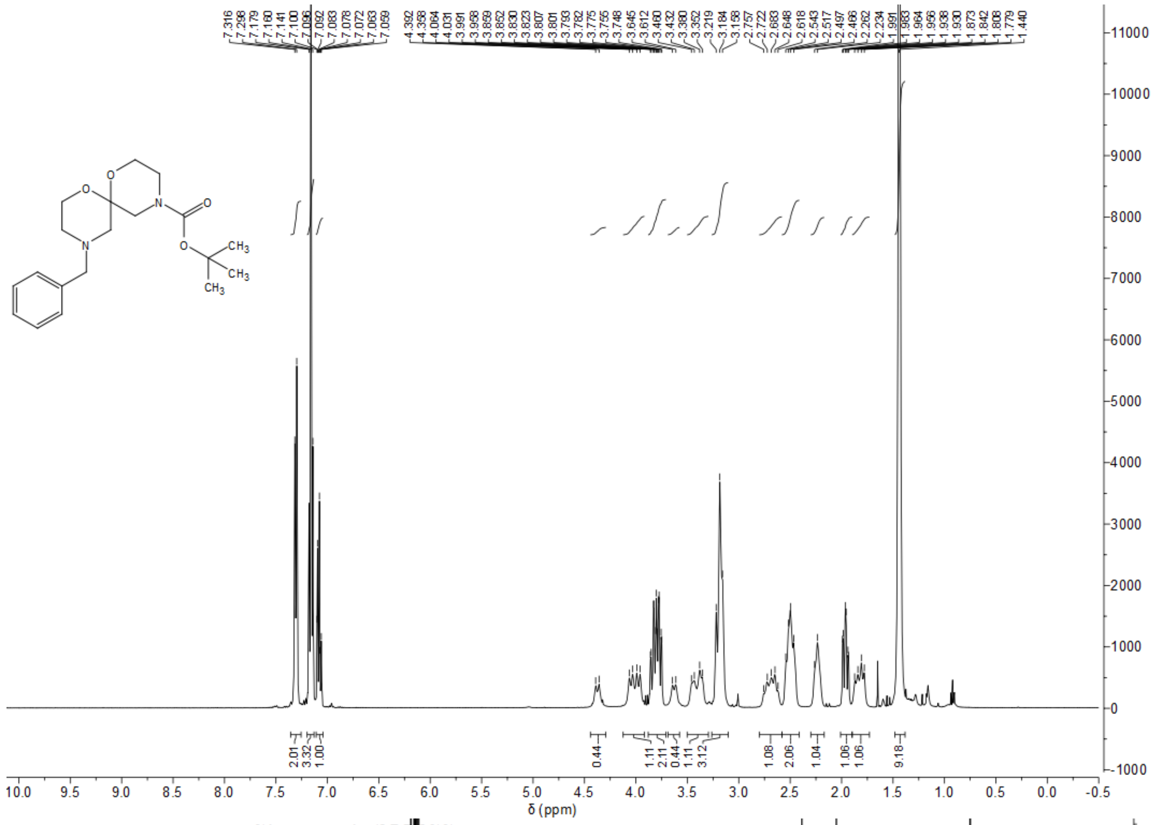

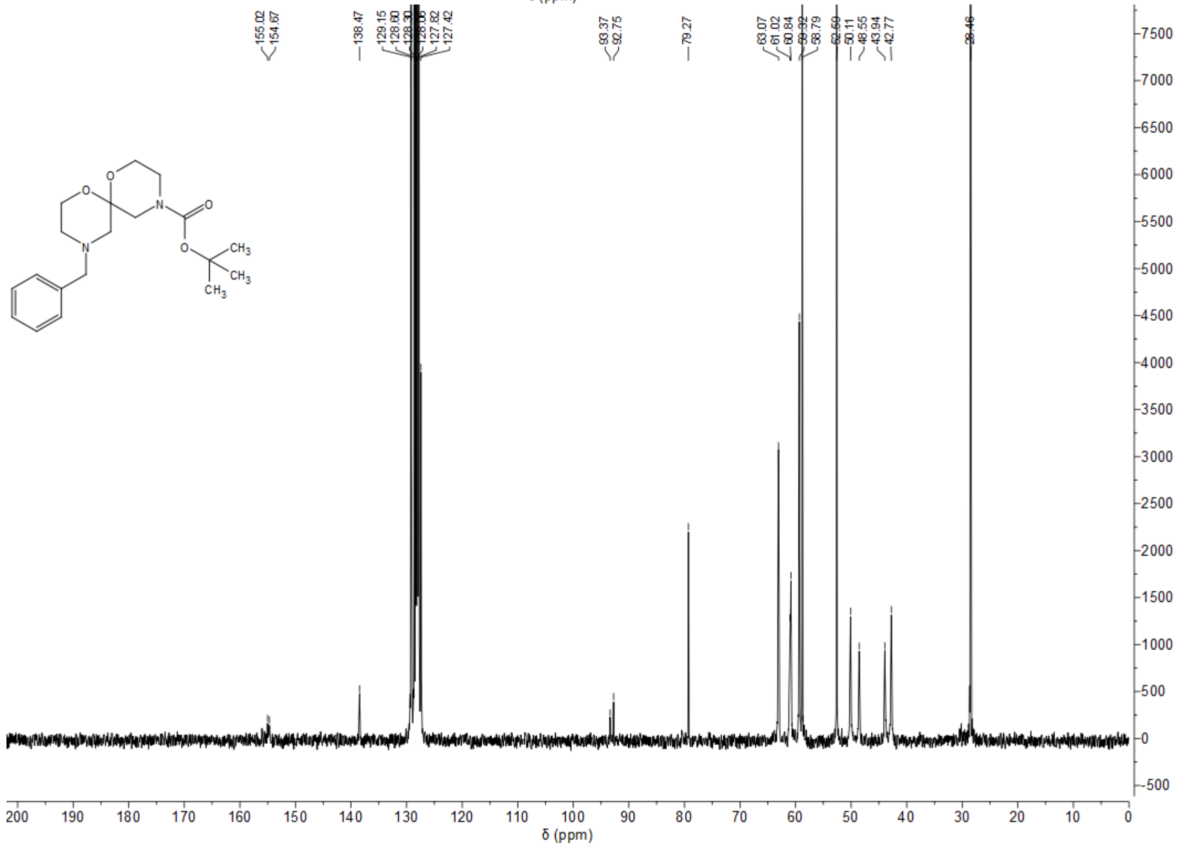

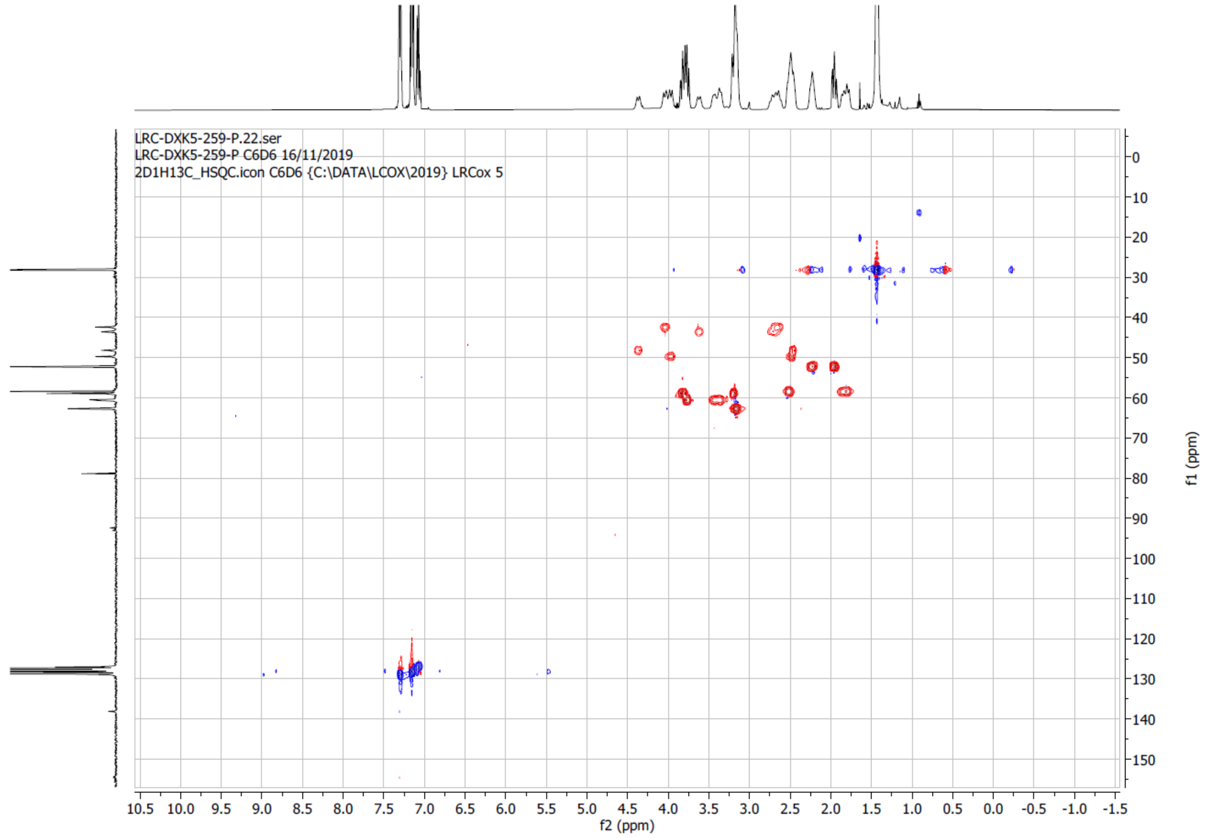
LRC-DXK5-259-P.22.ser
LRC-DXK5-259-P C6D6 16/11/2019
2D1H13C_HSQC.icon C6D6 {C:\DATA\LCOX\2019} LRCox 5
f2 (ppm)
f1 (ppm)

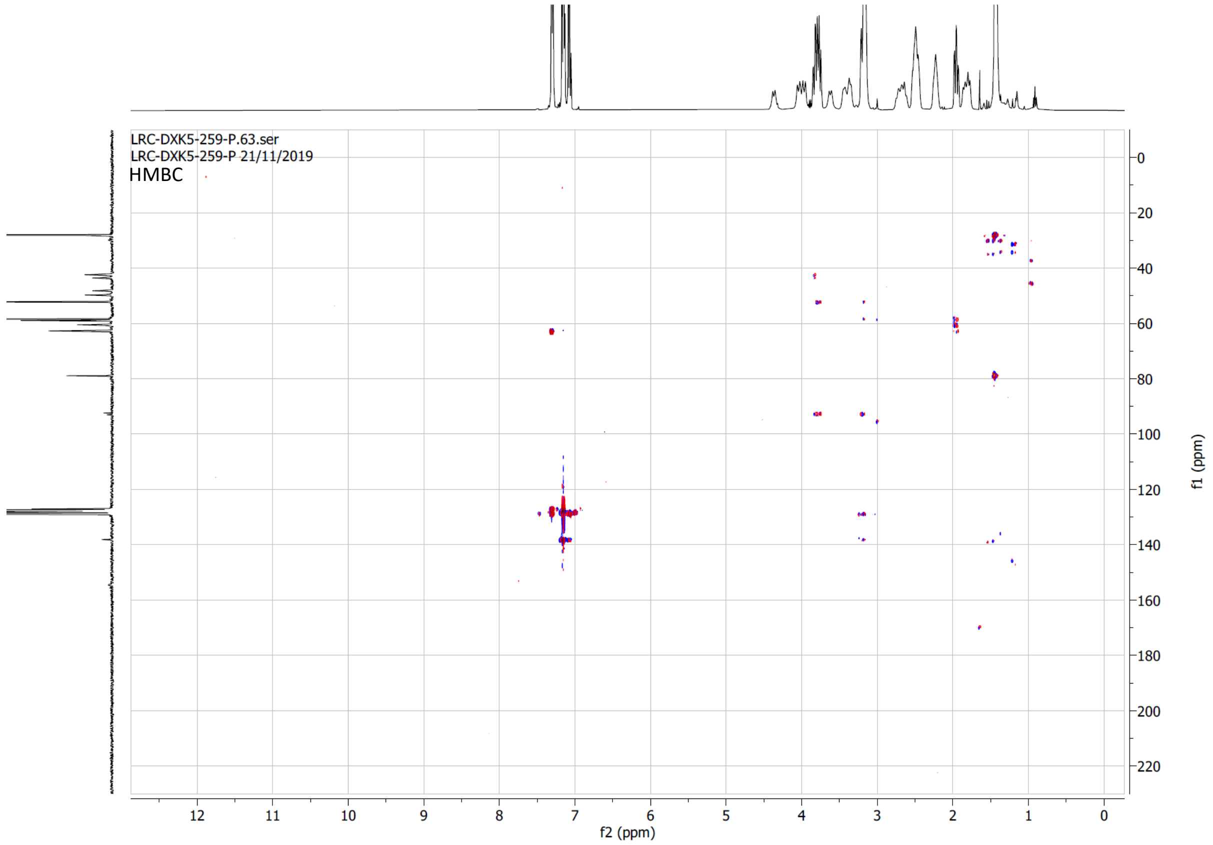
LRC-DXK5-259-P.63.ser
LRC-DXK5-259-P 21/11/2019
HMBC
f2 (ppm)
f1 (ppm)

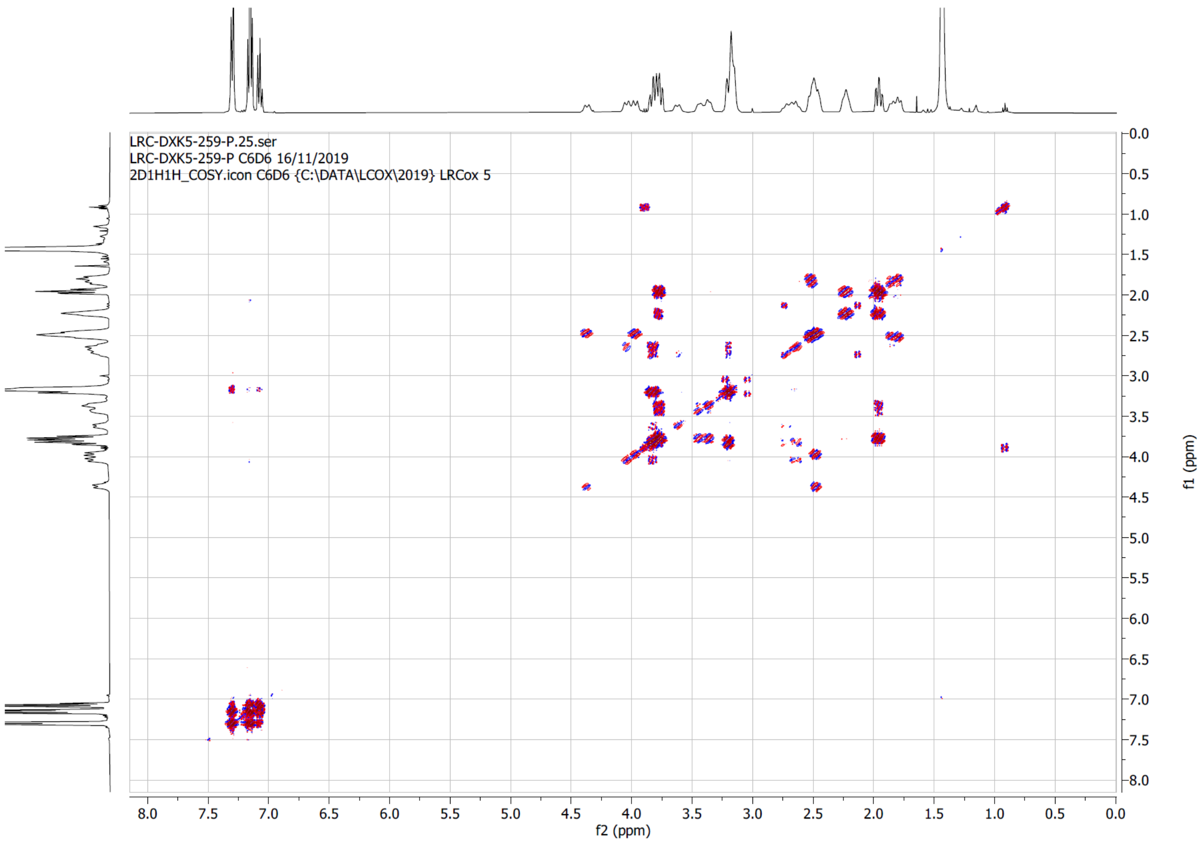
LRC-DXK5-259-P.25.ser
LRC-DXK5-259-P C6D6 16/11/2019
2D1H1H_COSY.icon C6D6 {C:\DATA\LCOX\2019} LRCox 5
f1 (ppm)
f2 (ppm)

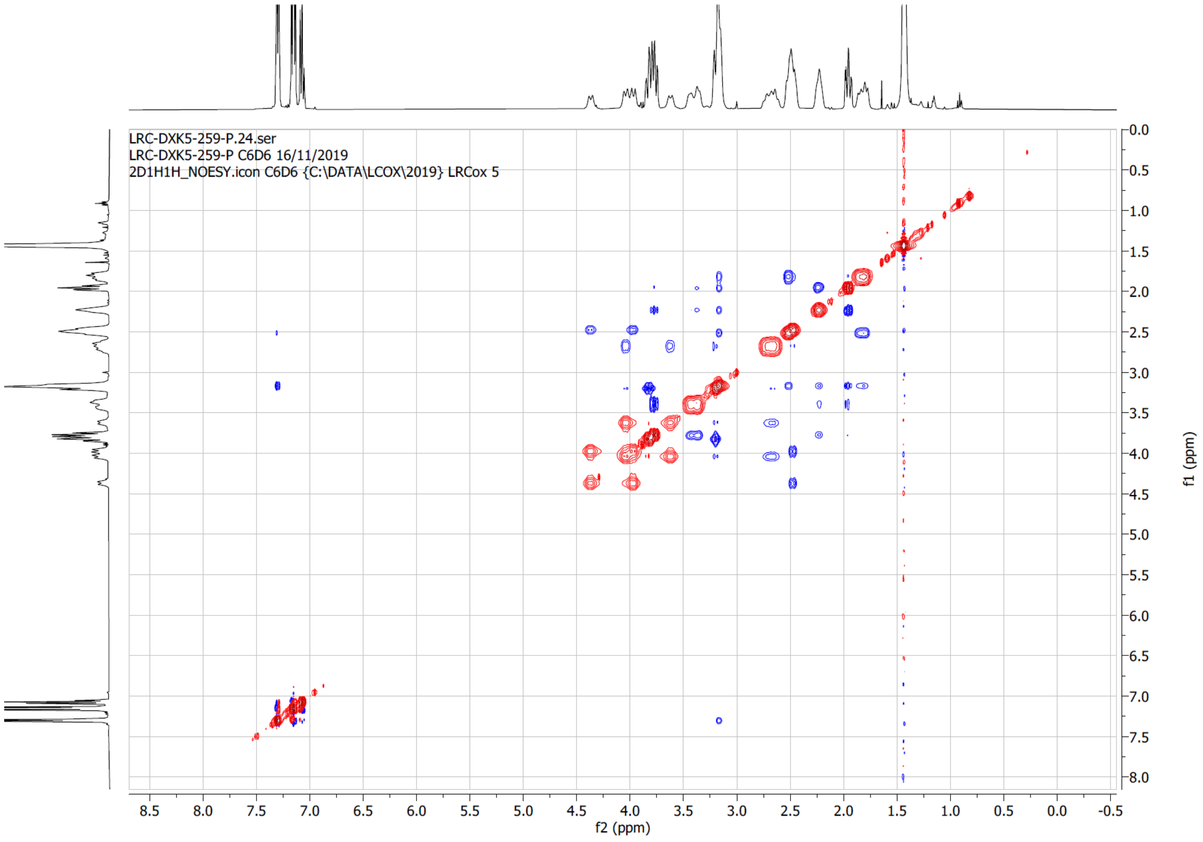
LRC-DXK5-259-P.24.ser
LRC-DXK5-259-P C6D6 16/11/2019
2D1H1H_NOESY.icon C6D6 {C:\DATA\LCOX\2019} LRCox 5
f1 (ppm)
f2 (ppm)

4-benzyl 10-(*tert*-butyl) 1,7-dioxa-4,10-diazaspiro[5.5]undecane-4,10-dicarboxylate (8) [^{1}H-NMR spectrum: 400 MHz, C_6D_6; $^{13}C\{^1H\}$-NMR spectrum: 101 MHz, C_6D_6; 2D NMR spectra: HSQC, HMBC, all in C_6D_6]:

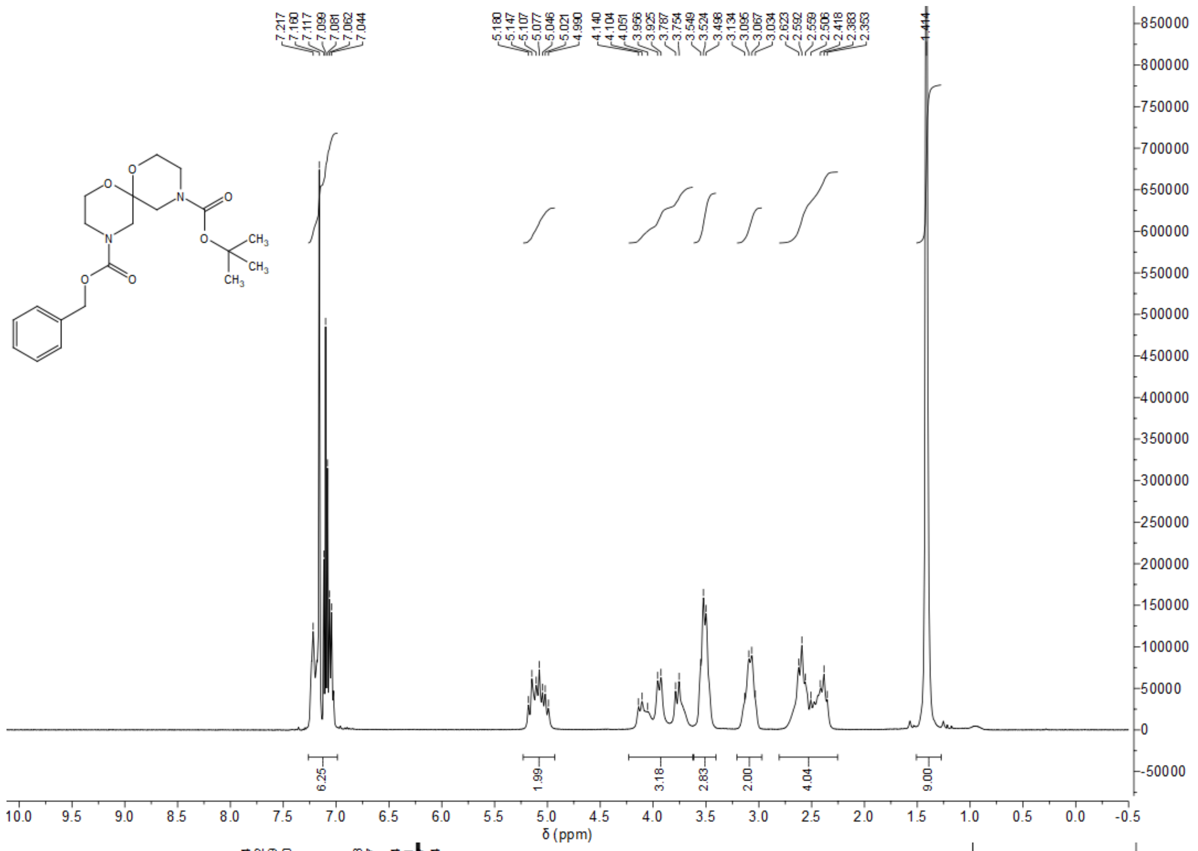

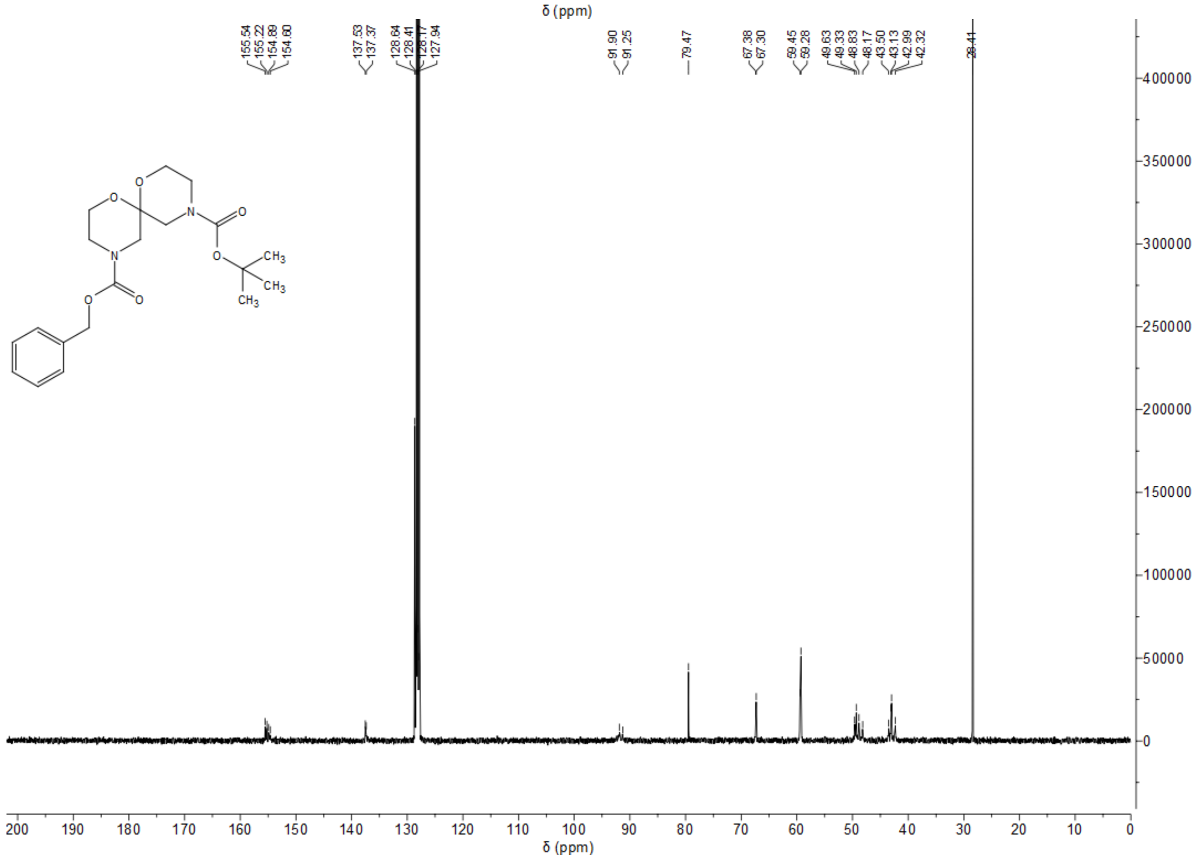

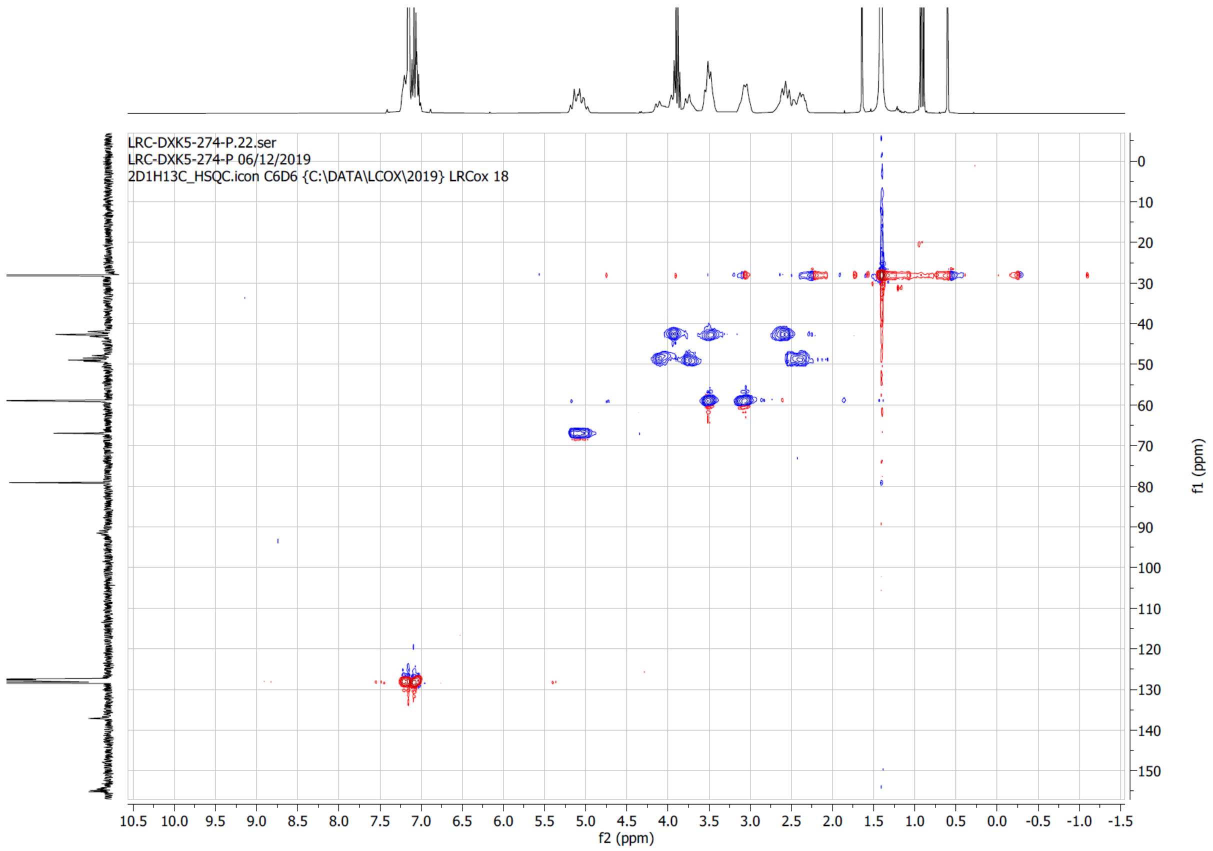
LRC-DXK5-274-P.22.ser
LRC-DXK5-274-P 06/12/2019
2D1H13C_HSQC.icon C6D6 {C:\DATA\LCOX\2019} LRCox 18
f2 (ppm)
f1 (ppm)

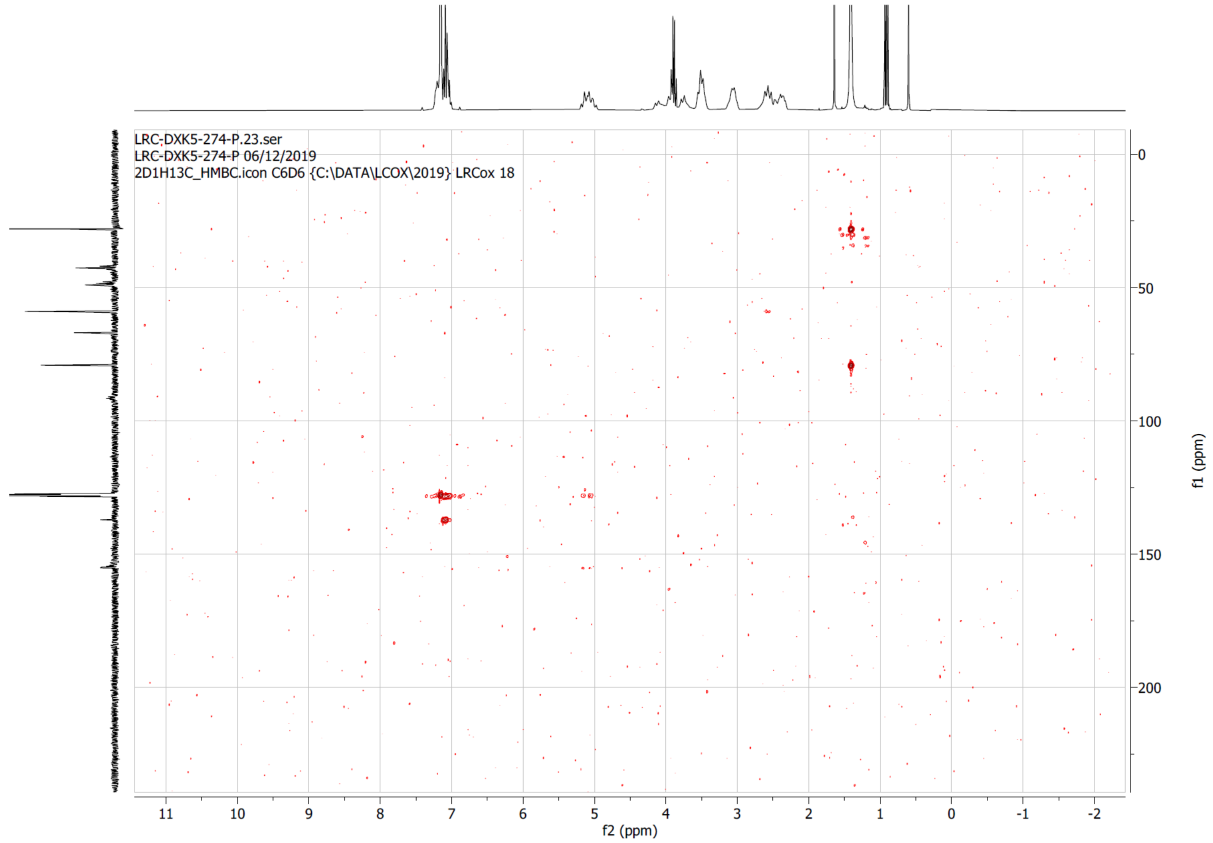
LRC-DXK5-274-P.23.ser
LRC-DXK5-274-P 06/12/2019
2D1H13C_HMBC.icon C6D6 {C:\DATA\LCOX\2019} LRCox 18
f2 (ppm)
f1 (ppm)

tert-butyl 1,7-dioxa-4,10-diazaspiro[5.5]undecane-4-carboxylate (9) [^{1}H-NMR data: 400 MHz, CD_3OD; $^{13}C\{^{1}H\}$-NMR data: 101 MHz, CD_3OD]:

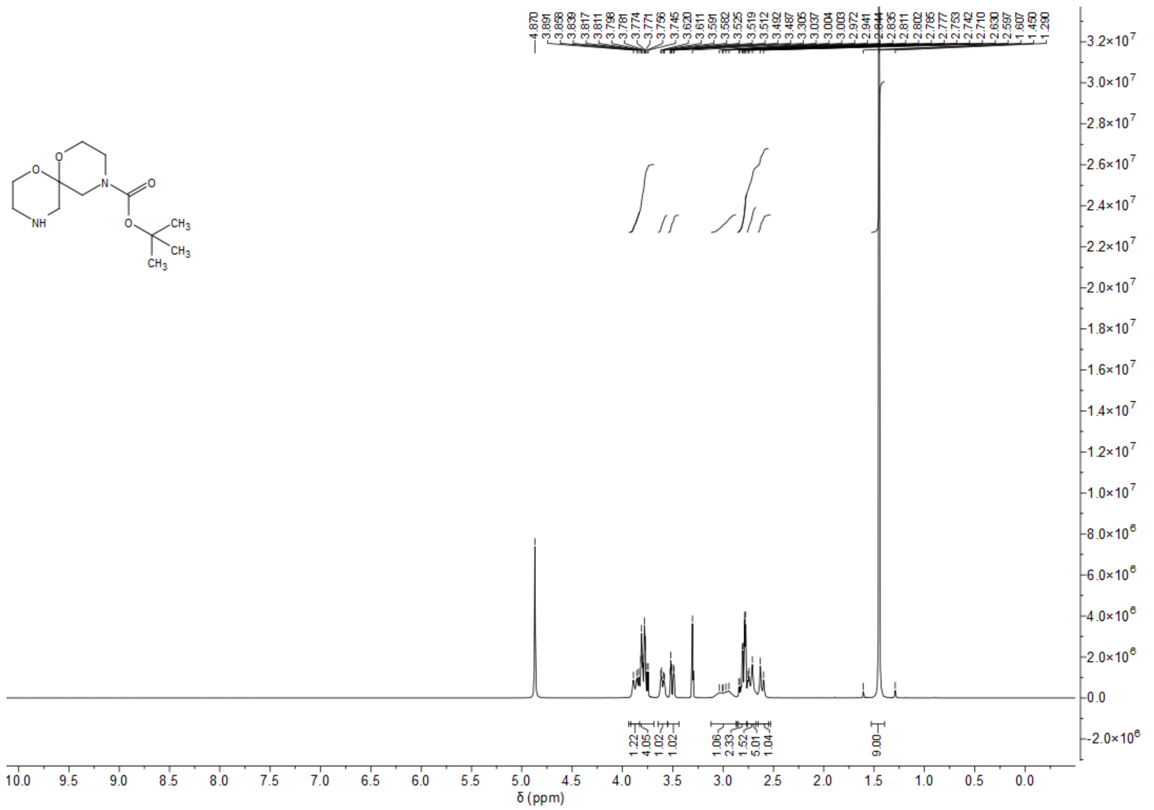

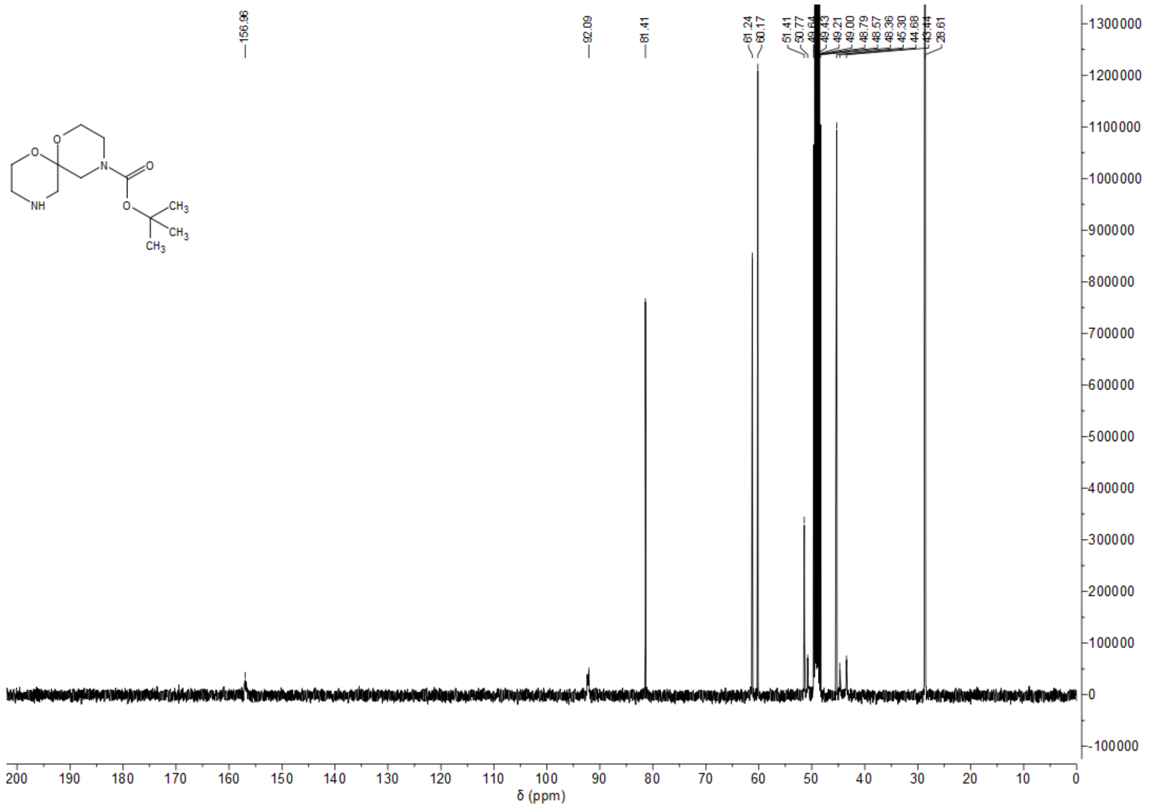

***tert*-butyl 10-methyl-1,7-dioxa-4,10-diazaspiro[5.5]undecane-4-carboxylate (S20) [^{1}H-NMR data: 400 MHz, CD_3OD; $^{13}C\{^{1}H\}$-NMR data: 101 MHz, CD_3OD]:**

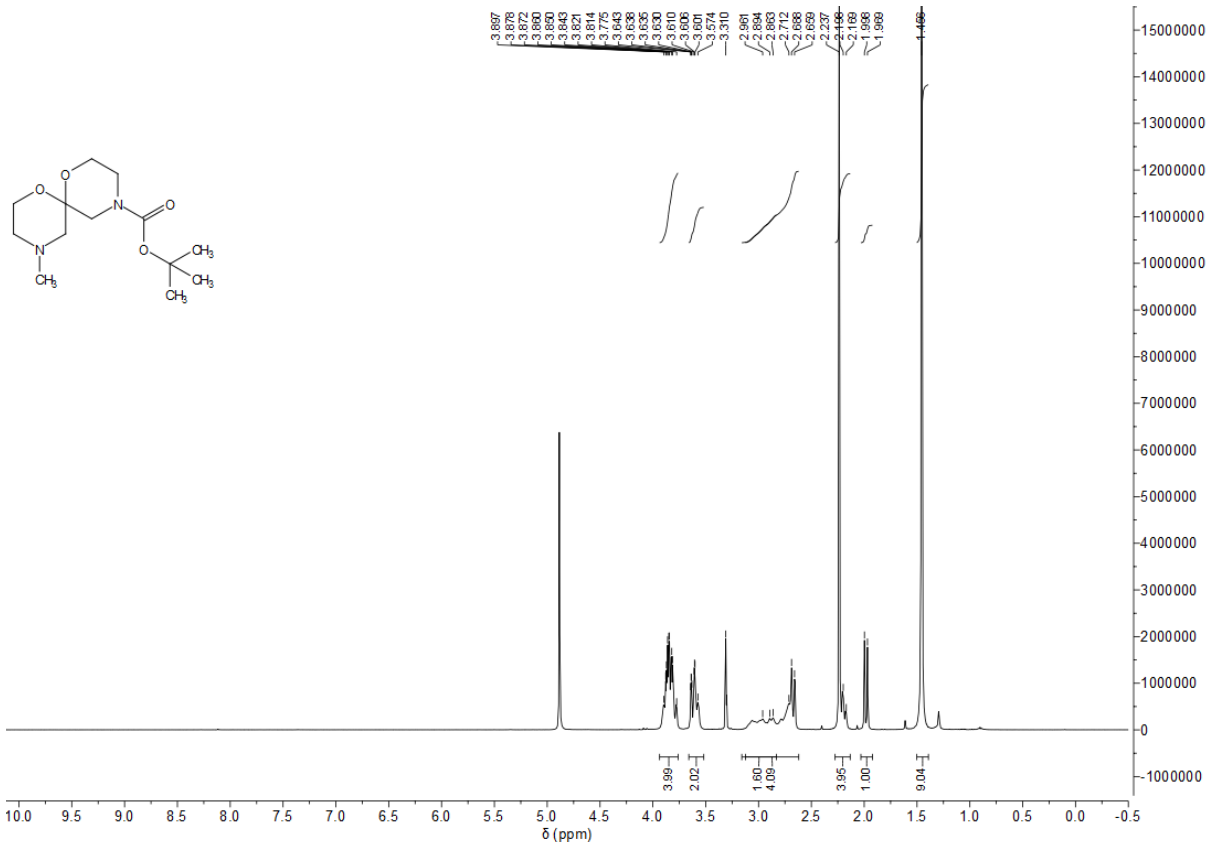

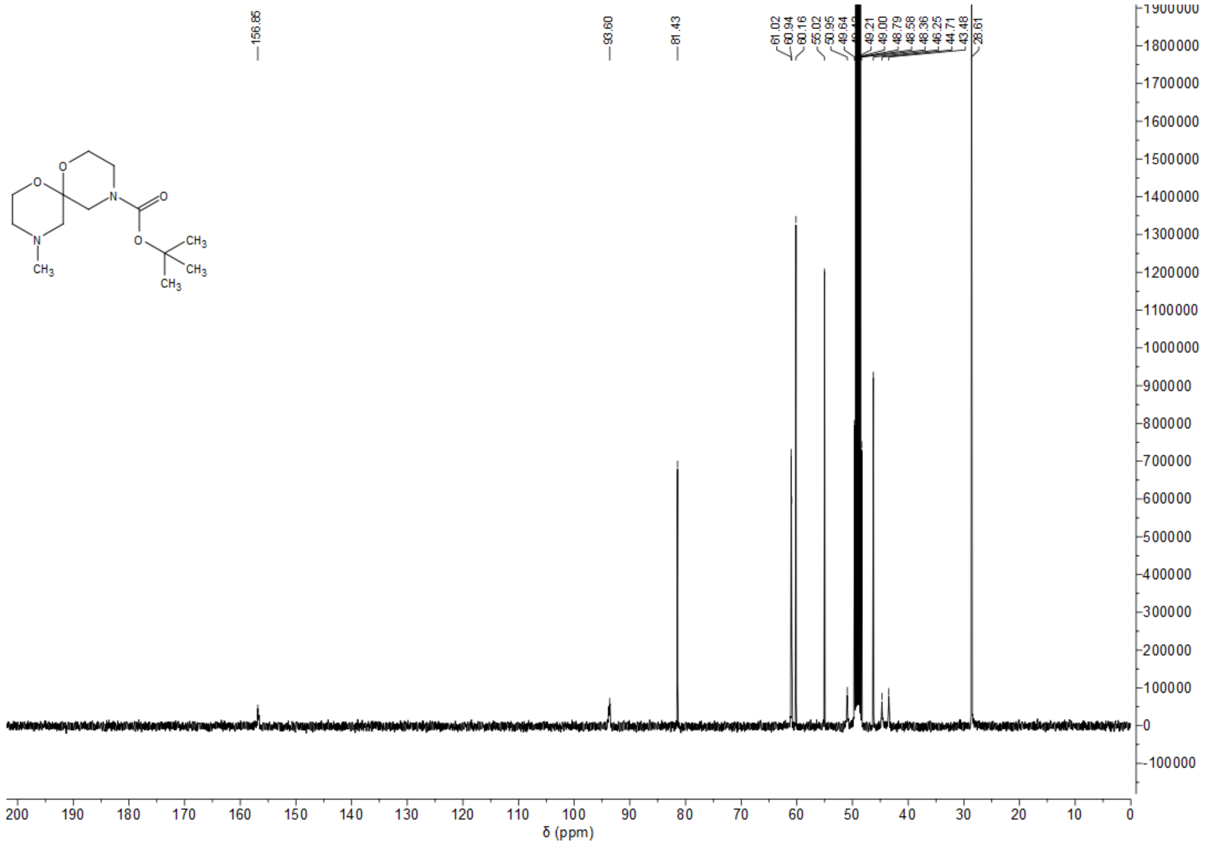

benzyl 1,7-dioxa-4,10-diazaspiro[5.5]undecane-4-carboxylate hydrochloride (10·HCl) [^{1}H-NMR data: 400 MHz, CD_3OD; $^{13}C\{^{1}H\}$-NMR data: 101 MHz, CD_3OD]:

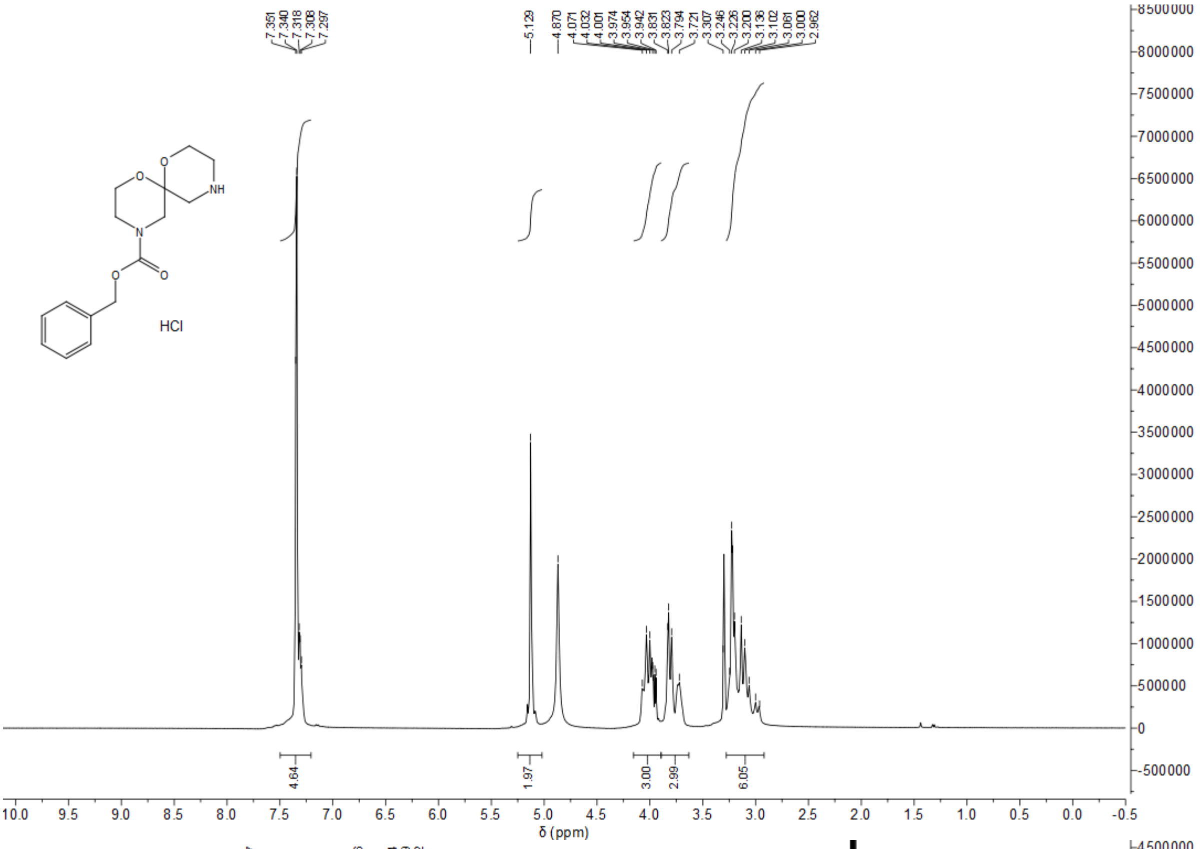

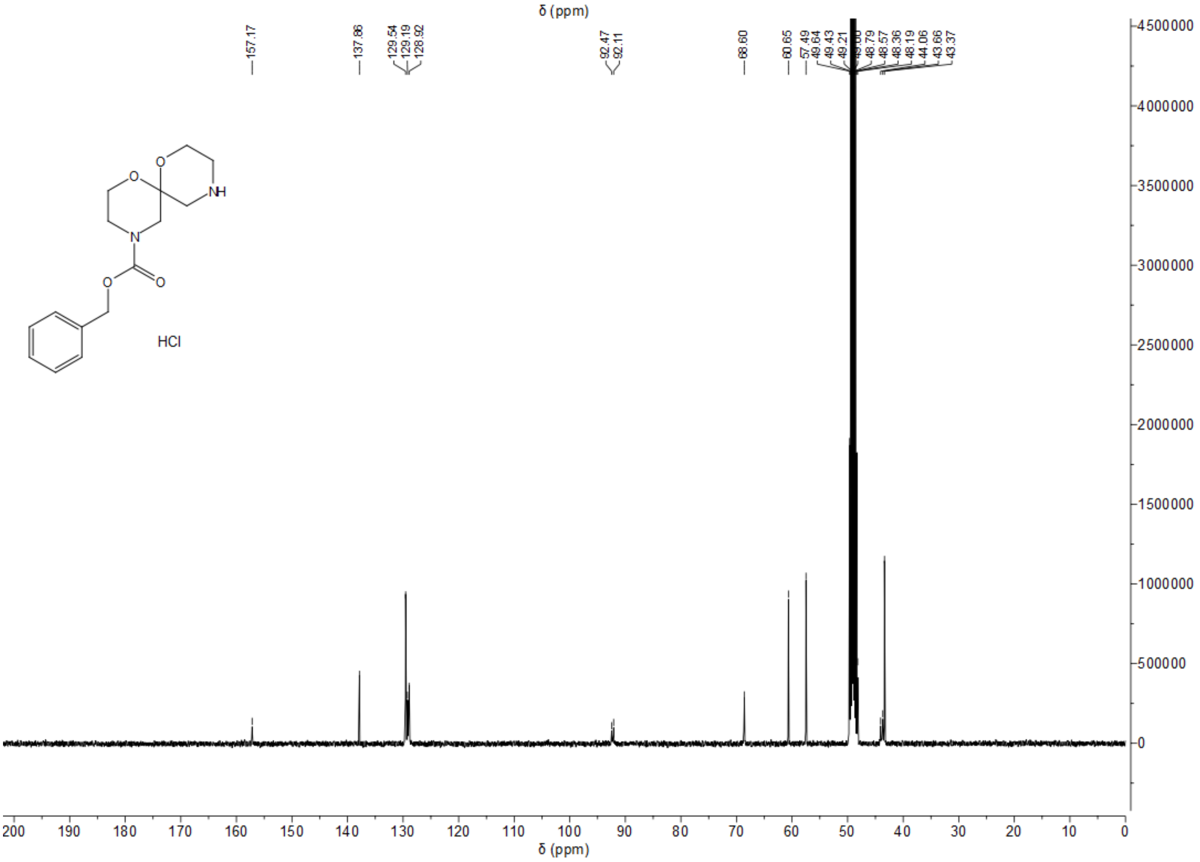

***tert*-butyl 4-benzyl-1,7-dioxa-4,11-diazaspiro[5.6]dodecane-11-carboxylate (19) [^{1}H-NMR data: 400 MHz, $CDCl_3$; $^{13}C\{^{1}H\}$-NMR data: 101 MHz, $CDCl_3$; 2D NMR spectra: HSQC, HMBC, COSY, all in $CDCl_3$]:**

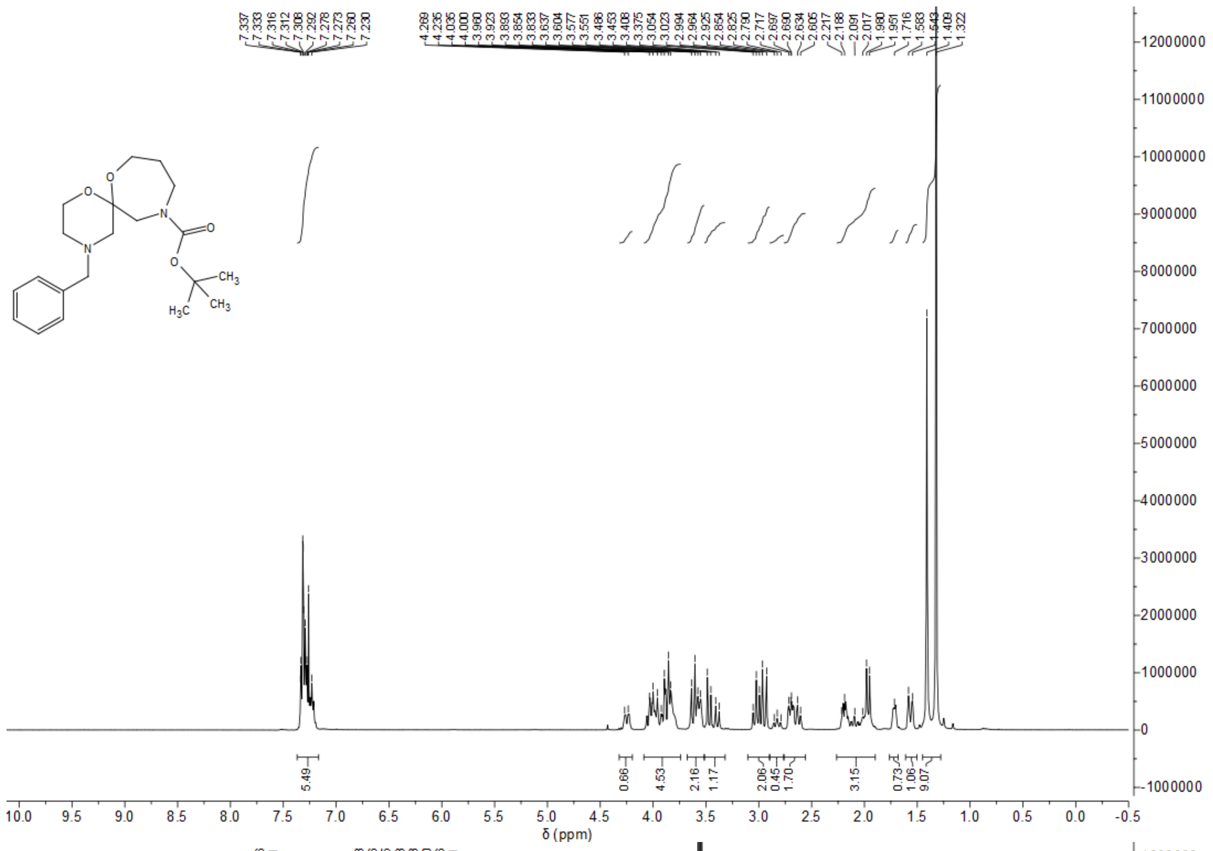

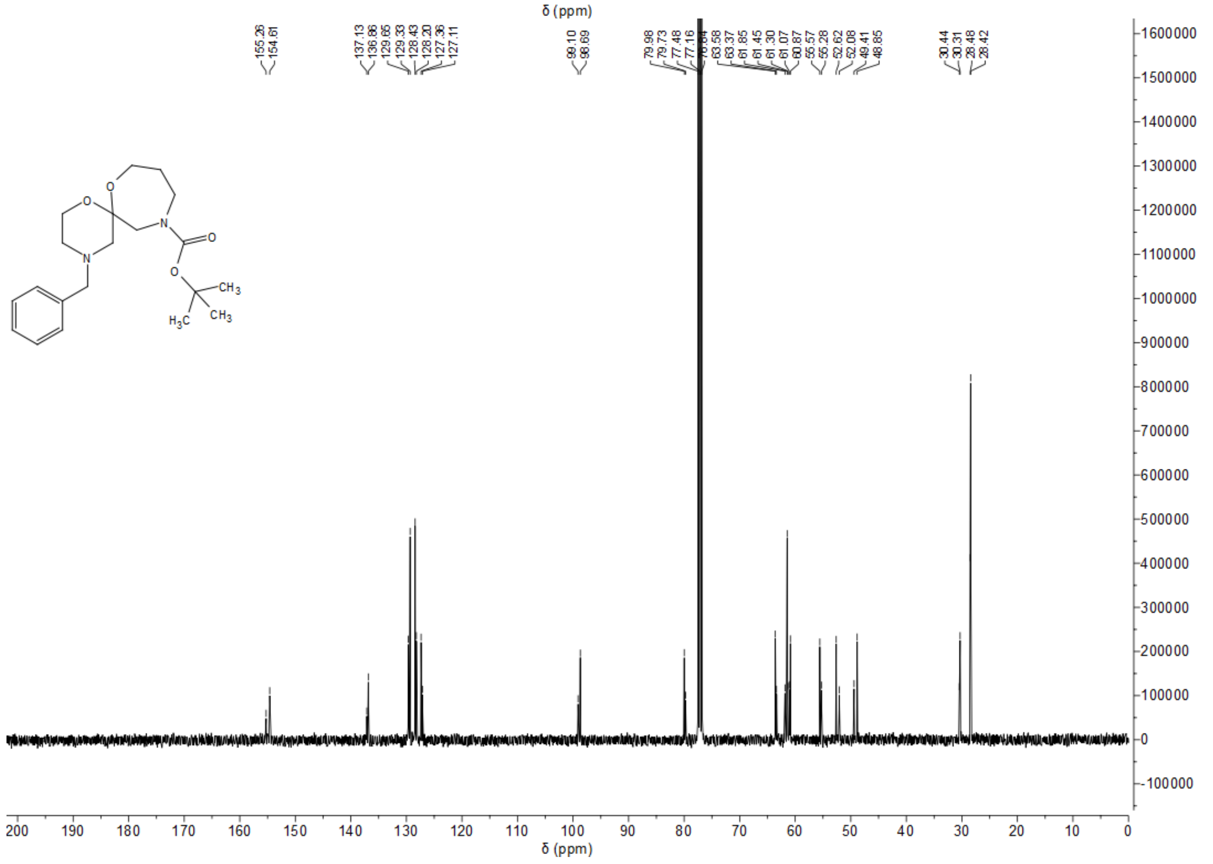

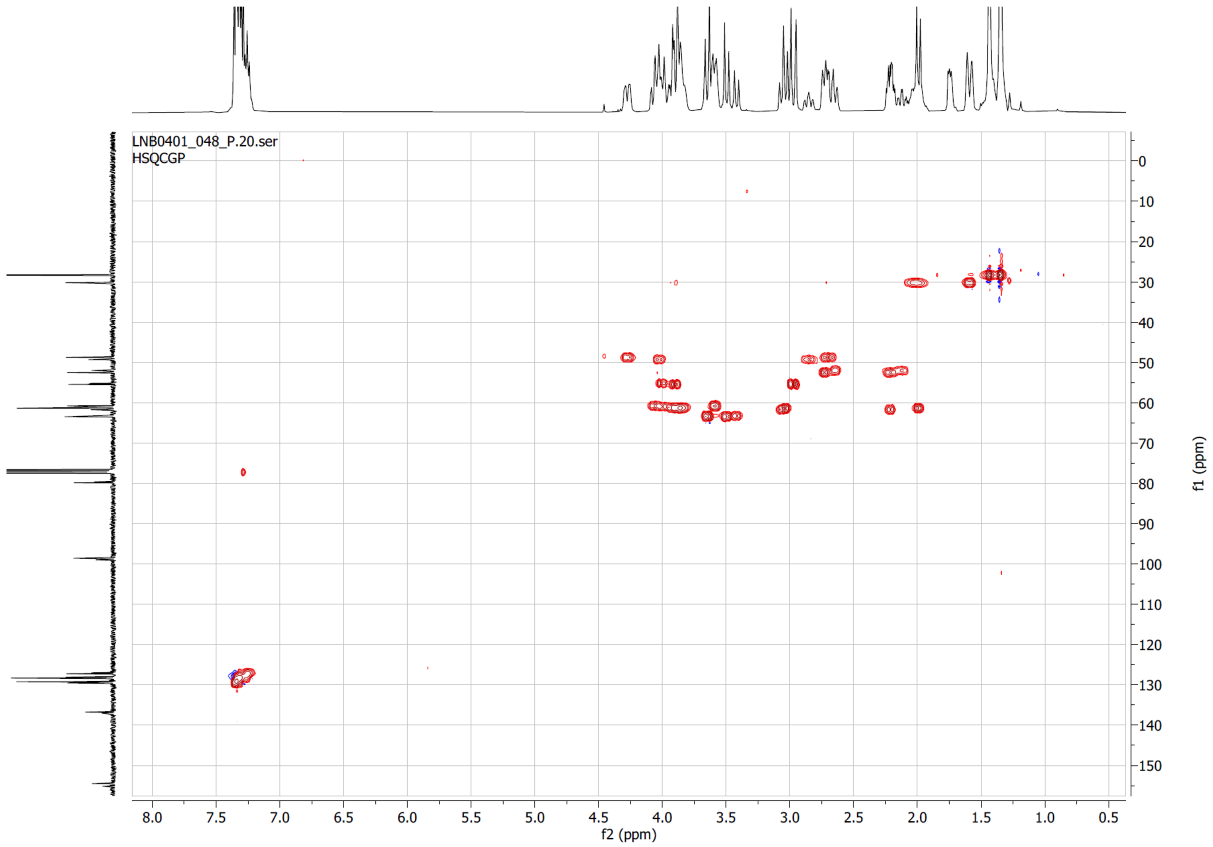
LNB0401_048_P.20.ser
HSQCGP
f2 (ppm)
f1 (ppm)

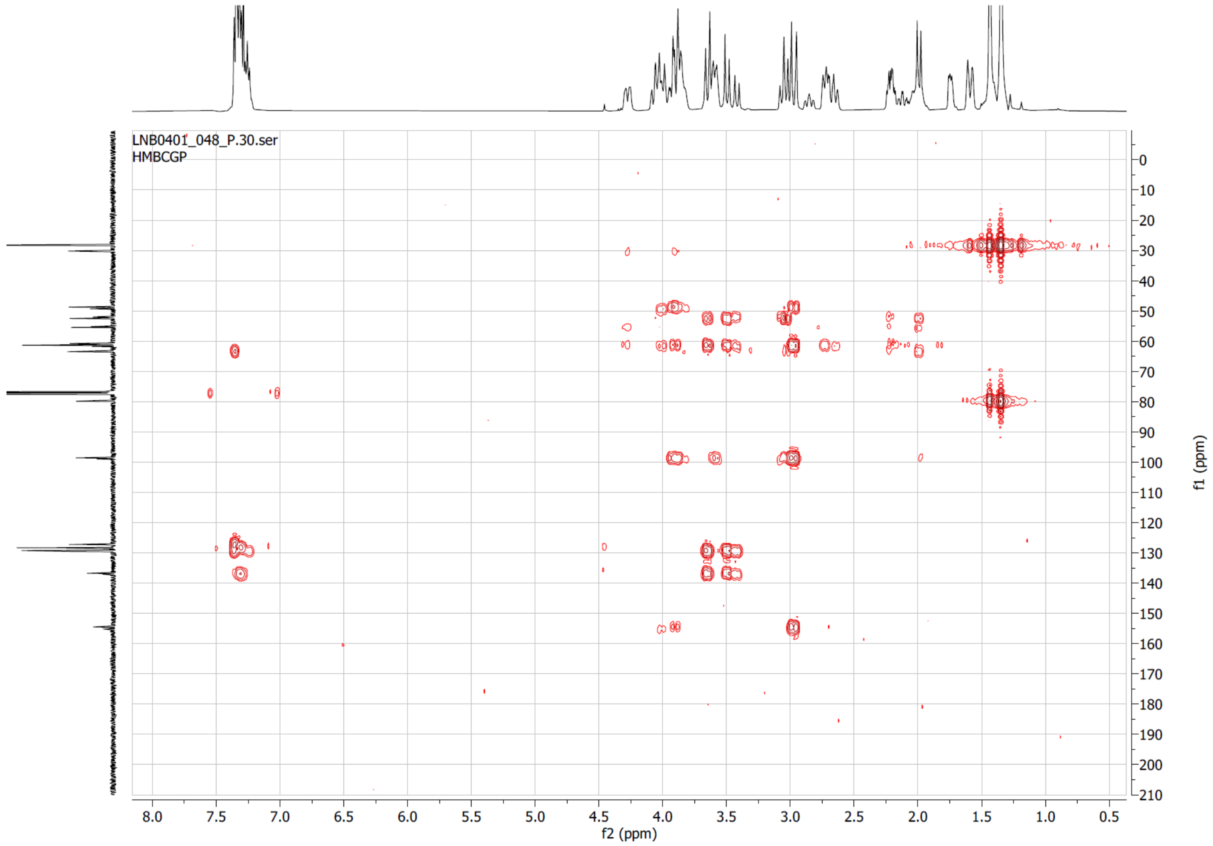
LNB0401_048_P.30.ser
HMBCGP
f2 (ppm)
f1 (ppm)

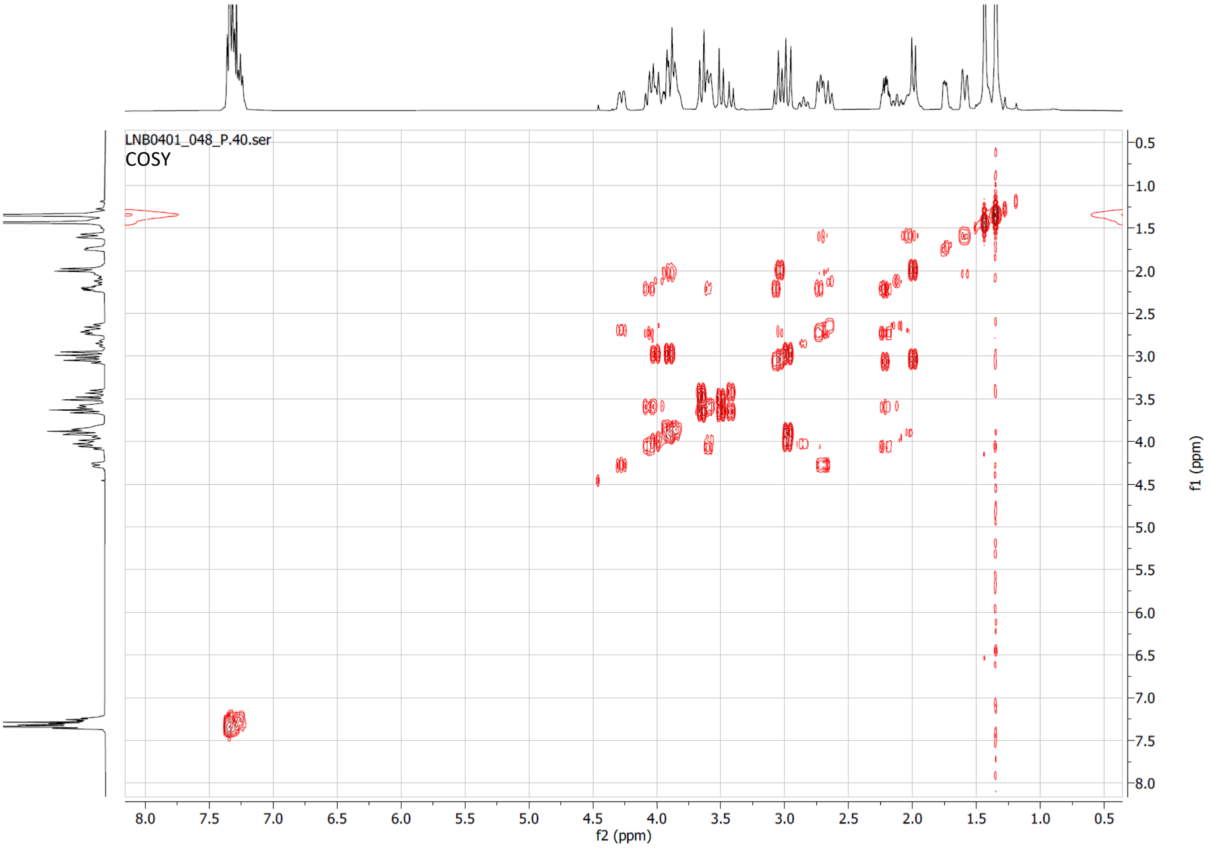
LNB0401_048_P.40.ser
COSY
f2 (ppm)
f1 (ppm)
8.0 7.5 7.0 6.5 6.0 5.5 5.0 4.5 4.0 3.5 3.0 2.5 2.0 1.5 1.0 0.5
0.5 1.0 1.5 2.0 2.5 3.0 3.5 4.0 4.5 5.0 5.5 6.0 6.5 7.0 7.5 8.0

4-benzyl 11-(*tert*-butyl) 1,7-dioxa-4,11-diazaspiro[5.6]dodecane-4,11-dicarboxylate (20) [^{1}H-NMR data: 400 MHz, $CDCl_3$; $^{13}C\{^1H\}$-NMR data: 101 MHz, $CDCl_3$]:

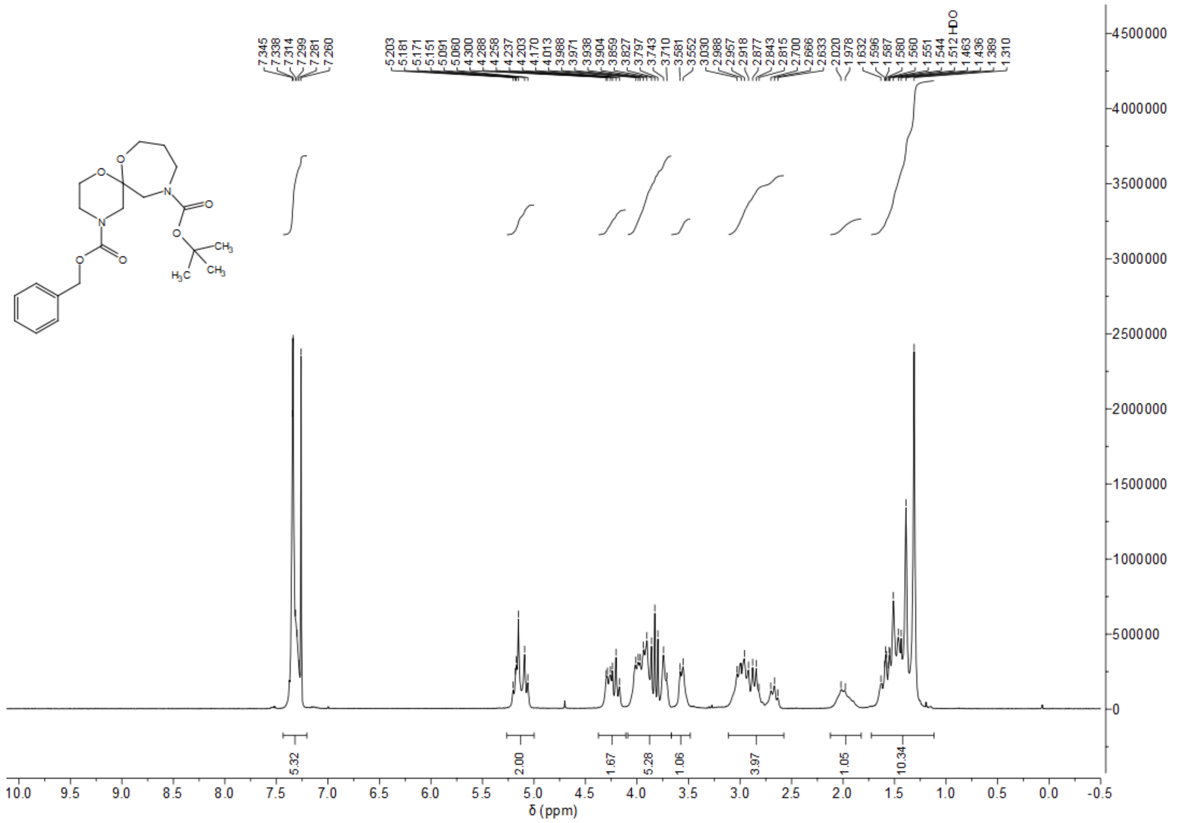

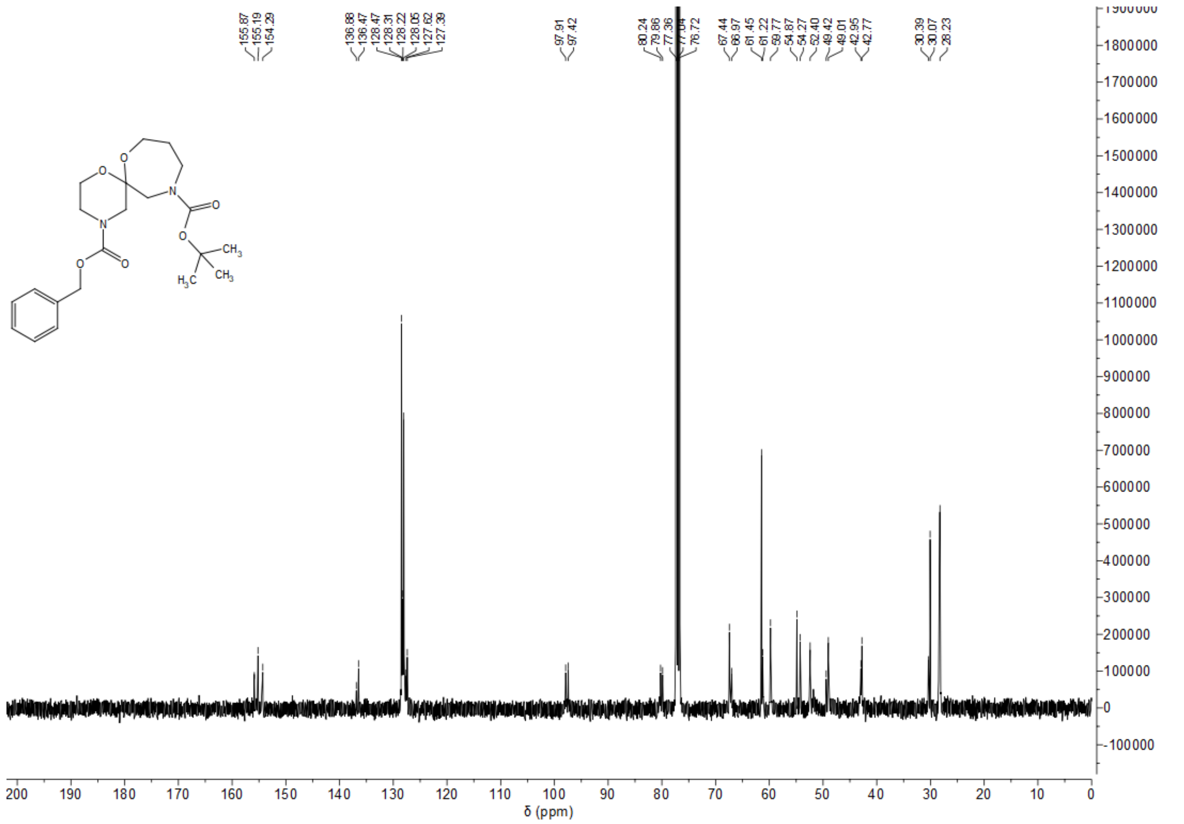

***tert*-butyl 1,7-dioxa-4,11-diazaspiro[5.6]dodecane-11-carboxylate (25)** **[^{1}H-NMR data: 400 MHz, CD_3OD; $^{13}C\{^1H\}$-NMR data: 101 MHz, CD_3OD; 2D NMR spectra: HSQC, HMBC, COSY, all in CD_3OD]:**

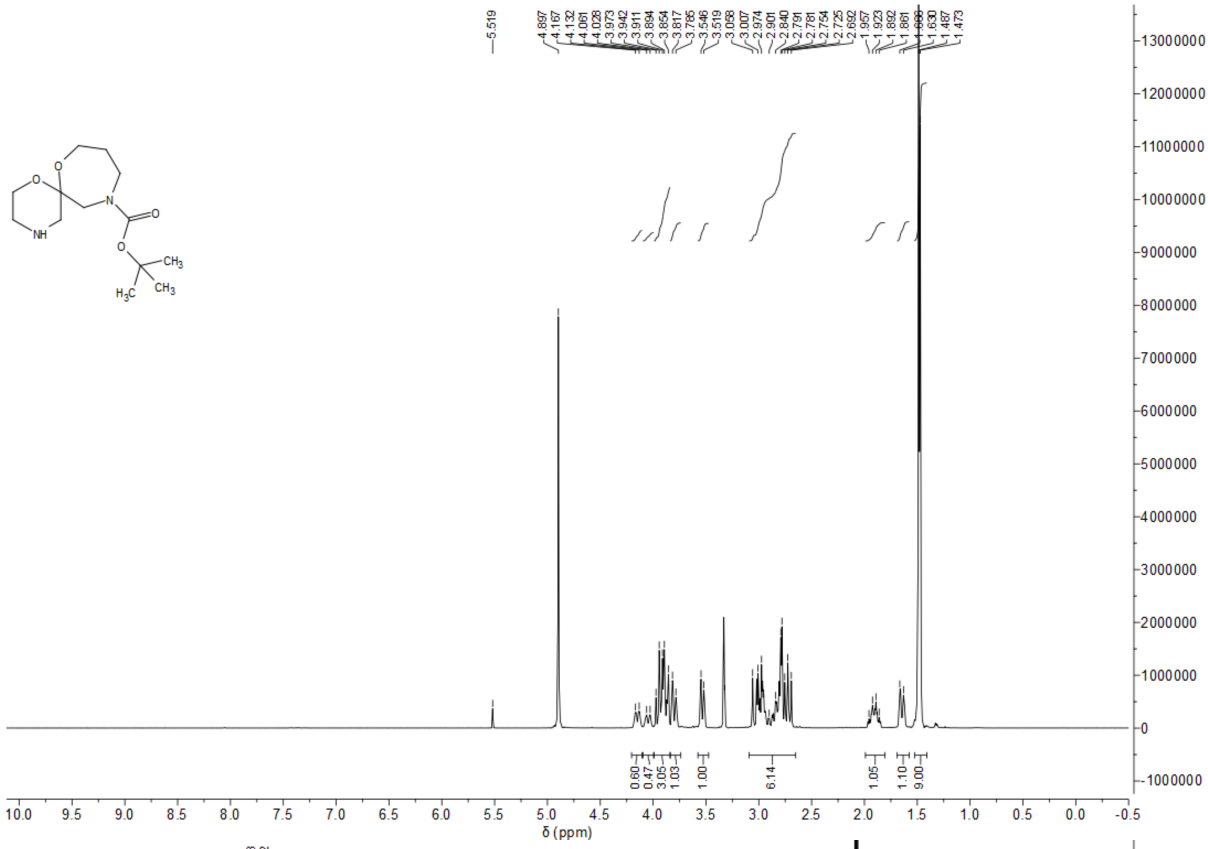

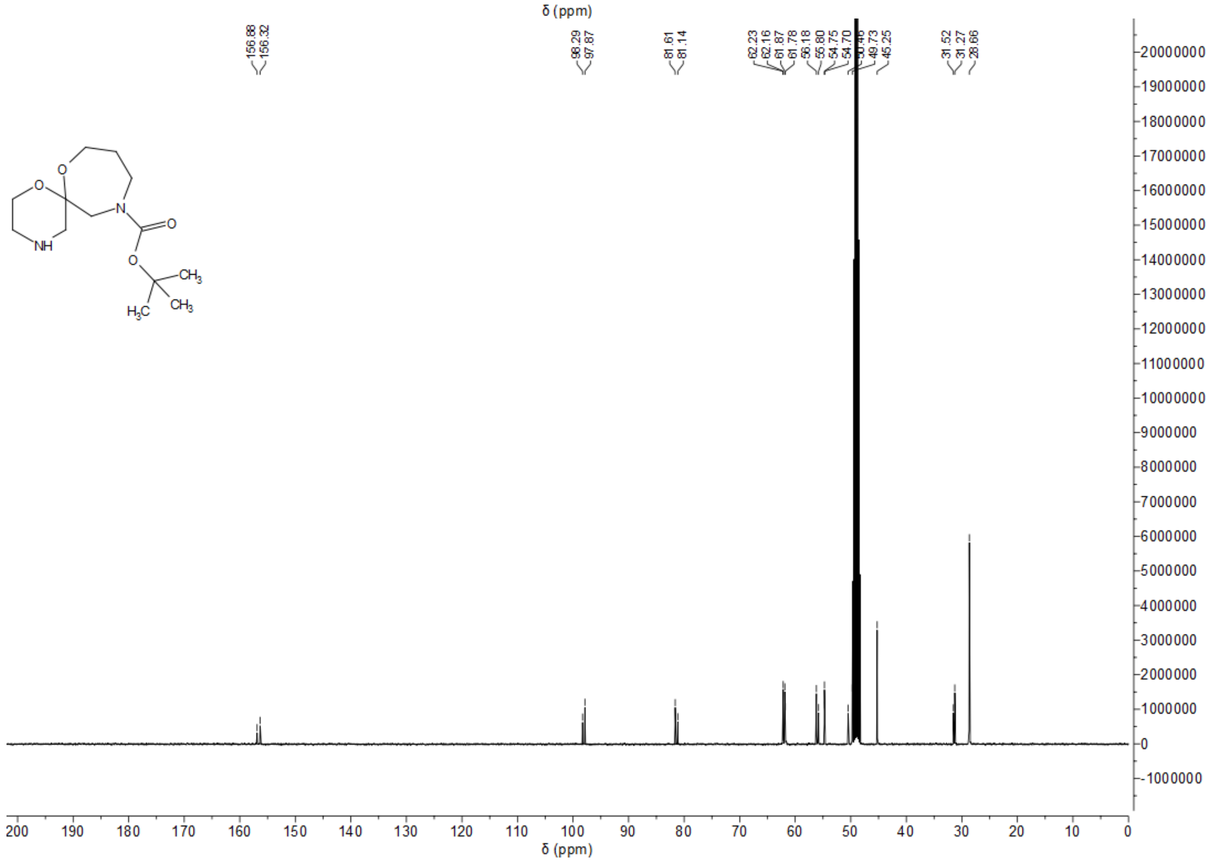

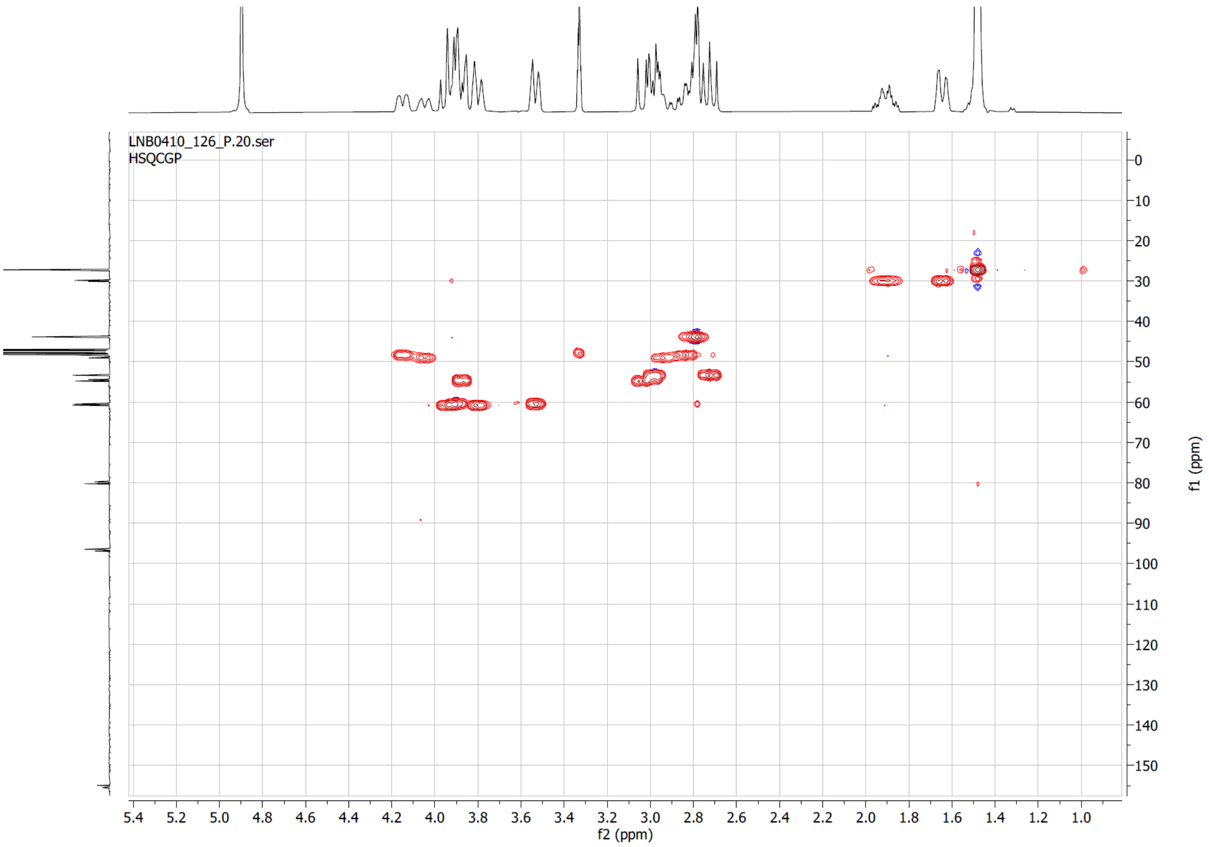
LNB0410_126_P.20.ser
HSQCGP
f2 (ppm)
f1 (ppm)

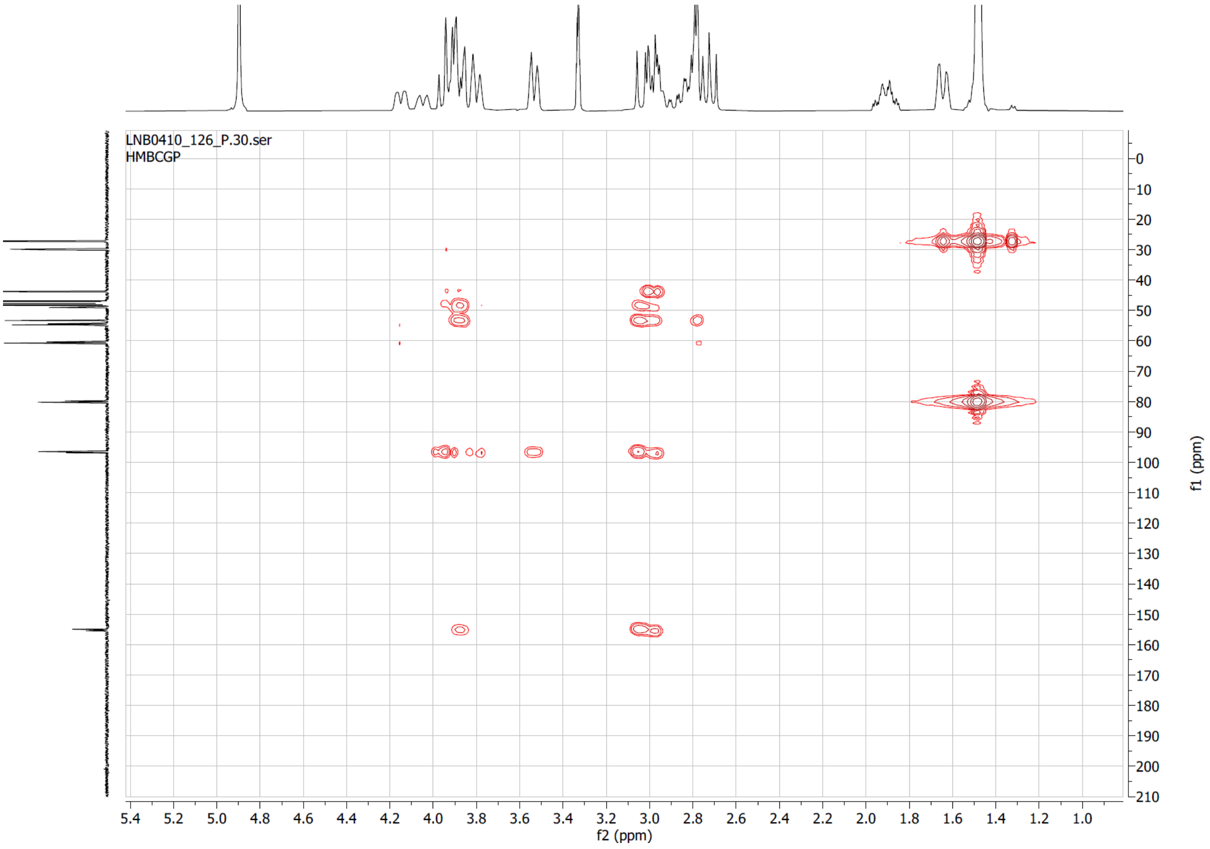
LNB0410_126_P.30.ser
HMBCGP
f2 (ppm)
f1 (ppm)

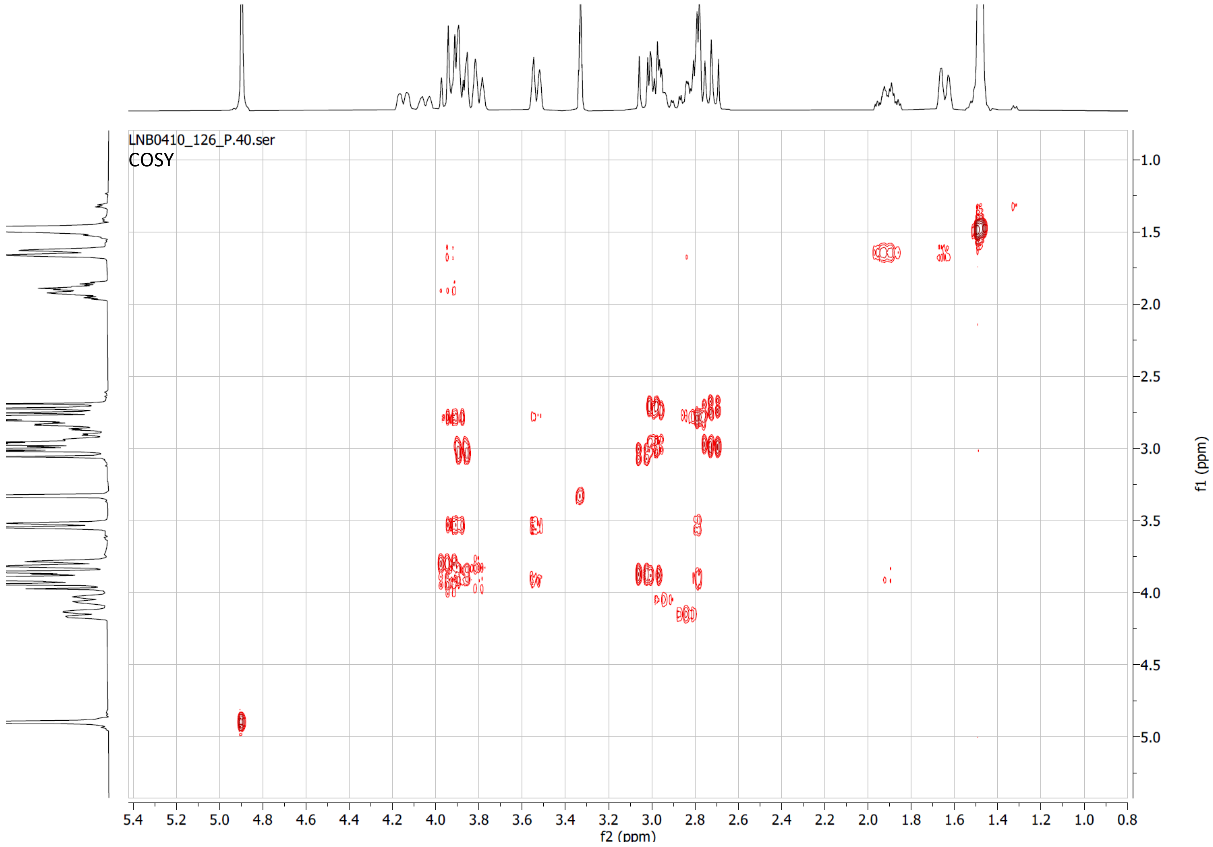
LNB0410_126_P.40.ser
COSY
f2 (ppm)
f1 (ppm)

***tert*-butyl 11-benzyl-1,7-dioxa-4,11-diazaspiro[5.6]dodecane-4-carboxylate (22) [^{1}H-NMR data: 400 MHz, CDCl$_3$; ^{13}C{^{1}H}-NMR data: 101 MHz, CDCl$_3$]:**

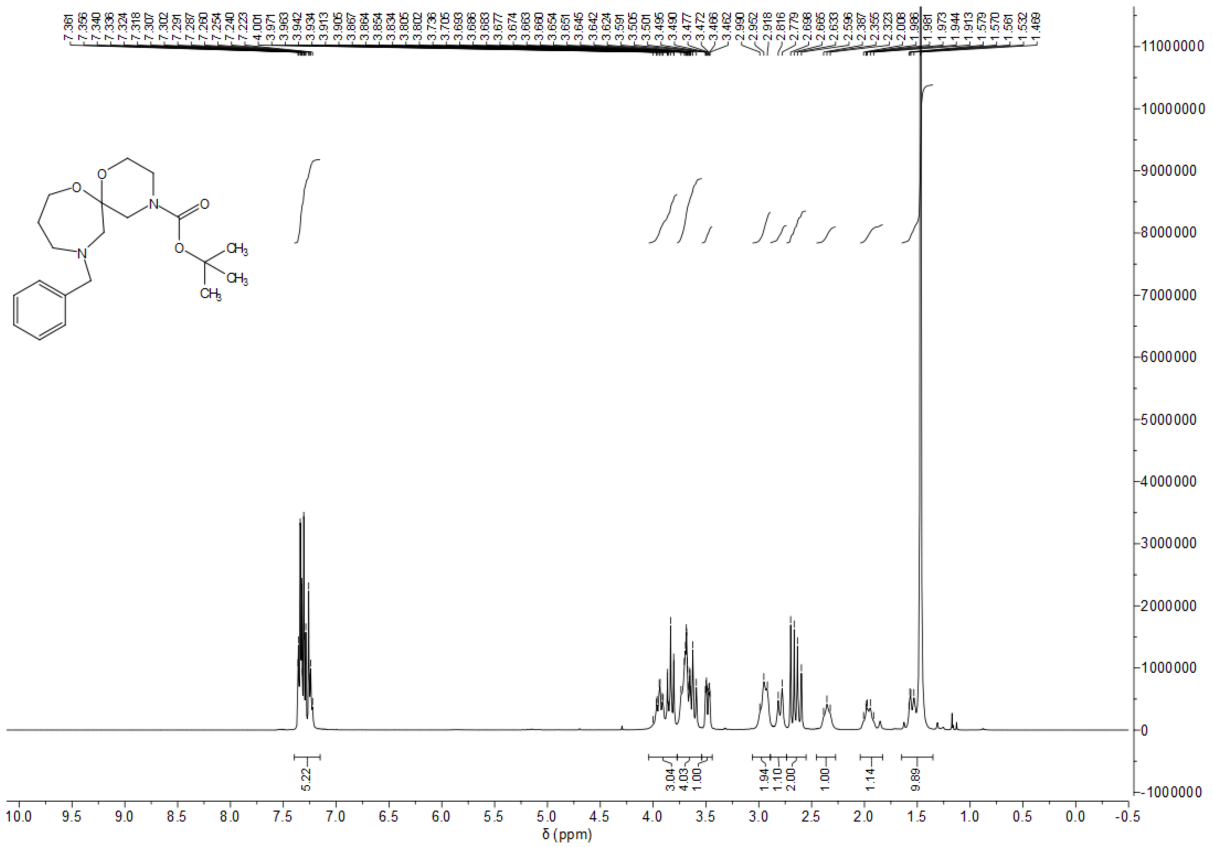

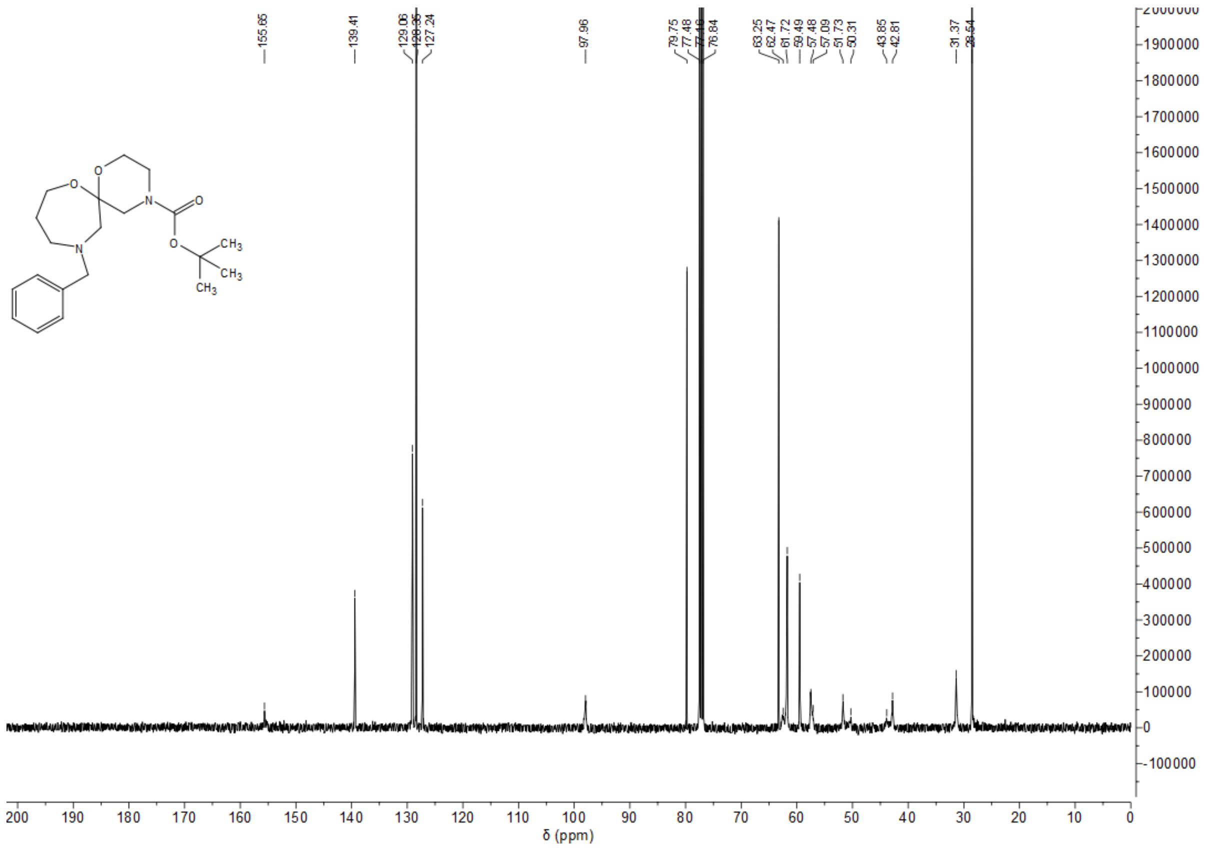

11-benzyl 4-(*tert*-butyl) 1,7-dioxa-4,11-diazaspiro[5.6]dodecane-4,11-dicarboxylate (23) [^{1}H-NMR data: 400 MHz, $CDCl_3$; $^{13}C\{^{1}H\}$-NMR data: 101 MHz, $CDCl_3$]:

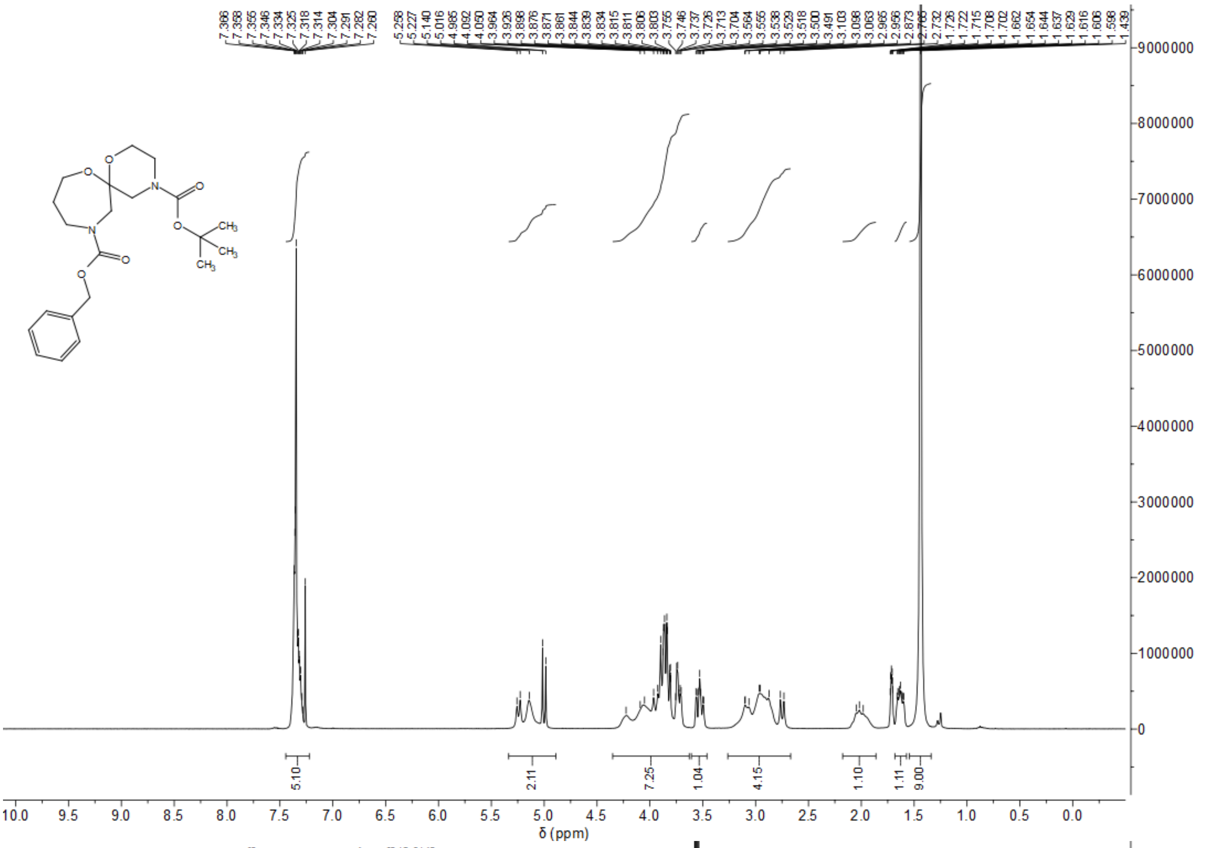

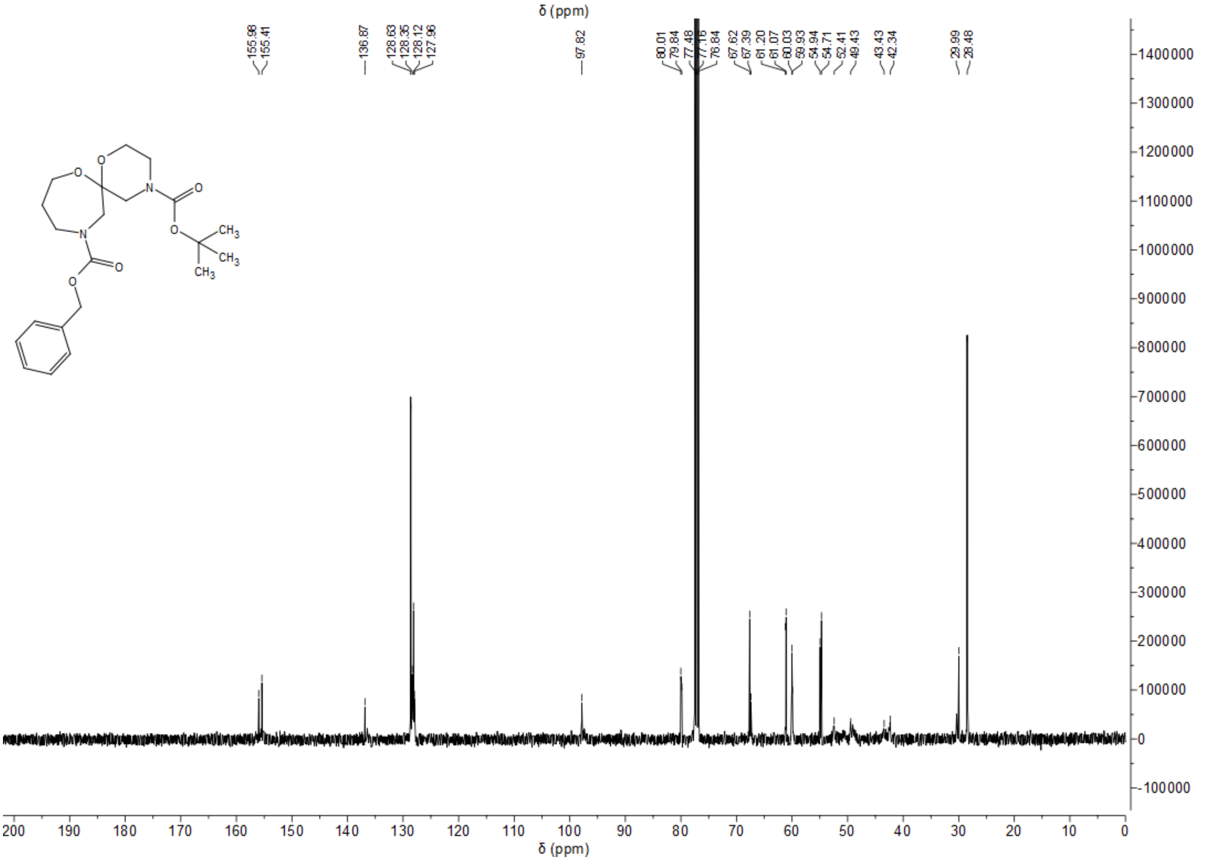

***tert*-butyl 12-benzyl-1,8-dioxa-5,12-diazaspiro[6.6]tridecane-5-carboxylate (24) [^{1}H-NMR data: 400 MHz, $CDCl_3$; ^{13}C{^{1}H}-NMR data: 101 MHz, $CDCl_3$]:**

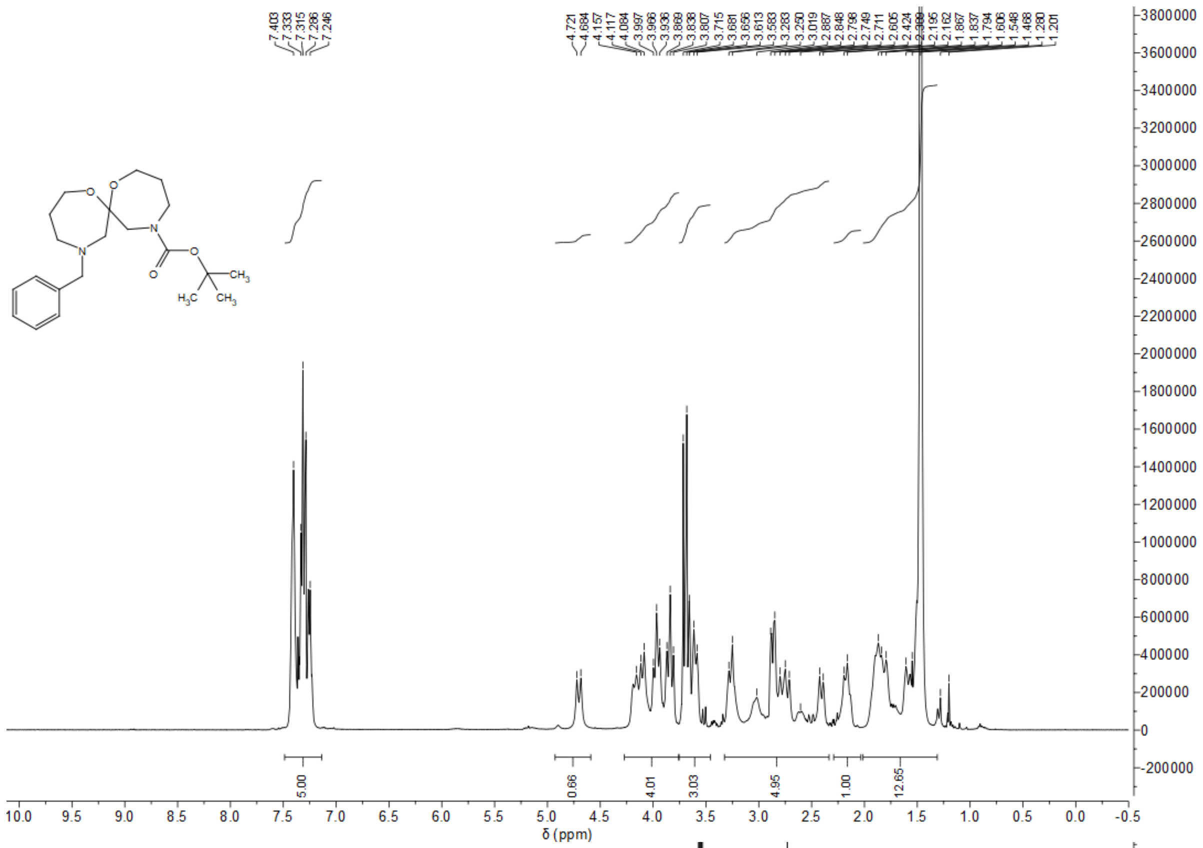

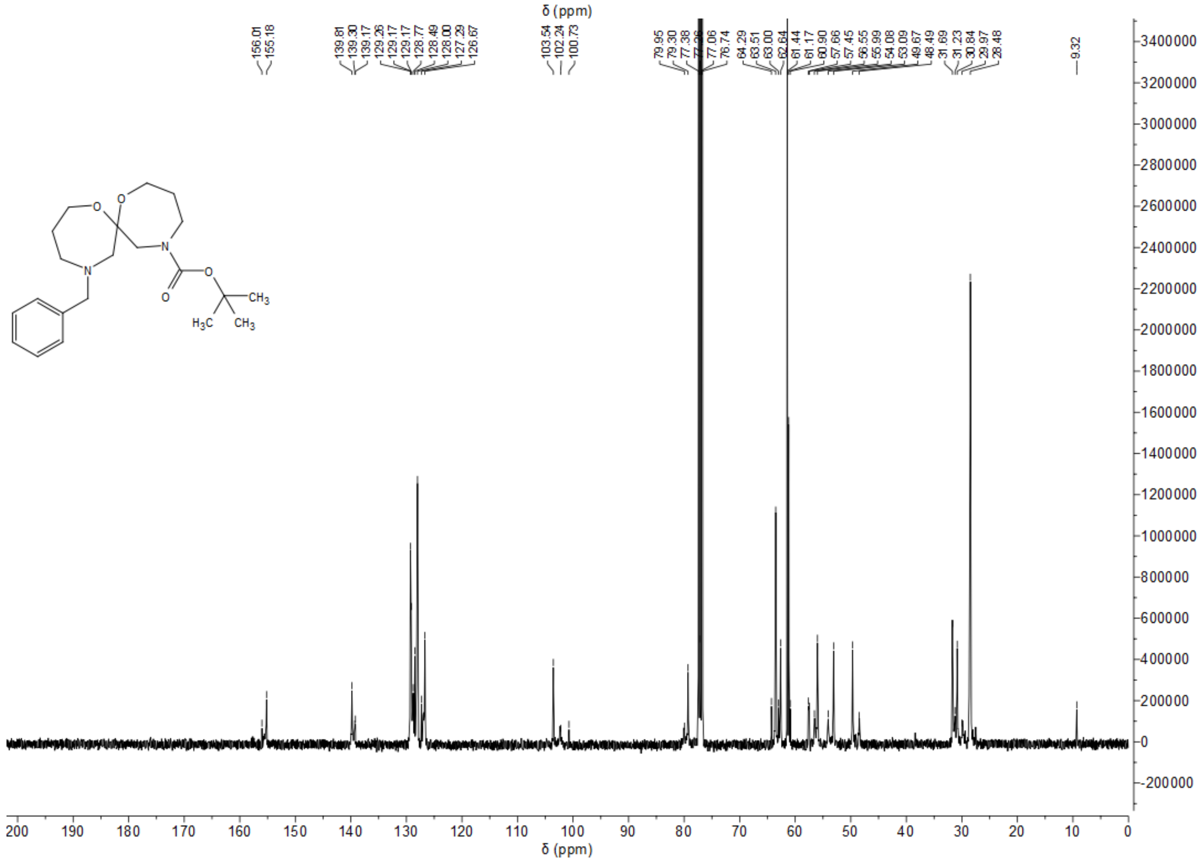

3-(3-((4-benzyl-2-(iodomethyl)-1,4-oxazepan-2-yl)oxy)propyl)-1,1-dimethylurea (S27) [^{1}H-NMR data: 400 MHz, $CDCl_3$; $^{13}C\{^1H\}$-NMR data: 101 MHz, $CDCl_3$]:

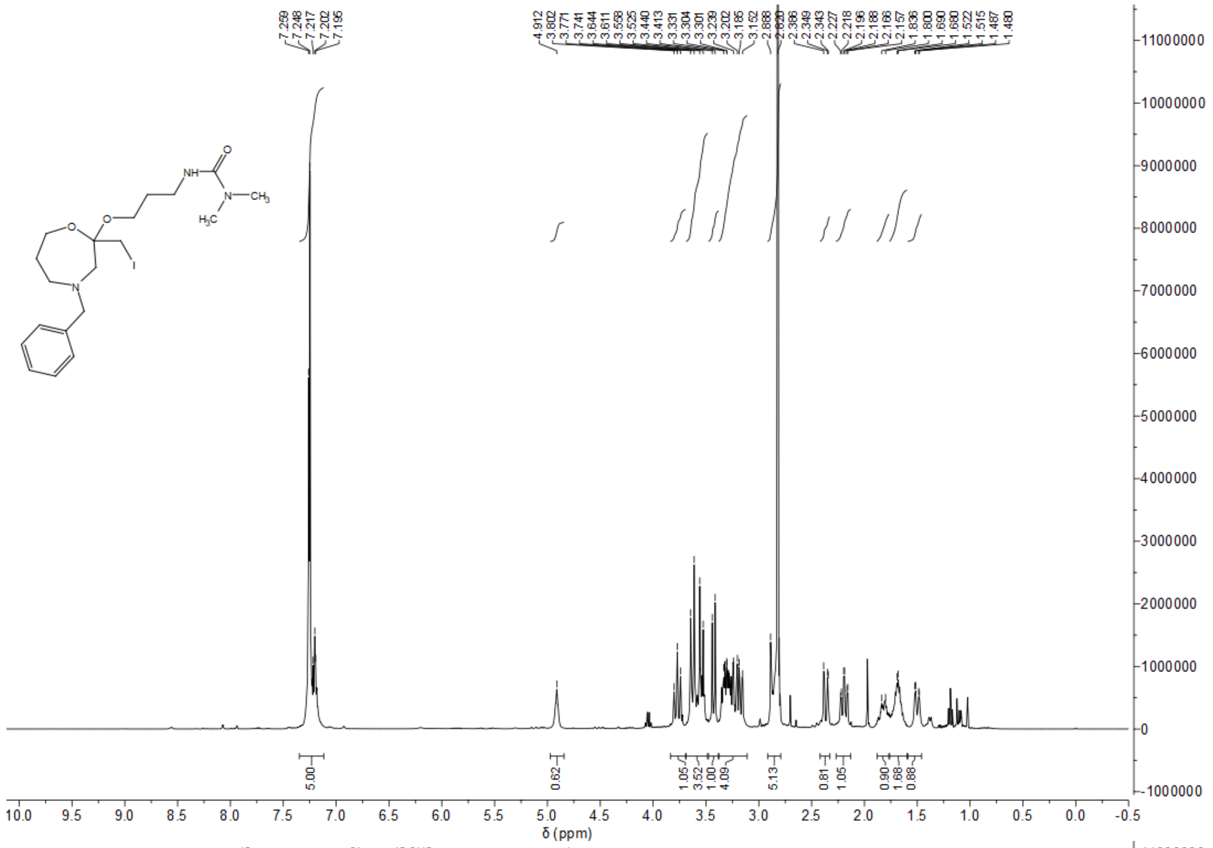

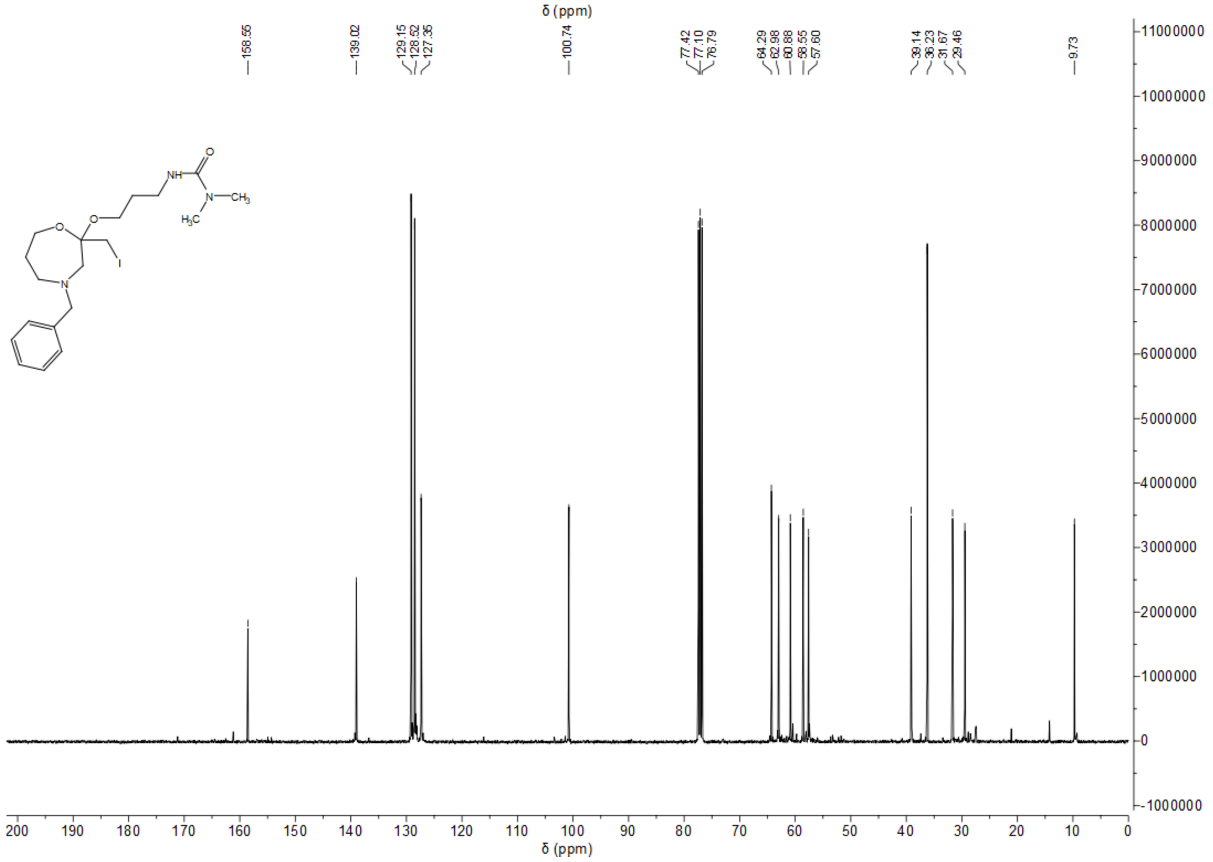

3-(4-((4-benzyl-2-(iodomethyl)morpholin-2-yl)oxy)butyl)-1,1-dimethylurea (S25) [^{1}H-NMR data: 400 MHz, $CDCl_3$]:

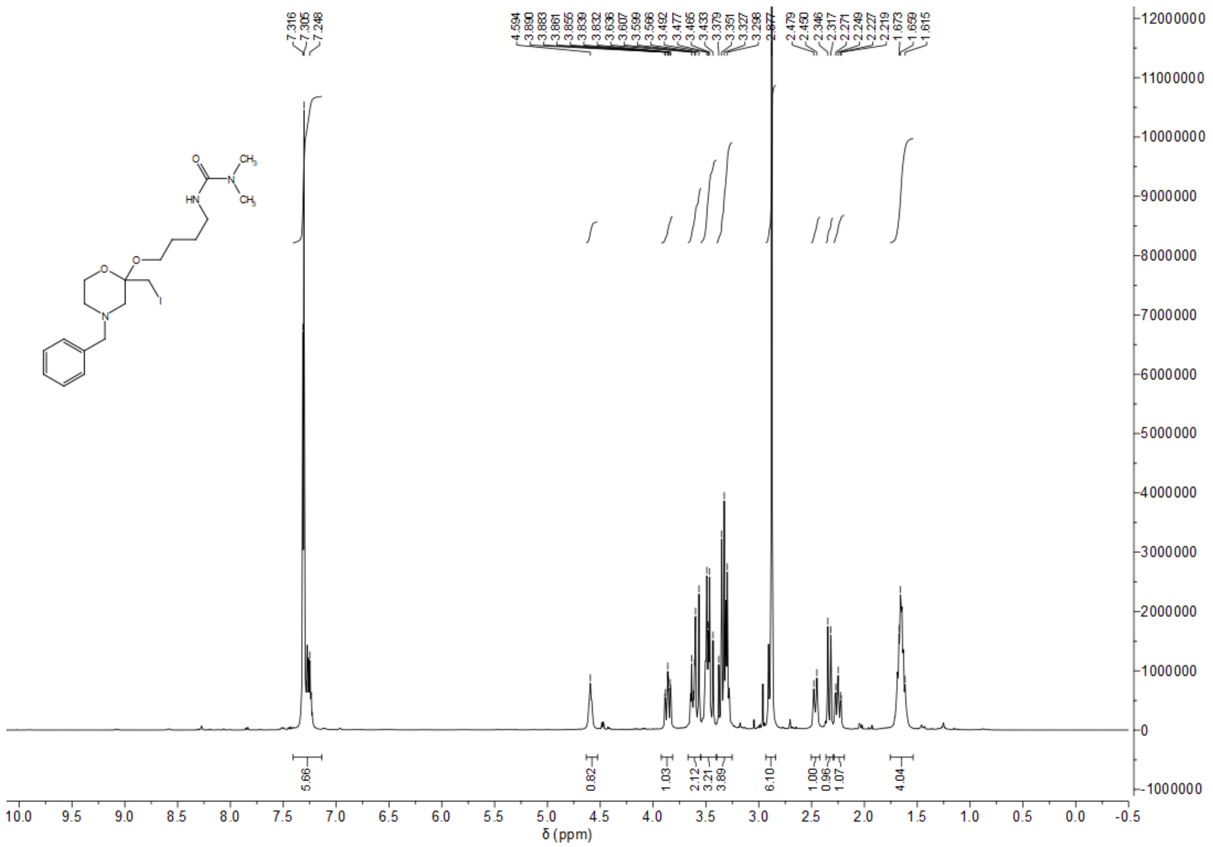

2-(2-((4-benzyl-2-(iodomethyl)morpholin-2-yl)oxy)ethoxy)ethan-1-amine (S26) [^{1}H-NMR data: 400 MHz, $CDCl_3$; $^{13}C\{^1H\}$-NMR data: 101 MHz, $CDCl_3$]:

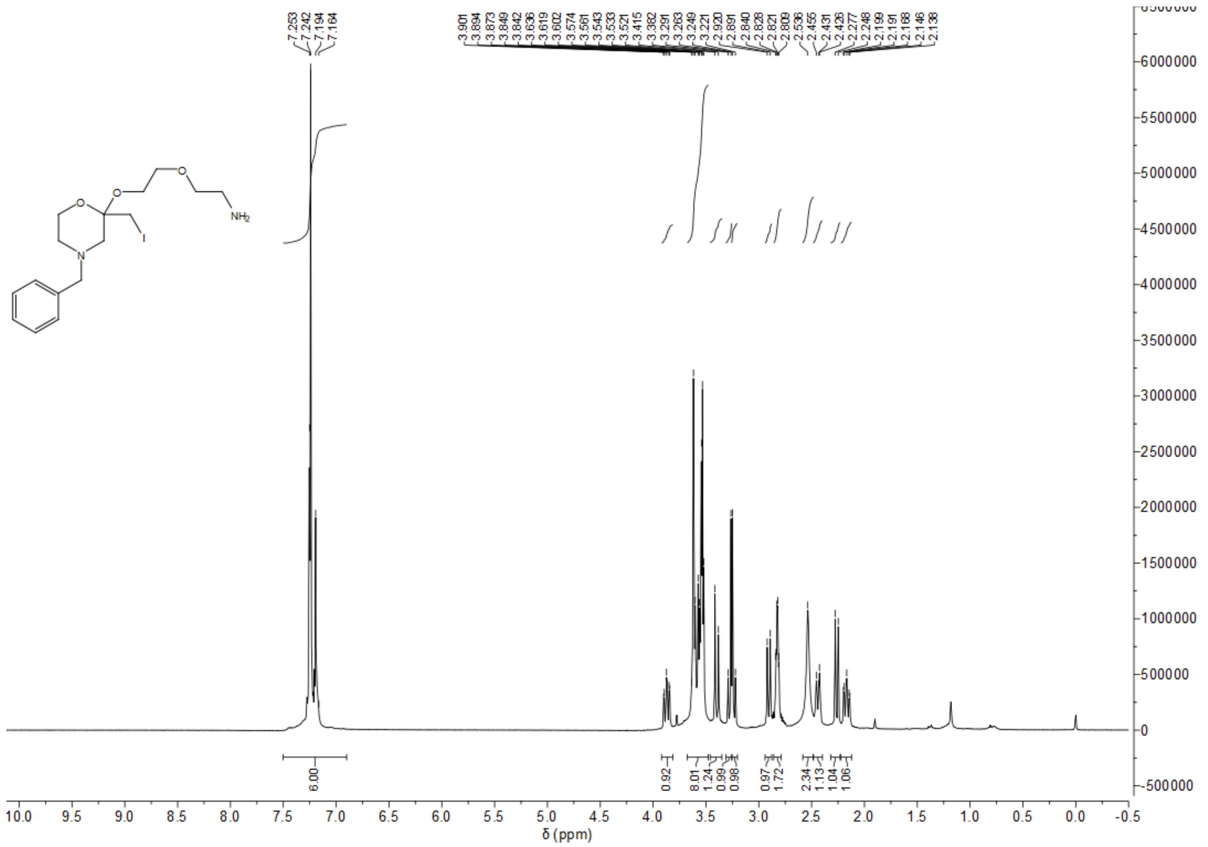

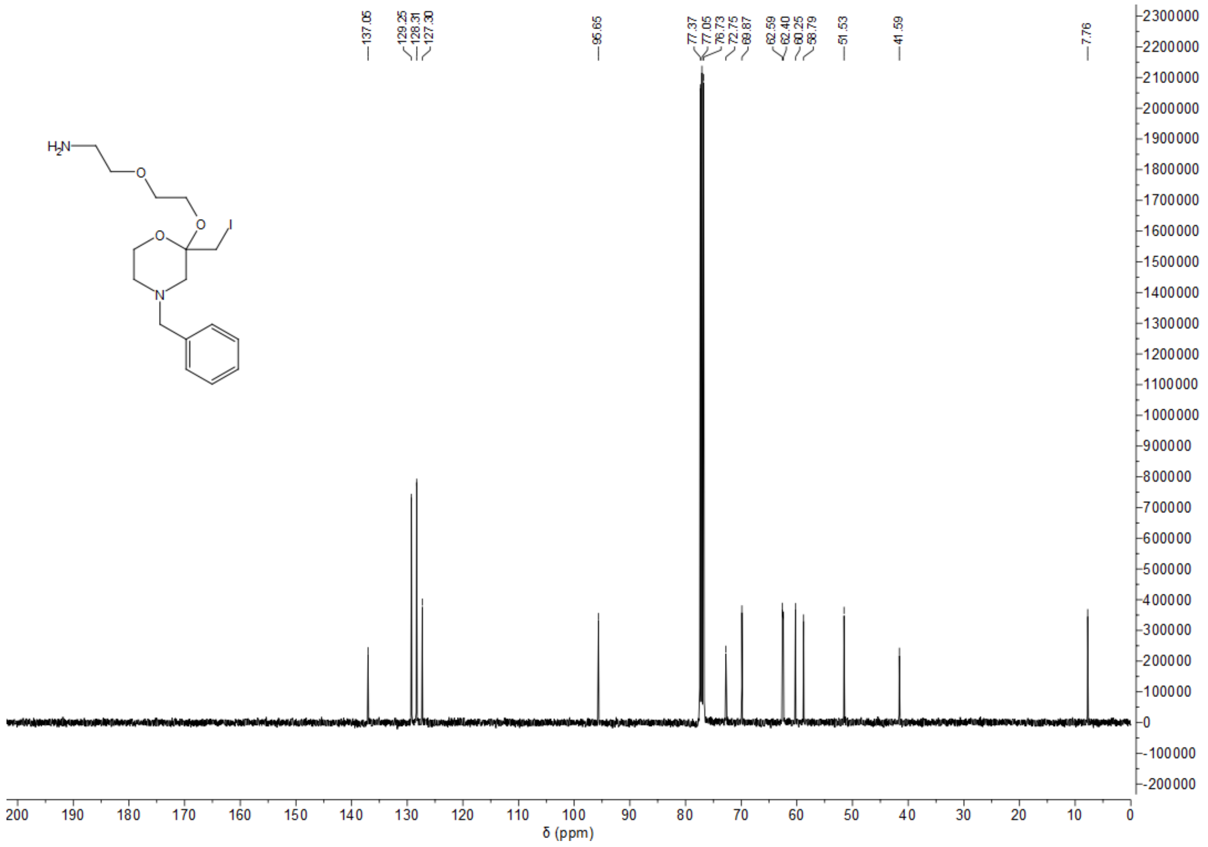

1.5.2 Substituted 6,6-spiroacetals

***tert*-butyl (3*S*)-10-benzyl-3-methyl-1,7-dioxa-4,10-diazaspiro[5.5]undecane-4-carboxylate (44) [^{1}H-NMR data: 400 MHz, $CDCl_3$; $^{13}C\{^1H\}$-NMR data: 101 MHz, $CDCl_3$; 2D NMR spectra: HSQC, HMBC, COSY, all in $CDCl_3$]:**

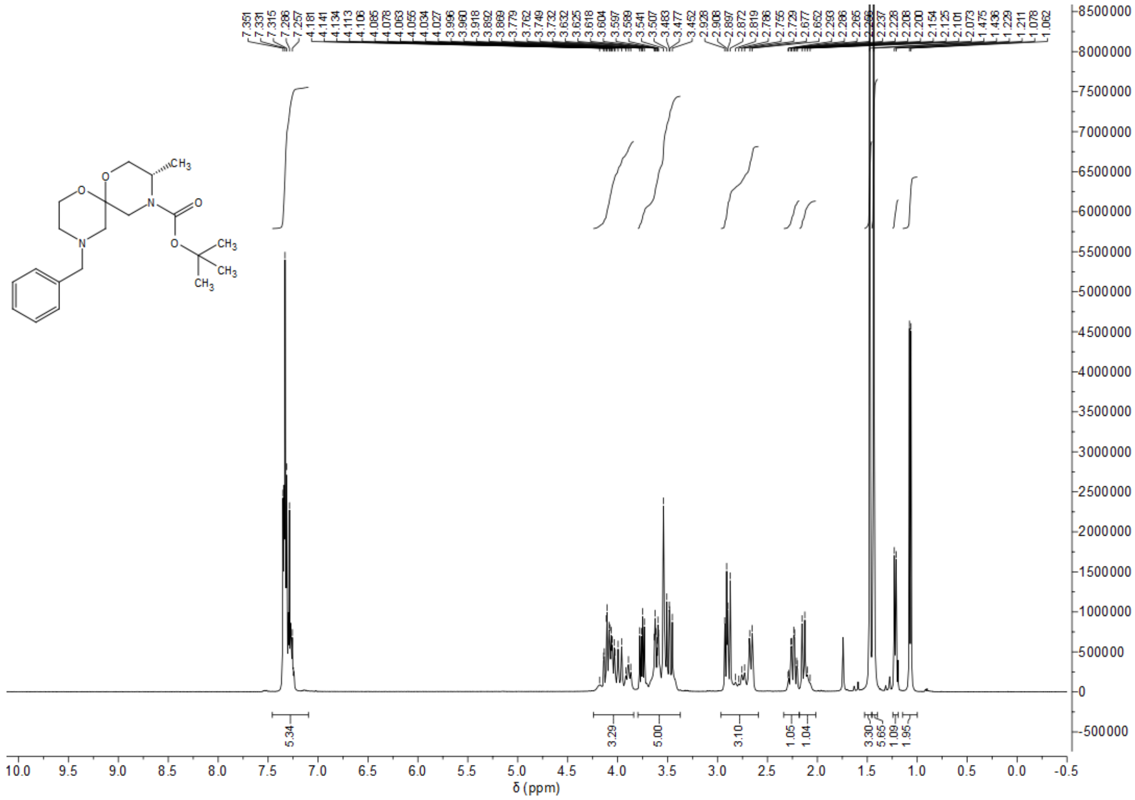

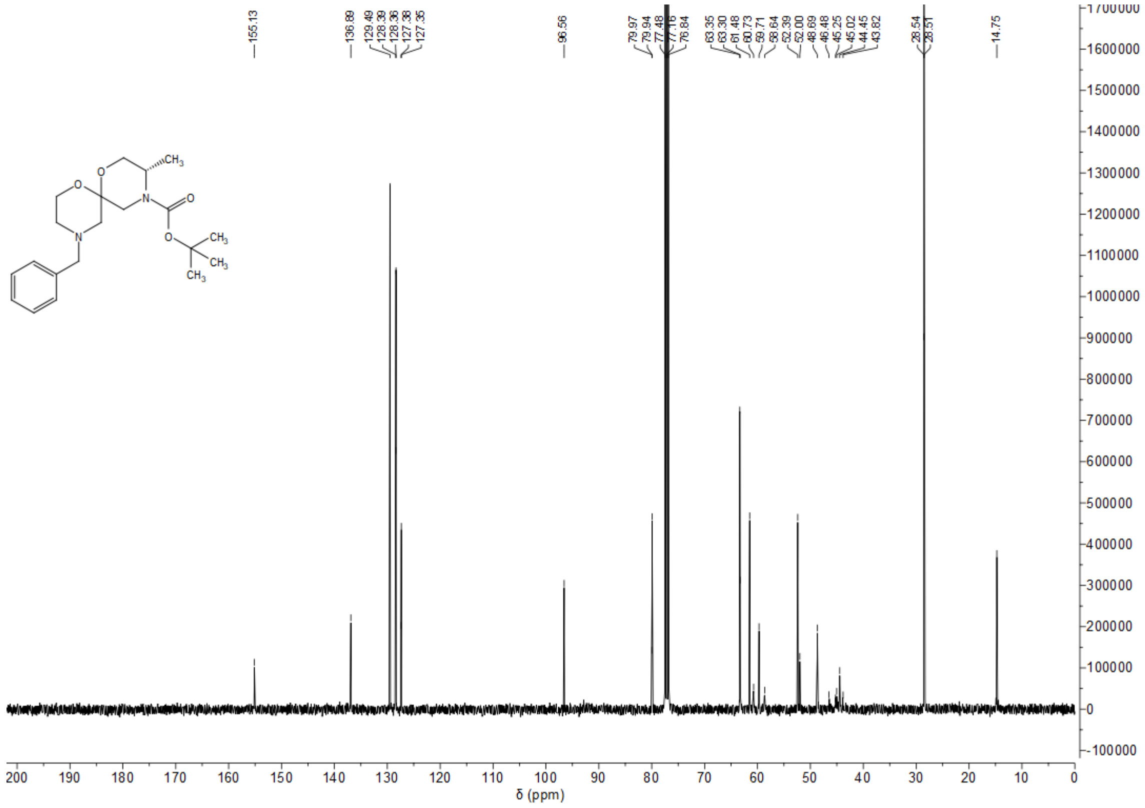

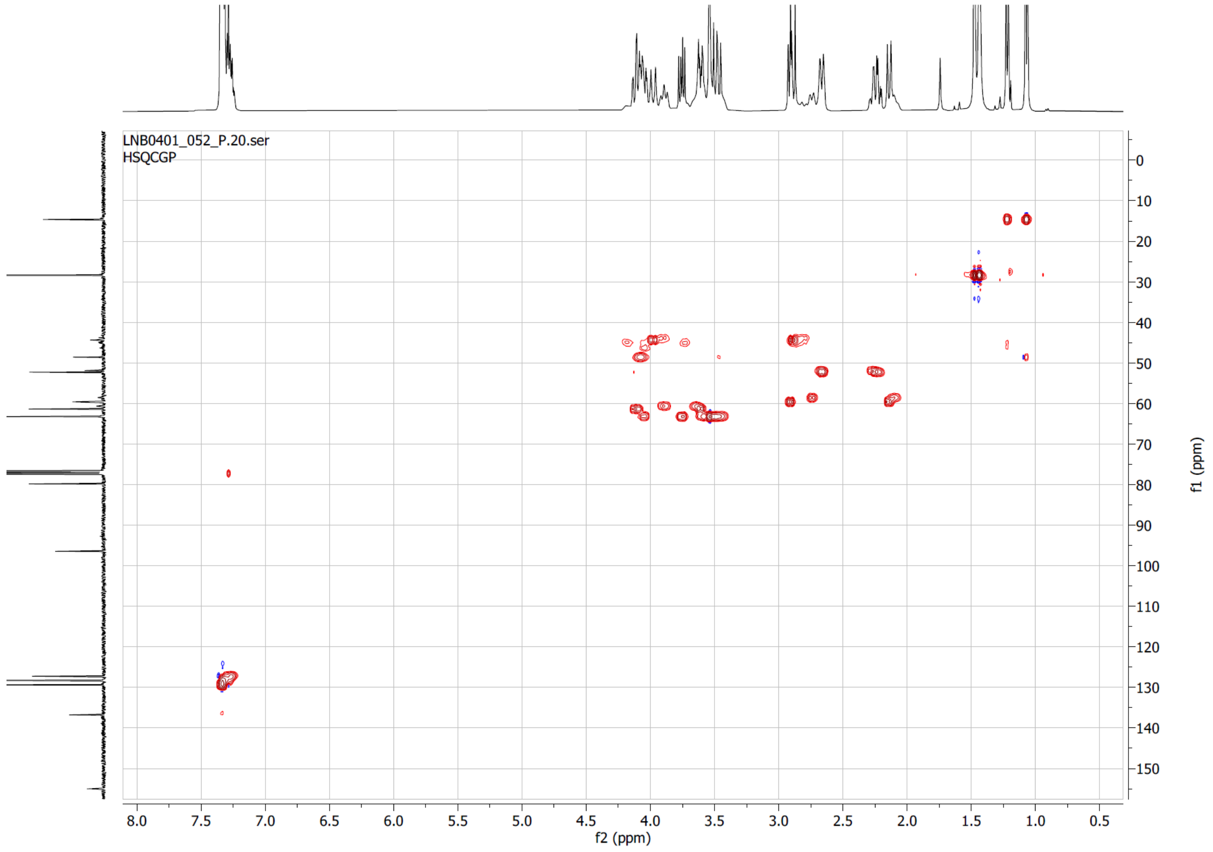
LNB0401_052_P.20.ser
HSQCGP
f2 (ppm)
f1 (ppm)

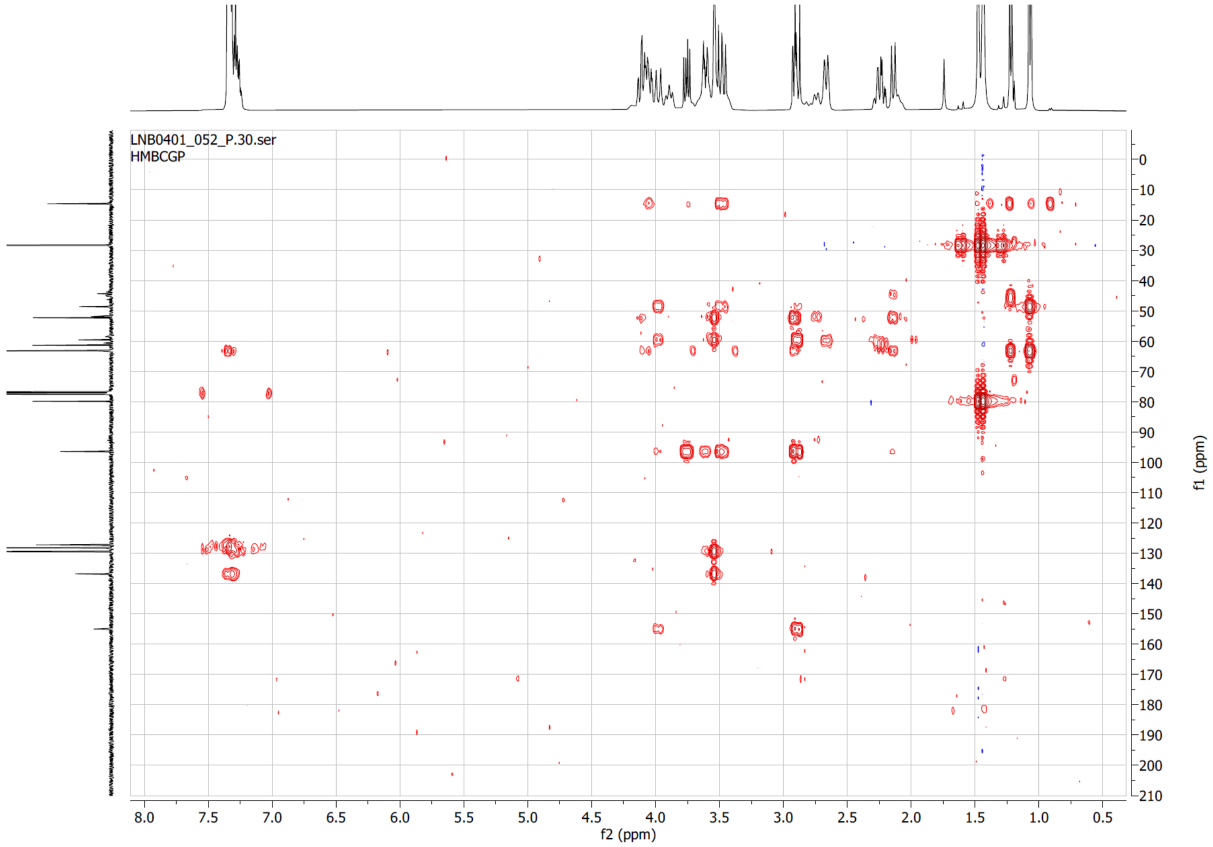
LNB0401_052_P.30.ser
HMBCGP
f2 (ppm)
f1 (ppm)

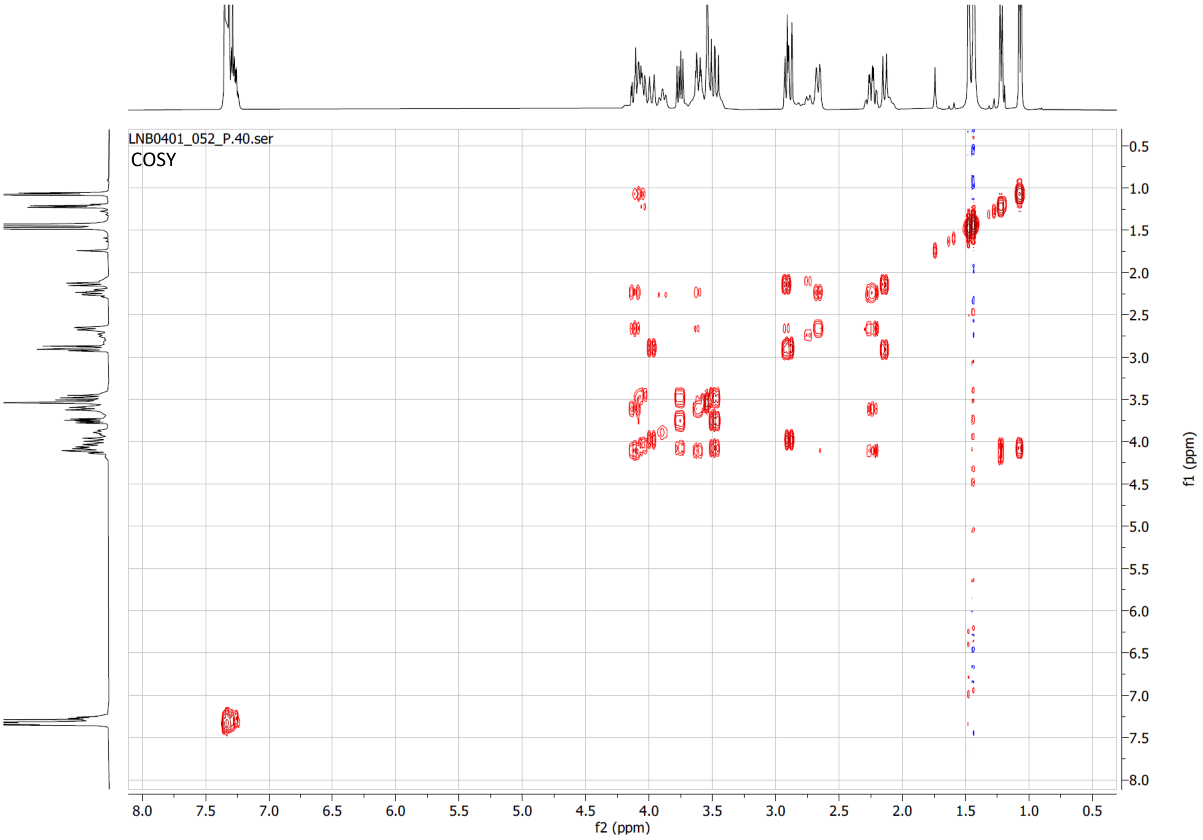

LNB0401_052_P.40.ser
COSY
f2 (ppm)
f1 (ppm)
8.0
7.5
7.0
6.5
6.0
5.5
5.0
4.5
4.0
3.5
3.0
2.5
2.0
1.5
1.0
0.5

***tert*-butyl (3*S*)-10-benzyl-3-isopropyl-1,7-dioxa-4,10-diazaspiro[5.5]undecane-4-carboxylate (45) [^{1}H-NMR data: 400 MHz, CDCl$_3$; ^{13}C{^{1}H}-NMR data: 101 MHz, CDCl$_3$; 2D NMR spectra: HSQC, HMBC, COSY, all in CDCl$_3$]:**

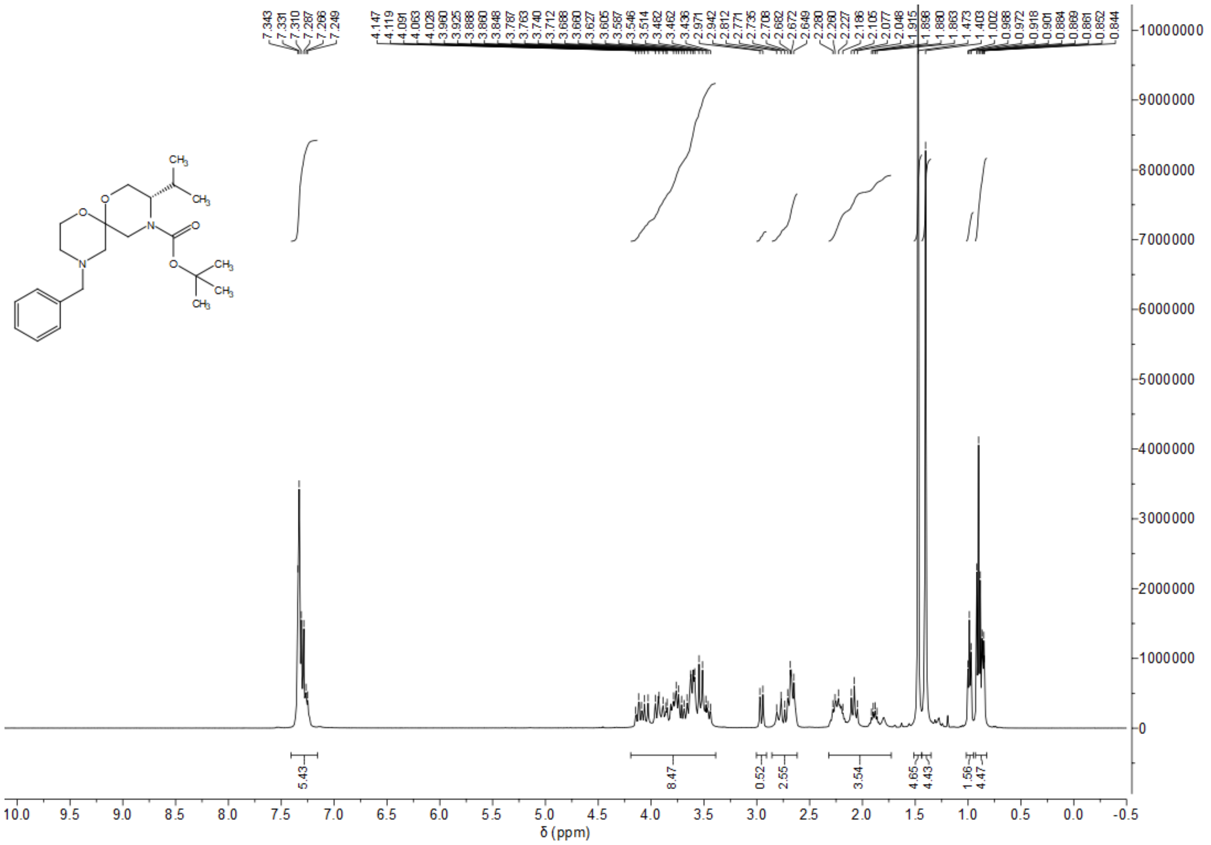

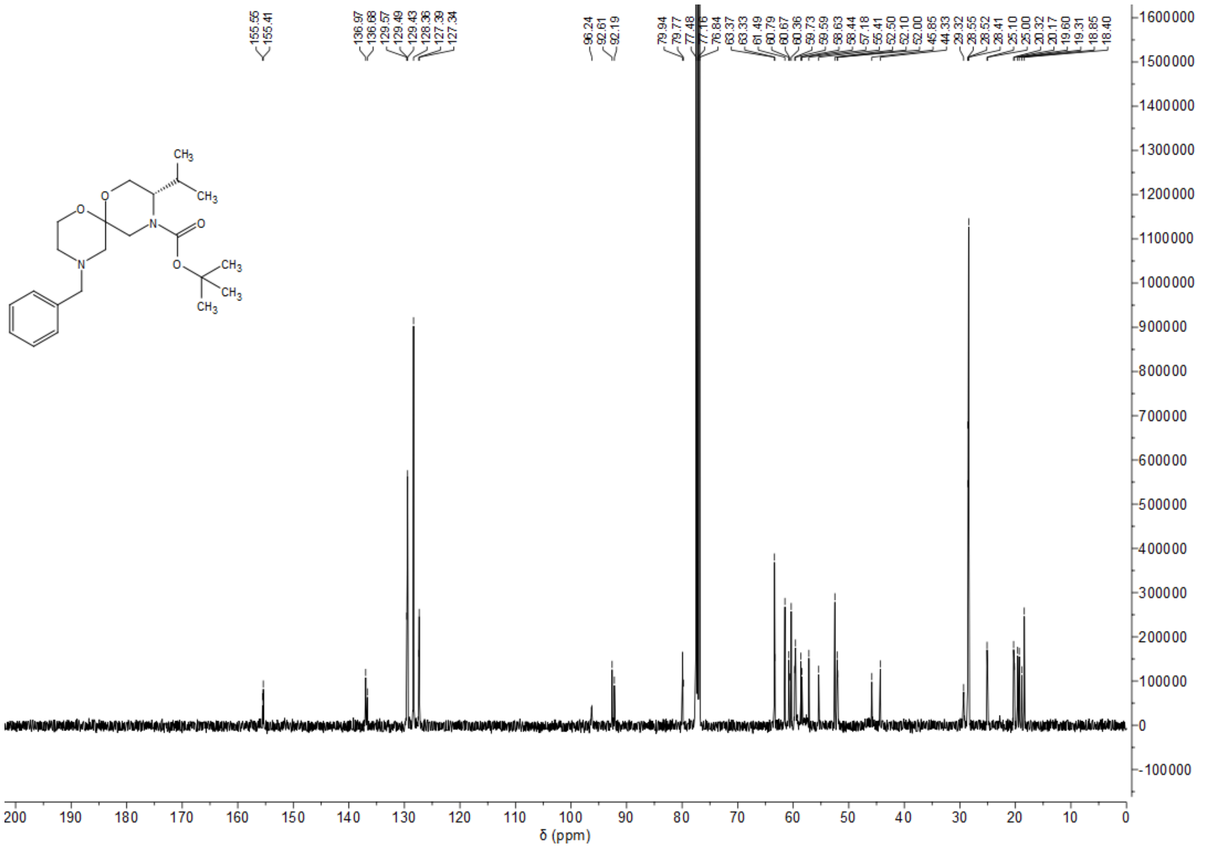

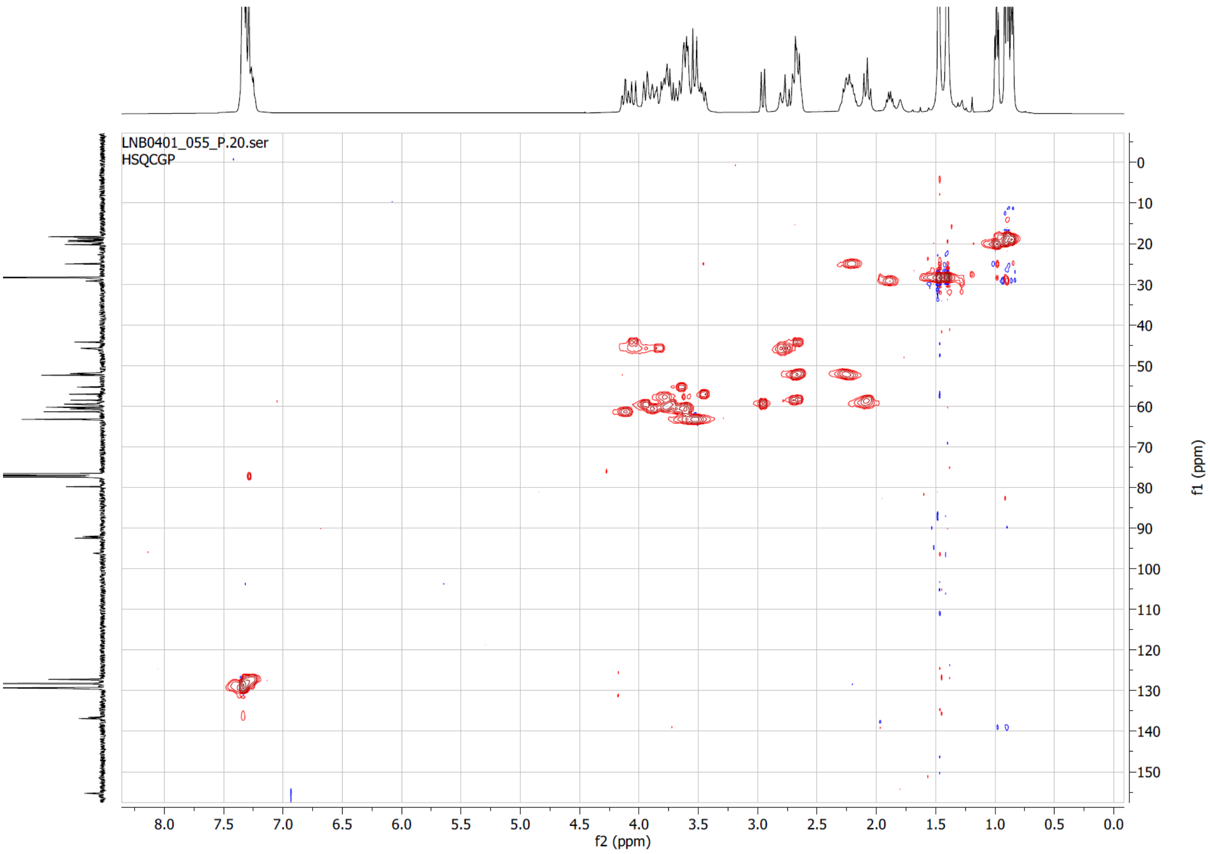
LNB0401_055_P.20.ser
HSQCGP
f2 (ppm)
f1 (ppm)

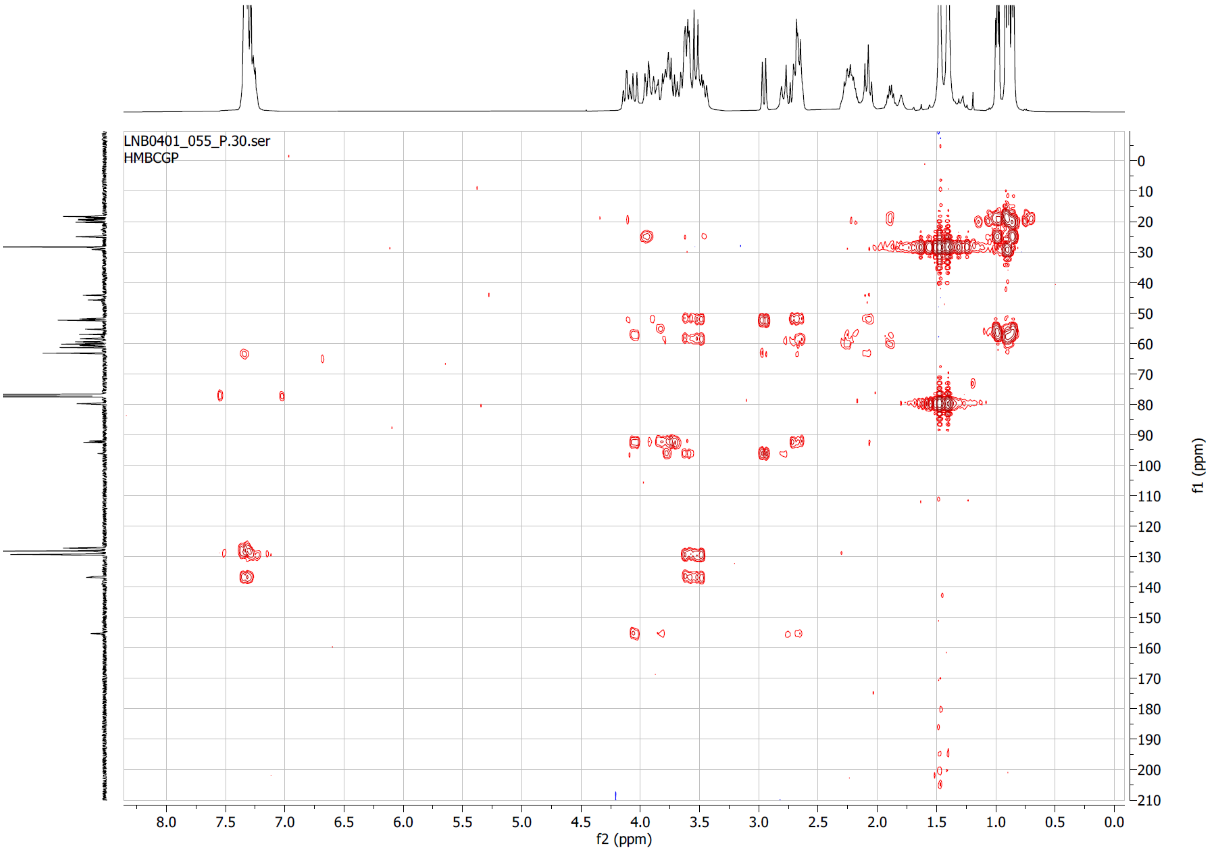
LNB0401_055_P.30.ser
HMBCGP
f2 (ppm)
f1 (ppm)

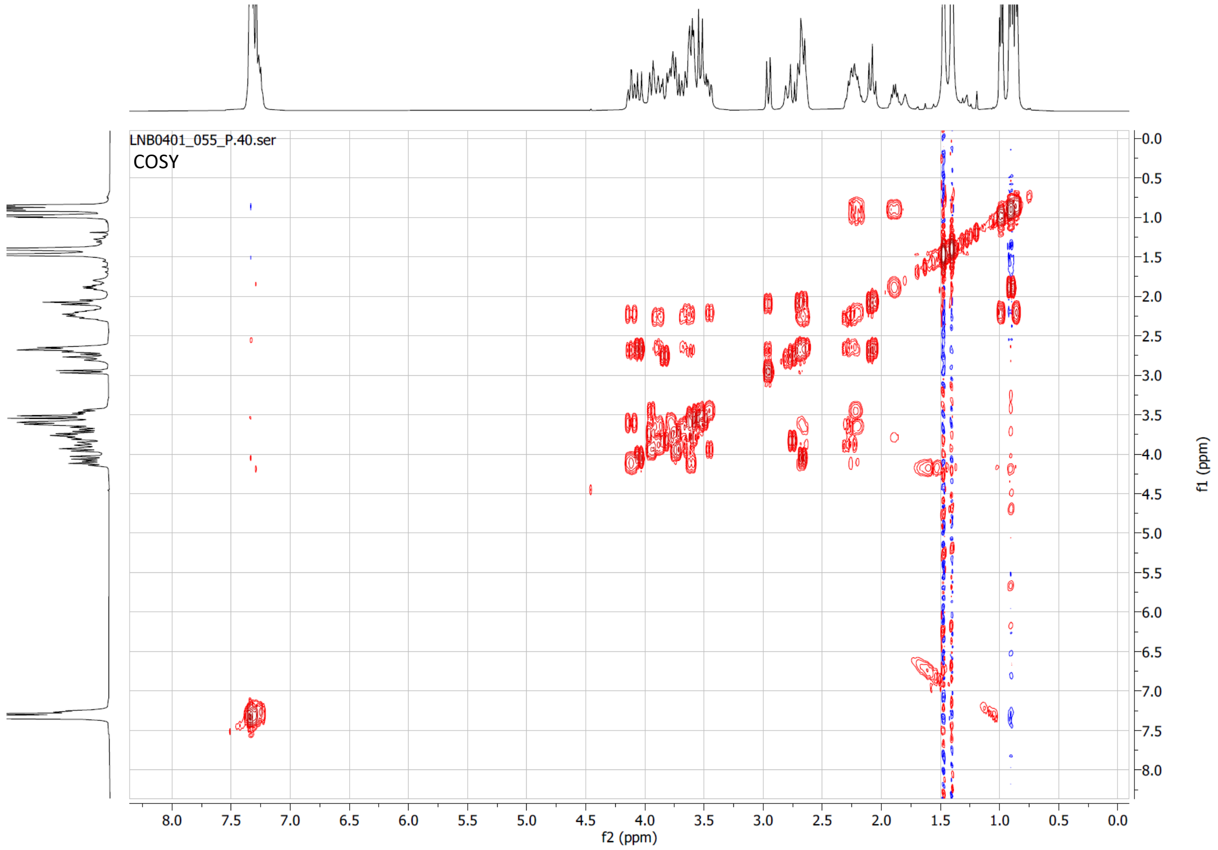

LNB0401_055_P.40.ser
COSY
f2 (ppm)
f1 (ppm)

***tert*-butyl (3*S*)-10-benzyl-3-isobutyl-1,7-dioxa-4,10-diazaspiro[5.5]undecane-4-carboxylate (46) [^{1}H-NMR data: 400 MHz, $CDCl_3$; $^{13}C\{^{1}H\}$-NMR data: 101 MHz, $CDCl_3$; 2D NMR spectra: HSQC, HMBC, COSY, all in $CDCl_3$]:**

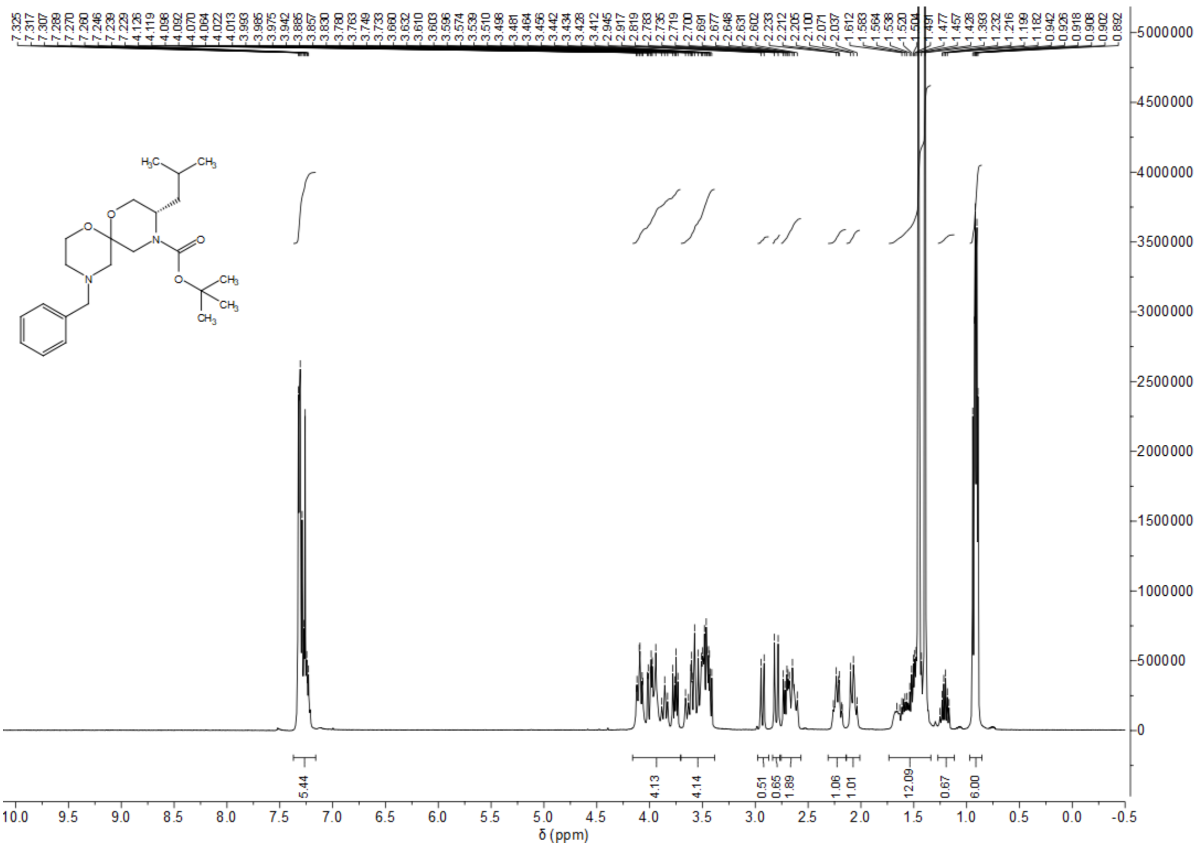

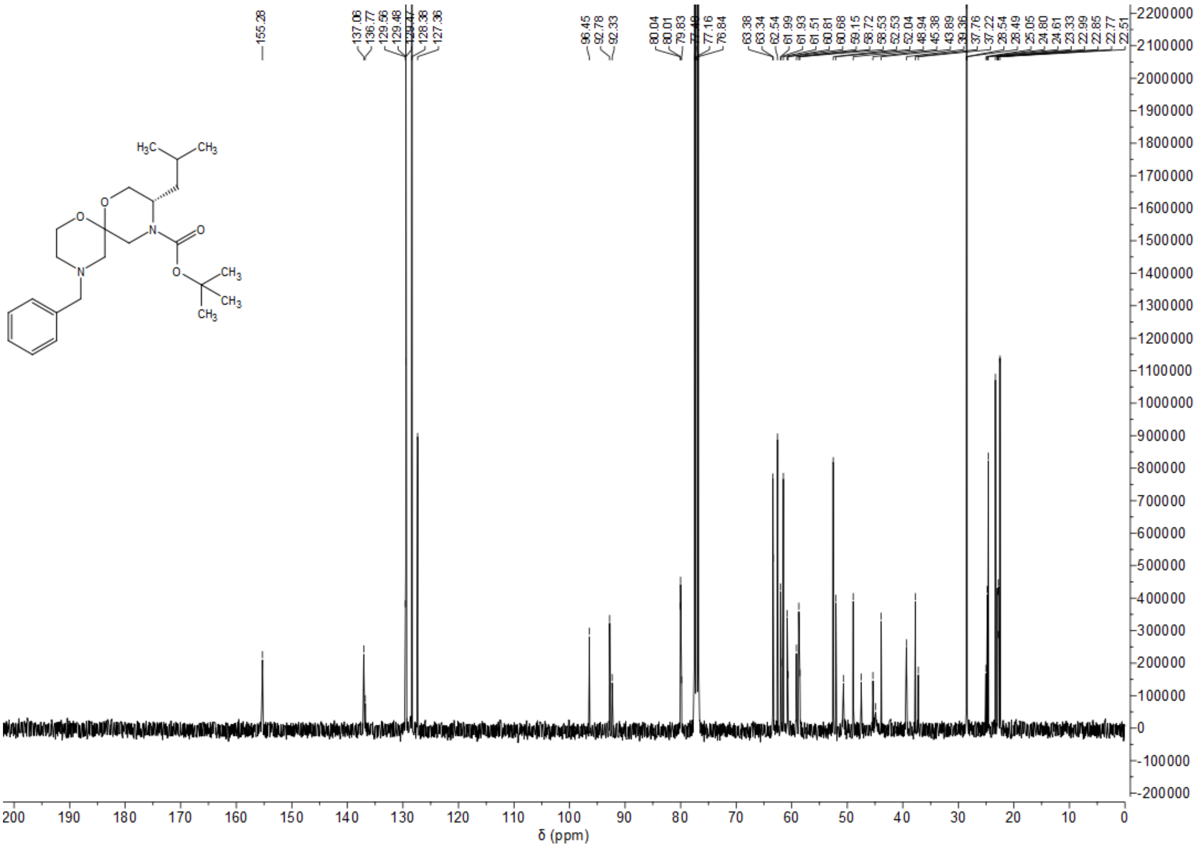

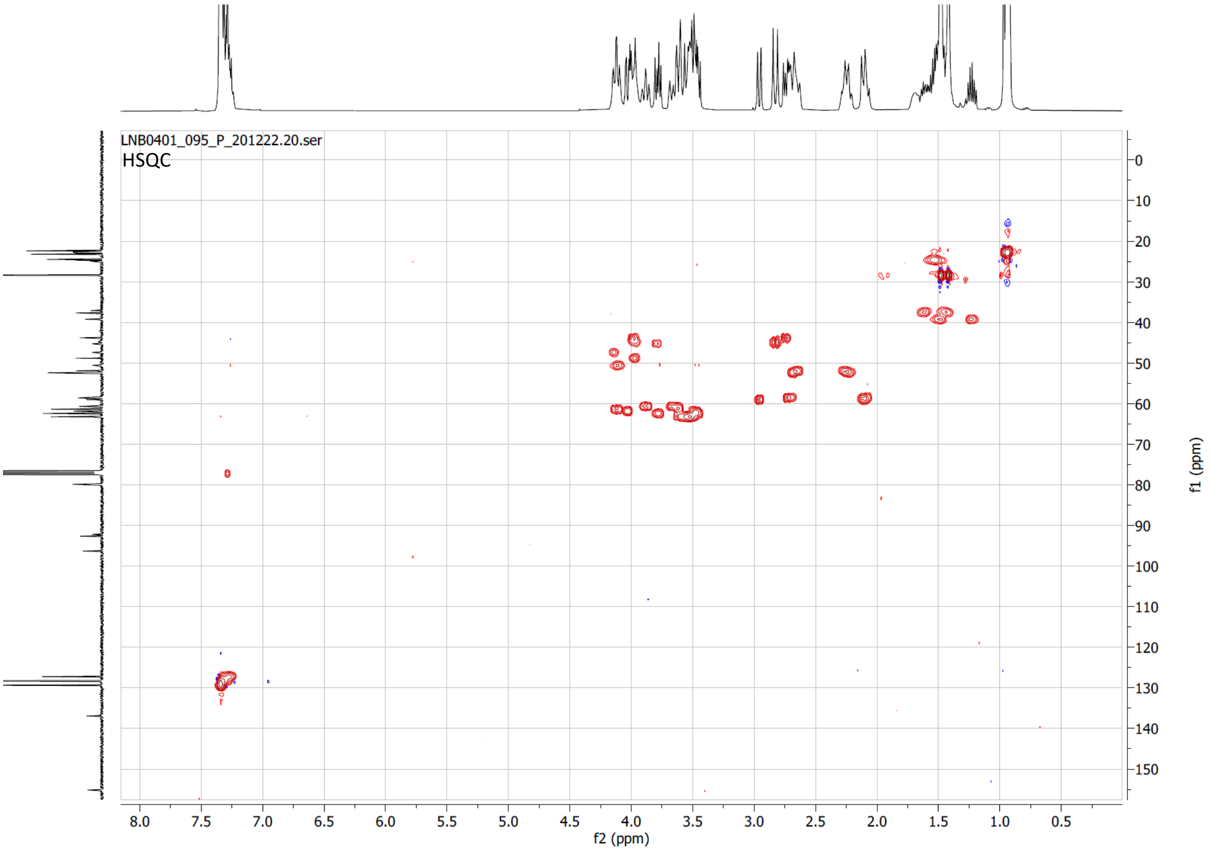
LNB0401_095_P_201222.20.ser
HSQC
f2 (ppm)
f1 (ppm)

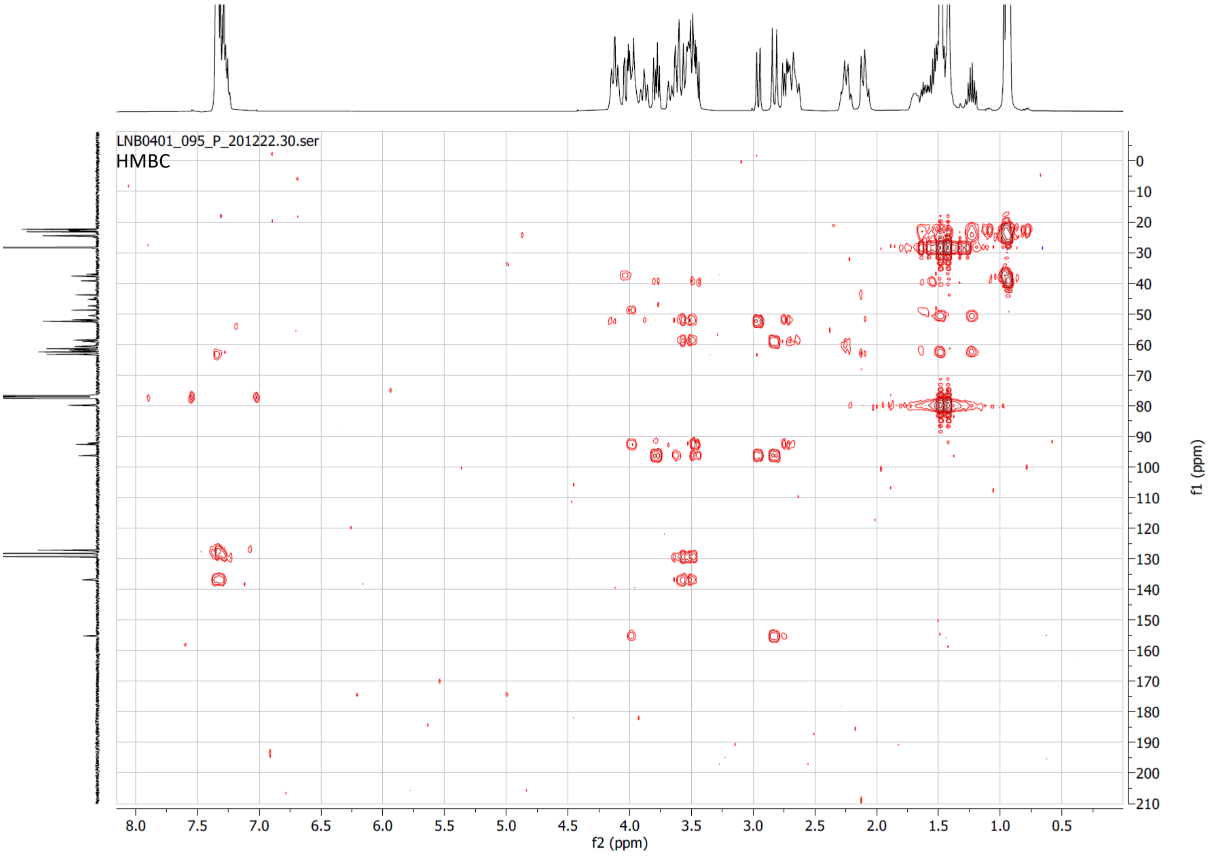
LNB0401_095_P_201222.30.ser
HMBC
f2 (ppm)
f1 (ppm)

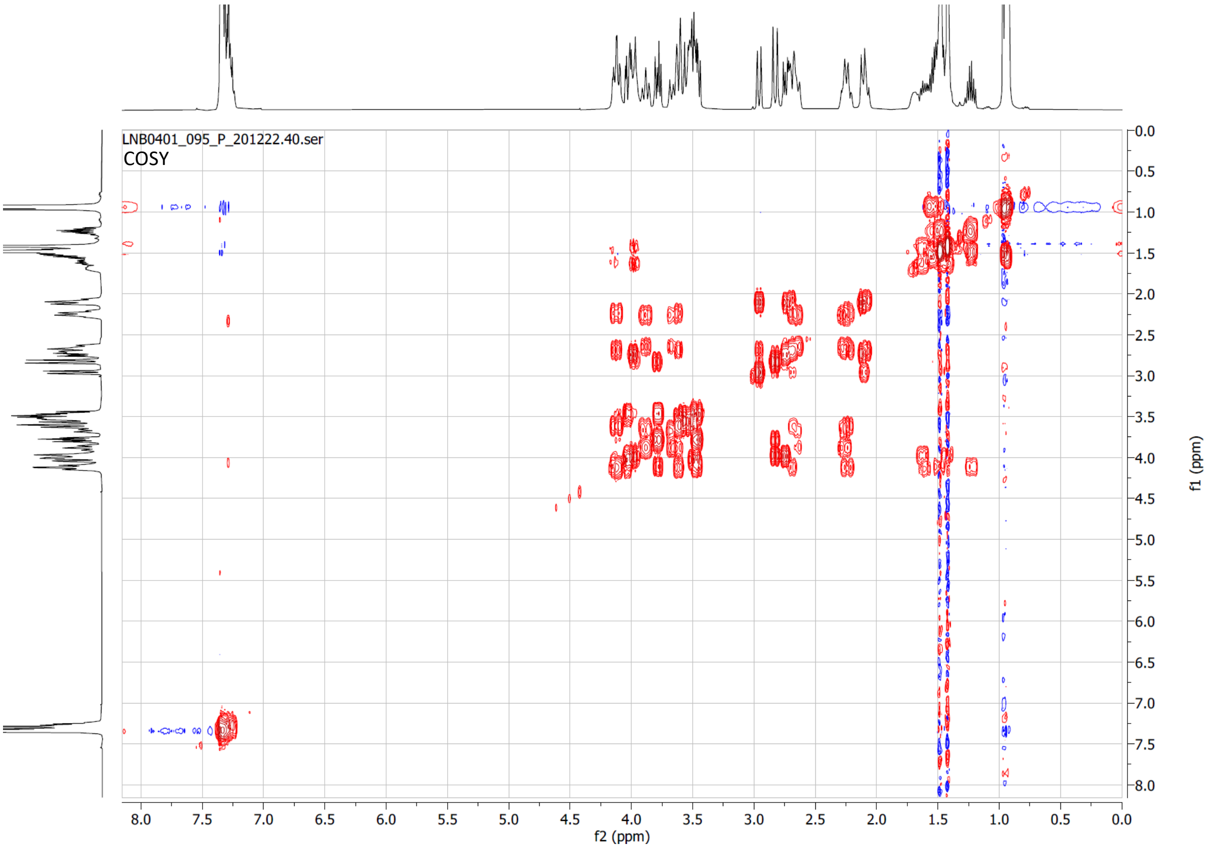

LNB0401_095_P_201222.40.ser
COSY
f2 (ppm)
f1 (ppm)

***tert*-butyl (3*S*)-10-benzyl-3-phenyl-1,7-dioxa-4,10-diazaspiro[5.5]undecane-4-carboxylate (47) [^{1}H-NMR data: 400 MHz, $CDCl_3$; $^{13}C\{^1H\}$-NMR data: 101 MHz, $CDCl_3$; 2D NMR spectra: HSQC, HMBC, COSY, all in $CDCl_3$]:**

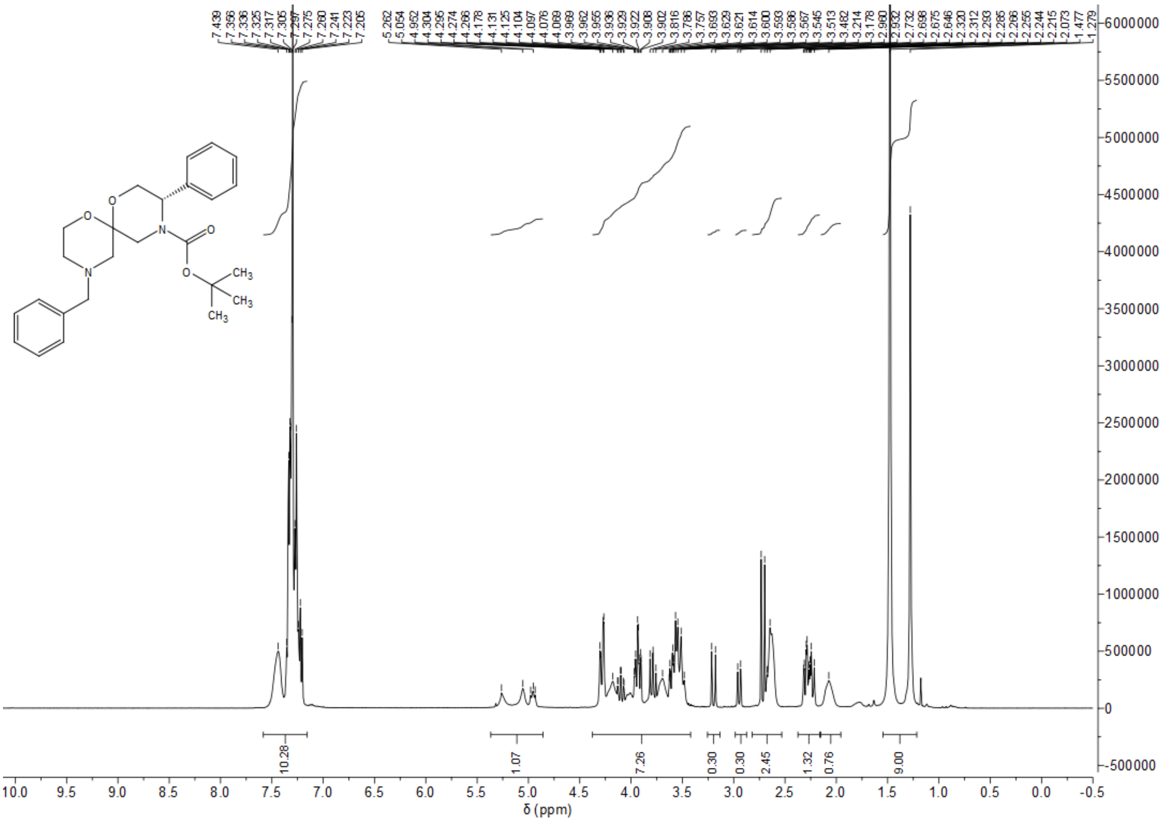

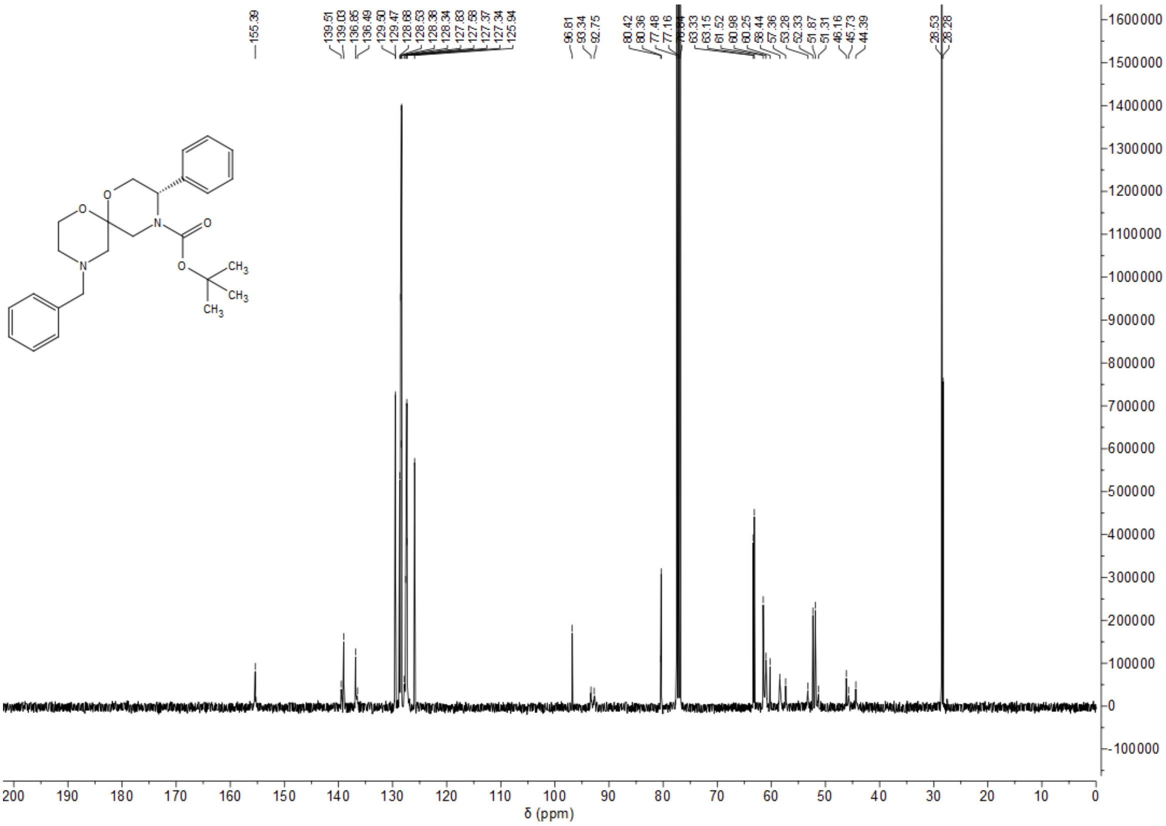

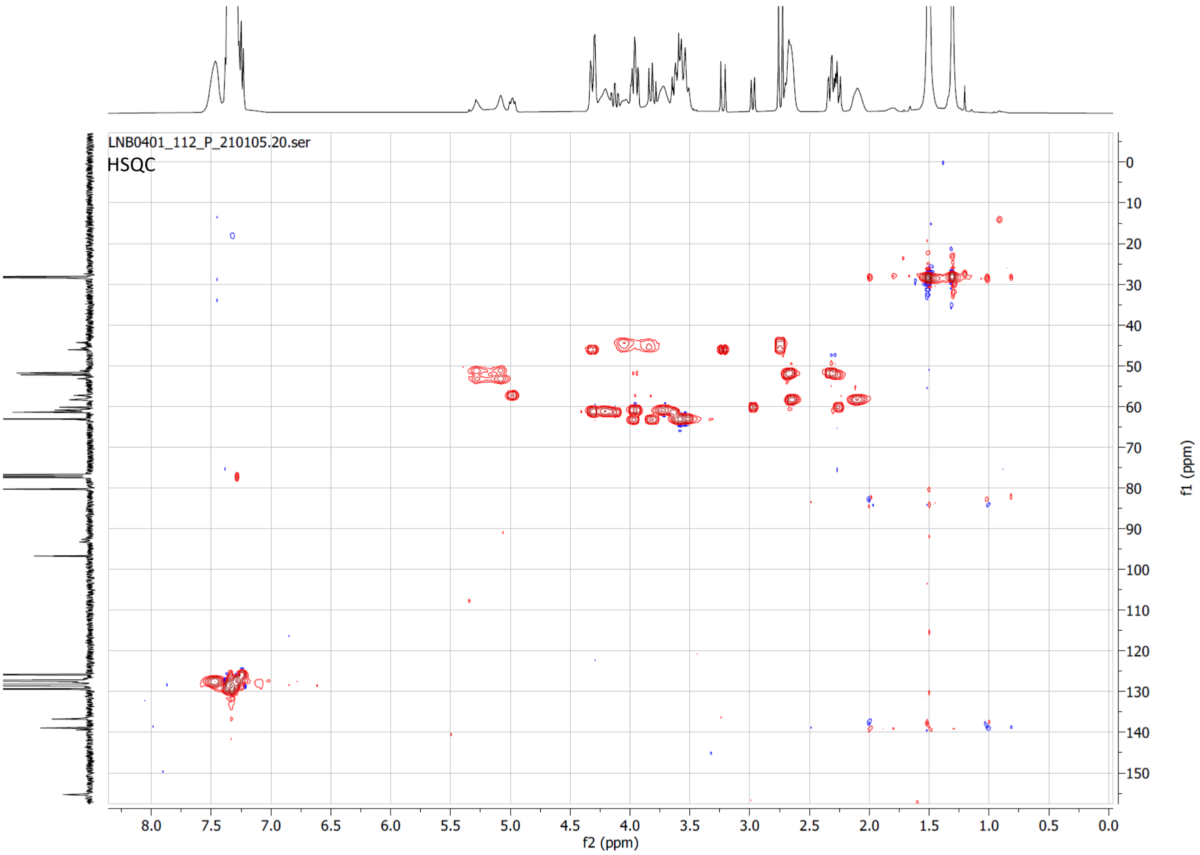
LNB0401_112_P_210105.20.ser
HSQC
f2 (ppm)
f1 (ppm)

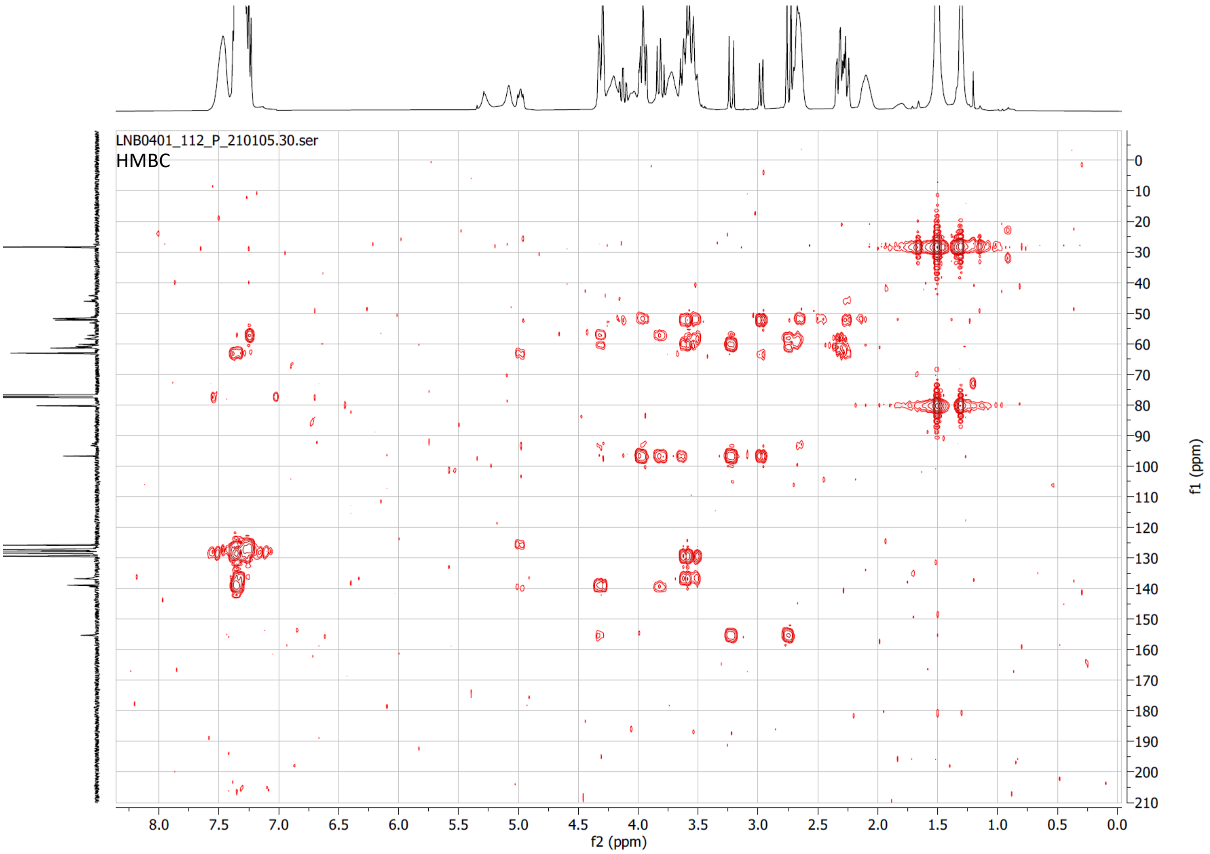
LNB0401_112_P_210105.30.ser
HMBC
f2 (ppm)
f1 (ppm)

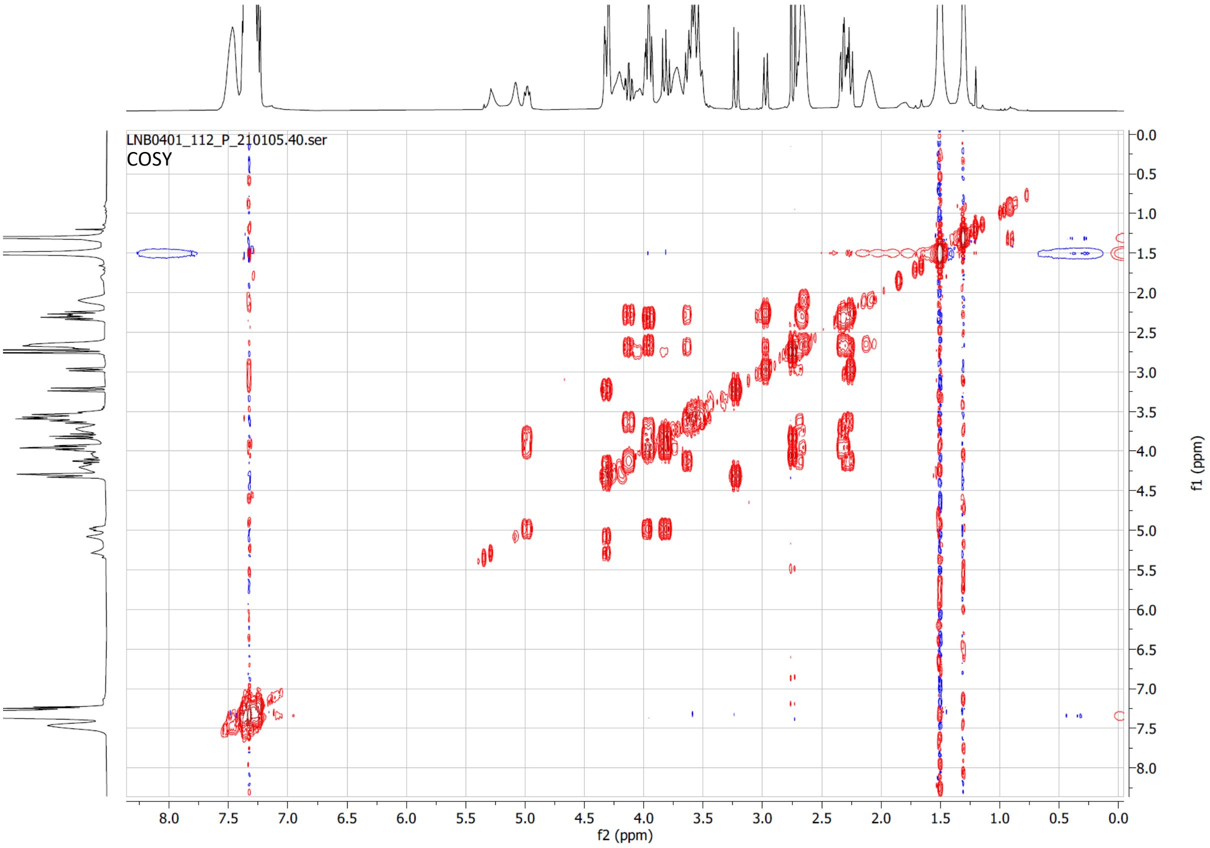
LNB0401_112_P_210105.40.ser
COSY
f2 (ppm)
f1 (ppm)

***tert*-butyl (3*S*)-3,10-dibenzyl-1,7-dioxa-4,10-diazaspiro[5.5]undecane-4-carboxylate (48) [^{1}H-NMR data: 400 MHz, CDCl$_3$; ^{13}C{^{1}H}-NMR data: 101 MHz, CDCl$_3$; 2D NMR spectra: HSQC, HMBC, COSY, all in CDCl$_3$]:**

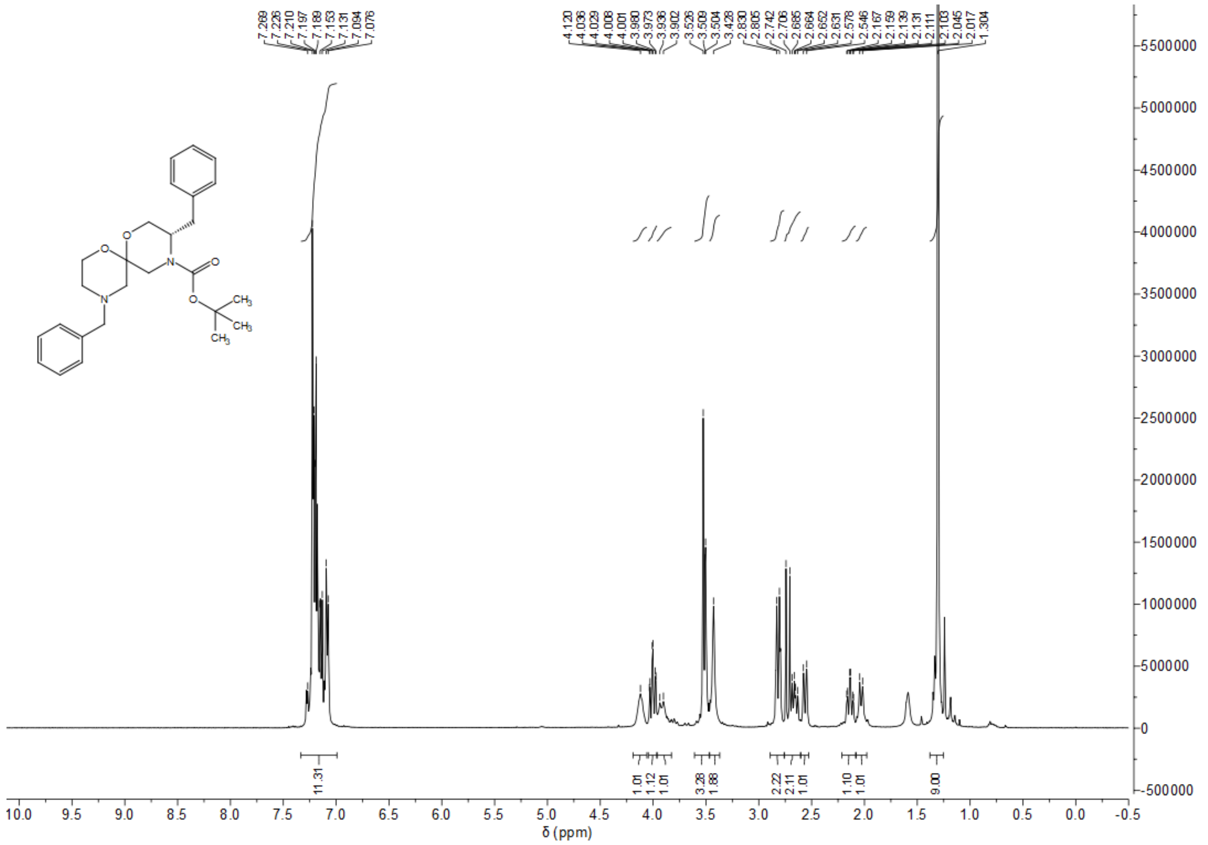

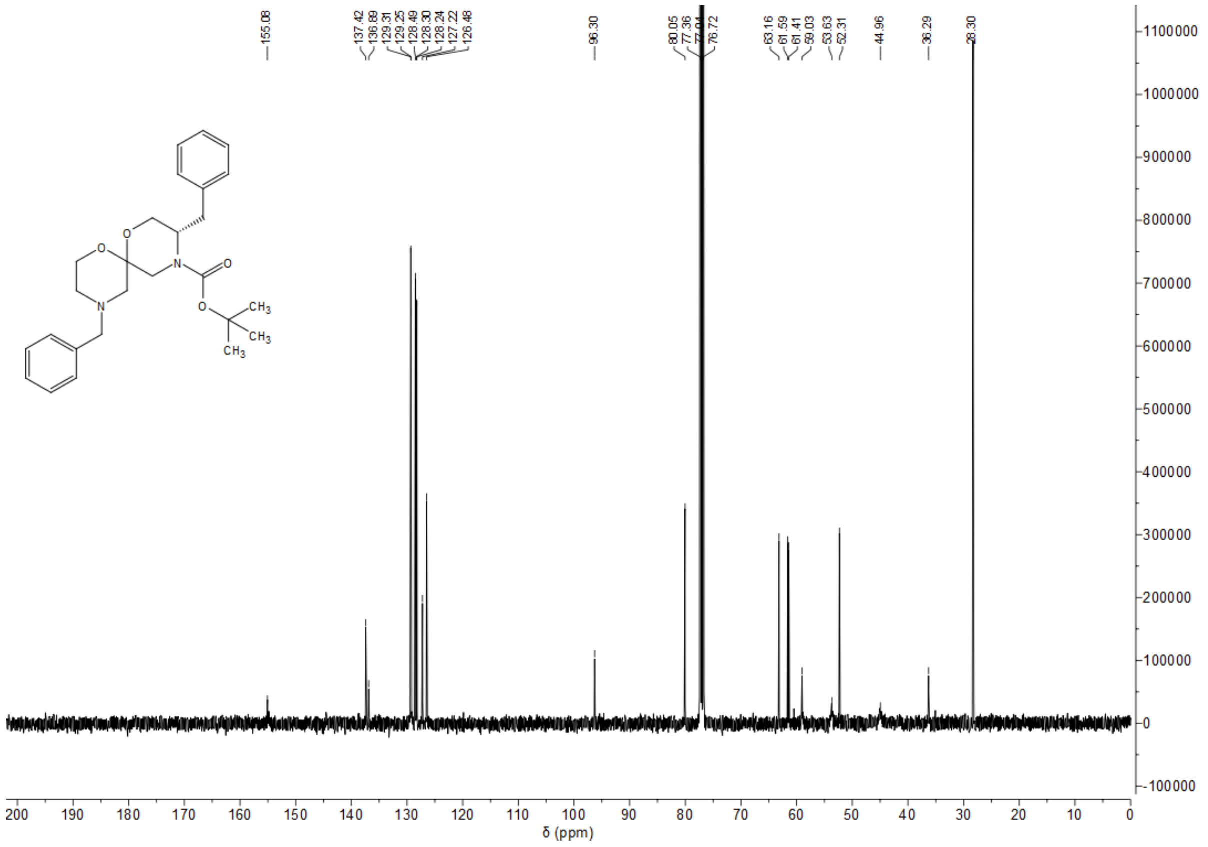

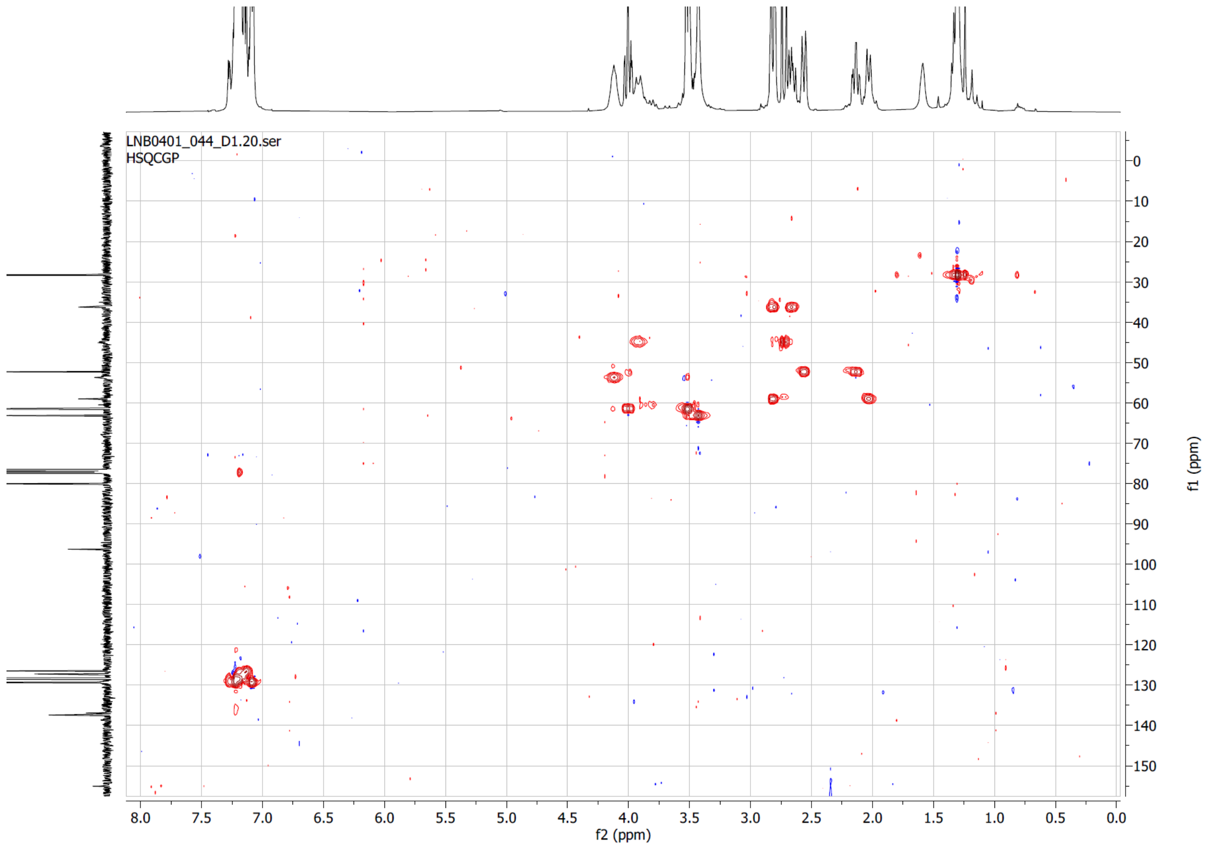
LNB0401_044_D1.20.ser
HSQCGP
f2 (ppm)
f1 (ppm)

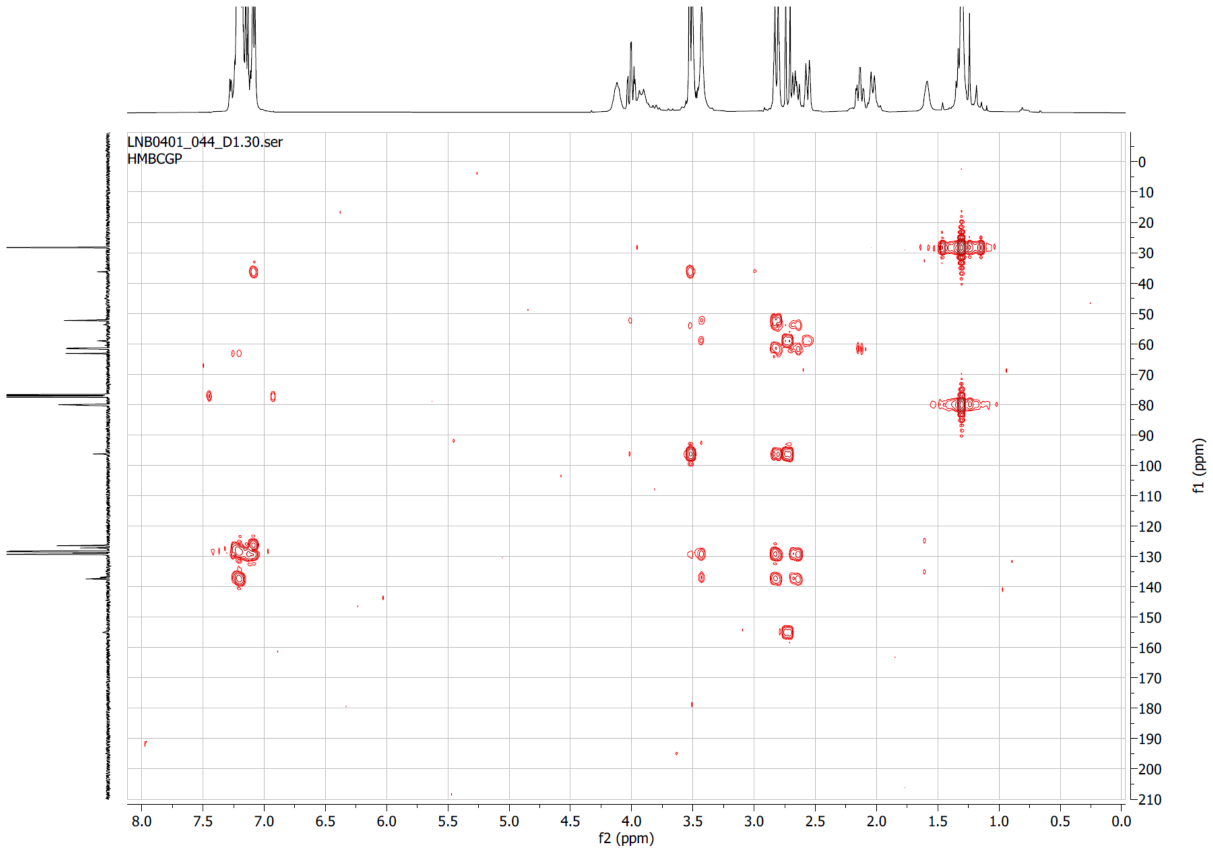
LNB0401_044_D1.30.ser
HMBCGP
f2 (ppm)
f1 (ppm)

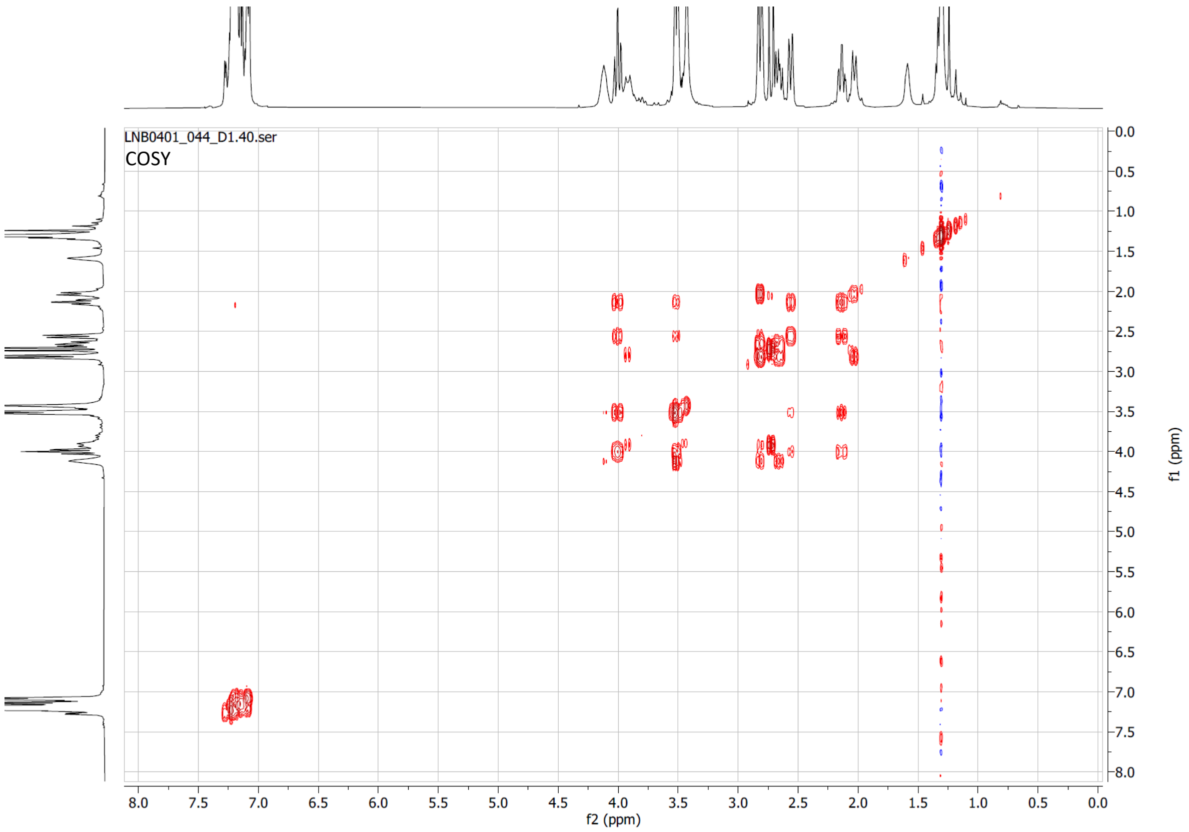
LNB0401_044_D1.40.ser
COSY
f2 (ppm)
f1 (ppm)

10-benzyl 4-(*tert*-butyl) (3*S*)-3-benzyl-1,7-dioxa-4,10-diazaspiro[5.5]undecane-4,10-dicarboxylate (S36) [^{1}H-NMR data: 400 MHz, CDCl$_3$; ^{13}C{^{1}H}-NMR data: 101 MHz, CDCl$_3$; 2D NMR spectra: HSQC, HMBC, COSY, all in CDCl$_3$]:

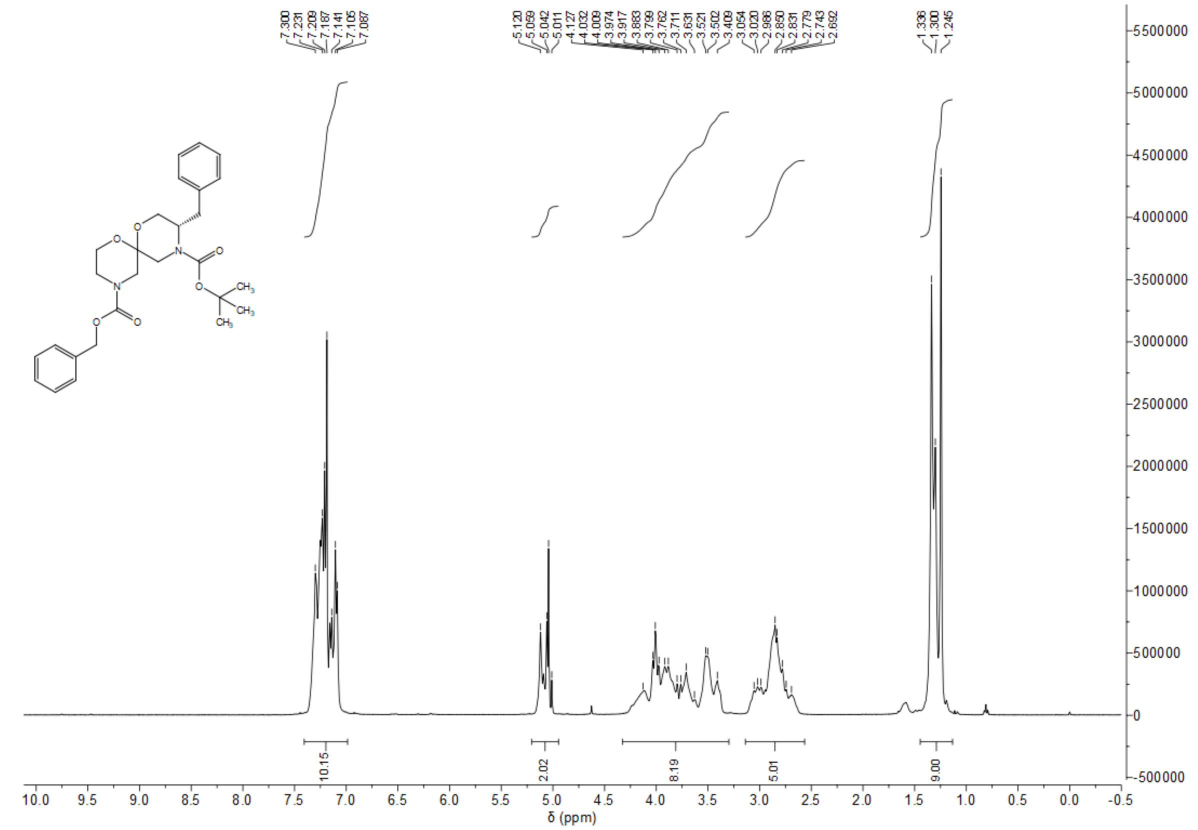

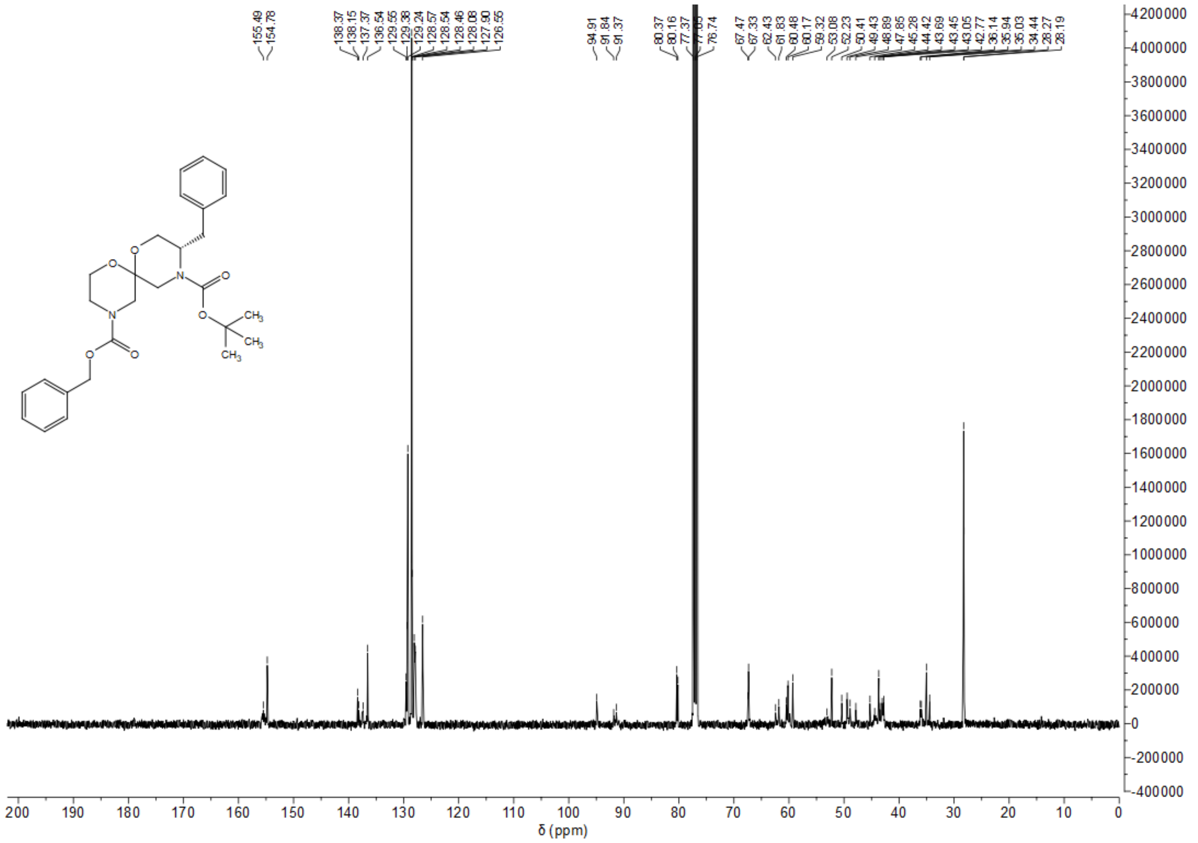

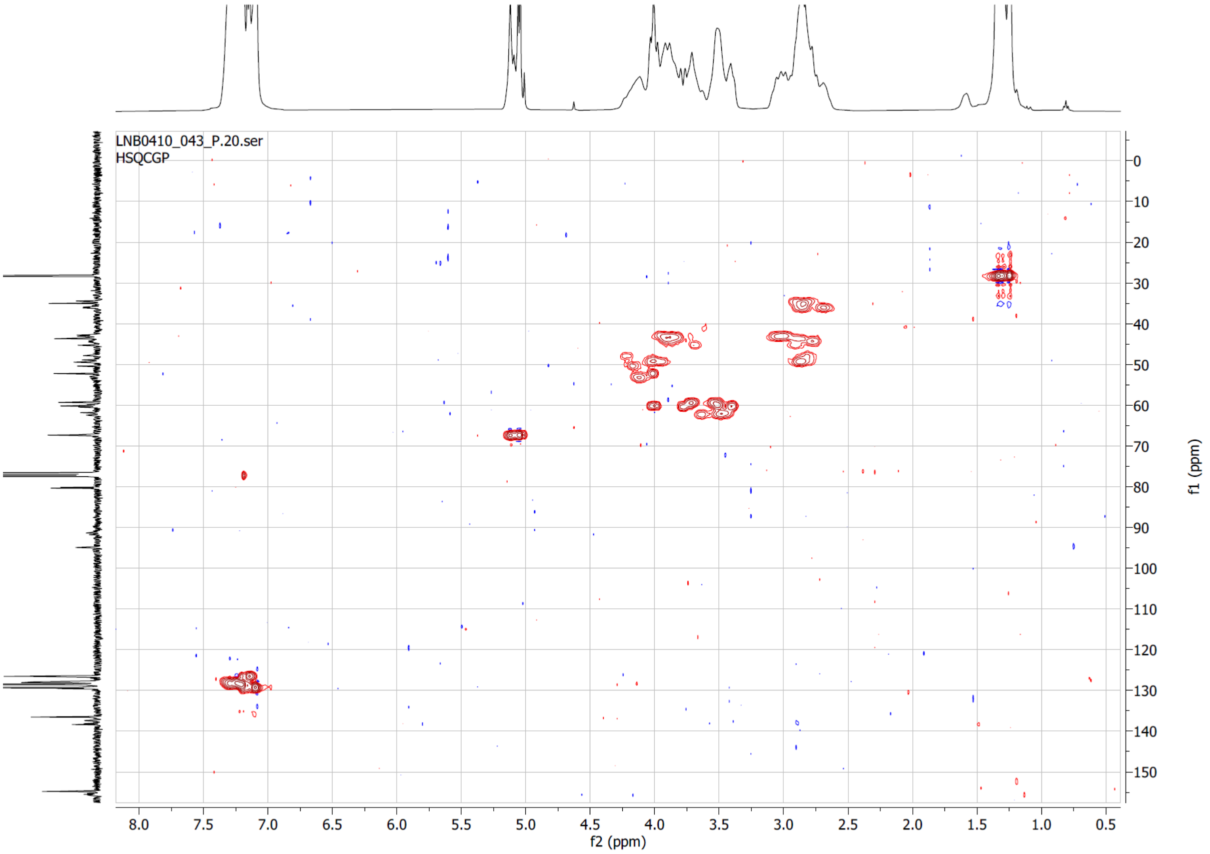
LNB0410_043_P.20.ser
HSQCGP
f2 (ppm)
f1 (ppm)

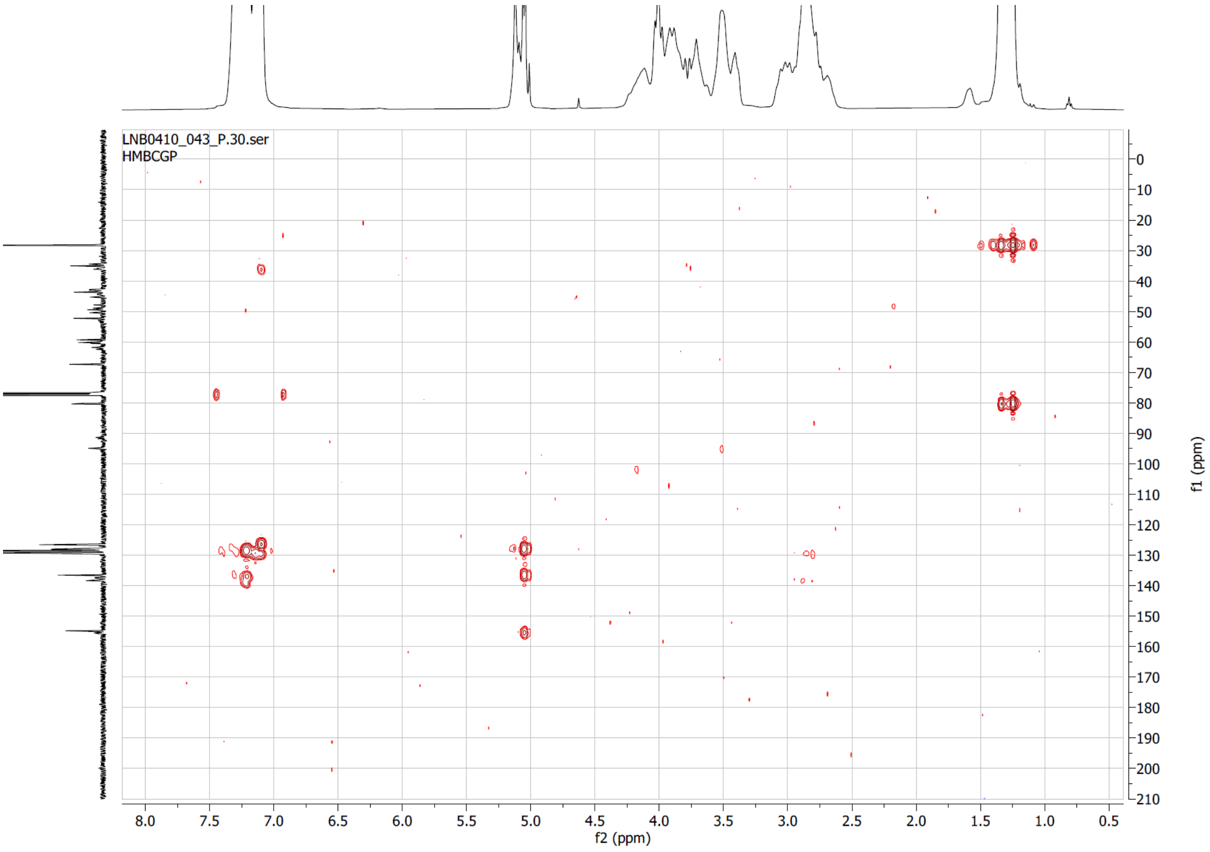
LNB0410_043_P.30.ser
HMBCGP
f2 (ppm)
f1 (ppm)

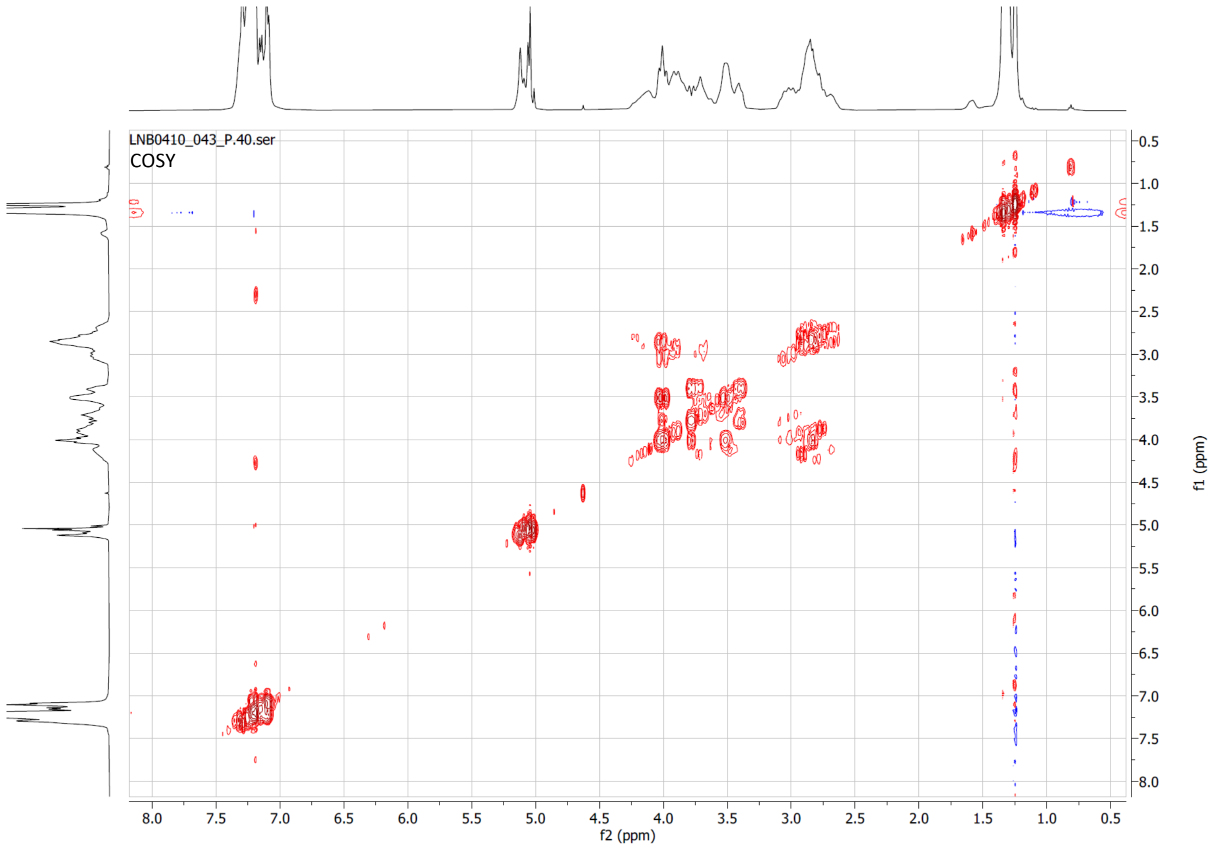
LNB0410_043_P.40.ser
COSY
f2 (ppm)
f1 (ppm)

4-((9*H*-fluoren-9-yl)methyl) 10-benzyl (3*S*)-3-benzyl-1,7-dioxa-4,10-diazaspiro[5.5]undecane-4,10-dicarboxylate (55) [^{1}H-NMR data: 400 MHz, $CDCl_3$; $^{13}C\{^{1}H\}$-NMR data: 101 MHz, $CDCl_3$; 2D NMR spectra: HSQC, HMBC, COSY, all in $CDCl_3$]:

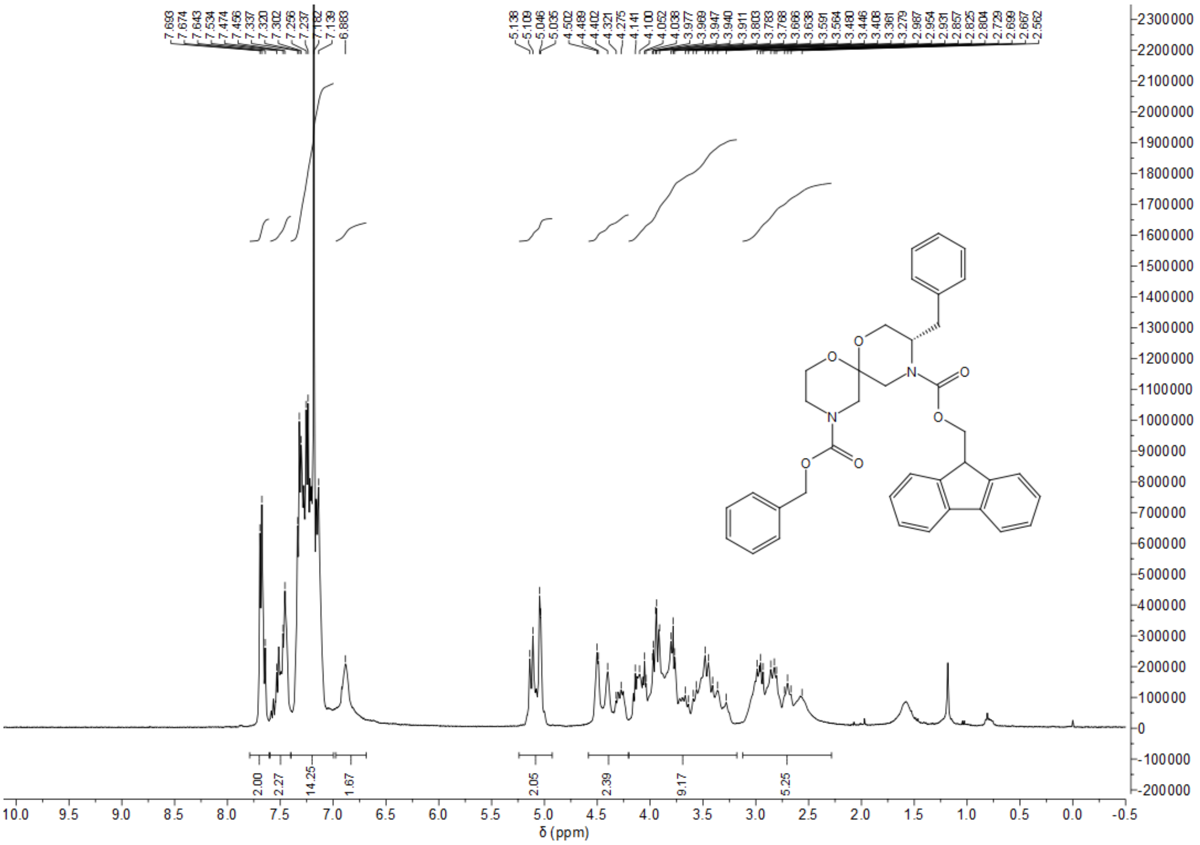

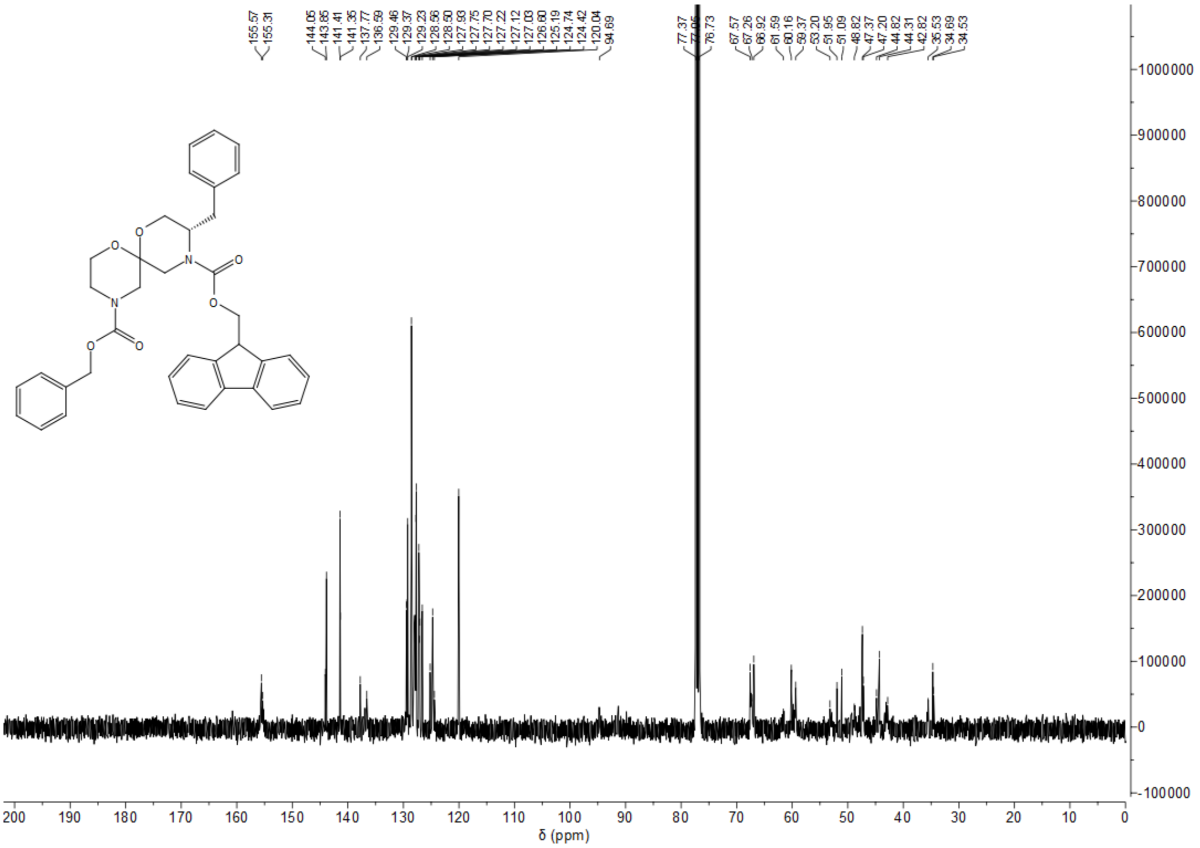

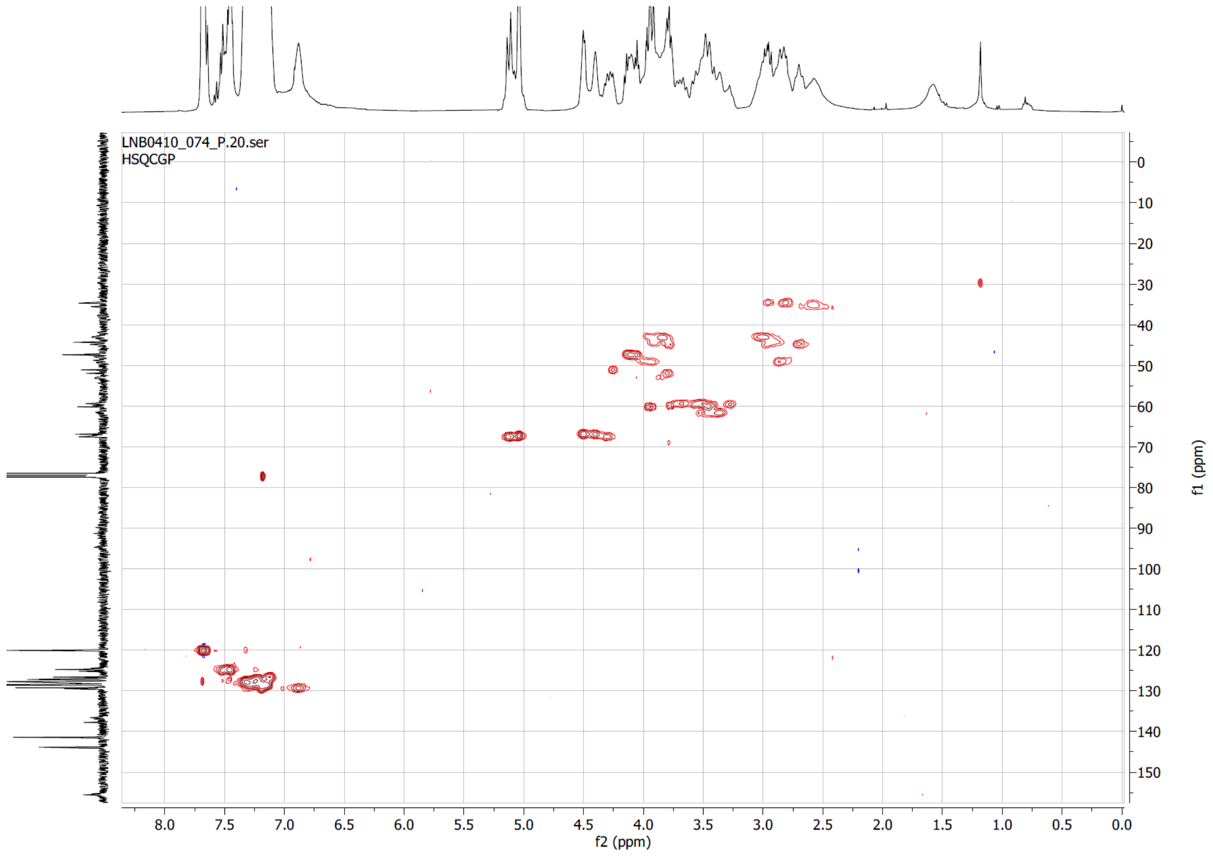
LNB0410_074_P.20.ser
HSQCGP
f2 (ppm)
f1 (ppm)

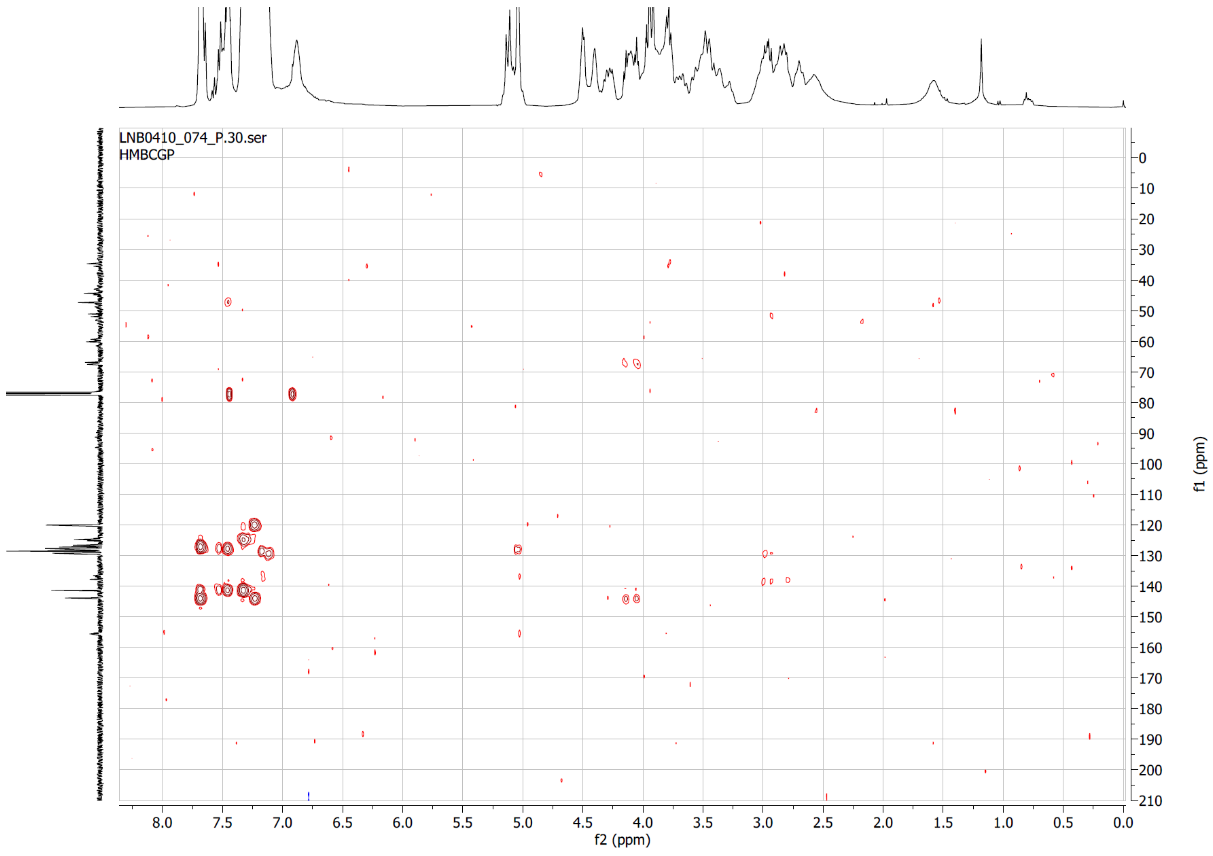
LNB0410_074_P.30.ser
HMBCGP
f2 (ppm)
f1 (ppm)

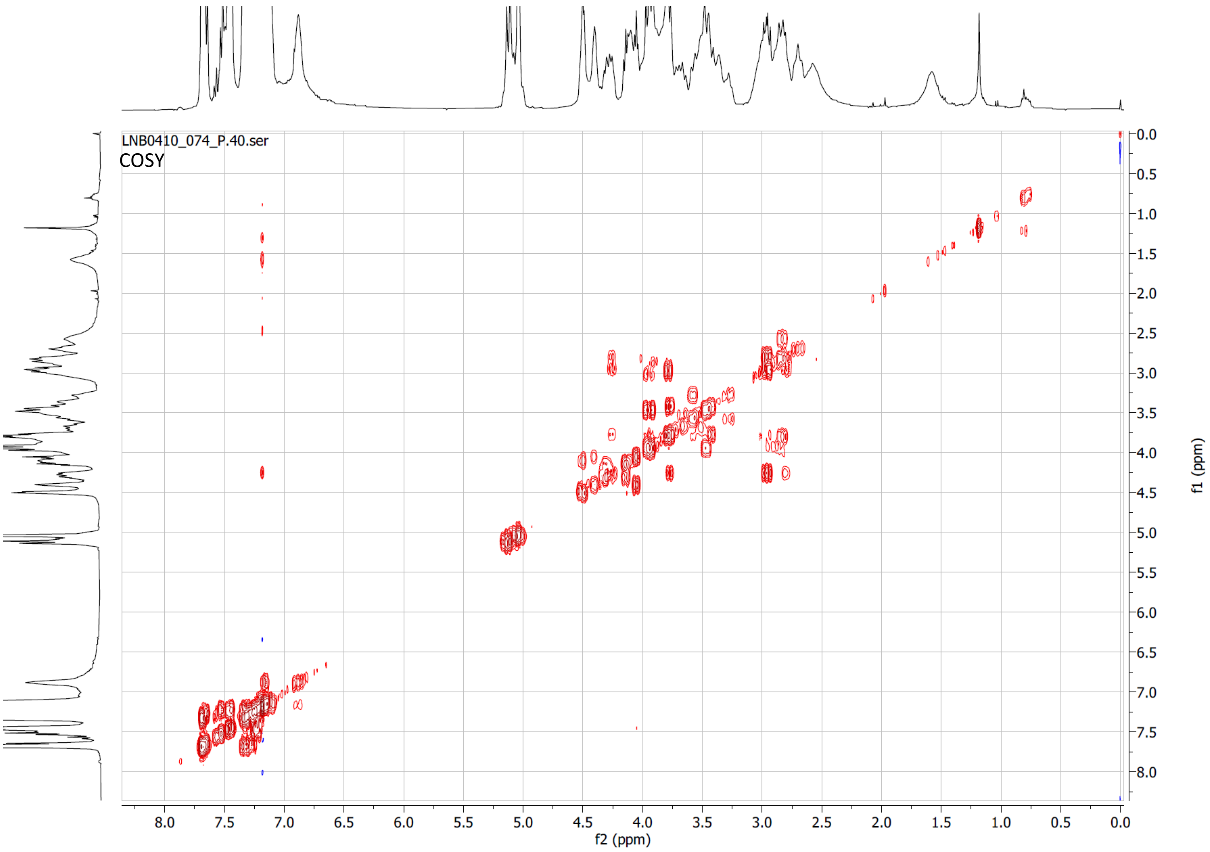
LNB0410_074_P.40.ser
COSY
f2 (ppm)
f1 (ppm)
8.0 7.5 7.0 6.5 6.0 5.5 5.0 4.5 4.0 3.5 3.0 2.5 2.0 1.5 1.0 0.5 0.0

***tert*-butyl 10-benzyl-3,3-dimethyl-1,7-dioxa-4,10-diazaspiro[5.5]undecane-4-carboxylate (49) [^{1}H-NMR data: 400 MHz, CDCl$_3$; ^{13}C{^{1}H}-NMR data: 101 MHz, CDCl$_3$; 2D NMR spectra: HSQC, HMBC, COSY, all in CDCl$_3$]:**

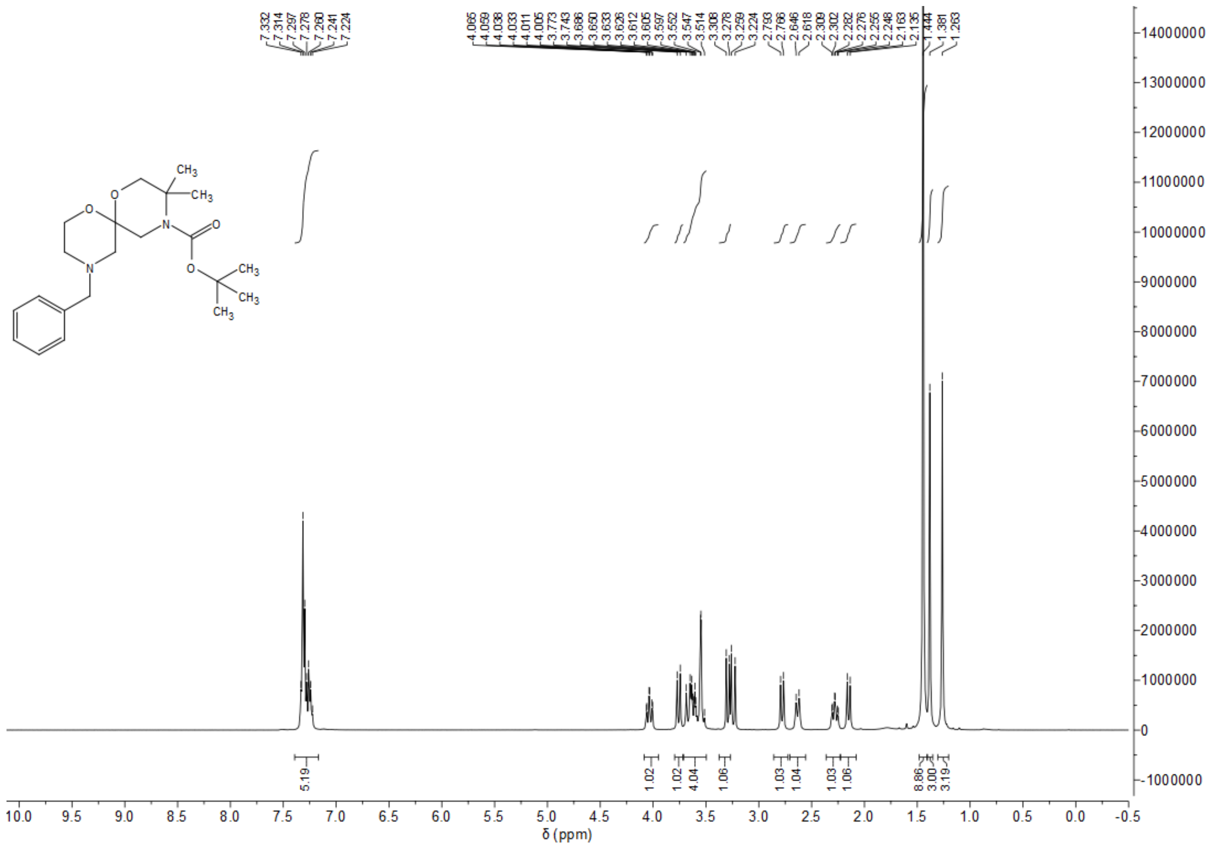

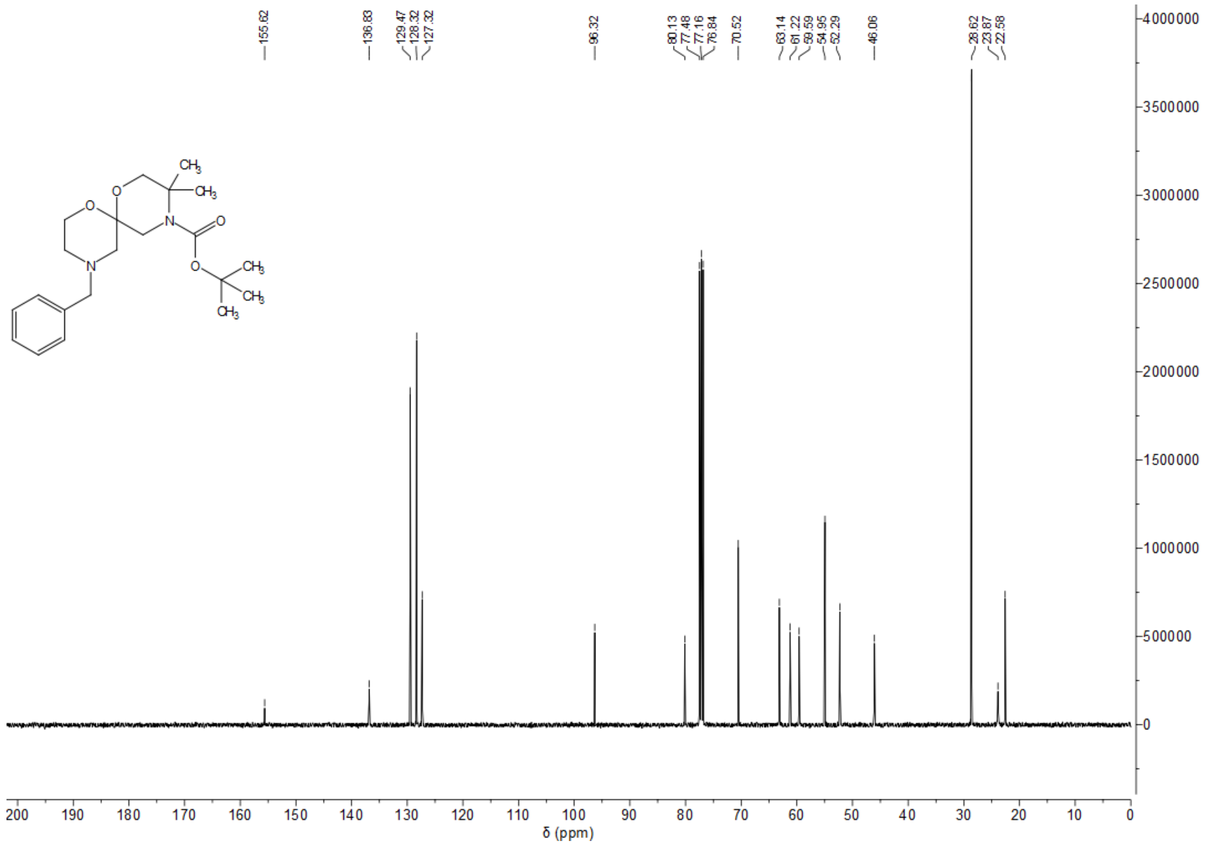

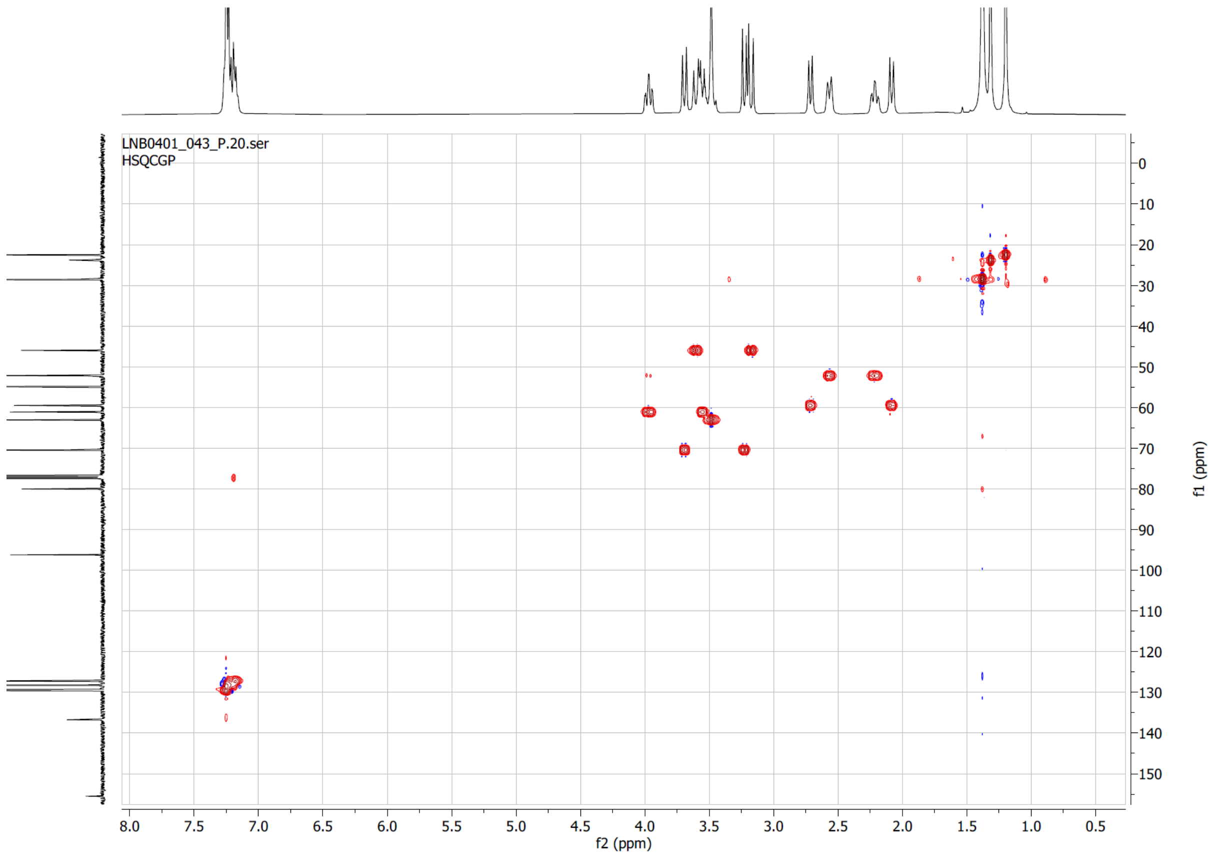
LNB0401_043_P.20.ser
HSQCGP
f2 (ppm)
f1 (ppm)

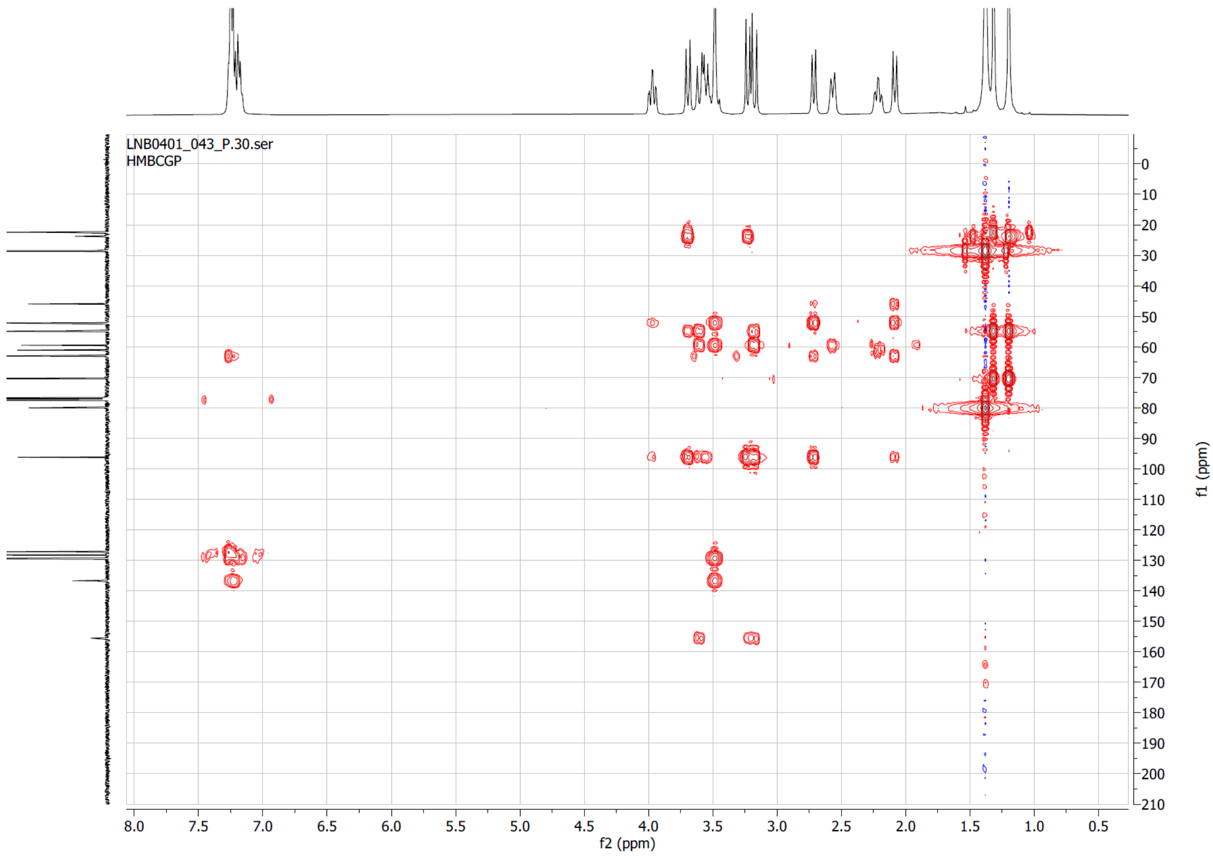
LNB0401_043_P.30.ser
HMBCGP
f2 (ppm)
f1 (ppm)

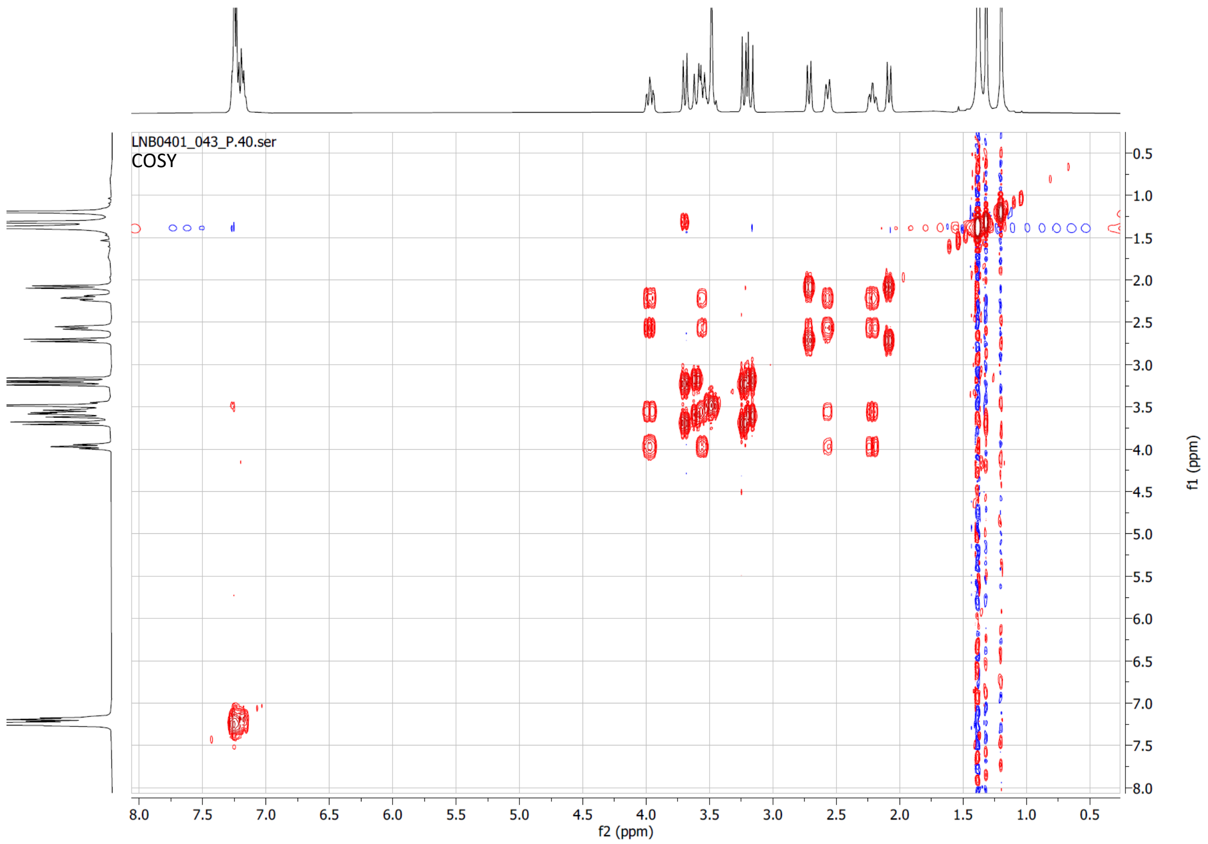

LNB0401_043_P.40.ser
COSY
f2 (ppm)
f1 (ppm)

***tert*-butyl (2*S*)-10-benzyl-2-methyl-1,7-dioxa-4,10-diazaspiro[5.5]undecane-4-carboxylate (50) [^{1}H-NMR data: 400 MHz, CDCl$_3$; ^{13}C{^{1}H}-NMR data: 101 MHz, CDCl$_3$; 2D NMR spectra: HSQC, HMBC, COSY, all in CDCl$_3$]:**

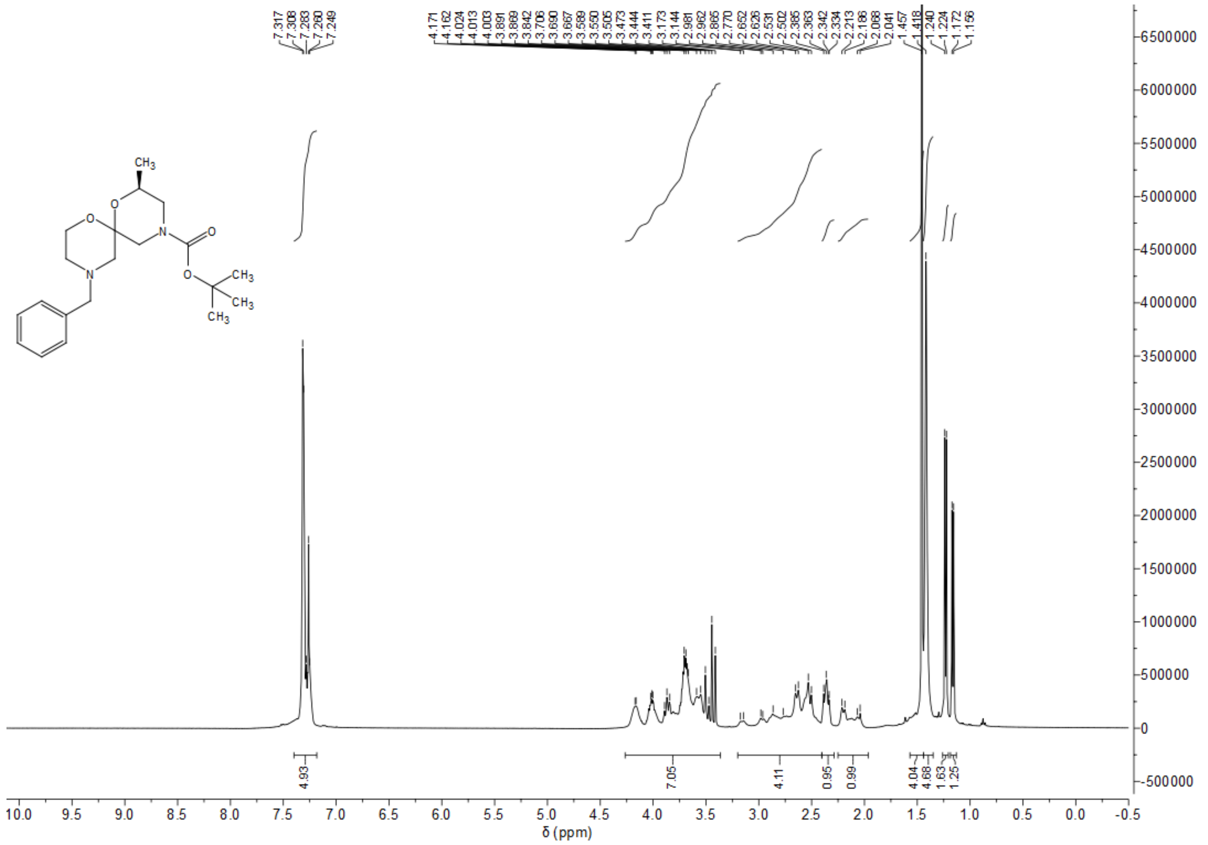

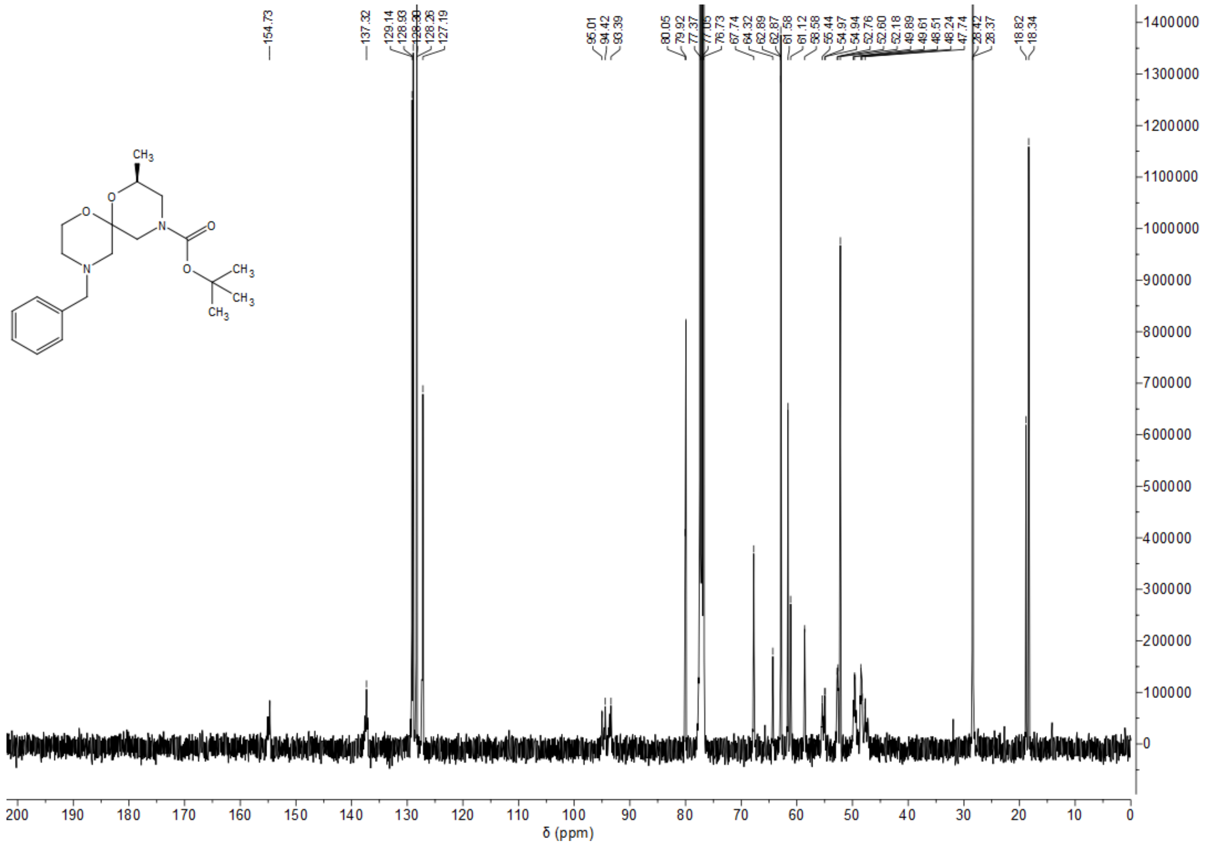

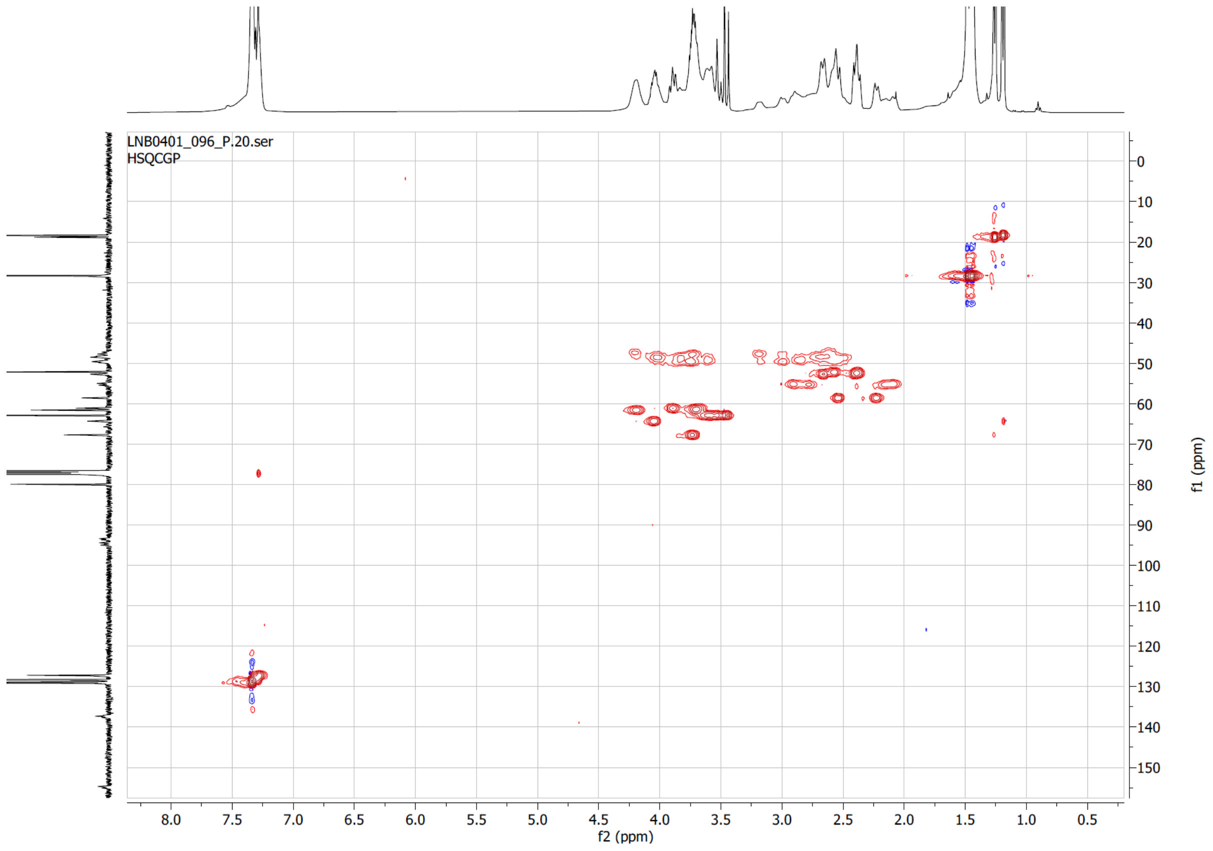
LNB0401_096_P.20.ser
HSQCGP
f2 (ppm)
f1 (ppm)

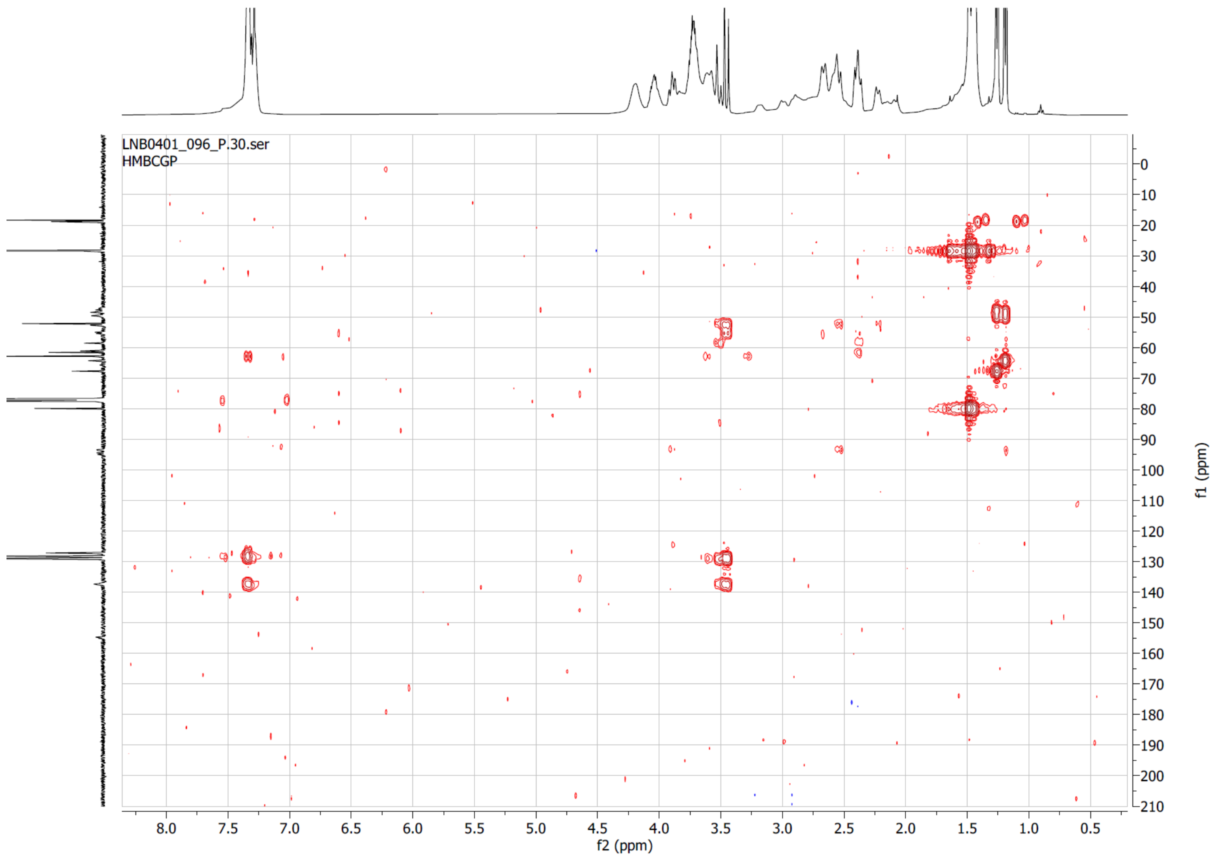
LNB0401_096_P.30.ser
HMBCGP
f2 (ppm)
f1 (ppm)

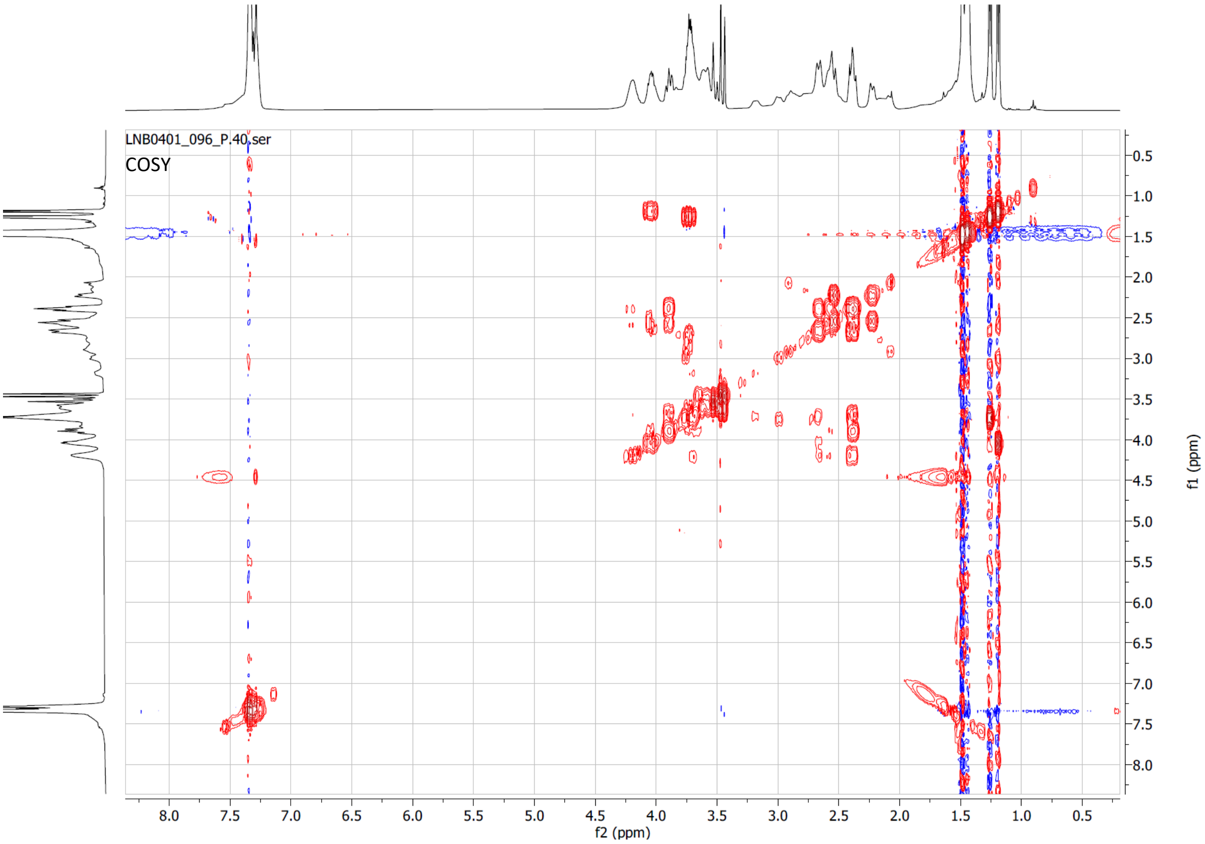
LNB0401_096_P.40.ser
COSY
f2 (ppm)
f1 (ppm)

tert-butyl (3_S_)-10-benzyl-3-isopropyl-2,2-dimethyl-1,7-dioxa-4,10-diazaspiro[5.5]undecane-4-carboxylate (51) [^{1}H-NMR data: 400 MHz, $CDCl_3$; $^{13}C\{^{1}H\}$-NMR data: 101 MHz, $CDCl_3$; 2D NMR spectra: HSQC, HMBC, COSY, all in $CDCl_3$]:

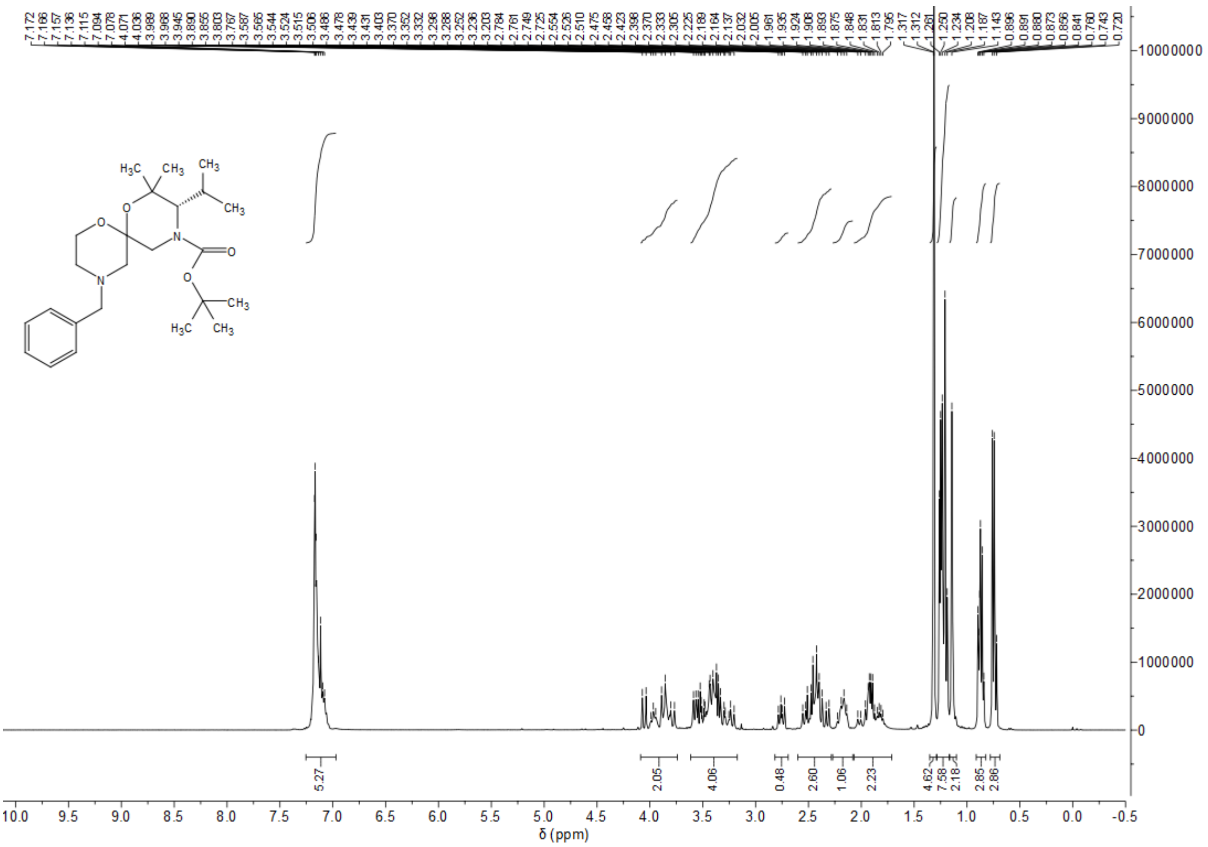

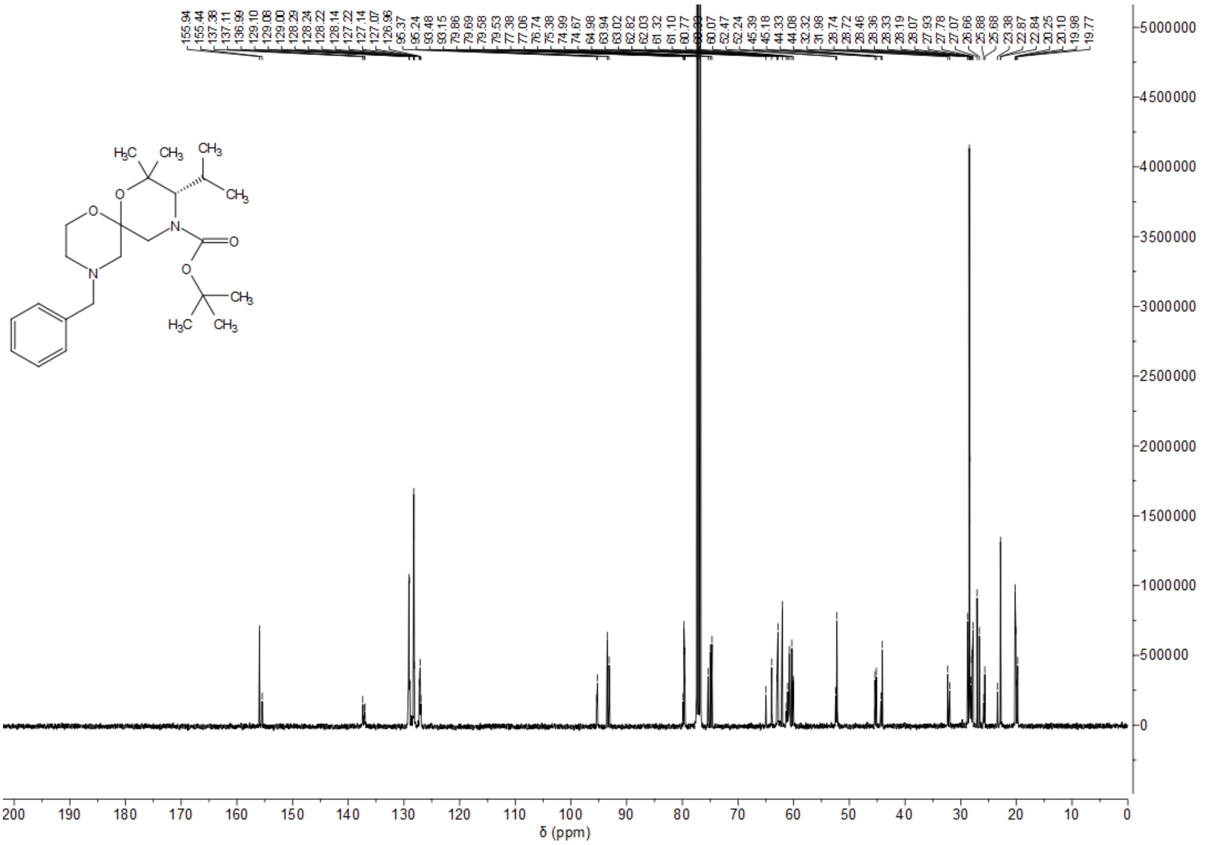

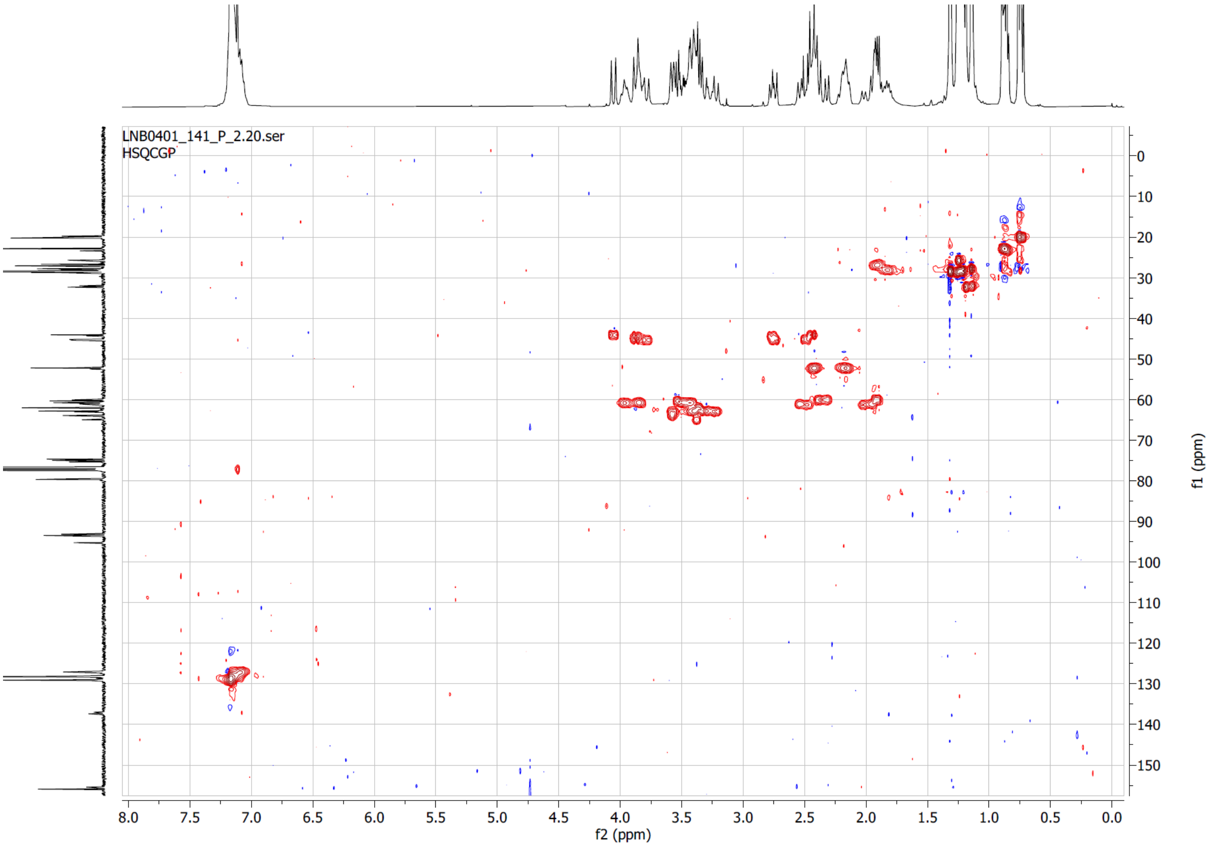
LNB0401_141_P_2.20.ser
HSQCGP
f2 (ppm)
f1 (ppm)

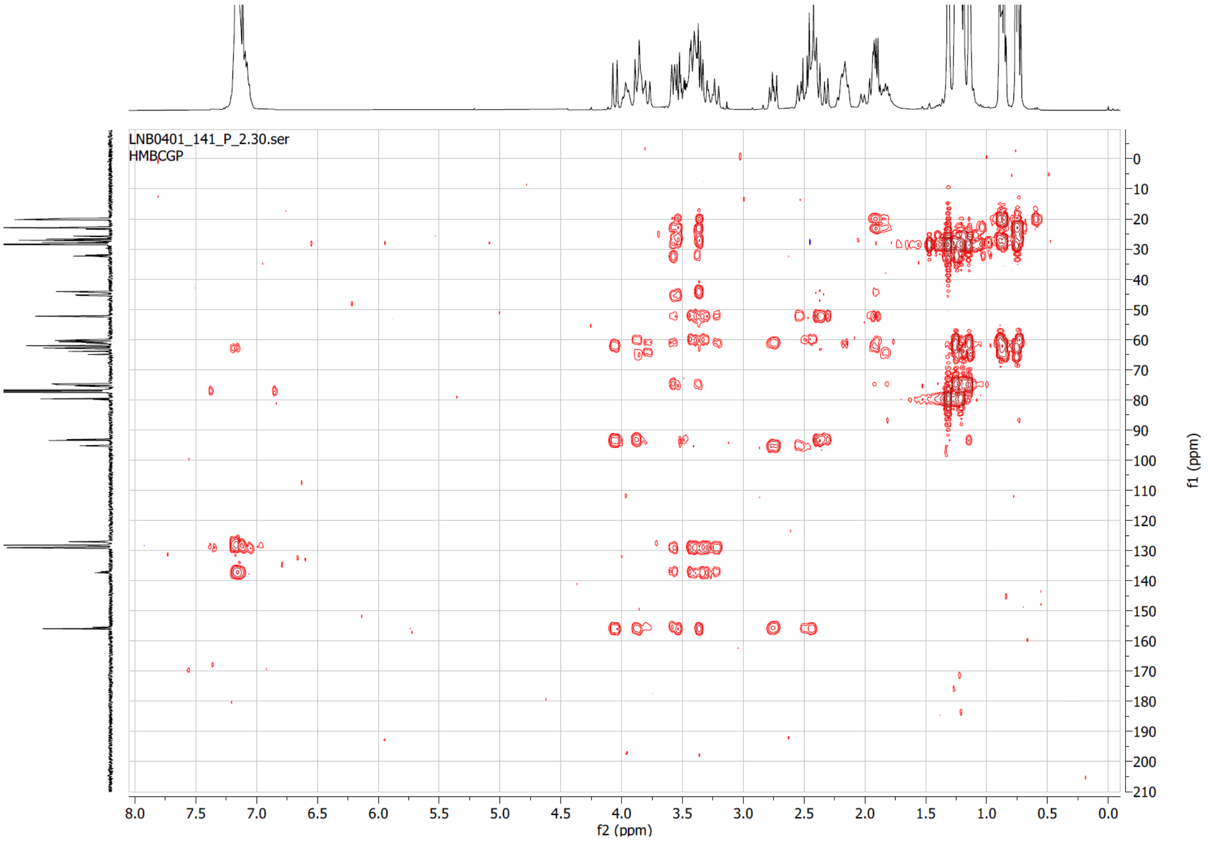
LNB0401_141_P_2.30.ser
HMBCGP
f2 (ppm)
f1 (ppm)

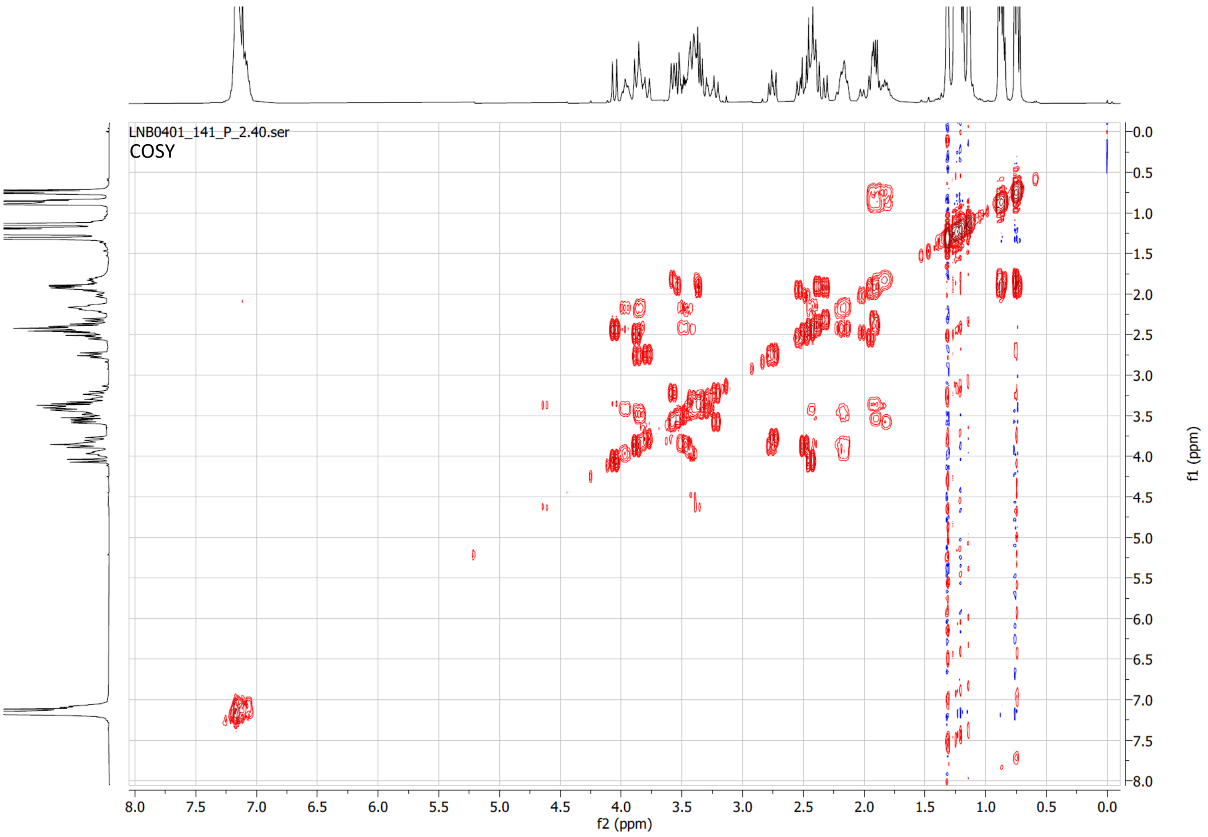

LNB0401_141_P_2.40.ser
COSY
f2 (ppm)
f1 (ppm)

***tert*-butyl (4*aS*,8*aS*)-4'-benzylhexahydrospiro[benzo[b][1,4]oxazine-2,2'-morpholine]-4(3*H*)-carboxylate (52) [^{1}H-NMR data: 400 MHz, $CDCl_3$; $^{13}C\{^{1}H\}$-NMR data: 101 MHz, $CDCl_3$; 2D NMR spectra: HSQC, HMBC, COSY, all in $CDCl_3$]:**

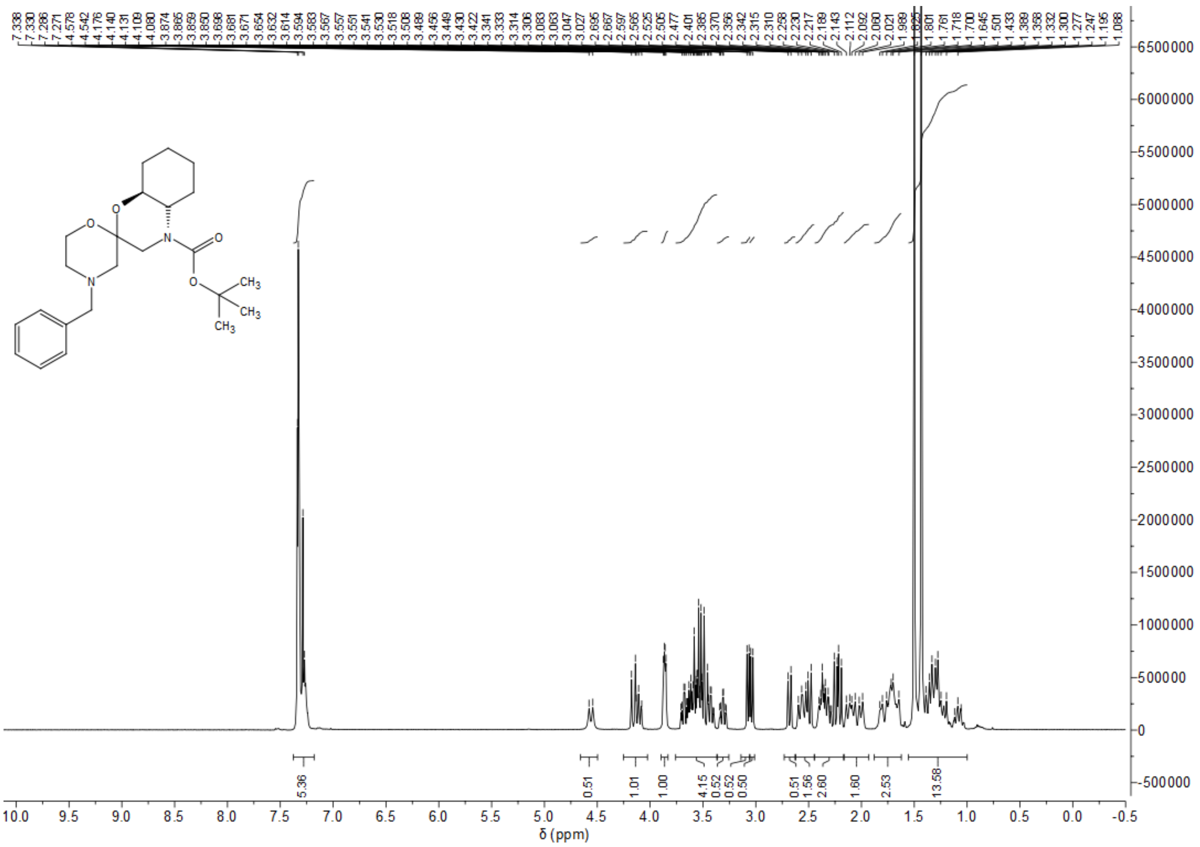

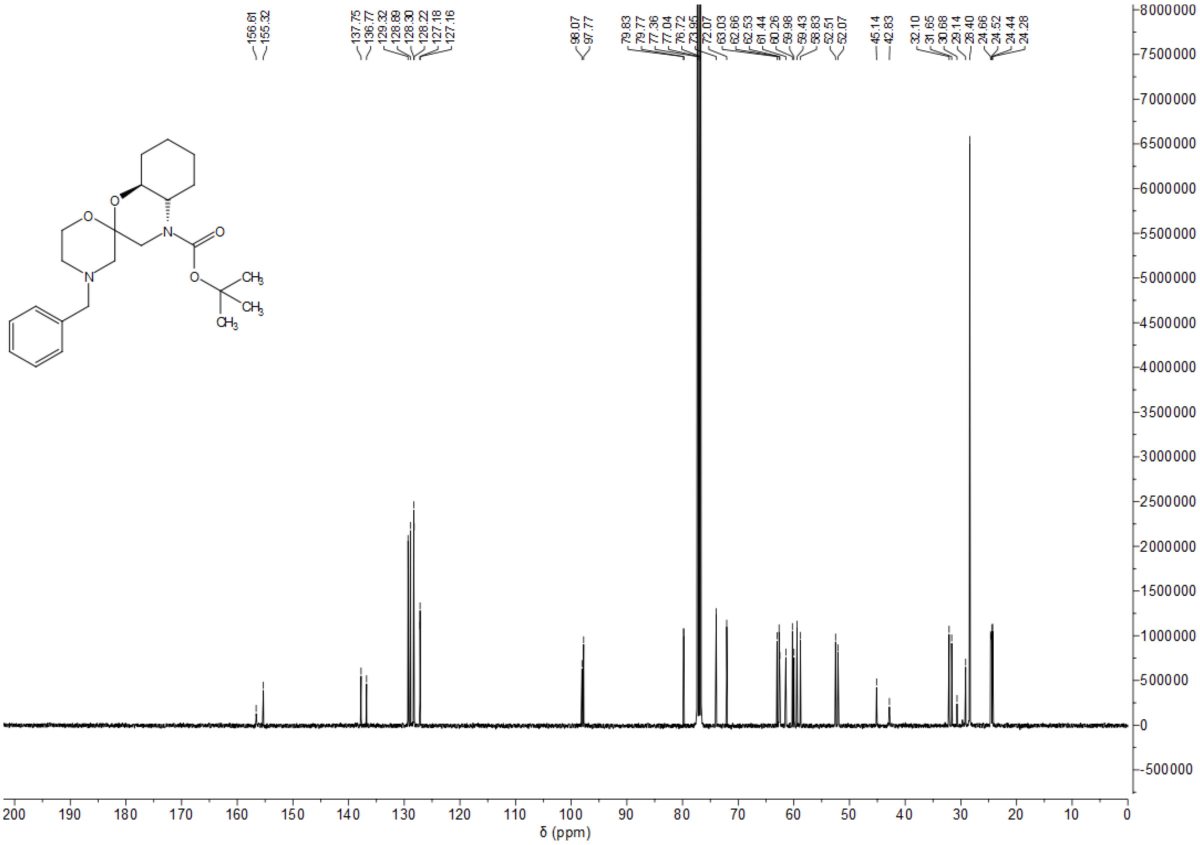

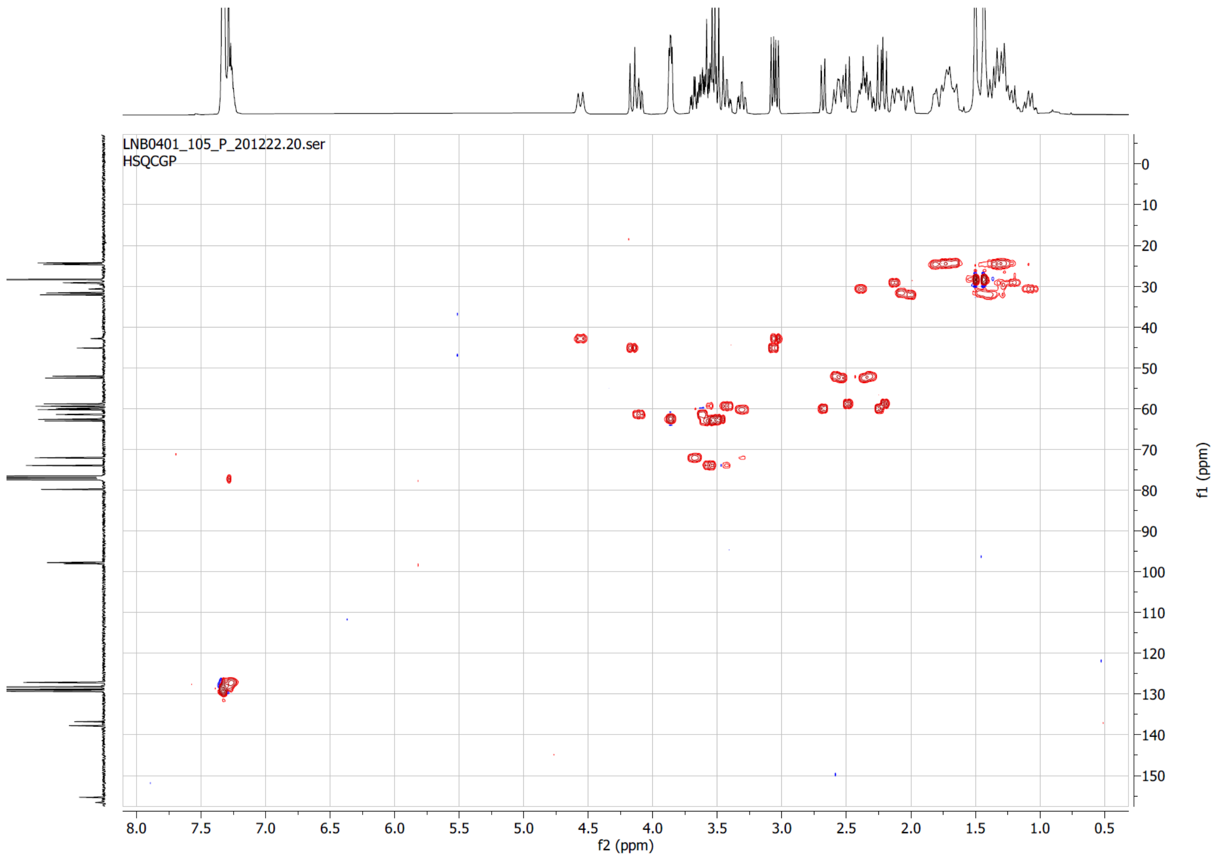
LNB0401_105_P_201222.20.ser
HSQCGP
f2 (ppm)
f1 (ppm)

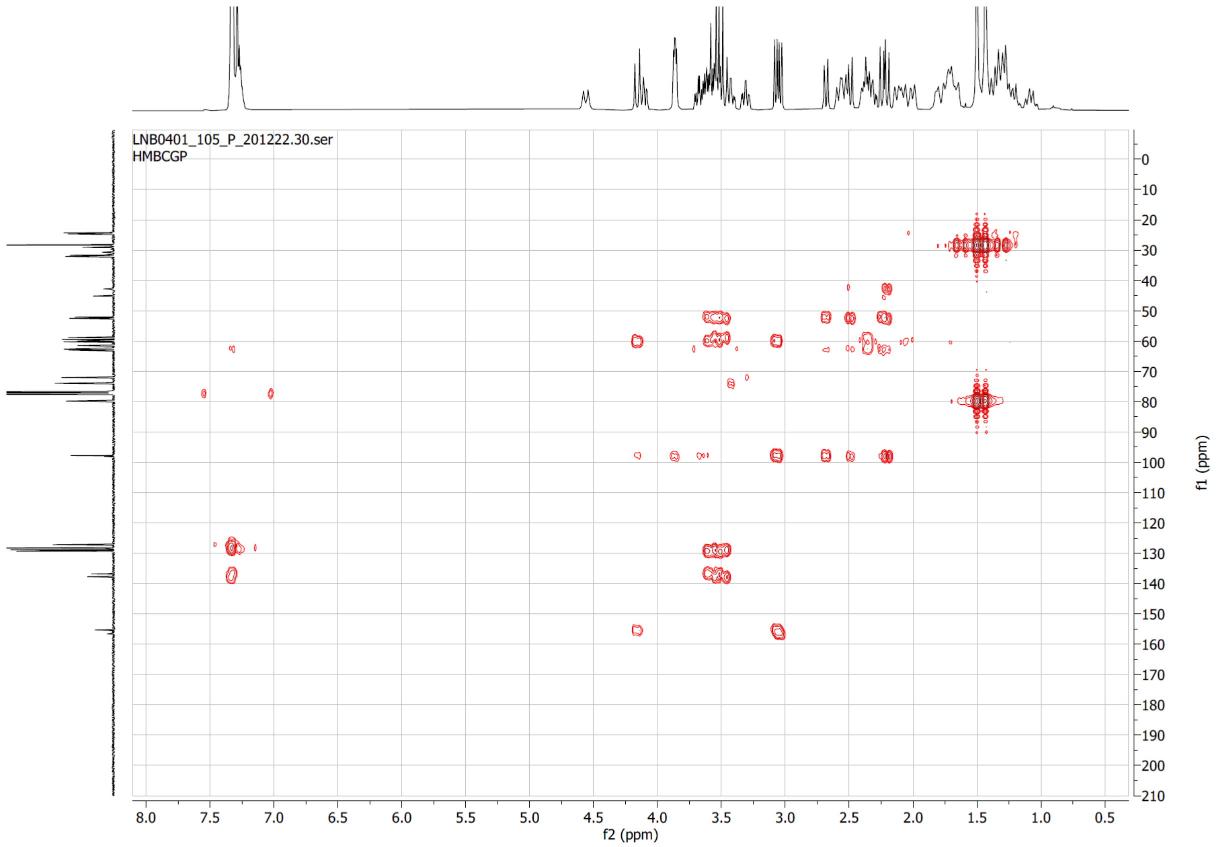
LNB0401_105_P_201222.30.ser
HMBCGP
f2 (ppm)
f1 (ppm)

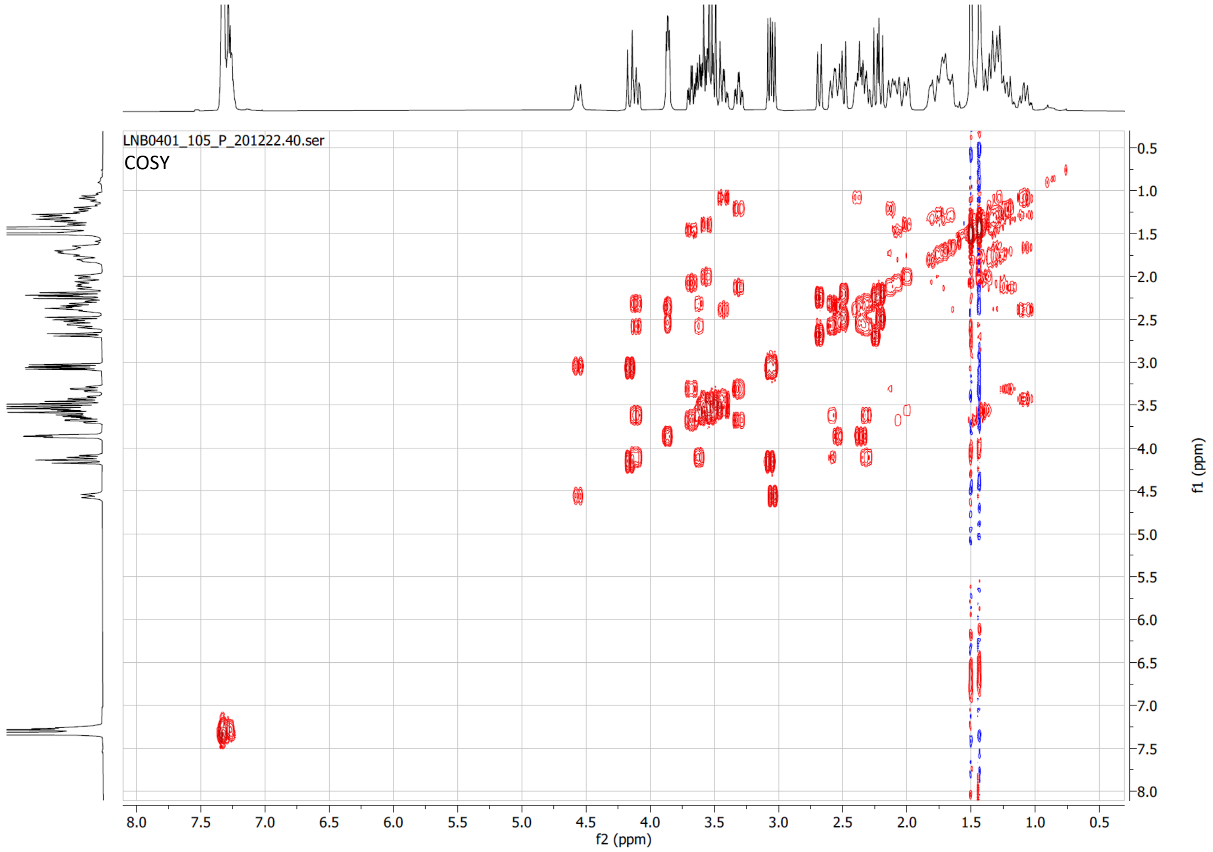
LNB0401_105_P_201222.40.ser
COSY
f2 (ppm)
f1 (ppm)

***tert*-butyl 4'-benzylspiro[benzo[b][1,4]oxazine-2,2'-morpholine]-4(3*H*)-carboxylate (53) [^{1}H-NMR data: 400 MHz, $CDCl_3$; ^{13}C{^{1}H}-NMR data: 101 MHz, $CDCl_3$; 2D NMR spectra: HSQC, HMBC, COSY, all in $CDCl_3$]:**

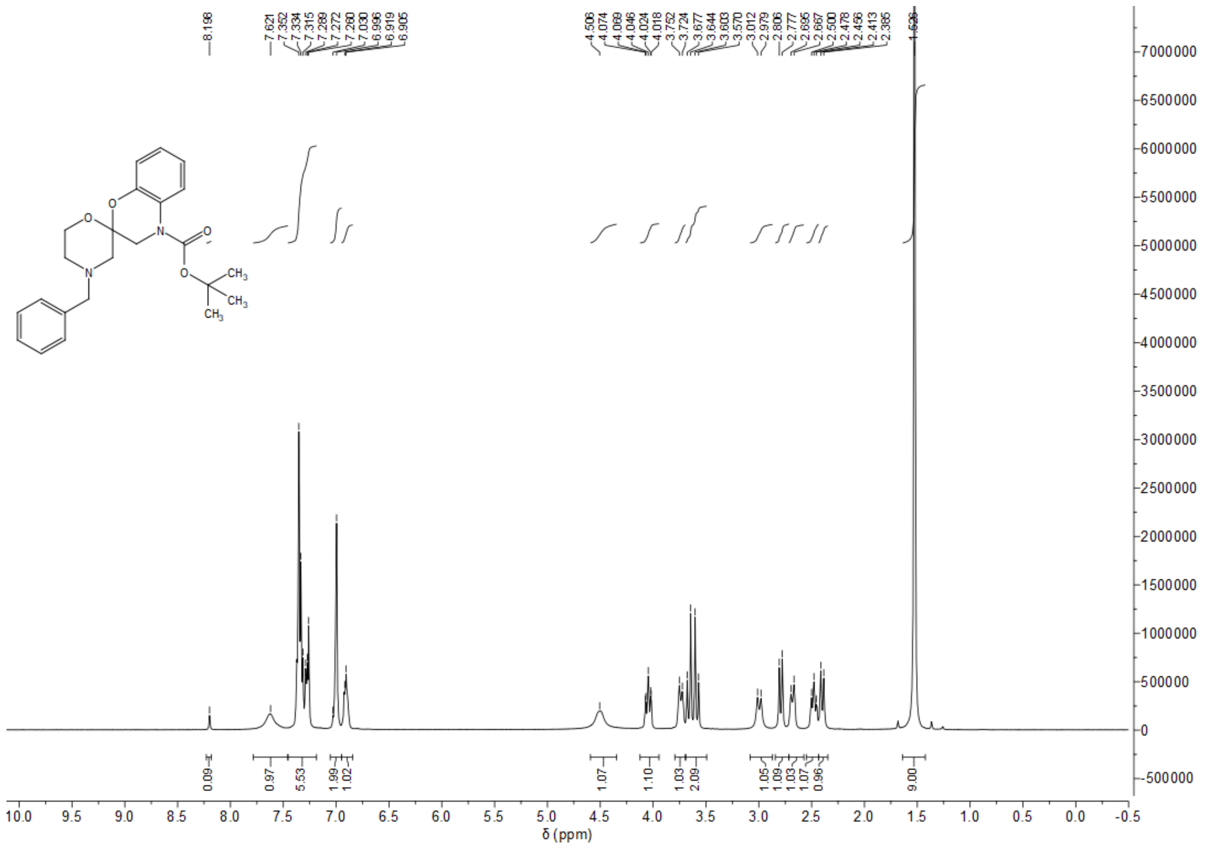

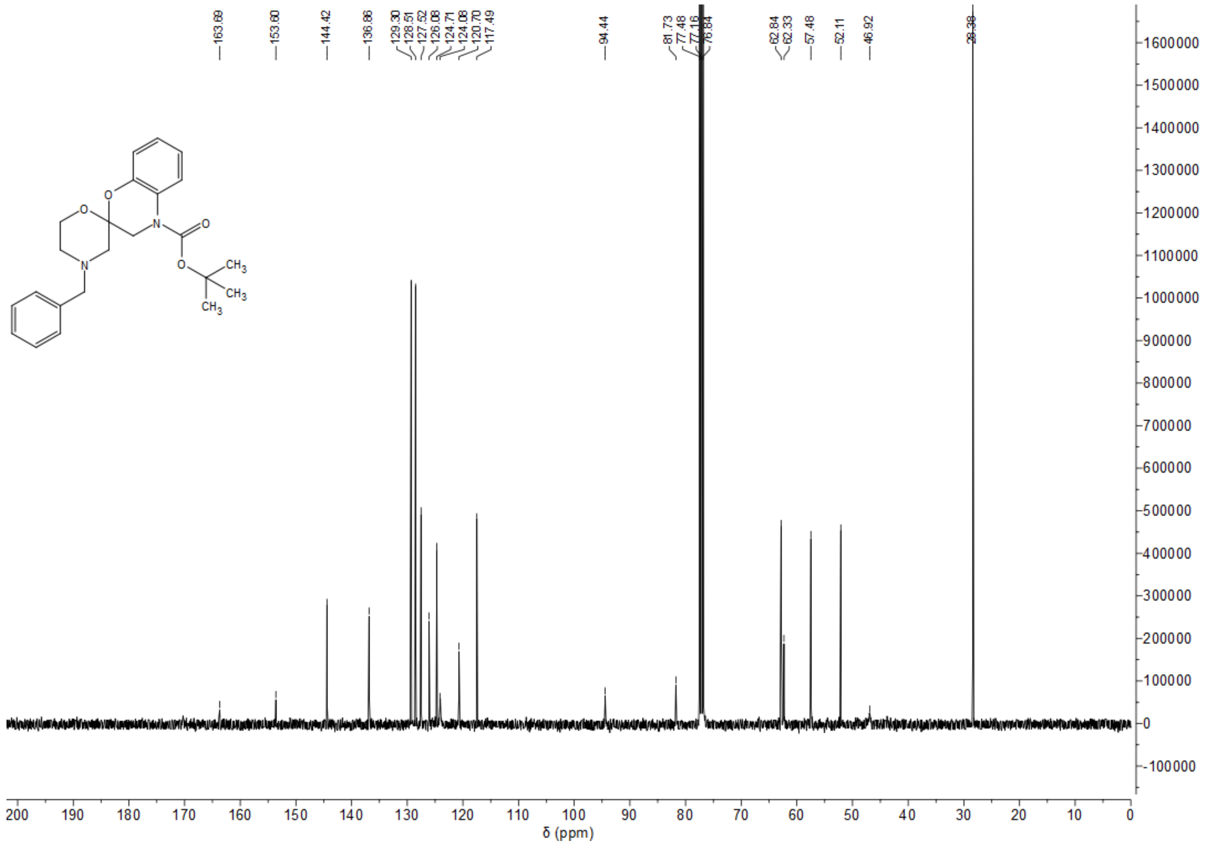

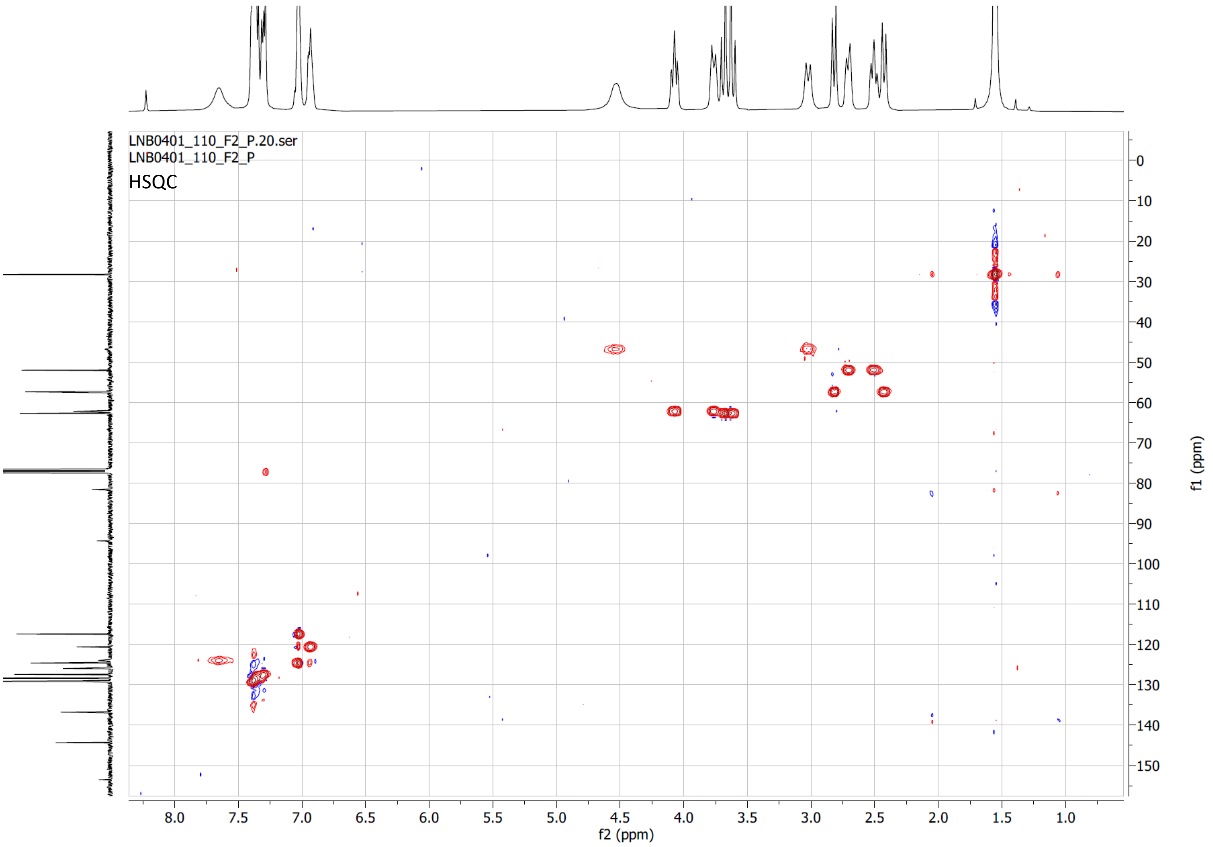
LNB0401_110_F2_P.20.ser
LNB0401_110_F2_P
HSQC
f2 (ppm)
f1 (ppm)

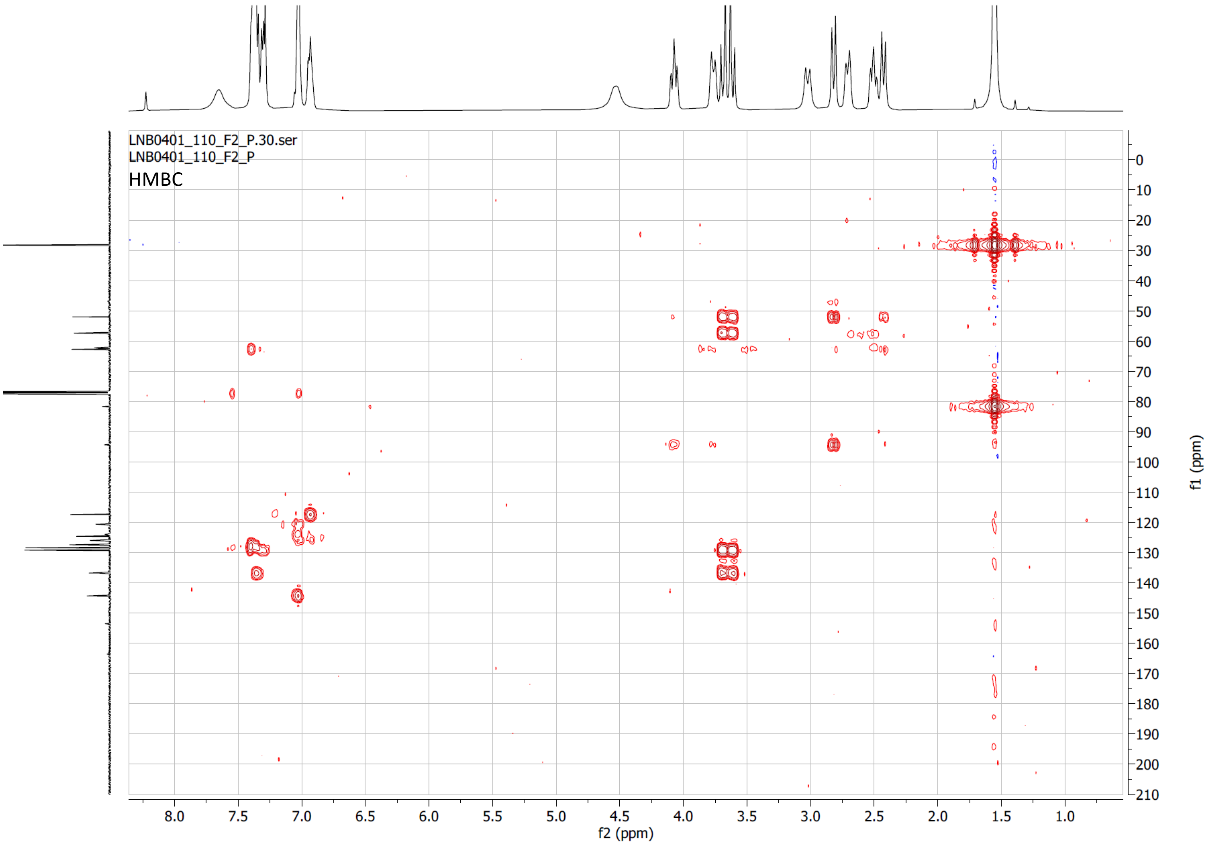
LNB0401_110_F2_P.30.ser
LNB0401_110_F2_P
HMBC
f2 (ppm)
f1 (ppm)

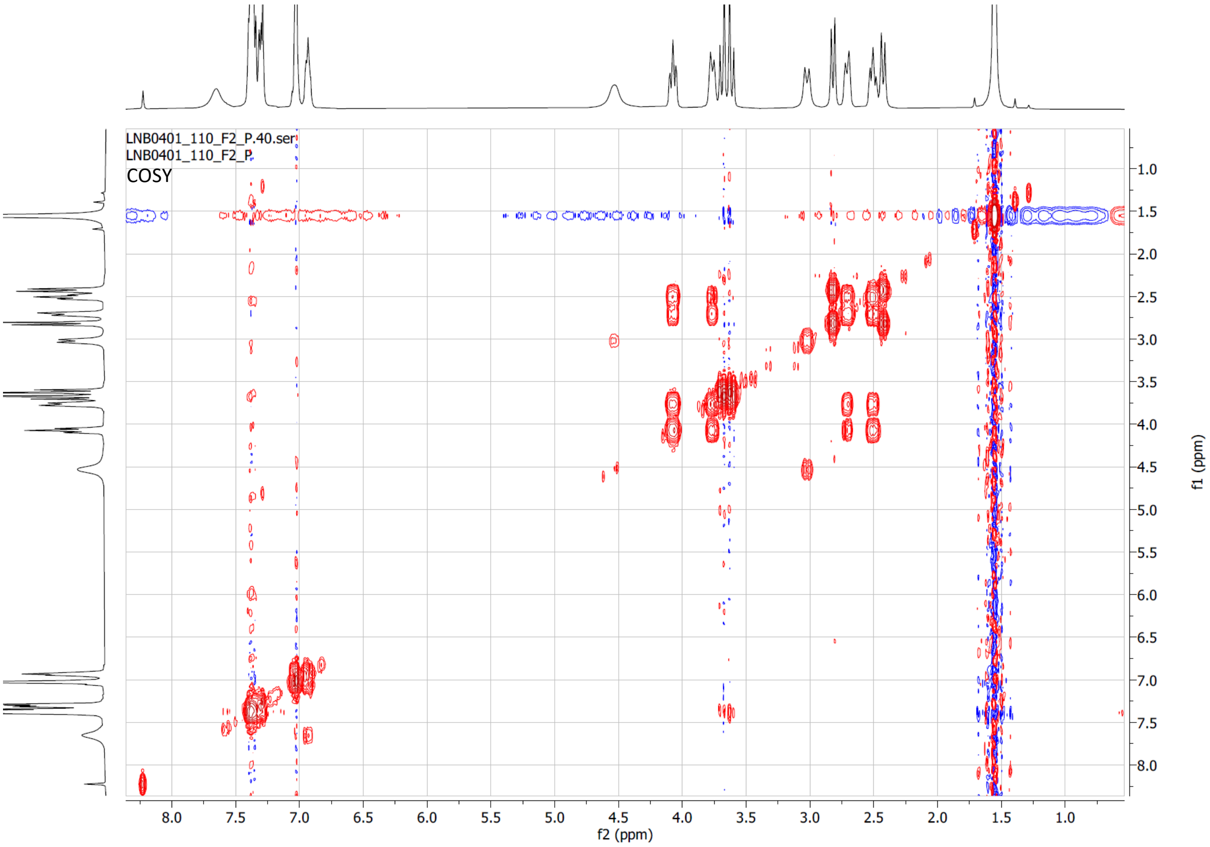
LNB0401_110_F2_P.40.ser
LNB0401_110_F2_P
COSY
f2 (ppm)
f1 (ppm)

***tert*-butyl (8*S*)-10-benzyl-8-methyl-1,7-dioxa-4,10-diazaspiro[5.5]undecane-4-carboxylate (43) [^{1}H-NMR data: 400 MHz, $CDCl_3$; $^{13}C\{^{1}H\}$-NMR data: 101 MHz, $CDCl_3$; 2D NMR spectra: HSQC, HMBC, COSY, all in $CDCl_3$]:**

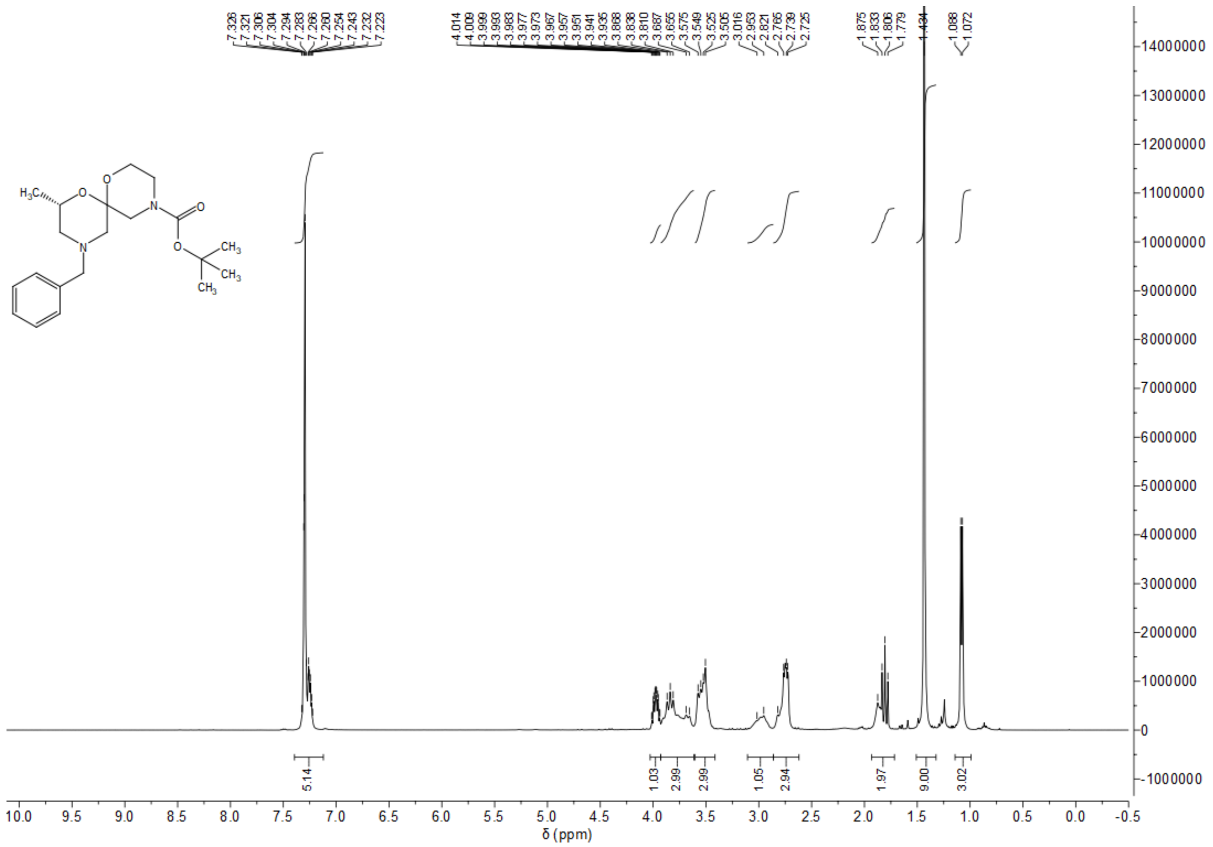

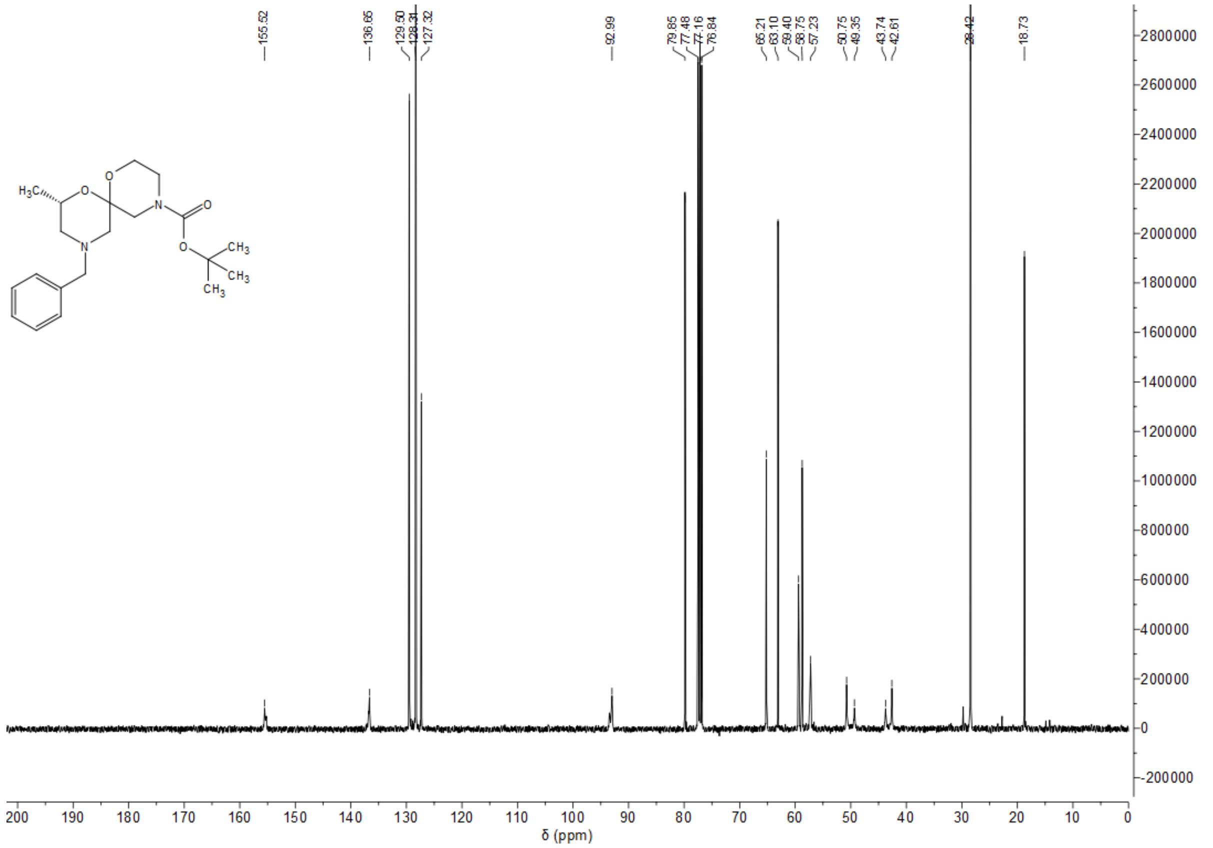

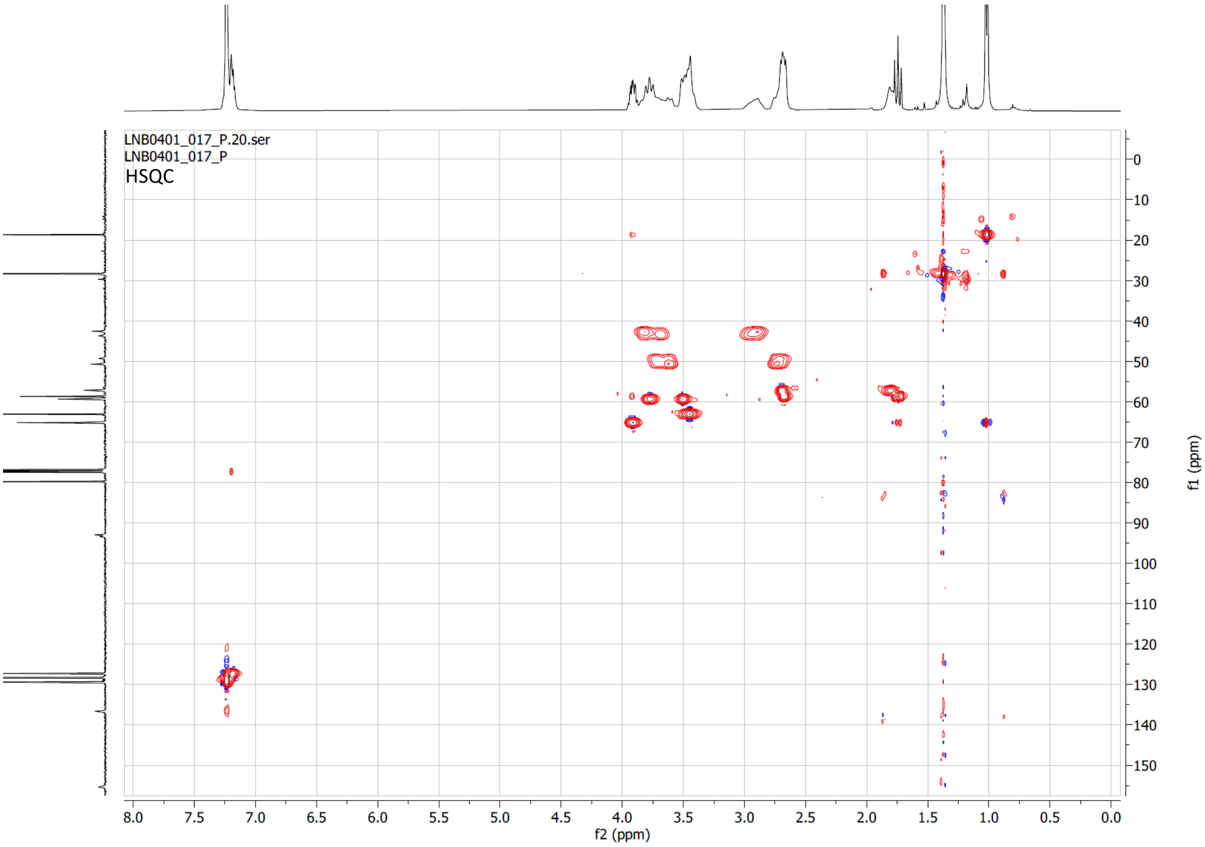
LNB0401_017_P.20.ser
LNB0401_017_P
HSQC
f2 (ppm)
f1 (ppm)

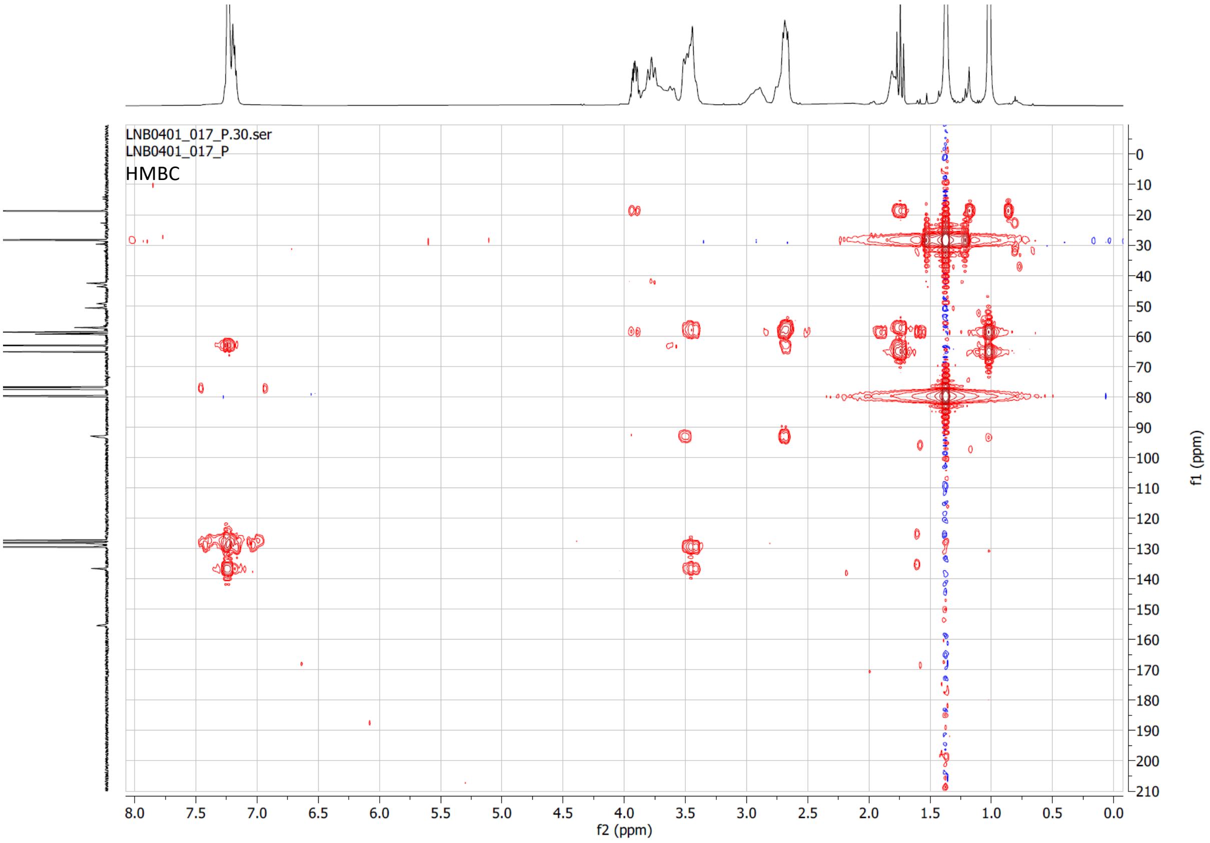
LNB0401_017_P.30.ser
LNB0401_017_P
HMBC
f2 (ppm)
f1 (ppm)

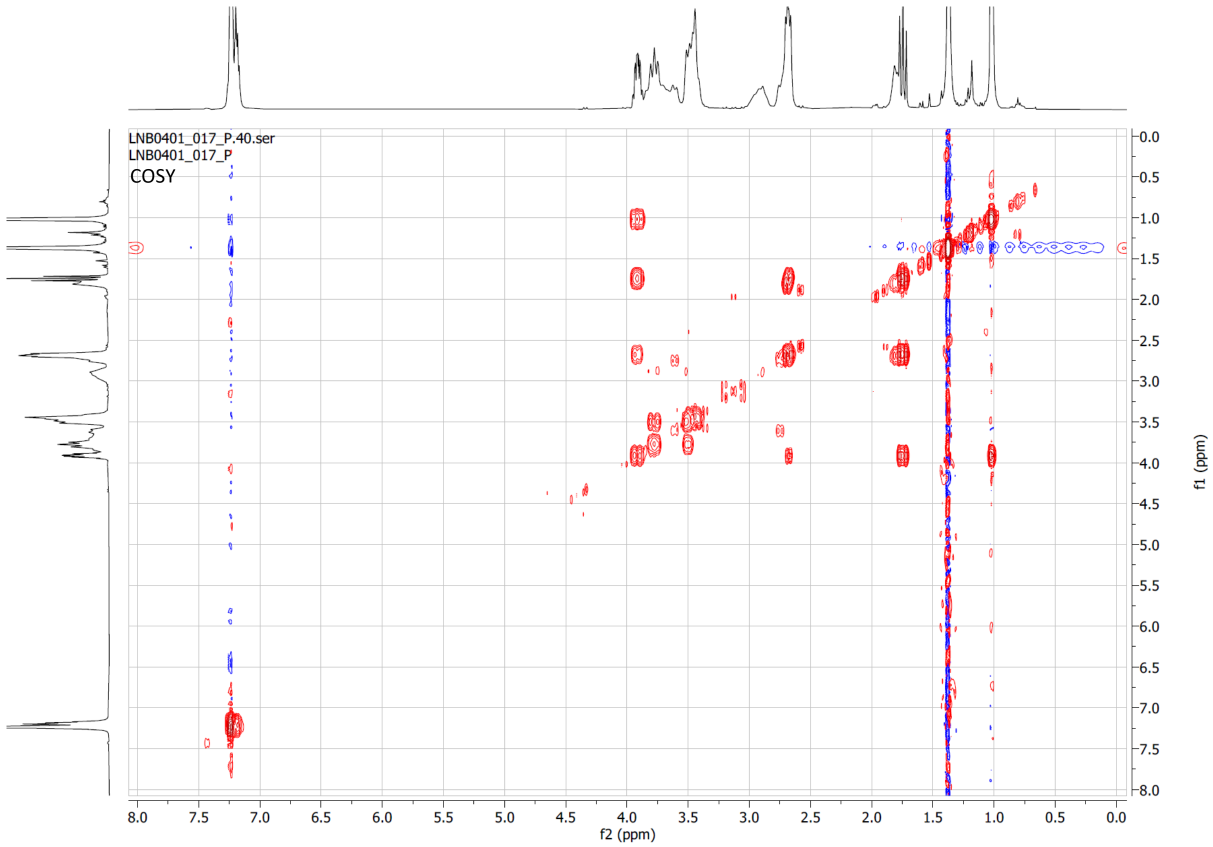
LNB0401_017_P.40.ser
LNB0401_017_P
COSY
f2 (ppm)
f1 (ppm)

***tert*-butyl (9*S*)-10-benzyl-9-methyl-1,7-dioxa-4,10-diazaspiro[5.5]undecane-4-carboxylate (42)** **[^{1}H-NMR data: 400 MHz, $CDCl_3$; $^{13}C\{^{1}H\}$-NMR data: 101 MHz, $CDCl_3$; 2D NMR spectra: HSQC, HMBC, COSY, all in $CDCl_3$]: Diasteromer A**

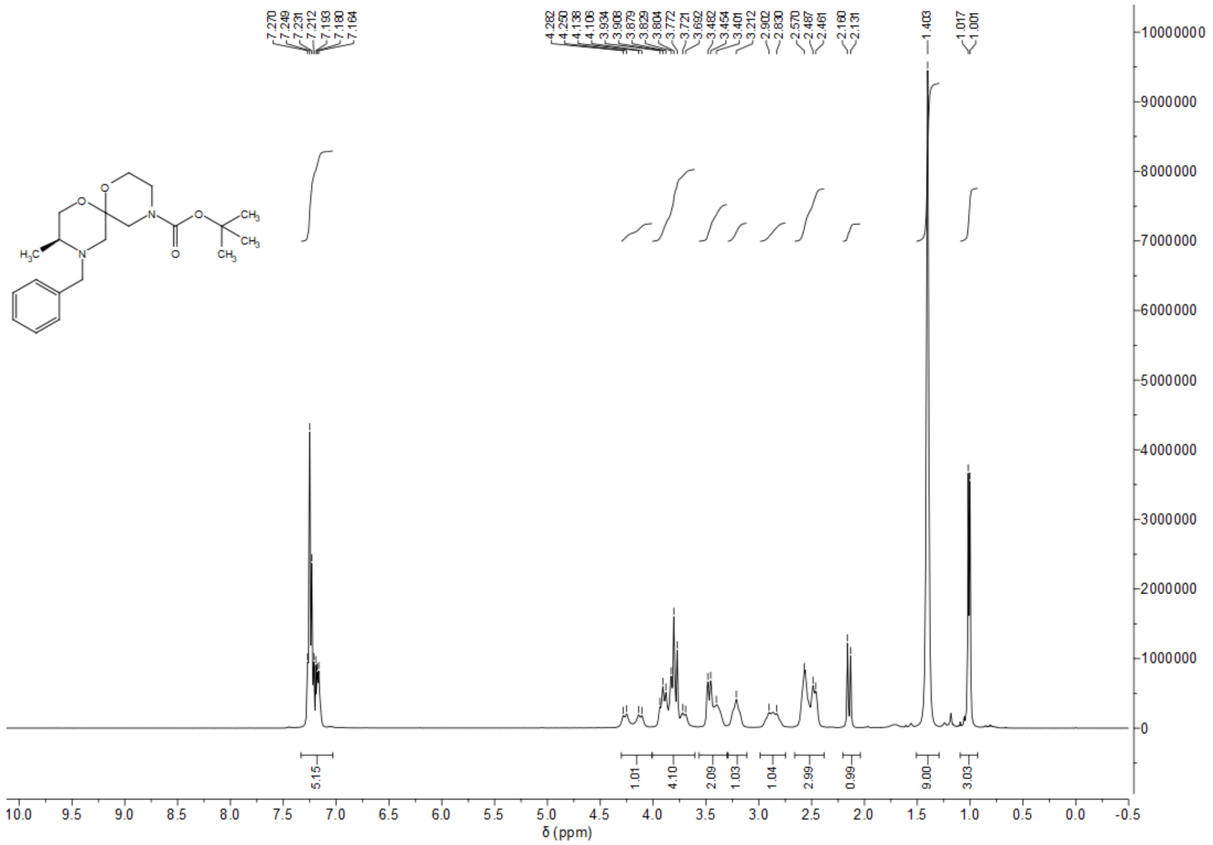

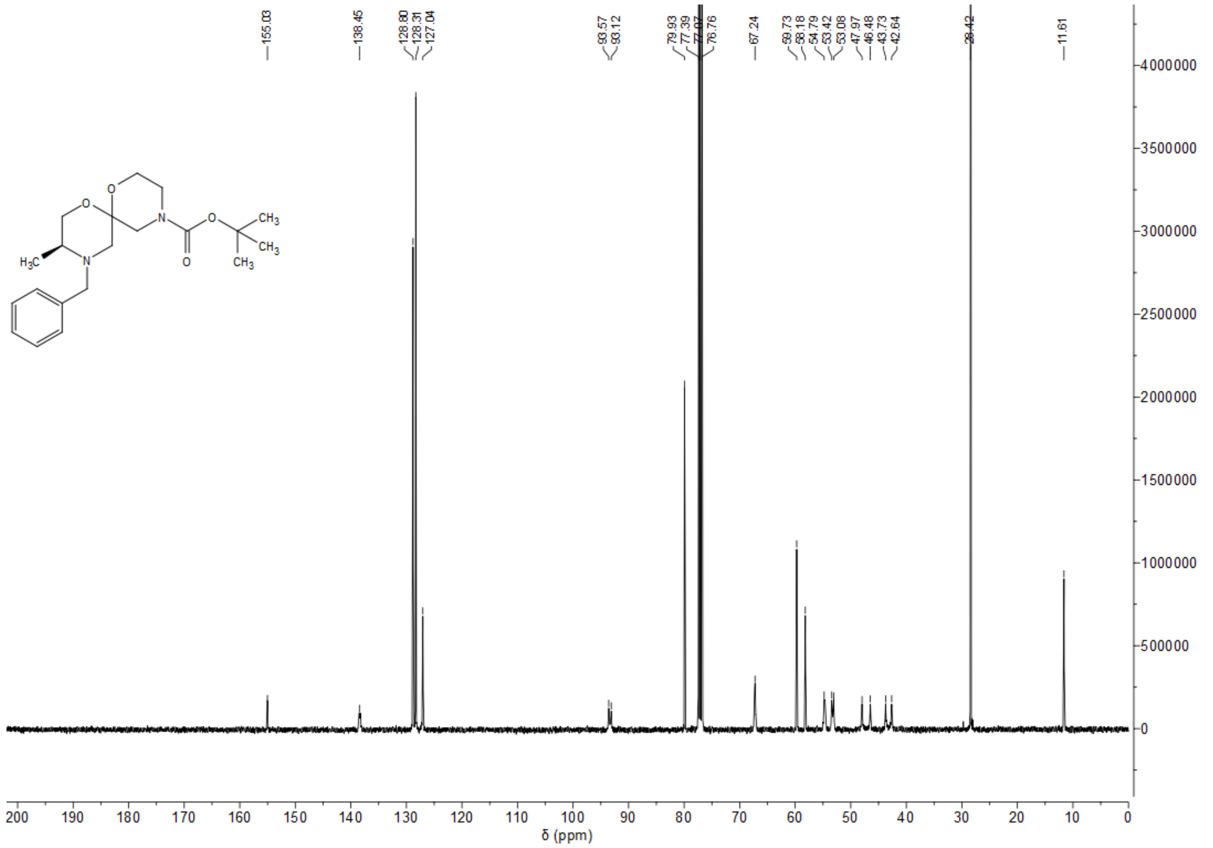

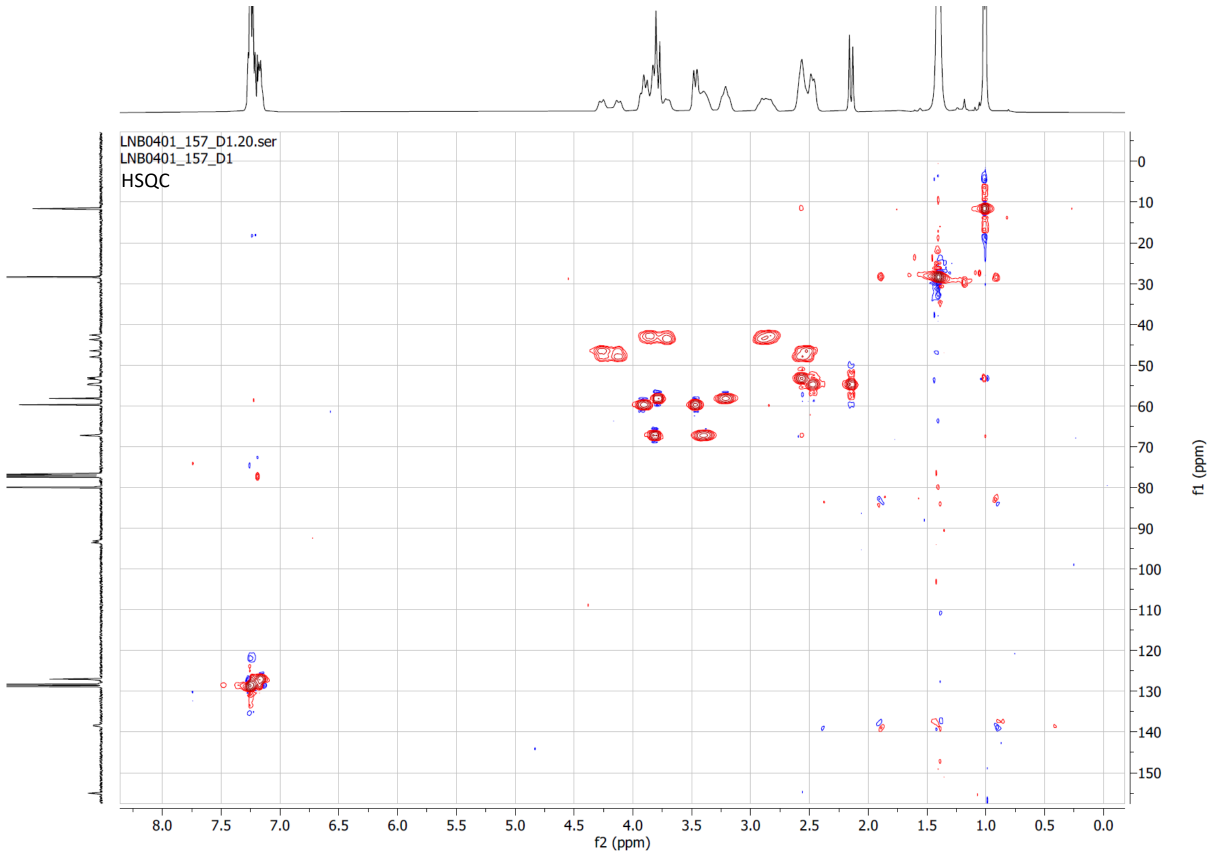
LNB0401_157_D1.20.ser
LNB0401_157_D1
HSQC
f2 (ppm)
f1 (ppm)

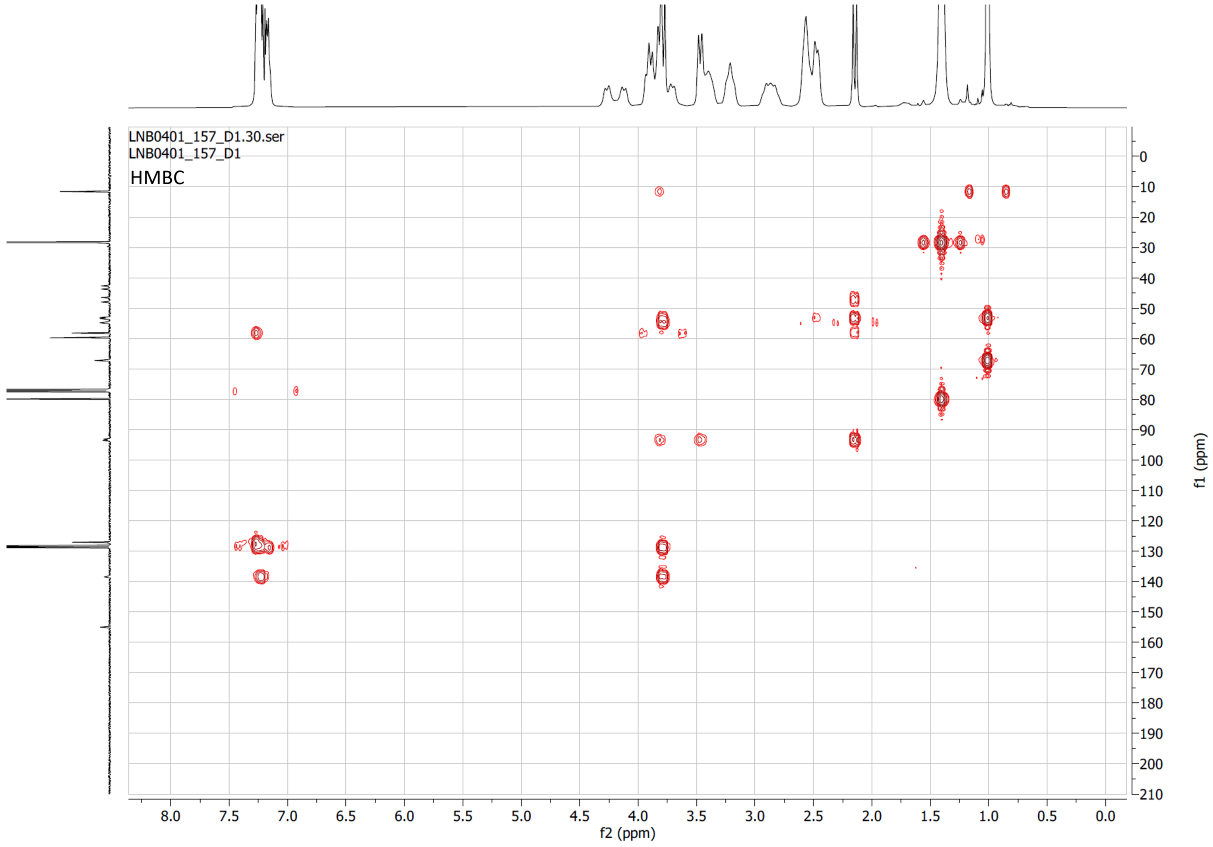
LNB0401_157_D1.30.ser
LNB0401_157_D1
HMBC
f2 (ppm)
f1 (ppm)

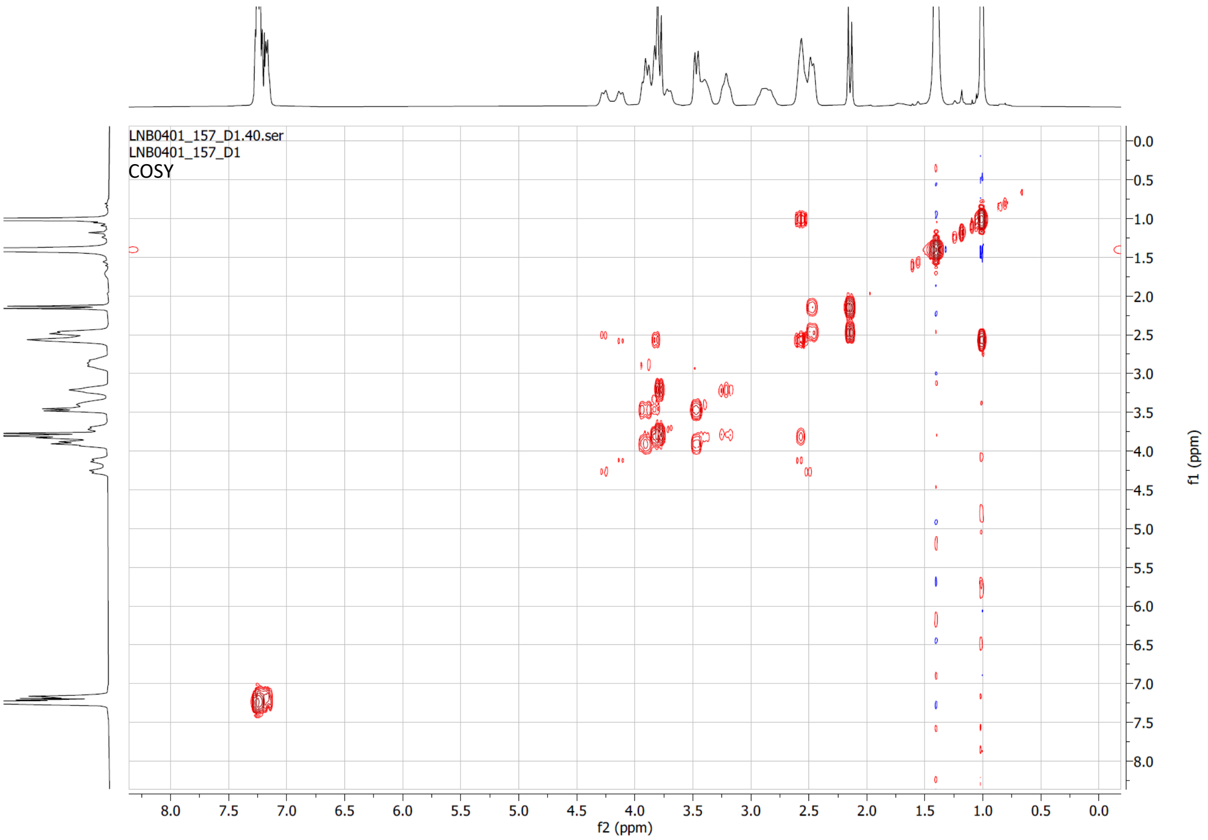

LNB0401_157_D1.40.ser
LNB0401_157_D1
COSY
f2 (ppm)
f1 (ppm)

***tert*-butyl (9*S*)-10-benzyl-9-methyl-1,7-dioxa-4,10-diazaspiro[5.5]undecane-4-carboxylate (42) [^{1}H-NMR data: 400 MHz, $CDCl_3$; $^{13}C\{^{1}H\}$-NMR data: 101 MHz, $CDCl_3$; 2D NMR spectra: HSQC, HMBC, COSY, all in $CDCl_3$]: Diastereomer B**

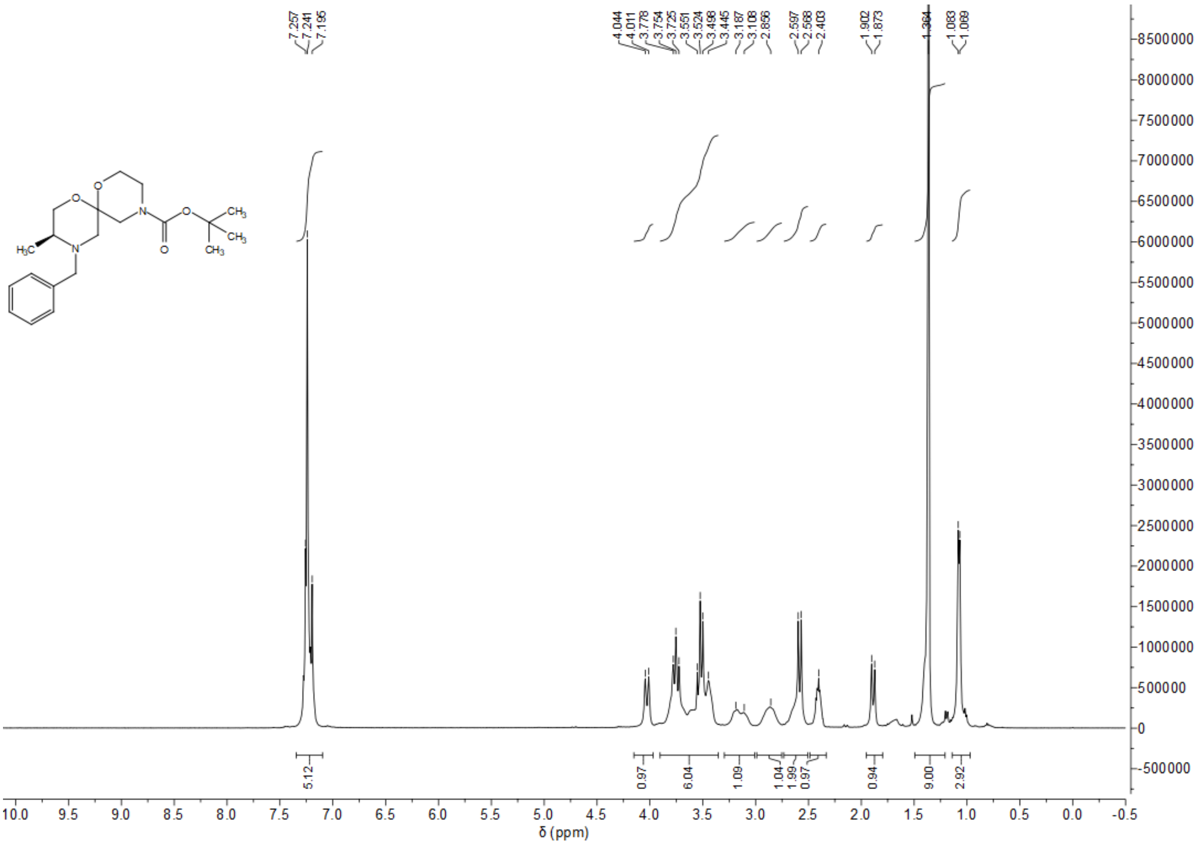

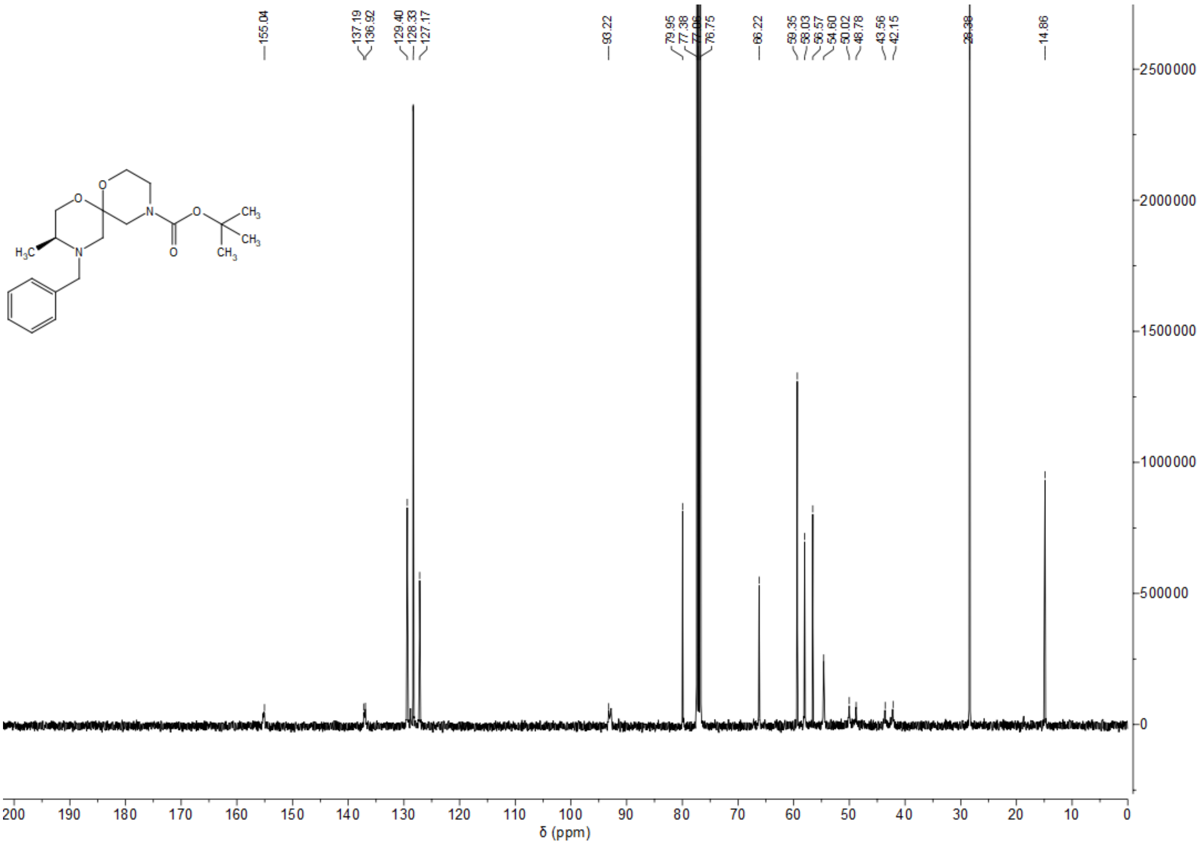

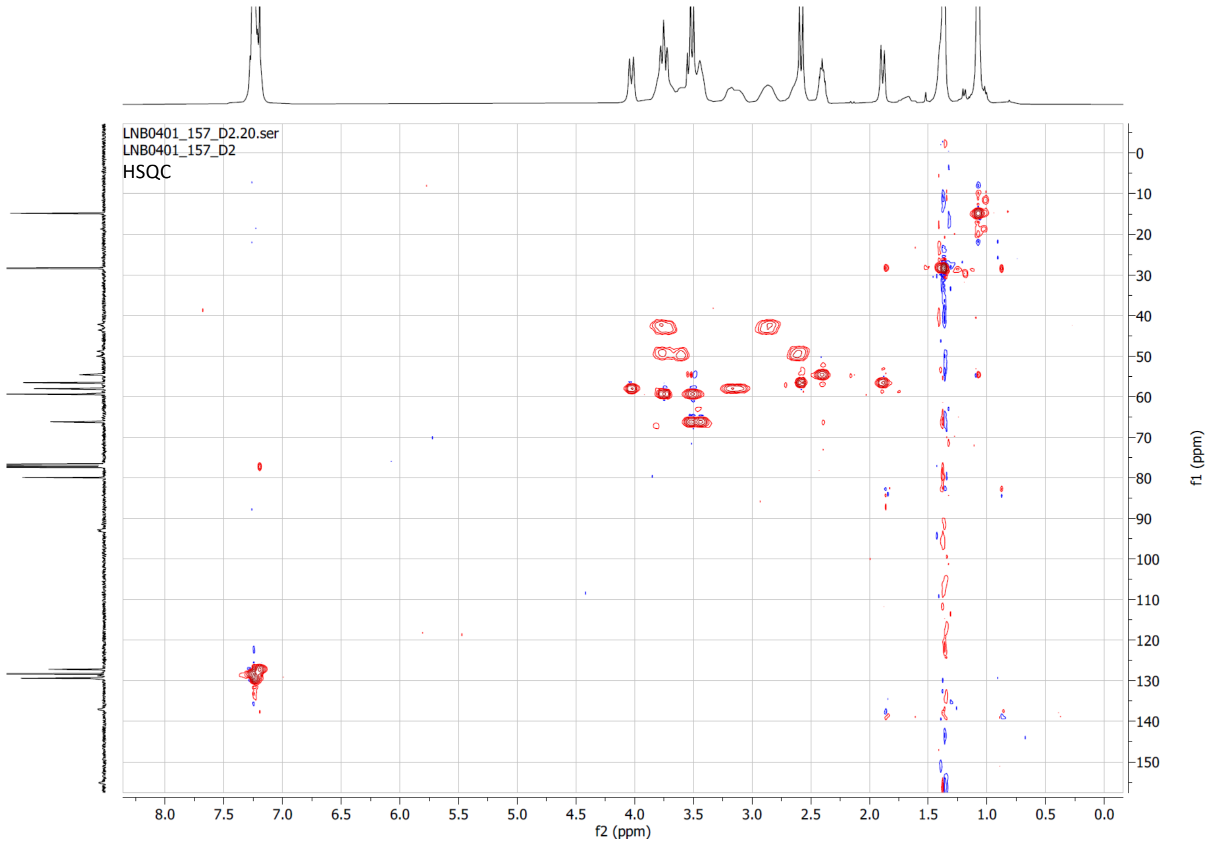
LNB0401_157_D2.20.ser
LNB0401_157_D2
HSQC
f2 (ppm)
f1 (ppm)

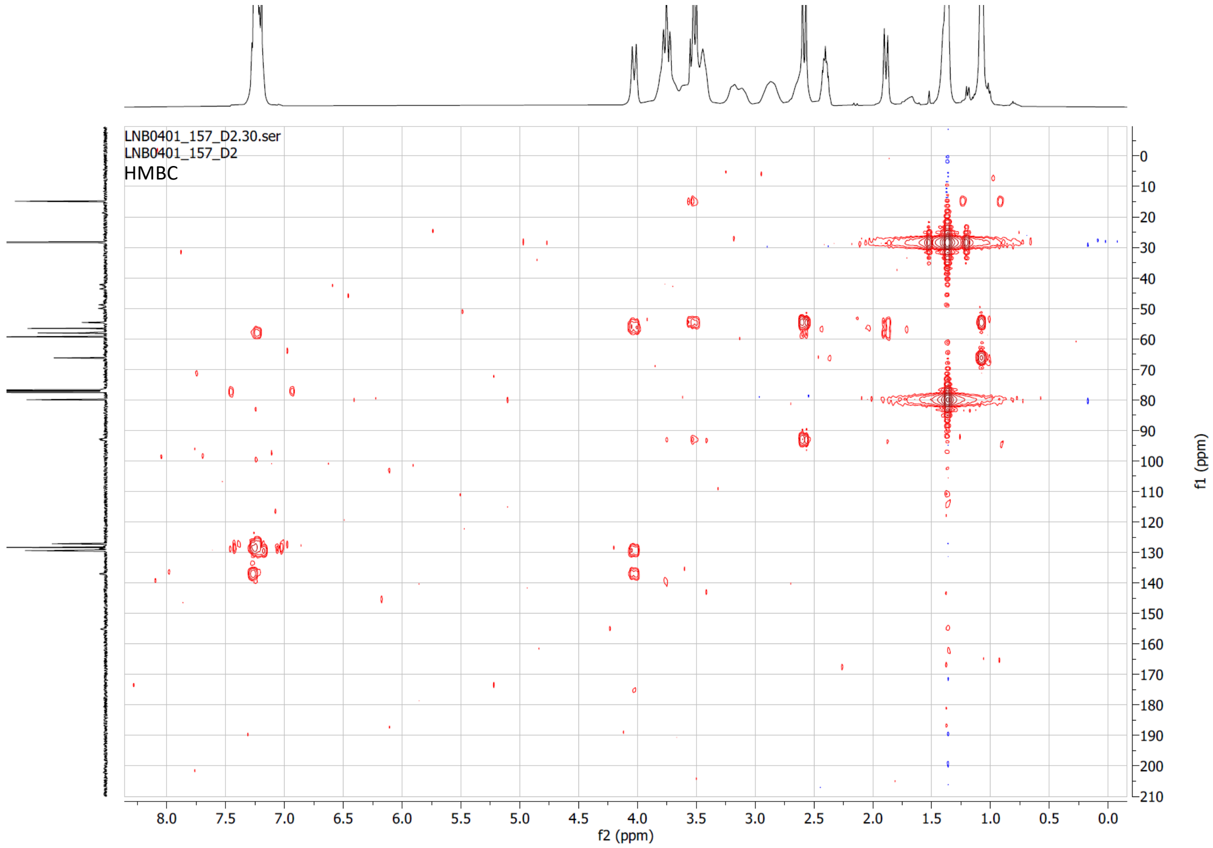
LNB0401_157_D2.30.ser
LNB0401_157_D2
HMBC
f2 (ppm)
f1 (ppm)

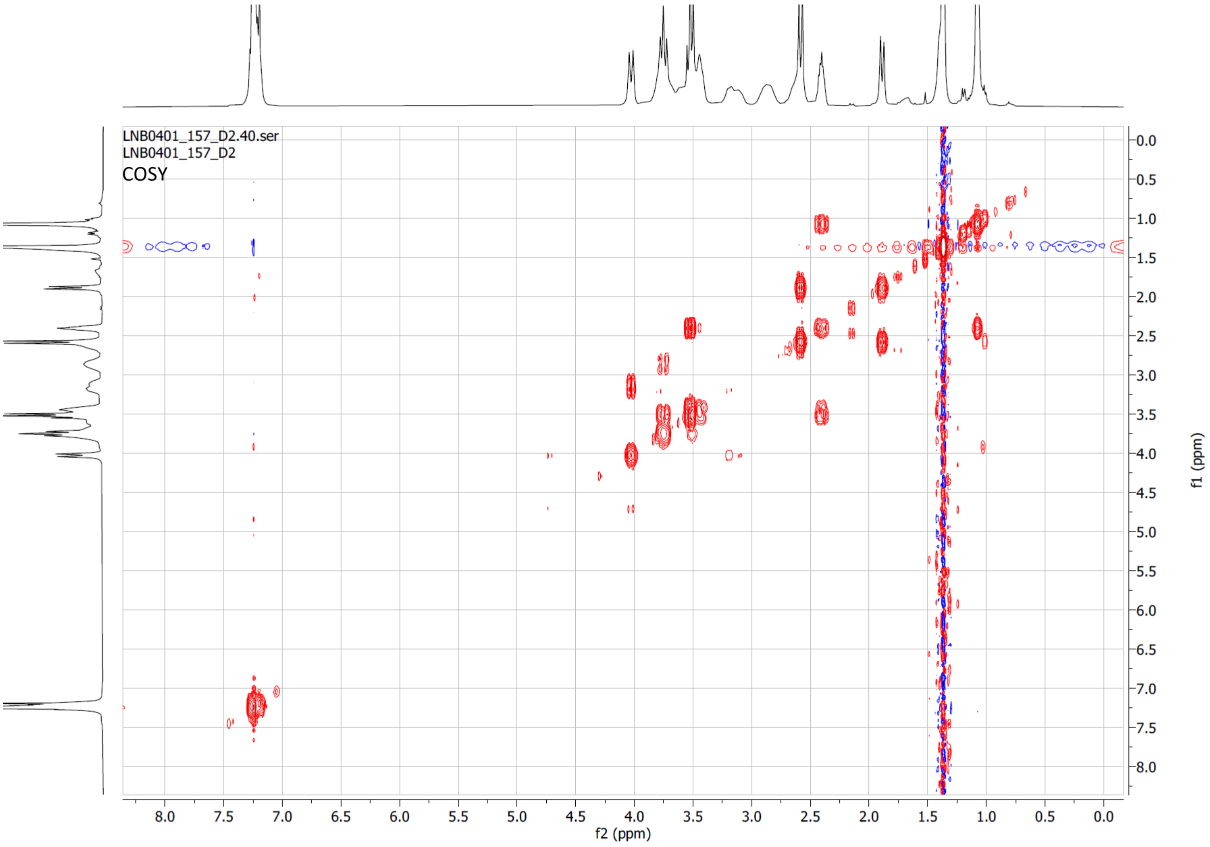

LNB0401_157_D2.40.ser
LNB0401_157_D2
COSY
f2 (ppm)
f1 (ppm)

***tert*-butyl (3*S*,9*S*)-3,10-dibenzyl-9-methyl-1,7-dioxa-4,10-diazaspiro[5.5]undecane-4-carboxylate (54) [^{1}H-NMR data: 400 MHz, $CDCl_3$; $^{13}C\{^{1}H\}$-NMR data: 101 MHz, $CDCl_3$; 2D NMR spectra: HSQC, HMBC, COSY, all in $CDCl_3$]:**

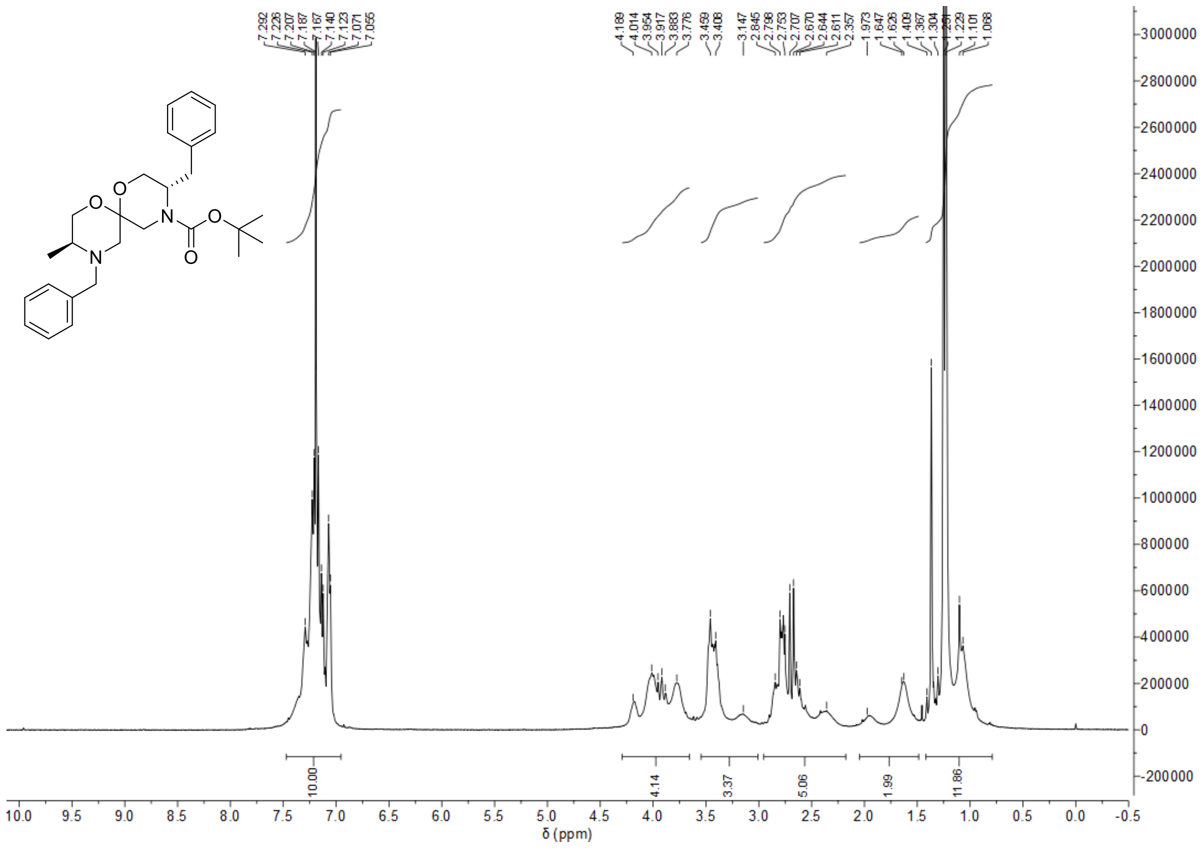

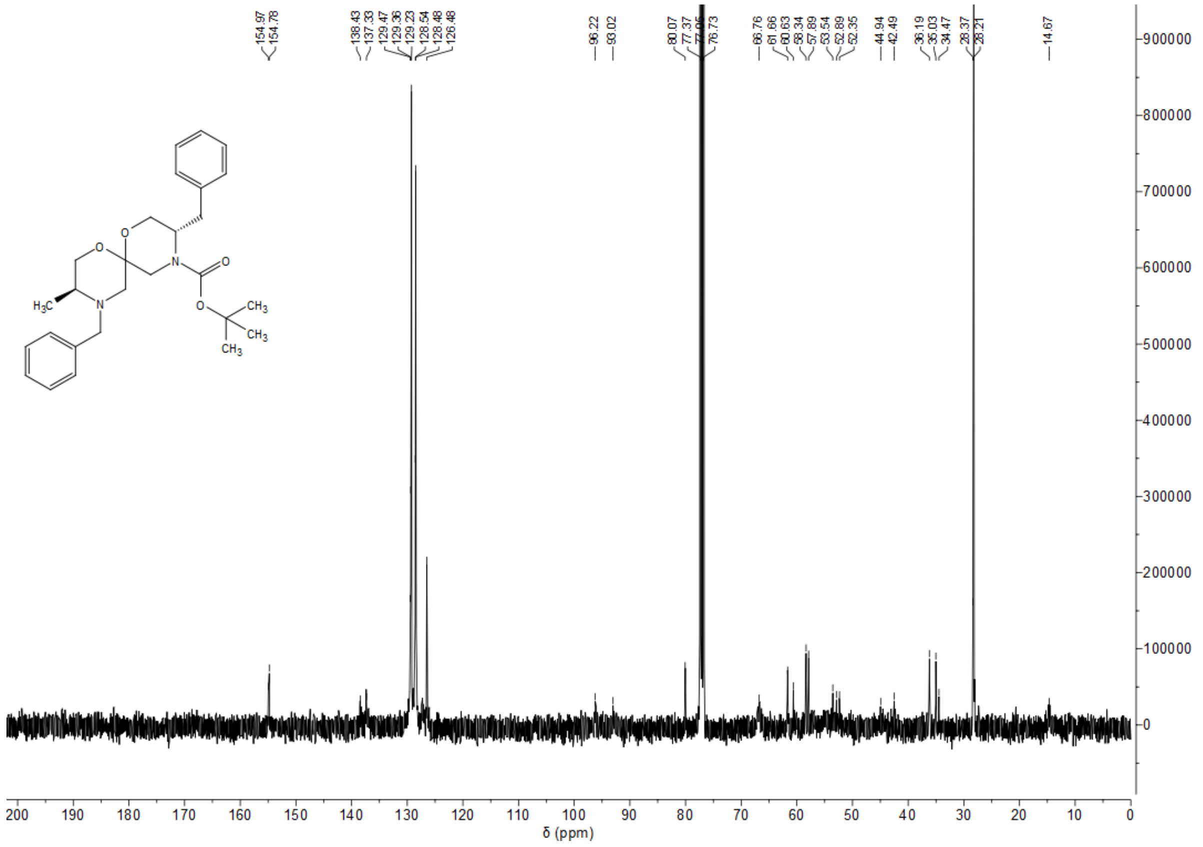

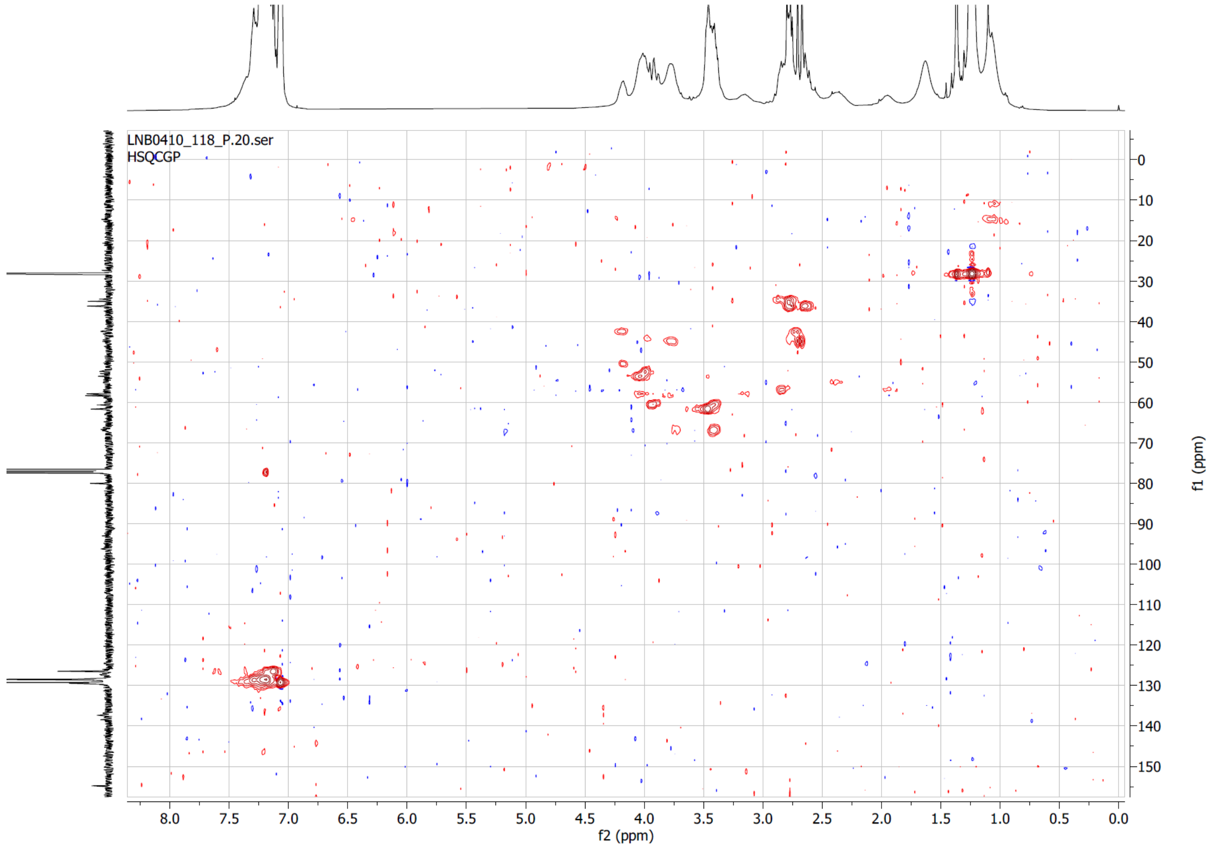
LNB0410_118_P.20.ser
HSQCGP
f2 (ppm)
f1 (ppm)

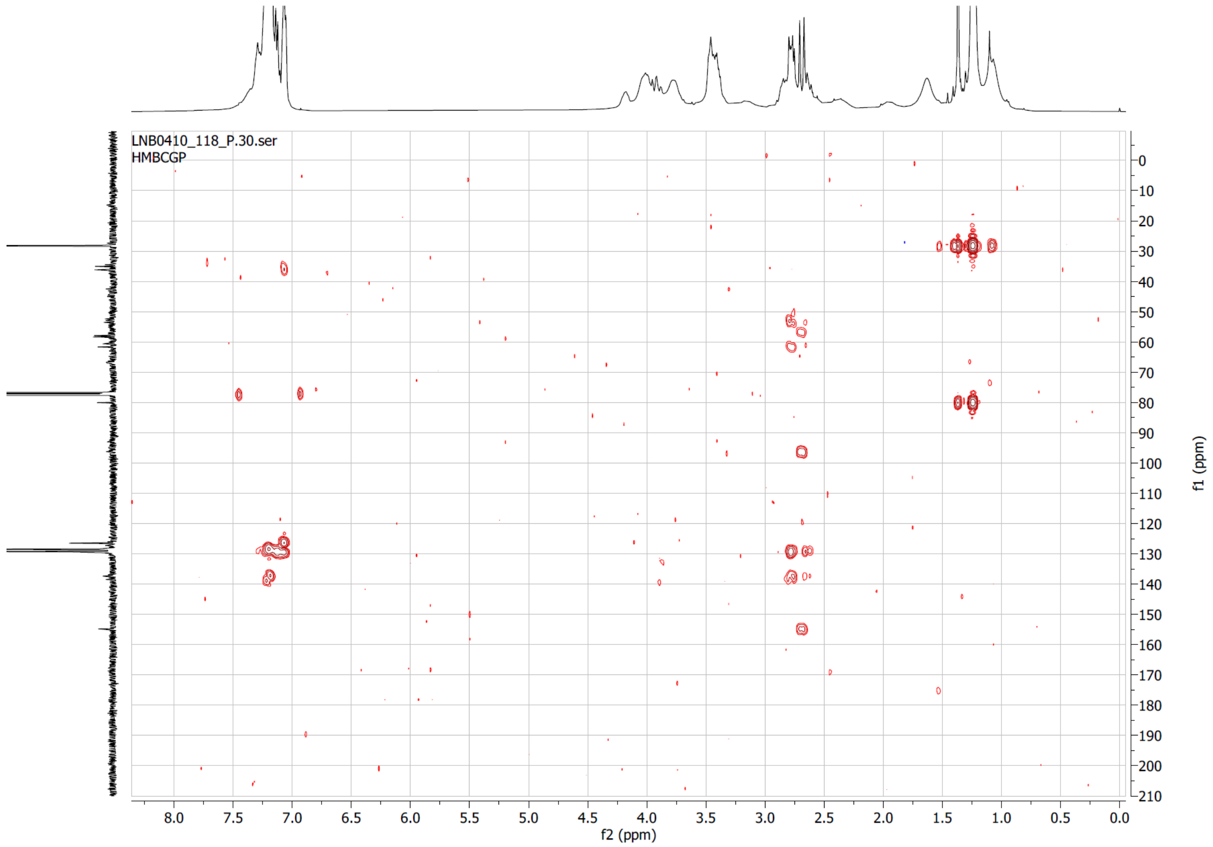
LNB0410_118_P.30.ser
HMBCGP
f2 (ppm)
f1 (ppm)

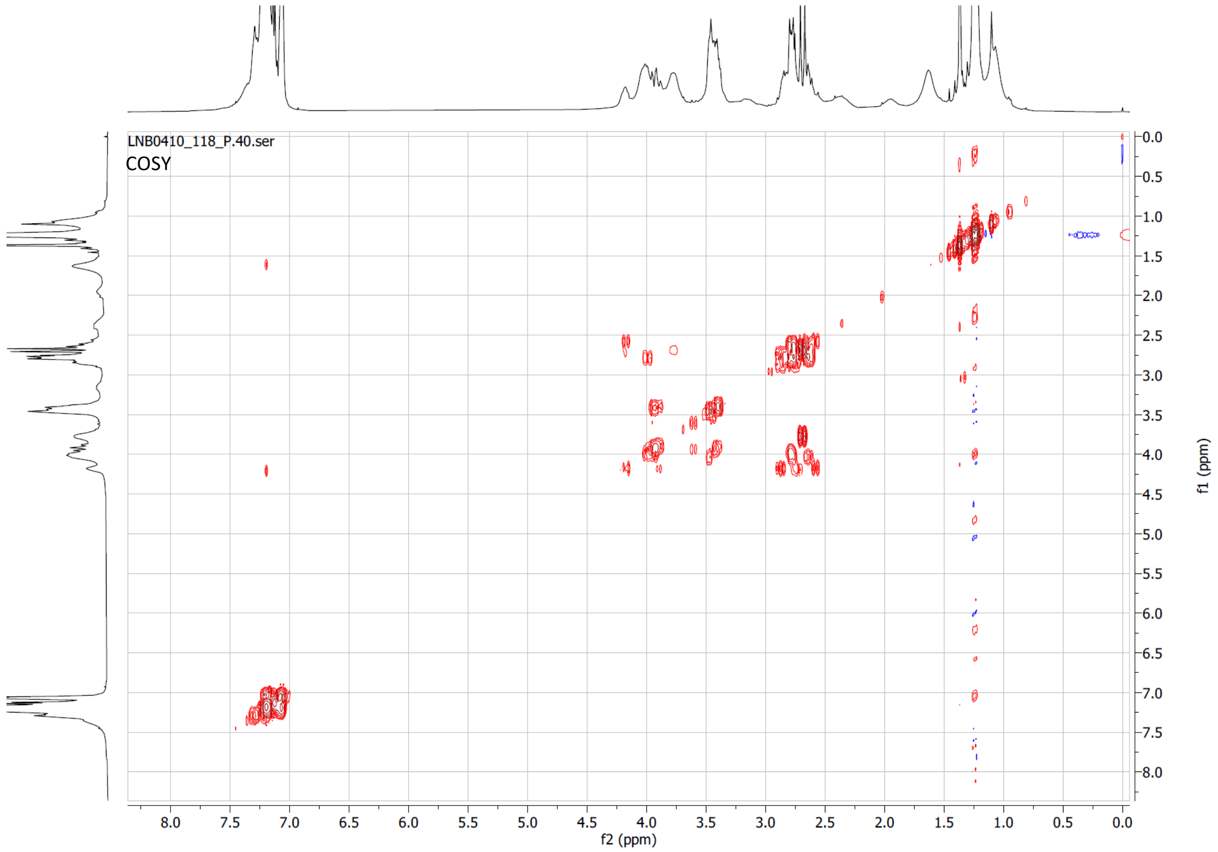

LNB0410_118_P.40.ser
COSY
f2 (ppm)
f1 (ppm)

(3*S*,9*S*)-4,9-dibenzyl-3-methyl-1,7-dioxa-4,10-diazaspiro[5.5]undecane (S38) [^{1}H-NMR data: 400 MHz, CD_3OD; $^{13}C\{^{1}H\}$-NMR data: 101 MHz, CD_3OD; 2D NMR spectra: HSQC, HMBC, COSY, all in CD_3OD]:

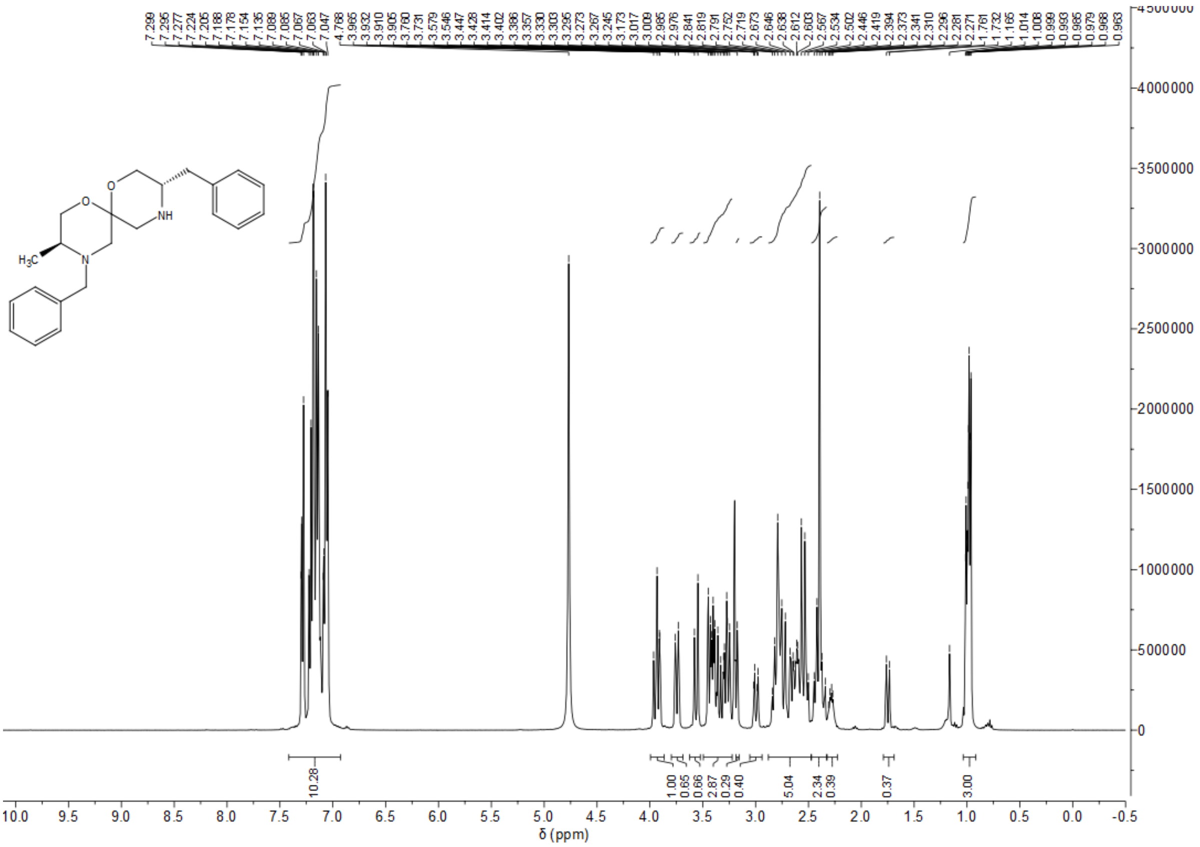

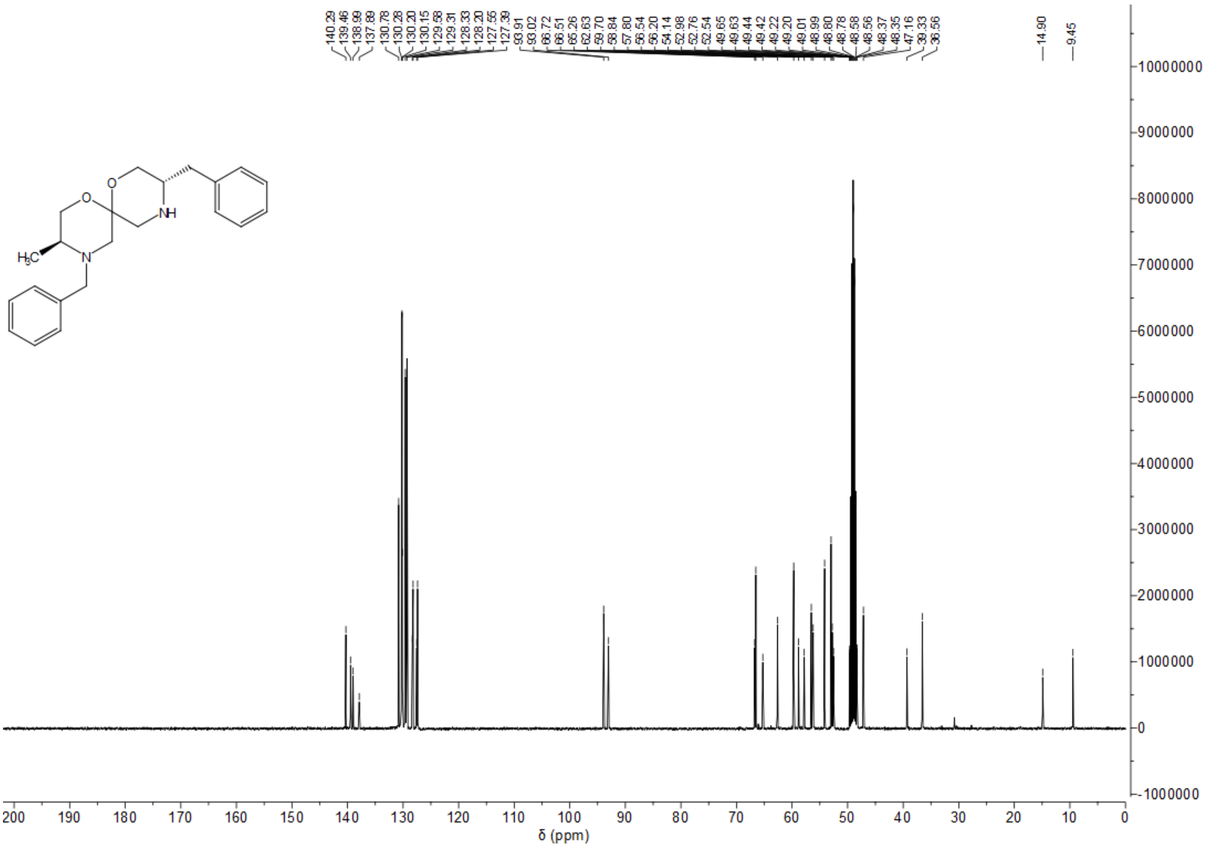

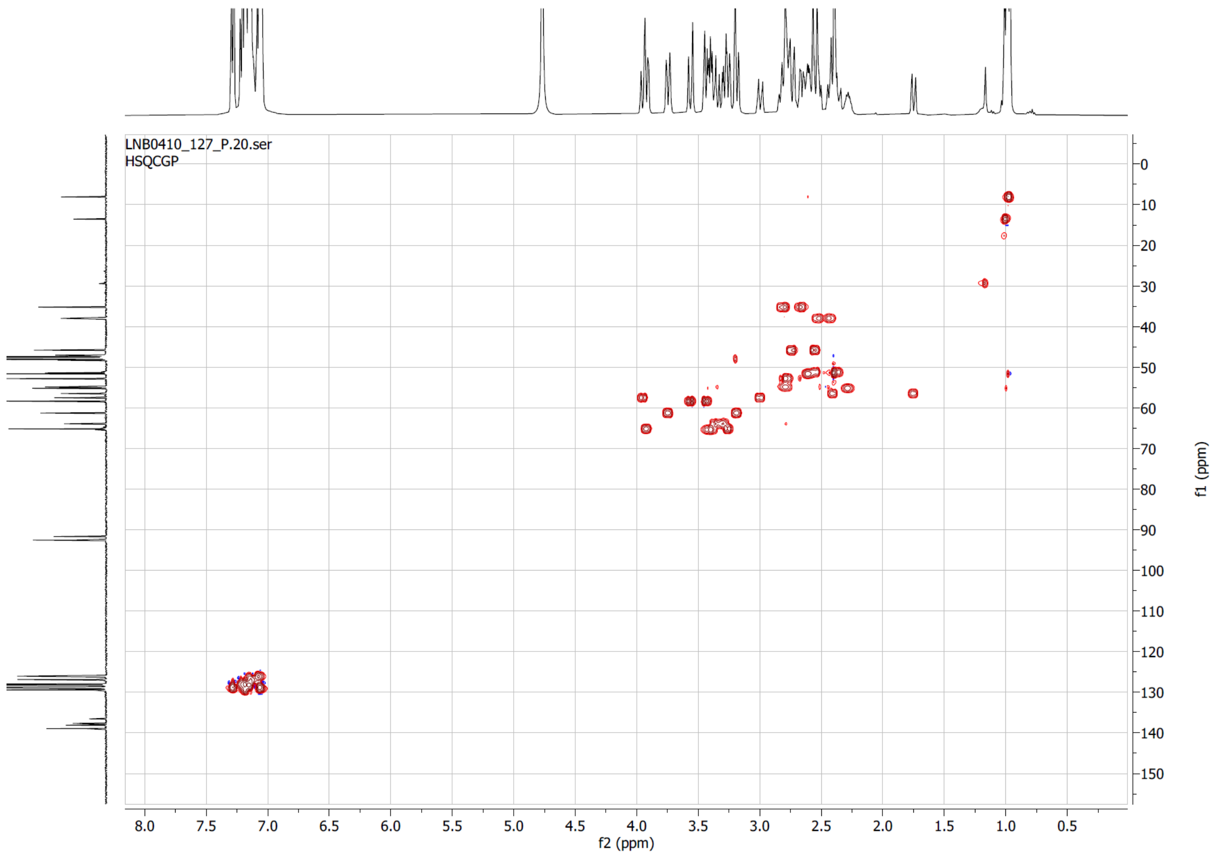
LNB0410_127_P.20.ser
HSQCGP
f2 (ppm)
f1 (ppm)

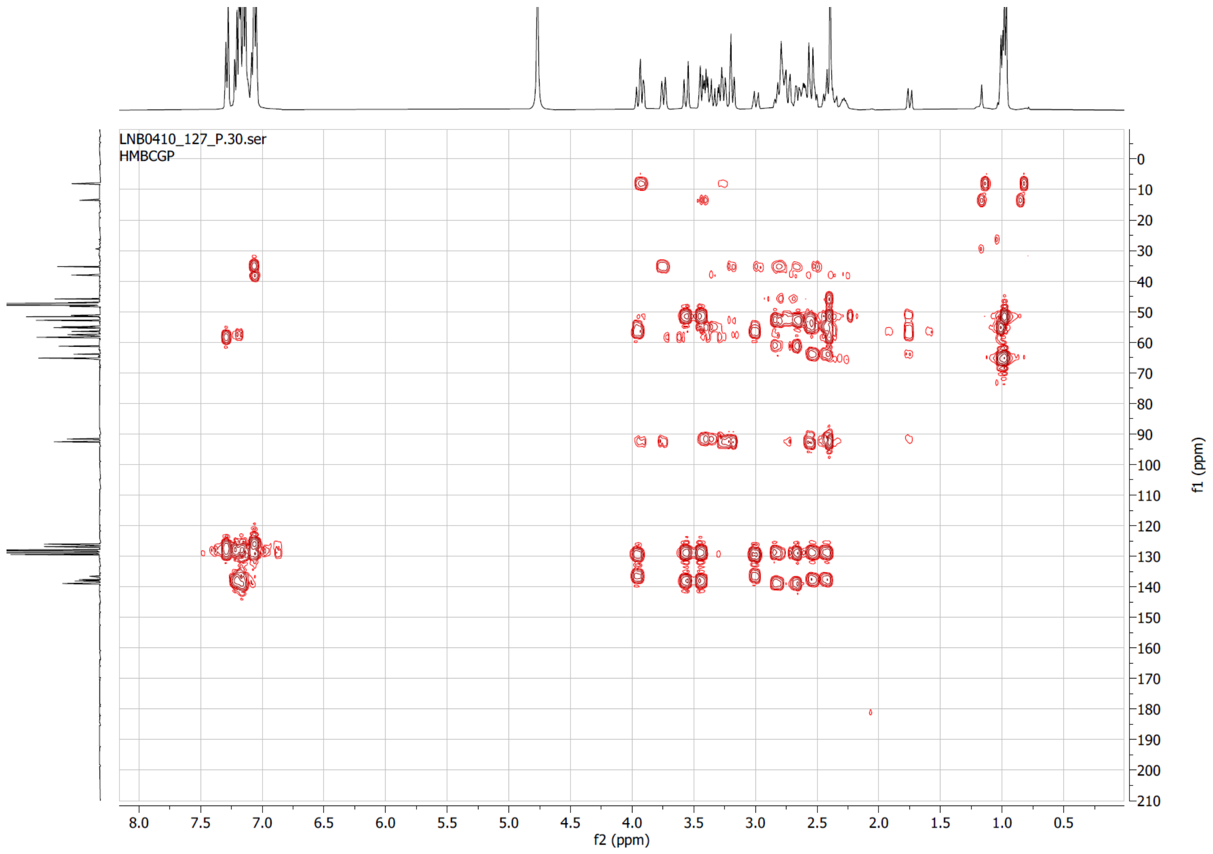
LNB0410_127_P.30.ser
HMBCGP
f2 (ppm)
f1 (ppm)

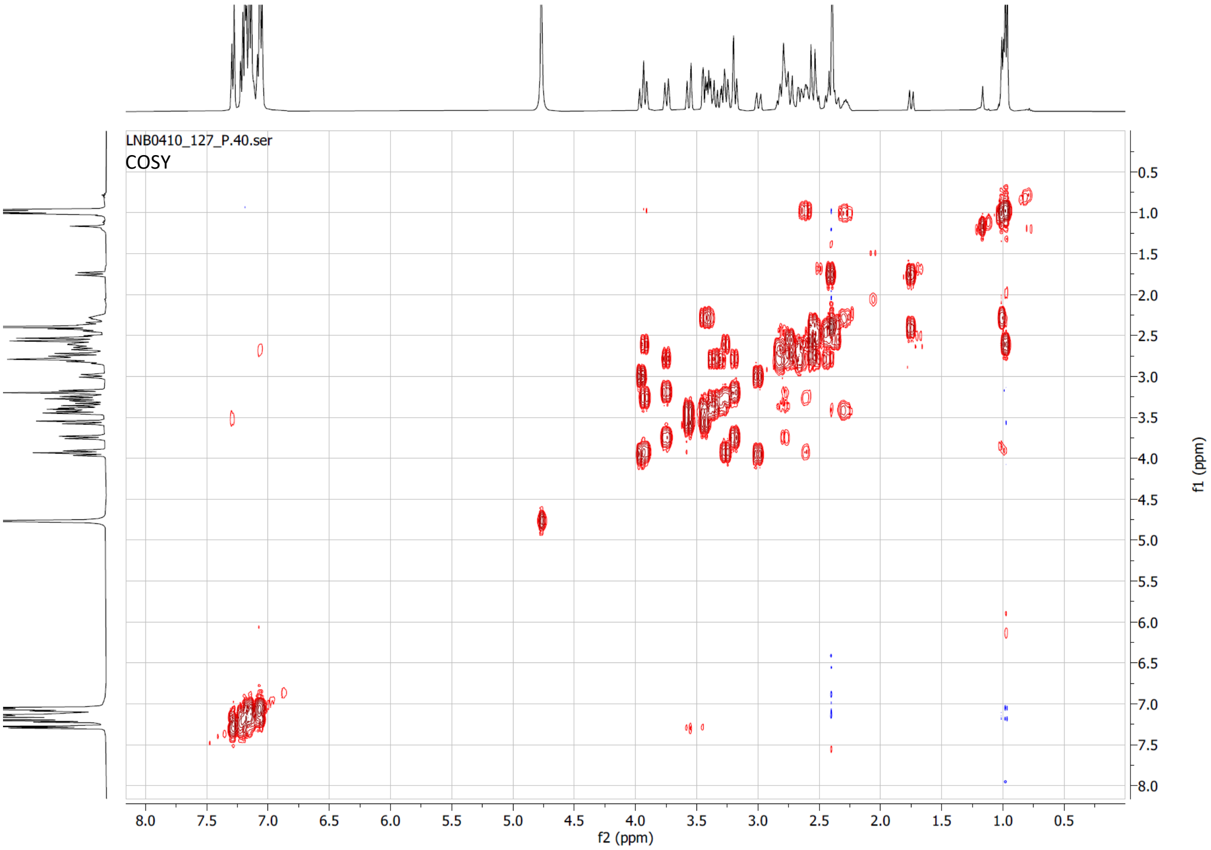
LNB0410_127_P.40.ser
COSY
f2 (ppm)
f1 (ppm)
8.0
7.5
7.0
6.5
6.0
5.5
5.0
4.5
4.0
3.5
3.0
2.5
2.0
1.5
1.0
0.5

(9*H*-fluoren-9-yl)methyl (3*S*,9*S*)-3,10-dibenzyl-9-methyl-1,7-dioxa-4,10-diazaspiro[5.5]undecane-4-carboxylate (*S*39) [^{1}H-NMR data: 400 MHz, $CDCl_3$; $^{13}C\{^{1}H\}$-NMR data: 101 MHz, $CDCl_3$; 2D NMR spectra: HSQC, HMBC, COSY, all in $CDCl_3$]:

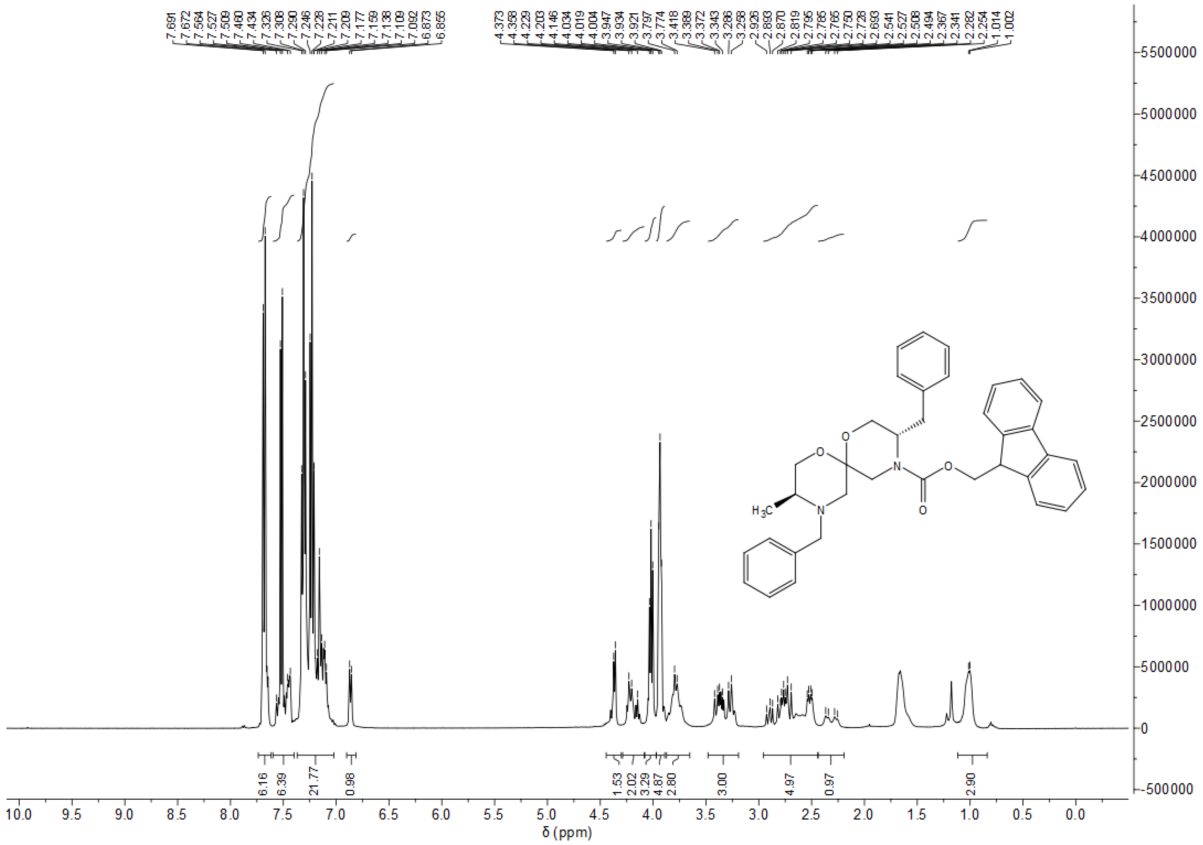

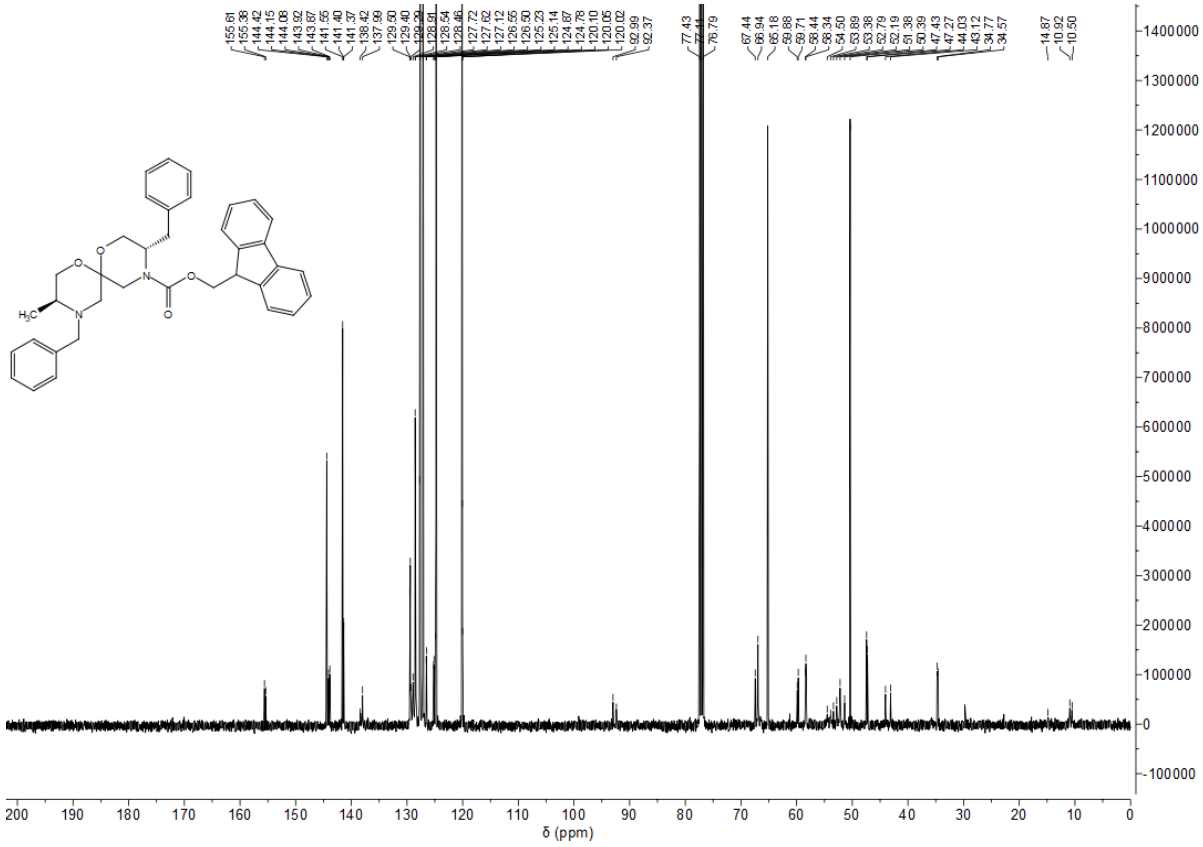

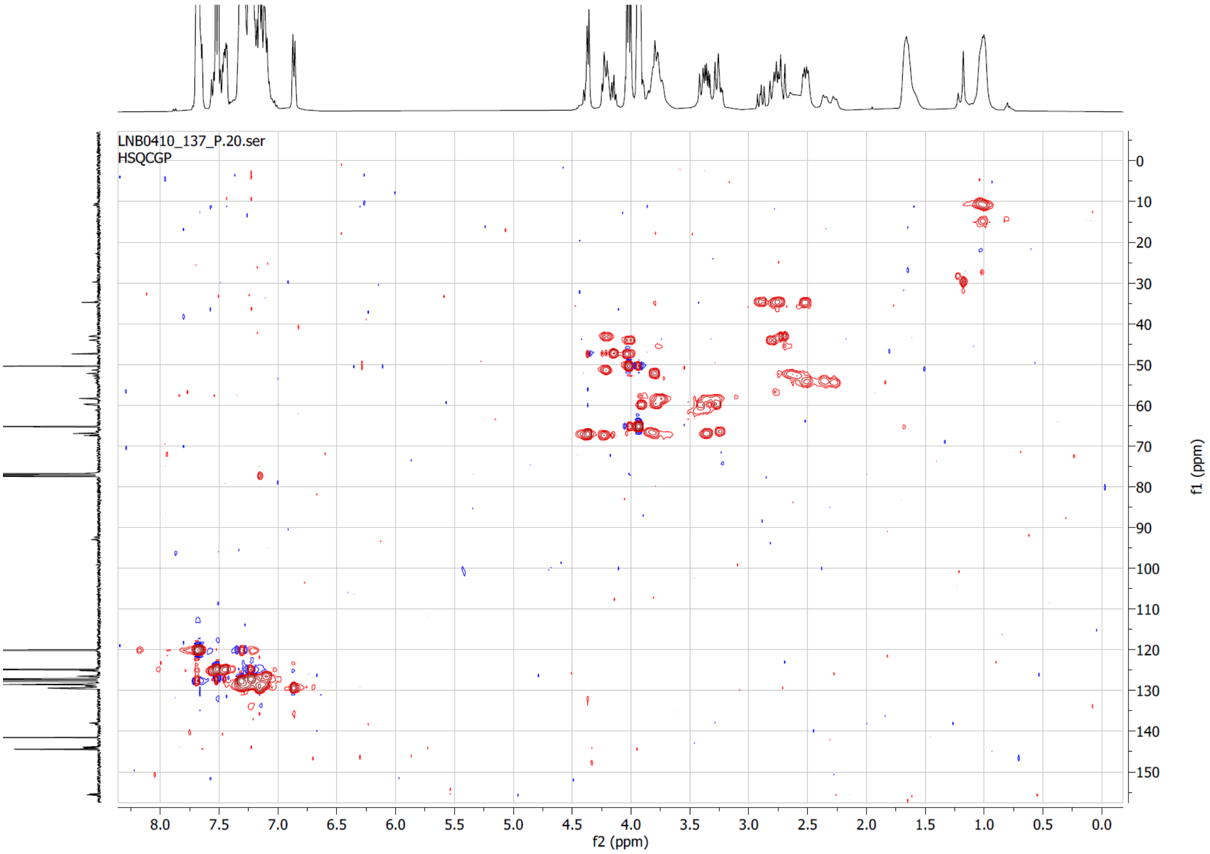
LNB0410_137_P.20.ser
HSQCGP
f2 (ppm)
f1 (ppm)

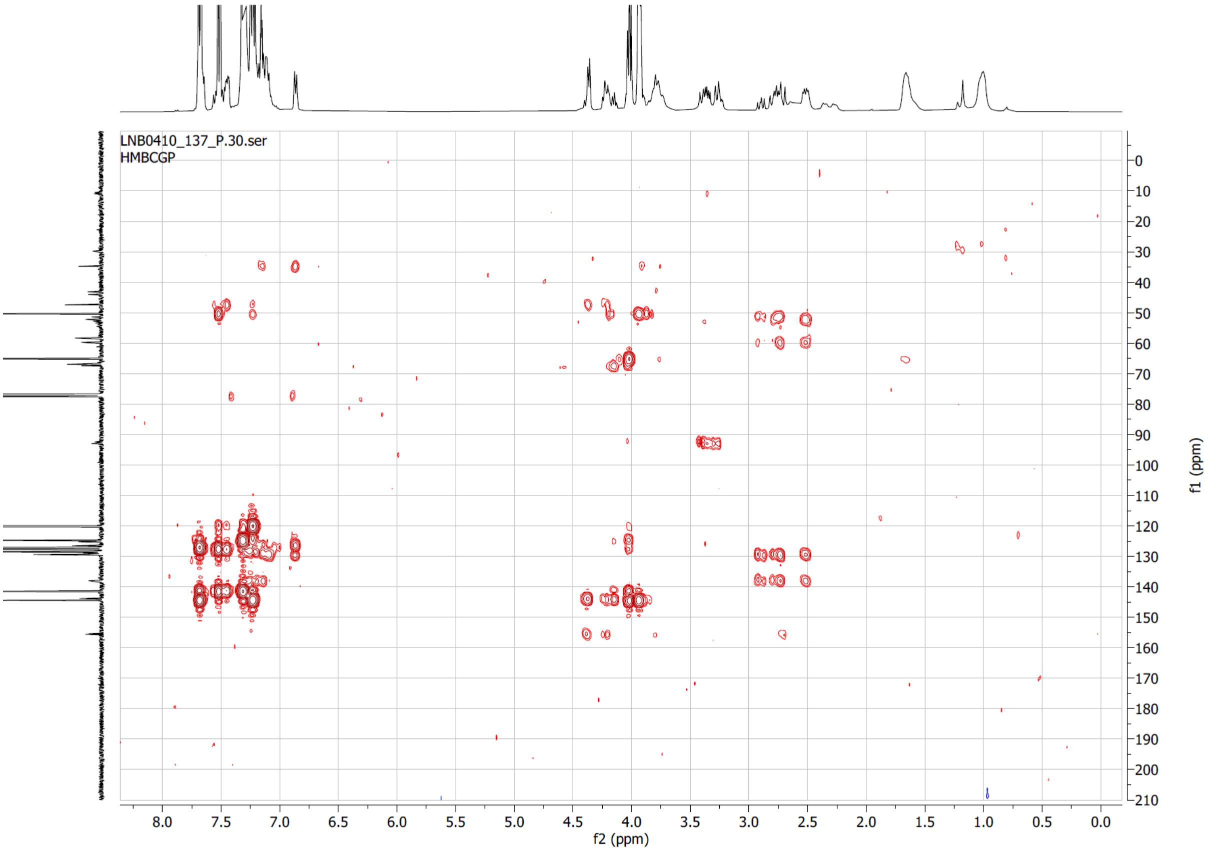
LNB0410_137_P.30.ser
HMBCGP
f2 (ppm)
f1 (ppm)

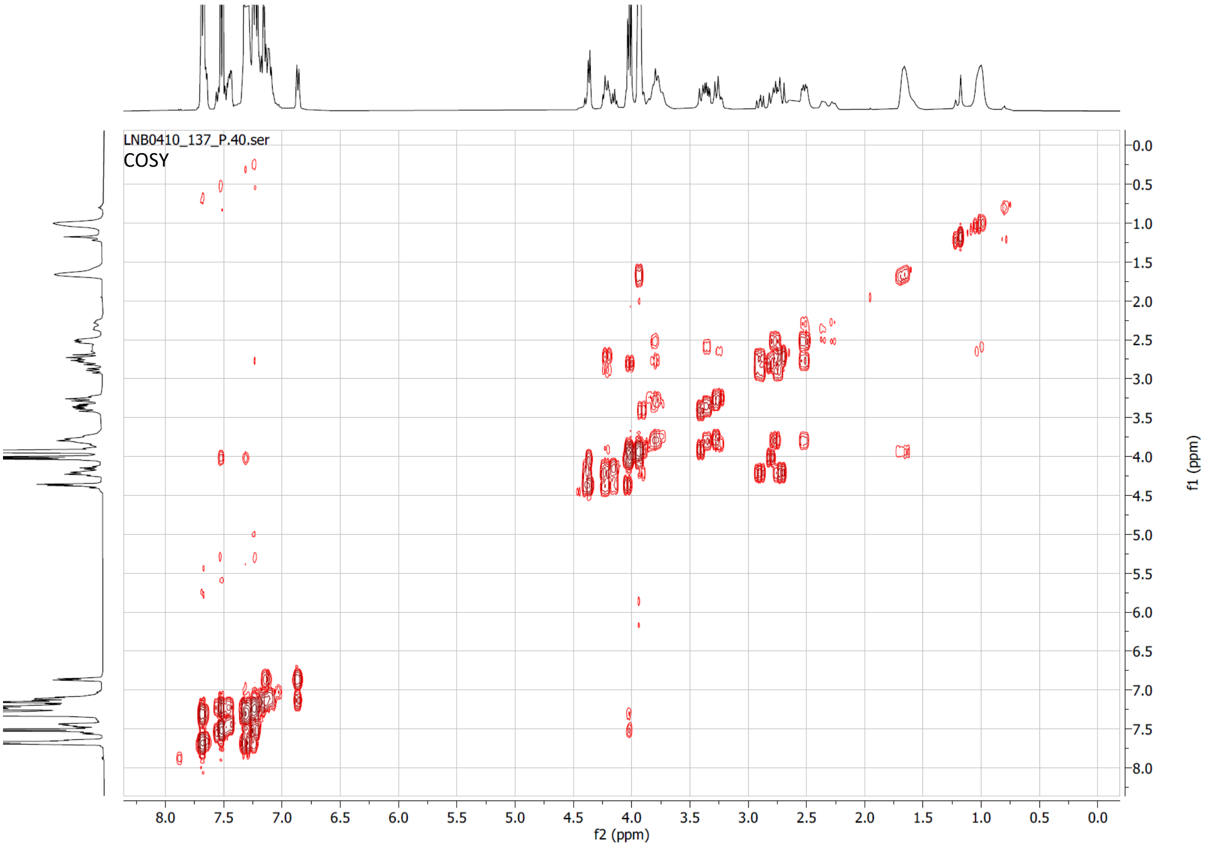

LNB0410_137_P.40.ser
COSY
f2 (ppm)
f1 (ppm)

1.6 Library compounds

1.6.1 First decoration step – Urea formation

benzyl 10-((2,4-difluorophenyl)carbamoyl)-1,7-dioxa-4,10-diazaspiro[5.5]undecane-4-carboxylate (10-I03 *or* 11 in the main article) [^{1}H-NMR data: 500 MHz, C_6D_6; $^{13}C\{^{1}H\}$-NMR data: 126 MHz, C_6D_6; ^{19}F-NMR data: 376 MHz, C_6D_6]:

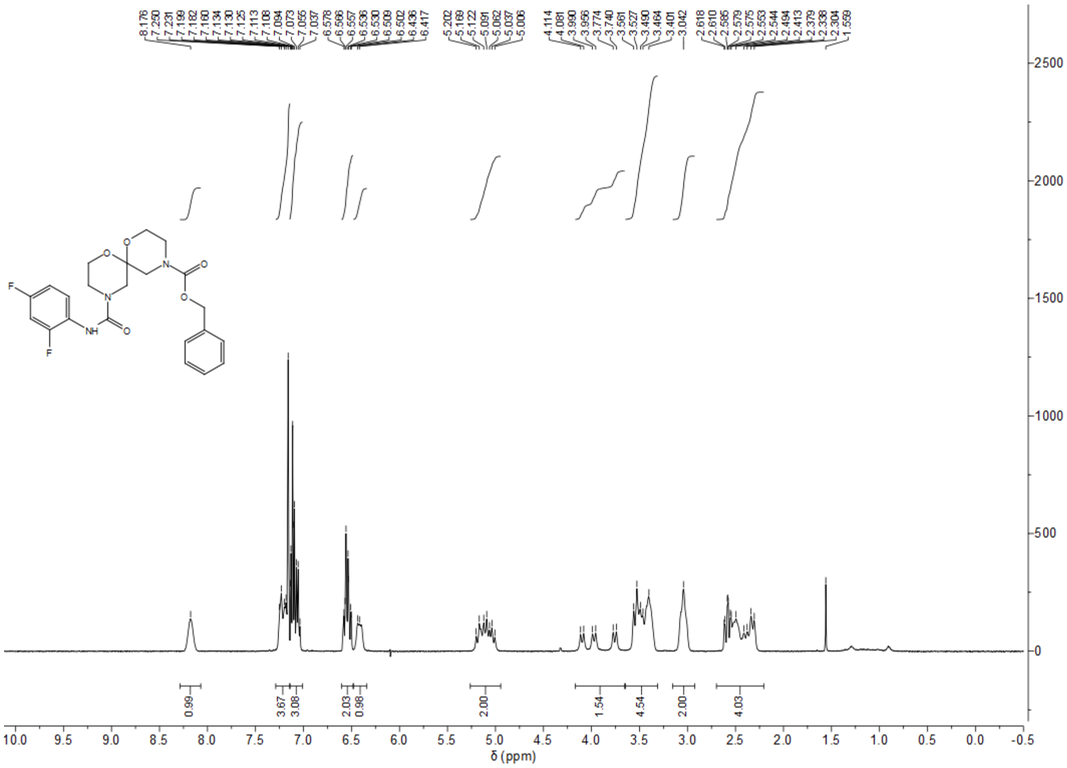

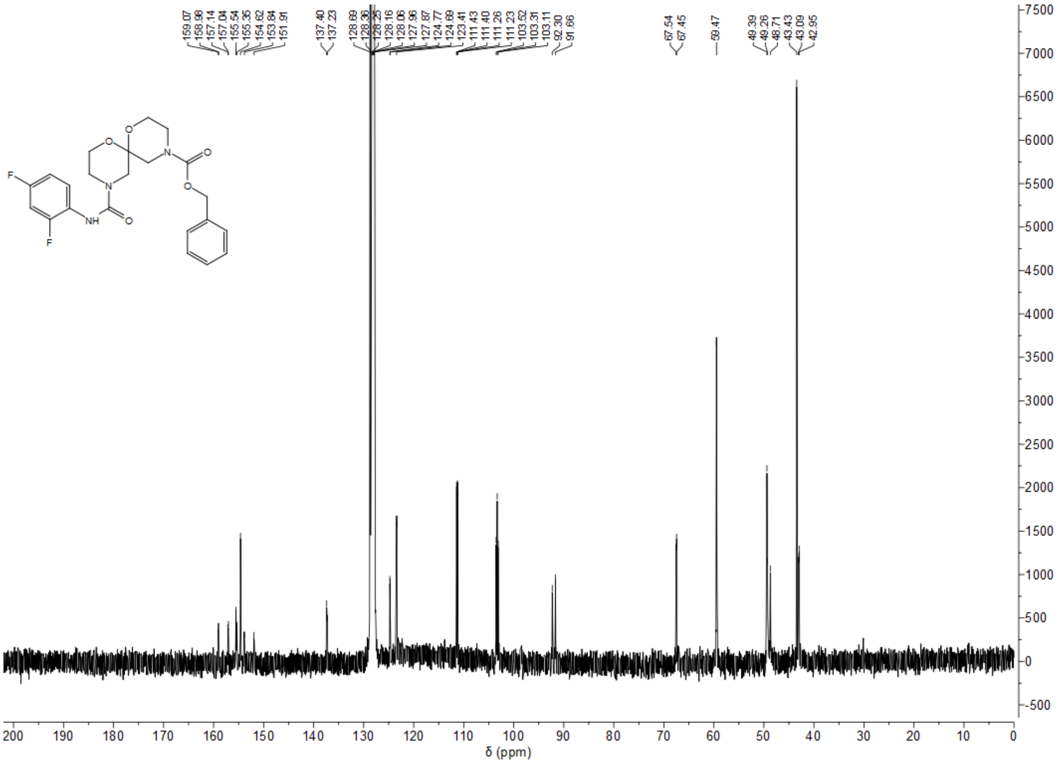

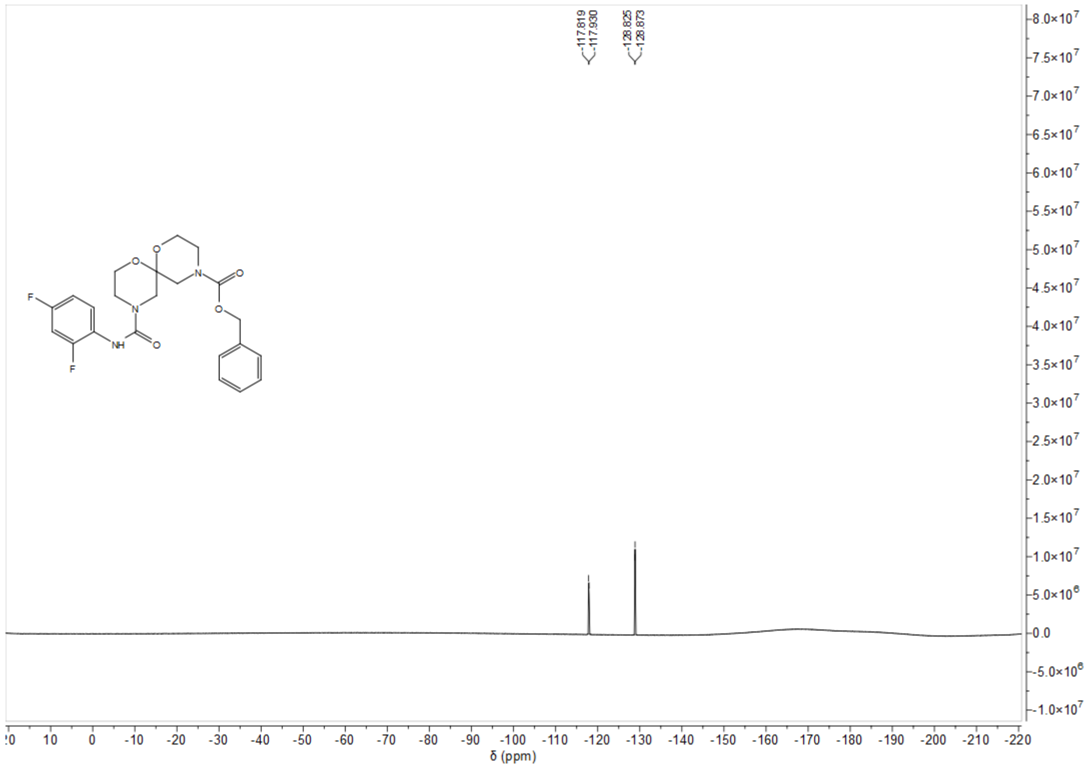

-117.819
-117.930
-128.825
-128.873
F
F
NH
O
N
O
O
N
O
O
δ (ppm)

***N*-(2,4-difluorophenyl)-1,7-dioxa-4,10-diazaspiro[5.5]undecane-4-carboxamide (L1-I03 *or* 13 in the main article) [^{1}H-NMR data: 400 MHz, CD_3OD; $^{13}C\{^{1}H\}$-NMR data: 101 MHz, CD_3OD; ^{19}F-NMR data: 376 MHz, CD_3OD]:**

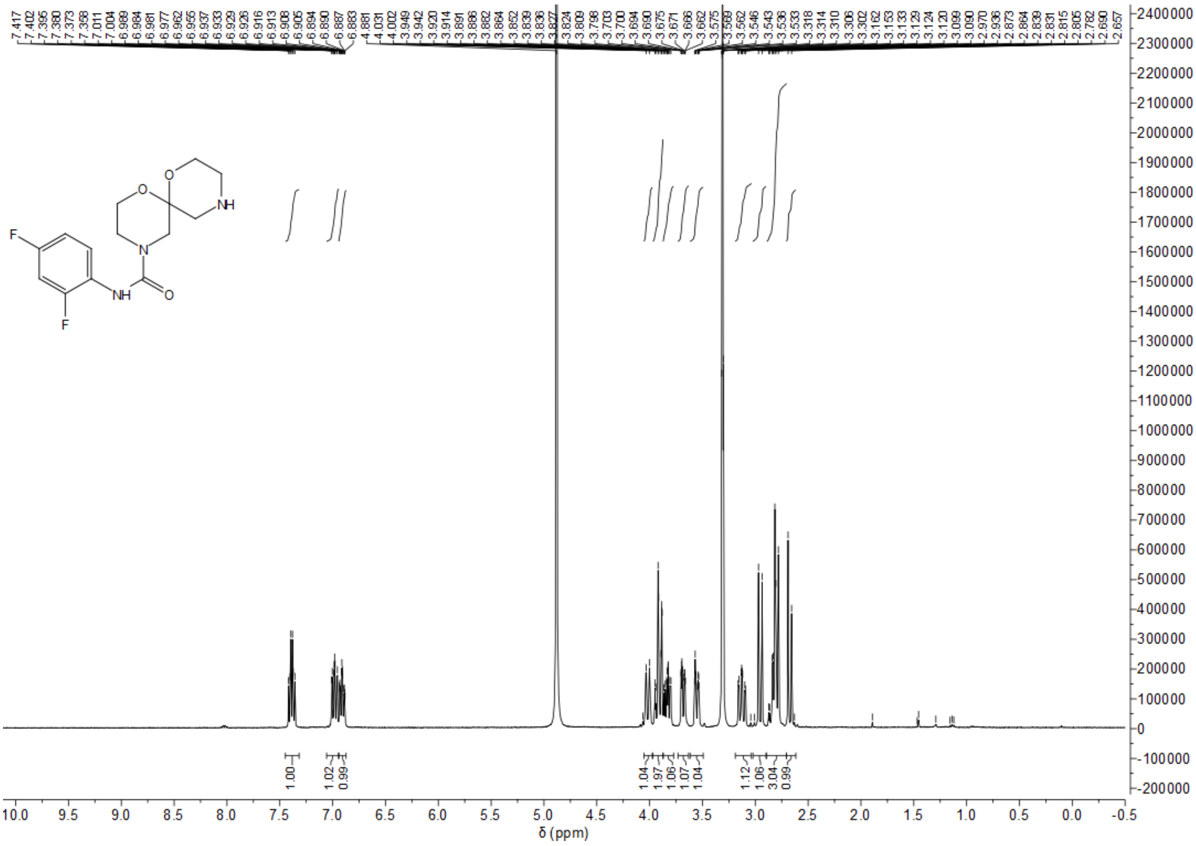

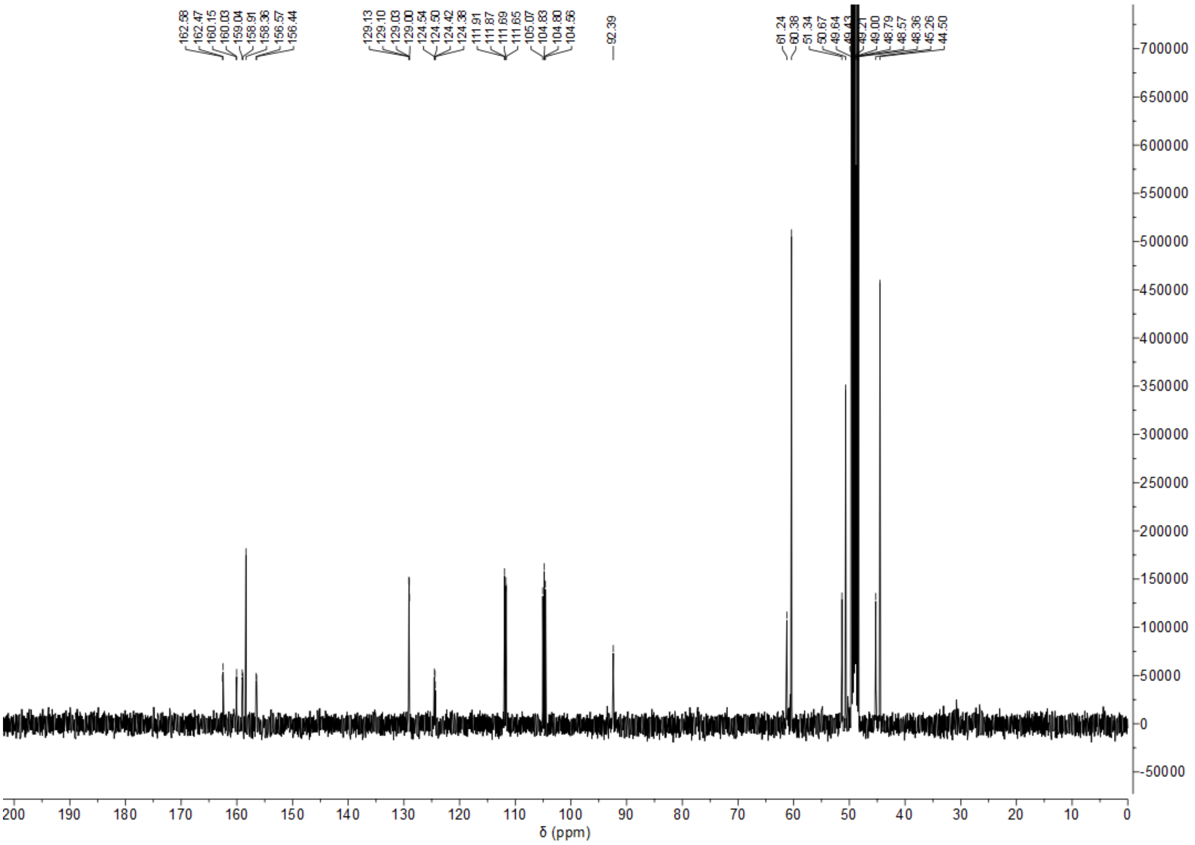

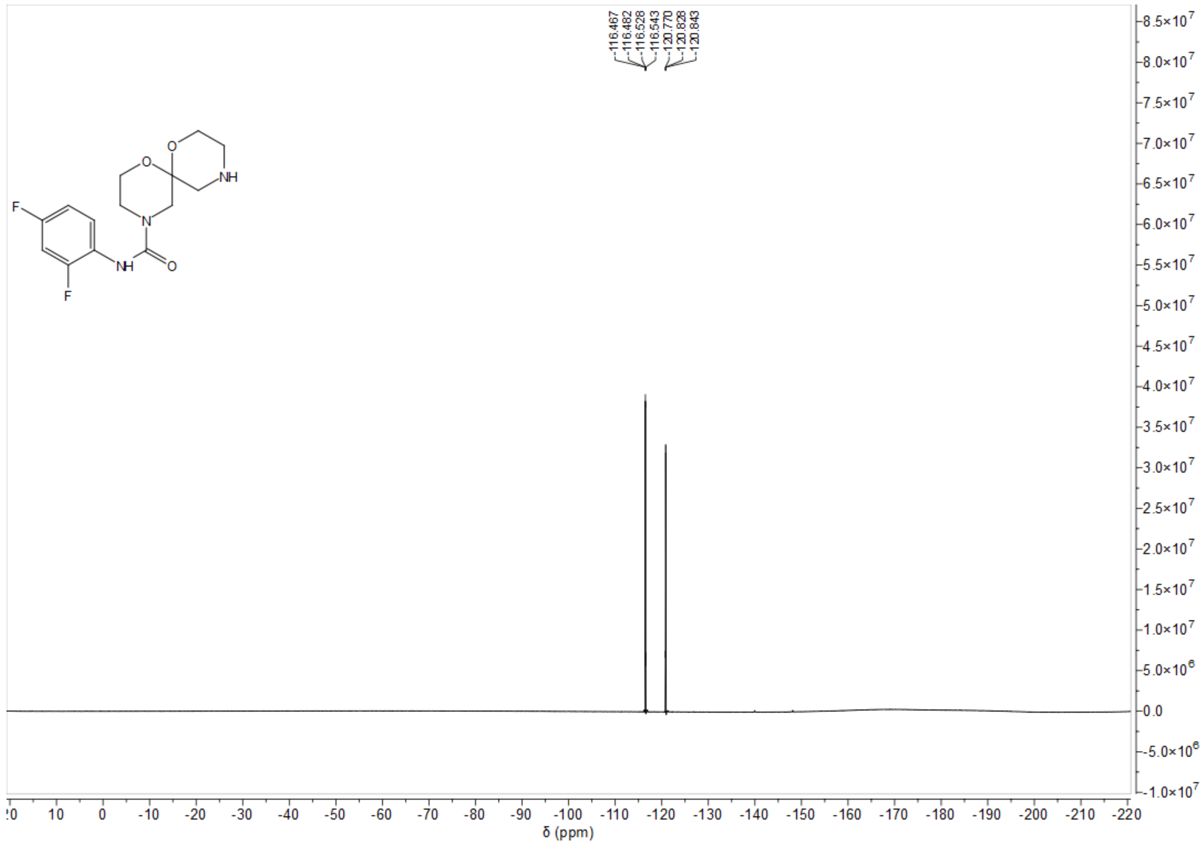

-116.467
-116.482
-116.528
-116.543
-120.770
-120.828
-120.843
F
F
NH
O
N
O
O
NH
δ (ppm)

N-(2,4-difluorophenyl)-10-(2-(pyridine-3-yl)acetyl)-1,7-dioxa-4,10-diazaspiro[5.5] undecane-4-carboxamide (L1-I03-C39) [^{1}H-NMR data: 400 MHz, CD_3OD; $^{13}C\{^{1}H\}$-NMR data: 101 MHz, CD_3OD]:

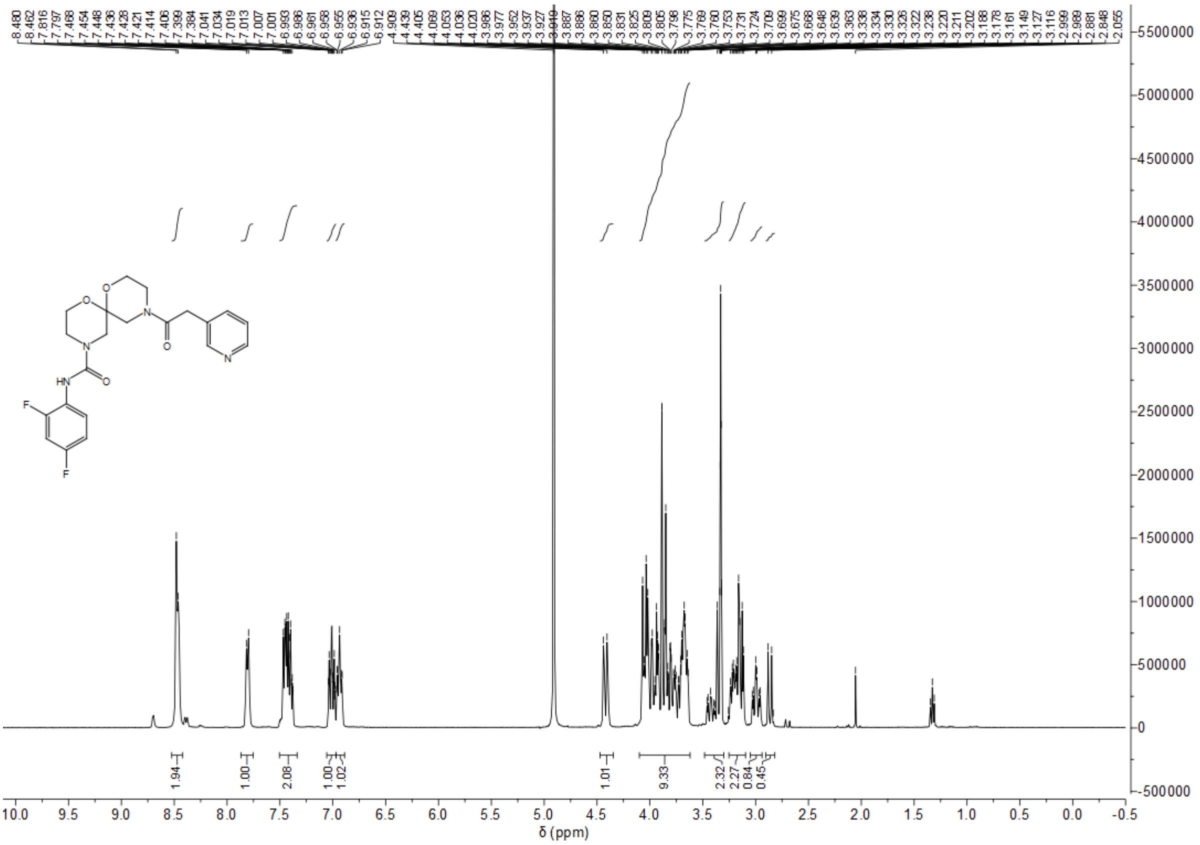

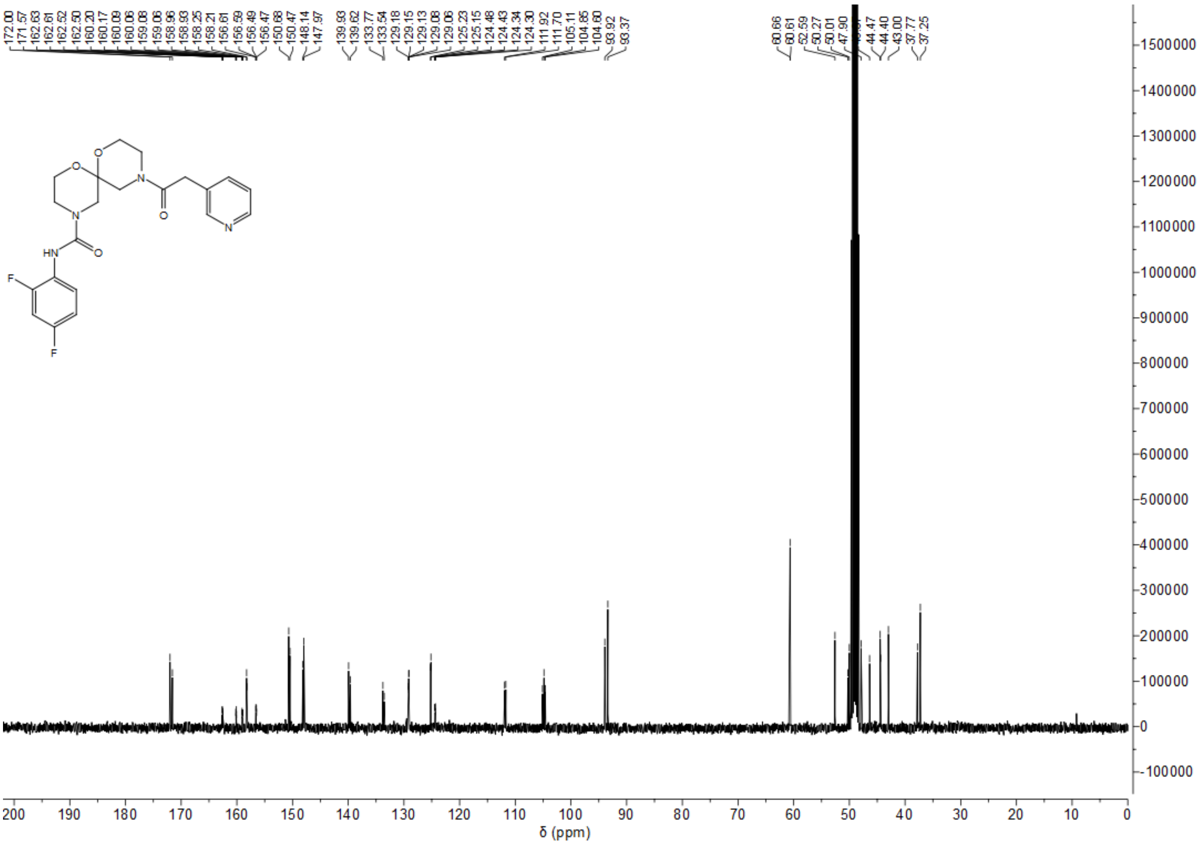

10-(4,4-difluorocyclohexyl)-*N*-(2,4-difluorophenyl)-1,7-dioxa-4,10-diazaspiro[5.5] undecane-4-carboxamide (L1-I03-K01) [^{1}H-NMR data: 400 MHz, CD_3OD; ^{13}C{^{1}H}-NMR data: 101 MHz, CD_3OD]:

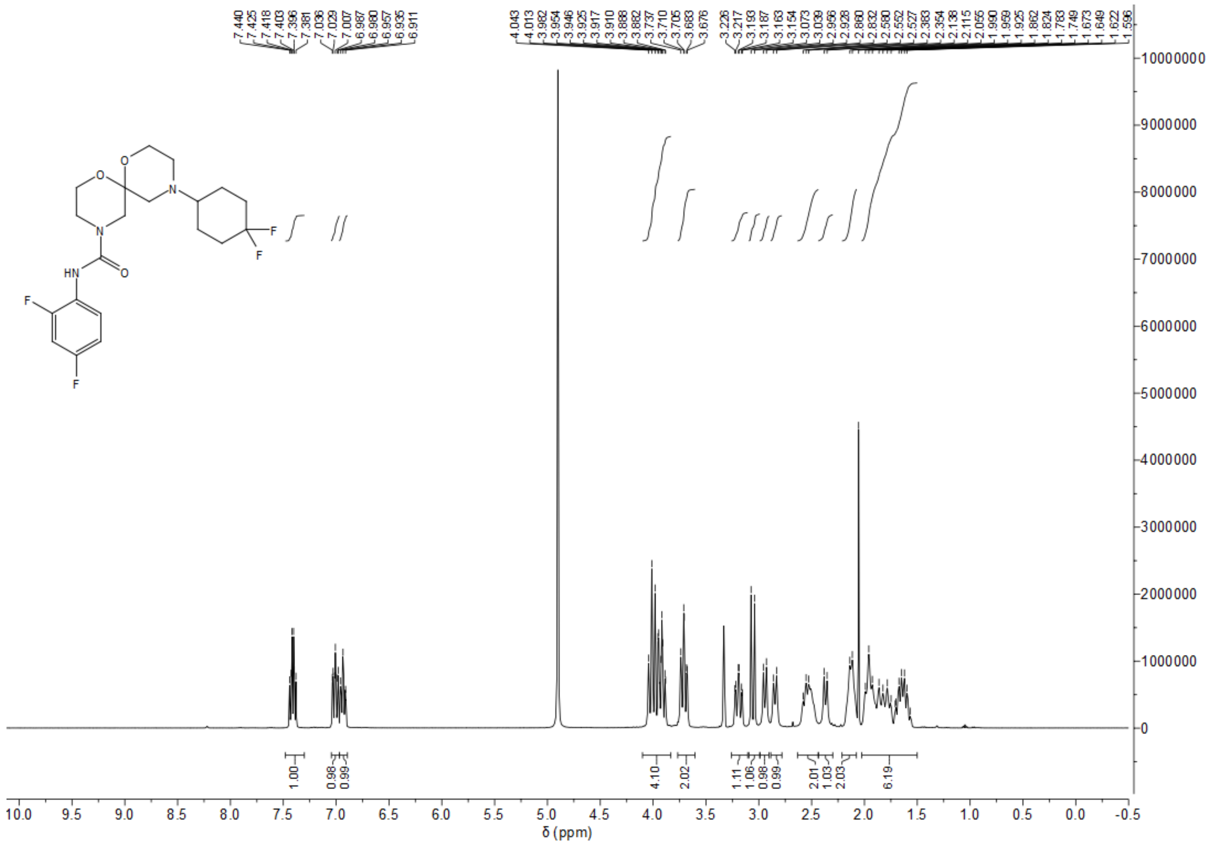

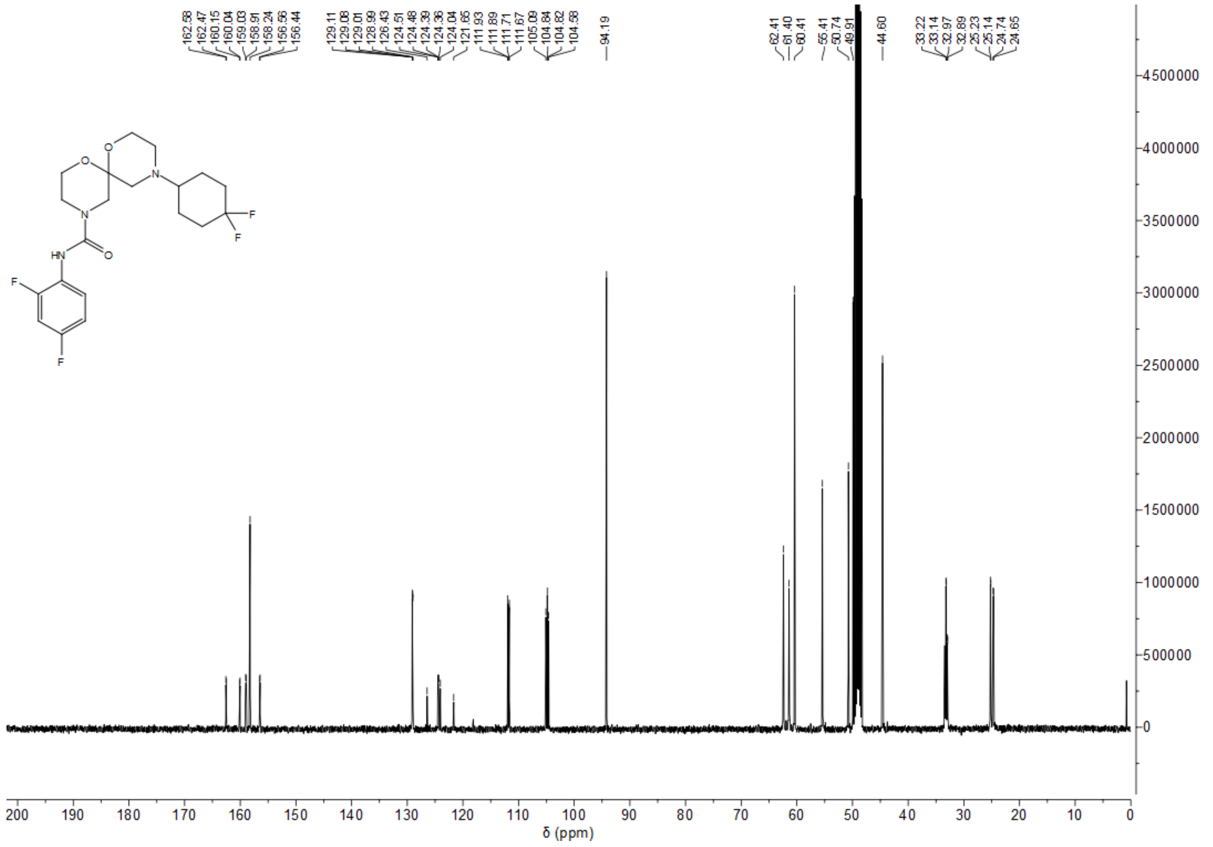

***N*-(2,4-difluorophenyl)-10-(imidazo[1,5-a]pyridin-3-ylmethyl)-1,7-dioxa-4,10-diazaspiro [5.5]undecane-4-carboxamide (L1-I03-A11) [^{1}H-NMR data: 400 MHz, CD_3OD; $^{13}C\{^{1}H\}$-NMR data: 101 MHz, CD_3OD]:**

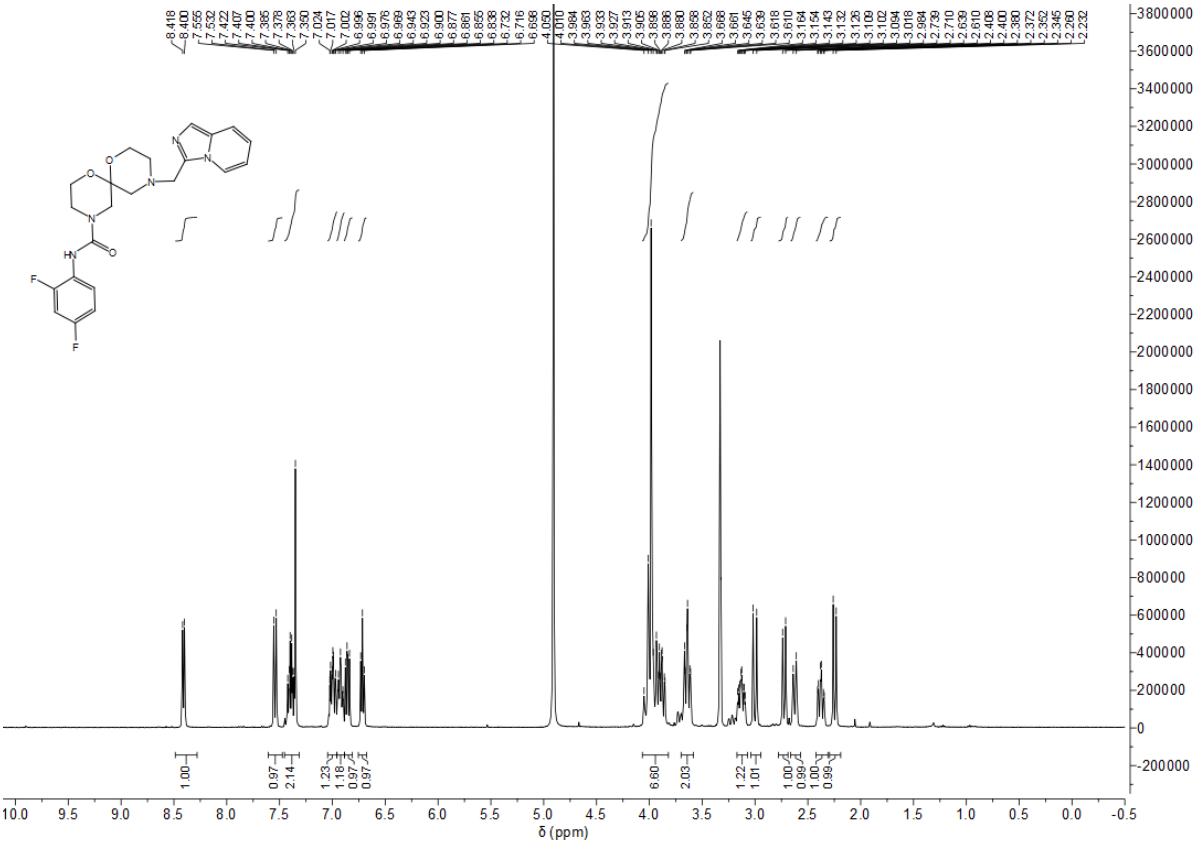

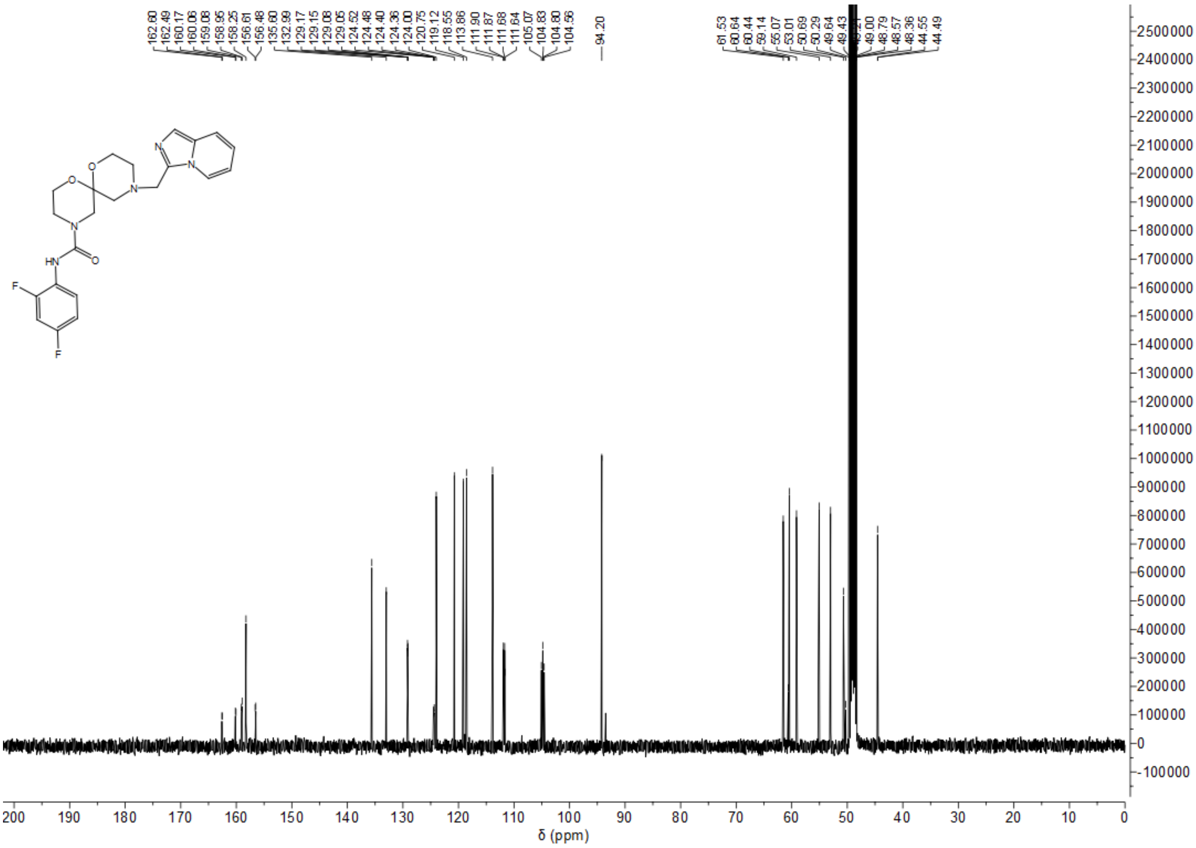

***tert*-butyl 10-(benzylcarbamoyl)-1,7-dioxa-4,10-diazaspiro[5.5]undecane-4-carboxylate (9-I04) [^{1}H-NMR data: 400 MHz, C_6D_6; $^{13}C\{^{1}H\}$-NMR data: 101 MHz, C_6D_6]:**

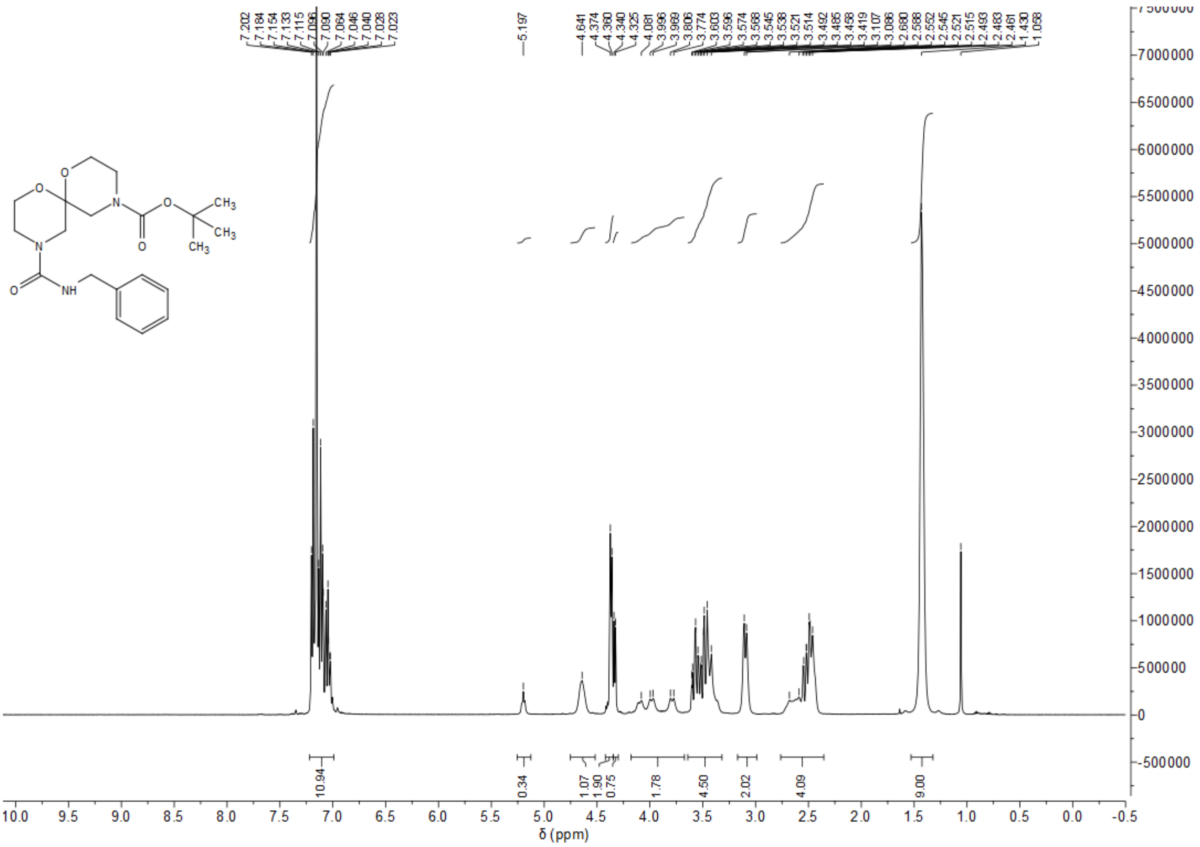

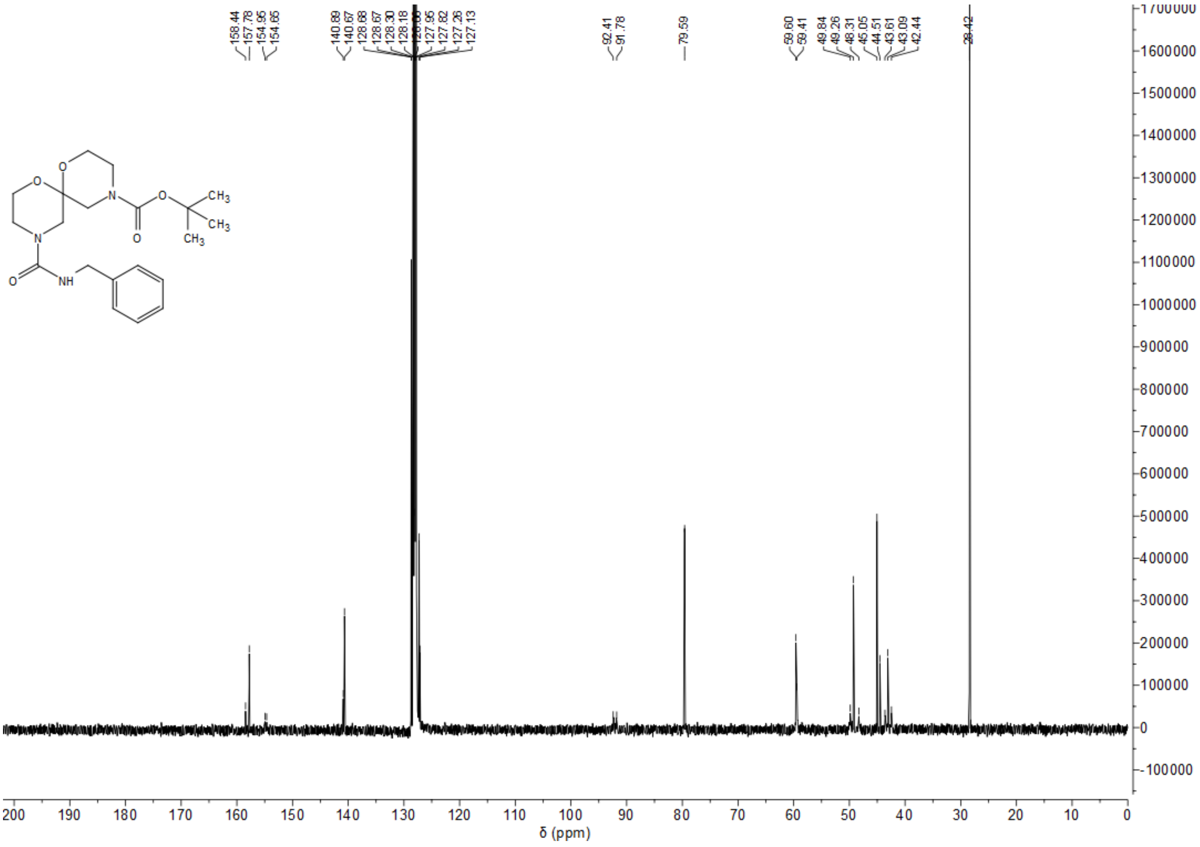

N-benzyl-1,7-dioxa-4,10-diazaspiro[5.5]undecane-4-carboxamide hydrochloride (L1-I04·HCl) [^{1}H-NMR data: 400 MHz, CD_3OD; $^{13}C\{^{1}H\}$-NMR data: 101 MHz, CD_3OD]:

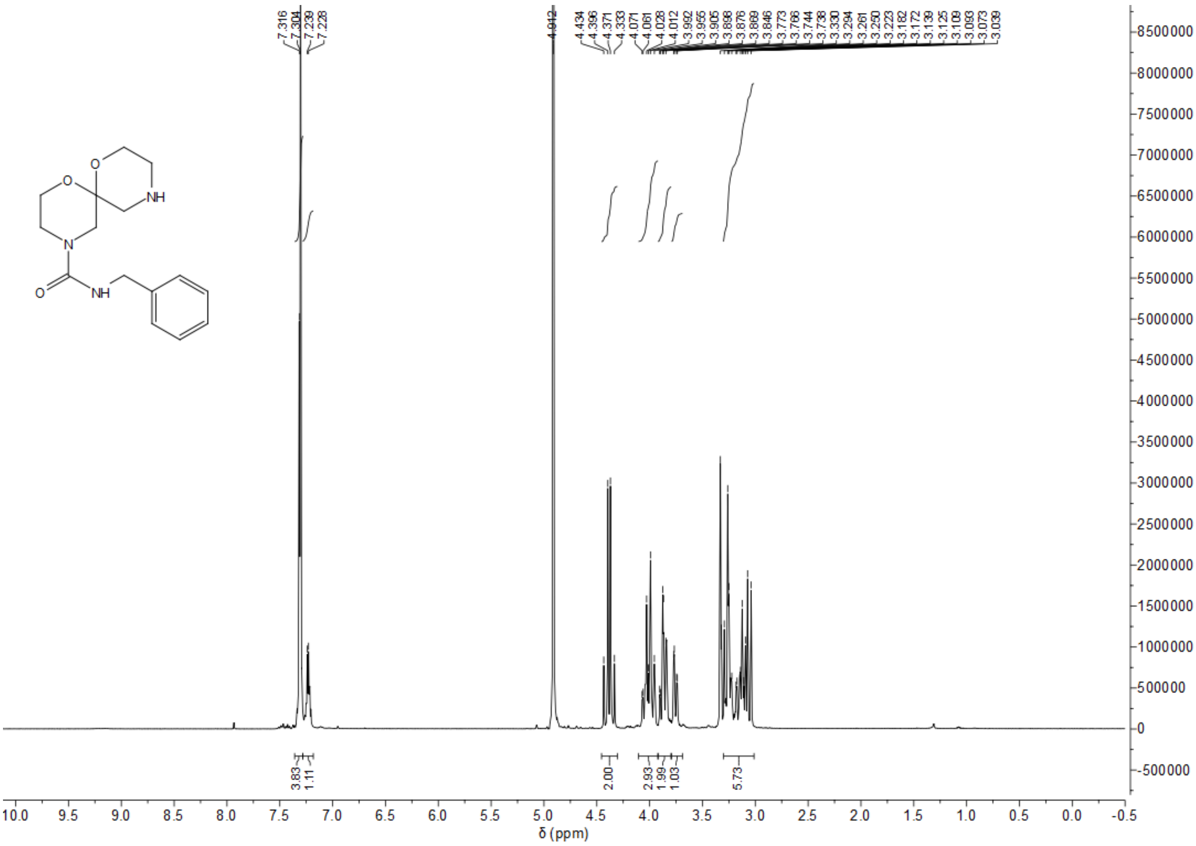

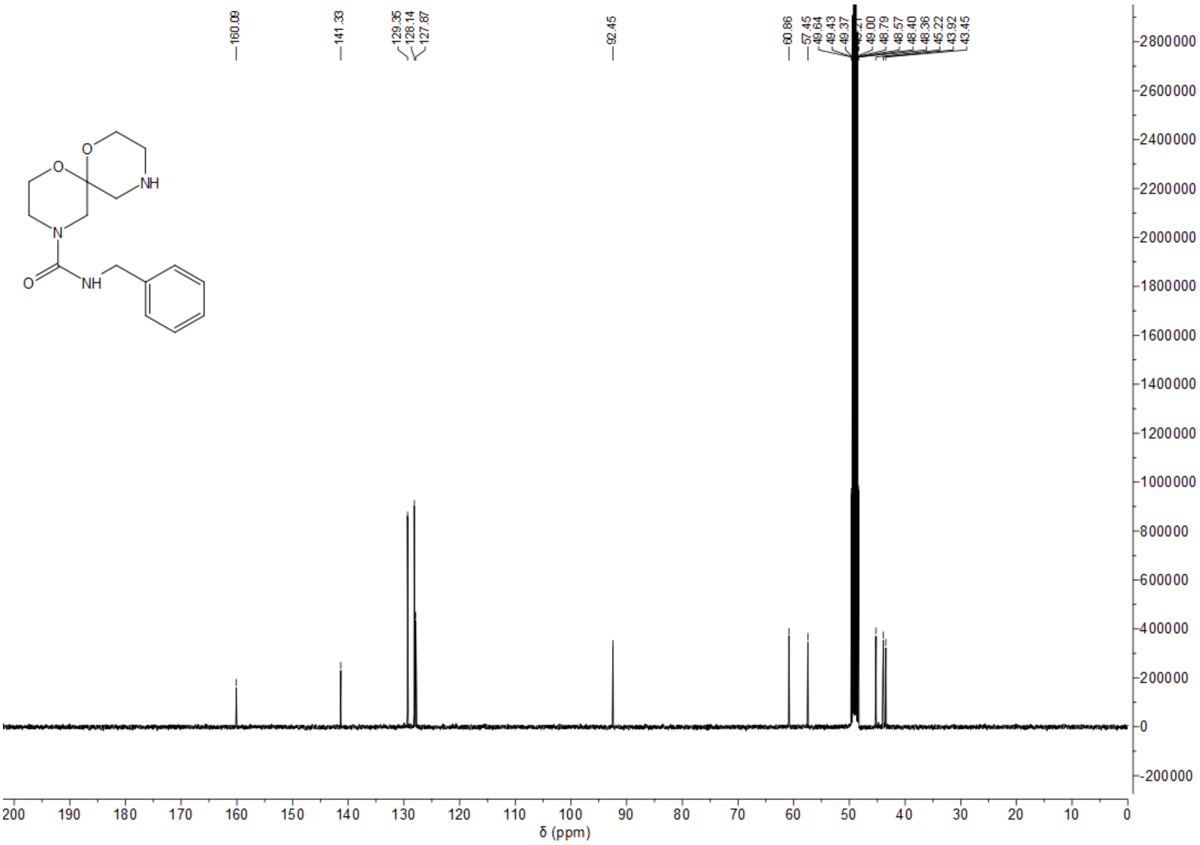

***N*-benzyl-10-((1,3,5-trimethyl-1*H*-pyrazol-4-yl)sulfonyl)-1,7-dioxa-4,10-diazaspiro[5.5] undecane-4-carboxamide (L1-I04-B10) [^{1}H-NMR data: 400 MHz, CD_3OD; ^{13}C{^{1}H}-NMR data: 101 MHz, CD_3OD]:**

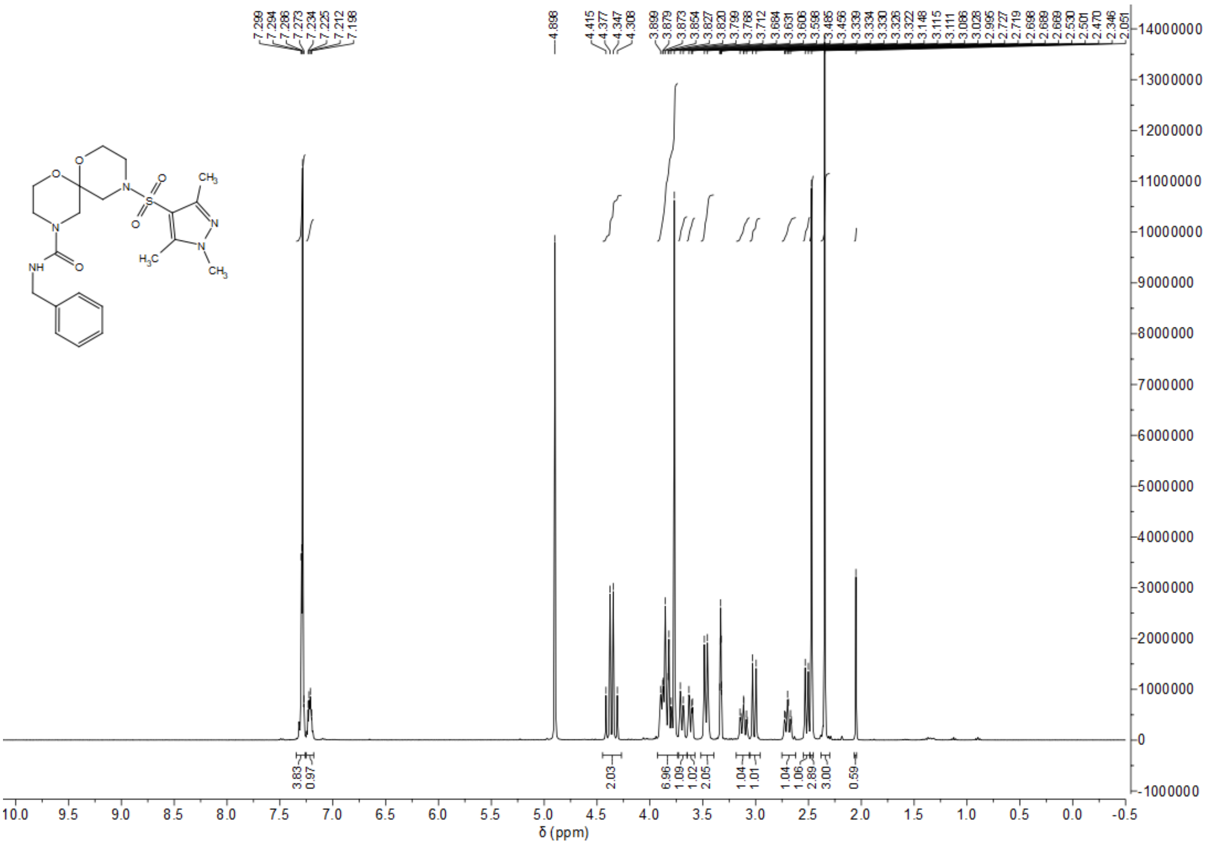

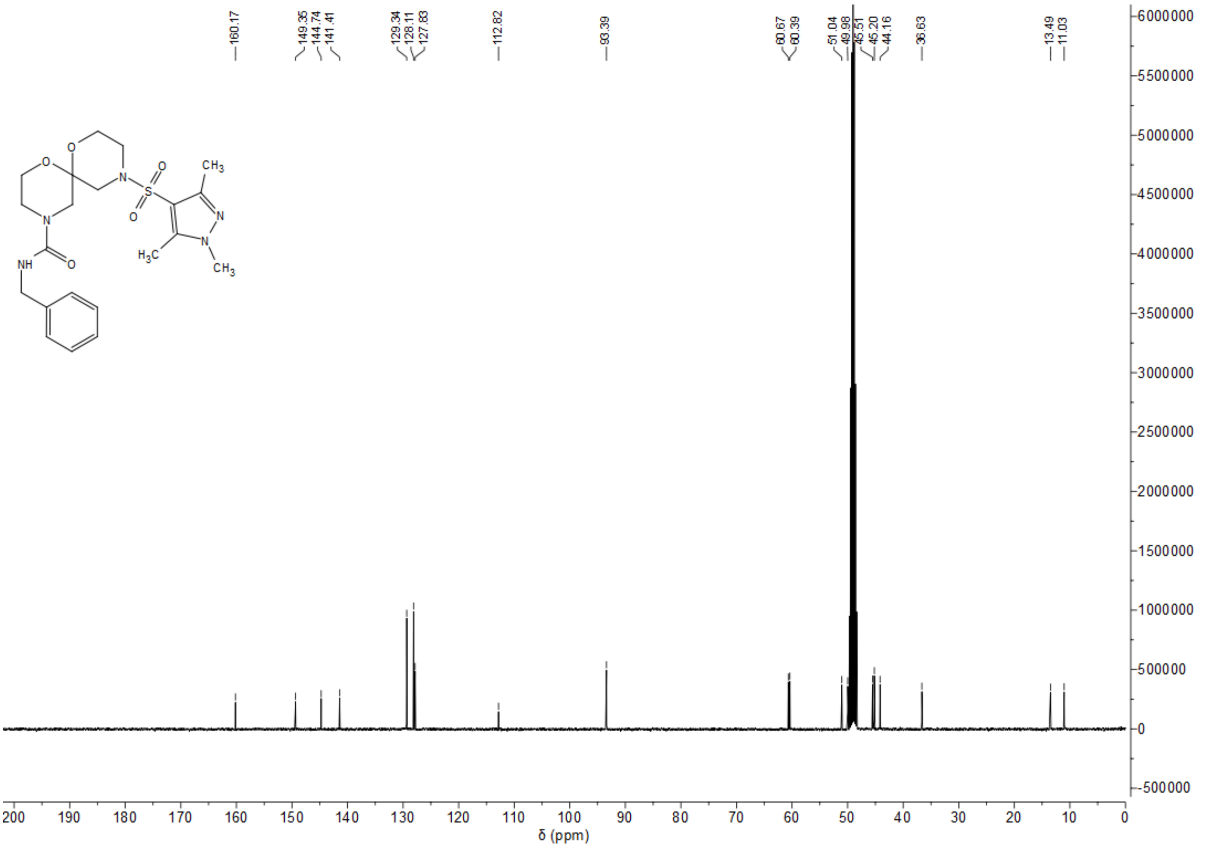

***N*-benzyl-10-((2,3-dihydrobenzo[*b*][1,4]dioxin-6-yl)methyl)-1,7-dioxa-4,10-diazaspiro[5.5]undecane-4-carboxamide (L1-I04-A13) [^{1}H-NMR data: 400 MHz, CD_3OD; $^{13}C\{^{1}H\}$-NMR data: 101 MHz, CD_3OD]:**

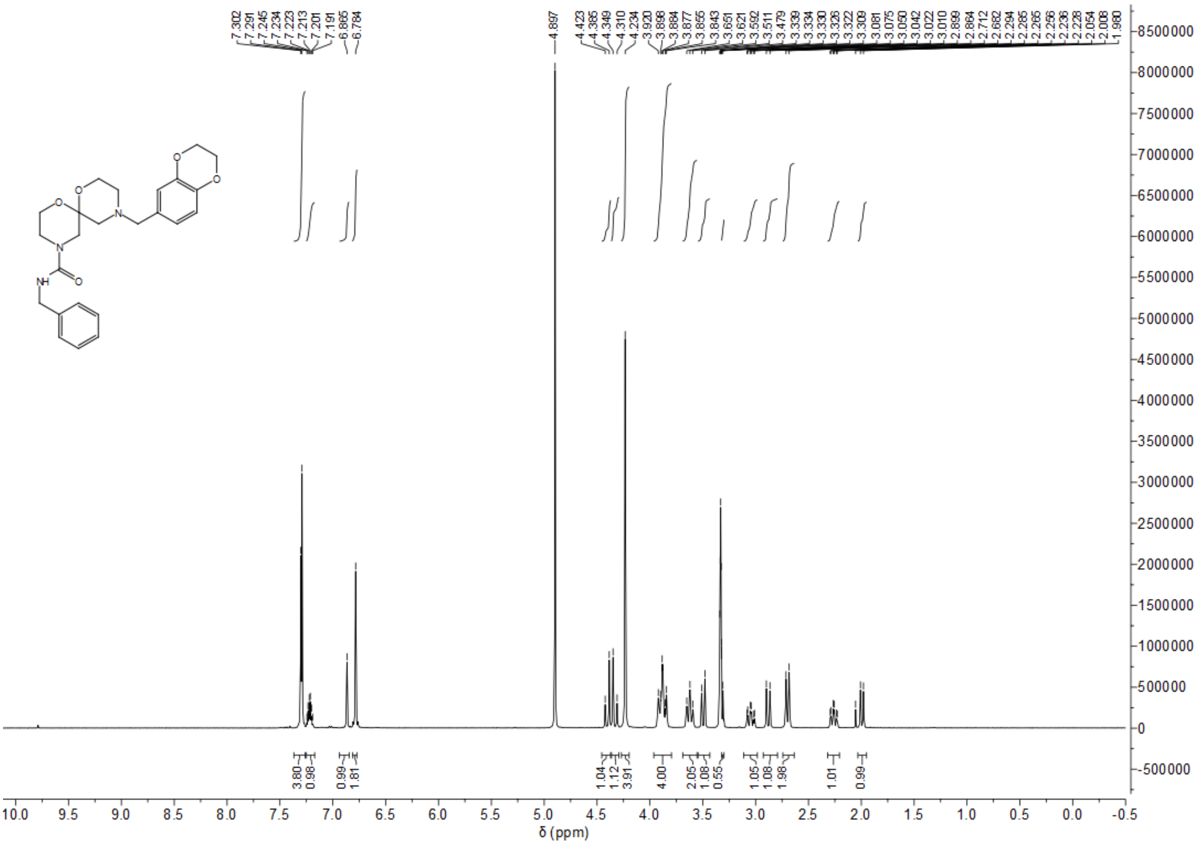

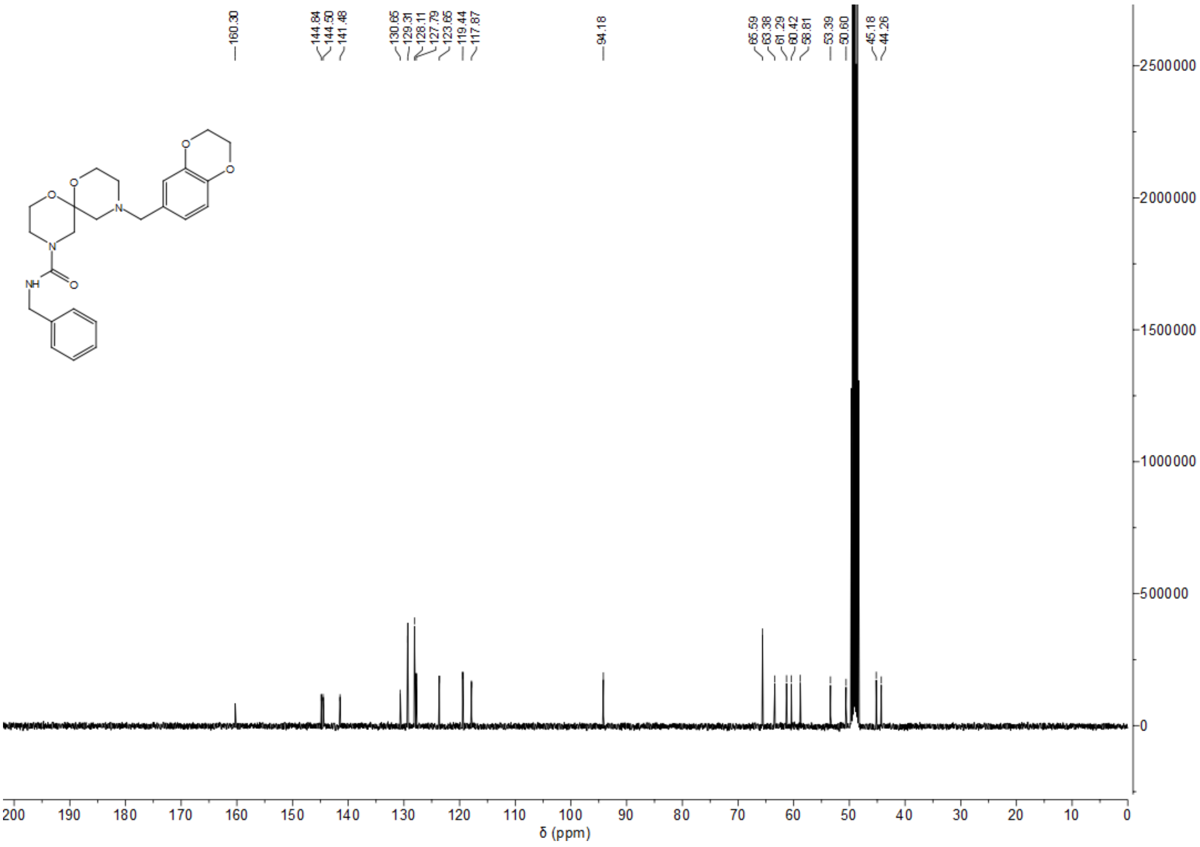

***N*-benzyl-10-((3-methoxypyridin-2-yl)methyl)-1,7-dioxa-4,10-diazaspiro[5.5]undecane-4-carboxamide (L1-I04-A08) [^{1}H-NMR data: 400 MHz, CD_3OD; $^{13}C\{^{1}H\}$-NMR data: 101 MHz, CD_3OD]:**

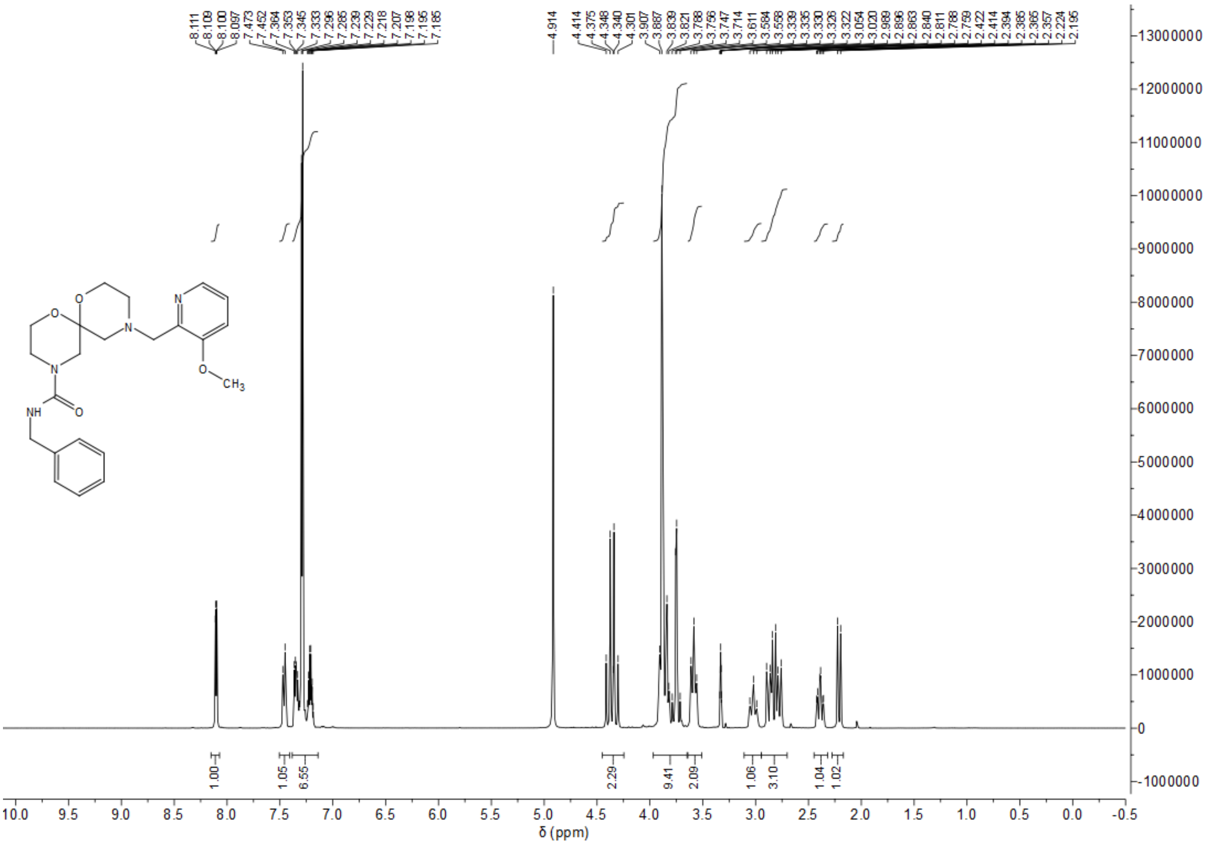

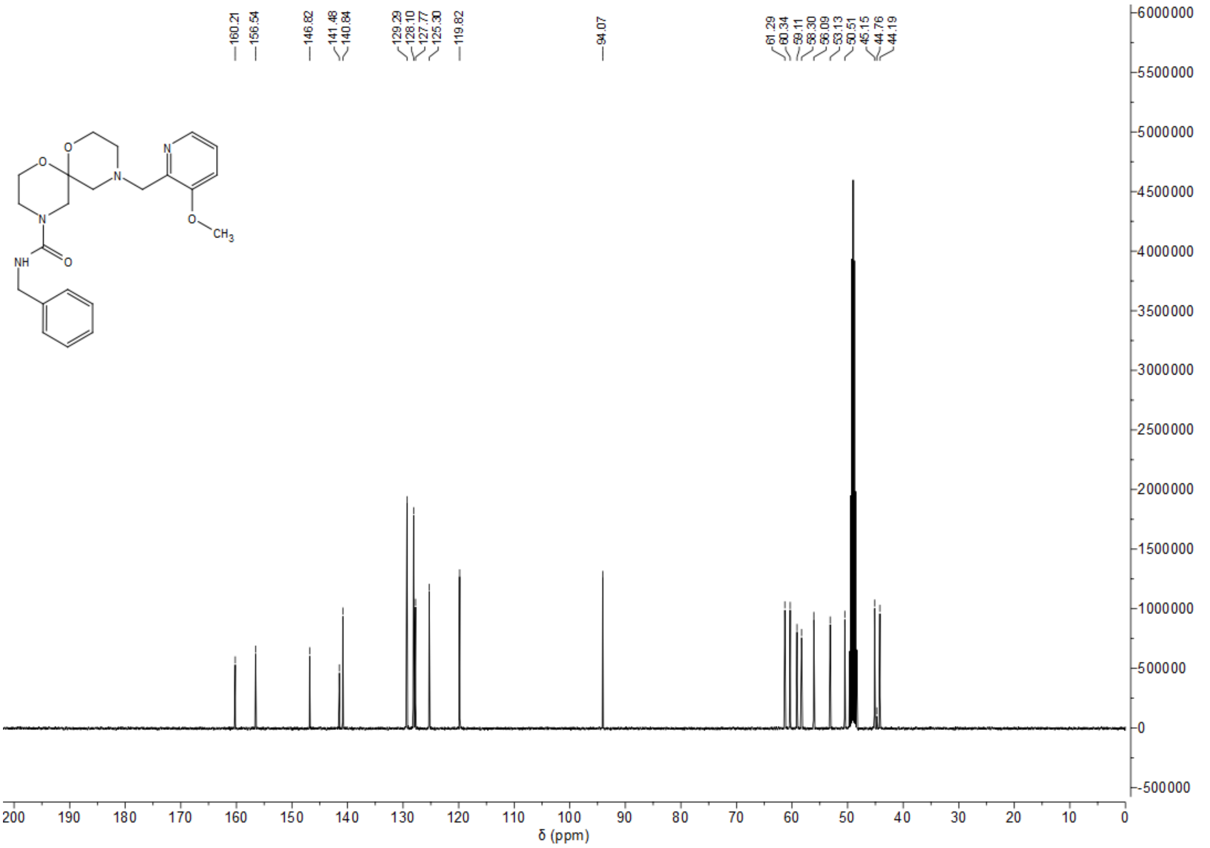

***tert*-butyl 10-((3,5-dimethylisoxazol-4-yl)carbamoyl)-1,7-dioxa-4,10-diazaspiro[5.5]undecane-4-carboxylate (9-I08) [^{1}H-NMR data: 400 MHz, C_6D_6; ^{13}C{^{1}H}-NMR data: 101 MHz, C_6D_6]:**

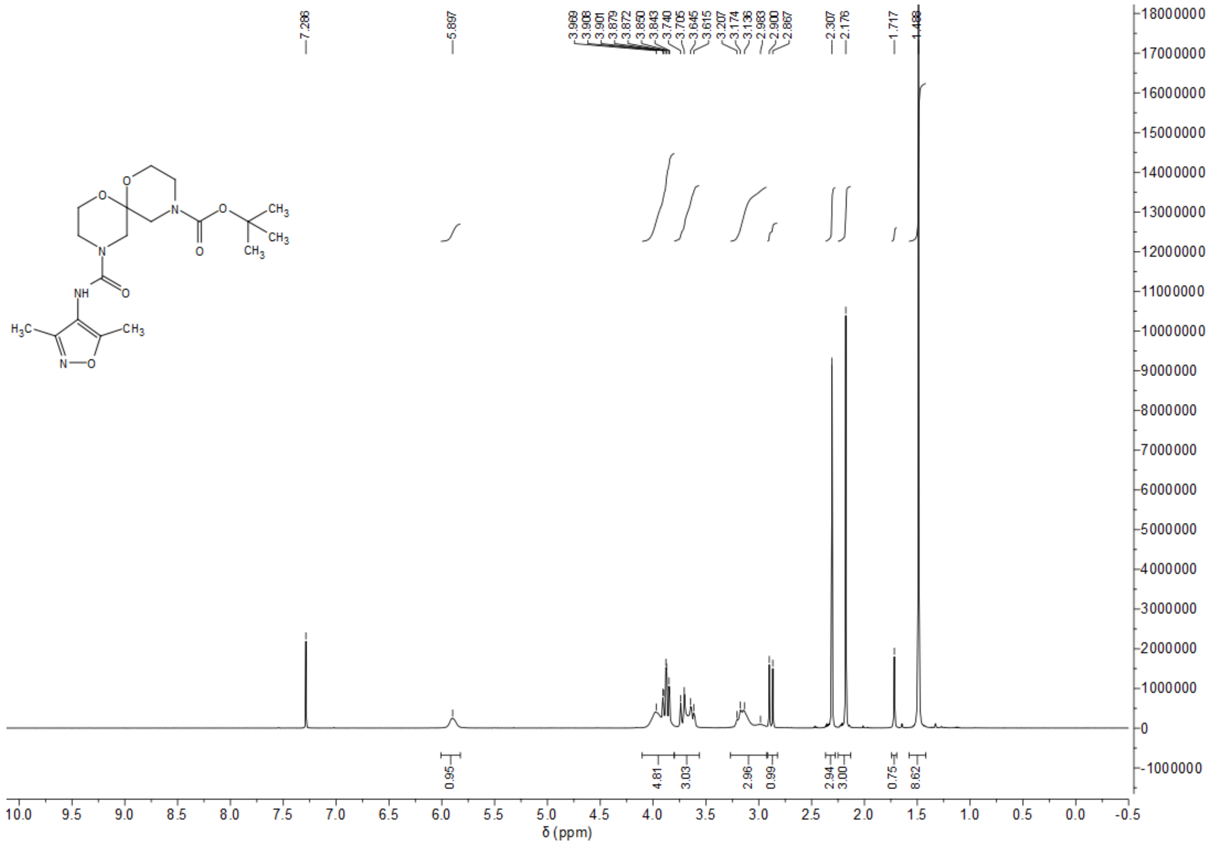

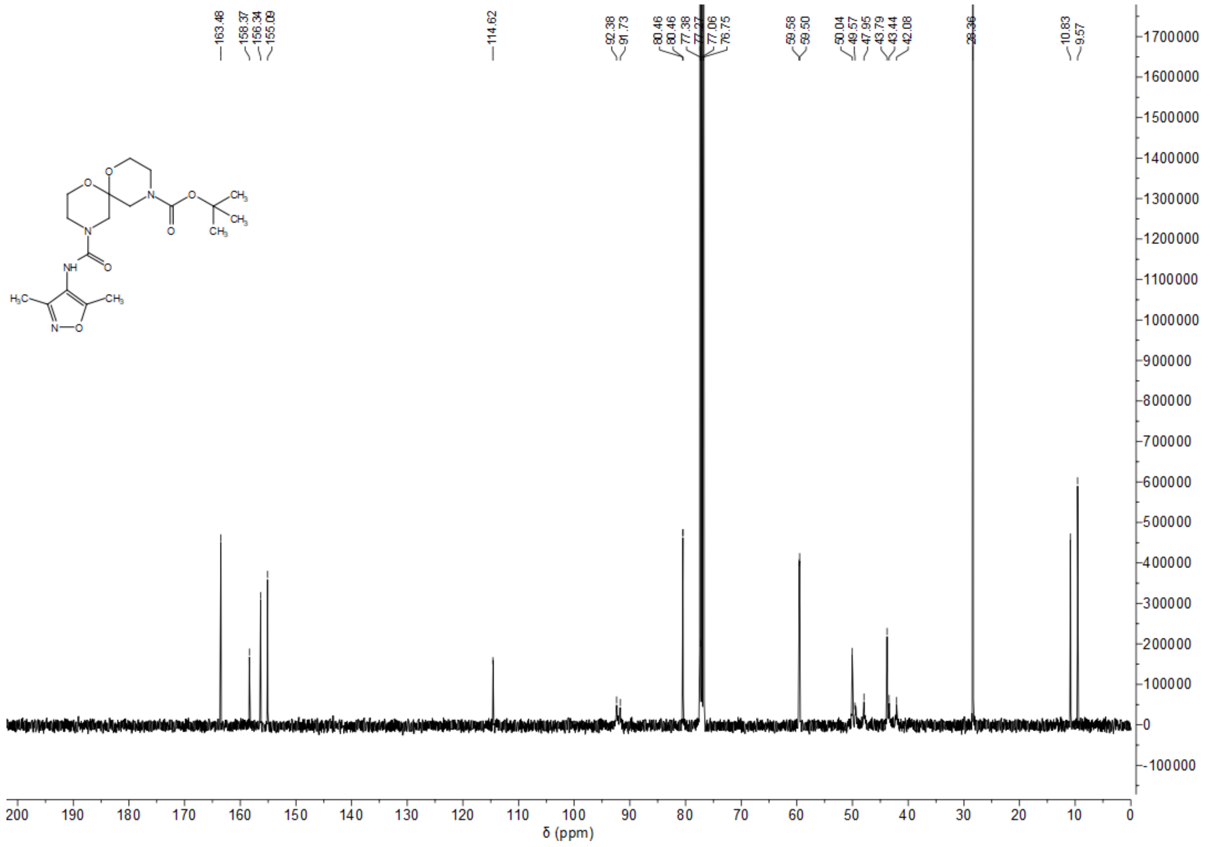

***N*-(3,5-dimethylisoxazol-4-yl)-1,7-dioxa-4,10-diazaspiro[5.5]undecane-4-carboxamide (L1-I08) [^{1}H-NMR data: 400 MHz, CD_3OD; ^{13}C{^{1}H}-NMR data: 101 MHz, CD_3OD]:**

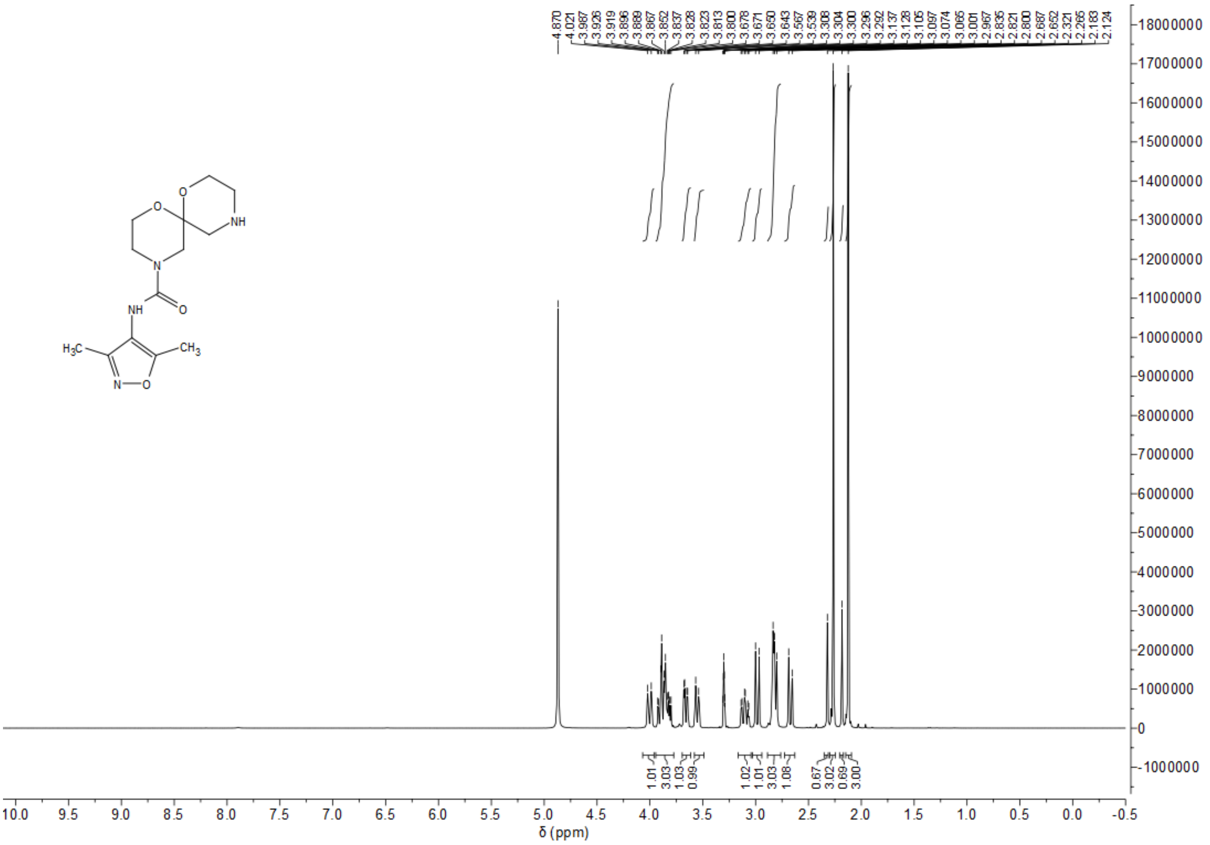

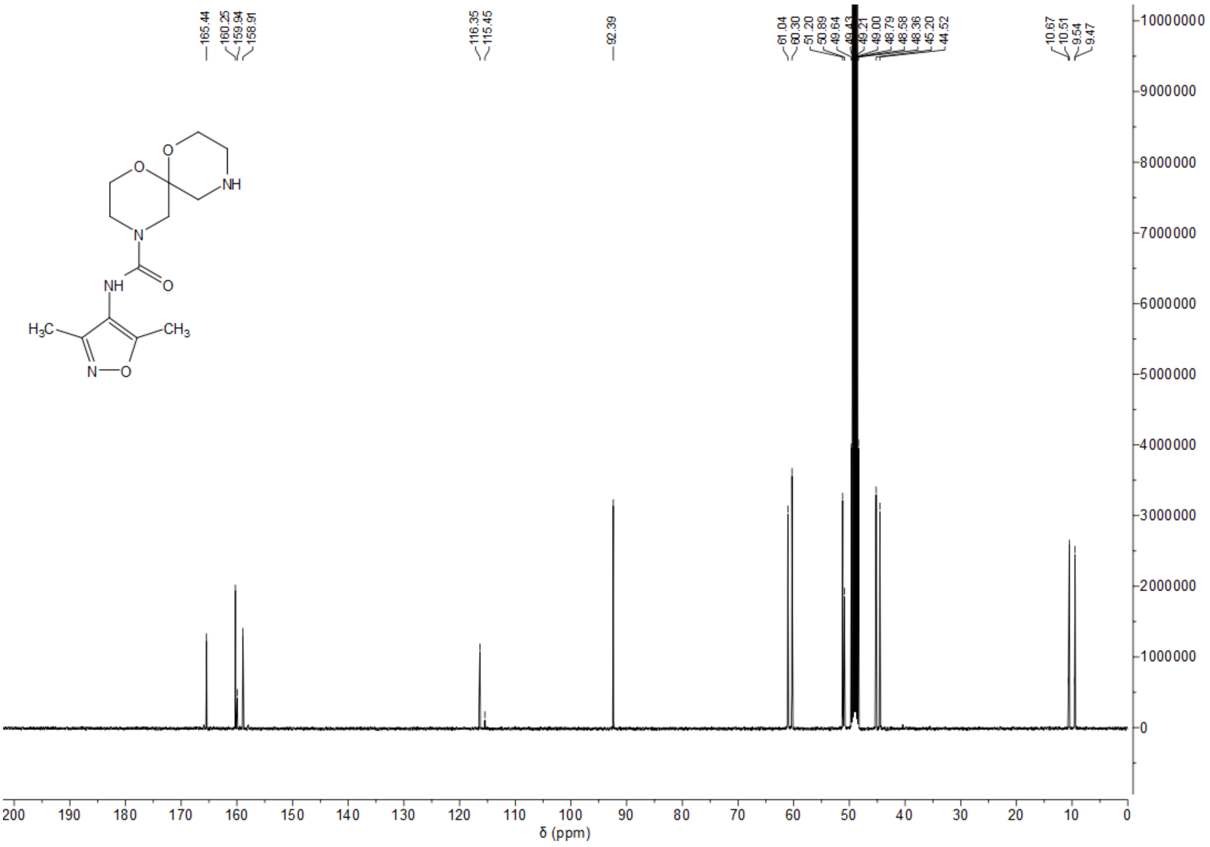

***N*[4],*N*[10]-bis(3,5-dimethylisoxazol-4-yl)-1,7-dioxa-4,10-diazaspiro[5.5]undecane-4,10-dicarboxamide (L1-I08-I08) [^{1}H-NMR data: 400 MHz, CD_3OD; $^{13}C\{^1H\}$-NMR data: 101 MHz, CD_3OD]:**

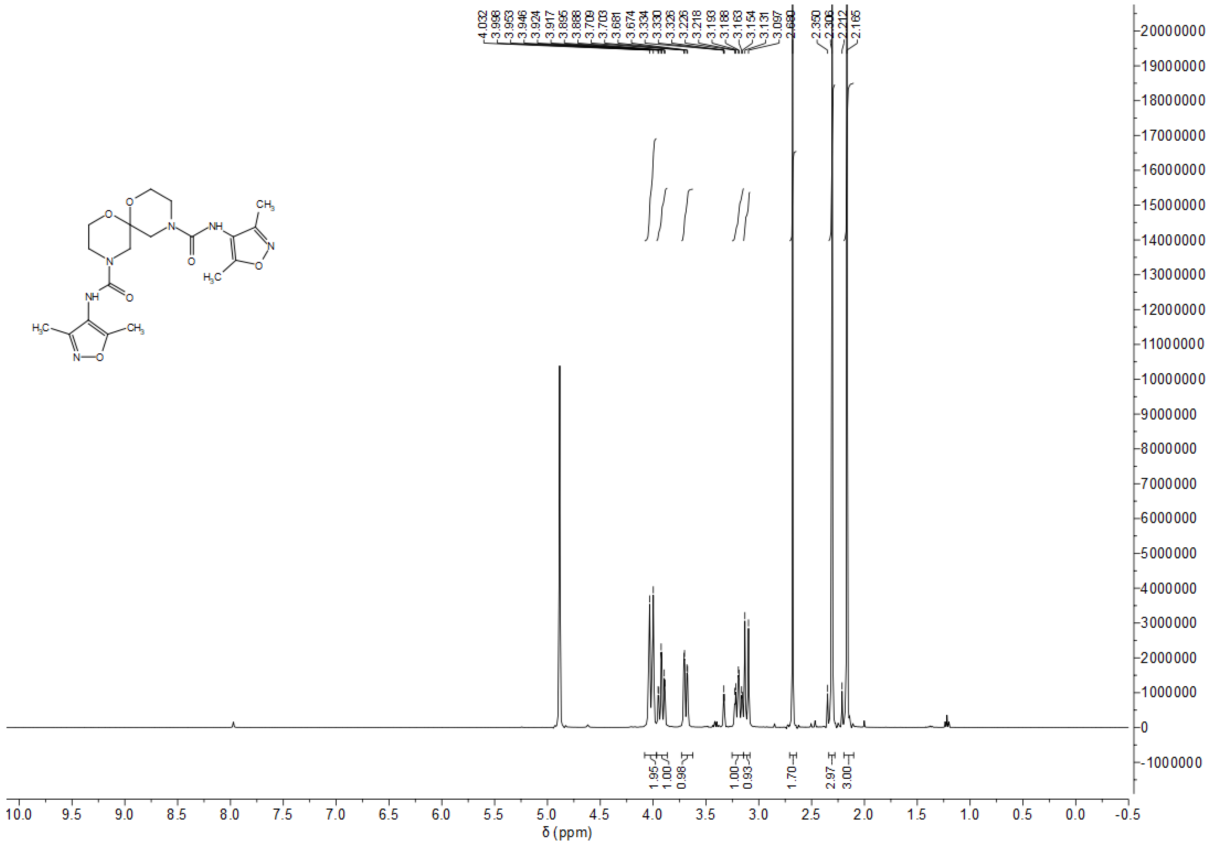

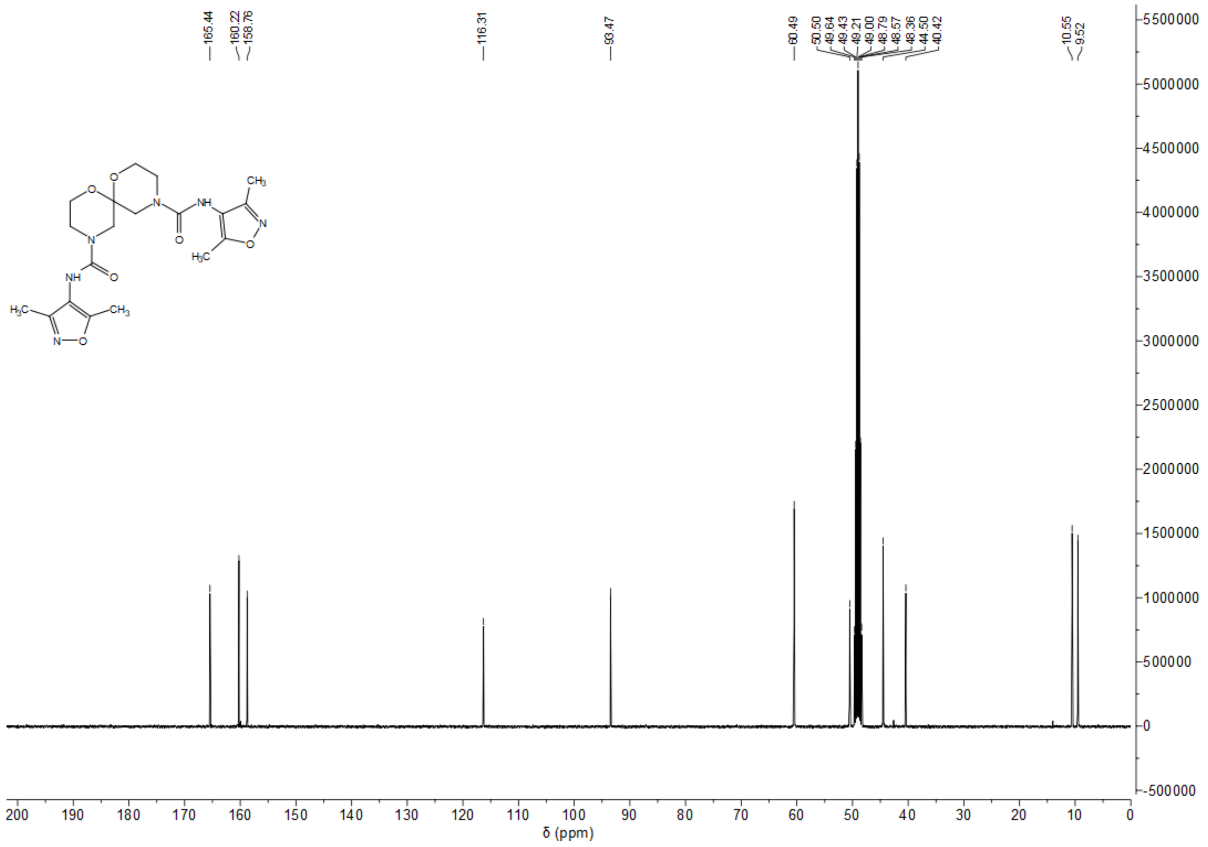

10-(3-chloro-4-(trifluoromethoxy)benzyl)-*N*-(3,5-dimethylisoxazol-4-yl)-1,7-dioxa-4,10-diazaspiro[5.5]undecane-4-carboxamide (L1-I08-A09) [^{1}H-NMR data: 400 MHz, CD_3OD; $^{13}C\{^{1}H\}$-NMR data: 101 MHz, CD_3OD]:

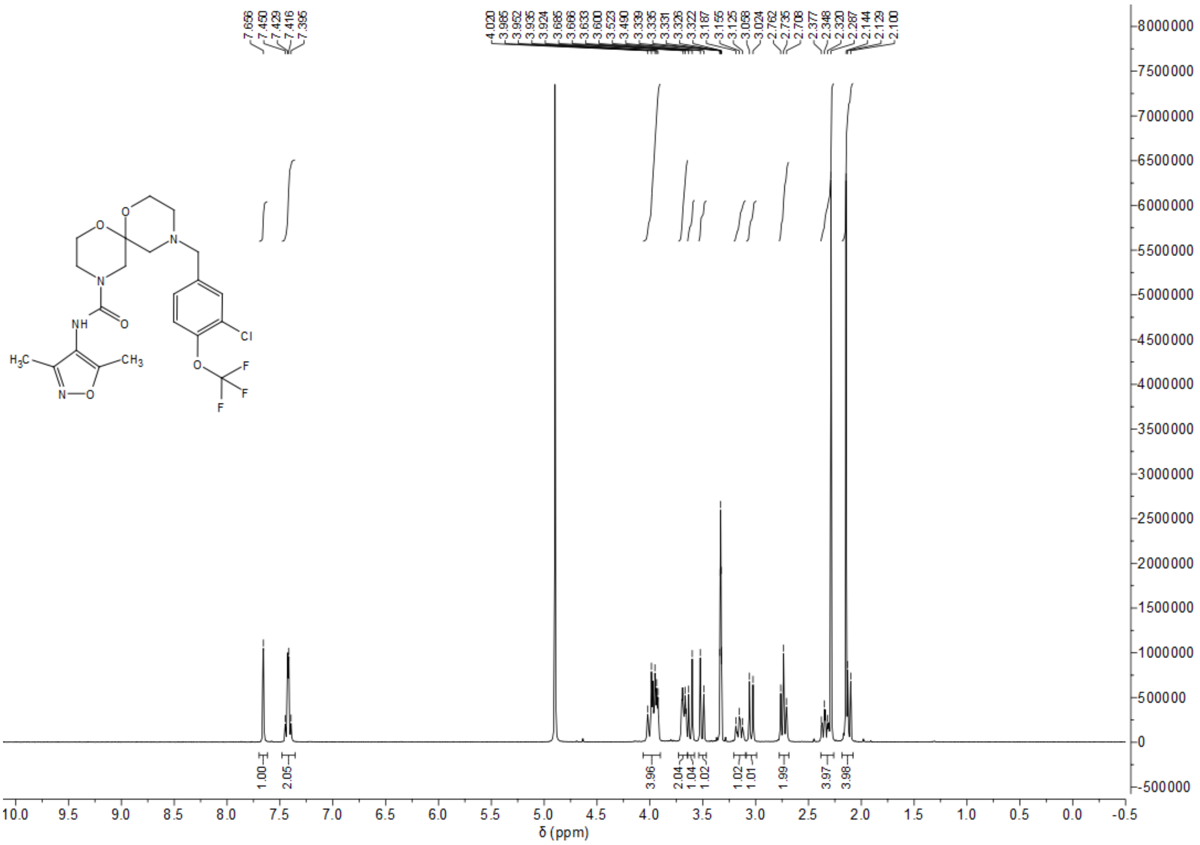

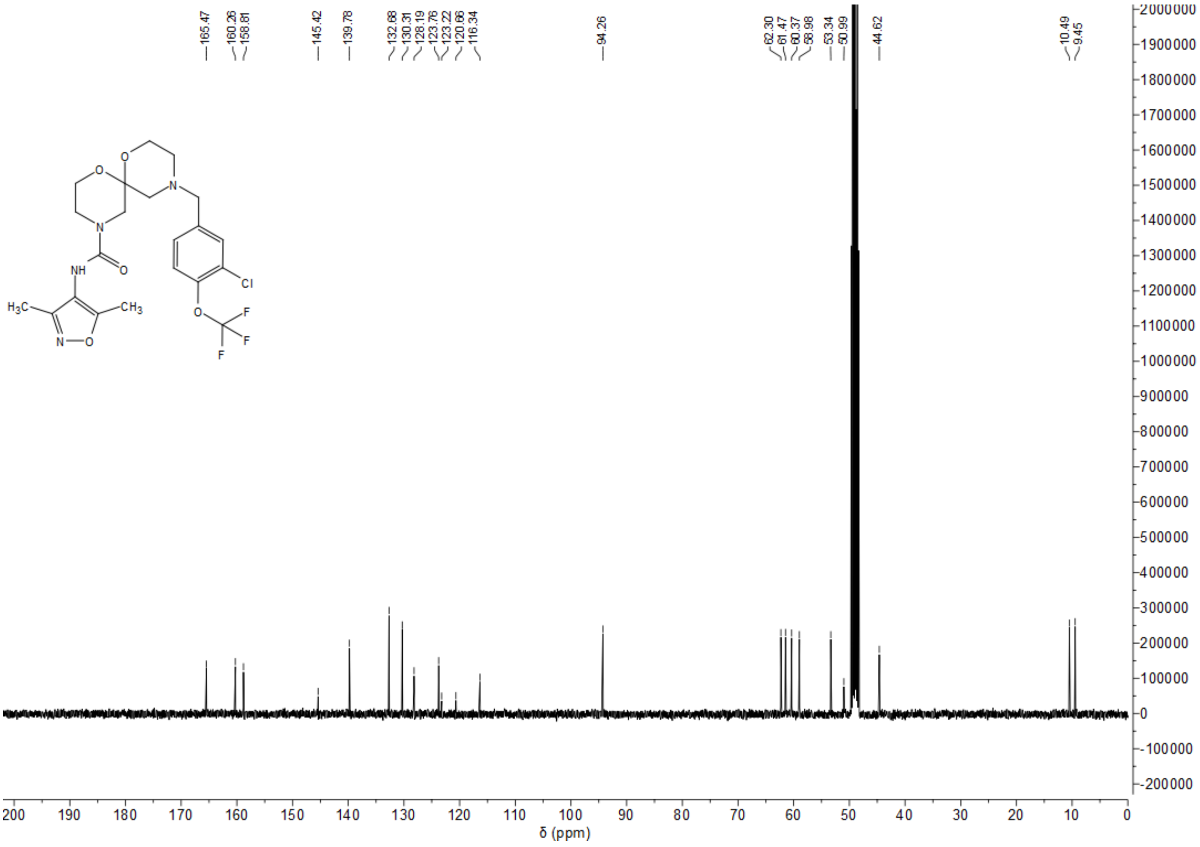

***tert*-butyl 4-((2,4-difluorophenyl)carbamoyl)-1,7-dioxa-4,11-diazaspiro[5.6]dodecane-11-carboxylate (25-I03) [^{1}H-NMR data: 400 MHz, $CDCl_3$; $^{13}C\{^{1}H\}$-NMR data: 101 MHz, $CDCl_3$; ^{19}F-NMR data: 376 MHz, $CDCl_3$; 2D NMR spectra: HSQC, HMBC, COSY, all in $CDCl_3$]:**

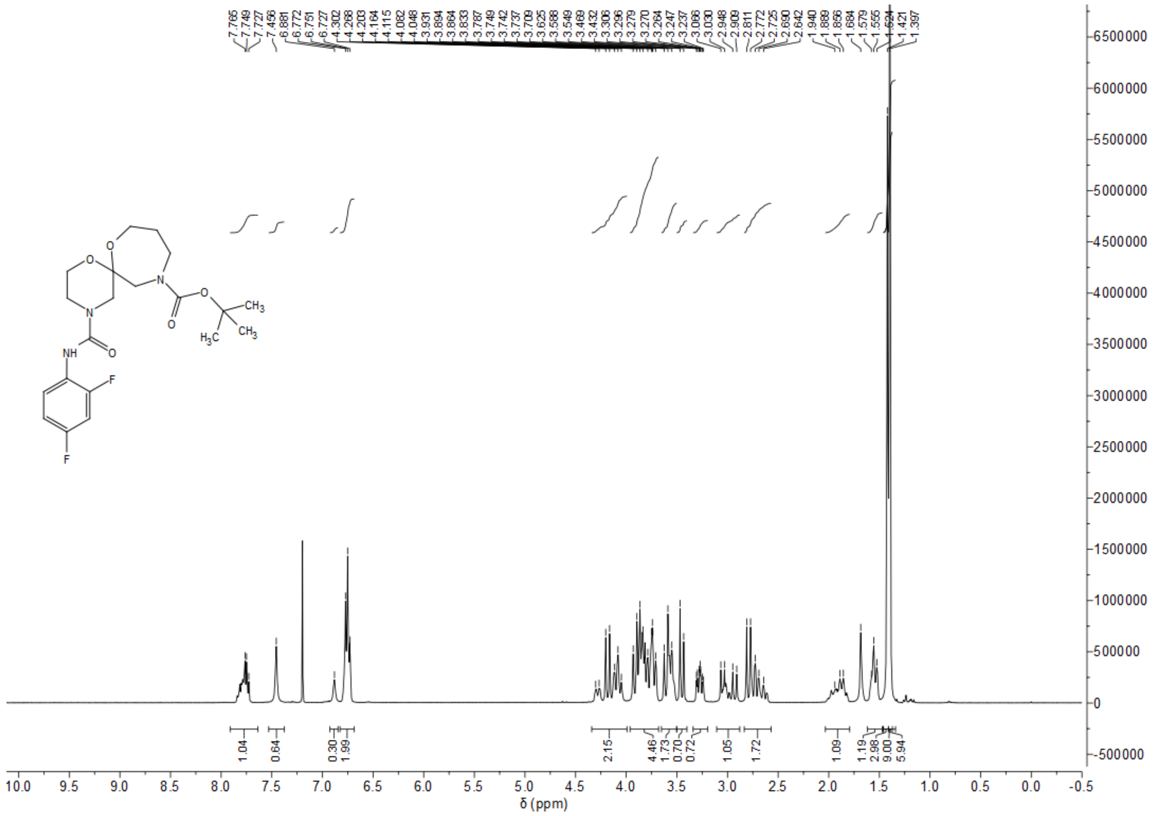

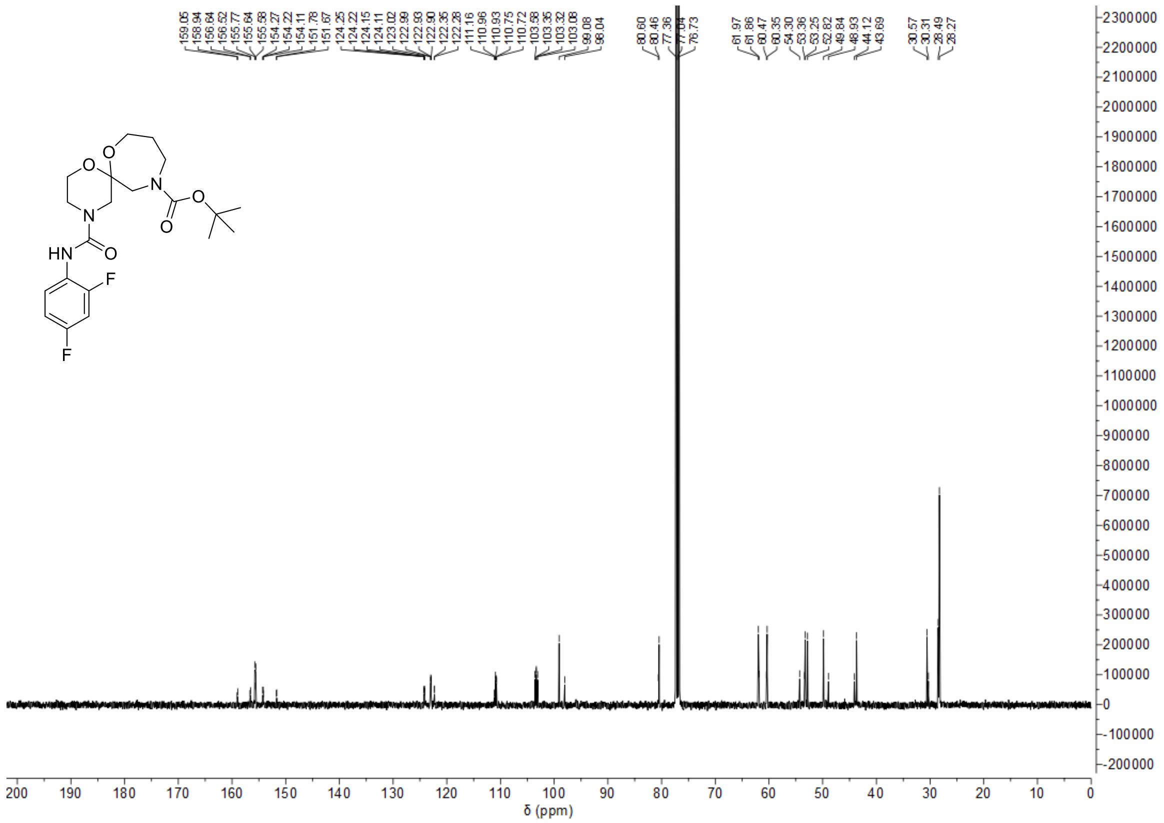

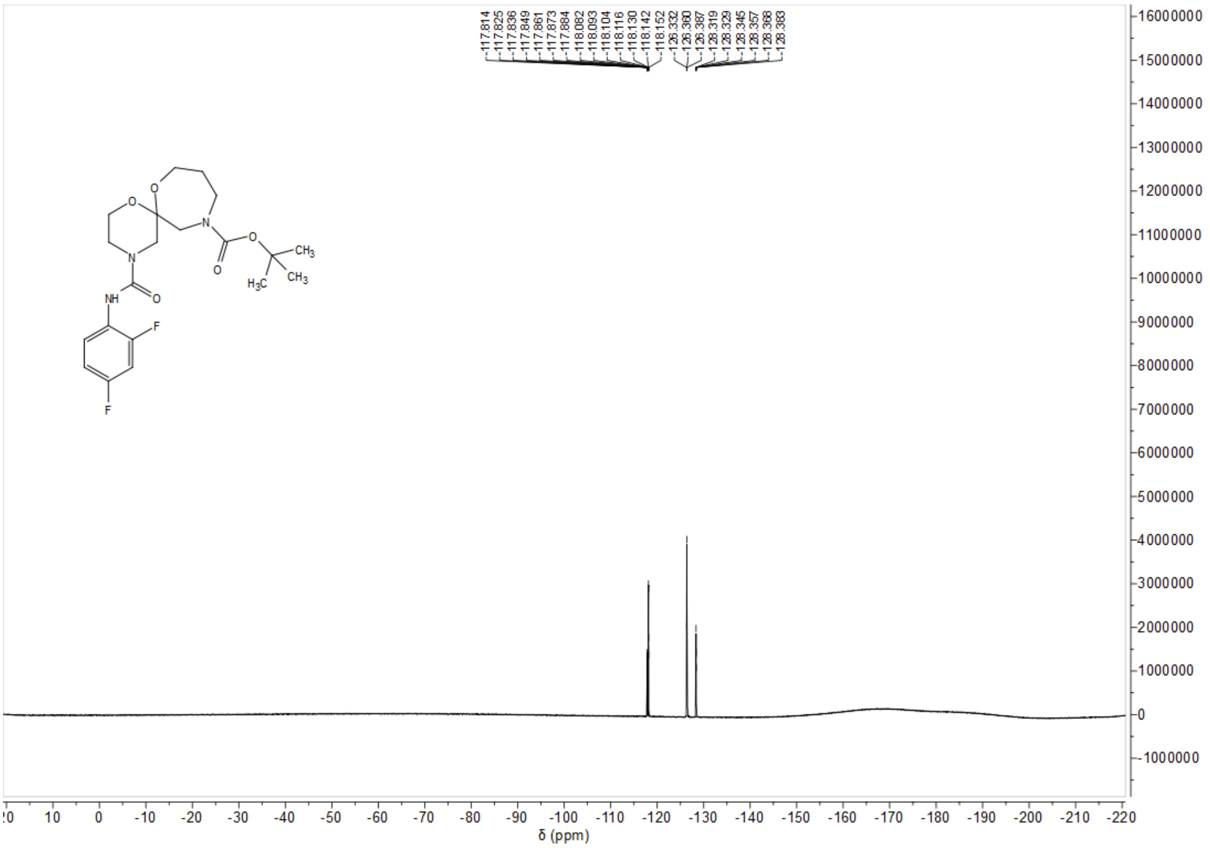

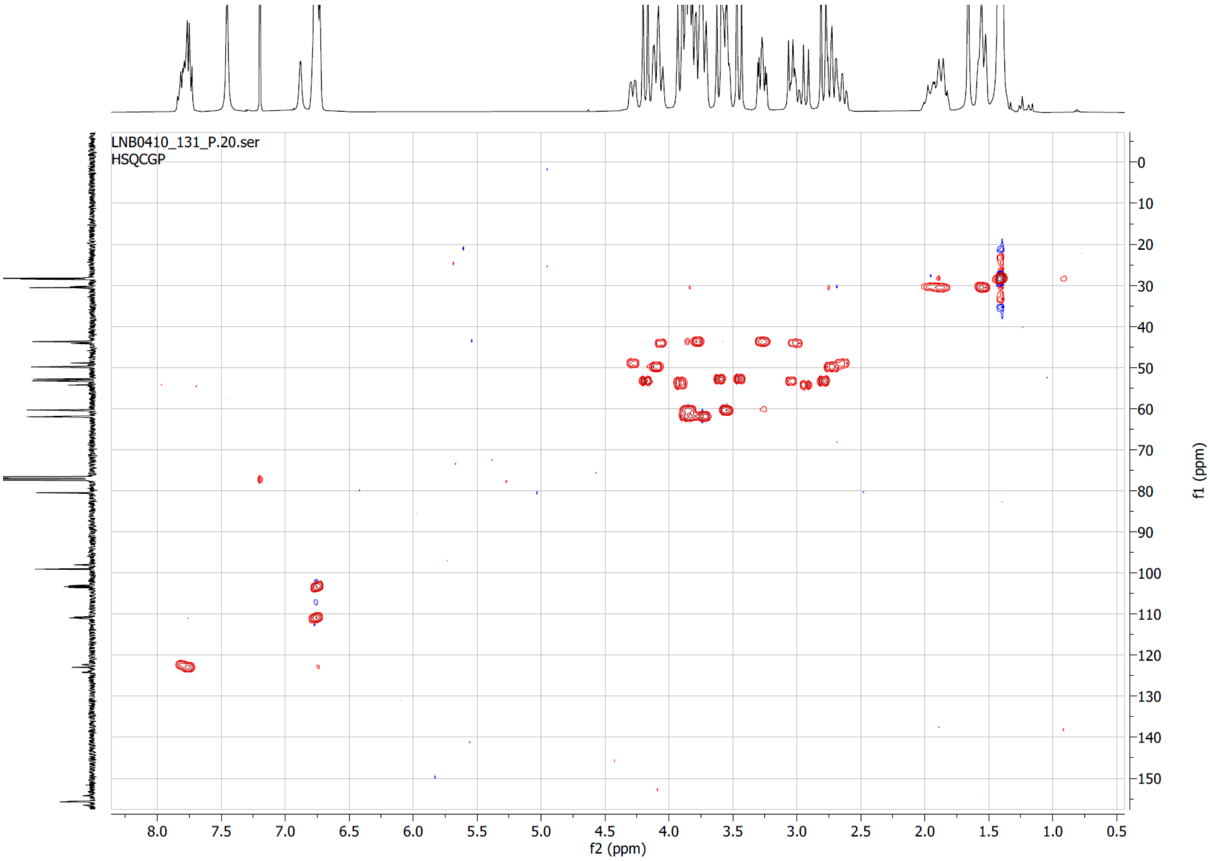
LNB0410_131_P.20.ser
HSQCGP
f2 (ppm)
f1 (ppm)

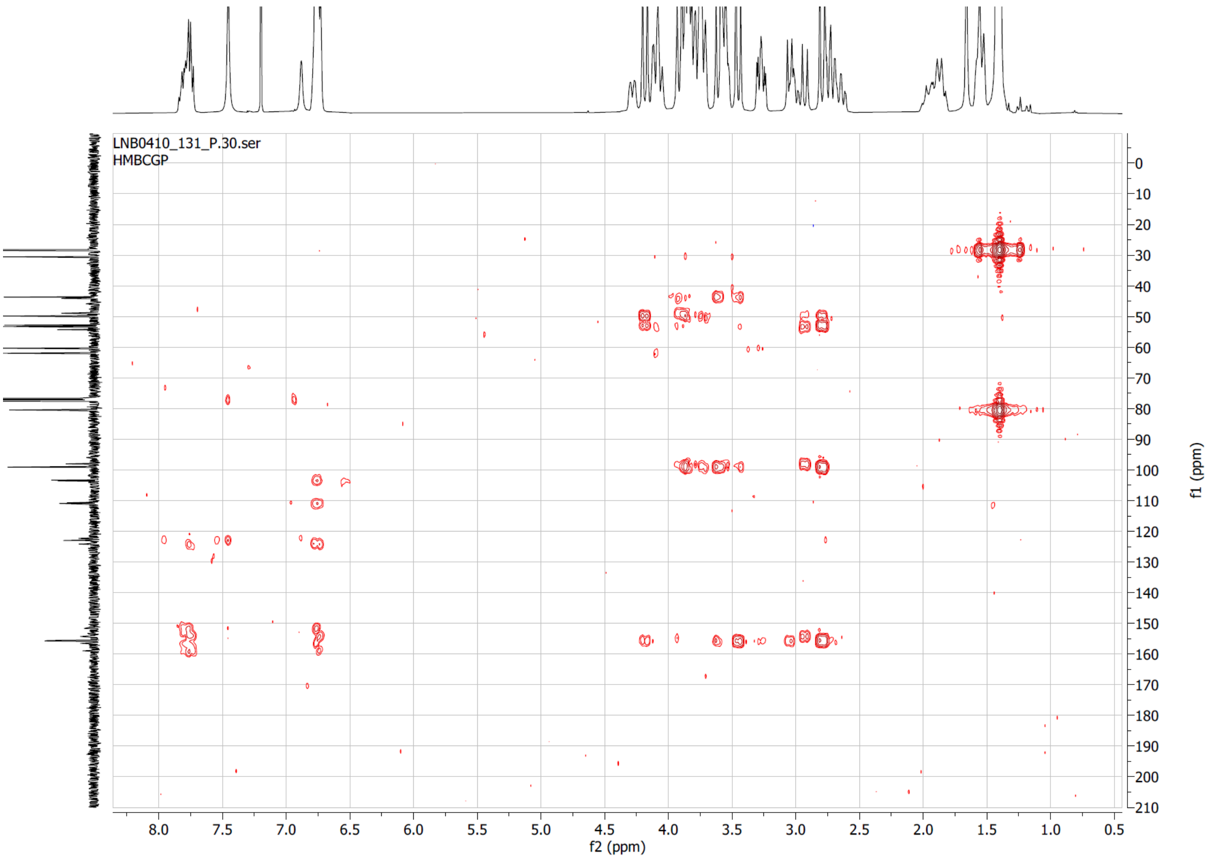
LNB0410_131_P.30.ser
HMBCGP
f2 (ppm)
f1 (ppm)

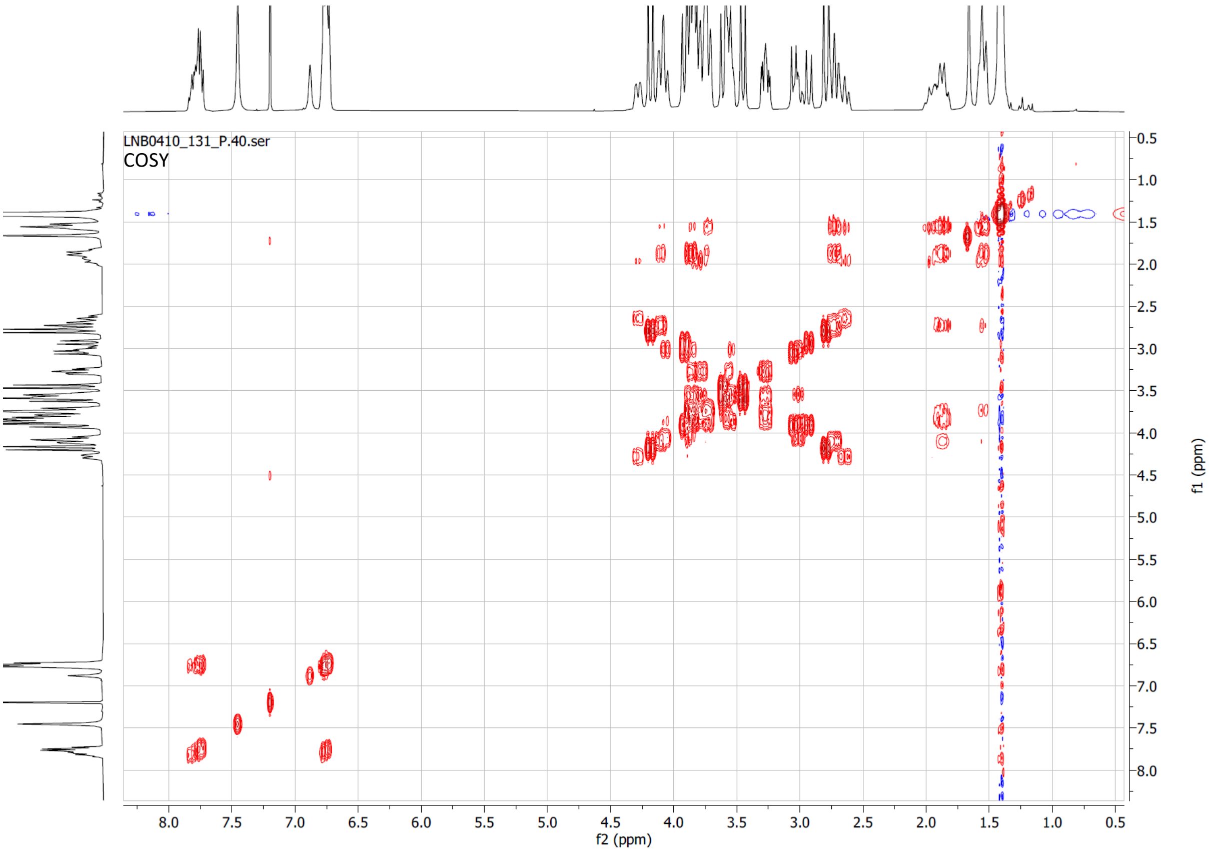
LNB0410_131_P.40.ser
COSY
f2 (ppm)
f1 (ppm)

***N*-(2,4-difluorophenyl)-1,7-dioxa-4,11-diazaspiro[5.6]dodecane-4-carboxamide hydrochloride (L2-I03·HCl *or* 31·HCl in the main article) [^{1}H-NMR data: 400 MHz, D_2O; $^{13}C\{^{1}H\}$-NMR data: 101 MHz, D_2O; ^{19}F-NMR data: 376 MHz, D_2O]:**

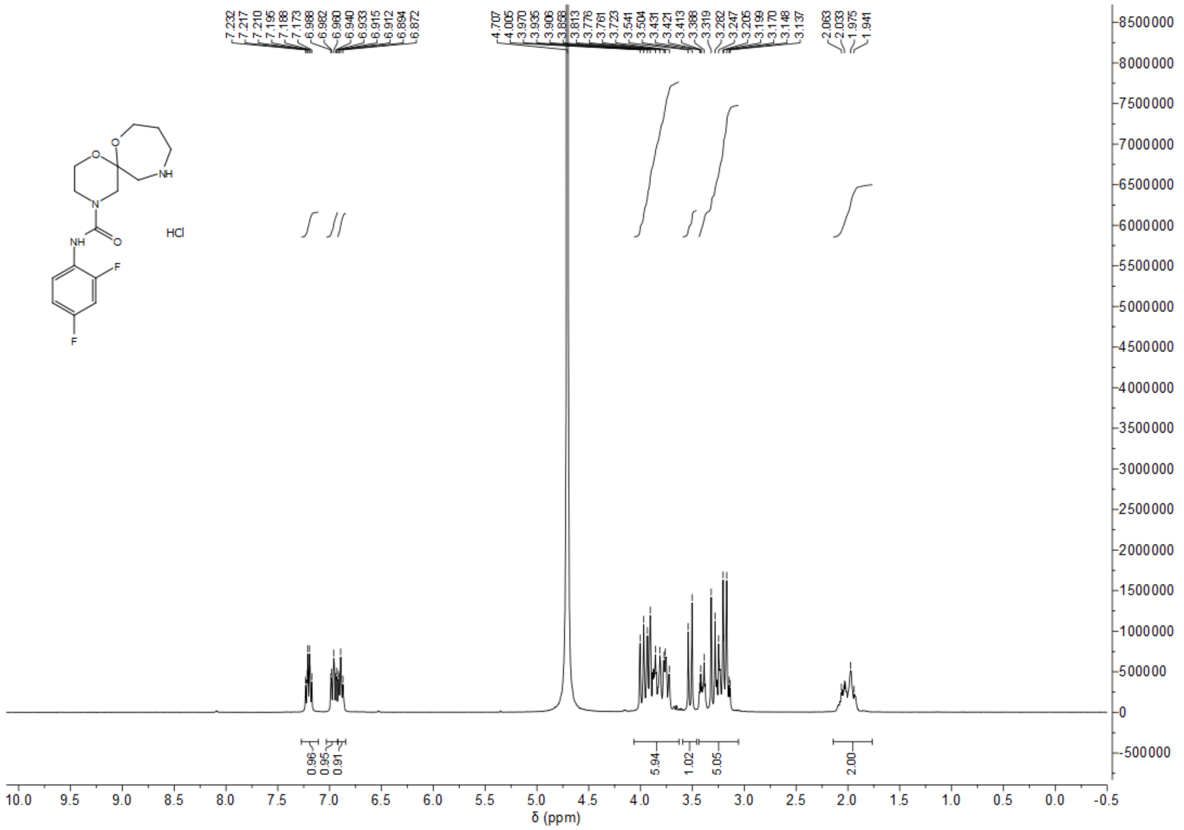

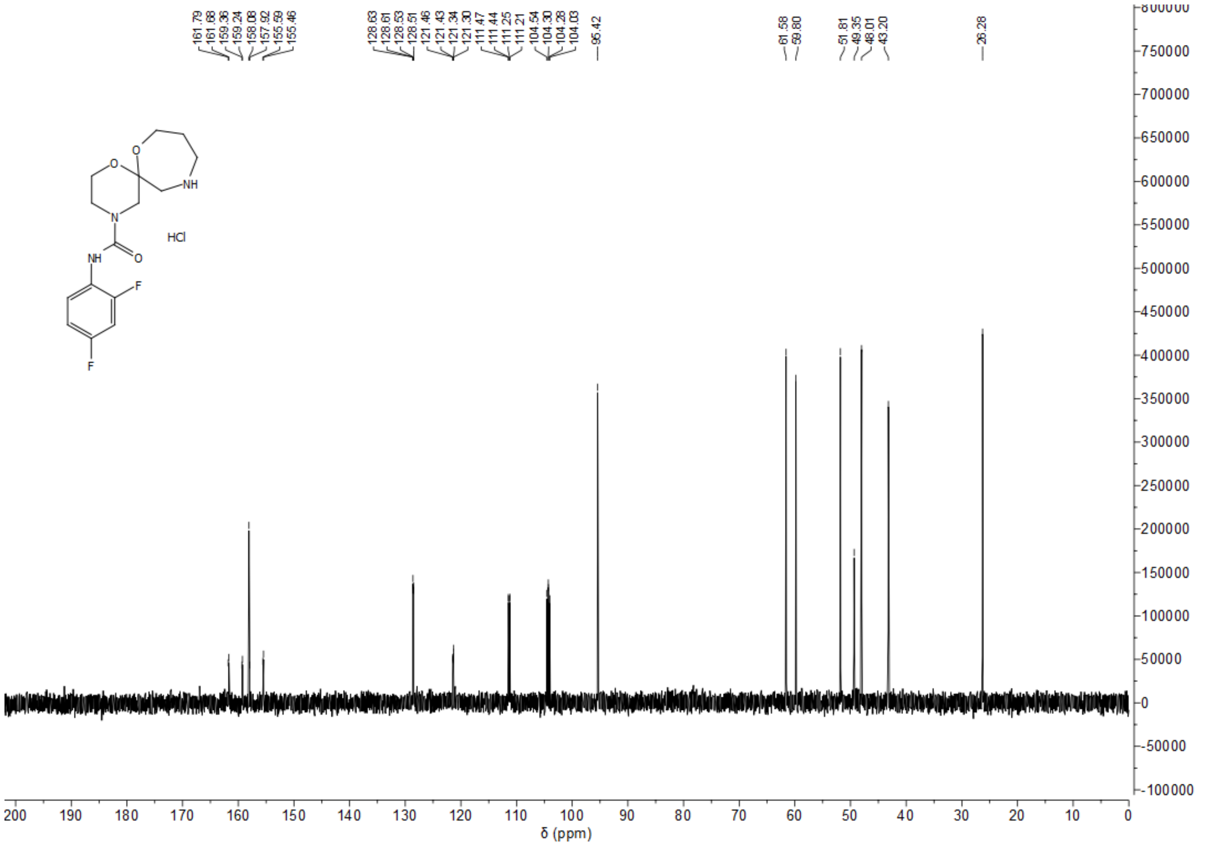

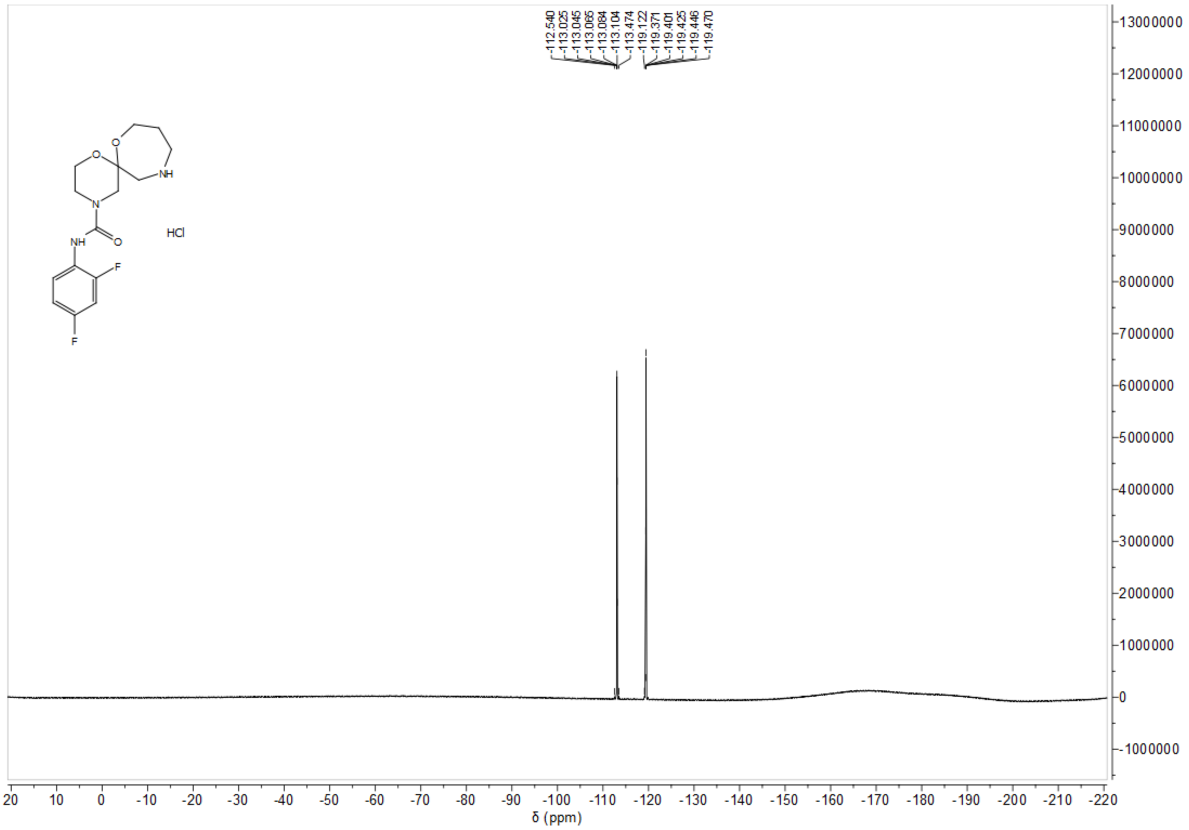
-112.540
-113.025
-113.045
-113.065
-113.084
-113.104
-113.474
-119.122
-119.371
-119.401
-119.425
-119.446
-119.470
HCl
δ (ppm)

***N*-(2,4-difluorophenyl)-11-((3-phenylisoxazol-5-yl)methyl)-1,7-dioxa-4,11-diazaspiro [5.6]dodecane-4-carboxamide (L2-I03-A05) [^{1}H-NMR data: 400 MHz, CD_3OD; ^{13}C{^{1}H}-NMR data: 101 MHz, CD_3OD]:**

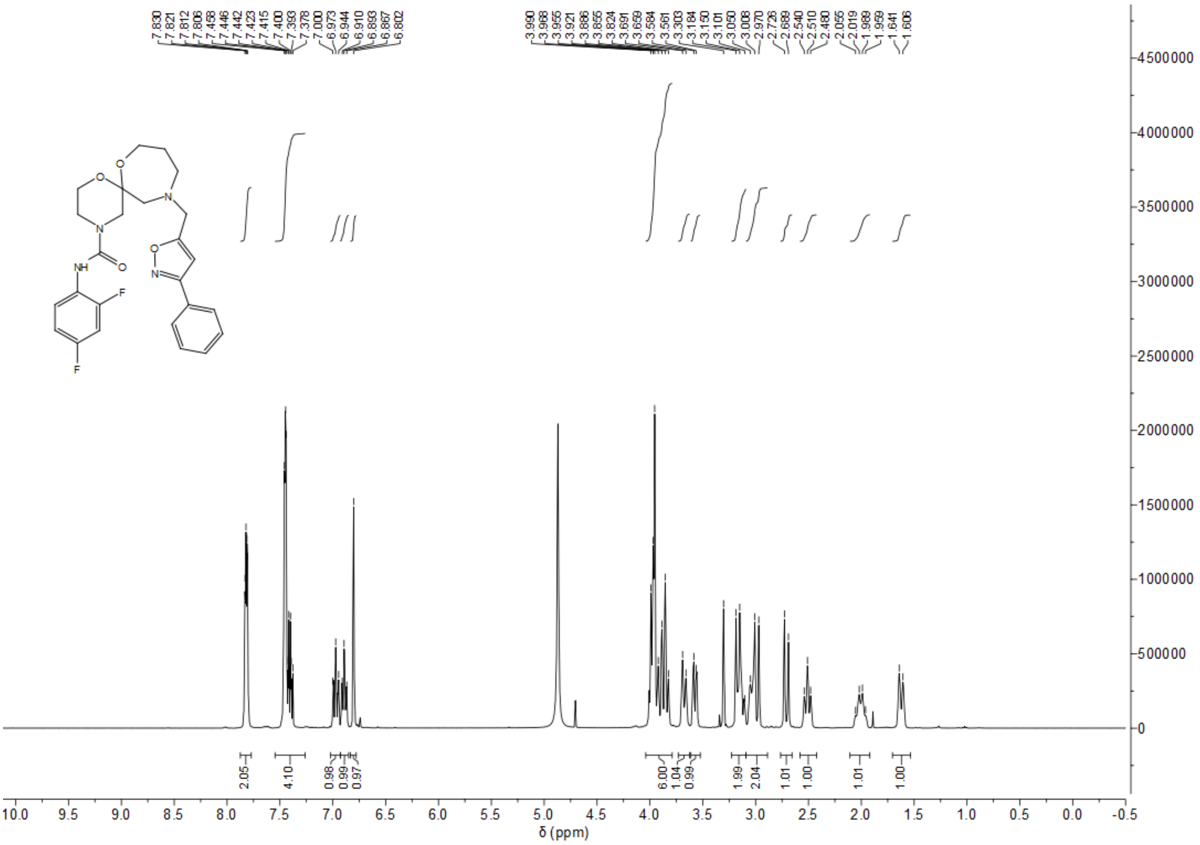

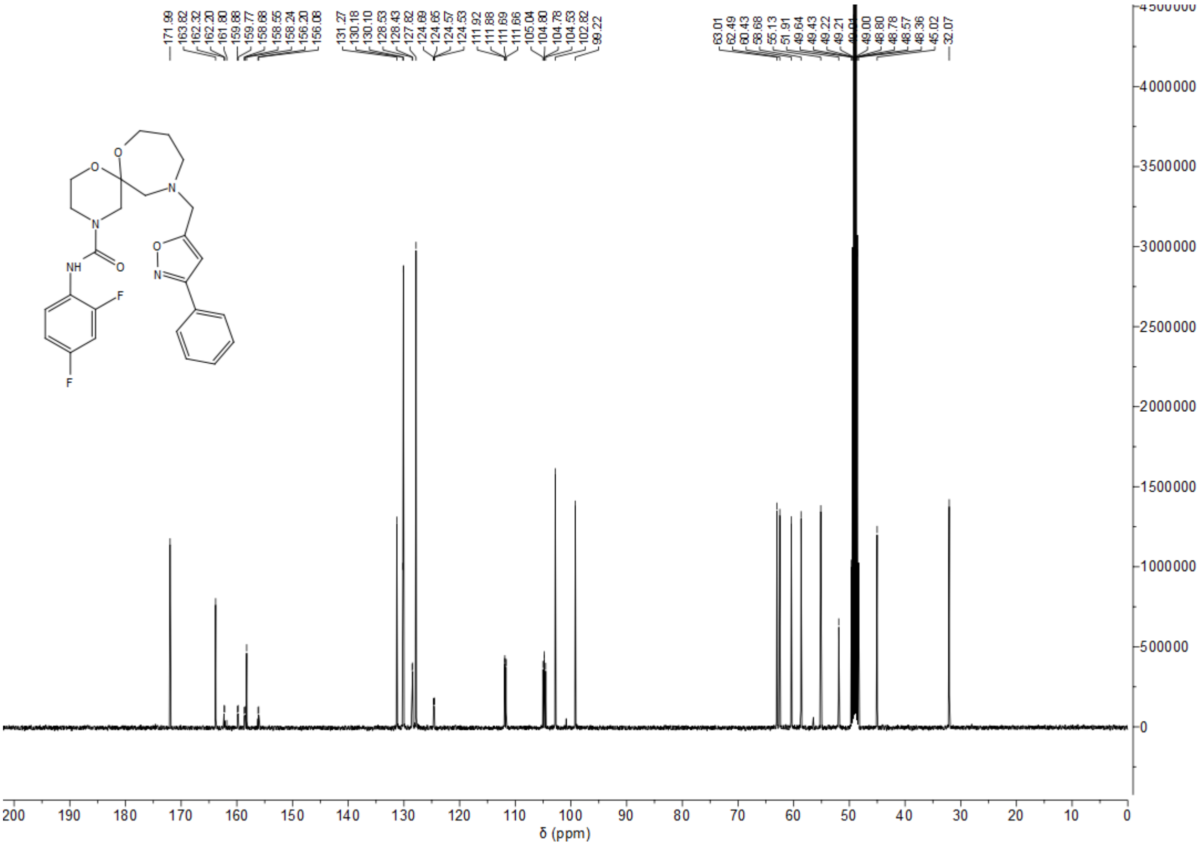

***N*-(2,4-difluorophenyl)-11-((1,3,5-trimethyl-1*H*-pyrazol-4-yl)sulfonyl)-1,7-dioxa-4,11-diazaspiro[5.6]dodecane-4-carboxamide (L2-I03-B10) [^{1}H-NMR data: 400 MHz, CD_3OD; $^{13}C\{^1H\}$-NMR data: 101 MHz, CD_3OD]:**

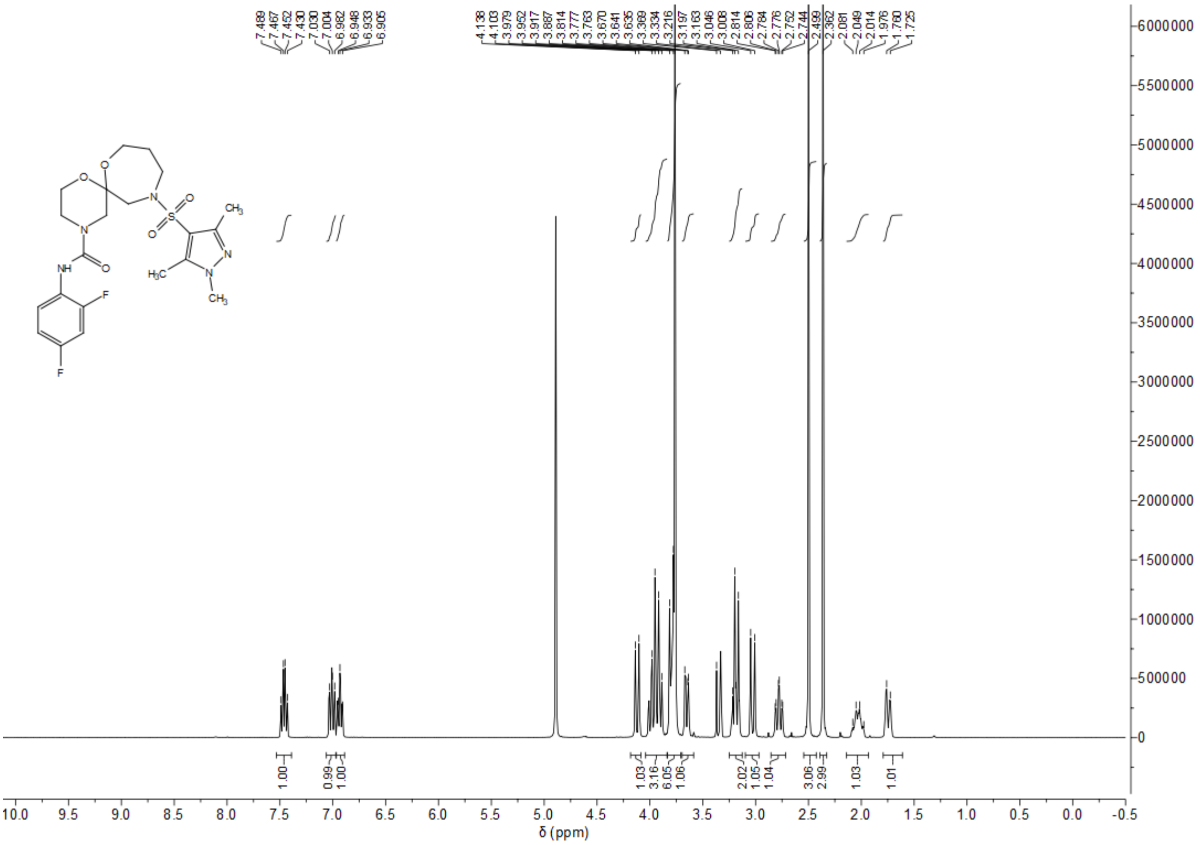

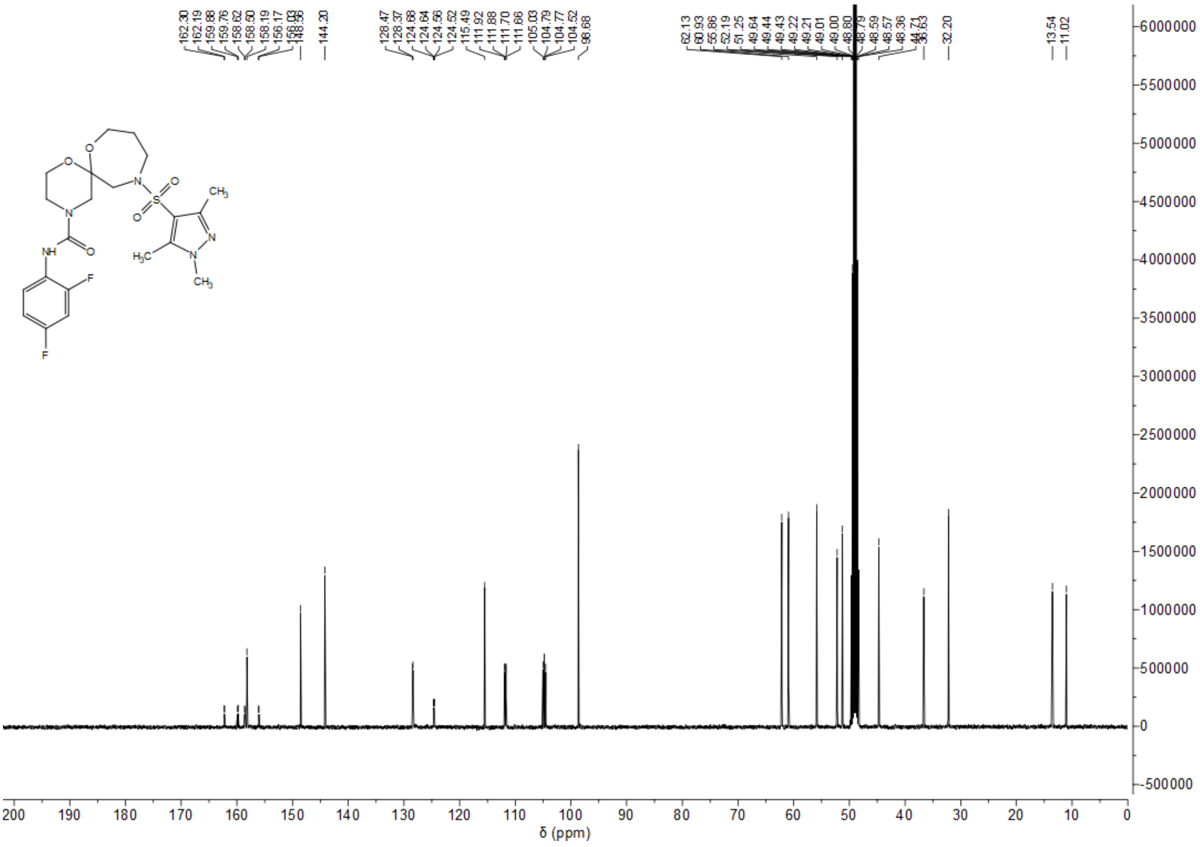

N-(2,4-difluorophenyl)-11-(2-methylthiazole-4-carbonyl)-1,7-dioxa-4,11-diazaspiro[5.6]dodecane-4-carboxamide (L2-I03-C42) [^{1}H-NMR data: 400 MHz, CD_3OD; $^{13}C\{^{1}H\}$-NMR data: 101 MHz, CD_3OD]:

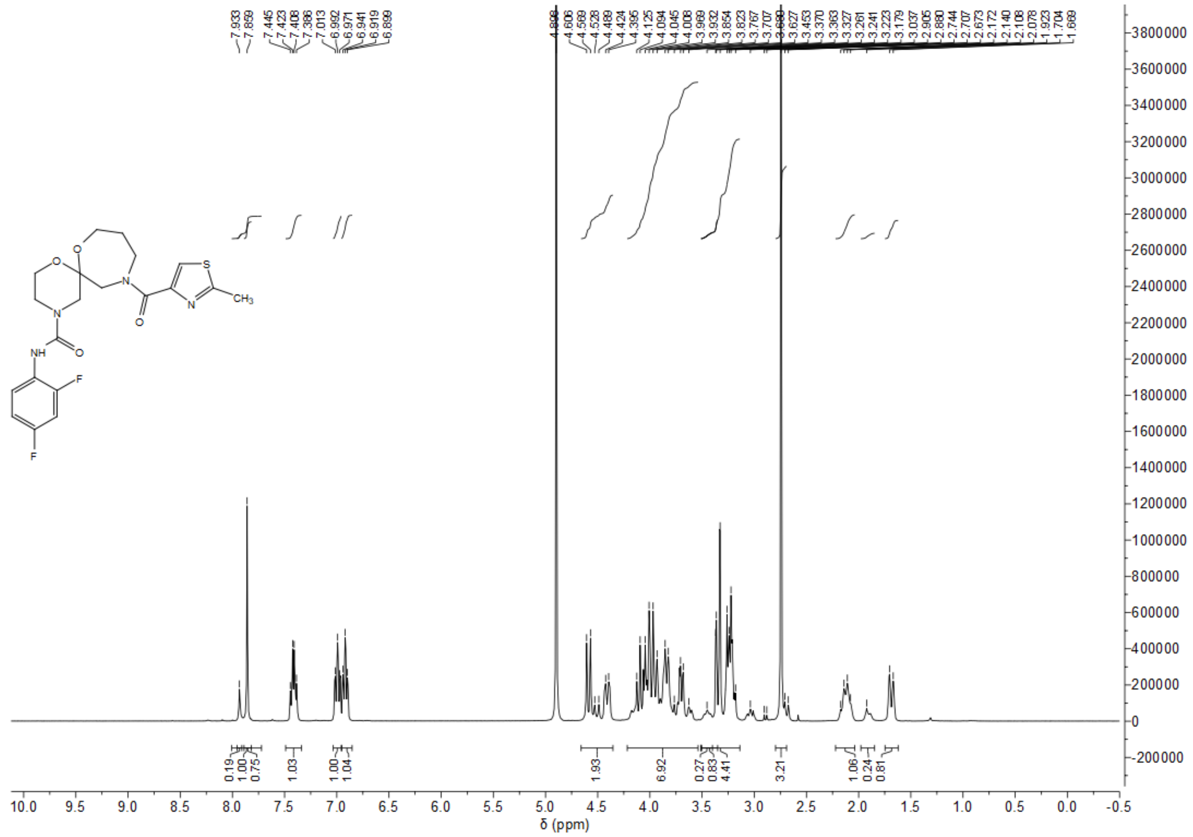

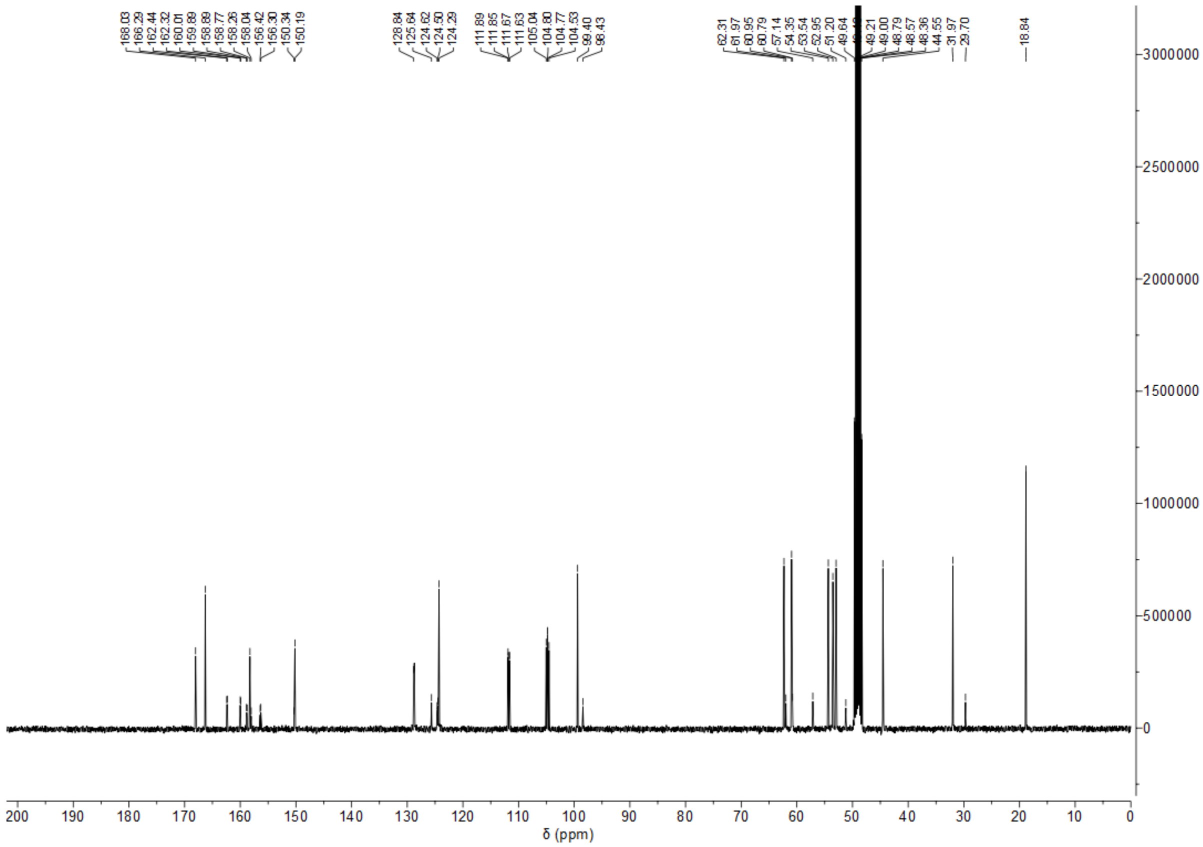

1.6.2 First decoration step – Sulfonylations

benzyl 10-(pyridin-3-ylsulfonyl)-1,7-dioxa-4,10-diazaspiro[5.5]undecane-4-carboxylate (10-B08 *or* 12 in the main article) [^{1}H-NMR data: 400 MHz, C_6D_6; ^{13}C{^{1}H}-NMR data: 101 MHz, C_6D_6]:

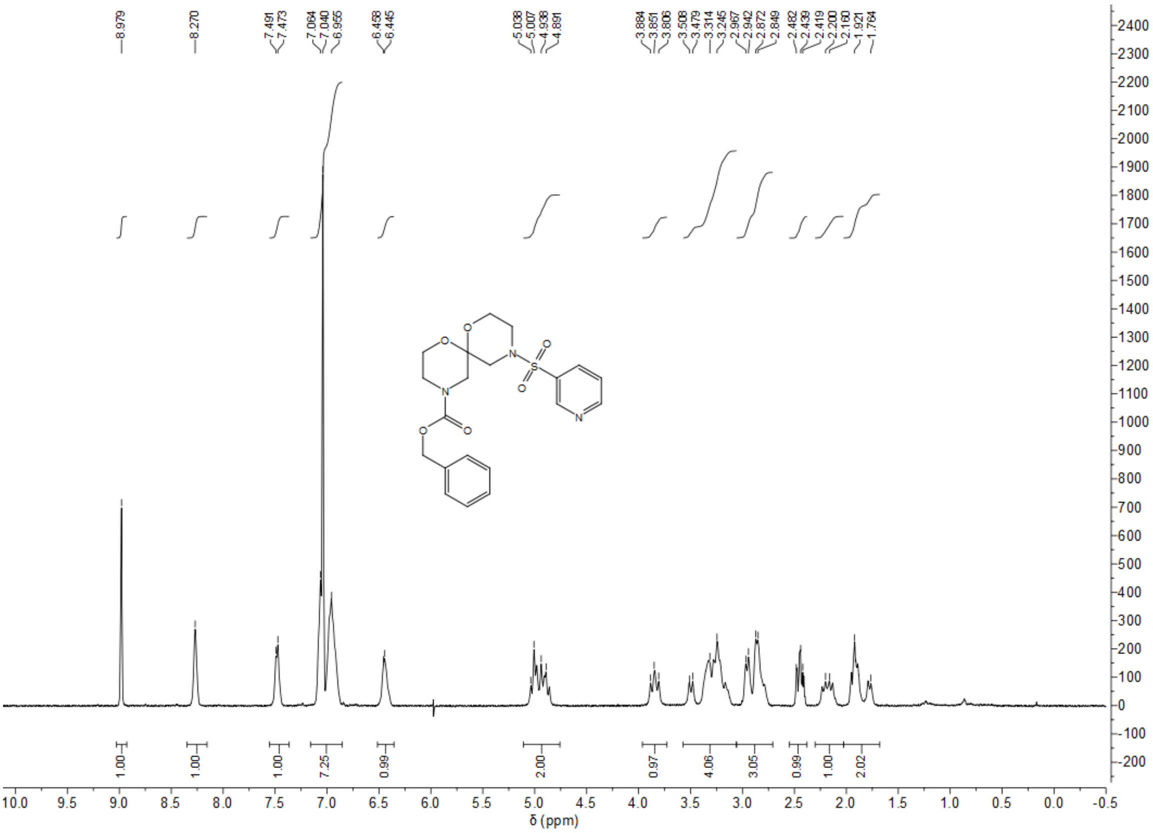

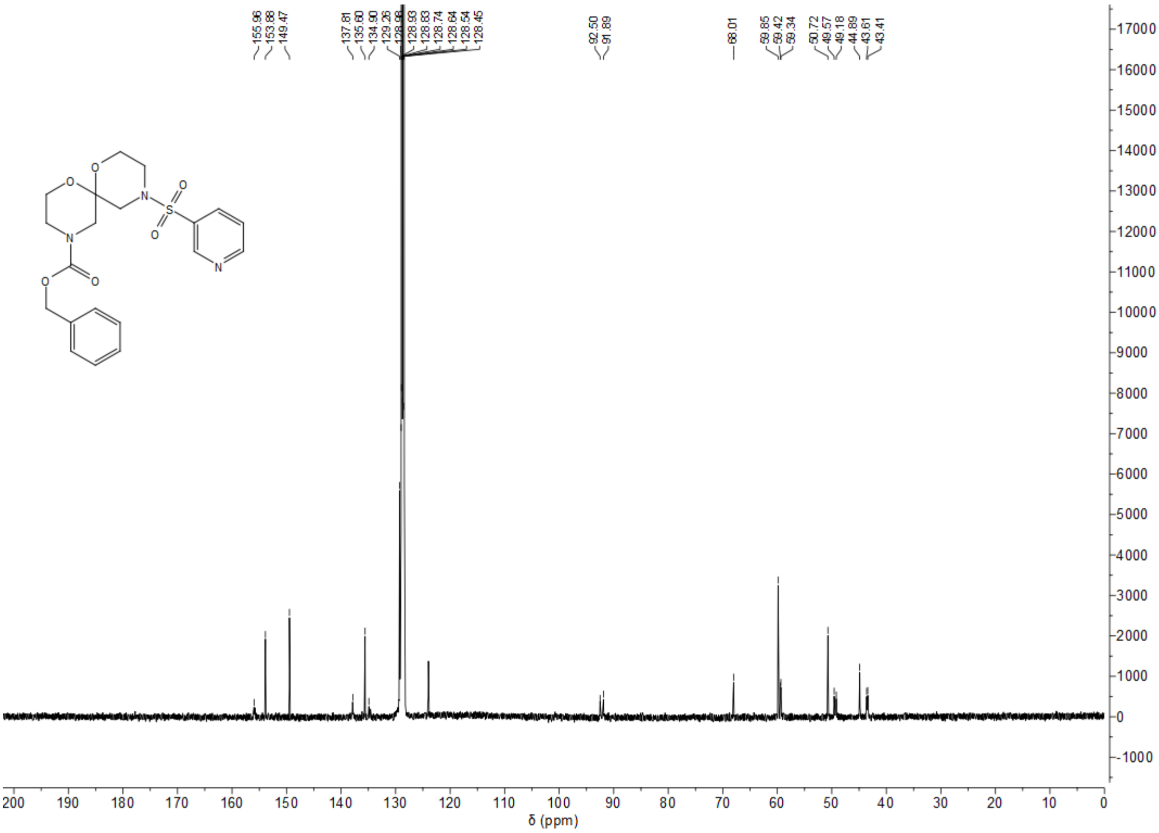

***tert*-butyl 10-(pyridin-3-ylsulfonyl)-1,7-dioxa-4,10-diazaspiro[5.5]undecane-4-carboxylate (9-B08 *or* 14 in the main article) [^{1}H-NMR data: 400 MHz, C_6D_6; ^{13}C{^{1}H}-NMR data: 101 MHz, C_6D_6]:**

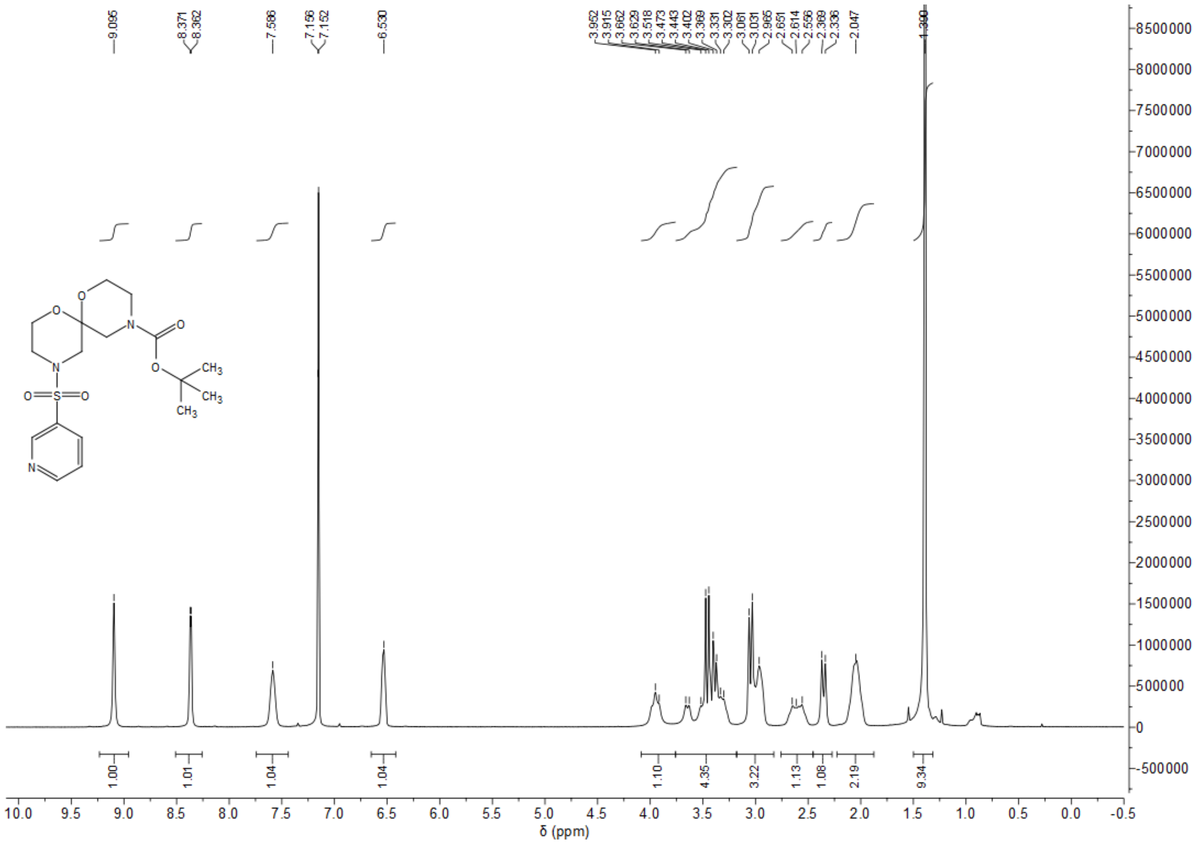

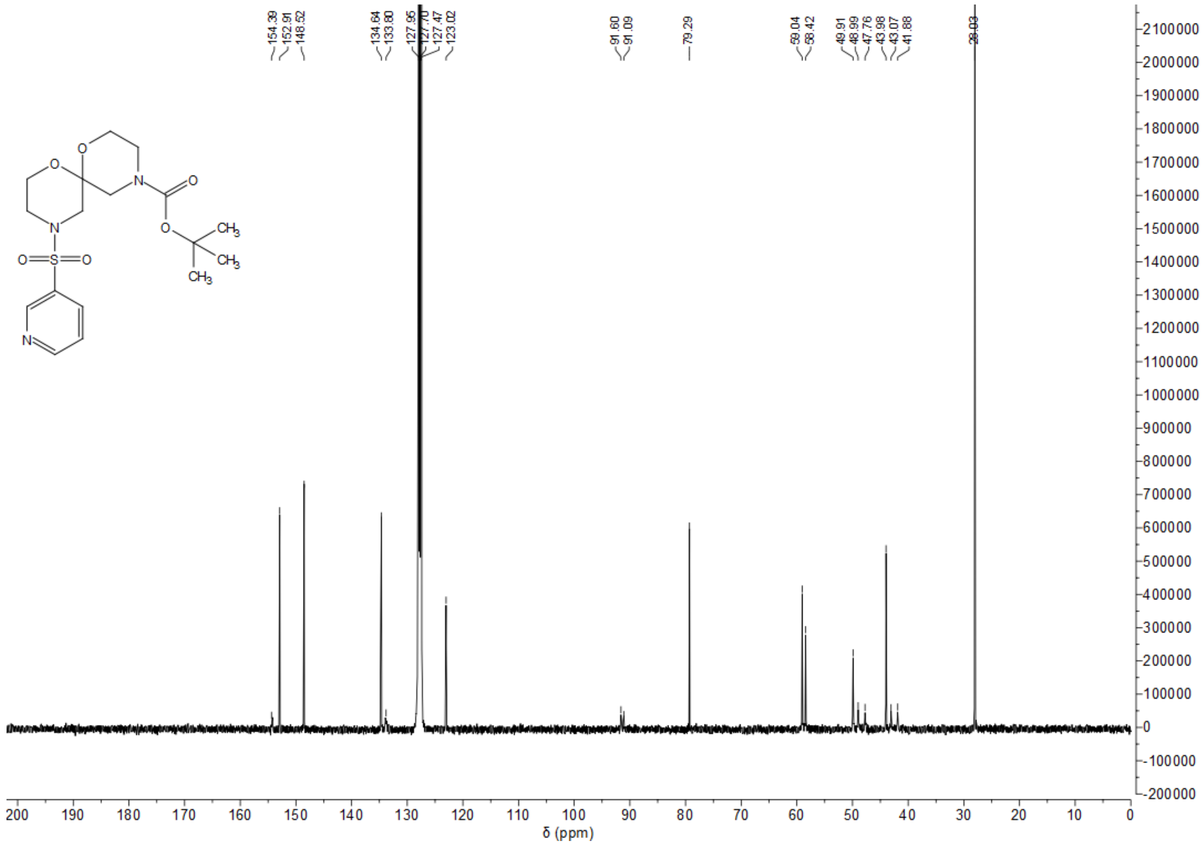

4-(pyridin-3-ylsulfonyl)-1,7-dioxa-4,10-diazaspiro[5.5]undecane dihydrochloride (L1-B08·2HCl *or* 16 in the main article) [^{1}H-NMR data: 400 MHz, CD_3OD; ^{13}C{^{1}H}-NMR data: 101 MHz, CD_3OD]:

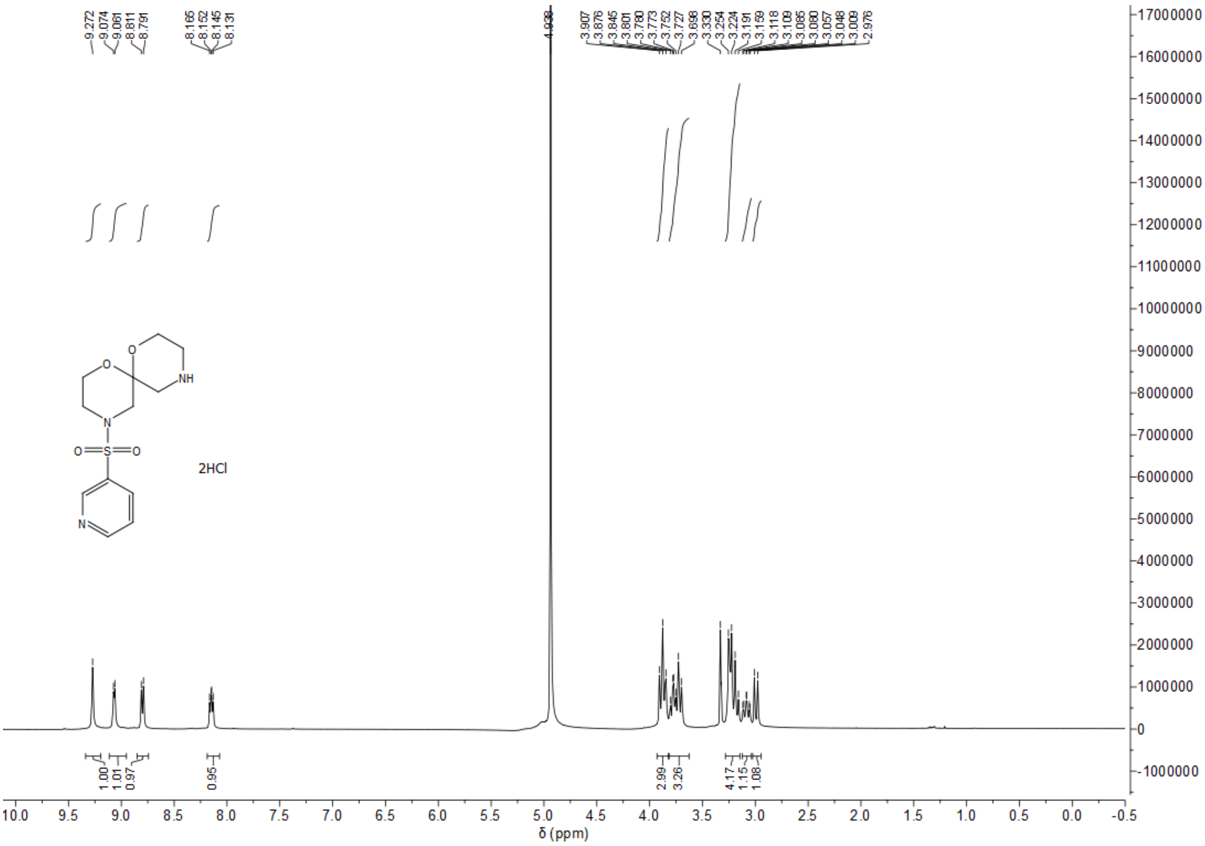

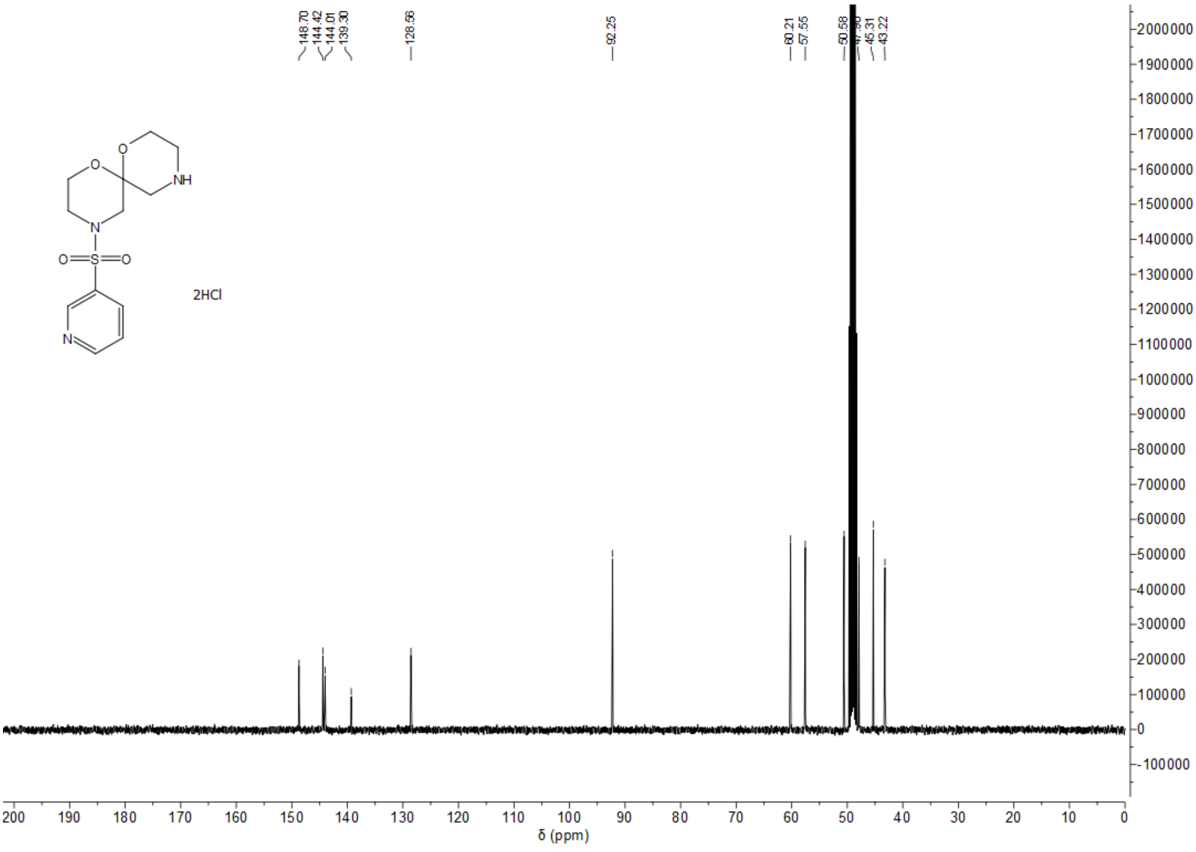

2-(pyridin-3-yl)-1-(10-(pyridin-3-ylsulfonyl)-1,7-dioxa-4,10-diazaspiro[5.5]undecan-4-yl)ethan-1-one (L1-B08-C39) [^{1}H-NMR data: 400 MHz, CD_3OD; ^{13}C{^{1}H}-NMR data: 101 MHz, CD_3OD]:

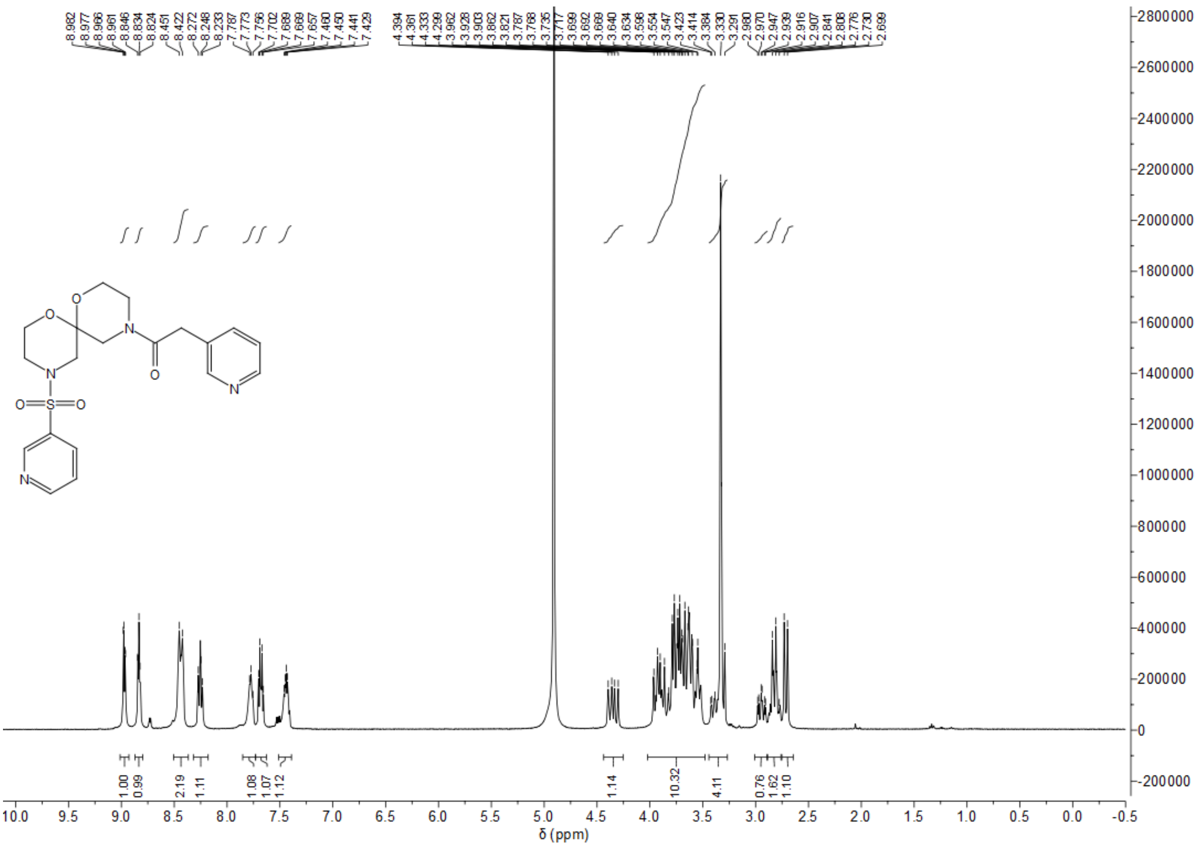

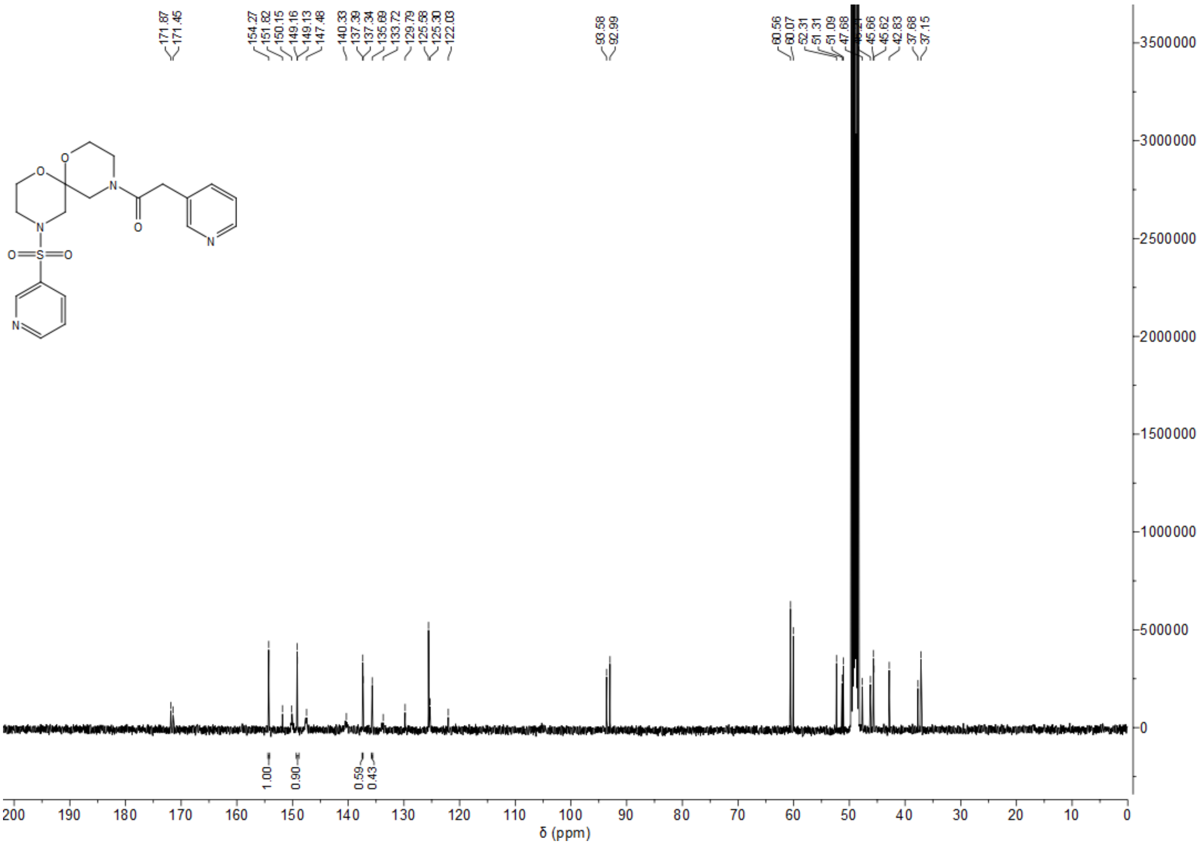

***tert*-butyl 10-(cyclohexylsulfonyl)-1,7-dioxa-4,10-diazaspiro[5.5]undecane-4-carboxylate (9-B05)**

[^{1}H-NMR data: 400 MHz, C_6D_6; ^{13}C{^{1}H}-NMR data: 101 MHz, C_6D_6]:

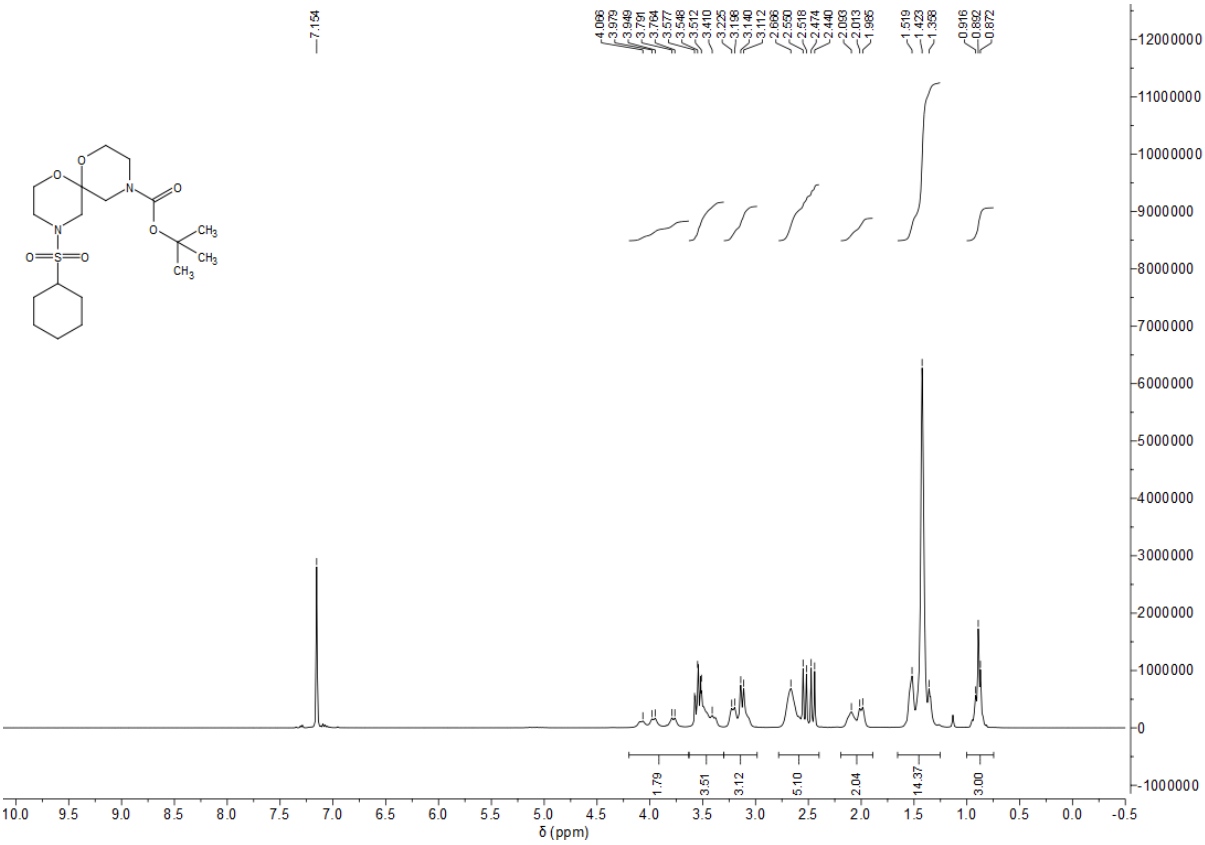

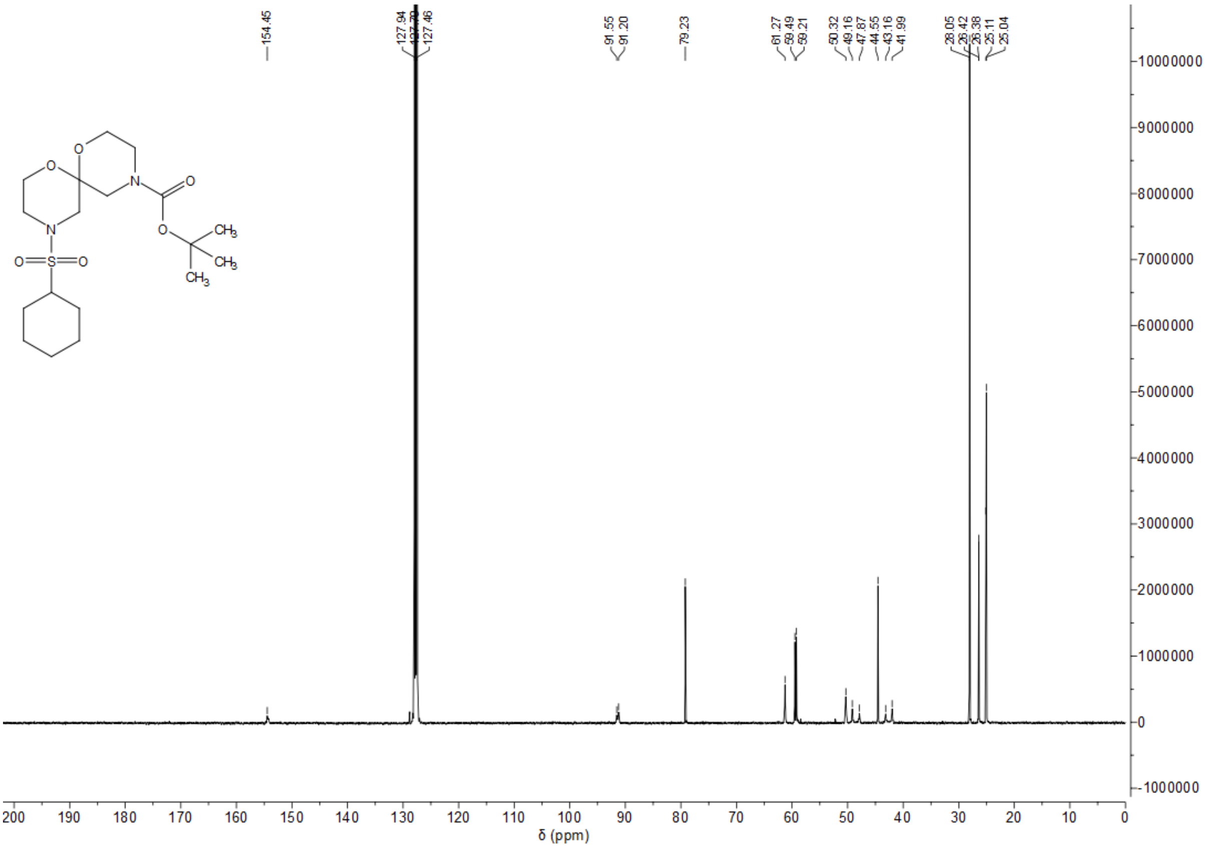

4-(cyclohexylsulfonyl)-1,7-dioxa-4,10-diazaspiro[5.5]undecane (L1-B05) [^{1}H-NMR data: 400 MHz, CD_3OD; ^{13}C{^{1}H}-NMR data: 101 MHz, CD_3OD]:

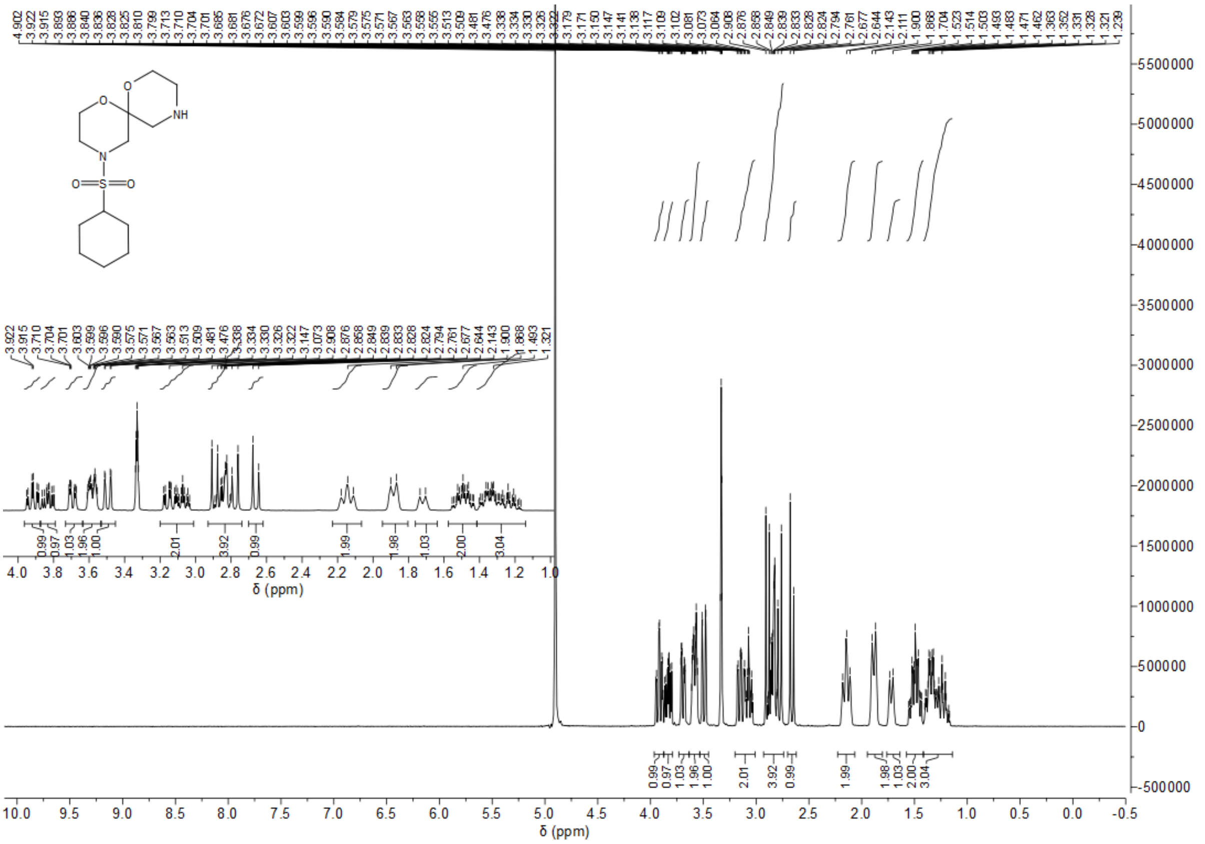

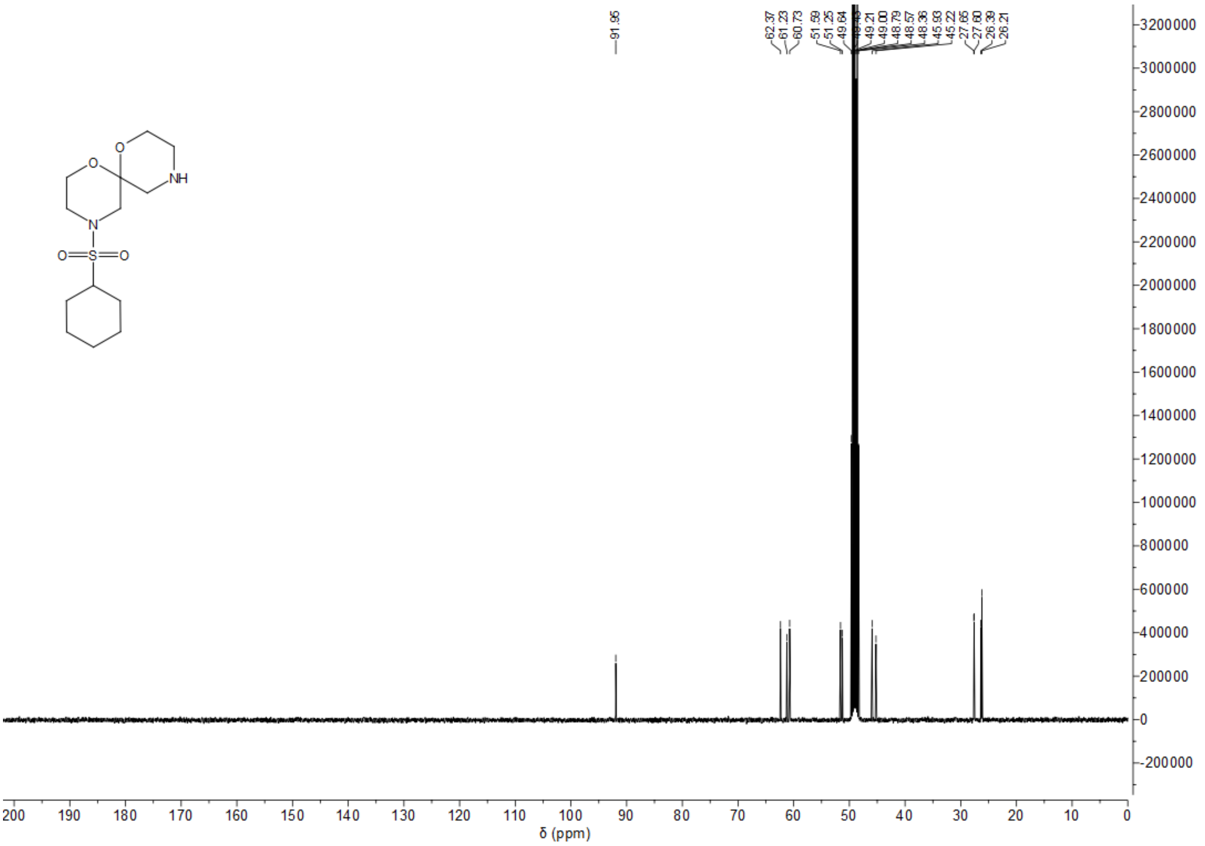

4-(cyclohexylsulfonyl)-10-((1,3,5-trimethyl-1*H*-pyrazol-4-yl)sulfonyl)-1,7-dioxa-4,10-diazaspiro[5.5]undecane (L1-B05-B10) [^{1}H-NMR data: 400 MHz, $(CD_3)_2SO$; ^{13}C{^{1}H}-NMR data: 101 MHz, $(CD_3)_2SO$]:

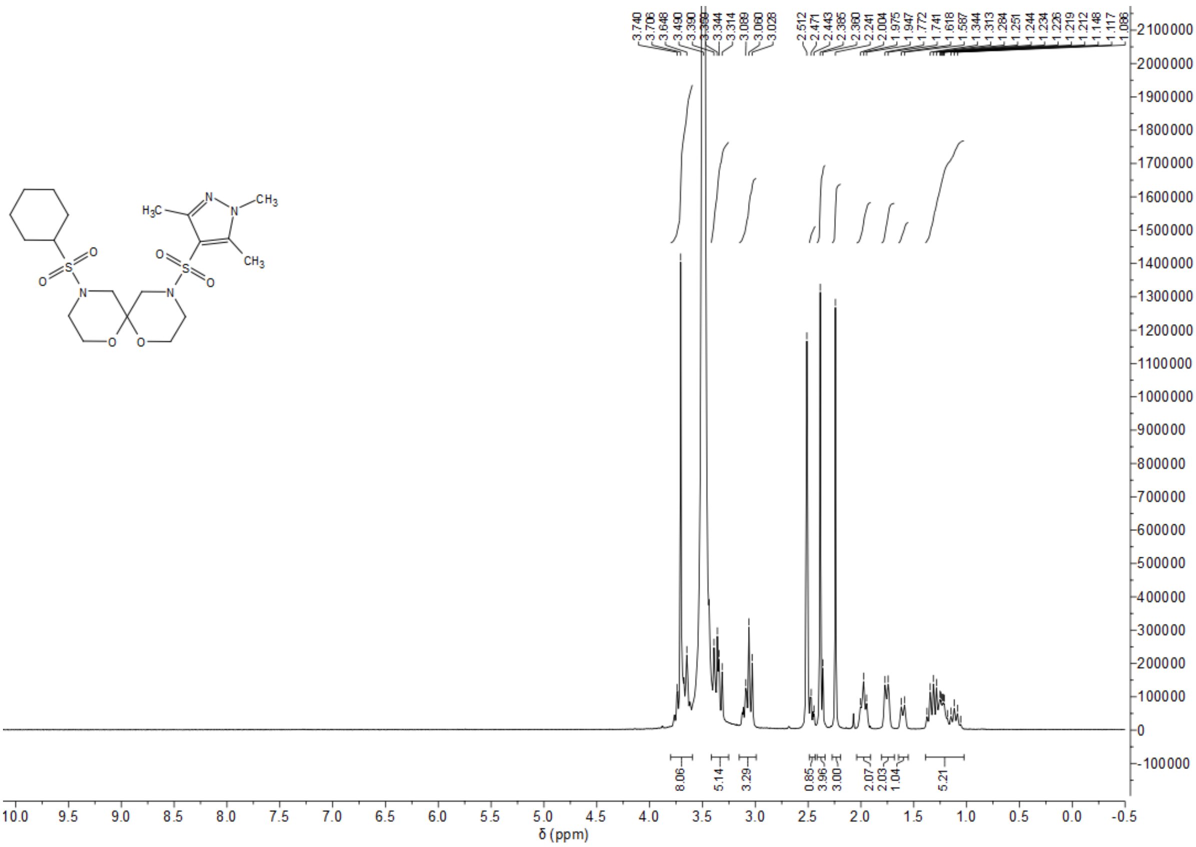

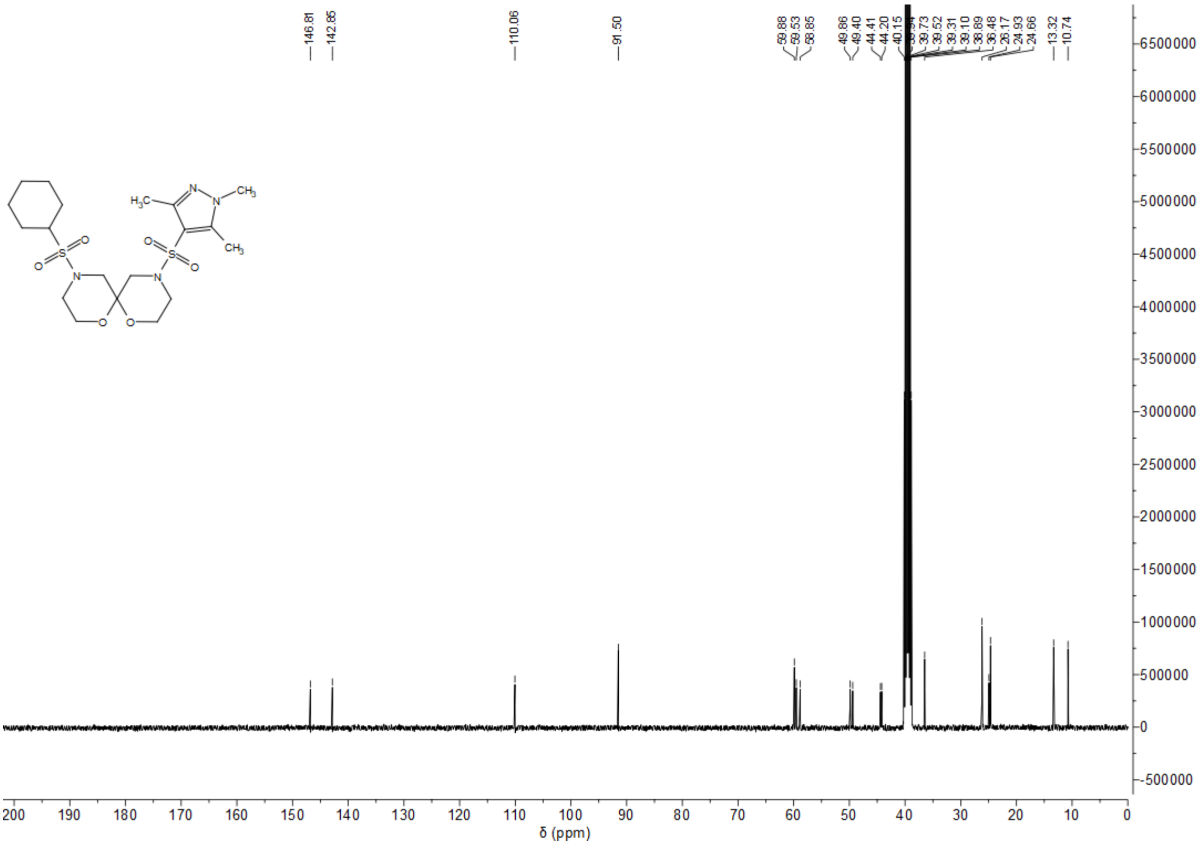

10-(cyclohexylsulfonyl)-*N*-(4-methoxybenzyl)-1,7-dioxa-4,10-diazaspiro[5.5]undecane-4-carboxamide (L1-B05-I11) [^{1}H-NMR data: 400 MHz, CD_3OD; $^{13}C\{^{1}H\}$-NMR data: 101 MHz, CD_3OD]:

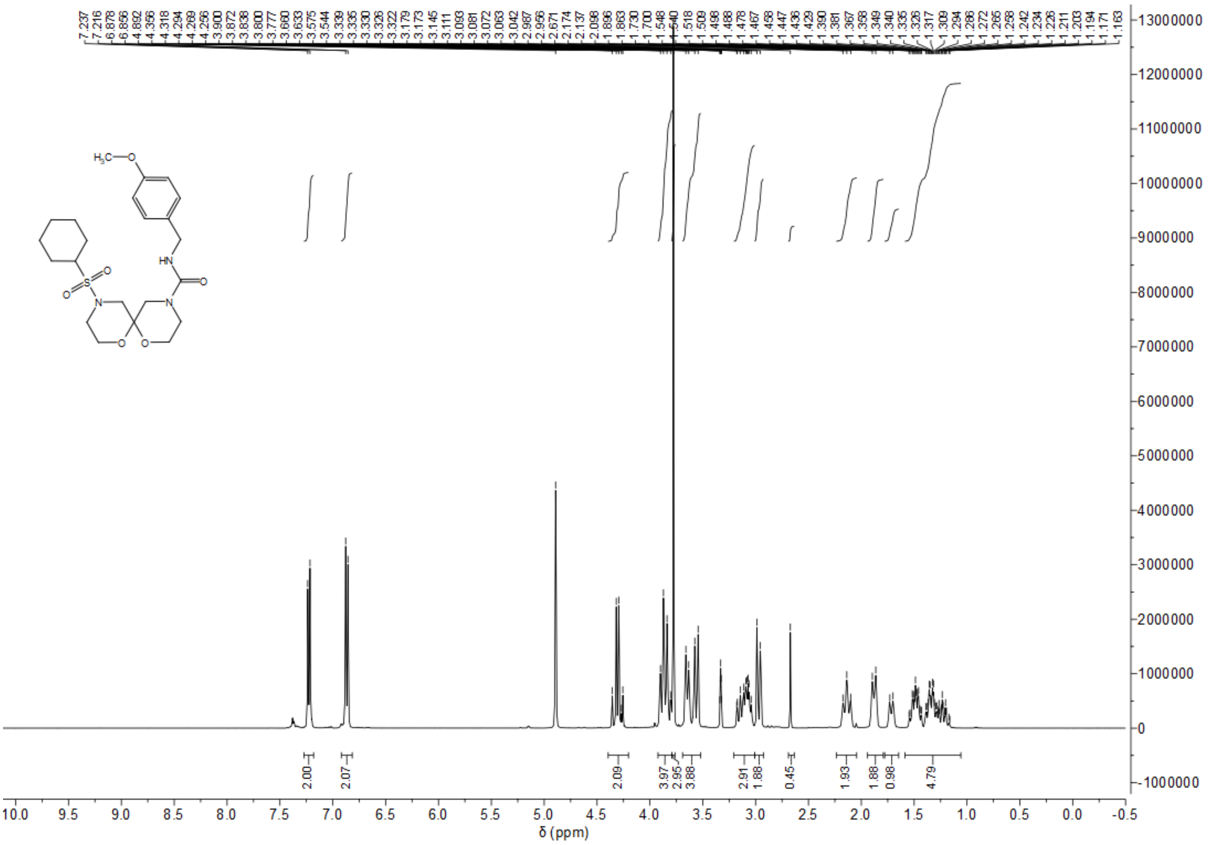

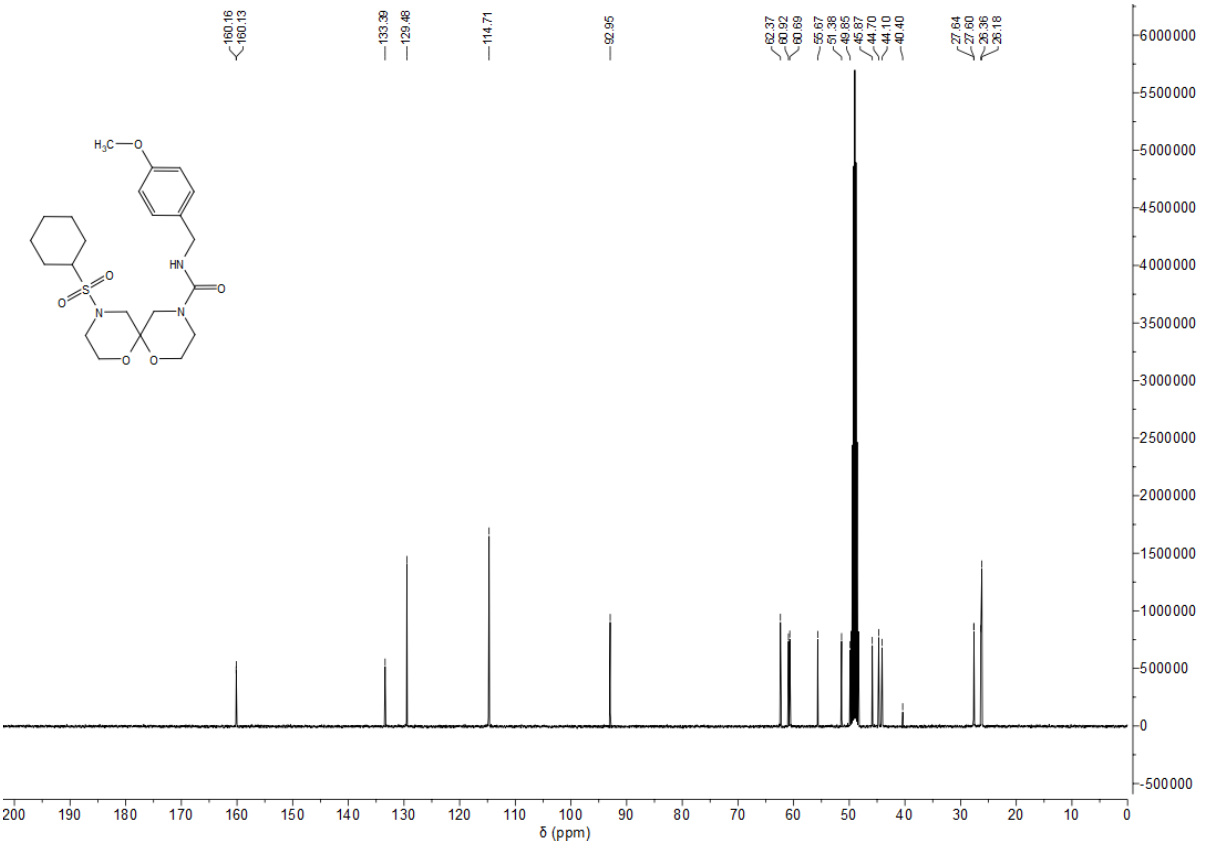

4-(cyclohexylsulfonyl)-10-(pyrimidin-2-ylmethyl)-1,7-dioxa-4,10-diazaspiro[5.5] undecane (L1-B05-A06) [^{1}H-NMR data: 400 MHz, CD_3OD; $^{13}C\{^1H\}$-NMR data: 101 MHz, CD_3OD]:

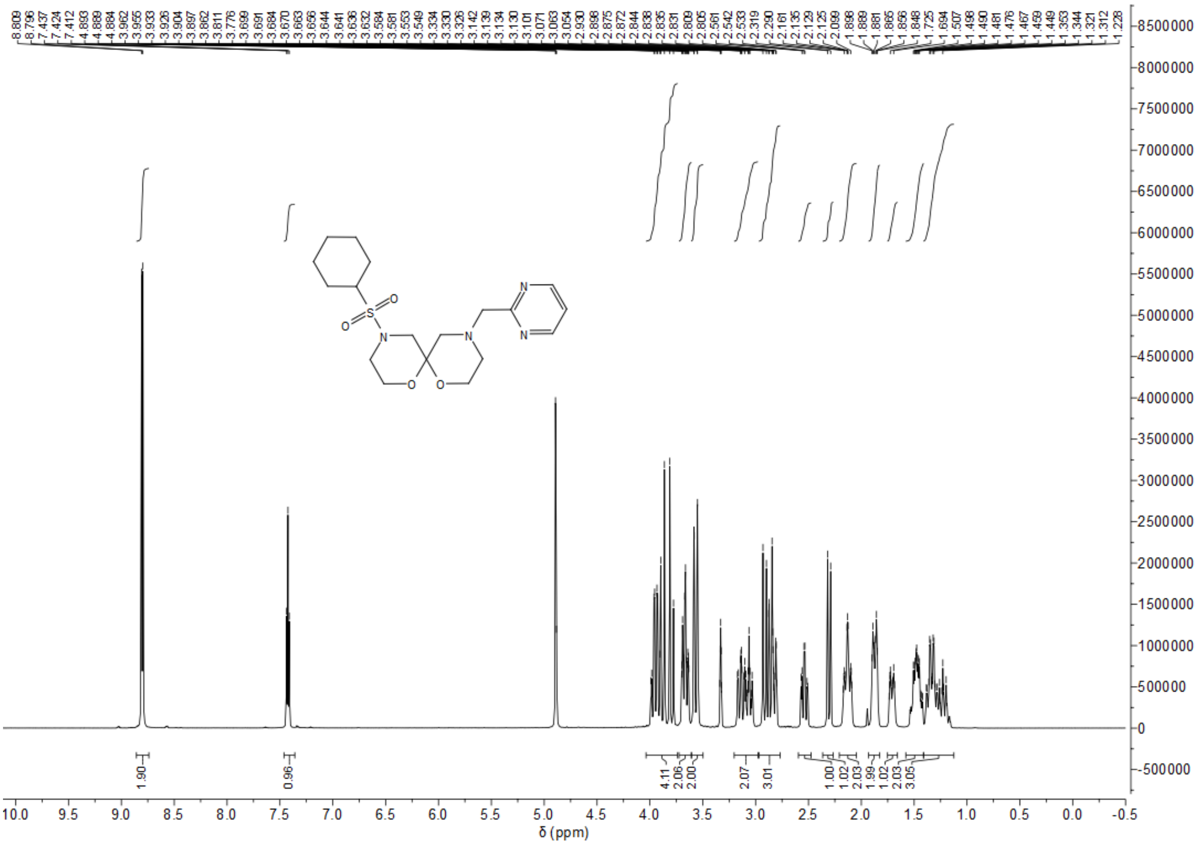

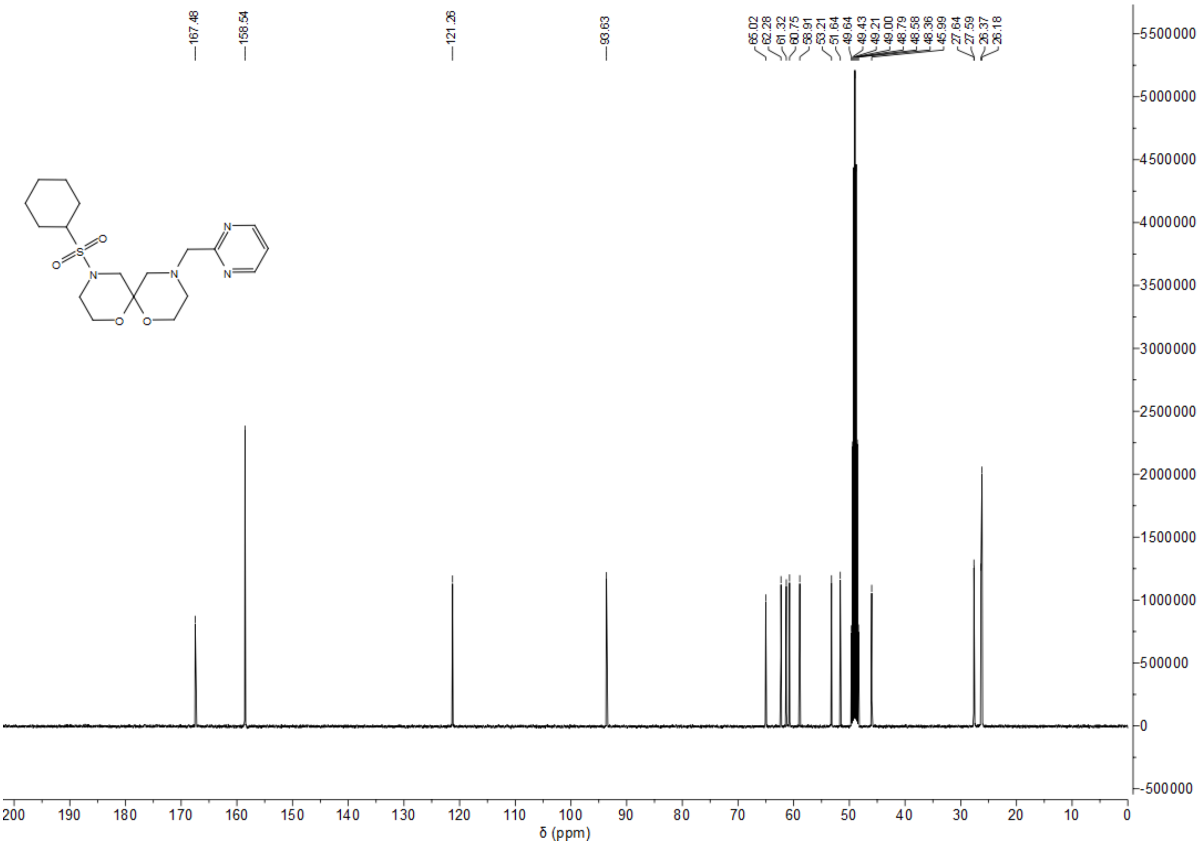

***tert*-butyl 4-(pyridin-3-ylsulfonyl)-1,7-dioxa-4,11-diazaspiro[5.6]dodecane-11-carboxylate (25-B08)**

[^{1}H-NMR data: 400 MHz, $CDCl_3$; ^{13}C{^{1}H}-NMR data: 101 MHz, $CDCl_3$]:

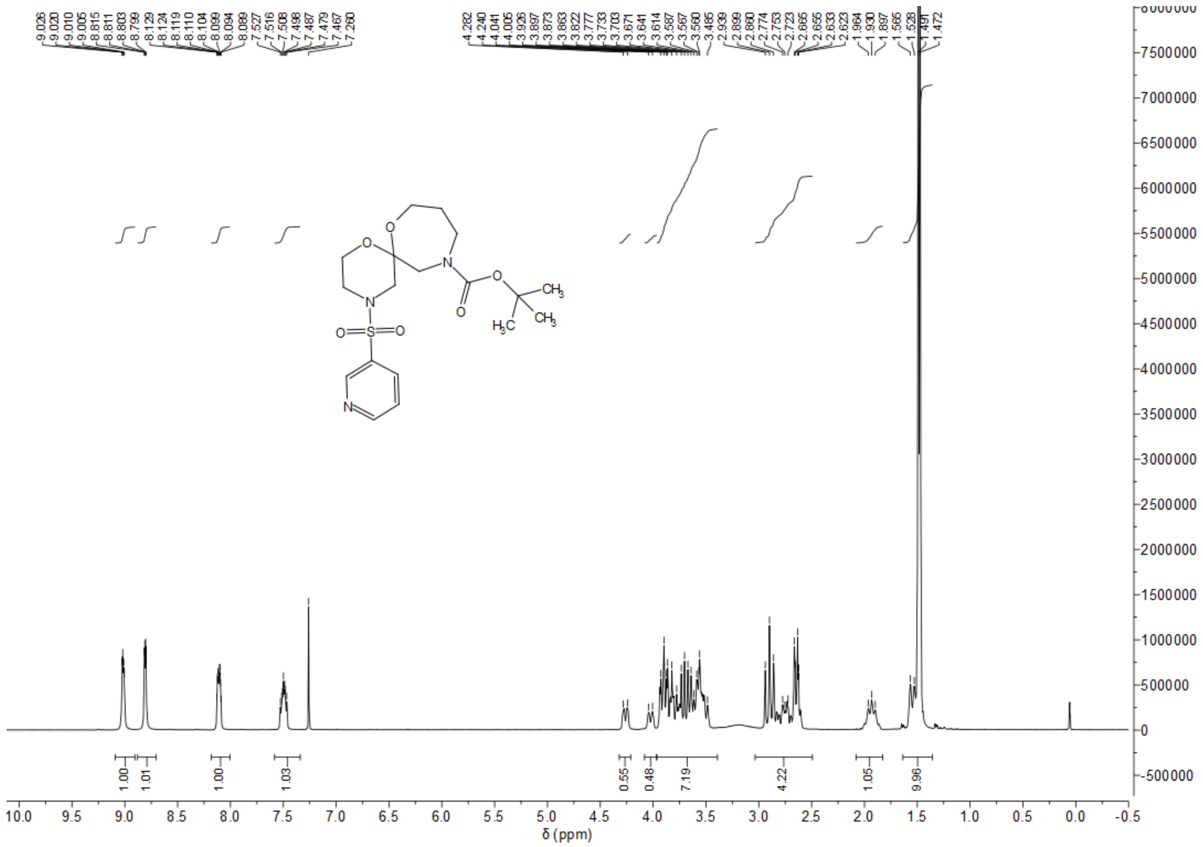

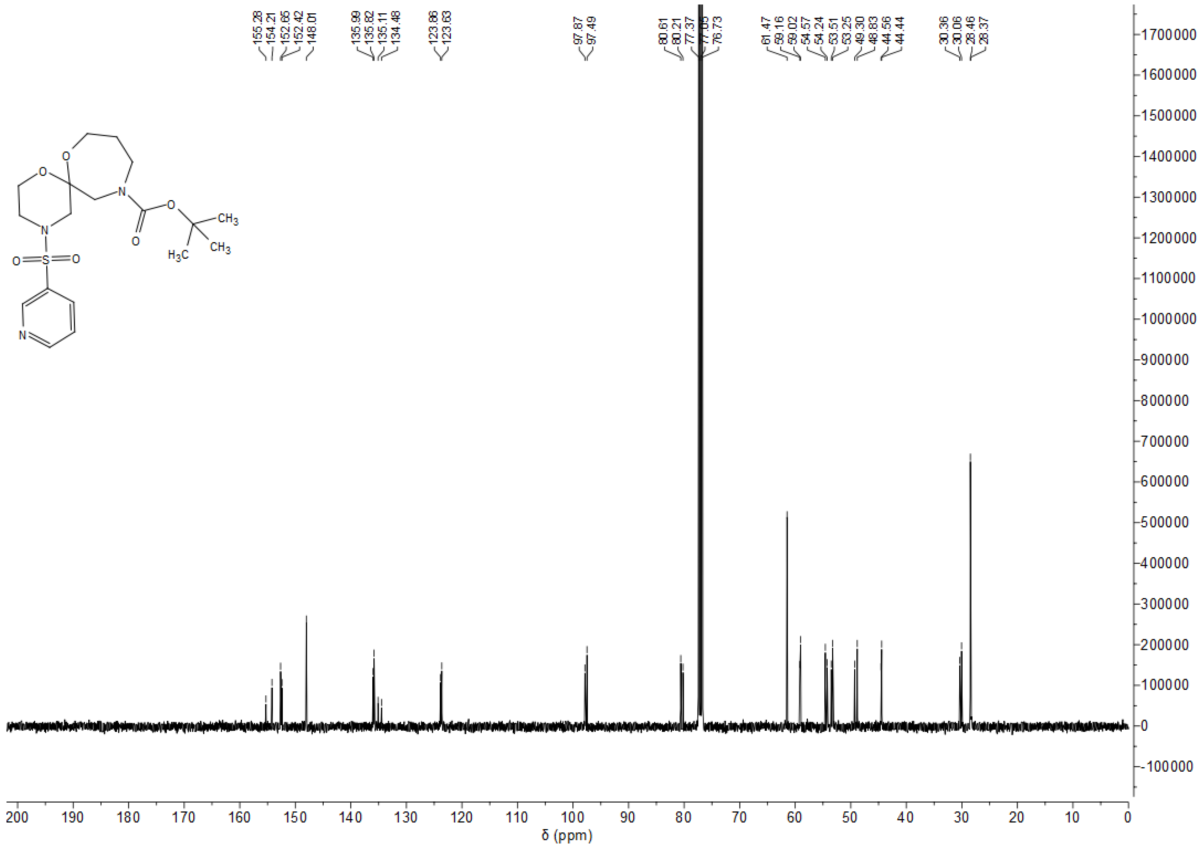

4-(pyridin-3-ylsulfonyl)-1,7-dioxa-4,11-diazaspiro[5.6]dodecane dihydrochloride (L2-B08·2HCl *or* 30·2HCl in the main article) [^{1}H-NMR data: 400 MHz, CD_3OD; ^{13}C{^{1}H}-NMR data: 101 MHz, CD_3OD]:

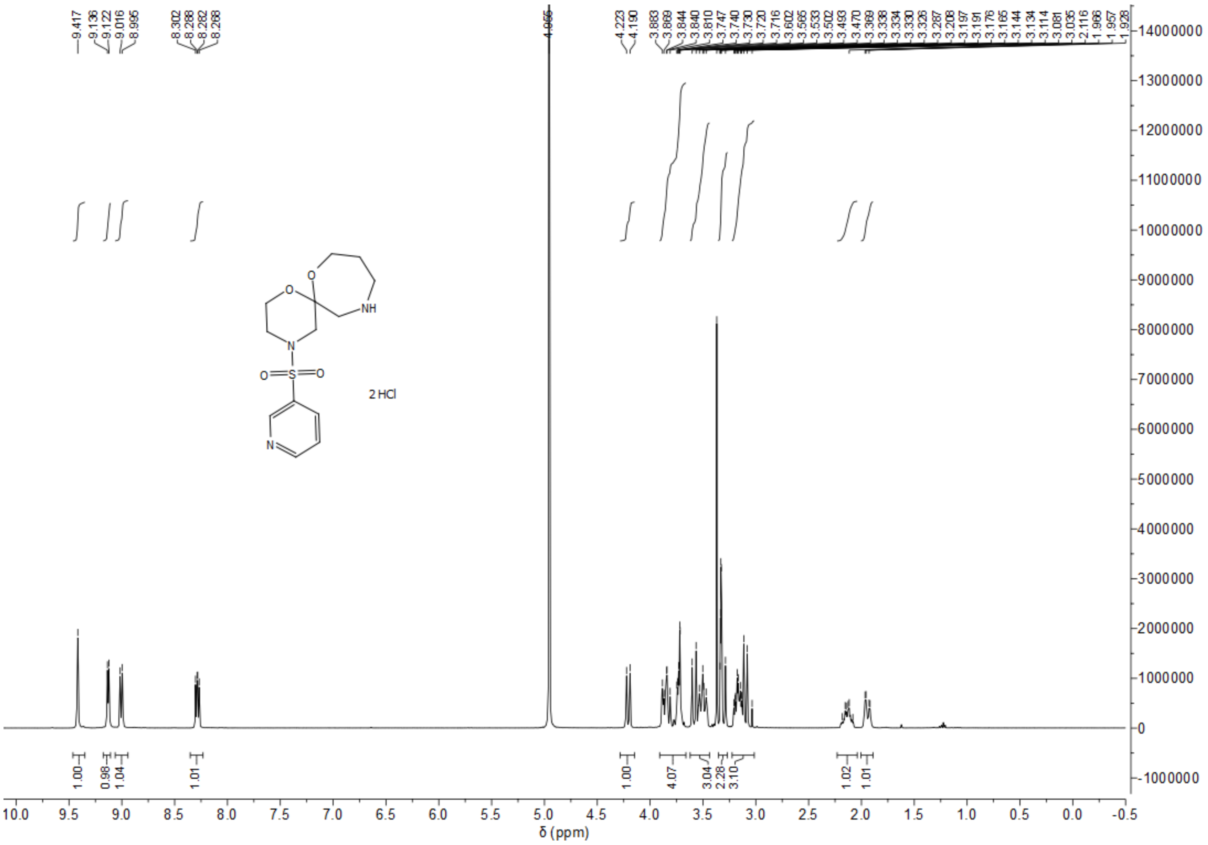

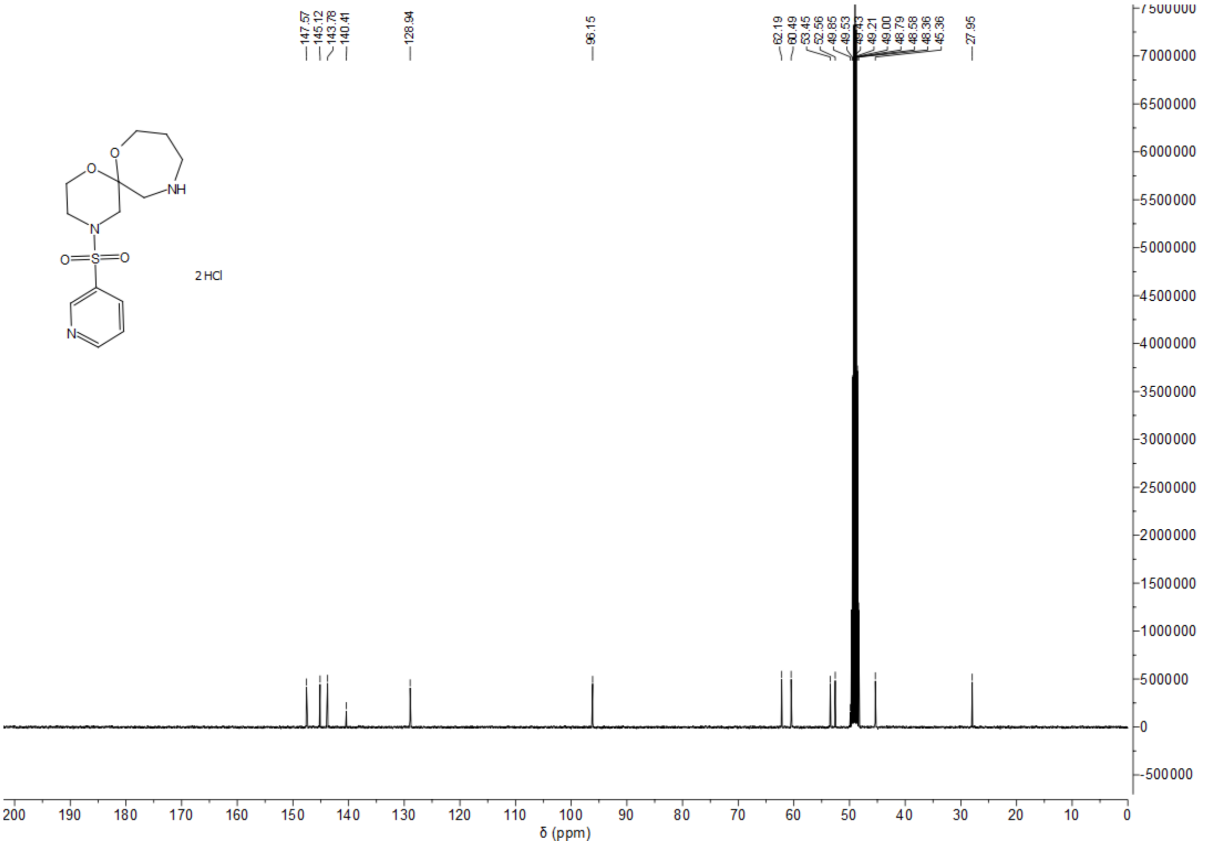

(1-(4-fluorophenyl)cyclopropyl)(4-(pyridin-3-ylsulfonyl)-1,7-dioxa-4,11-diazaspiro[5.6]dodecan-11-yl)methanone (L2-B08-C34) [^{1}H-NMR data: 400 MHz, CD_3OD; ^{13}C{^{1}H}-NMR data: 101 MHz, CD_3OD]:

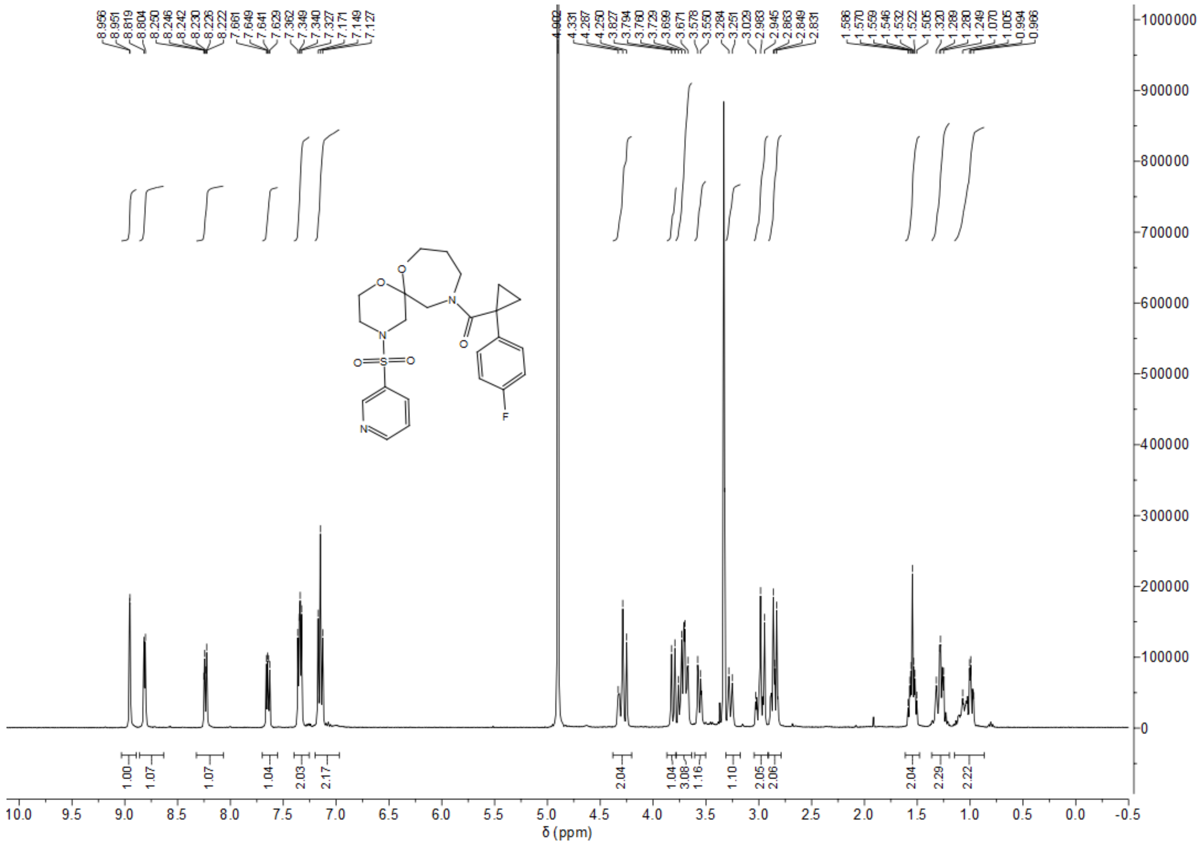

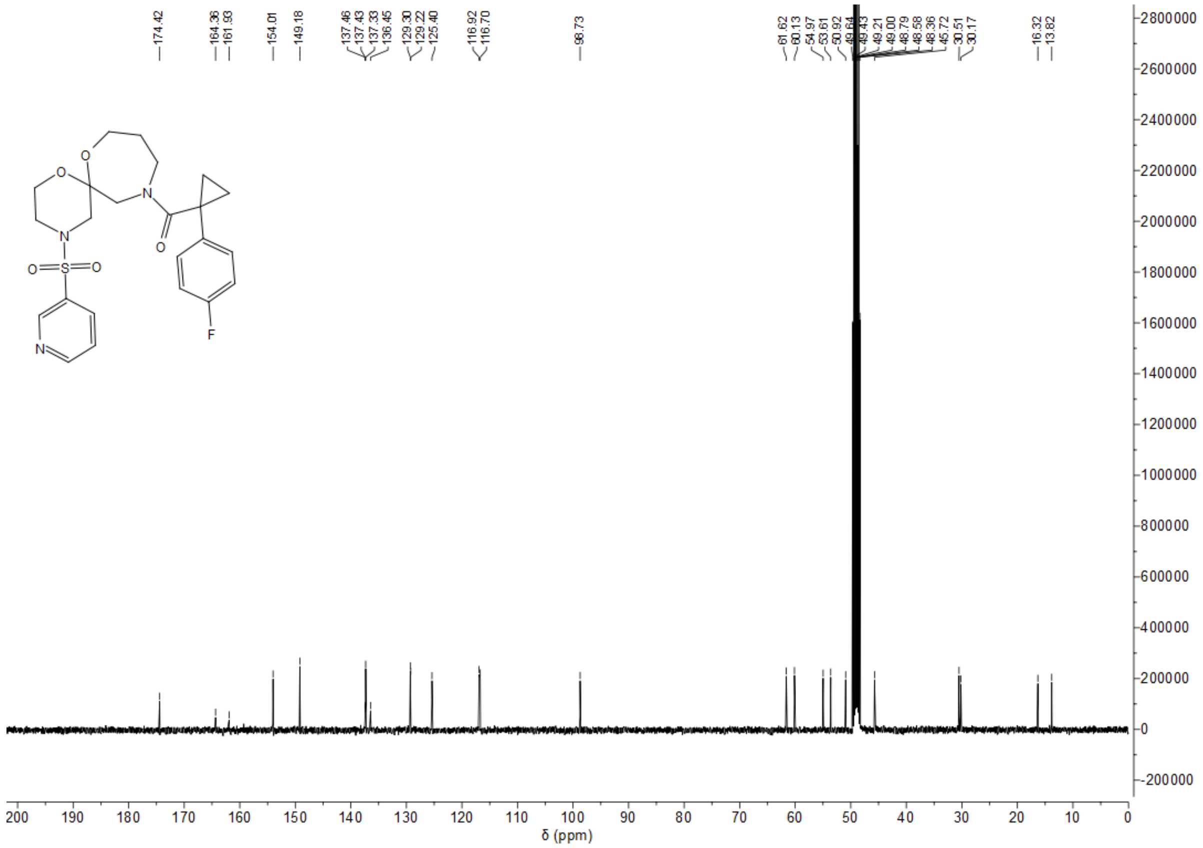

(2-methylpyridin-4-yl)(4-(pyridin-3-ylsulfonyl)-1,7-dioxa-4,11-diazaspiro[5.6]dodecan-11-yl)methanone (L2-B08-C38) [^{1}H-NMR data: 400 MHz, CD_3OD; $^{13}C\{^{1}H\}$-NMR data: 101 MHz, CD_3OD]:

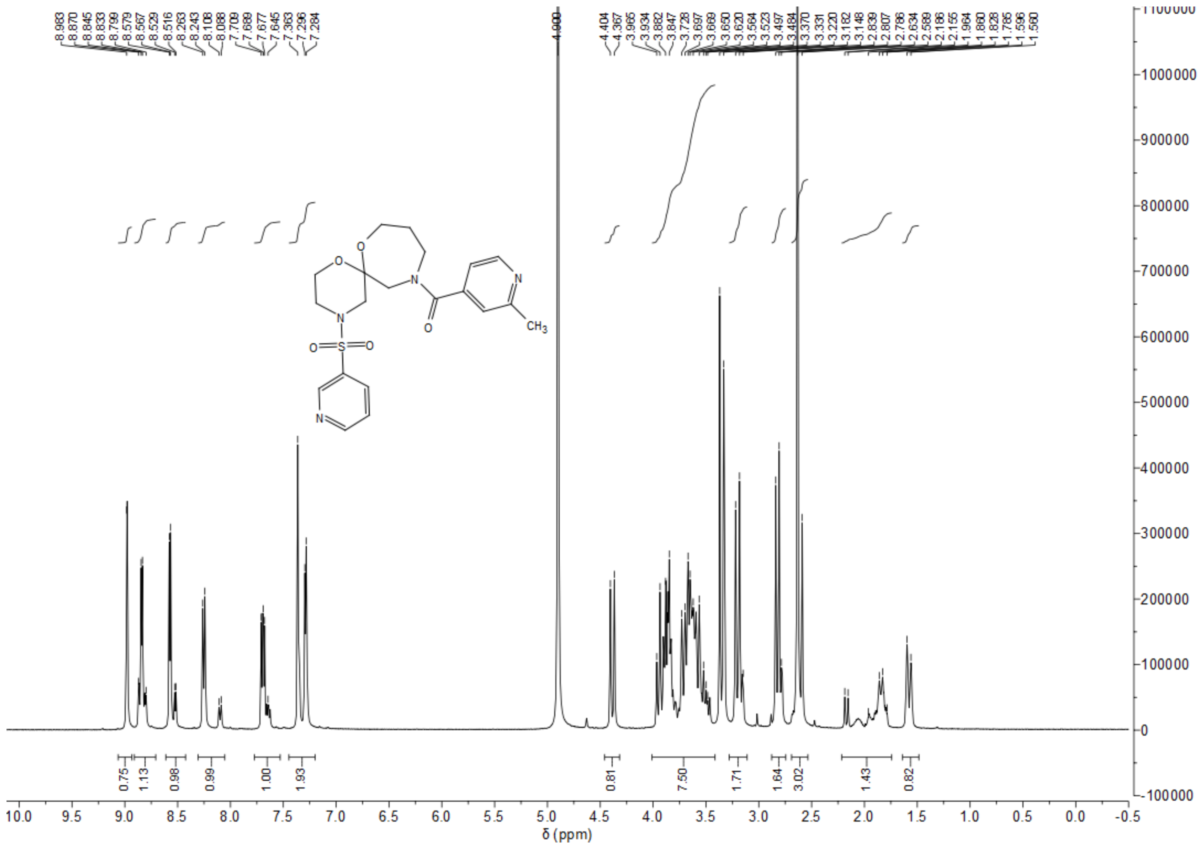

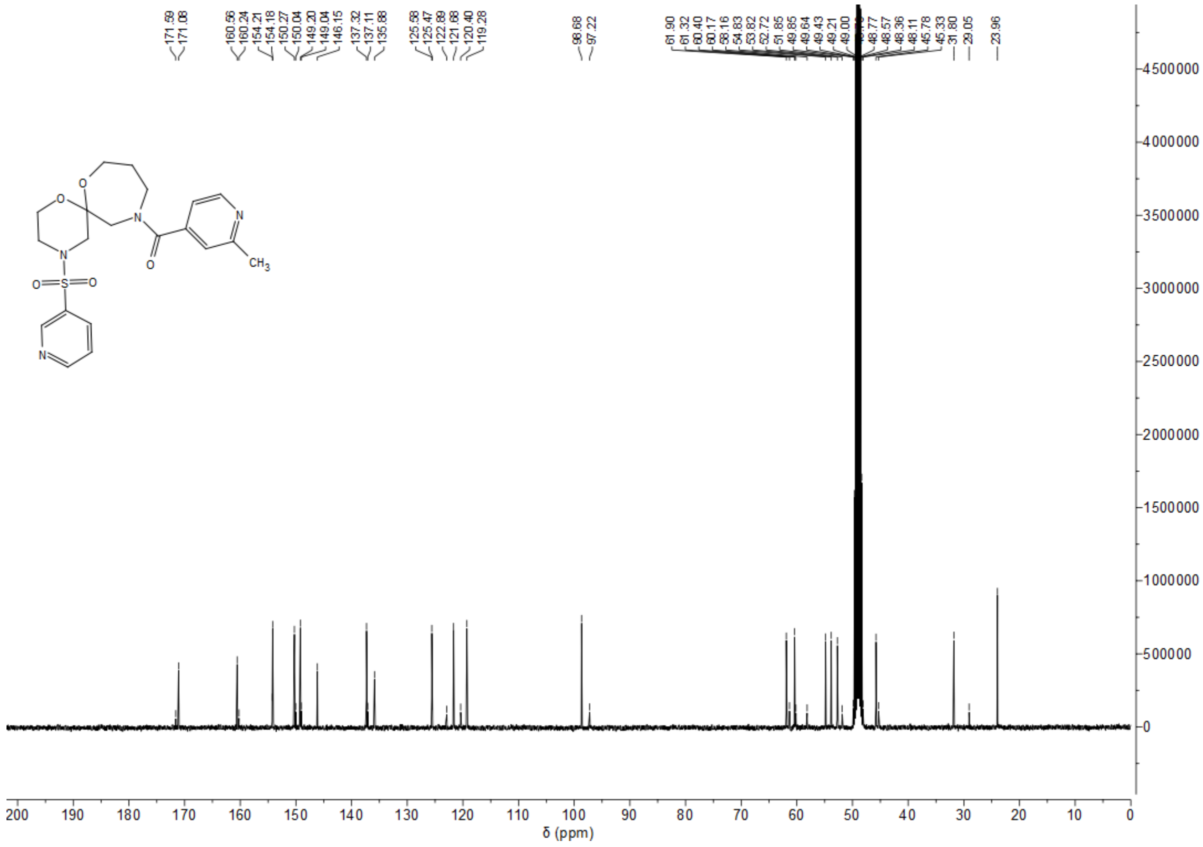

1.6.3 First decoration step – Amidations

***tert*-butyl 10-(1-methyl-1*H*-pyrazole-4-carbonyl)-1,7-dioxa-4,10-diazaspiro[5.5]undecane-4-carboxylate (9-C28 *or* 15 in the main article) [^{1}H-NMR data: 400 MHz, C_6D_6; ^{13}C{^{1}H}-NMR data: 101 MHz, C_6D_6]:**

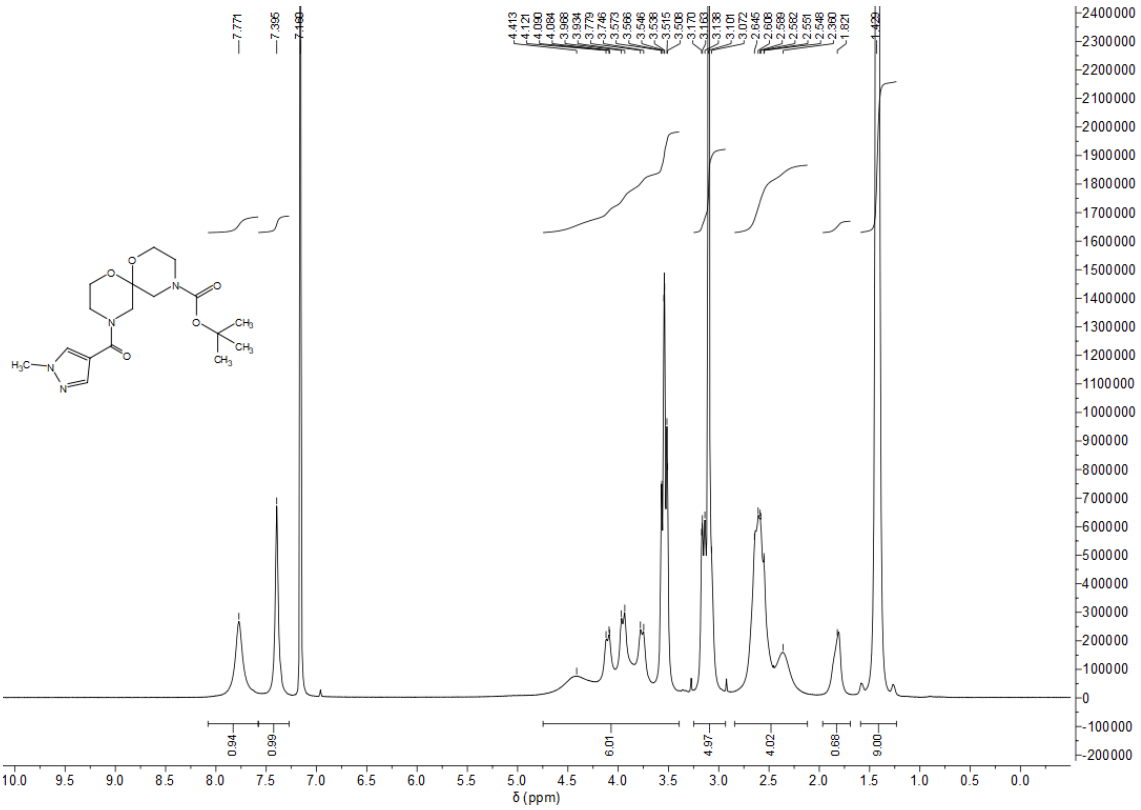

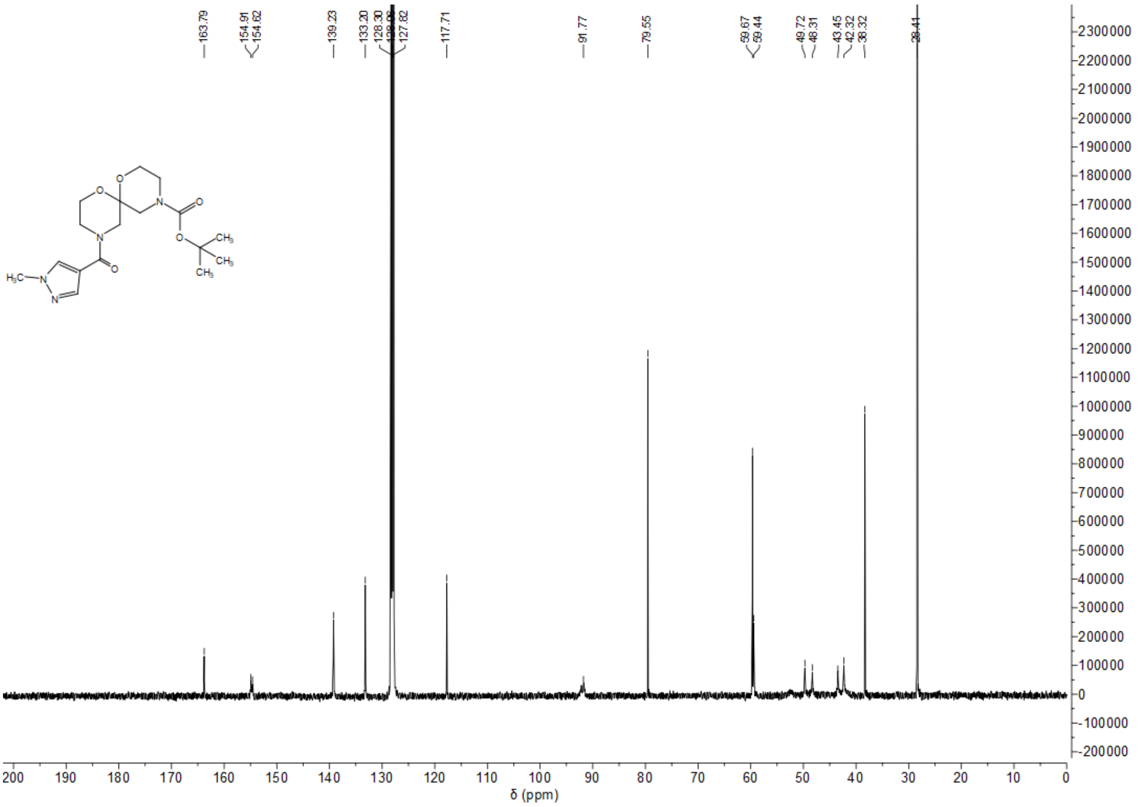

(1-methyl-1*H*-pyrazol-4-yl)(1,7-dioxa-4,10-diazaspiro[5.5]undecan-4-yl)methanone (L1-C28 *or* 17·HCl in the main article) [^{1}H-NMR data: 400 MHz, CD_3OD; $^{13}C\{^1H\}$-NMR data: 101 MHz, CD_3OD]:

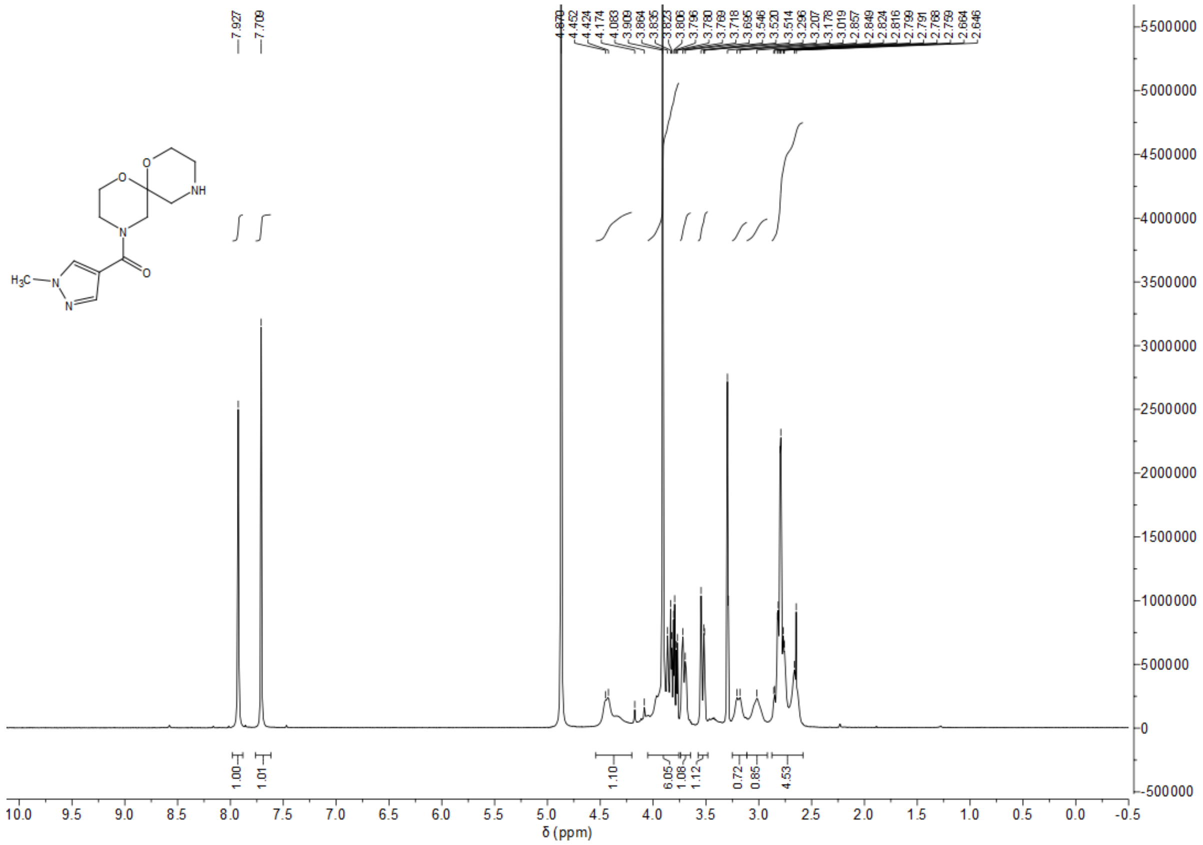

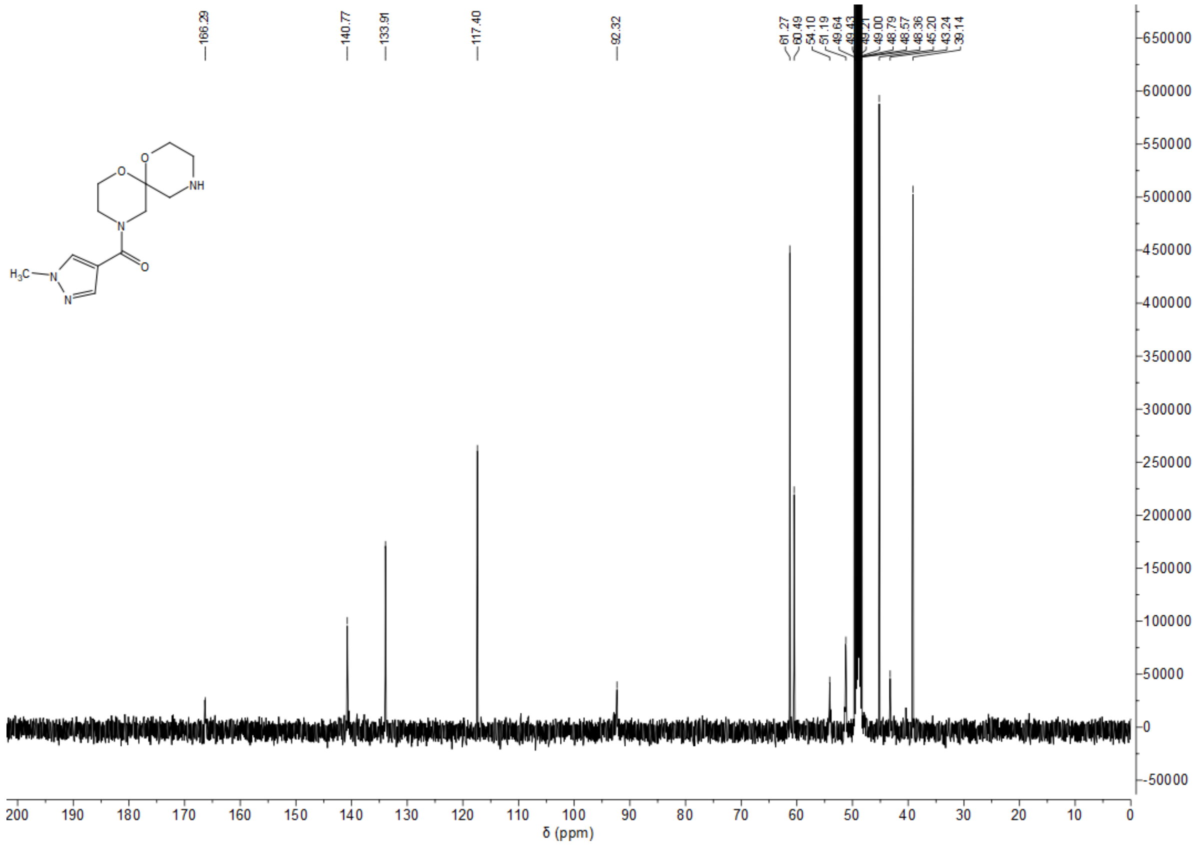

tert-butyl 10-(1-phenylcyclopropane-1-carbonyl)-1,7-dioxa-4,10-diazaspiro[5.5]undecane-4-carboxylate (9-C14) [^{1}H-NMR data: 400 MHz, C_6D_6; ^{13}C{^{1}H}-NMR data: 101 MHz, C_6D_6]:

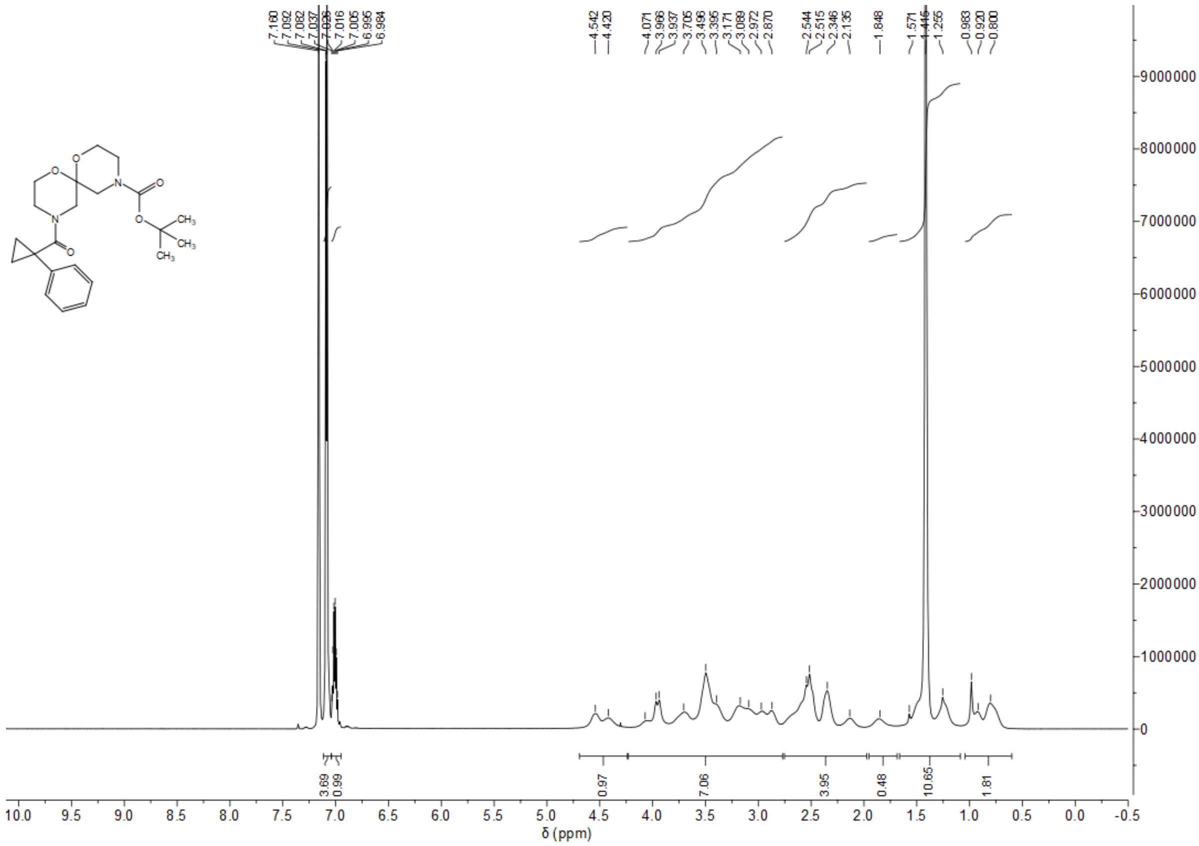

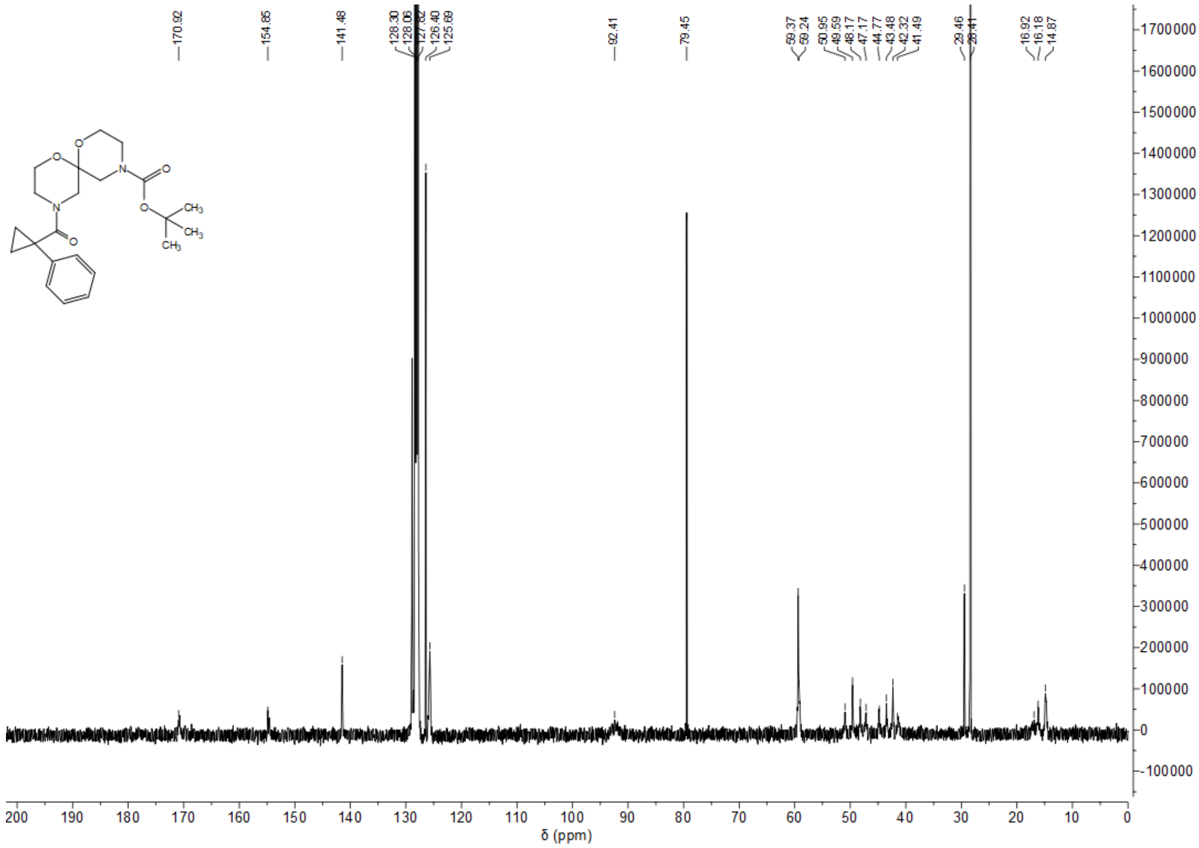

1-phenylcyclopropyl-(1,7-dioxa-4,10-diazaspiro[5.5]undecan-4-yl)methanone (L1-C14) [^{1}H-NMR data: 400 MHz, CD_3OD; $^{13}C\{^1H\}$-NMR data: 101 MHz, CD_3OD]:

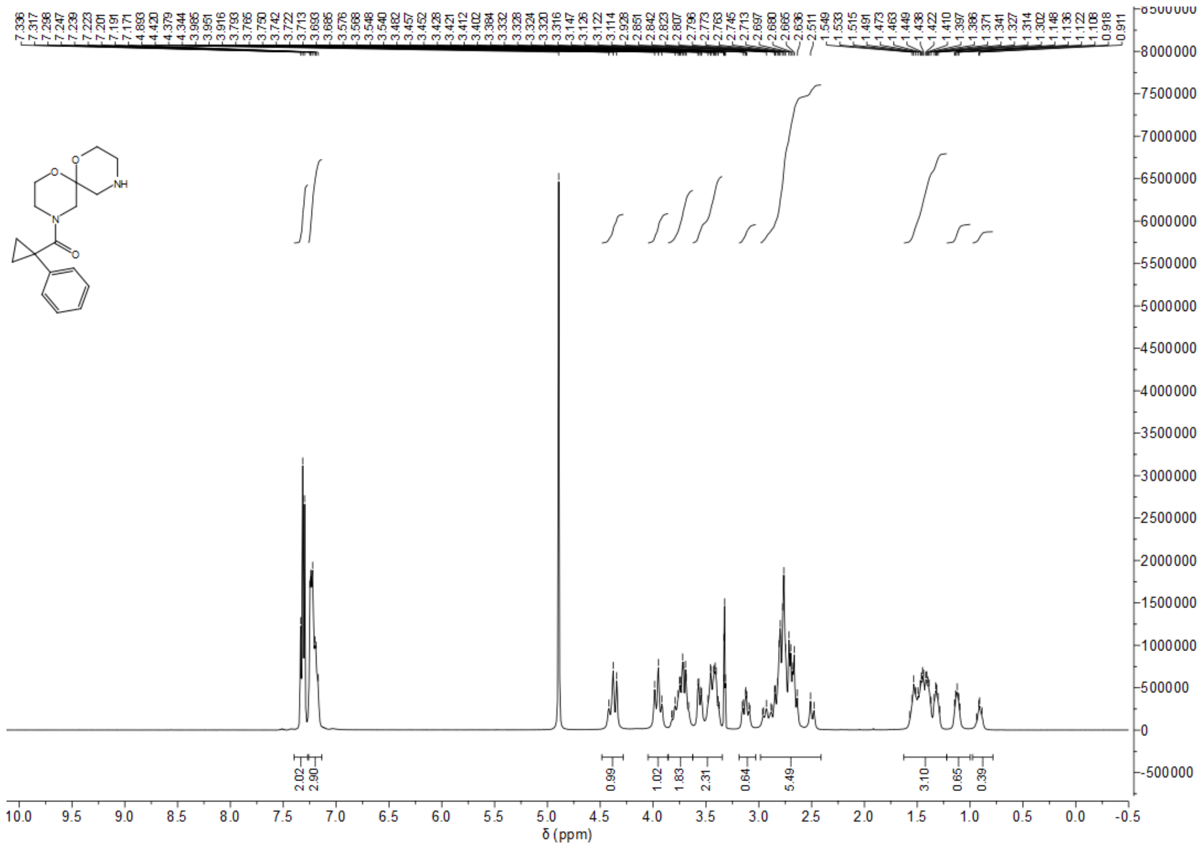

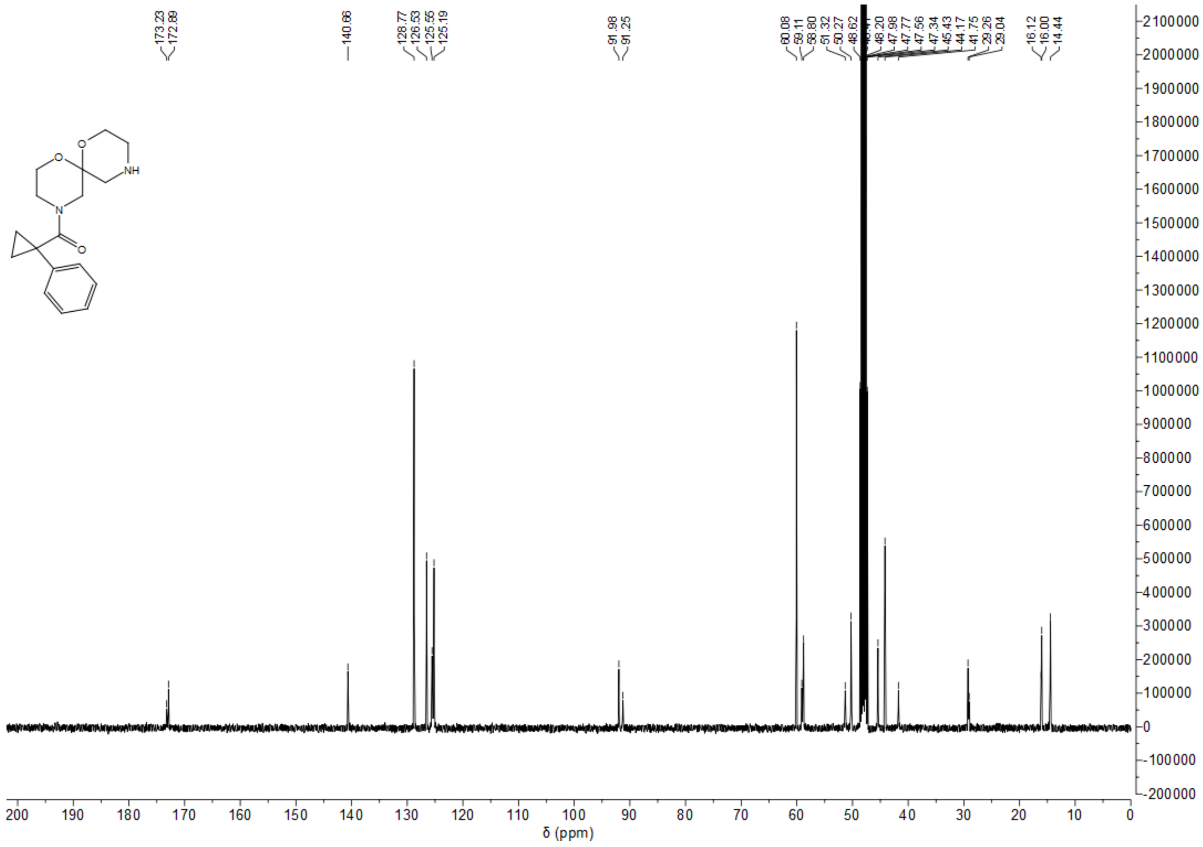

(2-methylthiazol-4-yl)(10-(1-phenylcyclopropane-1-carbonyl)-1,7-dioxa-4,10-diazaspiro[5.5]undecan-4-yl)methanone (L1-C14-C42) [^{1}H-NMR data: 400 MHz, CD_3OD; $^{13}C\{^1H\}$-NMR data: 101 MHz, CD_3OD]:

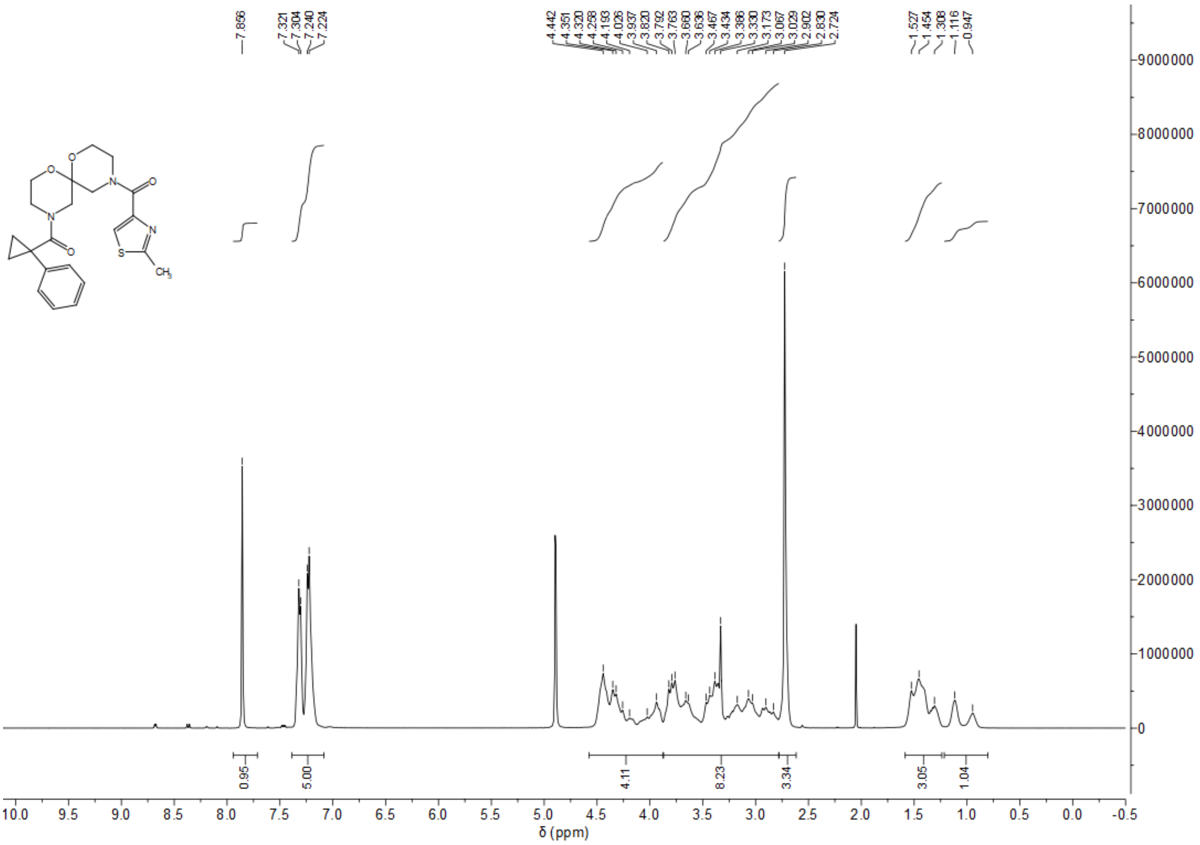

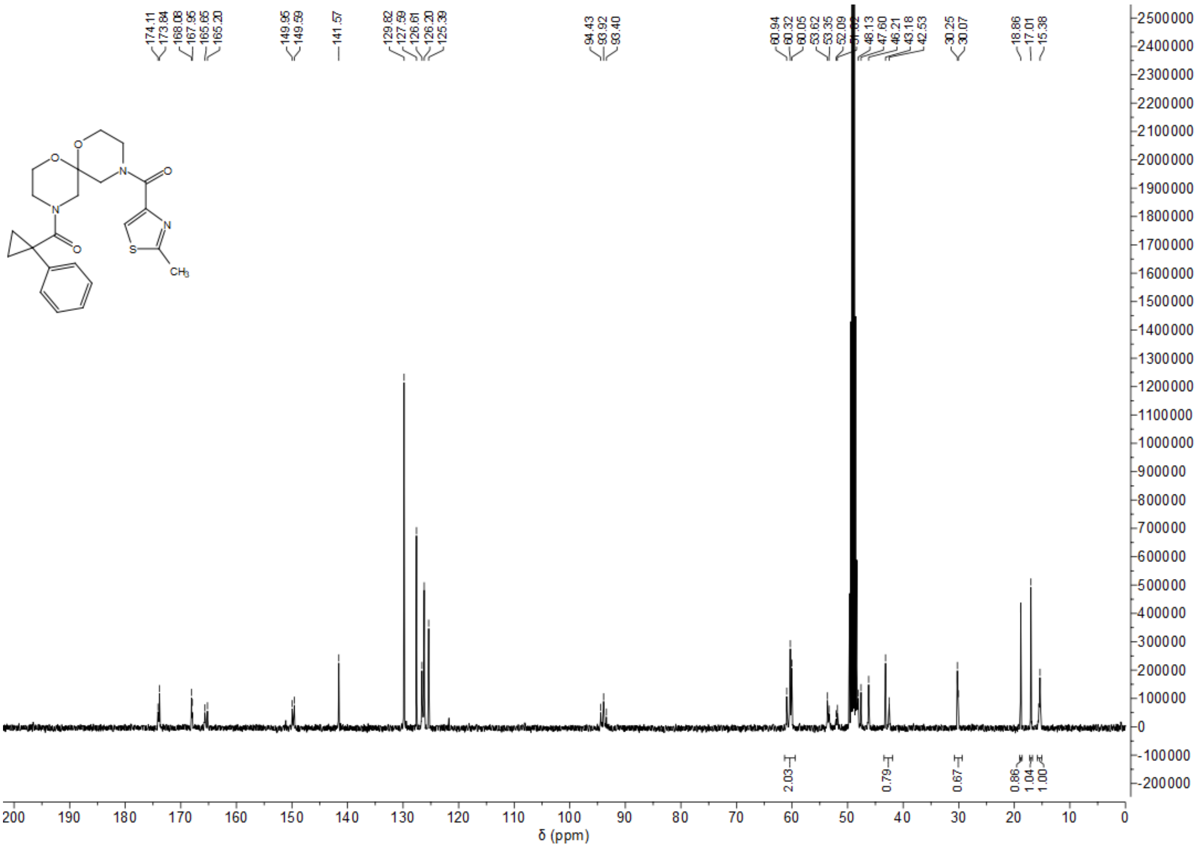

***tert*-butyl 10-(2-phenoxyacetyl)-1,7-dioxa-4,10-diazaspiro[5.5]undecane-4-carboxylate (9-C08) [^{1}H-NMR data: 400 MHz, $CDCl_3$; $^{13}C\{^{1}H\}$-NMR data: 101 MHz, $CDCl_3$]:**

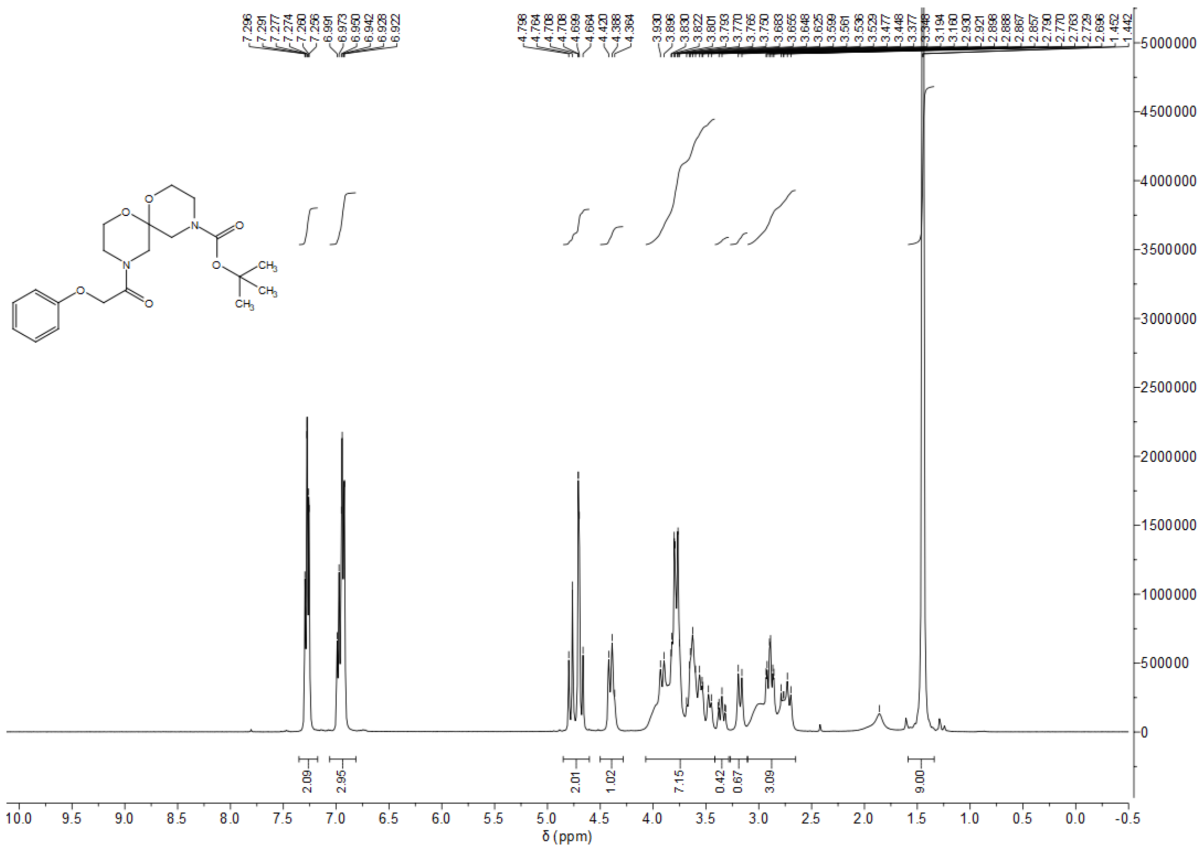

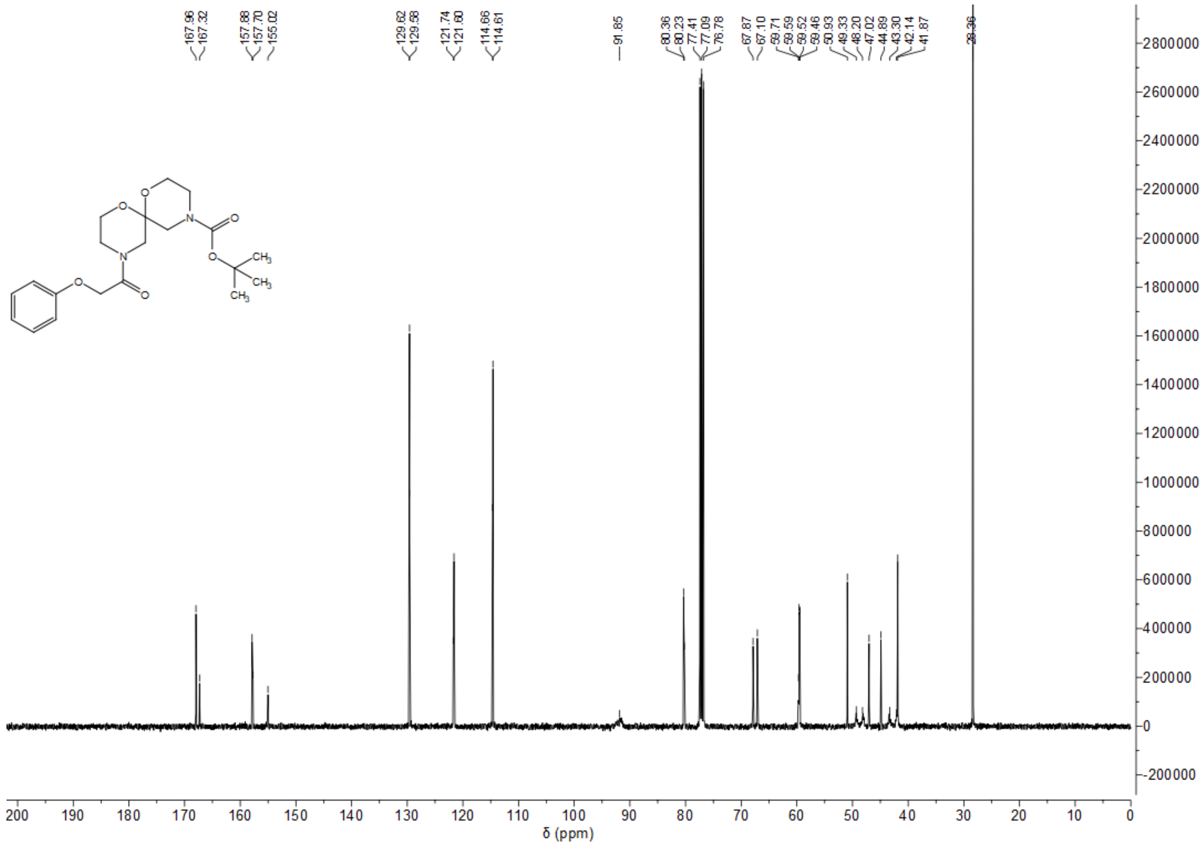

2-phenoxy-1-(1,7-dioxa-4,10-diazaspiro[5.5]undecan-4-yl)ethan-1-one (L1-C08) [^{1}H-NMR data: 400 MHz, CD_3OD; $^{13}C\{^{1}H\}$-NMR data: 101 MHz, CD_3OD]:

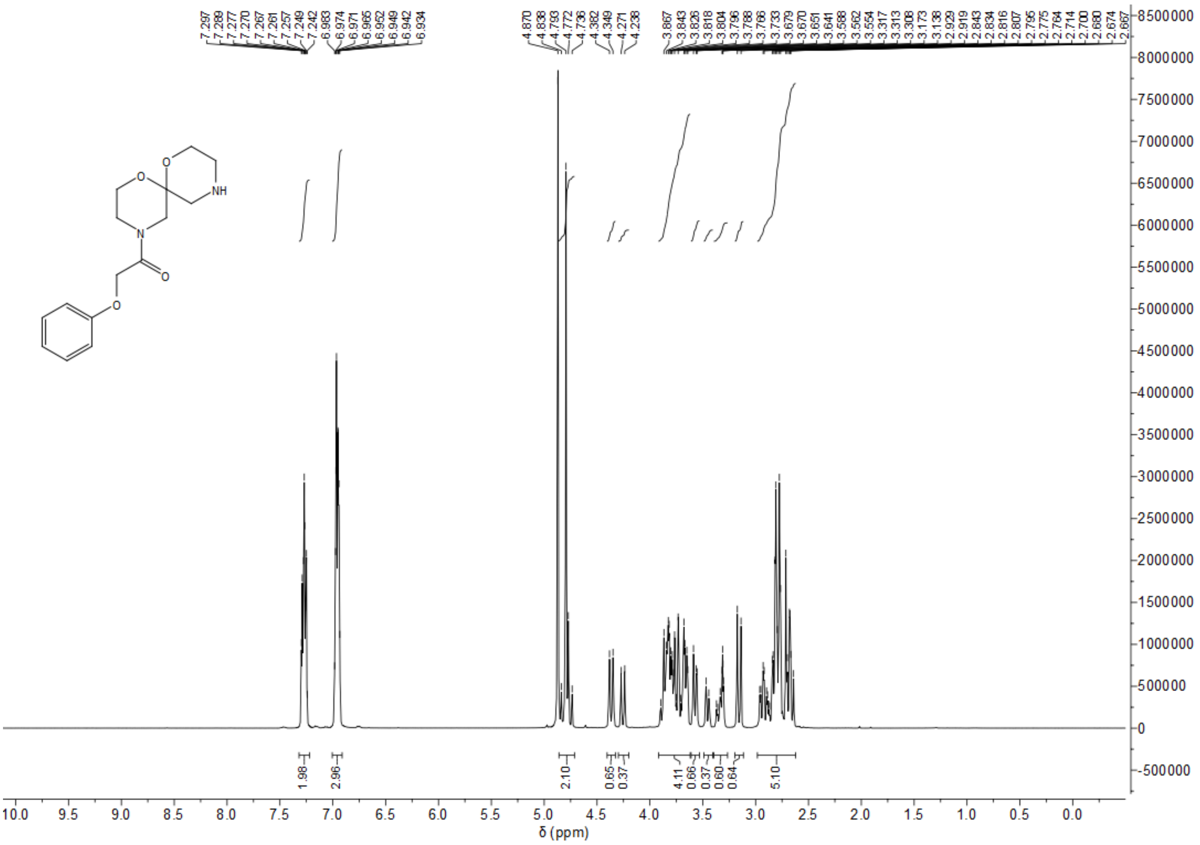

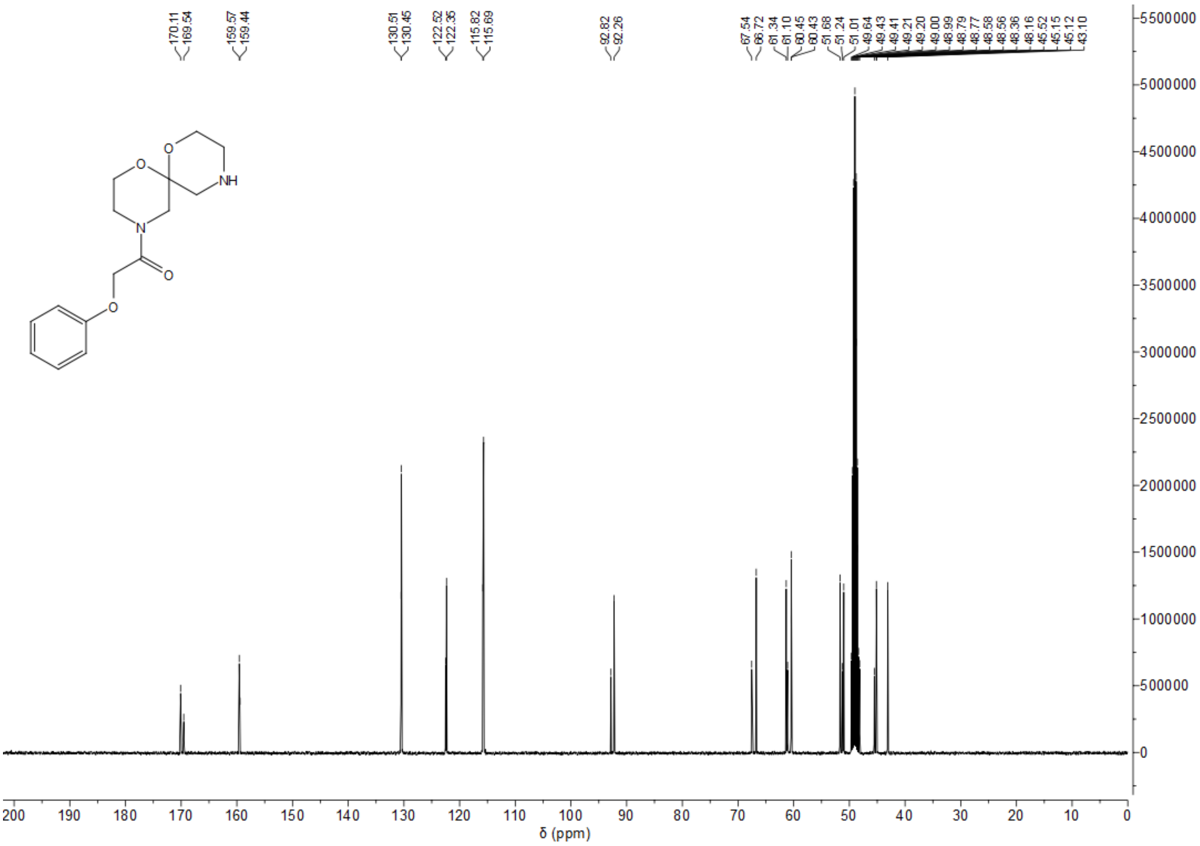

***tert*-butyl 10-(4-(trifluoromethoxy)benzoyl)-1,7-dioxa-4,10-diazaspiro[5.5]undecane-4-carboxylate (9-C16) [^{1}H-NMR data: 400 MHz, C_6D_6; ^{13}C{^{1}H}-NMR data: 101 MHz, C_6D_6]:**

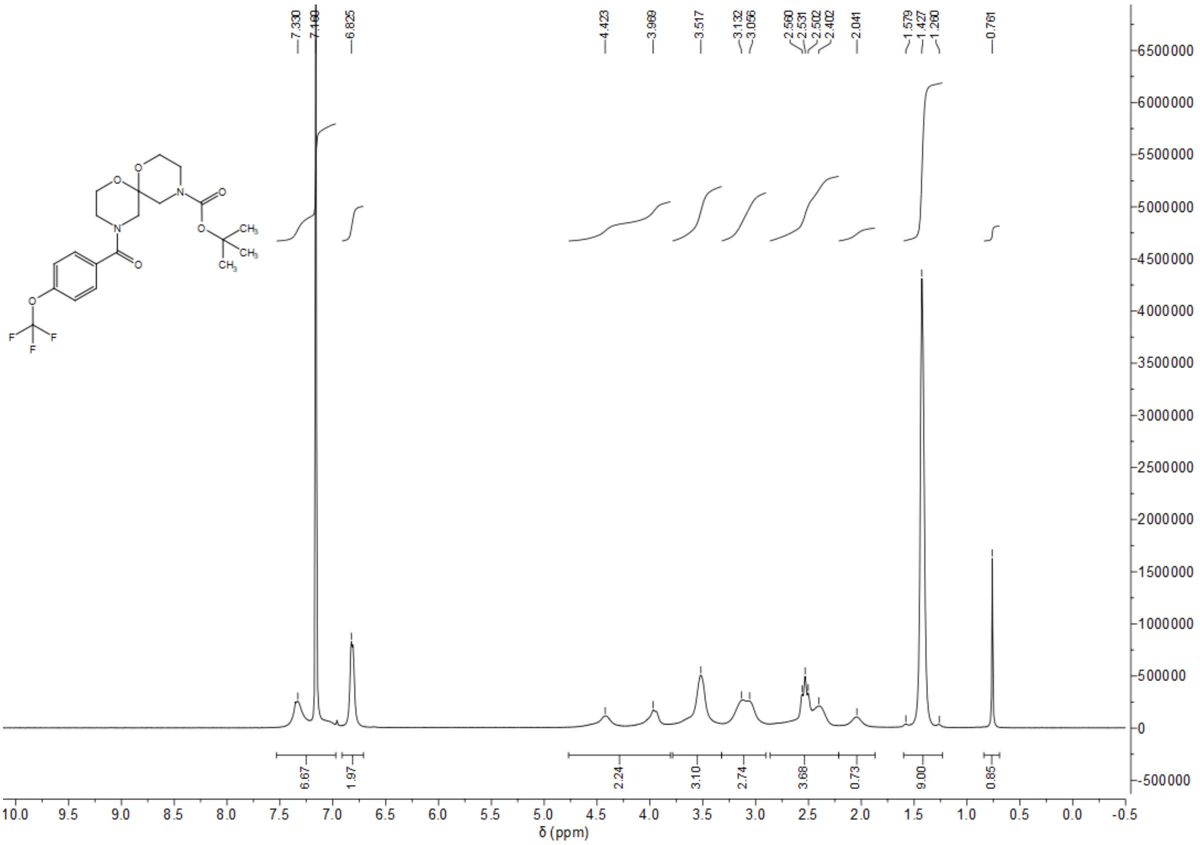

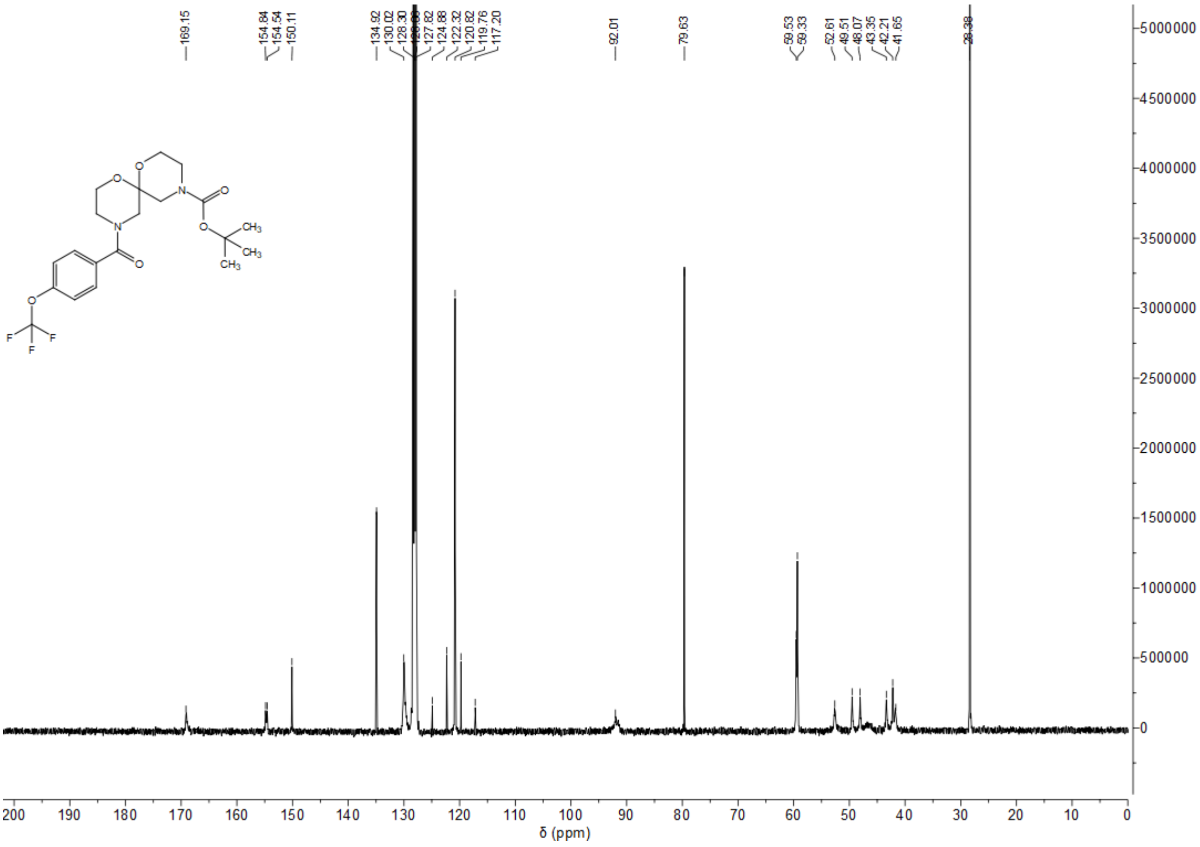

(1,7-dioxa-4,10-diazaspiro[5.5]undecan-4-yl)(4-(trifluoromethoxy)phenyl)methanone hydrochloride (L1-C16·HCl) [^{1}H-NMR data: 400 MHz, CD_3OD; ^{13}C{^{1}H}-NMR data: 101 MHz, CD_3OD; ^{19}F-NMR data: 376 MHz, CD_3OD]:

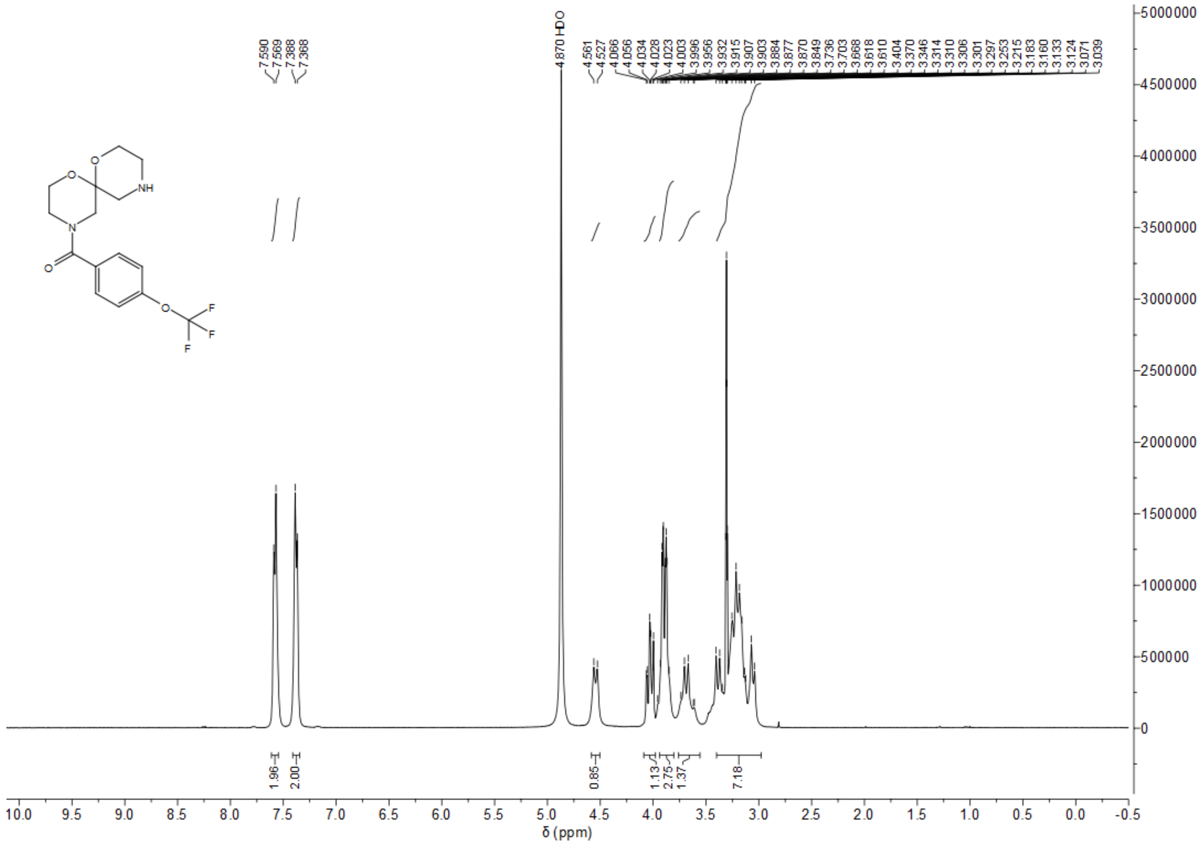

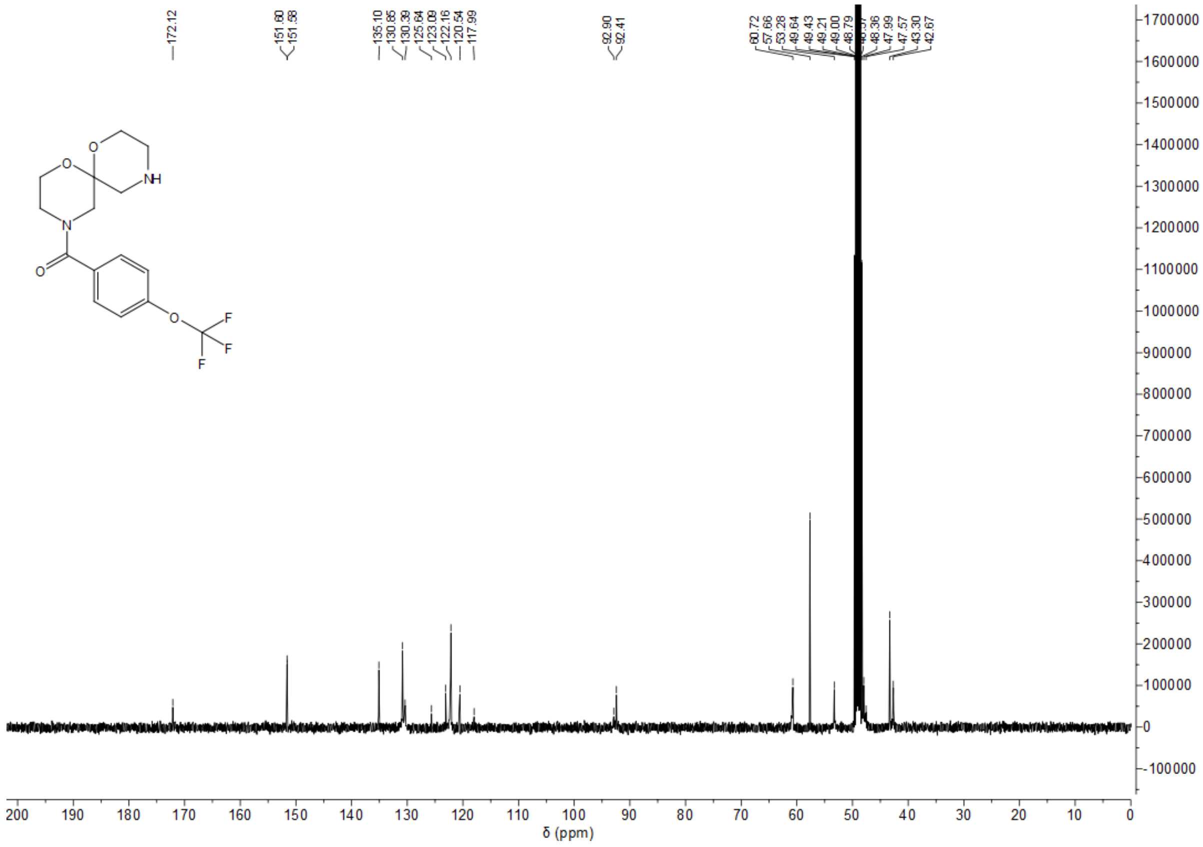

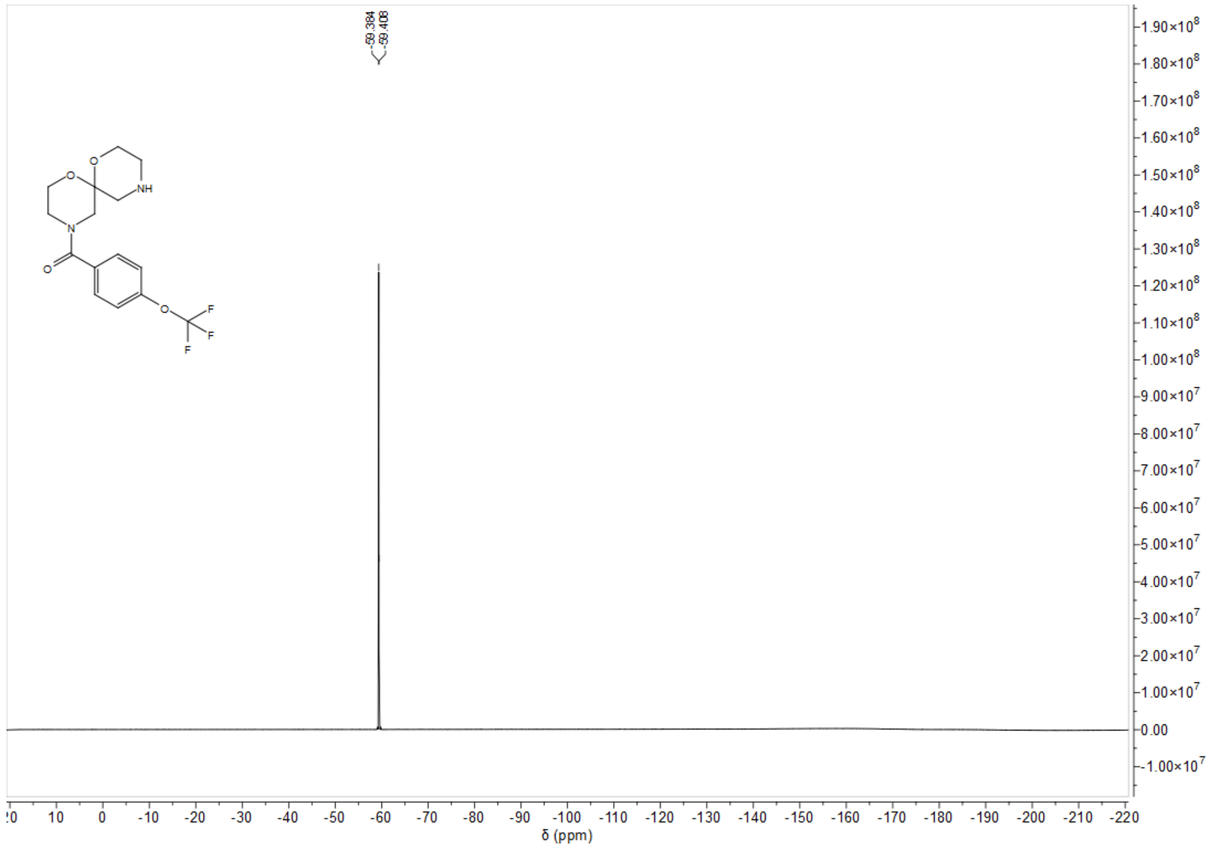

-59.384
-59.408
NH
O
O
N
O
O
F
F
F
δ (ppm)

(4-(trifluoromethoxy)phenyl)(10-((1,3,5-trimethyl-1*H*-pyrazol-4-yl)sulfonyl)-1,7-dioxa-4,10-diazaspiro[5.5]undecan-4-yl)methanone (L1-C16-B10) [^{1}H-NMR data: 400 MHz, CD_3OD; $^{13}C\{^1H\}$-NMR data: 101 MHz, CD_3OD]:

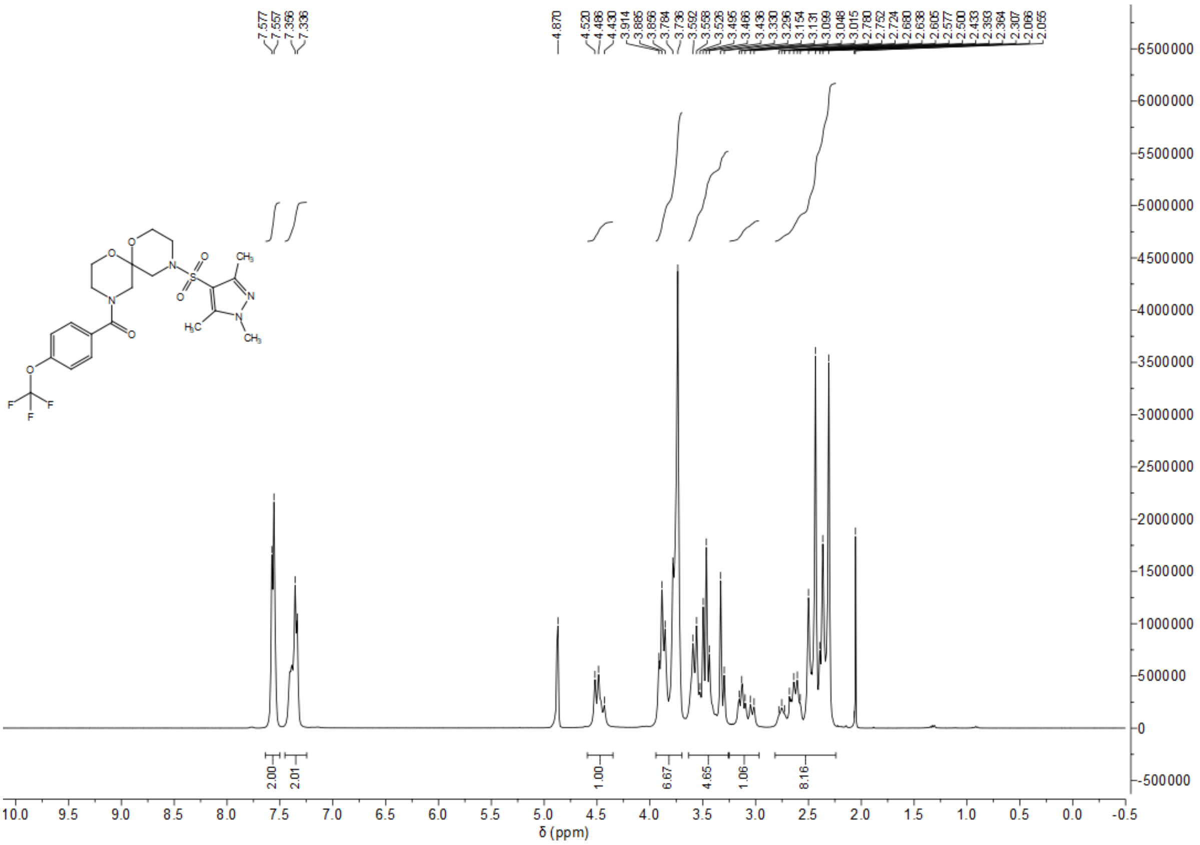

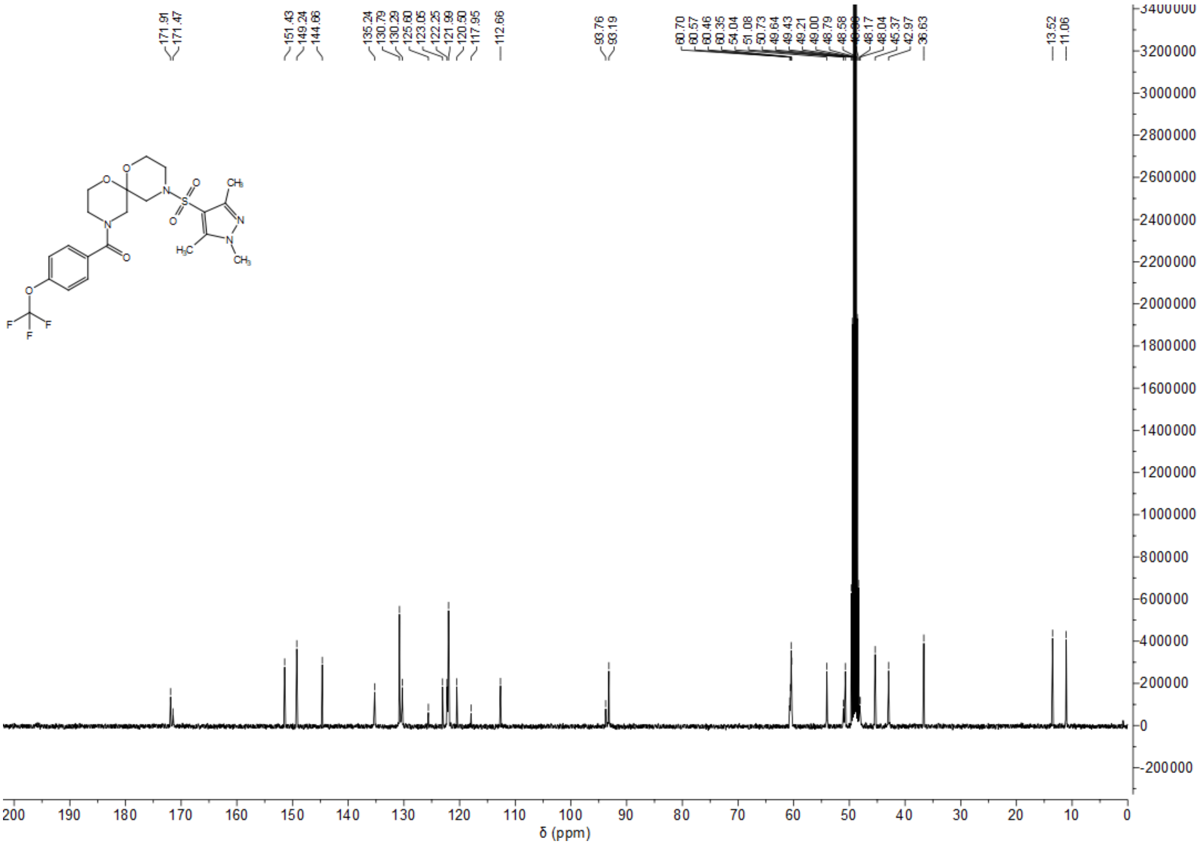

***tert*-butyl 10-(3-methylbutanoyl)-1,7-dioxa-4,10-diazaspiro[5.5]undecane-4-carboxylate (9-C09)**

[^{1}H-NMR data: 400 MHz, C_6D_6; ^{13}C{^{1}H}-NMR data: 101 MHz, C_6D_6]:

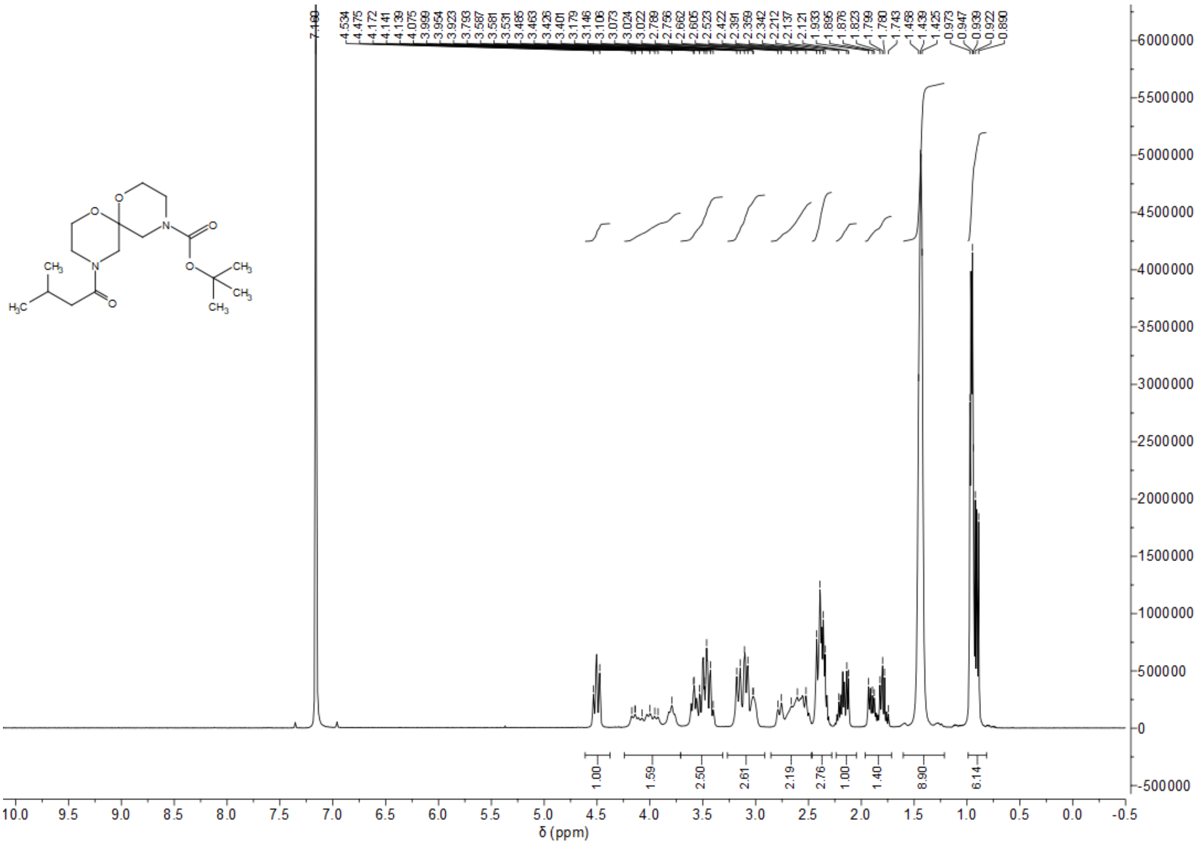

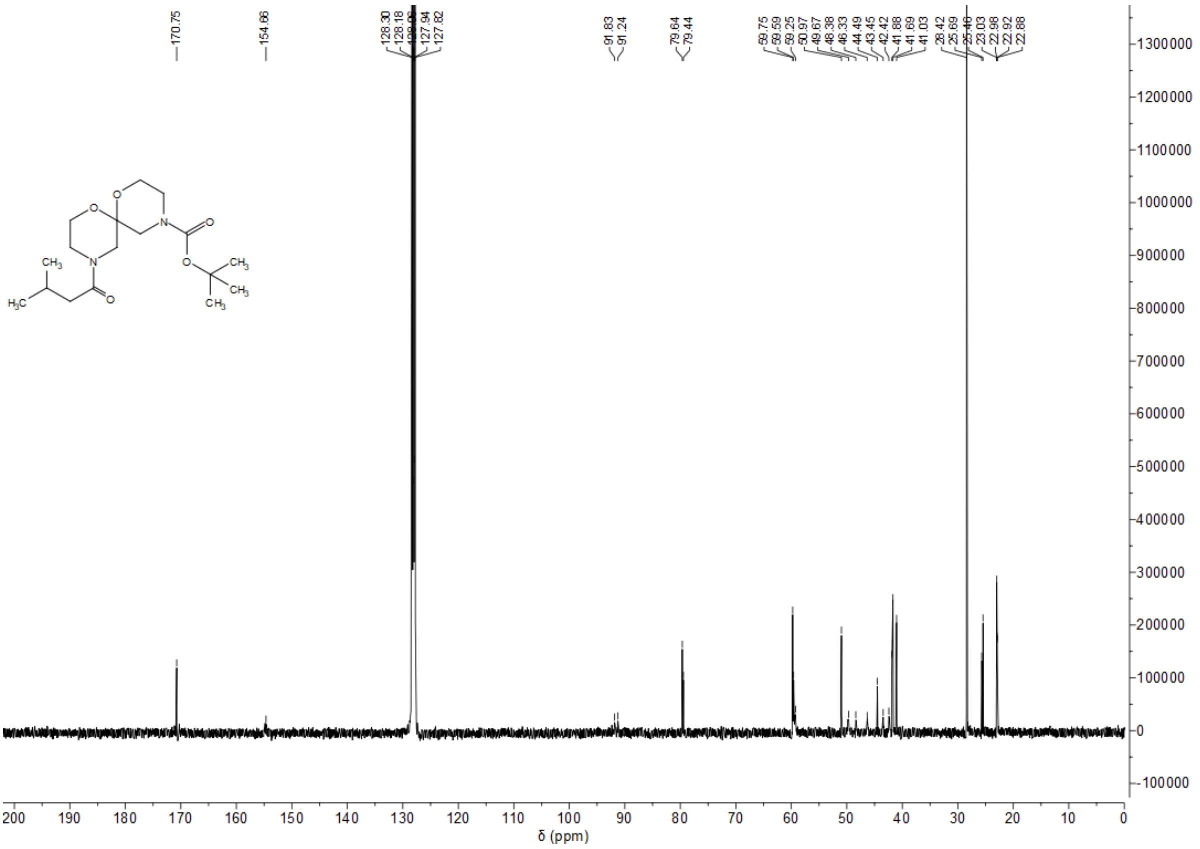

3-methyl-1-(1,7-dioxa-4,10-diazaspiro[5.5]undecan-4-yl)butan-1-one hydrochloride (L1-C09·HCl)

[^{1}H-NMR data: 400 MHz, CD_3OD; $^{13}C\{^{1}H\}$-NMR data: 101 MHz, CD_3OD]:

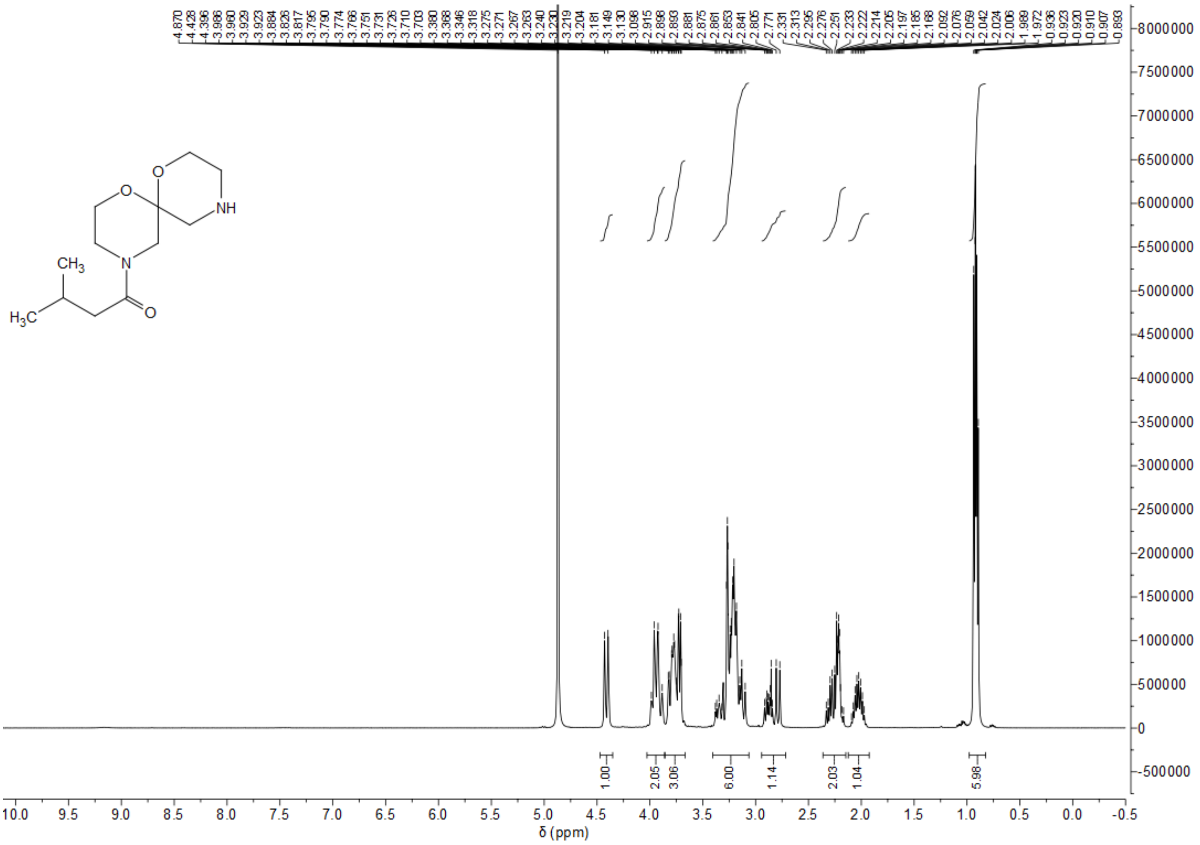

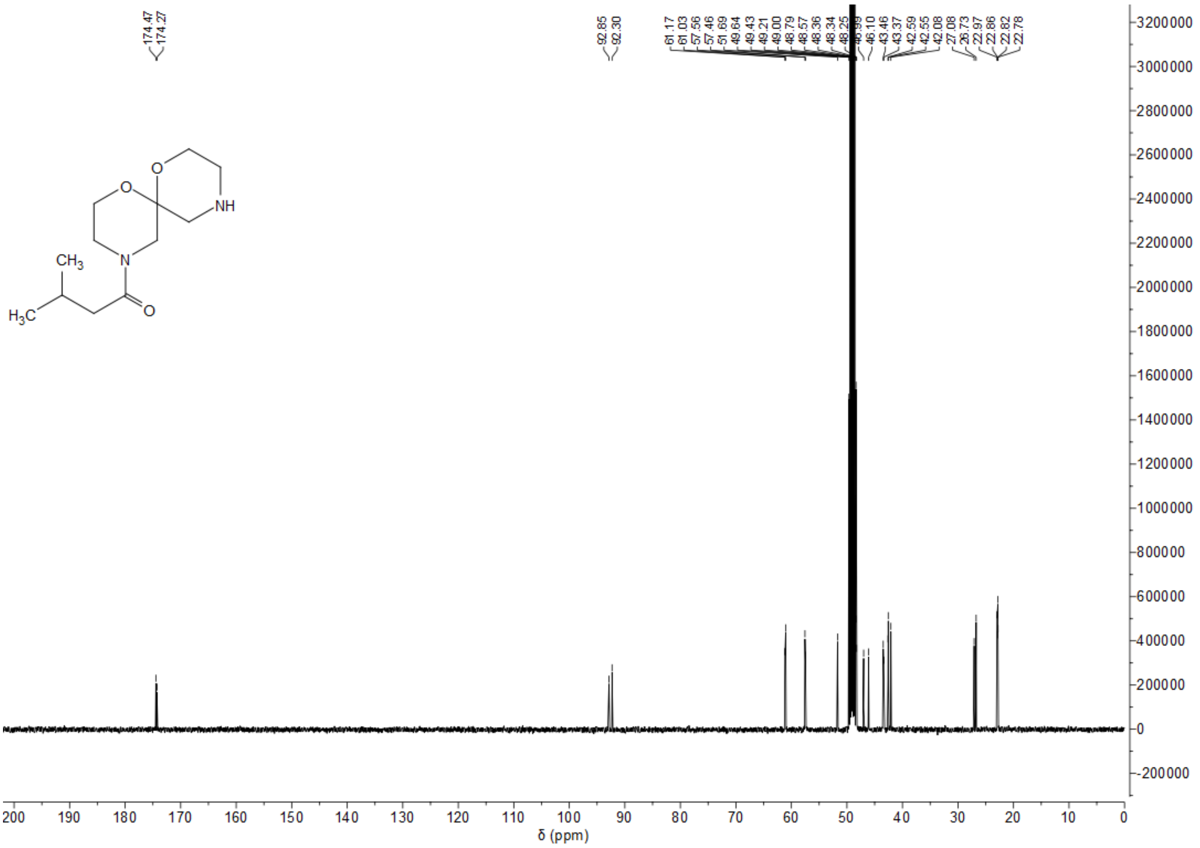

tert-butyl 10-(1-methylcyclohexane-1-carbonyl)-1,7-dioxa-4,10-diazaspiro[5.5]undecane-4-carboxylate (9-C10) [^{1}H-NMR data: 400 MHz, $CDCl_3$; $^{13}C\{^{1}H\}$-NMR data: 101 MHz, $CDCl_3$]:

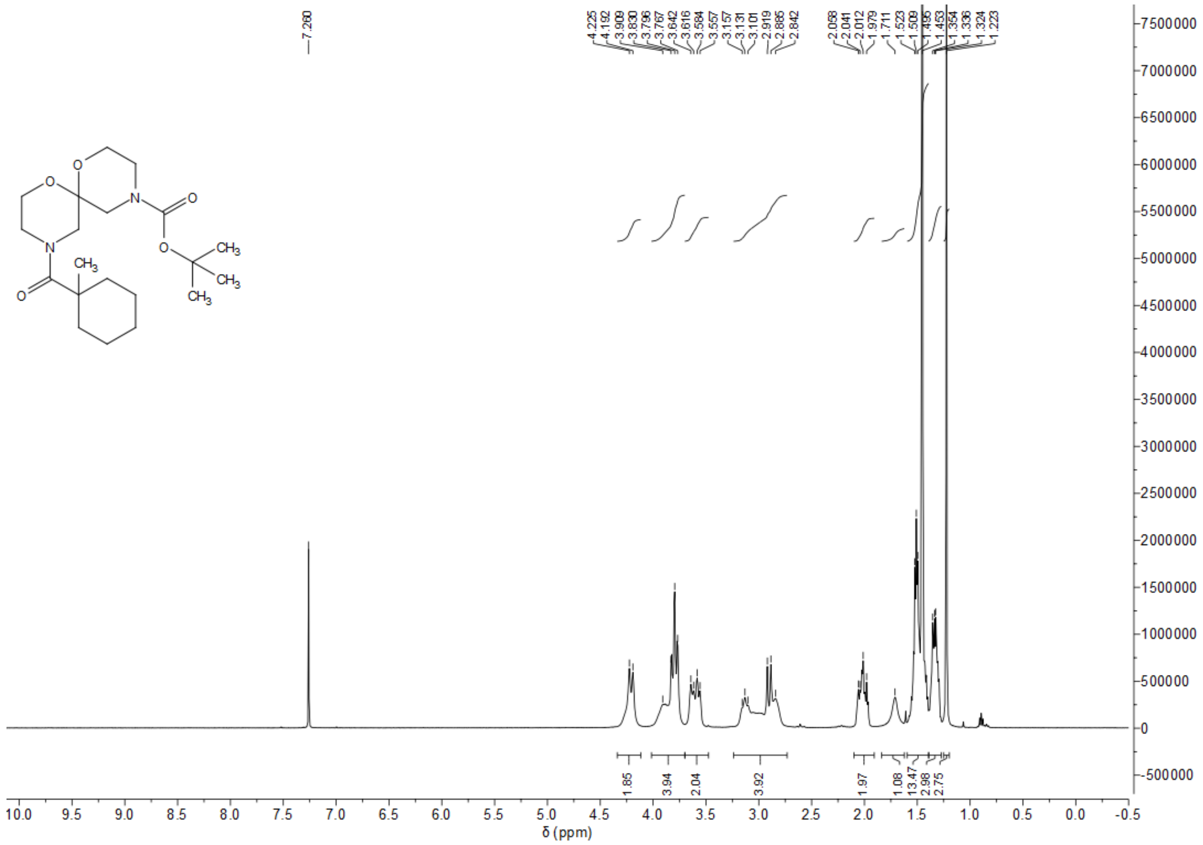

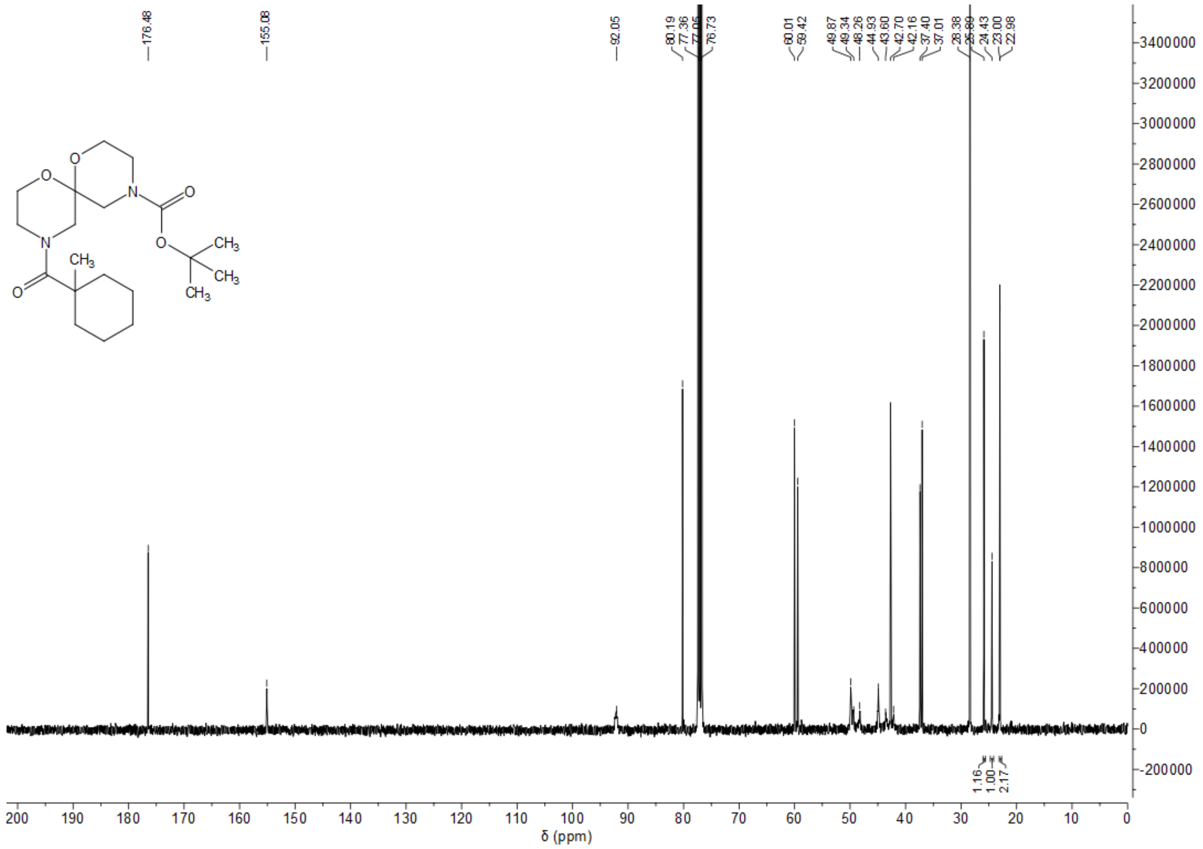

3*H*-[1,2,3]triazolo[4,5-b]pyridine-3-yl 1-methylcyclohexane-1-carboxylate (S54) [^{1}H-NMR data: 400 MHz, $CDCl_3$; $^{13}C\{^{1}H\}$-NMR data: 101 MHz, $CDCl_3$]:

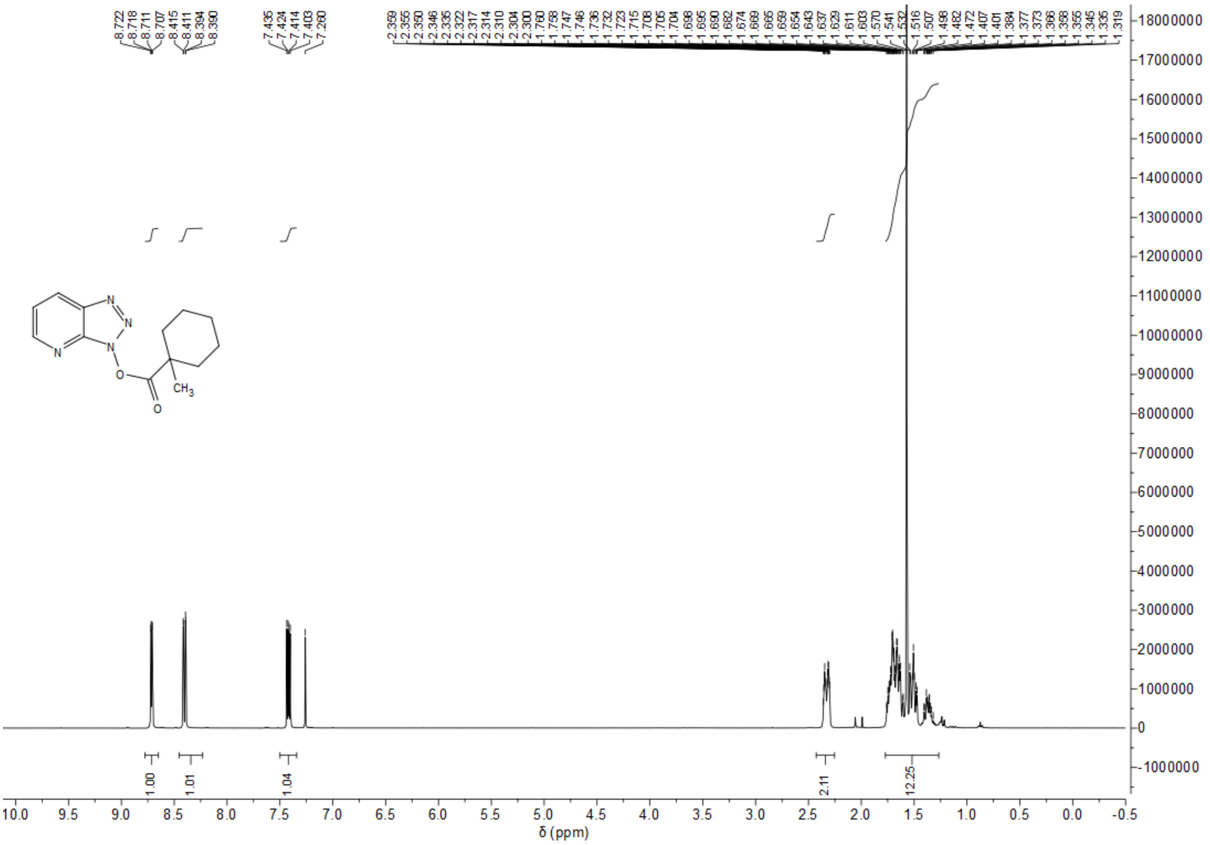

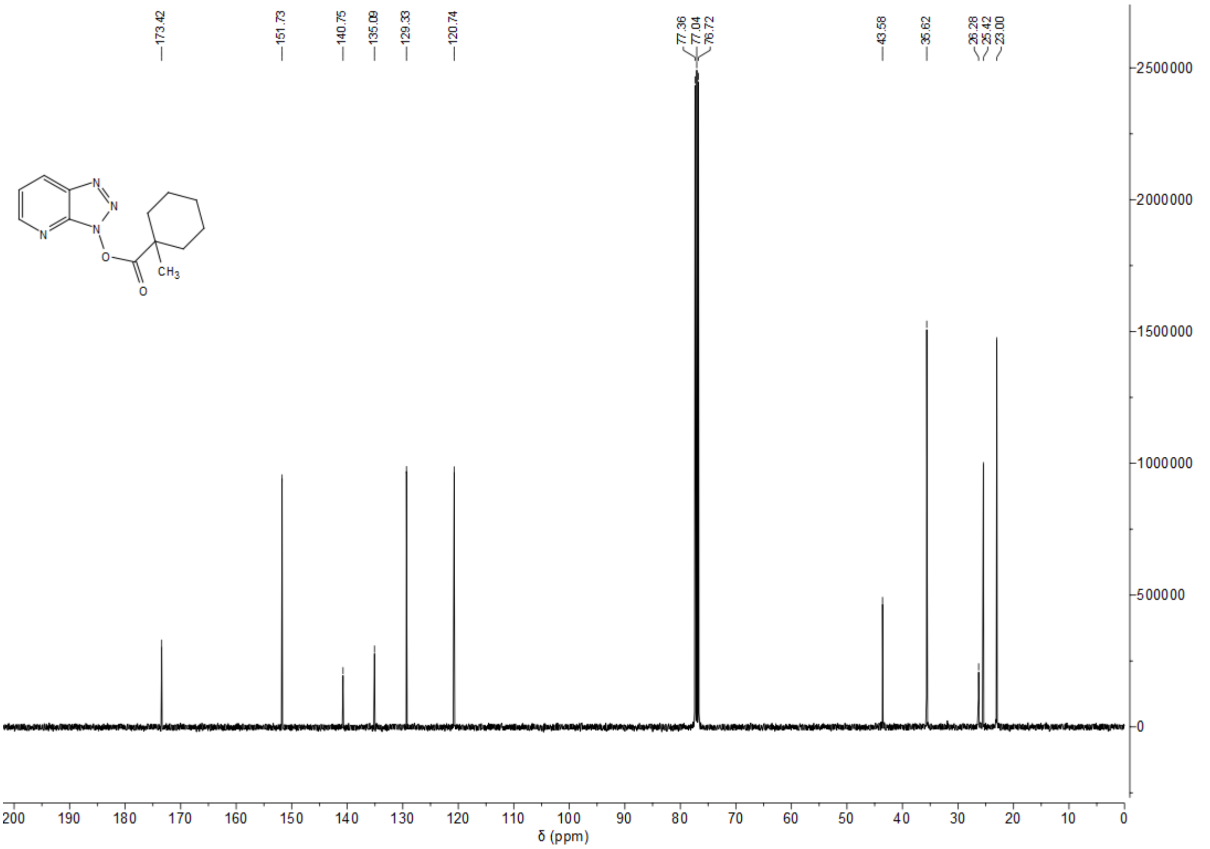

(1-methylcyclohexyl)(1,7-dioxa-4,10-diazaspiro[5.5]undecan-4-yl)methanone (L1-C10) [^{1}H-NMR data: 400 MHz, CD_3OD; $^{13}C\{^{1}H\}$-NMR data: 101 MHz, CD_3OD]:

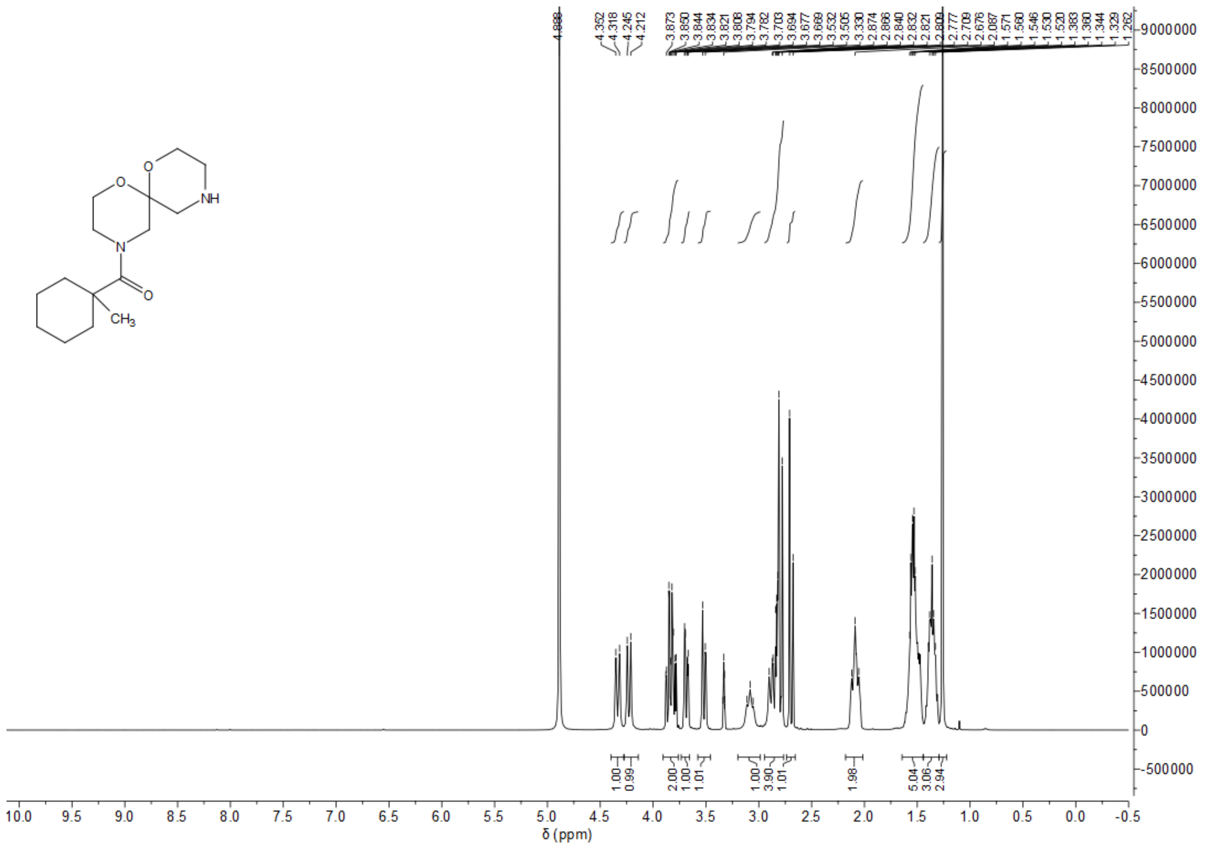

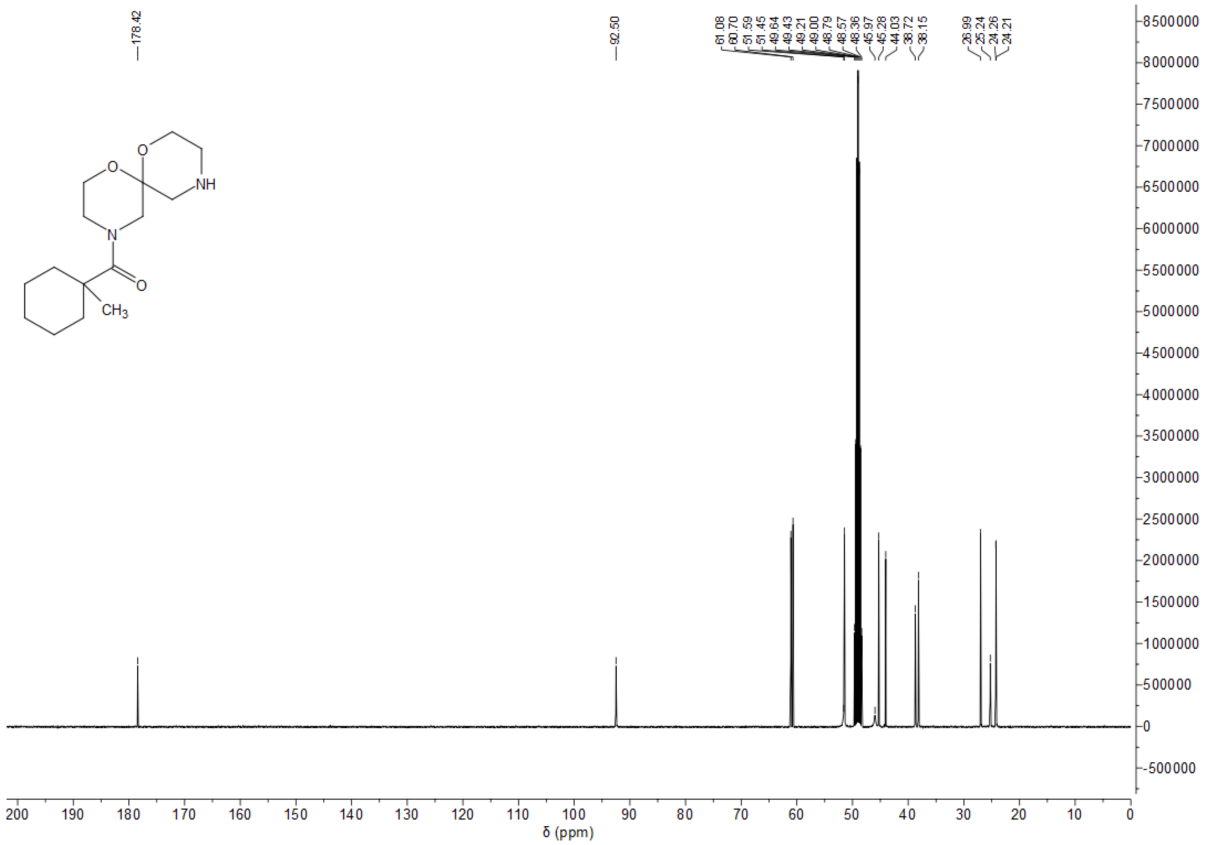

(10-(2,3-dihydro-1*H*-inden-2-yl)-1,7-dioxa-4,10-diazaspiro[5.5]undecan-4-yl)(1-methylcyclohexyl)methanone (L1-C10-K02) [^{1}H-NMR data: 400 MHz, CD_3OD; $^{13}C\{^{1}H\}$-NMR data: 101 MHz, CD_3OD]:

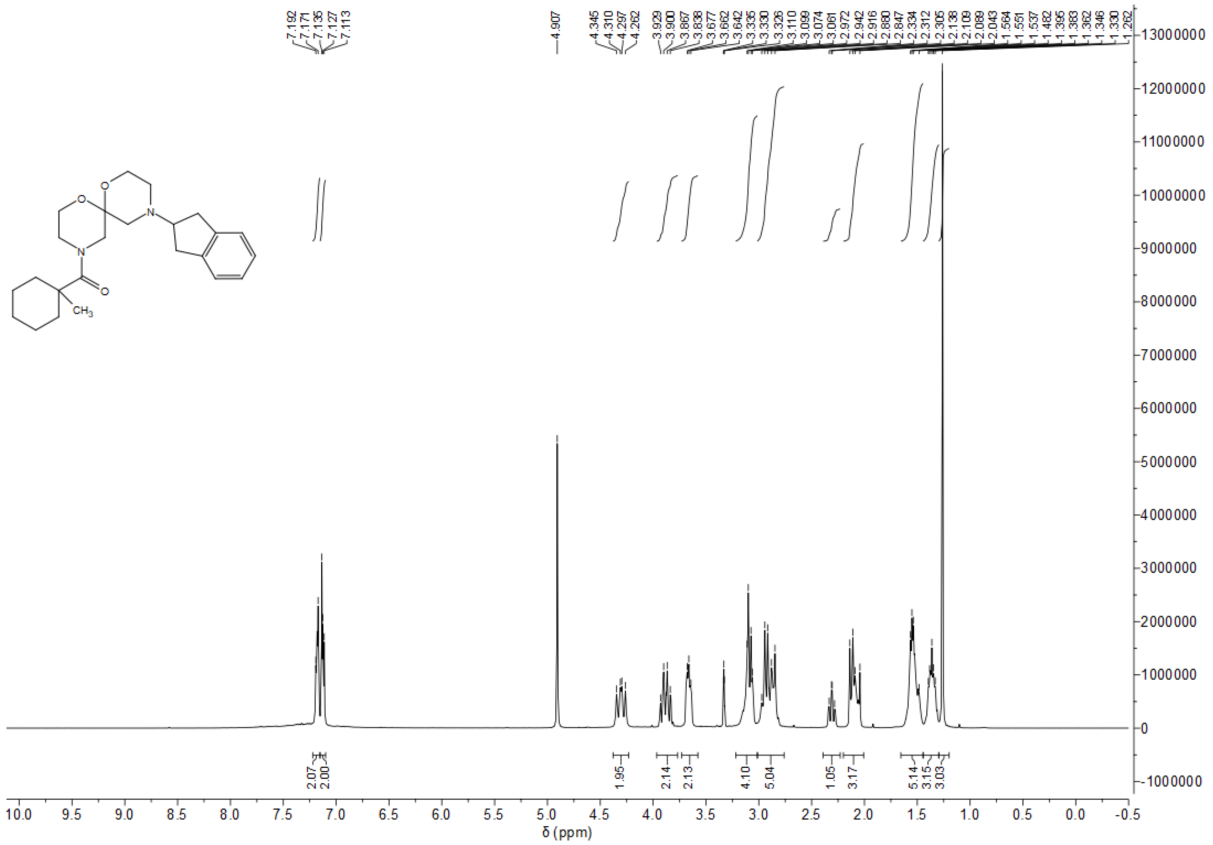

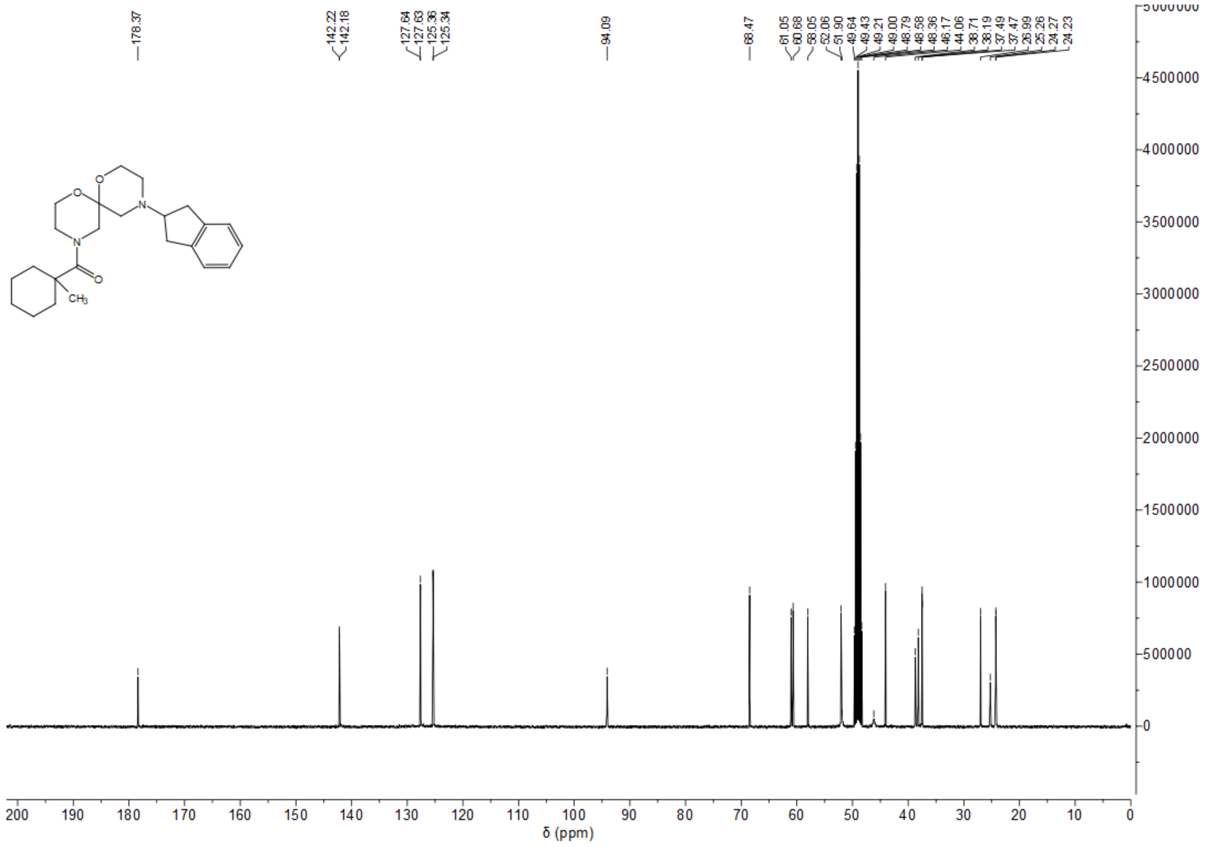

***tert*-butyl 10-(4-methyloxazole-5-carbonyl)-1,7-dioxa-4,10-diazaspiro[5.5]undecane-4-carboxylate (9-C30) [^{1}H-NMR data: 400 MHz, C_6D_6; ^{13}C{^{1}H}-NMR data: 101 MHz, C_6D_6]:**

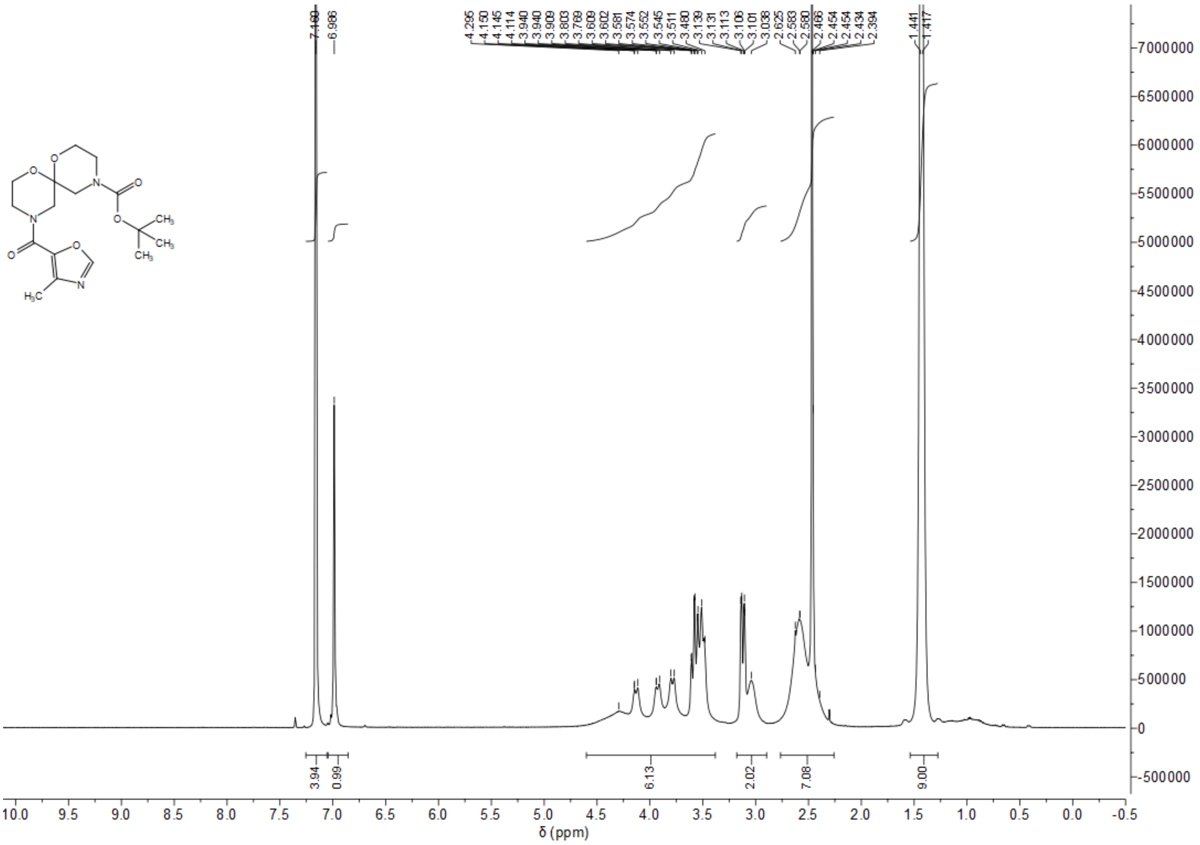

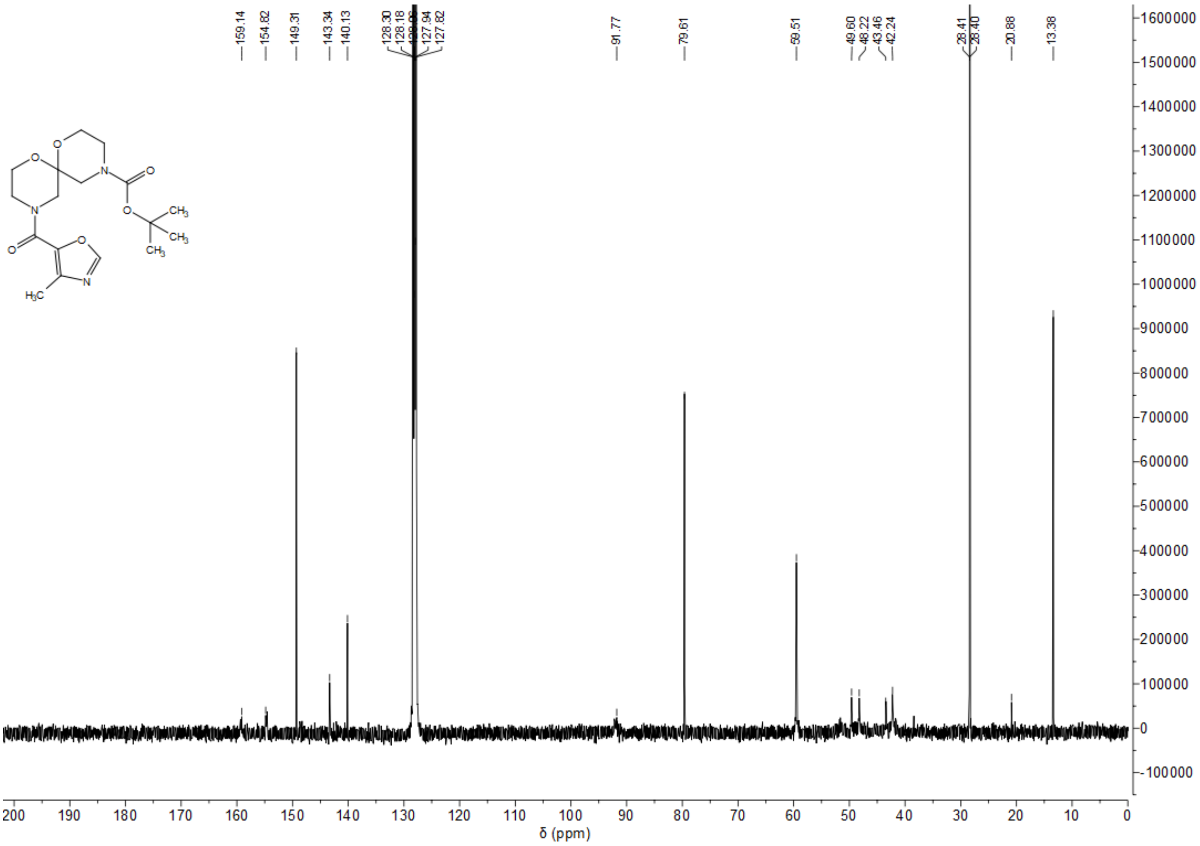

(4-methyloxazol-5-yl)(1,7-dioxa-4,10-diazaspiro[5.5]undecan-4-yl)methanone (L1-C30) [^{1}H-NMR data: 400 MHz, CD_3OD; $^{13}C\{^{1}H\}$-NMR data: 101 MHz, CD_3OD]:

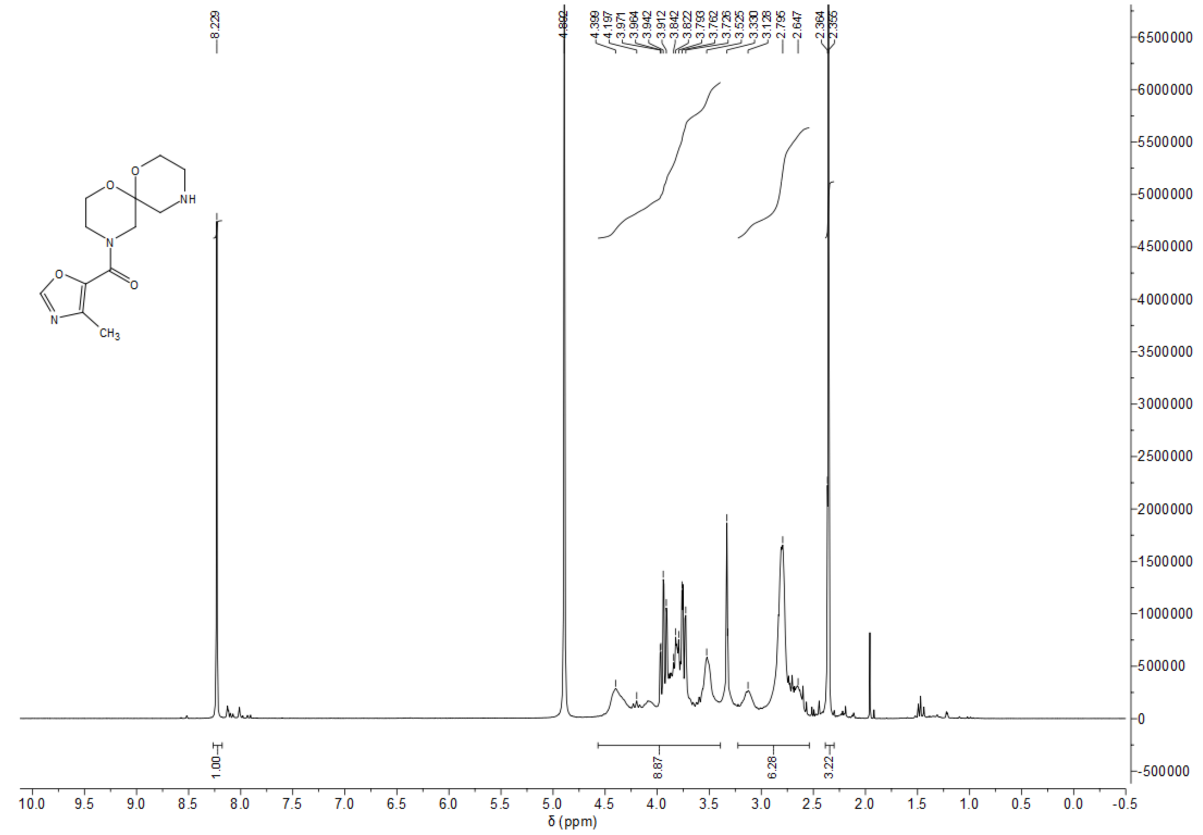

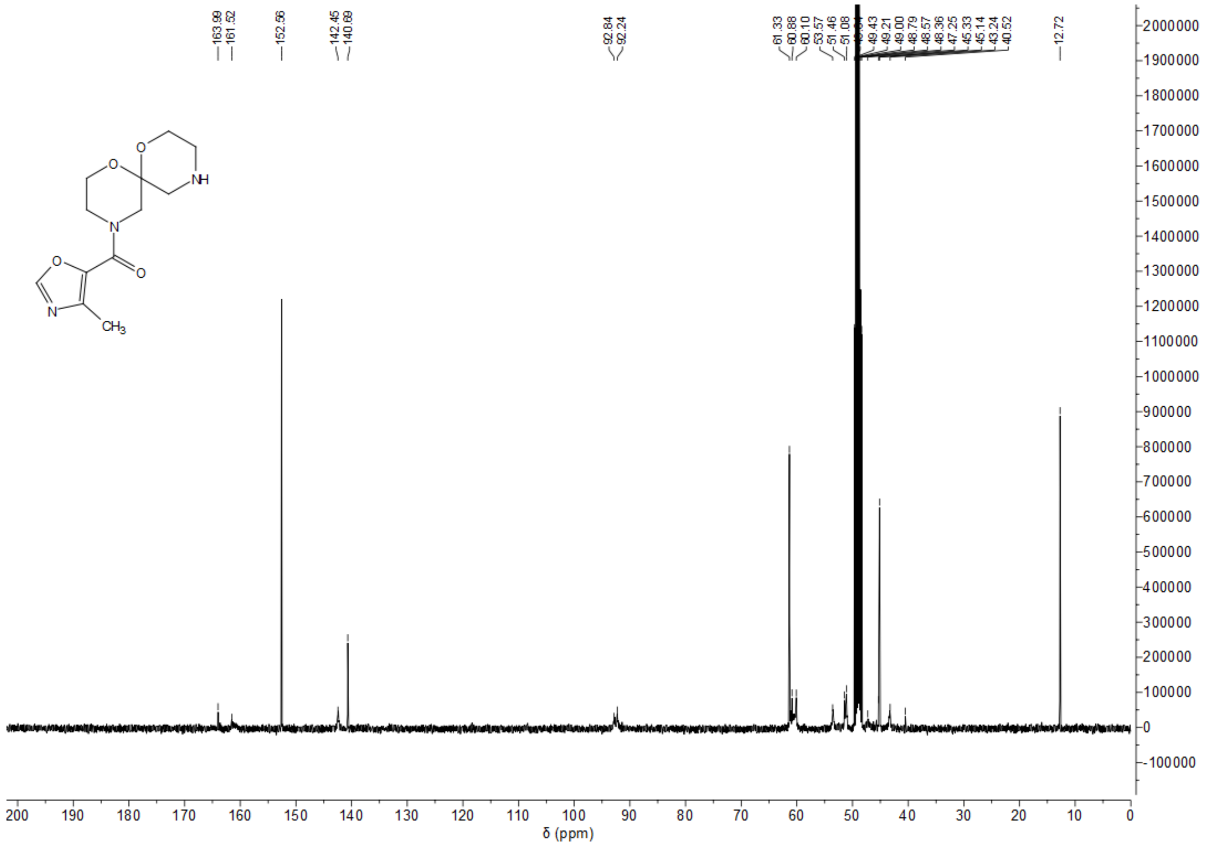

***tert*-butyl 10-(1-methyl-1*H*-imidazole-2-carbonyl)-1,7-dioxa-4,10-diazaspiro[5.5]undecane-4-carboxylate (9-C27) [^{1}H-NMR data: 400 MHz, C_6D_6; $^{13}C\{^{1}H\}$-NMR data: 101 MHz, C_6D_6]:**

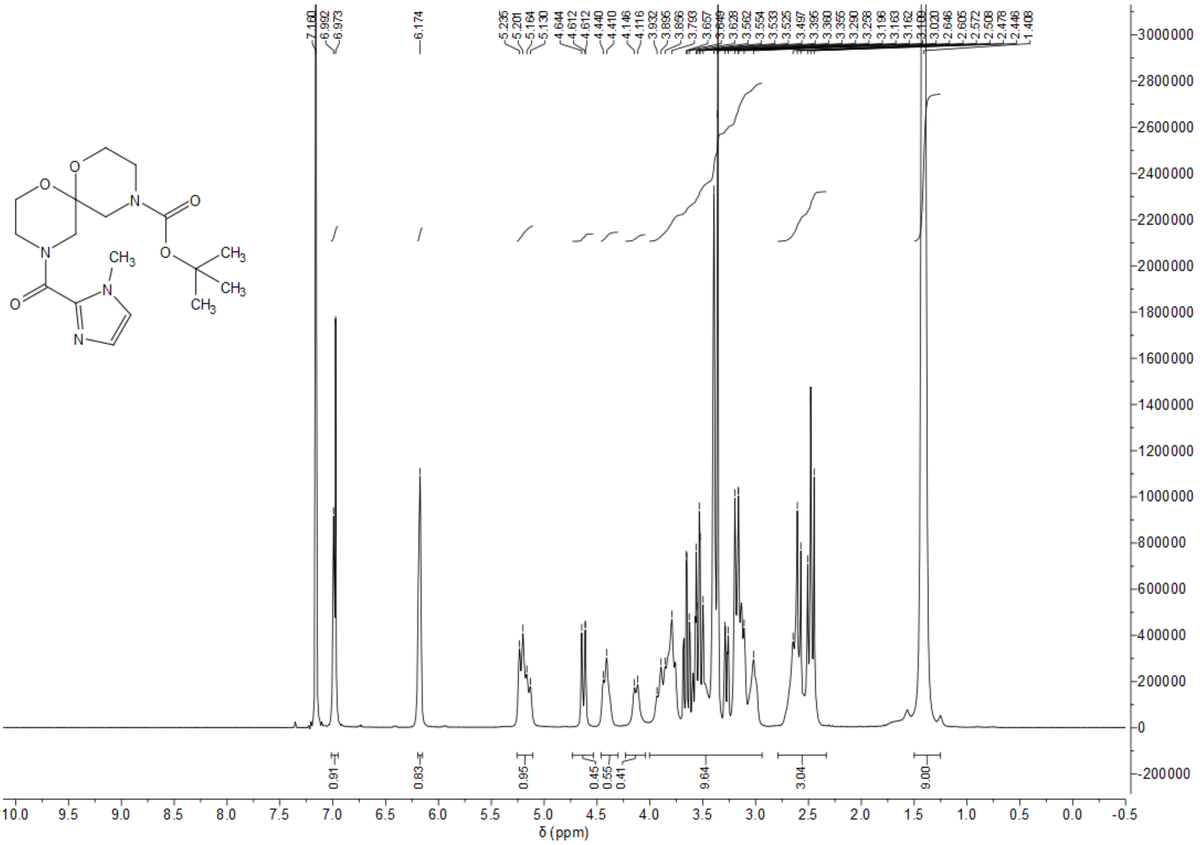

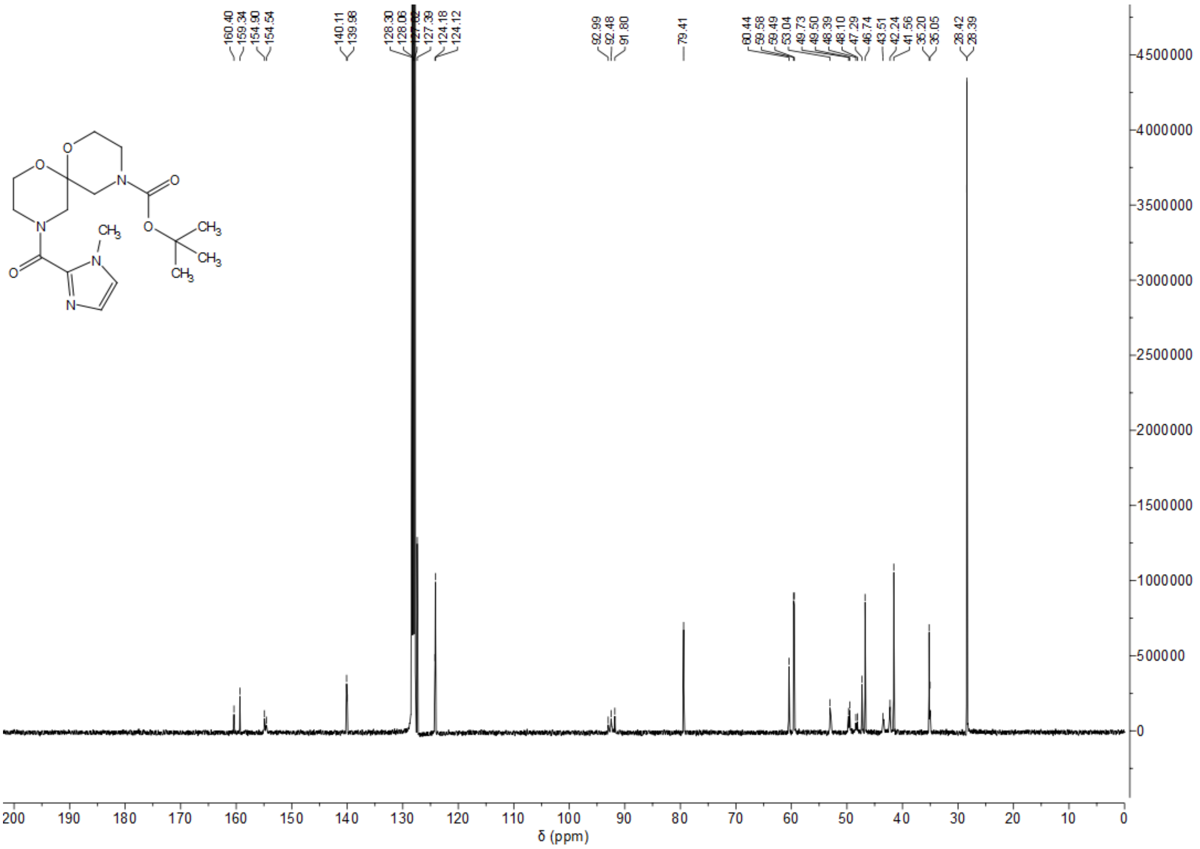

(1-methyl-1*H*-imidazol-2-yl)(1,7-dioxa-4,10-diazaspiro[5.5]undecan-4-yl)methanone (L1-C27) [^{1}H-NMR data: 400 MHz, CD_3OD; $^{13}C\{^{1}H\}$-NMR data: 101 MHz, CD_3OD]:

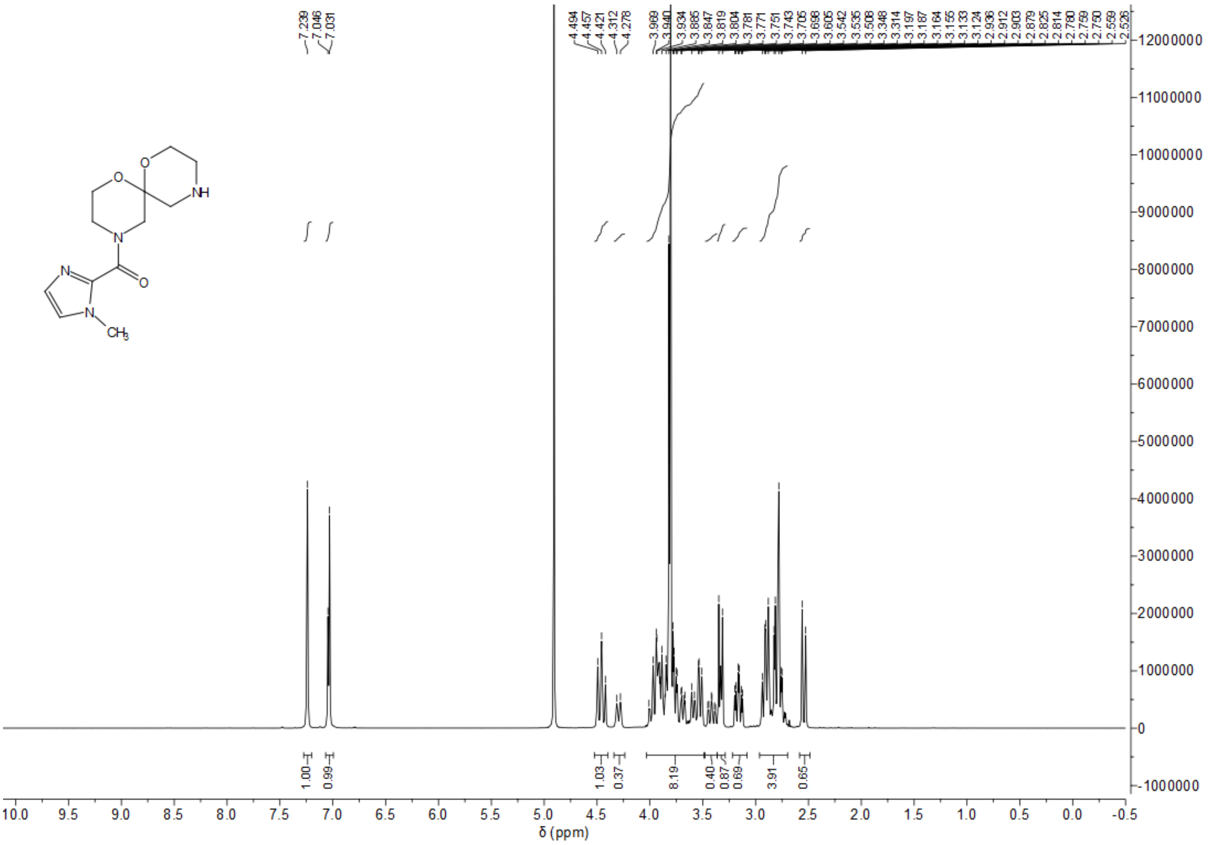

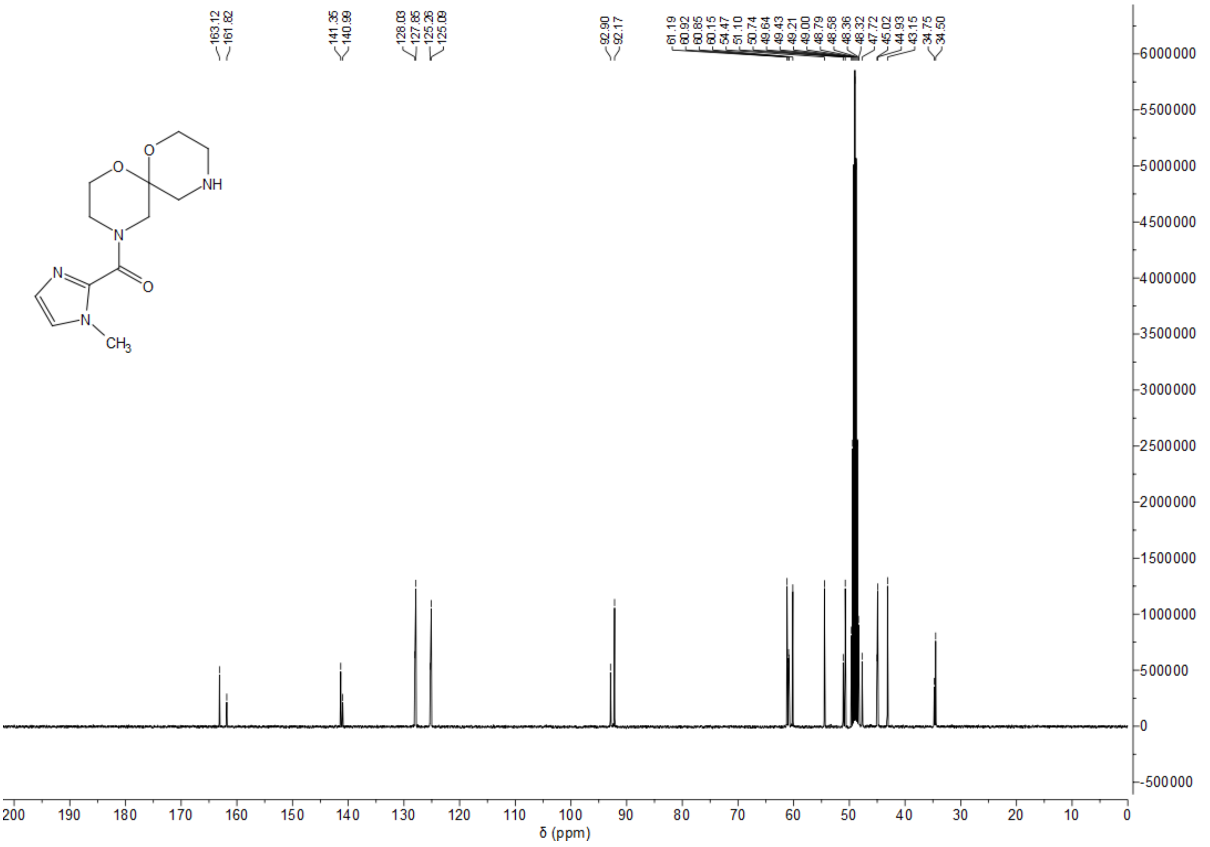

***N*-(4-methoxybenzyl)-10-(1-methyl-1*H*-imidazole-2-carbonyl)-1,7-dioxa-4,10-diazaspiro[5.5]undecane-4-carboxamide (L1-C27-I11) [^{1}H-NMR data: 400 MHz, CD_3OD; $^{13}C\{^{1}H\}$-NMR data: 101 MHz, CD_3OD]:**

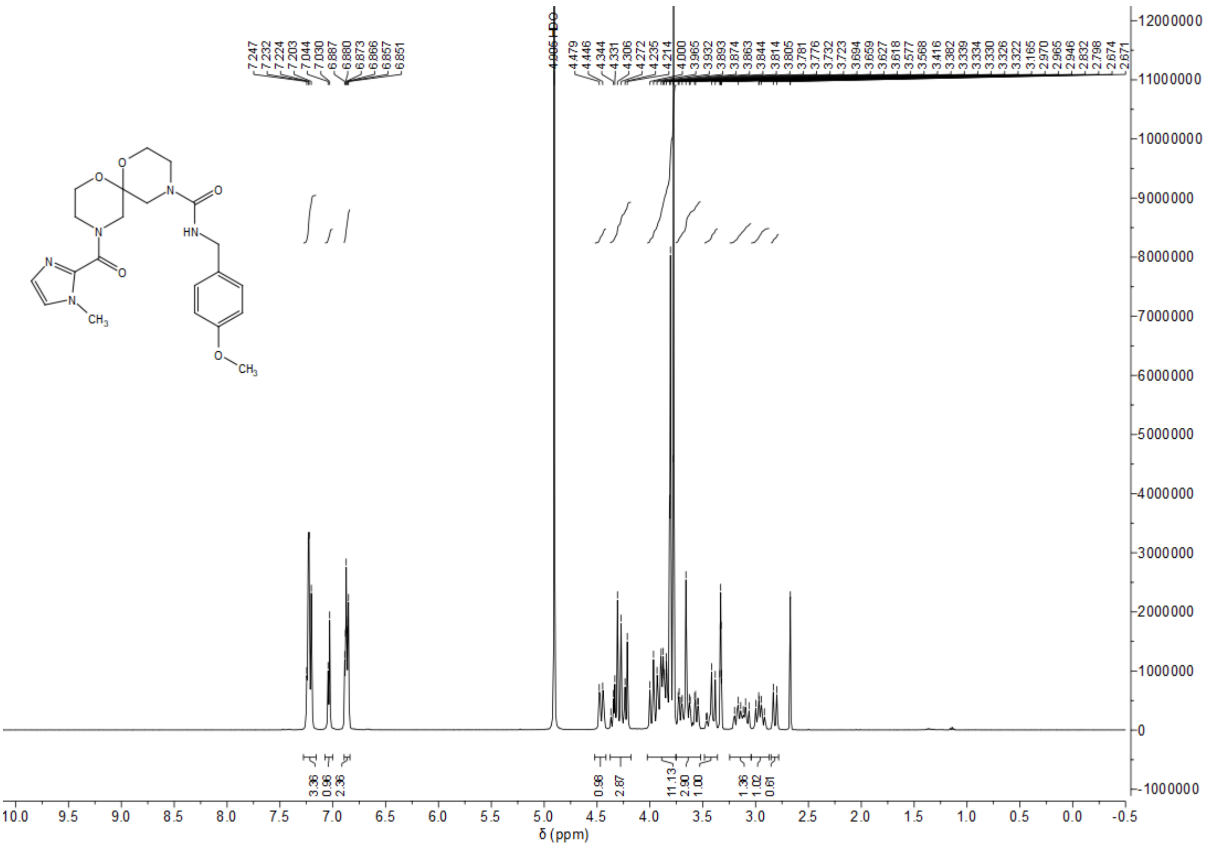

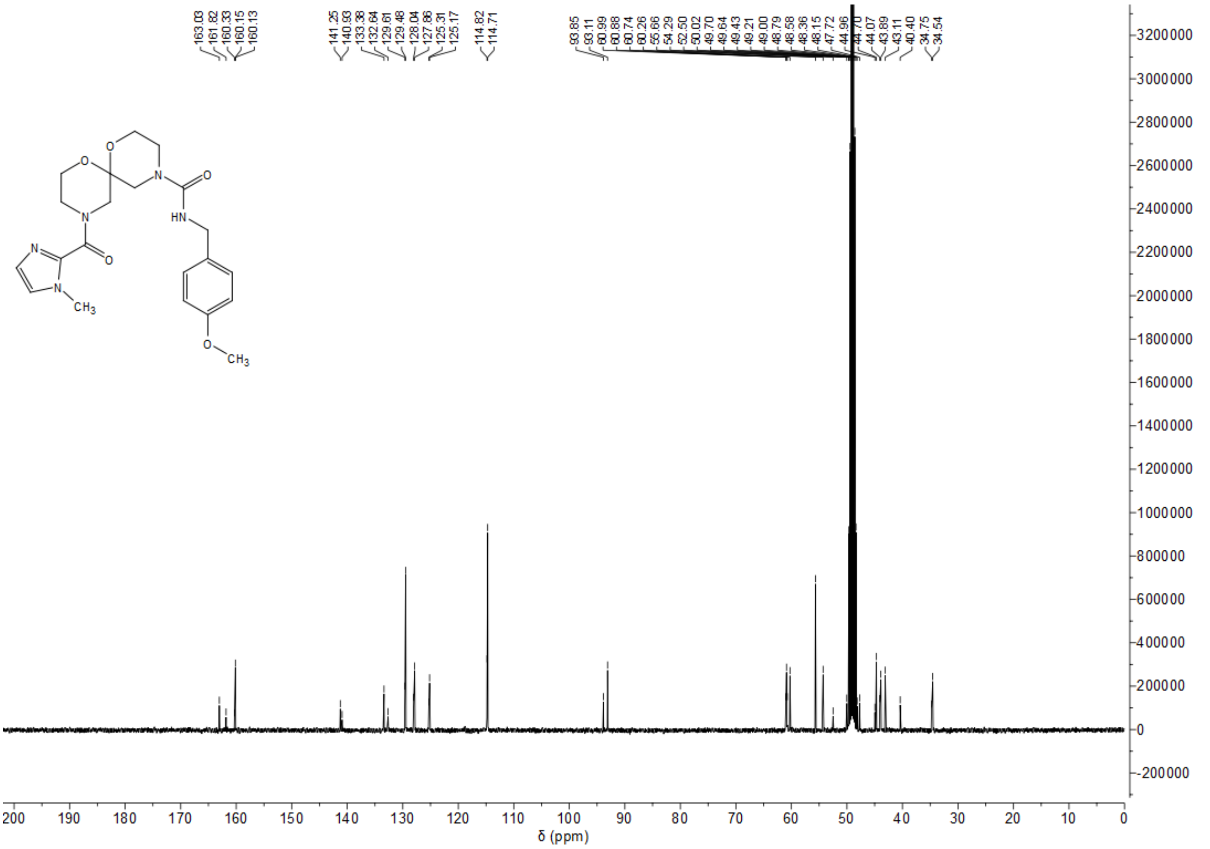

***tert*-butyl 4-(4-methyloxazole-5-carbonyl)-1,7-dioxa-4,11-diazaspiro[5.6]dodecane-11-carboxylate (25-C30 *or* 26 in main article) [^{1}H-NMR data: 400 MHz, $CDCl_3$; $^{13}C\{^{1}H\}$-NMR data: 101 MHz, $CDCl_3$]:**

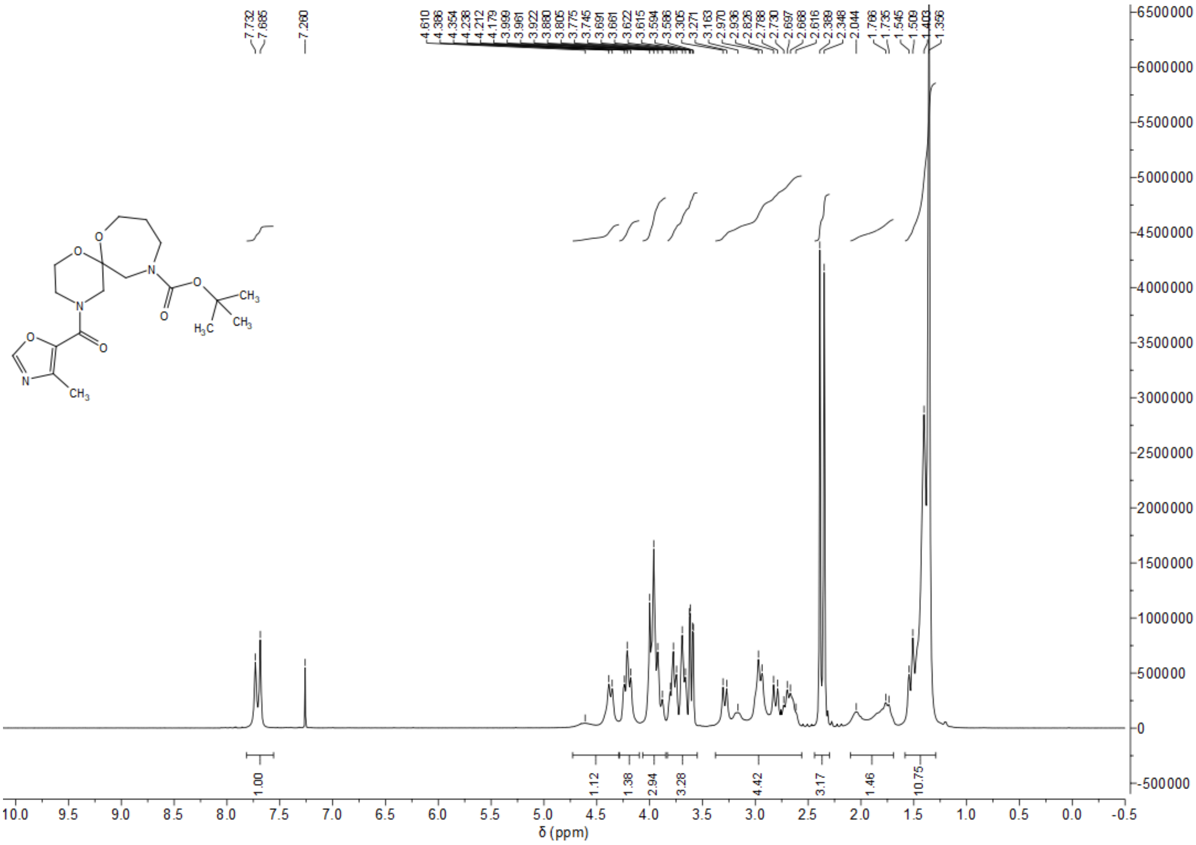

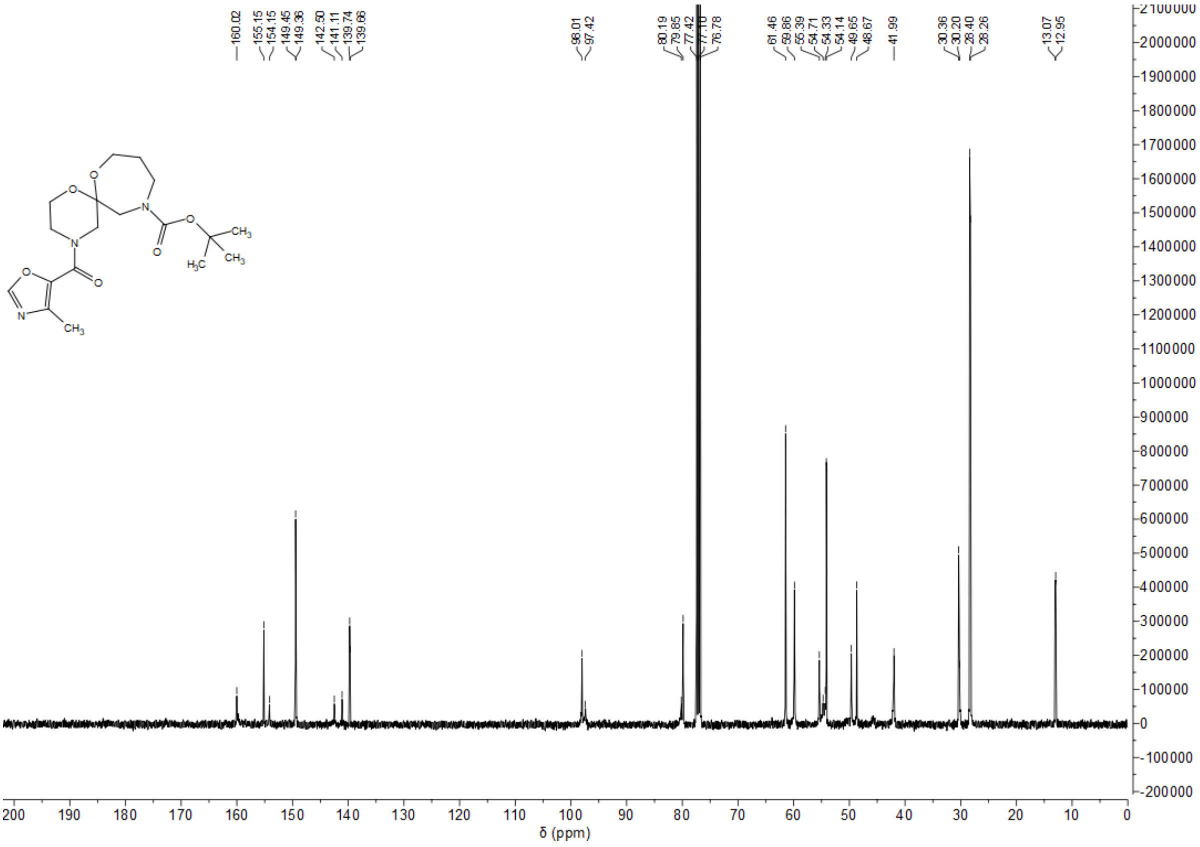

(4-methyloxazol-5-yl)(1,7-dioxa-4,11-diazaspiro[5.6]dodecan-4-yl)methanone (L2-C30 *or* 29 in the main article) [^{1}H-NMR data: 400 MHz, CD_3OD; $^{13}C\{^{1}H\}$-NMR data: 101 MHz, CD_3OD]:

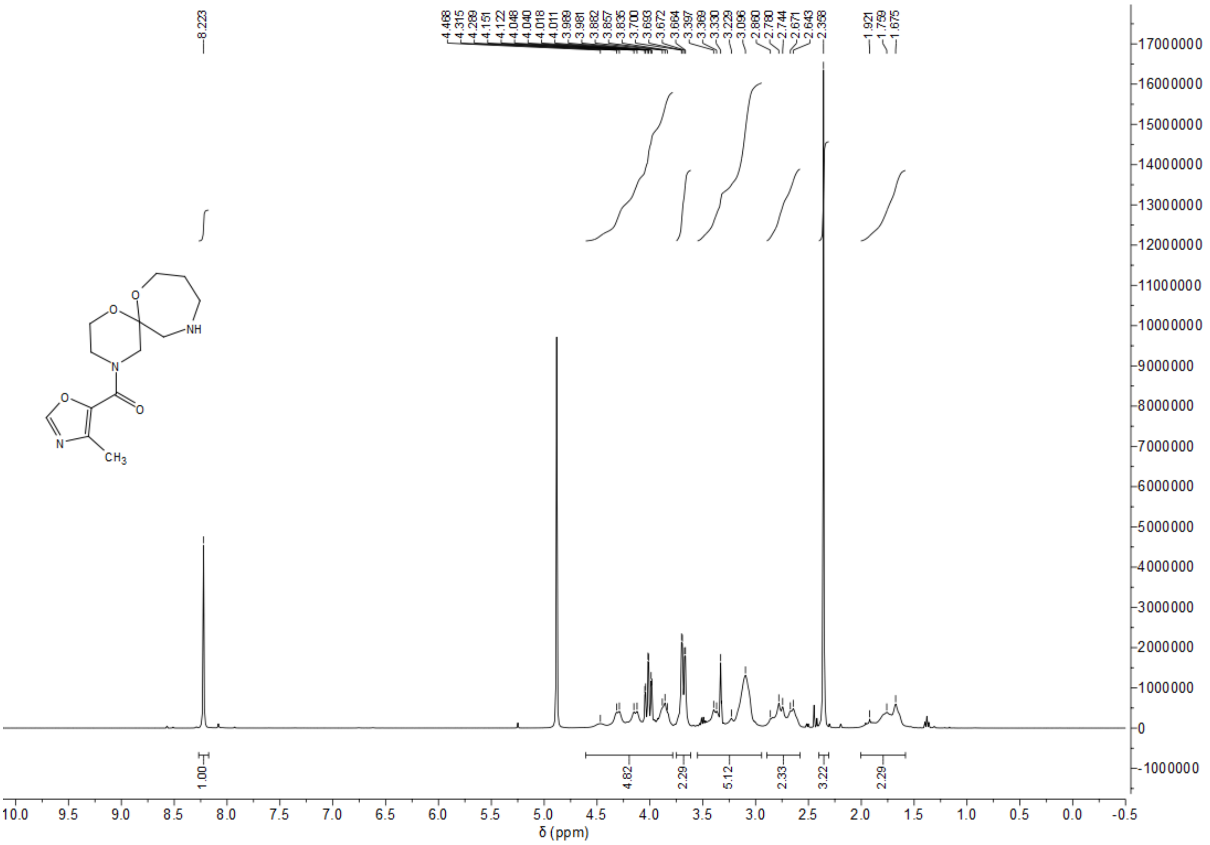

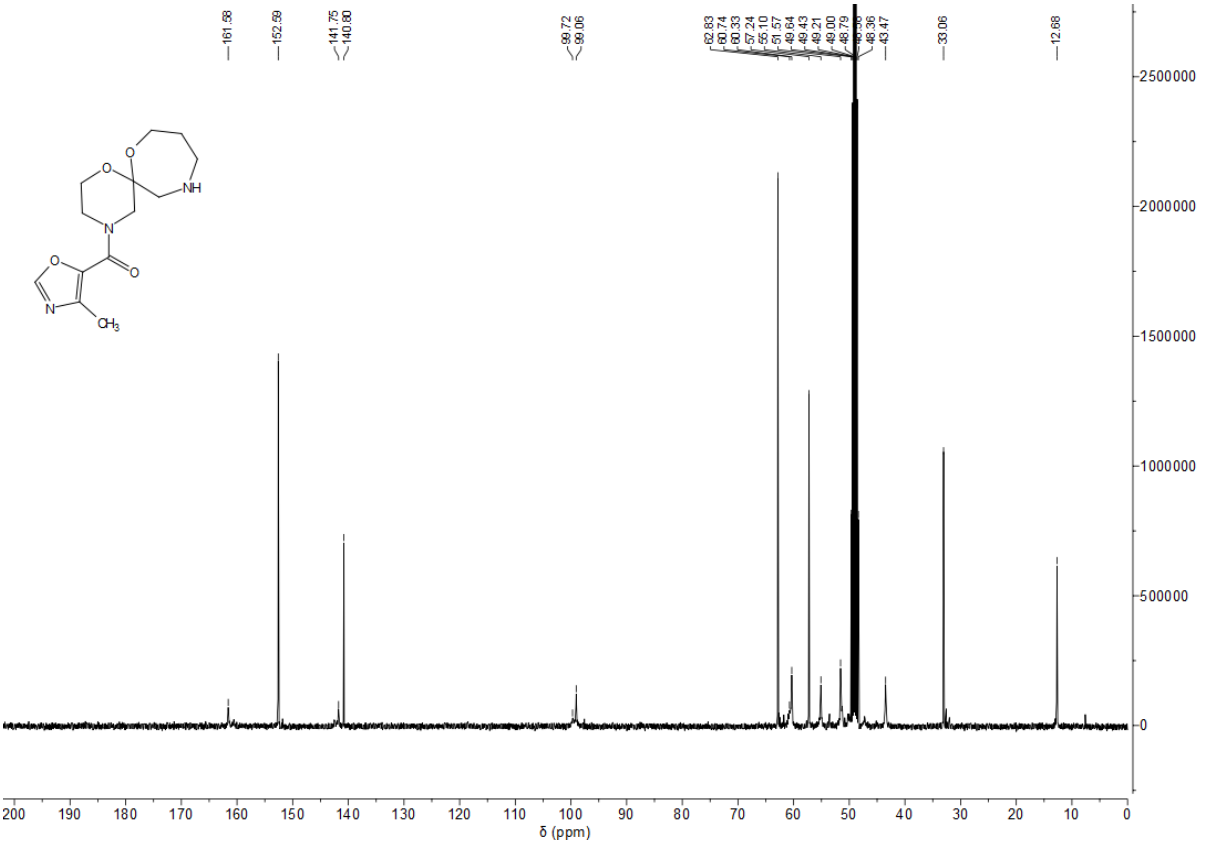

(4-methyloxazol-5-yl)(11-(methylsulfonyl)-1,7-dioxa-4,11-diazaspiro[5.6]dodecan-4-yl)methanone (L2-C30-B09) [^{1}H-NMR data: 400 MHz, CD_3OD; ^{13}C{^{1}H}-NMR data: 101 MHz, CD_3OD]:

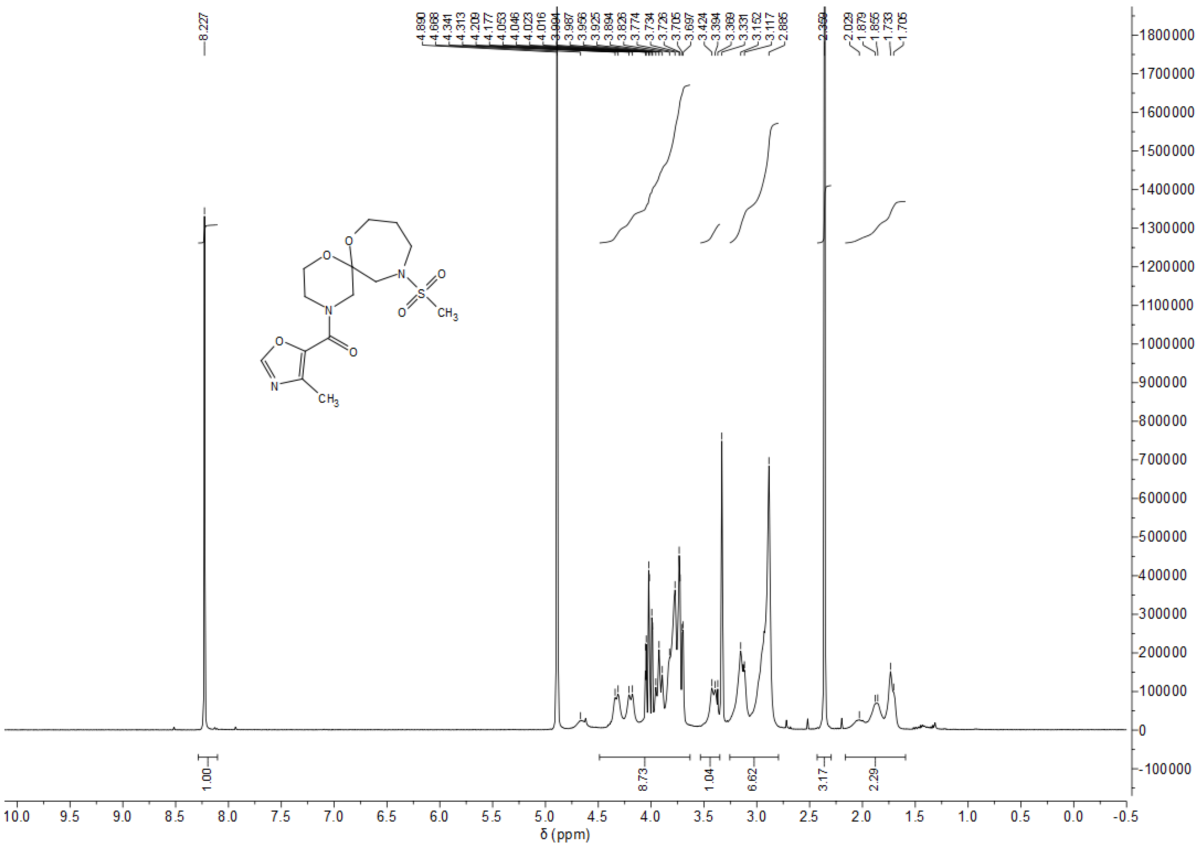

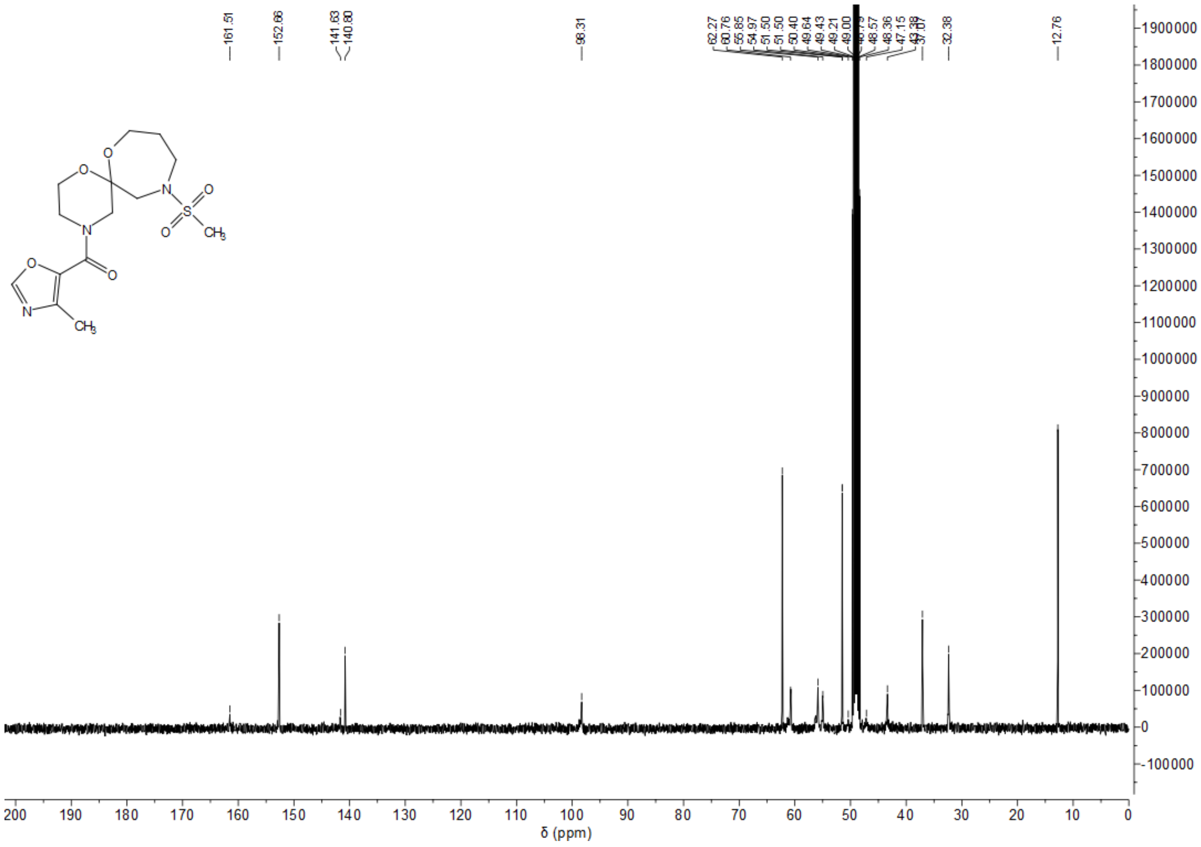

(4-methyloxazol-5-yl)(11-((3-phenylisoxazol-5-yl)methyl)-1,7-dioxa-4,11-diazaspiro[5.6]dodecan-4-yl)methanone (L2-C30-A05) [^{1}H-NMR data: 400 MHz, CD_3OD; $^{13}C\{^{1}H\}$-NMR data: 101 MHz, CD_3OD]:

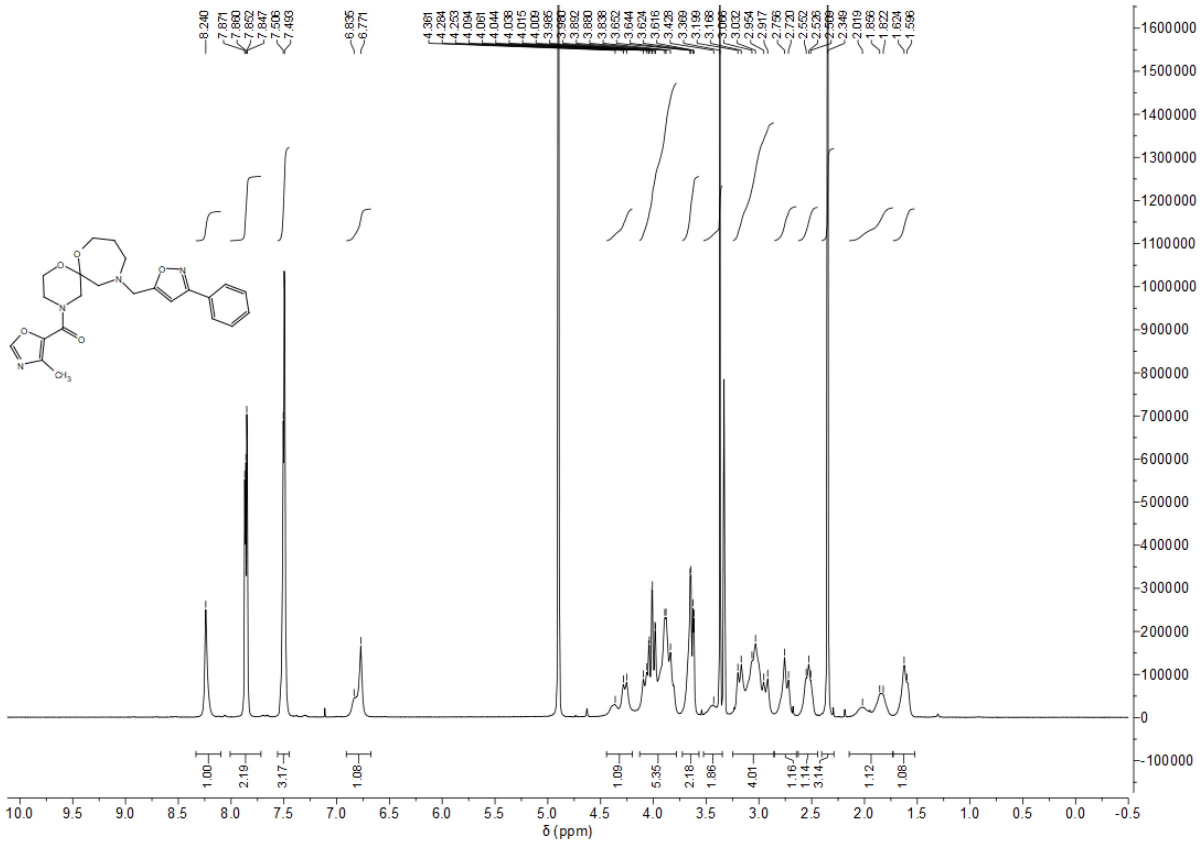

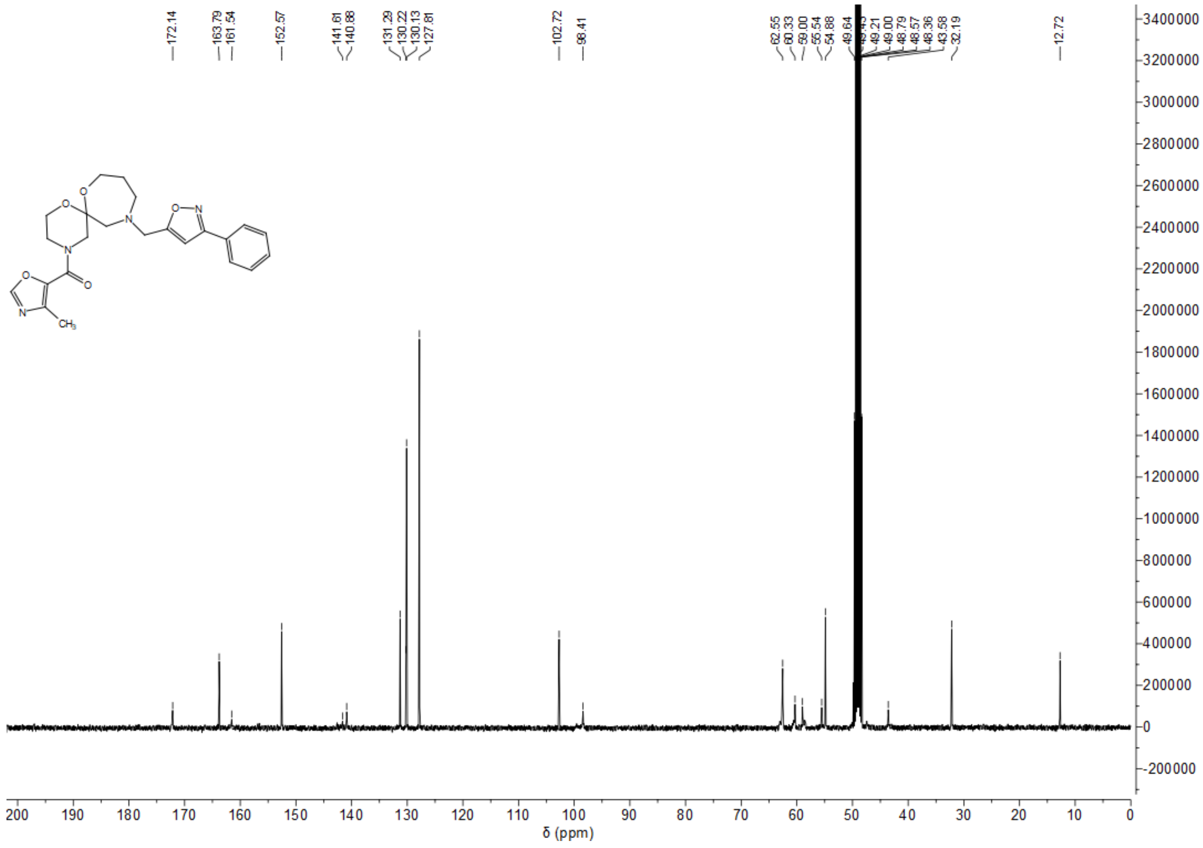

Supplement: Supplementary file 2 — jo4c02690_si_002.pdf [file jo4c02690_si_002.pdf]
